# The catalytic enantioselective [1,2]-Wittig rearrangement cascade of allylic ethers

**DOI:** 10.1038/s41557-025-02022-4

**Published:** 2026-01-06

**Authors:** Tengfei Kang, Justin O’Yang, Kevin Kasten, Samuel S. Allsop, Toby Lewis-Atwell, Elliot H. E. Farrar, Martin Juhl, David B. Cordes, Aidan P. McKay, Matthew N. Grayson, Andrew D. Smith

**Affiliations:** 1 EaStCHEM, School of Chemistry, University of St Andrews, St Andrews, UK; 2 Key Laboratory of Applied Surface and Colloid Chemistry, Ministry of Education, and School of Chemistry and Chemical Engineering, Shaanxi Normal University, Xi’an, China; 3 Department of Chemistry, University of Bath, Claverton Down, Bath, UK; 4 Department of Computer Science, University of Bath, Claverton Down, Bath, UK

**Keywords:** Stereochemistry, Synthetic chemistry methodology, Synthetic chemistry methodology

## Abstract

The catalytic enantioselective [1,2]-Wittig rearrangement of allylic ethers constitutes a recognized synthetic challenge as it is traditionally considered to arise from a non-concerted reaction pathway via formation and recombination of radical pairs. Here we show a catalytic enantioselective solution to this challenge, demonstrating that [1,2]-Wittig products are generated via an alternative reaction cascade to traditional dogma. The developed process employs a chiral bifunctional iminophosphorane catalyst to promote an initial enantioselective [2,3]-sigmatropic rearrangement. A subsequent base-promoted, stereoconvergent, fragmentation–recombination process that proceeds with high enantiospecificity and retention of configuration, formally equivalent to a Woodward–Hoffmann forbidden thermal [1,3]-sigmatropic rearrangement, generates [1,2]-Wittig products in up to 97:3 enantiomeric ratio. Supported by extensive quantum chemistry calculations, this chirality transfer process will have broad implications for fundamental stereocontrol in organic transformations.

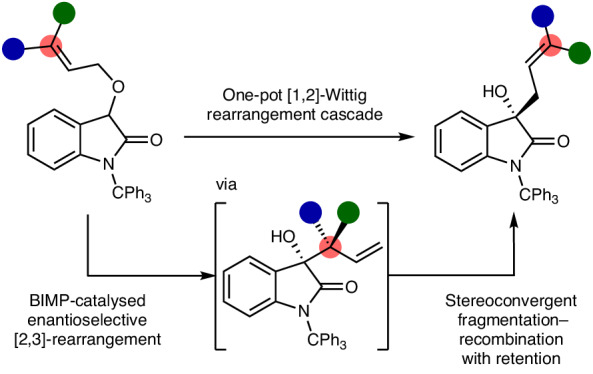

## Abstract (web summary)

The [1,2]-Wittig rearrangement of allylic ethers is traditionally considered to proceed via formation and recombination of radical pairs. Now it has been shown that an alternative reaction cascade, involving initial enantioselective [2,3]-rearrangement followed by base-promoted anionic fragmentation–recombination that proceeds with high enantiospecificity, allows a catalytic enantioselective [1,2]-Wittig process.

## Main

**Fig. 1:**
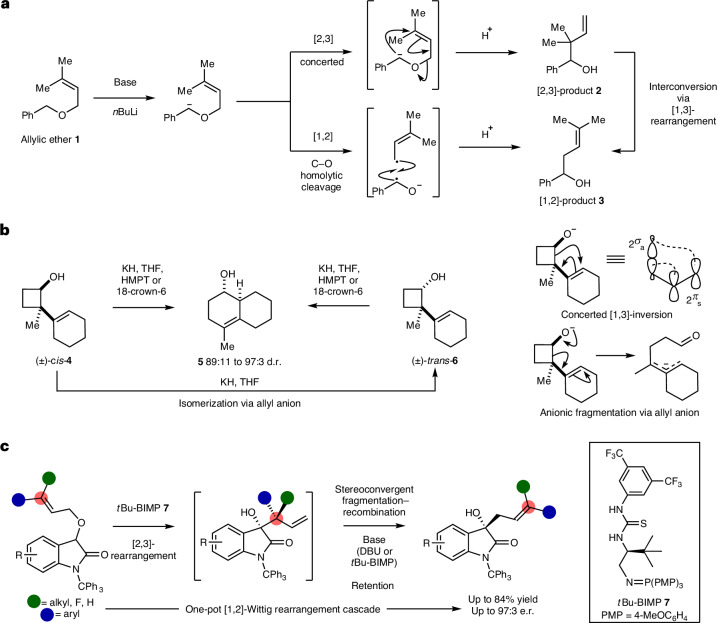
Overview of [2,3]-, [1,2]- and [1,3]-rearrangement pathways. **a**, Traditional mechanism and dichotomy between [2,3]- and [1,2]-Wittig rearrangements. **b**, Stereochemical ambiguity of [1,3]-rearrangement reactions via concerted or fragmentary pathways. **c**, This work: the catalytic enantioselective [1,2]-Wittig rearrangement cascade. HMPT, hexamethylphosphoric triamide; THF, tetrahydrofuran.

Sigmatropic rearrangements are useful and reliable atom-economic reactions, with their ability to form carbon–carbon and carbon–heteroatom bonds through well-defined and predictable transition states^1^ making these processes attractive for the synthesis of complex targets^2^. Among this broad set of reaction processes, [2,3]- and [1,2]-sigmatropic rearrangements are of synthetic and mechanistic significance^3,4^. The rearrangement of allylic ethers under basic reaction conditions typically leads to product mixtures proposed to arise from the thermally allowed concerted [2,3]-sigmatropic Wittig rearrangement, alongside a competitive non-concerted [1,2]-Wittig rearrangement generally thought to arise from homolytic fragmentation of the anionic intermediate to form a geminate radical pair and their subsequent recombination^5,6^ (Fig. 1a). As representative examples, both Rautenstrauch^7^ and Baldwin^8^ have shown that treatment of benzyl allyl ether **1** with *n*BuLi gives rise to a mixture of [2,3]- and [1,2]-products **2** and **3**, respectively, with increased [1,2]-product observed at higher temperatures. Although not widely recognized, sporadic control reactions have demonstrated the feasibility of converting [2,3]-Wittig products to [1,2]-Wittig products (formally via a [1,3]-rearrangement), although the generality, mechanism and configurational consequence have not been established^9–15^. The concerted or dissociative (via ionic or radical intermediates) nature of both the [1,2]- and [1,3]-processes has been much debated. For example, Danheiser considered a concerted [1,3]-pathway to account for the inversion observed in the ring expansion of *cis*-cyclobutanol **4**^16^. However, Gajewski^17^ and Cohen^18^ both postulated a non-concerted fragmentation pathway via an intermediate allylic anion that accounts for the observed in situ isomerism of *cis*-**4** to *trans*-**6**, and that use of enantiomerically pure *cis*-**4** or *trans*-**6** leads to racemic product **5** (Fig. 1b)^19^. Applying the Woodward–Hoffmann rules indicates that a concerted [1,2]-rearrangement is forbidden, while a thermal [1,3]-rearrangement is symmetry allowed but geometrically challenging, with a suprafacial carbon shift expected to proceed with inversion of configuration at the oxygen-bearing carbon^1^. Interestingly, Houk has previously shown that anionic Cope and amino-Cope reactions proceed through a stepwise dissociation–recombination process^20^, consistent with competitive non-concerted [1,3]-rearrangements observed in related systems^21,22^. Given the mechanistic ambiguity surrounding these processes the enantioselective [1,2]-Wittig rearrangement of allylic ethers is a recognized challenge and is currently unknown despite its synthetic potential^23^.

In this Article, the catalytic enantioselective [1,2]-Wittig rearrangement of allylic ethers is developed (up to 97:3 enantiomeric ratio (e.r.)) and is shown to proceed through a cascade process consisting of an initial enantioselective [2,3]-rearrangement (up to >99:1 e.r.) promoted by a bifunctional iminophosphorane (BIMP) catalyst. The resultant tertiary carbinol bearing an α-branched allylic substituent is transformed to the linear [1,2]-Wittig isomeric product with retention of configuration at the oxygen-bearing carbon (equivalent to a Woodward–Hoffmann forbidden [1,3]-sigmatropic rearrangement) through a dissociative intramolecular fragmentation–recombination event with high enantiospecificity (Fig. 1c). Substitution reactions that proceed with retention of configuration are rare, although recognized for alcohols via an S_N_i mechanism that proceeds via contact ion-paired intermediates^24–26^. Traditionally the stereospecificity of nucleophilic substitution processes leads to inversion of configuration in S_N_2 reaction processes at secondary centres and partial or complete racemization in S_N_1 processes at tertiary centres. However, recent advances have showcased stereospecific substitution at tertiary and even quaternary centres in which stereochemical information is conferred despite ionization of a substrate^27–32^. In this context, the high enantiospecificity of the observed chirality transfer protocol that leads to [1,2]-products with retention of configuration, while proceeding through an ionic fragmentation and recombination process, holds promise for the elucidation of alternative reaction pathways for generating chiral products with high enantioselectivity.

## Reaction development

**Fig. 2:**
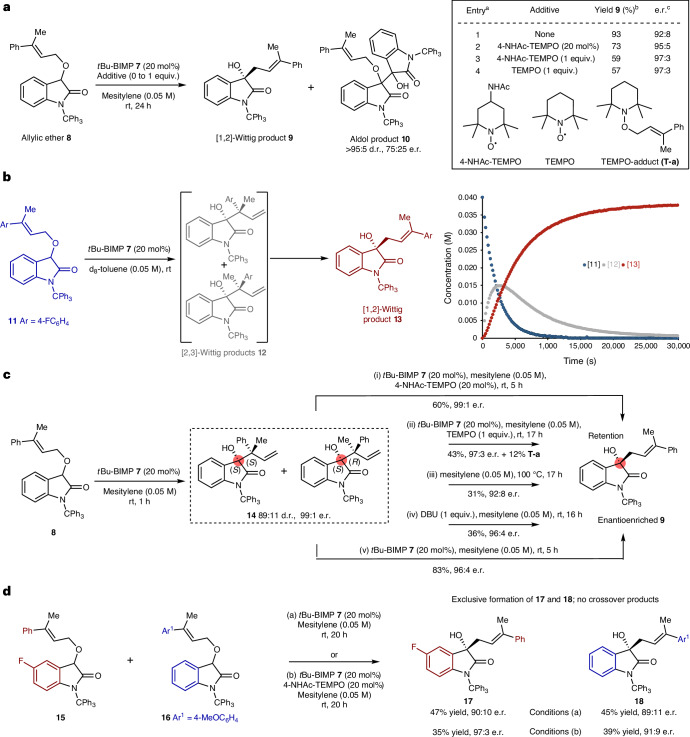
Demonstration of [1,2]-Wittig cascade and intermediate validation. **a**, Initial observations of [1,2]-Wittig reaction products and additive effect. **b**, In situ reaction monitoring of [1,2]-Wittig cascade by ^1^H NMR spectroscopy. [**12**] refers to combined concentration of two diastereoisomers. **c**, Control experiments validate [2,3]-Wittig products as intermediates in cascade. **d**, Crossover experiments indicate an intramolecular rearrangement process. ^a^Reaction performed on 0.1 mmol scale. ^b^All yields are isolated. ^c^Determined by high-performance liquid chromatography (HPLC) analysis on a chiral stationary phase. rt, room temperature.

Building upon the observation that disubstitution at the allylic ether terminus typically leads to increased preference for [1,2]-rearrangement products^14^, the use of BIMPs as organocatalysts to promote the enantioselective [1,2]-rearrangement process was considered. Originally developed by Dixon, BIMPs have shown widespread use in a plethora of stereoselective transformations^33^, possessing a Brønsted superbasic iminophosphorane with a hydrogen-bond donor to assert stereocontrol. Rearrangement of an allylic ether upon an oxindole skeleton was chosen, given the prevalence of this motif in natural products and bioactive molecules, as well as expected acidity. Following initial screening of the effect of N-substituent (*N*-Me, *N*-Bn, *N*-trityl), BIMP catalyst, solvent and temperature variation (see Supplementary Information Section 2) using *N*-trityl-substituted allylic ether **8** and *t*Bu-BIMP catalyst **7** showed that rearrangement to **9** in mesitylene led to selective formation of the [1,2]-product in excellent yield and promising enantioselectivity after 24 h (Fig. 2a; 92:8 e.r.). As [1,2]-Wittig products are traditionally expected to be generated via a radical recombination mechanism, the effect of adding 20 mol% of 4-acetamido-2,2,6,6-tetramethyl-1-oxopiperidinium (4-NHAc-TEMPO) as an additive was probed. Formation of the [1,2]-product was not significantly inhibited, giving **9** in 73% yield and improved 95:5 e.r., with no 4-NHAc-TEMPO adducts observed^34^. The mass balance consisted of the aldol side product **10** (>95:5 diastereomeric ratio (d.r.), 75:25 e.r.) that was isolated in 5% yield; addition of 1.0 equivalent of 4-NHAc-TEMPO was also tested, affording **9** in a further reduced 59% yield but enhanced 97:3 e.r. Control experiments indicated that taking a 1:1 mixture of allylic ether **8** and *N*-tritylisatin with *t*Bu-BIMP **7** gave aldol product **10** in 71% yield (>95:5 d.r., 75:25 e.r.), consistent with in situ formation of *N*-tritylisatin derivative in the presence of 4-NHAc-TEMPO. Further experiments showed that addition of 1.0 equivalent of TEMPO led to **9** in 57% isolated yield (97:3 e.r.) with 10% of TEMPO-allyl adduct (**T-a**) isolated as well as 11% *N*-tritylisatin. Intrigued by these observations, in situ temporal reaction analysis monitored consumption of allylic ether **11** (40 mm) upon treatment with *t*Bu-BIMP **7** (20 mol%) to give [1,2]-Wittig product **13** in d_8_-toluene using ^1^H NMR spectroscopy (Fig. 2b and Supplementary Information Section 5.1). The rearrangement showed a first-order consumption in substrate **11** (which was racemic throughout the reaction process), with a transient mixture of diastereoisomeric [2,3]-rearrangement products **12** detected (*δ*_H_ = 5.15 and 4.87 ppm) that accumulated to a maximum concentration of ≈15 mm and was subsequently transformed into the [1,2]-rearrangement product **13**, consistent with **12** being an intermediate in the generation of **13**. On a synthetic scale, stopping the reaction of allylic ether **8** after 1 h gave, at 75% conversion, a 63:37 mixture of [2,3]-product **14** and [1,2]-product **9** (96:4 e.r.). Purification gave **14** (89:11 d.r., both diastereoisomers 99:1 e.r.) in 21% yield whose absolute configuration was determined by *N*-trityl deprotection and subsequent single-crystal X-ray diffraction (Supplementary Information Section 6). The absolute configuration within **9** was confirmed by chemical synthesis (Supplementary Information Section 8.8), indicating stereoconvergence and retention of configuration at C(3) in the rearrangement of diastereoisomers **14** to **9**. Separate control experiments validated the [2,3]-rearrangement products **14** as intermediates to the [1,2]-product **9** (Fig. 2c). Treating **14** (89:11 d.r.) with *t*Bu-BIMP **7** and 4-NHAc-TEMPO for 5 h gave the [1,2]-product **9** in 60% yield (99:1 e.r.) alongside 10% of *N*-tritylisatin, while treatment with *t*Bu-BIMP **7** and TEMPO gave the [1,2]-product **9** in 43% yield (97:3 e.r.) alongside 12% of TEMPO-adduct (**T-a**) and 15% of *N*-tritylisatin. Treatment with *t*Bu-BIMP **7** alone gave **9** in 83% yield (96:4 e.r.) with 12% of *N*-tritylisatin observed, while the use of achiral base 1,8-diazabicycloundec-7-ene (DBU) on **14** also promoted rearrangement, but to moderate 50% conversion, giving **9** in 36% yield but with high enantioselectivity (96:4 e.r.). This is consistent with DBU or *t*Bu-BIMP **7** acting as a base and not notably influencing enantiospecificity in this [1,3]-rearrangement process. Consistent with this observation, monitoring the conversion of **14** (89:11 d.r.) to **9** upon treatment with racemic or enantiopure BIMP derivatives did not lead to significant rate differences (Supplementary Information Section 5.2) implying no matched and mismatched reactant combinations. Taken together these experiments are consistent with the addition of 4-NHAc-TEMPO or TEMPO leading to enhanced enantiospecificity in the base-promoted stereoconvergent [1,3]-rearrangement. This is consistent with selective fragment trapping within this process leading to enhanced selectivity, with the origin of this phenomenon discussed in ‘Mechanistic analysis’. The [2,3]-product **14** could also be transformed into the [1,2]-Wittig product **9** when heated at 100 °C without the addition of base, albeit with reduced yield (31%) and enantioselectivity (92:8 e.r.). Crossover experiments (Fig. 2d) using a 50:50 mixture of ethers **15** and **16** either with *t*Bu-BIMP **7** alone (conditions a), or with the addition of 4-NHAc-TEMPO (conditions b), resulted in only [1,2]-products **17** and **18**, consistent with an intramolecular process in operation, with enhanced product enantioselectivity again observed in the presence of 4-NHAc-TEMPO.

Having identified a viable reaction pathway, the scope of the enantioselective [1,2]-Wittig cascade was examined (Table 1). Changing the C(3′)-alkyl substituent from methyl to ethyl was tolerated, giving **19**, while using a C(3′)-cyclopropyl-substituted allylic ether as a radical probe generated only [1,2]-rearrangement product **20** (40% yield, 96:4 e.r.) with the cyclopropyl ring intact. Although indicative, the absence of ring-opened products does not exclude a radical mechanism since in-cage carbon–carbon radical recombination (rate constant typically estimated to be >10^11^ s^−1^) is considered much faster than ring opening of a cyclopropyl methyl radical (*k* = 1.3 × 10^8^ s^−1^)^35^. Increasing the steric hindrance through incorporation of a C(3′)-phenyl substituent resulted in moderate conversion (Supplementary Information Section 4). This necessitated changing the *N*-trityl substituent to an *N*-benzyl for increased reactivity, giving **21** in 61% yield but reduced product enantioselectivity (73:27 e.r.), consistent with screening studies that necessitated *N*-trityl substitution for optimal enantioselectivity (Supplementary Information Section 2). A limitation of this process showed that both a (*Z*)-configured allylic ether and a dimethyl terminal allylic ether returned only starting material under the reaction conditions. Variation of the C(3′)-aryl substituent showed that the incorporation of halogens (4-F, 4-Cl and 4-Br), electron-withdrawing (4-CF_3_) as well as electron-donating (4-Ph, 4-Me, 4-MeO) substituents were tolerated, giving the [1,2]-rearrangement products **13**, **18**, **22**–**26** in high yields and enantioselectivity. The incorporation of 3-Me and 2-Me substituents (**27**, **28**) was also tolerated, although with lower yields for the 2-Me-substituted example. In addition, 2-naphthyl-, thien-2-yl- and thiazol-2-yl-substituted ethers all afforded the corresponding [1,2]-Wittig products **29**–**31** in 71% to 82% yields with 93:7 to 94:6 e.r. Substituent variation within the 4-, 5- and 6-positions of the oxindole included the incorporation of halogen (5-F, 5-Cl, 5-I, 6-Br, 6-Cl), electron-withdrawing (5-O_2_N) and electron-donating (5-Me, 5-MeO) substituents that gave the corresponding products **17**, **32**–**39** in 67% to 80% yield and up to 96:4 e.r. While *N*-tritylation of 7-chloroisatin was unsuccessful, the *N*-benzyl analogue was prepared and tested, giving **40** in 81% yield but reduced enantioselectivity (73:27 e.r.).

**Table 1:**
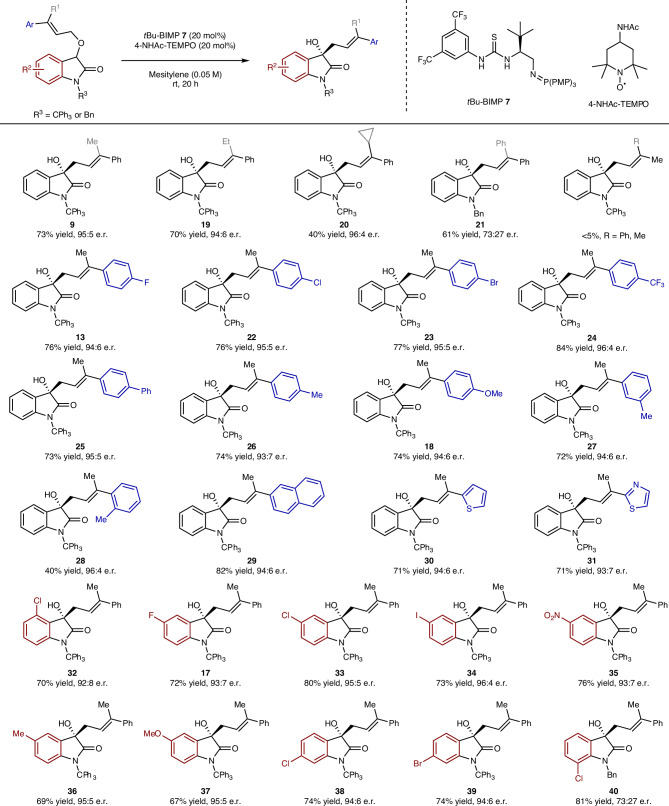
Substrate scope and limitations of the [1,2]-Wittig rearrangement cascade

All e.r. values determined by HPLC analysis on a chiral stationary phase; all yields are isolated yields.

**Fig. 3:**
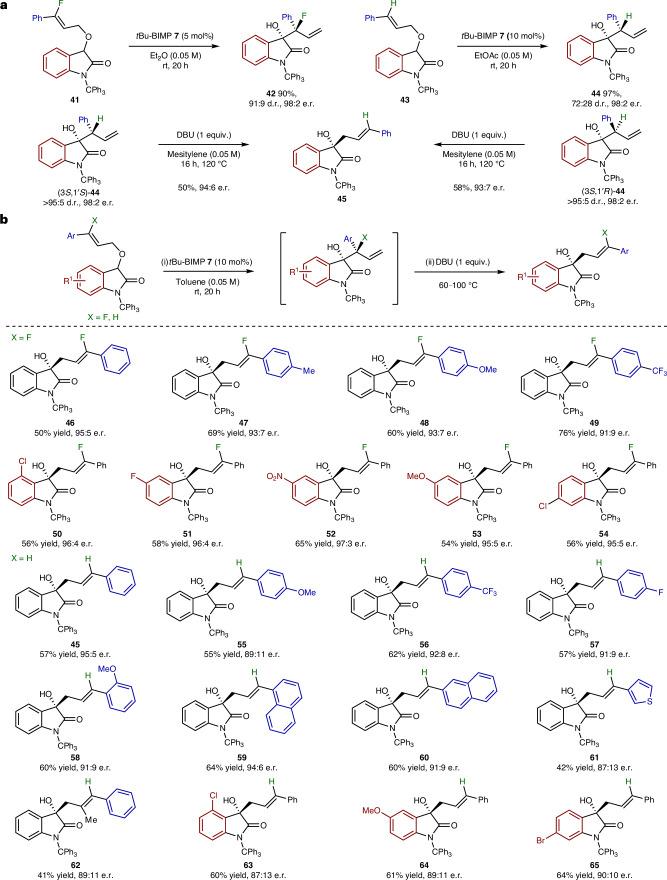
Effect of C(3′)-substitution on [2,3]- and [1,2]-reaction pathways. **a**, Observations with C(3′)-F or C(3′)-H substitution; selective formation of [2,3]-rearrangement product and temperature required to promote [1,3]-rearrangement. **b**, Substrate scope and limitations of the telescoped [1,2]-Wittig process; all e.r. values determined by HPLC analysis on a chiral stationary phase; all yields are isolated yields; *t*Bu-BIMP **7** (20 mol %) used to prepare **62**.

To further probe the generality of this transformation, the effect of incorporating C(3′)-F or C(3′)-H substituents within the allylic ether terminus was investigated (Fig. 3a). In contrast to the parent C(3′)-methyl series, treatment of **41** and **43** with *t*Bu-BIMP **7** at room temperature gave exclusively the corresponding [2,3]-rearrangement products **42** (91:9 d.r., 98:2 e.r.) and **44** (72:28 d.r., 98:2 e.r.) with no formation of the [1,2]-product. To convert these enantioenriched [2,3]-products to the corresponding [1,2]-Wittig products increased reaction temperatures (≥100 °C for **42** and **44**) and the addition of stoichiometric DBU (1 equiv.) was required. For example, stereoconvergence of the separable diastereoisomers (3*S*,1′*S*)- and (3*S*,1′*R*)-**44** upon treatment with DBU in mesitylene at 120 °C was observed, with both giving (*E*,*S*)-[1,2]-Wittig product **45** in 94:6 and 93:7 e.r., respectively. Solvent polarity has a substantial effect upon the enantiospecificity of the [1,3]-rearrangement at these increased reaction temperatures (Supplementary Information Section 3) with highest product enantioselectivity observed in solvents of low polarity (toluene and mesitylene) rather than polar solvents (DMF or MeCN). Rearrangement with retention of configuration is still observed, although addition of 4-NHAc-TEMPO or TEMPO does not lead to increased product e.r. in this series (Supplementary Information Section 3). Having demonstrated that high temperatures are required to promote the [1,3]-rearrangement of the initially formed [2,3]-products, a telescoped process to allow one-pot access to [1,2]-Wittig products was developed that utilized toluene as a solvent (Fig. 3b). Treatment of a range of allylic ethers with *t*Bu-BIMP **7** promoted enantioselective [2,3]-rearrangement, which was followed by the addition of DBU (1 equiv.) and heating to between 60 °C and 100 °C. Following this procedure, in the C(3′)-F series, inclusion of Ph-, 4-MeC_6_H_4_-, 4*-*MeOC_6_H_4_- and 4-F_3_CC_6_H_4_-substituted allylic ethers, as well as 4-Cl, 5-F, 5-NO_2_, 5-MeO and 6-Cl substituents within the oxindole were tolerated, giving the corresponding [1,2]-Wittig products (**46**–**54**) with good to excellent enantioselectivity (91:9 to 97:3 e.r.). In the C(3′)-H series, variation of the aryl substituent within the allylic ether showed that Ph-, 4*-*MeOC_6_H_4_, 4-F_3_CC_6_H_4_, 4-FC_6_H_4_, 2-MeOC_6_H_4_, 1-naphthyl and 2-naphthyl substitution, heteroaromatic 3-thienyl and C(2′)-methyl substitution, alongside 4-Cl, 5-OMe and 6-Br substituents within the oxindole were tolerated, allowing the formation of enantioenriched [1,2]-Wittig rearrangement products **45**, **55**–**65** (87:13 to 95:5 e.r.). Notably, lower product yields (41% to 73%) were observed in this one-pot process than noted in Fig. 3, reflecting the propensity for competitive decomposition at the elevated reaction temperatures required to promote the [1,3]-rearrangement.

## Mechanistic analysis

**Fig. 4:**
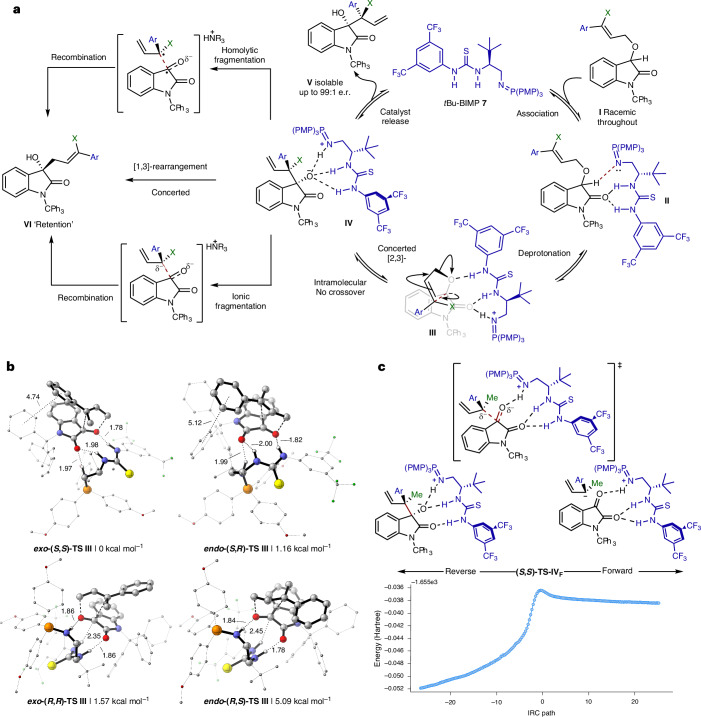
Catalytic cycle and computational analysis. **a**, Plausible mechanisms considered for the [1,2]-Wittig cascade. **b**, Lowest energy [2,3]-rearrangement TSs (M06-2X/Def2-TZVPP/IEFPCM(mesitylene)//ONIOM(M06-2X/6-31+G(d):AM1)) leading to the formation of the four possible stereoisomers; images were created in CYLview20^63^. Relative free energies are listed below each structure. Distances in Å. **c**, Intrinsic reaction coordinate (IRC) plot for the [1,3]-fragmentation TS **(*S***,***S*)**-**TS IV**_**F**_ (ONIOM(M06-2X/6-31+G(d):AM1)), (Ar = 4-FC_6_H_4_).

Having demonstrated the scope of this transformation, consideration to the mechanism of this cascade was given. Initial association of *t*Bu-BIMP **7** to substrate **I** by hydrogen bonding is assumed to direct deprotonation of the allylic ether–BIMP complex **II** to give **III**, with subsequent concerted [2,3]-sigmatropic rearrangement giving **IV** with high stereoselectivity (Fig. 4a). Protonation and catalyst release gives isolable **V**. A subsequent base-promoted (*t*Bu-BIMP or DBU), anion-accelerated [1,3]-rearrangement from **IV**^36^, which proceeds with retention of configuration at the carbinol centre, generates [1,2]-Wittig product **VI**. Computational methods were used to gain mechanistic insight into the [2,3]- and [1,3]-rearrangement processes. Eight binding modes between [*t*Bu-BIMP-H]^+^ and the substrate were considered (Supplementary Fig. 19) and conformationally searched using CREST (GFN2-xTB; Ar = Ph, X = Me)^37–39^. The lowest energy conformer from each search was then optimized in Gaussian 16^40^ using ONIOM^41,42^ to reduce the computational complexity for this large system. The peripheral components of both catalyst and substrate were treated with semi-empirical quantum mechanics and placed in the low layer, while the atoms involved in bond breaking and forming were treated with density functional theory (DFT). Full DFT single point energies were performed on all stationary points (Supplementary Information Section 7). These calculations were conducted on substrate **8** (Ar = Ph, X = Me; Fig. 4a) in the presence of catalyst **7** unless otherwise stated (Fig. 2c). Previous studies have shown that there is good agreement between ONIOM and full DFT calculations for other organocatalytic systems^43–49^.

## [2,3]-Sigmatropic rearrangement

To study the origins of stereoselectivity, transition states (TSs) leading to each of the four possible stereoisomers for the BIMP-catalysed [2,3]-sigmatropic rearrangement were located (Ar = Ph, X = Me). Consistent with experimental results (Fig. 2c), the calculated lowest energy *exo*-TS forms the (*S*,*S*)-stereoisomer, with the difference in free energy between this and the lowest energy *exo*-(*R*,*R*)-TS (1.57 kcal mol^−1^; Fig. 4b) resulting in a computed e.r. of 93:7^50^. The diastereomeric ratio computed from the ***exo*-(*S***,***S***)- and ***endo*-(*S***,***R*)**-**TS III** energies (ΔΔ*G*^‡^ = 1.16 kcal mol^−1^) is 86:14. These ratios are consistent with the experimentally observed levels of stereoselectivity in the [2,3]-sigmatropic rearrangement. To gain insight into the origins of enantioselectivity, the structures of ***exo*-(*S***,***S*)**- and ***exo***-**(*R***,***R*)-TS III** were compared, revealing key differences in hydrogen bonding capability between [*t*Bu-BIMP-H]^+^ and the substrate. In ***exo***-**(*S***,***S*)-TS III**, three stabilizing NH···O hydrogen bonds from the catalyst to the substrate oxygen atoms are observed, while in ***exo***-**(*R***,***R*)-TS III**, only two NH···O hydrogen bonds are seen alongside an unusual, longer and weaker NH···C interaction with a reacting carbon atom (Fig. 4b and Supplementary Fig. 21). The origins of diastereoselectivity can be understood by comparison of ***exo***-**(*S***,***S*)-TS III** and ***endo***-**(*S***,***R*)-TS III**. A shorter distance between the centroids of the allylic aryl substituent and an *N*-trityl Ph substituent in ***exo***-**(*S***,***S*)-TS III** (4.74 Å) relative to ***endo***-**(*S***,***R*)-TS III** (5.12 Å) is observed, suggesting a more favourable T-shaped π-stacking interaction, leading to the preference for the (*S*,*S*)-stereoisomer^51^ (Fig. 4b and Supplementary Fig. 22).

## [1,3]-Rearrangement

Computational analysis subsequently studied the feasibility of a concerted rearrangement or a stepwise fragmentation followed by a recombination process (Fig. 4a) for the onwards reaction of (***S***,***S*)-IV** (Ar = Ph, X = Me). Although examples of [1,3]-sigmatropic rearrangements are reported^52–58^, despite extensive efforts it was not possible to locate concerted TSs for this [1,3]-rearrangement process, even with constraints between the bond-forming and bond-breaking atoms; some unconstrained attempts resulted in geometries that resembled **TS IV** (fragmentation). Optimization of fragmentation TSs was possible but attempts to locate a TS that connects the fragment complex and the recombination product were all unsuccessful. An energy scan starting from the recombination product up to a fragment complex (Supplementary Fig. 23) showed no peak in the potential energy surface between 1.6 and 3.5 Å, which suggests that **VI** forms via a near-barrierless recombination event. Reoptimization of the lowest energy **(*S***,***S*)-TS IV** conformer with unrestricted methods showed no appreciable spin density on any atom, which is consistent with an ionic rather than a homolytic fragmentation.

The lowest energy conformer of **(*S***,***S*)-IV** and **(*S***,***S*)-TS IV** were then reoptimized with Ar = 4-FC_6_H_4_, X = Me such that the calculated reaction barrier could be directly compared to that derived from in situ reaction monitoring (Fig. 2b and Supplementary Information Section 5.4 and 7.3). The intrinsic reaction coordinate data for the reoptimized 4-FC_6_H_4_ TS (**(*S***,***S*)-TS IV**_**F**_) is shown in Fig. 4c (for optimized structures of **(*S***,***S*)-IV**_**F**_, **(*S***,***S*)-TS IV**_**F**_ and fragment complex see Supplementary Fig. 24). The computed barrier for the fragmentation step of this [1,3]-rearrangement (Δ*G*^‡^_comp_ = 20.97 kcal mol^−1^) closely matches that derived from kinetic fitting of the reaction profile generated via in situ monitoring of **11**→**12**→**13** (Δ*G*^‡^_exp_ = 21.90 kcal mol^−1^; see Fig. 2b and Supplementary Information Section 5.4).

**Fig. 5:**
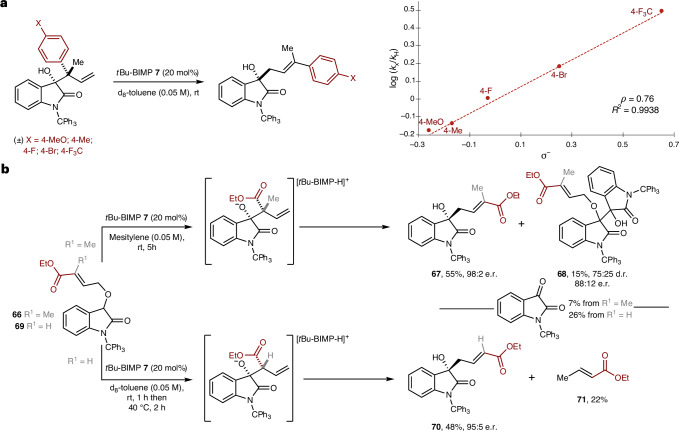
Mechanistic experiments. **a**, Hammett analysis of the [1,3]-rearrangement. **b**, Effect of anion-stabilizing ester substituents.

To experimentally probe the validity of this proposed fragmentation–recombination pathway, taking isolated racemic [2,3]-rearrangement products that differed in the electronic effect of C(1′)-aryl substituents with *t*Bu-BIMP **7** showed that inclusion of electron-withdrawing substituents led to enhanced reaction rates. Hammett analysis (Fig. 5a and Supplementary Information Section 5.2) revealed a *ρ* value of +0.76 when plotted against the substituent constant *σ*^−^. Although small, this *ρ* value is consistent with the build-up of partial negative charge within the rate-limiting TS of the reaction and the proposed anionic fragmentation. Notably, no significant difference in reaction rate was observed when using BIMP catalysts with either racemic or enantioenriched [2,3]-rearrangement products (Supplementary Information Section 5.3). Building on this observation, the effect of incorporating an electron-withdrawing ester substituent at C(3′) within the allylic ether terminus was investigated (Fig. 5b and Supplementary Information Section 3). Treatment of **66** (R^1^ = Me) with *t*Bu-BIMP **7** at room temperature promoted initial enantioselective [2,3]-rearrangement, leading to [1,2]-product **67** in 55% yield (98:2 e.r.) alongside aldol product **68** (15%, 75:25 d.r., 88:12 e.r.) and *N*-tritylisatin (7%). Notably, treatment of **69** (R^1^ = H) with *t*Bu-BIMP **7** at room temperature for 1 h, followed by warming at 40 °C for 2 h led to facile fragmentation, giving [1,2]-product **70** in 48% yield (95:5 e.r.) in conjunction with ethyl crotonate (22% yield) and *N*-tritylisatin (26%). The generation of ethyl crotonate **71** and *N*-tritylisatin is consistent with the ester substituent facilitating anionic fragmentation from an initially formed [2,3]-product, with protonation and isomerization giving **71**. Building on these observations, the observed [1,3]-rearrangement with retention of configuration and high enantiospecificity is consistent with anionic fragmentation from an alkoxide·[*t*Bu-BIMP-H]^+^ complex generating an intimate ion pair consisting of an allylic anion and *N*-tritylisatin·[*t*Bu-BIMP-H]^+^ (Fig. 4c). Recombination at the terminal allylic position upon the *Re*-face of the isatin occurs at a faster rate than ion pair dissociation, or conformational change and bond rotation, to allow *Si*-face addition of the isatin that would lead to reduced product enantioselectivity. Given this anionic fragmentation–recombination mechanism the observation of improved product enantioselectivity on addition of TEMPO derivatives (Fig. 2a) is ascribed to initial single-electron transfer oxidation of an anionic carbanion intermediate by TEMPO as it dissociates from the intimate ion pair, followed by trapping with a second equivalent of TEMPO, consistent with observations by Studer and co-workers alongside others^59–62^. Alternative mechanisms involving radical fragmentation and subsequent recombination, or anionic fragmentation and protonation, followed by C-H abstraction by TEMPO and subsequent radical recombination were ruled out (Supplementary Information Section 3).

## Summary

To conclude, a cascade process that allows the catalytic enantioselective [1,2]-Wittig rearrangement of allylic ethers has been developed, providing enantioenriched homoallylic tertiary alcohols with high enantioselectivity. Contrary to existing dogma, mechanistic studies and quantum chemistry calculations strongly support a cascade mechanism for this process that involves an initial enantioselective [2,3]-rearrangement of the allylic ether catalysed by a chiral *t*Bu-BIMP catalyst. This is followed by a base-promoted anionic fragmentation–recombination pathway (base, *t*Bu-BIMP or DBU) that proceeds with high levels of enantiospecificity even at elevated reaction temperatures. Substitution of the allylic fragment markedly affects the effectiveness of the fragmentation–recombination pathway, with anion-stabilizing ester substituents facilitating fragmentation, and with C(3′)-alkyl substitution leading to enhanced rates of [1,2]-product formation over C(3′)-F or C(3′)-H substitution. Given the importance of understanding fundamental reaction processes that lead to stereochemical control, the enantiospecificity observed in this non-concerted pathway will have broader implications for a plethora of other related synthetic transformations.

## Methods

Typical procedures for the [1,2]-rearrangement cascade at room temperature and upon heating are exemplified below.

### Procedure for BIMP-catalysed enantioselective [1,2]-rearrangement reaction at room temperature

The allylic ether substrate **8** (52.1 mg, 0.1 mmol), *t*Bu-BIMP **7** (14.7 mg, 0.02 mmol) and 4-NHAc-TEMPO (4.3 mg, 0.02 mmol) were added to a flame-dried Schlenk tube and the tube was flushed with N_2_ three times. Mesitylene (2 ml, 0.05 M) was added through the septum under a positive pressure of N_2_. The reaction was stirred at room temperature for 16 h until completion as indicated by thin-layer chromatography analysis. The reaction mixture was concentrated under reduced pressure to give the crude product, which was purified by flash column chromatography (silica gel:eluent hexane:EtOAc = 4:1→3:1) to afford **9** (38.0 mg, 73%) as a colourless amorphous solid. The e.r. was obtained by high-performance liquid chromatography (chiral stationary phase) of the purified products (Daicel CHIRALCEL OD-H column (10% *i*PrOH in hexane; 1.0 ml min^−1^; *T* = 30 °C; 211 nm); retention time (minor) = 7.0 min, retention time (major) =8.7 min; 5:95 e.r.).

### Procedure for BIMP-catalysed enantioselective [1,2]-rearrangement reaction cascade involving heating with DBU

The allylic ether substrate **43** (101.5 mg, 1.0 equiv.) and *t*Bu-BIMP **7** (14.8 mg, 10 mol%) were added to a vial before the addition of toluene (4 ml, 0.05 M). The mixture was stirred at room temperature for 24 h, then DBU (28 μl, 1.0 equiv.) was added, and the mixture was then heated to 100 °C and stirred for 16 h, then quenched by addition of saturated aqueous NH_4_Cl and diluted with EtOAc. The phases were separated, and the aqueous phase was extracted with EtOAc (×2). The combined organic phases were washed with brine, dried (MgSO_4_) and concentrated under reduced pressure. The residue was purified by flash column chromatography (silica gel:eluent 80:20→75:25 petrol:EtOAc) to afford **45** (58 mg, 57%) as a yellow amorphous solid. The e.r. was obtained by high-performance liquid chromatography (chiral stationary phase) of the purified products (Daicel CHIRALPAK AD-H column (10% *i*PrOH in hexane; 1.0 ml min^−1^; *T* = 30 °C; 211 nm); retention time (minor) = 12.5 min, retention time (major) =14.6 min; 5:95 e.r.).

## Online content

Any methods, additional references, Nature Portfolio reporting summaries, source data, extended data, supplementary information, acknowledgements, peer review information; details of author contributions and competing interests; and statements of data and code availability are available at 10.1038/s41557-025-02022-4.

## Supplementary information

Supplementary Information Supplementary Information Sections 1–11 and Figs. 1–486.

## Data Availability

Data are available in the paper and Supplementary Information. The research data supporting this publication can be accessed at 10.17630/5b5778a0-f337-4cbe-b336-c2afac22693b and 10.17630/6424d442-e1bc-4834-9456-6cfb7296580f: data underpinning ‘The Catalytic Enantioselective [1,2]-Wittig Rearrangement Cascade of Allylic Ethers’. University of St Andrews Research Portal; PURE ID: 295983644 and 320215223. Gaussian files plus three sets of in situ reaction monitoring data are openly available in a dataset for ‘The Catalytic Enantioselective [1,2]-Wittig Rearrangement Cascade of Allylic Ethers’ in the University of Bath Research Data Archive at 10.15125/BATH-01337. The supplementary crystallographic data for this paper are available free of charge from the Cambridge Crystallographic Data Centre (CCDC) at accession numbers 2305636 (for compound **S24**) and 2305637 (for compound **S25**).

## Code availability

A Python script that fits the rate constants of a first-order kinetics model to the experimental data is openly available in a dataset for ‘The Catalytic Enantioselective [1,2]-Wittig Rearrangement Cascade of Allylic Ethers’ in the University of Bath Research Data Archive at 10.15125/BATH-01337.
