# Supplementary material for: The catalytic enantioselective [1,2]-Wittig rearrangement cascade of allylic ethers
Source: Nat Chem. 2026 Jan 6;18(4):800–9. doi: 10.1038/s41557-025-02022-4 (PMC13061636; doi:10.1038/s41557-025-02022-4)
Supplement: Supplementary file 1 — Supplementary Information Sections 1–11 and Figs. 1–486. [file 41557_2025_2022_MOESM1_ESM.pdf]

nature chemistry

Supplementary information https://doi.org/10.1038/s41557-025-02022-4

The catalytic enantioselective [1,2]-Wittig rearrangement cascade of allylic ethers

In the format provided by the authors and unedited

Supplementary Materials for

The Catalytic Enantioselective [1,2]-Wittig Rearrangement Cascade of Allylic Ethers

Tengfei Kang[1,2], Justin O'Yang,[1] Kevin Kasten,[1] Samuel S. Allsop,[3] Toby Lewis-Atwell,[3,4] Elliot H. E. Farrar,[3] Martin Juhl,[1] David B. Cordes,[1] Aidan P. McKay,[1] Matthew N. Grayson,[3*] Andrew D. Smith[1*]

[1] EaStCHEM, School of Chemistry, University of St Andrews, North Haugh, St Andrews, KY16 9ST, UK.

[2] Key Laboratory of Applied Surface and Colloid Chemistry, Ministry of Education, and School of Chemistry and Chemical Engineering, Shaanxi Normal University, Xi'an, Shaanxi, China, 710119.

[3] Department of Chemistry, University of Bath, Claverton Down, Bath, BA2 7AY, UK.

[4] Department of Computer Science, University of Bath, Claverton Down, Bath, BA2 7AY, UK.

Corresponding authors: Andrew D. Smith, ads10@st-andrews.ac.uk ;
Matthew N. Grayson, M.N.Grayson@bath.ac.uk

The PDF file includes:

Materials and Methods
Supplementary Text
Figs. S1 to S24
Tables S1 to S11
References

Content

1 General Information 4
1.1 Materials and Methods 4
1.2 General Procedures 6
1.2.1 General Procedure A: Preparation of diazo compounds 6
1.2.2 General Procedure B: Preparation of 3-dissubstituted allylic alcohols 7
1.2.3 General Procedure C: Preparation of 3-fluoro-3-aryl allylic alcohols 8
1.2.4 General Procedure D: Preparation of 3-substitued allylic alcohols 9
1.2.5 General Procedure E: Preparation of (*E*)-1-cyclopropyl-3-phenylbut-2-en-1-ol (SS1) 10
1.2.6 General Procedure F: Preparation of allylic ether by Rh-catalyzed O-H insertion 11
1.2.7 General Procedure G: BIMP-catalyzed enantioselective [1,2]-rearrangement reaction at room temperature 11
1.2.8 General Procedure H: BIMP-catalyzed enantioselective formal [1,2]-rearrangement reaction under heating 12
1.2.9 General Procedure I: Preparation of racemic [1,2]-rearrangement product 13
1.2.10 General Procedure J: Preparation of [2,3]-Wittig rearrangement product ..13
1.2.11 General Procedure K: Determination of the absolute configuration of [1,2]-rearrangement product 15
1.2.12 General Procedure L: Determination of the absolute configuration of 14 and 46 16
1.2.13 General Procedure M: Preparation of BIMPs 16
2 Supplementary data of reaction optimization 17
3 Supplementary data for Control experiments 18
4 Supplementary data: limitations of the reaction 28
5 Supplementary Kinetic studies 29
5.1 Monitoring of the reaction using *in situ* ^{1}H NMR 29
5.2 Hammett plot analysis 42
5.3 Kinetic studies of 14 (89:11 dr) to 9 with racemic or enantiopure BIMPs 45
5.4 Fitting Kinetics Equations 46
6 Supplementary X-ray analysis details 49
7 Supplementary Computational details 51
7.1 Computational details 53
7.1.1 Binding modes 53
7.1.2 [2,3]-Sigmatropic rearrangement additional figures 54
7.2 [1,3]-Rearrangement additional figures 55
7.3 *N*-substituent effects 56
8 Supplementary data for substrates and scope of [1,2]-rearrangement reaction 58
8.1 Supplementary Data for protected isatins 58
8.2 Supplementary Data for diazo compounds 65
8.3 Supplementary Data for allylic alcohols 72
8.4 Supplementary Data for allylic ethers 85

8.5 Supplementary Data for [1,2]-rearrangement products 143
8.6 Supplementary Data for [2,3]-rearrangement products and additional compounds 192
8.7 Supplementary Data for interrupted [1,2]-rearrangement products 213
8.8 Supplementary Data for determination of absolute configuration of [2,3]- and formal [1,2]-rearrangement product 225
9 Supplementary HPLC data of chiral compounds 230
10 Supplementary NMR spectral data of compounds 294
11 Supplementary Reference 694

1 General Information

1.1 Materials and Methods

Reactions involving moisture sensitive reagents were carried out in flame-dried glassware under a nitrogen atmosphere using standard vacuum line techniques. Anhydrous solvents (THF, CH_2Cl_2, Et_2O and toluene) were obtained from an anhydrous solvent system (purified using an alumina column, MBraun SPS-800). Petrol is defined as petroleum ether 40–60 °C. All other solvents and commercial reagents were used as received without further purification unless otherwise stated.

Room temperature (rt) refers to 15–20 °C. Temperatures of 0 °C, –15 °C, and –78 °C were obtained using ice/water, ice/NaCl(s) and CO_2(s)/acetone baths, respectively. Temperatures of 0 °C to –78 °C for overnight reactions were obtained using an immersion cooler (HAAKE EK 90). Unless otherwise stated, reactions involving heating were performed using DrySyn blocks and a contact thermocouple.

Under reduced pressure or 'in vacuo' refers to the use of either a Büchi Rotavapor R200 with a Büchi V-491 heating bath and Büchi V-800 vacuum controller, a Büchi Rotavapor R-210 with a Büchi V-491 heating bath and Büchi V-850 vacuum controller, a Heidolph Laborota 4001 with vacuum controller, an IKA RV10 rotary evaporator with a IKA HB10 heating bath and ILMVAC vacuum controller, or an IKA RV10 rotary evaporator with a IKA HB10 heating bath and Vacuubrand CVC3000 vacuum controller. Rotary evaporator condensers are fitted to Julabo FL601 Recirculating Coolers filled with ethylene glycol and set to –6 °C.

Analytical thin layer chromatography (TLC) was performed on pre-coated aluminium plates (Kieselgel 60 F254 silica). TLC visualisation was carried out with ultraviolet light (254 nm) and/or staining with either aqueous $KMnO_4$ solution, ethanolic phosphomolybdic acid, or ethanolic Vanillin solution followed by heating. Flash column chromatography was performed in glass columns fitted with porosity 3 sintered discs over Kieselgel 60 silica using the solvent system stated. Automated chromatography was performed on Biotage® Selekt using Sfär™ Silica HC D or Biotage® Sfär™ Silica D columns. Melting points were recorded on an Electrothermal 9100 melting point apparatus. Optical rotations were measured on a PerkinElmer Precisely/Model-341 polarimeter operating at the sodium D line with a 100 mm path cell at rt.

Infrared spectra were recorded on a Shimadzu IRAffinity-1 Fourier transform IR spectrophotometer fitted with a Specac Quest ATR accessory (diamond puck). Spectra were recorded of either thin films or solids, with characteristic absorption wavenumbers (ν_{max}) reported in cm^{-1}.

HPLC analyses were obtained on either a Shimadzu HPLC consisting of a DGU-20A5 degassing unit, LC-20AT liquid chromatography pump, SIL-20AHT autosampler, CMB-20A communications bus module, SPD-M20A diode array detector and a CTO20A column oven or a Shimadzu HPLC consisting of a DGU-20A5R degassing unit, LC-20AD liquid chromatography pump, SIL-20AHT autosampler, SPD-20A UV/Vis detector and a CTO-20A column oven. Separation was achieved using either DAICEL S2 CHIRALPAK OD-H column or DAICEL CHIRALPAK AD-H, IB, IC columns using the method stated. HPLC traces of enantiomerically enriched compounds were compared with authentic racemic spectra. Racemic compounds were synthesized under analogous reaction conditions using DBU or Cs_2CO_3 as base.

^{1}H, ^{13}C, ^{19}F nuclear magnetic resonance (NMR) spectra were acquired on either a Bruker Avance 300 (^{1}H 300 MHz; ^{13}C 75 MHz; ^{19}F 282 MHz), Bruker Avance II 400 (^{1}H 400 MHz; ^{13}C 101 MHz; ^{19}F 376 MHz) or a Bruker Avance II 500 (^{1}H 500 MHz, ^{13}C 126 MHz, ^{19}F 470 MHz), spectrometer at ambient temperature in the deuterated solvent stated. All chemical shifts are quoted in parts per million (ppm) and referenced to the residual solvent peak. All coupling constants, *J*, are quoted in Hz. Multiplicities are indicated by: s (singlet), d (doublet), t (triplet), q (quartet), dd (doublet of doublets), dt (doublet of triplets), dq (doublet of quartets), td (triplet of doublets), ddd (doublet of doublet of doublets), ddt (doublet of doublet of triplets) and m (multiplet). The abbreviation Ar is used to denote aromatic, Ph to denote phenyl, Bn to denote benzyl, br to denote broad and app to denote apparent. NMR peak assignments were confirmed using 2D ^{1}H correlated spectroscopy (COSY), 2D $^{1}H-^{13}C$ heteronuclear multiple-bond correlation spectroscopy (HMBC), and 2D $^{1}H-^{13}C$ heteronuclear single quantum coherence (HSQC) where necessary. For diastereomers, not all minor signals have been resolved.

Mass spectrometry (*m*/*z*) data were acquired by either electrospray ionisation (ESI), electron impact (EI), or matrix-assisted laser desorption/ionisation with no matrix (MALDI (no matrix)) at either the University of St Andrews Mass Spectrometry Facility or SIRCAMS at University of Edinburgh.

1.2 General Procedures

1.2.1 General Procedure A: Preparation of diazo compounds

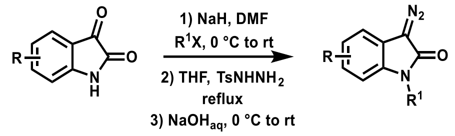

Step I: To a stirred solution of the appropriate isatin (1.0 equiv.) was added sodium hydride (60% dispersion in mineral oil, 1.05 equiv.) slowly in dry DMF (1.0 M) at 0 °C under N_2. The mixture was stirred at this temperature for 15 min. The alkyl halide (1.1–1.3 equiv.) was added during a period of 10 min and the reaction mixture was allowed to warm to rt and stirred overnight. After completion was determined by TLC, the mixture was quenched with saturated NH_4Cl solution and extracted with CH_2Cl_2 (×3). The combined extracts were washed sequentially with 1 M HCl (×2), H_2O (×3), and brine (×2). The organic layer was dried over $MgSO_4$ and concentrated under reduced pressure to afford the crude product which was then either crystallized or purified by flash column chromatography to give the pure protected isatin product.

Step II: To the above obtained isatin (1.0 equiv.) in THF (0.2 M) was added tosylhydrazine (1.1 equiv.). The reaction mixture was heated to reflux and allowed to stir for 2–3 h. After completion was determined by TLC, the reaction mixture was cooled to 0 °C, and aq. NaOH (1.0 equiv., 0.2 M) was added slowly. The reaction mixture was allowed to warm to rt and stirred for 1 h, before another 1.0 equiv. aq. NaOH was added slowly and the reaction mixture was stirred for another 1–2 h. After full transformation of starting material was determined by TLC, the reaction mixture was neutralized by dry ice and extracted with EA (×3), washed with brine, dried over $MgSO_4$, and concentrated under reduced pressure to afford the crude diazo compounds which was further purified by flash column chromatography (eluent: hexane/CH_2Cl_2/EtOAc = 15:5:2).

1.2.2 General Procedure B: Preparation of 3-dissubstituted allylic alcohols

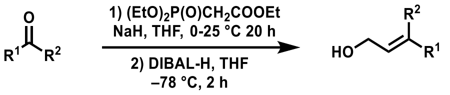

Step I: To a stirred solution of NaH (60% in mineral oil, 1.3 equiv.) in dry THF (0.5 M) at 0 °C was added triethylphosphonoacetate dropwise (1.3 equiv.) and the reaction was stirred for 0.5 h at 0 °C. The ketone (1.0 equiv.) was added to the above reaction mixture which was stirred for 1 h at 0 °C and then overnight at rt. After the starting material was fully consumed, a saturated aqueous NH_4Cl solution (10 mL) and H_2O (30 mL) were added at 0 °C, and the reaction mixture was allowed to warm to rt. The mixture was extracted with Et_2O (×3). The combined organic extracts were washed with brine, dried over $MgSO_4$, and concentrated under reduced pressure to afford crude α,β-unsaturated ester which was further purified by flash column chromatography to give title compound (eluent: hexane/EtOAc = 20:1 to 10:1).

Step II: The above obtained α,β-unsaturated ester (1.0 equiv.) was dissolved in anhydrous Et_2O (0.25 M) and cooled to –78 °C, DIBAL–H (2.2 equiv. 1.0 M in hexane) was added dropwise and the reaction mixture was stirred at the same temperature for 3 h. The reaction was then warmed to rt over the period of 1 h before saturated aqueous NH_4Cl solution was added (~1/10 Vol/Vol) dropwise at 0 °C until the reaction mixture turned cloudy. Saturated aqueous Rochelle's salt was added (equal Vol.) and the reaction mixture stirred vigorously for 1 h until the emulsion homogenized. The layers were separated, and the organic layer was washed with saturated aqueous NH_4Cl solution, brine, dried over $MgSO_4$, filtered, and concentrated under reduced pressure to afford crude allylic alcohol which was further purified by flash column chromatography to give the title compound (eluent: hexane/EtOAc = 4:1).

1.2.3 General Procedure C: Preparation of 3-fluoro-3-aryl allylic alcohols

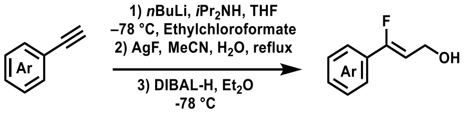

Step I: To a solution of *i*Pr_2NH (2.5 equiv.) in anhydrous tetrahydrofuran (0.48 m) was added *n*BuLi (as 2.5 M solution in hexanes, 2.5 equiv.) at –78 °C. The mixture was stirred for 15 minutes and the appropriate arylacetylene (1.0 equiv.) was added at –78 °C. After being stirred for 30 minutes ethyl chloroformate (4.5 equiv.) was added at –78 °C. The reaction was allowed to warm to room temperature and stirred for further 2 hours. The mixture was then cooled to 0 °C, quenched with saturated aqueous NH_4Cl solution, extracted with ethyl acetate (×3), dried over $MgSO_4$, filtered and concentrated in vacuo. The crude was purified by flash column chromatography with the eluent stated.

Step II: To a stirred solution of an arylpropiolate (1.0 equiv.) and water (6.0–7.0 equiv.) in acetonitrile (0.5 M) was added silver(I) fluoride (2.0 equiv.) at room temperature. The reaction was stirred under reflux (90 °C) overnight. The mixture was allowed to cool to room temperature and was then pressed through a plug of silica using diethyl ether as the eluent. Further purification was accomplished by flash column chromatography with the eluent stated.

Step III: A solution of appropriate 3-fluoro-3-arylacrylate (1.0 equiv.) in anhydrous Et_2O (0.25 M) was cooled to –78 °C, DIBAL–H (2.2 equiv. 1.0 M in hexane) was added dropwise, and the reaction mixture was stirred at the same temperature for 3 h. The reaction was then warmed to rt over a period of 1 h before saturated aqueous NH_4Cl was added (~1/10 Vol.) dropwise at 0 °C until the reaction mixture turned cloudy. Saturated aqueous Rochelle's salt was added (equal Vol.) and the reaction mixture was stirred vigorously for 1 h until the emulsion homogenized. The layers were separated, and the organic layer was washed with saturated aqueous NH_4Cl, brine, dried over $MgSO_4$, filtered, and concentrated under reduced pressure to afford crude allylic alcohol which was further purified by flash column chromatography to give the title compound (eluent: hexane/EtOAc = 4:1).

1.2.4 General Procedure D: Preparation of 3-substitued allylic alcohols

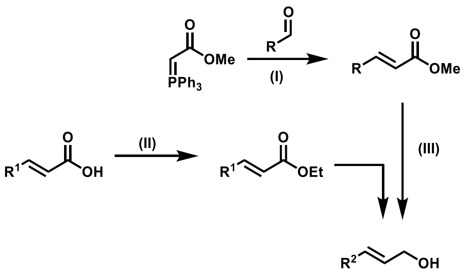

Step I: To a solution of methyl (triphenylphosphoranylidene)acetate (1.0 equiv.) in CH_2Cl_2 (1.0 M) was added the appropriate aldehyde (1.1 equiv.) while stirring at 0 °C. The mixture was allowed to warm to rt and stirred for 3 days, then concentrated under reduced pressure. The residue was suspended in Et_2O (0.06 M) and stirred at rt for 1 h, then cooled to 0 °C. The mixture was filtered, and the filter cake was washed with ice-cold Et_2O. The filtrate was concentrated under reduced pressure and the residue was purified by flash column chromatography to give the corresponding α,β-unsaturated methyl esters.

Step II: A solution of the appropriate acrylic acid (1.0 equiv.) and concentrated H_2SO_4 (0.1 mL/g) in ethanol (10 mL/g) was heated to reflux and stirred for 3 h, then allowed to cool to rt and concentrated under reduced pressure. The residue was basified with saturated aqueous $NaHCO_3$, diluted with EtOAc and separated. The aqueous phase was extracted with EtOAc (×2), then the combined organic extracts were washed with brine, dried over $MgSO_4$, and concentrated *in vacuo* to give the corresponding α,β-unsaturated ethyl esters, which were used without further purification unless otherwise specified.

Step III: To a solution of the appropriate α,β-unsaturated ester (1.0 equiv.) in anhydrous CH_2Cl_2 (0.25 M) was added DIBAL-H (25 wt% in toluene or 1.0 M in hexanes, 2.5 equiv.) dropwise while stirring at −78 or 0 °C as specified. The mixture was stirred for 3 h, then allowed to warm to rt. 10% *w*/*v* aqueous NaOH (12.5 equiv.) was added and the mixture was stirred for 1 h. The phases were separated and the aqueous phase was extracted with CH_2Cl_2 (×2). The combined organic extracts were washed with brine, dried over $MgSO_4$, and concentrated *in vacuo*. The residue was purified by flash column chromatography, unless otherwise specified, to give the corresponding allylic alcohols.

1.2.5 General Procedure E: Preparation of (*E*)-1-cyclopropyl-3-phenylbut-2-en-1-ol (SS1)

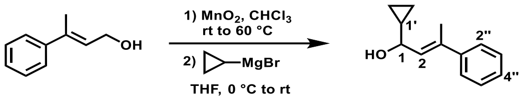

To a 50 mL round bottom flask containing the allylic alcohol (1.0 equiv.) obtained with **General Procedure B**, was added activated MnO_2 (5.0 equiv.) and anhydrous $CHCl_3$ (0.3 M) at rt. The reaction mixture was stirred at 60°C and monitored by TLC. After complete consumption of the starting material, the reaction mixture was filtered through a pad of celite. The resulting filtrate was concentrated under reduced pressure. The crude residue was purified by flash column chromatography to afford the corresponding product (eluent: hexanes/EtOAc = 19/1).

To a stirred solution of above obtained aldehyde (1.0 equiv.) in THF (0.5 M) at 0 °C was added cyclopropylmagnesium bromide (0.5 M in THF, 1.2 equiv.) dropwise. The reaction mixture was allowed to warm to rt and stirred until complete consumption of the starting material. The reaction mixture was quenched with sat. aq. NH_4Cl and extracted with Et_2O (×3). The combined organic extracts were washed with brine, dried over $MgSO_4$, and concentrated under reduced pressure to afford crude allylic alcohol which was purified by flash column chromatography to give the title compound (eluent: hexane/EtOAc = 5:1 to 3:1) (0.57 g, 65%) as a colourless oil. **IR** ν_{max} (film) 3003 (OH), 1597 (C=C), 1492, 1444, 1379; **^{1}H NMR** (400 MHz, $CDCl_3$) δ **0.30–0.44** (2H, m, C(2`)$H^A$H$^B$, C(3`)H^AH^B), **0.47–0.62** (2H, m, C(2`)H$^A$$H^B$, C(3`)H^AH^B), **1.08–1.19** (1H, m, C(1`)*H*), **1.82** (1H, s, O*H*), **2.09** (3H, d, *J* 1.3, CH_3), **4.05** (1H, t, *J* 7.6, C(1)*H*), **5.86** (1H, dq, *J* 8.5, 1.4, C(2)*H*), **7.24–7.30** (1H, m, ArC(4``)*H*), **7.30–7.36** (2H, m, ArC(3``,5``)*H*), **7.40–7.46** (m, 2H, ArC(2``,6``)*H*); **$^{13}$C{$^1$H} NMR** (101 MHz, $CDCl_3$) δ **1.9** (*C*(2`)$H_2$), **2.9** (*C*(3`)$H_2$), **16.5** (*C*$H_3$), **18.0** (*C*(1`)H), **72.8** (*C*(1)OH), **126.0** (Ar*C*(2``,6``)H), **127.4** (Ar*C*(4``)H), **128.3** (Ar*C*(3`,5`)H), **129.3** (*C*(2)H), **137.3** (*C*(3)H), **143.1** (*C*(1``)); **HRMS (ESI$^+$)** $C_{13}H_{16}ONa$ $[M+Na]^+$ found 211.1088, requires 211.1093 (–2.4 ppm).

1.2.6 General Procedure F: Preparation of allylic ether by Rh-catalyzed O-H insertion

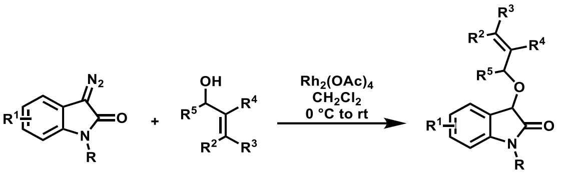

To a stirred solution of appropriate allylic alcohol (1.0 equiv.) in CH_2Cl_2 (0.05 M) was added $Rh_2(OAc)_4$ (0.005 equiv.) at 0 °C. The appropriate diazo compound (1.1 equiv.) was added at once, the reaction mixture was allowed to warm to rt and stirred for 2-4 h. After the consumption of the starting material was determined by TLC, the mixture was concentrated under reduced pressure to give the crude product, which was further purified by flash column chromatography to give the final allylic ether product.

1.2.7 General Procedure G: BIMP-catalyzed enantioselective [1,2]-rearrangement reaction at room temperature

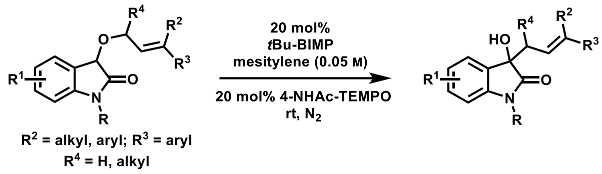

The appropriate allylic ether substrate (0.1 mmol), *t*Bu-BIMP (0.02 mmol) and 4-NHAc-TEMPO (0.02 mmol) were added to a flame-dried Schlenk tube and the tube was flushed with N_2 three times. Mesitylene (0.05 M) was added through the septum under a positive pressure of N_2. The reaction was stirred at rt until completion as indicated by TLC analysis. The reaction mixture was concentrated under reduced pressure to give the crude product, which was purified by flash column chromatography (eluent: hexane/EtOAc = 4:1 to 3:1).

1.2.8 General Procedure H: BIMP-catalyzed enantioselective formal [1,2]-rearrangement reaction under heating

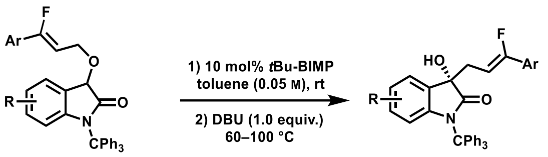

The appropriate allyloxy-oxindole (0.1 mmol), *t*Bu-BIMP (0.005 mmol) were added to a flame-dried Schlenk tube and the tube was flushed with N_2 three times. Toluene (0.05 M) was added through the septum under a positive pressure of N_2. The reaction was stirred at rt for 15–24 h. DBU (1.0 equiv.) was then added to the above reaction mixture and then heated at 60–100 °C for 5–20 h. The reaction mixture was concentrated under reduced pressure to give the crude product, which was purified by flash column chromatography.

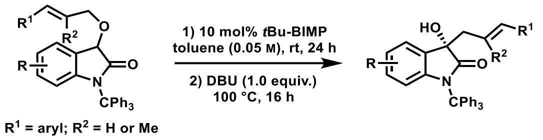

To a vial was added the appropriate allyloxy-oxindole (1.0 equiv.), *t*Bu-BIMP (10 mol%), and toluene (0.05 M). The mixture was stirred at rt for 24 h, then DBU (1.0 equiv.) was added, and the mixture was then heated to 100 °C and stirred for 16 h, then quenched by addition of sat. aq. NH_4Cl and diluted with EtOAc. The phases were separated, and the aqueous phase was extracted with EtOAc (×2). The combined organic phases were washed with brine, dried ($MgSO_4$) and concentrated under reduced pressure. The residue was purified as specified to give the corresponding products.

1.2.9 General Procedure I: Preparation of racemic [1,2]-rearrangement product

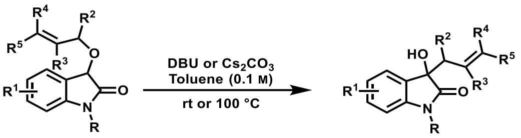

The appropriate allylic ether substrate (0.1 mmol), DBU or Cs_2CO_3 (0.1 mmol) were added to a flame-dried Schlenk tube and the tube was flushed with N_2 three times. Toluene (0.1 M) was added through the septum under a positive pressure of N_2. The reaction was stirred at rt until completion as indicated by TLC analysis. The reaction mixture was concentrated under reduced pressure to give the crude product, which was purified by flash flash column chromatography (eluent: hexane/EtOAc = 4:1 to 3:1).

1.2.10 General Procedure J: Preparation of [2,3]-Wittig rearrangement product

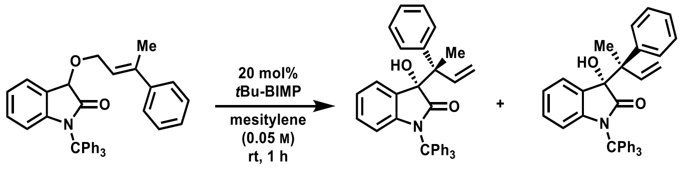

8 major diastereomer 14 minor diastereomer

To a stirred solution of allylic ether **8** (1.0 equiv.) in mesitylene (0.05 M) was added *t*Bu-BIMP (0.2 equiv.) at rt. The solution was stirred at rt for 1 h, then half of the volume of mesitylene was removed under reduced pressure, and the residue was purified by flash column chromatography (hexane/acetone = 8:1 to 4:1) to give the corresponding [2,3] products.

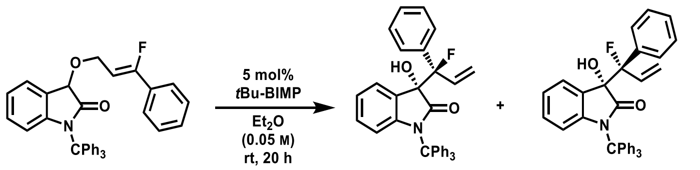

41 major diastereomer 42 minor diastereomer

To a stirred solution of allylic ether **41** (1.0 equiv.) in Et_2O (0.05 M) was added *t*Bu-BIMP (0.05 equiv.) at rt. The solution was stirred at rt for 20 h, then the reaction mixture was concentrated under reduced pressure, and the residue was purified by flash column chromatography (hexane/EtOAc = 4:1) to give the corresponding [2,3] products.

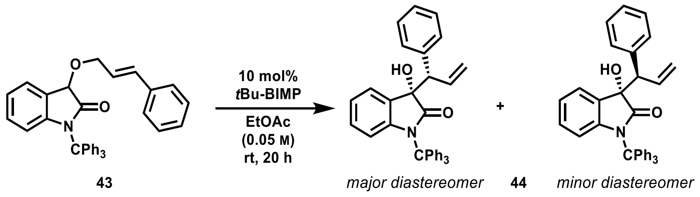

To a stirred solution of allylic ether **43** (1.0 equiv.) in EtOAc (0.05 M) was added *t*Bu-BIMP (0.10 equiv.) at rt. The solution was allowed to stir at rt for 20 h, then the reaction mixture was concentrated under reduced pressure, and the residue was purified by flash column chromatography (: Petrol/EtOAc = 4:1) to give the corresponding [2,3] products.

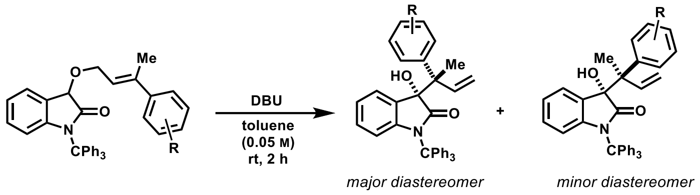

To a stirred solution of allylic ether (1.0 equiv.) in toluene (0.05 M) was added DBU (5.0 equiv.) at rt. The solution was allowed to stir at rt for 2 h. Half of the volume of mesitylene was removed under reduced pressure and the residue was summited to flash column chromatography to give the title compound (eluent: hexane/acetone = 8:1 to 4:1).

1.2.11 General Procedure K: Determination of the absolute configuration of [1,2]-rearrangement product

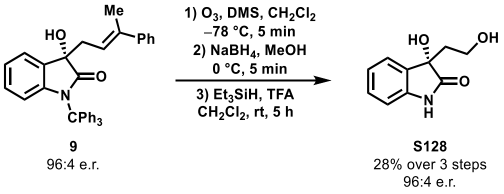

Step I: Through a stirred solution of [1,2]-rearrangement product **9** (0.20 mmol), obtained according to **General procedure G**, in CH_2Cl_2 (30 mL) was bubbled O_3 at –78 °C until a pale blue solution formed (~5 min). SMe_2 (3.0 mmol, 10 equiv.) was added at –78 °C and the reaction mixture was kept stirring for 0.5 h until light blue color disappeared. The mixture was concentrated to afford the crude aldehyde which was used without further purification.

Step II: To the above obtained aldehyde was added methanol (5 mL) at 0 °C. $NaBH_4$ (0.45 mmol) was added and the mixture stirred at 0 °C for 5 min. After completion was determined by TLC analysis, sat. aq. NH_4Cl (5 mL) was added. The reaction mixture was extracted with EA (×4). The combined organic extracts were dried over $MgSO_4$, filtered, and concentrated under reduced pressure to give crude alcohol product.

Step III: The above afforded alcohol product was dissolved in CH_2Cl_2 (2 mL). At rt, Et_3SiH (1.5 mmol) and TFA (1.0 mL) were added. The reaction mixture was stirred at rt for 5 h. After completion was determined by TLC analysis, excess TFA was removed under reduced pressure and the mixture was redissolved in EtOAc (10 mL) and neutralized by addition of sat. aq. $NaHCO_3$. The mixture was extracted with EtOAc (×4). The combined organic extracts were dried over $MgSO_4$, filtered, and concentrated under reduced pressure. Further purification by flash column chromatography (eluent: hexane/EtOAc = 3:1 to 1:1) gave the final product.

1.2.12 General Procedure L: Determination of the absolute configuration of 14 and 46

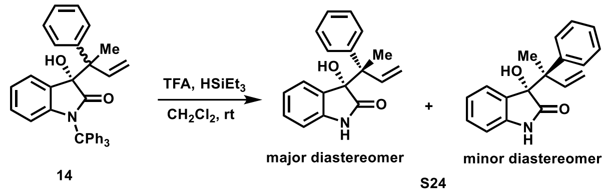

To a stirred solution of [2,3]-rearrangement product **14** (0.20 mmol), obtained according to **General procedure J,** in CH_2Cl_2 (2 mL) at rt was added, Et_3SiH (1.0 mmol) and TFA (1.0 mL). The reaction mixture was stirred at rt for 1.5 h. After completion was determined by TLC analysis, excess TFA was removed under reduced pressure and the mixture was redissolved in EtOAc (10 mL) and neutralized by sat. aq. $NaHCO_3$. The mixture was extracted with EtOAc (×4). The combined organic extracts were dried over $MgSO_4$, filtered, and concentrated under reuduced pressure. Further purification by flash column chromatography (eluent: hexane/EtOAc = 3:1 to 1:1) gave the final product.

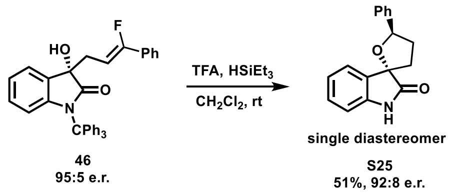

To a stirred solution of [1,2]-rearrangement product **46** (0.10 mmol), obtained according to **General procedure H**, in CH_2Cl_2 (1 mL) at rt was added, Et_3SiH (0.5 mmol) and TFA (0.5 mL) was added. The reaction mixture was stirred at rt for 16 h. After completion was determined by TLC analysis, excess TFA was removed under reduced pressure and the mixture was redissolved in EtOAc (10 mL) and neutralized by sat. aq. $NaHCO_3$. The mixture was extracted with EtOAc (×4). The combined organic extracts were dried over $MgSO_4$, filtered, and concentrated under reduced pressure. Further purification by flash column chromatography (eluent: hexane/EtOAc = 3:1 to 1:1) gave the final product in 51% yield as a single diastereomer with 92:8 e.r.

1.2.13 General Procedure M: Preparation of BIMPs

The BIMP catalysts (*t*Bu-BIMP, Ph-BIMP, Bn-BIMP) used in the paper were synthesized according to a literature procedure reported by Dixon et al[1,2].

2 Supplementary data of reaction optimization

Table S1. Conditions screening on allylic ethers

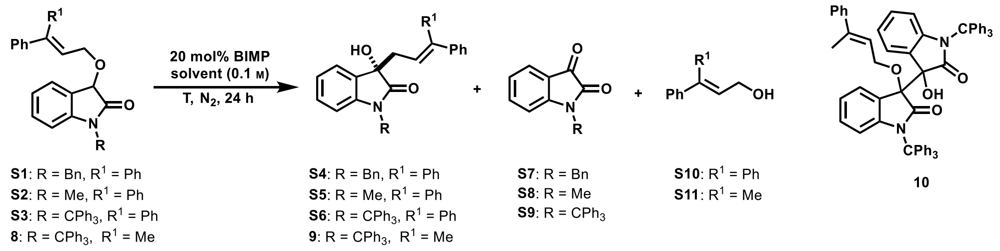

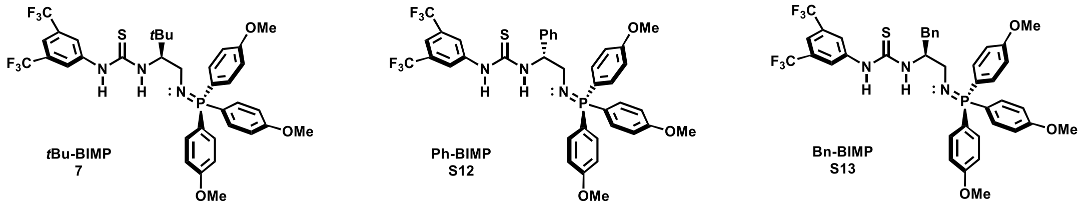

Entry	R	R^1	BIMP	T / °C	solvent	yield / %[a] **S4-S6, 9**	yield / %[a] **(S7-S9)** +**(S10-S11)**	e.r. [b] **S4-S6, 9**
1	Bn	Ph	**7**	50	Toluene	31	8	79:21
2	Bn	Ph	**7**	50	EA	88	11	72:28
3	Bn	Ph	**7**	50	MeCN	66	8	70:30
4	Bn	Ph	**7**	50	EC	95	<2	56:44
5	Bn	Ph	**S12**	50	EA	85	<5	40:60
6	Bn	Ph	**S13**	50	DMC	97	<5	72:28
7	Bn	Ph	**S13**	50	EA	92	<5	56:44
8	Me	Ph	**7**	50	Toluene	55	18	69:31
9	CPh_3	Ph	**7**	50	Toluene	20	<5	81:19
10	CPh_3	Ph	**7**	50	DMC	<5	<5	N.D.
11	CPh_3	Me	**7**	50	DMC	87	<2	85:15
12	CPh_3	Me	**7**	50	Toluene	89	<2	88:12
13	CPh_3	Me	**7**	50	2-MeTHF	93	<2	86:14
14	CPh_3	Me	**7**	50	DCE	14	<5	88:12
15[c]	CPh_3	Me	**7**	50	mesitylene	90	<2	88:12
16[c]	CPh_3	Me	**7**	50	chlorobenzene	95	<2	87:13
17[c]	CPh_3	Me	**7**	50	Trifluoromethy lbenzene	89	<2	87:13
18[c]	CPh_3	Me	**7**	40	toluene	92	<2	88:12
19[c]	CPh_3	Me	**7**	30	toluene	93	<2	89:11

20[c]	CPh_3	Me	**7**	rt	toluene	98	<2	91:9
21[c]	CPh_3	Me	**7**	rt	mesitylene	93	<2	92:8
22	CPh_3	Me	**7**	rt	MTBE	90	<2	91:9
23	CPh_3	Me	**7**	rt	Et_2O	63	<2	91:9
24	CPh_3	Me	**7**	rt	mesitylene	94	<2	92:8
25	CPh_3	Me	**7**	rt	Et_2O	63	<2	91:9
26[d]	CPh_3	Me	**7**	rt	mesitylene	94	<2	92:8
27[e]	CPh_3	Me	**7**	rt	mesitylene	91	<2	92:8
28[f]	CPh_3	Me	**7**	rt	mesitylene	92	<2	92:8
29[g,h]	CPh_3	Me	**7**	rt	mesitylene	53	<5	97:3
30[h,i]	CPh_3	Me	**7**	rt	mesitylene	59	<5	97:3
31[h,j]	CPh_3	Me	**7**	rt	mesitylene	90	<5	91:9
32[h,k]	CPh_3	Me	**7**	rt	mesitylene	48	<5	96:4
33[h,l]	CPh_3	Me	**7**	rt	mesitylene	73	<5	95:5

Unless otherwise noted, the reaction was performed at a 0.05 mmol scale under varied solvents and temperatures. EA = ethyl acetate; EC = ethylene carbonate; DMC = dimethyl carbonate; DCE = 1,2-dichloroethane; MTBE = methyl tert-butyl ether; TEMPO = (2,2,6,6-tetramethylpiperidin-1-yl)oxidanyl; 4-NHAc-TEMPO = (4-acetamido-2,2,6,6-tetramethylpiperidin-1-yl)oxidanyl; 4-MeO-TEMPO = 4-Methoxy-2,2,6,6-tetramethyl-1-piperidinyloxy; a Determined by ^{1}H NMR using 1,3,5-trimethoxybenzene as the internal standard. b Determined by HPLC analysis on a chiral stationary phase. c The reaction time was 18 h. d Mesitylene (0.05 M). e Mesitylene (0.025 M). f Mesitylene (0.02 M). g TEMPO (1.0 equiv.) was added as additive. h Isolated yield was given, the aldol product **10** was also observed with ~3-7% when adding TEMPO, 4-NHAc-TEMPO or 4-MeO-TEMPO as additives. i 4-NHAc-TEMPO (1.0 equiv.) was added as additive. j 1,4-cyclohexadiene (1.0 equiv.) was added as additive. k 4-MeO-TEMPO (1.0 equiv.) was added as additive. l 4-NHAc-TEMPO (0.2 equiv.) was added as additive, and the reaction time was 20 h.

3 Supplementary data for Control experiments

Scheme S1. Control reaction of **8** and **S9**

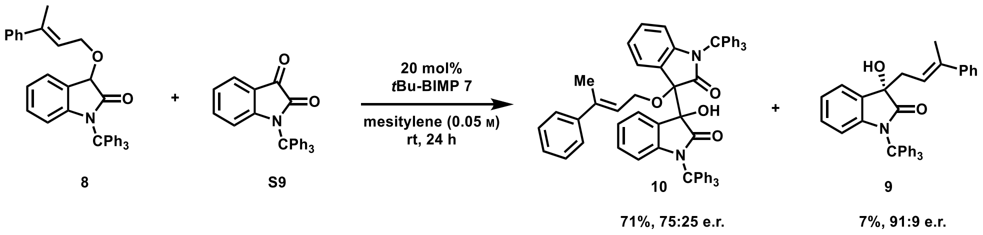

Scheme S2. Control reactions of **41** and **43** with TEMPO

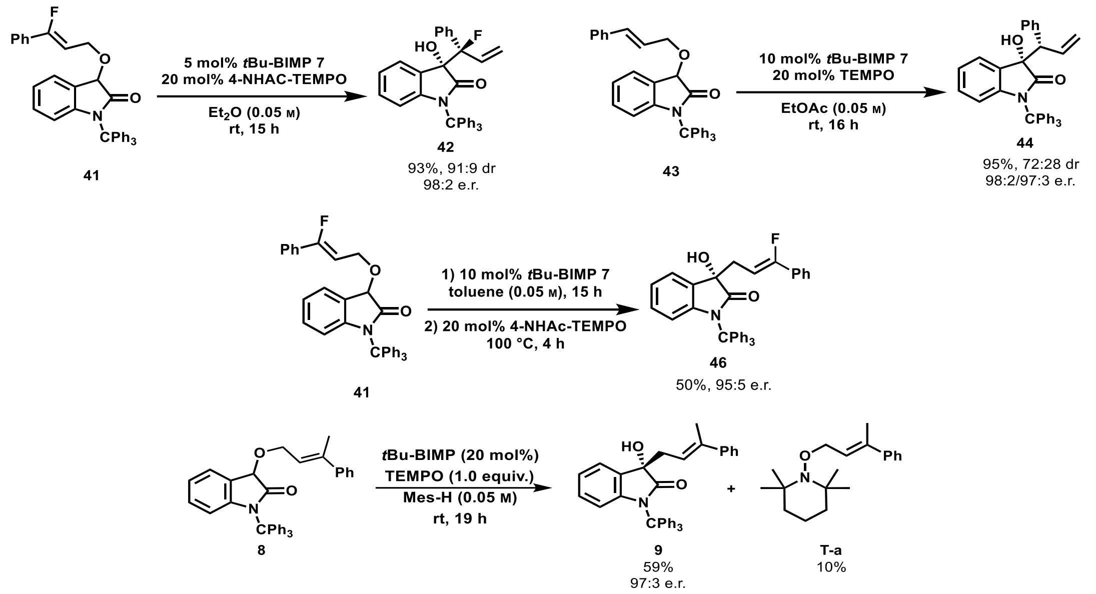

Table S2. Solvent effect on [1,3]-rearrangement

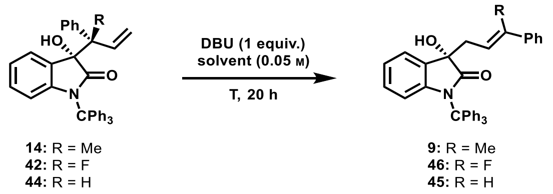

Entry	R	solvent	T / °C	yield / %[a]	e.r. [b]
1	Me	DMF	rt	57	85:15
2	Me	mesitylene	rt	36	96:4
3	F	DMF	100	10	85:15
4	F	Toluene	100	50	95:5
5	H	MeCN	80	49	86:14
6	H	DMF	100	40	91:9
7	H	toluene	100	57	95:5

Table S3: Isolation of [2,3]-Wittig rearrangement product

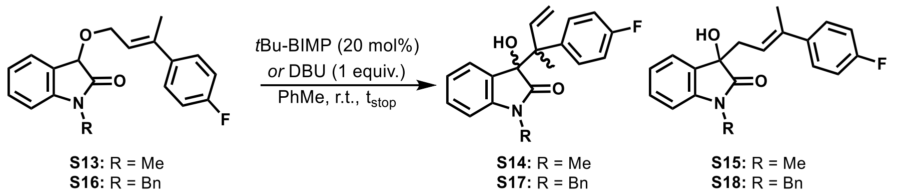

Entry	R	Base	t_{stop} / min	RSM / %	[1,2] Yield / %	[1,2] e.r.	[2,3] Yield / %	[2,3] dr	[2,3] e.r.
**1**	Me	*t*Bu-BIMP	30	19	40	68:32	39	60:40	60:40 / 71:29
**2**	Bn	*t*Bu-BIMP	15	11	24	81:19	46	59:41	79:21 / 79:21
**3**	Me	DBU	60	57	11	-	25	65:35	-
**4**	Bn	DBU	45	49	12	-	40	60:40	-

Table S4: 1,3-allylic shift and trap

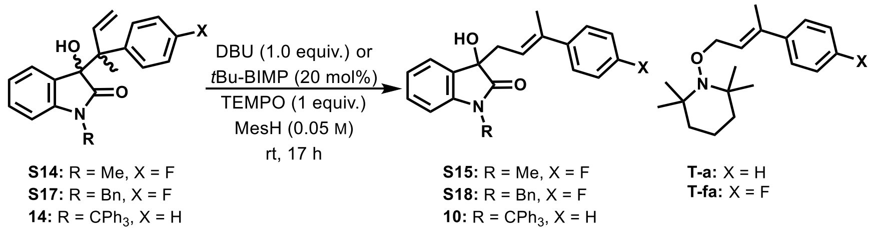

Entry	X	R	[2,3] dr	[2,3] e.r.	RSM / %	[1,2] Yield / %	[1,2] e.r.	T-a or T-fa Yield / %
**1**[a]	F	Me	63:37	59:41 / 71:29	29	49	63:37	-
**2**[a]	F	Bn	63:37	80:20 / 79:21	25	65	77:23	-
**3**[a]	F	Bn	61:39	80:20 / 79:21	19	60	77:23	14
**4**[a]	F	Bn	61:39	rac	26	58	rac	8
**5**[b]	H	CPh_3	86:14	95:5 / 98:2	N.D.	43	97:3	12

[a] DBU (1.0 equiv.) was used; [b] *t*Bu-BIMP (20 mol%) was used

Table S5: 1,3-allylic shift and trap

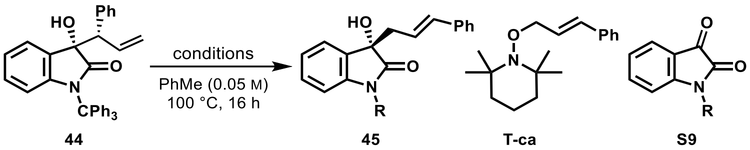

Entry	Conditions	RSM / %	45 yield / %	45 e.r.	S9 yield / %	T-ca yield / %
**1**	10 mol% BIMP + 1 equiv. DBU	N.D.	57	95:5	N.D.	-
**2**	20 mol% BIMP	76	7	98:2	7	-
**3**	20 mol% BIMP + 1 equiv. TEMPO	58	18	98:2	12	N.D.
**4**	1 equiv. DBU + 1 equiv. TEMPO	N.D.	51	94:6	N.D.	21
**5**	Heat to 100 °C only	80	N.D.	-	N.D.	-
**6**	20 mol% BIMP + 1 equiv. DBU + 1 equiv. TEMPO	10	45	94:6	N.D.	8
**7**	1 equiv. TEMPO	Quant.	N.D.	-	N.D.	N.D.

Scheme S3: One pot [1,2]-Wittig with TEMPO trap

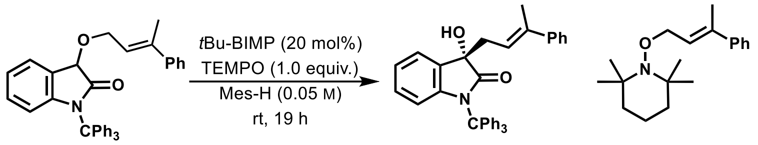

8 | **9** 59% 97:3 e.r. | **T-a** 10%

Table S6: Ester substituent [1,2]-Wittig rearrangement

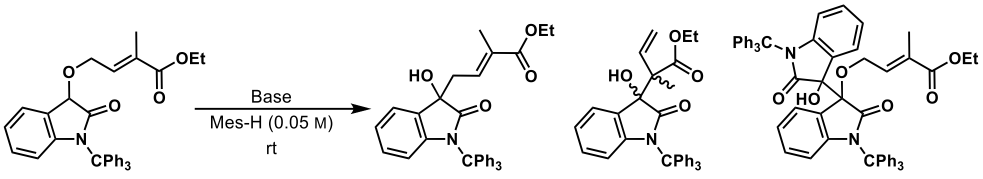

66 | **67** | **S19** | **68**

Entry	Base	t_{stop} / min	RSM / %	67 yield / %	67 e.r.	S19 Yield / %	S19 er	68 Yield / %	68 e.r.
**1**	*t*Bu-BIMP	330	N/A	55	98:2	9	89:11	15	88:12
**2**	DBU	60	24	9	50:50	11	50:50	25	50:50

Reactions were performed with either *t*Bu-BIMP **7** (20 mol%) or DBU (1 equiv.). For Entries 1 and 2, 9% and 7% of *N*-trityl isatin **S9** were also isolated, and only a single diastereomer was isolated for **S19**.

Scheme S4: Ester substituent [1,2]-Wittig rearrangement

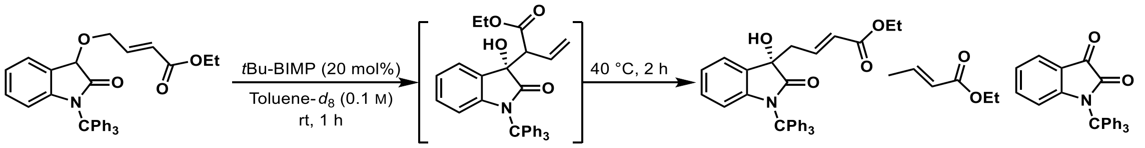

69 | **70** 48% 95:5 e.r. | **71** 22%[a] | **S9** 26%[a]

[a] As observed by ^{1}H NMR analysis of the reaction mixture

Scheme S5: Stability test of *t*Bu-BIMP **7** in PhMe-d_8 at 100 °C

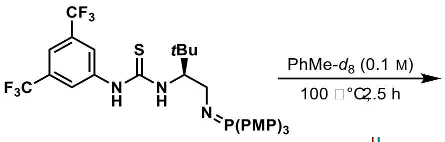

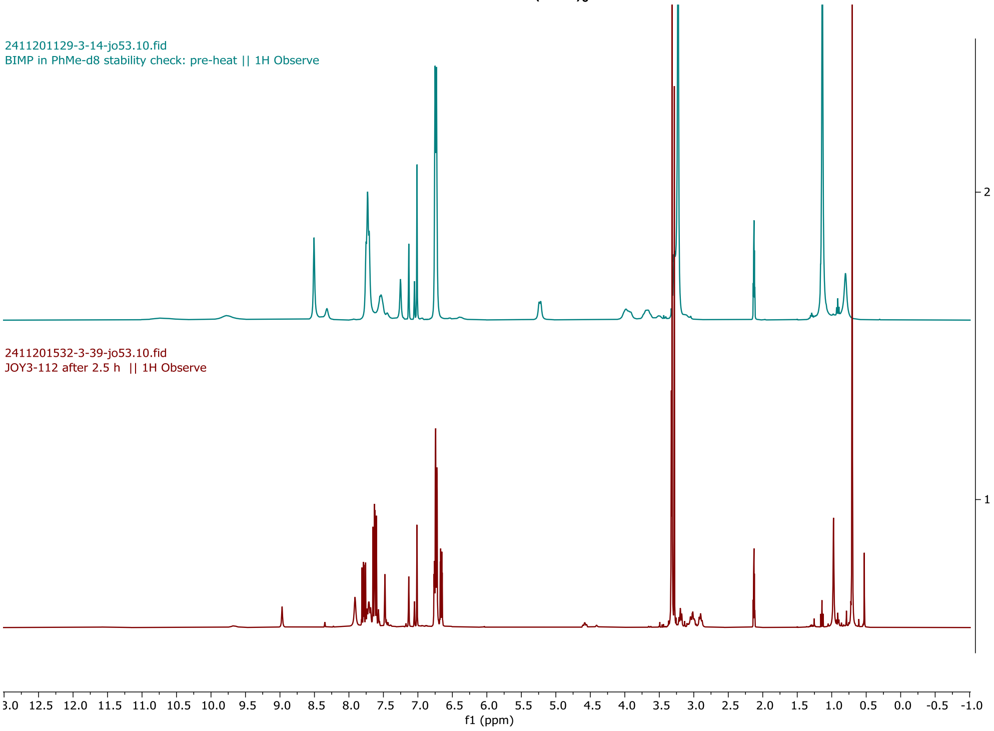

^{1}H NMR spectra (PhMe-d_8) – Top: pre-heat; Bottom: after 2.5 h at 100 °C

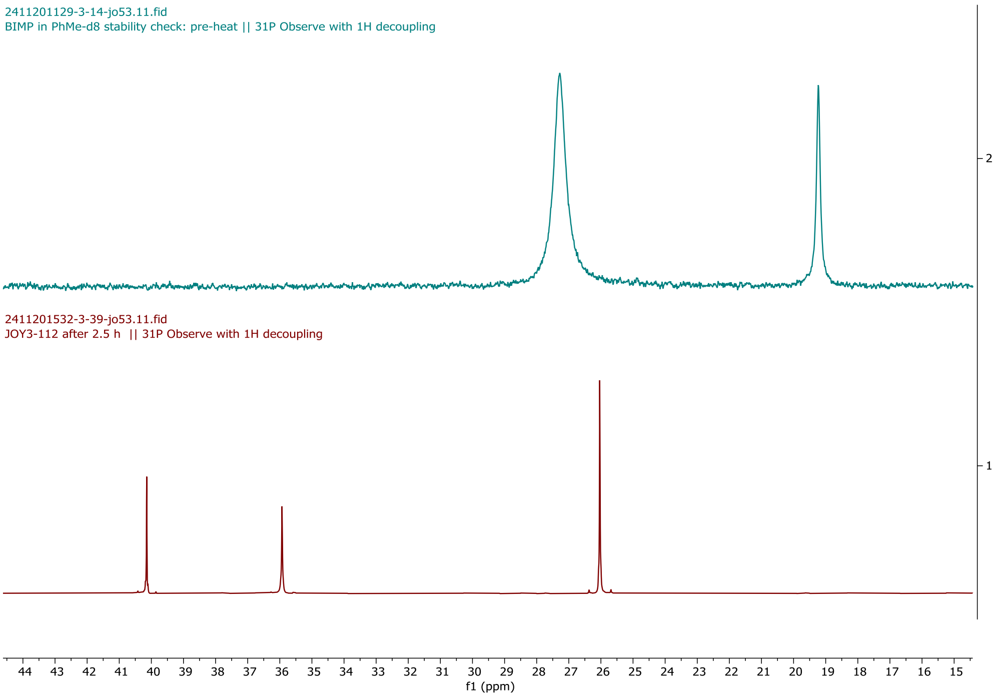

^{31}P{^{1}H} NMR spectra (PhMe-d_8) – Top: pre-heat; Bottom: after 2.5 h at 100 °C

Complete decomposition of *t*Bu-BIMP was seen after heating to 100 °C for 2.5 h.

Scheme S6: Reaction of N-trityl isatin **S9** with DBU in PhMe at 100 °C

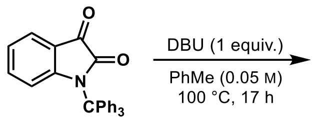

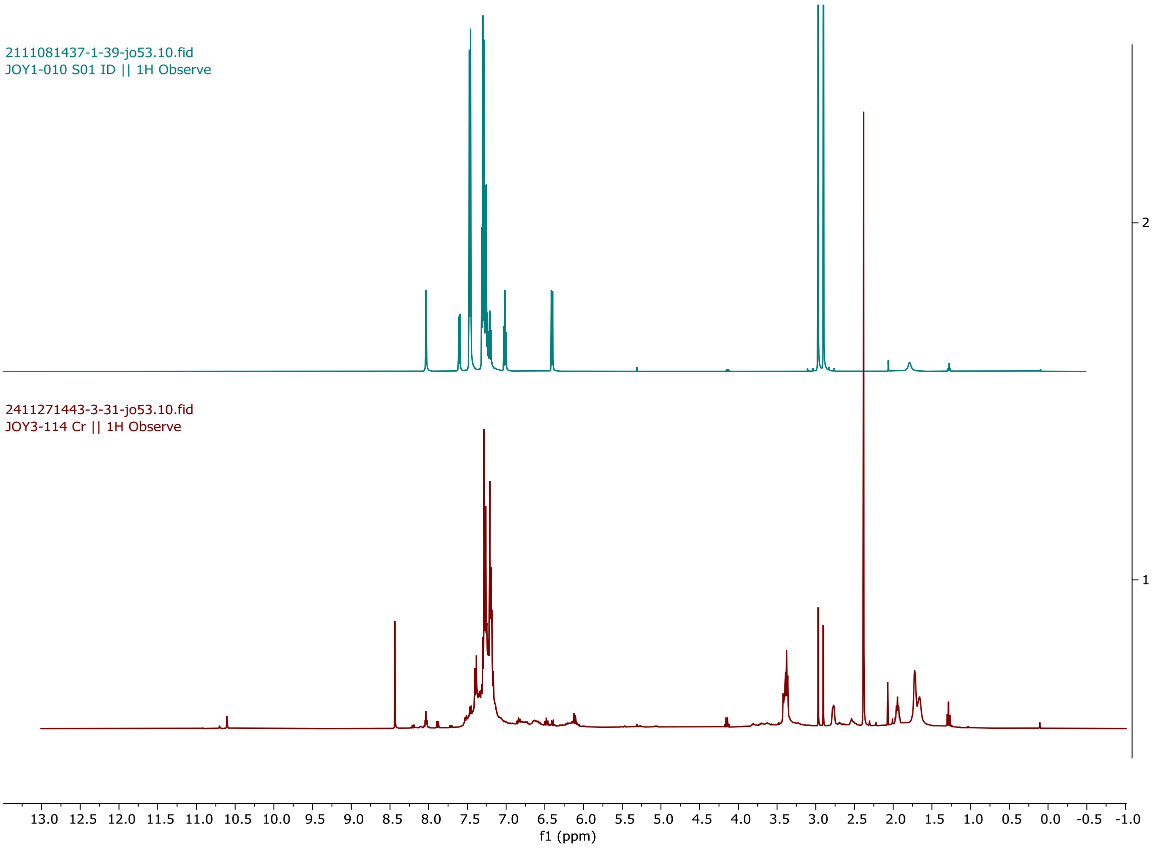

^{1}H NMR spectra ($CDCl_3$) – Top: N-Trityl isatin **S9** with residual DMF; Bottom: after heating at 100 °C for 17 h

Significant decomposition of N-trityl isatin **S9** observed after treatment with 1.0 equiv. DBU.

Scheme S7: Reaction of allylbenzene **S20** with TEMPO

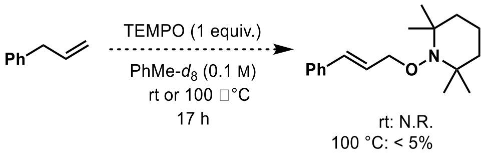

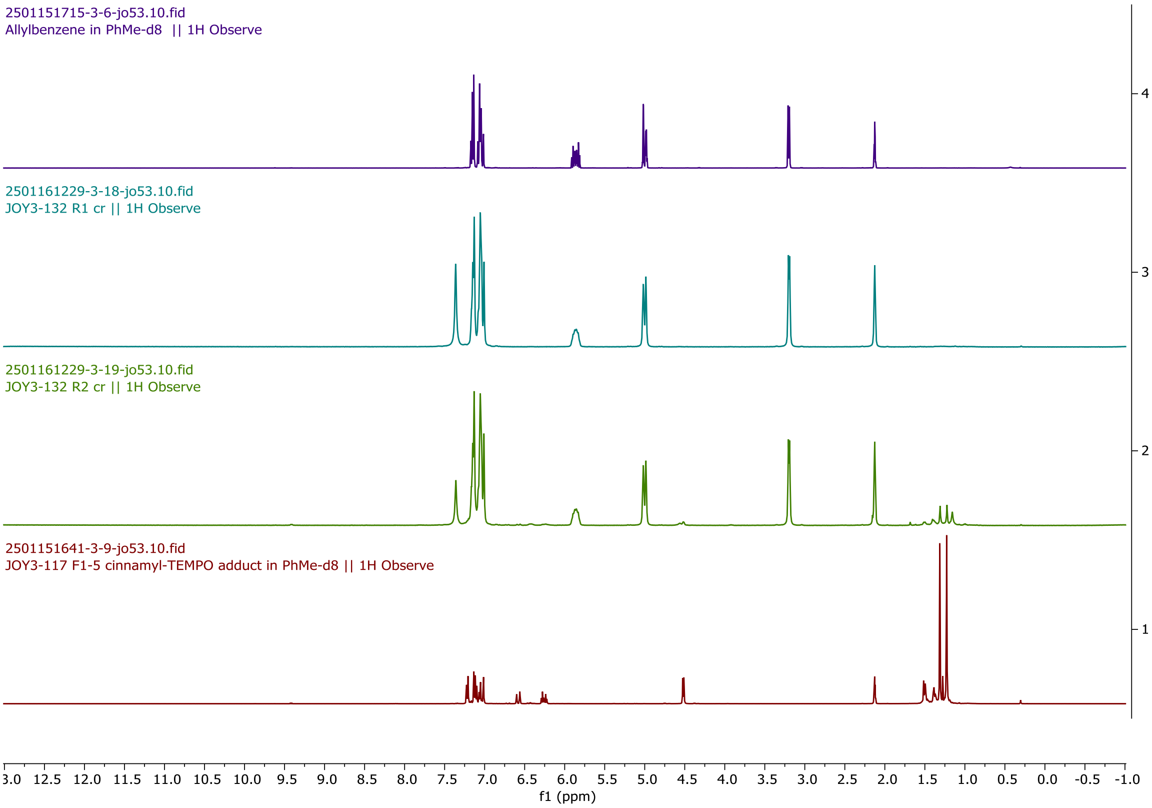

^{1}H NMR spectra (PhMe-d_8)– from top to bottom: starting material; reaction mixture after stirring at rt for 17 h; reaction mixture after stirring at 100 °C for 17 h; and authentic sample of desired product.

Scheme S8: Reaction of (*E*)-β-methylstyrene **S21** with TEMPO

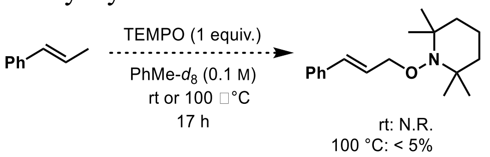

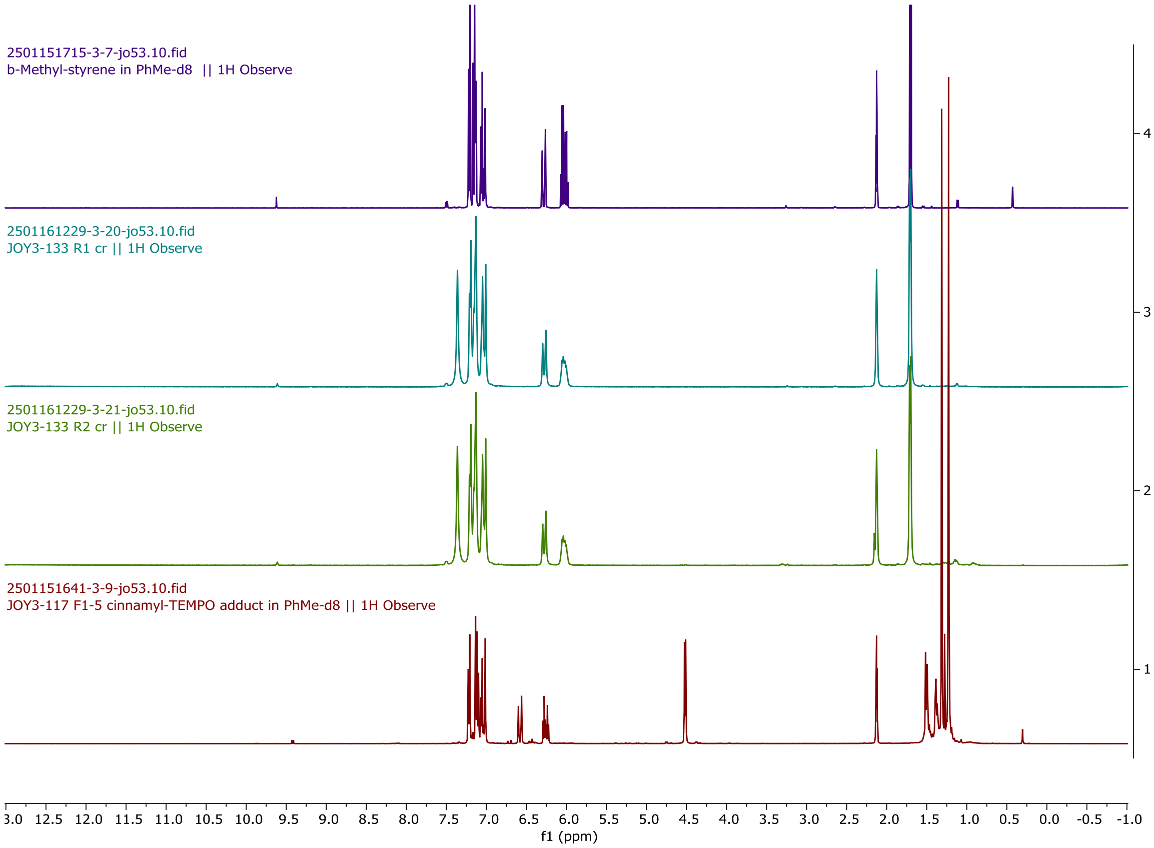

^{1}H NMR spectra (PhMe-d_8)– from top to bottom: starting material; reaction mixture after stirring at rt for 17 h; reaction mixture after stirring at 100 °C for 17 h; and authentic sample of desired product.

Scheme S9: Reaction of but-3-en-2-ylbenzene **S22** with TEMPO

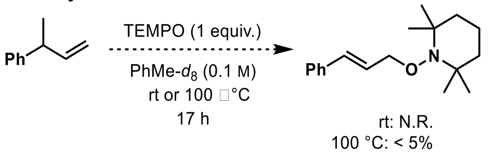

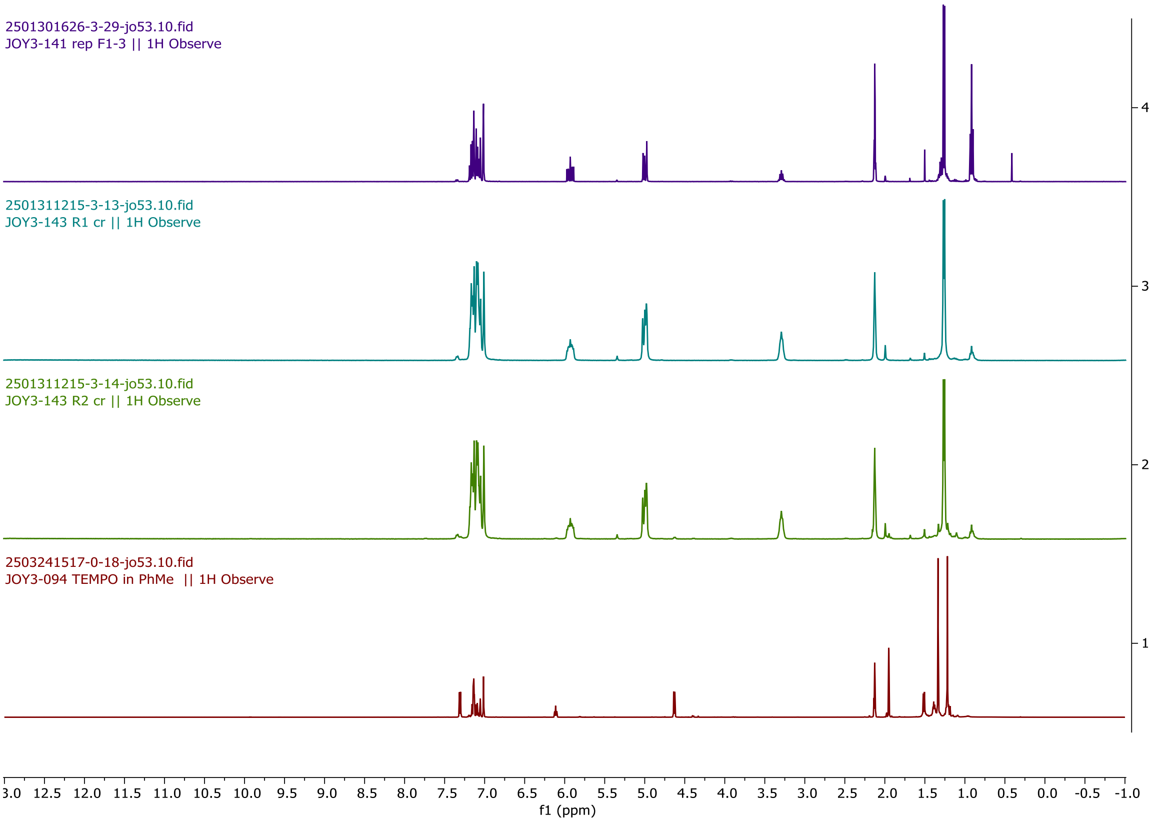

^{1}H NMR spectra (PhMe-d_8)– from top to bottom: starting material; reaction mixture after stirring at rt for 17 h; reaction mixture after stirring at 100 °C for 17 h; and authentic sample of desired product.

Scheme S10: Reaction of but-2-en-2-ylbenzene **S23** with TEMPO

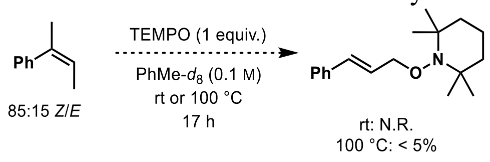

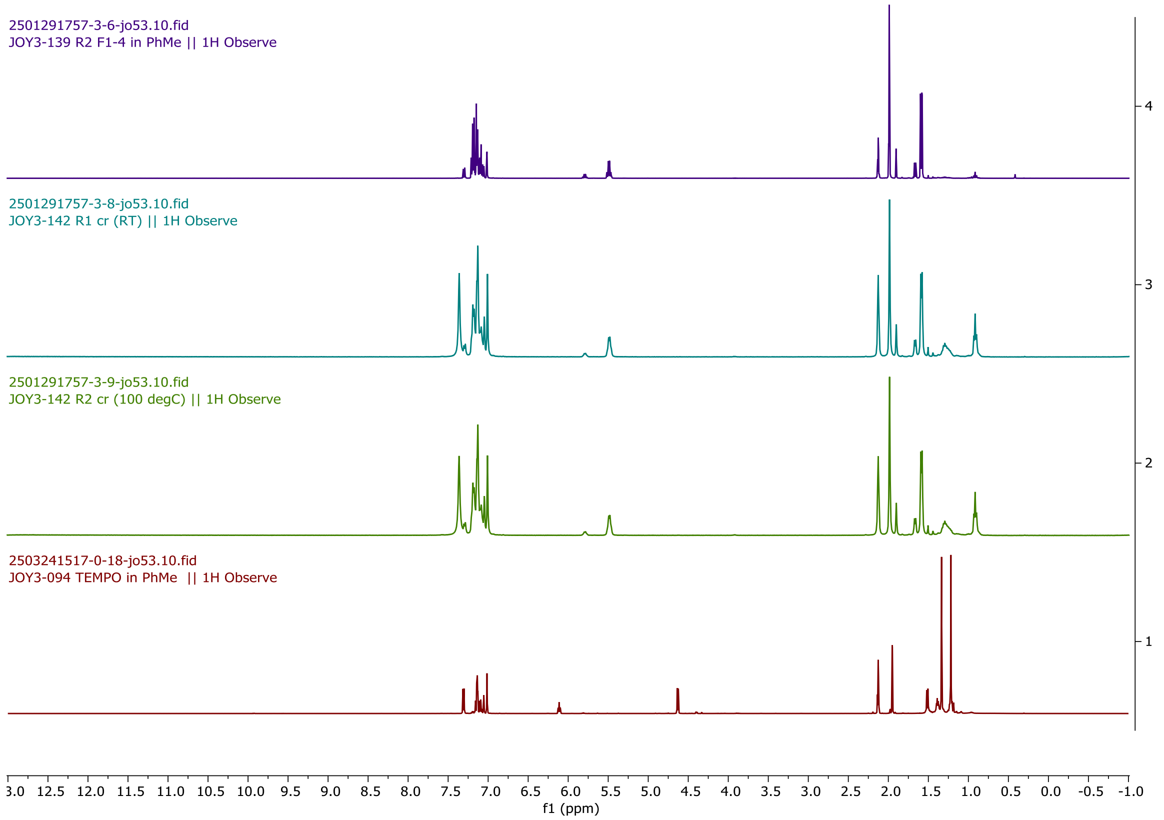

^{1}H NMR spectra (PhMe-d_8)– from top to bottom: starting material; reaction mixture after stirring at rt for 17 h; reaction mixture after stirring at 100 °C for 17 h; and authentic sample of desired product.

4 Supplementary data: limitations of the reaction

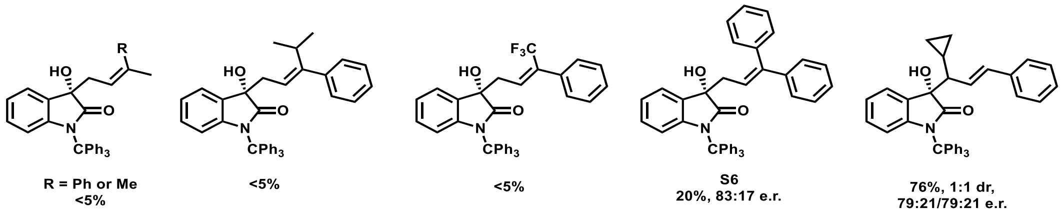

5 Supplementary Kinetic studies

5.1 Monitoring of the reaction using *in situ* ^{1}H NMR

Table S7: *In-situ* reaction monitoring via ^{1}H NMR

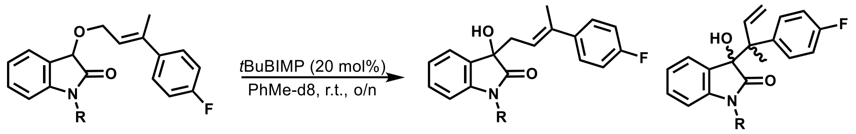

S13: R = Me
S16: R = F
11: R = CPh_3

S15: R = Me
S18: R = F
13: R = CPh_3

S14: R = Me
S17: R = F
12: R = CPh_3

**Entry**	**R**	**$t_{1/2}$ ([1,2]) / s**	***k* / s^{-1}**	**t_{Cmax} ([2,3]) / s**
**1**	Me	682	1.016×10^{-3}	1371
**2**	Bn	382	1.815×10^{-3}	901
**3**	CPh_3	2519	2.752×10^{-4}	3878

$t_{1/2}$ were obtained by linear regression model (1st order kinetics) shown in equation 1:

$$t_{\frac{1}{2}} = \frac{\ln\left(c_{\frac{1}{2}}\right) - \left(\ln\left(c_{\frac{1}{2}+t}\right) - \frac{\ln\left(c_{\frac{1}{2}+t}\right) - \ln\left(c_{\frac{1}{2}-t}\right)}{t_{\frac{1}{2}+t} - t_{\frac{1}{2}-t}} \cdot t_{\frac{1}{2}+t}\right)}{\frac{\ln\left(c_{\frac{1}{2}+t}\right) - \ln\left(c_{\frac{1}{2}-t}\right)}{t_{\frac{1}{2}+t} - t_{\frac{1}{2}-t}}} \quad \text{(equation 1)}$$

with the rate constant *k* (1st order kinetics) calculated as shown in equation 2:

$$k = \frac{\ln(2)}{t_{\frac{1}{2}}} \quad \text{(equation 2)}$$

t_{Cmax} were obtained from the 1st derivative of the integral or concentration of the [2,3]-intermediate, followed by linear regression model to find the abscissa intercept shown in equation 2:

$$t_{c_{max}} = 0 - \frac{\left(\dot{c}_{max+t} - \frac{\dot{c}_{max+t} - \dot{c}_{max-t}}{t_{c_{max+t}} - t_{c_{max-t}}} \cdot t_{c_{max+t}}\right)}{\frac{\dot{c}_{max+t} - \dot{c}_{max-t}}{t_{c_{max+t}} - t_{c_{max-t}}}} \quad \text{(equation 2)}$$

$$\text{with } \dot{c}_{[2,3]} = \frac{dc_{[2,3]}}{dt}$$

Table S7, entry 1: 1 mL stock solutions were prepared for 1,3,5-trimethoxybenzene (20.2 mg, 0.120 mmol, 0.120 M), (*E*)-3-((3-(4-fluorophenyl)but-2-en-1-yl)oxy)-1-methylindolin-2-one **S13** (37.4 mg, 0.120 mmol, 0.120 M) and *t*Bu-BIMP **7** (17.7 mg, 0.024 mmol, 0.024 M) using PhMe-*d*8 as solvent. To an oven-dried NMR tube was added each 0.25 mL of the 1,3,5-trimethoxybenzene (0.03 mmol) and (E)-3-((3-(4-fluorophenyl)but-2-en-1-yl)oxy)-1-methylindolin-2-one **S13** (0.03 mmol) stock solutions. The sample was used to shim the NMR machine. 0.25 mL of the *t*Bu-BIMP **7** (0.006 mmol) stock solution was added to the NMR tube. The NMR tube was shaken quickly three times and injected to the NMR machine. The reaction was monitored overnight. The reaction mixture was purified by flash column chromatography (CH_2Cl_2:Et_2O 47.5:2.5 → 45:5 → 40:10) to give the formal [1,2]-Wittig rearrangement product **S15** as a white solid (9.2 mg, 0.030 mmol, 99%, 65:35 e.r.).

Table S7, entry 2: 1 mL stock solutions were prepared for 1,3,5-trimethoxybenzene (20.2 mg, 0.120 mmol, 0.120 M), (*E*)-1-benzyl-3-((3-(4-fluorophenyl)but-2-en-1-yl)oxy)indolin-2-one **S16** (46.5 mg, 0.120 mmol, 0.120 M) and *t*Bu-BIMP **7** (17.7 mg, 0.024 mmol, 0.024 M) using PhMe-*d*8 as solvent. To an oven-dried NMR tube was added each 0.25 mL of the 1,3,5-trimethoxybenzene (0.03 mmol) and (*E*)-1-benzyl-3-((3-(4-fluorophenyl)but-2-en-1-yl)oxy)indolin-2-one **S16** (0.03 mmol) stock solutions. The sample was used to shim the NMR machine. 0.25 mL of the *t*Bu-BIMP **7** (0.006 mmol) stock solution was added to the NMR tube. The NMR tube was shaken quickly three times and injected to the NMR machine. The reaction was monitored overnight. The reaction mixture was purified by flash column chromatography (CH_2Cl_2:Et_2O 47.5:2.5 → 45:5 → 40:10) to give the formal [1,2]-Wittig rearrangement product **S18** as a white solid (10.8 mg, 0.028 mmol, 93%, 73:27 e.r.).

Table S7, entry 3: To a solution of (*E*)-3-((3-(4-fluorophenyl)but-2-en-1-yl)oxy)-1-tritylindolin-2-one **11** (0.03 mmol) and 1,3,5-trimethoxybenzene (0.03 mmol) in PhMe-d_8 (0.04 M) was added *t*Bu-BIMP (0.006 mmol) in NMR tube, then the reaction was monitored by ^{1}H NMR spectroscopy.

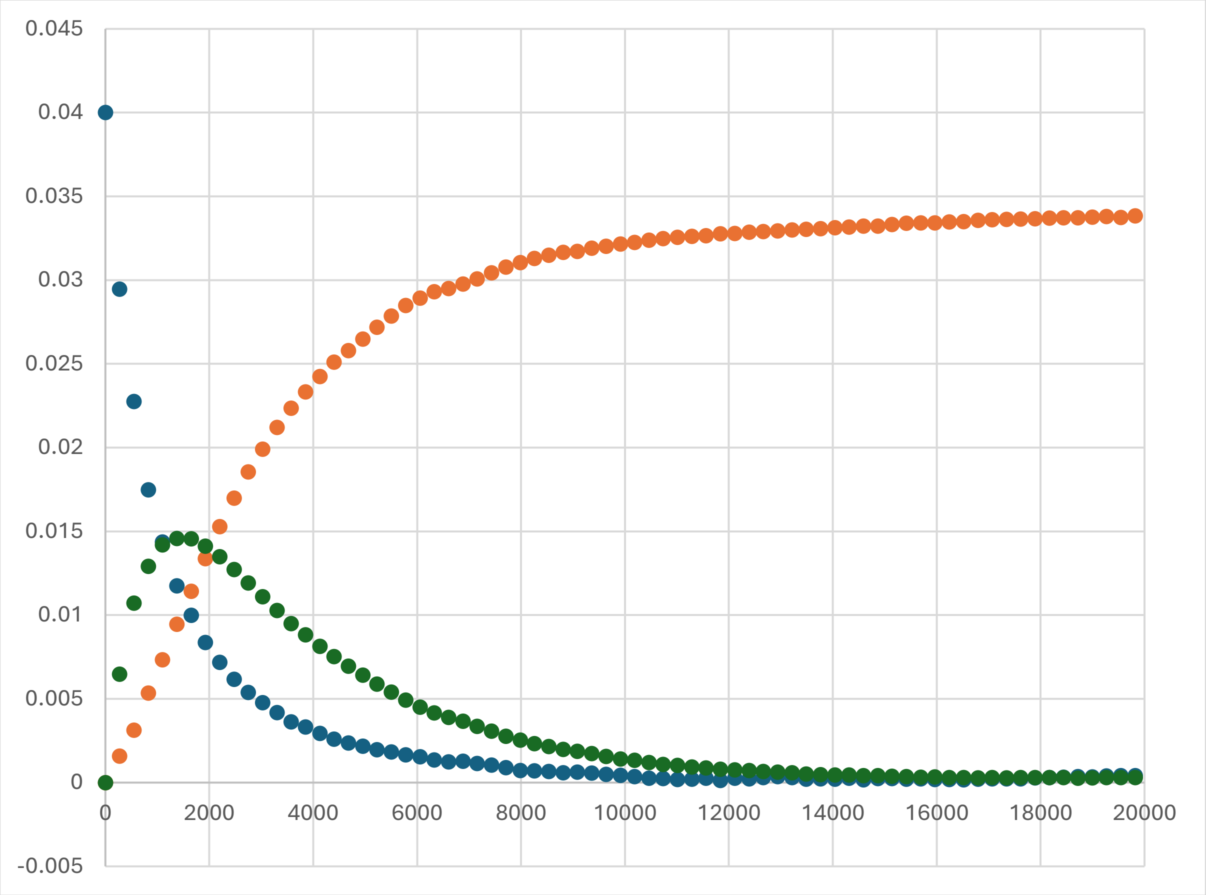

Fig. S1: Reaction Profile **S13** → **S14** + **S15** (N-Me)

The data was baseline corrected (blc) after integration by subtracting an average value obtained from the steady state region (>16000 s). Following integrals were used: SM (C$H_a$$H_b$ at 4.739 – 4.638, blc −0.0019), [2,3] (=CH_2, both diastereomers/2, 5.226 – 5.117 + 4.951 – 4.865, blc −0.0029), [1,2] (CH_2-C*H*=, 5.715 – 5.624, blc 0

t / s	SM	[2,3]	[1,2]
**0**	0.040000000	0.000000000	0.000000000
**273**	0.029464282	0.006470969	0.001581163
**549**	0.022753073	0.010721423	0.003130983
**825**	0.017473374	0.012917594	0.005346151
**1100**	0.014364868	0.014199426	0.007331981
**1375**	0.011742195	0.014567455	0.009446864
**1651**	0.009985281	0.014558602	0.011414944
**1926**	0.008357472	0.014118245	0.013378620
**2201**	0.007171341	0.013484831	0.015284287
**2476**	0.006156108	0.012726073	0.016972340
**2752**	0.005386651	0.011913852	0.018547734
**3027**	0.004771126	0.011104613	0.019909702
**3302**	0.004180777	0.010278000	0.021207695
**3577**	0.003627858	0.009498294	0.022341778
**3852**	0.003323563	0.008818329	0.023321323

**4128**	0.002935800	0.008136773	0.024242642
**4403**	0.002593880	0.007520468	0.025098214
**4678**	0.002358939	0.006950018	0.025795058
**4954**	0.002165862	0.006405318	0.026484784
**5229**	0.001971246	0.005881281	0.027182397
**5504**	0.001836743	0.005398582	0.027853643
**5780**	0.001659725	0.004915594	0.028482976
**6056**	0.001550045	0.004499712	0.028928386
**6331**	0.001358930	0.004161213	0.029308933
**6607**	0.001244541	0.003882761	0.029495871
**6882**	0.001278377	0.003670017	0.029763717
**7157**	0.001133791	0.003358274	0.030061739
**7432**	0.001037150	0.003072273	0.030430930
**7708**	0.000896694	0.002773674	0.030784837
**7984**	0.000723708	0.002543967	0.031045598
**8260**	0.000694258	0.002331709	0.031286011

**8535**	0.000663342	0.002152879	0.031492122
**8810**	0.000585067	0.001971614	0.031665586
**9085**	0.000623748	0.001857047	0.031718248
**9361**	0.000571363	0.001740761	0.031908674
**9636**	0.000493483	0.001555408	0.032013963
**9912**	0.000433194	0.001414052	0.032143786
**10188**	0.000362301	0.001324645	0.032251077
**10463**	0.000256593	0.001196969	0.032374468
**10737**	0.000238406	0.001083030	0.032486650
**11012**	0.000191785	0.001027083	0.032549270
**11288**	0.000196203	0.000938550	0.032608016
**11563**	0.000258403	0.000874005	0.032658688
**11838**	0.000127066	0.000802063	0.032755864
**12114**	0.000257826	0.000760363	0.032790290
**12389**	0.000231592	0.000714355	0.032866697
**12665**	0.000292155	0.000658436	0.032898173
**12940**	0.000349367	0.000616365	0.032930634
**13215**	0.000305993	0.000579977	0.033001581
**13490**	0.000208218	0.000510594	0.033032328
**13765**	0.000220235	0.000477588	0.033076232
**14041**	0.000206939	0.000460821	0.033133035

**14316**	0.000266969	0.000453431	0.033167294
**14591**	0.000169498	0.000420233	0.033219151
**14866**	0.000238255	0.000419741	0.033227109
**15142**	0.000238252	0.000381586	0.033323049
**15417**	0.000202085	0.000353721	0.033387479
**15692**	0.000224992	0.000324740	0.033418421
**15968**	0.000177916	0.000334248	0.033419909
**16243**	0.000178214	0.000302262	0.033475459
**16519**	0.000159216	0.000291723	0.033497049
**16794**	0.000196712	0.000276397	0.033568604
**17069**	0.000221526	0.000301150	0.033610419
**17344**	0.000227797	0.000276123	0.033626192
**17619**	0.000222388	0.000307212	0.033636443
**17894**	0.000289315	0.000299444	0.033663034
**18169**	0.000298753	0.000301893	0.033705685
**18444**	0.000312297	0.000295124	0.033718047
**18719**	0.000365558	0.000264681	0.033712411
**18994**	0.000332602	0.000277289	0.033749930
**19270**	0.000397073	0.000308350	0.033789669
**19546**	0.000409693	0.000296182	0.033747893
**19821**	0.000411091	0.000291942	0.033830181

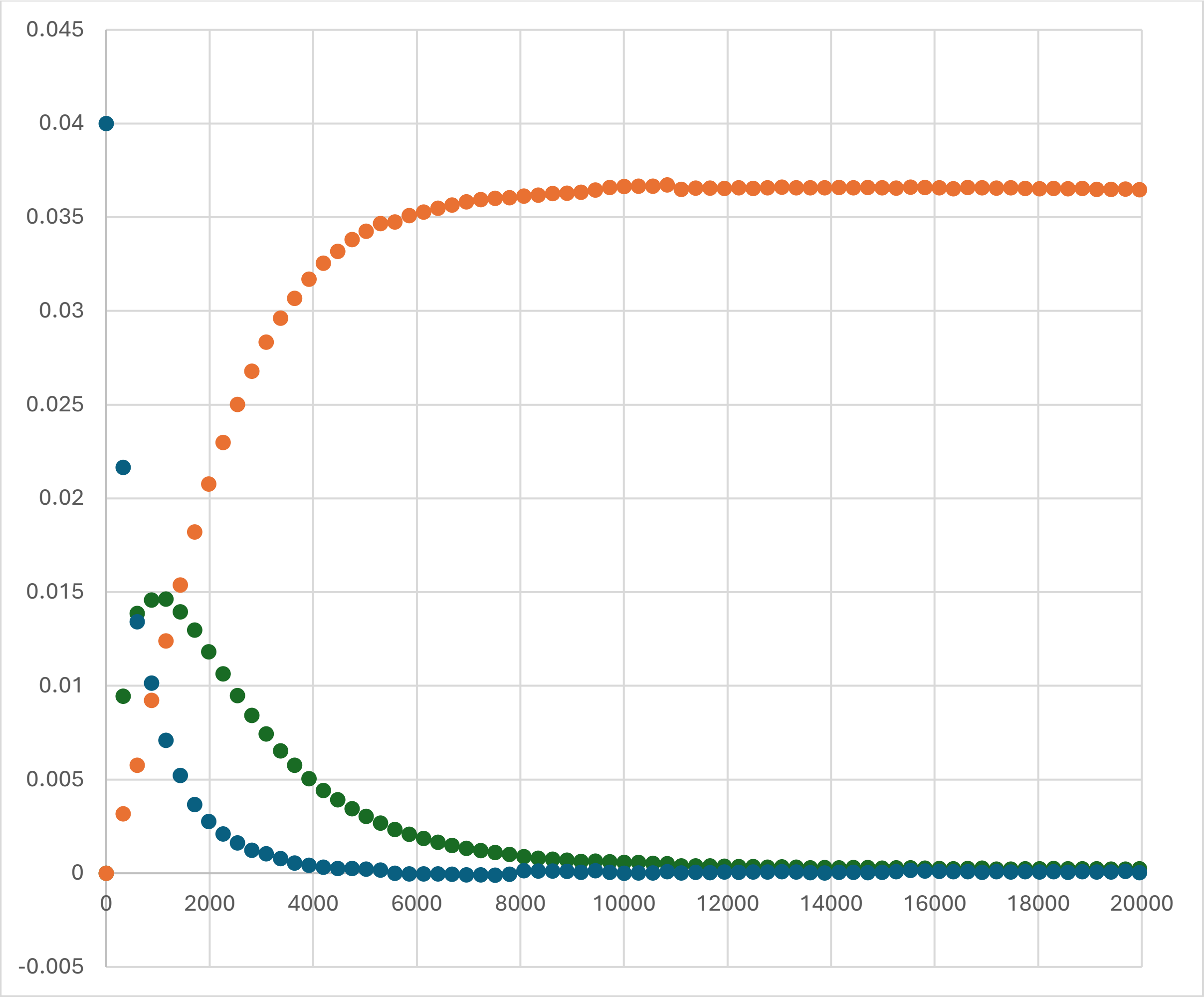

Fig. S2: Reaction Profile **S16** → **S17** + **S18** (N-Bn)

The data was baseline corrected (blc) after integration by subtracting an average value obtained from the steady state region (>15000 s). Following integrals were used: SM (average of CH_2-C*H*= at 6.030 – 5.965 and =C-C*H*$_3$ /3 at 1.908 – 1.846, blc −0.0021), [2,3] ((C*H*=CH_2 at 5.278 – 5.133 and CH=CH$_a$*H*$_b$ at 4.907 – 4.842 (half signal *2))/2, blc −0.0018), [1,2] (CH_2-C*H*= at 5.562 – 5.475, blc 0).

t [s]	SM	[2,3]	[1,2]
**0**	0.04000000	0.00000000	0.00000000
**327**	0.02165198	0.00945252	0.00317381
**603**	0.01340876	0.01386129	0.00575879
**879**	0.01015163	0.01458256	0.00921665
**1155**	0.00709796	0.01462231	0.01239815
**1430**	0.00521604	0.01394544	0.01537119
**1707**	0.00365649	0.01296888	0.01821360
**1984**	0.00276648	0.01181854	0.02075936
**2260**	0.0021004	0.01063168	0.02297867
**2537**	0.00162049	0.00948266	0.02500840
**2813**	0.00122812	0.00841419	0.02678375
**3089**	0.00104114	0.00743496	0.02834372
**3366**	0.00078787	0.00652550	0.02961552

**3642**	0.00053576	0.00576418	0.03067528
**3919**	0.00042543	0.00503764	0.03169997
**4195**	0.00032793	0.00441790	0.03254909
**4472**	0.00024582	0.00392077	0.03318246
**4748**	0.00024598	0.00344362	0.03380390
**5024**	0.00022332	0.00304135	0.03424835
**5301**	0.00017676	0.00268387	0.03465901
**5578**	0.00000053	0.00233408	0.03474912
**5855**	-0.00003187	0.00207447	0.03508254
**6130**	-0.00002835	0.00186130	0.03528157
**6407**	-0.00003168	0.00165358	0.03548486
**6683**	-0.00005604	0.00148068	0.03564857
**6960**	-0.00008127	0.00132382	0.03582039
**7236**	-0.00008188	0.00120116	0.03593976

**7514**	-0.00010727	0.00110456	0.03601571
**7791**	-0.00005068	0.00099728	0.03605088
**8068**	0.00013942	0.00088494	0.03612013
**8345**	0.00012428	0.00079890	0.03618694
**8621**	0.00012470	0.00074534	0.03627021
**8898**	0.00009367	0.00070134	0.03628818
**9174**	0.00005744	0.00062772	0.03633973
**9452**	0.00012744	0.00064955	0.03645300
**9728**	0.00004566	0.00061437	0.03659118
**10005**	0.00000149	0.00057932	0.03664720
**10281**	0.00001777	0.00057615	0.03664959
**10558**	0.00001464	0.00052267	0.03666293
**10834**	0.00007811	0.00050257	0.03672470
**11110**	0.00002405	0.00039381	0.03648381
**11387**	0.00004716	0.00039353	0.03656017
**11665**	0.00003484	0.00039075	0.03655431
**11942**	0.00006471	0.00036869	0.03654147
**12219**	0.00004932	0.00034935	0.03657260
**12495**	0.00007509	0.00034897	0.03654293
**12771**	0.00007148	0.00031623	0.03656687
**13049**	0.00007783	0.00033313	0.03660724
**13325**	0.00059278	0.00032050	0.03657108
**13601**	0.00002870	0.00029517	0.03657940

**13877**	0.00000779	0.00031260	0.03657241
**14154**	0.00005406	0.00028232	0.03659305
**14431**	0.00005025	0.00030949	0.03657025
**14707**	0.00003363	0.00030245	0.03658529
**14984**	0.00004301	0.00027052	0.03657967
**15261**	0.00008038	0.00028660	0.03656013
**15537**	0.00014809	0.00029359	0.03660323
**15813**	0.00012520	0.00027285	0.03658862
**16089**	0.00009394	0.00026274	0.03656886
**16365**	0.00009063	0.00024522	0.03652782
**16642**	0.00007595	0.00025036	0.03658687
**16918**	0.00005086	0.00026911	0.03657340
**17194**	0.00007631	0.00021873	0.03655226
**17471**	0.00006317	0.00022443	0.03657266
**17747**	0.00008669	0.00023759	0.03653992
**18024**	0.00005871	0.00024325	0.03652693
**18300**	0.00007664	0.00025138	0.03653034
**18577**	0.00005232	0.00023580	0.03651355
**18854**	0.00007946	0.00023193	0.03654505
**19130**	0.00006150	0.00023376	0.03649025
**19408**	0.00006644	0.00021611	0.03648274
**19684**	0.00009698	0.00021427	0.03649739
**19960**	0.00004049	0.00023672	0.03646222

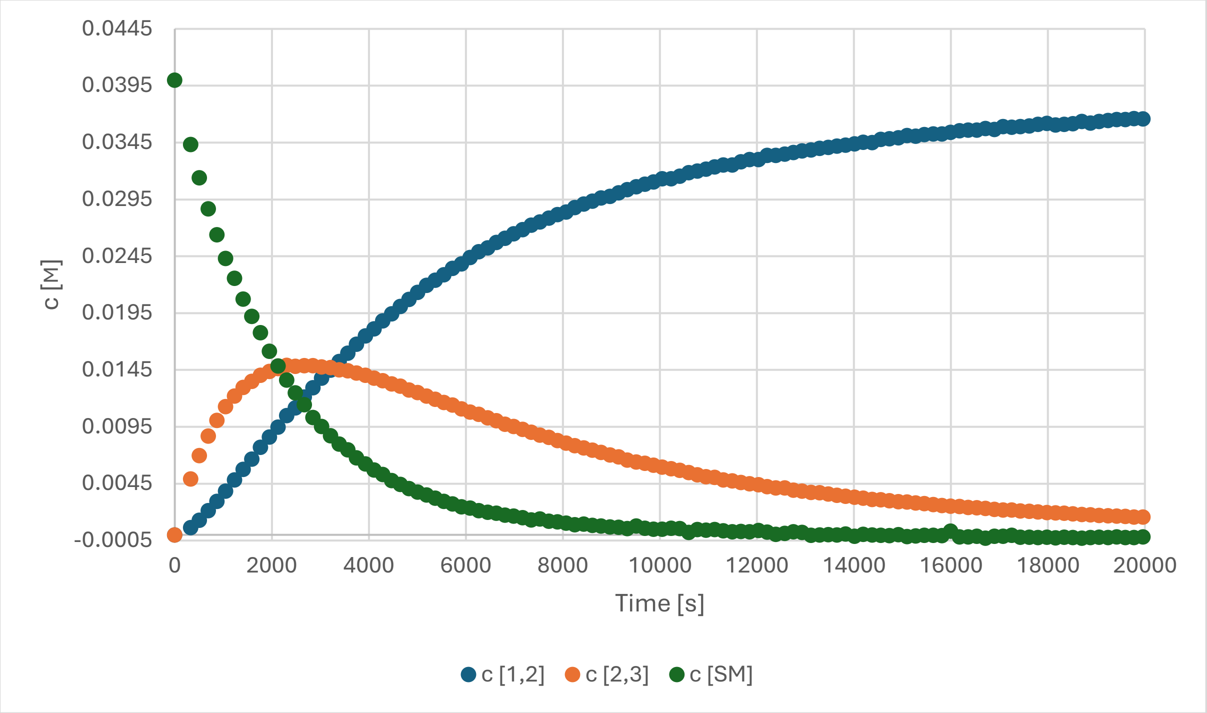

Fig. S3: Reaction Profile **11** → **12** + **13** (N-CPh$_3$)

The data was baseline corrected (blc) before integration using the integrated tool within MestReNova. Following integrals were used: SM (=CH-CH_aH$_b$ at 4.538 – 4.433), [2,3] (=CH_aH$_b$ at 5.195 – 5.125), [1,2] (=CH_aH$_b$= at 5.5672 – 5.602).

t [s]	SM	[2,3]	[1,2]
**0**	0.04000000	0.00000000	0.00000000
**328**	0.03433844	0.00491761	0.00064525
**508**	0.03139171	0.00697093	0.00126885
**688**	0.02868309	0.00869455	0.00211662
**868**	0.02637700	0.01005664	0.00293976
**1048**	0.02431776	0.01127833	0.00384040
**1228**	0.02255398	0.01219798	0.00483695
**1408**	0.02071927	0.01297021	0.00576168
**1587**	0.01921400	0.01350749	0.00666283
**1767**	0.01778925	0.01404529	0.00769814
**1947**	0.01614412	0.01436779	0.00859797
**2127**	0.01484672	0.01461577	0.00947384
**2307**	0.01360946	0.01491437	0.01049083
**2487**	0.01248190	0.01482945	0.01116613
**2667**	0.01143458	0.01487657	0.01210928
**2847**	0.01032929	0.01487873	0.01293804
**3028**	0.00951545	0.01477057	0.01377966
**3208**	0.00870785	0.01471319	0.01449250
**3388**	0.00799259	0.01451366	0.01524118
**3568**	0.00746208	0.01441948	0.01598430
**3748**	0.00676652	0.01423466	0.01675748

**3929**	0.00622775	0.01402926	0.01748232
**4109**	0.00568882	0.01377633	0.01811999
**4288**	0.00531860	0.01354714	0.01881564
**4469**	0.00478532	0.01328058	0.01943158
**4648**	0.00442829	0.01308062	0.02009094
**4828**	0.00408060	0.01273065	0.02069877
**5008**	0.00376282	0.01250537	0.02131319
**5188**	0.00350079	0.01219620	0.02194398
**5368**	0.00322144	0.01193280	0.02239794
**5548**	0.00293718	0.01164592	0.02288417
**5728**	0.00270678	0.01140418	0.02342193
**5908**	0.00246474	0.01107647	0.02384072
**6088**	0.00234253	0.01080368	0.02439264
**6268**	0.00211411	0.01058854	0.02489867
**6448**	0.00197914	0.01029647	0.02523634
**6628**	0.00190919	0.01004078	0.02571223
**6808**	0.00173985	0.00972639	0.02608679
**6988**	0.00163395	0.00951973	0.02646287
**7168**	0.00149441	0.00927293	0.02685197
**7349**	0.00130121	0.00903071	0.02724729
**7529**	0.00138895	0.00876499	0.02752327
**7709**	0.00119993	0.00855832	0.02785504

**7889**	0.00113156	0.00829221	0.02815805
**8069**	0.00102243	0.00805485	0.02839079
**8249**	0.00088418	0.00783898	0.02877108
**8429**	0.00093561	0.00765441	0.02908590
**8609**	0.00082577	0.00743788	0.02933512
**8789**	0.00076915	0.00725817	0.02962170
**8970**	0.00069910	0.00703588	0.02977547
**9150**	0.00066210	0.00684634	0.03009134
**9330**	0.00054058	0.00657995	0.03035556
**9510**	0.00076098	0.00639756	0.03060547
**9690**	0.00057084	0.00629398	0.03084766
**9870**	0.00049045	0.00613025	0.03103119
**10050**	0.00048028	0.00596332	0.03130327
**10232**	0.00058258	0.00581007	0.03133016
**10412**	0.00055223	0.00566366	0.03155227
**10592**	0.00019912	0.00548021	0.03185066
**10772**	0.00046645	0.00526638	0.03198682
**10952**	0.00039998	0.00511431	0.03215373
**11132**	0.00045577	0.00505018	0.03236217
**11311**	0.00034182	0.00483742	0.03253101
**11491**	0.00026934	0.00473140	0.03251626
**11672**	0.00030858	0.00460754	0.03280341
**11852**	0.00027103	0.00450204	0.03299416
**12032**	0.00038476	0.00441310	0.03299859
**12212**	0.00024308	0.00423808	0.03336423
**12392**	0.00005642	0.00413098	0.03338552
**12573**	0.00013153	0.00411593	0.03347907
**12753**	0.00027920	0.00391739	0.03363427
**12933**	0.00019978	0.00383193	0.03376902
**13113**	-0.00002936	0.00373070	0.03383634
**13292**	-0.00000910	0.00369290	0.03400332
**13472**	0.00001751	0.00358895	0.03406586
**13652**	-0.00000629	0.00348060	0.03419743
**13832**	0.00006056	0.00340625	0.03427549
**14014**	-0.00012571	0.00331903	0.03438120
**14194**	0.00003483	0.00322686	0.03451309
**14374**	-0.00000985	0.00311212	0.03450158
**14554**	-0.00004454	0.00308235	0.03478746
**14735**	-0.00006103	0.00300632	0.03482683
**14915**	0.00001143	0.00292195	0.03491322
**15095**	-0.00014587	0.00287877	0.03510574
**15275**	-0.00009285	0.00280198	0.03506048
**15455**	-0.00005241	0.00273894	0.03520381
**15635**	-0.00004644	0.00267007	0.03525248
**15815**	-0.00006948	0.00258703	0.03526917
**15995**	0.00031816	0.00251842	0.03541134

**16175**	-0.00017724	0.00247845	0.03552909
**16355**	-0.00018704	0.00241364	0.03558144
**16535**	-0.00013783	0.00236737	0.03558831
**16715**	-0.00030517	0.00232262	0.03573172
**16895**	-0.00012681	0.00224517	0.03566039
**17075**	-0.00011758	0.00218779	0.03590640
**17255**	-0.00005064	0.00218308	0.03583818
**17435**	-0.00021897	0.00211074	0.03591680
**17617**	-0.00019931	0.00205888	0.03596084
**17797**	-0.00023084	0.00201349	0.03609893
**17977**	-0.00021185	0.00195522	0.03617531
**18157**	-0.00026514	0.00194033	0.03604127
**18337**	-0.00020441	0.00189320	0.03611559
**18517**	-0.00024121	0.00184797	0.03616730
**18697**	-0.00029892	0.00179477	0.03636108
**18877**	-0.00022758	0.00177064	0.03621709
**19058**	-0.00020885	0.00171316	0.03635911
**19238**	-0.00023763	0.00167324	0.03644420
**19418**	-0.00019099	0.00165130	0.03651795
**19597**	-0.00022883	0.00162475	0.03652923
**19777**	-0.00023840	0.00157565	0.03661382
**19957**	-0.00018994	0.00156818	0.03659332
**20137**	-0.00022437	0.00154604	0.03659969
**20317**	-0.00029757	0.00148831	0.03660760
**20497**	-0.00021326	0.00146939	0.03664366
**20677**	-0.00024662	0.00144779	0.03672494
**20857**	-0.00025346	0.00141748	0.03667219
**21037**	-0.00039659	0.00135897	0.03676099
**21217**	-0.00032418	0.00134712	0.03677052
**21398**	-0.00027061	0.00131937	0.03695306
**21577**	-0.00022793	0.00131618	0.03701460
**21757**	-0.00026558	0.00128219	0.03696726
**21937**	-0.00024170	0.00125081	0.03693790
**22117**	-0.00020184	0.00122714	0.03699868
**22297**	-0.00032252	0.00119969	0.03703715
**22477**	-0.00022284	0.00117615	0.03714838
**22657**	-0.00034302	0.00118824	0.03702175
**22837**	-0.00031299	0.00114556	0.03708460
**23017**	-0.00024809	0.00111672	0.03717855
**23197**	-0.00021592	0.00110829	0.03716417
**23377**	-0.00020860	0.00110247	0.03716581
**23557**	-0.00021257	0.00108375	0.03715665
**23737**	-0.00022952	0.00105577	0.03717932
**23917**	-0.00022563	0.00104239	0.03722333
**24097**	-0.00021724	0.00102477	0.03720987
**24277**	-0.00018563	0.00100572	0.03723383

**24457**	-0.00022480	0.00097862	0.03723231
**24637**	-0.00020991	0.00097377	0.03731256
**24818**	-0.00022175	0.00095646	0.03740043
**24998**	-0.00021954	0.00094137	0.03733745
**25178**	-0.00020856	0.00092866	0.03739070
**25358**	-0.00019877	0.00090536	0.03736324
**25538**	-0.00020295	0.00090486	0.03753035
**25718**	-0.00021440	0.00090668	0.03732792
**25897**	-0.00017717	0.00086467	0.03740723
**26078**	-0.00020142	0.00087663	0.03739719
**26258**	-0.00019701	0.00085197	0.03747410
**26438**	-0.00020038	0.00078767	0.03749302
**26618**	-0.00022148	0.00080190	0.03752632
**26798**	-0.00019105	0.00080166	0.03753371
**26978**	-0.00019183	0.00080931	0.03762683
**27157**	-0.00020389	0.00078937	0.03758860
**27338**	-0.00019757	0.00077067	0.03755700
**27517**	-0.00018587	0.00076022	0.03755780
**27697**	-0.00016270	0.00074984	0.03765384
**27877**	-0.00016977	0.00072754	0.03763840
**28057**	-0.00020782	0.00071482	0.03765734
**28237**	-0.00017569	0.00069835	0.03760744
**28418**	-0.00019459	0.00071285	0.03774903
**28598**	-0.00016725	0.00068837	0.03767864
**28778**	-0.00017563	0.00068826	0.03778529
**28958**	-0.00016918	0.00067892	0.03763140
**29139**	-0.00017218	0.00067446	0.03764576
**29318**	-0.00017404	0.00066413	0.03770686
**29499**	-0.00018187	0.00065825	0.03774495
**29678**	-0.00017262	0.00065436	0.03770943
**29859**	-0.00015327	0.00063274	0.03777477
**30039**	-0.00017005	0.00063143	0.03781661
**30219**	-0.00017758	0.00061013	0.03781431
**30399**	-0.00015417	0.00063214	0.03775693
**30579**	-0.00016000	0.00059152	0.03783498
**30759**	-0.00015541	0.00059348	0.03781424
**30939**	-0.00015420	0.00057365	0.03778075
**31119**	-0.00015556	0.00058979	0.03784510
**31299**	-0.00015342	0.00057912	0.03787897
**31479**	-0.00015216	0.00057048	0.03783799
**31659**	-0.00015889	0.00056067	0.03788370
**31839**	-0.00014441	0.00055256	0.03783950
**32019**	-0.00014670	0.00056151	0.03782745
**32199**	-0.00015096	0.00055384	0.03790706
**32380**	-0.00015112	0.00053075	0.03794104
**32560**	-0.00015178	0.00053474	0.03790336

**32739**	-0.00014421	0.00052785	0.03793918
**32920**	-0.00015441	0.00051008	0.03794176
**33100**	-0.00014471	0.00052230	0.03787976
**33280**	-0.00013498	0.00051074	0.03783822
**33460**	-0.00013974	0.00051307	0.03788258
**33640**	-0.00015348	0.00049927	0.03808732
**33821**	-0.00014199	0.00047770	0.03795683
**34001**	-0.00014231	0.00049480	0.03802482
**34181**	-0.00014590	0.00048969	0.03799609
**34360**	-0.00013158	0.00045992	0.03788892
**34540**	-0.00013223	0.00047141	0.03803209
**34721**	-0.00014114	0.00046682	0.03805549
**34901**	-0.00013712	0.00045997	0.03805023
**35081**	-0.00014075	0.00041928	0.03802594
**35261**	-0.00012916	0.00046013	0.03798250
**35441**	-0.00014430	0.00044757	0.03792530
**35621**	-0.00013994	0.00044833	0.03804077
**35801**	-0.00012807	0.00044187	0.03803279
**35981**	-0.00013449	0.00042492	0.03806641
**36161**	-0.00012767	0.00043707	0.03813999
**36341**	-0.00012786	0.00041008	0.03802796
**36521**	-0.00012956	0.00041275	0.03805653
**36701**	-0.00012329	0.00041713	0.03801749
**36881**	-0.00013877	0.00040641	0.03804172
**37060**	-0.00013293	0.00040716	0.03802550
**37241**	-0.00013158	0.00038706	0.03807966
**37421**	-0.00013352	0.00039326	0.03807753
**37601**	-0.00013250	0.00039080	0.03803290
**37781**	-0.00012131	0.00042230	0.03812304
**37961**	-0.00012889	0.00038403	0.03800553
**38141**	-0.00012063	0.00032389	0.03807419
**38321**	-0.00012689	0.00036219	0.03805796
**38501**	-0.00012180	0.00038277	0.03806555
**38681**	-0.00011942	0.00037333	0.03807946
**38861**	-0.00012520	0.00033694	0.03798812
**39041**	-0.00011797	0.00038189	0.03812952
**39221**	-0.00012882	0.00035255	0.03808633
**39401**	-0.00012346	0.00037518	0.03835186
**39581**	-0.00012325	0.00035946	0.03812605
**39761**	-0.00012620	0.00035487	0.03807823
**39941**	-0.00011334	0.00036810	0.03812704
**40121**	-0.00012553	0.00036459	0.03808999
**40301**	-0.00011730	0.00035509	0.03807079
**40481**	-0.00011845	0.00035534	0.03814112
**40661**	-0.00012112	0.00034218	0.03811550
**40841**	-0.00012769	0.00033888	0.03809588

**41021**	-0.00012185	0.00027046	0.03822434
**41201**	-0.00010810	0.00034266	0.03812702
**41381**	-0.00011710	0.00032163	0.03818046
**41561**	-0.00010347	0.00033026	0.03813798
**41741**	-0.00011925	0.00034999	0.03815731
**41921**	-0.00010771	0.00033325	0.03813764
**42101**	-0.00011054	0.00030211	0.03824934
**42281**	-0.00011282	0.00032154	0.03815196
**42461**	-0.00011248	0.00033579	0.03818644
**42641**	-0.00011413	0.00032165	0.03810846
**42821**	-0.00011032	0.00030981	0.03813162
**43001**	-0.00011534	0.00033299	0.03822437
**43181**	-0.00012575	0.00030537	0.03816381
**43361**	-0.00010516	0.00032831	0.03806357
**43541**	-0.00011961	0.00028974	0.03814630
**43721**	-0.00010917	0.00026389	0.03817077
**43901**	-0.00010853	0.00031194	0.03820245
**44081**	-0.00011184	0.00026813	0.03809471
**44261**	-0.00011374	0.00027443	0.03810351
**44440**	-0.00009374	0.00026015	0.03814951
**44621**	-0.00010623	0.00025378	0.03815521
**44800**	-0.00011361	0.00025946	0.03822890

**44980**	-0.00010083	0.00023958	0.03818956
**45161**	-0.00010908	0.00026074	0.03819965
**45341**	-0.00011629	0.00025311	0.03820502
**45521**	-0.00011645	0.00025990	0.03826427
**45701**	-0.00010596	0.00028028	0.03814044
**45881**	-0.00011728	0.00023158	0.03815097
**46061**	-0.00011159	0.00026446	0.03816829
**46241**	-0.00010852	0.00022966	0.03809669
**46421**	-0.00011561	0.00022864	0.03815408
**46601**	-0.00011851	0.00024532	0.03811916
**46781**	-0.00011561	0.00023432	0.03815075
**46960**	-0.00010708	0.00025563	0.03820410
**47140**	-0.00010856	0.00023705	0.03824656
**47320**	-0.00012029	0.00024283	0.03817240
**47500**	-0.00011579	0.00025072	0.03821101
**47680**	-0.00011342	0.00025364	0.03812389
**47860**	-0.00011002	0.00023222	0.03817712
**48040**	-0.00010672	0.00021924	0.03821475
**48220**	-0.00011131	0.00022398	0.03814323
**48400**	-0.00011692	0.00021942	0.03809944

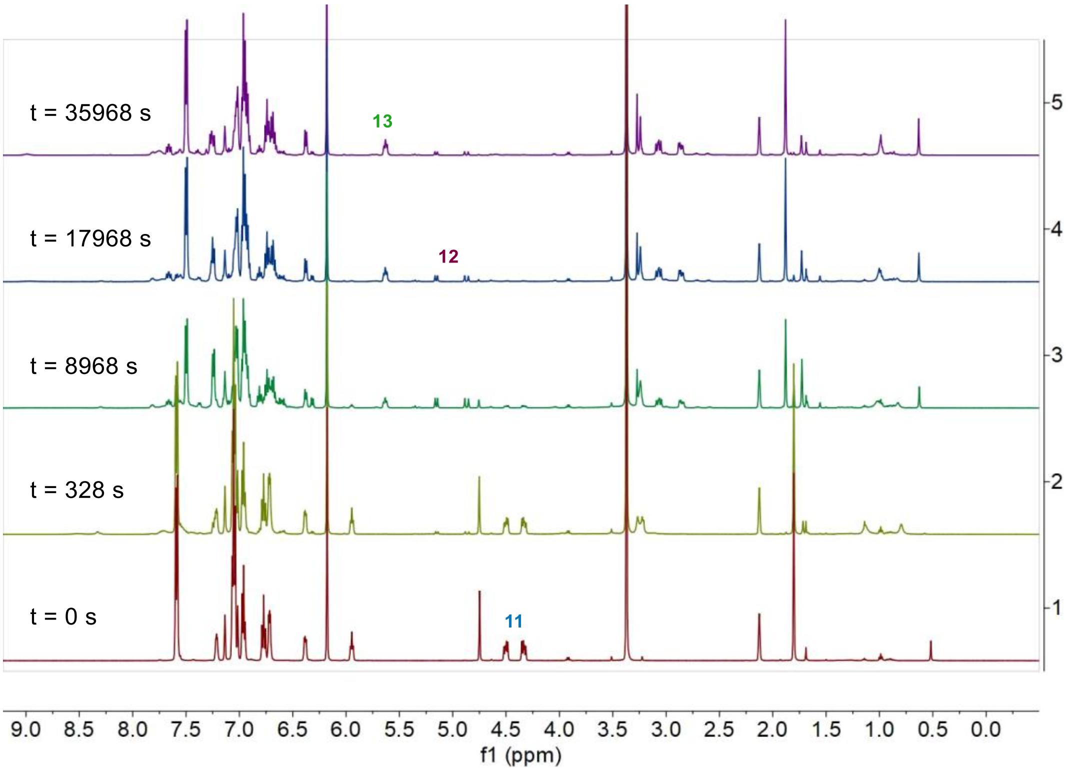

Fig. S4. Selected *in situ* ^{1}H NMR spectra for the monitoring of the [1,2]-rearrangement of the *N*-Tr substrate **11**, at different times

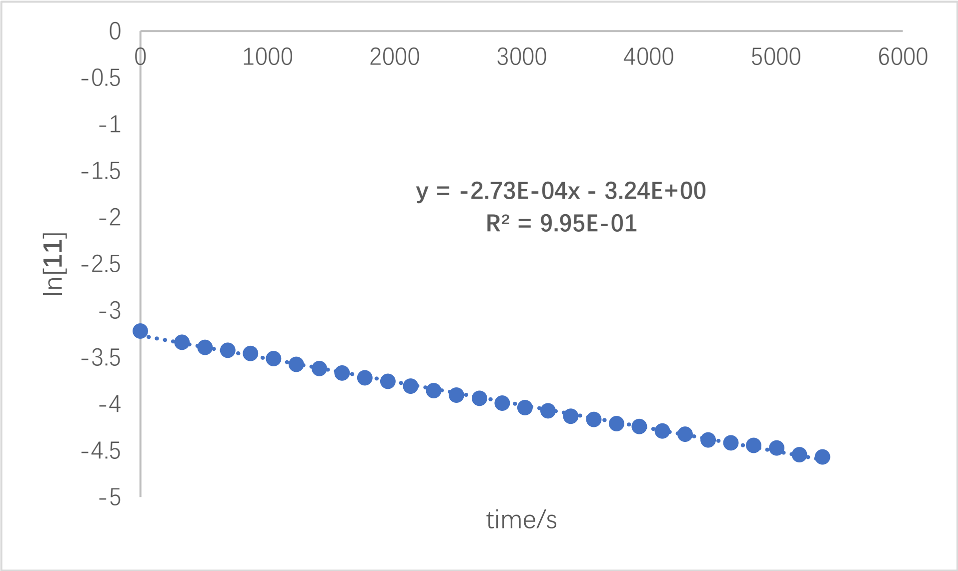

Fig. S5. First order decay of the *N*-CPh_3 substrate

Table S8: *In-situ* reaction monitoring for ester-bearing allylic ether **66** via ^{1}H NMR

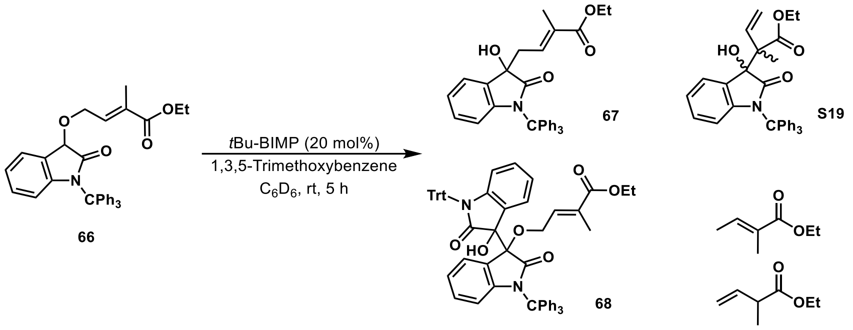

Entry	$t_{1/2}$ ([1,2]) [s]	t_{Cmax} ([2,3]) [s]
**1**	2960	3839

Table S8, Entry 1: To an NMR tube was added 1,3,5-trimethoxybenzene (2.6 mg, 0.016 mmol), ethyl (*E*)-4-((2-oxo-1-tritylindolin-3-yl)oxy)tiglate **66** (18.3 mg, 0.035 mmol) and *t*Bu-BIMP **7** (5.5 mg, 0.007 mmol). C_6D_6 was added and the sample was shook vigorously. The reaction was monitored using ^{1}H NMR occasionally over the period of 5 h.

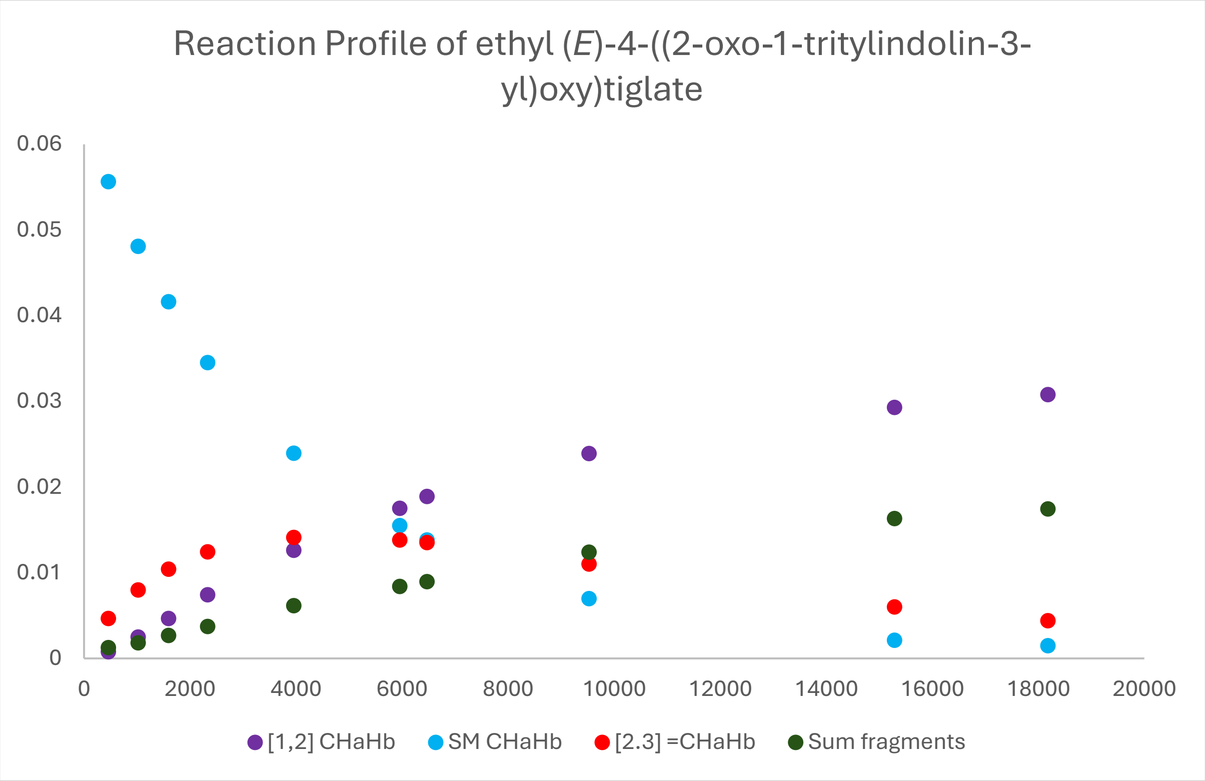

Fig. S6: Reaction Profile **66** → **S19** + **67** . *fragments based on tentative assignment of characteristic signals

t [s]	SM	[2,3]	[1,2]	Fragments
**463**	0.05564507	0.00465910	0.00078277	0.0012716
**1023**	0.04809253	0.00799629	0.00249922	0.0018350
**1597**	0.04163683	0.01041143	0.00466804	0.0026862
**2335**	0.03453515	0.01242809	0.00741852	0.0037335
**3957**	0.02396325	0.01410307	0.01262140	0.0061506
**5958**	0.01549137	0.01381587	0.01750893	0.0084166
**6469**	0.01381073	0.01353620	0.01891638	0.0089554
**9527**	0.00699083	0.01099967	0.02391557	0.0123856
**15290**	0.00213797	0.00600847	0.02927855	0.0163216
**18186**	0.00148282	0.00439533	0.03079245	0.0174408

5.2 Hammett plot analysis

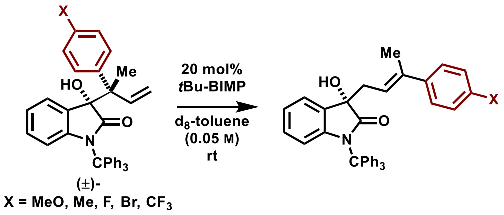

Procedure: To a solution of racemic-[2,3] Wittig rearrangement product (0.03 mmol) and 1,3,5-trimethoxybenzene (0.03 mmol) in toluene-d_8 (0.05 M) was added *t*Bu-BIMP (0.006 mmol) in NMR-tube, then the reaction was monitored by ^{1}H NMR spectroscopy.

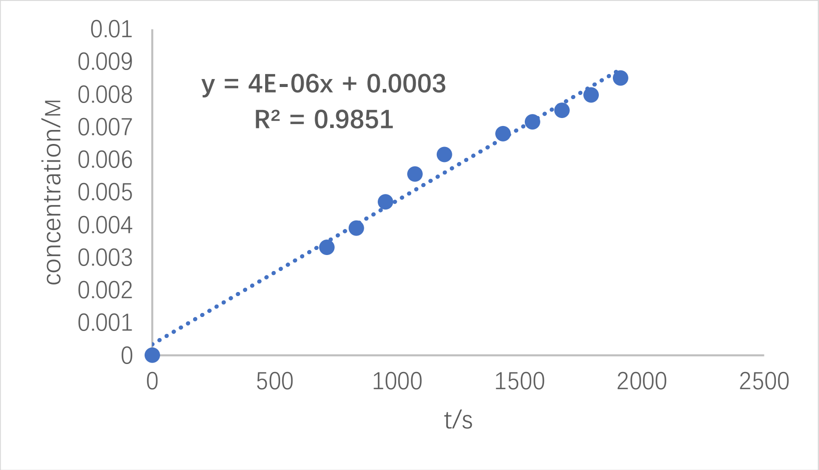

Fig. S7. Initial rate of the reaction, X = H

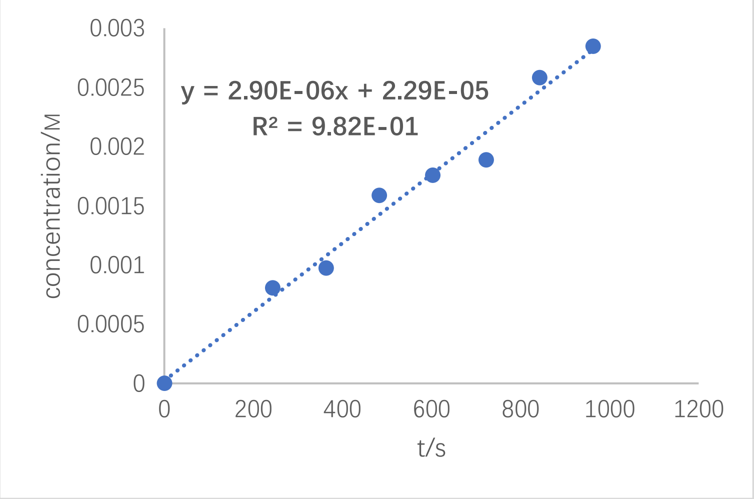

Fig. S8: Initial rate of the reaction, X = OMe

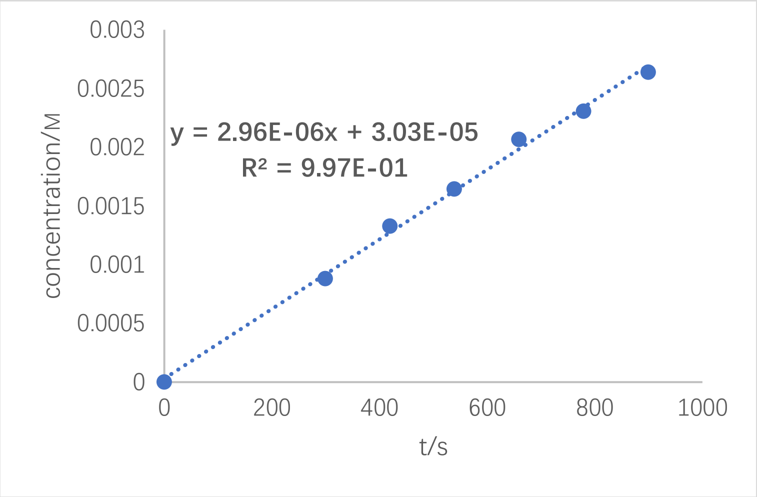

Fig. S9: Initial rate of the reaction, X = Me

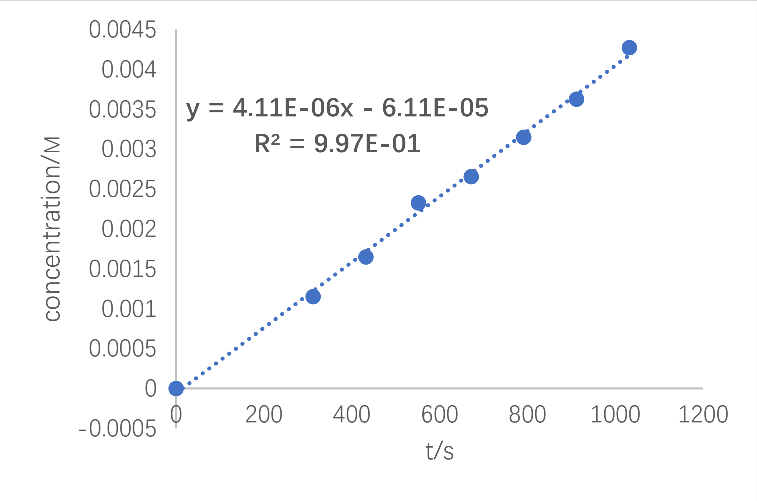

Fig. S10: Initial rate of the reaction, X = F

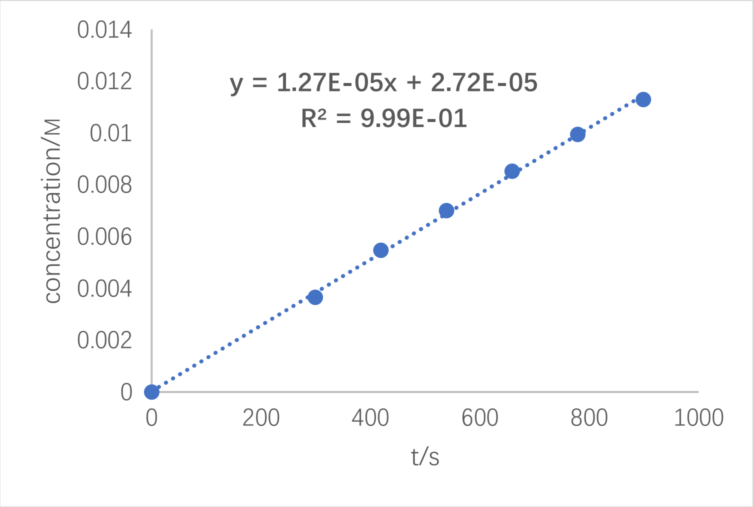

Fig. S11: Initial rate of the reaction, X = CF_3

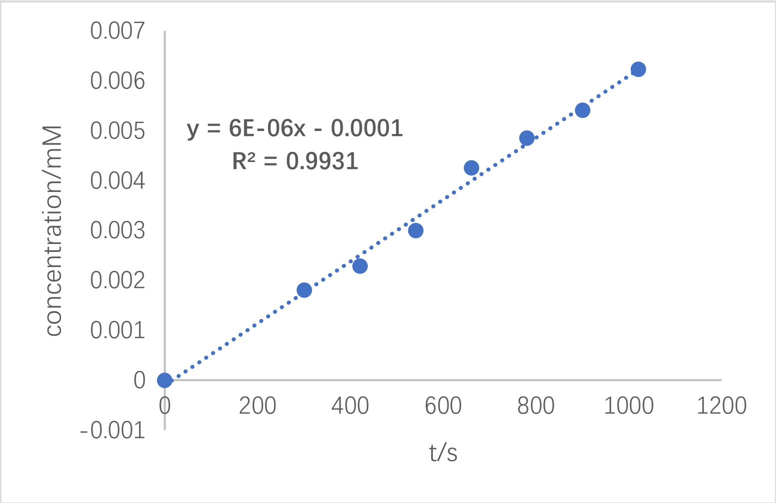

Fig. S12: Initial rate of the reaction, X = Br.

Table S9: Data of Hammett plot analysis

**Substituent**	**σ^-**	**$\log(K_X/K_H)$**
**4-F**	-0.03	0.011781
**4-Br**	0.25	0.19033
**4-Me**	-0.17	-0.13
**4-OMe**	-0.26	-0.169
**4-CF_3**	0.65	0.5017

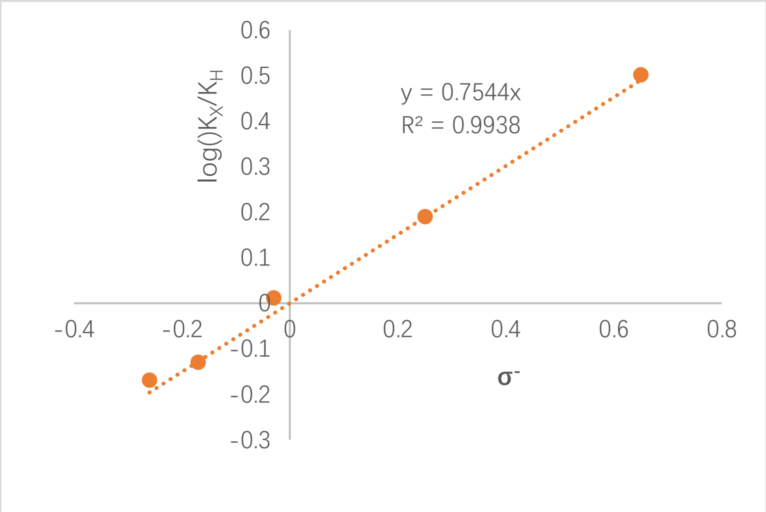

Fig. S13: Hammett analysis for conversion of [2,3]-intermediates to [1,2]-product with various aryl substituents.

5.3 Kinetic studies of 14 (89:11 dr) to 9 with racemic or enantiopure BIMPs

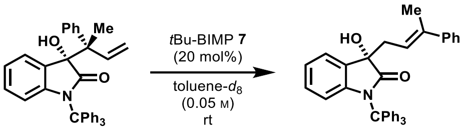

14 **9**

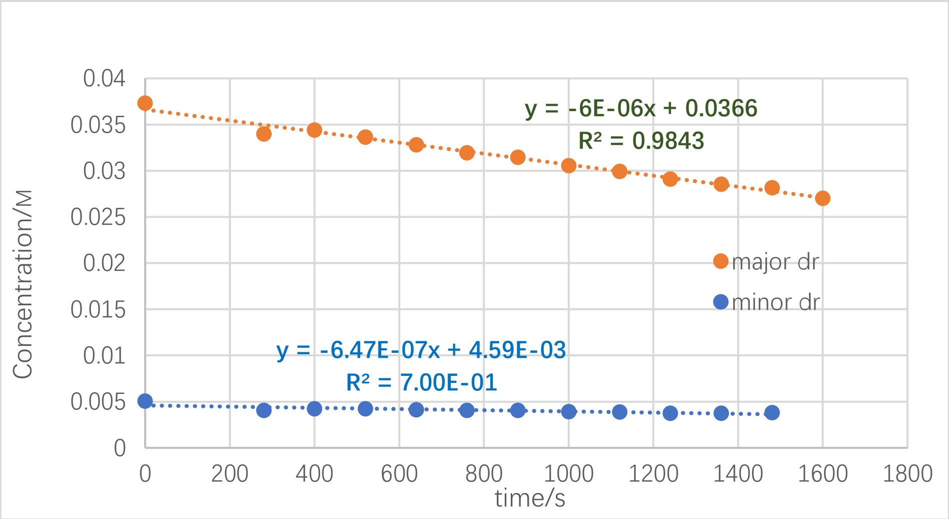

Fig. S14: Kinetic study of **14** with *t*Bu-BIMP **7** ($k_{(3S,1'S)\text{-}14}$ = 1.6×10^{-4} s^{-1}; $k_{(3S,1'R)\text{-}14}$ = 1.3×10^{-4} s^{-1}).

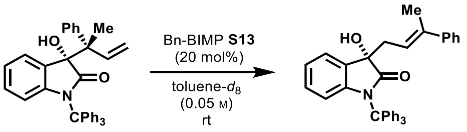

14 **9**

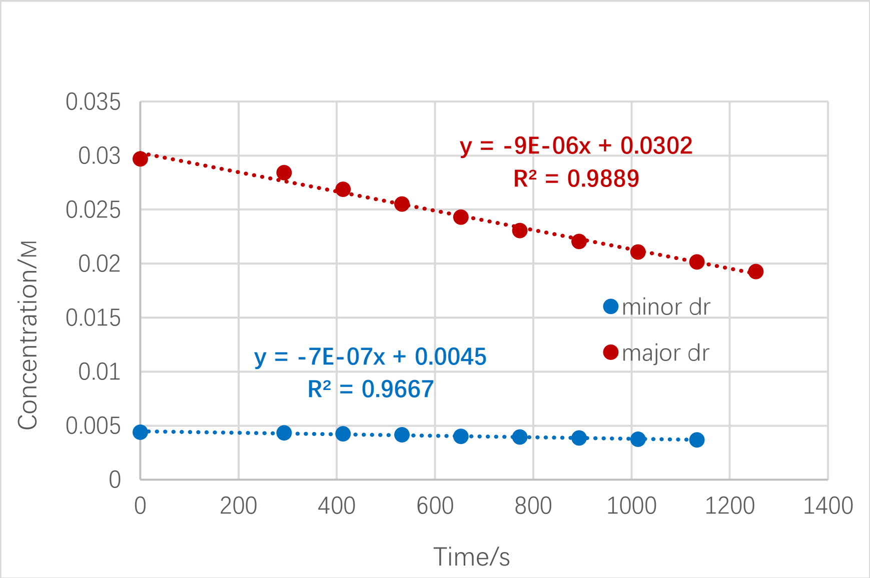

Fig. S15. Kinetic study of **14** with Bn-BIMP **S13** ($k_{(3S,1'S)\text{-}14}$ = 3.0×10^{-4} s^{-1}; $k_{(3S,1'R)\text{-}14}$ = 1.6×10^{-4} s^{-1}).

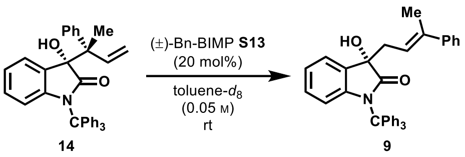

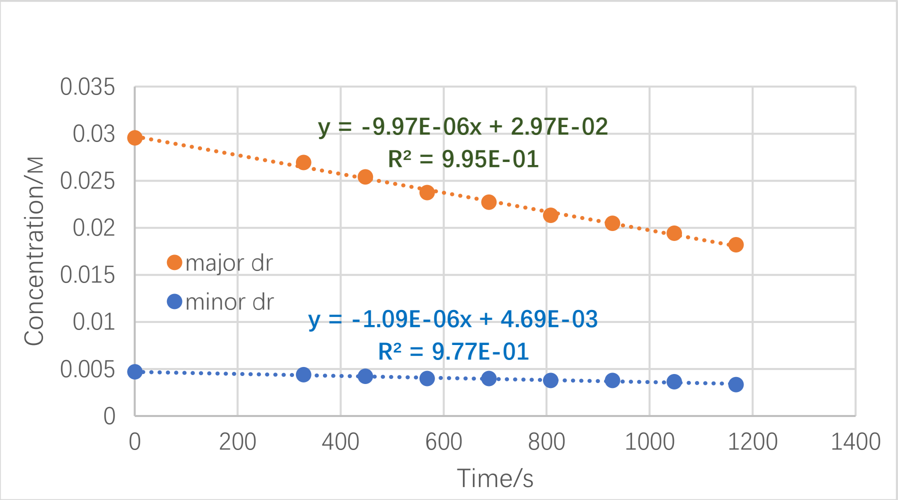

Fig. S16. Kinetic study of **14** with (±)-Bn-BIMP **S13** ($k_{(3S,1'S)\text{-}\mathbf{14}}$ = 3.3×10^{-4} s^{-1}; $k_{(3S,1'R)\text{-}\mathbf{14}}$ = 2.3×10^{-4} s^{-1}).

5.4 Fitting Kinetics Equations

Data from *in situ* reaction monitoring was used to obtain approximate barriers for each measured stage of the reaction. Assuming 1st order kinetics and no reverse reactions, based on **Scheme S11** rate equations can be derived as a function of time, *t,* and initial concentration of the starting material, $[A_0]$. Values for k_1, k_2 and k_3 were then optimised using stochastic gradient decent in Tensorflow v2.12.0[3], minimising the mean squared error between the predictions of equations (3), (4) and (5) and the experimental data. Rate constants k_1 and k_3 were optimised first, fitting them to the experimental concentrations of A using only equation (3). Once converged, k_2 was subsequently optimised for the concentration of B using equation (4). Once good initial values had been determined for k_1, k_2 and k_3, all rate constants were simultaneously optimised, fitting to the experimental concentrations of A, B and C utilising all three equations (see also **Fig. S17**). Values derived from this process were then converted to free energy barriers using the Eyring equation (6) to compare with computationally derived barriers (see *N*-substituent section).

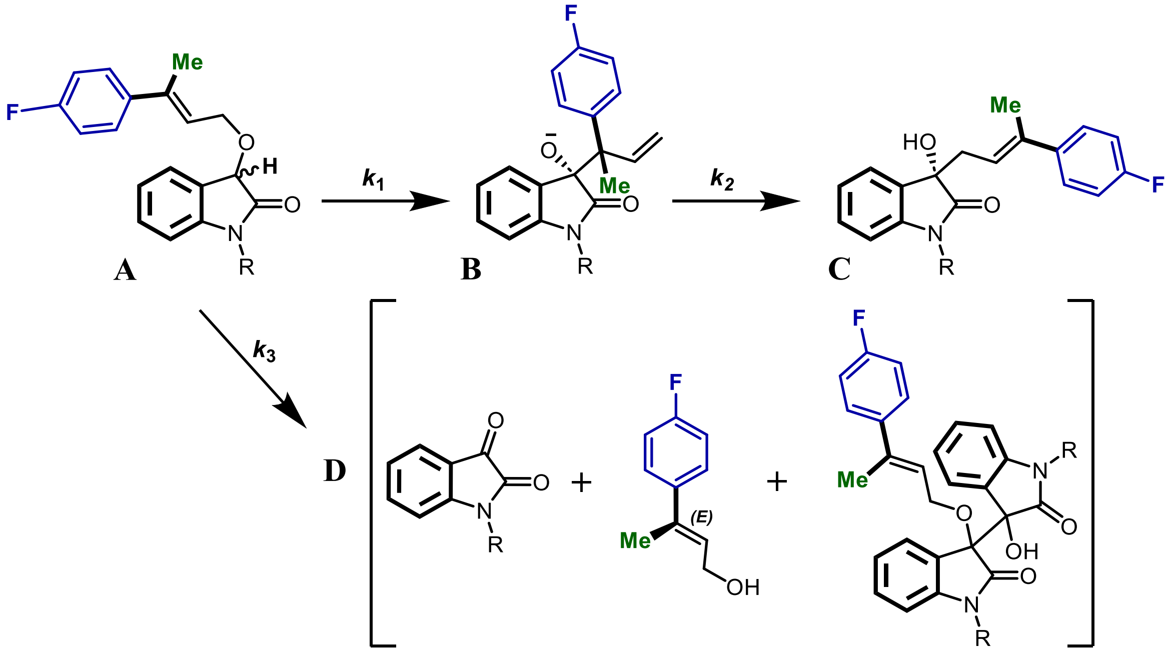

Scheme S11. Reaction scheme for *in situ* reaction monitoring (R = Me, Bn, Trityl).

$$[A] = [A_0]e^{-(k_1+k_3)t} \qquad \textbf{(3)}$$

$$[B] = \frac{[A_0]k_1}{k_2 - k_1 - k_3}\left(e^{-(k_1+k_3)t} - e^{-k_2 t}\right) \qquad \textbf{(4)}$$

$$[C] = \frac{[A_0]k_1}{k_1 + k_3}\left(1 - \frac{k_2 e^{-(k_1+k_3)t}}{k_2 - k_1 - k_3} + \frac{(k_1+k_3)e^{-k_2 t}}{k_2 - k_1 - k_3}\right) \qquad \textbf{(5)}$$

$$\Delta G^{\ddagger} = RT\left(\ln\left(\frac{k_B T}{hk}\right)\right) \qquad \textbf{(6)}$$

N-substituent	[2,3] experimental barrier (kcal mol^{-1})	[1,3] experimental barrier (kcal mol^{-1})
Methyl	21.39	21.58
Benzyl	21.03	21.35
Trityl	21.68	21.90

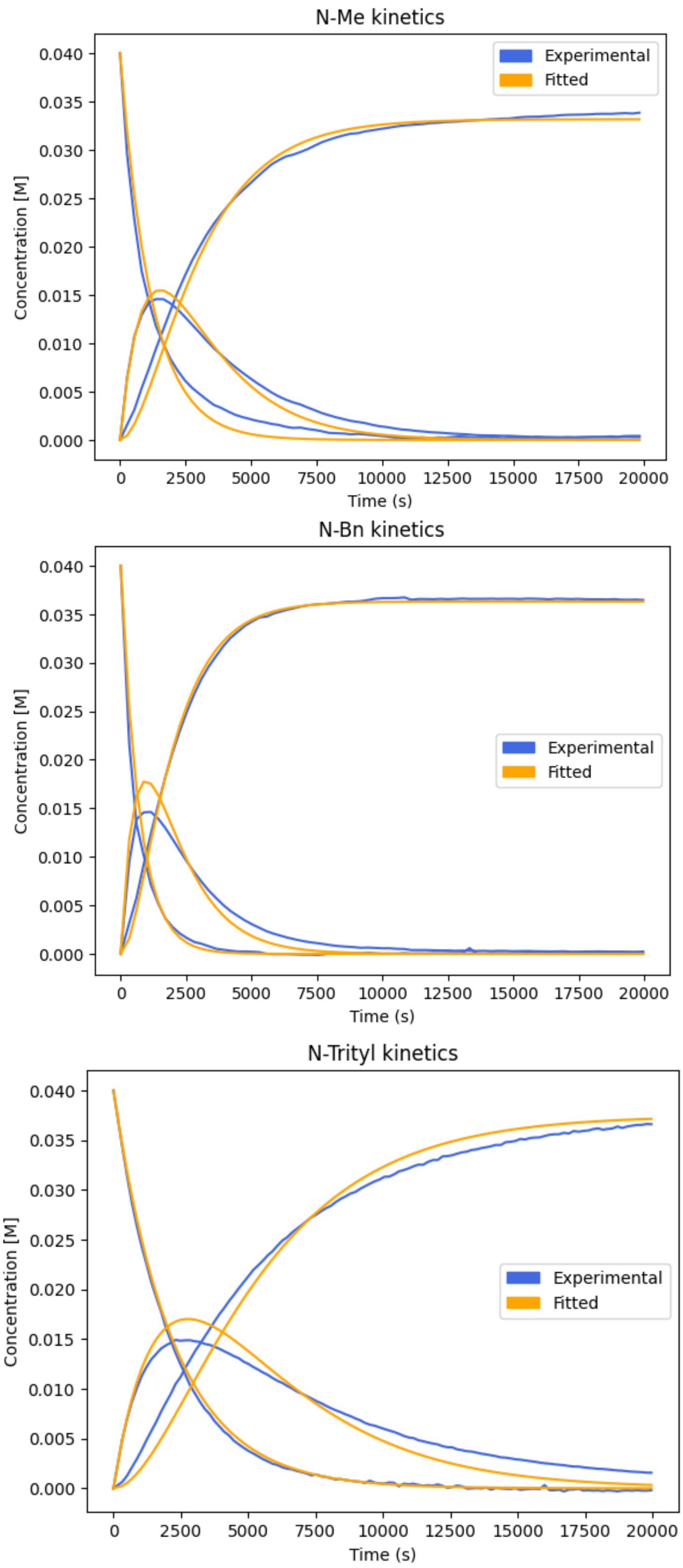

Fig. S17. Kinetics profiles with N-Me (top), N-Bn (middle) and N-Trityl (bottom).

6 Supplementary X-ray analysis details

X-ray quality crystals of **S24** were prepared by the vapour diffusion of dichloromethane into a methanol solution of the compound, while those of **S25** were prepared by the diffusion of hexane into a dichloromethane solution of the compound. X-ray diffraction data for compound **S24** and **S25** were collected at 173 K using a Rigaku MM-007HF High Brilliance RA generator/confocal optics [Cu Kα radiation (λ = 1.54187 Å)] with either an XtaLAB P200 (**S24**) or P100 (**S25**) diffractometer. Intensity data for both compounds were collected using either CrystalClear [4] (using ω steps and accumulating area detector images spanning at least a hemisphere of reciprocal space) or CrysAlisPro [5] (using a calculated data-collection strategy), and processed (including correction for Lorentz, polarization and absorption) using CrysAlisPro. Structures were solved by dual-space methods (SHELXT [6]) and refined by full-matrix least-squares against F^2 (SHELXL-2018/3 [7]). Non-hydrogen atoms were refined anisotropically, and carbon-bound hydrogen atoms were refined using a riding model. Hydrogen atoms bound to heteroatoms were located from the difference Fourier map and refined isotropically subject to a distance restraint. The structure of **S24** shows disorder in the orientation of the methyl and ethylene groups at C10, leading to the formation of two enantiopure diastereomers. The disorder was refined with some restraints to bond distances and thermal motion. All calculations were performed using the Olex2 [8] interface. Selected crystallographic data are presented in **Table S10**. CCDC 2305636-2305637 contains the supplementary crystallographic data for this paper. These data can be obtained free of charge from The Cambridge Crystallographic Data Centre via www.ccdc.cam.ac.uk/structures.

Table S10. Selected crystallographic data

Entry	**S24**	**S25**
formula	$C_{18}H_{17}NO_2$	$C_{17}H_{15}NO_2$
fw	279.32	265.30
crystal description	Colourless prism	Colourless plate
crystal size [mm^3]	0.20×0.15×0.10	0.28×0.15×0.05
space group	$P2_12_12_1$	$P2_12_12_1$

a [Å]	6.79230(6)	5.81666(6)
b [Å]	7.02749(5)	7.60297(6)
c [Å]	29.72211(19)	29.9904(2)
vol $[Å]^3$	1418.720(18)	1326.294(19)
Z	4	4
ρ (calc) $[g/cm^3]$	1.308	1.329
μ $[mm^{-1}]$	0.680	0.699
F(000)	592	560
reflections collected	16416	45274
independent reflections (R_{int})	2902 (0.0149)	2339 (0.0237)
parameters, restraints	210, 15	181, 1
GoF on F^2	1.077	1.097
R_1 $[I > 2\sigma(I)]$	0.0295	0.0236
wR_2 (all data)	0.0785	0.0607
largest diff. peak/hole $[e/Å^3]$	0.200, -0.204	0.099, -0.161
Flack parameter	0.00(5)	-0.03(2)

Table S11. Thermal ellipsoid plots (50 % probability ellipsoids) of the X-ray structures of (a) the major diastereomer of **S24**, (b) the minor diastereomer of **S24**, (c) **S25**

**(a) (3*S*,1'*S*)-S24**	**(b) (3*S*,1'*R*)-S24**
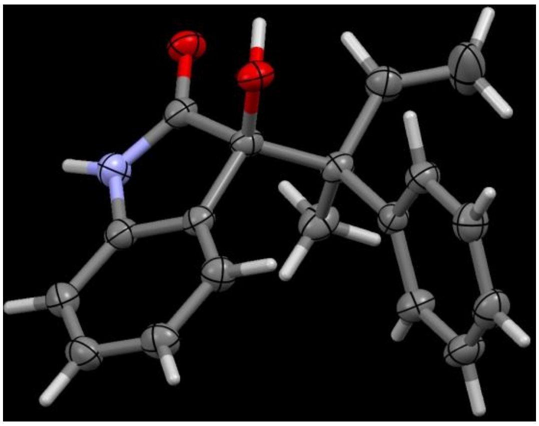	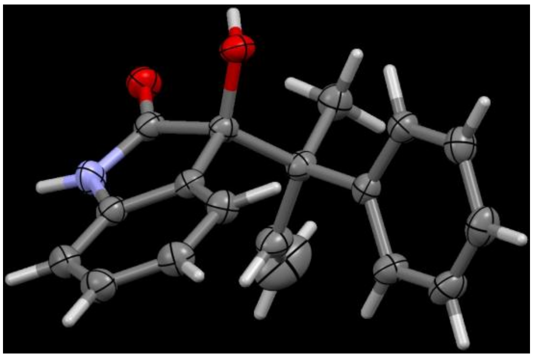
**(c) (2*S*,5*R*)-S25**	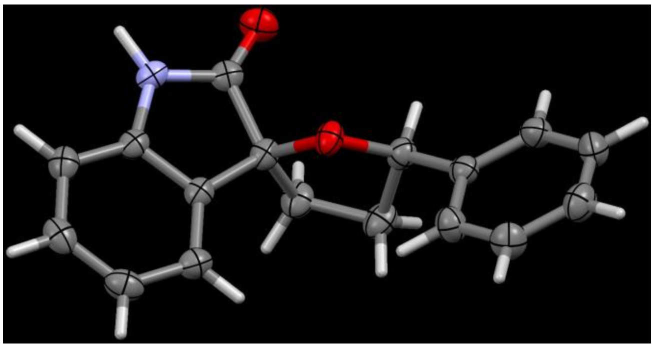

7 Supplementary Computational details

Conformational searches were performed with CREST[9] v2.12, using the GFN2-xTB[10] method; constraints were applied to the TS bond forming and breaking distances and all key hydrogen-bonding interactions. All other settings were left at their defaults. These searches were performed on the following structures (**Fig. 4A**, Ar = Ph, X = Me) across all eight binding modes (**Fig. S19**): (*S,S*)-, (*S,R*)-, (*R,S*)-, (*R,R*)-**TS-III**; (*S,S*)-**IV**; (*S,S*)-**TS-IV**. However, multiple CREST searches on the m23n1 binding mode for TS III (*R,S*) lead to violation of the hydrogen bond distance constraints (thus returning the incorrect binding mode) and were discarded. Each ensemble was then filtered using CREST's CREGEN function with an RMSD threshold of 1.25Å.

Geometry optimisations of the single lowest energy conformer from each of the CREST searches and in the exploratory calculations detailed in the main manuscript were performed with ONIOM[11,12] using Gaussian 16 revision C.01[13]. During these optimisations, some structures moved away from their initial binding modes. The ONIOM high layer was treated with the M06-2X functional[14] and the 6-31+G(d) basis set[15]; the low layer was treated with the AM1 semi-empirical method[16]. Previous studies have shown that there is good agreement between ONIOM and full DFT calculations for other organocatalytic systems[17-23]. The initial ONIOM layering scheme placed the Ph and Me groups of the allylic fragment in the high layer (**Fig. S18**, left). Within this scheme, calculations involving the [2,3]-sigmatropic rearrangement converged without issue. However, to address TS convergence problems when modelling the fragmentation step of the [1,3]-rearrangement, these groups were moved into the low layer (**Fig. S18**, right). Although convergence issues were still seen with this new layering, two of the eight conformers did converge to TSs. To make the calculated reaction barrier for this step directly comparable to that derived from *in situ* reaction monitoring data, we changed Ar = Ph to Ar = 4-FC_6H_4 in the lowest energy TS and pre-reaction complex and reoptimised. Despite the change in ONIOM layering, the computed barrier was in excellent agreement with that derived from experiment (see [1,3]-rearrangement section in the main manuscript).

Single point energy (SPE) calculations were used to correct the Gibbs free energy computed from the ONIOM optimisations[24]. These SPEs were performed using M06-2X with the Def2-TZVPP basis set[25] and the Integral Equation Formalism of the Polarizable Continuum Model (IEF-PCM)[26] implicit solvent model, with mesitylene as the solvent unless otherwise stated. It is generally understood that diffuse functions should be used to accurately describe systems with anions. However, previous work has shown that accurate energies can be computed without them when using Ahlrichs' (Def2-TZVP) or Dunning's (cc-pVTZ) triple-ζ basis sets[27]. Quasi-harmonic free energies were calculated with the GoodVibes Python package[28] using a temperature of 298.15 K, a concentration of 0.01 mol/L (to match the catalyst loading, **Fig. 2C**), Truhlar's quasi-harmonic treatment of entropy[29], the Head-Gordon enthalpy correction[30], and a vibrational scaling factor of 1.0[31] unless otherwise stated.

Computed structures were visualised with GaussView 6.1[32] and images of structures were generated in CYLview20[33]. Gaussian 16 output files are openly available in the Dataset for "The Catalytic Enantioselective [1,2]-Wittig Rearrangement Cascade of Allylic Ethers" in the University of Bath Research Data Archive at https://doi.org/10.15125/BATH-01337.

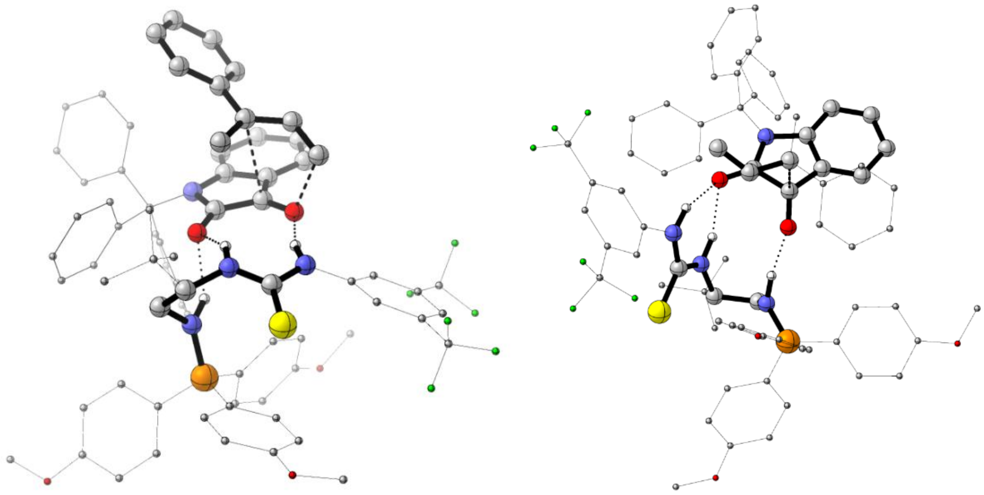

Fig. S18. ONIOM layering schemes for the [2,3]-rearrangement (left) and fragmentation (right) TSs. The ONIOM low layer is represented using smaller atoms. Note that there is a change between the two steps: the Ph and Me groups of the allylic fragment are placed in the high layer for the [2,3]-rearrangement and the low layer for the fragmentation step.

7.1 Computational details

7.1.1 Binding modes

Eight reasonable catalyst-substrate binding modes were considered (**Fig. S19**). For conformational searching, all hydrogen bonds were constrained. The binding mode codenames are based on which hydrogen interacts with which oxygen during the conformational searches. The allylic ether oxygen was labelled as *m* and the carbonyl oxygen as *n*. Hydrogens were numbered from 1 to 3 according to their distance from phosphorus.

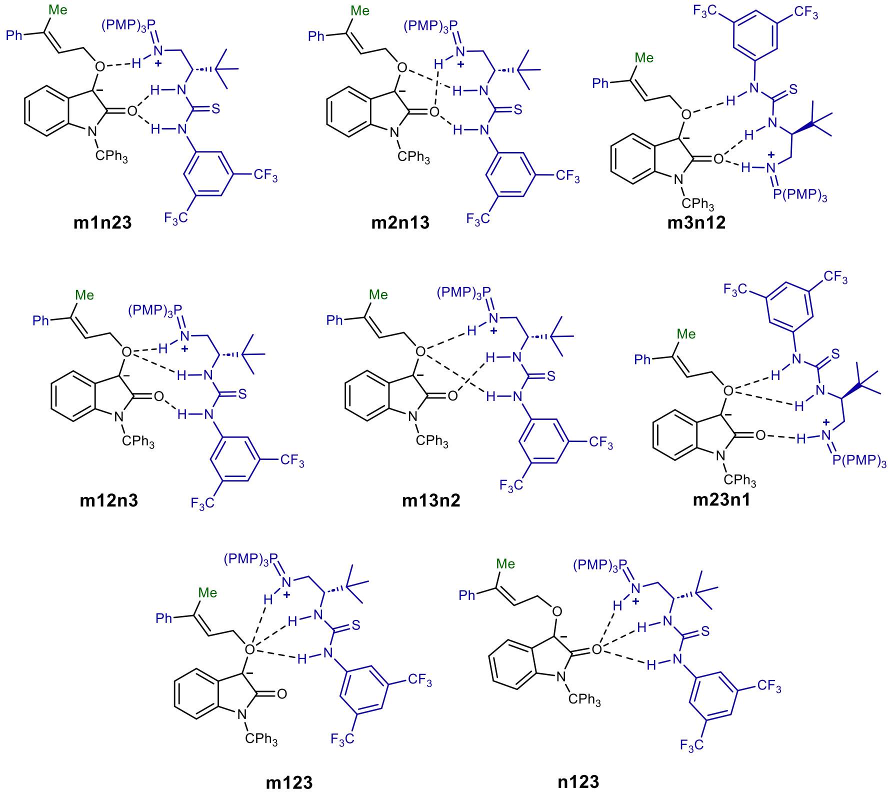

Fig. S19. Eight catalyst-substrate binding modes considered.

7.1.2 [2,3]-Sigmatropic rearrangement additional figures

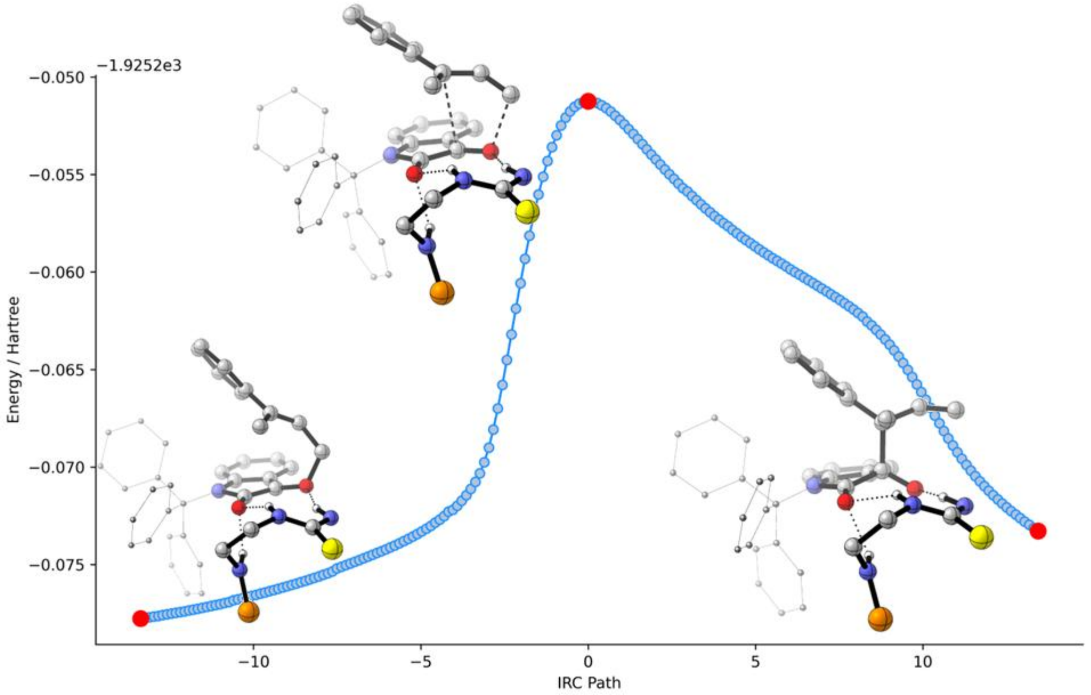

Fig. S20. IRC of *exo*-(*S*,*S*)-TS **III**. The ONIOM low layer atoms on [*t*Bu-BIMP-H]$^+$ have been omitted for clarity. Points along the IRC path that relate to the structures shown are highlighted in red.

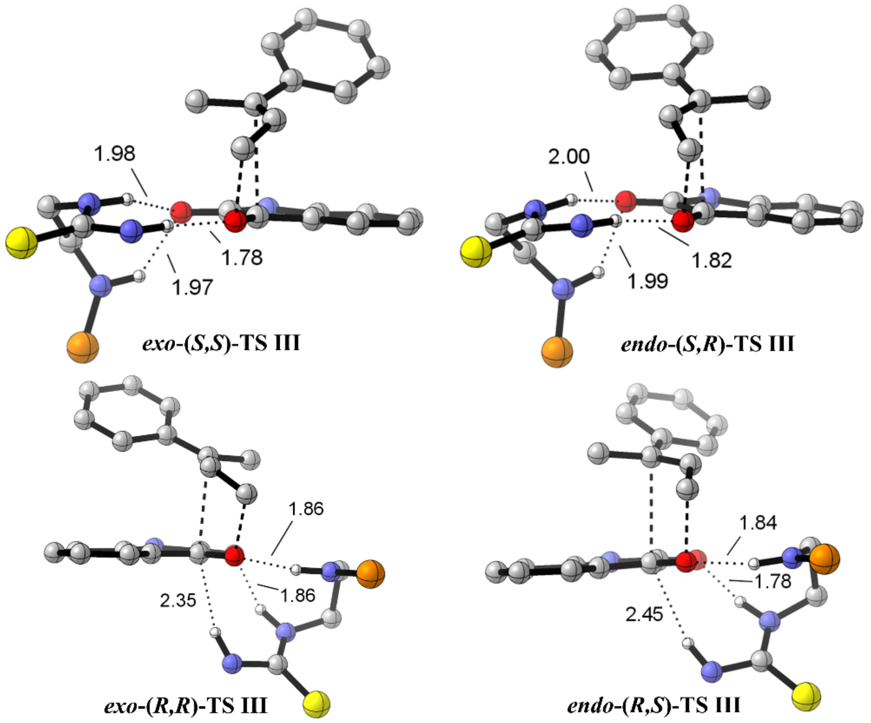

Fig. S21. Hydrogen bond distances in each of the lowest energy [2,3]-rearrangement TSs. ONIOM low layer atoms have been omitted for clarity.

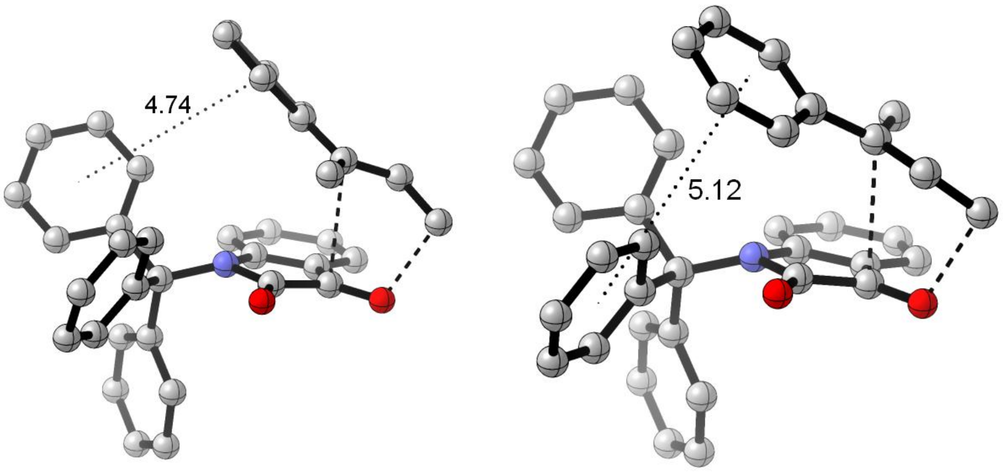

Fig. S22. Comparison of the closest π-ring centroid distances between *exo*-(*S*,*S*)-TS **III** (left) and *endo*-(*S*,*R*)-TS **III** (right). [*t*Bu-BIMP-H]$^+$ and ONIOM layering depiction have been omitted for clarity.

7.2 [1,3]-Rearrangement additional figures

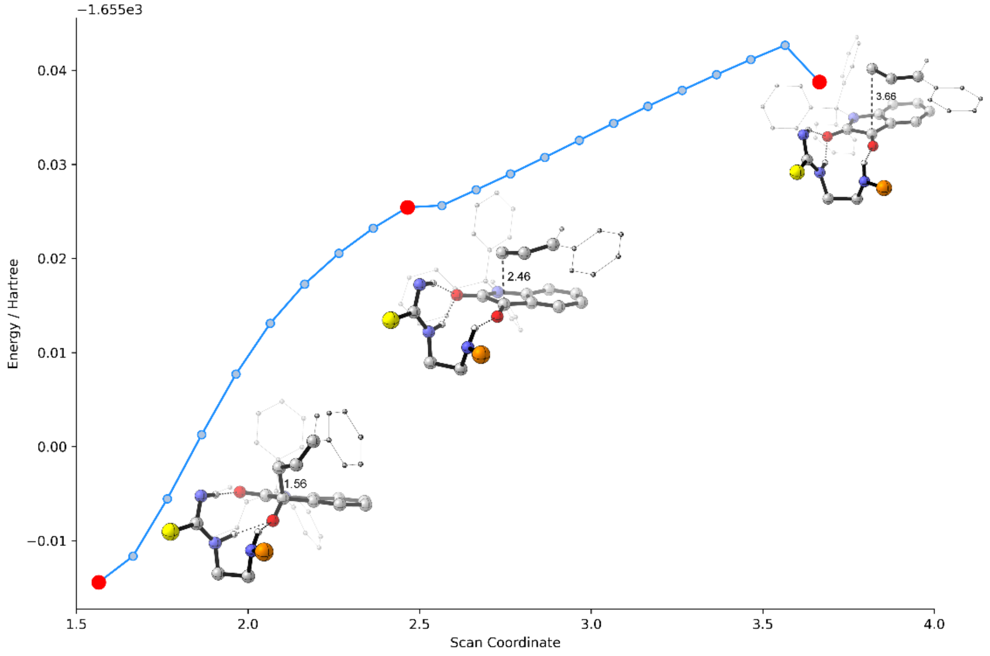

Fig. S23. Energy scan from a conformer of the recombination product (built and then optimised from a fragment complex) increasing the distance between the two carbons leading to a separated fragment pair. The ONIOM low layer atoms of [*t*Bu-BIMP-H]$^+$ have been omitted for clarity. Highlighted points along the scan coordinate relate to the displayed structures. The decrease in energy after the structure at 3.56 Å is due to a conformational change; optimisation of this structure did not result in a recombination TS. (**Fig. 4A**, X = Me, Ar = Ph).

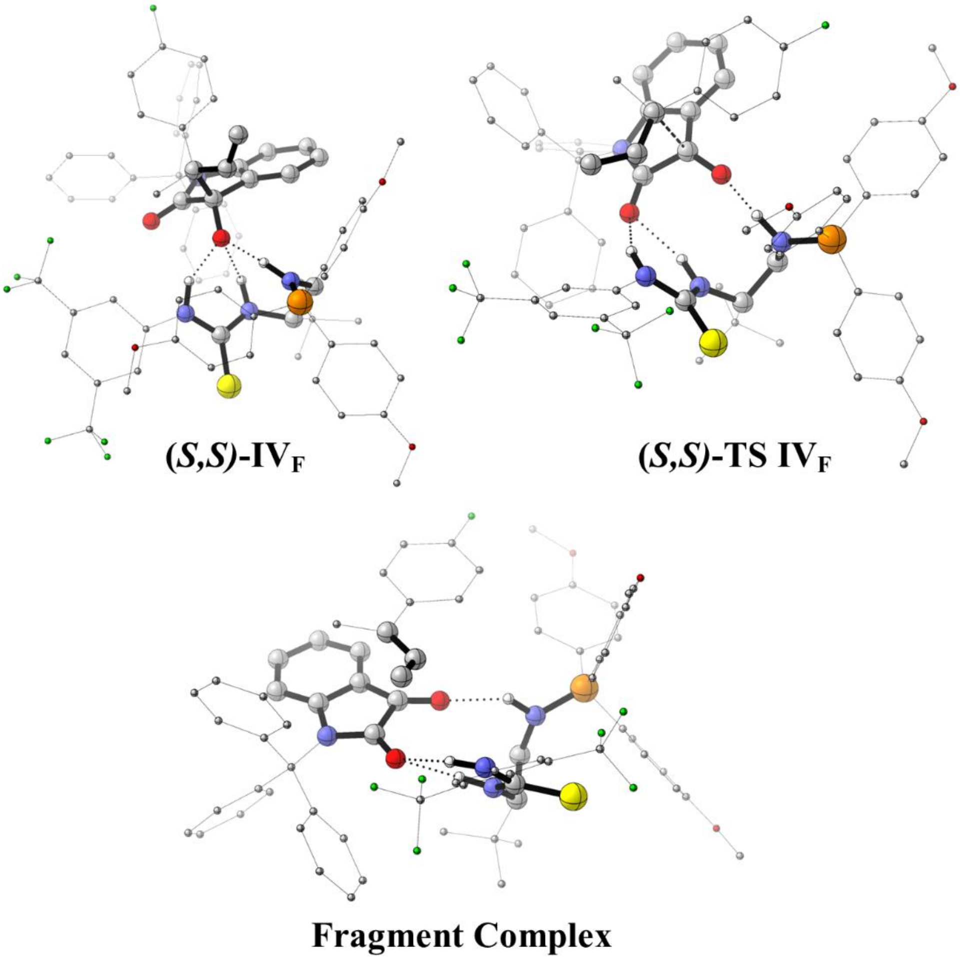

Fig. S24. Optimised structures in the [1,3]-rearrangement. X = Me, Ar = 4-FC_6H_4, (**Fig. 4A**). The fragment complex was obtained from geometry optimisation of the final structure of the IRC calculation of *(S,S)*-TS IV$_F$.

7.3 *N*-substituent effects

We studied entries 1, 8 and 9 from Table S1. These reactions were performed under identical conditions which allowed us to compute the effects of changing the N-substituent on stereoselectivity. We substituted X = Me for X = Ph in the lowest energy (*S*,*S*), (*S*,*R*), (*R*,*R*) and (*R*,*S*) [2,3] TSs and reoptimised them with each of the three N-Me, N-Bn and N-trityl substituents. SPE calculations were performed at the same level of theory as described above, but using toluene as the solvent instead of mesitylene in order to reflect the experimental data available from Table S1. Quasi-harmonic free energies were calculated with GoodVibes using a temperature of 323.15 K, a

concentration of 0.02 mol/L (to match the catalyst loading, Table S1), Truhlar's quasi-harmonic treatment of entropy, the Head-Gordon enthalpy correction, and a vibrational scaling factor of 1.0. The ONIOM layering scheme was the same as shown in **Fig. S18** (left) with both Ph groups of the allylic fragment placed in the high layer. Without full conformational analysis, the resulting $\Delta\Delta G^{\ddagger}$ values are approximate but qualitatively reproduce the trends seen in the experimental e.r. data:

N-substituent	$\Delta\Delta G^{\ddagger}_{comp}$ (kcal mol^{-1})	experimental e.r.
Methyl	0.09	69:31
Benzyl	0.53	79:21
Trityl	0.68	81:19

For the fragmentation step of the [1,3]-rearrangement, (*S*,*S*)-IV_F and (*S*,*S*)-TS IV_F were reoptimised with the N substituent changed to N-benzyl and N-methyl and reaction barriers computed. Without full conformational analysis, these barriers are approximate (see Kinetics section):

N-substituent	$\Delta G^{\ddagger}_{comp}$ (kcal mol^{-1})	$\Delta G^{\ddagger}_{exp}$ (kcal mol^{-1})
Methyl	13.82	21.58
Benzyl	16.85	21.35
Trityl	20.97	21.90

8 Supplementary data for substrates and scope of [1,2]-rearrangement reaction

8.1 Supplementary Data for protected isatins

1-Methylindoline-2,3-dione (S26)

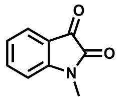

Following **general procedure A**, isatin (2.93 g, 19.9 mmol), sodium hydride (60% dispersion in mineral oil, 1.09 g, 27.3 mmol) and MeI (1.3 mL, 21 mmol) in DMF (15 mL + 20 mL) were stirred for 5 h to give, after purification by column chromatography (eluent: Hexane/EtOAc = 19:1), the title compound (1.96 g, 61%) as a yellow solid with spectroscopic data in accordance with the literature [34]. **^{1}H NMR (500 MHz, $CDCl_3$)** δ **3.25** (3H, s, CH_3), **6.89** (1H, dt, *J* 7.8, 0.8, ArC(7)*H*), **7.12** (1H, td, *J* 7.5, 0.8, ArC(5)*H*), **7.56–7.64** (2H, m, ArC(4,6)*H*); **^{13}C{^{1}H} NMR (126 MHz, $CDCl_3$)** δ **26.4**, **110.1**, **117.6**, **124.0**, **125.4**, **138.5**, **151.6**, **158.4**, **183.5**.

1-Benzylindoline-2,3-dione (S27)

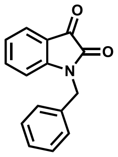

Following **general procedure A**, isatin (7.41 g, 50.4 mmol), potassium carbonate (10.5 g, 76 mmol) and benzyl bromide (6.5 mL, 55 mmol) in acetonitrile (180 mL) were stirred and refluxed for 2 h and filtered, the filtrate was concentrated under reduced pressure and the obtained residue was recrystallized from EtOAc to give the title compound (5.93 g, 50%) as an orange solid with ^{1}H spectroscopic data in accordance with the literature. [34] **^{1}H NMR (500 MHz, $CDCl_3$)** δ **4.93** (2H, s, NCH_2), **6.78** (1H, d, *J* 7.9, ArC(7)*H*), **7.09** (1H, ddd, *J* 7.5, 7.7, 1.1, ArC(5)*H*), **7.28–7.39** (5H, m, Ph*H*), **7.48** (1H, ddd, *J* 7.7, 7.9, 1.3, ArC(6)*H*), **7.61** (1H, dd, *J* 7.5, 1.3, ArC(4)*H*), **^{13}C{^{1}H} NMR (126 MHz, $CDCl_3$)** δ **44.2**, **111.1**, **117.8**, **124.0**, **125.6**, **127.6**, **128.3**, **129.2**, **134.6**, **138.5**, **150.8**, **158.4**, **183.4**.

1-Tritylindoline-2,3-dione (S9)

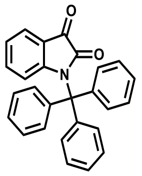

Following **General Procedure A**, isatin (1.47 g, 10 mmol), sodium hydride (60% dispersion in mineral oil, 0.42 g, 10.5 mmol), and trityl bromide (3.56 g, 11 mmol) in DMF (10 mL) were stirred overnight to give, after purification by column chromatography (eluent: Hexane/EtOAc = 4:1), the title compound (3.5 g, 90%) as a yellow-orange solid with ^{1}H spectroscopic data in accordance with the literature [35]. **^{1}H NMR (400 MHz, $CDCl_3$)** δ **6.40** (1H, dt, *J* 8.4, 0.8), **7.00** (1H, td, *J* 7.6, 0.8), **7.17–7.32** (10H, m), **7.41–7.50** (6H, m), **7.59** (1H, app dd, *J* 7.6, 1.6). **^{13}C{^{1}H} NMR (126 MHz, $CDCl_3$)** δ **75.5**, **117.7**, **119.1**, **123.4**, **124.8**, **127.4**, **128.1**, **129.3**, **136.8**, **141.2**, **152.3**, **159.4**, **183.0**.

4-Chloro-1-tritylindoline-2,3-dione (S28)

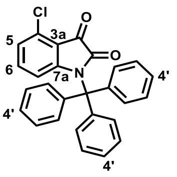

Following **General Procedure A**, 4-chloroisatin (1.81 g, 10 mmol), sodium hydride (60% dispersion in mineral oil, 0.42 g, 10.5 mmol), and trityl bromide (4.19 g, 13 mmol) in DMF (10 mL) were stirred overnight to give, after purification by column chromatography (eluent: Hexane/EtOAc = 4:1), the title compound (3.64 g, 86%) as a yellow-orange solid. **mp** 212–215 °C; **IR** ν_{max} (film) 1742 (C=O); **^{1}H NMR (500 MHz, $CDCl_3$)** δ **6.34** (1H, d, *J* 8.0, ArC(7)*H*), **6.94** (1H, d, *J* 8.0, ArC(5)*H*), **7.09** (1H, app t, *J* 8.5, ArC(6)*H*), **7.23–7.31** (9H, m, ArC(3`,4`,5`)*H*), **7.40–7.46** (6H, m, ArC(2`,6`)*H*). **$^{13}$C{$^1$H} NMR (126 MHz, $CDCl_3$)** δ **75.7** (N*C*$Ph_3$), **116.0** (Ar*C*(7)H), **116.1** (Ar*C*(4)Cl), **124.9** (Ar*C*(5)H), **127.6** (Ar*C*(4`)H), **128.1** (Ar*C*(3`,5`)H), **129.3** (Ar*C*(2`,6`)H), **133.2** (Ar*C*(3a)), **136.6** (Ar*C*(6)H), **141.0** (Ar*C*(1`)), **153.4** (Ar*C*(7a)), **158.5** (*C*(2)=O), **179.9** (*C*(3)=O).

5-Fluoro-1-tritylindoline-2,3-dione (S29)

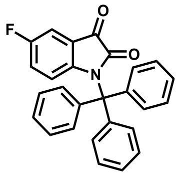

Following **General Procedure A**, 5-fluoroisatin (1.65 g, 10 mmol), sodium hydride (60% dispersion in mineral oil, 0.42 g, 10.5 mmol), and trityl bromide (4.19 g, 13 mmol) in DMF (10 mL) were stirred overnight to give, after purification by column chromatography (eluent: Hexane/EtOAc = 4:1), the title compound (2.64 g, 65%) as a yellow-orange solid with ^{1}H spectroscopic data in accordance with the literature [36]. **^{1}H NMR (500 MHz, $CDCl_3$)** δ **6.37** (1H, dd, *J* 9.0, 3.5), **6.91** (1H, td, *J* 8.5, 2.5), **7.23–7.32** (10H, m), **7.40–7.47** (6H, m); **^{13}C{^{1}H} NMR (126 MHz, $CDCl_3$)** δ **75.7**, **111.3** (d, *J* 23.6), **119.1** (d, *J* 6.8), **119.9** (d, *J* 7.0), **123.4** (d, *J* 23.6), **127.6**, **128.2**, **129.3**, **141.0**, **148.4** (d, *J* 2.2), **158.8** (d, *J* 247.0), **159.8** (d, *J* 1.1), **182.5** (d, *J* 1.9); **^{19}F{^{1}H} NMR (376 MHz, $CDCl_3$)** δ –118.65.

5-Chloro-1-tritylindoline-2,3-dione (S30)

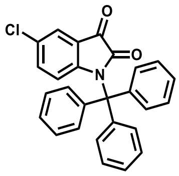

Following **General Procedure A**, 5-chloroisatin (1.81 g, 10 mmol), sodium hydride (60% dispersion in mineral oil, 0.42 g, 10.5 mmol), and trityl bromide (4.19 g, 13 mmol) in DMF (10 mL) were stirred overnight to give, after purification by column chromatography (eluent: Hexane/EtOAc = 4:1), the title compound (3.38 g, 80%) as a yellow-orange solid with ^{1}H spectroscopic data in accordance with the literature [37]. **^{1}H NMR (400 MHz, $CDCl_3$)** δ **6.37** (1H, dd, *J* 8.8, 0.4), **7.17** (1H, dd, *J* 8.8, 2.4), **7.24–7.37** (9H, m), **7.41–7.48** (6H, m), **7.57** (1H, dd, *J* 2.4, 0.4); **^{13}C{^{1}H} NMR (126 MHz, $CDCl_3$)** δ **75.7**, **119.0**, **120.0**, **124.5**, **127.7**, **128.2**, **129.3**, **129.5**, **136.2**, **140.9**, **150.6**, **158.8**, **182.1**.

5-Iodo-1-tritylindoline-2,3-dione (S31)

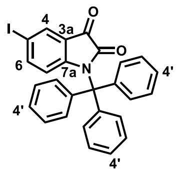

Following **General Procedure A**, 5-iodoisatin (2.73 g, 10 mmol), sodium hydride (60% dispersion in mineral oil, 0.42 g, 10.5 mmol), and trityl bromide (4.19 g, 13 mmol) in DMF (10 mL) were stirred overnight to give, after purification by column chromatography (eluent: Hexane/EtOAc = 4:1), the title compound (3.09 g, 60%) as a yellow-orange solid. **mp** 197–200 °C; **IR** ν_{max} (film) 1740 (C(3)=O), 1662, 1597, 1489, 1448, 1294, 1263, 1190, 1124; **^{1}H NMR (400 MHz, $CDCl_3$)** δ **6.20** (1H, d, *J* 8.7, ArC(7)*H*), **7.25–7.35** (9H, m, ArC(3`,4`,5`)*H*), **7.42–7.47** (6H, m, ArC(2`,6`)*H*), **7.50** (1H, dd, *J* 8.7, 2.0, ArC(6)*H*), **7.89** (1H, d, *J* 2.0, ArC(4)*H*); **$^{13}$C{$^1$H} NMR (101 MHz, $CDCl_3$)** δ **75.7** (N*C*$Ph_3$), **86.5** (Ar*C*(5)I), **119.7** (Ar*C*(7)H), **120.7** (ArC(3a)), **127.7** (Ar*C*(4`)H), **128.2** (Ar*C*(3`,5`)H), **129.3** (Ar*C*(2`,6`)H), **133.3** (Ar*C*(4)H), **140.9** (Ar*C*(1`)), **144.9** (Ar*C*(6)H), **151.8** (Ar*C*(7a)), **158.4(*C*(2)=O)**, **181.7 (*C*(3)=O)**; **HRMS (ESI$^+$)** $C_{27}H_{18}INO_2Na$ $[M+Na]^+$ found 538.0266, requires 538.0275 (1.67 ppm).

5-Nitro-1-tritylindoline-2,3-dione (S32)

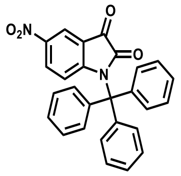

Following **General Procedure A**, 5-nitroisatin (1.92 g, 10 mmol), sodium hydride (60% dispersion in mineral oil, 0.42 g, 10.5 mmol), and trityl bromide (4.19 g, 13 mmol) in DMF (10 mL) were stirred overnight to give, after purification by column chromatography (eluent: Hexane/EtOAc = 3:1), the title compound (3.04 g, 70%) as a yellow-orange solid with ^{1}H spectroscopic data in accordance with the literature [38]. **^{1}H NMR (400 MHz, $CDCl_3$)** δ **6.56** (1H, d, *J* 9.2), **7.26–7.34** (9H, m), **7.39–7.44** (6H, m), **8.10** (1H, dd, *J* 9.2, 2.4), **8.43** (1H, d, *J* 2.4); **^{13}C{^{1}H} NMR (101 MHz, $CDCl_3$)** δ **76.4**, **117.9**, **118.8**, **120.1**, **128.0**, **128.4**, **129.2**, **131.5**, **140.4**, **143.6**, **156.6**, **158.9**, **181.2**.

5-Methyl-1-tritylindoline-2,3-dione (S33)

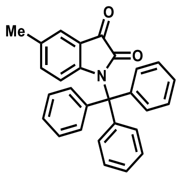

Following **General Procedure A**, 5-methylisatin (1.61 g, 10 mmol), sodium hydride (60% dispersion in mineral oil, 0.42 g, 10.5 mmol), and trityl bromide (4.19 g, 13 mmol) in DMF (10 mL) were stirred overnight to give, after purification by column chromatography (eluent: Hexane/EtOAc = 4:1), the title compound (2.62 g, 65%) as a yellow-orange solid with ^{1}H spectroscopic data in accordance with the literature [38]. **^{1}H NMR (400 MHz, $CDCl_3$)** δ **2.26** (3H, s), **6.30** (1H, d, *J* 8.4), **7.01–7.05** (1H, m), **7.22–7.36** (9H, m), **7.40–7.44** (1H, m), **7.45–7.52** (6H, m). **$^{13}C\{^1H\}$ NMR (101 MHz, $CDCl_3$)** δ **20.5**, **75.4**, **117.5**, **119.2**, **125.0**, **127.4**, **128.0**, **129.3**, **133.1**, **137.5**, **141.3**, **150.1**, **159.6**, **183.3**.

5-Methoxy-1-tritylindoline-2,3-dione (S34)

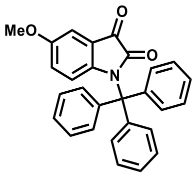

Following **General Procedure A**, 5-methoxyisatin (1.77 g, 10 mmol), sodium hydride (60% dispersion in mineral oil, 0.42 g, 10.5 mmol), and trityl bromide (4.19 g, 13 mmol) in DMF (10 mL) were stirred overnight to give, after purification by column chromatography (eluent: Hexane/EtOAc = 4:1), the title compound (2.81 g, 67%) as a yellow-orange solid with ^{1}H spectroscopic data in accordance with the literature [38]. **^{1}H NMR (400 MHz, $CDCl_3$)** δ **3.73** (3H, s), **6.30** (1H, d, *J* 9.2), **6.76** (1H, dd, *J* 9.2, 3.2), **7.09** (1H, d, *J* 3.2), **7.21–7.31** (9H, m), **7.41–7.48** (6H, m); **$^{13}C\{^1H\}$ NMR (126 MHz, $CDCl_3$)** δ **55.9**, **75.4**, **108.0**, **118.8**, **119.7**, **123.8**, **127.4**, **128.1**, **129.4**, **141.3**, **146.2**, **155.9**, **159.6**, **183.4**.

6-Chloro-1-tritylindoline-2,3-dione (S35)

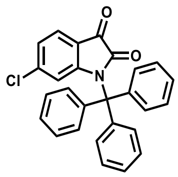

Following **General Procedure A**, 6-chloroisatin (1.81 g, 10 mmol), sodium hydride (60% dispersion in mineral oil, 0.42 g, 10.5 mmol), and trityl bromide (4.19 g, 13 mmol) in DMF (10 mL) were stirred overnight to give, after purification by column chromatography (eluent: Hexane/EtOAc = 4:1), the title compound (2.96 g, 70%) as a yellow-orange solid with ^{1}H spectroscopic data in accordance with the literature [38]. **^{1}H NMR (400 MHz, CDCl$_3$)** δ **6.32** (1H, d, *J* 1.6), **6.99** (1H, dd, *J* 8.0, 1.6), **7.23–7.33** (9H, m), **7.38–7.46** (6H, m), **7.52** (1H, d, *J* 8.0); **^{13}C{^{1}H} NMR (126 MHz, CDCl$_3$)** δ **75.8**, **117.5**, **118.1**, **123.8**, **125.7**, **127.8**, **128.2**, **129.3**, **140.8**, **143.2**, **153.1**, **159.3**, **181.5**.

6-Bromo-1-tritylindoline-2,3-dione (S36)

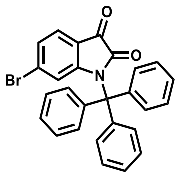

Following **General Procedure A**, 6-bromoisatin (2.25 g, 10 mmol), sodium hydride (60% dispersion in mineral oil, 0.42 g, 10.5 mmol), and trityl bromide (4.19 g, 13 mmol) in DMF (10 mL) were stirred overnight to give, after purification by column chromatography (eluent: Hexane/EtOAc = 4:1), the title compound (2.80 g, 60%) as a yellow-orange solid with ^{1}H spectroscopic data in accordance with the literature [38]. **^{1}H NMR (400 MHz, CDCl$_3$)** δ **6.47** (1H, d, *J* 1.6), **7.17** (1H, dd, *J* 8.0, 1.6), **7.24–7.35** (10H, m), **7.40–7.46** (7H, m); **^{13}C{^{1}H} NMR (101 MHz, CDCl$_3$)** δ **75.8**, **117.9**, **121.0**, **125.6**, **126.7**, **127.7**, **128.2**, **129.3**, **132.1**, **140.8**, **153.0**, **159.2**, **181.8**.

1-Benzyl-7-chloroindoline-2,3-dione (S37)

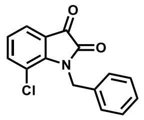

Following **General Procedure A**, 7-chloroisatin (1.81 g, 10 mmol), sodium hydride (60% dispersion in mineral oil, 0.42 g, 10.5 mmol), and benzyl bromide (2.21 g, 13 mmol) in DMF (10 mL) were stirred overnight to give, after purification by column chromatography (eluent: Hexane/EtOAc = 4:1), the title compound (2.57 g, 95%) as a red solid with ^{1}H spectroscopic data in accordance with the literature [39]. **^{1}H NMR (500 MHz, $CDCl_3$)** δ **5.37** (2H, s), **7.07** (1H, dd, *J* 8.1, 7.3), **7.28** (3H, d, *J* 7.2), **7.31–7.36** (2H, m), **7.47** (1H, dd, *J* 8.2, 1.3), **7.58** (1H, dd, *J* 7.3, 1.3); **$^{13}C\{^1H\}$ NMR (126 MHz, $CDCl_3$)** δ **45.2**, **117.6**, **120.6**, **124.3**, **125.1**, **126.8**, **127.8**, **128.9**, **136.2**, **140.9**, **146.3**, **159.0**, **182.5**.

8.2 Supplementary Data for diazo compounds

3-Diazo-1-methylindolin-2-one (S38)

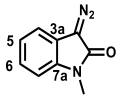

Following **General Procedure A**, 1-methylindoline-2,3-dione (1.96 g, 12.2 mmol), tosylhydrazide (2.50 g, 13.4 mmol), and 0.2 M aq. NaOH (61 mL + 20 mL) in THF (56 mL) gave, after purification by flash column chromatography (20:80 EtOAc/petrol), the title compound as a red solid (1.74 g, 82%) with ^{1}H spectroscopic data in accordance with the literature [40]. **^{1}H NMR (500 MHz, $CDCl_3$)** δ **3.33** (s, 3H, C*H*$_3$), **6.90–6.94** (m, 1H, ArC(7)*H*), **7.09** (app td, *J* 7.5, 1.0, 1H, ArC(6)*H*), **7.17–7.23** (m, 2H, ArC(4)*H* and ArC(5)*H*), **^{13}C{^{1}H} NMR (126 MHz, $CDCl_3$)** δ **26.9**, **60.9**, **108.7**, **116.8**, **118.3**, **122.2**, **125.6**, **134.6**, **166.9**.

1-Benzyl-3-diazoindolin-2-one (S39)

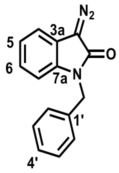

Following **General Procedure A**, 1-benzylindoline-2,3-dione (5.83 g, 24.6 mmol), tosylhydrazide (5.14 g, 27.6 mmol), and 0.2 M aq. NaOH (122 mL + 40 mL) in THF (112 mL) gave, after purification by flash column chromatography (eluent: petrol/EtOAc = 3:1), the title compound (5.37 g, 88%) as an orange solid with ^{1}H spectroscopic data in accordance with the literature [40]. **^{1}H NMR (500 MHz, $CDCl_3$)** δ **5.03** (s, 2H, C*H*$_2$), **6.79–6.87** (m, 1H, ArC(7)*H*), **7.03–7.12** (m, 2H, ArC(5)*H* and ArC(6)*H*), **7.19–7.23** (m, 1H, C(4)*H*), **7.23–7.28** (m, 1H, ArC(4`)*H*), **7.28–7.35** (m, 4H, ArC(2`,3`,5`,6`)*H*), **^{13}C{^{1}H} NMR (126 MHz, $CDCl_3$)** δ **44.4**, **61.0**, **109.7**, **116.9**, **118.4**, **122.3**, **125.6**, **127.4**, **127.8**, **128.9**, **133.8**, **136.1**, **167.0**.

3-Diazo-1-tritylindolin-2-one (S40)

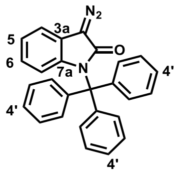

Following **General Procedure A**, *N*-trityl isatin (1.95 g, 5 mmol), tosylhydrazine (1.02 g, 5.5 mmol), and 0.2 M aq. NaOH (50 mL, 10 mmol) in THF (25 mL) gave an orange precipitate which was collected and purified by column chromatography (eluent: hexane/CH_2Cl_2/EtOAc = 15:5:2) to afford the the title compound (1.60 g, 80%) as an orange solid. **mp** 178–180 °C; **IR** ν_{max} (film) 2085 (C=N_2), 1686 (C=O), 1604 (C=C), 1491, 1458, 1448, 1400, 1336, 1265, 1146, 1101; **^{1}H NMR (500 MHz, $CDCl_3$)** δ **6.27** (1H, dd, *J* 8.0, 2.0, ArC(7)*H*), **6.76–6.84** (1H, m, ArC(6)*H*), **6.97** (1H, app t, *J* 7.5, ArC(5)*H*), **7.09–7.12** (1H, m, ArC(4)*H*), **7.21–7.25** (3H, m, ArC(4`)*H*), **7.26–7.31** (6H, m, ArC(3`,5`)*H*), **7.47–7.52** (6H, m, ArC(2`,6`)*H*); **$^{13}$C{$^1$H} NMR (126 MHz, $CDCl_3$)** δ **62.0** (*C*(3)), **75.4** (N*C*$Ph_3$), **116.2** (Ar*C*(7)H), **117.3** (Ar*C*(3a)), **117.7** (Ar*C*(4)H), **121.9** (Ar*C*(5)H), **124.2** (Ar*C*(6)H), **127.1** (Ar*C*(4`)H), **127.7** (Ar*C*(3`,5`)H), **129.5** (Ar*C*(2`,6`)H), **134.4** (Ar*C*(7a)), **142.2** (Ar*C*(1`)), **168.1** (*C*=O); **HRMS (ESI$^+$)** $C_{27}H_{19}N_3ONa$ $[M+Na]^+$ found 424.1417, requires 424.1420 (–0.7 ppm).

4-Chloro-3-diazo-1-tritylindolin-2-one (S41)

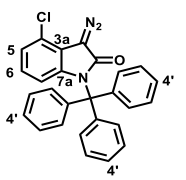

Following **General Procedure A**, *N*-trityl 4-chloroisatin (2.12 g, 5 mmol), tosylhydrazine (1.02 g, 5.5 mmol), and 0.2 M aq. NaOH (50 mL, 10 mmol) in THF (25 mL) gave an orange precipitate which was collected and purified by column chromatography (eluent: hexane/CH_2Cl_2/EtOAc = 15:5:2) to afford the title compound (1.94 g, 89%) as an orange solid. **mp** 165–167 °C; **IR** ν_{max} (film) 2100 (C=N_2), 1688 (C=O), 1439, 1394, 1332, 1248, 1140, 1014; **^{1}H NMR (500 MHz, $CDCl_3$)** δ **6.17** (1H, d, *J* 8.5, ArC(7)*H*), **6.69** (1H, app t, *J* 8.5, ArC(6)*H*), **6.89** (1H, d, *J* 8.0, ArC(5)*H*), **7.21–**

7.25 (3H, m, ArC(4`)*H*), **7.25–7.31** (6H, m, ArC(3`,5`)*H*), **7.42–7.47** (6H, m, ArC(2`,6`)*H*); **$^{13}$C{$^{1}$H} NMR (126 MHz, CDCl$_3$)** δ **62.5** (*C*(3)), **75.7** (N*C*Ph$_3$), **114.5** (Ar*C*(7)H), **115.1** (Ar*C*(4)Cl), **122.2** (Ar*C*(5)H), **124.6** (Ar*C*(6)H), **125.3** (Ar*C*(3a)), **127.2** (Ar*C*(4`)H), **127.8** (Ar*C*(3`,5`)H), **129.5** (Ar*C*(2`,6`)H), **135.4** (Ar*C*(7a)), **142.0** (Ar*C*(1`)), **167.8** (*C*=O); **HRMS (ESI$^+$)** $C_{27}H_{18}{}^{35}ClN_3ONa$ [M+Na]$^+$ found 458.1017, requires 458.1031 (–3.0 ppm).

3-Diazo-5-fluoro-1-tritylindolin-2-one (S42)

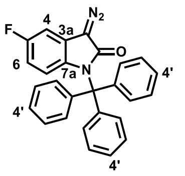

Following **General Procedure A**, *N*-trityl fluoroisatin (2.04 g, 5 mmol), tosylhydrazine (1.02 g, 5.5 mmol), and 0.2 M aq. NaOH (50 mL, 10 mmol) in THF (25 mL) gave an orange precipitate which was collected and purified by column chromatography (eluent: hexane/CH_2Cl_2/EtOAc = 15:5:2) to afford the title compound (1.51 g, 72%) as an orange solid. **mp** 186–188 °C; **IR** ν_{max} (film) 2087 (C=N$_2$), 1692 (C=O) 1593 (C=C), 1466, 1448, 1389, 1286, 1259, 1184, 1140 (C-F), 1111; **^{1}H NMR (500 MHz, CDCl$_3$)** δ **6.17** (1H, dd, *J* 9.0, 4.0, ArC(7)*H*), **6.49** (1H, app td, *J* 9.0, 2.5, ArC(6)*H*), **6.83** (1H, dd, *J* 8.0, 3.0, ArC(4)*H*), **7.21–7.25** (3H, m, ArC(4`)*H*), **7.26–7.31** (6H, m, ArC(3`,5`)*H*), **7.44–7.48** (6H, m, ArC(2`,6`)*H*); **$^{13}$C{$^{1}$H} NMR (126 MHz, CDCl$_3$)** δ **62.6** (*C*(3)), **75.6** (N*C*Ph$_3$), **105.1** (d, *J* 27.7, Ar*C*(4)H), **111.0** (d, *J* 22.7, Ar*C*(6)H), **116.8** (d, *J* 7.6, Ar*C*(7)H), **118.6** (d, *J* 10.1, Ar*C*(3a)), **127.2** (Ar*C*(4`)H), **127.8** (Ar*C*(3`,5`)H), **129.5** (Ar*C*(2`,6`)H), **130.3** (Ar*C*(7a)), **142.0** (Ar*C*(1`)), **158.5** (d, *J* 241.9, Ar*C*(5)F), **167.7** (*C*=O); **^{19}F{^{1}H} NMR (470 MHz, CDCl$_3$)** δ **–120.81** (s); **HRMS (ESI$^+$)** $C_{27}H_{18}FN_3ONa$ [M+Na]$^+$ found 442.1320, requires 442.1326 (–1.4 ppm).

5-Chloro-3-diazo-1-tritylindolin-2-one (S43)

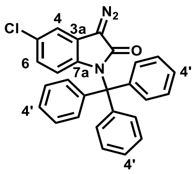

Following **General Procedure A**, *N*-trityl 5-chloroisatin (2.12 g, 5 mmol), tosylhydrazine (1.02 g, 5.5 mmol), and 0.2 M aq. NaOH (50 mL, 10 mmol) in THF (25 mL) gave an orange precipitate which was collected and purified by column chromatography (eluent: hexane/CH_2Cl_2/EtOAc = 15:5:2) to afford the title compound (1.37 g, 63%) as an orange solid. **mp** 196–198 °C; **IR** ν_{max} (film) 2095 (C=N_2), 1694 (C=O), 1604 (C=C), 1491, 1466, 1448, 1381, 1327, 1290, 1265, 1182, 1159, 1118, 1082 (C-Cl); **^{1}H NMR (500 MHz, $CDCl_3$)** δ **6.20** (1H, d, *J* 8.5, ArC(7)*H*), **6.77** (1H, dd, *J* 9.0, 2.5, ArC(6)*H*), **7.11** (1H, d, *J* 2.0, ArC(4)*H*), **7.24–7.29** (3H, m, ArC(4`)*H*), **7.29–7.34** (6H, m, ArC(3`,5`)*H*), **7.46–7.51** (6H, m, ArC(2`,6`)*H*); **$^{13}$C{$^1$H} NMR (126 MHz, $CDCl_3$)** δ **62.0** (*C*(3)), **75.6** (N*C*$Ph_3$), **116.9** (Ar*C*(7)H), **117.5** (Ar*C*(4)H), **118.9** (Ar*C*(3a)), **124.2** (Ar*C*(6)H), **127.2** (Ar*C*(4`)H), **127.5** (Ar*C*(5)Cl), **127.8** (Ar*C*(3`,5`)H), **129.5** (Ar*C*(2`,6`)H), **132.8** (Ar*C*(7a)), **141.9** (Ar*C*(1`)), **167.4** (*C*=O); **HRMS (ESI$^+$)** $C_{27}H_{18}{}^{35}ClN_3ONa$ $[M+Na]^+$ found 458.1023, requires 458.1031 (–1.7 ppm).

3-Diazo-5-iodo-1-tritylindolin-2-one (S44)

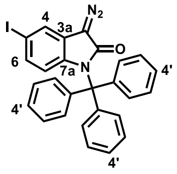

Following **General Procedure A**, *N*-trityl 5-iodoisatin (2.58 g, 5 mmol), tosylhydrazine (1.02 g, 5.5 mmol), and 0.2 M aq. NaOH (50 mL, 10 mmol) in THF (25 mL) gave an orange precipitate which was collected and purified by column chromatography (eluent: hexane/CH_2Cl_2/EtOAc = 15:5:2) to afford the title compound (1.84 g, 70%) as an orange solid. **mp** 188–190 °C; **IR** ν_{max} (film) 2098 (C=N_2), 1697 (C=O), 1593, 1560, 1489, 1460, 1448, 1435, 1377, 1286, 1257, 1147, 1111, 1085 (C-I); **^{1}H NMR (400 MHz, $CDCl_3$)** δ **6.01** (1H, d, *J* 8.8, ArC(7)*H*), **7.09** (1H, dd, *J* 8.8, 1.8, ArC(6)*H*), **7.23–7.34** (9H, m, ArC(3`,4`,5`)*H*), **7.42** (1H, d, *J* 1.8, ArC(4)*H*), **7.43–7.47** (6H, m,

ArC(2`,6`)*H*); **$^{13}C\{^1H\}$ NMR (101 MHz, $CDCl_3$)** δ **61.4** (*C*(3)), **75.6** (N*C*Ph_3), **84.7** (Ar*C*(5)I), **117.8** (Ar*C*(7)H), **119.7** (Ar*C*(3a)), **126.0** (Ar*C*(4)H), **127.2** (Ar*C*(4`)H), **127.8** (Ar*C*(3`,5`)H), **129.5** (Ar*C*(2`,6`)H), **132.9** (Ar*C*(6)H), **134.0** (Ar*C*(7a)), **141.9** (Ar*C*(1`)), **167.2** (*C*=O); **HRMS (ESI$^+$)** $C_{27}H_{18}IN_3ONa$ $[M+Na]^+$ found 550.0382, requires 550.0387 (–0.9 ppm).

3-Diazo-5-nitro-1-tritylindolin-2-one (S45)

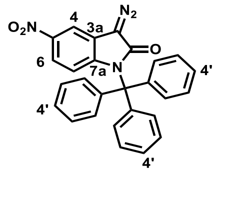

Following **General Procedure A**, *N*-trityl 5-nitroisatin (2.17 g, 5 mmol), tosylhydrazine (1.02 g, 5.5 mmol), and 0.2 M aq. NaOH (50 mL, 10 mmol) in THF (25 mL) gave an orange precipitate which was collected and purified by column chromatography (eluent: hexane/CH_2Cl_2/EtOAc = 15:5:2) to afford the title compound (1.74 g, 80%) as an orange solid. **mp** 187–190 °C; **IR** ν_{max} (film) 2108 (C=N_2), 1680 (C=O), 1606 (C=C), 1514 (NO_2), 1490, 1467, 1444, 1382 (NO_2), 1332, 1294, 1273, 1180, 1163, 1118, 1089; **^1H NMR (400 MHz, $CDCl_3$)** δ **6.34** (1H, d, *J* 9.2, ArC(7)*H*), **7.24–7.33** (9H, m, ArC(3`,4`,5`)*H*), **7.41–7.46** (6H, m, ArC(2`,6`)*H*), **7.71** (1H, dd, *J* 9.2, 2.4, ArC(6)*H*), **8.02** (1H, d, *J* 2.4, ArC(4)*H*); **$^{13}C\{^1H\}$ NMR (126 MHz, $CDCl_3$)** δ **62.6** (*C*(3)), **76.2** (N*C*$Ph_3$), **112.8** (Ar*C*(4)H), **115.2** (Ar*C*(7)H), **118.3** (Ar*C*(3a)), **120.3** (Ar*C*(6)H), **127.5** (Ar*C*(4`)H), **128.0** (Ar*C*(3`,5`)H), **129.4** (Ar*C*(2`,6`)H), **139.2** (Ar*C*(7a)), **141.4** (Ar*C*(1`)H), **142.6** (Ar*C*(5)NO_2), **167.1** (*C*=O): **HRMS (ESI$^+$)** $C_{27}H_{18}N_4O_3Na$ $[M+Na]^+$ found 469.1263, requires 469.1271 (–1.7 ppm).

3-Diazo-5-methyl-1-tritylindolin-2-one (S46)

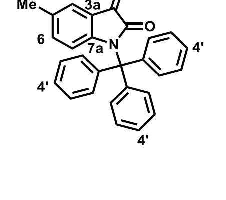

Following **General Procedure A**, *N*-trityl 5-methylisatin (2.02 g, 5 mmol), tosylhydrazine (1.02 g, 5.5 mmol), and 0.2 M aq. NaOH (50 mL, 10 mmol) in THF (25 mL) gave an orange precipitate which was collected and purified by column chromatography (eluent: hexane/CH_2Cl_2/EtOAc = 15:5:2) to afford the title compound (1.41 g, 68%) as an orange solid. **mp** 176–178 °C; **IR** ν_{max} (film) 2087 (C=N_2), 1682 (C=O), 1471, 1448, 1384, 1327, 1303, 1263, 1182, 1161, 1124; **^{1}H NMR (500 MHz, $CDCl_3$)** δ **2.32** (3H, s, C*H₃*), **6.20** (1H, d, *J* 8.5, ArC(7)*H*), **6.66** (1H, d, *J* 8.0, ArC(6)*H*), **6.98** (1H, s, ArC(4)*H*), **7.25–7.30** (3H, m, ArC(4`)*H*), **7.31–7.39** (6H, m, ArC(3`,5`)*H*), **7.55** (6H, d, *J* 7.7, ArC(2`,6`)*H*); **$^{13}$C{$^1$H} NMR (126 MHz, $CDCl_3$)** δ **21.0** (*C*$H_3$), **61.8** (*C*(3)), **75.3** (N*C*$Ph_3$), **115.9** (Ar*C*(7)H), **117.3** (Ar*C*(5)Me), **118.3** (Ar*C*(4)H), **125.0** (Ar*C*(6)H), **127.0** (Ar*C*(4`)H), **127.7** (Ar*C*(3`,5`)H), **129.5** (Ar*C*(2`,6`)H), **131.5** (Ar*C*(3a)), **132.1** (Ar*C*(7a)), **142.3** (Ar*C*(1`)), **168.1** (*C*=O); **HRMS (ESI$^+$)** $C_{28}H_{21}N_3ONa$ $[M+Na]^+$ found 438.1572, requires 438.1577 (–1.1 ppm).

3-Diazo-5-methoxy-1-tritylindolin-2-one (S47)

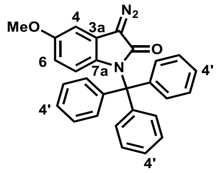

Following **General Procedure A**, *N*-trityl 5-methoxyisatin (2.10 g, 5 mmol), tosylhydrazine (1.02 g, 5.5 mmol), and 0.2 M aq. NaOH (50 mL, 10 mmol) in THF (25 mL) gave a red precipitate which was collected and purified by column chromatography (eluent: hexane/CH_2Cl_2/EtOAc = 15:5:2) to afford the title compound (1.94 g, 90%) as a red solid. **mp** 180–182 °C; **IR** ν_{max} (film) 2092 (C=N_2), 1681 (C=O), 1587 (C=C), 1483, 1448, 1388, 1271, 1244, 1151 (C-O); **^{1}H NMR (500 MHz, $CDCl_3$)** δ **3.73** (3H, s, OC*H₃*), **6.15** (1H, d, *J* 9.0, ArC(7)*H*), **6.35** (1H, dd, *J* 9.0, 2.5, ArC(6)*H*), **6.68** (1H, d, *J* 2.5, ArC(4)*H*), **7.21–7.25** (3H, m, ArC(4`)*H*), **7.26–7.31** (6H, m, ArC(3`,5`)*H*), **7.46–7.51** (6H, m, ArC(2`,6`)*H*); **$^{13}$C{$^1$H} NMR (126 MHz, $CDCl_3$)** δ **55.7** (O*C*$H_3$), **62.3** (*C*(3)), **75.3** (N*C*$Ph_3$), **103.7** (Ar*C*(4)H), **110.1** (Ar*C*(6)H), **116.8** (Ar*C*(7)H), **118.4** (Ar*C*(3a)), **127.0** (Ar*C*(4`)H), **127.7** (Ar*C*(3`,5`)H), **128.1** (Ar*C*(7a)), **129.5**

(Ar*C*(2`,6`)H), **142.3** (Ar*C*(1`)), **155.2** (Ar*C*(5)OMe), **168.0** (*C*=O); **HRMS (ESI$^+$)** $C_{28}H_{21}N_3O_2Na$ $[M+Na]^+$ found 454.1520, requires 454.1526 (–1.3 ppm).

6-Chloro-3-diazo-1-tritylindolin-2-one (S48)

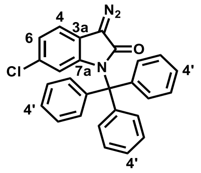

Following **General Procedure A**, *N*-trityl 6-choloroisatin (2.12 g, 5 mmol), tosylhydrazine (1.02 g, 5.5 mmol), and 0.2 M aq. NaOH (50 mL, 10 mmol) in THF (25 mL) gave an orange precipitate which was collected and purified by column chromatography (eluent: hexane/CH_2Cl_2/EtOAc = 15:5:2) to afford the title compound (1.78 g, 82%) as an orange solid. **mp** 166–168 °C; **IR** ν_{max} (film) 2089 (C=N_2), 1690 (C=O), 1606 (C=C), 1469, 1448, 1400, 1338, 1263, 1180, 1147, 1109, 1078 (C-Cl), 1010; **^{1}H NMR (500 MHz, $CDCl_3$)** δ **6.20** (1H, d, *J* 1.5, ArC(7)*H*), **6.98** (1H, dd, *J* 8.5, 2.0, ArC(5)*H*), **7.02** (1H, d, *J* 8.5, ArC(4)*H*), **7.26**–**7.30** (3H, m, ArC(4`)*H*), **7.31**–**7.35** (6H, m, ArC(3`,5`)*H*), **7.47**–**7.52** (6H, m, ArC(2`,6`)*H*); **$^{13}$C{$^1$H} NMR (126 MHz, $CDCl_3$)** δ **61.9** (*C*(3)), **75.7** (N*C*$Ph_3$), **115.7** (Ar*C*(3a)), **116.3** (Ar*C*(7)H), **118.1** (Ar*C*(4)H), **122.0** (Ar*C*(5)H), **127.3** (Ar*C*(4`)H), **127.9** (Ar*C*(3`,5`)H), **129.5** (Ar*C*(2`,6`)H), **129.9** (Ar*C*(6)Cl), **135.2** (Ar*C*(7a)), **141.8** (Ar*C*(1`)), **167.7** (*C*=O); **HRMS (ESI$^+$)** $C_{27}H_{18}{}^{35}ClN_3ONa$ $[M+Na]^+$ found 458.1024, requires 458.1031 (–1.4 ppm).

6-Bromo-3-diazo-1-tritylindolin-2-one (S49)

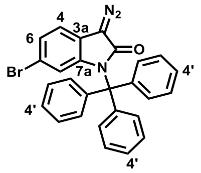

Following **General Procedure A**, *N*-trityl 6-bromoisatin (2.34 g, 5 mmol), tosylhydrazine (1.02 g, 5.5 mmol), and 0.2 M aq. NaOH (50 mL, 10 mmol) in THF (25 mL) gave an orange precipitate which was collected and purified by column

chromatography (eluent: hexane/CH_2Cl_2/EtOAc = 15:5:2) to afford the title compound (2.04 g, 85%) as an orange solid. **mp** 175–177 °C; **IR** ν_{max} (film) 2089 (C=N_2), 1682 (C=O), 1604, 1467, 1448, 1417, 1400, 1338, 1261, 1145, 1109, 1080, 1068 (C-Br), 1008; **^{1}H NMR (400 MHz, $CDCl_3$)** δ **6.28** (1H, d, *J* 1.6, ArC(7)*H*), **6.98** (1H, d, *J* 8.0, ArC(4)*H*), **7.12** (1H, dd, *J* 8.4, 1.6, ArC(5)*H*), **7.24–7.34** (9H, m, ArC(3`,4`,5`)*H*), **7.44–7.49** (6H, m, ArC(2`,6`)*H*); **$^{13}$C{$^1$H} NMR (126 MHz, $CDCl_3$)** δ **62.0** (*C*(3)), **75.7** (N*C*$Ph_3$), **116.2** (Ar*C*(3a)), **117.5** (Ar*C*(6)Br), **118.5** (Ar*C*(4)H), **119.0** (Ar*C*(7)H), **124.8** (Ar*C*(5)H), **127.3** (Ar*C*(4`)H), **127.9** (Ar*C*(3`,5`)H), **129.5** (Ar*C*(2`,6`)H), **135.4** (Ar*C*(7a)), **141.8** (Ar*C*(1`)), **167.6** (*C*=O); **HRMS (ESI$^+$)** $C_{27}H_{18}{}^{79}BrN_3ONa$ $[M+Na]^+$ found 502.0525, requires 502.0525 (–1.7 ppm).

1-Benzyl-7-chloro-3-diazoindolin-2-one (S50)

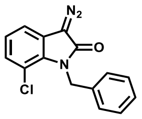

Following **General Procedure A**, *N*-benzyl 7-chloroisatin (1.19 g, 5 mmol), tosylhydrazine (1.02 g, 5.5 mmol), and 0.2 M aq. NaOH (50 mL, 10 mmol) in THF (25 mL) gave an orange precipitate which was collected and purified by column chromatography (eluent: hexane/CH_2Cl_2/EtOAc = 15:5:2) to afford the title compound (2.04 g, 64%) as an orange solid with ^{1}H spectroscopic data in accordance with the literature [41]. **^{1}H NMR (400 MHz, $CDCl_3$)** δ **5.47** (2H, s), **7.00** (1H, dd, *J* 8.0, 7.2), **7.07** (1H, dd, *J* 8.0, 1.2), **7.11** (1H, dd, *J* 7.6, 1.2), **7.20–7.26** (3H, m), **7.27–7.33** (2H, m); **^{13}C{^{1}H} NMR (101 MHz, $CDCl_3$)** δ **45.5**, **116.4**, **116.7**, **119.5**, **123.0**, **126.7**, **127.4**, **127.7**, **128.7**, **129.8**, **137.8**, **167.0**.

8.3 Supplementary Data for allylic alcohols

3,3-Diphenylprop-2-en-1-ol (S10)

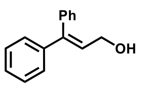

Following **General Procedure B**, ethyl 3,3-diphenylacrylate (1.26 g, 5 mmol), DIBAL-H (11 mL, 11 mmol) in Et_2O (20 mL) gave a crude allylic alcohol product

which was purified by flash column chromatography (eluent: hexane/EtOAc = 5:1 to 3:1) to afford the title compound (0.84 g, 80%) as a pale yellow amorphous solid with ^{1}H spectroscopic data in accordance with the literature [42]. **IR** ν_{max} (film) 3288 (OH), 1599 (C=C), 1575 (C=C), 1492, 1442, 1369, 1221 (C-O), 1074, 1012; **^{1}H NMR (400 MHz, CDCl$_3$)** δ_H **1.55** (1H, s), **4.13** (2H, d, *J* 6.8), **6.16** (1H, t, *J* 6.8), **7.03–7.11** (2H, m), **7.15–7.24** (5H, m), **7.24–7.32** (3H, m).

(*E*)-3-Phenylbut-2-en-1-ol (S11)

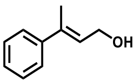

Following **General Procedure B**, ethyl (*E*)-3-phenylbut-2-enoate (0.95 g, 5 mmol), DIBAL-H (11 mL, 11 mmol) in Et$_2$O (20 mL) gave a crude allylic alcohol product which was purified by flash column chromatography (eluent: hexane/EtOAc = 4:1) to afford the title compound (0.68 g, 92%) as a colourless oil with ^{1}H spectroscopic data in accordance with the literature [43]. **IR** ν_{max} (film) 3292 (OH), 1647, 1597 (C=C), 1575 (C=C), 1492, 1444, 1379, 1269, 1244 (C-O), 1109. **^{1}H NMR (500 MHz, CDCl$_3$)** δ_H **1.75** (1H, s), **2.09** (3H, dt, *J* 1.2, 0.8), **4.37** (2H, dq, *J* 6.7, 0.8), **5.99** (1H, tq, *J* 6.7, 1.2), **7.25–7.29** (1H, m), **7.31–7.36** (2H, m), **7.40–7.44** (2H, m).

(*E*)-3-Phenylpent-2-en-1-ol (S51)

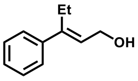

Following **General Procedure B**, ethyl (*E*)-3-phenylpent-2-enoate (1.02 g, 5 mmol), DIBAL-H (11 mL, 11 mmol) in Et$_2$O (20 mL) gave a crude allylic alcohol product which was purified by flash column chromatography (eluent: hexane/EtOAc = 4:1) to afford the title compound (0.67 g, 83%) as a colourless oil with ^{1}H spectroscopic data in accordance with the literature [43]. **IR** ν_{max} (film, cm^{-1}) 3294 (OH), 2966 (C-H), 1492, 1454, 1444, 1375, 1091, 1064; **^{1}H NMR (400 MHz, CDCl$_3$)** δ_H **1.00** (3H, t, *J* 7.6), **1.63** (1H, s), **2.55** (2H, app q, *J* 7.6), **4.36** (2H, d, *J* 6.8), **5.84** (1H, tq, *J* 6.8, 0.5), **7.24–7.29** (1H, m), **7.30–7.35** (2H, m), **7.36–7.40** (2H, m).

(*E*)-3-Cyclopropyl-3-phenylprop-2-en-1-ol (S52)

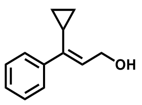

Following **General Procedure B**, ethyl (*E*)-3-cyclopropyl-3-phenylacrylate (1.08 g, 5 mmol), DIBAL-H (11 mL, 11 mmol) in Et_2O (20 mL) gave a crude allylic alcohol product which was purified by flash column chromatography (eluent: hexane/EtOAc = 4:1) to afford the title compound (0.70 g, 80%) as a colourless oil with ^1H spectroscopic data in accordance with the literature [44]. **IR** ν_{max} (film) 3275 (OH), 1492, 1442, 1311, 1215 (C-O); **^{1}H NMR (400 MHz, CDCl$_3$)** δ_H **0.32–0.38** (2H, m), **0.79–0.82** (1H, m), **0.82–0.85** (1H, m), **1.65** (1H, s), **1.72–1.80** (1H, m), **4.53** (2H, d, *J* 6.6), **5.87** (1H, td, *J* 6.6, 1.7), **7.21–7.27** (1H, m), **7.27–7.36** (1H, m).

(*Z*)-3-Phenylbut-2-en-1-ol (S53)

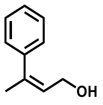

Following **General Procedure B**, ethyl (*Z*)-3-phenylbut-2-enoate (0.95 g, 5 mmol), DIBAL-H (11 mL, 11 mmol) in Et_2O (20 mL) gave a crude allylic alcohol product which was purified by flash column chromatography (eluent: hexane/EtOAc = 4:1) to afford the title compound (0.67 g, 85%) as a colourless oil with ^1H spectroscopic data in accordance with the literature [45]. **IR** ν_{max} (film) 3321 (OH), 1653, 1600, 1573, 1492, 1435, 1375, 1246 (C-O), 1082, 1064; **^{1}H NMR (500 MHz, CDCl$_3$)** δ_H **1.70** (1H, s), **2.09** (3H, dt, *J* 1.5, 1.2), **4.06** (2H, app d, *J* 7.1), **5.71** (1H, tq, *J* 7.1, 1.5), **7.16–7.21** (2H, m), **7.25–7.30** (1H, m), **7.31–7.37** (2H, m).

(*E*)-3-(4-Fluorophenyl)but-2-en-1-ol (S54)

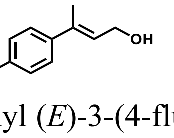

Following **General Procedure B**, ethyl (*E*)-3-(4-fluorophenyl)but-2-enoate (1.04 g, 5 mmol), DIBAL-H (11 mL, 11 mmol) in Et_2O (20 mL) gave a crude allylic alcohol product which was purified by flash column chromatography (eluent: hexane/EtOAc = 4:1) to afford the title compound (0.72 g, 87%) as a colourless oil with ^1H spectroscopic data in accordance with the literature [46]. **IR** ν_{max} (film) 3319 (OH), 1602 (C=C), 1508,

1442, 1408, 1379, 1224 (C-O), 1161 (C-F), 1103, 1064; **^{1}H NMR (400 MHz, $CDCl_3$)** δ_H **2.02** (3H, dt, *J* 1.4, 0.9), **2.61** (1H, s), **4.33** (2H, dq, *J* 6.7, 0.9), **5.90** (1H, tq, *J* 6.7, 1.4), **6.92–7.01** (2H, m), **7.30–7.37** (2H, m).

(*E*)-3-(4-chlorophenyl)but-2-en-1-ol (S55)

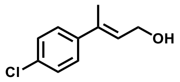

Following **General Procedure B**, ethyl (*E*)-3-(4-chlorophenyl)but-2-enoate (1.12 g, 5 mmol), DIBAL-H (11 mL, 11 mmol) in Et_2O (20 mL) gave a crude allylic alcohol product which was purified by flash column chromatography (eluent: hexane/EtOAc = 4:1) to afford the title compound (0.73 g, 80%) as a colourless oil with ^{1}H spectroscopic data in accordance with the literature [47]. **IR** ν_{max} (film) 3282 (OH), 1487, 1440, 1402, 1377, 1093 (C-Cl), 1062; **^{1}H NMR (400 MHz, $CDCl_3$)** δ_H **1.66** (1H, s), **2.05** (3H, dt, *J* 1.5, 0.8), **4.35** (2H, dq, *J* 6.7, 0.8), **5.95** (1H, tq, *J* 6.7, 1.4), **7.26–7.31** (2H, m), **7.31–7.35** (2H, m).

(*E*)-3-(4-Bromophenyl)but-2-en-1-ol (S56)

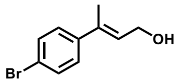

Following **General Procedure B**, ethyl (*E*)-3-(4-bromophenyl)but-2-enoate (1.34 g, 5 mmol), DIBAL-H (11 mL, 11 mmol) in Et_2O (20 mL) gave a crude allylic alcohol product which was purified by flash column chromatography (eluent: hexane/EtOAc = 4:1) to afford the title compound (1.02 g, 90%) as a colourless oil [48]. **IR** ν_{max} (film) 3313 (OH), 1483, 1442, 1400, 1105, 1078 (C-Br), 1006; **^{1}H NMR (400 MHz, $CDCl_3$)** δ_H **1.54** (1H, s), **2.05** (3H, dt, *J* 1.4, 0.8), **4.35** (2H, dq, *J* 6.6, 0.8), **5.96** (1H, tq, *J* 6.6, 1.4), **7.25–7.29** (2H, m), **7.42–7.46** (2H, m).

(*E*)-3-(4-(Trifluoromethyl)phenyl)but-2-en-1-ol (S57)

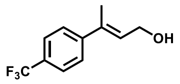

Following **General Procedure B**, ethyl (*E*)-3-(4-(trifluoromethyl)phenyl)but-2-enoate (1.29 g, 5 mmol), DIBAL-H (11 mL, 11 mmol) in Et_2O (20 mL) gave a crude allylic

alcohol product which was purified by flash column chromatography (eluent: hexane/EtOAc = 4:1) to afford the title compound (0.95 g, 88%) as a colourless oil with ^{1}H spectroscopic data in accordance with the literature [44]. **IR** ν_{max} (film) 3309 (OH), 1616 (C=C), 1411,1321 (CF_3), 1163, 1111, 1074, 1058; **^{1}H NMR (500 MHz, $CDCl_3$)** δ_H **1.96** (1H, s), **2.07** (3H, s), **4.38** (2H, d, *J* 6.5), **6.02** (1H, tq, *J* 6.5, 1.5), **7.48** (2H, app d, *J* 8.3), **7.56** (2H, app d, *J* 8.2).

(*E*)-3-([1,1'-Biphenyl]-4-yl)but-2-en-1-ol (S58)

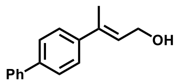

Following **General Procedure B**, ethyl (*E*)-3-([1,1'-biphenyl]-4-yl)but-2-enoate (1.33 g, 5 mmol), DIBAL-H (11 mL, 11 mmol) in Et_2O (20 mL) gave a crude allylic alcohol product which was purified by flash column chromatography (eluent: hexane/EtOAc = 4:1) to afford the title compound (0.95 g, 85%) as a colourless amorphous solid with ^{1}H spectroscopic data in accordance with the literature [43]. **IR** ν_{max} (film) 3242 (OH), 1485, 1406, 1363, 1267, 1070; **^{1}H NMR (500 MHz, $CDCl_3$)** δ_H **1.55** (1H, t, *J* 4.4), **2.13** (3H, dt, *J* 1.4, 0.8), **4.40** (2H, app dd, *J* 6.8, 3.5), **6.07** (1H, tq, *J* 6.7, 1.4), **7.33–7.39** (1H, m), **7.42–7.47** (2H, m), **7.49–7.53** (2H, m), **7.56–7.60** (2H, m), **7.60–7.64** (2H, m).

(*E*)-3-(p-Tolyl)but-2-en-1-ol (S59)

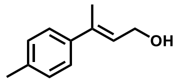

Following **General Procedure B**, ethyl (*E*)-3-(p-tolyl)but-2-enoate (1.02 g, 5 mmol), DIBAL-H (11 mL, 11 mmol) in Et_2O (20 mL) gave a crude allylic alcohol product which was purified by flash column chromatography (eluent: hexane/EtOAc = 4:1) to afford the title compound (0.71 g, 88%) as a colourless oil with ^{1}H spectroscopic data in accordance with the literature [43]. **IR** ν_{max} (film) 3311 (OH), 1600 (C=C), 1512, 1440, 1377, 1112, 1064; **^{1}H NMR (400 MHz, $CDCl_3$)** δ_H **1.62** (1H, s), **2.07** (1H, dt, *J* 1.4, 0.8), **2.36** (3H, s), **4.36** (2H, dq, *J* 6.7, 0.8), **5.97** (1H, tq, *J* 6.7, 1.4), **7.10–7.19** (2H, m), **7.28–7.37** (2H, m).

(*E*)-3-(4-methoxyphenyl)but-2-en-1-ol (S60)

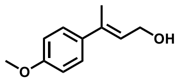

Following **General Procedure B**, ethyl (*E*)-3-(4-methoxyphenyl)but-2-enoate (1.10 g, 5 mmol), DIBAL-H (11 mL, 11 mmol) in Et_2O (20 mL) gave a crude allylic alcohol product which was purified by flash column chromatography (eluent: hexane/EtOAc = 4:1) to afford the title compound (0.71 g, 80%) as a colourless oil with ^1H spectroscopic data in accordance with the literature [43]. **IR** ν_{max} (film) 3349 (OH), 1604 (C=C), 1510, 1465, 1440, 1282, 1246 (C-O), 1180, 1066, 1026; **^{1}H NMR (400 MHz, CDCl$_3$)** δ_H **1.46** (1H, s), **2.06** (1H, dt, *J* 1.4, 0.8), **3.81** (3H, s), **4.35** (2H, dq, *J* 6.8, 0.8), **5.92** (1H, tq, *J* 6.8, 1.4), **6.85–6.89** (2H, m), **7.34–7.39** (2H, m).

(*E*)-3-(m-Tolyl)but-2-en-1-ol (S61)

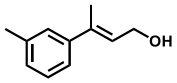

Following **General Procedure B**, ethyl (*E*)-3-(m-tolyl)but-2-enoate (1.10 g, 5 mmol), DIBAL-H (11 mL, 11 mmol) in Et_2O (20 mL) gave a crude allylic alcohol product which was purified by flash column chromatography (eluent: hexane/EtOAc = 4:1) to afford the title compound (0.71 g, 80%) as a colourless oil with ^1H spectroscopic data in accordance with the literature [43]. **IR** ν_{max} (film) 3325 (OH), 2920 (C-H), 1649 (C=C), 1602 (C=C), 1276, 1095; **^{1}H NMR (300 MHz, CDCl$_3$)** δ_H **1.47** (1H, s), **2.08** (3H, dt, *J* 1.4, 0.8), **2.37** (3H, s), **4.36** (2H, d (br), *J* 6.7), **5.97** (1H, tq, *J* 6.7, 1.4), **7.06–7.13** (1H, m), **7.19–7.25** (3H, m).

(*E*)-3-(o-Tolyl)but-2-en-1-ol (S62)

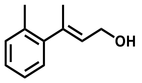

Following **General Procedure B**, ethyl (*E*)-3-(o-tolyl)but-2-enoate (1.02 g, 5 mmol), DIBAL-H (11 mL, 11 mmol) in Et_2O (20 mL) gave a crude allylic alcohol product which was purified by flash column chromatography (eluent: hexane/EtOAc = 4:1) to afford the title compound (0.73 g, 91%) as a colourless oil with ^1H spectroscopic data in accordance with the literature [49]. **IR** ν_{max} (film) 3307 (OH), 1485, 1436, 1377, 1215

(C-O) 1099; **^{1}H NMR (300 MHz, $CDCl_3$)** δ_H **1.76** (1H, s), **1.98** (1H, dt, *J* 1.5, 0.8), **2.30** (3H, s), **4.35** (2H, dq, *J* 6.7, 0.8), **5.55** (1H, tq, *J* 6.7, 1.5), **7.06–7.13** (1H, m), **7.14–7.21** (3H, m).

(*E*)-3-(Naphth-2-yl)but-2-en-1-ol (S63)

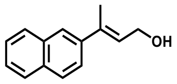

Following **General Procedure B**, ethyl (*E*)-3-(naphth-2-yl)but-2-enoate (1.20 g, 5 mmol), DIBAL-H (11 mL, 11 mmol) in Et_2O (20 mL) gave a crude allylic alcohol product which was purified by flash column chromatography (eluent: hexane/EtOAc = 4:1) to afford the title compound (0.81 g, 82%) as a colourless oil with ^{1}H spectroscopic data in accordance with the literature [43]. **IR** ν_{max} (film) 3250 (OH), 3055 (C-H), 1595 (C=C), 1369, 1168, 1130; **^{1}H NMR (400 MHz, $CDCl_3$)** δ_H **1.59** (1H, s), **2.20** (3H, s) **4.44** (2H, app dd, *J* 6.9, 1,4), **6.15** (1H, tq, *J* 6.7, 1.4), **7.46** (1H, ddd, *J* 7.5, 6.9, 1.6), **7.48** (1H, ddd, *J* 7.5, 6.8, 1.8), **7.60** (1H, dd, *J* 8.6, 1.8), **7.80** (1H, d, *J* 8.6), **7.80–7.85** (3H, m).

(*E*)-3-(Thiophen-2-yl)but-2-en-1-ol (S64)

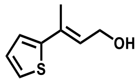

Following **General Procedure B**, ethyl (*E*)-3-(thiophen-2-yl)but-2-enoate (0.98 g, 5 mmol), DIBAL-H (11 mL, 11 mmol) in Et_2O (20 mL) gave a crude allylic alcohol product which was purified by flash column chromatography (eluent: hexane/EtOAc = 4:1) to afford the title compound (0.59 g, 76%) as a colourless oil with ^{1}H spectroscopic data in accordance with the literature [50]. **IR** ν_{max} (film) 3329 (OH), 1639 (C=C), 1435, 1381, 1246 (C-O); **^{1}H NMR (500 MHz, $CDCl_3$)** δ_H **2.08** (3H, s), **2.10** (1H, s), **4.32** (2H, d, *J* 6.9), **6.11** (1H, dq, *J* 6.8, 1.4, 1H), **6.97** (1H, dd, *J* 5.1, 3.6), **7.03** (1H, dd, *J* 3.6, 1.2), **7.15** (1H, dd, *J* 5.1, 1.2).

(*E*)-3-(Thiazol-2-yl)but-2-en-1-ol (S65)

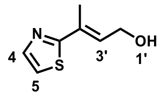

Following **General Procedure B**, ethyl (*E*)-3-(thiazol-2-yl)but-2-enoate (0.99 g, 5 mmol), DIBAL-H (11 mL, 11 mmol) in Et_2O (20 mL) gave a crude allylic alcohol product which was purified by flash column chromatography (eluent: hexane/EtOAc = 4:1) to afford the title compound (0.54 g, 70%) as a colourless oil. **IR** ν_{max} (film) 3307 (OH), 1487, 1417, 1381, 1249 (C-O), 1149, 1066, 1002; **^{1}H NMR (500 MHz, $CDCl_3$)** δ_H **2.15** (3H, s, C*H*$_3$), **2.61** (1H, s, O*H*), **4.39** (2H, dd, *J* 6.5, 1.1, C(2`)*H*$_2$), **6.55** (1H, tq, *J* 6.4, 1.4, C(3`)*H*), **7.20** (1H, d, *J* 3.3, C(5)*H*), **7.73** (1H, d, *J* 3.3, C(4)*H*); **^{13}C NMR (126 MHz, $CDCl_3$)** δ **15.2** (*C*H$_3$), **59.5** (*C*(2`)H$_2$), **118.4**(*C*(5)H), **131.3** (*C*(3`)H), **131.5** (*C*(4`)), **143.0** (*C*(3)H), **171.0** (*C*(2)); **HRMS (ESI$^+$)** $C_7H_{11}ONS$ $[M+H]^+$ found 156.0475, requires 156.0478 (–1.8 ppm).

(*Z*)-3-Fluoro-3-phenylprop-2-en-1-ol (S66)

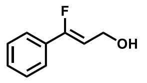

Following **General Procedure C**, ethyl (*Z*)-3-fluoro-3-phenylacrylate (0.97 g, 5 mmol), DIBAL-H (11 mL, 11 mmol) in Et_2O (20 mL) gave a crude allylic alcohol product which was purified by flash column chromatography (eluent: hexane/EtOAc = 4:1) to afford the title compound (0.66 g, 87%) as a colourless oil with ^{1}H and ^{19}F spectroscopic data in accordance with the literature [51]. **IR** ν_{max} (film) 3286 (OH), 1678 (C=C), 1494, 1448, 1280 (C-F), 1099 (C-O), 1033; **^{1}H NMR (500 MHz, $CDCl_3$)** δ_H **1.74** (1H, t, *J* 5.7), **4.45** (2H, ddd, *J* 7.1, 5.7, 2.1), **5.66** (1H, dt, *J* 36.6, 7.1), **7.31–7.43** (m, 3H), **7.48–7.58** (m, 2H); **^{19}F{^{1}H} NMR (470 MHz, $CDCl_3$)** δ **−117.24**.

(*Z*)-3-Fluoro-3-(p-tolyl)prop-2-en-1-ol (S67)

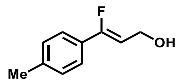

Following **General Procedure C**, ethyl (*Z*)-3-fluoro-3-(p-tolyl)acrylate (1.04 g, 5 mmol), DIBAL-H (11 mL, 11 mmol) in Et_2O (20 mL) gave a crude allylic alcohol product which was purified by flash column chromatography (eluent: hexane/EtOAc = 4:1) to afford the title compound (0.67 g, 81%) as a colourless oil with ^{1}H and ^{19}F spectroscopic data in accordance with the literature [51]. **IR** ν_{max} (film) 3334 (OH), 2953

(C-H), 1674 (C=C), 1512, 1384, 1278 (C-F), 1188, 1107 (C-O); **^{1}H NMR (500 MHz, CDCl$_3$)** δ_H **1.75** (1H, t, *J* 5.8), **2.37** (3H, s), **4.43** (2H, ddd, *J* 7.2, 5.8, 2.1), **5.60** (2H, dt, *J* 36.6, 7.2), **7.18** (2H, app d, *J* 8.0), **7.42** (2H, app d, *J* 8.3); **^{19}F{^{1}H} NMR (470 MHz, CDCl$_3$)** δ **−117.10**.

(*Z*)-3-Fluoro-3-(4-methoxyphenyl)prop-2-en-1-ol (S68)

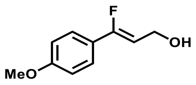

Following **General Procedure C**, ethyl (*Z*)-3-fluoro-3-(4-methoxyphenyl)acrylate (1.12 g, 5 mmol), DIBAL-H (11 mL, 11 mmol) in Et$_2$O (20 mL) gave a crude allylic alcohol product which was purified by flash column chromatography (eluent: hexane/EtOAc = 4:1) to afford the title compound (0.68 g, 75%) as a pale-yellow solid with ^{1}H and ^{19}F spectroscopic data in accordance with the literature [51]. **IR** ν_{max} (film) 3460 (OH), 2954 (C-H), 1674 (C=C), 1604 (C=C), 1510, 1415, 1280 (C-F), 1180, 1101 (C-O); **^{1}H NMR (500 MHz, CDCl$_3$)** δ_H **1.71** (1H, s), **3.82** (3H, s), **4.41** (2H, dd, *J* 7.2, 2.0), **5.51** (1H, dt, *J* 36.7, 7.2), **6.83–6.94** (2H, m), **7.39–7.52** (2H, m); **^{19}F{^{1}H} NMR (470 MHz, CDCl$_3$)** δ **−116.59**.

(*Z*)-3-Fluoro-3-[4-(trifluoromethyl)phenyl]prop-2-en-1-ol (S69)

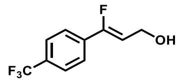

Following **General Procedure C**, ethyl (*Z*)-3-fluoro-3-(4-(trifluoromethyl)phenyl) acrylate (1.31 g, 5 mmol), DIBAL-H (11 mL, 11 mmol) in Et$_2$O (20 mL) gave a crude allylic alcohol product which was purified by flash column chromatography (eluent: hexane/EtOAc = 4:1) to afford the title compound (0.94 g, 86%) as a pale-yellow solid with ^{1}H and ^{19}F spectroscopic data in accordance with the literature [51]. **IR** ν_{max} (film) 3298 (OH), 1676 (C=C), 1620 (C=C), 1419, 1321 (CF$_3$), 1286, 1165, 1111 (C-O); **^{1}H NMR (400 MHz, CDCl$_3$)** δ_H **2.11** (1H, s), **4.47** (2H, dd, *J* 7.0, 2.2), **5.76** (1H, dt, *J* 36.3, 7.0), **7.61** (5H, s); **^{19}F{^{1}H} NMR (470 MHz, CDCl$_3$)** δ **−62.88** (3F, s), **−117.78** (1F, s).

(*E*)-3-(4-Methoxyphenyl)prop-2-en-1-ol (S70)

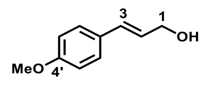

To a solution of (*E*)-3-(4-methoxyphenyl)acrylaldehyde (1.50 g, 9.27 mmol, 1.00 equiv.) in methanol (14 mL) was added $NaBH_4$ (0.458 g, 12.1 mmol, 1.31 equiv.) portionwise over 2 min while stirring at 0 °C. The mixture was allowed to warm to rt and stirred for 2 h, then cooled to 0 °C and acetone (5 mL) was added. The solution was stirred for 10 min, then allowed to warm to rt and diluted with sat. aq. NH_4Cl and Et_2O. The aqueous phase was extracted with Et_2O (×2), then the combined organic phases were washed sequentially with sat. aq. $NaHCO_3$ and brine, dried over $MgSO_4$, filtered, and concentrated *in vacuo*. The residue was purified by flash column chromatography (25:75 → 50:50 EtOAc/petrol) to give the title compound as a white solid (1.09 g, 62%) with ^{1}H spectroscopic data in accordance with the literature [52]. **^{1}H NMR (500 MHz, $CDCl_3$)** δ **1.58** (1H, s, O*H*), **3.81** (3H, s, C*H*$_3$), **4.29** (2H, d, *J* 5.9, C(1)*H*$_2$), **6.24** (1H, dt, *J* 15.9, 5.9, C(2)*H*), **6.55** (1H, dt, *J* 15.9, 1.6, C(3)*H*), **6.81–6.90** (2H, m, ArC(3`,5`)*H*), **7.28–7.36** (2H, m, ArC(2`,6`)*H*); **^{13}C{^{1}H} NMR (126 MHz, $CDCl_3$)** δ **55.4**, **64.0**, **114.1**, **126.4**, **127.8**, **129.5**, **131.1**, **159.4**.

(*E*)-3-[4-(Trifluoromethyl)phenyl]prop-2-en-1-ol (S71)

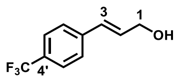

Following **General Procedure D**, ethyl (*E*)-3-(4-(trifluoromethyl)phenyl)acrylate (1.78 g, 8.80 mmol) and DIBAL-H (1.75 M in toluene, 11.5 mL, 20.2 mmol) in anhydrous CH_2Cl_2 (40 mL) at –78 °C gave, after purification by flash column chromatography (30:70 EtOAc/petrol), the title compound as a white solid (1.12 g, 63%) with ^{1}H spectroscopic data in accordance with the literature [53,54]. **^{1}H NMR (500 MHz, $CDCl_3$)** δ **1.66** (1H, t, *J* 5.6, O*H*), **4.26–4.44** (2H, m, C(1)*H*$_2$), **6.46** (1H, dt, *J* 16.0, 5.6, C(2)*H*), **6.66** (1H, dt, *J* 16.0, 1.8, C(3)*H*), **7.47** (2H, app. d, *J* 8.1, ArC(2`,6`)*H*), **7.57** (2H, app. d, *J* 8.1, ArC(3`,5`)*H*); **^{13}C{^{1}H} NMR (126 MHz, $CDCl_3$)** δ **63.5**, **124.3** (q, $^1J_{CF}$ 271.8, *C*F$_3$), **125.7** (q, $^3J_{CF}$ 3.6, Ar*C*(3`,5`)H), **126.7**, **129.4**, **129.6** (q, $^2J_{CF}$ 32.8, Ar*C*(4`)CF$_3$), **131.4**, **140.3**; **^{19}F{^{1}H} NMR (471 MHz, $CDCl_3$)** δ **−62.5** (C*F*$_3$).

(*E*)-3-(4-Fluorophenyl)prop-2-en-1-ol (S72)

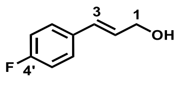

Following **General Procedure D**, ethyl (*E*)-3-(4-fluorophenyl)acrylate (1.774 g, 9.134 mmol) and DIBAL-H (1.0 m in hexanes, 22.8 mL, 22.8 mmol) in anhydrous CH_2Cl_2 (36.5 mL) gave, after purification by flash column chromatography (25:75 EtOAc/petrol), the title compound as a colourless solid (0.995 g, 72%) with spectroscopic data in accordance with the literature. [55] **^{1}H NMR (500 MHz, $CDCl_3$)** δ **1.46** (t, *J* 5.9, 1H, O*H*), **4.32** (td, *J* 5.8, 1.6, 2H, C(1)H_2), **6.29** (dt, *J* 15.8, 5.7, 1H, C(2)*H*), **6.59** (dt, *J* 15.8, 1.6, 1H, C(3)*H*), **6.95–7.10** (m, 2H, ArC(3`,5`)*H*), **7.32–7.41** (m, 2H, ArC(2`,6`)*H*). **^{13}C{^{1}H} NMR (126 MHz, $CDCl_3$)** δ **63.7** (*C*(1)), **115.6** (d, *J* 21.5, Ar*C*(3`,5`)H), **128.1** (d, *J* 8.1, Ar*C*(2`,6`)H), **128.4** (d, *J* 2.2, *C*(2)H), **130.1** (*C*(3)H), **133.0** (d, *J* 3.4, Ar*C*(1`)), **162.5** (d, *J* 246.9, Ar*C*(4`)F). **^{19}F{^{1}H} NMR (470 MHz, $CDCl_3$)** δ –114.3 (Ar*C*(4`)F).

(*E*)-3-(2-methoxyphenyl)prop-2-en-1-ol (S73)

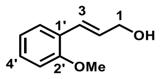

Following **General Procedure D**, ethyl (*E*)-3-(2-methoxyphenyl)acrylate (3.03 g, 14.7 mmol) and DIBAL-H (1.0 M in hexanes, 37.0 mL, 37.0 mmol) in anhydrous CH_2Cl_2 (40 mL) gave, after purification by flash column chromatography (25:75 EtOAc/petrol), the title compound as a yellow oil (2.00 g, 83%) with ^{1}H spectroscopic data in accordance with the literature [56]. **^{1}H NMR (500 MHz, $CDCl_3$)** δ **1.47** (1H, s, O*H*), **3.85** (3H, s, OCH_3), **4.33** (2H, d, *J* 5.9, C(1)H_2), **6.39** (1H, dt, *J* 16.0, 5.9, C(2)*H*), **6.88** (1H, dd, *J* 8.6, 1.1, ArC(3`)*H*), **6.90–6.97** (2H, m, C(3)*H* and ArC(5`)*H*), **7.24** (1H, ddd, *J* 8.6, 7.5, 1.7, ArC(4`)*H*), **7.44** (1H, dd, *J* 7.6, 1.7, ArC(6`)*H*); **^{13}C{^{1}H} NMR (126 MHz, $CDCl_3$)** δ **55.6**, **64.4**, **110.9**, **120.8**, **125.8**, **126.3**, **127.1**, **128.9**, **129.4**, **156.9**.

(*E*)-3-(1-naphthyl)prop-2-en-1-ol (S74)

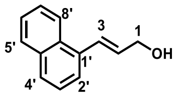

Following **General Procedure D**, methyl (*E*)-3-(1-naphthyl)acrylate (2.08 g, 9.80 mmol) and DIBAL-H (1.0 M in hexanes, 25.0 mL, 25.0 mmol) in anhydrous CH_2Cl_2 (40 mL) at 0 °C gave, after purification by flash column chromatography (25:75 EtOAc/petrol), the title compound as a yellow oil (1.78 g, quant.) with ^{1}H spectroscopic data in accordance with the literature [56]. **^{1}H NMR (500 MHz, $CDCl_3$)** δ **1.53** (1H, s, O*H*), **4.45** (2H, app td, *J* 5.8, 1.8, C(1)H_2), **6.41** (1H, dt, *J* 15.7, 5.6, C(2)*H*), **7.39** (1H, dd, *J* 15.7, 1.8, C(3)*H*), **7.45** (1H, dd, *J* 8.1, 7.2, ArC(3`)*H*), **7.47–7.54** (2H, m, ArC(6`, 7`)*H*), **7.60** (1H, dt, *J* 7.2, 1.0, ArC(2`)*H*), **7.79** (1H, d, *J* 8.1, ArC(4`)*H*), **7.86** (1H, dd, *J* 8.0, 1.6, ArC(5`)*H*), **8.13** (1H, dt, *J* 8.5, 1.2, ArC(8`)*H*); **^{13}C{^{1}H} NMR (126 MHz, $CDCl_3$)** δ **64.1**, **123.9**, **124.1**, **125.8**, **125.9**, **126.2**, **128.2**, **128.4**, **128.7**, **131.3**, **131.9**, **133.7**, **134.6**.

(*E*)-3-(2-naphthyl)prop-2-en-1-ol (S75)

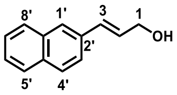

Following **General Procedure D**, methyl (*E*)-3-(2-naphthyl)acrylate (1.71 g, 8.06 mmol) and DIBAL-H (1.0 M in hexanes, 20.5 mL, 20.5 mmol) in anhydrous CH_2Cl_2 (40 mL) at 0 °C gave, after purification by flash column chromatography (20:80 EtOAc/petrol), the title compound as a colourless solid (1.38 g, 93%) with ^{1}H spectroscopic data in accordance with the literature [56]. **^{1}H NMR (500 MHz, $CDCl_3$)** δ **1.54** (t, *J* 5.7, 1H, O*H*), **4.39** (app td, *J* 5.7, 1.6, 2H, C(1)H_2), **6.50** (1H, dt, *J* 15.8, 5.7, C(2)*H*), **6.79** (1H, dt, *J* 15.8, 1.6, C(3)*H*), **7.39–7.50** (2H, m, ArC(6`,7`)*H*), **7.61** (1H, dd, *J* 8.6, 1.8, ArC(3`)*H*), **7.74** (1H, d, *J* 1.8, ArC(1`)*H*), **7.77–7.86** (3H, m, ArC(4`,5`,8`)*H*); **^{13}C{^{1}H} NMR (126 MHz, $CDCl_3$)** δ **64.0**, **123.7**, **126.1**, **126.4**, **126.6**, **127.8**, **128.1**, **128.4**, **129.0**, **131.4**, **133.2**, **133.7**, **134.3**.

(*E*)-3-(thiophen-3-yl)prop-2-en-1-ol (S76)

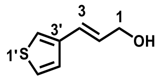

Following **General Procedure D**, ethyl (*E*)-3-(thiophen-3-yl)acrylate (576 mg, 3.16 mmol) and DIBAL-H (1.0 M in hexanes, 8.0 mL, 8.0 mmol) in anhydrous CH_2Cl_2 (16 mL) gave the title compound as a brown oil (419 mg, 95%) with ^{1}H spectroscopic data in accordance with the literature [57], which was used without further purification. **^{1}H NMR (500 MHz, $CDCl_3$)** δ **4.29** (2H, dd, *J* 5.8, 1.5, C(1)*H*$_2$), **6.22** (1H, dt, *J* 15.8, 5.8, C(2)*H*), **6.63** (1H, dt, *J* 15.9, 1.7, C(3)*H*), **7.16** (1H, dd, *J* 3.1, 1.2, ArC(2`)*H*), **7.22** (1H, dd, *J* 5.1, 1.3, ArC(4`)*H*), **7.28** (1H, dd, *J* 5.1, 2.9, ArC(5`)*H*). **$^{13}$C NMR (126 MHz, $CDCl_3$)** δ **63.8** (*C*H$_2$), **122.5** (Ar*C*(2`)), **125.1** (Ar*C*(4`)), **125.6** (*C*(3)), **126.3** (Ar*C*(5`)), **128.4** (*C*(2)), **139.4** (Ar*C*(3`)).

8.4 Supplementary Data for allylic ethers

1-Benzyl-3-[(3,3-diphenylallyl)oxy]indolin-2-one (S1)

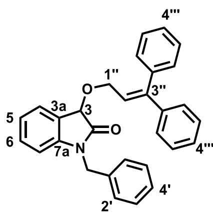

Following **General Procedure F**, 1-benzyl-3-diazoindolin-2-one (0.548 g, 2.2 mmol), 3,3-diphenylprop-2-en-1-ol (1.05 g, 2.0 mmol), $Rh_2(OAc)_4$ (0.0044 g, 0.01 mmol) in CH_2Cl_2 (40 mL) gave a crude allylic ether product which was purified by flash column chromatography (eluent: hexane/CH_2Cl_2/EtOAc = 15:5:2) to afford the title compound (0.56 g, 65%) as a colourless amorphous solid. **IR** ν_{max} (film) 1716 (C=O), 1614 (C=C), 1489, 1465, 1442, 1355, 1348, 1170, 1105, 1078 (C-O); **^{1}H NMR (400 MHz, $CDCl_3$)** δ **4.35** (1H, ddd, *J* 11.7, 7.2, 1.2, OC(1``)$H^AH^B$), **4.51** (1H, ddd, *J* 11.7, 6.6, 1.2, OC(1``)H^AH^B), **4.78–4.92** (2H, m, NCH_2Ph), **5.01** (1H, s, C(3)*H*), **6.29–6.40** (1H, m, C(2``)*H*), **6.66** (1H, d, *J* 7.8, ArC(7)*H*), **7.00** (1H, t, *J* 7.5, ArC(5)*H*), **7.15–7.22** (3H, m, ArC*H*), **7.25–7.33** (11H, m, ArC*H*), **7.33–7.40** (3H, m, ArC*H*); **$^{13}$C{$^1$H} NMR (101 MHz, $CDCl_3$)** δ **43.8** (N*C*$H_2$Ph), **67.1** (O*C*(1``)H_2), **75.4** (O*C*(3)H), **109.5** (Ar*C*(7)H), **123.0** (Ar*C*(5)H), **124.8** (*C*(2``)*H*), **125.3** (Ar*C*(3a)), **125.5** (Ar*C*H), **127.4** (Ar*C*(2`,6`)H), **127.7** (Ar*C*H), **127.8** (Ar*C*H), **127.8** (Ar*C*H), **128.3** (Ar*C*(6)H), **128.3** (Ar*C*H), **128.3** (Ar*C*H), **128.9** (Ar*C*H), **129.9** (Ar*C*H), **135.6** (*C*(1`)), **139.1** (Ar*C*), **141.9** (Ar*C*(1```)), **143.4** (Ar*C*(7a)), **145.8** (Ar*C*(3``)), **174.8** (*C*=O); **HRMS (ESI$^+$)** $C_{30}H_{25}NO_2Na$ [M+Na]$^+$ found 454.1777, requires 454.1778 (–0.18 ppm).

3-[(3,3-Diphenylallyl)oxy]-1-methylindolin-2-one (S2)

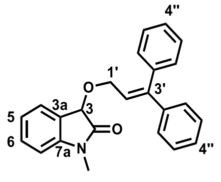

Following **General Procedure F**, 3-diazo-1-methylindolin-2-one (0.381 g, 2.2 mmol), 3,3-diphenylprop-2-en-1-ol (1.05 g, 2.0 mmol), $Rh_2(OAc)_4$ (0.0044 g, 0.01 mmol) in CH_2Cl_2 (40 mL) gave a crude allylic ether product which was purified by flash column chromatography (eluent: hexane/CH_2Cl_2/EtOAc = 15:5:2) to afford the title compound (0.43 g, 60%) as a colourless amorphous solid. **IR** ν_{max} (film) 1714 (C=O), 1612 (C=C), 1492, 1467, 1371, 1342, 1257, 1111, 1087 (C-O); **^{1}H NMR (500 MHz, $CDCl_3$)** δ **3.14** (3H, s, C*H*$_3$), **4.31** (1H, dd, *J* 11.9, 7.2, OC(1`)*H*$^A$H$^B$), **4.47** (1H, dd, *J* 11.8, 6.6, OC(1`)H^A*H*^{B}), **4.89** (1H, s, C(3)*H*), **6.30** (1H, t, *J* 6.9, C(2`)*H*), **6.77** (1H, d, *J* 7.8, ArC(7)*H*), **7.03** (1H, t, *J* 7.5, ArC(5)*H*), **7.10–7.16** (2H, m, ArC*H*), **7.22–7.38** (10H, m, ArC*H*); **$^{13}$C{$^1$H} NMR (126 MHz, $CDCl_3$)** δ **26.2** (C*H*$_3$), **67.1** (O*C*(1`)H$_2$), **75.4** (O*C*(3)H), **108.4** (Ar*C*(7)H), **122.9** (Ar*C*(5)H), **124.9** (*C*(2`)*H*), **125.3** (Ar*C*(3a)), **125.4** (Ar*C*H), **127.7** (Ar*C*H), **127.8** (Ar*C*H), **127.8** (Ar*C*H), **128.3** (Ar*C*H), **129.9** (Ar*C*H), **130.0** (Ar*C*(6)H), **135.6** (*C*(1`)), **139.1** (Ar*C*), **141.9** (Ar*C*(1``)), **144.3** (Ar*C*(7a)), **145.7** (Ar*C*), **174.7** (*C*=O); **HRMS (ESI$^+$)** $C_{24}H_{21}NO_2Na$ [M+Na]$^+$ found 378.1456, requires 378.1464 (–2.25 ppm).

3-[(3,3-Diphenylallyl)oxy]-1-tritylindolin-2-one (S3)

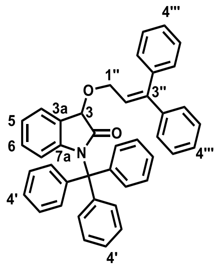

Following **General Procedure F**, 3-diazo-1-tritylindolin-2-one (0.882 g, 2.2 mmol), 3,3-diphenylprop-2-en-1-ol (1.05 g, 2.0 mmol), $Rh_2(OAc)_4$ (0.0044 g, 0.01 mmol) in CH_2Cl_2 (40 mL) gave a crude allylic ether product which was purified by flash column chromatography (eluent: hexane/CH_2Cl_2/EtOAc = 15:5:2) to afford the title compound (0.79 g, 68%) as a colourless amorphous solid. **IR** ν_{max} (film) 1732 (C=O), 1608 (C=C), 1597 (C=C), 1490, 1477, 1463, 1448, 1371, 1338, 1303, 1267, 1283, 1184, 1114 (C=C), 1041; **^{1}H NMR (500 MHz, CDCl$_3$)** δ **4.19** (1H, dd, *J* 11.7, 7.2, OC(1``)$H^AH^B$), **4.40** (1H, dd, *J* 11.7, 6.5, OC(1``)H^AH^B), **4.96** (1H, s, C(3)*H*), **6.21–6.31** (2H, m, ArC(7)*H*, C(2``)*H*), **6.88–6.95** (2H, m, ArC(5,6)*H*), **7.16–7.29** (17H, m, ArC*H*), **7.32–7.36** (3H, m, ArC*H*), **7.42–7.47** (6H, m, ArC(2`,6`)*H*); **$^{13}$C{$^1$H} NMR (126 MHz, CDCl$_3$)** δ **67.2** (O*C*(1``)H_2), **74.5** (*NC*Ph_3), **75.7** (O*C*(3)HCO), **116.0** (Ar*C*(7)H), **122.4** (Ar*C*(5)H), **124.7** (Ar*C*(4)H), **125.1** (*C*(2``)), **126.0** (Ar*C*(3a)), **127.0** (Ar*C*(4`)H), **127.6** (Ar*C*(4```)H), **127.7** (Ar*C*H), **127.8** (Ar*C*(3`,5`)H), **127.9** (Ar*C*(3```,5```)), **128.2** (Ar*C*(6)H), **128.3** (Ar*C*H), **128.3** (Ar*C*H), **129.3** (Ar*C*(2`,6`)H), **129.9** (Ar*C*H), **139.1** (*C*(1```)), **142.0** (Ar*C*(1`)), **144.0** (Ar*C*(7a)), **145.4** (*C*(3``)), **176.6** (*C*=O); **HRMS (ESI$^+$)** $C_{42}H_{33}NO_2Na$ $[M+Na]^+$ found 606.2400, requires 606.2404 (–0.66 ppm).

(*E*)-3-[(3-Phenylbut-2-en-1-yl)oxy]-1-tritylindolin-2-one (8)

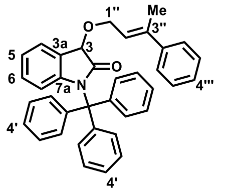

Following **General Procedure F**, 3-diazo-1-tritylindolin-2-one (0.882 g, 2.2 mmol), (*E*)-3-phenylbut-2-en-1-ol (0.296 g, 2.0 mmol), $Rh_2(OAc)_4$ (0.0044 g, 0.01 mmol) in CH_2Cl_2 (40 mL) gave a crude allylic ether product which was purified by flash column chromatography (eluent: hexane/CH_2Cl_2/EtOAc = 15:5:2) to afford the title compound (0.70 g, 67%) as a colourless amorphous solid. **IR** ν_{max} (film) 1738 (C=O), 1674, 1606 (C=C), 1593 (C=C), 1490, 1463, 1446, 1330, 1309, 1265 (C-O), 1091; **^{1}H NMR** (500 MHz, $CDCl_3$) δ 1.94 (3H, s, C*H*$_3$), 4.23 (1H, dd, *J* 11.8, 6.9, OC(1``)$H^A$H$^B$), 4.34 (1H, dd, *J* 11.8, 6.5, OC(1``)H^AH^B), 4.95 (1H, s, C(3)*H*), 5.84–5.93 (1H, m, C(2``)*H*), 6.18 (1H, dd, *J* 7.5, 1.5, ArC(7)*H*), 6.82–6.89 (2H, m ArC(5,6)*H*), 7.10–7.14 (3H, m, ArC(4`)*H*), 7.15–7.20 (7H, m, ArC(3`,5`,4```)*H*), 7.21–7.28 (3H, m, ArC(3```,5```,4)*H*), 7.30–7.33 (2H, m, ArC(2```,6```)*H*), 7.37–7.42 (6H, m, ArC(2`,6`)*H*); **^{13}C{^{1}H} NMR** (126 MHz, $CDCl_3$) δ 16.4 (*C*H$_3$), 66.0 (O*C*(1``)H$_2$), 74.5 (*NC*Ph$_3$), 75.3 (O*C*(3)HCO), 116.1 (Ar*C*(7)H), 122.5 (Ar*C*(5)H), 123.7 (*C*(2``)H), 124.7 (Ar*C*(4)H), 126.0 (Ar*C*(2```,6```)H), 126.1 (Ar*C*(3a)), 127.0 (Ar*C*(4`)H), 127.4 (Ar*C*(4```)), 127.8 (Ar*C*(3`,5`)H), 128.3 (Ar*C*(3```,5```)), 128.4 (Ar*C*(6)H), 129.3 (Ar*C*(2`,6`)H), 139.7 (*C*(3``)), 142.1 (Ar*C*(1`)), 142.9 (Ar*C*(1```)), 144.1 (Ar*C*(7a)), 176.9 (*C*=O); **HRMS (ESI$^+$)** $C_{37}H_{31}NO_2Na$ $[M+Na]^+$ found 524.2241, requires 524.2247 (–1.1 ppm).

(*E*)-3-[(3-Phenylpent-2-en-1-yl)oxy]-1-tritylindolin-2-one (S77)

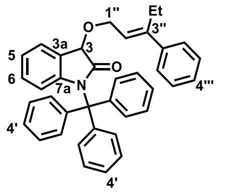

Following **General Procedure F**, 3-diazo-1-tritylindolin-2-one (0.882 g, 2.2 mmol), (*E*)-3-phenylpent-2-en-1-ol (0.324 g, 2.0 mmol), $Rh_2(OAc)_4$ (0.0044 g, 0.01 mmol) in CH_2Cl_2 (40 mL) gave a crude allylic ether product which was purified by flash column chromatography (eluent: hexane/CH_2Cl_2/EtOAc = 15:5:2) to afford the title compound (0.73 g, 68%) as a colourless amorphous solid. **IR** ν_{max} (film) 1726 (C=O), 1608 (C=C), 1597 (C=C), 1490, 1477, 1463, 1448, 1338, 1303, 1265, 1186, 1155, 1107 (C-O), 1031, 1001; ^{1}**H NMR (500 MHz, CDCl$_3$)** δ **0.85** (3H, t, *J* 7.5, CH_2CH_3), **2.32–2.48** (2H, m, CH_2CH_3), **4.24** (1H, dd, *J* 11.8, 7.1, OC(1``)$H^AH^B$), **4.32** (1H, dd, *J* 11.8, 6.5, OC(1``)H^AH^B), **4.95** (1H, s, C(3)*H*), **5.74** (1H, t, *J* 6.8, C(2``)*H*), **6.18** (1H, dd, *J* 7.4, 1.6, ArC(7)*H*), **6.80–6.89** (2H, m, ArC(5,6)*H*), **7.09–7.14** (3H, m, ArC(4`)*H*), **7.15–7.20** (7H, m, ArC(3`,5`,4```)*H*), **7.20–7.30** (5H, m, ArC(2```,3```,5````,6```,4)*H*), **7.36–7.43** (6H, m, ArC(2`,6`)*H*); ^{13}**C{^{1}H} NMR (126 MHz, CDCl$_3$)** δ **14.0** (CH_2CH_3), **23.5** (CH_2CH_3), **65.7** (O*C*(1``)$H_2$), **74.5** (*NC*$Ph_3$), **75.2** (O*C*(3)HCO), **116.0** (Ar*C*(7)H), **122.4** (Ar*C*(5)H), **123.4** (*C*(2``)H), **124.7** (Ar*C*(4)), **126.1** (Ar*C*(3a)), **126.6** (Ar*C*(3```,5```)H), **127.0** (Ar*C*(4`)H), **127.3** (Ar*C*(4```)), **127.8** (Ar*C*(3`,5`)H), **128.4** (Ar*C*(6,2```,6````)H), **129.3** (Ar*C*(2`,6`)H), **142.0** (*C*(3``)), **142.1** (*C*(1``)), **144.1** (Ar*C*(7a)), **146.5** (Ar*C*(1```)), **177.0** (*C*=O); **HRMS (ESI$^+$)** $C_{38}H_{33}NO_2Na$ $[M+Na]^+$ found 558.2399, requires 558.2404 (–0.81 ppm).

(*E*)-3-[(3-Cyclopropyl-3-phenylallyl)oxy]-1-tritylindolin-2-one (S78)

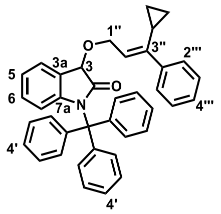

Following **General Procedure F**, 3-diazo-1-tritylindolin-2-one (0.882 g, 2.2 mmol), (*E*)-3-cyclopropyl-3-phenylprop-2-en-1-ol, $Rh_2(OAc)_4$ (0.0044 g, 0.01 mmol) in CH_2Cl_2 (40 mL) gave a crude allylic ether product which was purified by flash column chromatography (eluent: hexane/CH_2Cl_2/EtOAc = 15:5:2) to afford the title compound (0.71 g, 65%) as a colourless amorphous solid. **IR** ν_{max} (film) 1732 (C=O), 1606 (C=C), 1597 (C=C), 1490, 1477, 1463, 1448, 1334, 1303, 1269, 1232, 1184, 1151, 1116 (C-O), 1085; **^{1}H NMR (500 MHz, $CDCl_3$)** δ **0.34** (2H, d, *J* 5.4, C(3``)CHC*H*$_2$), **0.78** (2H, d, *J* 8.5, C(3``)CHC*H*$_2$), **1.74** (1H, q, *J* 7.0, C(3``)C*H*$CH_2CH_2$), **4.54** (1H, dd, *J* 12.0, 6.9, OC(1``)H^AH^B), **4.61** (1H, dd, *J* 11.9, 6.4, OC(1``)$H^AH^B$), **5.10** (1H, s, C(3)*H*$OCH_2$), **5.90** (1H, t, *J* 6.7, C(2``)*H*), **6.30** (1H, d, *J* 7.7, ArC(7)*H*), **6.87–7.03** (2H, m, ArC(5,6)*H*), **7.19–7.42** (15H, m, ArC*H*), **7.52** (6H, d, *J* 8.0, ArC(2`,6`)*H*); **^{13}C{^{1}H} NMR (126 MHz, $CDCl_3$)** δ **6.5** (C(3``)CH*C*$(H^A)_2C(H^B)_2$), **6.6** (C(3``)CHC$(H^A)_2$*C*$(H^B)_2$), **11.8** (C(3``)*C*HC$(H^A)_2C(H^B)_2$), **65.9** (O*C*(1`)$H_2$CH), **74.5** (*N*C$Ph_3$), **75.4** (O*C*(3)HCO), **116.0** (Ar*C*(7)H), **122.4** (Ar*C*(5)H), **124.7** Ar(*C*(4)H), **126.1** (Ar*C*(3a)), **126.5** (*C*(2``)*H*), **127.0** (Ar*C*(4`)H), **127.5** (Ar*C*(4```)H), **127.8** (Ar*C*(3`,5`)H), **127.9** (Ar*C*H), **128.3** (Ar*C*(6)H), **129.3** (Ar*C*(2`,6`)), **141.3** (*C*(3``)), **142.1** (ArC(1`)), **144.1** (Ar*C*(7a)), **144.9** (Ar*C*(1```)), **176.9** (*C*=O); **HRMS (ESI$^+$)** $C_{39}H_{33}NO_2Na$ $[M+Na]^+$ found 570.2383, requires 570.2404 (–3.6 ppm).

(*E*)-3-[(4-Methyl-3-phenylpent-2-en-1-yl)oxy]-1-tritylindolin-2-one (S79)

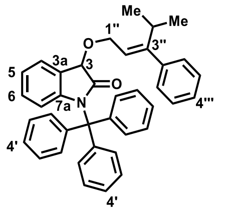

Following **General Procedure F**, 3-diazo-1-tritylindolin-2-one (0.882 g, 2.2 mmol), (*E*)-4-methyl-3-phenylpent-2-en-1-ol (0.352 g, 2.0 mmol), $Rh_2(OAc)_4$ (0.0044 g, 0.01 mmol) in CH_2Cl_2 (40 mL) gave a crude allylic ether product which was purified by flash column chromatography (eluent: hexane/CH_2Cl_2/EtOAc = 15:5:2) to afford the title compound (0.66 g, 60%) as a colourless amorphous solid. **IR** ν_{max} (film) 2962 (C-H), 1728 (C=O), 1608 (C=C), 1597 (C=C), 1490, 1463, 1448, 1303, 1111 (C-O); **^{1}H NMR (400 MHz, $CDCl_3$)** δ **0.99** (3H, d, *J* 7.0, CH(C*H*$_3$)^A(CH$_3$)^B), **1.01** (3H, d, *J* 7.0, CH(CH$_3$)^A(C*H*$_3$)^B), **2.95** (1H, hept, *J* 7.4, C*H*(CH$_3$)$_2$), **4.29–4.45** (2H, m, OC(1``)*H*$_2$CH), **5.04** (1H, s, C(3)*H*), **5.47** (1H, app t, *J* 6.7, C(2``)*H*), **6.21–6.29** (1H, m, ArC(7)*H*), **6.90–6.97** (2H, m, ArC(5,6)*H*), **7.15–7.25** (8H, m, ArC*H*), **7.27–7.43** (7H, m, ArC*H*), **7.45–7.51** (6H, m, ArC(2`,6`)*H*); **^{13}C{^{1}H} NMR (101 MHz, $CDCl_3$)** δ **22.0** (CH(*C*H$_3$)$_2$), **29.9** (*C*H(CH$_3$)$_2$), **65.0** (O*C*(1``)H$_2$), **74.5** (*NC*Ph$_3$), **75.2** (O*C*(3)HCO), **116.0** (Ar*C*(7)H), **122.4** (Ar*C*(5)H), **124.6** (*C*(2``)H), **124.7** (Ar*C*(4)H), **126.1** (Ar*C*(3a)), **126.7** (Ar*C*H), **127.0** (Ar*C*(4`)H), **127.7** (Ar*C*(2```,6```)H), **127.8** (Ar*C*(3`,5`)H), **128.1** (Ar*C*(4```)H), **128.4** (Ar*C*(6)H), **128.6** (Ar*C*(3```,5```)H), **129.3** (Ar*C*(2`,6`)H), **142.1** (Ar*C*(1`)), **142.3** (*C*(3``)), **144.1** (Ar*C*(7a)), **151.4** (Ar*C*(1```)), **177.0** (*C*=O); **HRMS (ESI$^+$)** $C_{39}H_{35}NO_2Na$ [M+Na]$^+$ found 572.2552, requires 572.2560 (–1.4 ppm).

3-[(3-Methylbut-2-en-1-yl)oxy]-1-tritylindolin-2-one (S80)

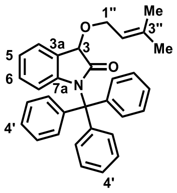

Following **General Procedure F**, 3-diazo-1-tritylindolin-2-one (0.882 g, 2.2 mmol), 3-methylbut-2-en-1-ol (0.172 g, 2.0 mmol), $Rh_2(OAc)_4$ (0.0044 g, 0.01 mmol) in CH_2Cl_2 (40 mL) gave a crude allylic ether product which was purified by flash column chromatography (eluent: hexane/CH_2Cl_2/EtOAc = 15:5:2) to afford the title compound (0.32 g, 35%) as a colourless amorphous solid. **IR** ν_{max} (film) 1728 (C=O), 1608 (C=C), 1597 (C=C), 1490, 1477, 1463, 1448, 1336, 1303, 1269, 1186, 1155, 1103 (C-O), 1083; **^{1}H NMR (400 MHz, $CDCl_3$)** δ **1.66** (3H, s, C(4``)$H_3$), **1.76** (3H, s, C(5``)H_3), **4.09** (1H, dd, *J* 10.7, 7.5, OC(1``)$H^AH^B$), **4.23** (1H, dd, *J* 10.7, 7.0, OC(1``)H^AH^B), **4.96** (1H, s, C(3)*H*OCH$_2$), **5.34–5.47** (1H, m, C(2``)*H*), **6.26** (1H, d, *J* 7.3, ArC(7)*H*), **6.87–6.98** (2H, m, ArC(5,6)*H*), **7.17–7.23** (3H, m, ArC(4`)*H*), **7.26** (6H, dd, *J* 9.9, 4.8, ArC(3`,5`)*H*), **7.31–7.35** (1H, m, ArC(4)*H*), **7.43–7.53** (6H, m, ArC(2`,6`)*H*); **$^{13}$C{$^1$H} NMR (126 MHz, $CDCl_3$)** δ **18.2** (*C*(4``)H$_3$), **25.9** (*C*(5``)H$_3$), **65.4** (O*C*(1``)H$_2$CH), **74.4** (*NC*Ph$_3$), **75.0** (O*C*(3)HCO), **115.9** (Ar*C*(7)H), **120.5** (*C*(2``)*H*), **122.2** (Ar*C*(5)H), **124.6** (Ar*C*(4)H), **126.2** (Ar*C*(3a)), **126.9** (Ar*C*(4`)H), **127.7** (Ar*C*(3`,5`)H), **128.2** (ArC(6)H), **129.2** (Ar*C*(2`,6`)H), **138.3** (*C*(3``)), **142.0** (Ar*C*(1`)), **144.0** (Ar*C*(7a)), **176.9** (*C*=O); **HRMS (ESI$^+$)** $C_{32}H_{29}NO_2Na$ $[M+Na]^+$ found 482.2084, requires 482.2091 (–1.3 ppm).

(*Z*)-3-[(3-Phenylbut-2-en-1-yl)oxy]-1-tritylindolin-2-one (S81)

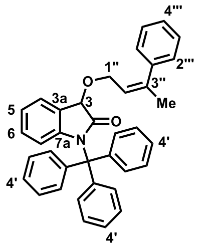

Following **General Procedure F**, 3-diazo-1-tritylindolin-2-one (0.882 g, 2.2 mmol), (*Z*)-3-phenylbut-2-en-1-ol, $Rh_2(OAc)_4$ (0.0044 g, 0.01 mmol) in CH_2Cl_2 (40 mL) gave a crude allylic ether product which was purified by flash column chromatography (eluent: hexane/CH_2Cl_2/EtOAc = 15:5:2) to afford the title compound (0.53 g, 51%) as a colourless amorphous solid. **IR** ν_{max} (film) 1728 (C=O), 1608 (C=C), 1597 (C=C), 1490, 1477, 1463, 1448, 1338, 1303, 1267, 1184, 1155, 1103 (C-O), 1085; **^{1}H NMR (400 MHz, CDCl$_3$)** δ **2.11** (3H, d, *J* 1.3, C*H*$_3$), **3.93–4.13** (1H, m, OC(1``)$H^A$H$^B$), **4.20–4.34** (1H, m, OC(1``)H^AH^B), **4.92** (1H, s, C(3)*H*OCH$_2$), **5.70–5.81** (1H, m, C(2``)*H*), **6.22–6.28** (1H, m, ArC(7)*H*), **6.87–6.93** (2H, m, ArC(5,6)*H*), **7.17–7.30** (13H, m, ArC*H*), **7.31–7.36** (2H, m, ArC*H*), **7.42–7.47** (6H, m, ArC(2`,6`)*H*); **$^{13}$C{$^1$H} NMR (101 MHz, CDCl$_3$)** δ **25.5** (*C*H$_3$), **66.8** (O*C*(1``)H$_2$), **74.4** (*NC*Ph$_3$), **75.5** (O*C*(3)HCO), **116.0** (Ar*C*(7)H), **122.3** (Ar*C*(5)H), **123.4** (*C*(2``)*H*), **124.7** (Ar*C*(4)H), **126.0** (Ar*C*(3a)), **126.9** (Ar*C*(4`)H), **127.3** (Ar*C*(4```)H), **127.8** (Ar*C*(3`,5`)H), **127.9** (Ar*C*H), **128.2** (Ar*C*(6)H), **129.3** (Ar*C*(2`,6`)H), **140.7** (*C*(3``)), **141.8** (Ar*C*(1```)), **142.0** (Ar*C*(1`)), **144.0** (Ar*C*(7a)), **176.6** (*C*=O); **HRMS (ESI$^+$)** $C_{37}H_{31}NO_2Na$ $[M+Na]^+$ found 524.2242, requires 524.2247 (–0.86 ppm).

(*E*)-3-[(1-Cyclopropyl-3-phenylbut-2-en-1-yl)oxy]-1-tritylindolin-2-one (S82)

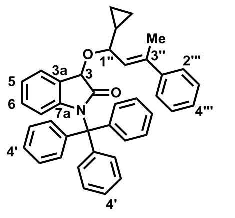

Following **General Procedure F**, 3-diazo-1-tritylindolin-2-one (0.882 g, 2.2 mmol), (*E*)-1-cyclopropyl-3-phenylbut-2-en-1-ol (0.376 g, 2.0 mmol), $Rh_2(OAc)_4$ (0.0044 g, 0.01 mmol) in CH_2Cl_2 (40 mL) gave a crude allylic ether product which was purified by flash column chromatography (eluent: hexane/CH_2Cl_2/EtOAc = 15:5:2) to afford the title compound (0.50 g, 45%) as a colourless amorphous solid. **IR** ν_{max} (film) 1722 (C=O), 1610 (C=C), 1597 (C=C), 1492, 1479, 1463, 1448, 1338, 1305, 1273, 1186, 1155, 1101 (C-O), 1083, 1062; **^{1}H NMR (400 MHz, $CDCl_3$)** δ **0.28–0.39** (1H, m, CH((C$H^A$$H^B$)(C$H^C$$H^D$))), **0.45–0.54** (2H, m, CH((C$H^A$$H^B$)(C$H^C$$H^D$))), **0.64–0.55** (1H, m, CH(C$H^A$$H^B$)(C$H^C$$H^D$)), **1.10–1.21** (1H, m, C*H*(C$H^A$$H^B$)(C$H^C$$H^D$)), **2.13** (3H, d, *J* 1.3, CH_3), **4.67** (1H, dd, *J* 9.6, 7.9, OC(1``)*H*), **4.81** (1H, s, C(3)*H*OCH), **5.87** (1H, m, C(2``)*H*), **6.18** (1H, dd, *J* 7.1, 1.7, ArC(7)*H*), **6.84–6.93** (2H, m, C(5,6)*H*), **7.17–7.22** (3H, m, ArC(4`)*H*), **7.22–7.26** (5H, m, ArC*H*), **7.26–7.28** (2H, m, ArC*H*), **7.29–7.32** (1H, m, ArC*H*), **7.34–7.39** (2H, m, ArC(3```,5```)*H*), **7.45–7.50** (8H, m, ArC(2`,6`,2```,6```)*H*); **^{13}C{^{1}H} NMR (101 MHz, $CDCl_3$) 2.3** (CH((*C*$H^A$$H^B$)(C$H^C$$H^D$))), **3.6** (CH((C$H^A$$H^B$)(*C*$H^C$$H^D$))), **16.1** (*C*H(C$H^A$$H^B$)(C$H^C$$H^D$))), **16.9** (*C*$H_3$), **72.0** (O*C*(3)HCO), **74.0** (*N*CPh$_3$), **79.7** (O*C*(1``)H), **115.6** (Ar*C*(7)H), **122.3** (Ar*C*(5)H), **124.8** Ar(*C*(4)H), **126.1** (Ar*C*(2```,6```)H), **126.9** (Ar*C*(4`)H), **127.1** (*C*(2``)H), **127.6** (Ar*C*(4```)H), **127.8** (Ar*C*(3`,5`)H), **128.2** (Ar*C*H), **128.5** (Ar*C*(6)H), **129.3** (Ar*C*(2`,6`)), **140.3** (*C*(3``)), **142.2** (ArC(1`)), **143.0** (Ar*C*(7a)), **143.9** (Ar*C*(1```)), **177.5** (*C*=O); **HRMS (ESI$^+$)** $C_{40}H_{35}NO_2Na$ $[M+Na]^+$ found 584.2548, requires 584.2560 (–2.0 ppm).

(*Z*)-3-[(4,4,4-Trifluoro-3-phenylbut-2-en-1-yl)oxy]-1-tritylindolin-2-one (S83)

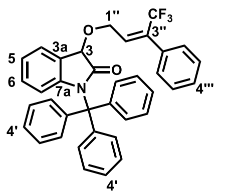

Following **General Procedure F**, 3-diazo-1-tritylindolin-2-one (0.882 g, 2.2 mmol), (*Z*)-4,4,4-trifluoro-3-phenylbut-2-en-1-ol (0.404 g, 2.0 mmol), $Rh_2(OAc)_4$ (0.0044 g, 0.01 mmol) in CH_2Cl_2 (40 mL) gave a crude allylic ether product which was purified by flash column chromatography (eluent: hexane/CH_2Cl_2/EtOAc = 15:5:2) to afford the title compound (0.62 g, 54%) as a colourless amorphous solid. **IR** ν_{max} (film) 1738 (C=O), 1608 (C=C), 1599 (C=C), 1467, 1446, 1298, 1184, 1170 (C-F), 1124 (C-O), 1105, 1089; **^{1}H NMR (400 MHz, $CDCl_3$)** δ **3.98–4.10** (1H, m, OC(1``)$H^A$H$^B$), **4.25–4.37** (1H, m, OC(1``)H^AH^B), **4.88** (1H, app s, C(3)*H*), **6.20–6.27** (1H, m, ArC(7)*H*), **6.50–6.65** (1H, m, C(2``)*H*), **6.88–6.96** (2H, m, ArC(5,6)*H*), **7.16–7.26** (12H, m, ArC*H*), **7.33–7.38** (3H, m, ArC*H*), **7.38–7.43** (6H, m, ArC(2`,6`)*H*); **^{13}C{^{1}H} NMR (101 MHz, $CDCl_3$)** δ **65.5** (O*C*(1`)$H_2$), **74.6** (*NC*$Ph_3$), **76.0** (O*C*(3)HCO), **116.2** (Ar*C*(7)H), **122.6** (Ar*C*(5)H), **123.1** (q, $^1J_{C\text{-}F}$ 274.6, *C*$F_3$), **124.7** (Ar*C*(4)H), **125.3** (Ar*C*(3a)), **127.1** (Ar*C*(4`)H), **127.8** (Ar*C*(3`,5`)H), **128.7** (Ar*C*(6)H), **129.1** (Ar*C*H), **129.3** (Ar*C*(2`,6`)), **129.4** (Ar*C*H), **132.8** (q, $^3J_{C\text{-}F}$ 5.5, *C*(2``)H), **141.9** (Ar*C*(1```)), **144.1** (Ar*C*(7a)), **176.4** (*C*=O); **^{19}F NMR (376 MHz, $CDCl_3$)** δ **–66.07**; **HRMS (ESI$^+$)** $C_{37}H_{28}F_3NO_2Na$ [M+Na]$^+$ found 598.1964, requires 598.1964 (0 ppm).

(*E*)-3-{[3-(4-Fluorophenyl)but-2-en-1-yl]oxy}-1-tritylindolin-2-one (11)

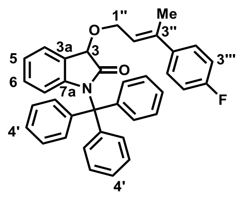

Following **General Procedure F**, 3-diazo-1-tritylindolin-2-one (0.882 g, 2.2 mmol), (*E*)-3-(4-fluorophenyl)but-2-en-1-ol (0.332 g, 2.0 mmol), $Rh_2(OAc)_4$ (0.0044 g, 0.01 mmol) in CH_2Cl_2 (40 mL) gave a crude allylic ether product which was purified by flash column chromatography (eluent: hexane/CH_2Cl_2/EtOAc = 15:5:2) to afford the title compound (0.65 g, 60%) as a colourless amorphous solid. **IR** ν_{max} (film) 1724 (C=O), 1598 (C=C), 1508, 1490, 1477, 1463, 1448, 1336, 1303, 1267, 1228, 1186, 1159, 1103 (C-O), 1085 (C-F); **^{1}H NMR** (500 MHz, $CDCl_3$) δ 1.90 (3H, s, C*H*$_3$), 4.18 (1H, dd, *J* 11.8, 6.8, OC(1``)$\mathit{H}^{A}\mathrm{H}^{B}$), 4.32 (1H, dd, *J* 11.7, 6.5, OC(1``)$\mathrm{H}^{A}\mathit{H}^{B}$), 4.93 (1H, s, C(3)*H*), 5.82 (1H, app t, *J* 6.5, C(2``)*H*), 6.18 (1H, d, *J* 7.5, ArC(7)*H*), 6.78–7.86 (2H, m, ArC(5,6)*H*), 6.86–6.92 (2H, m, ArC(2```,6```)*H*), 7.08–7.13 (3H, m, ArC(4`)*H*), 7.13–7.18 (6H, m, ArC(3`,5`)*H*), 7.22–7.28 (3H, m, ArC(3```,5```,4)*H*), 7.35–7.43 (6H, m, ArC(2`,6`)*H*); **^{13}C{^{1}H} NMR** (126 MHz, $CDCl_3$) δ 16.5 (*C*H$_3$), 65.8 (O*C*(1``)H$_2$), 74.5 (N*C*Ph$_3$), 75.3 (O*C*(3)HCO), 115.1 (d, $^2J_{C\text{-}F}$ 21.4, Ar*C*(3```,5```)H), 116.0 (Ar*C*(7)H), 122.4 (Ar*C*(5)H), 123.6 (*C*(2``)*H*), 124.7 (Ar*C*(4)H), 126.0 (Ar*C*(3a)), 127.0 (Ar*C*(4`)H), 127.4 (d, $^3J_{C\text{-}F}$ 7.9, Ar*C*(2```,6```)), 127.8 (Ar*C*(3`,5`)H), 128.4 (Ar*C*(6)H), 129.3 (Ar*C*(2`,6`)H), 138.6 (*C*(3``)), 138.9 (d, $^4J_{C\text{-}F}$ 3.2, Ar*C*(1```)), 142.1 (Ar*C*(1`)H), 144.1 (Ar*C*(7a)), 161.3 (d, $^1J_{C\text{-}F}$ 246.7, Ar*C*(4```)), 176.9 (*C*=O); **^{19}F{^{1}H} NMR** (470 MHz, $CDCl_3$) δ **–115.43**. **HRMS (ESI$^+$)** $C_{37}H_{30}FNO_2Na$ $[M+Na]^+$ found 562.2145, requires 562.2153 (–1.38 ppm);

(*E*)-3-{[3-(4-Chlorophenyl)but-2-en-1-yl]oxy}-1-tritylindolin-2-one (S84)

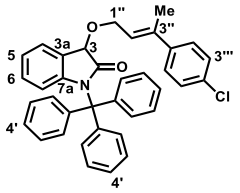

Following **General Procedure F**, 3-diazo-1-tritylindolin-2-one (0.882 g, 2.2 mmol), (*E*)-3-(4-chlorophenyl)but-2-en-1-ol (0.364 g, 2.0 mmol), $Rh_2(OAc)_4$ (0.0044 g, 0.01 mmol) in CH_2Cl_2 (40 mL) gave a crude allylic ether product which was purified by flash column chromatography (eluent: hexane/CH_2Cl_2/EtOAc = 15:5:2) to afford the title compound (0.63 g, 57%) as a colourless amorphous solid. **IR** ν_{max} (film) 1722 (C=O), 1608 (C=C), 1595 (C=C), 1487, 1463, 1450, 1402, 1346, 1307, 1276, 1238, 1180, 1095 (C-O), 1033 (C-Cl); **^{1}H NMR (500 MHz, $CDCl_3$)** δ **1.90** (3H, s, CH_3), **4.18** (1H, dd, *J* 11.8, 6.8, OC(1``)$H^AH^B$), **4.33** (1H, dd, *J* 11.7, 6.5, OC(1``)H^AH^B), **4.94** (1H, s, C(3)*H*), **5.81–5.91** (1H, m, C(2``)*H*), **6.14–6.22** (1H, m, ArC(7)*H*), **6.80–7.90** (2H, m, ArC(5,6)*H*), **7.09–7.14** (3H, m, ArC(4`)*H*), **7.15–7.21** (8H, m, ArC(3`,5`,2```,6```)*H*), **7.21–7.28** (3H, m, ArC(3```,5```,4)*H*), **7.36–7.42** (6H, m, ArC(2`,6`)*H*); **^{13}C{^{1}H} NMR (101 MHz, $CDCl_3$)** δ **16.3** (*C*H_3), **65.8** (O*C*(1``)$H_2$), **74.6** (N*C*$Ph_3$), **75.4** (O*C*(3)HCO), **116.1** (Ar*C*(7)H), **122.5** (Ar*C*(5)H), **124.3** (*C*(2``)*H*), **124.7** (Ar*C*(4)H), **125.9** (Ar*C*(3a)), **127.0** (Ar*C*(4`)H), **127.2** (Ar*C*(2```,6```)H), **127.8** (Ar*C*(3`,5`)H), **128.4** (Ar*C*(3```,5```,6)H), **129.3** (Ar*C*(2`,6`)H), **133.2** (ArC(4```)Cl), **138.4** (*C*(3``)), **141.3** (Ar*C*(1```)), **142.1** (ArC(1`)), **144.1** (Ar*C*(7a)), **176.9** (*C*=O); **HRMS (ESI$^+$)** $C_{37}H_{30}{}^{35}ClNO_2Na$ $[M+Na]^+$ found 578.1851, requires 578.1857 (–1.1 ppm).

(*E*)-3-{[3-(4-Bromophenyl)but-2-en-1-yl]oxy}-1-tritylindolin-2-one (S85)

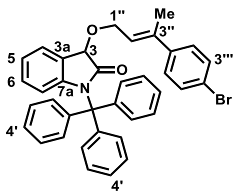

Following **General Procedure F**, 3-diazo-1-tritylindolin-2-one (0.882 g, 2.2 mmol), (*E*)-3-(4-bromophenyl)but-2-en-1-ol (0.452 g, 2.0 mmol), $Rh_2(OAc)_4$ (0.0044 g, 0.01 mmol) in CH_2Cl_2 (40 mL) gave a crude allylic ether product which was purified by flash column chromatography (eluent: hexane/CH_2Cl_2/EtOAc = 15:5:2) to afford the title compound (0.84 g, 70%) as a colourless amorphous solid. **IR** ν_{max} (film) 1721 (C=O), 1608 (C=C), 1595 (C=C), 1477, 1463, 1448, 1400, 1307, 1276, 1182, 1166, 1153 (C-O), 1116, 1053 (C-Br); **^{1}H NMR (500 MHz, $CDCl_3$)** δ **2.01** (3H, s, C*H*$_3$), **4.29** (1H, dd, *J* 11.88, 6.8, OC(1``)$H^A$H$^B$), **4.43** (1H, dd, *J* 11.8, 6.5, OC(1``)H^AH^B), **5.05** (1H, s, C(3)*H*), **5.98** (1H, t, *J* 6.2, C(2``)*H*), **6.30** (1H, d, *J* 7.4, ArC(7)*H*), **6.92–7.02** (2H, m, ArC(5,6)*H*), **7.21–7.45** (3H, m, ArC(4`)*H*), **7.26–7.32** (8H, m, ArC(3`,5`,2```,6```)*H*), **7.37** (1H, d, *J* 6.7, ArC(4)*H*), **7.45** (2H, app d, *J* 8.5, ArC(3```,5```)*H*), **7.50** (6H, app d, *J* 7.6, ArC(2`,6`)*H*); **^{13}C{^{1}H} NMR (126 MHz, $CDCl_3$)** δ **16.3** (*C*H$_3$), **65.8** (O*C*(1``)H$_2$), **74.6** (N*C*Ph$_3$), **75.4** (O*C*(3)HCO), **116.1** (Ar*C*(7)H), **121.3** (Ar*C*(4```)Br), **122.5** (Ar*C*(5)H), **124.4** (*C*(2``)*H*), **124.7** (Ar*C*(4)H), **125.9** (Ar*C*(3a)), **127.0** (Ar*C*(4`)H), **127.6** (Ar*C*(2```,6```)H), **127.8** (ArC(3`,5`)H), **128.4** (Ar*C*(6)H), **129.3** (Ar*C*(2`,6`)H), **131.4** (Ar*C*(3```,5```)), **138.4** (*C*(3``)), **141.8** (Ar*C*(1```)), **142.1** (Ar*C*(1`)), **144.1** (Ar*C*(7a)), **176.9** (*C*=O); **HRMS (ESI$^+$)** $C_{37}H_{30}{}^{79}BrNO_2Na$ [M+Na]$^+$ found 622.1339, requires 622.1352 (–2.1 ppm).

(*E*)-3-{[3-(4-(Trifluoromethyl)phenyl)but-2-en-1-yl]oxy}-1-tritylindolin-2-one (S86)

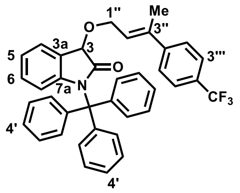

Following **General Procedure F**, 3-diazo-1-tritylindolin-2-one (0.882 g, 2.2 mmol), (*E*)-3-(4-(trifluoromethyl)phenyl)but-2-en-1-ol (0.432 g, 2.0 mmol), $Rh_2(OAc)_4$ (0.0044 g, 0.01 mmol) in CH_2Cl_2 (40 mL) gave a crude allylic ether product which was purified by flash column chromatography (eluent: hexane/CH_2Cl_2/EtOAc = 15:5:2) to afford the title compound (0.74 g, 63%) as a colourless amorphous solid. **IR** ν_{max} (film) 1724 (C=O), 1604 (C=C), 1489, 1463, 1446, 1384, 1323 (CF_3), 1305, 1261, 1165, 1151 (C-O), 1112, 1101, 1074; **^{1}H NMR (400 MHz, CDCl$_3$)** δ **1.92** (3H, s, C*H*$_3$), **4.18** (1H, ddd, *J* 12.0, 6.7, 0.9, OC(1``)$H^AH^B$), **4.35** (1H, ddd, *J* 12.0, 6.4, 1.0, OC(1``)H^AH^B), **4.95** (1H, d, *J* 1.0, C(3)*H*), **5.93** (1H, tq, *J* 6.5, 1.3, C(2``)*H*), **6.16–6.22** (1H, m, ArC(7)*H*), **6.81–6.90** (2H, m, ArC(5,6)*H*), **7.09–7.14** (3H, m, ArC(4`)*H*), **7.14–7.20** (6H, m, ArC(3`,5`)*H*), **7.24–7.28** (1H, m, ArC(4)*H*), **7.46–6.57** (8H, m, ArC(2`,6`,2```,6```)*H*), **7.44–7.50** (2H, m, ArC(3```,5```)*H*); **^{13}C{^{1}H} NMR (126 MHz, CDCl$_3$)** δ **16.3** (*C*H$_3$), **65.8** (O*C*(1``)H$_2$CH), **74.6** (N*C*Ph$_3$), **75.5** (O*C*(3)HCO), **116.1** (Ar*C*(7)H), **122.5** (Ar*C*(5)H), **124.4** (q, $^1J_{C-F}$ 272.9, ArC(4```)*C*F$_3$), **124.7** (Ar*C*(4)H), **125.3** (q, $^3J_{C-F}$ 3.6, ArC(3```,5```)H), **125.8** (Ar*C*(3a)), **125.9** (*C*(2``)H), **126.2** (ArC(2```,6```)H), **127.1** (Ar*C*(4`)H), **127.8** (Ar*C*(3`,5`)H), **128.5** (Ar*C*(6)H), **129.3** (Ar*C*(2`,6`)H), **138.1** (*C*(3``)), **142.0** (Ar*C*(1`)), **144.1** (Ar*C*(7a)), **146.4** (Ar*C*(1```)), **176.8** (*C*=O); **^{19}F NMR (376 MHz, CDCl$_3$)** δ **–62.38**; **HRMS (ESI$^+$)** $C_{38}H_{30}F_3NO_2Na$ $[M+Na]^+$ found 612.2110, requires 612.2121 (–1.8 ppm).

(*E*)-3-{[3-((1,1'-Biphenyl)-4-yl)but-2-en-1-yl]oxy}-1-tritylindolin-2-one (S87)

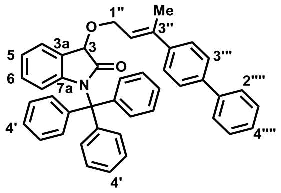

Following **General Procedure F**, 3-diazo-1-tritylindolin-2-one (0.882 g, 2.2 mmol), (*E*)-3-([1,1'-biphenyl]-4-yl)but-2-en-1-ol (0.448 g, 2.0 mmol), $Rh_2(OAc)_4$ (0.0044 g, 0.01 mmol) in CH_2Cl_2 (40 mL) gave a crude allylic ether product which was purified by flash column chromatography (eluent: hexane/CH_2Cl_2/EtOAc = 15:5:2) to afford the title compound (0.84 g, 70%) as a colourless amorphous solid. **IR** ν_{max} (film) 1726 (C=O), 1608 (C=C), 1597 (C=C), 1487, 1463, 1448, 1338, 1303, 1267, 1240, 1182, 1155 (C-O), 1103, 1083; **^{1}H NMR (500 MHz, $CDCl_3$)** δ **2.07** (3H, s, C*H*$_3$), **4.35** (1H, dd, *J* 11.8, 6.9, OC(1``)*H*$^A$H$^B$), **4.46** (1H, dd, *J* 11.7, 6.5, OC(1``)H^A*H*^{B}), **5.06** (1H, s, C(3)*H*OCH$_2$), **6.06** (1H, app td, *J* 6.7, 1.4, C(2``)*H*), **6.25–6.33** (1H, m, ArC(7)*H*), **6.90–7.01**(2H, m, ArC(5,6)*H*), **7.20–7.24** (3H, m, ArC(4`)*H*), **7.25–7.30** (6H, m, ArC(3`,5`)*H*), **7.33–7.39** (2H, m, ArC(4,4````)*H*), **7.43–7.47** (2H, m, ArC(3````,5````)*H*), **7.48–7.54** (8H, m, ArC(2`,6`,3```,5```)*H*), **7.55–7.59** (2H, m, ArC(2```,6```)*H*), **7.60–7.64** (2H, m, ArC(2````,6````)*H*); **^{13}C{^{1}H} NMR (126 MHz, $CDCl_3$)** δ **16.3** (*C*H$_3$), **66.0** (O*C*(1``)H$_2$CH), **74.6** (N*C*Ph$_3$), **75.3** (O*C*(3)HCO), **116.1** (Ar*C*(7)H), **122.5** (Ar*C*(5)H), **123.7** (*C*(2``)*H*), **124.7** (Ar*C*(4)H), **126.1** (Ar*C*(3a)), **126.3** (Ar*C*(3```,5```)H), **127.0** (Ar*C*(4`,2```,6```)H), **127.1** (Ar*C*(2````,6````)H), **127.4** (Ar*C*(4````)H), **127.8** (Ar*C*(3`,5`)H), **128.4** (Ar*C*(6)H), **128.9** (Ar*C*(3````,5````)H), **129.3** (Ar*C*(2`,6`)H), **139.1** (*C*(3``)), **140.2** (*C*(4```)), **140.8** (*C*(1````)), **141.8** (Ar*C*(1```)), **142.1** (Ar*C*(1`)), **144.1** (Ar*C*(7a)), **176.9** (*C*=O); **HRMS (ESI$^+$)** $C_{43}H_{35}NO_2Na$ $[M+Na]^+$ found 620.2560, requires 620.2560 (0.0 ppm).

(*E*)-3-{[3-(p-Tolyl)but-2-en-1-yl]oxy}-1-tritylindolin-2-one (S88)

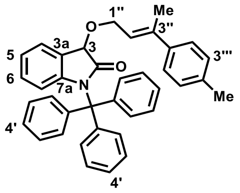

Following **General Procedure F**, 3-diazo-1-tritylindolin-2-one (0.882 g, 2.2 mmol), (*E*)-3-(p-tolyl)but-2-en-1-ol (0.324 g, 2.0 mmol), $Rh_2(OAc)_4$ (0.0044 g, 0.01 mmol) in CH_2Cl_2 (40 mL) gave a crude allylic ether product which was purified by flash column chromatography (eluent: hexane/CH_2Cl_2/EtOAc = 15:5:2) to afford the title compound (0.71 g, 66%) as a colourless amorphous solid. **IR** ν_{max} (film) 1726 (C=O), 1608 (C=C), 1597 (C=C), 1512, 1490, 1477, 1463, 1448, 1338, 1303, 1265, 1186, 1155 (C-O), 1105, 1085; **^{1}H NMR (500 MHz, $CDCl_3$)** δ **1.93** (3H, s, =C(3``)C*H*$_3$), **2.26** (3H, s, ArC(4```)C*H*$_3$), **4.22** (1H, dd, *J* 11.8, 7.0, OC(1``)*H*$^A$H$^B$), **4.33** (1H, dd, *J* 11.7, 6.5, OC(1``)H^A*H*^{B}), **4.94** (1H, s, C(3)*H*OCH$_2$), **5.81–5.91** (1H, m, C(2``)*H*), **6.17** (1H, dd, *J* 7.6, 1.5, ArC(7)*H*), **6.80–6.89** (2H, m, ArC(5,6)*H*), **7.04** (2H, d, *J* 8.0, C(3```,5```)*H*), **7.10–7.14** (3H, m, ArC(4`)*H*), **7.15–7.19** (6H, m, ArC(3`,5`)*H*), **7.20–7.24** (2H, m, ArC(2```,6```)*H*), **7.24–7.28** (1H, m, ArC(4)*H*), **7.37–7.45** (6H, m, ArC(2`,6`)*H*); **^{13}C{^{1}H} NMR (126 MHz, $CDCl_3$)** δ **16.4** (=C(3``)*C*H$_3$), **21.2** (ArC(4```)*C*H$_3$), **66.0** (O*C*(1``)H$_2$), **74.5** (N*C*Ph$_3$), **75.2** (O*C*(3)HCO), **116.0** (Ar*C*(7)H), **122.4** (Ar*C*(5)H), **122.8** (*C*(2``)H), **124.7** (Ar*C*(4)H), **125.8** (Ar*C*(3```,5```)H), **126.1** (Ar*C*(3a)), **127.0** (Ar*C*(4`)H), **127.8** (Ar*C*(3`,5`)H), **128.3** (Ar*C*(6)H), **129.0** (Ar*C*(2```,6```)H), **129.3** (Ar*C*(2`,6`)H), **137.2** (Ar*C*(4```)CH$_3$), **140.0** (*C*(3``)), **142.1** (Ar*C*(1`)), **143.1** (Ar*C*(1```)), **144.1** (Ar*C*(7a)), **177.0** (*C*=O); **HRMS (ESI$^+$)** $C_{38}H_{33}NO_2Na$ $[M+Na]^+$ found 558.2391, requires 558.2404 (–2.3 ppm).

(*E*)-3-{[3-(4-Methoxyphenyl)but-2-en-1-yl]oxy}-1-tritylindolin-2-one (16)

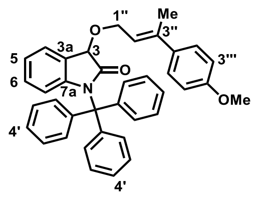

Following **General Procedure F**, 3-diazo-1-tritylindolin-2-one (0.882 g, 2.2 mmol), (*E*)-3-(4-methoxyphenyl)but-2-en-1-ol (0.356 g, 2.0 mmol), $Rh_2(OAc)_4$ (0.0044 g, 0.01 mmol) in CH_2Cl_2 (40 mL) gave a crude allylic ether product which was purified by flash column chromatography (eluent: hexane/CH_2Cl_2/EtOAc = 15:5:2) to afford the title compound (0.53 g, 48%) as a colourless amorphous solid. **IR** ν_{max} (film) 1726 (C=O), 1606 (C=C), 1510, 1490, 1477, 1463, 1448, 1336, 1303, 1265, 1246 (C-O), 1180, 1155, 1105 (C-O), 1083; **^{1}H NMR** (500 MHz, $CDCl_3$) δ 1.92 (3H, s, C(3``)C$H_3$), 3.72 (3H, s, OC$H_3$), 4.21 (1H, dd, *J* 11.6, 7.1, OC(1``)H^AH^B), 4.32 (1H, dd, *J* 11.6, 6.6, OC(1``)$H^AH^B$), 4.94 (1H, s, C(3)*H*OCH$_2$), 5.83 (1H, app t, *J* 6.3, C(2``)*H*), 6.13–7.21 (1H, m, ArC(7)*H*), 6.73–6.79 (2H, m, ArC(3```,5```)*H*), 6.80–6.90 (2H, m, ArC(5,6)*H*), 7.09–7.20 (9H, m, ArC(3`,4`,5`)*H*), 7.23–7.29 (3H, m, ArC(2```,6```,4)*H*), 7.39 (6H, d, *J* 7.4, ArC(2`,6`)*H*); **^{13}C{^{1}H} NMR** (126 MHz, $CDCl_3$) δ 16.4 (C(3``)*C*H$_3$), 55.4 (O*C*H$_3$), 66.0 (O*C*(1``)H$_2$CH), 74.5 (*NC*Ph$_3$), 75.2 (O*C*(3)HCO), 113.7 (Ar*C*(6)H), 116.0 (Ar*C*(7)H), 121.9 (*C*(2``)H), 122.4 (Ar*C*(5)H), 124.6 (Ar*C*(4)H), 126.1 (Ar*C*(3a)), 127.0 (Ar*C*(4`)H), 127.8 (Ar*C*(3`,5`)H), 127.9 (Ar*C*(3```,5```)H), 128.3 (Ar*C*(2```,6```)H), 129.3 (Ar*C*(2`,6`)H), 135.3 (*C*(3``)), 139.2 (Ar*C*(1```)), 142.1 (Ar*C*(1`)), 144.1 (Ar*C*(7a)), 159.1 (Ar*C*(4```)OMe), 177.0 (*C*=O); **HRMS (ESI$^+$)** $C_{38}H_{33}NO_3Na$ $[M+Na]^+$ found 574.2353, requires 574.2353 (0 ppm).

(*E*)-3-{[3-(*m*-Tolyl)but-2-en-1-yl]oxy}-1-tritylindolin-2-one (S89)

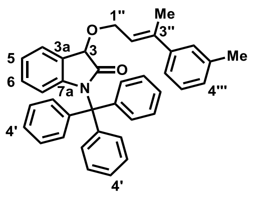

Following **General Procedure F**, 3-diazo-1-tritylindolin-2-one (0.882 g, 2.2 mmol), (*E*)-3-(m-tolyl)but-2-en-1-ol (0.356 g, 2.0 mmol), $Rh_2(OAc)_4$ (0.0044 g, 0.01 mmol) in CH_2Cl_2 (40 mL) gave a crude allylic ether product which was purified by flash column chromatography (eluent: hexane/CH_2Cl_2/EtOAc = 15:5:2) to afford the title compound (0.73 g, 68%) as a colourless amorphous solid. **IR** ν_{max} (film) 1726 (C=O), 1608 (C=C), 1595 (C=C), 1489, 1463, 1450, 1348, 1307, 1274, 1242, 1190, 1178, 1161, 1097 (C-O); **^{1}H NMR (400 MHz, $CDCl_3$)** δ **1.93** (3H, s, C(3``)CH_3), **2.27** (3H, s, ArC(3```)CH_3), **4.22** (H, dd, *J* 11.8, 6.9, OC(1``)$H^A$H$^B$), **4.33** (H, dd, *J* 11.6, 6.6, OC(1``)H^AH^B), **4.95** (1H, s, C(3)*H*OCH_2), **5.87** (1H, app td, *J* 6.7, 1.4, C(2``)*H*), **6.18** (1H, dd, *J* 7.2, 1.8, ArC(7)*H*), **6.80–6.90** (2H, m, ArC(5,6)*H*), **6.96–7.01** (1H, m, ArC(2```)*H*), **7.09–7.15** (6H, m, ArC(4`,4```,5```,6```)*H*), **7.15–7.20** (6H, m, ArC(3`,5`)*H*), **7.24–7.28** (1H, m, ArC(4)*H*), **7.37–7.42** (6H, m, ArC(2`,6`)*H*); **^{13}C{^{1}H} NMR (101 MHz, $CDCl_3$)** δ **16.5** (C(3``)*C*H_3), **21.6** (ArC(3```)*C*H_3), **66.0** (O*C*(1``)H_2CH), **74.5** (*NC*Ph_3), **75.2** (O*C*(3)HCO), **116.0** (Ar*C*(7)H), **122.4** (Ar*C*(5)H), **123.1** (Ar*C*(6```)H), **123.5** (*C*(2``)H), **124.7** (Ar*C*(4)H), **126.1** (Ar*C*(3a)), **126.8** (Ar*C*(4```)H)), **127.0** (Ar*C*(4`)H), **127.8** (Ar*C*(3`,5`)H), **128.1** (Ar*C*(2```)H), **128.2** (Ar*C*(6)H), **128.4** (Ar*C*(5```)H), **129.3** (Ar*C*(2`,6`)), **137.8** (Ar*C*(3```)), **139.8** (*C*(3``)), **142.1** (Ar*C*(1`)), **143.0** (Ar*C*(1```)), **144.1** (Ar*C*(7a)), **177.0** (*C*=O); **HRMS (ESI$^+$)** $C_{38}H_{33}NO_2Na$ $[M+Na]^+$ found 558.2386, requires 558.2404 (–3.1 ppm).

(*E*)-3-{[3-(o-Tolyl)but-2-en-1-yl]oxy}-1-tritylindolin-2-one (S90)

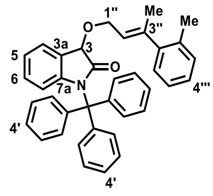

Following **General Procedure F**, 3-diazo-1-tritylindolin-2-one (0.882 g, 2.2 mmol), (*E*)-3-(o-tolyl)but-2-en-1-ol (0.356 g, 2.0 mmol), $Rh_2(OAc)_4$ (0.0044 g, 0.01 mmol) in CH_2Cl_2 (40 mL) gave a crude allylic ether product which was purified by flash column chromatography (eluent: hexane/CH_2Cl_2/EtOAc = 15:5:2) to afford the title compound (0.55 g, 51%) as a colourless amorphous solid. **IR** ν_{max} (film) 1726 (C=O), 1689, 1608 (C=C), 1595 (C=C), 1489, 1479, 1463, 1448, 1377, 1336, 1303, 1265, 1242, 1184, 1155 (C-O), 1112, 1103, 1085; **^{1}H NMR (400 MHz, $CDCl_3$)** δ **1.83** (3H, d, *J* 0.5, C(3``)C*H*$_3$), **2.18**, (3H, s, ArC(2```)C*H*$_3$), **4.18–4.32** (2H, m, OC(1``)*H*$_2$), **4.95** (1H, s, C(3)*H*OCH$_2$), **5.44** (1H, td, *J* 7.0, 1.4, C(2``)*H*), **6.14–6.22** (1H, m, ArC(7)*H*), **6.80–6.89** (2H, m, ArC(5,6)*H*), **6.96–7.08** (4H, m, ArC(3```,4```,5```,6```)*H*), **7.09–7.14** (3H, m, ArC(4`)*H*), **7.14–7.21** (6H, m, ArC(3`,5`)*H*), **7.22–7.27** (1H, m, ArC(4)*H*), **7.36–7.43** (6H, m, ArC(2`,6`)*H*); **^{13}C{^{1}H} NMR (101 MHz, $CDCl_3$)** δ **18.7** (C(3``)*C*H$_3$), **20.0** (Ar(C2```)*C*H$_3$), **65.6** (O*C*(1`)H$_2$CH), **74.5** (*NC*Ph$_3$), **75.1** (O*C*(3)HCO), **116.0** (Ar*C*(7)H), **122.4** (Ar*C*(5)H), **124.7** (Ar*C*(4)H), **125.3** (*C*(2``)*H*), **125.6** (Ar*C*(6```)H), **126.1** (Ar*C*(3a)), **126.9** (Ar*C*(5```)H), **127.0** (Ar*C*(4`)H), **127.8** (Ar*C*(3`,5`)H), **128.2** (Ar*C*(4```)H), **128.3** (Ar*C*(6)H), **129.3** (Ar*C*(2`,6`)H), **130.2** (Ar*C*(3```)H), **134.6** (Ar*C*(2```)CH$_3$), **141.4** (*C*(3``)), **142.1** (Ar*C*(1`)), **144.1** (Ar*C*(7a)), **144.7** (Ar*C*(1```)), **177.0** (*C*=O); **HRMS (ESI$^+$)** $C_{38}H_{33}NO_2Na$ $[M+Na]^+$ found 558.2395, requires 558.2404 (–1.5 ppm).

(*E*)-3-{[3-(Naphtha-2-yl)but-2-en-1-yl]oxy}-1-tritylindolin-2-one (S91)

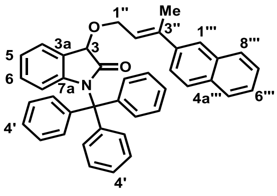

Following **General Procedure F**, 3-diazo-1-tritylindolin-2-one (0.882 g, 2.2 mmol), (*E*)-3-(naphtha-2-yl)but-2-en-1-ol (0.396 g, 2.0 mmol), $Rh_2(OAc)_4$ (0.0044 g, 0.01 mmol) in CH_2Cl_2 (40 mL) gave a crude allylic ether product which was purified by flash column chromatography (eluent: hexane/CH_2Cl_2/EtOAc = 15:5:2) to afford the title compound (0.80 g, 70%) as a colourless amorphous solid. **IR** ν_{max} (film) 1732 (C=O), 1606 (C=C), 1595 (C=C), 1490, 1475, 1463, 1448, 1381, 1334, 1301, 1267, 1232, 1184, 1153, 1126 (C-O), 1116; **^{1}H NMR (400 MHz, $CDCl_3$)** δ **2.14**, (3H, s, CH_3), **4.37** (1H, dd, *J* 11.7, 6.9, OCH^AH^B), **4.50** (1H, dd, *J* 11.7, 6.4, OCH^AH^B), **5.07** (1H, s, C(3)*H*OCH$_2$), **6.15** (1H, app t, *J* 6.3, C(2``)*H*), **6.29** (1H, d, *J* 7.8, ArC(7)*H*), **6.90–7.01**(2H, m, ArC(5,6)*H*), **7.22** (3H, app t, *J* 7.2, ArC(4`)*H*), **7.28** (6H, app t, *J* 7.5, ArC(3`,5`)*H*), **7.38** (1H, d, *J* 6.8, ArC(4)*H*), **7.42–7.48** (2H, m, ArC*H*), **7.50** (6H, d, *J* 7.4, ArC(2`,6`)*H*), **7.60** (1H, d, *J* 8.6, ArC(3```)*H*), **7.79** (1H, d, *J* 8.7, ArC(4```)*H*), **7.80–7.87** (3H, m, ArC*H*); **^{13}C{^{1}H} NMR (101 MHz, $CDCl_3$)** δ **16.4** (*C*H$_3$), **66.0** (O*C*(1``)H$_2$CH), **74.6** (*NC*Ph$_3$), **75.4** (O*C*(3)HCO), **116.1** (Ar*C*(7)H), **122.5** (Ar*C*(5)H), **124.3** (Ar*C*(3```)H), **124.4** (*C*(2``)H), **124.7** (Ar*C*H), **124.8** (Ar*C*(4)H), **125.9** (Ar*C*(3a)), **126.1** (Ar*C*H), **126.2** (Ar*C*H), **127.0** (Ar*C*(4`)H), **127.6** (Ar*C*(5```)H), **127.8** (Ar*C*(3`,5`)H, Ar*C*(4```)*H*), **128.3** (Ar*C*(6)H), **128.4** (Ar*C*H), **129.3** (Ar*C*(2`,6`)H), **132.8** (Ar*C*(4a```)), **133.5** (Ar*C*(8a```)), **139.4** (*C*(3``)), **140.0** (Ar*C*(2```)), **142.1** (Ar*C*(1`)H), **144.1** (Ar*C*(7a)), **176.9** (*C*=O); **HRMS (ESI$^+$)** $C_{41}H_{33}NO_2Na$ [M+Na]$^+$ found 594.2389, requires 594.2404 (–2.4 ppm).

(*E*)-3-{[3-(Thiophen-2-yl)but-2-en-1-yl]oxy}-1-tritylindolin-2-one (S92)

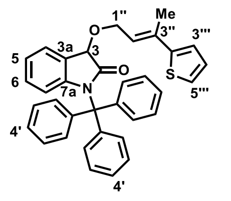

Following **General Procedure F**, 3-diazo-1-tritylindolin-2-one (0.882 g, 2.2 mmol), (*E*)-3-(thiophen-2-yl)but-2-en-1-ol (0.308 g, 2.0 mmol), $Rh_2(OAc)_4$ (0.0044 g, 0.01 mmol) in CH_2Cl_2 (40 mL) gave a crude allylic ether product which was purified by flash column chromatography (eluent: hexane/CH_2Cl_2/EtOAc = 15:5:2) to afford the title compound (0.54 g, 51%) as a colourless amorphous solid. **IR** ν_{max} (film) 1726 (C=O), 1608 (C=C), 1595 (C=C), 1490, 1463, 1450, 1440, 1346, 1309, 1273, 1267, 1240, 1199, 1182, 1161, 1101, 1051; **^{1}H NMR (400 MHz, $CDCl_3$)** δ **1.96** (3H, m, C*H*$_3$), **4.20** (1H, dd, *J* 11.8, 7.2, OC(1``)*H*$^A$H$^B$), **4.32** (1H, d, *J* 11.8, 6.7, OC(1``)H^A*H*^{B}), **4.93** (1H, s, C(3)*H*OCH$_2$), **5.97–6.06** (1H, m, C(2``)*H*), **6.15–6.20** (1H, m, ArC(7)*H*), **6.80–6.89** (3H, m, ArC(5,6,5```)*H*), **6.95** (1H, dd, *J* 3.6, 0.9, C(3```)*H*), **7.06** (1H, dd, *J* 5.1, 0.9, C(4```)*H*), **7.09–7.15** (3H, m, Ar*C*(4`)*H*), **7.15–7.21** (m, 6H, Ar*C*(3`,5`)*H*), **7.24–7.27** (m, 1H, Ar*C*(4)*H*), **7.35–7.42** (m, 6H, Ar*C*(2`,6`)H); **^{13}C{^{1}H} NMR (101 MHz, $CDCl_3$)** δ **16.3** (*C*H$_3$), **65.5** (O*C*(1``)H$_2$CH), **74.5** (*N*CPh$_3$), **75.1** (O*C*(3)HCO), **116.0** (Ar*C*(7)H), **121.9** (*C*(2``)H), **122.5** (Ar*C*(5)H), **123.5** (Ar*C*(3```)H), **124.3** (Ar*C*(4```)H), **124.7** (Ar*C*(4)H), **126.0** (Ar*C*(3a)), **127.0** (Ar*C*(4`)H), **127.4** (Ar*C*(5```)H), **127.8** (Ar*C*(3`,5`)H), **128.4** (Ar*C*(6)H), **129.3** (Ar*C*(2`,6`)H), **133.7** (*C*(3``)), **142.1** (Ar*C*(1`)), **144.0** (Ar*C*(7a)), **146.7** (Ar*C*(2```)), **176.8** (*C*=O); **HRMS (ESI$^+$)** $C_{35}H_{29}NO_2SNa$ $[M+Na]^+$ found 550.1812, requires 550.1811 (0.14 ppm).

(*E*)-3-{[3-(Thiazol-2-yl)but-2-en-1-yl]oxy}-1-tritylindolin-2-one (S93)

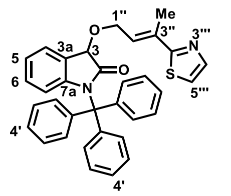

Following **General Procedure F**, 3-diazo-1-tritylindolin-2-one (0.882 g, 2.2 mmol), (*E*)-3-(thiazol-2-yl)but-2-en-1-ol (0.31 g, 2.0 mmol), $Rh_2(OAc)_4$ (0.0044 g, 0.01 mmol) in CH_2Cl_2 (40 mL) gave a crude allylic ether product which was purified by flash column chromatography (eluent: hexane/CH_2Cl_2/EtOAc = 15:5:2) to afford the title compound (0.58 g, 55%) as a colourless amorphous solid. **IR** ν_{max} (film) 1722 (C=O), 1606 (C=C), 1593, 1487, 1463, 1448, 1330, 1305, 1265, 1190, 1170, 1153, 1118 (C-O), 1089, 1066; **^{1}H NMR (500 MHz, $CDCl_3$)** δ **2.12** (3H, m, CH_3), **4.33** (1H, dd, *J* 12.5, 6.7, OC(1``)$H^A$H$^B$), **4.46** (1H, d, *J* 12.5, 6.3, OC(1``)H^AH^B), **5.03** (1H, s, C(3)*H*OCH$_2$), **6.27** (1H, d, *J* 7.2, ArC(7)*H*), **6.54** (1H, app t, *J* 6.0, C(2`)*H*), **6.88–6.99** (2H, m, ArC(5,6)*H*), **7.18–7.23** (4H, m, ArC(4`)*H*, ArC(5```)*H*), **7.24–7.30** (6H, m, ArC(3`,5`)*H*), **7.35** (1H, d, *J* 6.6, ArC(4)*H*), **7.48** (6H, d, *J* 7.6, ArC(2`,6`)*H*), **7.75** (1H, d, *J* 3.3, C(4```)*H*); **^{13}C{^{1}H} NMR (126 MHz, $CDCl_3$)** δ **16.4** (*C*H$_3$), **65.4** (O*C*(1``)H$_2$CH), **74.6** (*N*CPh$_3$), **75.4** (O*C*(3)HCO), **116.1** (Ar*C*(7)H), **118.5** (Ar*C*(5```)H), **124.8** (Ar*C*(4)H), **122.5** (Ar*C*(5)H), **125.7** (Ar*C*(3a)), **127.0** (Ar*C*(4`)H), **127.8** (Ar*C*(3`,5`)H), **128.0** (*C*(2``)H), **128.5** (Ar*C*(6)H), **129.3** (Ar*C*(2`,6`)H), **133.6** (*C*(3``)), **142.0** (Ar*C*(1`)), **143.1** (Ar*C*(4```)), **144.1** (Ar*C*(7a)), **170.7** (Ar*C*(2```)), **176.8** (*C*=O); **HRMS (ESI$^+$)** $C_{34}H_{28}N_2O_2SNa$ [M+Na]$^+$ found 551.1745, requires 551.1764 (–3.4 ppm).

(*E*)-4-Chloro-3-[(3-phenylbut-2-en-1-yl)oxy]-1-tritylindolin-2-one (S94)

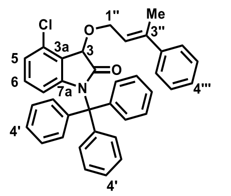

Following **General Procedure F**, 4-chloro-3-diazo-1-tritylindolin-2-one (0.957 g, 2.2 mmol), (*E*)-3-phenylbut-2-en-1-ol (0.296 g, 2.0 mmol), $Rh_2(OAc)_4$ (0.0044 g, 0.01 mmol) in CH_2Cl_2 (40 mL) gave a crude allylic ether product which was purified by flash column chromatography (eluent: hexane/CH_2Cl_2/EtOAc = 15:5:2) to afford the title compound (0.80 g, 72%) as a colourless amorphous solid. **IR** ν_{max} (film) 1730 (C=O), 1600 (C=C), 1490, 1446, 1338, 1271, 1253, 1139 (C-O), 1083, 1026 (C-Cl); **^{1}H NMR (500 MHz, $CDCl_3$)** δ **2.08** (3H, s, *CH3*), **4.45** (1H, dd, *J* 11.6, 7.1, OC(1``)$H^AH^B$), **4.57** (1H, dd, *J* 11.6, 6.6, OC(1``)H^AH^B), **4.95** (1H, s, C(3)*H*), **6.07** (1H, app td, *J* 6.8, 1.4, C(2``)*H*), **6.15** (1H, d, *J* 8.0, ArC(7)*H*), **6.86** (1H, t, *J* 8.1, ArC(6)*H*), **6.89–6.94** (1H, m, ArC(5)*H*), **7.19–7.24** (3H, m, ArC(4`)*H*, **7.25–7.29** (7H, m, ArC(3`,5`,4```,)*H*), **7.30–7.34** (2H, m, ArC(3```,5```)*H*), **7.41–7.44** (2H, m, ArC(2```,6```)*H*), **7.44–7.50** (6H, m, ArC(2`,6`)*H*); **^{13}C{^{1}H} NMR (126 MHz, $CDCl_3$)** δ **16.4** (*C*H_3), **66.7** (O*C*(1``)$H_2$CH), **74.6** (O*C*(3)HCO), **74.7** (N*C*$Ph_3$), **114.4** (Ar*C*(7)H), **123.2** (Ar*C*(5)H), **123.5** (*C*(2``)H), **123.7** (Ar*C*(3a)), **126.0** (Ar*C*(2```,6```)H), **127.2** (Ar*C*(4`)H), **127.4** (Ar*C*(4```)H), **127.9** (Ar*C*(3`,5`)H), **128.3** (Ar*C*(3```,5```)H), **129.4** (Ar*C*(2`,6`)H), **129.7** (Ar*C*(6)H), **132.1** (Ar*C*(4)Cl), **140.0** (*C*(3``)), **141.8** (Ar*C*(1`)), **146.0** (Ar*C*(7a)), **142.9** (Ar*C*(1```)), **175.3** (*C*=O); **HRMS (ESI$^+$)** $C_{37}H_{30}{}^{35}ClNO_2Na$ $[M+Na]^+$ found 578.1843, requires 578.1857 (–2.5 ppm).

(*E*)-5-Fluoro-3-[(3-phenylbut-2-en-1-yl)oxy]-1-tritylindolin-2-one (15)

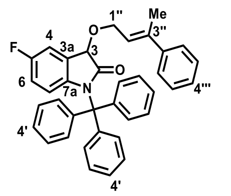

Following **General Procedure F**, 3-diazo-5-fluoro-1-tritylindolin-2-one (0.922 g, 2.2 mmol), (*E*)-3-phenylbut-2-en-1-ol (0.36 g, 2.0 mmol), $Rh_2(OAc)_4$ (0.0044 g, 0.01 mmol) in CH_2Cl_2 (40 mL) gave a crude allylic ether product which was purified by flash column chromatography (eluent: hexane/CH_2Cl_2/EtOAc = 15:5:2) to afford the title compound (0.65 g, 60%) as a colourless amorphous solid. **IR** ν_{max} (film) 1728 (C=O), 1606 (C=C), 1479, 1450, 1292, 1274 (C-F), 1163; **^{1}H NMR** (500 MHz, $CDCl_3$) δ 1.96 (3H, s, C*H*$_3$), 4.26 (1H, dd, *J* 11.8, 7.0, OC(1``)*H*$^A$H$^B$), 4.37 (1H, dd, *J* 11.8, 6.5, OC(1``)H^A*H*^{B}), 4.92 (1H, s, C(3)*H*), 5.88 (1H, app t, *J* 6.7, C(2``)*H*), 6.11 (1H, dd, *J* 8.9, 4.1, ArC(7)*H*), 6.54 (1H, td, *J* 9.0, 2.8, ArC(6)*H*), 6.96–7.01 (1H, m, ArC(4)*H*), 7.10–7.15 (3H, m, ArC(4`)*H*), 7.16–7.21 (7H, m, ArC(3`,5`)*H*, ArC(4``)*H*), 7.24 (2H, t, *J* 7.3, ArC(3``,5``)*H*), 7.32 (2H, d, *J* 7.6, ArC(2``,6``)*H*), 7.37 (6H, d, *J* 7.8, ArC(2`,6`)*H*); **^{13}C{^{1}H} NMR** (126 MHz, $CDCl_3$) δ 16.4 (*C*H$_3$), 66.3 (O*C*(1``)H$_2$CH), 74.7 (N*C*Ph$_3$), 75.1 (O*C*(3)HCO), 112.4 (d, *J* 24.2, Ar*C*(4)H), 114.8 (d, *J* 22.9, Ar*C*(6)H), 116.8 (d, *J* 7.6, Ar*C*(7)H), 123.4 (*C*(2``)H), 126.0 (Ar*C*(2```,6```)H), 127.2 (Ar*C*(4`)H), 127.5 (Ar*C*(4```)H), 127.8 (Ar*C*(3a)), 127.9 (Ar*C*(3`,5`)H), 128.4 (Ar*C*(3```,5```)H), 129.3 (Ar*C*(2`,6`)H), 139.8 (Ar*C*(7a)), 140.1 (*C*(3``)), 141.9 (Ar*C*(1`)H), 142.8 (Ar*C*(1```)), 158.8 (d, *J* 243.4, Ar*C*(5)), 176.8 (*C*=O); **^{19}F{^{1}H} NMR** (470 MHz, $CDCl_3$) δ –120.75; **HRMS (ESI$^+$)** $C_{37}H_{30}FNO_2Na$ [M+Na]$^+$ found 562.2149, requires 562.2153 (–0.71 ppm).

(*E*)-5-Chloro-3-[(3-phenylbut-2-en-1-yl)oxy]-1-tritylindolin-2-one (S95)

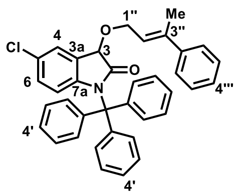

Following **General Procedure F**, 5-chloro-3-diazo-1-tritylindolin-2-one (0.957 g, 2.2 mmol), (*E*)-3-phenylbut-2-en-1-ol (0.296 g, 2.0 mmol), $Rh_2(OAc)_4$ (0.0044 g, 0.01 mmol) in CH_2Cl_2 (40 mL) gave a crude allylic ether product which was purified by flash column chromatography (eluent: hexane/CH_2Cl_2/EtOAc = 15:5:2) to afford the title compound (0.64 g, 58%) as a colourless amorphous solid. **IR** ν_{max} (film) 1740 (C=O), 1593 (C=C), 1494, 1465, 1450, 1325, 1259, 1159, 1124 (C-O), 1111 (C-O), 1089 (C-Cl); **^{1}H NMR (400 MHz, $CDCl_3$)** δ **1.96** (3H, s, CH_3), **4.27** (1H, dd, *J* 11.8, 7.0, OC(1``)$H^AH^B$), **4.39** (1H, dd, *J* 11.8, 6.4, OC(1``)H^AH^B), **4.92** (1H, s, C(3)*H*), **5.88** (1H, app td, *J* 6.7, 1.5, C(2``)*H*), **6.09** (1H, d, *J* 8.7, ArC(7)*H*), **6.80** (1H, dd, *J* 8.8, ArC(6)*H*), **7.11–7.21** (10H, m, ArC(3`,4`,5`)*H*, ArC(4```)*H*, **7.21–7.27** (3H, m, ArC(3```,5```,)*H*, ArC(4)*H*), **7.30–7.34** (2H, m, ArC(2```,6```)*H*), **7.34–7.41** (6H, m, ArC(2`,6`)*H*); **^{13}C{^{1}H} NMR (101 MHz, $CDCl_3$)** δ **16.5** (CH_3), **66.4** (O*C*(1``)$H_2$CH), **74.7** (N*C*$Ph_3$), **74.8** (O*C*(3)HCO), **117.0** (Ar*C*(7)H), **123.3** (*C*(2``)*H*), **125.0** (Ar*C*(4)H), **126.0** (Ar*C*(2```,6```)H), **127.2** (Ar*C*(4`)H), **127.5** (Ar*C*(4```)H), **127.8** (Ar*C*(3a)), **127.9** (Ar*C*(3`,5`)H), **128.1** (Ar*C*(5)Cl), **128.3** (Ar*C*(6)H), **128.4** (Ar*C*(3```,5```)H), **129.3** (Ar*C*(2`,6`)H), **140.1** (*C*(3``)), **141.7** (Ar*C*(1`)H), **142.5** (*C*(7a)), **142.8** (Ar*C*(1```)), **176.5** (*C*=O); **HRMS (ESI$^+$)** $C_{37}H_{30}{}^{35}ClNO_2Na$ $[M+Na]^+$ found 578.1851, requires 578.1857 (–1.1 ppm).

(*E*)-5-Iodo-3-[(3-phenylbut-2-en-1-yl)oxy]-1-tritylindolin-2-one (S96)

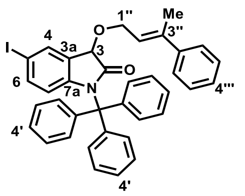

Following **General Procedure F**, 3-diazo-5-iodo-1-tritylindolin-2-one (1.16 g, 2.2 mmol), (*E*)-3-phenylbut-2-en-1-ol (0.296 g, 2.0 mmol), $Rh_2(OAc)_4$ (0.0044 g, 0.01 mmol) in CH_2Cl_2 (40 mL) gave a crude allylic ether product which was purified by flash column chromatography (eluent: hexane/CH_2Cl_2/EtOAc = 15:5:2) to afford the title compound (0.75 g, 58%) as a colourless amorphous solid. **IR** ν_{max} (film) 1732 (C=O), 1597 (C=C), 1492, 1467, 1440, 1419, 1381, 1311, 1286, 1261, 1193, 1161, 1122 (C-O), 1091, 1035 (C-I); **^{1}H NMR (400 MHz, CDCl$_3$)** δ **1.93–1.99** (3H, m, CH_3), **4.28** (1H, dd, *J* 11.8, 7.0, OC(1``)$H^A$H$^B$), **4.36–4.34** (1H, m, OC(1``)H^AH^B), **4.91** (1H, s, C(3)*H*), **5.88** (1H, app td, *J* 6.9, 1.3, C(2``)*H*), **5.94** (1H, d, *J* 8.6, ArC(7)*H*), **7.11–7.19** (10H, m, ArC(3`,4`,5`)*H*, ArC(6)*H*), **7.19–7.27** (3H, m, ArC(3```,4```,5```)*H*), **7.31–7.37** (8H, m, ArC(2`,6`)*H*, ArC(2```,6```)*H*), **7.54** (1H, dd, *J* 1.7, 1.1, ArC(4)*H*); **^{13}C{^{1}H} NMR (101 MHz, CDCl$_3$)** δ **16.4** (*C*H$_3$), **66.4** (O*C*(1``)H$_2$CH), **74.5** (O*C*(3)HCO), **85.6** (Ar*C*(5)), **117.8** (Ar*C*(7)H), **123.2** (*C*(2``)H), **125.9** (Ar*C*(2```,6```)H), **127.1** (Ar*C*(4`)H), **127.4** (Ar*C*(4```)H), **127.8** (Ar*C*(3`,5`)H), **128.3** (Ar*C*(3```,5```)H), **128.4** (Ar*C*(3a)), **129.2** (Ar*C*(2`,6`)H), **133.4** (Ar*C*(4)H), **137.1** (Ar*C*(6)H), **140.0** (*C*(3``)), **141.6** (Ar*C*(1`)), **142.7** (Ar*C*(1```)), **143.7** (Ar*C*(7a)), **176.1** (*C*=O); **HRMS (ESI$^+$)** $C_{37}H_{30}INO_2Na$ [M+Na]$^+$ found 670.1212, requires 670.1213 (–0.2 ppm).

(*E*)-5-Nitro-3-[(3-phenylbut-2-en-1-yl)oxy]-1-tritylindolin-2-one (S97)

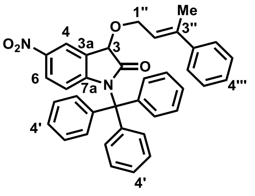

Following **General Procedure F**, 3-diazo-5-nitro-1-tritylindolin-2-one (0.981 g, 2.2 mmol), (*E*)-3-phenylbut-2-en-1-ol (0.296 g, 2.0 mmol), $Rh_2(OAc)_4$ (0.0044 g, 0.01 mmol) in CH_2Cl_2 (40 mL) gave a crude allylic ether product which was purified by flash column chromatography (eluent: hexane/CH_2Cl_2/EtOAc = 15:5:2) to afford the title compound (0.51 g, 45%) as a colourless amorphous solid. **IR** ν_{max} (film) 1748 (C=O), 1747, 1610 (C=O), 1514 (NO_2), 1490, 1467, 1442, 1338, 1271, 1182, 1159, 1122 (C-O), 1097 (C-O), 1066; **^{1}H NMR (500 MHz, $CDCl_3$)** δ **2.09** (3H, m, C*H*$_3$), **4.49** (1H, dd, *J* 11.8, 7.2, OCH^AH^B), **4.61** (1H, dd, *J* 11.8, 6.5, OCH^AH^B), **5.06** (1H, s, C(3)*H*), **6.00** (1H, app td, *J* 6.7, 1.4, C(2``)*H*), **6.37** (1H, d, *J* 9.1, ArC(7)*H*), **7.23–7.27** (3H, m, ArC(4`)*H*), **7.27–7.31** (7H, m, ArC(3`,5`,4```)*H*), **7.32–7.36** (2H, m, ArC(3```,5```)*H*), **7.40–7.46** (8H, m, C(2`,6`,2```,6```)*H*), **7.86** (1H, dd, *J* 9.0, 2.2, ArC(6)*H*), **8.21** (1H, dd, *J* 2.5, 1.1, ArC(4)*H*); **^{13}C{^{1}H} NMR (126 MHz, $CDCl_3$)** δ **16.5** (*C*H$_3$), **67.2** (O*C*(1``)H$_2$CH), **74.1** (*C*(3)HOCH$_2$), **75.2** (N*C*Ph$_3$), **115.5** (Ar*C*(7)H), **120.4** (Ar*C*(4)H), **122.8** (*C*(2``)H), **124.9** (Ar*C*(6)H), **126.0** (Ar*C*(2```,6```)H), **127.1** (Ar*C*(3a)), **127.5** (Ar*C*(4`)H), **127.7** (Ar*C*(4```)H), **128.2** (Ar*C*(3`,5`)H), **128.4** (Ar*C*(3```,5```)H), **129.2** (Ar*C*(2`,6`)H), **140.9** (*C*(3``)), **141.2** (Ar*C*(1`)), **142.6** (Ar*C*(1```)), **143.0** (Ar*C*(7a)), **149.8** (Ar*C*(5)NO$_2$), **177.0** (*C*=O); **HRMS (ESI$^+$)** $C_{37}H_{30}N_2O_4Na$ [M+Na]$^+$ found 589.2085, requires 589.2098 (–2.2 ppm).

(*E*)-5-Methyl-3-[(3-phenylbut-2-en-1-yl)oxy]-1-tritylindolin-2-one (S98)

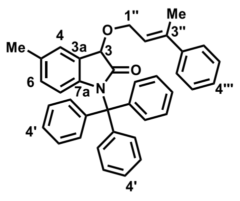

Following **General Procedure F**, 3-diazo-5-methyl-1-tritylindolin-2-one (0.913 g, 2.2 mmol), (*E*)-3-phenylbut-2-en-1-ol (0.296 g, 2.0 mmol), $Rh_2(OAc)_4$ (0.0044 g, 0.01 mmol) in CH_2Cl_2 (40 mL) gave a crude allylic ether product which was purified by flash column chromatography (eluent: hexane/CH_2Cl_2/EtOAc = 15:5:2) to afford the title compound (0.66 g, 62%) as a colourless amorphous solid. **IR** ν_{max} (film) 1728 (C=O), 1595 (C=C), 1487, 1448, 1321, 1300, 1265, 1188, 1151, 1126 (C-O), 1085, 1033; **^{1}H NMR (400 MHz, $CDCl_3$)** δ **1.94** (3H, s, C*H*3), **2.14** (3H, s, ArC(5)C*H*3), **4.23** (1H, dd, *J* 11.8, 6.9, OC(1``)$H^AH^B$), **4.36** (1H, dd, *J* 11.7, 6.6, OC(1``)H^AH^B), **4.91** (1H, s, C(3)*H*), **5.89** (1H, app td, *J* 6.6, 1.4, C(2``)*H*), **6.05** (1H, d, *J* 8.3, ArC(7)*H*), **6.64** (1H, d, *J* 8.2, ArC(6)*H*), **7.06–7.09** (1H, m, ArC(4)*H*), **7.10–7.14** (3H, m, ArC(4`)*H*), **7.15–7.20** (7H, m, ArC(3`,5`)*H*, ArC(4```)*H*), **7.21–7.26** (2H, m, ArC(3```,5```)*H*), **7.31–7.34** (2H, m, ArC(2```,6```)*H*), **7.35–7.43** (6H, m, ArC(2`,6`)*H*); **^{13}C{^{1}H} NMR (101 MHz, $CDCl_3$)** δ **16.4** (*CH3*), **20.9** (C*H*3Ar), **66.0** (O*C*(1``)$H_2$CH), **74.4** (N*C*$Ph_3$), **75.4** (O*C*(3)HCO), **115.8** (Ar*C*(7)H), **123.8** (*C*(2``)H), **125.4** (Ar*C*(4)H), **126.0** (Ar*C*(2```,6```)H), **126.1** (Ar*C*(5)CH_3), **127.0** (Ar*C*(4`)H), **127.4** (Ar*C*(4```)H), **127.8** (Ar*C*(3`,5`)H), **128.3** (Ar*C*(3```,5```)H), **128.8** (Ar*C*(6)H), **129.3** (Ar*C*(2,6)H), **132.0** (Ar*C*(3a)), **139.6** (*C*(3``)), **141.6** (Ar*C*(7a)), **142.2** (Ar*C*(1`)), **142.9** (Ar*C*(1``), **176.9** (*C*=O); **HRMS (ESI$^+$)** $C_{38}H_{33}NO_2Na$ $[M+Na]^+$ found 558.2397, requires 558.2404 (–1.2 ppm).

(*E*)-5-Methoxy-3-[(3-phenylbut-2-en-1-yl)oxy]-1-tritylindolin-2-one (S99)

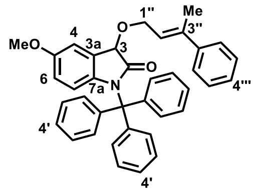

Following **General Procedure F**, 3-diazo-5-methoxy-1-tritylindolin-2-one (0.948 g, 2.2 mmol), (*E*)-3-phenylbut-2-en-1-ol (0.296 g, 2.0 mmol), $Rh_2(OAc)_4$ (0.0044 g, 0.01 mmol) in CH_2Cl_2 (40 mL) gave a crude allylic ether product which was purified by flash column chromatography (eluent: hexane/CH_2Cl_2/EtOAc = 15:5:2) to afford the title compound (0.58 g, 53%) as a colourless amorphous solid. **IR** ν_{max} (film) 1724 (C=O), 1595, 1485, 1446, 1265 (C-O), 1205, 1182, 1155; **^{1}H NMR (400 MHz, $CDCl_3$)** δ **1.93** (3H, s, C*H*$_3$), **3.60** (3H, s, ArC(5)OC*H*$_3$), **4.19** (1H, dd, *J* 11.8, 6.9, OC(1``)$H^A$H$^B$), **4.30** (1H, dd, *J* 11.8, 6.5, OCH$^A$$H^B$), **4.93** (1H, s, C(3)*H*), **5.87** (1H, app td, *J* 6.7, 1.4, C(2``)*H*), **6.07** (1H, d, *J* 8.9, ArC(7)*H*), **6.37** (1H, dd, *J* 8.9, 2.8, ArC(6)*H*), **6.85** (1H, d, *J* 2.0, ArC(4)*H*), **7.08–7.13** (3H, m, ArC(4)*H*), **7.14–7.19** (7H, m, ArC(3`,5`)*H*, ArC(4```)*H*), **7.20–7.24** (2H, m, ArC(3```,5```)*H*), **7.29–7.33** (2H, m, ArC(2```,6```)*H*), **7.35–7.43** (6H, m, ArC(2`,6`)*H*); **^{13}C{^{1}H} NMR (101 MHz, $CDCl_3$)** δ **16.4** (C(Ph)*C*H$_3$), **55.7** (ArC(5)O*C*H$_3$), **65.8** (O*C*(1``)H$_2$CH), **74.5** (N*C*Ph$_3$), **75.5** (O*C*(3)HCO), **110.9** (Ar*C*(4)H), **113.5** (Ar*C*(6)H), **116.7** (Ar*C*(7)H), **123.7** (*C*(2``)H), **125.9** (Ar*C*(2```,6```)H), **127.0** (Ar*C*(4)H), **127.3** (*C*(3a)), **127.4** (Ar*C*(4```)H), **127.8** (Ar*C*(3`,5`)H), **128.3** (Ar*C*(3```,5```)H), **129.3** (Ar*C*(2`,6`)H), **137.2** (Ar*C*(7a)), **139.6** (*C*(3``)), **142.1** (Ar*C*(1`)), **142.9** (Ar*C*(1```)), **155.5** (ArC(5)O*C*H$_3$), **176.7** (*C*=O); **HRMS (ESI$^+$)** $C_{38}H_{33}NO_3Na$ [M+Na]$^+$ found 574.2351, requires 574.2353 (–0.29 ppm).

(*E*)-6-Chloro-3-[(3-phenylbut-2-en-1-yl)oxy]-1-tritylindolin-2-one (S100)

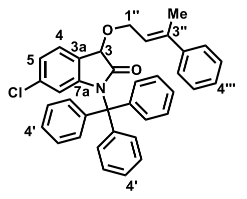

Following **General Procedure F**, 6-chloro-3-diazo-1-tritylindolin-2-one (0.957 g, 2.2 mmol), (*E*)-3-phenylbut-2-en-1-ol (0.296 g, 2.0 mmol) (0.296 g, 2.0 mmol), $Rh_2(OAc)_4$ (0.0044 g, 0.01 mmol) in CH_2Cl_2 (40 mL) gave a crude allylic ether product which was purified by flash column chromatography (eluent: hexane/CH_2Cl_2/EtOAc = 15:5:2) to afford the title compound (0.58 g, 52%) as a colourless amorphous solid. **IR** ν_{max} (film) 1732 (C=O), 1608 (C=C), 1585 (C=C), 1490, 1473, 1448, 1417, 1381, 1332, 1274, 1263, 1186, 1159, 1107, 1076 (C-Cl); **^{1}H NMR (500 MHz, $CDCl_3$)** δ **1.94** (3H, m, C*H*$_3$), **4.22** (1H, dd, *J* 11.9, 7.0, OC(1``)$H^AH^B$), **4.34** (1H, dd, *J* 11.8, 6.4, OC(1``)H^AH^B), **4.88** (1H, s, C(3)*H*), **5.86** (1H, app td, *J* 6.6, 1.3, C(2``)*H*), **6.10** (1H, d, *J* 1.8, ArC(7)*H*), **6.85** (1H, dd, *J* 7.9, 1.8, ArC(5)*H*), **7.13–7.26** (13H, m, ArC*H*), **7.30–7.33** (2H, m, ArC(2```,6```)*H*), **7.34–7.40** (6H, m, ArC(2`,6`)*H*); **^{13}C{^{1}H} NMR (126 MHz, $CDCl_3$)** δ **16.4** (*CH3*), **66.1** (O*C*(1``)$H_2$CH), **74.6** (O*C*(3)HCO), **74.8** (N*C*$Ph_3$), **116.3** (Ar*C*(7)H), **122.5** (Ar*C*(5)H), **123.4** (*C*(2``)H), **124.5** (Ar*C*(6)Cl), **125.6** (Ar*C*(4)H), **126.0** (Ar*C*(2```,6```)H), **127.3** (Ar*C*(4`)H), **127.5** (Ar*C*(4```)H), **128.0** (Ar*C*(3`,5`)H), **128.4** (Ar*C*(3```,5```)H), **129.3** (Ar*C*(2`,6`)H), **134.1** (Ar*C*(3a)), **140.0** (*C*(3``)), **141.7** (Ar*C*(1`)), **142.8** (Ar*C*(1```)), **145.2** (Ar*C*(7a)), **176.7** (*C*=O); **HRMS (ESI$^+$)** $C_{37}H_{30}{}^{35}ClNO_2Na$ [M+Na]$^+$ found 578.1851, requires 578.1857 (–1.1 ppm).

(*E*)-6-Bromo-3-[(3-phenylbut-2-en-1-yl)oxy]-1-tritylindolin-2-one (S101)

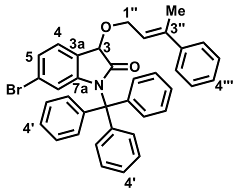

Following **General Procedure F**, 6-bromo-3-diazo-1-tritylindolin-2-one (1.05 g, 2.2 mmol), (*E*)-3-phenylbut-2-en-1-ol (0.296 g, 2.0 mmol) (0.296 g, 2.0 mmol), $Rh_2(OAc)_4$ (0.0044 g, 0.01 mmol) in CH_2Cl_2 (40 mL) gave a crude allylic ether product which was purified by flash column chromatography (eluent: hexane/CH_2Cl_2/EtOAc = 15:5:2) to afford the title compound (0.71 g, 59%) as a colourless amorphous solid. **IR** ν_{max} (film) 1732 (C=O), 1606 (C=C), 1575, 1490, 1471, 1448, 1411, 1334, 1186, 1157, 1109 (C-O), 1085, 1066 (C-Br); **^{1}H NMR (400 MHz, $CDCl_3$)** δ **1.94** (3H, s, C*H*3), **4.21** (1H, dd, *J* 11.8, 7.0, OC(1``)$H^AH^B$), **4.33** (1H, dd, *J* 11.8, 6.5, OC(1``)H^AH^B), **4.86** (1H, s, C(3)*H*), **5.86** (1H, app td, *J* 6.7, 1.4, C(2``)*H*), **6.23** (1H, d, *J* 1.6, ArC(7)*H*), **7.01** (1H, dd, *J* 7.9, 1.6, ArC(5)*H*), **7.11** (1H, dd, *J* 7.9, 1.1 ArC(4)*H*), **7.13–7.26** (13H, m, ArC*H*), **7.29–7.33** (2H, m, ArC(2```,6```)*H*), **7.34–7.40** (6H, m, ArC(2`,6`)*H*); **^{13}C{^{1}H} NMR (126 MHz, $CDCl_3$)** δ **16.4** (*C*H3), **66.1** (O*C*(1``)$H_2$CH), **74.6** (O*C*(3)HCO), **74.8** (N*C*Ph3), **119.1** (Ar*C*(7)H), **122.1** (Ar*C*(3a)), **123.4** (*C*(2``)H), **125.0** (Ar*C*(6)Br), **125.4** (Ar*C*(5)H), **125.9** (Ar*C*(4)H), **125.9** (Ar*C*(2```,6```)H), **127.3** (Ar*C*(4`)H), **127.5** (*C*(4```)H), **128.0** (Ar*C*(3`,5`)H), **128.4** (Ar*C*(3```,5```)H), **129.2** (Ar*C*(2`,6`)H), **140.0** (*C*(3``)), **141.6** (Ar*C*(1`)), **142.8** (Ar*C*(1```)), **145.4** (Ar*C*(7a)), **176.6** (*C*=O); **HRMS (ESI$^+$)** $C_{37}H_{30}{}^{79}BrNO_2Na$ $[M+Na]^+$ found 622.1332, requires 622.1352 (–3.2 ppm).

(*E*)-1-Benzyl-7-chloro-3-[(3-phenylbut-2-en-1-yl)oxy]indolin-2-one (S102)

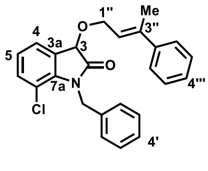

Following **General Procedure F**, 1-benzyl-7-chloro-3-diazoindolin-2-one (0.623 g, 2.2 mmol), (*E*)-3-phenylbut-2-en-1-ol (0.296 g, 2.0 mmol), $Rh_2(OAc)_4$ (0.0044 g, 0.01 mmol) in CH_2Cl_2 (40 mL) gave a crude allylic ether product which was purified by flash column chromatography (eluent: hexane/CH_2Cl_2/EtOAc = 15:5:2) to afford the title compound (0.56 g, 70%) as a colourless amorphous solid. **IR** ν_{max} (film) 1714 (C=O), 1610 (C=C), 1585, 1494, 1454, 1429, 1369, 1357, 1336, 1307, 1124 (C-O), 1105, 1082, 1062 (C-Cl), 1041, 1029, 1008; **^{1}H NMR (500 MHz, $CDCl_3$)** δ **2.11** (3H, s, C*H*$_3$), **4.55–4,67** (2H, m, OC(1``)*H*$_2$), **5.03** (1H, s, C(3)*H*), **5.29–5.37** (2H, m, NC*H*$_2$Ph), **5.98–6.09** (1H, m, C(2``)*H*), **7.01** (1H, dd, *J* 8.2, 7.3, ArC(5)*H*), **7.21** (1H, app dt, *J* 8.2, 1.0, ArC(6)*H*), **7.23–7.32** (6H, m, ArC*H*), **7.32–7.36** (3H, m, ArC(3```,4,5```)*H*), **7.41–7.46** (2H, m, ArC(2```,6```)*H*); **^{13}C{^{1}H} NMR (126 MHz, $CDCl_3$)** δ **16.4** (*C*H$_3$), **44.8** (N*C*H$_2$Ph), **66.1** (O*C*(1``)H$_2$CH), **74.1** (O*C*(3)HCO), **115.8** (Ar*C*(7)Cl), **123.2** (*C*(2``)*H*), **124.0** (Ar*C*(5)H), **124.2** (Ar*C*(4)H), **126.0** (Ar*C*(2```,6```)H), **126.7** (Ar*C*(2`,6`)H), **127.4** (Ar*C*(4```)H), **127.6** (Ar*C*(4`)H), **128.4** (Ar*C*(3`,5`)H), **128.5** (Ar*C*(3a)), **128.7** (Ar*C*(3```,5```)H), **132.5** (Ar*C*(6)H), **137.4** (Ar*C*(7a)), **139.5** (Ar*C*(1`)), **140.5** (*C*(3``)), **142.8** (Ar*C*(1```)), **175.7** (*C*=O); **HRMS (ESI$^+$)** $C_{25}H_{22}{}^{35}ClNO_2Na$ $[M+Na]^+$ found 626.1229, requires 426.1231 (–0.5 ppm).

(*Z*)-3-[(3-Fluoro-3-phenylallyl)oxy]-1-tritylindolin-2-one (41)

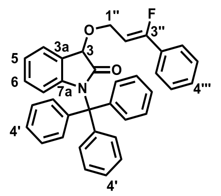

Following **General Procedure F**, 3-diazo-1-tritylindolin-2-one (2.21 g, 5.5 mmol), (*Z*)-3-fluoro-3-phenylprop-2-en-1-ol (0.76 g, 5.0 mmol), $Rh_2(OAc)_4$ (0.0044 g, 0.01 mmol) in CH_2Cl_2 (40 mL) gave a crude allylic ether product which was purified by flash column chromatography (eluent: hexane/CH_2Cl_2/EtOAc = 15:5:2) to afford the title compound (2.13 g, 81%) as a pale-yellow solid. **mp** 132–134 °C; **IR** ν_{max} (film) 1724 (C=O), 1685 (C=C), 1598 (C=C), 1492, 1465, 1448, 1334, 1305, 1276 (C-F), 1186, 1157, 1114 (C-O), 1089; **^{1}H NMR (400 MHz, $CDCl_3$)** δ **4.41** (1H, ddd, *J* 11.8, 7.3, $^4J_{H-F}$ 2.0, OC(1``)$H^A$H$^B$), **4.57** (1H, ddd, *J* 11.8, 6.9, $^4J_{H-F}$ 2.2, OC(1``)H^AH^B), **5.03** (1H, s, C(3)*H*), **5.66** (1H, app dt, $^3J_{H-F}$ 36.5, *J* 7.1, C(2``)*H*), **6.27** (1H, dd, *J* 7.4, 1.6, ArC(7)*H*), **6.88**–**7.00** (2H, m, ArC(5,6)*H*), **7.18**–**7.23** (3H, m, ArC(4`)*H*), **7.23**–**7.29** (6H, m, ArC(3`,5`)*H*), **7.33**–**7.40** (4H, m, ArC(4, 3```,4```,5```)*H*), **7.43**–**7.49** (6H, m, ArC(2`,6`)*H*), **7.50**–**7.57** (2H, m, ArC(2```,6```)*H*). **^{13}C{^{1}H} NMR (101 MHz, $CDCl_3$)** δ **62.5** (d, $^3J_{C-F}$ 7.1, O*C*(1``)$H_2$), **74.6** (*NC*$Ph_3$), **75.6** (O*C*(3)H), **102.4** (d, $^2J_{C-F}$ 15.0, *C*(2``)H), **116.1** (Ar*C*(7)H), **122.5** (Ar*C*(5)H), **124.7** (Ar*C*(3```,5```)H), **124.7** (Ar*C*(4)H), **125.9** (Ar*C*(3a)), **127.0** (Ar*C*(4`)H), **127.8** (Ar*C*(3`,5`)H), **128.4** (Ar*C*(6)H), **128.6** (Ar*C*(2```,6```)H), **129.4** (Ar*C*(2`,6`)H), **129.5** (Ar*C*(4```)H), **131.8** (d, $^2J_{C-F}$ 28.5, Ar*C*(1```)), **142.1** (Ar*C*(1`)), **144.1** (Ar*C*(7a)), **158.9** (d, $^1J_{C-F}$ 252.6, *C*(3``)F), **176.7** (*C*=O); **^{19}F{^{1}H} NMR (470 MHz, $CDCl_3$)** δ **–115.51**. **HRMS (ESI$^+$)** $C_{36}H_{28}FNO_2Na$ $[M+Na]^+$ found 548.2010, requires 548.1996 (–2.55 ppm).

(*Z*)-3-{[3-fluoro-3-(p-tolyl)allyl]oxy}-1-tritylindolin-2-one (S103)

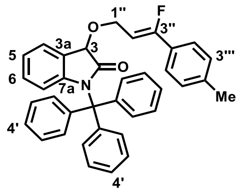

Following **General Procedure F**, 3-diazo-1-tritylindolin-2-one (0.441 g, 1.1 mmol), (*Z*)-3-fluoro-3-(p-tolyl)prop-2-en-1-ol, $Rh_2(OAc)_4$ (0.0044 g, 0.01 mmol) in CH_2Cl_2 (40 mL) gave a crude allylic ether product which was purified by flash column chromatography (eluent: hexane/CH_2Cl_2/EtOAc = 15:5:2) to afford the title compound (0.469 g, 87%) as a colourless amorphous solid. **mp** 130–132 °C; **IR** ν_{max} (film) 1734 (C=O), 1680 (C=C), 1606 (C=C), 1465, 1448, 1303, 1278 (C-F), 1240, 1180, 1151, 1128 (C-O); **^{1}H NMR (500 MHz, $CDCl_3$)** δ **2.37** (3H, s, C*H*$_3$), **4.41** (1H, ddd, *J* 11.7, 7.3, $^4J_{H-F}$ 1.9, OC(1``)*H*$^A$H$^B$), **4.57** (1H, ddd, *J* 11.8, 7.0, $^4J_{H-F}$ 2.1, OC(1``)H^A*H*^{B}), **5.02** (1H, s, C(3)*H*), **5.60** (1H, dt, $^3J_{H-F}$ 36.5, *J* 7.1, C(2``)*H*), **6.26** (1H, d, *J* 7.5, ArC(7)*H*), **6.88**–**7.00** (2H, m, ArC(5,6)*H*), **7.14**–**7.24** (5H, m, ArC(4`,3```,5```)*H*), **7.23**–**7.29** (6H, m, ArC(3`,5`)*H*), **7.36** (1H, d, *J* 6.5, ArC(4)*H*), **7.42** (2H, app d, *J* 8.0, ArC(2```,6```)*H*), **7.47** (2H, app dd, *J* 7.6, 1.7, ArC(2`,6`)*H*). **^{13}C{^{1}H} NMR (126 MHz, $CDCl_3$)** δ **21.5** (*C*H$_3$), **62.5** (d, $^4J_{C-F}$ *J* 7.2, O*C*(1``)H$_2$), **74.6** (*NC*Ph$_3$), **75.5** (O*C*(3)H), **101.4** (d, $^2J_{C-F}$ 15.0, *C*(2``)H), **116.1** (Ar*C*(7)H), **122.5** (Ar*C*(5)H), **124.6** (Ar*C*(2```,6```)H), **124.7** (Ar*C*(4)H), **125.9** (Ar*C*(3a)), **127.0** (Ar*C*(4`)H), **127.8** (Ar*C*(3`,5`)H), **128.4** (Ar*C*(6)H), **129.1** (d, $^2J_{C-F}$ 28.6, Ar*C*(1```)), **129.3** (Ar*C*(3```,5```)), **129.4** (Ar*C*(2`,6`)H), **139.6** (Ar*C*(4```)*C*H$_3$), **142.1** (Ar*C*(1`)), **144.1** (Ar*C*(7a)), **159.2** (d, $^1J_{C-F}$ 252.3, *C*(3``)F), **176.8** (*C*=O); **^{19}F{^{1}H} NMR (470 MHz, $CDCl_3$)** δ **–115.32**. **HRMS (ESI$^+$)** $C_{37}H_{31}FNO_2Na$ $[M+H]^+$ found 540.2311, requires 540.2333 (–4.13 ppm).

(*Z*)-3-{[3-Fluoro-3-(4-methoxyphenyl)allyl]oxy}-1-tritylindolin-2-one (S104)

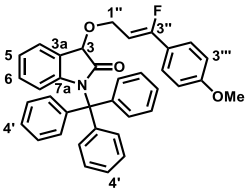

Following **General Procedure F**, 3-diazo-1-tritylindolin-2-one (0.441 g, 1.1 mmol), (*Z*)-3-fluoro-3-(4-methoxyphenyl)prop-2-en-1-ol (0.182 g, 1.0 mmol), $Rh_2(OAc)_4$ (0.0044 g, 0.01 mmol) in CH_2Cl_2 (40 mL) gave a crude allylic ether product which was purified by flash column chromatography (eluent: hexane/CH_2Cl_2/EtOAc = 15:5:2) to afford the title compound (0.461 g, 83%) as a pale pink solid. **mp** 140–142 °C; **IR** ν_{max} (film) 1734 (C=O), 1681 (C=C), 1608 (C=C), 1595 (C=C), 1514, 1485, 1307, 1253 (C-F), 1240, 1178, 1151, 1118 (C-O), 1089; **^{1}H NMR (400 MHz, $CDCl_3$)** δ **3.83** (3H, s, OC*H*$_3$), **4.40** (1H, ddd, *J* 11.7, 7.4, $^4J_{H-F}$ 1.9, OC(1``)$H^A$H$^B$), **4.56** (1H, ddd, *J* 11.6, 7.0, $^4J_{H-F}$ 2.1, OC(1``)H^AH^B), **5.03** (1H, s, C(3)*H*), **5.52** (1H, app dt, $^3J_{H-F}$ 36.6, *J* 7.2, C(2``)*H*), **6.14–6.41** (1H, m, ArC(7)*H*), **6.85–6.92** (2H, m, ArC(3```,5```)*H*), **6.89–6.99** (2H, m, ArC(5,6)*H*), **7.18–7.23** (3H, m, ArC(4`)*H*), **7.24–7.30** (6H, m, ArC(3`,5`)*H*), **7.37** (1H, ddd, *J* 6.7, 2.4, 1.1, ArC(4)*H*), **7.43–7.53** (8H, m, ArC(2`,6`,2```,6```)*H*). **^{13}C{^{1}H} NMR (101 MHz, $CDCl_3$)** δ **55.5** (O*C*H$_3$), **62.6** (d, $^3J_{C-F}$ 7.2, O*C*(1``)H$_2$), **74.6** (*NC*Ph$_3$), **75.4** (O*C*(3)H), **100.3** (d, $^2J_{C-F}$ 15.2, *C*(2``)H), **114.0** (d, $^4J_{C-F}$ 1.8, Ar*C*(3```,5```)H), **116.1** (Ar*C*(7)H), **122.4** (Ar*C*(5)H), **124.4** (d, $^2J_{C-F}$ 29.2, Ar*C*(1```)), **124.7** (Ar*C*(4)H), **126.0** (Ar*C*(3a)), **126.2**(d, $^3J_{C-F}$ 7.2, Ar*C*(2```,6```)H), **127.0** (Ar*C*(4`)H), **127.8** (Ar*C*(3`,5`)H), **128.4** (Ar*C*(6)H), **129.4** (Ar*C*(2`,6`)H), **142.1** (Ar*C*(1`)), **144.1** (Ar*C*(7a)), **159.1** (d, $^1J_{C-F}$ 251.9, *C*(3``)F), **160.6** (Ar*C*(4```)O*C*H$_3$), **176.8** (*C*=O); **^{19}F{^{1}H} NMR (377 MHz, $CDCl_3$)** δ **–114.80**. **HRMS (ESI$^+$)** $C_{37}H_{30}FNO_3Na$ $[M+H]^+$ found 578.2100, requires 578.2102 (–0.33 ppm).

(*Z*)-3-{[3-Fluoro-3-(4-(trifluoromethyl)phenyl)allyl]oxy}-1-tritylindolin-2-one (S105)

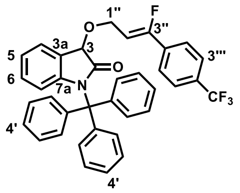

Following **General Procedure F**, 3-diazo-1-tritylindolin-2-one (0.441 g, 1.1 mmol), (*Z*)-3-fluoro-3-(4-(trifluoromethyl)phenyl)prop-2-en-1-ol (0.22 g, 1.0 mmol), $Rh_2(OAc)_4$ (0.0044 g, 0.01 mmol) in CH_2Cl_2 (40 mL) gave a crude allylic ether product which was purified by flash column chromatography (eluent: hexane/CH_2Cl_2/EtOAc = 15:5:2) to afford the title compound (0.415 g, 70%) as a colourless amorphous solid. **mp** 150–152 °C; **IR** ν_{max} (film) 1734 (C=O), 1681 (C=C), 1608 (C=C), 1465, 1450, 1323, 1303, 1170, 1126 (C-F), 1114 (C-O), 1068; **^{1}H NMR (500 MHz, $CDCl_3$)** δ **4.40** (1H, ddd, *J* 12.1, 7.1, $^4J_{H\text{-}F}$ 2.1, OC(1``)$H^AH^B$), **4.59** (1H, ddd, *J* 12.2, 6.8, $^4J_{H\text{-}F}$ 2.2, OC(1``)H^AH^B), **5.04** (1H, s, C(3)*H*), **5.77** (1H, app dt, $^3J_{H\text{-}F}$ 36.3, *J* 6.9, C(2``)*H*), **6.28** (1H, d, *J* 7.5, ArC(7)*H*), **6.87–7.02** (2H, m, ArC(5,6)*H*), **7.17–7.23** (3H, m, ArC(4`)*H*), **7.24–7.30** (6H, m, ArC(2`,6`)*H*), **7.36** (1H, d, *J* 6.8, ArC(4)*H*), **7.43–7.51** (6H, m, ArC(2`,6`)*H*), **7.62** (4H, s, ArC(2```,3```,5```,6```)*H*). **^{13}C{^{1}H} NMR (126 MHz, $CDCl_3$)** δ **62.4** (d, $^3J_{C\text{-}F}$ 6.9, O*C*(1``)$H_2$), **74.6** (*NC*$Ph_3$), **75.8** (*C*(3)H), **105.0** (d, $^2J_{C\text{-}F}$ 14.5, *C*(2``)H), **116.2** (Ar*C*(7)H), **122.5** (Ar*C*(5)H), **124.0** (q, $^1J_{C\text{-}F}$ 263.3, *C*F_3), **124.7** (Ar*C*(4)H), **124.9** (q, $^3J_{C\text{-}F}$ 7.1, Ar*C*(2```,6```)), **125.7** (q, $^4J_{C\text{-}F}$ 4.5, Ar*C*(3```,5```)H), **127.1** (Ar*C*(4`)H), **127.9** (Ar*C*(3`,5`)H), **128.6** (Ar*C*(6)H), **129.3** (Ar*C*(2`,6`)H), **131.2** (q, $^2J_{C\text{-}F}$ 32.7, Ar*C*(4```)), **135.2** (d, $^2J_{C\text{-}F}$ 28.6, Ar*C*(1```)), **142.0** (Ar*C*(1`)), **144.1** (*C*(7a)), **157.2** (d, $^1J_{C\text{-}F}$ 252.4, *C*(3``)F), **176.7** (*C*=O); **$^{19}$F NMR (470 MHz, $CDCl_3$)** δ **–62.77** (3F, s, C*F*$_3$), **–116.09** (1F, s, C(3``)*F*). **HRMS (ESI$^+$)** $C_{37}H_{27}F_4NO_2Na$ $[M+Na]^+$ found 616.1868, requires 616.1870 (–0.35 ppm).

(*Z*)-4-Chloro-3-[(3-fluoro-3-phenylallyl)oxy]-1-tritylindolin-2-one (S106)

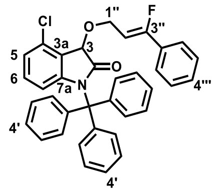

Following **General Procedure F**, 4-chloro-3-diazo-1-tritylindolin-2-one (0.479 g, 1.1 mmol), (*Z*)-3-fluoro-3-phenylprop-2-en-1-ol (0.152 g, 1.0 mmol), $Rh_2(OAc)_4$ (0.0044 g, 0.01 mmol) in CH_2Cl_2 (40 mL) gave a crude allylic ether product which was purified by flash column chromatography (eluent: hexane/CH_2Cl_2/EtOAc = 15:5:2) to afford the title compound (0.352 g, 63%) as a pale-yellow solid. **mp** 136–138 °C; **IR** ν_{max} (film) 1728 (C=O), 1604 (C=C), 1597 (C=C), 1489, 1446, 1253 (C-F), 1141 (C-O), 1001; **^{1}H NMR (500 MHz, $CDCl_3$)** δ **4.56** (1H, ddd, *J* 11.8, 7.3, $^4J_{H-F}$ 1.9, OC(1``)$H^AH^B$), **4.65** (1H, ddd, *J* 11.8, 6.8, $^4J_{H-F}$ 2.2, OC(1``)H^AH^B), **4.95** (1H, s, C(3)*H*), **5.73** (1H, app dt, $^3J_{H-F}$ 36.4, *J* 7.1, C(2``)*H*), **6.15** (1H, d, *J* 8.0, ArC(7)*H*), **6.85** (1H, t, *J* 8.1, ArC(5)*H*), **6.90** (1H, d, *J* 8.1, ArC(6)*H*), **7.19–7.23** (3H, m, ArC(4`)*H*), **7.24–7.29** (6H, m, ArC(3`,5`)*H*), **7.33–7.40** (3H, m, ArC(3```,4```,5```)*H*), **7.40–7.47** (6H, m, ArC(2`,6`)*H*), **7.50–7.56** (2H, m, ArC(2```,6```)*H*). **^{13}C{^{1}H} NMR (126 MHz, $CDCl_3$)** δ **63.2** (d, $^3J_{C-F}$ 7.3, O*C*(1``)$H_2$), **74.8** (*NC*$Ph_3$), **74.9** (O*C*(3)H), **102.3** (d, $^2J_{C-F}$ 14.8, *C*(2``)H), **114.5** (Ar*C*(7)H), **123.2** (Ar*C*(5)H), **123.4** (Ar*C*(3a)), **124.7** (Ar*C*(3```,5```)H), **127.2** (Ar*C*(4`)H), **127.9** (Ar*C*(3`,5`)H), **128.6** (Ar*C*(2```,6```)H), **129.3** (Ar*C*(2`,6`)H), **129.4** (Ar*C*(4```)H), **129.8** (Ar*C*(6)H), **131.9** (d, $^2J_{C-F}$ 28.5, Ar*C*(1```)), **132.0** (Ar*C*(4)Cl), **141.7** (Ar*C*(1`)), **145.9** (Ar*C*(7a)), **158.9** (d, $^1J_{C-F}$ 252.9, *C*(3``)F), **175.3** (*C*=O); **^{19}F{^{1}H} NMR (470 MHz, $CDCl_3$)** δ **–115.47**. **HRMS (ESI$^+$)** $C_{36}H_{27}F^{35}ClNO_2Na$ $[M+Na]^+$ found 582.1601, requires 582.1607 (–1.03 ppm).

(*Z*)-5-Fluoro-3-[(3-fluoro-3-phenylallyl)oxy]-1-tritylindolin-2-one (S107)

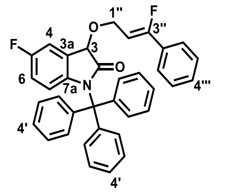

Following **General Procedure F**, 3-diazo-5-fluoro-1-tritylindolin-2-one (0.461 g, 1.1 mmol), (*Z*)-3-fluoro-3-phenylprop-2-en-1-ol, $Rh_2(OAc)_4$ (0.0044 g, 0.01 mmol) in CH_2Cl_2 (40 mL) gave a crude allylic ether product which was purified by flash column chromatography (eluent: hexane/CH_2Cl_2/EtOAc = 15:5:2) to afford the title compound (0.418 g, 77%) as a pale-yellow amorphous solid. **mp** 134–136 °C; **IR** ν_{max} (film) 1730 (C=O), 1685 (C=C), 1608 (C=C), 1481, 1448, 1282 (C-F), 1267 (C-F), 1163, 1122 (C-O), 1089; **^{1}H NMR (500 MHz, $CDCl_3$)** δ **4.44** (1H, ddd, *J* 11.8, 7.4, $^4J_{C\text{-}F}$ 1.9, OC(1``)$H^A$H$^B$), **4.59** (1H, ddd, *J* 11.8, 6.9, $^4J_{C\text{-}F}$ 2.2, OC(1``)H^AH^B), **5.02** (1H, s, C(3)*H*), **5.66** (1H, app dt, $^3J_{H\text{-}F}$ 36.3, 7.1, C(2``)*H*), **6.20** (1H, dd, *J* 9.0, $^4J_{C\text{-}F}$ 4.1, ArC(7)*H*), **6.63** (1H, app td, $^3J_{H\text{-}F}$ 9.0, 2.8, ArC(6)*H*), **7.10** (1H, dd, *J* 7.3, 2.3, ArC(4)*H*), **7.20–7.24** (3H, m, ArC(4`)*H*), **7.24–7.31** (6H, m, ArC(3`,5`)*H*), **7.33–7.42** (3H, m, ArC(3```,4```,5```)*H*), **7.41–7.48** (6H, m, ArC(2`,6`)*H*), **7.49–7.57** (2H, m, ArC(2```,6```)*H*). **^{13}C{^{1}H} NMR (126 MHz, $CDCl_3$)** δ **62.8** (d, $^3J_{C\text{-}F}$ 7.2, O*C*(1``)$H_2$), **74.7** (*NC*$Ph_3$), **75.3** (O*C*(3)H), **102.0** (d, $^2J_{C\text{-}F}$ 14.9, *C*(2``)H), **112.4** (d, $^2J_{C\text{-}F}$ 24.2, Ar*C*(4)H), **114.9** (d, $^2J_{C\text{-}F}$ 22.9, Ar*C*(6)H), **116.9** (d, $^3J_{C\text{-}F}$ 7.5, Ar*C*(6)H), **124.7** (d, $^3J_{C\text{-}F}$ 7.0, Ar*C*(2```,6```)H), **127.2** (Ar*C*(4`)H), **127.5** (d, $^3J_{C\text{-}F}$ 7.9, Ar*C*(3a)), **127.9** (Ar*C*(3`,5`)H), **128.6** (d, $^4J_{C\text{-}F}$ 1.9, Ar*C*(3```,5```)H), **129.3** (Ar*C*(2`,6`)H), **129.6** (Ar*C*(4```)H), **131.7** (d, $^2J_{C\text{-}F}$ 28.4, Ar*C*(1```)), **139.8** (d, $^4J_{C\text{-}F}$ 1.9, Ar*C*(7a)), **141.8** (Ar*C*(1`)), **158.8** (d, $J_{C\text{-}F}$ 242.7, Ar*C*(5)F), **159.1** (d, $^1J_{C\text{-}F}$ 253.0, *C*(3``)F), **176.6** (*C*=O); **$^{19}$F{$^1$H} NMR (470 MHz, $CDCl_3$)** δ **–115.17** (1F, s, C(3``)*F*), **–120.66** (1F, s, ArC(5)*F*). **HRMS (ESI$^+$)** $C_{36}H_{27}F_2NO_2Na$ [M+Na]$^+$ found 566.1895, requires 566.1902 (–1.25 ppm).

(*Z*)-3-[(3-Fluoro-3-phenylallyl)oxy]-5-nitro-1-tritylindolin-2-one (S108)

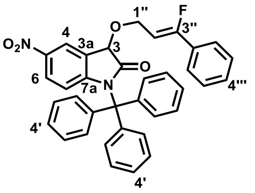

Following **General Procedure F**, 3-diazo-5-nitro-1-tritylindolin-2-one (0.491 g, 1.1 mmol), (*Z*)-3-fluoro-3-phenylprop-2-en-1-ol, $Rh_2(OAc)_4$ (0.0044 g, 0.01 mmol) in CH_2Cl_2 (40 mL) gave a crude allylic ether product which was purified by flash column chromatography (eluent: hexane/CH_2Cl_2/EtOAc = 15:5:2) to afford the title compound (0.456 g, 80%) as a pale-yellow solid. **mp** 138–140 °C; **IR** ν_{max} (film) 1745 (C=O), 1678 (C=C), 1612 (C=C), 1514 (NO_2), 1490, 1444, 1338 (NO_2), 1273 (C-F), 1122 (C-O), 1093, 1066; **^{1}H NMR (500 MHz, $CDCl_3$)** δ **4.60** (1H, ddd, *J* 11.9, 7.6, $^4J_{H\text{-}F}$ 1.8, OC(1``)$H^AH^B$), **4.70** (1H, ddd, *J* 11.9, 6.9, $^4J_{H\text{-}F}$ 2.2, OC(1``)H^AH^B), **5.07** (1H, s, C(3)*H*), **5.69** (1H, app dt, $^3J_{H\text{-}F}$ 36.0, *J* 7.2, C(2``)*H*), **6.38** (1H, d, *J* 9.0, ArC(7)*H*), **7.22–7.27** (3H, m, ArC(4`)*H*), **7.27–7.32** (6H, m, ArC(3`,5`)*H*), **7.37–7.41** (3H, m, ArC(3```,4```,5```)*H*), **7.40–7.46** (6H, m, ArC(2`,6`)*H*), **7.53–7.60** (2H, m, ArC(2```,6```)*H*), **7.86** (1H, dd, *J* 9.0, 2.5, ArC(6)*H*), **8.23** (1H, dd, *J* 2.5, 1.1, ArC(4)*H*). **^{13}C{^{1}H} NMR (126 MHz, $CDCl_3$)** δ **63.7** (d, $^3J_{C\text{-}F}$ 7.1, O*C*(1``)$H_2$), **74.4** (O*C*(3)H), **75.2** (*NC*$Ph_3$), **101.5** (d, $^2J_{C\text{-}F}$ 14.9, *C*(2``)H), **115.6** (Ar*C*(7)H), **120.4** (Ar*C*(4)H), **124.7** (d, $^3J_{C\text{-}F}$ 7.2, Ar*C*(2```,6```)H), **124.9** (Ar*C*(4)H), **126.9** (Ar*C*(3a)), **127.5** (Ar*C*(4`)H), **128.1** (Ar*C*(3`,5`)H), **128.7** (d, $^4J_{C\text{-}F}$ 1.9, Ar*C*(3```,5```)H), **129.2** (Ar*C*(2`,6`)H), **129.7** (Ar*C*(4```)H), **131.5** (d, $^2J_{C\text{-}F}$ 28.4, Ar*C*(1```)), **142.2** (Ar*C*(1`)), **143.0** (Ar*C*(5)NO_2), **149.8** (Ar*C*(7a)), **159.6** (d, $^1J_{C\text{-}F}$ 253.6, *C*(3``)F), **176.8** (*C*=O); **^{19}F{^{1}H} NMR (470 MHz, $CDCl_3$)** δ **–114.50**. **HRMS (ESI$^+$)** $C_{36}H_{27}FN_2O_4Na$ $[M+Na]^+$ found 593.1844, requires 593.1847 (–0.57 ppm).

(*Z*)-3-[(3-Fluoro-3-phenylallyl)oxy]-5-methoxy-1-tritylindolin-2-one (S109)

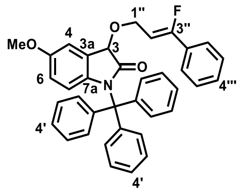

Following **General Procedure F**, 3-diazo-5-methoxy-1-tritylindolin-2-one (0.474 g, 1.1 mmol), (*Z*)-3-fluoro-3-phenylprop-2-en-1-ol, $Rh_2(OAc)_4$ (0.0044 g, 0.01 mmol) in CH_2Cl_2 (40 mL) gave a crude allylic ether product which was purified by flash column chromatography (eluent: hexane/CH_2Cl_2/EtOAc = 15:5:2) to afford the title compound (0.394 g, 71%) as a pale pink amorphous solid. **IR** ν_{max} (film) 1724 (C=O), 1595 (C=C), 1485, 1448, 1280 (C-F), 1186, 1157, 1126, 1085 (C-O), 1031; **^{1}H NMR (500 MHz, $CDCl_3$)** δ **3.71** (3H, s, OC*H*$_3$), **4.38** (1H, ddd, *J* 11.8, 7.2, $^4J_{H\text{-}F}$ 2.0, OC(1``)$H^A$H$^B$), **4.56** (1H, ddd, *J* 11.8, 6.9, $^4J_{H\text{-}F}$ 2.2, OC(1``)H^AH^B), **5.03** (1H, s, C(3)*H*), **5.67** (1H, app dt, $^3J_{H\text{-}F}$ 36.4, *J* 7.1, C(2``)*H*), **6.17** (1H, d, *J* 8.9, ArC(7)*H*), **6.37–6.57** (1H, m, ArC(6)*H*), **6.97** (1H, dd, *J* 2.9, 1.1, ArC(4)*H*), **7.19–7.23** (3H, m, ArC(4`)*H*), **7.23–7.30** (6H, m, ArC(3`,5`)*H*), **7.34–7.41** (3H, m, ArC(3```,4```,5```)*H*), **7.44–7.50** (6H, m, ArC(2`,6`)*H*), **7.50–7.56** (2H, m, ArC(2```,6```)*H*). **^{13}C{^{1}H} NMR (126 MHz, $CDCl_3$)** δ **55.7** (O*C*H$_3$), **62.4** (d, $^3J_{C\text{-}F}$ 7.1, O*C*(1``)H$_2$), **74.5** (*NC*Ph$_3$), **74.8** (O*C*(3)H), **102.3** (d, $^2J_{C\text{-}F}$ 14.9, *C*(2``)H), **110.9** (Ar*C*(4)H), **113.6** (Ar*C*(6)H), **116.8** (Ar*C*(7)H), **124.6** (d, $^3J_{C\text{-}F}$ 7.2, Ar*C*(2```,6```)H), **127.0** (Ar*C*(4`)H), **127.8** (Ar*C*(3`,5`)H), **128.6** (d, $^4J_{C\text{-}F}$ 2.0, Ar*C*(3```,5```)H), **129.3** (Ar*C*(2`,6`)H), **129.4** (Ar*C*(4```)H), **131.8** (d, $^2J_{C\text{-}F}$ 28.4, Ar*C*(1```)), **137.2** (Ar*C*(7a)), **142.1** (Ar*C*(1`)), **155.5** (Ar*C*(5)OMe), **158.8** (d, $^1J_{C\text{-}F}$ 252.6, *C*(3``)F), **176.5** (*C*=O); **^{19}F{^{1}H} NMR (470 MHz, $CDCl_3$)** δ **–115.50**. **HRMS (ESI$^+$)** $C_{37}H_{30}FNO_3Na$ [M+Na]$^+$ found 578.2107, requires 578.2102 (+0.88 ppm).

(*Z*)-6-Chloro-3-[(3-fluoro-3-phenylallyl)oxy]-1-tritylindolin-2-one (S110)

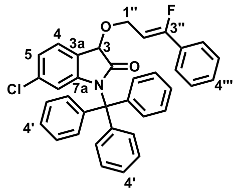

Following **General Procedure F**, 6-chloro-3-diazo-1-tritylindolin-2-one (0.479 g, 1.1 mmol), (*Z*)-3-fluoro-3-phenylprop-2-en-1-ol (0.152 g, 1.0 mmol), $Rh_2(OAc)_4$ (0.0044 g, 0.01 mmol) in CH_2Cl_2 (40 mL) gave a crude allylic ether product which was purified by flash column chromatography (eluent: hexane/CH_2Cl_2/EtOAc = 15:5:2) to afford the title compound (0.419 g, 75%) as a colourless amorphous solid. **IR** ν_{max} (film) 1730 (C=O), 1674, 1608 (C=C), 1577, 1489, 1473, 1448, 1413, 1330, 1286 (C-F), 1118, 1083 (C-O); **^{1}H NMR (500 MHz, $CDCl_3$)** δ **4.41** (1H, ddd, *J* 11.8, 7.3, $^4J_{H\text{-}F}$ 1.9, OC(1``)$H^A$H$^B$), **4.56** (1H, ddd, *J* 11.8, 7.0, $^4J_{H\text{-}F}$ 2.2, OC(1``)H^AH^B), **4.97** (1H, s, C(3)*H*), **5.64** (1H, app dt, $^3J_{H\text{-}F}$ 36.3, *J* 7.1, CH_2C(2``)*H*), **6.20** (1H, d, *J* 1.7, ArC(7)*H*), **6.94** (1H, dd, *J* 7.9, 1.8, ArC(5)*H*), **7.21–7.25** (3H, m, ArC(4`)*H*), **7.25–7.31** (7H, m, ArC(4,3`,5`)*H*), **7.35–7.40** (3H, m, ArC(3```,4```,5```)*H*), **7.42–7.48** (6H, m, ArC(2`,6`)*H*), **7.49–7.56** (2H, m, ArC(2```,6```)*H*). **^{13}C{^{1}H} NMR (126 MHz, $CDCl_3$)** δ **62.6** (d, $^3J_{C\text{-}F}$ 7.1, O*C*(1``)$H_2$), **74.8** (*NC*$Ph_3$), **74.9** (O*C*(3)H), **102.0** (d, $^2J_{C\text{-}F}$ 14.9, *C*(2``)H), **116.4** (Ar*C*(7)H), **122.5** (Ar*C*(5)H), **124.3** (Ar*C*(3a)), **124.7** (Ar*C*(2```,6```)H), **125.6** (Ar*C*(4)H), **127.3** (Ar*C*(4`)H), **128.0** (Ar*C*(3`,5`)H), **128.6** (d, *J* 1.9, Ar*C*(3```,5```)H), **129.3** (Ar*C*(2`,6`)H), **129.5** (Ar*C*(4```)H), **131.7** (d, $^2J_{C\text{-}F}$ 28.3, Ar*C*(1```)), **134.2** (Ar*C*(6)Cl), **141.6** (Ar*C*(1`)), **145.2** (Ar*C*(7a)), **159.1** (d, $^1J_{C\text{-}F}$ 253.0, *C*(3``)F), **176.5** (*C*=O); **^{19}F{^{1}H} NMR (470 MHz, $CDCl_3$)** δ **–115.25**. **HRMS (ESI$^+$)** $C_{36}H_{27}F^{35}ClNO_2Na$ [M+Na]$^+$ found 582.1595, requires 582.1607 (–1.99 ppm).

(*E*)-3-(Cinnamyloxy)-1-tritylindolin-2-one (43)

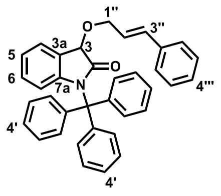

Following **General Procedure F**, 3-diazo-1-tritylindolin-2-one (5.91 g, 14.7 mmol), cinnamyl alcohol (2.2 mL, 17 mmol), $Rh_2(OAc)_4$ (67.4 mg, 0.152 mmol) in anhydrous CH_2Cl_2 (225 mL) gave a crude product which was purified by flash column chromatography (8:92 EtOAc/petrol) to afford the title compound (4.14 g, 56%) as an off-white amorphous solid. **IR** ν_{max} (film) 1724 (C=O), 1609, 1597 (C=C), 1115, 1084 (C-O); **^{1}H NMR (500 MHz, $CDCl_3$)** δ **4.33** (1H, ddd, *J* 12.2, 6.5, 1.4, OC(1``)$H^A$*$H^B$*), **4.46** (1H, ddd, *J* 12.2, 5.9, 1.5, OC(1``)*H^A*H^B), **5.07** (1H, s, C(3)*H*), **6.26–6.30** (1H, m, ArC(7)*H*), **6.33** (1H, app dt, *J* 15.9, 6.2, C(2``)*H*), **6.65** (1H, dd, *J* 15.9, 1.6, C(3``)*H*), **6.89–6.99** (m, 2H, ArC(5,6)*H*), **7.18–7.26** (4H, m, ArC(4`,4```)*H*), **7.26–7.30** (6H, m, ArC(3`,5`)*H*), **7.30–7.35** (2H, m, ArC(3```,5```)*H*), **7.37** (1H, app dt, *J* 7.0, 1.3, ArC(4)*H*), **7.38–7.42** (m, 2H, ArC(2```,6```)*H*), **7.45–7.53** (m, 6H, ArC(2`,6`)*H*); **^{13}C{^{1}H} NMR (126 MHz, $CDCl_3$)** δ **69.8** (O*C*(1``)$H_2$), **74.5** (N*C*$Ph_3$), **75.0** (*C*(3)H), **116.1** (Ar*C*(7)H), **122.4** (Ar*C*(6)H), **124.7** (Ar*C*(4)H), **125.6** (*C*(2``)H), **126.0** (Ar*C*(3a)), **126.7** (Ar*C*(2```,6```)H), **127.0** (Ar*C*(4`)H), **127.8** (Ar*C*(3`,5`)H), **127.9** (Ar*C*(4```)H), **128.4** (Ar*C*(5)H), **128.6** (Ar*C*(3```,5```)H), **129.3** (Ar*C*(2`,6`)H), **133.5** (*C*(3``)H), **136.7** (Ar*C*(1```)), **142.0** (Ar*C*(1`)), **144.1** (Ar*C*(7a)), **176.9** (*C*=O); **HRMS** (ESI$^+$) $C_{36}H_{29}NO_2Na$ $[M+Na]^+$ found 530.2086, requires 530.2091 (−0.9 ppm).

(*E*)-3-{[3-(4-methoxyphenyl)allyl]oxy}-1-tritylindolin-2-one (S111)

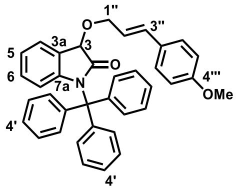

Following **General Procedure F**, 3-diazo-1-tritylindolin-2-one (2.408 g, 5.998 mmol), (*E*)-3-(4-methoxyphenyl)prop-2-en-1-ol (1.125 g, 6.851 mmol), $Rh_2(OAc)_4$ (13.2 mg, 0.0299 mmol) in anhydrous CH_2Cl_2 (38 mL) gave a crude product which was purified by flash column chromatography (1:6:14 EtOAc/CH_2Cl_2/petrol), then the product-containing fractions were combined. Most of the CH_2Cl_2 was removed under reduced pressure (380 mbar, 40 °C) and the resulting suspension was left to stand overnight at rt. The precipitate was filtered off then washed with EtOAc and Et_2O to give the title compound as a pale-orange solid (1.630 g, 51%). **IR** ν_{max} (film) 1724 (C=O), 1607, 1597 (C=C), 1124, 1026 (C-O); **^{1}H NMR (500 MHz, CDCl$_3$)** δ **3.81** (3H, s, OC*H*$_3$), **4.28** (1H, dd, *J* 11.9, 6.8, OC(1``)H$^A$*H*$^B$), **4.41** (1H, dd, *J* 12.0, 6.0, OC(1``)*H*^{A}H^B), **5.03** (1H, s, C(3)*H*), **6.17** (1H, app dt, *J* 15.8, 6.4, C(2``)*H*), **6.26** (1H, d, *J* 7.9, ArC(7)*H*), **6.57** (1H, d, *J* 15.9, C(3``)*H*), **6.85** (2H, d, *J* 8.2, ArC(3```, 5```)*H*), **6.87–6.97** (2H, m, ArC(5, 6)*H*), **7.17–7.22** (3H, m, ArC(4`)*H*), **7.21–7.30** (6H, m, ArC(3`, 5`)*H*), **7.32** (2H, d, *J* 8.3, ArC(2```, 6```)*H*), **7.35** (1H, d, *J* 7.3, ArC(4)*H*), **7.46** (6H, d, *J* 7.7, ArC(2`,5`)*H*); **^{13}C{^{1}H} NMR (126 MHz, CDCl$_3$)** δ **55.4** (O*C*H$_3$), **70.1** (O*C*(1``)H$_2$), **74.5** (N*C*Ph$_3$), **74.9** (*C*(3)H), **114.1** (Ar*C*(3```,5```)), **116.1** (Ar*C*(7)H), **122.4** (Ar*C*(6)H), **123.3** (*C*(2``)H), **124.7** (Ar*C*(4)H), **126.1** (Ar*C*(3a)), **127.0** (Ar*C*(4`)H), **127.8** (Ar*C*(3`,5`)H), **127.9** (Ar*C*(2```,6```)H), **128.3** (Ar*C*(5)H), **129.4** (Ar*C*(2`,6`)H), **129.5** (Ar*C*(1```)), **133.4** (*C*(3``)H), **142.1** (Ar*C*(1`)), **144.0** (Ar*C*(7a)), **159.5** (Ar*C*(4```)H), **177.0** (*C*=O); **HRMS** (ESI$^+$) $C_{37}H_{31}NO_3Na$ [M+Na]$^+$ found 560.2189, requires 560.2196 (−1.2 ppm).

(*E*)-3-{[3-(4-(trifluoromethyl)phenyl)allyl]oxy}-1-tritylindolin-2-one (S112)

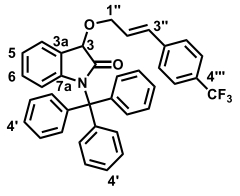

Following **General Procedure F**, 3-diazo-1-tritylindolin-2-one (1.61 g, 4.01 mmol), (*E*)-3-(4-(trifluoromethyl)phenyl)prop-2-en-1-ol (0.893 g, 4.42 mmol), $Rh_2(OAc)_4$ (16.0 mg, 0.0362 mmol) in anhydrous CH_2Cl_2 (60 mL) gave a crude product which was purified by flash column chromatography (10:90 EtOAc/petrol) to afford the title compound (0.981 g, 42%) as a yellow oil. **IR** ν_{max} (film) 1726 (C=O), 1611, 1597 (C=C), 1119 (C-O), 1067 (C-F); **^{1}H NMR (500 MHz, CDCl$_3$)** δ **4.28** (1H, ddd, *J* 12.8, 6.1, 1.5, OC(1``)H$^A$*H*$^B$), **4.46** (1H, ddd, *J* 12.7, 5.7, 1.5, OC(1``)*H*^{A}H^B), **5.08** (1H, s, C(3)*H*), **6.30** (1H, d, *J* 7.2, ArC(7)*H*), **6.39** (1H, app dt, *J* 15.9, 5.9, C(2``)*H*), **6.65** (1H, d, *J* 15.9, C(3``)*H*), **6.88–7.01** (2H, m, ArC(5, 6)*H*), **7.18–7.24** (3H, m, ArC(4`)*H*), **7.22–7.31** (6H, m, ArC(3`, 5`)*H*), **7.37** (1H, d, *J* 6.9, ArC(4)*H*), **7.43–7.50** (8H, m, ArC(2`, 6`, 2```, 6```)*H*), **7.57** (2H, d, *J* 8.0, ArC(3```, 5```)*H*); **^{13}C{^{1}H} NMR (126 MHz, CDCl$_3$)** δ **69.3** (O*C*(1``)H$_2$), **74.6** (N*C*Ph$_3$), **75.4** (*C*(3)H), **116.2** (Ar*C*(7)H), **122.5** (Ar*C*(5)H), **124.3** (q, $^1J_{CF}$ 271.8, *C*F$_3$), **124.6** (Ar*C*(4)H), **125.6** (q, $^3J_{CF}$ 3.8, Ar*C*(3```,5```)H), **125.7** (Ar*C*(3a)), **126.8** (Ar*C*(2```,6```)H), **127.1** (Ar*C*(4`)H), **127.8** (Ar*C*(3`,5`)H), **128.4** (*C*(2)H), **128.5** (Ar*C*(6)H), **129.3** (Ar*C*(2`)H), **129.6** (q, $^2J_{CF}$ 32.3, Ar*C*(4```)CF$_3$), **131.4** (*C*(3``)H), **140.2** (Ar*C*(1```)), **142.0** (Ar*C*(1`)), **144.1** (Ar*C*(7a)), **176.8** (*C*=O), **^{19}F{^{1}H} NMR (471 MHz, CDCl$_3$)** δ **−62.4** (C*F*$_3$), **HRMS** (ESI$^+$) $C_{37}H_{28}F_3NO_2Na$ $[M+Na]^+$ found 598.1955, requires 598.1964 (−0.9 ppm).

(*E*)-3-{[3-(4-Fluorophenyl)allyl]oxy}-1-tritylindolin-2-one (S113)

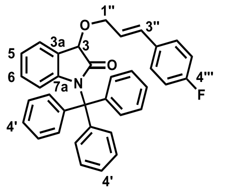

Following **General Procedure F**, (*E*)-3-(4-fluorophenyl)prop-2-en-1-ol (0.502 g, 3.30 mmol), dirhodium tetraacetate (6.7 mg, 0.015 mmol), anhydrous CH_2Cl_2 (19 mL) and 3-diazo-1-tritylindolin-2-one (1.201 g, 2.992 mmol) gave, after purification by flash column chromatography (10:90 EtOAc/petrol), the title compound as an off-white amorphous solid (0.374 g, 24%). **IR** ν_{max} (film) 3086, 3055, 3034, 2926, 2859 (C-H), 1726 (C=O), 1609, 1601 (C=C), 1117, 1092 (C-O); **^{1}H NMR (500 MHz, $CDCl_3$)** δ **4.28** (dd, *J* 12.2, 6.5, 1H, OC(1``)$H^AH^B$), **4.43** (dd, *J* 12.1, 5.9, 1H, OC(1``)H^AH^B), **5.05** (s, 1H, C(3)*H*), **6.22** (dt, *J* 15.9, 6.1, C(2``)*H*), **6.27** (d, *J* 7.9, 1H, ArC(7)*H*), **6.59** (d, *J* 15.9, 1H, C(3``)*H*), **6.86–6.97** (m, 2H, ArC(5,6)*H*), 7.00 (dd, *J* 8.7, 2H, ArC(3```,5```)*H*), **7.16–7.24** (m, 3H, ArC(4`)*H*), **7.23–7.30** (m, 6H, ArC(3`,5`)*H*), **7.31–7.38** (m, 3H, ArC(4,2```,6```)*H*), **7.44–7.51** (m, 6H, ArC(2`,6`)*H*); **^{13}C{^{1}H} NMR (126 MHz, $CDCl_3$)** δ **69.7** (O*C*(1``)H_2), **74.5** (N*C*Ph_3), **75.1** (*C*(3)H), **115.6** (d, *J* 21.6, ArC(3```)H), **116.1** (Ar*C*(7)H), **122.5** (Ar*C*(5)H), **124.6** (Ar*C*(4)H), **125.3** (d, *J* 2.3, *C*(2``)H), **125.9** (Ar*C*(3a)), **127.0** (Ar*C*(4`)H), **127.8** (Ar*C*(3`,5`)H), **128.2** (d, *J* 8.0, Ar*C*(2```)H), **128.4** (Ar*C*(6)H), **129.3** (Ar*C*(2`,6`)H), **132.2** (*C*(3``)H), **132.9** (d, *J* 3.3, Ar*C*(1```)), **142.0** (Ar*C*(1`)), **144.0** (Ar*C*(7a)), **162.5** (d, *J* 247.0, Ar*C*(4```)F), **176.9** (*C*=O); **^{19}F{^{1}H} NMR (470 MHz, $CDCl_3$)** δ –114.2 (ArC(4```)*F*); **HRMS** (ESI^+) $C_{36}H_{28}FNO_2Na$ $[M+Na]^+$ found 548.1982, requires 548.1996 (–2.6 ppm)

(*E*)-3-{[3-(2-Methoxyphenyl)allyl]oxy}-1-tritylindolin-2-one (S114)

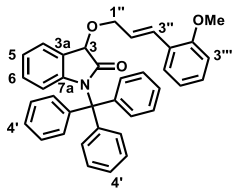

Following **General Procedure F**, 3-diazo-1-tritylindolin-2-one (0.597 g, 1.49 mmol), (*E*)-3-(4-methoxyphenyl)prop-2-en-1-ol (0.268 g, 1.63 mmol), $Rh_2(OAc)_4$ (3.5 mg, 0.0078 mmol) in anhydrous CH_2Cl_2 (9 mL) gave a crude product which was purified by flash column chromatography (10:90 → 15:85 EtOAc/petrol) to afford the title compound (0.565 g, 71%) as a yellow amorphous solid. **IR** ν_{max} (film) 3057, 3032, 2920, 2849, (C-H), 1734 (C=O), 1609, 1597 (C=C). 1030 (C-O); **^{1}H NMR (500 MHz, $CDCl_3$)** δ **3.86** (3H, s, OC*H*$_3$), **4.39** (1H, dd, *J* 12.2, 6.7, OC(1``)$H^AH^B$), **4.51** (1H, dd, *J* 12.1, 5.9, OC(1``)H^AH^B), **5.09** (1H, s, C(3)*H*), **6.30** (1H, d, *J* 7.9, ArC(7)*H*), **6.39** (1H, app dt, *J* 16.0, 6.3, C(2``)*H*), **6.89** (1H, d, *J* 8.3, ArC(3```)*H*), **6.90–7.01** (3H, m, ArC(5,6,5```)*H*), **7.03** (1H, d, *J* 16.1, C(3``)*H*), **7.16–7.35** (10H, m, ArC(3`,4`,5`,4```)*H*), **7.41** (1H, d, *J* 7.1, ArC(4)*H*), **7.45–7.56** (7H, m, ArC(2`,5`,6```)*H*). **^{13}C NMR (126 MHz, $CDCl_3$)** δ **55.5** (O*C*H$_3$), **70.5** (O*C*(1``)H$_2$), **74.4** (N*C*Ph$_3$), **74.9** (*C*(3)H), **110.9** (Ar*C*(3```)H), **116.0** (Ar*C*(7)H), **120.7** (Ar*C*(6)H), **122.3** (Ar*C*(5)H), **124.7** (Ar*C*(4)H), **125.6** (Ar*C*(1```)H), **126.1** (*C*(2``)H), **126.1** (Ar*C*(3a)), **127.0** (Ar*C*(4`)H), **127.1** (Ar*C*(6```)H), **127.8** (Ar*C*(3`,5`)H), **128.2** (Ar*C*(5```)H), **128.5** (*C*(3``)H), **128.9** (Ar*C*(4```)H), **129.3** (Ar*C*(2`,6`)H), **142.0** (Ar*C*(1`)), **144.0** (Ar*C*(7a)), **156.9** (Ar*C*(2```)OMe), **177.0** (*C*=O). **HRMS** (ESI$^+$) $C_{37}H_{31}NO_3Na$ $[M+Na]^+$ found 560.2192, requires 560.2196 (−0.7 ppm).

(*E*)-3-{[3-(Naphthalen-1-yl)allyl]oxy}-1-tritylindolin-2-one (S115)

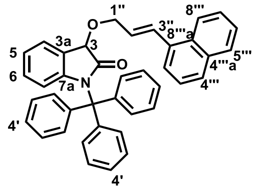

Following **General Procedure F**, 3-diazo-1-tritylindolin-2-one (504 mg, 1.26 mmol), (*E*)-3-(1-naphthyl)prop-2-en-1-ol (255 mg, 1.38 mmol), $Rh_2(OAc)_4$ (2.8 mg, 0.0063 mmol) in anhydrous CH_2Cl_2 (8 mL) gave a crude product which was purified by flash column chromatography (10:90 EtOAc/petrol) to afford the title compound (516 mg, 73%) as an off-white amorphous solid. **IR** ν_{max} (film) 3057, 2932, 2860 (C-H), 1718 (C=O), 1609, 1595 (C=C), 1119 (C-O); **^{1}H NMR (500 MHz, $CDCl_3$)** δ **4.43** (1H, ddd, *J* 12.4, 6.4, 1.5, OC(1``)$H^A$H$^B$), **4.55** (1H, ddd, *J* 12.4, 5.9, 1.6, OC(1``)H^AH^B), **5.13** (1H, s, C(3)*H*), **6.29** (1H, dd, *J* 7.6, 1.3, ArC(7)*H*), **6.35** (1H, app dt, *J* 15.6, 6.1, C(2``)*H*), **6.90–7.00** (2H, m, ArC(5,6)*H*), **7.18–7.25** (3H, m, ArC(4`)*H*), **7.25–7.32** (6H, m, ArC(3`,5`)*H*), **7.37–7.42** (2H, m, C(3``)*H*, ArC(4)*H*), **7.45** (1H, dd, *J* 8.2, 7.2 ArC(3```)*H*), **7.47–7.55** (8H, m, ArC(2`,6`,6```,7```)*H*), **7.56–7.63** (1H, m, ArC(2```)*H*), **7.79** (1H, d, *J* 8.2, ArC(4```)*H*), **7.82–7.88** (1H, m, ArC(5```)*H*), **8.04–8.14** (1H m, ArC(8```)*H*). **^{13}C NMR (126 MHz, $CDCl_3$)** δ **70.0** (O*C*(1``)H_2), **74.6** (N*C*Ph_3), **75.2** (*C*(3)H), **116.1** (Ar*C*(7)H), **122.5** (Ar*C*(5)H), **124.0** (Ar*C*(8```)H), **124.2** (Ar*C*(2```)H), **124.7** (Ar*C*(4)H), **125.7** (Ar*C*(3```)H), **125.9** (Ar*C*(6```)H), **126.0** (Ar*C*(3a)), **126.2** (Ar*C*(7```)H), **127.0** (Ar*C*(4`)H), **127.8** (Ar*C*(3`,5`)H), **128.2** (Ar*C*(4```)H), **128.4** (Ar*C*(6)H), **128.6** (Ar*C*(5```)H), **128.8** (*C*(2``)H), **129.4** (Ar*C*(2`,6`)H), **130.5** (*C*(3``)H), **131.3** (Ar*C*(8```a)), **133.7** (Ar*C*(4```a)), **134.5** (Ar*C*(1```)), **142.1** (Ar*C*(1`)), **144.1** (Ar*C*(7a)), **177.0** (*C*=O). **HRMS** (ESI$^+$) $C_{40}H_{31}NO_2Na$ $[M+Na]^+$ found 580.2247, requires 580.2247 (0 ppm).

(*E*)-3-{[3-(Naphthalen-2-yl)allyl]oxy}-1-tritylindolin-2-one (S116)

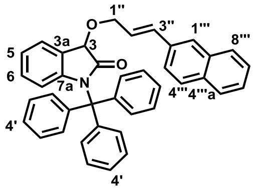

Following **General Procedure F**, 3-diazo-1-tritylindolin-2-one (0.502 g, 1.25 mmol), (*E*)-3-(2-naphthyl)prop-2-en-1-ol (0.253 g, 1.37 mmol), $Rh_2(OAc)_4$ (2.9 mg, 0.0066 mol) in anhydrous CH_2Cl_2 (7.5 mL) gave a crude product which was purified by flash column chromatography (10:90 EtOAc/petrol) to afford the title compound (0.523 g, 75%) as an amorphous yellow solid. **IR** ν_{max} (solid) 1732 (C=O), 1605, 1593 (C=C), 1117, 1088 (C-O); **^{1}H NMR (500 MHz, $CDCl_3$)** δ **4.42** (1H, dd, *J* 12.3, 6.5, OC(1``)$H^AH^B$), **4.57** (1H, dd, *J* 12.3, 5.9, OC(1``)H^AH^B), **5.14** (1H, s, C(3)*H*), **6.34** (1H, d, *J* 7.9, ArC(7)*H*), **6.50** (1H, app dt, *J* 15.9, 6.2, C(2``)*H*), **6.85** (1H, d, *J* 15.8, C(3``)*H*), **6.97** (1H, app t, *J* 7.8, ArC(6)*H*), **7.00** (1H, app t, *J* 7.4, ArC(5)*H*), **7.22–7.29** (3H, m, ArC(4`)*H*), **7.31** (6H, app t, *J* 7.6, ArC(3`,5`)*H*), **7.44** (1H, d, *J* 7.1, ArC(4)*H*), **7.45 – 7.51** (2H, m, ArC(6```,7```)*H*), **7.54** (6H, d, *J* 7.8, ArC(2`,6')*H*), **7.65** (1H, d, *J* 8.4, ArC(3```)*H*), **7.77** (1H, s, ArC(1```)*H*), **7.79–7.89** (3H, m, ArC(4```,5```,8```)*H*); **^{13}C{^{1}H} NMR (126 MHz, $CDCl_3$)** δ **69.9** (O*C*(1``)H_2), **74.5** (N*C*Ph_3), **75.1** (*C*(3)H), **116.0** (Ar*C*(7)H), **122.4** (Ar*C*(5)H), **123.7** (Ar*C*(3```)H), **124.6** (Ar*C*(4)H), **126.0** (Ar*C*(3a), *C*(2``)H), **126.0** (Ar*C*(6```)H), **126.3** (Ar*C*(7)H), **126.7** (Ar*C*(1```)H), **127.0** (Ar*C*(4`)H), **127.7** (Ar*C*(8```)H), **127.8** (Ar*C*(3`,5`)H), **128.1** (Ar*C*(5```)H), **128.3** (Ar*C*(4```)H), **128.5** (Ar*C*(6)H), **129.3** (Ar*C*(2`,6`)H), **133.1** (Ar*C*(4```a)), **133.4** (*C*(3``)H), **133.6** (Ar*C*(8```a)), **134.1** (Ar*C*(2```)), **142.0** (Ar*C*(1`)), **144.0** (Ar*C*(7a)), **176.9** (*C*=O); **HRMS** (ESI^+) $C_{40}H_{31}NO_2Na$ $[M+Na]^+$ found 580.2240, requires 580.2247 (−1.2 ppm).

(*E*)-3-{[3-(Thiophen-3-yl)allyl]oxy}-1-tritylindolin-2-one (S117)

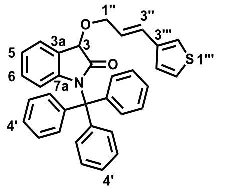

Following **General Procedure F**, 3-diazo-1-tritylindolin-2-one (0.665 g, 1.66 mmol), (*E*)-3-(thiophen-3-yl)prop-2-en-1-ol (0.189 g, 1.81 mmol), $Rh_2(OAc)_4$ (3.7 mg, 0.0083 mmol) in anhydrous CH_2Cl_2 (10 mL) gave a crude product which was purified by flash column chromatography (10:90 → 15:85 EtOAc/petrol) to afford the title compound (405 mg, 47%) as a yellow amorphous solid. **IR** ν_{max} (film) 1724 (C=O), 1115, 1086 (C-O); **^{1}H NMR (500 MHz, $CDCl_3$)** δ **4.27** (1H, ddd, *J* 12.1, 6.6, 1.4, OC(1``)$H^AH^B$), **4.41** (1H, ddd, *J* 12.1, 6.0, 1.5, OC(1``)H^AH^B), **5.04** (1H, s, C(3)*H*), **6.16** (1H, app dt, *J* 15.9, 6.3, C(2``)*H*), **6.26** (1H, dd, *J* 7.6, 1.3, ArC(7)*H*), **6.64** (1H, d, *J* 15.8, C(3``)*H*), **6.89–6.98** (2H, m, ArC(5,6)*H*), **7.16** (1H, dd, *J* 3.0, 1.2, ArC(2```)*H*), **7.18–7.23** (4H, m, ArC(4`,4```)*H*), **7.23–7.29** (7H, m, ArC(3`,5`,5```)*H*), 7.34 (1H, dt, *J* 7.0, 1.4, ArC(4)*H*), **7.44–7.50** (6H, m, ArC(2`,6`)*H*). **^{13}C NMR (126 MHz, $CDCl_3$)** δ **69.8** (O*C*(1``)H_2), **74.5** (N*C*Ph_3), **75.0** (*C*(3)H), **116.1** (Ar*C*(7)H), **122.4** (Ar*C*(5)H), **122.7** (Ar*C*(2```)H), **124.6** (Ar*C*(4)H), **125.2** (Ar*C*(4```)H), **125.4** (*C*(2``)H), **126.0** (Ar*C*(3a)), **126.1** (Ar*C*(5```)H), **127.0** (Ar*C*(4`)H), **127.8** (*C*(3``)H), **127.8** (Ar*C*(3`,5`)H), **128.4** (Ar*C*(6)H), **129.4** (Ar*C*(2`,6`)H), **139.4** (Ar*C*(3```)H), **142.0** (Ar*C*(1`)), **144.0** (Ar*C*(7a)), **176.9** (*C*=O). **HRMS** (ESI^+) $C_{34}H_{27}NO_2SNa$ $[M+Na]^+$ found 536.1650, requires 536.1655 (−0.9 ppm).

(*E*)-3-[(2-Methyl-3-phenylallyl)oxy)-1-tritylindolin-2-one (S118)

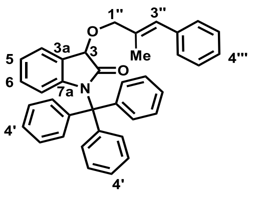

Following **General Procedure F**, 3-diazo-1-tritylindolin-2-one (2.01 g, 5.01 mmol), (*E*)-2-methyl-3-phenylprop-2-en-1-ol (0.80 mL, 5.56 mmol), $Rh_2(OAc)_4$ (11.5 mg, 0.0260 mmol), anhydrous CH_2Cl_2 (30 mL) and gave, after purification by flash column chromatography (10:90 EtOAc/petrol), the title compound as an off-white amorphous solid (2.48 g, 95%). **IR** ν_{max} (solid) 1728 (C=O), 1607, 1597 (C=C), 1105, 1084 (C-O); **^{1}H NMR (500 MHz, CDCl$_3$)** δ **1.94** (3H, d, *J* 1.4, C*H*$_3$), **4.20** (1H, dd, *J* 11.7, 1.2, OC(1``)*H*$^A$H$^B$), **4.29** (1H, dd, *J* 11.8, 1.3, OC(1``)H^A*H*^{B}), **5.04** (1H, s, C(3)*H*), **6.27** (1H, dd, *J* 7.6, 1.4, ArC(7)*H*), **6.55** (1H, s, C(3``)*H*), **6.88–6.99** (2H, m, ArC(5,6)*H*), **7.17–7.23** (4H, m, ArC(4`,4```)*H*), **7.23–7.30** (8H, m, ArC(3`,5`,3```,5```)*H*), **7.31–7.33** (1H, m, ArC(4)*H*), **7.33–7.37** (2H, m, ArC(2```,6```)*H*), **7.43–7.53** (6H, m, ArC(2`,6`)*H*); **^{13}C{^{1}H} NMR (126 MHz, CDCl$_3$)** δ **15.9** (*C*H$_3$), **74.5** (N*C*Ph$_3$), **74.8** (*C*(3)H), **75.3** (O*C*(1``)H$_2$), **116.1** (Ar*C*(7)H), **122.4** (Ar*C*(6)H), **124.7** (Ar*C*(4)H), **126.1** (Ar*C*(3a)), **126.7** (Ar*C*(4```)H), **127.0** (Ar*C*(4`)H), **127.8** (Ar*C*(3`,5`)H), **128.2** (*C*(3``)H), **128.2** (Ar*C*(2```,6```)H), **128.4** (Ar*C*(5)H), **129.1** (Ar*C*(3```,5```)H), **129.3** (Ar*C*(2`,6`)H), **134.7** (*C*(2``)), **137.5** (Ar*C*(1```)), **142.1** (Ar*C*(1`)), **144.1** (Ar*C*(7a)), **177.0** (*C*=O); **HRMS** (ESI$^+$) $C_{37}H_{31}NO_2Na$ [M+Na]$^+$ found 544.2240, requires 544.2247 (−1.3 ppm).

4-Chloro-3-(cinnamyloxy)-1-tritylindolin-2-one (S119)

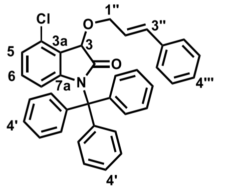

Following **General Procedure F**, (*E*)-cinnamyl alcohol (0.29 mL, 2.2 mmol), dirhodium tetraacetate (4.3 mg, 0.0097 mmol), anhydrous CH_2Cl_2 (12.5 mL) and 4-chloro-3-diazo-1-tritylindolin-2-one (0.865 g, 1.98 mmol) gave, after purification by flash column chromatography (15:5:1 petrol/EtOAc/CH_2Cl_2), the title compound as an off-white amorphous solid (0.789 g, 74%). **IR** ν_{max} (film) 3084, 3057, 3024 (C-H), 1728 (C=O), 1601, 1589 (C=C), 1141, 1107 (C-O); **^{1}H NMR (500 MHz, $CDCl_3$)** δ **4.41** (1H, dd, *J* 1.9, 6.8, OC(1``)$H^A$H$^B$), **4.53** (1H, dd, *J* 1.8, 6.0, 1H, OC(1``)H^AH^B), **4.99** (1H, s, C(3)*H*), **6.15** (1H, d, *J* 8.0, ArC(7)*H*), **6.39** (1H, dt, *J* 15.9, 6.4, C(2``)*H*), **6.66** (1H, d, *J* 15.9, C(3``)*H*), **6.85** (1H, dd, *J* 8.1, ArC(6)*H*), **6.91** (1H, d, *J* 8.1, ArC(5)*H*), **7.19–7.29** (10H, m, ArC(3`,4`,4```)*H*), **7.31** (2H, dd, *J* 7.6, ArC(3```)*H*), **7.39** (2H, d, *J* 7.6, ArC(2```)*H*), **7.44** (6H, d, *J* 8.0, ArC(2`,6`)*H*); **^{13}C{^{1}H} NMR (126 MHz, $CDCl_3$)** δ **70.6** (O*C*(1``)$H_2$), **74.3** (*C*(3)*H*), **74.7** (N*C*$Ph_3$), **114.5** (Ar*C*(7)H), **123.2** (Ar*C*(5)H), **123.5** (Ar*C*(3a)), **125.3** (*C*(2``)H), **126.8** (Ar*C*(2```)H), **127.2** (Ar*C*(4`)H), **127.9** (Ar*C*(2`,5`)H), **128.6** (Ar*C*(3```)H), **129.3** (Ar*C*(2`,6`)H), **129.7** (Ar*C*(6)H), **131.9** (Ar*C*(4)), **134.0** (*C*(3``)H), **136.7** (Ar*C*(1``)), **141.7** (Ar*C*(1`)), **145.9** (Ar*C*(7a)), **175.4** (*C*(2)); **HRMS** (ESI$^+$) $C_{36}H_{28}NO_2{}^{35}ClNa$ [M+Na]$^+$ found 564.1678, requires 564.1701 (–4.0 ppm).

3-(Cinnamyloxy)-5-methoxy-1-tritylindolin-2-one (S120)

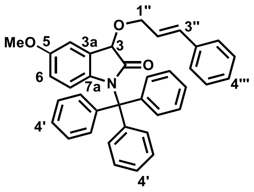

Following **General Procedure G**, (*E*)-cinnamyl alcohol (0.43 mL, 3.3 mmol), dirhodium tetraacetate (6.5 mg, 0.015 mmol), anhydrous CH_2Cl_2 (19 mL) and 3-diazo-5-methoxy-1-tritylindolin-2-one (1.295 g, 3.001 mmol) gave, after purification by flash column chromatography (50:50 → 0:100 petrol/ CH_2Cl_2), the title compound as an off-white amorphous solid (1.308 g, 81%). **IR** ν_{max} (film) 3084, 3057, 3024 (C-H), 1722 (C=O), 1595 (C=C), 1126, 1086 (C-O); **^{1}H NMR (500 MHz, $CDCl_3$)** δ **3.70** (3H, s, OCH_3), **4.27** (1H, ddd, *J* 12.2, 6.6, 1.4, OC(1``)$H^A$H$^B$), **4.42** (1H, ddd, *J* 12.1, 5.9, 1.5, OC(1``)H^AH^B), **5.04** (1H, s, C(3)*H*), **6.16** (1H, d, *J* 8.9, ArC(7)*H*), **6.31** (1H, dt, *J* 15.9, 6.2, C(2``)*H*), **6.46** (1H, ddd, *J* 8.9, 2.8, 0.7, ArC(6)*H*), **6.63** (1H, d, *J* 15.9, C(3``)*H*), **6.95** (1H, dd, *J* 2.8, 1.1, ArC(4)*H*), **7.18–7.23** (3H, m, ArC(4`)*H*), **7.22–7.30** (7H, m, ArC(3`,4```)*H*), **7.29–7.34** (2H, m, ArC(3```)*H*), **7.36–7.41** (2H, m, ArC(2```)*H*), **7.43–7.50** (6H, m, ArC(2`,6`)*H*); **^{13}C{^{1}H} NMR (126 MHz, $CDCl_3$)** δ **55.7** (O*C*H_3), **69.7** (O*C*(1``)$H_2$), **74.5** (N*C*$Ph_3$), **75.3** (*C*(3)H), **110.9** (Ar*C*(4)H), **113.5** (Ar*C*(6)H), **116.8** (Ar*C*(7)H), **125.6** (*C*(2``)H), **126.7** (Ar*C*(2```)H), **127.0** (Ar*C*(4`)H), **127.2** (Ar*C*(3a)), **127.8** (Ar*C*(3`,5`)H), **127.9** (Ar*C*(4```)H), **128.7** (Ar*C*(3```)H), **129.3** (Ar*C*(2`,6`)H), **133.5** (*C*(3``)H), **136.7** (Ar*C*(1```)), **137.2** (Ar*C*(7a)), **142.1** (Ar*C*(1`)), **155.5** (Ar*C*(5)), **176.7** (*C*=O); **HRMS** (ESI^+) $C_{37}H_{31}NO_3Na$ $[M+Na]^+$ found 560.2178, requires 560.2196 (–3.2 ppm).

6-Bromo-3-(cinnamyloxy)-1-tritylindolin-2-one (S121)

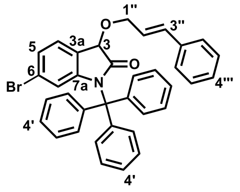

Following **General Procedure F**, (*E*)-cinnamyl alcohol (0.29 mL, 2.2 mmol), dirhodium tetraacetate (4.4 mg, 0.010 mmol), anhydrous CH_2Cl_2 (12.5 mL) and 6-bromo-3-diazo-1-tritylindolin-2-one (0.954 g, 1.99 mmol) gave, after purification by flash column chromatography (15:5:1 petrol/EtOAc/CH_2Cl_2), the title compound as an off-white amorphous solid (0.845 g, 72%). **IR** ν_{max} (film) 3084, 3057, 3024 (C-H), 1732 (C=O), 1607, 1578 (C=C), 1111, 1086 (C-O); **^{1}H NMR (500 MHz, $CDCl_3$)** δ **4.30** (1H, ddd, *J* 12.2, 6.6, 1.4, OC(1``)$H^AH^B$), **4.44** (1H, ddd, *J* 12.2, 5.9, 1.5, OC(1``)H^AH^B), **4.96** (1H, d, *J* 1.1, C(3)*H*), **6.29** (1H, dt, *J* 15.9, 6.3, C(2``)*H*), **6.32** (1H, d, *J* 1.6, ArC(7)*H*), **6.63** (1H, d, *J* 16.0, C(3``)*H*), **7.09** (1H, dd, *J* 7.9, 1.6, ArC(5)*H*), **7.20** (1H, dd, *J* 7.9, 1.1, ArC(4)*H*), **7.22–7.35** (12H, m, ArC(3`,4`,5`,3```,4```,5```)*H*), **7.36–7.40** (2H, m, ArC(2```)*H*), **7.41–7.46** (6H, m, ArC(2`,6`)*H*); **^{13}C{^{1}H} NMR (126 MHz, $CDCl_3$)** δ **70.0** (O*C*(1``)$H_2$), **74.4** (*C*(3)H), **74.8** (N*C*$Ph_3$), **119.1** (Ar*C*(7)), **122.1** (Ar*C*(6)), **124.9** (Ar*C*(3a)), **125.3** (*C*(2``)H), **125.4** (Ar*C*(5)H), **125.8** (Ar*C*(4)H), **126.7** (Ar*C*(2```)H), **127.3** (Ar*C*(4`)H), **128.0** (Ar*C*(3`,5`)H), **128.0** (Ar*C*(4```)H), **128.7** (Ar*C*(3```)H), **129.3** (Ar*C*(2`,6`)H), **133.8** (*C*(3``)H), **136.6** (Ar*C*(1```)), **141.6** (Ar*C*(1`)), **145.3** (Ar*C*(7a)), **176.6** (*C*=O); **HRMS** (ESI$^+$) $C_{36}H_{28}NO_2{}^{79}BrNa$ $[M+Na]^+$ found 608.1186, requires 608.1201 (–2.5 ppm).

(*E*)-3-((3-(4-fluorophenyl)but-2-en-1-yl)oxy)-1-methylindolin-2-one (S13)

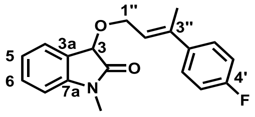

Following **General Procedure F**, 3-diazo-1-methylindolin-2-one (1.71 g, 9.90 mmol), 3-(4-fluorophenyl)but-2-en-1-ol (1.51 g, 9.00 mmol), $Rh_2(OAc)_4$ (19.9 mg, 0.045 mmol) in anhydrous CH_2Cl_2 (180 mL) gave a crude product which was purified by flash column chromatography (500-X:X starting from X = 5 increasing by 5 each 500 mL, PhMe/EtOAc) to afford the title compound (1.68 g, 5.39 mmol, 60%) as a yellow oil.
IR ν_{max}(film) 3055, 2936, 2876 (C-H), 1713 (C=O), 1614 , 1508, 1493, 1470, 1373, 1101, 1090, 1042 (C-O), 829, 820, 750; **^{1}H NMR** (400 MHz, $CDCl_3$) δ_H **2.01** (dt, *J* = 1.4, 0.8 Hz, 3H, C(4")H_3), **3.16** (s, 3H, N-CH_3), **4.47** (ddq, *J* = 11.8, 7.3, 0.8 Hz, 1H, C(1")$H^A$$H^B$), **4.54** (ddq, *J* = 11.8, 6.4, 0.8 Hz, 1H, C(1")$H^A$$H^B$), **4.97** (s, 1H, C(3)*H*), **5.94** (ddq, *J* = 7.3, 6.4, 1.4 Hz, 1H, C(2")*H*), **6.81** (d, *J* = 7.8 Hz, 1H, ArC(7)*H*), **6.94** – **7.03** (2H, m, ArC(3',5')*H*), **7.10** (app td, *J* = 7.5, 1.0 Hz, 1H, ArC(5)*H*), **7.30** – **7.39** (m, 3H, ArC(6)*H*, ArC(2',6')*H*), **7.39** – **7.43** (m, 1H, ArC(4)*H*); **^{13}C{^{1}H} NMR** (100 MHz, $CDCl_3$) δ **16.4** (*C*(4")H_3), **26.2** (N-*C*H_3), **65.7** (*C*(1")H_2), **75.0** (*C*(3)H), **108.5** (Ar*C*(7)H), **115.1** (d, *J* = 21.4 Hz, Ar*C*(3',5')H), **123.1** (Ar*C*(5)H), **123.4** (*C*(2")H), **125.3** (Ar*C*(3a)), **125.4** (Ar*C*(4)H), **127.5** (d, *J* = 8.0 Hz, Ar*C*(2',6')H), **130.1** (Ar*C*(6)H), **138.9** (d, *J* = 3.7 Hz, Ar*C*(1')), **139.0** (*C*(3)), **144.4** (Ar*C*(7a)), **162.4** (d, *J* = 246.1 Hz, Ar*C*(4')F), **175.0** (*C*(2)); **^{19}F{^{1}H} NMR** (377 MHz, $CDCl_3$) δ **−115.41** (s, 1F, ArC(4')*F*); **HRMS** (ESI^+) $C_{19}H_{18}O_2NFNa$ $[M+Na]^+$ found 334.1212, requires 334.1214 (−0.5 ppm).

(*E*)-1-benzyl-3-((3-(4-fluorophenyl)but-2-en-1-yl)oxy)indolin-2-one (S16)

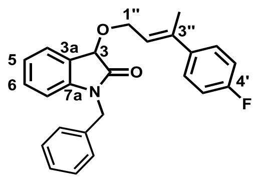

Following **General Procedure F**, 1-benzyl-3-diazoindolin-2-one (2.47 g, 9.90 mmol), 3-(4-fluorophenyl)but-2-en-1-ol (1.51 g, 9.00 mmol), $Rh_2(OAc)_4$ (19.9 mg, 0.045 mmol) in anhydrous CH_2Cl_2 (180 mL) gave a crude product which was purified by flash column chromatography (pure PhMe until elution of red band then 1350:150 → 400:100 PhMe/EtOAc, re-purify in pure CH_2Cl_2) to afford the title compound (1.61 g, 4.16 mmol, 46%) as a yellow oil.

IR ν_{max} (film): 3061, 3032, 2920, 2862 (C-H), 1717 (C=O), 1614, 1603, 1508, 1489, 1466 (C=C), 1360, 1225, 1171, 1163, 1105, 1080, 1009 (C-O), 829, 818, 750; **^{1}H NMR** (400 MHz, $CDCl_3$) δ **2.06** (dd, *J* = 1.4, 0.5 Hz, 3H, C(4”)H_3), **4.52** (dd, *J* = 11.8, 7.1 Hz, 1H, C(1”)H^AH^B), **4.59** (ddq, *J* = 11.8, 6.5, 0.5 Hz, 1H, C(1”)H^AH^B), **4.83** (d, *J* = 15.6 Hz, 1H, NCH^AH^B), **4.92** (d, *J* = 15.6 Hz, 1H, NCH^AH^B), **5.07** (s, 1H, C(3)*H*), **5.98** (ddq, *J* = 7.1, 6.5, 1.4 Hz, 1H, C(2”)*H*), **6.71** (d, *J* = 7.8 Hz, 1H, ArC(7)*H*), **6.97** – **7.04** (m, 2H, ArC(3’,5’)*H*), **7.06** (app td, *J* = 7.6, 1.0 Hz, 1H, ArC(5)*H*), **7.19** – **7.24** (m, 1H, ArC(6)*H*), **7.24** – **7.30** (m, 1H, PhC(4)*H*), **7.30** – **7.33** (m, 4H, ArC(2”’,3”’,5”’,6”’)*H*), **7.35** – **7.41** (m, 2H, ArC(2’,6’)*H*), **7.41** – **7.44** (m, 1H, ArC(4)*H*); **^{13}C{^{1}H} NMR** (100 MHz, $CDCl_3$) δ **16.5** (*C*(4”)H_3), **43.8** (N*C*H_2), **65.8** (*C*(1”)H_2), **75.1** (*C*(3)H), **109.6** (Ar*C*(7)H), **115.2** (d, *J* = 21.3 Hz, Ar*C*(3’,5’)H), **123.1** (Ar*C*(5)H), **123.4** (*C*(2”)H), **125.3** (Ar*C*(3a)), **125.5** (Ar*C*(4)H), **127.4**$_7$ (Ar*C*(2”’,6”’)H), **127.5**$_4$ (d, *J* = 8.0 Hz, Ar*C*(2’,6’)H), **127.9** (Ar*C*(4”’)H), **129.0** (Ar*C*(3”’,5”’)H), **130.0** (Ar*C*(6)H), **135.7** (Ar*C*(1”’)), **138.9** (d, *J* = 3.3 Hz, Ar*C*(1’)), **139.1** (*C*(3”)), **143.5** (Ar*C*(7a)), **162.4** (d, *J* = 246.3 Hz, Ar*C*(4’)F), **175.1** (*C*(2)); **^{19}F{^{1}H} NMR** (377 MHz, $CDCl_3$) δ **−115.37** (s, 1F, ArC(4’)*F*); **HRMS** (ESI$^+$) $C_{25}H_{22}O_2NFNa$ [M+Na]$^+$ found 410.1524, requires 410.1527 (−0.8 ppm).

Ethyl 4-(1-trityloxindol-3-oxy)tiglate (66)

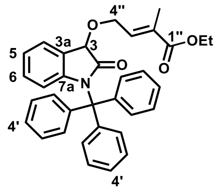

Following **General Procedure F**, 3-diazo-1-trityloxindole (2.23 g, 5.54 mmol), ethyl 4-hydroxytiglate (726 mg, 5.04 mmol), $Rh_2(OAc)_4$ (11.1 mg, 0.025 mmol) in anhydrous CH_2Cl_2 (101 mL, 0.05 M) gave a crude product which was purified by flash column chromatography (30:10:2 → 30:10:4 → 30:10:10 hexane/CH_2Cl_2/EtOAc) to afford the title compound (1.978 g, 76%) as an amorphous yellow solid. **mp** 54 – 56 °C (CH_2Cl_2); **IR** ν_{max} (film) 3057, 2980, 2928, 1728 (C=O), 1711 (C=O), 1611, 1597, 1491, 1477, 1464, 1449 (C=C), 1337, 1306, 1267, 1246, 1186, 1153, 1132, 1098, 1084, 1034 (C-O), 746, 739, 706; **^{1}H NMR** (700 MHz, CD_2Cl_2) δ **1.27** (t, *J* = 7.1 Hz, 3H, CH_2CH_3), **1.72** (ddd, *J* = 1.5, 1.3, 1.1 Hz, 3H, C(2")CH_3), **4.15**$_6$ (q, *J* = 7.1 Hz, 2H, CH_2CH$_3$), **4.16**$_2$ (ddq, *J* = 13.6, 6.3, 1.1 Hz, 1H, C(4")H^AH^B), **4.29** (ddq, *J* = 13.6, 5.7, 1.3 Hz, 1H, C(4")H^AH^B), **5.00** (s, 1H, C(3)*H*), **6.28** (app d, *J* = 7.6 Hz, 1H, ArC(7)*H*), **6.76** (ddq, *J* = 6.3, 5.7, 1.5 Hz, 1H, C(3")*H*), **6.94** (app td, *J* = 7.7, 1.7 Hz, 1H, ArC(6)*H*), **6.96** (app td, *J* = 7.7, 1.1 Hz, 1H, ArC(5)*H*), **7.20** – **7.24** (m, 3H, C(ArC$^{4'}$*H*)$_3$), **7.25** – **7.29** (m, 6H, C(ArC(3',5')*H*)$_3$), **7.31** – **7.34** (m, 1H, ArC(4)*H*), **7.45** – **7.48** (m, 6H, C(PhC(2',6')*H*)$_3$); **^{13}C{^{1}H} NMR** (176 MHz, CD_2Cl_2) δ **12.7** (C(2")*C*H$_3$), **14.0** (CH$_2$*C*H$_3$), **60.6** (*C*H$_2$CH$_3$), **65.3** (*C*(4")H$_2$), **74.4** (*C*Ph$_3$), **75.6** (*C*(3)H), 115.9 (Ar*C*(7)H), **122.4** (Ar*C*(5)H), **124.6** (Ar*C*(4)H), **125.6** (Ar*C*(3a)), **126.8** (C(Ar*C*(4')H)$_3$), 127.7 (C(Ar*C*(3',5')H)$_3$), **128.3** (Ar*C*(6)H), **129.0** (C(Ph*C*(2,6)H)$_3$), **129.9** (*C*(2")), **136.9** (*C*(3")H), **142.1** (C(Ar*C*(1'))$_3$), **143.8** (Ar*C*(7a)), **167.2** (*C*OOEt), **176.6** (*C*(2)); **HRMS** (ESI$^+$) $C_{34}H_{31}O_4NNa$ [M+Na]$^+$ found 540.2149, requires 540.2145 (+0.75 ppm).

Ethyl (*E*)-4-((2-oxo-1-tritylindolin-3-yl)oxy)crotonate (69)

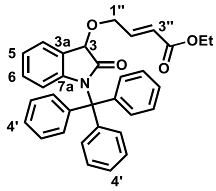

Following **General Procedure F**, 3-diazo-1-tritylindolin-2-one (2.00 g, 4.98 mmol), ethyl (*E*)-4-hydroxybut-2-enoate (721 mg, 5.54 mmol), $Rh_2(OAc)_4$ (10.8 mg, 0.0244 mmol) in anhydrous CH_2Cl_2 (31 mL) gave a crude product which was purified by flash column chromatography (15:5:1 hexane/CH_2Cl_2/EtOAc) to afford the title compound (1.103 g, 44%) as a yellow oil. **IR** ν_{max} (film) 3057, 3032, 2980, 2934 (C-H), 1726, 1717 (C=O), 1609, 1597 (C=C), 1126 (C-O); **^{1}H NMR** (500 MHz, $CDCl_3$) δ **1.28** (t, *J* = 7.1 Hz, 3H, C*H*$_3$), **4.19** (q, *J* = 7.1 Hz, 2H, OC*H*$_2$CH$_3$), **4.23** (ddd, *J* = 16.0, 4.7, 2.1 Hz, 1H, C(3")$H^A$$H^B$), **4.42** (ddd, *J* = 15.7, 4.2, 2.1 Hz, 1H, C(3")$H^A$$H^B$), **5.00** (d, *J* = 1.1 Hz, 1H, C(3)*H*), **6.10** (dt, *J* = 15.7, 2.0 Hz, 1H, C(1")*H*), **6.27** (dd, *J* = 7.5, 1.6 Hz, 1H, ArC(7)*H*), **6.88** – **6.98** (m, 3H, C(1")*H* and ArC(5,6)*H*), **7.16** – 7.**24** (m, 3H, ArC(4')*H*), **7.23** – **7.29** (m, 6H, ArC(3',5')*H*), **7.30** – **7.35** (m, 1H, ArC(4)*H*), 7.40 – **7.51** (m, 6H, ArC(2',6')*H*); **^{13}C{^{1}H} NMR** (126 MHz, $CDCl_3$) δ **14.4** (*C*H$_3$), **60.5** (O*C*H$_2$CH$_3$), **67.4** (*C*(3")H$_2$), **74.6** (N*C*Ph$_3$), **75.7** (*C*(3)H), **116.2** (Ar*C*(7)H), **121.7** (*C*(1")H), **122.6** (Ar*C*(5)H), **124.7** (Ar*C*(4)H), **125.4** (Ar*C*(3a)), **127.1** (Ar*C*(4')H), **127.9** (Ar*C*(3',5')H), **128.6** (Ar*C*(6)H), **129.3** (Ar*C*(2',6')H), **142.0** (Ar*C*(1')), **143.7** (*C*(2")H), **144.1** (Ar*C*(7a)), **166.4** (*C*(O)OEt), **176.6** (*C*(2)=O). **HRMS** (ESI$^+$) $C_{33}H_{29}NO_4Na$ $[M+Na]^+$ found 526.1981, requires 526.1989 (−1.5 ppm).

8.5 Supplementary Data for [1,2]-rearrangement products

(*S*,*E*)-3-Hydroxy-3-(3-phenylbut-2-en-1-yl)-1-tritylindolin-2-one (9)

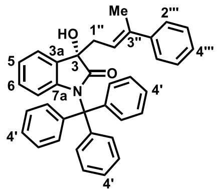

Following **General Procedure G**, allylic ether **8** (52.1 mg, 0.1 mmol), *t*Bu-BIMP (14.7 mg, 0.02 mmol), 4-NHAc-TEMPO (4.3 mg, 0.02 mmol) in mesitylene (2 mL) gave a crude product which was purified by flash column chromatography (eluent: hexane/ EtOAc = 4:1 to 3:1) to afford the product (38.0 mg, 73%) as a colourless amorphous solid. **IR** ν_{max} (film) 1713 (C=O), 1610 (C=C), 1598 (C=C), 1490, 1463, 1448, 1319, 1265, 1184, 1157, 1109, 1083; $\mathbf{[a]_D^{20}}$ +38.8 (*c* 0.25, $CHCl_3$); **Chiral HPLC analysis,** Chiralcel ODH (90:10 hexane:IPA, flow rate 1.0 ml·min^{-1}, 211 nm, 30 °C) t_R (*R*)-**9**: 7.0min, t_R (*S*)-**9**: 8.7min, 5:95 e.r.; **^{1}H NMR** (400 MHz, $CDCl_3$) δ **1.94** (3H, s, C*H*$_3$), **2.75** (1H, br, O*H*), **2.81**–**2.97** (2H, m, OC(1``)*H*$_2$), **5.53** (1H, app dt, *J* 7.9, 5.2, C(2``)*H*), **6.16** (1H, d, *J* 8.1, ArC(7)*H*), **6.80** (1H, app td, *J* 7.9, 1.5, ArC(6)*H*), **6.87** (1H, app td, *J* 7.5, 1.0, ArC(5)*H*), **6.98**–7.**11** (9H, m, ArC*H*), **7.15**–**7.18** (1H, m, ArC(4```)*H*), **7.18**–**7.23** (4H, m, ArC*H*), **7.23**–**7.28** (6H, m, ArC*H*), **7.30** (1H, dd, *J* 7.3, 1.5, ArC(4)*H*); **^{13}C{^{1}H} NMR** (75 MHz, $CDCl_3$) δ **16.5** (*C*H$_3$), **39.5** (*C*(1``)H$_2$), **74.6** (N*C*Ph$_3$), **76.4** (*C*(3)OH), **116.3** (Ar*C*(7)H), **120.0** (*C*(2``)H), **122.7** (Ar*C*(5)H), **123.4** (Ar*C*(4)H), **125.8** (Ar*C*(2```,6```)H), **127.0** (Ar*C*(4`)H), **127.2** (Ar*C*(4```)H), **127.7** (Ar*C*(3`,5`)H), **128.2** (Ar*C*(6)H), **128.4** (Ar*C*(3```,5```)H), **129.4** (Ar*C*(2`,6`)H), **130.0** (Ar*C*(3a)), **139.4** (*C*(3``)), **141.8** (Ar*C*(1`)), **143.2** (*C*(7a)), **143.3** (Ar*C*(1```)), **180.2** (*C*=O); **HRMS (ESI$^+$)** $C_{37}H_{31}NO_2Na$ [M+Na]$^+$ found 544.2237, requires 544.2247 (–1.80 ppm).

(*E*)-3-Hydroxy-3'-[(3-phenylbut-2-en-1-yl)oxy]-1,1'-ditrityl-[3,3'-biindoline]-2,2'-dione (10)

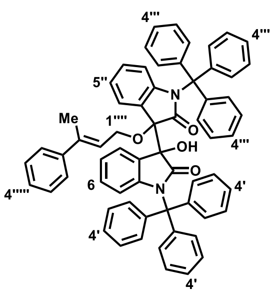

Following **General Procedure G**, allylic ether **8** (52.1 mg, 0.1 mmol), *t*Bu-BIMP (14.7 mg, 0.02 mmol), 4-NHAc-TEMPO (21.3 mg, 0.1 mmol) in mesitylene (2 mL) gave a crude product which was purified by flash column chromatography (eluent: hexane/ EtOAc = 4:1 to 3:1) to afford the product (6.0 mg, 7%) as a colourless amorphous solid. **IR** ν_{max} (film) 3431 (OH), 1720 (C=O), 1602 (C=C), 1490 (C=C), 1462, 1448, 1307, 1267, 1188, 1111 (C-O); $\mathbf{[a]_D^{20}}$ –48.6 (*c* 0.50, $CHCl_3$); **Chiral HPLC analysis** Chiralpak IB (98:2 hexane:IPA, flow rate 1.0 ml·min^{-1}, 211 nm, 30 °C) t_R major-**10**: 12.18min, t_R minor-**10**: 17.50min, 75:25 e.r.; ^{1}**H NMR** (400 MHz, $CDCl_3$) δ **1.66** (3H, s, C*H*$_3$), **2.80–3.00** (1H, m, OC(1````)H^AH^B), **3.16–3.34** (1H, m, OC(1````)H^AH^B), **5.64–5.73** (1H, m, C(2````)*H*), **6.25** (1H, dd, *J* 6.1, 3.3, ArC(7)*H*), **6.45–6.72** (2H, m, ArC(7``)*H*), **6.87** (1H, app t, *J* 7.5, ArC(5``)*H*), **6.96–7.02** (2H, m, ArC(5,6)*H*), **7.10** (1H, app td, *J* 7.9, 1.5, ArC(6``)*H*), **7.13–7.36** (31H, m, ArC*H*), **7.66** (6H, dd, *J* 7.3, 1.9, ArC*H*), **7.74–7.90** (1H, m, ArC*H*); ^{13}**C{^{1}H} NMR** (101 MHz, $CDCl_3$) δ **16.2** (*C*H_3), **61.6** (O*C*(1````)H_2), **74.6** (N(1``)*C*$Ph_3$), **76.2** (N(1)*C*$Ph_3$), **115.7** (Ar*C*(7)H), **116.2** (Ar*C*(7``)H), **122.4** (Ar*C*(5)H), **122.7** (Ar*C*(5``)H), 123**.5** (Ar*C*(2````)H), **124.5** (Ar*C*), **125.8** (Ar*C*), **126.9** (Ar*C*), **127.3** (Ar*C*), **127.5** (Ar*C*), **127.7** (Ar*C*), **127.9** (Ar*C*), **128.1** (Ar*C*), **128.3** (Ar*C*), **128.8** (Ar*C*), **129.2** (Ar*C*), **129.3** (Ar*C*), **129.4** (Ar*C*), **129.7** (Ar*C*), **136.8** (Ar*C*), **138.2** (Ar*C*(3````)), **141.2** (Ar*C*), **142.1** (Ar*C*(1```)), **142.4** (Ar*C*(1`)), **142.8** (Ar*C*(1```)), **144.5** (Ar*C*(7a)), **145.4** (Ar*C*(7a``)), **176.2** (*C*=O), **176.9** (*C*=O). **HRMS (ESI$^+$)** $C_{64}H_{50}N_2O_4Na$ $[M+Na]^+$ found 933.3649, requires 933.3663 (–1.48 ppm).

(*S*,*E*)-3-Hydroxy-3-(3-phenylpent-2-en-1-yl)-1-tritylindolin-2-one (19)

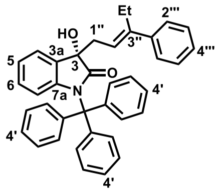

Following **General Procedure G**, allylic ether **S77** (53.5 mg, 0.1 mmol), *t*Bu-BIMP (14.7 mg, 0.02 mmol), 4-NHAc-TEMPO (4.3 mg, 0.02 mmol) in mesitylene (2 mL) gave a crude [1,2]-rearrangement product which was purified by flash column chromatography (eluent: hexane/ EtOAc = 4:1 to 3:1) to afford the product (37.5 mg, 70%) as a colourless amorphous solid. **IR** ν_{max} (film) 3394 (OH), 1712 (C=O), 1610 (C=C), 1599 (C=C), 1490, 1463, 1448, 1319, 1265, 1184, 1157, 1109; $\mathbf{[a]_D^{20}}$ +38.4 (*c* 0.25, $CHCl_3$); **Chiral HPLC analysis**, Chiralcel ODH (90:10 hexane:IPA, flow rate 1.0 ml·min^{-1}, 211 nm, 30 °C) t_R (*R*)-**19**: 8.9 min, t_R (*S*)-**19**: 11.2 min, 6:94 e.r.; **^{1}H NMR** (500 MHz, $CDCl_3$) δ **0.85** (3H, t, *J* 7.5, CH_2CH_3), **2.03–2.36** (1H, m, O*H*), **2.37–2.52** (2H, m, CH_2CH_3), **2.84** (1H, dd, *J* 13.8, 6.3, C(1``)H$^A$*H$^B$*), **2.92** (1H, dd, *J* 13.8, 9.1, C(1``)H^A*H^B*), **5.37** (1H, dd, *J* 9.0, 6.4, C(2``)*H*), **6.17** (1H, d, *J* 8.1, ArC(7)*H*), **6.80** (1H, app td, *J* 8.0, 1.4, ArC(6)*H*), **6.88** (1H, app td, *J* 7.6, 0.7, ArC(5)*H*), **7.02–7.10** (9H, m, ArC*H*), **7.15–7.22** (5H, m, ArC*H*), **7.25–7.29** (6H, m, ArC*H*), **7.30** (1H, dd, *J* 7.3, 1.2, ArC(4)*H*); **^{13}C{^{1}H} NMR** (126 MHz, $CDCl_3$) δ **13.5** (CH_2CH_3), **23.4** (CH_2CH_3), **39.1** (*C*(1``)$H_2$), **74.7** (N*C*$Ph_3$), **76.3** (*C*(3)OH), **116.3** (Ar*C*(7)H), **119.6** (*C*(2``)H), **122.7** (Ar*C*(5)H), **123.4** (Ar*C*(4)H), **126.6** (Ar*C*(2```,6```)H), **127.0** (Ar*C*(4`)H), **127.1** (Ar*C*(4```)H), **127.8** (Ar*C*(3`,5`)H), **128.2** (Ar*C*(6)H), **128.4** (Ar*C*(3```,5```)H), **129.4** (Ar*C*(2`,6`)H), **130.0** (Ar*C*(3a)), **141.8** (Ar*C*(1`)), **142.5** (Ar*C*(1```)), **143.3** (*C*(7a)), **146.4**, (*C*(3``)Et), **180.2** (*C*=O); **HRMS (ESI$^+$)** $C_{38}H_{33}NO_2Na$ [M+Na]$^+$ found 558.2395, requires 558.2404 (–1.5 ppm).

(*S*,*E*)-3-(3-Cyclopropyl-3-phenylallyl)-3-hydroxy-1-tritylindolin-2-one (20)

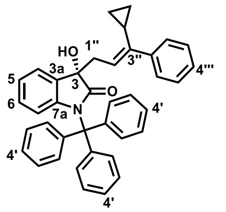

Following **General Procedure G**, allylic ether **S78** (54.7 mg, 0.1 mmol), *t*Bu-BIMP (14.7 mg, 0.02 mmol), 4-NHAc-TEMPO (4.3 mg, 0.02 mmol) in mesitylene (2 mL) gave a crude [1,2]-rearrangement product which was purified by flash column chromatography (eluent: hexane/ EtOAc = 4:1 to 3:1) to afford the product (21.9 mg, 40%) as a colourless amorphous solid. **IR** ν_{max} (film) 3379 (OH), 1717 (C=O), 1610 (C=C), 1598 (C=C), 1463, 1448, 1317, 1263, 1111; $[\mathbf{a}]_{\mathbf{D}}^{\mathbf{20}}$ –10.4 (*c* 0.25, $CHCl_3$); **Chiral HPLC analysis**, Chiralpak IA (95:5 hexane:IPA, flow rate 1 ml·min^{-1}, 211 nm, 30 °C) t_R (*R*)-**20**: 17.4min, t_R (*S*)-**20**: 24.3min, 4:96 e.r.; ^{1}**H NMR** (500 MHz, $CDCl_3$) δ **0.23–0.34** (2H, m, CH((C*H*$_2$)^A(CH$_2$)^B)), 0.78–0.87 (2H, m, CH((CH$_2$)^A(C*H*$_2$)^B)), **1.67–1.78** (1H, m, CH((C*H*$_2$)$_2$)), **2.72** (1H, s, O*H*), **3.03** (1H, ddd, *J* 13.7, 6.0, 1.2, C(1``)*H*$^A$H$^B$), **3.30** (1H, dd, *J* 13.8, 9.4, C(1``)H^A*H*^{B}), **5.53** (1H, ddd, *J* 9.3, 5.9, 1.8, C(2``)*H*), **6.26** (1H, d, *J* 8.0, ArC(7)*H*), **6.89** (1H, app td, *J* 7.9, ArC(6)*H*), **6.96** (1H, app td, *J* 7.94, ArC(5)*H*), **7.11–7.19** (9H, m, ArC*H*), **7.20–7.28** (5H, m, ArC*H*), **7.33–7.40** (7H, m, ArC*H*); ^{13}**C{**^{1}**H} NMR** (126 MHz, $CDCl_3$) δ 6.8 (CH((*C*H$_2$)^A(CH$_2$)^B)), **7.2** (CH((CH$_2$)^A(*C*H$_2$)^B)), **11.8** (*C*H(CH$_2$)$_2$), **39.3** (C(1``)H$_2$), **74.6** (N*C*Ph$_3$), **76.4** (*C*(3)OH), **116.3** (Ar*C*(7)H), **122.7** (Ar*C*(5)H), **123.1** (C(2``)H), **123.4** (Ar*C*(4)H), **126.8** (Ar*C*(4```)H), **127.0** (Ar*C*(4`)H), **127.5** (Ar*C*(3```,5```)*H*), **127.8** (Ar*C*(3`,5`)H), **128.0** (Ar*C*(2```,6```)H), **128.2** (Ar*C*(6)H), **129.4** (Ar*C*(2`,6`)H), **130.1** (Ar*C*(3a)), **141.8** (*C*(3``)Ar), **142.0** (Ar*C*(1`)), **143.2** (Ar*C*(7a)), **145.4** (Ar*C*(1```)), **180.4** (*C*=O); **HRMS (ESI$^+$)** $C_{39}H_{33}NO_2Na$ [M+Na]$^+$ found 570.2390, requires 570.2404 (–2.4 ppm).

(*S*)-1-Benzyl-3-(3,3-diphenylallyl)-3-hydroxyindolin-2-one (21)

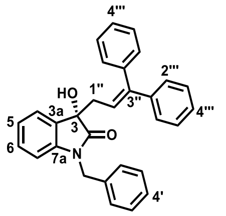

Following **General Procedure G**, allylic ether **S1** (43.1 mg, 0.1 mmol), *t*Bu-BIMP (14.7 mg, 0.02 mmol), 4-NHAc-TEMPO (4.3 mg, 0.02 mmol) in mesitylene (2 mL) gave a crude [1,2]-rearrangement product which was purified by flash column chromatography (eluent: hexane/ EtOAc = 4:1 to 3:1) to afford the product (26.3 mg, 61%) as a colourless amorphous solid. **IR** ν_{max} (film) 3365 (OH), 1701 (C=O), 1614 (C=C), 1489, 1465, 1352, 11701114, 1076; $[\mathbf{a}]_{\mathbf{D}}^{\mathbf{20}}$ –5.6 (*c* 0.25, $CHCl_3$); **Chiral HPLC analysis**, Chiralcel ODH (90:10 hexane:IPA, flow rate 1.0 ml·min^{-1}, 211 nm, 30 °C) t_R (*S*)-**21**: 13.1 min, t_R (*R*)-**21**: 11.2 min, 27:73 e.r.; **^{1}H NMR** (400 MHz, $CDCl_3$) δ **2.76–2.93** (2H, m, C(1``)*H*$_2$), **3.02** (1H, br s, O*H*), **4.65** (1H, d, *J* 15.7, NC*H*$^A$H$^B$Ph), **5.12** (1H, d, *J* 15.7, NCH$^A$*H*$^B$Ph), **5.97** (1H, app t, *J* 7.5, C(2``)*H*), **6.70** (1H, d, *J* 7.8, ArC(7)*H*), **6.92–7.00** (2H, m, ArC*H*), **7.00–7.08** (3H, m, ArC*H*), **7.13–7.25** (9H, m, ArC*H*), **7.26–7.34** (4H, m, ArC*H*); **^{13}C{^{1}H} NMR** (101 MHz, $CDCl_3$) δ **39.2** (*C*(1``)H$_2$), **44.0** (N*C*H$_2$Ph), **76.5** (*C*(3)OH), **109.6** (Ar*C*(7)H), **120.8** (*C*(2``)H), **123.2** (Ar*C*(5)H), **124.5** (Ar*C*(4)H), **127.2** (Ar*C*H), **127.3** (Ar*C*H), **127.3** (Ar*C*H), **127.4** (Ar*C*H), **127.8** (Ar*C*H), **128.2** (Ar*C*(6)H), **128.4** (Ar*C*H), **129.0** (Ar*C*H), **129.7** (Ar*C*(3a)), **129.8** (Ar*C*H), **129.9** (Ar*C*H), **135.5** (Ar*C*(1`)), **139.4** (Ar*C*), **142.0** (Ar*C*(1```)), **142.5** (*C*(7a)), **145.9** (*C*(3``)Ar$_2$), **178.0** (*C*=O); **HRMS (ESI$^+$)** $C_{30}H_{25}NO_2Na$ [M+Na]$^+$ found 454.1775, requires 454.1778 (–0.5 ppm).

(S,*E*)-3-(3,3-diphenylallyl)-3-hydroxy-1-tritylindolin-2-one (S6)

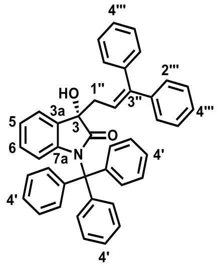

Following **General Procedure G**, allylic ether **S3** (58.3 mg, 0.1 mmol), *t*Bu-BIMP (14.7 mg, 0.02 mmol), 4-NHAc-TEMPO (4.3 mg, 0.02 mmol) in mesitylene (2 mL) gave a crude [1,2]-rearrangement product which was purified by flash column chromatography (eluent: hexane/ EtOAc = 4:1 to 3:1) to afford the product (11.7 mg, 20%) as a colourless amorphous solid. **IR** ν_{max} (film) 3317 (OH), 1705 (C=O), 1599 (C=C), 1490, 1462, 1446, 1251, 1197, 1138, 1031; $\mathbf{[a]_D^{20}}$+14.3 (*c* 0.35, $CHCl_3$); **Chiral HPLC analysis**, Chiralpak IA (90:10 hexane:IPA, flow rate 1.0 ml·min^{-1}, 211 nm, 30 °C) t_R (*S*)-**S6**: 11.0 min, t_R (*R*)-**S6**: 14.4 min, 83:17 e.r.; **^{1}H NMR (400 MHz, $CDCl_3$)** δ **2.61** (1H, br s, O*H*), **2.76–2.91** (2H, m, C(1``)$H_2$), **6.07**, (1H, dd, *J* 8.4, 6.7, C(2``)*H*), **6.24–6.32** (1H, m, ArC(7)*H*), **6.88–6.98** (2H, m, ArC(5,6)*H*), **6.98–7.02** (2H, m, ArC*H*), **7.13–7.20** (11H, m, ArC*H*), **7.22–7.26** (4H, m, ArC*H*, ArC(4)*H*), **7.31–7.38** (9H, m, ArC*H*); **^{13}C{^{1}H} NMR (101 MHz, $CDCl_3$)** δ **40.3** (*C*(1``)$H_2$), **76.3** (*C*(3)OH), **77.4** (N*C*$Ph_3$), **116.3** (Ar*C*(7)H), **121.4** (*C*(2``)H), **122.7** (Ar*C*(5)H), **123.8** (Ar*C*(4)H), **127.1** (Ar*C*(4`)H), **127.3** (Ar*C*H), **127.4** (Ar*C*H), **127.5** (Ar*C*H), **127.8** (Ar*C*(3`,5`)H), **128.2** (Ar*C*(6)H), **128.3** (Ar*C*H), **128.4** (Ar*C*H), **129.4** (Ar*C*(2`,6`)H), **130.0** (*C*(3a)), **139.4** (Ar*C*), **141.8** (Ar*C*(1`)), **142.2** (Ar*C*(1```)), **143.0** (*C*(7a)), **145.7** (*C*(3``)Ar_2), **180.3** (*C*=O); **HRMS (ESI$^+$)** $C_{42}H_{33}NO_2Na$ $[M+Na]^+$ found 606.2403, requires 606.2404 (–0.1 ppm).

(*S*,*E*)-3-[3-(4-Fluorophenyl)but-2-en-1-yl]-3-hydroxy-1-tritylindolin-2-one (13)

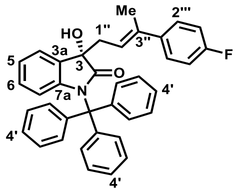

Following **General Procedure G**, allylic ether **11** (53.9 mg, 0.1 mmol), *t*Bu-BIMP (14.7 mg, 0.02 mmol), 4-NHAc-TEMPO (4.3 mg, 0.02 mmol) in mesitylene (2 mL) gave a crude [1,2]-rearrangement product which was purified by flash column chromatography (eluent: hexane/ EtOAc = 4:1 to 3:1) to afford the product (41.0 mg, 76%) as a colourless amorphous solid. **IR** ν_{max} (film) 3350 (OH), 1719 (C=O), 1598 (C=C), 1508, 1463, 1448, 1222, 1184, 1159 (C-F), 1107; $[\mathbf{a}]_{\mathbf{D}}^{\mathbf{20}}$ +25.6 (*c* 0.25, $CHCl_3$); **Chiral HPLC analysis**, Chiralcel ODH (95:5 hexane:IPA, flow rate 1 ml·min^{-1}, 211 nm, 30 °C) t_R (*R*)-**13**: 11.1 min, t_R (*S*)-**13**: 15.3 min, 6:94 e.r.; **^{1}H NMR** (500 MHz, $CDCl_3$) δ **2.01** (3H, s, C*H*$_3$), **2.71–2.83** (1H, m, O*H*), **2.92** (1H, dd, *J* 13.8, 6.6, C(1``)*H*$^A$H$^B$), **3.00** (1H, dd, *J* 13.7, 9.0, C(1``)*H*^{A}H^B), **5.48–5.61** (1H, m, C(2``)*H*), **6.26** (1H, d, *J* 8.1, ArC(7)*H*), **6.91** (1H, app td, *J* 7.9, 1.5, ArC(6)*H*), **6.95–7.02** (3H, m, ArC(5`,3```,5```)*H*), **7.11–7.20** (9H, m, ArC(3`,4`,5`)*H*), **7.22–7.26** (2H, m, ArC(2```,6```)*H*), **7.30–7.37** (6H, m, ArC(2`,6`)*H*), **7.39** (1H, dd, *J* 7.4, 1.5, ArC(4)*H*); **^{13}C{^{1}H} NMR** (126 MHz, $CDCl_3$) δ **16.6** (*C*H$_3$), **39.4** (*C*(1``)H$_2$), **74.6** (N*C*Ph$_3$), **76.3** (*C*(3)OH), **115.2** (d, $^2J_{C\text{-}F}$ 21.3, Ar*C*(3```,5```)H), **116.3** (Ar*C*(7)H), **120.0** (*C*(2``)H), **122.8** (Ar*C*(5)H), **123.4** (Ar*C*(4)H), **127.1** (Ar*C*(4`)H), **127.3** (d, $^3J_{C\text{-}F}$ 7.8, Ar*C*(2```,6```)H), **127.7** (Ar*C*(3`,5`)H), **128.3** (Ar*C*(6)H), **129.4** (Ar*C*(2`,6`)H), **129.9** (Ar*C*(3a)), **138.4** (*C*(3``)Ar), **139.4** (d, $^4J_{C\text{-}F}$ 3.2, Ar*C*(1```)), **141.8** (Ar*C*(1`)), **143.2** (Ar*C*(7a)), **162.2** (d, $^1J_{C\text{-}F}$ 246.3, Ar*C*(4```)F), **180.1** (*C*=O); **^{19}F{^{1}H} NMR** (377 MHz, $CDCl_3$) δ **–115.73** (ArC(4```)*F*). **HRMS (ESI$^+$)** $C_{37}H_{30}FNO_2Na$ [M+Na]$^+$ found 562.2145, requires 562.2153 (–1.38 ppm);

(*S*,*E*)-3-[3-(4-Chlorophenyl)but-2-en-1-yl]-3-hydroxy-1-tritylindolin-2-one (22)

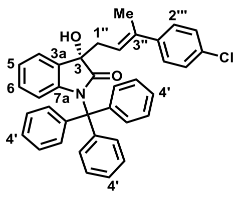

Following **General Procedure G**, allylic ether **S84** (55.5 mg, 0.1 mmol), *t*Bu-BIMP (14.7 mg, 0.02 mmol), 4-NHAc-TEMPO (4.3 mg, 0.02 mmol) in mesitylene (2 mL) gave a crude [1,2]-rearrangement product which was purified by flash column chromatography (eluent: hexane/ EtOAc = 4:1 to 3:1) to afford the product (42.2 mg, 76%) as a colourless amorphous solid. **IR ν$_{max}$ (film)** 3402 (OH), 1717 (C=O), 1610 (C=C), 1490, 1463, 1446, 1319, 1186, 1091 (C-Cl); **[a]$_D^{20}$** +19.2 (*c* 0.25, $CHCl_3$); **Chiral HPLC analysis**, Chiralpak IA (90:10 hexane:IPA, flow rate 1 ml·min^{-1}, 211 nm, 30 °C) t$_R$ (*R*)-**22**: 11.4 min, t$_R$ (*S*)-**22**: 14.4 min, 5:95 e.r.; **^{1}H NMR (400 MHz, $CDCl_3$)** δ **1.92** (3H, s, C*H*$_3$), **2.25–2.78** (1H, br s, O*H*), **2.79–2.87** (1H, m, C(1``)*H*$^A$H$^B$), **2.91** (1H, dd, *J* 13.7, 8.9, C(1``)H^A*H*^{B}), **5.51** (1H, ddd, *J* 8.9, 6.7, 1.4, C(2``)*H*), **6.17** (1H, d, *J* 8.0, ArC(7)*H*), **6.82** (1H, app td, *J* 7.9, 1.5, ArC(6)*H*), **6.89** (1H, app td, *J* 7.5, 1.0, ArC(5)*H*), **7.03–7.19** (13H, m, ArC(3`,4`,5`,2```,6```)*H*), **7.21–7.28** (6H, m, ArC(2`,6`)*H*), **7.30** (1H, dd, *J* 7.3, 1.5, ArC(4)*H*); **^{13}C{^{1}H} NMR (101 MHz, $CDCl_3$)** δ **16.4** (*C*H$_3$), **39.3** (*C*(1``)H$_2$), **74.6** (N*C*Ph$_3$), **76.3** (*C*(3)OH), **116.3** (Ar*C*(7)H), **120.7** (*C*(2``)H), **122.8** (Ar*C*(5)H), **123.4** (Ar*C*(4)H), **127.0** (Ar*C*(4`)H), **127.1** (Ar*C*(3```,5```)), **127.7** (Ar*C*(3`,5`)H), **128.3** (Ar*C*(6)H), **128.5** (Ar*C*(2```,6```)H), **129.4** (Ar*C*(2`,6`)H), **129.9** (Ar*C*(3a)), **132.9** (Ar*C*(4```)Cl), **138.2** (*C*(3``)Ar), **141.7** (Ar*C*(1`,1```), **143.2** (Ar*C*(7a)), **180.1** (*C*=O); **HRMS (ESI$^+$)** $C_{37}H_{30}{}^{35}ClNO_2Na$ [M+Na]$^+$ found 578.1851, requires 578.1857 (–1.1 ppm)..

(*S*,*E*)-3-[3-(4-Bromophenyl)but-2-en-1-yl]-3-hydroxy-1-tritylindolin-2-one (23)

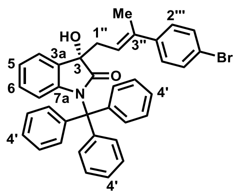

Following **General Procedure G**, allylic ether **S85** (59.9 mg, 0.1 mmol), *t*Bu-BIMP (14.7 mg, 0.02 mmol), 4-NHAc-TEMPO (4.3 mg, 0.02 mmol) in mesitylene (2 mL) gave a crude [1,2]-rearrangement product which was purified by flash column chromatography (eluent: hexane/ EtOAc = 4:1 to 3:1) to afford the product (46.1 mg, 77%) as a colourless amorphous solid. **IR ν_{max} (film)** 3361 (OH), 1716 (C=O), 1610 (C=C), 1481, 1465, 1448, 1319, 1265, 1186, 1109, 1078 (C-Br), 1033 (C-Br); **$[a]_D^{20}$** +20.0 (*c* 0.25, $CHCl_3$); **Chiral HPLC analysis**, Chiralpak IA (90:10 hexane:IPA, flow rate 1 ml·min^{-1}, 211 nm, 30 °C) t_R (*R*)-**23**: 11.7 min, t_R (*S*)-**23**: 14.7 min, 5:95 er; **^{1}H NMR (400 MHz, $CDCl_3$)** δ **2.00** (3H, s, C*H*$_3$), **2.63** (1H, br s, O*H*), **2.91** (1H, app dd, *J* 13.7, 9.0, OC*H*^{A}H^B), **2.99** (1H, app dd, *J* 13.7, 6.6, OCH^A*H*^{B}), **5.60** (1H, app t, *J* 7.7, =C(2``)*H*), **6.26** (1H, d, *J* 8.1, ArC(7)*H*), **6.91** (1H, app t, *J* 7.8, ArC(6)*H*), **6.98** (1H, app t, *J* 7.5, ArC(5)*H*), **7.10–7.20** (11H, m, ArC*H*), **7.31–7.36** (6H, m, ArC*H*), **7.37–7.44** (3H, m, ArC*H*); **^{13}C{^{1}H} NMR (75 MHz, $CDCl_3$)** δ **16.3** (*C*H$_3$), **39.4** (*C*(1``)H$_2$), **74.6** (N*C*Ph$_3$), **76.2** (*C*(3)OH), **116.3** (Ar*C*(7)H), **120.8** (*C*(2``)H), **121.1** (Ar*C*(4```)Br), **122.8** (Ar*C*(5)H), **123.4** (Ar*C*(4)H), **127.1** (Ar*C*(4`)H), **127.4** (Ar*C*(3```,5```)H), **127.7** (Ar*C*(3`,5`)H), **128.3** (Ar*C*(6)H), **129.4** (Ar*C*(2`,6`)H), **129.9** (Ar*C*(3a)), **131.5** (Ar*C*(2```,6```)H), **138.3** (*C*(3``)Ar), **141.7** (Ar*C*(1`)H), **142.2** (Ar*C*(1```)), **143.2** (Ar*C*(7a)), **180.1** (*C*=O); **HRMS (ESI$^+$)** $C_{37}H_{30}{}^{79}BrNO_2Na$ [M+Na]$^+$ found 622.1346, requires 622.1352 (–0.96 ppm)..

(*S*,*E*)-3-Hydroxy-3-{3-[4-(trifluoromethyl)phenyl]but-2-en-1-yl}-1-tritylindolin-2-one (24)

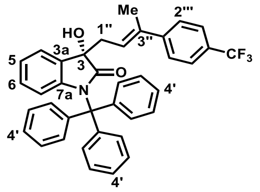

Following **General Procedure G**, allylic ether **S86** (58.9 mg, 0.1 mmol), *t*Bu-BIMP (14.7 mg, 0.02 mmol), 4-NHAc-TEMPO (4.3 mg, 0.02 mmol) in mesitylene (2 mL) gave a crude [1,2]-rearrangement product which was purified by flash column chromatography (eluent: hexane/ EtOAc = 4:1 to 3:1) to afford the product (49.5 mg, 84%) as a colourless amorphous solid. **IR** ν_{max} (film) 3377 (OH), 1717 (C=O), 1614 (C=C), 1465, 1448, 1325 (C-F), 1166, 1116, 1074; $[a]_D^{20}$ +24.8 (*c* 0.25, $CHCl_3$); **Chiral HPLC analysis**, Chiralpak IA (90:10 hexane:IPA, flow rate 1 ml·min^{-1}, 211 nm, 30 °C) t_R (*R*)-**24**: 10.6 min, t_R (*S*)-**24**: 12.8 min, 4:96 e.r.; **^{1}H NMR (400 MHz, $CDCl_3$)** δ **2.06** (3H, s, C*H*$_3$), **2.87–2.99** (2H, m, C(1``)*H*$^A$H$^B$, O*H*), **3.04** (1H, app dd, *J* 13.8, 8.9, OCH$^A$*H*$^B$), **5.63–5.72** (1H, m, C(2``)*H*), **6.28** (1H, d, *J* 8.1, ArC(7)*H*), **6.92** (1H, app t, *J* 7.9, 1.5, ArC(6)*H*), **6.99** (1H, app td, *J* 7.5, 1.0, ArC(5)*H*), **7.11–7.19** (9H, m, ArC(3`,4`,5`)*H*), **7.31–7.36** (6H, m, ArC(2`,6`)*H*), **7.37** (2H, d, *J* 8.4, ArC(2```,6```)*H*), **7.40** (1H, dd, *J* 7.3, 1.5, ArC(4)*H*), **7.56** (2H, d, *J* 8.2, ArC(3```,5```)*H*); **^{13}C{^{1}H} NMR (101 MHz, $CDCl_3$)** δ **16.3** (*C*H$_3$), **39.4** (*C*(1``)H$_2$), **74.6** (N*C*Ph$_3$), **76.2** (*C*(3)OH), **116.4** (Ar*C*(7)H), **122.5** (*C*(2``)H), **122.8** (Ar*C*(5)H), **123.4** (Ar*C*(4)H), **124.4** (q, $^1J_{C\text{-}F}$ 272.9, *C*F$_3$), **125.3** (q, $^3J_{C\text{-}F}$ 3.9, Ar*C* (3```,5```)H), **126.1** (ArC(2```,6```)H), **127.1** (Ar*C*(4`)H), **127.7** (Ar*C*(3`,5`)H), **128.4** (Ar*C*(6)H), **129.4** (Ar*C*(2`,6`)H), **129.8** (Ar*C*(3a)), **138.3** (*C*(3``)Ar), **141.7** (Ar*C*(1`)), **143.2** (Ar*C*(7a)), **146.9** (Ar*C*(1```)), **180.0** (*C*=O); **^{19}F NMR (376 MHz, $CDCl_3$)** δ **–62.44** (C*F*$_3$); **HRMS (ESI$^+$)** $C_{38}H_{30}F_3NO_2Na$ [M+Na]$^+$ found 612.2107, requires 612.2121 (–2.3 ppm)..

(*S*,*E*)-3-{3-[(1,1'-biphenyl)-4-yl]but-2-en-1-yl}-3-hydroxy-1-tritylindolin-2-one (25)

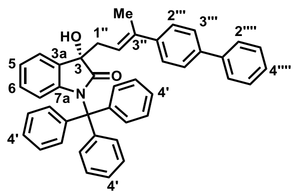

Following **General Procedure G**, allylic ether **S87** (59.7 mg, 0.1 mmol), *t*Bu-BIMP (14.7 mg, 0.02 mmol), 4-NHAc-TEMPO (4.3 mg, 0.02 mmol) in mesitylene (2 mL) gave a crude [1,2]-rearrangement product which was purified by flash column chromatography (eluent: hexane/ EtOAc = 4:1 to 3:1) to afford the product (43.6 mg, 73%) as a colourless amorphous solid. **IR** ν_{max} (film) 3421 (OH), 1716 (C=O), 1610 (C=C), 1597 (C=C), 1487, 1463, 1448, 1317, 1182, 1107; $\mathbf{[a]_D^{20}}$ +13.6 (*c* 0.25, $CHCl_3$); **Chiral HPLC analysis**, Chiralpak IA (90:10 hexane:IPA, flow rate 1 ml·min^{-1}, 211 nm, 30 °C) t_R (*R*)-**25**: 13.5 min, t_R (*S*)-**25**: 15.8 min, 5:95 e.r.; **^{1}H NMR (500 MHz, $CDCl_3$)** δ **2.09** (3H, s, C*H*$_3$), **2.64** (1H, br s, O*H*), **2.98** (1H, dd, *J* 13.5, 6.5, C(1``)$H^AH^B$), **3.06** (1H, dd, *J* 13.5, 9.1, C(1``)H^AH^B), **5.70**, (1H, app t, *J* 7.4, C(2``)*H*), **6.27** (1H, d, *J* 8.1, ArC(7)*H*), **6.92** (1H, app td, *J* 7.9, 1.5, ArC(6)*H*), **7.00** (1H, app t, *J* 7.2, ArC(5)*H*), **7.09–7.23** (9H, m, Ar*C*(3`,4`,5`)*H*), **7.31–7.41** (9H, m, ArC(2`,6`,3```,5```,4````)*H*), **7.43** (1H, d, *J* 7.3, ArC(4)*H*), **7.47** (2H, app t, *J* 7.6, ArC(3````,5````)*H*), **7.55** (2H, app d, *J* 8.3, ArC(2```,6```)*H*), **7.62** (2H, app d, *J* 7.3, ArC(2````,6````)*H*); **^{13}C{^{1}H} NMR (126 MHz, $CDCl_3$)** δ **16.4** (*C*H$_3$), **39.5** (*C*(1``)H$_2$), **74.6** (N*C*Ph$_3$), **76.4** (*C*(3)OH), **116.3** (Ar*C*(7)H), **120.1** (=*C*(2``)H), **122.7** (Ar*C*(5)H), **123.4** (Ar*C*(4)H), **126.2** (Ar*C*(3```,5```)H), **127.0** (Ar*C*(4`)H), **127.1** (Ar*C*(2````,6````)H), **127.2** (Ar*C*(2```,6```)H), **127.4** (Ar*C*(4````)H), **127.7** (Ar*C*(3`,5`)H), **128.3** (Ar*C*(6)H), **128.9** (Ar*C*(3````,5````)H), **129.4** (Ar*C*(2`,6`)H), **129.9** (Ar*C*(3a)), **138.9** (*C*(3``)Ar), **140.0** (Ar*C*(4```)), **140.9** (Ar*C*(1````)), **141.7** (Ar*C*(1`)), **142.2** (Ar*C*(1```)), **143.3** (Ar*C*(7a)), **180.2** (*C*=O); **HRMS (ESI$^+$)** $C_{43}H_{35}NO_2Na$ [M+Na]$^+$ found 620.2544, requires 620.2560 (–2.5 ppm).

(*S*,*E*)-3-Hydroxy-3-[3-(p-tolyl)but-2-en-1-yl]-1-tritylindolin-2-one (26)

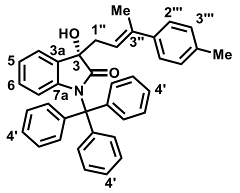

Following **General Procedure G**, allylic ether **S88** (53.5 mg, 0.1 mmol), *t*Bu-BIMP (14.7 mg, 0.02 mmol), 4-NHAc-TEMPO (4.3 mg, 0.02 mmol) in mesitylene (2 mL) gave a crude [1,2]-rearrangement product which was purified by flash column chromatography (eluent: hexane/ EtOAc = 4:1 to 3:1) to afford the product (39.6 mg, 74%) as a colourless amorphous solid. **IR** ν_{max} (film) 3392 (OH), 1715 (C=O), 1610 (C=C), 1600 (C=C), 1490, 1465, 1448, 1319, 1265, 1186, 1112, 1083; $[\mathbf{a}]_{\mathbf{D}}^{\mathbf{20}}$ +27.2 (*c* 0.25, $CHCl_3$); **Chiral HPLC analysis**, Chiralpak IA (90:10 hexane:IPA, flow rate 1 ml·min^{-1}, 211 nm, 30 °C) t_R (*R*)-**26**: 9.6 min, t_R (*S*)-**26**: 11.8 min, 7:93 e.r.; **^{1}H NMR (300 MHz, $CDCl_3$)** δ **2.03** (3H, s, =C(3``)CH_3), **2.37** (3H, s, ArC(4```)CH_3), **2.46–2.87** (1H, br s, O*H*), **2.90–3.07** (2H, m, C(1``)$H_2$), **5.63** (1H, app t, *J* 7.8, C(2``)*H*), **6.27** (1H, d, *J* 8.0, ArC(7)*H*), **6.91** (1H, app td, *J* 7.8, 1.6, ArC(6)*H*), **6.98** (1H, app t, *J* 7.4, ArC(5)*H*), **7.05–7.21** (11H, m, ArC*H*), **7.21–7.27** (3H, m, ArC*H*), **7.32–7.45** (7H, m, ArC*H*); **^{13}C{^{1}H} NMR (75 MHz, $CDCl_3$)** δ **16.4** (*C*H_3), **21.2** (ArC(4```)*C*H_3), **39.4** (*C*(1``)$H_2$), **74.6** (N*C*$Ph_3$), **76.4** (*C*(3)OH), **116.3** (Ar*C*(7)H), **119.1** (*C*(2``)H), **122.7** (Ar*C*(5)H), **123.4** (Ar*C*(4)H), **125.7** (Ar*C*(3```,5```)H), **127.0** (Ar*C*(4`)H), **127.7** (Ar*C*(3`,5`)H), **128.2** (Ar*C*(6)H), **129.1** (Ar*C*(2```,6```)H), **129.4** (Ar*C*(2`,6`)H), **130.0** (Ar*C*(3a)), **136.9** (Me*C*(4```)), **139.2** (*C*(3``)Ar), **140.4** (Ar*C*(1```)), **141.8** (Ar*C*(1`)), **143.2** (Ar*C*(7a)), **180.3** (*C*=O); **HRMS (ESI$^+$)** $C_{38}H_{33}NO_2Na$ $[M+Na]^+$ found 558.2391, requires 558.2404 (–2.2 ppm).

(*S*,*E*)-3-Hydroxy-3-[3-(4-methoxyphenyl)but-2-en-1-yl]-1-tritylindolin-2-one (18)

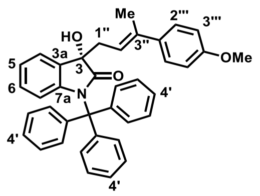

Following **General Procedure G**, allylic ether **16** (55.1 mg, 0.1 mmol), *t*Bu-BIMP (14.7 mg, 0.02 mmol), 4-NHAc-TEMPO (4.3 mg, 0.02 mmol) in mesitylene (2 mL) gave a crude [1,2]-rearrangement product which was purified by flash column chromatography (eluent: hexane/ EtOAc = 4:1 to 3:1) to afford the product (40.8 mg, 74%) as a colourless amorphous solid. **IR** ν_{max} (film) 3396 (OH), 1717 (C=O), 1606 (C=C), 1510, 1463, 1448, 1246 (C-O), 1180, 1031 (C-O); $[\mathbf{a}]_{\mathbf{D}}^{\mathbf{20}}$ +28.0 (*c* 0.25, $CHCl_3$); **Chiral HPLC analysis**, Chiralpak IA (90:10 hexane:IPA, flow rate 1 ml·min^{-1}, 211 nm, 30 °C) t_R (*R*)-**18**: 13.3 min, t_R (*S*)-**18**: 15.8 min, 6:94 e.r.; **^{1}H NMR** (300 MHz, $CDCl_3$) δ 2.01 (3H, s, C(3``)C*H*$_3$), 2.68–2.88 (1H, br s, O*H*), 2.92 (1H, dd, *J* 14.2, 6.7, C(1``)*H*^{A}H^B), 3.01 (1H, dd, *J* 13.6, 8.9, C(1``)H$^A$*H*$^B$), 3.81 (3H, s, OC*H*$_3$), 5.50–5.63 (1H, m, C(2``)*H*), 6.26 (1H, d, *J* 7.7, ArC(7)*H*), 6.82–6.88 (2H, app t, *J* 7.8, C(3```,5```)*H*), 6.91 (1H, dd, *J* 7.9, 1.6, ArC(6)*H*), 6.98 (1H, app td, *J* 7.5, 1.1, ArC(5)*H*), 7.10–7.20 (9H, m, ArC(3`,5`)*H*), 7.23–7.28 (2H, m, ArC(2```,6```)*H*), 7.31–7.38 (6H, m, ArC(2`,6`)*H*), 7.39–7.42 (1H, m, ArC(4)*H*); **^{13}C{^{1}H} NMR** (75 MHz, $CDCl_3$) δ 16.4 (C(3``)*C*H$_3$), 39.5 (*C*(1``)H$_2$), 55.5 (O*C*H$_3$), 74.6 (N*C*Ph$_3$), 76.4 (*C*(3)OH), 113.8 (Ar*C*(3```,5```)H), 116.3 (Ar*C*(7)H), 118.3 (*C*(2``)H), 122.7 (Ar*C*(5)H), 123.4 (Ar*C*(4)H), 126.8 (Ar*C*(2```,6```)H), 127.0 (Ar*C*(4`)H), 127.7 (Ar*C*(3`,5`)H), 128.2 (Ar*C*(6)H), 129.4 (Ar*C*(2`,6`)H), 130.1 (Ar*C*(3a)), 135.9 (*C*(3``)Ar), 138.7 (Ar*C*(1```)), 141.8 (Ar*C*(1`)), 143.2 (Ar*C*(7a)), 158.9 (Ar*C*(4```)OCH$_3$), 180.3 (*C*=O); **HRMS (ESI$^+$)** $C_{38}H_{33}NO_3Na$ [M+Na]$^+$ found 574.2344, requires 574.2353 (–1.6 ppm).

(*S*,*E*)-3-Hydroxy-3-[3-(m-tolyl)but-2-en-1-yl]-1-tritylindolin-2-one (27)

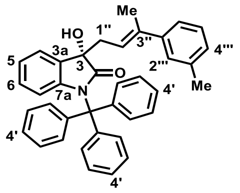

Following **General Procedure G**, allylic ether **S89** (53.5 mg, 0.1 mmol), *t*Bu-BIMP (14.7 mg, 0.02 mmol), 4-NHAc-TEMPO (4.3 mg, 0.02 mmol) in mesitylene (2 mL) gave a crude [1,2]-rearrangement product which was purified by flash column chromatography (eluent: hexane/ EtOAc = 4:1 to 3:1) to afford the product (38.5 mg, 72%) as a colourless amorphous solid. **IR** ν_{max} (film) 3408 (OH), 1713 (C=O), 1600 (C=C), 1489, 1463, 1448, 1319, 1263, 1184, 1107; $[\mathbf{a}]_{\mathbf{D}}^{\mathbf{20}}$ +34.4 (*c* 0.25, $CHCl_3$); **Chiral HPLC analysis**, Chiralpak IA (90:10 hexane:IPA, flow rate 1 ml·min^{-1}, 211 nm, 30 °C) t_R (*R*)-**27**: 9.2 min, t_R (*S*)-**27**: 11.7 min, 6:94 e.r.; **^{1}H NMR (500 MHz, $CDCl_3$)** δ **2.02** (3H, s, C(3``)C*H*$_3$), **2.33** (3H, s, ArC(3```)C*H*$_3$), **2.90–3.05** (2H, m, C(1``)*H*$_2$), **5.58**, (1H, m, C(2``)*H*), **6.26** (1H, d, *J* 8.1, ArC(7)*H*), **6.90** (1H, app td, *J* 8.0, 1.4, ArC(6)*H*), **6.98** (1H, app td, *J* 7.6, 0.7, ArC(5)*H*), **7.07–7.22** (13H, m, ArC(3`,4`,5`,2```,4```,5```,6```)*H*), **7.33–7.39** (6H, m, ArC(2`,6`)*H*), **7.40** (1H, dd, *J* 7.3, 1.1, ArC(4)*H*); **^{13}C{^{1}H} NMR (126 MHz, $CDCl_3$)** δ **16.5** (C(3``)*C*H$_3$), **21.7** (ArC(3```)*C*H$_3$), **39.5** (*C*(1``)H$_2$), **74.7** (N*C*Ph$_3$), **76.4** (*C*(3)OH), **116.2** (Ar*C*(7)H), **119.8** (*C*(2``)H), **122.7** (Ar*C*(5)H), **123.0** (Ar*C*H), **123.4** (Ar*C*(4)H), **126.6** (Ar*C*(4```)), **127.0** (Ar*C*(4`)H), **127.7** (Ar*C*(3`,5`)H), **127.9** (Ar*C*H), **128.2** (Ar*C*(6)H), **128.3** (Ar*C*H), **129.4** (Ar*C*(2`,6`)H), **130.1** (Ar*C*(3a)), **137.9** (Ar*C*(3```)CH$_3$), **139.6** (*C*(3``)Ar), **141.8** (Ar*C*(1`)), **143.3** (Ar*C*(7a)), **143.4** (Ar*C*(1```)), **180.2** (*C*=O); **HRMS (ESI$^+$)** $C_{38}H_{33}NO_2Na$ $[M+Na]^+$ found 558.2389, requires 558.2404 (–2.6 ppm).

(*S*,*E*)-3-Hydroxy-3-[3-(0-tolyl)but-2-en-1-yl]-1-tritylindolin-2-one (28)

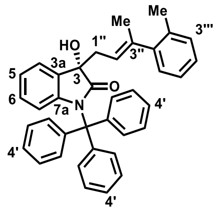

Following **General Procedure G**, allylic ether **S90** (53.5 mg, 0.1 mmol), *t*Bu-BIMP (14.7 mg, 0.02 mmol), 4-NHAc-TEMPO (4.3 mg, 0.02 mmol) in mesitylene (2 mL) gave a crude [1,2]-rearrangement product which was purified by flash column chromatography (eluent: hexane/ EtOAc = 4:1 to 3:1) to afford the product (21.4 mg, 40%) as a colourless amorphous solid. **IR** ν_{max} (film) 3398 (OH), 1717 (C=O), 1610 (C=C), 1598 (C=C), 1489, 1463, 1448, 1276, 1184; $[a]_D^{20}$ +62.0 (*c* 0.25, $CHCl_3$); **Chiral HPLC analysis**, Chiralpak IA (90:10 hexane:IPA, flow rate 1 ml·min^{-1}, 211 nm, 30 °C) t_R (*R*)-**28**: 8.2 min, t_R (*S*)-**28**: 12.2 min, 4:96 e.r.; **^{1}H NMR (500 MHz, $CDCl_3$)** δ **1.88** (3H, s, C(3``)C*H*3), **1.95** (3H, s, ArC(2```)C*H*3), **2.71** (1H, s, O*H*), **2.88** (1H, dd, *J* 13.7, 7.1, C(1``)*H*$^A$H$^B$), **2.97** (1H, dd, *J* 13.7, 8.2, C(1``)H^A*H*^{B}), **5.07–5.19** (1H, m, =C(2``)*H*), **6.27** (1H, d, *J* 8.1, ArC(7)*H*), **6.87–6.93** (2H, m, ArC*H*), **6.96** (1H, app t, *J* 7.4, ArC(5)*H*), **7.06–7.20** (12H, m, ArC*H*), **7.33–7.43** (7H, m, ArC(2`,6`,4)*H*); **$^{13}$C{$^1$H} NMR (126 MHz, $CDCl_3$)** δ **18.6** (C(3``)*C*H3), **19.6** (ArC(2```)*C*H3), **38.9** (*C*(1``)H2), **74.8** (N*C*Ph3), **76.3** (*C*(3)OH), **116.4** (Ar*C*(7)H), **121.2** (*C*(2``)H), **122.7** (Ar*C*(5)H), **123.4** (Ar*C*(4)H), **125.8** (Ar*C*(5```)H), **126.8** (Ar*C*(6```)H), **127.1** (Ar*C*(4`)H), **127.8** (Ar*C*(3`,5`)H), **128.2** (Ar*C*(4```)H), **128.2** (Ar*C*(6)H), **129.4** (Ar*C*(2`,6`)H), **130.0** (Ar*C*(3a)), **130.2** (Ar*C*(3```)H), **134.8** (Ar*C*(2```)CH3), **141.2** (*C*(3``)Ar), **141.9** (Ar*C*(1`)), **143.3** (Ar*C*(7a)), **145.2** (Ar*C*(1```)), **180.0** (*C*=O); **HRMS (ESI$^+$)** $C_{38}H_{33}NO_2Na$ [M+Na]$^+$ found 558.2387, requires 558.2404 (–2.9 ppm).

(*S*,*E*)-3-Hydroxy-3-[3-(naphthalen-2-yl)but-2-en-1-yl]-1-tritylindolin-2-one (29)

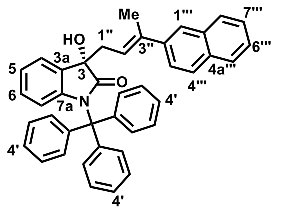

Following **General Procedure G**, allylic ether **S91** (57.1 mg, 0.1 mmol), *t*Bu-BIMP (14.7 mg, 0.02 mmol), 4-NHAc-TEMPO (4.3 mg, 0.02 mmol) in mesitylene (2 mL) gave a crude [1,2]-rearrangement product which was purified by flash column chromatography (eluent: hexane/ EtOAc = 4:1 to 3:1) to afford the product (46.8 mg, 82%) as a colourless amorphous solid. **IR** ν_{max} (film) 3356 (OH), 1715 (C=O), 1610 (C=C), 1597 (C=C), 1463, 1448, 1319, 1267, 1112; $[\mathbf{a}]_{\mathbf{D}}^{\mathbf{20}}$ –4.8 (*c* 0.50, MeOH); **Chiral HPLC analysis**, Chiralcel ODH (90:10 hexane:IPA, flow rate 1 ml·min^{-1}, 211 nm, 30 °C) t_R (*R*)-**29**: 8.2 min, t_R (*S*)-**29**: 11.8 min, 6:94 e.r.; **^{1}H NMR (500 MHz, $CDCl_3$)** δ **2.14** (3H, s, CH_3), **2.33–2.96** (1H, m, O*H*), **3.00** (1H, dd, *J* 13.8, 6.7, C(1``)$H^A$H$^B$), **3.08** (1H, dd, *J* 13.7, 8.9, C(1``)H^AH^B), **5.76–5.86** (1H, m, C(2``)*H*), **6.26** (1H, d, *J* 8.1, ArC(7)*H*), **6.91**(1H, app td, *J* 7.9, 1.5, ArC(6)*H*), **7.00** (1H, app t, *J* 7.5, ArC(5)*H*), **7.06–7.16** (9H, m, ArC(3`,4`,5`)*H*), **7.32–7.40** (6H, m, ArC(2`,6`)*H*), **7.41–7.50** (4H, m, ArC(4`)*H*, ArC*H*), **7.71–7.84** (4H, m, ArC*H*); **^{13}C{^{1}H} NMR (126 MHz, $CDCl_3$)** δ **16.5** (*C*H_3), **39.5** (*C*(1``)$H_2$), **74.7** (N*C*$Ph_3$), **76.4** (*C*(3)OH), **116.3** (Ar*C*(7)H), **120.7** (*C*(2``)H), **122.8** (Ar*C*(5)H), **123.5** (Ar*C*H), **124.3** (Ar*C*H), **124.5** (Ar*C*(4)H), **125.9** (Ar*C*H), **126.3** (Ar*C*H), **127.0** (Ar*C*(4`)H), **127.6** (Ar*C*(3```)H), **127.7** (Ar*C*(3`,5`)H, **127.9** (Ar*C*H), **128.3** (Ar*C*(6)H), **129.4** (Ar*C*(2`,6`)H), **130.0** (Ar*C*(3a)), **132.7** (Ar*C*(4a```)), **133.5** (Ar*C*(8a```)), **139.2** (*C*(3``)Ar), **140.5** (Ar*C*(2```)), **141.8** (Ar*C*(1`)), **143.3** (Ar*C*(7a)), **180.2** (*C*=O); **HRMS (ESI$^+$)** $C_{41}H_{33}NO_2Na$ [M+Na]$^+$ found 594.2393, requires 594.2404 (–1.8 ppm).

(*S*,*E*)-3-Hydroxy-3-[3-(thiophen-2-yl)but-2-en-1-yl]-1-tritylindolin-2-one (30)

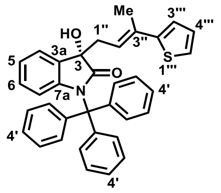

Following **General Procedure G**, allylic ether **S92** (52.7 mg, 0.1 mmol), *t*Bu-BIMP (14.7 mg, 0.02 mmol), 4-NHAc-TEMPO (4.3 mg, 0.02 mmol) in mesitylene (2 mL) gave a crude [1,2]-rearrangement product which was purified by flash column chromatography (eluent: hexane/ EtOAc = 4:1 to 3:1) to afford the product (37.4 mg, 71%) as a colourless amorphous solid. **IR** ν_{max} (film) 3396 (OH), 1713 (C=O), 1610 (C=C), 1600 (C=C), 1463, 1448, 1319, 1271, 1164, 1112; $[a]_D^{20}$ +33.6 (*c* 0.25, $CHCl_3$); **Chiral HPLC analysis**, Chiralpak IA (90:10 hexane:IPA, flow rate 1 ml·min^{-1}, 211 nm, 30 °C) t_R (*R*)-**30**: 11.0 min, t_R (*S*)-**30**: 14.2 min, 6:94 e.r.; **^{1}H NMR (400 MHz, $CDCl_3$)** δ **2.02** (3H, s, C*H*3), **2.64–2.91** (1H, m, O*H*), **2.91–3.02** (2H, m, C(1``)*H*2), **5.74–5.83**, (1H, m, C(2``)*H*), **6.27** (1H, d, *J* 8.1, ArC(7)*H*), **6.91** (1H, app td, *J* 7.9, 1.5, ArC(6)*H*), **6.95–7.02** (3H, m, ArC*H*), **7.10–7.23** (10H, m, ArC*H*), **7.33–7.43** (7H, m, ArC*H*); **^{13}C{^{1}H} NMR (101 MHz, $CDCl_3$)** δ **16.3** (*C*H3), **39.1** (*C*(1``)H2), **74.7** (N*C*Ph3), **76.4** (*C*(3)OH), **116.4** (Ar*C*(7)H), **118.5** (*C*(2``)H), **122.8** (Ar*C*(5)H), **123.1** (Ar*C*(4)H), **123.4** (Ar*C*H), **123.9** (Ar*C*H), **127.0** (Ar*C*(4`)H), **127.5** (Ar*C*H), **127.7** (Ar*C*(3`,5`)H), **128.3** (Ar*C*(6)H), **129.5** (Ar*C*(2`,6`)H), **129.8** (Ar*C*(3a)), **133.3** (*C*(3``)Ar), **141.7** (Ar*C*(1`)), **143.3** (Ar*C*(7a)), **147.2** (ArC(2```)), **180.2** (*C*=O); **HRMS (ESI$^+$)** $C_{35}H_{29}NO_2SNa$ [M+Na]$^+$ found 550.1798, requires 550.1811 (–2.4 ppm)..

(*S*,*E*)-3-Hydroxy-3-[3-(thiazol-2-yl)but-2-en-1-yl]-1-tritylindolin-2-one (31)

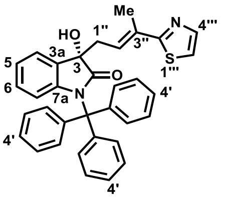

Following **General Procedure G**, allylic ether **S93** (52.8 mg, 0.1 mmol), *t*Bu-BIMP (14.7 mg, 0.02 mmol), 4-NHAc-TEMPO (4.3 mg, 0.02 mmol) in mesitylene (2 mL) gave a crude [1,2]-rearrangement product which was purified by flash column chromatography (eluent: hexane/ EtOAc = 4:1 to 3:1) to afford the product (37.5 mg, 71%) as a colourless amorphous solid. **IR** ν_{max} (film) 3360 (OH), 1726 (C=O), 1610 (C=C), 1600 (C=C), 1489, 1463, 1448, 1267, 1112; $\mathbf{[a]_D^{20}}$ +37.6 (*c* 0.25, $CHCl_3$); **Chiral HPLC analysis**, Chiralpak IA (90:10 hexane:IPA, flow rate 1 ml·min^{-1}, 211 nm, 30 °C) t_R (*R*)-**31**: 13.2 min, t_R (*S*)-**31**: 16.2 min, 7:93 e.r.; **^{1}H NMR (400 MHz, $CDCl_3$)** δ **2.13** (3H, s, C*H*3), **2.71–3.27** (3H, m, C(1``)*H*2 and O*H*), **6.28** (1H, d, *J* 8.0, ArC(7)*H*), **6.33–6.41**, (1H, m, C(2``)*H*), **6.91** (1H, app td, *J* 7.9, 1.5, ArC(6)*H*), **6.98** (1H, dd, *J* 7.5, 1.0, ArC(5)*H*), **7.09–7.22** (10H, m, ArC(3`,4`,5`,5```)*H*), **7.30–7.38** (6H, m, ArC(2`,6`)*H*), **7.40** (1H, dd, *J* 7.3, 1.5, ArC(4)*H*), **7.74** (1H, d, *J* 3.3, ArC(4```)*H*); **^{13}C{^{1}H} NMR (101 MHz, $CDCl_3$)** δ **15.5** (*C*H3), **39.1** (*C*(1``)H2), **74.7** (N*C*Ph3), **76.1** (*C*(3)OH), **116.4** (Ar*C*(7)H), **118.2** (Ar*C*(5```)H), **122.9** (Ar*C*(5)H), **123.4** (Ar*C*(4)H), **124.5** (*C*(2``)H), **127.0** (Ar*C*(4`)H), **127.8** (Ar*C*(3`,5`)H), **128.4** (Ar*C*(6)H), **129.4** (Ar*C*(2`,6`)H), **129.7** (Ar*C*(3a)), **134.0**, (*C*(3``)Ar), **141.7** (Ar*C*(1`)), **143.2** (Ar*C*(7a)), **171.0** (Ar*C*(2```)), **179.9** (*C*=O); **HRMS (ESI$^+$)** $C_{34}H_{28}N_2O_2SNa$ [M+Na]$^+$ found 551.1745, requires 551.1764 (–3.4 ppm).

(*S*,*E*)-4-Chloro-3-hydroxy-3-(3-phenylbut-2-en-1-yl)-1-tritylindolin-2-one (32)

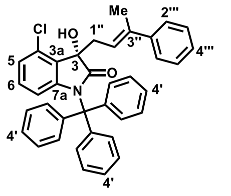

Following **General Procedure G**, allylic ether **S94** (55.5 mg, 0.1 mmol), *t*Bu-BIMP (14.7 mg, 0.02 mmol), 4-NHAc-TEMPO (4.3 mg, 0.02 mmol) in mesitylene (2 mL) gave a crude [1,2]-rearrangement product which was purified by flash column chromatography (eluent: hexane/ EtOAc = 4:1 to 3:1) to afford the product (38.9 mg, 70%) as a colourless amorphous solid. **IR** ν_{max} (film) 3412 (OH), 1732 (C=O), 1598 (C=C), 1490, 1463, 1444, 1265, 1242, 1141 (C-Cl); $[\mathbf{a}]_{\mathbf{D}}^{\mathbf{20}}$ +65.2 (*c* 0.25, $CHCl_3$); **Chiral HPLC analysis**, Chiralcel OD-H (95:5 hexane:IPA, flow rate 1 ml·min^{-1}, 211 nm, 30 °C) t_R (*R*)-**32**: 6.5 min, t_R (*S*)-**32**: 8.2 min, 8:92 e.r.; **^{1}H NMR (500 MHz, $CDCl_3$)** δ **2.08** (3H, s, C*H*$_3$), **2.83** (1H, s, O*H*), **3.07** (1H, dd, *J* 13.5, 9.0 C(1``)*H*$^A$H$^B$), **3.36–3.53** (1H, m, C(1``)H^A*H*^{B}), **5.57** (1H, ddq, *J* 8.4, 6.9, 1.4, =C(2``)*H*), **6.16** (1H, dd, *J* 8.2, 0.8, ArC(7)*H*), **6.81** (1H, app t, *J* 8.2, ArC(6)*H*), **6.93** (1H, dd, *J* 8.2, 0.8, ArC(5)*H*), **7.08–7.13** (6H, m, ArC*H*), **7.13–7.17** (3H, m, ArC*H*), **7.24–7.32** (11H, m, ArC*H*); **^{13}C{^{1}H} NMR (126 MHz, $CDCl_3$)** δ **16.4** (*C*H$_3$), **37.0** (*C*(1``)H$_2$), **75.1** (N*C*Ph$_3$), **77.3** (*C*(3)OH), **115.0** (Ar*C*(7)H), **119.6** (*C*(2``)H), **123.8** (Ar*C*(5)H), **125.9** (Ar*C*(2```,6```)H), **126.3** (Ar*C*(3a)), **127.2** (Ar*C*(4`)*H*), **127.3** (Ar*C*(4```)H), **127.8** (Ar*C*(3`,5`)H), **128.5** (Ar*C*(3```,5```)H), **129.2** (Ar*C*(6)H), **129.5** (Ar*C*(2`,6`)H), **130.9** (Ar*C*(4)Cl), **139.8** (*C*(3``)Ar), **141.4** (Ar*C*(1`)), **143.3** (Ar*C*(1```)), **145.4** (Ar*C*(7a)), **179.2** (*C*=O); **HRMS (ESI$^+$)** $C_{37}H_{30}{}^{35}ClNO_2Na$ $[M+Na]^+$ found 578.1841, requires 578.1857 (–2.8 ppm).

(*S*,*E*)-5-Fluoro-3-hydroxy-3-(3-phenylbut-2-en-1-yl)-1-tritylindolin-2-one (17)

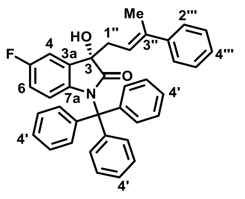

Following **General Procedure G**, allylic ether **15** (53.9 mg, 0.1 mmol), *t*Bu-BIMP (14.7 mg, 0.02 mmol), 4-NHAc-TEMPO (4.3 mg, 0.02 mmol) in mesitylene (2 mL) gave a crude [1,2]-rearrangement product which was purified by flash column chromatography (eluent: hexane/ EtOAc = 4:1 to 3:1) to afford the product (38.8 mg, 72%) as a colourless amorphous solid. **IR** ν_{max} (film) 3392(OH), 1717 (C=O), 1610 (C=C), 1475, 1448, 1263, 1184, 1153, 1120; $[\mathbf{a}]_{\mathbf{D}}^{\mathbf{20}}$ +32.4 (*c* 0.25, $CHCl_3$); **Chiral HPLC analysis**, Chiralpak IA (90:10 hexane:IPA, flow rate 1 ml·min^{-1}, 211 nm, 30 °C) t_R (*R*)-**17**: 8.7 min, t_R (*S*)-**17**: 10.7 min, 7:93 e.r.; **^{1}H NMR** (500 MHz, $CDCl_3$) δ 2.05 (3H, s,C*H*3), 2.51–3.17 (3H, m, C(1``)*H*2, O*H*), 5.56–5.64, (1H, m, C(2``)*H*), 6.17 (1H, dd, *J* 8.9, 4.2, ArC(7)*H*), 6.60 (1H, app td, *J* 9.0, 2.8, ArC(6)*H*), 7.07–7.21 (10H, m, ArC*H*), 7.25–7.34 (11H, m, ArC*H*); **^{13}C{^{1}H} NMR** (126 MHz, $CDCl_3$) δ 16.5 (*C*H3), 39.5 (*C*(1``)H2), 74.8 (N*C*Ph3), 76.5 (*C*(3)OH), 111.2 (d, $^2J_{C\text{-}F}$ 23.9, Ar*C*(4)H), 114.7 (d, $^2J_{C\text{-}F}$ 22.7, Ar*C*(6)H), 117.1 (d, $^3J_{C\text{-}F}$ 7.6, Ar*C*(7)H), 119.5 (*C*(2``)H), 125.8 (Ar*C*(2```,6```)H), 127.2 (Ar*C*(4`)H), 127.3 (Ar*C*(4```)H), 127.8 (Ar*C*(3`,5`)H), 128.5 (Ar*C*(3```,5```)H), 129.4 (Ar*C*(2`,6`)H), 131.8 (d, $^3J_{C\text{-}F}$ 7.6, Ar*C*(3a)), 139.0 (Ar*C*(7a)), 139.7 (*C*(3``)Ar), 141.4 (Ar*C*(1`)), 143.1 (Ar*C*(1```)), 158.9 (d, $^1J_{C\text{-}F}$ 243.2, ArC(5)F), 179.9 (*C*=O); **^{19}F{^{1}H} NMR** (470 MHz, $CDCl_3$) δ –120.17 (ArC(5)*F*); **HRMS (ESI$^+$)** $C_{37}H_{30}FNO_2Na$ $[M+Na]^+$ found 562.2152, requires 562.2153 (–0.2 ppm).

(*S*,*E*)-5-Chloro-3-hydroxy-3-(3-phenylbut-2-en-1-yl)-1-tritylindolin-2-one (33)

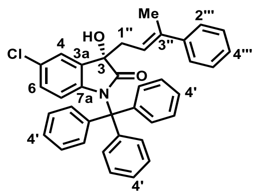

Following **General Procedure G**, allylic ether **S95** (55.5 mg, 0.1 mmol), *t*Bu-BIMP (14.7 mg, 0.02 mmol), 4-NHAc-TEMPO (4.3 mg, 0.02 mmol) in mesitylene (2 mL) gave a crude [1,2]-rearrangement product which was purified by flash column chromatography (eluent: hexane/ EtOAc = 4:1 to 3:1) to afford the product (44.4 mg, 80%) as a colourless amorphous solid. **IR** ν_{max} (film) 3379 (OH), 1717 (C=O), 1593 (C=C), 1490, 1467, 1448, 1319, 1257, 1163, 1126; $[\mathbf{a}]_{\mathbf{D}}^{\mathbf{20}}$ +96.0 (*c* 0.25, $CHCl_3$); **Chiral HPLC analysis**, Chiralpak IA (90:10 hexane:IPA, flow rate 1 ml·min^{-1}, 211 nm, 30 °C) t_R (*R*)-**33**: 8.9 min, t_R (*S*)-**33**: 10.9 min, 5:95 e.r.; **^{1}H NMR (300 MHz, $CDCl_3$)** δ **2.06** (3H, s, ArCC*H*3), **2.40–2.82** (1H, br, O*H*), **2.87–2.96** (1H, m, C(1``)$H^AH^B$), **3.03** (1H, dd, *J* 13.6, 9.2, C(1``)H^AH^B), **5.63** (1H, ddd, *J* 9.0, 6.5, 1.3, CH_2C(2``)*H*), **6.18** (1H, d, *J* 8.7, ArC(7)*H*), **6.88** (1H, dd, *J* 8.7, 2.3, ArC(6)*H*), **7.10–7.21** (9H, m, ArC*H*), **7.26–7.35** (11H, m, ArC*H*), **7.41** (1H, d, *J* 2.2, ArC(4)*H*); **$^{13}$C{$^1$H} NMR (75 MHz, $CDCl_3$)** δ **16.5** (ArC*C*$H_3$), **39.5** (*C*(1``)H_2), **74.8** (N*C*Ph_3), **76.4** (*C*(3)OH), **117.2** (Ar*C*(7)H), **119.4** (*C*(2``)H), **123.8** (Ar*C*(4)H), **125.8** (Ar*C*(2```,6```)H), **127.2** (Ar*C*(4`)*H*), **127.3** (Ar*C*(4`)H), **127.8** (Ar*C*(3`,5`)H), **128.2** (Ar*C*(6)H), **128.4** (Ar*C*(5)Cl), **128.5** (Ar*C*(3```,5```)H), **129.3** (Ar*C*(2`,6`)H), **131.7** (Ar*C*(3a)), **139.9** (*C*(3``)Ar), **141.4** (Ar*C*(1`)), **141.7** (Ar*C*(7a)), **143.1** (Ar*C*(1```)), **179.7** (*C*=O); **HRMS (ESI$^+$)** $C_{37}H_{30}{}^{35}ClNO_2Na$ [M+Na]$^+$ found 578.1851, requires 578.1857 (–1.1 ppm).

(*S*,*E*)-3-Hydroxy-5-iodo-3-(3-phenylbut-2-en-1-yl)-1-tritylindolin-2-one (34)

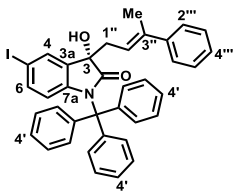

Following **General Procedure G**, allylic ether **S96** (64.7 mg, 0.1 mmol), *t*Bu-BIMP (14.7 mg, 0.02 mmol), 4-NHAc-TEMPO (4.3 mg, 0.02 mmol) in mesitylene (2 mL) gave a crude [1,2]-rearrangement product which was purified by flash column chromatography (eluent: hexane/ EtOAc = 4:1 to 3:1) to afford the product (47.2 mg, 73%) as a colourless amorphous solid. **IR** ν_{max} (film) 3387 (OH), 1716 (C=O), 1597 (C=C), 1490, 1465, 1448, 1309, 1263, 1180, 1166, 1126, 1085; $[\mathbf{a}]_{\mathbf{D}}^{\mathbf{20}}$ +170.4 (*c* 0.25, $CHCl_3$); **Chiral HPLC analysis**, Chiralpak IA (90:10 hexane:IPA, flow rate 1 ml·min^{-1}, 211 nm, 30 °C) t_R (*R*)-**34**: 9.8 min, t_R (*S*)-**34**: 11.8 min, 4:96 e.r.; **^{1}H NMR (500 MHz, $CDCl_3$)** δ **2.03** (3H, s, C*H*$_3$), **2.65–2.85** (1H, br s, O*H*), **2.87–2.93** (1H, m, C(1``)*H*$^A$H$^B$), **3.00** (1H, dd, *J* 13.6, 9.2, C(1``)H^A*H*^{B}), **5.56–5.63** (1H, m, C(2``)*H*), **6.00** (1H, d, *J* 8.6, ArC(7)*H*), **7.10–7.18** (9H, m, ArC*H*), **7.20** (1H, dd, *J* 8.6, 1.9, ArC(6)*H*), **7.26–7.34** (11H, m, ArC*H*), **7.69** (1H, d, *J* 1.9, ArC(4)*H*); **$^{13}$C{$^1$H} NMR (126 MHz, $CDCl_3$)** δ **16.5** (*C*H$_3$), **39.45**(*C*(1``)H$_2$), **74.8** (N*C*Ph$_3$), **76.2** (*C*(3)OH), **86.1** (Ar*C*(5)I), **118.2** (Ar*C*(7)H), **119.4** (*C*(2``)H), **125.9** (Ar*C*(2```,6```)H), **127.2** (Ar*C*(4`)H), **127.4** (Ar*C*(4```)H), **127.8** (Ar*C*(3`,5`)H), **128.5** (Ar*C*(3```,5```)H), **129.3** (Ar*C*(2`,6`)H), **132.3** (Ar*C*(4)H), **132.4** (Ar*C*(3a)), **137.1** (Ar*C*(6)H), **139.9** (*C*(3``)Ar), **141.3** (Ar*C*(1`)), **143.0** (Ar*C*(7a)), **143.1** (*C*(1```)Ar), **179.4** (*C*=O); **HRMS (ESI$^+$)** $C_{37}H_{30}INO_2Na$ $[M+Na]^+$ found 670.1202, requires 670.1213 (–1.7 ppm).

(*S*,*E*)-3-Hydroxy-5-nitro-3-(3-phenylbut-2-en-1-yl)-1-tritylindolin-2-one (35)

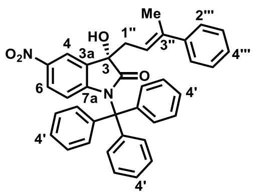

Following **General Procedure G**, allylic ether **S97** (56.6 mg, 0.1 mmol), *t*Bu-BIMP (14.7 mg, 0.02 mmol), 4-NHAc-TEMPO (4.3 mg, 0.02 mmol) in mesitylene (2 mL) gave a crude [1,2]-rearrangement product which was purified by flash column chromatography (eluent: hexane/ EtOAc = 4:1 to 3:1) to afford the product (43.0 mg, 76%) as a colourless amorphous solid. **IR** ν_{max} (film) 3367 (OH), 1728 (C=O), 1610 (C=C), 1517 (NO_2), 1490, 1469, 1448, 1338 (NO_2), 1271, 1163, 1076; $[\mathbf{a}]_{\mathbf{D}}^{\mathbf{20}}$ +145.6 (*c* 0.25, $CHCl_3$); **Chiral HPLC analysis**, Chiralpak IA (90:10 hexane:IPA, flow rate 1 ml·min^{-1}, 211 nm, 30 °C) t_R (*R*)-**35**: 9.0 min, t_R (*S*)-**35**: 12.0 min, 7:93 e.r.; **^{1}H NMR (500 MHz, $CDCl_3$)** δ **2.05** (3H, s, C*H*$_3$), **2.64–3.45** (3H, m, C(1``)*H*$_2$ and O*H*), **5.52–5.59** (1H, m, C(2``)*H*), **6.35** (1H, d, *J* 9.0, ArC(7)*H*), **7.12–7.21** (9H, m, ArC*H*), **7.32–7.26** (11H, m, ArC*H*), **7.83** (1H, dd, *J* 9.0, 2.5, ArC(6)*H*), **8.27** (1H, d, *J* 2.5, ArC(4)*H*); **^{13}C{^{1}H} NMR (126 MHz, $CDCl_3$)** δ **16.5** (*C*H_3), **39.3** (*C*(1``)$H_2$), **75.4** (N*C*$Ph_3$), **76.0** (*C*(3)OH), **115.9** (Ar*C*(7)H), **118.6** (*C*(2``)H), **119.1** (Ar*C*(4)H), **124.7** (Ar*C*(6)H), **125.8** (Ar*C*(3```,5```)H), **127.5** (Ar*C*(4`)H), **127.6** (Ar*C*(4```)H), **128.0** (Ar*C*(3`,5`)H), **128.6** (Ar*C*(2```,6```)H), **129.2** (Ar*C*(2`,6`)H), **130.1** (Ar*C*(3a)), **140.7** (*C*(3``)Ar), **140.9** (Ar*C*(1`)), **142.9** (Ar*C*(1```)), **143.2** (Ar*C*(7a)), **149.2** (Ar*C*(5)NO_2), **180.2** (*C*=O); **HRMS (ESI$^+$)** $C_{37}H_{30}N_2O_4Na$ $[M+Na]^+$ found 589.2082, requires 589.2098 (–2.7 ppm).

(*S*,*E*)-3-Hydroxy-5-methyl-3-(3-phenylbut-2-en-1-yl)-1-tritylindolin-2-one (36)

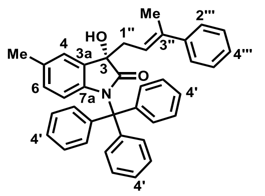

Following **General Procedure G**, allylic ether **S98** (53.5 mg, 0.1 mmol), *t*Bu-BIMP (14.7 mg, 0.02 mmol), 4-NHAc-TEMPO (4.3 mg, 0.02 mmol) in mesitylene (2 mL) gave a crude [1,2]-rearrangement product which was purified by flash column chromatography (eluent: hexane/ EtOAc = 4:1 to 3:1) to afford the product (36.9 mg, 69%) as a colourless amorphous solid. **IR** ν_{max} (film) 3400 (OH), 1712 (C=O), 1597 (C=C), 1489, 1448, 1321, 1263, 1192, 1132, 1083; $[\mathbf{a}]_{\mathbf{D}}^{\mathbf{20}}$ +84.4 (*c* 0.25, $CHCl_3$); **Chiral HPLC analysis**, Chiralpak IA (90:10 hexane:IPA, flow rate 1 ml·min^{-1}, 211 nm, 30 °C) t_R (*R*)-**36**: 9.1 min, t_R (*S*)-**36**: 11.1 min, 5:95 e.r.; **^{1}H NMR (300 MHz, $CDCl_3$)** δ **2.06** (3H, s, =C(3``)C*H*$_3$), **2.26** (3H, s, ArC(5)C*H*$_3$), **2.36**–**2.72** (1H, br s, O*H*), **2.86**–**2.97** (1H, m, C(1``)*H*^{A}H^B), **3.03** (1H, dd, *J* 13.7, 9.1, C(1``)H$^A$*H*$^B$), **5.53**–**5.69** (1H, m, C(2``)*H*), **6.13** (1H, d, *J* 8.3, ArC(7)*H*), **6.71** (1H, dd, *J* 8.3, 1.2, ArC(6)*H*), **7.09**–**7.20** (9H, m, ArC*H*), **7.23** (1H, d, *J* 1.8, ArC(4)*H*), **7.26**–**7.41** (11H, m, ArC*H*); **^{13}C{^{1}H} NMR (75 MHz, $CDCl_3$)** δ **16.5** (C(3``)*C*H$_3$), **21.0** (ArC(5)*C*H$_3$), **39.5** (*C*(1``)H$_2$), **74.5** (N*C*Ph$_3$), **76.4** (*C*(3)OH), **116.0** (Ar*C*(7)H), **120.2** (*C*(2``)H), **124.0** (Ar*C*(4)H), **125.8** (Ar*C*(3```,5```)H), **127.0** (Ar*C*(4`)H), **127.2** (Ar*C*(3`,5`)H), **127.7** (Ar*C*(4```)H), **128.4** (Ar*C*(2```,6```)H), **128.6** (Ar*C*(6)H), **129.4** (Ar*C*(2`,6`)H), **129.9** (Ar*C*(3a)), **132.3** (Ar*C*(5)CH$_3$), **139.3** (*C*(3``)Ar), **140.8** (Ar*C*(7a)), **141.8** (Ar*C*(1`)), **143.4** (Ar*C*(1```)), **180.2** (*C*=O); **HRMS (ESI$^+$)** $C_{38}H_{33}NO_2Na$ [M+Na]$^+$ found 558.2395, requires 558.2404 (–1.6 ppm).

(*S*,*E*)-3-Hydroxy-5-methoxy-3-(3-phenylbut-2-en-1-yl)-1-tritylindolin-2-one (37)

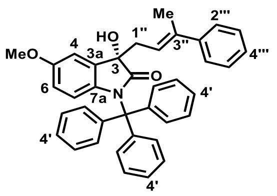

Following **General Procedure G**, allylic ether **S99** (55.1 mg, 0.1 mmol), *t*Bu-BIMP (14.7 mg, 0.02 mmol), 4-NHAc-TEMPO (4.3 mg, 0.02 mmol) in mesitylene (2 mL) gave a crude [1,2]-rearrangement product which was purified by flash column chromatography (eluent: hexane/ EtOAc = 4:1 to 3:1) to afford the product (36.9 mg, 67%) as a colourless amorphous solid. **IR** ν_{max} (film) 3400 (OH), 1711 (C=O), 1597 (C=C), 1483, 1448, 1436, 1276, 1195, 1186, 1161 (C-O), 1083; $\mathbf{[a]_D^{20}}$ +86.8 (*c* 0.25, $CHCl_3$); **Chiral HPLC analysis**, Chiralpak IA (90:10 hexane:IPA, flow rate 1 ml·min^{-1}, 211 nm, 30 °C) t_R (*R*)-**37**: 11.6 min, t_R (*S*)-**37**: 14.3 min, 5:95 e.r.; **^{1}H NMR (300 MHz, $CDCl_3$)** δ **2.06** (3H, s, C(3``)C*H*$_3$), **2.34–2.85** (1H, br s, O*H*), **2.85–2.91** (1H, dd, *J* 14.0, 6.0, C(1``)H^AH^B), **3.02** (1H, dd, *J* 13.6, 9.1, C(1``)$H^AH^B$), **3.71** (3H, s, OC*H*$_3$), **5.55–5.68** (1H, m, C(2``)*H*), **6.14** (1H, d, *J* 8.9, ArC(7)*H*), **6.44** (1H, dd, *J* 8.9, 2.8, ArC(6)*H*), **6.99** (1H, d, *J* 2.7, ArC(4)*H*), **7.08–7.18** (9H, m, ArC*H*), **7.26–7.42** (11H, m, ArC*H*); **^{13}C{^{1}H} NMR (75 MHz, $CDCl_3$)** δ **16.5** (C(3``)*C*H$_3$), **39.6** (*C*(1``)H$_2$), **55.7** (O*C*H$_3$), **74.6** (N*C*Ph$_3$), **76.6** (*C*(3)OH), **109.4** (Ar*C*(4)H), **113.5** (Ar*C*(6)H), **117.0** (Ar*C*(7)H), **120.0** (*C*(2``)H), **125.8** (Ar*C*(3```,5```)H), **127.0** (Ar*C*(4`)H), **127.2** (Ar*C*(3`,5`)H), **127.7** (Ar*C*(4```)H), **128.5** (Ar*C*(2```,6```)H), **129.4** (Ar*C*(2`,6`)H), **131.2** (Ar*C*(3a)), **136.3** (Ar*C*(7a)), **139.4** (*C*(3``)Ar), **141.8** (Ar*C*(1`)), **143.3** (Ar*C*(1```)), **155.7** (Ar*C*(5)OMe), **180.0** (*C*=O); **HRMS (ESI$^+$)** $C_{38}H_{33}NO_3Na$ $[M+Na]^+$ found 574.2345, requires 574.2353 (–1.4 ppm).

(*S*,*E*)-6-Chloro-3-hydroxy-3-(3-phenylbut-2-en-1-yl)-1-tritylindolin-2-one (38)

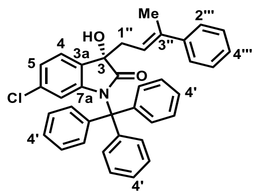

Following **General Procedure G**, allylic ether **S100** (55.5 mg, 0.1 mmol), *t*Bu-BIMP (14.7 mg, 0.02 mmol), 4-NHAc-TEMPO (4.3 mg, 0.02 mmol) in mesitylene (2 mL) gave a crude [1,2]-rearrangement product which was purified by flash column chromatography (eluent: hexane/ EtOAc = 4:1 to 3:1) to afford the product (41.1 mg, 74%) as a colourless amorphous solid. **IR** ν_{max} (film) 3383 (OH), 1717 (C=O), 1608 (C=C), 1587, 1490, 1477, 1448, 1419, 1330, 1280, 1263, 1161, 1112 (C-Cl), 1076; $\mathbf{[a]_D^{20}}$ +65.6 (*c* 0.25, $CHCl_3$); **Chiral HPLC analysis**, Chiralpak IA (90:10 hexane:IPA, flow rate 1 ml·min^{-1}, 211 nm, 30 °C) t_R (*R*)-**38**: 9.4 min, t_R (*S*)-**38**: 11.5 min, 6:94 e.r.; **^{1}H NMR (300 MHz, $CDCl_3$)** δ **2.04** (3H, s, C*H*3), **2.65–3.25** (3H, m, C(1``)*H*2, O*H*), **5.60**, (1H, app t, *J* 7.2, C(2``)*H*), **6.21** (1H, d, *J* 1.6, ArC(7)*H*), **6.96** (1H, dd, *J* 7.9, 1.7, ArC(5)*H*), **7.11–7.23** (9H, m, ArC*H*), **7.27–7.39** (12H, m, ArC*H*); **^{13}C{^{1}H} NMR (75 MHz, $CDCl_3$)** δ **16.5** (*C*H3), **39.3** (*C*(1``)H2), **74.9** (N*C*Ph3), **76.0** (*C*(3)OH), **116.6** (Ar*C*(7)H), **119.5** (*C*(2``)H), **122.8** (Ar*C*(5)H), **124.2** (Ar*C*(4)H), **125.8** (Ar*C*(3```,5```)H), **127.2** (Ar*C*(4`)H), **127.4** (Ar*C*(4```)H), **127.9** (Ar*C*(3`,5`)H), **128.4** (Ar*C*(3a)), **128.5** (Ar*C*(2```,6```)H), **129.3** (Ar*C*(2`,6`)H), **133.9** (Ar*C*(6)Cl), **139.8** (*C*(3``)Ar), **141.3** (ArC(1`)), **143.2** (Ar*C*(4```)), **144.4** (Ar*C*(7a)), **180.0** (*C*=O); **HRMS (ESI$^+$)** $C_{37}H_{30}{}^{35}ClNO_2Na$ [M+Na]$^+$ found 578.1852, requires 578.1857 (–0.9 ppm).

(*S*,*E*)-6-Bromo-3-hydroxy-3-(3-phenylbut-2-en-1-yl)-1-tritylindolin-2-one (39)

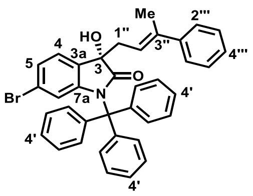

Following **General Procedure G**, allylic ether **S101** (59.9 mg, 0.1 mmol), *t*Bu-BIMP (14.7 mg, 0.02 mmol), 4-NHAc-TEMPO (4.3 mg, 0.02 mmol) in mesitylene (2 mL) gave a crude [1,2]-rearrangement product which was purified by flash column chromatography (eluent: hexane/ EtOAc = 4:1 to 3:1) to afford the product (44.3 mg, 74%) as a colourless amorphous solid. **IR** ν_{max} (film) 3365 (OH), 1716 (C=O), 1606 (C=C), 1585 (C=C), 1490, 1473, 1448, 1415, 1328, 1263, 1184, 1114, 1066 (C-Br); $\mathbf{[a]_D^{20}}$ +66.8 (*c* 0.25, $CHCl_3$); **Chiral HPLC analysis**, Chiralpak IA (90:10 hexane:IPA, flow rate 1 ml·min^{-1}, 211 nm, 30 °C) t_R (*R*)-**39**: 9.8 min, t_R (*S*)-**39**: 11.7 min, 6:94 e.r.; **^{1}H NMR (500 MHz, $CDCl_3$)** δ **2.03** (3H, s, C*H*$_3$), **2.44–3.17** (3H, m, C(1``)*H*$_2$, O*H*), **5.60**, (1H, app t, *J* 7.5, C(2``)*H*), **6.30–6.37** (1H, d, *J* 1.1, ArC(7)*H*), **7.12** (1H, dd, *J* 7.9, 1.3, ArC(5)*H*), **7.14–7.22** (9H, m, ArC*H*), **7.22–7.31** (3H, m, ArC*H*), **7.31–7.36** (9H, m, ArC*H*); **^{13}C{^{1}H} NMR (126 MHz, $CDCl_3$)** δ **16.5** (*C*H$_3$), **39.3** (*C*(1``)H$_2$), **74.9** (N*C*Ph$_3$), **76.0** (*C*(3)OH), **119.3** (Ar*C*(7)H), **119.4** (*C*(2``)H), **121.9** (Ar*C*(6)Br), **124.6** (Ar*C*(4)H), **125.7** (Ar*C*(5)H), **125.8** (Ar*C*(3```,5```)H), **127.2** (Ar*C*(4`)H), **127.4** (Ar*C*(4```)H), **127.9** (Ar*C*(3`,5`)H), **128.5** (Ar*C*(2```,6```)H), **129.0** (Ar*C*(3a)), **129.3** (Ar*C*(2`,6`)H), **139.8** (*C*(3``)Ar), **141.2** (ArC(1`)), **143.2** (Ar*C*(1```)), **144.5** (Ar*C*(7a)), **179.9** (*C*=O); **HRMS (ESI$^+$)** $C_{37}H_{30}{}^{79}BrNO_2Na$ $[M+Na]^+$ found 622.1336, requires 622.1352 (–2.6 ppm).

(*S*,*E*)-1-Benzyl-7-chloro-3-hydroxy-3-(3-phenylbut-2-en-1-yl)indolin-2-one (40)

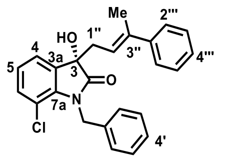

Following **General Procedure G**, allylic ether **S102** (40.3 mg, 0.1 mmol), *t*Bu-BIMP (14.7 mg, 0.02 mmol), 4-NHAc-TEMPO (4.3 mg, 0.02 mmol) in mesitylene (2 mL) gave a crude [1,2]-rearrangement product which was purified by flash column chromatography (eluent: hexane/ EtOAc = 4:1 to 3:1) to afford the product (32.2 mg, 81%) as a colourless amorphous solid. **IR** ν_{max} (film) 3365 (OH), 1705 (C=O), 1610 (C=C), 1585 (C=C), 1494, 1452, 1352, 1163, 1130, 1072 (C-Cl), 1024; $[\mathbf{a}]_\mathbf{D}^{\mathbf{20}}$ –35.6 (*c* 0.25, $CHCl_3$); **Chiral HPLC analysis**, Chiralpak IA (90:10 hexane:IPA, flow rate 1 ml·min^{-1}, 211 nm, 30 °C) t_R (*R*)-40: 11.0 min, t_R (*S*)-40: 15.4 min, 27:73 e.r.; **^{1}H NMR (500 MHz, $CDCl_3$)** δ **1.95** (3H, s, CH_3), **2.93** (1H, dd, *J* 13.8, 6.9, C(1``)$H^A$$H^B$), **3.00** (1H, dd, *J* 13.9, 8.7, C(1``)$H^A$$H^B$), **3.04–3.28** (1H, m, O*H*), **5.21–5.39** (2H, m, NCH_2Ph), **5.50–5.61** (1H, m, C(2``)*H*), **7.03** (1H, dd, *J* 8.2, 7.3, ArC(5)*H*), **7.06–7.12** (2H, m, ArC(2```,6```)*H*), **7.12–7.18** (3H, m, ArC(2`,6`,6)*H*), **7.19–7.30** (6H, m, ArC(3`,3```,4`,4```,5`,5```)*H*), **7.38** (1H, dd, *J* 7.3, 1.2, ArC(4)*H*); **^{13}C{^{1}H} NMR (126 MHz, $CDCl_3$)** δ **16.4** (*C*H_3), **38.5** (*C*(1``)$H_2$), **45.1** (N$CH_2$Ph), **75.9** (*C*(3)OH), **116.0** (Ar*C*(7)Cl), **118.9** (*C*(2``)H), **122.9** (Ar*C*(4)H), **124.3** (Ar*C*(5)H), **126.0** (Ar*C*(3```,5```)H), **126.4** (Ar*C*(2`,6`)H), **127.2** (Ar*C*(4`)H), **127.3** (Ar*C*(4```)H), **128.4** (Ar*C*(3`,5`)H), **128.7** (Ar*C*(2```,6```)H), **132.4** (Ar*C*(6)H), **132.9** (Ar*C*(3a)), **137.2** (Ar*C*(7a)), **138.9** (ArC(1`)), **140.3** (*C*(3``)Ar), **143.3** (Ar*C*(1```)), **178.8** (*C*=O); **HRMS (ESI$^+$)** $C_{25}H_{22}{}^{35}ClNO_2Na$ $[M+Na]^+$ found 626.1230, requires 426.1231 (–0.4 ppm).

(*S*,*Z*)-3-(3-fluoro-3-phenylallyl)-3-hydroxy-1-tritylindolin-2-one (46)

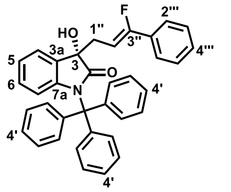

Following **General Procedure H**, allylic ether **41** (52.5 mg, 0.1 mmol), *t*Bu-BIMP (7.4 mg, 0.01 mmol), in toluene (2 mL) for 15 h and then the reaction mixture was treated with DBU (0.10 mmol) at 100 °C for 15 h to give a crude formal [1,2]-rearrangement product which was purified by flash column chromatography (eluent: hexane/ EtOAc = 4:1 to 3:1) to afford the product (26.3 mg, 50%) as a pale yellow oil. **IR** ν_{max} (film) 3350 (OH), 2974 (C-H), 1717 (C=O), 1610 (C=C), 1598 (C=C), 1463, 1448, 1317, 1265, 1182 (C-F), 1114; $[\mathbf{a}]_{\mathbf{D}}^{\mathbf{20}}$ +8.4 (*c* 0.25, $CHCl_3$); **Chiral HPLC analysis** Chiralpak ADH (90:10 hexane:IPA, flow rate 1 ml·min^{-1}, 211 nm, 30 °C) t_R (*R*)-**46**: 13.2 min, t_R (*S*)-**46**: 14.7 min, 5:95 e.r.; **^{1}H NMR (400 MHz, $CDCl_3$)** δ **2.81** (1H, s, O*H*), **2.96** (1H, ddd, *J* 13.9, 7.2, 2.0, C(1``)$H^AH^B$), **3.07** (1H, ddd, *J* 13.9, 8.7, 1.2, C(1``)H^AH^B), **5.35** (1H, ddd, *J* 35.7, 8.7, 7.1, C(2``)*H*), **6.29** (1H, d, *J* 8.0, ArC(7)*H*), **6.91** (1H, app td, *J* 7.8, 1.6, ArC(6)*H*), **6.98** (1H, app td, *J* 7.5, 1.0, ArC(5)*H*), **7.13–7.21** (9H, m, ArC(3`,4`,5`)*H*), **7.32–7.43** (10H, m, ArC(4,2`,6`,3```,4```,5```)*H*), **7.42–7.50** (2H, m, ArC(2```,6```)*H*); **^{13}C{^{1}H} NMR (101 MHz, $CDCl_3$)** δ **34.9** (*C*(1``)$H_2$), **74.6** (N*C*$Ph_3$), **75.9** (*C*(3)OH), **98.4** (d, $^2J_{C\text{-}F}$ 16.4, *C*(2``)H), **116.3** (Ar*C*(7)H), **122.8** (Ar*C*(5)H), **123.4** (Ar*C*(4)H), **124.3** (d, $^3J_{C\text{-}F}$ 7.0, Ar*C*(2```,6```)H), **127.1** (Ar*C*(4`)H), **127.8** (Ar*C*(3`,5`)H), **128.3** (Ar*C*(6)H), **128.7** (d, $^4J_{C\text{-}F}$ 2.1, Ar*C*(3```,5```)H), **129.2** (Ar*C*(4```)H), **129.3** (Ar*C*(2`,6`)H), **129.9** (Ar*C*(3a)), **132.0** (d, $^2J_{C\text{-}F}$ 28.8, Ar*C*(1```)), **141.8** (Ar*C*(1`)), **143.0** (Ar*C*(7a)), **159.1** (d, $^1J_{C\text{-}F}$ 251.0, *C*(3``)F), **179.9** (*C*=O); **$^{19}$F{$^1$H} NMR (377 MHz, $CDCl_3$)** δ **–116.25** (C(3``)*F*). **HRMS (ESI$^+$)** $C_{36}H_{28}FNO_2Na$ $[M+Na]^+$ found 548.1988, requires 548.1996 (–1.46 ppm).

(*S*,*Z*)-3-(3-Fluoro-3-(p-tolyl)allyl)-3-hydroxy-1-tritylindolin-2-one (47)

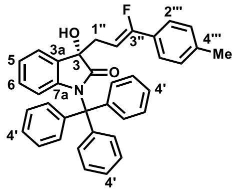

Following **General Procedure H**, allylic ether **S103** (53.9 mg, 0.1 mmol), *t*Bu-BIMP (7.4 mg, 0.01 mmol), in toluene (2 mL) for 15 h and then the reaction mixture was treated with DBU (0.10 mmol) at 90 °C for 15 h to give a crude formal [1,2]-rearrangement product which was purified by flash column chromatography (eluent: hexane/ EtOAc = 4:1 to 3:1) to afford the product (37.2 mg, 69%) as a pale yellow oil. **IR** ν_{max} (film) 3381 (OH), 1715 (C=O), 1610 (C=C), 1463, 1446, 1290, 1184 (C-F), 1165, 1083; $[\mathbf{a}]_{\mathbf{D}}^{\mathbf{20}}$ +11.2 (*c* 1.10, $CHCl_3$); **Chiral HPLC analysis**, Chiralpak IB (95:5 hexane:IPA, flow rate 1 ml·min^{-1}, 211 nm, 30 °C) t_R (*R*)-**47**: 9.8 min, t_R (*S*)-**47**: 14.9 min, 7:93 e.r.; **^{1}H NMR (400 MHz, CDCl$_3$)** δ **2.37** (1H, s, CH_3), **2.77** (1H, s, O*H*), **2.94** (1H, ddd, *J* 13.9, 7.1, 1.9, C(1``)$H^AH^B$), **3.06** (1H, ddd, *J* 13.9, 8.7, 1.1, C(1``)H^AH^B), **5.29** (1H, ddd, *J* 35.8, 8.7, 7.1, C(2``)*H*), **6.28** (1H, d, *J* 7.9, ArC(7)*H*), **6.91** (1H, app td, *J* 7.8, 1.6, ArC(6)*H*), **6.97** (1H, app td, *J* 7.5, 1.1, ArC(5)*H*), **7.13–7.24** (11H, m, ArC(3`,4`,5`,3```,5```)*H*), **7.33–7.37** (2H, m, ArC(2```,6```)*H*), **7.37–7.44** (7H, m, ArC(4,2`,6`)*H*); **^{13}C{^{1}H} NMR (101 MHz, CDCl$_3$)** δ **21.4** (*C*H$_3$), **34.9** (*C*(1``)H$_2$), **74.6** (N*C*Ph$_3$), **75.9** (d, $^4J_{C-F}$ 1.6, *C*(3)OH), **97.3** (d, $^2J_{C-F}$ 16.4, *C*(2``)H), **116.2** (Ar*C*(7)H), **122.8** (Ar*C*(5)H), **123.4** (Ar*C*(4)H), **124.3** (d, $^3J_{C-F}$ 7.0, Ar*C*(2```,6```)H), **127.1** (Ar*C*(4`)H), **127.8** (Ar*C*(3`,5`)H), **128.3** (Ar*C*(6)H), **129.2** (Ar*C*(1```)), **129.3** (Ar*C*(2`,6`,3```,5```)H), **130.0** (Ar*C*(3a)), **139.3** (Ar*C*(4```)Me), **141.9** (Ar*C*(1`)), **143.0** (Ar*C*(7a)), **159.3** (d, J_{C-F} 250.6, *C*(3``)F), **180.0** (*C*=O); **$^{19}$F{$^1$H} NMR (377 MHz, CDCl$_3$)** δ **–116.02** (C(3``)*F*). **HRMS (ESI$^+$)** $C_{37}H_{30}FNO_2Na$ [M+Na]$^+$ found 562.2143, requires 562.2153 (–1.74 ppm).

(*S*,*Z*)-3-[3-Fluoro-3-(4-methoxyphenyl)allyl]-3-hydroxy-1-tritylindolin-2-one (48)

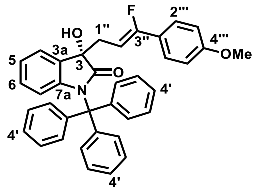

Following **General Procedure H**, allylic ether **S104** (55.5 mg, 0.1 mmol), *t*Bu-BIMP (7.4 mg, 0.01 mmol), in toluene (2 mL) for 15 h and then the reaction mixture was treated with DBU (0.10 mmol) at 90 °C for 15 h to give a crude formal [1,2]-rearrangement product which was purified by flash column chromatography (eluent: hexane/ EtOAc = 4:1 to 3:1) to afford the product (33.3 mg, 60%) as a pale yellow oil. **IR** ν_{max} (film) 3419 (OH), 1717 (C=O), 1606 (C=C), 1512, 1463, 1448, 1251, 1178 (C-F), 1114 (C-O), 1031; $[\mathbf{a}]_{\mathbf{D}}^{\mathbf{20}}$ +8.8 (*c* 1.10, $CHCl_3$); **Chiral HPLC analysis**, Chiralpak IB (90:10 hexane:IPA, flow rate 1 ml·min^{-1}, 211 nm, 30 °C) t_R (*R*)-**48**: 8.6 min, t_R (*S*)-**48**: 12.2 min, 7:93 e.r.; **^{1}H NMR (400 MHz, $CDCl_3$)** δ **2.77** (1H, s, O*H*), **2.92** (1H, ddd, *J* 13.9, 7.1, 1.9, C(1``)$H^AH^B$), **3.00–3.13** (1H, m, OC(1``)H^AH^B), **3.83** (3H, s, OCH_3), **5.19** (1H, ddd, *J* 36.0, 8.7, 7.1, C(2``)*H*), **6.28** (1H, d, *J* 7.9, ArC(7)*H*), **6.80–6.94** (3H, m, ArC(6,3```,5```)*H*), **6.97** (1H, app td, *J* 7.5, 1.1, ArC(5)*H*), **7.09–7.25** (9H, m, ArC(3`,4`,5`)*H*), **7.28–7.52** (2H, m, ArC(4,2`,6`,2```,6```)*H*); **^{13}C{^{1}H} NMR (101 MHz, $CDCl_3$)** δ **34.9** (*C*(1``)$H_2$), **55.5** (O$CH_3$), **74.6** (N*C*$Ph_3$), **75.9** (d, $^4J_{C\text{-}F}$ 1.5, *C*(3)OH), **96.3** (d, $^2J_{C\text{-}F}$ 16.5, *C*(2``)H), **114.1** (d, $^4J_{C\text{-}F}$ 1.8, Ar*C*(3```,5```)H), **116.2** (Ar*C*(7)H), **122.8** (Ar*C*(5)H), **123.4** (Ar*C*(4)H), **124.8** (d, $^2J_{C\text{-}F}$ 29.3, Ar*C*(1```)), **125.8** (d, $^3J_{C\text{-}F}$ 7.1, Ar*C*(2```,6```)H), **127.1** (Ar*C*(4`)H), **127.8** (Ar*C*(3`,5`)H), **128.3** (Ar*C*(6)H), **129.4** (Ar*C*(1```)), **130.0** (Ar*C*(3a)), **141.9** (Ar*C*(1`)), **143.0** (Ar*C*(7a)), **159.1** (d, $J_{C\text{-}F}$ 250.2, *C*(3``)F), **160.4** (Ar*C*(4```)OMe), **180.0** (*C*=O); **^{19}F{^{1}H} NMR (377 MHz, $CDCl_3$)** δ –**115.48** (C(3``)*F*). **HRMS (ESI$^+$)** $C_{37}H_{30}FNO_3Na$ $[M+Na]^+$ found 578.2095, requires 578.2102 (–1.20 ppm).

(*S*,*Z*)-3-{3-Fluoro-3-[4-(trifluoromethyl)phenyl]allyl}-3-hydroxy-1-tritylindolin-2-one (49)

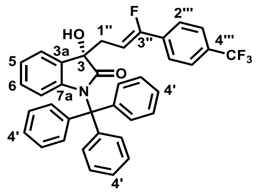

Following **General Procedure H**, allylic ether **S105** (59.3 mg, 0.1 mmol), *t*Bu-BIMP (7.4 mg, 0.01 mmol), in toluene (2 mL) for 15 h and then the reaction mixture was treated with DBU (0.10 mmol) at 90 °C for 15 h to give a crude formal [1,2]-rearrangement product which was purified by flash column chromatography (eluent: hexane/ EtOAc = 4:1 to 3:1) to afford the product (45.1 mg, 76%) as a pale yellow oil. **IR** ν_{max} (film) 3361 (OH), 1715 (C=O), 1612 (C=C), 1463, 1448, 1323 (CF_3), 1166 (C-F), 1112, 1068; $[\mathbf{a}]_{\mathbf{D}}^{\mathbf{20}}$ –48.0 (*c* 1.90, $CHCl_3$); **Chiral HPLC analysis**, Chiralpak IB (95:5 hexane:IPA, flow rate 1 ml·min^{-1}, 211 nm, 30 °C) t_R (*R*)-**49**: 10.6 min, t_R (*S*)-**49**: 18.5 min, 9:91 e.r.; **^{1}H NMR (400 MHz, $CDCl_3$)** δ **2.90** (1H, s, O*H*), **2.92–3.04** (1H, m, C(1``)$H^A$H$^B$), **3.07** (1H, dd, *J* 14.0, 8.5, OC(1``)H^AH^B), **5.49** (1H, app dt, *J* 35.4, 7.8, 7.1, C(2``)*H*), **6.30** (1H, d, *J* 8.0, ArC(7)*H*), **6.87–7.05** (2H, m, ArC(5,6)*H*), **7.10–7.24** (9H, m, ArC(3`,4`,5`)*H*), **7.33–7.47** (7H, m, ArC(4,2`,6`)*H*), **7.48–7.67** (4H, m, ArC(2```,3```,5```,6```)*H*); **^{13}C{^{1}H} NMR (101 MHz, $CDCl_3$)** δ **34.9** (d, $^3J_{C\text{-}F}$ 3.9, *C*(1``)$H_2$), **74.6** (N*C*$Ph_3$), **75.7** (*C*(3)OH), **101.2** (d, $^2J_{C\text{-}F}$ 16.0, *C*(2``)H), **116.3** (Ar*C*(7)H), **122.9** (Ar*C*(5)H), **123.4** (Ar*C*(4)H), **124.6** (d, $^3J_{C\text{-}F}$ 7.0, Ar*C*(2```,6```)), **124.0** (q, $^1J_{C\text{-}F}$ 272.7, *C*F_3), **125.7** (br, Ar*C*(3```,5```)H), **127.1** (Ar*C*(4`)H), **127.8** (Ar*C*(3`,5`)H), **128.5** (Ar*C*(6)H), **129.3** (Ar*C*(2`,6`)H), **129.9** (Ar*C*(3a)), **131.0** (q, $^2J_{C\text{-}F}$ 32.5, Ar*C*(4```)), **135.4** (d, $^2J_{C\text{-}F}$ 29.0, Ar*C*(1```)), **141.8** (Ar*C*(1`)), **142.9** (Ar*C*(7a)), **157.7** (d, $^1J_{C\text{-}F}$ 250.8, *C*(3``)F), **179.7** (*C*=O); **$^{19}$F{$^1$H} NMR (377 MHz, $CDCl_3$)** δ –**62.74** (3F, C*F*$_3$), **–116.84** (1F, C(3``)*F*). **HRMS (ESI$^+$)** $C_{37}H_{27}F_4NO_2Na$ [M+Na]$^+$ found 616.1860, requires 616.1870 (–1.64 ppm).

(*S*,*Z*)-4-Chloro-3-(3-fluoro-3-phenylallyl)-3-hydroxy-1-tritylindolin-2-one (50)

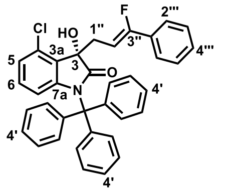

Following **General Procedure H**, allylic ether **S106** (55.9 mg, 0.1 mmol), *t*Bu-BIMP (7.4 mg, 0.01 mmol), in toluene (2 mL) for 15 h and then the reaction mixture was treated with DBU (0.10 mmol) at 70 °C for 15 h to give a crude formal [1,2]-rearrangement product which was purified by flash column chromatography (eluent: hexane/ EtOAc = 4:1 to 3:1) to afford the product (31.3 mg, 56%) as a pale yellow oil. **IR** ν_{max} (film) 3365 (OH), 1732 (C=O), 1600 (C=C), 1589 (C=C), 1490, 1444, 1282, 1143, 1085 (C-Cl); $[\mathbf{a}]_{\mathbf{D}}^{\mathbf{20}}$ +64.0 (*c* 0.25, $CHCl_3$); **Chiral HPLC analysis**, Chiralpak IB (95:5 hexane:IPA, flow rate 1 ml·min^{-1}, 211 nm, 30 °C) t_R (*R*)-**50**: 11.6 min, t_R (*S*)-**50**: 16.1 min, 4:96 e.r.; **^{1}H NMR (400 MHz, $CDCl_3$)** δ **2.85–2.98** (1H, m, O*H*), **3.21** (1H, ddd, *J* 13.6, 8.7, 1.2, C(1``)$H^AH^B$), **3.42** (1H, ddd, *J* 13.6, 7.5, 1.9, C(1``)H^AH^B), **5.25** (1H, ddd, *J* 35.6, 8.7, 7.5, C(2``)*H*), **6.20** (1H, d, *J* 8.1, ArC(7)*H*), **6.83** (1H, app t, *J* 8.2, ArC(6)*H*), **6.95** (1H, d, *J* 8.2, ArC(5)*H*), **7.08–7.21** (9H, m, ArC(3`,4`,5`)*H*), **7.29–7.40** (9H, m, ArC(2`,6`,3```,4```,5```)*H*), **7.42–7.47** (2H, m, ArC(2```,6```)*H*); **^{13}C{^{1}H} NMR (101 MHz, $CDCl_3$)** δ **32.4** (*C*(1``)$H_2$), **75.0** (N*C*$Ph_3$), **76.9** (d, $^4J_{C\text{-}F}$ 2.1, *C*(3)OH), **98.0** (d, $^2J_{C\text{-}F}$ 16.7, *C*(2``)H), **114.9** (Ar*C*(7)H), **123.9** (Ar*C*(5)H), **124.3** (d, $^3J_{C\text{-}F}$ 7.0, Ar*C*(2```,6```)H), **126.2** (Ar*C*(3a)), **127.2** (Ar*C*(4`)H), **127.8** (Ar*C*(3`,5`)H), **128.7** (d, $^4J_{C\text{-}F}$ 2.1, Ar*C*(3```,5```)H), **129.2** (Ar*C*(6)H), **129.3** (Ar*C*(2`,6`,4```)H), **131.0** (Ar*C*(4)Cl), **131.9** (d, $^2J_{C\text{-}F}$ 28.6, Ar*C*(1```)), **141.4** (Ar*C*(1`)), **145.0** (Ar*C*(7a)), **159.3** (d, $^1J_{C\text{-}F}$ 251.9, *C*(3``)F), **179.0** (*C*=O); **$^{19}$F{$^1$H} NMR (377 MHz, $CDCl_3$) δ –115.62** (C(3``)*F*). **HRMS (ESI$^+$)** $C_{36}H_{27}{}^{35}ClFNO_2Na$ $[M+Na]^+$ found 582.1596, requires 582.1607 (–1.81 ppm).

(*S*,*Z*)-5-Fluoro-3-(3-fluoro-3-phenylallyl)-3-hydroxy-1-tritylindolin-2-one (51)

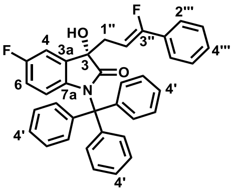

Following **General Procedure H**, allylic ether **S107** (54.3 mg, 0.1 mmol), *t*Bu-BIMP (7.4 mg, 0.01 mmol), in toluene (2 mL) for 15 h and then the reaction mixture was treated with DBU (0.10 mmol) at 100 °C for 15 h to give a crude formal [1,2]-rearrangement product which was purified by flash column chromatography (eluent: hexane/ EtOAc = 4:1 to 3:1) to afford the product (31.5 mg, 58%) as a pale yellow oil. **IR** ν_{max} (film) 3369 (OH), 1716 (C=O), 1606 (C=C), 1597 (C=C), 1477, 1448, 1263, 1182 (C-F), 1155 (C-F), 1122; $[\mathbf{a}]_{\mathbf{D}}^{\mathbf{20}}$ +10.4 (*c* 0.25, $CHCl_3$); **Chiral HPLC analysis**, Chiralpak IB (95:5 hexane:IPA, flow rate 1 ml·min^{-1}, 211 nm, 30 °C) t_R (*R*)-**51**: 10.4 min, t_R (*S*)-**51**: 15.0 min, 4:96 e.r.; **^{1}H NMR (400 MHz, $CDCl_3$)** δ **2.87–3.00** (m, 2H, O*H*, C(1``)*H*$^A$H$^B$), **2.99–3.11** (m, 1H, C(1``)H^A*H*^{B}), **5.21–5.50** (1H, m, C(2``)*H*), **6.21** (1H, dd, *J* 9.0, 4.2, ArC(7)*H*), **6.61** (1H, app td, *J* 9.0, 2.8, ArC(7)*H*), **7.13** (1H, dd, *J* 7.4, 2.8, ArC(4)*H*), **7.13–7.22** (9H, m, ArC(3`,4`,5`)*H*), **7.31–7.44** (9H, m, ArC(2`,6`,3```,4```,5```)*H*), **7.43–7.52** (2H, m, ArC(2```,6```)*H*); **^{13}C{^{1}H} NMR (101 MHz, $CDCl_3$)** δ **34.9** (*C*(1``)$H_2$), **74.8** (N*C*$Ph_3$), **76.0** (*C*(3)OH), **97.9** (d, $^2J_{C\text{-}F}$ 16.3, *C*(2``)H), **111.3** (d, $^2J_{C\text{-}F}$ 24.3, Ar*C*(4)H), **114.8** (d, $^2J_{C\text{-}F}$ 23.1, Ar*C*(6)H), **117.1** (d, $^3J_{C\text{-}F}$ 7.6, Ar*C*(7)H), **124.3** (d, $^3J_{C\text{-}F}$ 7.0, Ar*C*(2```,6```)H), **127.2** (Ar*C*(4`)H), **127.9** (Ar*C*(3`,5`)H), **128.7** (d, $^4J_{C\text{-}F}$ 2.0, Ar*C*(3```,5```)H), **129.3** (Ar*C*(2`,6`,4```)H), **131.7** (d, $^3J_{C\text{-}F}$ 4.4, Ar*C*(3a)), **131.8** (d, $^2J_{C\text{-}F}$ 41.0, Ar*C*(1```)), **138.8** (Ar*C*(7a)), **141.6** (Ar*C*(1`)), **159.0** (d, $J_{C\text{-}F}$ 243.2, Ar*C*(5)F), **159.4** (d, $J_{C\text{-}F}$ 251.5, *C*(3``)F), **179.7** (*C*=O); **$^{19}$F{$^1$H} NMR (377 MHz, $CDCl_3$)** δ **–115.77** (1F, C(3``)*F*), **–119.98** (1F, ArC(5)*F*). **HRMS (ESI$^+$)** $C_{36}H_{27}F_2NO_2Na$ [M+Na]$^+$ found 566.1890, requires 566.1902 (–2.13 ppm).

(*S*,*Z*)-3-(3-Fluoro-3-phenylallyl)-3-hydroxy-5-nitro-1-tritylindolin-2-one (52)

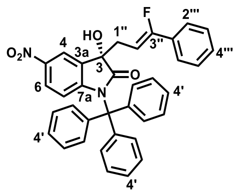

Following **General Procedure H**, allylic ether **S108** (57.0 mg, 0.1 mmol), *t*Bu-BIMP (7.4 mg, 0.01 mmol), in toluene (2 mL) for 15 h and then the reaction mixture was treated with DBU (0.10 mmol) at 60 °C for 5 h to give a crude formal [1,2]-rearrangement product which was purified by flash column chromatography (eluent: hexane/ EtOAc = 4:1 to 3:1) to afford the product (37.1 mg, 65%) as a pale yellow amorphous solid. **IR** ν_{max} (film) 3350 (OH), 1728 (C=O), 1610 (C=C), 1517 (NO_2), 1448, 1336 (NO_2), 1271, 1163 (C-F), 1074; $[a]_D^{20}$ +137.2 (*c* 0.25, $CHCl_3$); **Chiral HPLC analysis**, Chiralpak IB (90:10 hexane:IPA, flow rate 1 ml·min^{-1}, 211 nm, 30 °C) t_R (*R*)-**52**: 9.1 min, t_R (*S*)-**52**: 13.4 min, 3:97 e.r.; **^{1}H NMR (400 MHz, CDCl$_3$)** δ **2.93** (1H, s, O*H*), **3.01** (1H, ddd, *J* 13.9, 7.4, 1.8, C(1``)$H^AH^B$), **3.07–3.19** (1H, m, C(1``)H^AH^B), **5.32** (1H, ddd, $^3J_{C-F}$ 35.1, 8.6, 7.4, C(2``)*H*), **6.38** (1H, d, *J* 9.0, ArC(7)*H*), **7.15–7.23** (9H, m, ArC(3`,4`,5`)*H*), **7.30–7.40** (9H, m, ArC(2`,6`,3```,4```,5```)*H*), **7.42–7.50** (2H, m, ArC(2```,6```)*H*), **7.85** (1H, dd, *J* 9.0, 2.5, ArC(6)*H*), **8.27** (1H, d, *J* 2.5, ArC(4)*H*); **^{13}C{^{1}H} NMR (101 MHz, CDCl$_3$)** δ **34.8** (*C*(1``)$H_2$), **75.4** (N*C*Ph$_3$), **75.6** (d, $^4J_{C-F}$ 1.6, *C*(3)OH), **97.0** (d, $^2J_{C-F}$ 16.5, *C*(2``)H), **115.8** (Ar*C*(7)H), **119.3** (Ar*C*(4)H), **124.4** (d, $^3J_{C-F}$ 7.1, Ar*C*(2```,6```)H), **124.8** (Ar*C*(6)H), **127.6** (Ar*C*(4`)H), **128.1** (Ar*C*(3`,5`)H), **128.8** (d, $^4J_{C-F}$ 2.1, Ar*C*(3```,5```)H), **129.2** (Ar*C*(2`,6`)H), **129.6** (Ar*C*(4```)H), **130.9** (Ar*C*(3a)), **131.6** (d, $^2J_{C-F}$ 28.6, Ar*C*(1```)), **141.0** (Ar*C*(1`)), **143.3** (Ar*C*(5)NO_2), **148.9** (Ar*C*(7a)), **159.9** (d, J_{C-F} 252.6, *C*(3``)F), **179.9** (*C*=O); **$^{19}$F{$^1$H} NMR (377 MHz, CDCl$_3$)** δ **–114.6** (C(3``)*F*). **HRMS (ESI$^+$)** $C_{36}H_{27}FN_2O_4Na$ $[M+Na]^+$ found 593.1840, requires 593.1847 (–1.19 ppm).

(*S*,*Z*)-3-(3-Fluoro-3-phenylallyl)-3-hydroxy-5-methoxy-1-tritylindolin-2-one (53)

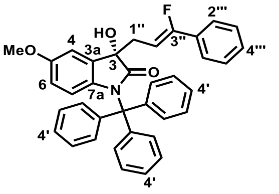

Following **General Procedure H**, allylic ether **S109** (55.5 mg, 0.1 mmol), *t*Bu-BIMP (7.4 mg, 0.01 mmol), in toluene (2 mL) for 15 h and then the reaction mixture was treated with DBU (0.10 mmol) at 90 °C for 15 h to give a crude formal [1,2]-rearrangement product which was purified by flash column chromatography (eluent: hexane/ EtOAc = 4:1 to 3:1) to afford the product (30.0 mg, 54%) as a pale yellow oil. **IR** ν_{max} (film) 3388 (OH), 1709 (C=O), 1597 (C=C), 1483, 1448, 1276, 1184 (C-F), 1161, 1128 (C-O), 1031; $[\mathbf{a}]_{\mathbf{D}}^{\mathbf{20}}$ +54.8 (*c* 0.25, $CHCl_3$); **Chiral HPLC analysis**, Chiralpak IB (90:10 hexane:IPA, flow rate 1 ml·min^{-1}, 211 nm, 30 °C) t_R (*R*)-**53**: 8.5 min, t_R (*S*)-**53**: 10.1 min, 5:95 e.r.; **^{1}H NMR (400 MHz, $CDCl_3$)** δ **2.80–2.99** (m, 2H, O*H*, OC(1``)$H^AH^B$), **3.08** (1H, ddd, *J* 13.9, 8.8, 1.1, OC(1``)H^AH^B), **3.70** (3H, s, OCH_3), **5.34** (1H, ddd, $^3J_{H\text{-}F}$ 35.7, 8.8, 7.1, C(2``)*H*), **6.17** (1H, d, *J* 8.9, ArC(7)*H*), **6.45** (1H, dd, *J* 8.9, 2.8, ArC(6)*H*), **7.01** (1H, d, *J* 2.7, ArC(4)*H*), **7.12–7.23** (9H, m, ArC(3`,4`,5`)*H*), **7.31–7.43** (9H, m, ArC(2`,6`,3```,4```,5```)*H*), **7.43–7.51** (2H, m, ArC(2```,6```)*H*); **^{13}C{^{1}H} NMR (101 MHz, $CDCl_3$)** δ **35.0** (*C*(1``)$H_2$), **55.7** (O*C*$H_3$), **74.6** (N*C*$Ph_3$), **76.1** (d, $^4J_{C\text{-}F}$ 1.6, *C*(3)OH), **98.4** (d, $^2J_{C\text{-}F}$ 16.3, *C*(2``)H), **109.5** (Ar*C*(4)H), **113.7** (Ar*C*(6)H), **117.0** (Ar*C*(7)H), **124.3** (d, $^3J_{C\text{-}F}$ 7.1, Ar*C*(2```,6```)H), **127.1** (Ar*C*(4`)H), **127.8** (Ar*C*(3`,5`)H), **128.7** (d, $^4J_{C\text{-}F}$ 2.1, Ar*C*(3```,5```)H), **129.2** (Ar*C*(4```)H), **129.3** (Ar*C*(2`,6`)H), **131.2** (Ar*C*(3a)), **132.1** (d, $^2J_{C\text{-}F}$ 28.8, Ar*C*(1```)), **136.1** (Ar*C*(7a)), **141.9** (Ar*C*(1`)), **155.8** (Ar*C*(5)OMe), **159.1** (d, $^1J_{C\text{-}F}$ 251.0, *C*(3``)F), **179.7** (*C*=O); **$^{19}$F NMR (377 MHz, $CDCl_3$)** δ **–116.1** (C(3``)*F*). **HRMS (ESI$^+$)** $C_{37}H_{30}FNO_3Na$ [M+H]$^+$ found 578.2079, requires 578.2102 (–3.96 ppm).

(*S*,*Z*)-6-Chloro-3-(3-fluoro-3-phenylallyl)-3-hydroxy-1-tritylindolin-2-one (54)

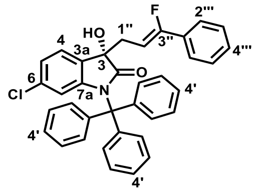

Following **General Procedure H**, allylic ether **S110** (55.9 mg, 0.1 mmol), *t*Bu-BIMP (7.4 mg, 0.01 mmol), in toluene (2 mL) for 15 h and then the reaction mixture was treated with DBU (0.10 mmol) at 70 °C for 20 h to give a crude formal [1,2]-rearrangement product which was purified by flash column chromatography (eluent: hexane/ EtOAc = 4:1 to 3:1) to afford the product (31.3 mg, 56%) as a pale yellow oil. **IR** ν_{max} (film) 3381 (OH), 1716 (C=O), 1608 (C=C), 1587 (C=C), 1490, 1475, 1448, 1284, 1182 (C-F), 1116, 1076 (C-Cl); $[\mathbf{a}]_{\mathbf{D}}^{\mathbf{20}}$ +36.0 (*c* 0.25, $CHCl_3$); **Chiral HPLC analysis**, Chiralpak IB (90:10 hexane:IPA, flow rate 1 ml·min^{-1}, 211 nm, 30 °C) t_R (*R*)-**54**: 7.2 min, t_R (*S*)-**54**: 9.4 min, 5:95 e.r.; **^{1}H NMR (400 MHz, $CDCl_3$)** δ **2.83** (s, 1H, O*H*), **2.92** (1H, ddd, *J* 13.9, 7.2, 2.0, C(1``)$H^A$H$^B$), **3.04** (1H, ddd, *J* 13.9, 8.7, 1.1, C(1``)H^AH^B), **5.31** (1H, ddd, *J* 35.5, 8.7, 7.2, C(2``)*H*), **6.23** (1H, d, *J* 1.8, ArC(7)*H*), **6.96** (1H, dd, *J* 8.0, 1.8, ArC(5)*H*), **7.14–7.24** (9H, m, ArC(3`,4`,5`)*H*), **7.30** (1H, d, *J* 8.0, ArC(4)*H*), **7.33–7.41** (9H, m, ArC(2`,6`,3```,4```,5```)*H*), **7.42–7.51** (2H, m, ArC(2```,6```)*H*); **^{13}C{^{1}H} NMR (101 MHz, $CDCl_3$)** δ **34.8** (d, $^3J_{C\text{-}F}$ 4.2, *C*(1``)$H_2$), **74.9** (N*C*$Ph_3$), **75.5** (d, $^4J_{C\text{-}F}$ 1.8, *C*(3)OH), **97.9** (d, $^2J_{C\text{-}F}$ 16.4, *C*(2``)H), **116.6** (Ar*C*(7)H), **122.9** (Ar*C*(5)H), **124.3** (Ar*C*(2```,6```)H), **124.4** (Ar*C*(4)H), **127.3** (Ar*C*(4`)H), **128.0** (Ar*C*(3`,5`)H), **128.3** (Ar*C*(3a)), **128.7** (d, $^4J_{C\text{-}F}$ 2.0, Ar*C*(3```,5```)H), **129.3** (Ar*C*(2`,6`)H), **129.4** (Ar*C*(4```)H), **131.9** (d, $^2J_{C\text{-}F}$ 28.6, Ar*C*(1```)), **134.0** (Ar*C*(6)Cl), **141.4** (Ar*C*(1`)), **144.2** (Ar*C*(7a)), **159.3** (d, $J_{C\text{-}F}$ 251.4, *C*(3``)F), **179.7** (*C*=O); **$^{19}$F{$^1$H} NMR (377 MHz, $CDCl_3$)** δ **–115.81** (C(3``)*F*). **HRMS (ESI$^+$)** $C_{36}H_{27}F^{35}ClNO_2Na$ [M+Na]$^+$ found 582.1605, requires 582.1607 (–0.27 ppm).

(*S*,*E*)-3-Cinnamyl-3-hydroxy-1-tritylindolin-2-one (45)

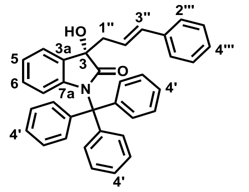

Following **General Procedure H**, allylic ether **43** (101.5 mg, 0.1999 mmol), *t*Bu-BIMP (14.8 mg, 0.0201 mmol) in toluene (4 mL) followed by DBU (28 μL, 0.19 mmol) gave a crude product which was purified by flash column chromatography (20:80 → 25:75 EtOAc/petrol) to afford the title compound (58 mg, 57%) as a yellow amorphous solid. **IR** ν_{max} (film) 3385 (O-H), 3086, 3057, 3028 (C-H), 1713 (C=O), 1611, 1599 (C=C), 1186 (C-O); $[\mathbf{a}]_{\mathbf{D}}^{\mathbf{20}}$ +13.6 (*c* 1.2, $CHCl_3$); **Chiral HPLC analysis,** Chiralpak AD-H (90:10 hexane:IPA, flow rate 1 mL·min^{-1}, 211 nm, 30 °C) t_R (*R*)-**45**: 12.5 min, t_R (*S*)-**45**: 14.6 min, 5:95 e.r.; **^{1}H NMR (500 MHz, $CDCl_3$)** δ **2.82** (1H, s, O*H*), **2.89** (1H, dd, *J* 13.0, 9.0, C(1``)*H*$^A$H$^B$), **2.99** (1H, ddd, *J* 13.0, 6.2, 1.6, C(1``)H^A*H*^{B}), **6.01** (1H, ddd, *J* 15.6, 9.0, 6.1, C(2``)*H*), **6.27** (1H, d, *J* 8.1, ArC(7)*H*), **6.52** (1H, d, *J* 15.8, C(3``)*H*), **6.91** (1H, app td, *J* 7.9, 1.5, ArC(6)*H*), **7.00** (1H, app td, *J* 7.5, 0.9, ArC(5)*H*), **7.09–7.18** (9H, m, ArC(3`,4`,5`)*H*), **7.21–7.26** (1H, m, ArC(4```)*H*), **7.29–7.33** (4H, m, ArC(2```,3```,5```,6```)*H*), **7.35** (6H, app dd, *J* 7.7, 2.1, ArC(2`,6`)*H*), **7.40** (1H, dd, *J* 7.4, 1.4, ArC(4)*H*). **^{13}C{^{1}H} NMR (126 MHz, $CDCl_3$)** δ 43.6 (*C*H_2), **74.7** (N*C*Ph_3), **76.3** (*C*(3)), **116.4** (Ar*C*(7)H), **122.1** (*C*(2``)H), **122.8** (Ar*C*(5)H), **123.4** (Ar*C*(4)H), **126.4** (Ar*C*(2```,6```)H), **127.0** (Ar*C*(4`)H), **127.7** (Ar*C*(3`,5`)H), **127.8** (Ar*C*(4```)H), **128.3** (Ar*C*(6)H), **128.8** (Ar*C*(3```,5```)H), **129.4** (Ar*C*(2`,6`)H), **129.8** (Ar*C*(3a)), **135.3** (*C*(3``)H), **136.9** (Ar*C*(1```)), **141.7** (Ar*C*(1`)), **143.2** (Ar*C*(7a)), **180.1** (*C*=O). **HRMS (ESI$^+$)** $C_{36}H_{29}NO_2Na$ [M+Na]$^+$ found 530.2090, requires 530.2091 (–0.2 ppm).

(*S*,*E*)-3-Hydroxy-3-[3-(4-methoxyphenyl)allyl]-1-tritylindolin-2-one (55)

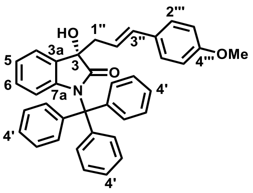

Following **General Procedure H,** allylic ether **S111** (107.6 mg, 0.2001 mmol), *t*Bu-BIMP (14.8 mg, 0.0201 mmol) in toluene (4 mL) followed by DBU (30 µL, 0.20 mmol) gave a crude product which was purified by flash column chromatography (30:70 EtOAc/hexane) to afford the title compound (58.9 mg, 55%) as a yellow amorphous solid. **IR** ν_{max} (film) 3401 (O-H), 3088, 3057, 3034, 2934, 2907 (C-H), 1715 (C=O), 1607, 1578, 1510 (C=C), 1175, 1111 (C-O); $[\mathbf{a}]_{\mathbf{D}}^{\mathbf{20}}$ +13.6 (*c* 1.2, $CHCl_3$); **Chiral HPLC analysis,** Chiralpak IB (90:10 hexane:IPA, flow rate 1 mL·min^{-1}, 211 nm, 30 °C) t_R (*R*)-**55**: 9.0 min, t_R (*S*)-**55**: 12.5 min, 11:89 e.r.; **^{1}H NMR (400 MHz, $CDCl_3$)** δ **2.69–2.77** (1H, m, O*H*), **2.86** (1H, ddd, *J* 13.1, 9.1, 0.9, C(1``)*H*$^A$H$^B$), **2.96** (1H, ddd, *J* 13.0, 6.1, 1.5, C(1``)H^A*H*^{B}), **3.80** (3H, s, OC*H*$_3$), **5.87** (1H, ddd, *J* 15.5, 9.0, 6.1, C(2``)*H*), **6.27** (1H, d, *J* 8.0, ArC(7)*H*), **6.47** (1H, d, *J* 15.8, C(3``)*H*), **6.81–6.87** (2H, m, ArC(3```,5```)*H*), **6.91** (1H, ddd, *J* 8.0, 7.7, 1.5, ArC(6)*H*), **6.99** (1H, app td, *J* 7.7, 1.0, ArC(5)*H*), **7.10–7.20** (9H, m, ArC(3`,4`,5`)*H*), **7.21–7.29** (2H, m, ArC(2```,6```)*H*), **7.32–7.41** (7H, m, ArC(4,2`,6`)*H*). **^{13}C{^{1}H} NMR (126 MHz, $CDCl_3$)** δ **43.7** (*C*H$_2$), **55.4** (O*C*H$_3$), **74.6** (N*C*Ph$_3$), **76.3** (*C*(3)), **114.2** (Ar*C*(3```,5```)H), **116.3** (Ar*C*(7)), **119.7** (*C*(2``)H), **122.8** (Ar*C*(5)H), **123.4** (Ar*C*(4)H), **127.0** (Ar*C*(4`)H), **127.6** (Ar*C*(2```,6```)H), **127.7** (Ar*C*(3`,5`)H), **128.2** (Ar*C*(6)H), **129.4** (Ar*C*(2`,6`)H), **129.7** (Ar*C*(1```)), **129.9** (Ar*C*(3a)), **134.7** (*C*(3``)H), **141.8** (Ar*C*(1`)), **143.2** (Ar*C*(7a)), **159.4** (Ar*C*(4```)), **180.1** (*C*=O). **HRMS (ESI$^+$)** $C_{37}H_{31}NO_3Na$ $[M+Na]^+$ found 560.2185, requires 560.2196 (–2.0 ppm).

(*S*,*E*)-3-Hydroxy-3-{3-[4-(trifluoromethyl)phenyl]allyl}-1-tritylindolin-2-one (56)

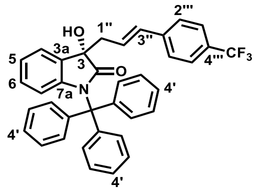

Following **General Procedure H**, allylic ether **S112** (115.1 mg, 0.2000 mmol), *t*Bu-BIMP (14.8 mg, 0.0201 mmol) in toluene (4 mL) followed by DBU (30 µL, 0.20 mmol) gave a crude product which was purified by flash column chromatography (30:70 EtOAc/hexane) to afford the title compound (71 mg, 62%) as a yellow amorphous solid. **IR** ν_{max} (film) 3389 (O-H), 3088, 3057, 3034, 2926 (C-H), 1713 (C=O), 1614 (C=C), 1325 (C-F), 1165 (C-O); $[\mathbf{a}]_{\mathbf{D}}^{\mathbf{20}}$ +7.5 (*c* 0.98, $CHCl_3$); **Chiral HPLC analysis,** Chiralcel OD-H (90:10 hexane:, flow rate 1 mLmin^{-1}, 211 nm, 30 °C) t_R (*R*)-**56**: 9.4 min, t_R (*S*)-**56**: 18.0 min, 8:92 e.r.; **^{1}H NMR (500 MHz, $CDCl_3$)** δ **2.84** (1H, s, O*H*), **2.91** (1H, dd, *J* 13.1, 9.0, C(1``)*H*$^A$H$^B$), **2.99** (1H, ddd, *J* 13.1, 6.2, 1.6, C(1``)H^A*H*^{B}), **6.12** (1H, ddd, *J* 15.6, 8.9, 6.2, C(2``)*H*), **6.29** (1H, d, *J* 8.0, ArC(7)*H*), **6.54** (1H, d, *J* 15.8, C(3``)*H*), **6.93** (1H, ddd, *J* 8.0, 7.8, 1.5, ArC(6)*H*), **7.00** (1H, dd, *J* 7.8, 7.6, ArC(5)*H*), **7.09–7.20** (9H, m, ArC(3`,4`,5`)*H*), **7.30–7.37** (6H, m, ArC(2`,6`)*H*), **7.37–7.42** (3H, m, ArC(4,2```,6```)*H*), **7.56** (2H, d, *J* 8.1, ArC(3```,5```)*H*). **^{13}C{^{1}H} NMR (126 MHz, $CDCl_3$)** δ **43.4** (*C*H_2), **74.7** (N*C*Ph_3), **76.1** (*C*(3)), **116.5** (Ar*C*(7)H), **122.9** (Ar*C*(5)H), **123.4** (Ar*C*(4)H), **124.3** (q, $^1J_{CF}$ 271.8, *C*F_3), **125.4** (*C*(2``)H), **125.7** (q, $^3J_{CF}$ 3.8, Ar*C*(3```,5```)H), **126.5** (Ar*C*(2```,6```)H), **127.1** (Ar*C*(4`)H), **127.7** (Ar*C*(3`,5`)H), **128.4** (Ar*C*(6)H), **129.4** (Ar*C*(2`,6`)H), **129.6** (Ar*C*(3a)), **129.6** (q, $^2J_{CF}$ 32.8, Ar*C*(4```)CF_3), **133.9** (*C*(3``)H), **140.4** (Ar*C*(1```)), **141.7** (Ar*C*(1`)), **143.2** (Ar*C*(7a)), **179.8** (*C*=O). **^{19}F{^{1}H} NMR (470 MHz, $CDCl_3$)** δ **–62.5** (C*F*$_3$); **HRMS (ESI$^+$)** $C_{37}H_{28}F_3NO_2Na$ [M+Na]$^+$ found 598.1957, requires 598.1964 (–1.2 ppm).

(*S*,*E*)-3-(3-(4-fluorophenyl)allyl)-3-hydroxy-1-tritylindolin-2-one (57)

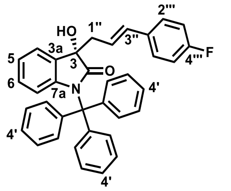

Following **General Procedure H**, allylic ether **S113** (105.1 mg, 0.2000 mmol), *t*Bu-BIMP (14.9 mg, 0.0202 mmol) in toluene (4 mL) followed by DBU (30 μL, 0.20 mmol) gave, after purification by flash column chromatography (20:80 EtOAc/hexane), the title compound as a yellow amorphous solid (60 mg, 57%). **IR** ν_{max} (film) 3377 (O-H), 3088, 3055, 3034 (C-H), 1713 (C=O), 1611, 1601 (C=C), 1157 (C-O); $[\mathbf{a}]_{\mathbf{D}}^{\mathbf{20}}$ +15.6 (*c* 0.63, $CHCl_3$); **Chiral HPLC analysis,** Chiralcel IB (90:10 hexane:IPA, flow rate 1 $mLmin^{-1}$, 211 nm, 30 °C) t_R (*R*)-**57**: 7.9 min, t_R (*S*)-**57**: 11.0 min, 9:91 e.r.; ^{1}**H NMR (500 MHz, $CDCl_3$)** δ **2.79** (1H, s, O*H*), **2.87** (1H, ddd, *J* 13.0, 9.1, 1.0, C(1``)$H^AH^B$), **2.96** (1H, ddd, *J* 13.1, 6.1, 1.6, C(1``)H^AH^B), **5.92** (1H, ddd, *J* 15.5, 9.0, 6.1, C(2``)*H*), **6.28** (1H, d, *J* 8.1, ArC(7)*H*), **6.48** (1H, d, *J* 15.8, C(3``)*H*), **6.92** (1H, ddd, *J* 7.9, 1.5, ArC(6)*H*), **6.96–7.04** (3H, m, ArC(5,3```)*H*), **7.10–7.19** (9H, m, ArC(3,4)*H*), **7.23–7.30** (2H, m, ArC(2```)*H*), **7.32–7.38** (6H, m, ArC(2`,6`)*H*), **7.39** (1H, dd, *J* 7.4, 1.4, ArC(4)*H*); ^{13}**C{^{1}H} NMR (126 MHz, $CDCl_3$)** δ **43.5** (*C*(1``)H), **74.6** (N*C*Ph_3), **76.2** (*C*(3)), **115.7** (d, *J* 21.4, Ar*C*(3```)H), **116.4** (Ar*C*(7)H), **121.9** (d, *J* 2.3, *C*(2``)H), **122.8** (Ar*C*(5)H), **123.4** (Ar*C*(4)H), **127.1** (Ar*C*(4`)H), **127.7** (Ar*C*(3`,5`)H), **127.9** (d, *J* 8.1, Ar*C*(2```)H), **128.3** (Ar*C*(6)H), **129.4** (Ar*C*(2`,6`)H), **129.8** (Ar*C*(3a)), **133.08** (d, *J* 3.3, Ar*C*(1```)), **134.1** (*C*(3``)H), **141.7** (Ar*C*(1```)), **143.2** (Ar*C*(7a)), **162.5** (d, *J* 247.0, Ar*C*(4```)F), 179.9 (*C*(2)); ^{19}**F{^{1}H} NMR (377 MHz, $CDCl_3$)** δ –114.4 (ArC(4```)*F*); **HRMS (ESI$^+$)** $C_{36}H_{28}FNO_2Na$ $[M+Na]^+$ found 548.1983, requires 548.1996 (–2.4 ppm);

(*S*,*E*)-3-Hydroxy-3-[3-(2-methoxyphenyl)allyl]-1-tritylindolin-2-one (58)

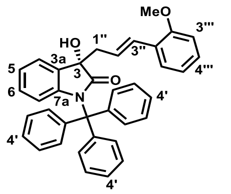

Following **General Procedure H**, allylic ether **S114** (108.0 mg, 0.2009 mmol), *t*Bu-BIMP (14.8 mg, 0.0201 mmol) in toluene (4 mL) followed by DBU (30 μL, 0.20 mmol) gave a crude product which was purified by flash column chromatography (20:80 EtOAc/hexane) to afford the title compound (65 mg, 60%) as a yellow amorphous solid. **IR** ν_{max} (film) 3399 (O-H), 3086, 3055, 3034, 2957, 2930 (C-H), 1713 (C=O), 1611, 1597 (C=C), 1244, 1182 (C-O); $\mathbf{[a]_D^{20}}$ +55.6 (*c* 0.40, $CHCl_3$); **Chiral HPLC analysis,** Chiralpak IB (95:5 hexane:IPA, flow rate 1 mL·min^{-1}, 211 nm, 30 °C) t_R (*R*)-**58**: 15.1 min, t_R (*S*)-**58**: 24.0 min, 9:91 e.r.; **^{1}H NMR (500 MHz, $CDCl_3$)** δ **2.75** (1H, s, O*H*), **2.92** (1H, dd, *J* 13.0, 9.0, C(1``)$H^AH^B$), **2.99** (1H, dd, *J* 12.5, 6.0, C(1``)H^AH^B), **3.74** (3H, s, OCH_3), **6.02** (1H, ddd, *J* 15.6, 8.9, 6.2, C(2``)*H*), **6.26** (1H, d, *J* 8.1, ArC(7)*H*), **6.83** (1H, d, *J* 8.2, ArC(3```)*H*), **6.86** (1H, d, *J* 16.1, C(3``)*H*), **6.89–6.94** (2H, m, ArC(6,5```)*H*), **6.99** (1H, app t, *J* 7.5, ArC(5)*H*), **7.07** – **7.18** (9H, m, ArC(3`, 4`,5`)*H*), **7.19–7.24** (1H, m, ArC(6```)*H*), **7.33** (1H, dd, *J* 7.7, 1.7, ArC(4```)*H*), **7.35–7.38** (6H, m, ArC(2`,6`)*H*), **7.38–7.45** (1H, m, ArC(4)*H*). **^{13}C{^{1}H} NMR (126 MHz, $CDCl_3$)** δ **44.1** (CH_2), **55.5** (OCH_3), **74.7** ($NCPh_3$), **76.4** (*C*(3)), **111.0** (Ar*C*(3```)H), **116.3** (Ar*C*(7)H), **120.7** (Ar*C*(5```)H), **122.7** (*C*(2``)H), **122.7** (Ar*C*(5)H), **123.3** (Ar*C*(4)H), **125.9** (Ar*C*(1```)), **126.9** (Ar*C*(4```)H), **127.0** (Ar*C*(4`)H), **127.7** (Ar*C*(3`,5`)H), **128.1** (Ar*C*(6)H), **128.8** (Ar*C*(6```)H), **129.5** (Ar*C*(2`,6`)H), **130.0** (Ar*C*(3a)), **130.0** (*C*(3``)H) **141.8** (Ar*C*(1`)), **143.2** (Ar*C*(7a)), **156.7** (Ar*C*(2```)), **180.3** (*C*=O). **HRMS (ESI$^+$)** $C_{37}H_{31}NO_3Na$ $[M+Na]^+$ found 560.2178, requires 560.2196 (–3.2 ppm).

(*S*,*E*)-3-Hydroxy-3-[3-(1-naphthyl)allyl]-1-tritylindolin-2-one (59)

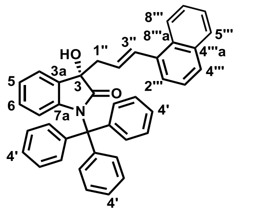

Following **General Procedure H**, allylic ether **S115** (111.7 mg, 0.2003 mmol), *t*Bu-BIMP (14.8 mg, 0.0201 mmol) in toluene (4 mL) followed by DBU (30 μL, 0.20 mmol) gave a crude product which was purified by flash column chromatography (30:70 EtOAc/hexane) to afford the title compound (71 mg, 64%) as a yellow amorphous solid. **IR** ν_{max} (film) 3393 (O-H), 3086, 3055, 3034, 2922, 2853 (C-H), 1709 (C=O), 1611, 1599 (C=C), 1263 (C-O); $\mathbf{[a]^{20}_{D}}$ –12.4 (*c* 0.86, $CHCl_3$); **Chiral HPLC analysis,** Chiralcel OD-H (90:10 hexane:IPA, flow rate 1 mL·min^{-1}, 211 nm, 30 °C) t_R (*R*)-**59**: 10.4 min, t_R (*S*)-**59**: 14.9 min, 6:94 e.r.; **^{1}H NMR (500 MHz, $CDCl_3$)** δ **2.83** (1H, s, O*H*), **3.01** (1H, dd, *J* 12.9, 8.0, C(1``)*H*$^A$H$^B$), **3.08** (1H, ddd, *J* 12.9, 7.2, 1.4, C(1``)H^A*H*^{B}), **6.05** (1H, app dt, *J* 15.4, 7.6, C(2``)*H*), **6.24** (1H, d, *J* 8.2, ArC(7)*H*), **6.95** (1H, app td, *J* 7.9, 1.4, ArC(6)*H*), **7.01** (6H, app t, *J* 7.7, ArC(3`,5`)*H*), **7.05–7.10** (4H, m, ArC(5, 4`)*H*), **7.13** (1H, d, *J* 15.5, C(3``)*H*), **7.28–7.32** (6H, m, ArC(2`,6`)*H*), **7.37–7.51** (m, 5H, ArC(4,3```,4```,6```, 7```)*H*), **7.66** (1H, d, *J* 8.4, ArC(2```)*H*), **7.79** (1H, d, *J* 8.0, ArC(8```)*H*), **7.85** (1H, d, *J* 8.1, ArC(5```)*H*). **^{13}C{^{1}H} NMR (126 MHz, $CDCl_3$)** δ **43.8** (*C*H_2), **74.7** (N*C*Ph_3), **76.5** (*C*(3)), **116.6** (Ar*C*(7)H), **122.8** (Ar*C*(5)H), **123.5** (Ar*C*(4)H), **123.8** (Ar*C*(2```)H), **124.0** (Ar*C*(7```)H), **125.1** (*C*(2``)H), **125.8** (Ar*C*(3```)H), **125.9** (Ar*C*(4```)H), **126.1** (Ar*C*(6```)H), **127.0** (Ar*C*(4`)H), **127.7** (Ar*C*(3`,5`)H), **128.1** (Ar*C*(8```)H), **128.3** (Ar*C*(6)H), **128.5** (Ar*C*(5```)H), **129.5** (Ar*C*(2`,6`)H), **129.9** (Ar*C*(3a)), **131.1** (Ar*C*(8```a)), **133.0** (*C*(3``)H), **133.7** (Ar*C*(4```a)), **134.7** (Ar*C*(1```)), **141.7** (Ar*C*(1`)), **143.5** (Ar*C*(7a)), **179.9** (*C*=O). **HRMS (ESI$^+$)** $C_{40}H_{31}NO_2Na$ [M+Na]$^+$ found 580.2234, requires 580.2247 (–2.2 ppm).

(*S*,*E*)-3-Hydroxy-3-[3-(2-naphthyl)allyl]-1-tritylindolin-2-one (60)

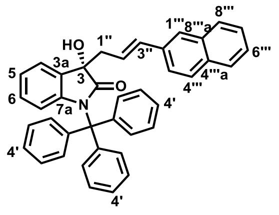

Following **General Procedure H**, allylic ether **S116** (110.8 mg, 0.1987 mmol), *t*Bu-BIMP (14.8 mg, 0.0201 mmol) in toluene (4 mL) followed by DBU (30 µL, 0.20 mmol) gave a crude product which was purified by flash column chromatography (30:70 EtOAc/hexane) to afford the title compound (66 mg, 60%) as a yellow amorphous solid. **IR** ν_{max} (film) 3397 (O-H), 3086, 3055, 3034, 2955, 2924 (C-H), 1715 (C=O), 1611, 1599 (C=C), 1113 (C-O); **$[a]_D^{20}$** –5.6 (*c* 1.1, $CHCl_3$); **Chiral HPLC analysis,** Chiralcel OD-H (90:10 hexane:IPA, flow rate 1 mL·min^{-1}, 211 nm, 30 °C) t_R (*R*)-**60**: 15.4 min, t_R (*S*)-**60**: 31.9 min, 9:91 e.r.; **^{1}H NMR (500 MHz, $CDCl_3$)** δ **2.83** (1H, s, O*H*), **2.95** (1H, dd, *J* 13.1, 9.0, C(1``)$H^A$H$^B$), **3.05** (1H, ddd, *J* 13.1, 6.1, 1.5, C(1``)H^AH^B), **6.16** (1H, ddd, *J* 15.4, 9.0, 6.1, C(2``)*H*), **6.28** (1H, d, *J* 8.1, ArC(7)*H*), **6.69** (1H, d, *J* 15.8, C(3``)*H*), **6.93** (1H, app td, *J* 7.9, 1.5, ArC(6)*H*), **7.01** (1H, app t, *J* 7.5, ArC(5)*H*), **7.07–7.17** (9H, m, ArC(3`,4`,5`)*H*), **7.33–7.40** (6H, m, ArC(2`,6`)*H*), **7.41–7.48** (3H, m, ArC(4,6```, 7```)*H*), **7.53** (1H, dd, *J* 8.6, 1.7, ArC(3```)*H*), **7.67** (1H, s, ArC(1```)*H*), **7.75–7.83** (3H, m, ArC(4```,5```,8```)*H*). **^{13}C{^{1}H} NMR (126 MHz, $CDCl_3$)** δ **43.8** (*C*H_2), **74.7** (N*C*Ph_3), **76.3** (*C*(3)), **116.4** (Ar*C*(7)H), **122.5** (*C*(2``)H), **122.8** (Ar*C*(5)H), **123.4** (Ar*C*(4)H), **123.5** (Ar*C*(3```)H), **126.0** (Ar*C*(6```)H), **126.4** (Ar*C*(1```)H), **126.4** (Ar*C*(7```)H), **127.0** (Ar*C*(4`)H), **127.7** (Ar*C*(3`,5`)H), **127.8** (Ar*C*(8```)H), **128.1** (Ar*C*(5```)H), **128.3** (Ar*C*(6)H), **128.4** (Ar*C*(4```)H), **129.4** (Ar*C*(2`,6`)H), **129.9** (Ar*C*(3a)), **133.1** (Ar*C*(4```a)), **133.7** (Ar*C*(8```a)), **134.3** (Ar*C*(2```)), **135.5** (*C*(3``)H), **141.7** (Ar*C*(1`)), **143.2** (Ar*C*(7a)), **180.0** (*C*=O). **HRMS (ESI$^+$)** $C_{40}H_{31}NO_2Na$ $[M+Na]^+$ found 580.2232, requires 580.2247 (–2.6 ppm).

(*S*,*E*)-3-Hydroxy-3-[3-(thiophen-3-yl)allyl]-1-tritylindolin-2-one (61)

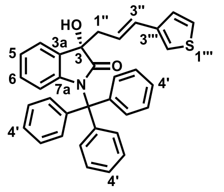

Following **General Procedure H**, allylic ether **S117** (82.8 mg, 0.161 mmol), *t*Bu-BIMP (11.9 mg, 0.0161 mmol) in toluene (3.2 mL) followed by DBU (24 µL, 0.16 mmol) gave a crude product which was purified by flash column chromatography (25:75 EtOAc/hexane) to afford the title compound (35 mg, 42%) as a yellow amorphous solid. **IR** ν_{max} (film) 3385 (O-H), 3055, 3032, 3022, (C-H), 1713 (C=O), 1611, 1601 (C=C), 1186 (C-O); $\mathbf{[a]_D^{20}}$ +23.4 (*c* 0.54, $CHCl_3$); **Chiral HPLC analysis,** Chiralpak AD-H (95:5 hexane:IPA, flow rate 1 mL·min^{-1}, 211 nm, 30 °C) t_R (*R*)-**61**: 15.2 min, t_R (*S*)-**61**: 17.3 min, 13:87: e.r.; **^{1}H NMR (500 MHz, $CDCl_3$)** δ **2.70–2.77** (1H, m, O*H*), 2.83 (1H, dd, *J* 13.0, 9.1, C(1``)*H*$^A$H$^B$), **2.95** (1H, ddd, *J* 12.9, 6.2, 1.6, C(1``)H^A*H*^{B}), **5.85** (1H, ddd, *J* 15.5, 9.0, 6.2, C(2``)*H*), **6.27** (1H, d, *J* 8.1, ArC(7)*H*), **6.52** (1H, d, *J* 15.8, C(3``)*H*), **6.91** (1H, app td, *J* 7.9, 1.5, ArC(6)*H*), **6.99** (1H, app td, *J* 7.5, 0.9, ArC(5)*H*), **7.09** (1H, dd, *J* 3.0, 1.2, ArC(2```)*H*), **7.11–7.19** (10H, m, ArC(3`,4`,5`, 4```)*H*), **7.23–7.30** (1H, m, ArC(5```)*H*), **7.31–7.37** (6H, m, ArC(2`,6`)*H*), **7.38** (1H, dd, *J* 7.3, 1.4, ArC(4)*H*). **^{13}C{^{1}H} NMR (126 MHz, $CDCl_3$)** δ **43.5** (*C*H_2), **74.7** (N*C*Ph_3), **76.3** (*C*(3)), **116.4** (Ar*C*(7)H), **122.0** (*C*(2``)H), **122.1** (Ar*C*(2```)H), **122.8** (Ar*C*(5)H), **123.3** (Ar*C*(4)H), **125.0** (Ar*C*(4```)H), **126.3** (Ar*C*(5''')H), **127.0** (Ar*C*(4`)H), **127.7** (Ar*C*(3`,5`)H), **128.3** (Ar*C*(6)H), **129.5** (Ar*C*(2`,6`)H), **129.5** (*C*(3``)H), **129.8** (Ar*C*(3a)), **139.6** (Ar*C*(3```)), **141.7** (Ar*C*(1`)), **143.2** (Ar*C*(7a)), **180.0** (*C*=O). **HRMS (ESI$^+$)** $C_{34}H_{27}NO_2SNa$ [M+Na]$^+$ found 536.1651, requires 536.1655 (–0.7 ppm).

(*S*,*E*)-3-Hydroxy-3-(2-methyl-3-phenylallyl)-1-tritylindolin-2-one (62)

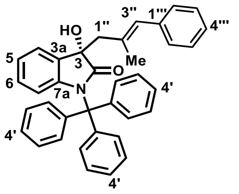

Following **General Procedure H**, allylic ether **S118** (104.9 mg, 0.2011 mmol), *t*Bu-BIMP (29.6 mg, 0.0401 mmol) in toluene (4 mL) followed by DBU (30 µL, 0.20 mmol) gave a crude product which was purified by flash column chromatography (25:75 EtOAc/hexane) to afford the title compound (43 mg, 41%) as a yellow amorphous solid. **IR** ν_{max} (film) 3399 (O-H), 3055, 3022, 2953, 2922, 2855 (C-H), 1717 (C=O), 1611, 1599 (C=C), 1184 (C-O); **[a]$^{20}_{D}$** –13.0 (*c* 0.60, $CHCl_3$); **Chiral HPLC analysis,** Chiralpak AD-H (95:5 hexane:IPA, flow rate 1 mL·min^{-1}, 211 nm, 30 °C) t_R (*R*)-**62**: 17.4 min, t_R (*S*)-**62**: 21.8 min, 11:89 e.r.; **^{1}H NMR (500 MHz, $CDCl_3$)** δ **1.81** (3H, d, *J* 1.4, C*H*$_3$), **2.76–2.84** (1H, m, O*H*), **2.89–2.92** (2H, m, C(1``)*H*$_2$), **6.24** (1H, s, C(3``)*H*), **6.27** (1H, d, *J* 8.2, ArC(7)*H*), **6.92** (1H, app td, *J* 7.9, 1.5, ArC(6)*H*), **6.99** (1H, app t, *J* 7.4, ArC(5)*H*), **7.10–7.19** (11H, m, ArC(3`,4`,5`, 3```,5```)*H*), **7.19–7.24** (1H, m, ArC(4```)*H*), **7.31** (2H, app d, *J* 7.6, ArC(2```,6```)*H*), **7.35** (6H, app dd, *J* 8.1, 1.8, ArC(2`,6`)*H*), **7.41** (1H, dd, *J* 7.3, 1.5, ArC(4)*H*). **^{13}C{^{1}H} NMR (126 MHz, $CDCl_3$)** δ **20.4** (*C*H$_3$), **50.5** (*C*H$_2$), **74.9** (N*C*Ph$_3$), **76.6** (*C*(3)), **116.6** (Ar*C*(7)H), **122.6** (Ar*C*(5)H), **123.9** (Ar*C*(4)H), **126.6** (Ar*C*(4```)H), **127.1** (Ar*C*(4`)H), **127.7** (Ar*C*(3`,5`)H), **128.3** (Ar*C*(6, 2```,6```)H), **129.1** (Ar*C*(3```,5```)H), **129.6** (Ar*C*(2`,6`)H), **130.1** (Ar*C*(3a)), **130.9** (*C*(3``)H), **132.0** (*C*(2``)H), **137.6** (Ar*C*(1```)), **141.7** (Ar*C*(1`)), **143.5** (Ar*C*(7a)), **180.2** (*C*=O). **HRMS (ESI$^+$)** $C_{37}H_{31}NO_2Na$ $[M+Na]^+$ found 544.2232, requires 544.2247 (–2.8 ppm).

(*S,E*)-4-Chloro-3-cinnamyl-3-hydroxy-1-tritylindolin-2-one (63)

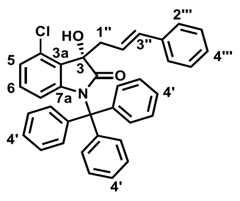

Following **General Procedure H**, allylic ether **S119** (107.9 mg, 0.1990 mmol), *t*Bu-BIMP (14.8 mg, 0.0201 mmol) in toluene (4 mL) followed by DBU (30 µL, 0.20 mmol) gave, after purification by flash column chromatography (20:80 EtOAc/hexane), the title compound as a yellow amorphous solid (65 mg, 60%); **IR** ν_{max} (film) 3404 (O-H), 3057, 3026 (C-H), 1724 (C=O), 1601, 1587 (C=C), 1144 (C-O); $\mathbf{[a]_D^{20}}$ +23.9 (*c* 0.82, $CHCl_3$); **Chiral HPLC analysis,** Chiralcel IB (90:10 hexane:IPA, flow rate 1 mLmin^{-1}, 211 nm, 30 °C) t_R (*R*)-**63**: 7.0 min, t_R (*S*)-**63**: 8.5 min, 13:87 e.r.; **^{1}H NMR (500 MHz, $CDCl_3$)** δ **2.86** (1H, s, O*H*), **2.95** (1H, ddd, *J* 12.8, 9.0, 1.0, C(1``)$H^AH^B$), **3.49** (1H, ddd, J 12.8, 6.5, 1.5, C(1``)H^AH^B), **5.94** (1H, ddd, *J* 15.6, 8.9, 6.5, C(2)*H*), **6.17** (1H, dd, *J* 8.2, 0.7, ArC(7)*H*), **6.55** (1H, d, *J* 15.8, C(3)*H*), **6.82** (1H, dd, *J* 8.2, ArC(6)*H*), **6.95** (1H, dd, *J* 8.2, 0.8, ArC(5)*H*), **7.11** (6H, dd, *J* 8.3, 6.3, ArC(3`,5`)*H*), **7.13–7.18** (3H, m, ArC(4`)*H*), **7.21–7.34** (11H, m, ArC(2`,6`,2```,3```,4```,5```,6```)*H*); **^{13}C{^{1}H} NMR (126 MHz, $CDCl_3$)** δ **40.6** (*C*(1``)H), **75.1** (N*C*Ph$_3$), **77.3**(*C*(3)), **115.0** (Ar*C*(7)H), **121.7** (*C*(2``)H), **123.9** (Ar*C*(5)H), **126.0** (Ar*C*(3a)), **126.4** (Ar*C*(2```)H), **127.2** (Ar*C*(4`)H), **127.8** (Ar*C*(3`,5`)H), **127.9** (Ar*C*(4```)H), **128.8** (Ar*C*(3```)H), **129.2** (Ar*C*(6)H), **129.5** (Ar*C*(2`,6`)H), **130.8** (Ar*C*(4)), **135.4** (*C*(3``)H), **136.7** (Ar*C*(1```)H), **141.3** (Ar*C*(1`)), **145.2** (Ar*C*(7a)), **179.1** (*C*=O); **HRMS (ESI$^+$)** $C_{36}H_{28}NO_2{}^{35}ClNa$ $[M+Na]^+$ found 564.1679, requires 564.1701 (–3.9 ppm);

(*S,E*)-3-cinnamyl-3-hydroxy-5-methoxy-1-tritylindolin-2-one (64)

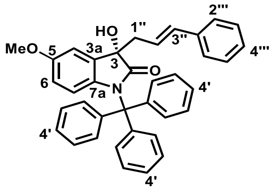

Following **General Procedure H**, allylic ether **S120** (107.2 mg, 0.1994 mmol), *t*Bu-BIMP (14.8 mg, 0.0201 mmol) in toluene (4 mL) followed by DBU (30 μL, 0.20 mmol) gave, after purification by flash column chromatography (20:80 EtOAc/hexane), the title compound as an off-white amorphous solid (65 mg, 61%); **IR** ν_{max} (film) 3377 (O-H), 3084, 3057, 3026 (C-H), 1717 (C=O), 1597 (C=C), 1159 (C-O); $\mathbf{[a]_D^{20}}$ +32.4 (*c* 0.88, $CHCl_3$); **Chiral HPLC analysis,** Chiralcel IB (90:10 hexane:IPA, flow rate 1 mLmin^{-1}, 211 nm, 30 °C) t_R (*R*)-**64**: 9.0 min, t_R (*S*)-**64**: 10.7 min, 11:89 e.r.; **^{1}H NMR (500 MHz, $CDCl_3$)** δ **2.79** (1H, s, O*H*), **2.88** (1H, dd, *J* 13.0, 9.2, C(1``)$H^A$$H^B$), **2.96** (1H, dd, *J* 13.1, 6.0, C(1``)$H^A$$H^B$), **3.72** (3H, s, OC$H_3$), **6.01** (1H, ddd, *J* 15.5, 9.1, 6.0, C(2``)*H*), **6.16** (1H, d, *J* 8.9, 1H, ArC(7)*H*), **6.45** (1H, dd, *J* 8.9, 2.8, ArC(6)*H*), **6.54** (1H, d, *J* 15.8, C(3``)*H*), **6.98** (1H, d, *J* 2.8, ArC(4)*H*), **7.09–7.25** (10H, m, ArC(3`,4`,4```)*H*), **7.28–7.40** (10H, m, ArC(2`,6`,2```,3```,5```,6```)*H*). **^{13}C{^{1}H} NMR (126 MHz, $CDCl_3$)** δ **43.7** (*C*(1``)H), **55.7** (O*C*$H_3$), **74.6** (N*C*$Ph_3$), **76.6** (*C*(3)), **109.4** (Ar*C*(4)H), **113.6** (Ar*C*(6)H), **117.1** (Ar*C*(7)H), **122.1** (*C*(2``)H), **126.4** (Ar*C*(2```)H), **127.0** (Ar*C*(4`)H), **127.7** (Ar*C*(3`,5`)H), **127.8** (Ar*C*(4```)H), **128.8** (Ar*C*(3```)H), **129.4** (Ar*C*(2`,6`)H), **131.0** (Ar*C*(3a)), **135.3** (*C*(3``)H), **136.2** (Ar*C*(7a)), **136.9** (Ar*C*(1```)), **141.8** (Ar*C*(1`)), **155.7** (Ar*C*(5)), **179.8** (*C*=O); **HRMS (ESI$^+$)** $C_{37}H_{31}NO_3Na$ $[M+Na]^+$ found 560.2182, requires 560.2196 (–2.5 ppm).

(*S,E*)-6-bromo-3-cinnamyl-3-hydroxy-1-tritylindolin-2-one (65)

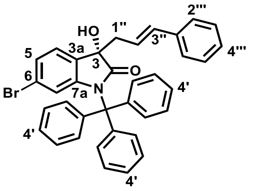

Following **General Procedure H**, allylic ether **S121** (117.1 mg, 0.1996 mmol), *t*Bu-BIMP (14.8 mg, 0.0201 mmol) in toluene (4 mL) followed by DBU (30 µL, 0.20 mmol) gave, after purification by flash column chromatography (20:80 EtOAc/hexane), the title compound as a yellow amorphous solid (75 mg, 64%); **IR** ν_{max} (film) 3374 (O-H), 3057, 3026 (C-H), 1717 (C=O), 1607, 1585 (C=C); $\mathbf{[a]_D^{20}}$ +50.8 (*c* 1.2, $CHCl_3$); **Chiral HPLC analysis,** Chiralcel IB (90:10 hexane:IPA, flow rate 1 $mLmin^{-1}$, 211 nm, 30 °C) t_R (*R*)-**65**: 8.4 min, t_R (*S*)-**65**: 10.7 min, 90:10 e.r.; **^{1}H NMR (500 MHz, $CDCl_3$)** δ **2.75** (1H, s, O*H*), **2.85** (1H, dd, *J* 13.0, 9.2, C(1``)$H^AH^B$), **2.95** (1H, ddd, *J* 13.1, 6.1, 1.6, 1H, C(1``)H^AH^B), **5.99** (1H, ddd, *J* 15.5, 9.1, 6.1, C(2``)*H*), **6.35** (1H, d, *J* 1.6, ArC(7)*H*), **6.52** (1H, d, *J* 15.9, C(3``)*H*), **7.11–7.20** (10H, m, ArC(5,3`,4`)*H*), **7.22–7.25** (2H, m, ArC(4,4```)*H*), **7.29–7.36** (10H, m, ArC(2`,3```,4```,5```)*H*); **^{13}C{^{1}H} NMR (126 MHz, $CDCl_3$)** δ **43.4** (*C*(1``)H), **74.9** (N*C*$Ph_3$), **75.9** (*C*(3)), **119.5** (Ar*C*(7)H), **121.5** (*C*(2``)H), **122.0** (Ar*C*(6)Br), **124.6** (Ar*C*(4)H), **125.8** (Ar*C*(5)H), **126.4** (Ar*C*(2```)H), **127.3** (Ar*C*(4`)H), **127.9** (Ar*C*(3`,5`)H), **128.0** (Ar*C*(4```)H), **128.8** (Ar*C*(3a)), **128.8** (Ar*C*(3```)H), **129.3** (Ar*C*(2`,6`)H), **135.7** (*C*(3``)H), **136.7** (Ar*C*(1```)), **141.3** (Ar*C*(1`)), **144.5** (Ar*C*(7a)), **179.7** (*C*=O); **HRMS (ESI$^+$)** $C_{36}H_{28}NO_2{}^{79}BrNa$ $[M+Na]^+$ found 608.1187, requires 608.1201 (–2.3 ppm).

8.6 Supplementary Data for [2,3]-rearrangement products and additional compounds

3-(1-Fluoro-1-phenylallyl)-3-hydroxy-1-tritylindolin-2-one (42)

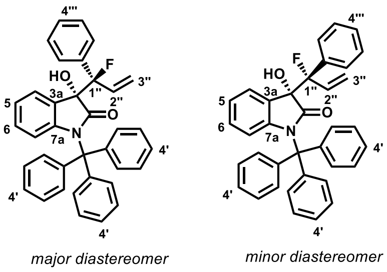

Following **General Procedure J**, allylic ether **41** (52.5 mg, 0.1 mmol), *t*Bu-BIMP (3.7 mg, 0.005 mmol), in diethylether (2 mL) gave a crude [2,3]-rearrangement product which was purified by flash column chromatography (eluent: hexane/EtOAc = 4:1) to afford the product (47.3 mg, 90%, 91:9 mixture of diastereomers) as a colourless amorphous solid. **IR** ν_{max} (film) 3412 (OH), 1724 (C=O), 1606 (C=C), 1490, 1463, 1448, 1309, 1184, 1159 (C-F), 1122; $[a]_D^{20}$ +84.8 (*c* 0.25, $CHCl_3$); **Chiral HPLC analysis**, Chiralpak ADH (90:10 hexane:IPA, flow rate 1ml·min^{-1}, 211 nm, 30 °C), major diastereomer: t_R (3*R*,1``*R*)-**42**: 7.56 min, $t_R$ (3*S*,1``*S*)-**42**: 12.03 min, 98:2 e.r.; minor diastereomer, t_R (3*S*,1``*R*)-**42**: 9.37 min, $t_R$ (3*R*,1``*S*)-**42**: 10.11 min, 91:9 e.r. ; **^{1}H NMR (500 MHz, $CDCl_3$)** δ **3.12** (0.09H, s, O*H* (minor)), **3.34** (0.91H, s, O*H* (major)), **5.43–5.66** (1.91H, m, C(3``)$H_2$ (major) and C(3``)H^AH^B (minor)), **5.79** (0.09H, d, *J* 17.1, C(3``)$H^AH^B$ (minor)), **6.05** (0.09H, d, *J* 8.3, ArC(7)*H* (minor)), **6.24** (0.91H, d, *J* 8.3, ArC(7)*H* (major)), **6.62–6.79** (1H, m, C(2``)*H* (major and minor)), **6.82** (1H, m, ArC(4)*H* (major) and ArC*H* (minor)), **6.87–6.93** (1.82H, m, ArC(5,6)*H* (major)), **6.95–6.99** (0.19H, m, ArC(4)*H* (minor) and ArC*H* (minor)), **7.00–7.06** (0.63H, m, ArC*H* (minor)), **7.14–7.23** (13.7H, m, ArC*H* (major)), **7.27–7.36** (2.53H, m, ArC*H* (major and minor)), **7.37–7.45** (2.83H, m, ArC*H* (major and minor)), **7.59–7.63** (0.19H, m, ArC*H* (minor)); **$^{13}C\{^1H\}$ NMR (126 MHz, $CDCl_3$)** δ **74.9** (N*C*Ph_3 (major)), **78.9** (d, $^2J_{C\text{-}F}$ 27.2, *C*(3)OH (major)), **98.7** (d, $^1J_{C\text{-}F}$ 187.9, *C*(1``)F (major)), **116.1** (Ar*C*(7)H (major)), **116.4** (Ar*C*(7)H (minor)), **118.8** (d, $^3J_{C\text{-}F}$ 14.2, *C*(3``)H_2 (minor)), **119.2** (d, $^3J_{C\text{-}F}$ 12.7,

C(3``)H_2 (major)), **122.1** (Ar*C*(4)H (major)), **122.6** (Ar*C*(4)H (minor)), **125.3** (Ar*C*(5)H (major)), **126.4** (d, $^{3}J_{C\text{-}F}$ 11.0, Ar*C*(2```,6```)H (major)), **127.0** (Ar*C*(4`)H (major)), **127.1** (Ar*C*(4`)H (minor)), **127.6** (Ar*C*(3`,5`)H (minor)), **127.7** (Ar*C*(3`,5`)H (major)), **128.0** (d, $^{4}J_{C\text{-}F}$ 2.2, Ar*C*(3```,5```)H (major)), **128.7** (Ar*C*(4```)H (major and minor)), **128.8** (Ar*C*(6)H (major and minor)), **129.3** (Ar*C*(2`,6`)H (major)), **129.7** (Ar*C*(2`,6`)H (minor)), **132.9** (d, $^{2}J_{C\text{-}F}$ 17.5, *C*(2``)H (minor)), **134.0** (d, $^{2}J_{C\text{-}F}$ 18.6, *C*(2``)H (major)), **137.2** (d, $^{2}J_{C\text{-}F}$ 21.8, Ar*C*(1```) (major)), **141.4** (Ar*C*(1`) (minor)), **141.8** (Ar*C*(1`) (major)), **144.5** (Ar*C*(7a) (major)), **177.8** (*C*=O (major)); **^{19}F NMR (470 MHz, $CDCl_3$)** δ **–167.20** (C(1``)*F*, major), **–167.37** (C(1``)*F*, minor). **HRMS (ESI$^+$)** $C_{36}H_{28}FNO_2Na$ $[M+Na]^+$ found 548.1997, requires 548.1996 (0.13 ppm).

3-Hydroxy-3-(1-phenylallyl)-1-tritylindolin-2-one (44)

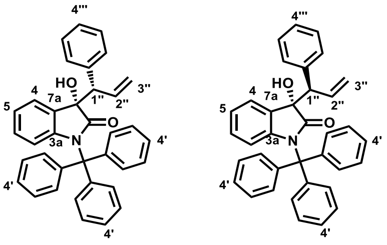

major diastereomer *minor diastereomer*

Following **General Procedure J**, **43** (101.5 mg, 0.1999 mmol), *t*Bu-BIMP (14.7 mg, 0.0199 mmol) in EtOAc (4 mL) gave a crude product which was purified by flash column chromatography (20:80 EtOAc/petrol) to afford:

44$_{major}$ as a colourless amorphous solid (73 mg, 72%); **IR** ν_{max} (film) 3397 (O-H), 3084, 3057, 3032 (C-H), 1709 (C=O), 1609, 1602 (C=C), 1188 (C-O); $[\mathbf{a}]_{\mathbf{D}}^{\mathbf{20}}$ +146.6 (*c* 0.47, $CHCl_3$); **Chiral HPLC analysis,** Chiralpak AD-H (90:10 hexane/IPA, flow rate 1 mL·min^{-1}, 211 nm, 30 °C) t_R (3*R*,1``*R*)-**44**: 8.5 min, $t_R$ (3*S*,1``*S*)-**44**: 27.5 min, 2:98 e.r.; **^{1}H NMR (500 MHz, $CDCl_3$)** δ **2.81** (1H, s, O*H*), **3.88** (1H, d, *J* 7.5, C(1``)*H*), **5.18** (1H, d, *J* 17.1, C(3``)H^AH^B), **5.37** (d, *J* 10.3, 1H, C(3``)$H^AH^B$), **6.15** (1H, d, *J* 8.2, ArC(7)*H*), **6.58** (1H, ddd, *J* 17.4, 10.2, 7.4, C(2``)*H*), **6.89** (1H, app t, *J* 7.9, ArC(6)*H*), **6.93–7.01** (9H, m, ArC(5,2`,6`,2```,6```)*H*), **7.10–7.17** (9H, m, ArC(3`,4`,5`)*H*), **7.29–7.36** (3H, m, ArC(3``,5``,4```)*H*), **7.46** (1H, d, *J* 7.4, ArC(4)*H*); **^{13}C{^{1}H} NMR (126 MHz, $CDCl_3$)** δ **58.7** (*C*(1``)H), **74.8** (N*C*Ph$_3$), **77.9** (*C*(3)), **116.5** (Ar*C*(7)H), **119.5** (*C*(3``)H$_2$), **122.5** (Ar*C*(5)H), **124.6** (Ar*C*(4)H), **126.9** (Ar*C*(4`)H), **127.6** (Ar*C*(3`,5`)H), **128.0** (Ar*C*(4```)H), **128.3** (Ar*C*(6)H), **128.4** (Ar*C*(3a), Ar*C*(3```,5```)H), **129.4** (Ar*C*(2`,6`)H), **129.9** (Ar*C*(2```,6```)H), **135.4** (*C*(2``)), **137.2** (Ar*C*(1```)), **141.6** (Ar*C*(1`)), **143.9** (Ar*C*(7a)), **179.8** (*C*=O); **HRMS** (ESI$^+$) $C_{36}H_{29}NO_2Na$ $[M+Na]^+$ found 530.2086, requires 530.2096 (–0.9 ppm);

44$_{minor}$ as a colourless oil (28 mg, 28%); **IR** ν_{max} (film) 3415 (O-H), 3086, 3057, 3032 (C-H), 1721 (C=O), 1609 (C=C), 1184 (C-O); $[\mathbf{a}]_{\mathbf{D}}^{\mathbf{20}}$ +75.6 (*c* 0.39, $CHCl_3$); **Chiral HPLC analysis** Chiralpak AD-H (90:10 hexane/IPA, flow rate 1 mL·min^{-1}, 211 nm, 30 °C) t_R (3*R*,1``*S*)-**44**: 10.0 min, $t_R$ (3*S*,1``*R*)-**44**: 17.1 min, 3:97 er; **^{1}H NMR (500 MHz, $CDCl_3$)** δ **3.06** (1H, s, O*H*), **3.91** (1H, d, *J* 10.3, C(1``)*H*), **5.42** (1H, dd, *J* 10.1,

1.8, C(3``)$H^A$$H^B$), **5.46** (1H, dd, *J* 17.0, 1.8, C(3``)$H^A$$H^B$), **6.04** (1H, d, *J* 8.2, ArC(7)*H*), **6.64** (1H, app dt, *J* 16.9, 10.2, C(2``)*H*), **6.79** (1H, app td, *J* 7.9, 1.5, ArC(6)*H*), **6.94** (1H, app t, *J* 7.5, ArC(5)*H*), **7.05–7.11** (6H, m, ArC(2`,6`)*H*), **7.11–7.14** (2H, m, ArC(2```,6```)*H*), **7.16–7.21** (9H, m, ArC(3`,4`,5`)*H*), **7.23–7.33** (4H, m, ArC(4, 3```,4```,5```)*H*); **^{13}C{^{1}H} NMR (126 MHz, $CDCl_3$)** δ **58.9** (*C*(1``)), **74.9** (N*C*$Ph_3$), **77.4** (*C*(3)OH), **116.5** (Ar*C*(7)H), **121.2** (*C*(3``)H_2), **122.4** (Ar*C*(5)H), **123.6** (Ar*C*(4)H), **127.0** (Ar*C*(4`)H), **127.5** (Ar*C*(4```)H), **127.6** (Ar*C*(3`,5`)H), **128.2** (Ar*C*(6)H), **128.3** (Ar*C*(3```,5```)H), **128.9** (Ar*C*(3a)), **129.5** (Ar*C*(2```,6```)H), **129.8** (Ar*C*(2`,6`)H), **134.1** (*C*(2``)H), **137.5** (Ar*C*(1```)), **141.6** (Ar*C*(1`)), **143.9** (Ar*C*(7a)), **179.1** (*C*=O); **HRMS** (ESI^+) $C_{36}H_{29}NO_2Na$ $[M+Na]^+$ found 530.2086, requires 530.2096 (–0.9 ppm).

(±)-3-Hydroxy-3-[2-(p-tolyl)but-3-en-2-yl]-1-tritylindolin-2-one (S122)

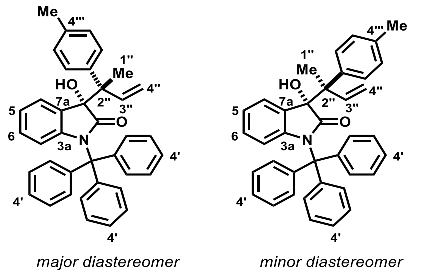

Following **General Procedure J**, allylic ether **S88** (535 mg, 1.0 mmol) and DBU (0.75 mL, 5.0 mmol) in toluene gave a crude product which was purified by flash column chromatography (eluent: hexane/ acetone = 8:1 to 4:1) to afford the product as a 89:11 mixture of diastereomers (150 mg, 28%) as a colourless amorphous solid. **IR** ν_{max} (film) 3423 (OH), 1711 (C=O), 1608 (C=C), 1595 (C=C), 1462, 1446, 1309, 1184, 1120; **^{1}H NMR (400 MHz, $CDCl_3$)** δ **1.74** (2.55H, s, C(2``)C*H*$_3$ (major)), **1.76** (0.35H, s, C(2``)C*H*$_3$ (minor)), **2.36** (0.35H, s, ArC(4```)C*H*$_3$ (minor)), **2.38** (2.60H, s, Ar*C*(4```)C*H*$_3$ (major)), **2.82** (0.78H, s, O*H* (major)), **2.90** (0.11H, s, O*H* (minor)), **4.99** (0.88H, dd, *J* 17.5, 1.3, C(4``)*H*$^A$H$^B$ (major)), **5.27** (0.88H, d, *J* 10.9, 1.3, C(4``)H^A*H*^{B} (major)), **5.46** (0.11H, dd, *J* 17.5, 1.3, C(4``)*H*$^A$H$^B$ (minor)), **5.53** (0.11H, d, *J* 11.0, 1.3, C(4``)H^A*H*^{B} (minor)), **6.13–6.19** (0.88H, m, ArC(7)*H* (major)), **6.19–6.22** (0.12H, m, ArC(7)*H* (minor)), **6.82**–**7.02** (3.16H, m, ArC*H* (major and minor) and ArC(3```)*H* (major and minor)), **7.05–7.13** (8.78H, m, ArC*H* (major and minor)), **7.13**–**7.21** (11.28H, m, ArC*H* (major and minor)); **^{13}C{^{1}H} NMR (101 MHz, $CDCl_3$)** δ **19.3** (C(2``)*C*H$_3$ (minor)), **20.1** (C(2``)*C*H$_3$ (major)), **21.2** (ArC(4```)*C*H$_3$ (minor)), **21.3** (ArC(4```)*C*H$_3$ (major)), **52.0** (*C*(2``) (major)), **75.0** (N*C*Ph$_3$ (major)), **79.4** (*C*(3)OH (major)), **115.9** (*C*(4``)H$_2$ (major)), **116.1** (Ar*C*(7)H (major)), **117.7** (*C*(4``)H$_2$ (minor)), **121.9** (Ar*C*(5)H (minor)), **122.0** (Ar*C*(5)H (major)), **125.0** (Ar*C*(4)H (major)), **125.4** (Ar*C*(4)H (minor)), **126.9** (Ar*C*(4`)H (major)), **127.6** (Ar*C*(3`,5`)H (major)), **128.0** (Ar*C*(6)H (major)), **128.1** (Ar*C*(6)H (minor)), **128.2** (Ar*C*(3```,5```)H (minor)), **128.3** (Ar*C*(3```,5```)H (major)), **128.7** (Ar*C*H (minor)), **129.4** (Ar*C*(2`,6`)H (major and minor)), **129.6** (Ar*C*(2```,6```)H (major)), **129.8** (Ar*C*(3a) (major)), **137.0** (Ar*C*(4```)CH$_3$ (major)), **137.5** (Ar*C*(1```) (major)), **139.7** (*C*(3``)H (minor)), **142.0**

(Ar*C*(1`) (major)), **142.1** (Ar*C*(1`) (minor)), **142.2** (*C*(3``)H (major)), **143.8** (Ar*C*(7a) (major)), **180.0** (*C*=O (major)), **180.2** (*C*=O (minor)),; **HRMS (ESI$^+$)** $C_{38}H_{33}NO_2Na$ $[M+Na]^+$ found 558.2403, requires 558.2404 (–0.17 ppm).

(±)-3-Hydroxy-3-[2-(4-methoxyphenyl)but-3-en-2-yl]-1-tritylindolin-2-one (S123)

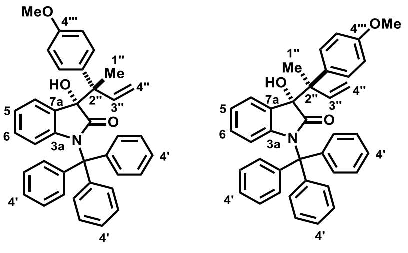

major diastereomer *minor diastereomer*

Following **General Procedure J**, allylic ether **16** (551 mg, 1.0 mmol) and DBU (0.75 mL, 5.0 mmol) in toluene gave a crude product which was purified by flash column chromatography (eluent: hexane/ acetone = 8:1 to 4:1) to afford the product as a 93:7 mixture of diastereomers (165 mg, 30%) as a colourless amorphous solid. **IR** ν_{max} (film) 3431 (OH), 1711 (C=O), 1608 (C=C), 1514, 1460, 1309, 1257, 1184, 1120 (C-O), 1035; **^{1}H NMR (500 MHz, CDCl$_3$)** δ δ **1.72** (2.70H, s, C(2``)C*H*$_3$ (major)), **1.75** (0.25H, s, C(2``)C*H*$_3$ (minor)), **2.82** (0.80H, s, O*H* (major)), **2.89** (0.08H, s, O*H* (minor)), **3.80** (0.26H, s, OC*H*$_3$ (minor)), **3.80** (2.75H, s, OC*H*$_3$ (major)), **4.97** (0.93H, dd, *J* 17.5, 1.3, C(4``)*H*$^A$H$^B$ (major)), **5.27** (0.93H, dd, *J* 10.9, 1.3, C(4``)H^A*H*^{B} (major)), **5.47** (0.07H, dd, *J* 17.5, 1.3, C(4``)*H*$^A$H$^B$ (minor)), **5.52** (0.07H, dd, *J* 10.9, 1.3, C(4``)H^A*H*^{B} (minor)), **6.12–6.19** (0.92H, m, ArC(7)*H* (major)), **6.19–6.24** (0.08H, m, ArC(7)*H* (minor)), **6.80–6.96** (5H, m, ArC*H* (major and minor) and ArC(3``)*H* (major and minor)), **7.05–7.24** (18H, m, ArC*H* (major and minor)); **$^{13}$C{$^1$H} NMR (126 MHz, CDCl$_3$)** δ **19.3** (C(2``)*C*H$_3$ (minor)), **20.4** (C(2``)*C*H$_3$ (major)), **51.9** (*C*(2``) (major)), **55.3** (O*C*H$_3$ (major)), **75.0** (N*C*Ph$_3$ (major), **79.5** (*C*(3)OH (major)), **112.9** (Ar*C*H (major)), **113.3** (Ar*C*H (minor)), **115.9** (*C*(4``)H$_2$ (major)), **116.1** (Ar*C*(7)H (major)), **117.6** (*C*(4``)H$_2$ (minor)), **121.9** (Ar*C*(5)H (minor)), **122.0** (Ar*C*(5)H (major)), **125.0** (Ar*C*(4)H (major)), **125.4** (Ar*C*(4)H (minor)), **126.9** (Ar*C*(4`)*H* (major)), **127.6** (Ar*C*(3`,5`)H (major)), **128.0** (Ar*C*(6)H (major)), **128.1** (Ar*C*(6)H (minor)), **129.4** (Ar*C*(major)H), **129.8** (Ar*C*(3a) (major)), **130.9** (Ar*C* (major)), **132.5** (Ar*C*(1```) (minor)), **142.0** (Ar*C*(1`) (major)), **142.1** (Ar*C*(1`) (minor)), **142.2** (*C*(3``)H (major)), **143.8** (Ar*C*(7a) (major)), **159.0** (Ar*C*(4```)OMe (major)), **180.1** (*C*=O (major)); **HRMS (ESI$^+$)** $C_{38}H_{33}NO_3Na$ [M+Na]$^+$ found 574.2354, requires 574.2353 (–0.18 ppm).

(±)-3-[2-(4-Fluorophenyl)but-3-en-2-yl]-3-hydroxy-1-tritylindolin-2-one (S124)

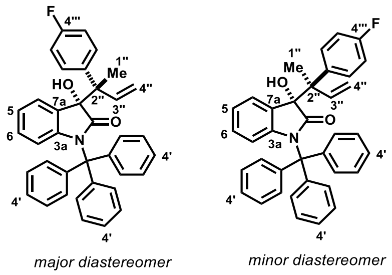

Following **General Procedure J**, allylic ether **11** (539 mg, 1.0 mmol) and DBU (0.75 mL, 5.0 mmol) in toluene gave a crude product which was purified by flash column chromatography (eluent: hexane/ acetone = 8:1 to 4:1) to afford the product as a 89:11 mixture of diasteromers (189 mg, 35%) as a colourless amorphous solid. **IR** ν_{max} (film) 3423 (OH), 1707 (C=O), 1602 (C=C), 1508, 1460, 1446, 1309, 1226 (C-F), 1184, 1165, 1118; **^{1}H NMR (500 MHz, CDCl$_3$)** δ **1.73** (2.60H, s, C*H*$_3$ (major)), **1.75** (0.36H, s, C*H*$_3$ (minor)), **2.85** (0.83H, s, O*H* (major)), **2.91** (0.12H, s, O*H* (major)), **4.95** (0.88H, dd, *J* 17.6, 1.2, C(4``)$H^A$H$^B$ (major)), **5.29** (0.88H, dd, *J* 11.0, 1.2, C(4``)H^AH^B (major)), **5.48** (0.11H, dd, *J* 17.5, 1.2, C(4``)$H^A$H$^B$ (minor)), **5.55** (0.11H, dd, *J* 11.0, 1.2, C(4``)H^AH^B (minor)), **6.13–6.22** (0.88H, m, ArC(7)*H* (major)), **6.24** (0.12H, d, *J* 8.0, ArC(7)*H* (minor)), **6.80–6.96** (3.15H, m, ArC(5,6)*H* (major), C(3``)*H* and ArC*H* (minor)), **6.96–7.02** (2.07H, m, ArC(3```,5```)*H* (major)), **7.03–7.14** (6.21H, m, ArC*H* (major and minor)), **7.14–7.25** (12H, m, ArC*H* (major and minor)), **7.27–7.37** (0.34H, m, ArC*H* (minor)); **^{13}C{^{1}H} NMR (126 MHz, CDCl$_3$)** δ **19.2** (*C*H$_3$ (major)), **20.7** (*C*H$_3$ (minor)), **52.1** (*C*(2``) (major)), **75.2** (N*C*Ph$_3$ (major)), **79.4** (*C*(3)OH (major)), **114.3** (d, $^2J_{C\text{-}F}$ 20.8, Ar*C*(3```,5```)H (major)), **114.6** (d, $^2J_{C\text{-}F}$ 20.9, Ar*C*(3```,5```)H (minor)), **116.2** (Ar*C*(7)H (minor)), **116.3** (Ar*C*(7)H (major)), **116.3** (*C*(4``)H$_2$ (major)), **118.0** (*C*(4``)H$_2$ (minor)), **122.0** (Ar*C*(5)H (minor)), **122.2** (Ar*C*(5)H (major)), **124.9** (Ar*C*(4)H (major)), **125.3** (Ar*C*(4)H (minor)), **127.0** (Ar*C*(4`)H (major)), **127.7** (Ar*C*(3`,5`)H (major)), **128.2** (Ar*C*(6)H (major)), **128.3** (Ar*C*(6)H (minor)), **129.4** (Ar*C*(2`,6`)H (major)), **130.1** (d, $^3J_{C\text{-}F}$ 7.7, Ar*C*(2```,6```)H (minor)), **131.6** (d, $^3J_{C\text{-}F}$ 7.7, Ar*C*(2```,6```)H (major)), **136.4** (d, $^4J_{C\text{-}F}$ 3.2, Ar*C*(1```) (major)), **139.6** (*C*(3``)H (minor)), **141.9** (*C*(3``)H (major)), **141.9** (Ar*C*(1```) (major)), **142.0** (Ar*C*(1```) (minor)),

143.8 (Ar*C*(7a) (major)), **144.0** (Ar*C*(7a) (minor)), **162.1** (d, $^{1}J_{C\text{-}F}$ 241.9, Ar*C*(4```)F (major)), **162.3** (d, $^{1}J_{C\text{-}F}$ 247.3, Ar*C*(4```)F (major)), **179.9** (*C*=O); **^{19}F{^{1}H} NMR (376 MHz, $CDCl_3$)** δ **–115.6** (ArC(4```)*F* (minor)), **–115.3** (ArC(4```)*F* (major)); **HRMS (ESI^+)** $C_{37}H_{30}FNO_2Na$ $[M+Na]^+$ found 562.2150, requires 562.2153 (–0.43 ppm).

(±)-3-[2-(4-Bromophenyl)but-3-en-2-yl]-3-hydroxy-1-tritylindolin-2-one (S125)

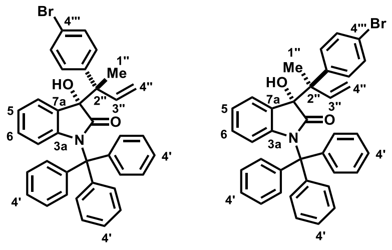

major diastereomer *minor diastereomer*

Following **General Procedure J**, allylic ether **S85** (599 mg, 1.0 mmol) and DBU (0.75 mL, 5.0 mmol) in toluene gave a crude product which was purified by flash column chromatography (eluent: hexane/ acetone = 8:1 to 4:1) to afford the product as a 88:12 mixture of diastereomers (251 mg, 42%) as a colourless amorphous solid. **IR** ν_{max} (film) 3473 (OH), 1707 (C=O), 1606 (C=C), 1485, 1463, 1309, 1182, 1165; **^{1}H NMR (500 MHz, $CDCl_3$)** δ **1.71** (2.60H, s, C*H*$_3$ (major)), **1.74** (3H, s, C*H*$_3$ (minor)), **2.84** (0.78H, s, O*H* (major)), **2.90** (0.13H, s, O*H* (minor)), **4.93** (0.87H, d, *J* 17.6, C(4``)*H*$^A$H$^B$ (major)), **5.29** (0.88H, d, *J* 11.0, C(4``)H^A*H*^{B} (major)), **5.47** (0.12H, d, *J* 17.5, C(4``)*H*$^A$H$^B$ (minor)), **5.55** (0.12H, d, *J* 11.0, C(4``)H^A*H*^{B} (minor)), **6.12–6.22** (0.87H, m, ArC(7)*H* (major)), **6.22–6.28** (0.13H, m, ArC(7)*H* (minor)), **6.79–6.94** (3H, m, ArC(5,6)*H* (major and minor), C(3``)*H* (major and minor)), **7.02–7.15** (8H, m, ArC*H* (major and minor)), **7.15–7.24** (10H, m, ArC*H* (major and minor)), **7.36–7.49** (2H, m, ArC(2```,6```)*H* (major and minor)); **^{13}C{^{1}H} NMR (126 MHz, $CDCl_3$)** δ **19.1** (*C*H$_3$ (minor)), **20.4** (*C*H$_3$ (major)), **52.3** (*C*(2``) (major)), **75.2** (*NC*Ph$_3$ (major)), **79.2** (*C*(3)OH (major)), **116.3** (Ar*C*(7)H (minor)), **116.3** (Ar*C*(7)H (major)), **116.4** (*C*(4``)H$_2$ (major)), **118.2** (*C*(4``)H$_2$ (minor)), **121.5** (Ar*C*(4```)Br (minor)), **121.7** (Ar*C*(4```)Br (major)), **122.1** (Ar*C*(5)H (minor)), **122.2** (Ar*C*(5)H (major)), **124.9** (Ar*C*(4)H (major)), **125.3** (Ar*C*(4)H (minor)), **127.0** (Ar*C*(4`)H (major and minor)), **127.7** (Ar*C*(3`,5`)H (major and minor)), **128.2** (Ar*C*(6)H (major)), **128.3** (Ar*C*(6)H (minor)), **129.3** (Ar*C*(2`,6`)H (major)), **129.4** (Ar*C*(2`,6`)H (minor)), **130.3** (Ar*C*(2```,6```)H (minor)), **130.7** (Ar*C*(2```,6```)H (major)), **131.0** (Ar*C*(3```,5```)H (minor)), **131.7** (Ar*C*(3```,5```)H (major)), **139.2** (*C*(3``)H (minor)), **139.8** (Ar*C*(1```) (major)), **141.6** (*C*(3``)H (major)), **141.9** (Ar*C*(1`) (major)), **142.0** (Ar*C*(1`) (minor)), **142.1** (Ar*C*(7a)

(minor)), **143.7** (Ar*C*(7a) (major)), **179.9** (*C*=O (major));

(±)-3-Hydroxy-3-{2-[4-(trifluoromethyl)phenyl]but-3-en-2-yl}-1-tritylindolin-2-one (S126)

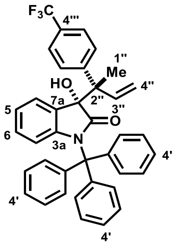
major diastereomer

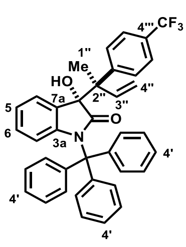
minor diastereomer

Following **General Procedure J**, allylic ether **S86** (589 mg, 1.0 mmol) and DBU (0.75 mL, 5.0 mmol) in toluene gave a crude product which was purified by flash column chromatography (eluent: hexane/ acetone = 8:1 to 4:1) to afford the product as 80:20 mixture of diasteromers (218 mg, 37%) as a colourless amorphous solid. **IR** ν_{max} (film) 3448 (OH), 1709 (C=O), 1606 (C=C), 1463, 1325 (C-F), 1157, 1122, 1074; **^{1}H NMR (500 MHz, $CDCl_3$)** δ **1.76** (2.42H, s, CH_3 (major)), **1.77** (0.61H, s, CH_3 (minor)), **2.85** (0.71H, s, O*H* (major)), **2.94** (0.19H, s, O*H* (minor)), **4.92** (0.79H, dd, *J* 17.5, 1.0, C(4``)$H^A$H$^B$ (major)), **5.31** (1H, dd, *J* 10.9, 1.0, C(4``)H^AH^B (major)), **5.50** (0.20H, dd, *J* 17.5, 1.1, C(4``)$H^A$H$^B$ (minor)), **5.58** (1H, dd, *J* 11.0, 1.0, C(4``)H^AH^B (minor)), **6.23** (0.80H, dd, *J* 7.5, 1.4, ArC(7)*H* (major)), **6.28** (0.20H, d, *J* 8.1, ArC(7)*H* (minor)), **6.77–7.00** (4.09H, m, ArC(4,5,6)*H* (major and minor), ArC(3``)*H* (major and minor)), **7.09–7.21** (15.22H, m, ArC(3`,4`,5`)*H* (major and minor)), **7.39–7.60** (4.10H, m, ArC(2```,3```,5```,6```)*H* (major and minor)); **^{13}C{^{1}H} NMR (126 MHz, $CDCl_3$)** δ **19.1** (*C*H_3 (minor)), **20.5** (*C*H_3 (major)), **51.0** (*C*(2``) (minor))**,** **52.5** (*C*(2``) (major))**,** **75.3** (*N*CPh_3 (major)), **79.1** (*C*(3)OH (major)), **116.3** (Ar*C*(7)H (minor)), **116.4** (Ar*C*(7)H (major)), **116.7** (*C*(4``)$H_2$ (major)), **118.3** (*C*(4``)H_2 (minor)), **122.2** (Ar*C*(5)H (minor)), **122.3** (Ar*C*(5)H (major)), **124.4** (q, $^3J_{C\text{-}F}$ 3.5, *C*(3```,5```)H (major)), **124.7** (q, $^3J_{C\text{-}F}$ 3.3, *C*(3```,5```)H (minor)), **125.0** (Ar*C*(4)H (major)), **125.3** (Ar*C*(4)H (minor)), **126.5** (q, $^1J_{C\text{-}F}$ 277.3, *C*F_3 (major)), **127.1** (Ar*C*(4')H (major)), **127.7** (Ar*C*(3`,5`)H (major)), **128.4** (Ar*C*(6)H (major)), **128.4** (Ar*C*(6)H (minor)), **128.9** (Ar*C*), **129.2** (Ar*C*(2`,6`)H (major)), **129.3** (Ar*C*(2`,6`)H), **130.3** (Ar*C*), **139.3** (*C*(3``)H (minor)), **141.3** (*C*(3``)H (major)), **141.9** (Ar*C*(1`) (major)), **142.0** (Ar*C*(1`) (minor)), **143.7** (Ar*C*(7a) (major)),

143.9 (Ar*C*(7a) (minor)), **145.0** (Ar*C*(1```) (major)), **147.3** (Ar*C*(1```) (minor)), **179.7** (*C*=O (major)), **179.9** (*C*=O (minor)); **^{19}F{^{1}H} NMR (376 MHz, $CDCl_3$)** δ **–62.2** (C*F*3, major); –**62.3** (C*F*3, minor). **HRMS (ESI^{+})** $C_{38}H_{30}F_3NO_2Na$ $[M+Na]^+$ found 612.2113, requires 612.2121 (–1.28 ppm).

((*E*)-1-Cyclopropyl-3-phenylbut-2-en-1-yl)-3-hydroxy-1-tritylindolin-2-one (S127)

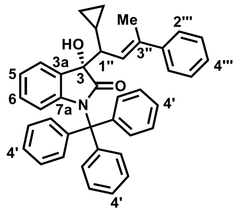

Following **General Procedure G**, allylic ether **S82** (56.1 mg, 0.1 mmol), *t*Bu-BIMP (14.7 mg, 0.02 mmol), 4-NHAc-TEMPO (4.3 mg, 0.02 mmol) in mesitylene (2 mL) gave a crude [1,2]-rearrangement product which was purified by flash column chromatography (eluent: hexane/ EtOAc = 4:1 to 3:1) to afford the product as 1.1:1 mixture of diastereomers (42.6 mg, 76%) as a colourless amorphous solid. **IR** ν_{max} (film) 3392 (OH), 1713 (C=O), 1608 (C=C), 1597 (C=C), 1490, 1463, 1448, 1317, 1263, 1182; **Chiral HPLC analysis**, Chiralpak ID (95:5 hexane:IPA, flow rate 1 ml·min^{-1}, 211 nm, 30 °C) major diastereomer: t_R (*R*,*R*)-**S118**:13.3min, t_R (*S*,*S*)-**S118**: 23.1min; 21:79 e.r.; minor diastereomer t_R (*S,R*)-**S118**: 14.5min, t_R (*R,S*)-**S118**: 15.7min; 79:21 e.r.; **^{1}H NMR (500 MHz, CDCl$_3$)** δ **0.01–0.08** (0.42H, m, CH((C*H*^{A}H^B)(CH^{C}H^D)) (minor)), **0.11–0.18** (0.57H, m, CH((C*H*^{A}H^B)(CH$_2$^{C}H^D) (major)), **0.18–0.24** (0.58H, m, CH((CH^{A}H^B)(C*H*^{C}H^D) (major)), **0.38–0.47** (1.57H, m, CH((CH^A*H*^{B})(CH^C*H*^{D}) (major and minor)), **0.49–0.58** (1H, m, CH((CH^A*H*^{B})(CH^{C}H^D)) (minor)), **0.59–0.68** (1H, m, CH((CH^{A}H^B)(CH^C*H*^{D})) (minor)), **1.01–1.11** (0.42H, m, *H*(CH$_2$)$_2$ (minor)), **1.15–1.23** (0.59H, m, C*H*(CH$_2$)$_2$ (minor)), **1.90** (1.26H, d, *J* 1.0, C*H*$_3$ (minor)), **2.12** (3H, d, *J* 1.5, C*H*$_3$ (major)), **2.51** (1H, dd, *J* 9.0, 10.5, C(1``)*H* (minor)), **2.83** (0.45H, s, O*H* (major)), **2.86–2.91** (0.64H, m, C(1``)*H* (major)), **3.12** (0.42H, s, O*H* (minor)), **5.55** (0.57H, dd, *J* 10.8, 1.6, C(2``)*H* (major)), **5.94** (0.43H, dd, *J* 10.7, 1.6, C(2``)*H* (minor)), **6.24** (0.43H, d, *J* 8.1, ArC(7)*H*, (minor)), **6.27** (0.57H, d, *J* 8.1, ArC(7)*H*, (major)), **6.86** (0.44H, app td, *J* 7.9, 1.5, ArC(6)*H* (minor)), **6.89–6.95** (1H, m, ArC(6)*H* (major) and ArC*H* (minor)), **6.97** (0.62H, app td, *J* 7.5, 1.0, ArC(5)*H* (major)), **7.10**–**7.19** (9H, m, ArC*H* (major and minor)), **7.26**–**7.32** (2.47H, m, ArC*H* (major and minor)), **7.32**–**7.40** (9H, m, ArC*H* (major and minor)), **7.50** (0.56H, dd, *J* 7.0, 1.5, ArC(4)*H* (major)); **^{13}C{^{1}H} NMR (126 MHz, CDCl$_3$) 1.8** (CH((*C*H^{A}H^B)(CHC^{C}H^D)) (major)), **2.1**

(CH((*C*H^{A}H^B)(CH^{C}H^D)) (minor)), **3.05** (CH((CH^{A}CH^B)(*C*H^{C}H^D)) (major)), **5.7** (CH((CH^{A}H^B)(*C*H^{C}H^D)) (minor)), **10.5** (*C*H(CH$_2$)$_2$ (major)), **10.8** (*C*H(CH$_2$)$_2$ (minor)), **16.7** (*C*H$_3$ (minor)), **16.8** (*C*H$_3$ (major)), **49.9** (*C*(1``)H (major)), **52.0** (*C*(1``)H (minor)), **74.7** (N*C*Ph$_3$ (major)), **75.0** (N*C*Ph$_3$ (minor)), **78.4** (*C*(3)OH (major)), **78.6** (*C*(3)OH (minor)), **116.2** (Ar*C*(7)H (major)), **116.4** (Ar*C*(7)H (minor)), **122.3** (Ar*C*(5)H) (minor)), **122.3** (Ar*C*(5)H (major)), **123.4** (*C*(2``)H (major)), **123.7** (Ar*C*(4)H (minor)), **124.5** (Ar*C*(4)H (major)), **124.8** (*C*(2``)H (minor)), **126.0** (Ar*C*(2```,6```)H (minor)), **126.0** (Ar*C*(2```,6```)H (major)), **126.9** (Ar*C*(4`)H (major)), **127.0** (Ar*C*(4`)H (minor)), **127.2** (Ar*C*(4```)H (minor)), **127.4** (Ar*C*(4```)H (major)), **127.6** (Ar*C*(3`,5`)H (major)), **127.7** (Ar*C*(3`,5`)H (minor)), **128.0** (Ar*C*(6)H) (minor), **128.1** (Ar*C*(6)H (major)), **128.5** (Ar*C*(3```,5```)H (major and minor)), **129.4** (Ar*C*(2`,6`)H (major)), **129.5** (Ar*C*(3a) (major)), **129.6** (Ar*C*(2`,6`)H (minor)), **130.0** (Ar*C*(3a) (minor)), **138.0** (*C*(3``)Ar (minor)), **139.9** (*C*(3``)Ar (major)), **141.9** (ArC(1`) (minor)), **141.9** (ArC(1`)(major)), **143.3** (Ar*C*(1```) (major)), **143.5** (Ar*C*(1```) (minor)), **143.8** (Ar*C*(7a) (minor)), **143.9** (Ar*C*(7a) (major)), **179.9** ((*C*=O) (minor)), **180.2** ((*C*=O) (major)); **^{13}C{^{1}H} NMR (126 MHz, CDCl$_3$)**; **HRMS (ESI$^+$)** $C_{39}H_{33}NO_2Na$ [M+Na]$^+$ found 584.2535, requires 584.2560 (–4.3 ppm).

Ethyl (*E*)-4-(3-hydroxy-2-oxo-1-tritylindolin-3-yl)crotonate (70)

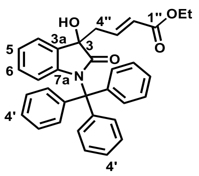

To a 7 mL vial was placed ethyl (*E*)-4-((2-oxo-1-tritylindolin-3-yl)oxy)crotonate **69** (100.5 mg, 0.1996 mmol) and *t*Bu-BIMP (29.6 mg, 0.0401 mmol), then PhMe (4 mL) was added. The reaction was stirred at rt for 1 h, then heated to 40 °C and stirred for 2 h. The mixture was then filtered through a short silica plug, eluting with hexane then EtOAc. The filtrate was concentrated under reduced pressure and the residue was purified by flash column chromatography (15:5:3 CH_2Cl_2/hexane/EtOAc) to give the title compound as a colourless oil (34 mg, 34%). $\mathbf{[a]_D^{20}}$ +29.4 (*c* 0.78, $CHCl_3$); **Chiral HPLC analysis,** Chiralpak IA (90:10 hexane:IPA, flow rate 1 mL·min^{-1}, 211 nm, 30 °C) t_R (*R*)-**70**: 13.3 min, t_R (*S*)-**70**: 15.0 min, 5:95 e.r.; **IR** ν_{max} (film) 3420 (O-H), 3088, 3057, 3034, 2982 (C-H), 1717 (C=O), 1611 (C=C), 1114 (C-O); ^{1}**H NMR** (500 MHz, $CDCl_3$) δ 1.21 (t, *J* = 7.1 Hz, 3H, OCH_2CH_3), 2.71 (s, 1H, O*H*), 2.83 (ddd, *J* = 13.1, 9.1, 1.1 Hz, 1H, C(4")H^AH^B), 2.94 (ddd, *J* = 13.2, 6.5, 1.6 Hz, 1H, C(4")H^AH^B), 4.16 (app. qd, *J* = 7.2, 2.1 Hz, 2H, OCH_2CH_3), 5.96 (dt, *J* = 15.6, 1.3 Hz, 1H, C(2")*H*), 6.31 (d, *J* = 8.1 Hz, 1H, ArC(7)*H*), 6.80 (ddd, *J* = 15.6, 9.1, 6.5 Hz, 1H, C(3')*H*), 6.92 (td, *J* = 7.9, 1.5 Hz, 1H, ArC(6)*H*), 6.98 (td, *J* = 7.5, 1.0 Hz, 1H, ArC(5)*H*), 7.17 – 7.23 (m, 3H, ArC(4')*H*), 7.22 – 7.28 (m, 6H, ArC(3',5')*H*), 7.34 (dd, *J* = 7.3, 1.5 Hz, 1H, ArC(4)*H*), 7.35 – 7.40 (m, 6H, ArC(2',6')*H*); ^{13}**C{^{1}H} NMR** (126 MHz, $CDCl_3$) δ 14.3 (CH_3), 42.6 (*C*(4")H_2), 60.6 (OCH_2), 74.8 ($NCPh_3$), 75.6 (*C*(3)), 116.6 (Ar*C*(7)H), 123.1 (Ar*C*(5)H), 123.5 (Ar*C*(4)H), 126.4 (Ar*C*(2")H), 127.2 (Ar*C*(4')H), 127.9 (Ar*C*(3',5')H), 128.6 (Ar*C*(6)H), 129.1 (Ar*C*(3a)), 129.5 (Ar*C*(2',6')H), 140.7 (Ar*C*(3")H), 141.7 (Ar*C*(1')), 143.0 (Ar*C*(7a)), 165.9 (*C*(1")=O), 179.5 (*C*(2)=O); **HRMS** (ESI$^+$) $C_{33}H_{29}NO_4Na$ [M+Na]$^+$ found 526.1983, requires 526.1989 (−1.1 ppm);

But-3-en-2-ylbenzene (S22)

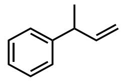

To a flame-dried 250 mL 2-necked round-bottomed flask was placed potassium tert-butoxide (2.71 g, 24.2 mmol) and methyltriphenylphosphonium bromide (5.73 g, 16.0 mmol) under N_2 atmosphere, then anhydrous THF (40 mL) was added. The mixture was stirred for 50 min at rt, then cooled to 0 °C and 2-phenylpropanal (1.60 mL, 11.9 mmol) was added dropwise. The reaction was allowed to warm to rt and stirred overnight at rt, then pentane (40 mL) was added and the mixture was filtered. The filtrate was concentrated under reduced pressure (40 °C, 280 mbar) and the residue was purified by flash column chromatography (pentane) to give the title compound (529 mg, 33%) as a colourless oil, with spectroscopic data in accordance with the literature.[58] Some product loss occurred during removal of THF under reduced pressure. **^{1}H NMR** (500 MHz, $CDCl_3$) δ **1.37** (d, *J* = 7.1 Hz, 3H, CH_3), **3.48** (app. p, *J* = 7.0 Hz, 1H, CH_3CH), **5.04** (app. dt, *J* = 10.3, 1.5 Hz, 1H, $CH{=}CH_{cis}H_{trans}$), **5.06** (app. dt, *J* = 17.2, 1.7 Hz, 1H, $CH{=}CH_{cis}H_{trans}$), **6.02** (ddd, *J* = 16.9, 10.3, 6.4 Hz, 1H, $CH{=}CH_2$), **7.17 – 7.25** (m, 3H, ArC(2,4,6)*H*), **7.28 – 7.37** (m, 2H, ArC(5)*H*); **^{13}C{^{1}H} NMR** (126 MHz, $CDCl_3$) δ **20.9** (CH_3), **43.3** (CH_3CH), **113.2**, ($CH{=}CH_2$), **126.3** (Ar*C*(4)H), **127.4** (Ar*C*(2,6)H), **128.6** (Ar*C*(3,5)H), **143.4** ($CH{=}CH_2$), **145.7** (Ar*C*(1)).

But-2-en-2-ylbenzene (S23)

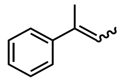

To a flame-dried 250 mL 2-necked round-bottomed flask was placed potassium tert-butoxide (348.7 mg, 3.108 mmol) and ethyltriphenylphosphonium bromide (984.5 mg, 2.652 mmol) under N_2 atmosphere, then anhydrous THF (6.7 mL) was added. The mixture was stirred for 1 h at rt, then cooled to 0 °C and acetophenone (0.23 mL, 1.9 mmol) was added dropwise. The reaction was allowed to warm to rt and stirred overnight at rt, then filtered through a short silica plug, eluting with pentane. The filtrate was concentrated under reduced pressure (40 °C, 280 mbar) and the residue was purified by flash column chromatography (pentane) to give the title compound (85:15 *Z*/*E*, 254 mg, 98%) as a colourless oil, with spectroscopic data in accordance with literature.[59] **^{1}H NMR** (500 MHz, $CDCl_3$) δ **1.60** (dq, *J* = 6.9, 1.6 Hz, 2.55H, (*Z*)-CHC*H*$_3$), **1.80** (dq, *J* = 6.8, 1.1 Hz, 0.45H, (*E*)-CHC*H*$_3$), **2.01 – 2.06** (m, 3H, HC=CC*H*$_3$), **5.57** (qq, *J* = 6.9, 1.5 Hz, 0.85H, (*Z*)-CH$_3$C*H*), **5.86** (qq, *J* = 6.9, 1.4 Hz, 0.15H, (*E*)-CH$_3$C*H*), **7.18** – **7.22** (m, 1.70H, (*Z*)-ArC(2,6)*H*), **7.20** – **7.27** (m, 1H, ArC(4)*H*), **7.27** – **7.40** (m, 2.30H, (*Z*)-ArC(3,5)*H* and (*E*)-ArC(2,3,5,6)*H*); **^{13}C{^{1}H} NMR** (126 MHz, $CDCl_3$) δ **14.5** ((*E*)-CH*C*H$_3$), **15.0** ((*Z*)-CH*C*H$_3$), **15.6** ((*E*)-HC=C*C*H$_3$), **25.5** ((*Z*)-HC=C*C*H$_3$), **121.7** ((*Z*)-*C*HCH$_3$), **122.6** ((*E*)-*C*HCH$_3$), **125.7** ((*E*)-Ar*C*(4)H), **126.5** ((*Z*)-Ar*C*(4)H), **126.5** ((*E*)-Ar*C*(3,5)H), **128.2** ((*Z*)-Ar*C*(2,6)H), **128.2** ((*Z*)-Ar*C*(3,5)H), **128.3** ((*E*)-Ar*C*(2,6)H), **135.7** ((*E*)-HC=*C*CH$_3$), **136.9** ((*Z*)-HC=*C*CH$_3$), **142.0** ((*Z*)-Ar*C*(1)), **144.2** ((*E*)-Ar*C*(1)).

(*E*)-2,2,6,6-tetramethyl-1-((3-(4-fluorophenyl)but-2-en-1-yl)oxy)piperidine (T-fa)

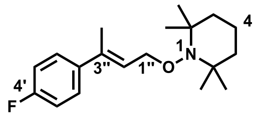

To a 5 mL round bottomed flask charged with 1-benzyl-3-(2-(4-fluorophenyl)but-3-en-2-yl)-3-hydroxyoxindole **S17** (26.0 mg, 0.067 mmol) and TEMPO (2,2,6,6-tetramethyl-piperidin-1-yloxyl) (10.5 mg, 0.067 mmol) was added mesitylene (1.3 mL, 0.05 M) and DBU (10 μL, 0.067 mmol) while under Ar. The reaction was stirred at rt overnight, then directly submitted to flash column chromatography (CH_2Cl_2:Et_2O 49:1 → 47.5:2.5 → 45:5) to give three fractions: TEMPO-adduct **T-fa** as colourless glassy solid (2.9 mg, 0.009 mmol, 14%), unreacted [2,3]-Wittig rearrangement product **S17** as yellow oil (5.0 mg, 0.013 mmol, 19%) and formal [1,2]-Wittig rearrangement product **S18** as white solid (15.7 mg, 0.040 mmol, 60%).

IR ν_{max} (film) 2974, 2928 (C-H), 1603, 1508, 1470, 1449 (C=C), 1373, 1358, 1231, 1161, 1132, 1015 (C-O) 837, 818; **^{1}H NMR** (700 MHz, $CDCl_3$) δ **1.12** (s, 6H, N(C(2,6)(CH_3)^A(CH$_3$)^B)), **1.22** (s, 6H, N(C(2,6)(CH$_3$)^A(CH_3)^B)$_2$), **1.24** – **1.39** (m, 2H, C(4)H_2), **1.42** – **1.52** (m, 4H, C(3,5)H_2), **2.03** (dt, *J* = 1.4, 1.0 Hz, 3H, C(4")H_3), **4.48** (dq, *J* = 6.5, 1.0 Hz, 2H, C(1")H_2), **5.88** (tq, *J* = 6.5, 1.4 Hz, 1H, C(2")*H*), 6**.94** – **7.04** (2H, m, ArC(3',5')*H*), **7.34** – **7.42** (2H, m, ArC(2',6')*H*); **^{13}C{^{1}H} NMR** (176 MHz, $CDCl_3$) δ **16.6** (*C*(4)H$_2$), **17.3** (*C*(4")H$_3$), **20.3** (N(C(2,6)(*C*H$_3$)^A(CH$_3$)^B)$_2$), **33.3** (N(C(2,6)(CH$_3$)^A(*C*H$_3$)^B)$_2$), **39.8** (*C*(3,5)H$_2$), **59.9** ((*C*(2,6)), **75.0** (*C*(1")H$_2$), **115.1** (d, *J* = 21.2 Hz, Ar*C*(3',5')H), **123.9** (*C*(2")H), **127.4** (d, *J* = 7.9 Hz, Ar*C*(2',6')H), **136.0** (*C*(3")), **139.3** (d, *J* = 3.4 Hz, Ar*C*(1')), **162.2** (d, *J* = 245.5 Hz, Ar*C*(4')F); **^{19}F NMR** (659 MHz, $CDCl_3$) δ **−116.01** (tt, *J* = 8.8, 5.3 Hz, 1F, ArC^4*F*); **HRMS** (ESI$^+$) $C_{19}H_{29}FNO$ [M+H]$^+$ found 306.2229, requires 306.2228 (+0.4 ppm).

1-(cinnamyloxy)-2,2,6,6-tetramethylpiperidine (T-ca)

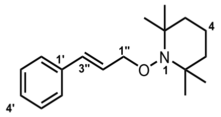

To a 7 mL vial was added 3-hydroxy-3-(1-phenylallyl)-1-tritylindolin-2-one **44** (101.2 mg, 0.1994 mmol, 72:28 d.r., 99:1 / 97:3 e.r.), TEMPO (2,2,6,6-tetramethyl-piperidin-1-yloxyl) (10.5 mg, 0.067 mmol) (31.7 mg, 0.203 mmol) and PhMe (4 mL), then DBU (30 µL, 0.20 mmol) was added and the mixture was heated to 100 °C and stirred for 16 h. The reaction was allowed to cool to rt, then filtered through a short silica plug, eluting with hexane then EtOAc. The filtrate was concentrated under reduced pressure and the residue was purified by flash column chromatography (0:100 → 5:95 → 10:90 EtOAc/hexane) to give the title compound as a colourless oil (8 mg, 15%), with spectroscopic data in accordance with literature. [60] **^{1}H NMR** (500 MHz, $CDCl_3$) δ **1.15** (s, 6H, C(2,6)CH_3^ACH$_3^B$), **1.22** (s, 6H, C(2,6)CH$_3^A$CH_3^B), **1.31** – **1.39** (m, 1H, C(4)H^AH^B), **1.45** – **1.52** (m, 4H, C(3,5)H_2), **1.54** – **1.66** (m, 1H, C(4)H^AH^B), **4.46** (dd, J = 6.0, 1.6 Hz, 2H, C(1'')H_2), **6.30** (dt, J = 16.0, 5.9 Hz, 1H, C(2'')H), **6.61** (dt, J = 15.9, 1.7 Hz, 1H, C(3'')H), **7.21** – **7.25** (m, 1H, C(4')H), **7.29** – **7.34** (m, 2H, C(3',5')H), **7.38** – **7.43** (m, 2H, C(2',6')H); **^{13}C{^{1}H} NMR** (126 MHz, $CDCl_3$) δ **17.3**, **20.3**, **33.2**, **39.8**, **59.9**, **78.2**, **125.7**, **126.6**, **127.6**, **128.6**, **131.5**, **137.2**.

(*E*)-2,2,6,6-tetramethyl-1-((3-phenylbut-2-en-1-yl)oxy)piperidine (T-a)

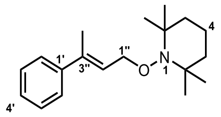

To a vial was added (E)-3-((3-phenylbut-2-en-1-yl)oxy)-1-tritylindolin-2-one **8** (260.3 mg, 0.4990 mmol), *t*Bu-BIMP **7** (74.6 mg, 0.101 mmol), TEMPO (78.3 mg, 0.501 mmol) and mesitylene (10 mL). The mixture was stirred at rt for 16 h, then filtered through a short silica plug, eluting with hexane then EtOAc. The filtrate was concentrated under reduced pressure and the residue was purified by flash column chromatography (0:100 → 5:95 → 10:90 EtOAc/hexane) to give the title compound as a colourless oil (14 mg, 10%), with spectroscopic data in accordance with literature. [61] **^{1}H NMR** (500 MHz, $CDCl_3$) δ **1.12** (s, 6H, C(2,6)CH_3^ACH_3^B), **1.23** (s, 6H, C(2,6)CH_3^ACH_3^B), **1.31 – 1.38** (m, 1H, C(4)$H^A$$H^B$), **1.46 – 1.51** (m, 4H, C(3,5)H_2), **1.53 – 1.64** (m, 1H, C(4)$H^A$$H^B$), **2.06** (d, *J* = 1.2 Hz, 3H, C(3'')CH_3), **4.50** (dd, *J* = 6.5, 1.1 Hz, 2H, C(1'')H_2), **5.94** (ddt, *J* = 6.4, 5.1, 1.4 Hz, 1H, C(2'')*H*),), **7.22 – 7.25** (m, 1H, C(4')*H*), **7.30 – 7.35** (m, 2H, C(3',5')*H*), **7.42 – 7.47** (m, 2H, C(2',6')*H*); **^{13}C{^{1}H} NMR** (126 MHz, $CDCl_3$) δ **16.5**, **17.3**, **20.3**, **33.2**, **39.8**, **59.9**, **75.1**, **124.1**, **125.9**, **127.1**, **128.3**, **136.9**, **143.3**.

8.7 Supplementary Data for interrupted [1,2]-rearrangement products

Interrupted [1,2]-rearrangement of (*E*)-3-((3-(4-fluorophenyl)but-2-en-1-yl)oxy)-1-methylindolin-2-one S13

To a 25 mL round bottomed flask charged with (*E*)-3-((3-(4-fluorophenyl)but-2-en-1-yl)oxy)-1-methylindolin-2-one **S13** (124.5 mg, 0.40 mmol) was added anhydrous PhMe (8.0 mL) and *t*Bu-BIMP **7** (59.0 mg, 0.080 mmol) while under Ar. The reaction was stirred for 0.5 h and then directly submitted to flash column chromatography (CH_2Cl_2:Et_2O 50:0 → 49:1 → 48:2 → 46:4 → 42.5:7.5 → 40:10) to give three fractions: unreacted starting material **S13** as yellow oil (23.7 mg, 0.076 mmol, 19%), [2,3]-Wittig rearrangement product **S14** as yellow oil/semi solid (47.9 mg, 0.154 mmol, 39%) and formal [1,2]-Wittig rearrangement product **S15** as colourless glassy solid (50.3 mg, 0.162 mmol, 40%).

(3*S*)-3-(2-(4-fluorophenyl)but-3-en-2-yl)-3-hydroxy-1-methylindolin-2-one (S14)

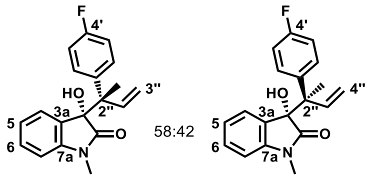

Analysed as (34:24)/(28:14) mixture of rotamers (:) and diastereomers ()/():

$[\alpha]_D^{20}$ = −19 (CH_2Cl_2, *c* 1.650, 58:42 dr, 60:40/71:29 e.r.); **chiral HPLC analysis**: ChiralCel® OJ-H, 94:6 Hexane:IPA flowrate 1 mL·min^{-1}, 211 nm, t_R (3*S*,2"*R*)-**S14** 13.0 min, t_R (3*R*,2"*S*)-**S14** 24.3 min, 71:29 e.r., t_R (3*R*,2"*R*)-**S14** 18.8 min, t_R (3*S*,2"*S*)-**S14** 27.9 min, 40:60 e.r.; **IR** ν_{max} (film): 3395, 3082, 3055, 2984, 2940, 2887 (C-H), 1699 (C=O), 1611, 1508, 1493, 1470 (C=C), 1371, 1350, 1227, 1165, 1109, 1090, 1036 (C-O), 928, 837, 820; **^{1}H NMR** (400 MHz, CD_2Cl_2) δ **1.44** (app s, 1.25H, C(1")H_3, 2 rotamers, minor diastereomer), **1.69** (app s, 1.75H, C(1")H_3, 2 rotamers, major diastereomer), **2.91** (s, 1.00H, NCH_3, major rotamer, major diastereomer), **2.92** (s, 0.70H, NCH_3, minor rotamer, major diastereomer), **2.93** (s, 0.80H, NCH_3, major rotamer, minor diastereomer), **2.94** (0.40H, s, NCH_3, minor rotamer, minor diastereomer), **3.14**$_0$ (app s, 0.42H, O*H*, 2 rotamers, minor diastereomer), 3.14$_4$ (app s, 0.58H, O*H*, 2 rotamers, major diastereomer), **4.96** (app dd, *J* = 17.5, 1.3 Hz, 0.42H, C(4")$H_{cis}H_{trans}$, 2 rotamers, minor diastereomer), **5.27** (dd, *J* = 10.9, 1.0 Hz, 0.14H, C(4")$H_{cis}H_{trans}$, minor rotamer, minor diasteromer), **5.28** (dd, *J* = 10.9, 1.2 Hz, 0.28H, C(4")$H_{cis}H_{trans}$, major rotamer, minor diastereomer), **5.30** (app dd, *J* = 17.5, 1.2 Hz, 0.58H, C(4")$H_{cis}H_{trans}$, 2 rotamers, major diastereomer), **5.35** (app dd, *J* = 11.0, 1.2 Hz, 0.58H, C(4")$H_{cis}H_{trans}$, 2 rotamers, major diastereomer), **6.41** – **6.57** (m, 1.00H, C(3") 2 rotamers, major diastereomer, ArC(4)*H* 2 rotamers, minor diastereomer), **6.64** – **6.69** (m, 1.00H, ArC(7)*H*, 2 rotamers, 2 diastereomers), **6.74** (app d (br), *J* = 7.6 Hz, 0.58H, ArC(4)*H*, 2 rotamers, major diastereomer), **6.83** – **6.94** (m, 3.00H, ArC(5)*H*, ArC(3',5')*H*, 2 rotamers, 2 diastereomers), **6.99** (dd, *J* = 17.5, 10.9 Hz, 0.14H, C(3")*H*, minor rotamer, minor diastereomer), **7.00** (dd, *J* = 17.5 Hz, 10.9 Hz, 0.28H, C(3")*H*, major rotamer, minor diastereomer), **7.17** – **7.23** (m, 0.84H, ArC(2',6')*H*, 2 rotamers,

minor diasteromer), **7.23** – **7.29** (m, 2.16H, ArC(6)*H* (2 rotamers, 2 diastereomers), ArC(2',6')*H* (2 rotamers, major diastereomer)); **^{13}C{^{1}H} NMR** (101 MHz, CD_2Cl_2) δ **18.7** (*C*(1'')H$_3$, major diastereomer), **20.7** (*C*(1'')H$_3$, minor diastereomer), **26.1** (N*C*H$_3$, major diastereomer), **26.2** (N*C*H$_3$, minor diastereomer), **50.6** (*C*(2''), major diastereomer), **51.5** (*C*(2''), minor diastereomer), **80.9** (*C*(3)OH, major diastereomer), **81.0** (*C*(3)OH, minor diastereomer), **108.3** (Ar*C*(7)H, minor diastereomer), **108.4** (Ar*C*(7)H, major diastereomer), **114.2** (d, *J* = 20.8 Hz, Ar*C*(3',5')H, minor diastereomer), **114.5** (d, *J* = 20.7 Hz, Ar*C*(3',5')H, major diastereomer), **116.6** (*C*(4'')H$_2$, minor diastereomer), **117.5** (*C*(4'')H$_2$, major diastereomer), **122.5**$_0$ (Ar*C*(5)H, major diastereomer), **122.5**$_1$ (Ar*C*(5)H, minor diastereomer), **125.9** (Ar*C*(4)H, major diastereomer), **126.1** (Ar*C*(4)H, minor diastereomer), **128.9** (Ar*C*(3a), major diastereomer), **129.1** (Ar*C*(3a), minor diastereomer), **130.1**$_7$ (d, *J* = 8.8 Hz, Ar*C*(3',5')H, major diastereomer), **130.2**$_1$ (Ar*C*(6)H, 2 diastereomers), **131.4** (d, *J* = 7.9 Hz, Ar*C*(3',5')H, minor diastereomer), **137.2** (d, *J* = 3.3 Hz, Ar*C*(1'), minor diastereomer), **139.2** (d, *J* = 2.7 Hz, Ar*C*(1'), major diastereomer), **139.9** (*C*(3'')H, major diastereomer), **141.6** (*C*(3'')H, minor diastereomer), **144.6** (Ar*C*(7a), 2 diastereomers), **162.2** (d, *J* = **245.1** Hz, Ar*C*(4')F, major diastereomer), **162.3** (d, *J* = 245.3 Hz, Ar*C*(4')F, minor diastereomer), **177.4** (*C*(2), minor diastereomer), **177.5** (*C*(2), major diastereomer); **^{19}F{^{1}H} NMR** (377 MHz, $CDCl_3$) δ **−117.04** (s, 0.58F, ArC(4')*F*), **−116.94** (s, 0.42F, Ar(C4')*F*); **HRMS** (ESI$^+$) $C_{19}H_{18}O_2NFNa$ [M+Na]$^+$ found 334.1208, requires 334.1214 (−1.9 ppm).

(*S*,*E*)-3-(3-(4-fluorophenyl)but-2-en-1-yl)-3-hydroxy-1-methylindolin-2-one (S15)

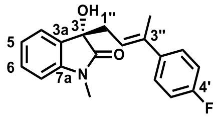

The [1,2]-Wittig product was analysed as 67:33 mixture of rotamers or dimer/monomer:

$[\alpha]_D^{20}$ (CH_2Cl_2, *c* 1.085) −14 (68:32 e.r.); **HPLC analysis:** ChiralCel® OD-H, 95:5 Hexane:IPA, flowrate 1 mL·min^{-1}, 254 nm, t_R (*R*)-**S15** 15.3 min, t_R (*S*)-**S15** 17.5 min, 35:65 e.r.; **IR** ν_{max} (film): 3366, 3055, 2936, 2918, 2887 (C-H), 1701 (C=O), 1614, 1508, 1495, 1470 (C=C), 1375, 1348, 1223, 1159, 1086, 1011 (C-O), 753; **^{1}H NMR** (400 MHz, $CDCl_3$) δ **1.89**$_7$ (q, *J* = 1.4 Hz, 0.67H, C(4”)H_3), **1.89**$_9$ (q, *J* = 1.3 Hz, 0.33H, C(4”)H_3), **2.83** (app dd, *J* = 14.5, 8.4 Hz, 1H, C(1”)H^AH^B), **2.87** (app dd, *J* = 14.5, 7.1 Hz, 1H, C(1”)H^AH^B), **3.16**$_9$ (s, 2H, NCH_3), **3.17**$_1$ (s, 1H, NCH_3), **3.44** (s, 0.67H, O*H*), **3.58** (s, 0.33H, O*H*), **5.54**$_7$ (ddq, *J* = 8.4, 7.2, 1.3 Hz, 0.33H, C(2”)), **5.55**$_3$ (ddq, *J* = 8.4, 7.1, 1.4 Hz, 0.67H, C(2”)*H*), **6.83** (app d, *J* = 7.8 Hz, 1H, ArC(7)*H*), **6.89** – **6.97** (m, 2H, ArC(3’,5’)*H*), **7.09** (app td, *J* = 7.6, 0.9 Hz, 1H, ArC(5)*H*), **7.14** – **7.21** (m, 2H, ArC(2’,6’)*H*), **7.33** (app td, *J* = 7.7, 1.3 Hz, 1H, ArC(6)*H*), **7.40** – **7.45** (m, 1H, ArC(4)*H*); **^{13}C{^{1}H} NMR** (101 MHz, $CDCl_3$) δ **16.5** (*C*(4”)H_3), **26.4** (N*C*H_3), **38.1** (*C*(1”)H_2), **76.5**$_0$ (*C*(3)OH, major), **76.5**$_4$ (*C*(3)OH, minor), **108.5** (Ar*C*(7)H), **115.0** (d, *J* = 21.2 Hz, Ar*C*(3’,5’)H), **119.7** (*C*(2”)H), **123.3** (Ar*C*(5)H), **124.1** (Ar*C*(4)H), **127.4** (d, *J* = 7.8 Hz, Ar*C*(2’,6’)H), **129.8** (Ar*C*(6)H), **130.0**$_3$ (Ar*C*(3a), major), **130.0**$_5$ (Ar*C*(3a), minor), **138.5**$_6$ (*C*(3”), minor), **138.5**$_7$ (*C*(3”), major), **139.7** (d, *J* = 3.3 Hz, Ar*C*(1’)), **143.3** (Ar*C*(7a)), **162.1** (d, *J* = 245.7 Hz, Ar*C*(4’)F), **178.2** (*C*(2), major), **178.3** (*C*(2), minor); **^{19}F{^{1}H} NMR** (377 MHz, $CDCl_3$) δ **−115.98** (s, 0.33F, ArC(4’)*F*), **−115.97** (s, 0.67F, ArC(4’)*F*); **HRMS** (ESI$^+$) $C_{19}H_{18}O_2NFNa$ [M+Na]$^+$ found 334.1209, requires 334.1214 (−1.6 ppm).

Interrupted [1,2]-rearrangement of (*E*)-1-benzyl-3-((3-(4-fluorophenyl)but-2-en-1-yl)oxy)indolin-2-one S16

To a 25 mL round bottomed flask charged with (*E*)-1-benzyl-3-((3-(4-fluorophenyl)but-2-en-1-yl)oxy)indolin-2-one **S16** (155.0 mg, 0.40 mmol) was added anhydrous PhMe (8.0 mL) and *t*Bu-BIMP **7** (59.0 mg, 0.080 mmol) while under Ar. The reaction was stirred for 0.5 h and then directly submitted to flash column chromatography (Hexanes:EtOAc 49:1 → 47.5:2.5 → 90:10 → 40:10 → 30:20) to give three fractions: unreacted starting material **S16** as yellow oil (23.7 mg, 0.076 mmol, 19%), [2,3]-Wittig rearrangement product **S17** as yellow oil (47.9 mg, 0.154 mmol, 39%) and formal [1,2]-Wittig rearrangement product **S18** as white solid (50.3 mg, 0.162 mmol, 40%).

(3*S*)-1-benzyl-3-(2-(4-fluorophenyl)but-3-en-2-yl)-3-hydroxyindolin-2-one (S17)

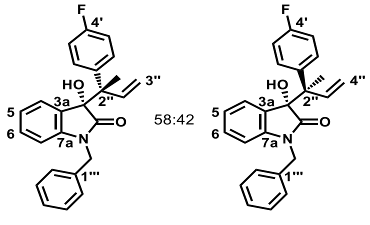

The [2,3]-Wittig product was analysed as (47:11)/(37:5) mixture of rotamers (:) and diastereomers ()/():

$[\alpha]_D^{20}$ = −28 (CH_2Cl_2, *c* 2.855, 58:42 dr, 79:21/79:21 e.r.); **HPLC analysis:** ChiralCel® AD-H, 95:5 Hexane:IPA, flowrate 1 mL·min^{-1}, 211 nm, t_R (3*R*,2”*R*)-**S17** 16.6 min, t_R (3*S*,2”*S*)- **S17** 24.7 min, 21:79 e.r., t_R (3*R*,2”*S*)- **S17** 18.4 min, t_R (3*S*,2”*R*)-**S17** 21.6 min, 21:79 e.r.; **IR** ν_{max} (film): 3412, 3084, 3061, 3032, 3007, 2984, 2943, 2922 (C-H), 1699 (C=O), 1611, 1508, 1497, 1487, 1466, 1456, 1435, 1412 (C=C), 1364, 1350, 1227, 1165, 1115, 1078, 1030, 1013 (C-O), 928, 835, 819; **^{1}H NMR** (500 MHz, CD_2Cl_2) δ **1.58** (s, 1.74H, C(1”)*H*$_3$ 2 rotamers, major diastereomer), **1.76** (s, 1.26H, C(1”)*H*$_3$ 2 rotamers, minor diastereomer), **3.24**$_0$ (s, 0.47H, O*H*, major rotamer, major diastereomer), **3.24**$_3$ (s, 0.37H, O*H*, major rotamer, minor diastereomer), **3.27** (app s, 0.16H, O*H*, minor rotamers, 2 diastereomers), **4.42** (d, *J* = 15.5, 0.37H, NC*H*^{A}H^B, major rotamer, minor diastereomer), **4.44** (d, *J* = 15.7 Hz, 0.05H, NC*H*^{A}H^B, minor rotamer, minor diastereomer), **4.45** (0.47H, d, *J* = 15.6 Hz, NC*H*^{A}H^B, major rotamer, major diastereomer), **4.46** (0.11H, d, *J* = 15.7 Hz, NC*H*^{A}H^B, minor rotamer, major diastereomer), **4.83**$_9$ (d, *J* = 15.6 Hz, 0.47H, NCH^A*H*^{B}, major rotamer, major diastereomer), **4.84**$_4$ (d, *J* = 15.7 Hz, 0.05H, NCH^A*H*^{B}, minor rotamer, minor diastereomer), **4.86** (d, *J* = 15.7 Hz, 0.11H, NCH^A*H*^{A}, minor rotamer, major diastereomer), **4.86** (d, *J* = 15.5 Hz, 0.37H, NCH^A*H*^{A}, major rotamer, minor diastereomer), **4.94** (app d, *J* = 17.5 Hz, 0.58H, C(4”)H$_{cis}$*H*$_{trans}$, 2 rotamers, major diastereomer), **5.29** (app d, $^3J_{HH}$ = 11.5 Hz, 0.58H, C(4”)*H*$_{cis}$H$_{trans}$, 2 rotamers, major diastereomer), **5.39** (app d, $^3J_{HH}$ = 18.0 Hz, 0.42H, C(4”)H$_{cis}$*H*$_{trans}$, 2 rotamers, minor diastereomers), **5.40** (app d, *J* = 11.1 Hz, 0.42H, C(4”)*H*$_{cis}$H$_{trans}$, 2 rotamers, minor diastereomer), **6.53** – **6.64** (m, 0.42H, C(3”)*H*, 2 rotamers, minor diastereomer), **6.55**

(app d, J = 7.8 Hz, 0.58H, ArC(7)*H*, 2 rotamers, major diastereomer), **6.58** (app d, J = 8.0 Hz, 0.42H, ArC(7)*H*, 2 rotamers, minor diastereomer), **6.66** – **6.74** (m, 0.58H, ArC(4)*H*, 2 rotamers, major diastereomer), **6.74** – **6.84** (m, 0.42H, ArC(4)*H*, 2 rotamers, minor diastereomer), **6.84** – **6.93** (m, 3H, ArC(5)*H*, ArC(3',5')*H*, 2 rotamers, 2 diastereomers), **6.94** – **7.01** (m, 1.16H, ArC(2''',6''')*H*, 2 rotamers, major diastereomer), **7.01** – **7.08** (m, 1.42H, *C*(3'')H 2 rotamers, major diastereomer, ArC(2''',6''')*H* 2 rotamers, minor diastereomer), **7.13** – **7.21** (m, 2.16H, ArC(6)*H* 2 rotamers, 2 diastereomers, ArC(2',6')*H* 2 rotamers, major diastereomer), **7.21** – **7.31** (m, 3.84H, ArC(3''',4''',5''')*H* 2 rotamers, 2 diastereomers, ArC(2',6')*H* 2 rotamers, minor diastereomer); **^{13}C{^{1}H} NMR** (125 MHz, CD_2Cl_2) δ **19.1** (*C*(1'')H_3, minor), **20.9** (*C*(1'')H_3, major), **44.3$_0$** (N*C*H_2, major), 44.3$_2$ (N*C*H_2, minor), **50.7** (*C*(2''), minor), **51.7** (*C*(2''), major), **80.5$_6$** (*C*(3)OH, minor), **80.5$_9$** (*C*(3)OH, major), **109.4** (Ar*C*(7)H, major), **109.5** (Ar*C*(7)H, minor), **114.5** (d, J = 20.8 Hz, Ar*C*(3',5')H, major), **114.8** (d, J = 20.8 Hz, Ar*C*(3',5')H, minor), **116.5** (*C*(4'')H_2, major), **118.2** (*C*(4'')H_2, minor), **122.7** (Ar*C*(5)H, minor), **122.8** (Ar*C*(5)H, major), **126.2** (Ar*C*(4')H, major), **126.4** (Ar*C*(4')H, minor), **127.6** (Ar*C*(2''',6''')H, major), **127.8** (Ar*C*(2''',6''')H, minor), **128.0** (Ar*C*(4''')H, 2 diastereomers), **129.0** (Ar*C*(3a), minor), **129.1** (Ar*C*(3''',5''')H, minor), **129.2** (Ar*C*(3''',5''')H, major), **129.4** (Ar*C*$^{(3a)}$, major), **130.2** (Ar*C*(6)H, 2 diastereomers), **130.3** (d, J = 8.8 Hz, Ar*C*(2',6')H, minor), **131.6** (d, J = 7.8 Hz, Ar*C*(2',6')H, major), **136.1** (Ar*C*(1'''), major), **136.2** (Ar*C*(1'''), minor), **137.0** (d, J = 3.3 Hz, Ar*C*$^{(1')}$, minor), **139.4** (Ar*C*(1'), major), **139.8** (*C*(3'')H, minor), **142.0** (*C*(3'')H, major), **143.9** (Ar*C*(7a), major), **144.1** (Ar*C*(7a), minor), **162.3** (d, J = 245.3 Hz, Ar*C*(4')F, minor), **162.4** (d, J = 245.5 Hz, Ar*C*(4')F, major), **177.6** (*C*(2), major), **177.7** (*C*(2), minor); **^{19}F{^{1}H} NMR** (470 MHz, CD_2Cl_2) δ **−116.52** (s, 0.58F, ArC(4')*F*), **−116.67** (s, 0.42F, ArC(4')*F*); **HRMS** (ESI^{+}) $C_{25}H_{22}O_2NFNa$ [M+Na]$^{+}$ found 410.1520, requires 410.1527 (−1.6 ppm).

(*S*,*E*)-1-benzyl-3-(3-(4-fluorophenyl)but-2-en-1-yl)-3-hydroxyindolin-2-one (S18)

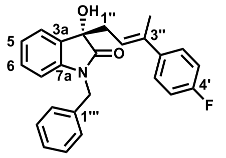

The [1,2]-Wittig product was analysed as 81:19 mixture of rotamers or dimer/monomer:

mp 114 °C (CH_2Cl_2/Et_2O); $[\alpha]_D^{20} = 3$ (CH_2Cl_2, *c* 0.570, 81:19 e.r.); **Chiral HPLC analysis**: ChiralCel® AS-H, 95:5 Hexane:IPA, flowrate 1 mL·min^{-1}, 254 nm, t_R (*R*)-**S18** 15.9 min, t_R (*S*)-**S18** 21.2 min, 19:81 e.r.; **IR** ν_{max} (film): 3385, 3055, 3032, 2914 (C-H), 1701 (C=O), 1614, 1601, 1508, 1489, 1468 (C=C), 1369, 1356, 1224, 1071 (C-O), 831, 752; **^{1}H NMR** (500 MHz, CD_2Cl_2) δ **1.94** (app s, 3H, C(4”)H_3), **2.92** (app ddq, *J* = 13.8, 7.0, 1.3 Hz, 1H, C(1”)H^AH^B), **2.99** (app dd, *J* = 13.8, 8.5 Hz, 1H, C(1”)H^AH^B), **3.10** (s, 0.81H, O*H*), **3.22** (s, 0.19H, O*H*), **4.62$_6$** (d, *J* = 15.8 Hz, 0.81H, NCH^AH^B), **4.63$_0$** (d, *J* = 15.8 Hz, 0.19H, NCH^AH^B), **5.06$_6$** (d, *J* = 15.8 Hz, 0.81H, NCH^AH^B), **5.07$_1$** (d, *J* = 15.8 Hz, 0.19H, NCH^AH^B), **5.45** (app ddq, *J* = 8.5, 7.0, 1.3 Hz, 1H, C(2”)*H*), **6.71** (app d, *J* = 7.8 Hz, 1H, ArC(7)*H*), **6.90** – **6.97** (m, 2H, ArC(3’,5’)*H*), **7.08** (app td, *J* = 7.6, 1.0 Hz, 1H, ArC(5)*H*), **7.09** – **7.16** (m, 4H, ArC(2’,6’)*H*, ArC(3’’’,5’’’)*H*), **7.16** – **7.22** (m, 3H, ArC(2’’’,4’’’,6’’’)*H*), **7.23** (app td, *J* = 7.8, 1.3, 0.5 Hz, 1H, ArC(6)*H*); **^{13}C{^{1}H} NMR** (125 MHz, CD_2Cl_2) δ **16.6** (*C*(4”)H_3), 38.6 (*C*(1”)H_2), **44.3** (N*C*H_2), **76.8$_5$** (*C*(3)OH, major), **76.8$_7$** (*C*(3)OH, minor), **109.9** (Ar*C*(7)H), **115.3** (d, *J* = 21.3 Hz, Ar*C*(3’,5’)H), **120.1** (*C*(2”)H), **123.6** (Ar*C*(5)H), **124.5** (Ar*C*(4)H), **127.6** (Ar*C*(2’’’,6’’’)H), **127.9** (d, *J* = 7.8 Hz, Ar*C*(2’,6’)H), **128.1** (Ar*C*(4’’’)H), **129.3** (Ar*C*(3’’’,5’’’)H), **130.2** (Ar$C^{(6)}$H), **130.4** (Ar*C*(3a)), **136.2** (Ar*C*(1’’’)), **138.9** (*C*(3”)H), **140.0** (d, *J* = 3.2 Hz, Ar*C*(1’)), **143.2** (Ar*C*(7a)), **162.5** (d, *J* = 145.1 Hz, Ar*C*(4’)F), **178.3$_5$** (*C*(2), major), **178.4$_3$** (*C*(2), minor); **^{19}F{^{1}H} NMR** (470 MHz, CD_2Cl_2) δ **−116.75$_3$** (s, 0.81F, ArC(4’)*F*), **−116.74$_7$** (s, 0.19F, ArC(4’)*F*); **HRMS** (ESI$^+$) $C_{25}H_{22}O_2NFNa$ [M+Na]$^+$ found 410.1521, requires 410.1527 (−1.5 ppm).

Interrupted [1,2]-rearrangement of ethyl 4-(1-trityloxindol-3-oxy)tiglate 66

To a 5 mL round bottomed flask was added ethyl 4-(1-trityloxindol-3-oxy)tiglate **66** (103.5 mg, 0.200 mmol), *t*Bu-BIMP **7** (29.5 mg, 0.040 mmol) and mesitylene (4.0 mL, 0.05 M). The mixture was stirred for 5.5 h and then directly submitted to flash column chromatography (Hexane:EtOAc 49:1 → 47.5:2.5 → 45:5 → 42.5:7.5 → 40:10 → 35:15 → 0:50) to give four fractions: [2,3]-Wittig product **S19** as yellow oil (9.2 mg, 0.018 mmol, 9%), *N*-trityl isatin **S9** as yellow solid (7.3 mg, 0.019 mmol, 9%), Aldol product **68** as white solid (27.8 mg, 0.031 mmol, 15%), and [1,2]-Wittig product **67** as white solid (57.0 mg, 0.110 mmol, 57%).

Ethyl (3*R*,2”*S*)-2-(3-hydroxy-2-oxo-1-tritylindolin-3-yl)-2-methylbut-3-enoate (S19)

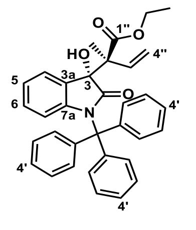

$[\alpha]_D^{20} =$ 0.8 (*c* 0.6, CH_2Cl_2); **Chiral HPLC analysis**: ChiralPak® IC, 95:5 Hexane:IPA, flowrate 1 mL·min^{-1}, 254 nm, t_R (3S,2”*R*)-**S19** 11.0 min, t_R (3*R*,2”*S*)- **S19** 18.2 min, 11:89 e.r.; **IR** ν_{max} (film) 3485, 3374, 3088, 3057, 3034, 2988, 2928 (C-H), 1730 (C=O), 1697, 1607, 1491, 1464, 1449 (C=C), 1331, 1310, 1273, 1234, 1186, 1105 (C-O), 745, 704; **^{1}H NMR** (700 MHz, CD_2Cl_2) δ **1.04** (s, 3H, C(2”)C*H*$_3$), **1.23** (t, *J* = 7.2 Hz, 3H, OCH$_2$C*H*$_3$), **4.14** (dq, *J* = 10.5, 7.2 Hz, 1H, OC*H*^{A}H^{B}CH$_3$), **4.19** (dq, *J* = 10.5, 7.2 Hz, 1H, OCH^A*H*^{B}CH$_3$), **5.19** (d, *J* = 17.5 Hz, 1H, C(4”)*H*^{A}H^B), **5.33** – **5.35** (m, 1H, O*H*), 5.43 (d, *J* = 10.8 Hz, 1H, C(4)H^A*H*^{B}), **6.31** (d, *J* = 8.1 Hz, 1H, ArC(7)*H*), **6.50** (dd, *J* = 17.5,10.8 Hz, 1H, C(3”)*H*), **6.88** (app t, *J* = 7.6 Hz, 1H, ArC(5)*H*), **6.93** (ddd, *J* = 8.1, 7.6, 1.4 Hz, 1H, ArC(6)*H*), **7.1** (app t, *J* = 7.4 Hz, 3H, (ArC(4’)*H*)$_3$), **7.26** (app t, *J* = 7.6 Hz, 6H, (C(3’,5’)*H*)$_3$), **7.28** (d, *J* = 7.6 Hz, 1H, ArC(4)*H*), **7.44** (app d, *J* = 7.8 Hz, 6H, (PhC(2’,6’)*H*)$_3$); **^{1}H{^{13}C} NMR** (176 MHz, CD_2Cl_2) δ **14.3** (OCH$_2$*C*H$_3$), **15.5** (C(2”)*C*H$_3$), **53.6** (*C*(2”)), **62.4** (O*C*H$_2$CH$_3$), **75.3** (*C*Ph$_3$), **79.2** (*C*(3)), **116.4** (Ar*C*(7)H), **118.4** (*C*(4”)H$_2$), **122.6** (Ar*C*(5)H), **125.3** (Ar*C*(4)H), **127.4** ((Ph*C*(4’)H)$_3$), **128.2** ((Ph*C*(3’,5’)H)$_3$), **128.5** (Ar*C*(3a)), **128.8** (Ar*C*(6)H), **129.6** (C(Ph*C*(2’,6’)H)$_3$), **136.6** (*C*(3”)H), **142.7** ((Ph*C*(1’))$_3$), **144.2** (Ar*C*(7a)), **174.7** (*C*(1”)), **178.4** (*C*(2)); **HRMS** (ESI$^+$) $C_{34}H_{31}O_4NNa$ [M+Na]$^+$ found 540.2145, requires 540.2145 (−0.0 ppm).

Ethyl (*E*)-4-((3-hydroxy-2,2'-dioxo-1,1'-ditrityl-[3,3'-biindolin]-3'-yl)oxy)tiglate (68)

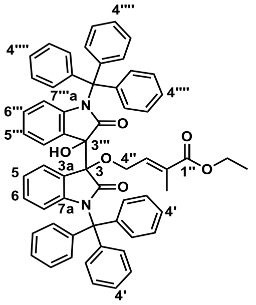

mp 114 – 116 °C (hexanes:EtOAc); $[\alpha]_D^{20} = -4.8$ (*c* 1.4, CH_2Cl_2); **Chiral HPLC analysis**: ChiralCel® OD-H, 95:5 Hexane:IPA, flowrate 1 mL·min^{-1}, 211 nm, t_R major 7.7 min, t_R minor 16.7 min, 75:25 e.r.; **IR** ν_{max} (film) 3441, 3055, 3022, 2980, 1728, 1717 (C=O), 1603, 1489, 1462, 1449 (C=C), 1329, 1308, 1269, 1234, 1190, 1113, 1034 (C-O), 743, 704, 629; **^{1}H NMR** (400 MHz, CD_2Cl_2) δ **1.24** (t, *J* = 7.1 Hz, 3H, CH_2CH_3), **1.38** (app dt, *J* = 1.5, 1.0 Hz, 3H, C(2'')CH_3), **2.81** (br dd, *J* = 12.8, 6.2 Hz, C(4'')H^AH^B), **3.03** (1H, br dd, *J* = 12.8, 5.8 Hz, 1H, C(4'')H^AH^B), **4.85** (br s, 1H, O*H*), **6.25** – **6.35** (m, 1H, Ar*C*(7)H), **6.50** (ddq, *J* = 6.2, 5.8, 1.5 Hz, 1H, C(3'')*H*), **6.52** (d, *J* = 8.3 Hz, 1H, ArC(7''')*H*), **6.93** (app t, *J* = 7.5 Hz, 1H, ArC(5''')*H*), **6.98** – **7.06** (m, 2H, ArC(5,6)*H*), **7.14** (ddd, *J* = 8.3, 7.5, 1.5 Hz, 1H, ArC(6''')*H*), **7.14** – **7.35** (m, 26H, ArC(4)*H*, ArC(4''')*H*, (ArC(3',4',5')*H*)$_3$, (ArC(2'''',3'''',4'''',5'''',6'''')*H*)$_3$), 7.56 – **7.63** (m, 6H, C(PhC(2',6')*H*)$_3$); **^{13}C{^{1}H} NMR** (101 MHz, CD_2Cl_2) δ **13.2** (C(2'')*C*H$_3$), **14.6** (CH$_2$*C*H$_3$), **61.2** (*C*H$_2$CH$_3$), **61.7** *C*(4'')H$_2$), **75.2** (*C*'''Ph$_3$), **76.8** (*C*Ph$_3$), **79.4** (br, Ar*C*(3), Ar*C*(3''')), **116.3** (Ar*C*(7)H), **116.7** (Ar*C*(7''')H), **123.0** (Ar*C*(5)H), **123.4** (Ar*C*(5''')H), **124.5** (Ar*C*(4''')H), **126.2** (Ar*C*(4)H), **127.2** (C(Ph*C*(4)H)$_3$), **127.3** (Ar*C*(3a)), **127.4** (C(Ph*C*(4''')H)$_3$), **128.3** ((Ar*C*(3',5')H)$_3$), **128.4** ((Ar*C*(3''',5''')H)$_3$), **129.3**$_9$ (Ar*C*(6)H), **129.4**$_5$ ((Ar*C*(2''',6''')H)$_3$), **129.5**$_0$ ((Ar*C*(2',6')H)$_3$), **130.3** (*C*(2'')), **130.6** (Ar*C*(3a'''), Ar*C*(6''')H), **136.5** (*C*(3'')H), **142.6** ((Ar*C*(1''''))$_3$), **142.9** ((Ar*C*(1'))$_3$), **144.7** (Ar*C*(7a)), **145.8** (Ar*C*(7a''')), **167.6** (*C*(1'')), **176.9** (*C*(2)), **177.2** (*C*(2''')); **HRMS** (ESI$^+$) $C_{61}H_{50}O_6N_2Na$ [M+Na]$^+$ found 929.3557, requires 929.3561 (−0.4 ppm).

Ethyl (*S*,*E*)-4-(3-hydroxy-2-oxo-1-tritylindolin-3-yl)tiglate (67)

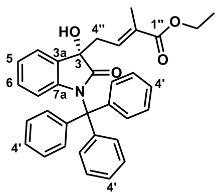

mp 70 °C (PhH); $[\alpha]_D^{20} =$ 56.9 (*c* 2.5, CH_2Cl_2), **Chiral HPLC analysis**: ChiralCel® OD-H, 95:5 Hexane:IPA, flowrate 1 mL·min^{-1}, 211 nm, t_R (*R*)-**67** 10.0 min, t_R (*S*)-**67** 14.4 min, 2:98 e.r.; **IR** ν_{max} (film): 3418 (br, O-H), 3057, 2980, 2928 (C-H), 1732, 1709 (C=O), 1611, 1491, 1479, 1466, 1449, 1331, 1319, 1261, 1211, 1186, 1111, 1088 (C-O), 746, 735, 704, 650, 633; **^{1}H NMR** (400 MHz, CD_2Cl_2) δ **1.22** (t, *J* = 7.1 Hz, 3H, OCH_2CH_3), **1.83** (app dt, *J* = 1.5, 1.0 Hz, 3H, C(2")CH_3), **2.89** – **2.96** (m, 3H, C(4")H_2, O*H*), **4.15** (dq, *J* = 10.8 , 7.1 Hz, 1H, OCH^AH^BCH$_3$), **4.19** (dq, *J* = 10.8, 7.1 Hz, 1H, OCH^AH^BCH$_3$), **6.36** (app dd, *J* = 7.9, 1.2 Hz, 1H, ArC(7)*H*), **6.68** (app tq, *J* = 7.8, 1.5 Hz, 1H, C(3")*H*), **6.96** (ddd, *J* = 7.9, 7.5, 1.7 Hz, 1H, ArC(6)*H*), **7.01** (ddd, *J* = 7.5, 7.1, 1.2 Hz, 1H, ArC(5)*H*), **7.04** (app t, *J* = 7.3 Hz, 3H, (ArC(4')*H*)$_3$), **7.18** – **7.31** (m, 9H, (ArC(3',4',5')*H*)$_3$), **7.38** (app dd, *J* = 7.1, 1.7 Hz, 1H, ArC(4)*H*), **7.40** – **7.47** (m, 6H, (ArC(2',6')*H*)$_3$); **^{13}C{^{1}H} NMR** (101 MHz, CD_2Cl_2) δ **13.2** (C(2")*C*H$_3$), **14.6** (OCH$_2$*C*H$_3$), **39.4** (*C*(4")H$_2$), **61.3** (O*C*H$_2$CH$_3$), **75.1** (*C*Ph$_3$), 76.1 (*C*(3)OH), **116.7** (Ar*C*(7)H), **123.3** (Ar*C*(5)H), **123.9** (Ar*C*(4)H), **127.4** (C(Ar*C*(4')H)$_3$), **128.3** (C(Ar*C*(3',5')H)$_3$), **128.8** (Ar*C*(6)H), **129.7** (C(*C*(2',6')H)$_3$), **130.4** (Ar*C*(3a)), **132.6** (*C*(2")), **133.8** (*C*(3")), **142.5** ((Ar*C*(1'))$_3$), **143.3** (Ar*C*(7a)), **167.9** (*C*(1")), **180.2** (*C*(2)); **HRMS** (ESI$^+$) $C_{34}H_{31}O_4NNa$ $[M+Na]^+$ found 540.2142, requires 540.2145 (−0.6 ppm).

8.8 Supplementary Data for determination of absolute configuration of [2,3]- and formal [1,2]-rearrangement product

(*S*)-3-Hydroxy-3-(2-hydroxyethyl)indolin-2-one (S128)

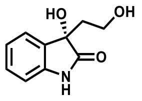

Following **General Procedure K**, formal [1,2]-rearrangement product **9** (156.3 mg, 0.3 mmol) was treated with O_3, $NaBH_4$ and Et_3SiH/TFA to give a crude product which was purified by flash column chromatography (eluent: hexane/ EtOAc = 3:1 to 1:1) to afford the product (16.2 mg, 28%) as a colourless amorphous solid. $[\mathbf{a}]_{\mathbf{D}}^{\mathbf{20}}$ –31.5 (*c* 0.39, $CHCl_3$); **Chiral HPLC analysis**, Chiralpak ADH (80:20 hexane:IPA, flow rate 1ml·min^{-1}, 211 nm, 30 °C) t_R (*S*)-**9**: 7.1min, t_R (*R*)-**9**: 9.2min, 96:4 e.r.; ^{1}**H NMR** (500 MHz, Acetone-d_6) δ **2.97** (2H, s), **3.60–3.77** (2H, m), **3.78–3.93** (1H, m), **5.15** (1H, s), **6.89** (1H, d, *J* 7.8), **7.01** (1H, td, *J* 7.5, 1.0), **7.22** (1H, td, *J* 7.7, 1.3), **7.35** (1H, dd, *J* 7.4, 1.2), **9.30** (1H, s); ^{13}**C{^{1}H} NMR** (126 MHz, Acetone-d_6) δ **41.1**, **58.3**, **76.1**, **110.6**, **122.8**, **124.9**, **129.9**, **133.0**, **142.4**, **180.1**.

3-Hydroxy-3-(2-phenylbut-3-en-2-yl)-1-tritylindolin-2-one (14)

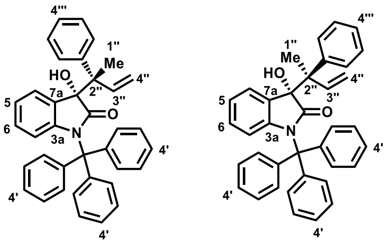

major diastereomer *minor diastereomer*

Following **General Procedure J**, allylic ether **8** (521 mg, 1.0 mmol) and *t*Bu-BIMP (147 mg, 0.2 mmol) in mesitylene (20 mL) gave a crude product which was purified by flash column chromatography (eluent: hexane/ acetone = 8:1 to 4:1) to afford the product as 89:11 mixture of diastereomers (109.4 mg, 21%) as a light yellow amorphous solid. **IR** ν_{max} (film) 3394 (OH), 1715 (C=O), 1610 (C=C), 1598 (C=C), 1463, 1448, 1242, 1043; $[\mathbf{a}]_{\mathbf{D}}^{\mathbf{20}}$ +85.6 (*c* 0.25, $CHCl_3$); **Chiral HPLC analysis,** Chiralpak IB (98:2 hexane:IPA, flow rate 1 ml·min^{-1}, 211 nm, 30 °C) major diastereomer: t_R (2``*R*,3*R*)-**14**: 8.5min, $t_R$ (2``*S*,3*S*)-**14**: 10.6min, 1:99 e.r.; minor diastereomer: t_R (2``*S*,3*R*)-**14**: 9.5min, $t_R$ (2``*R*,3*S*)-**14**: 11.6min, 1:99 e.r.; ^{1}**H NMR** (400 MHz, $CDCl_3$) δ **1.76** (2.53H, s, C*H*$_3$ (major)), **1.79** (0.45H, s, C*H*$_3$ (minor)), **2.87** (0.87H, s, O*H* (major)), **2.94** (0.11H, s, O*H* (minor)), **4.99** (0.88H, d, *J* 17.5, C(4``)$H^A$H$^B$ (major)), **5.29** (0.88H, d, *J* 11.2, C(4``)H^AH^B (major)), **5.50** (0.11H, d, *J* 17.2, C(4``)$H^A$H$^B$ (minor)), **5.56** (0.11H, d, *J* 11.2, C(4``)H^AH^B (minor)), **6.10–6.28** (1H, m, ArC(7)*H* (major and minor)), **6.78–7.02** (3.33H, m, ArC*H* (major and minor) and ArC(3``)*H* (major and minor)), **7.04–7.26** (18.29H, m, ArC*H* (major and minor)), **7.29–7.38** (3.31H, m, ArC*H* (major and minor)); $^{13}$**C{**$^{1}$**H} NMR** (101 MHz, $CDCl_3$) δ **19.2** (*C*H$_3$ (minor)), **20.1** (*C*H$_3$ (major)), **50.9** (*C*(2``) (minor)), **52.4** (*C*(2``) (major)), **74.9** (*N*CPh$_3$ (major and minor)), **79.3** (*C*(3)OH (major and minor)), **116.1** (Ar*C*(7)H (major)), **116.1** (Ar*C*(7)H (minor)), **116.1** (*C*(4``)H$_2$ (major)), **117.9** (*C*(4``)H$_2$ (minor)), **122.0** (Ar*C*(5)H (minor)), **122.1** (Ar*C*(5)H (major)), **125.0** (Ar*C*(4)H (major)), **125.3** (Ar*C*(4)H (minor)), **126.9** (Ar*C*(4`,4```)H (major)), **127.4** (Ar*C*(3```,5```)*H* (minor)), **127.4** (Ar*C*(3```,5```)*H* (major)), **127.6** (Ar*C*(3`,5`)*H* (major)), **128.0** (Ar*C*(2```,6```)*H* (minor)), **128.0** (Ar*C*(2```,6```)*H* (major)), **128.1** (Ar*C*(6)H (minor)), **128.3** (Ar*C*(6)H (major)), **129.3**

(Ar(2`,6`)H (major)), **129.6** (Ar*C*(3a) (major)), **129.7** (Ar*C*H), **139.5** (*C*(3``)H (minor)), **140.5** (Ar*C*), **141.9** (*C*(3``)H (major)), **141.9** (Ar*C*(1`) (major)), **142.0** (Ar*C*), **143.7** (Ar*C*(7a)), **179.9** (*C*=O); **HRMS (ESI$^+$)** $C_{37}H_{31}NO_2Na$ $[M+Na]^+$ found 524.2244, requires 524.2247 (–0.49 ppm).

3-Hydroxy-3-(2-phenylbut-3-en-2-yl)indolin-2-one (S24)

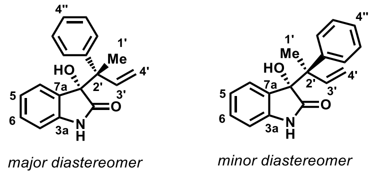

Following **General Procedure L**, [2,3]-rearrangement product **14** (52.1 mg, 0.1 mmol) was treated with Et_3SiH/TFA in CH_2Cl_2 gave a crude product which was purified by flash column chromatography (eluent: hexane/ EtOAc = 3:1 to 1:1) to afford the product as 89:11 mixture of diastereomers (18.1 mg, 65%) as a colourless amorphous solid. **IR** ν_{max} (film) 3361 (OH), 1697 (C=O), 1614 (C=C), 1598 (C=C), 1373, 1184, 1109; $[\mathbf{a}]_{\mathbf{D}}^{\mathbf{20}}$ –63.2 (*c* 0.25, $CHCl_3$); **Chiral HPLC analysis**, Chiralpak ID (95:5 hexane:IPA, flow rate 1 ml·min^{-1}, 211 nm, 30 °C), major diastereomer: t_R (2``*R*,3*R*)-**S24**: 20.5min, $t_R$ (2``*S*,3*S*)-**S24**: 23.8min, 2:98 e.r.; minor diastereomer: t_R (2``*S*,3*R*)-**S24**: 21.1min, $t_R$ (2``*R*,3*S*)-**S24**: 25.9min, 1:99 e.r.; **^{1}H NMR (500 MHz, CD_3OD)** δ **1.48** (2.58H, s, CH_3 (major)), **1.80** (0.42H, s, CH_3 (minor)), **4.87** (0.87H, dd, *J* 17.6, 1.5, C(4`)$H^A$$H^B$ (major)), **5.19** (0.87H, dd, *J* 11.0, 1.5, C(4`)$H^A$$H^B$ (major)), **5.23–5.29** (0.28H, m, C(4`)H_2 (minor)), **6.18–6.39** (1.08H, m, ArC*H* (major and minor)), **6.66–6.79** (2H, m, ArC(5,7)H (major and minor)), **7.12–7.25** (5H, m, ArC*H* (major and minor) and ArC(3``)*H* (major and minor)), **7.25–7.33** (1.75H, m, ArC(2``,6``)*H* (major)), **7.41–7.49** (0.29H, m, ArC*H* (minor)). **^{13}C{^{1}H} NMR (126 MHz, CD_3OD)** δ **19.0** (*C*H_3 (minor)), **20.8** (*C*H_3 (major)), **51.2** (*C*(2`) (minor)), **51.9** (*C*(2`) (major)), **81.8** (*C*(3)OH (major)), **110.3** (Ar*C*(7)*H* (major)), **110.4** (Ar*C*(7)*H* (minor)), **115.1** (*C*(4`)$H_2$ (major)), **116.4** (*C*(4`)H_2 (minor)), **122.4** (Ar*C*(5)H (major and minor)), **127.2** (Ar*C*H (major)), **127.3** (Ar*C*H (minor)), **127.6** (Ar*C*H (minor)), **127.7** (Ar*C*H (major)), **127.9** (Ar*C*(3``,5``)H (major)), **128.2** (Ar*C*H (minor)), **129.7** (Ar*C* (minor)), **130.4** (Ar*C* (major and minor)), **130.6** (Ar*C*(2``,6``)), **131.6** (Ar*C*(3a)), **142.0** (*C*(3`)H (minor)), **142.8** (Ar*C*(1``)), **143.3** (Ar*C*(7a) (minor)), **143.5** (Ar*C*(7a) (major)), **143.9** (*C*(3`)H (major)), **181.6** (*C*=O (major) and minor); **HRMS (ESI$^+$)** $C_{18}H_{17}NO_2Na$ $[M+Na]^+$ found 302.1148, requires 302.1151 (–1.18 ppm).

5-Phenyl-4,5-dihydro-3H-spiro[furan-2,3'-indolin]-2'-one (S25)

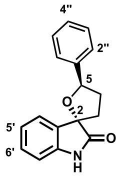

Following **General Procedure L**, formal [1,2]-rearrangement product **46** (52.5 mg, 0.1 mmol) was treated with Et_3SiH/TFA in CH_2Cl_2 to give a crude product which was purified by flash column chromatography (eluent: hexane/ EtOAc = 3:1 to 1:1) to afford a single diastereomer of the product (13.5 mg, 51%) as a colourless amorphous solid. **IR** ν_{max} (film) 3246 (N-H), 2924 (C-H), 1717 (C=O), 1620 (C=C), 1469, 1332, 1207, 1109 (C-O), 1049; $[\mathbf{a}]_{\mathbf{D}}^{\mathbf{20}}$ –71.2 (*c* 0.65, $CHCl_3$); **Chiral HPLC analysis**, Chiralcel ODH (90:10 hexane:IPA, flow rate 1 ml·min^{-1}, 211 nm, 30 °C) t_R (2'*R*,3*S*)-**S25**: 11.7 min, t_R (2'*S*,3*R*)-**S25**: 15.5min, 92:8 e.r.; **^{1}H NMR (500 MHz, $CDCl_3$)** δ **2.21** (1H, app dq, *J* 12.2, 8.5, C(4)H^AH^B), **2.30** (1H, ddd, *J* 12.4, 8.3, 3.1, C(2)H^AH^B), **2.53–2.63** (1H, m, C(2)H^AH^B), **2.73–2.85** (1H, m, C(4)H^AH^B), **5.55** (1H, dd, *J* 8.4, 6.2, C(5)*H*), **6.88** (1H, d, *J* 7.7, ArC(7`)*H*), **7.08** (1H, app t, *J* 7.4, ArC(5`)*H*), **7.23–7.33** (2H, m, ArC(4`,6`)*H*), **7.33–7.40** (3H, m, ArC(3``,4``,5``)*H*), **7.45** (2H, app d, *J* 7.1, ArC(2``,6``)*H*), **7.97–8.23** (1H, m, N*H*). **$^{13}$C{$^1$H} NMR (101 MHz, $CDCl_3$)** δ **35.3** (*C*(4)$H_2$), **36.3** (*C*(3)$H_2$), **83.1** (*C*(5)HPh), **83.7** (*C*(2)O), **110.3** (Ar*C*(7`)H), **123.3** (Ar*C*(5`)H), **124.3** (Ar*C*(4``)H), **126.0** (Ar*C*(2``,6``)H), **127.8** (Ar*C*(4`)H), **128.6** (Ar*C*(3``,5``)H), **129.8** (Ar*C*(6`)H), **131.3** (Ar*C*(3a`)), **140.7** (Ar*C*(1``)), **141.8** (Ar*C*(7a`)), **180.2** (*C*=O); **HRMS (ESI$^+$)** $C_{17}H_{15}NO_2Na$ $[M+Na]^+$ found 288.0988, requires 288.0995 (–2.25 ppm).

9 Supplementary HPLC data of chiral compounds

Fig. S25: Chiral HPLC analysis for (*S*,*E*)-3-Hydroxy-3-(3-phenylbut-2-en-1-yl)-1-tritylindolin-2-one (**9**), Chiralcel OD-H (90:10 hexane:IPA, flow rate 1mlmin^{-1}, 211 nm, 30 °C) t_R (*R*)-**9**: 7.0min, t_R (*S*)-**9**: 8.7min, 5:95 e.r.

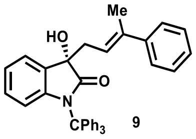

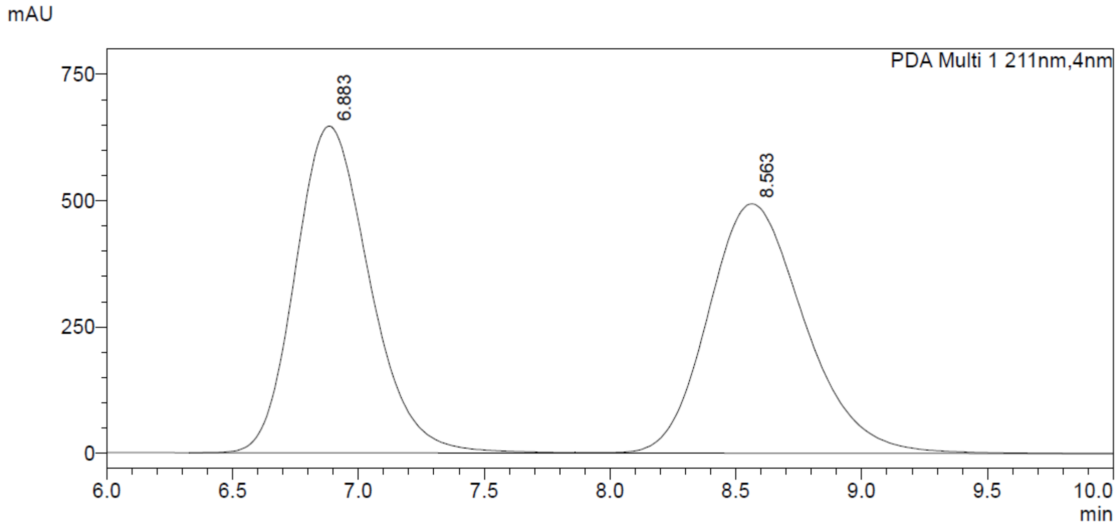

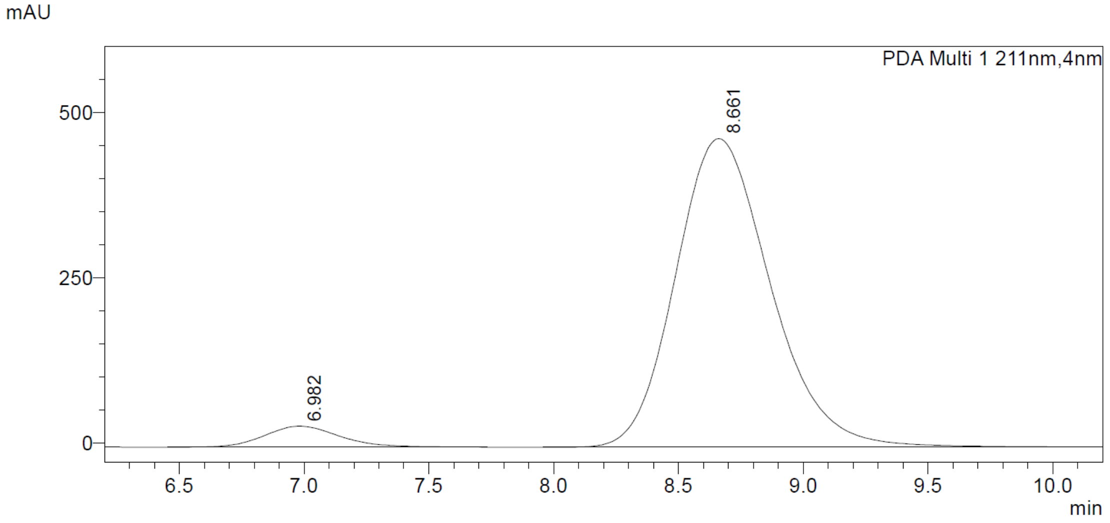

Racemic

<Peak Table>

PDA Ch1 211nm

Peak#	Ret. Time	Area%
1	6.883	50.180
2	8.563	49.820
Total		100.000

Enantioenriched

<Peak Table>

PDA Ch1 211nm

Peak#	Ret. Time	Area%
1	6.982	5.124
2	8.661	94.876
Total		100.000

Fig. S26: Chiral HPLC analysis for (*E*)-3-Hydroxy-3'-[(3-phenylbut-2-en-1-yl)oxy]-1,1'-ditrityl-[3,3'-biindoline]-2,2'-dione (**10**), Chiralpak IB (98:2 hexane:IPA, flow rate 1mlmin^{-1}, 211 nm, 30 °C) t_R (major)-**10**: 12.18 min, t_R (minor)-**10**: 17.50 min, 75:25 e.r.

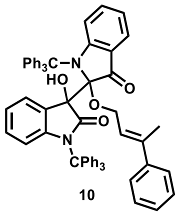

10

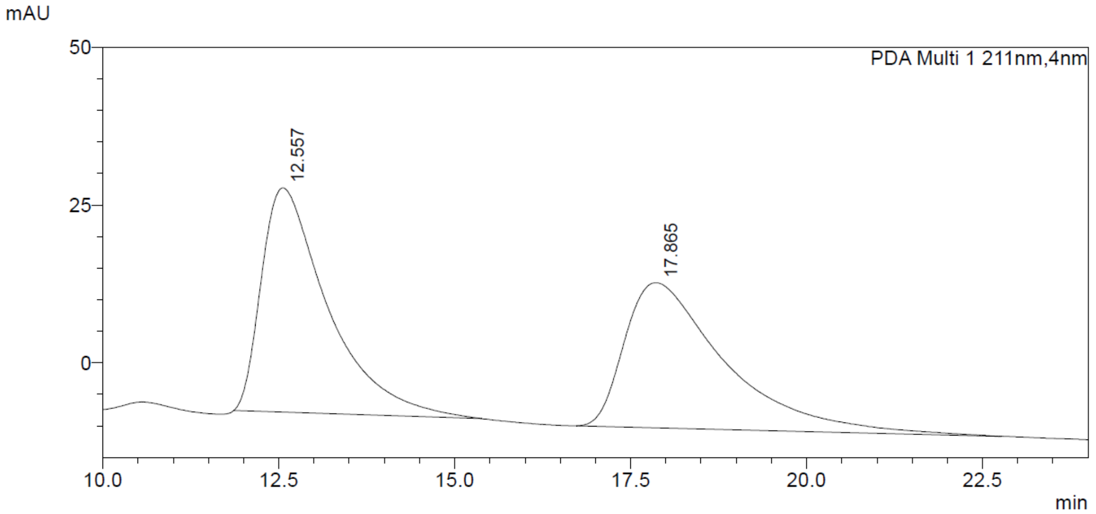

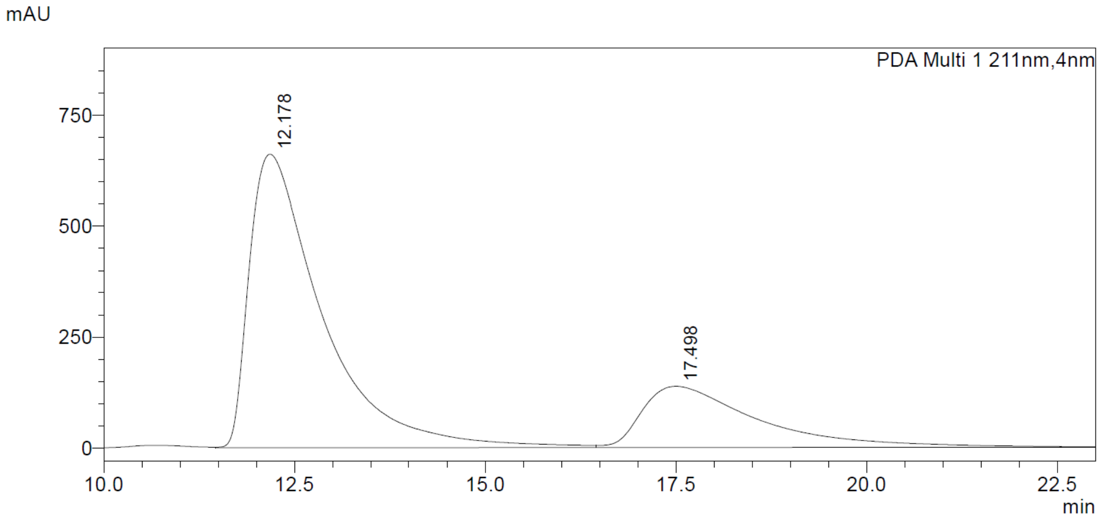

Racemic	Enantioenriched

Racemic

<Peak Table>

PDA Ch1 211nm

Peak#	Ret. Time	Area%
1	12.557	50.613
2	17.865	49.387
Total		100.000

Enantioenriched

<Peak Table>

PDA Ch1 211nm

Peak#	Ret. Time	Area%
1	12.178	75.019
2	17.498	24.981
Total		100.000

Fig. S27: Chiral HPLC analysis for (*S*,*E*)-3-Hydroxy-3-(3-phenylpent-2-en-1-yl)-1-tritylindolin-2-one (**19**), Chiralcel OD-H (90:10 hexane:IPA, flow rate 1mlmin^{-1}, 211 nm, 30 °C) t_R (*R*)-**19**: 8.9 min, t_R (*S*)-**19**: 11.2 min, 94:6 e.r.

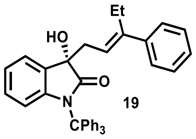

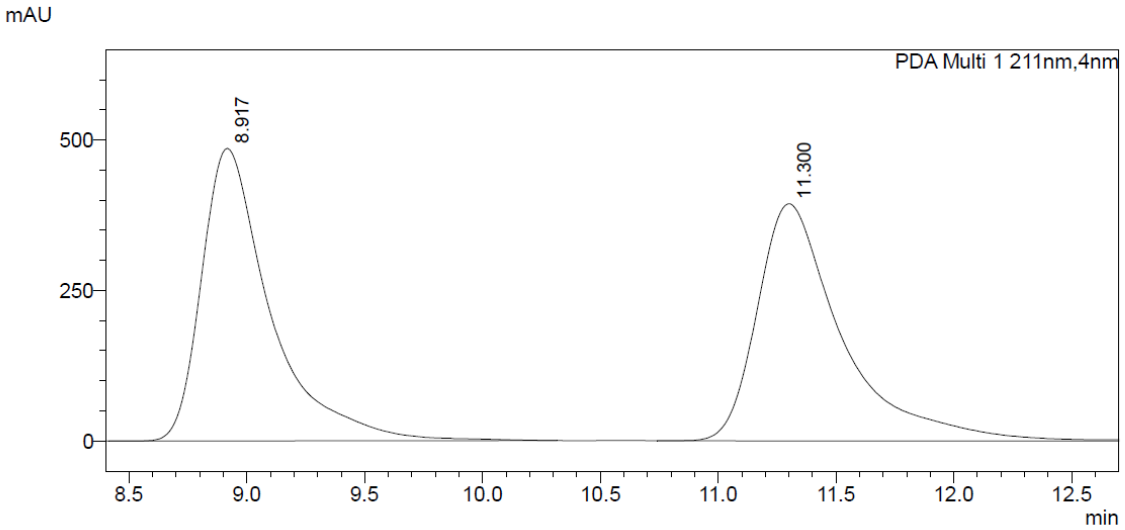

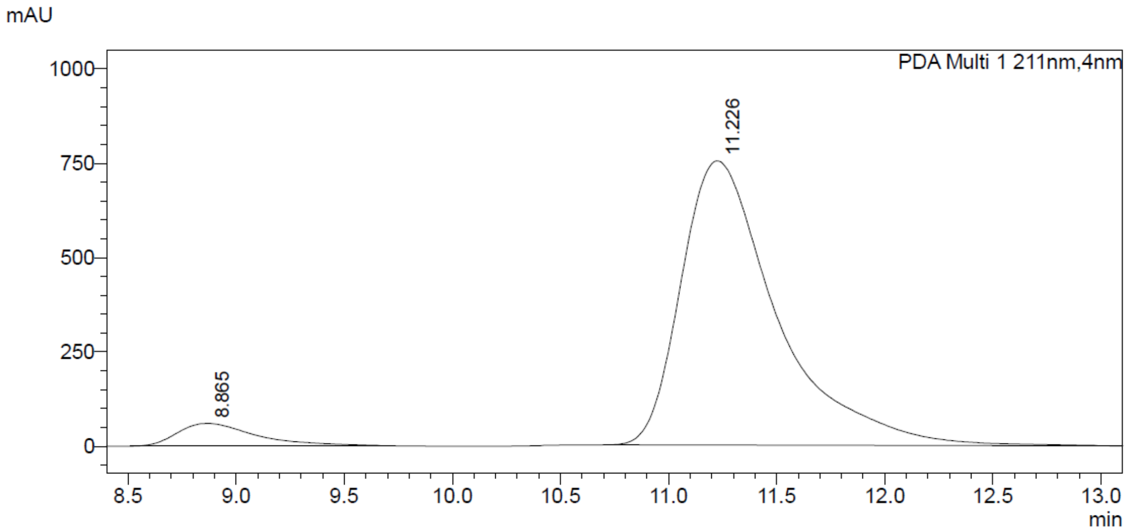

Racemic			Enantioenriched		
<Peak Table>			<Peak Table>		
PDA Ch1 211nm			PDA Ch1 211nm		
Peak#	Ret. Time	Area%	Peak#	Ret. Time	Area%
1	8.917	49.995	1	8.865	6.289
2	11.300	50.005	2	11.226	93.711
Total		100.000	Total		100.000

Fig. S28: Chiral HPLC analysis for (*S*,*E*)-3-(3-Cyclopropyl-3-phenylallyl)-3-hydroxy-1-tritylindolin-2-one (**20**), Chiralpak IA (95:5 hexane:IPA, flow rate 1mlmin^{-1}, 211 nm, 30 °C) t_R (*R*)-**20**: 17.4min, t_R (*S*)-**20**: 24.3min, 4:96 e.r.

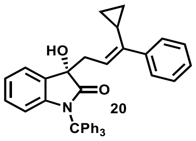

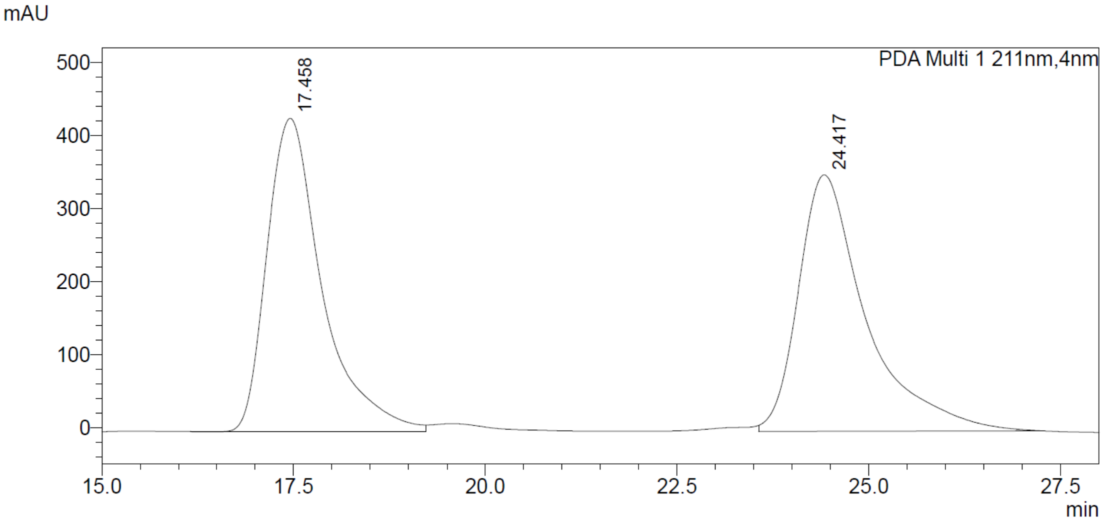

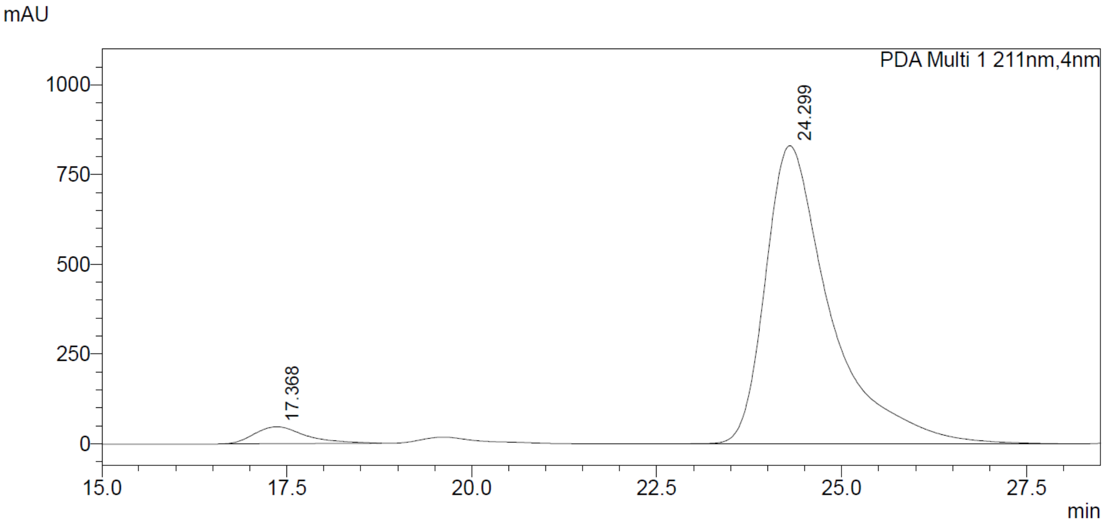

Racemic			Enantioenriched		
**<Peak Table>**			**<Peak Table>**		
PDA Ch1 211nm			PDA Ch1 211nm		
Peak#	Ret. Time	Area%	Peak#	Ret. Time	Area%
1	17.458	49.883	1	17.368	4.246
2	24.417	50.117	2	24.299	95.754
Total		100.000	Total		100.000

Fig. S29: Chiral HPLC analysis for (*S*)-1-Benzyl-3-(3,3-diphenylallyl)-3-hydroxyindolin-2-one (**21**), Chiralcel OD-H (90:10 hexane:IPA, flow rate 1mlmin^{-1}, 211 nm, 30 °C) t_R (*S*)-**21**: 11.2 min, t_R (*R*)-**21**: 13.1 min, 73:27 e.r.

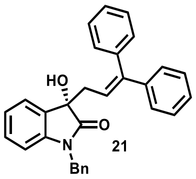

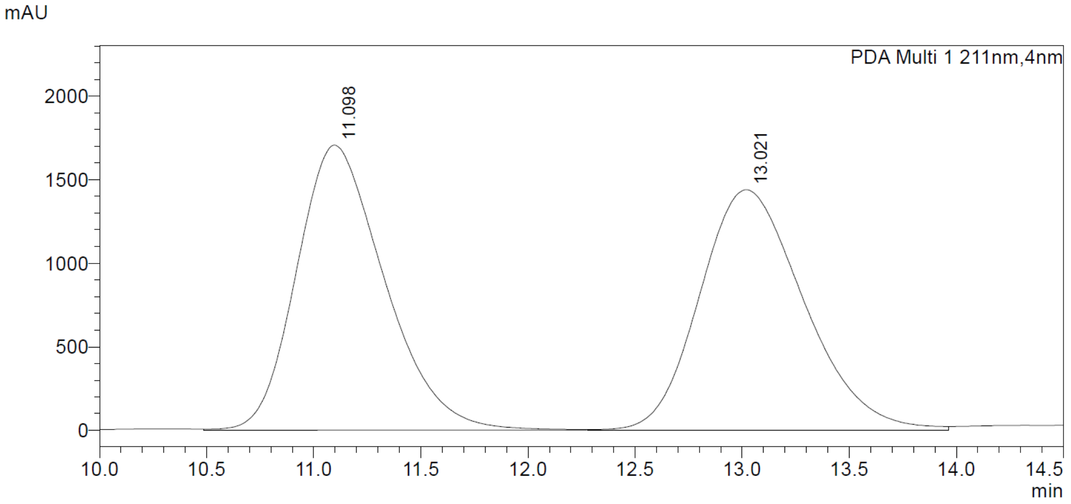

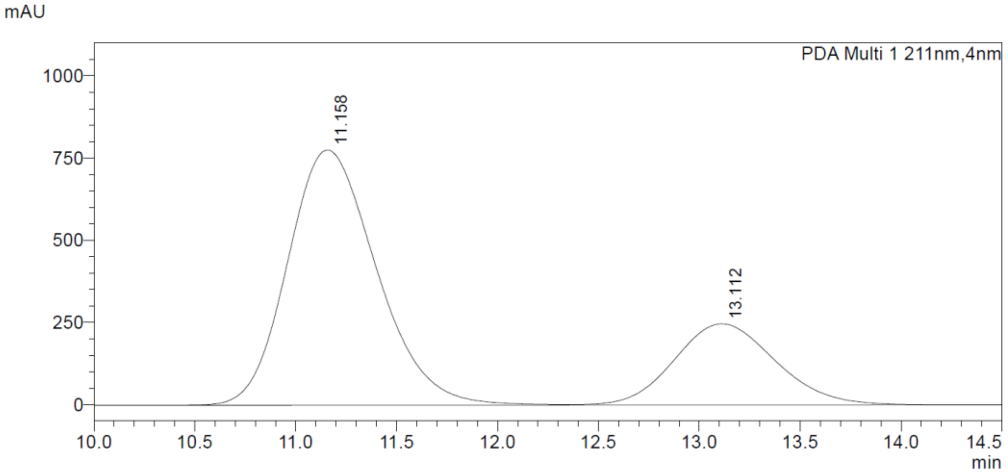

Racemic

<Peak Table>

PDA Ch1 211nm

Peak#	Ret. Time	Area%
1	11.098	50.045
2	13.021	49.955
Total		100.000

Enantioenriched

<Peak Table>

PDA Ch1 211nm

Peak#	Ret. Time	Area%
1	11.158	73.371
2	13.112	26.629
Total		100.000

Fig. S30: Chiral HPLC analysis for (*S*,*E*)-3-(3,3-diphenylallyl)-3-hydroxy-1-tritylindolin-2-one (**S6**), Chiralpak IA (90:10 hexane:IPA, flow rate 1mlmin^{-1}, 211 nm, 30 °C) t_R (*S*)-**S6**: 11.0 min, t_R (*R*)-**S6**: 14.4 min, 83:17 e.r.

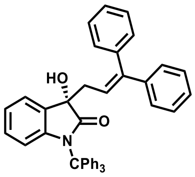

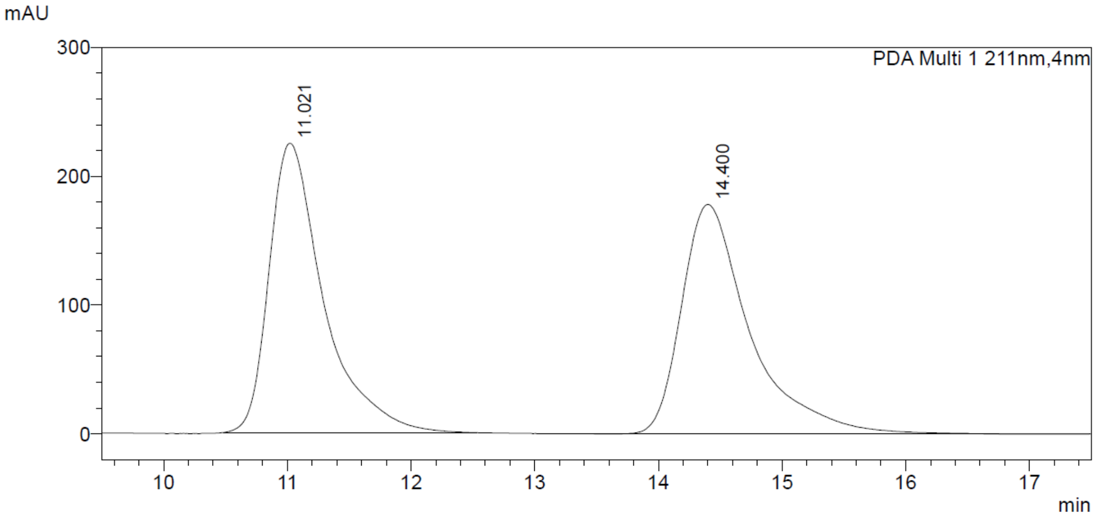

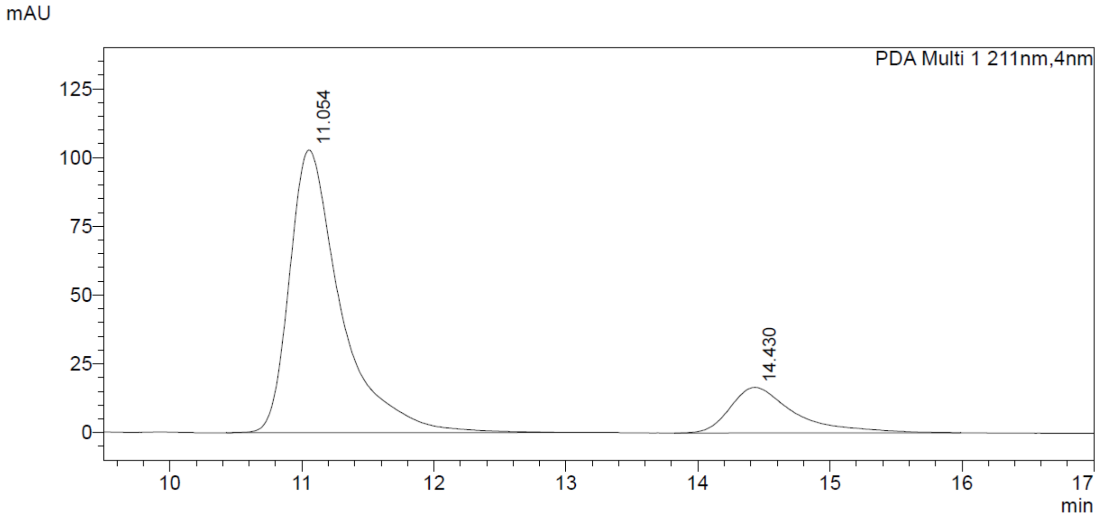

Racemic			Enantioenriched		
**<Peak Table>**			**<Peak Table>**		
PDA Ch1 211nm			PDA Ch1 211nm		
Peak#	Ret. Time	Area%	Peak#	Ret. Time	Area%
1	11.021	50.114	1	11.054	83.201
2	14.400	49.886	2	14.430	16.799
Total		100.000	Total		100.000

Fig. S31: Chiral HPLC analysis for (*S*,*E*)-3-[3-(4-Fluorophenyl)but-2-en-1-yl]-3-hydroxy-1-tritylindolin-2-one (**13**), Chiralcel OD-H (95:5 hexane:IPA, flow rate 1mlmin^{-1}, 211 nm, 30 °C) t_R (*R*)-**13**: 11.1 min, t_R (*S*)-**13**: 15.3 min, 6:94 e.r.

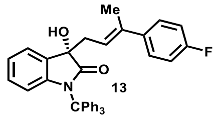

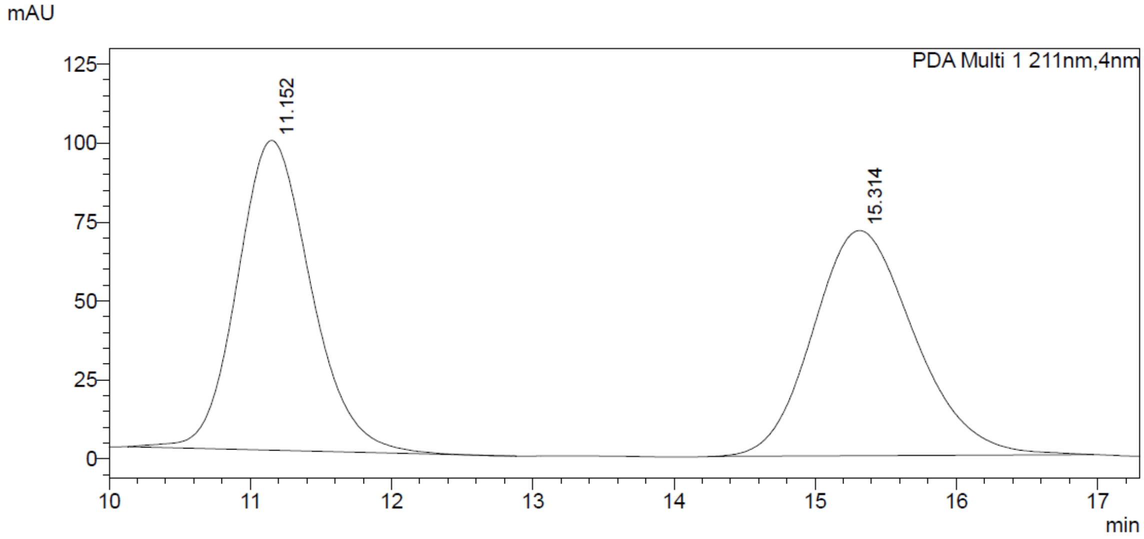

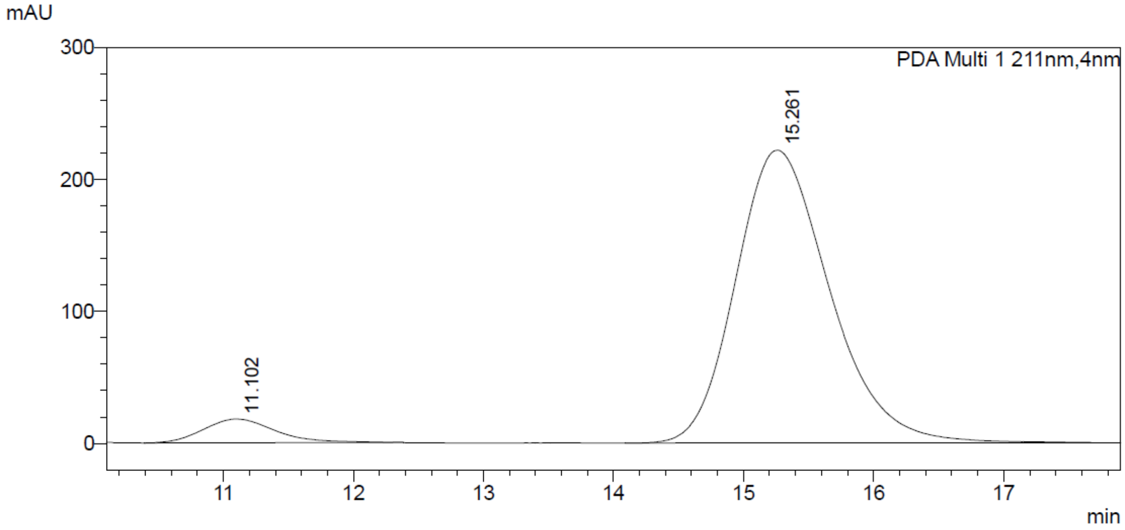

Racemic	Enantioenriched

Racemic

<Peak Table>

PDA Ch1 211nm

Peak#	Ret. Time	Area%
1	11.152	50.000
2	15.314	50.000
Total		100.000

Enantioenriched

<Peak Table>

PDA Ch1 211nm

Peak#	Ret. Time	Area%
1	11.102	5.933
2	15.261	94.067
Total		100.000

Fig. S32: Chiral HPLC analysis for (*S*,*E*)-3-[3-(4-Chlorophenyl)but-2-en-1-yl]-3-hydroxy-1-tritylindolin-2-one (**22**), Chiralpak IA (90:10 hexane:IPA, flow rate 1mlmin^{-1}, 211 nm, 30 °C) t_R (*R*)-**22**: 11.4 min, t_R (*S*)-**22**: 14.4 min, 5:95 e.r.

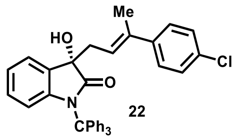

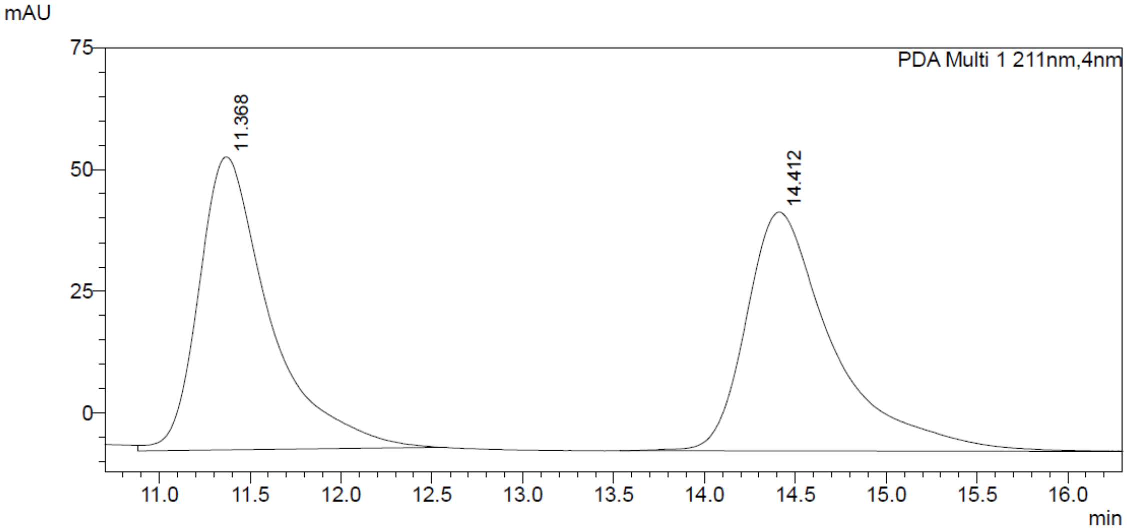

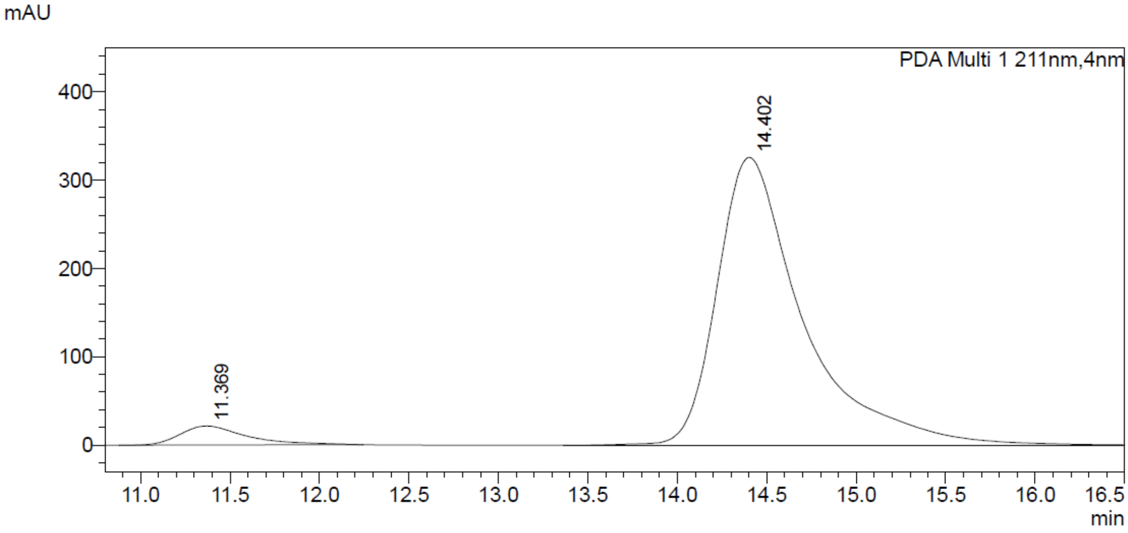

Racemic

<Peak Table>

PDA Ch1 211nm

Peak#	Ret. Time	Area%
1	11.368	50.048
2	14.412	49.952
Total		100.000

Enantioenriched

<Peak Table>

PDA Ch1 211nm

Peak#	Ret. Time	Area%
1	11.369	4.848
2	14.402	95.152
Total		100.000

Fig. S33: Chiral HPLC analysis for (*S*,*E*)-3-[3-(4-Bromophenyl)but-2-en-1-yl]-3-hydroxy-1-tritylindolin-2-one (**23**), Chiralpak IA (90:10 hexane:IPA, flow rate 1mlmin^{-1}, 211 nm, 30 °C) t_R (*R*)-**23**: 11.7 min, t_R (*S*)-**23**: 14.7 min, 5:95 e.r.

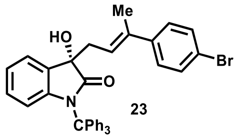

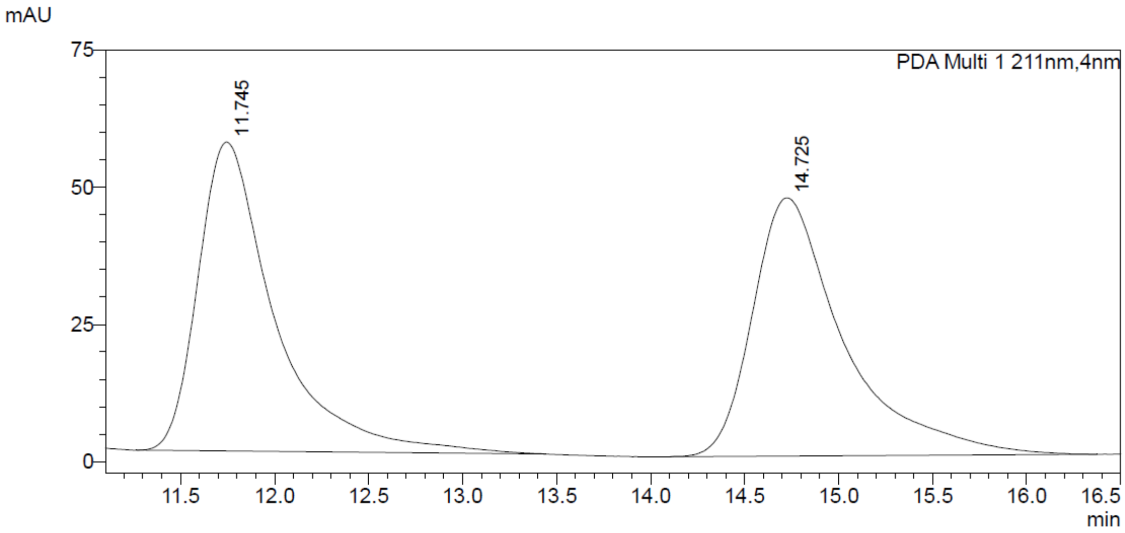

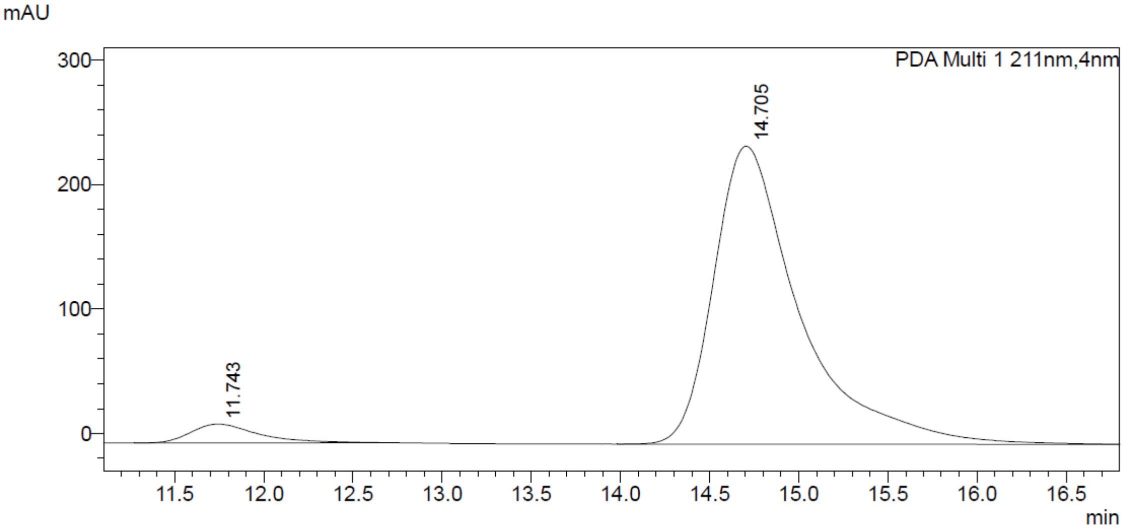

Racemic	Enantioenriched

Racemic

<Peak Table>

PDA Ch1 211nm

Peak#	Ret. Time	Area%
1	11.745	50.147
2	14.725	49.853
Total		100.000

Enantioenriched

<Peak Table>

PDA Ch1 211nm

Peak#	Ret. Time	Area%
1	11.743	4.715
2	14.705	95.285
Total		100.000

Fig. S34: Chiral HPLC analysis for (*S*,*E*)-3-Hydroxy-3-{3-[4-(trifluoromethyl)phenyl]but-2-en-1-yl}-1-tritylindolin-2-one (**24**), Chiralpak IA (90:10 hexane:IPA, flow rate 1mlmin^{-1}, 211 nm, 30 °C) t_R (*R*)-**24**: 10.6 min, t_R (*S*)-**24**: 12.8 min, 4:96 e.r.

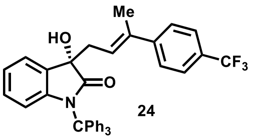

<Chromatogram>

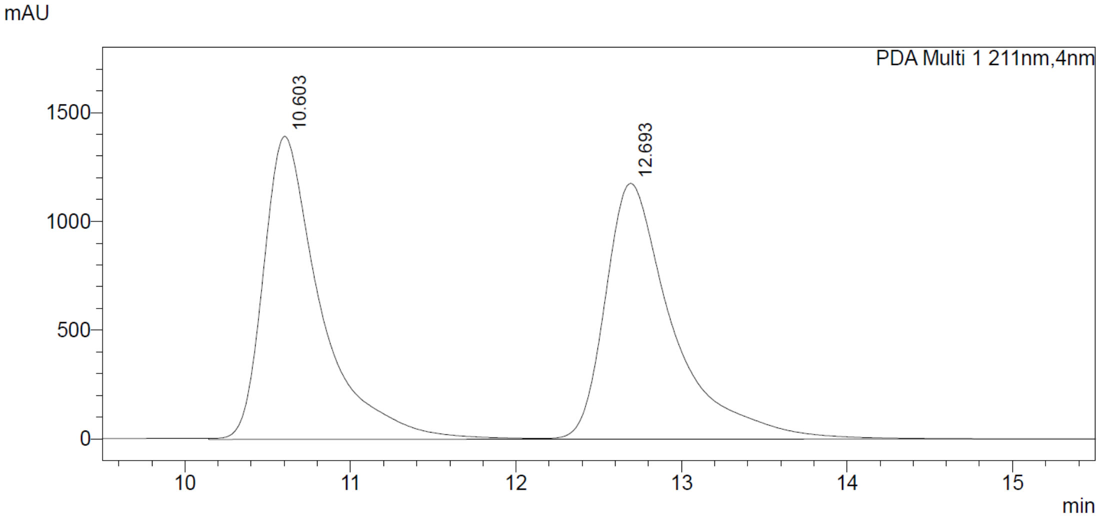

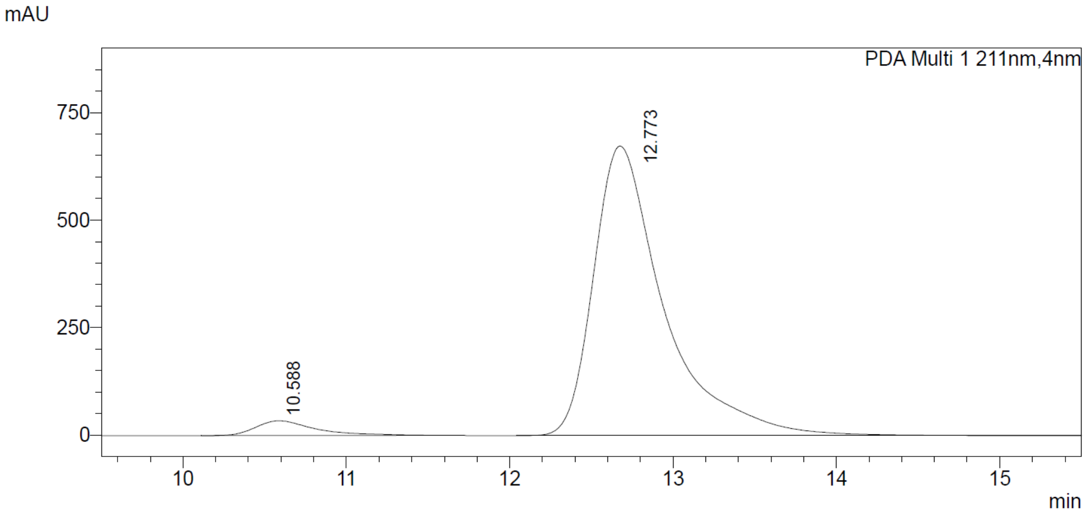

Racemic

<Peak Table>

PDA Ch1 211nm

Peak#	Ret. Time	Area%
1	10.603	49.983
2	12.693	50.017
Total		100.000

Enantioenriched

<Peak Table>

PDA Ch1 211nm

Peak#	Ret. Time	Area%
1	10.588	4.131
2	12.773	95.869
Total		100.000

Fig. S35: Chiral HPLC analysis for (*S*,*E*)-3-{3-[(1,1'-biphenyl)-4-yl]but-2-en-1-yl}-3-hydroxy-1-tritylindolin-2-one (**25**), Chiralpak IA (90:10 hexane:IPA, flow rate 1mlmin^{-1}, 211 nm, 30 °C) t_R (*R*)-**25**: 13.5 min, t_R (*S*)-**25**: 15.8 min, 5:95 e.r.

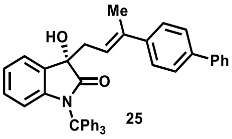

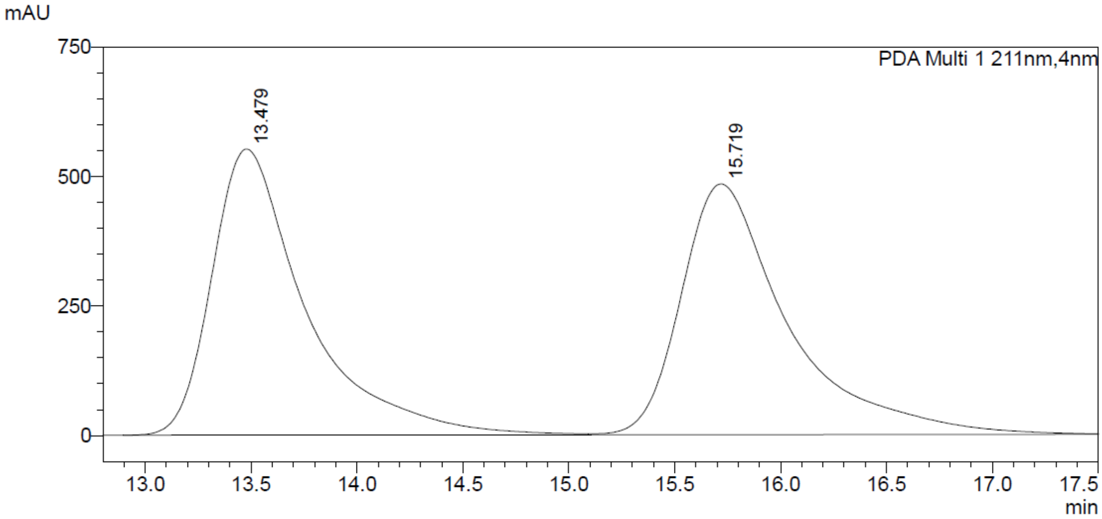

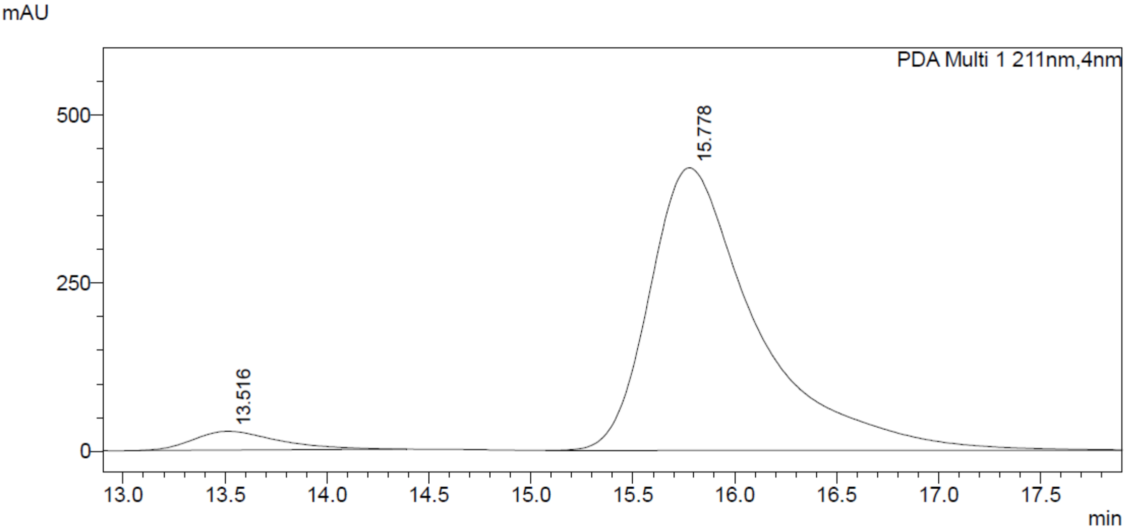

Racemic

<Peak Table>

PDA Ch1 211nm

Peak#	Ret. Time	Area%
1	13.479	50.032
2	15.719	49.968
Total		100.000

Enantioenriched

<Peak Table>

PDA Ch1 211nm

Peak#	Ret. Time	Area%
1	13.516	5.044
2	15.778	94.956
Total		100.000

Fig. S36: Chiral HPLC analysis for (*S*,*E*)-3-Hydroxy-3-[3-(p-tolyl)but-2-en-1-yl]-1-tritylindolin-2-one (**26**), Chiralpak IA (90:10 hexane:IPA, flow rate 1mlmin^{-1}, 211 nm, 30 °C) t_R (*R*)-**26**: 9.6 min, t_R (*S*)-**26**: 11.8 min, 7:93 e.r.

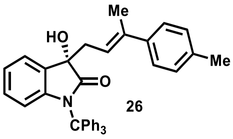

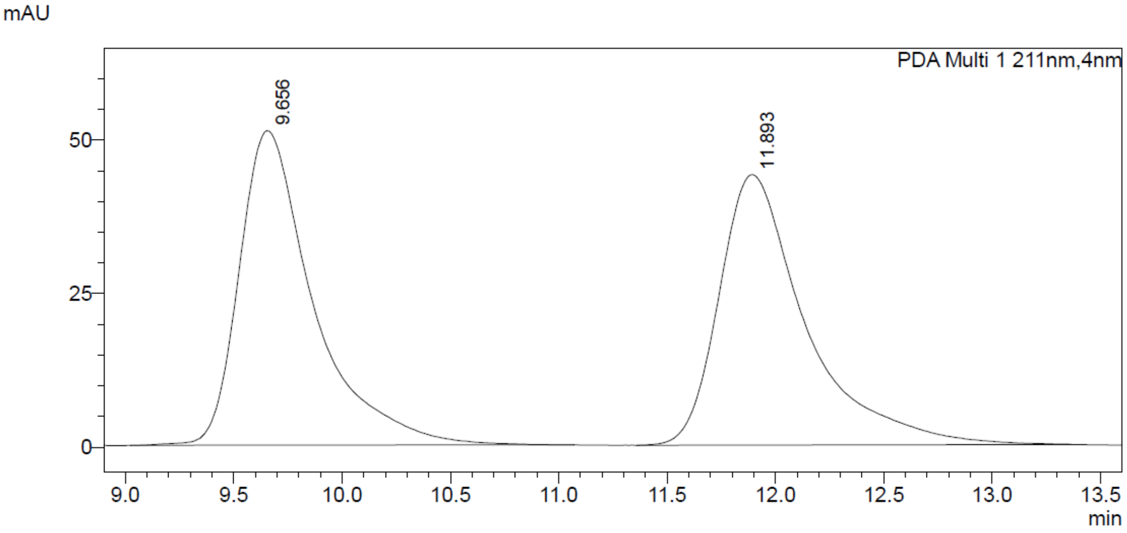

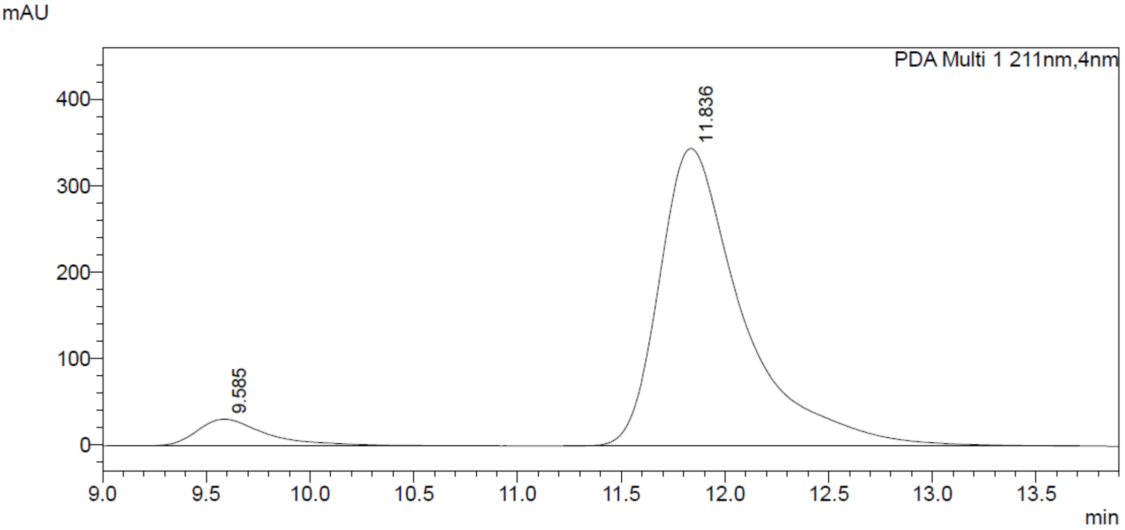

Racemic

<Peak Table>

PDA Ch1 211nm

Peak#	Ret. Time	Area%
1	9.656	49.851
2	11.893	50.149
Total		100.000

Enantioenriched

<Peak Table>

PDA Ch1 211nm

Peak#	Ret. Time	Area%
1	9.585	6.948
2	11.836	93.052
Total		100.000

Fig. S37: Chiral HPLC analysis for (*S*,*E*)-3-Hydroxy-3-[3-(4-methoxyphenyl)but-2-en-1-yl]-1-tritylindolin-2-one (**18**), Chiralpak IA (90:10 hexane:IPA, flow rate 1mlmin^{-1}, 211 nm, 30 °C) t_R (*R*)-**18**: 13.3 min, t_R (*S*)-**18**: 15.8 min, 6:94 e.r.

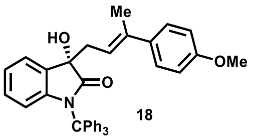

18

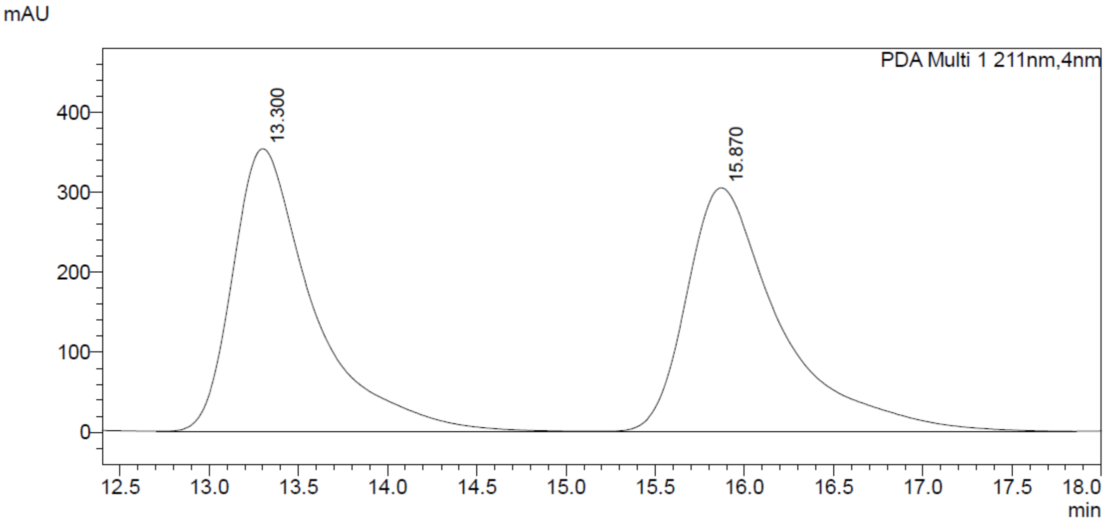

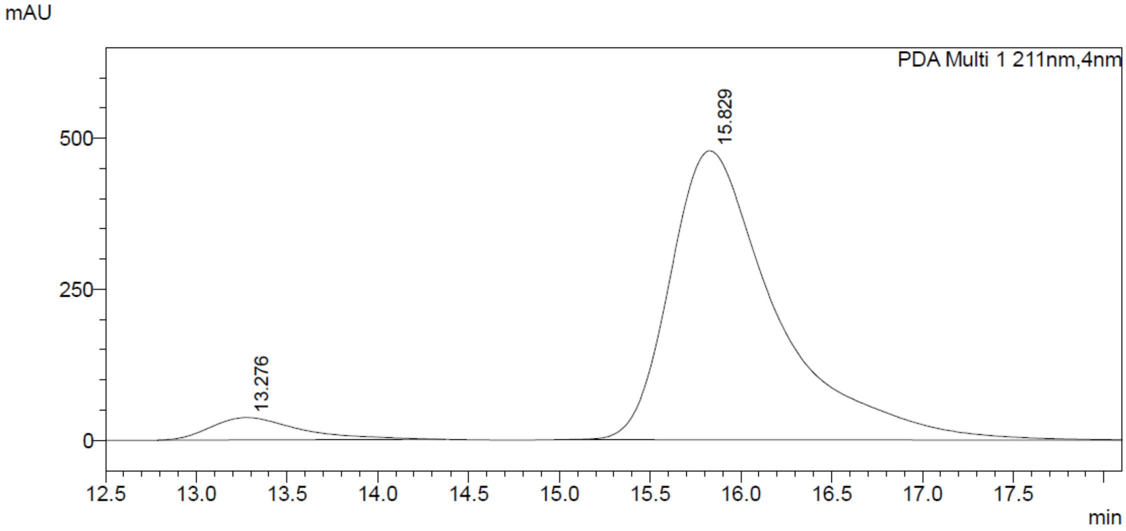

Racemic			Enantioenriched		
**<Peak Table>**			**<Peak Table>**		
PDA Ch1 211nm			PDA Ch1 211nm		
Peak#	Ret. Time	Area%	Peak#	Ret. Time	Area%
1	13.300	49.959	1	13.276	6.125
2	15.870	50.041	2	15.829	93.875
Total		100.000	Total		100.000

Fig. S38: Chiral HPLC analysis for (*S*,*E*)-3-Hydroxy-3-[3-(m-tolyl)but-2-en-1-yl]-1-tritylindolin-2-one (**27**), Chiralpak IA (90:10 hexane:IPA, flow rate 1mlmin^{-1}, 211 nm, 30 °C) t_R (*R*)-**27**: 9.2 min, t_R (*S*)-**27**: 11.7 min, 6:94 e.r.

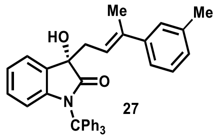

27

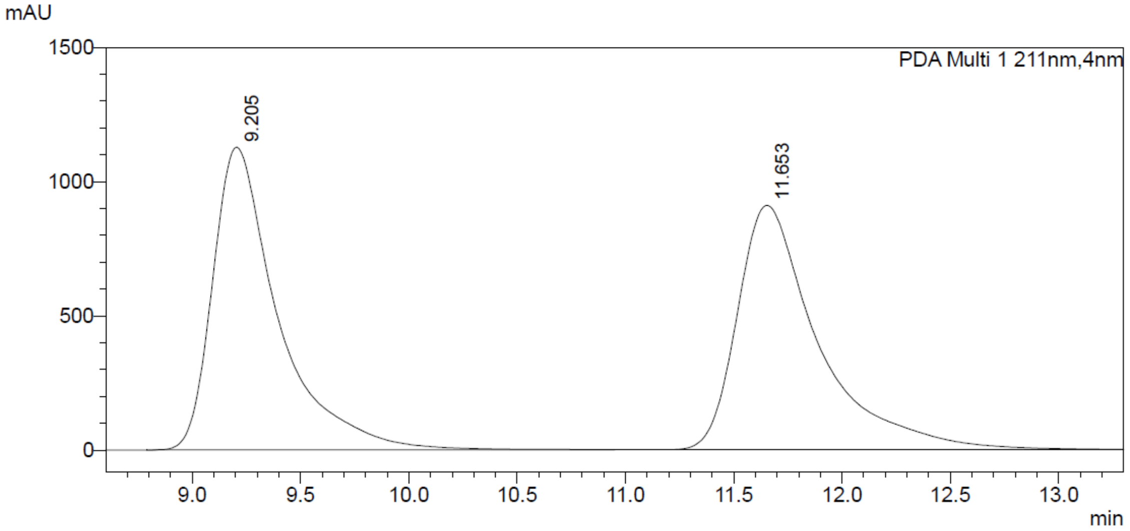

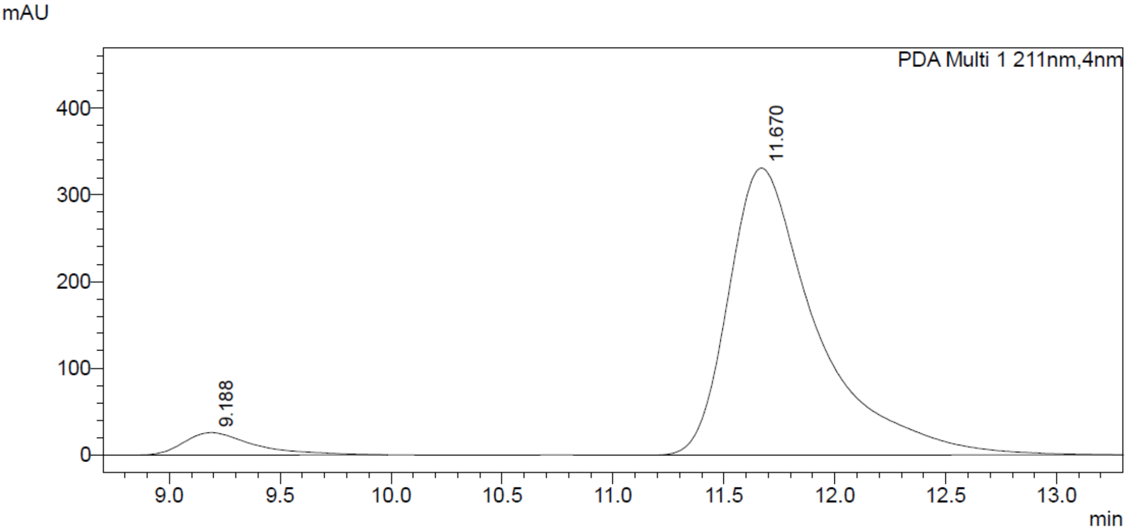

Racemic

<Peak Table>

PDA Ch1 211nm

Peak#	Ret. Time	Area%
1	9.205	49.933
2	11.653	50.067
Total		100.000

Enantioenriched

<Peak Table>

PDA Ch1 211nm

Peak#	Ret. Time	Area%
1	9.188	5.832
2	11.670	94.168
Total		100.000

Fig. S39: Chiral HPLC analysis for (*S*,*E*)-3-Hydroxy-3-[3-(0-tolyl)but-2-en-1-yl]-1-tritylindolin-2-one (**28**), Chiralpak IA (90:10 hexane:IPA, flow rate 1mlmin^{-1}, 211 nm, 30 °C) t_R (*R*)-**28**: 8.2 min, t_R (*S*)-**28**: 12.2 min, 4:96 e.r.

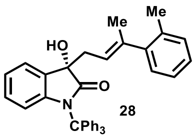

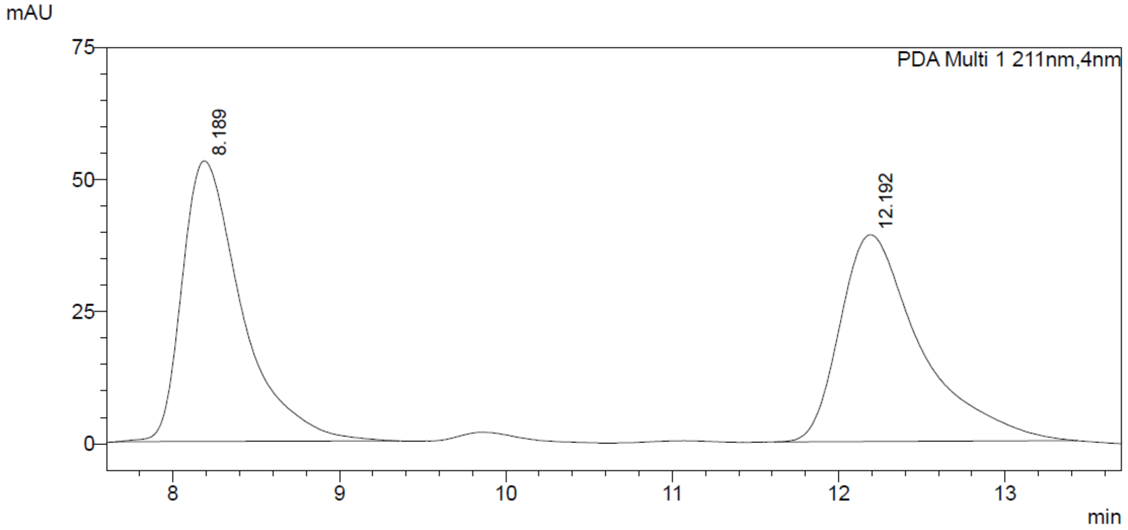

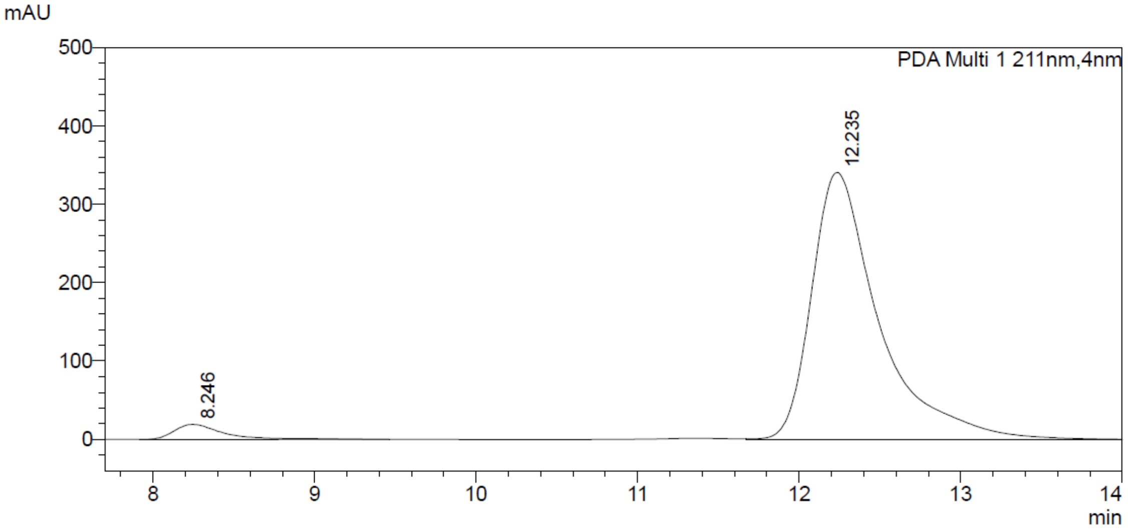

Racemic

<Peak Table>

PDA Ch1 211nm

Peak#	Ret. Time	Area%
1	8.189	50.139
2	12.192	49.861
Total		100.000

Enantioenriched

<Peak Table>

PDA Ch1 211nm

Peak#	Ret. Time	Area%
1	8.246	4.012
2	12.235	95.988
Total		100.000

Fig. S40: Chiral HPLC analysis for (*S*,*E*)-3-Hydroxy-3-[3-(naphthalen-2-yl)but-2-en-1-yl]-1-tritylindolin-2-one (**29**), Chiralcel OD-H (90:10 hexane:IPA, flow rate 1mlmin^{-1}, 211 nm, 30 °C) t_R (*R*)-**29**: 8.2 min, t_R (*S*)-**29**: 11.8 min, 6:94 e.r.

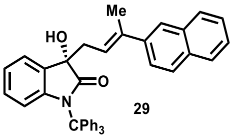

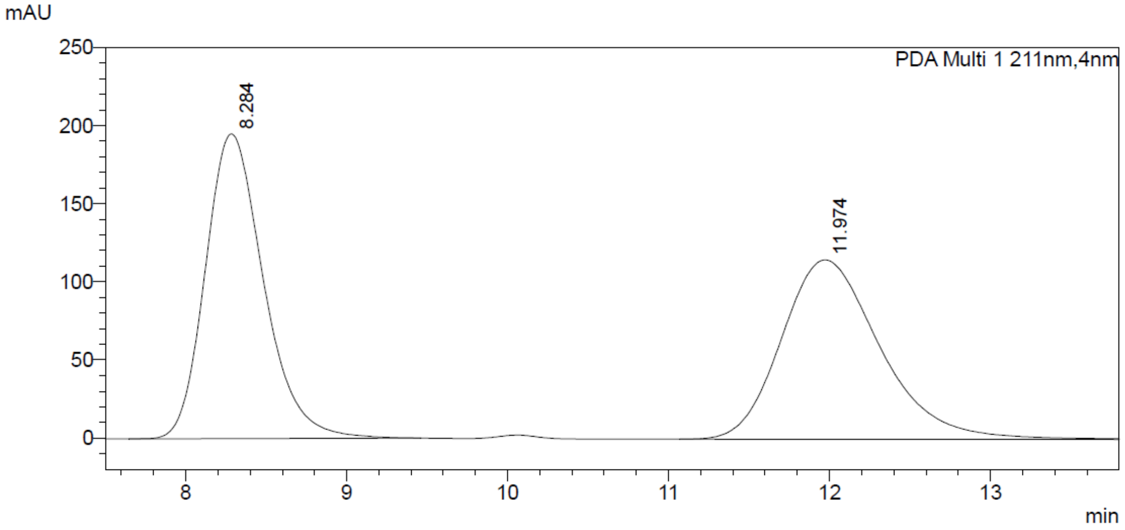

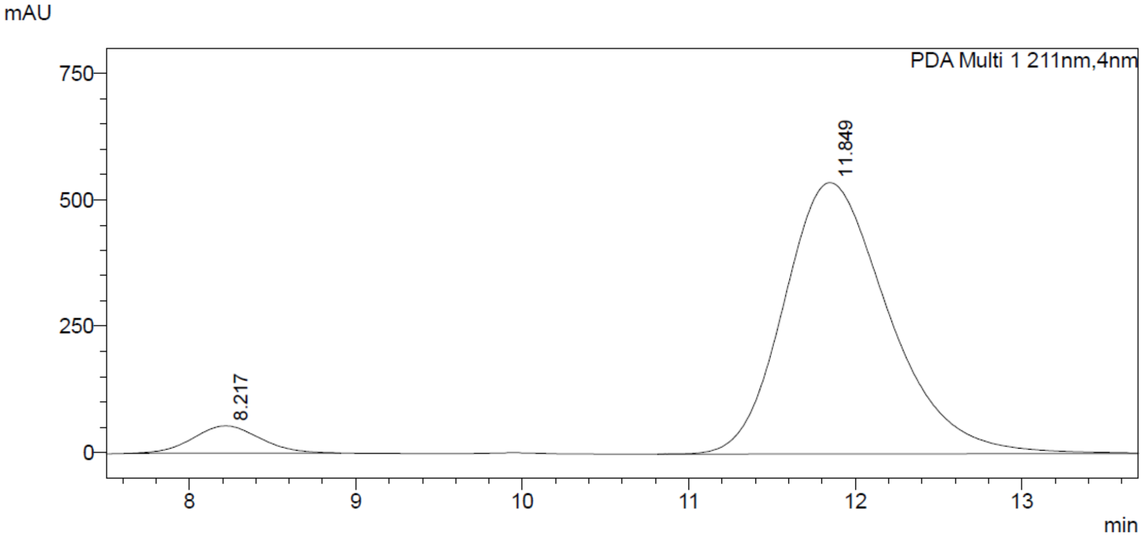

Racemic			Enantioenriched		
**<Peak Table>** PDA Ch1 211nm			**<Peak Table>** PDA Ch1 211nm		
Peak#	Ret. Time	Area%	Peak#	Ret. Time	Area%
1	8.284	49.915	1	8.217	6.066
2	11.974	50.085	2	11.849	93.934
Total		100.000	Total		100.000

Fig. S41: Chiral HPLC analysis for (*S*,*E*)-3-Hydroxy-3-[3-(thiophen-2-yl)but-2-en-1-yl]-1-tritylindolin-2-one (**30**), Chiralpak IA (90:10 hexane:IPA, flow rate 1mlmin^{-1}, 211 nm, 30 °C) t_R (*R*)-**30**: 11.0 min, t_R (*S*)-**30**: 14.2 min, 6:94 e.r.

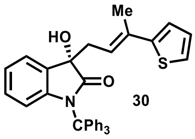

30

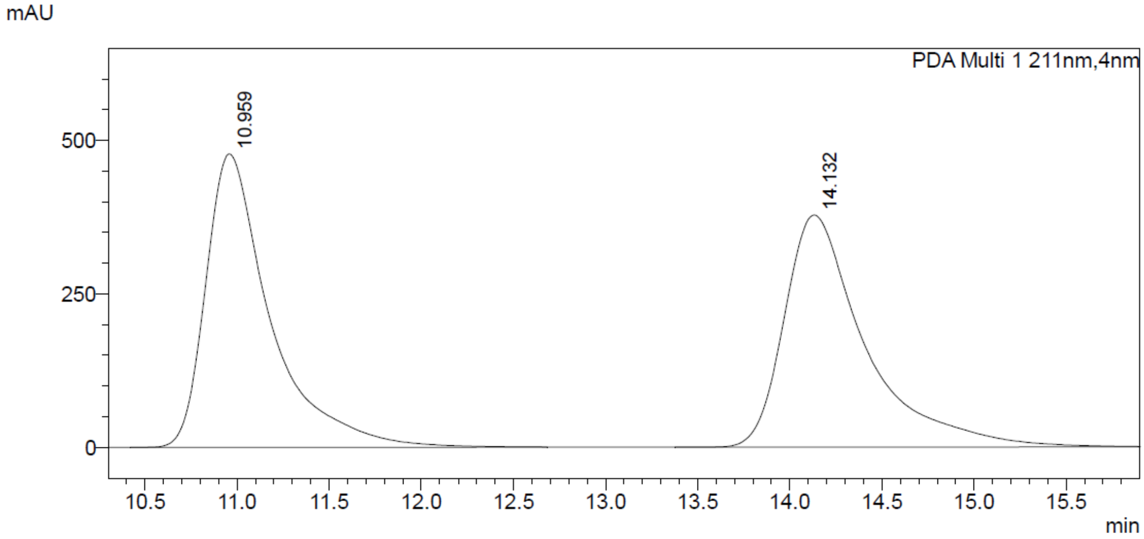

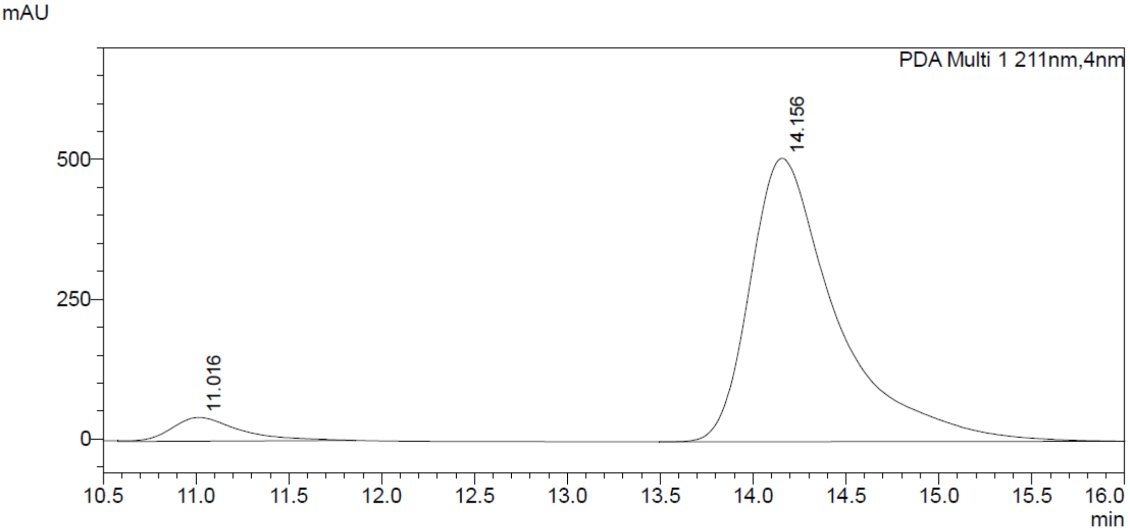

Racemic

<Peak Table>

PDA Ch1 211nm

Peak#	Ret. Time	Area%
1	10.959	50.068
2	14.132	49.932
Total		100.000

Enantioenriched

<Peak Table>

PDA Ch1 211nm

Peak#	Ret. Time	Area%
1	11.016	6.137
2	14.156	93.863
Total		100.000

Fig. S42: Chiral HPLC analysis (*S*,*E*)-3-Hydroxy-3-[3-(thiazol-2-yl)but-2-en-1-yl]-1-tritylindolin-2-one (**31**), Chiralpak IA (90:10 hexane:IPA, flow rate 1mlmin^{-1}, 211 nm, 30 °C) t_R (*R*)-**31**: 13.2 min, t_R (*S*)-**31**: 16.2 min, 7:93 e.r.

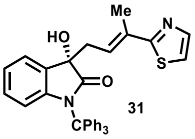

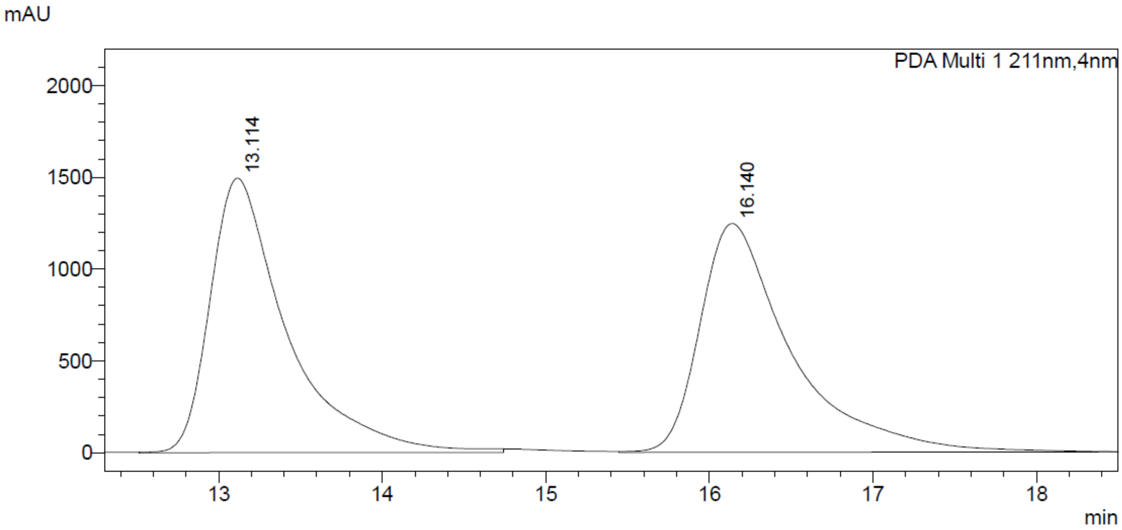

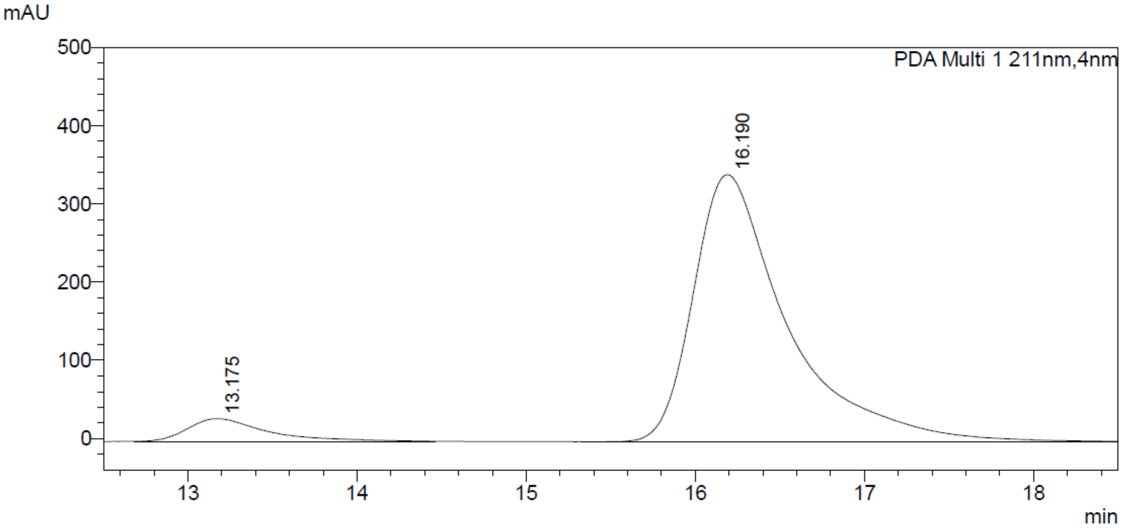

Racemic

<Peak Table>

PDA Ch1 211nm

Peak#	Ret. Time	Area%
1	13.114	50.228
2	16.140	49.772
Total		100.000

Enantioenriched

<Peak Table>

PDA Ch1 211nm

Peak#	Ret. Time	Area%
1	13.175	6.644
2	16.190	93.356
Total		100.000

Fig. S43: Chiral HPLC analysis for (*S*,*E*)-4-Chloro-3-hydroxy-3-(3-phenylbut-2-en-1-yl)-1-tritylindolin-2-one (**32**), Chiralcel OD-H (95:5 hexane:IPA, flow rate 1mlmin^{-1}, 211 nm, 30 °C) t_R (*R*)-**32**: 6.5 min, t_R (*S*)-**32**: 8.2 min, 8:92 e.r.

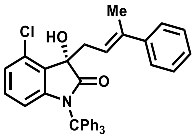

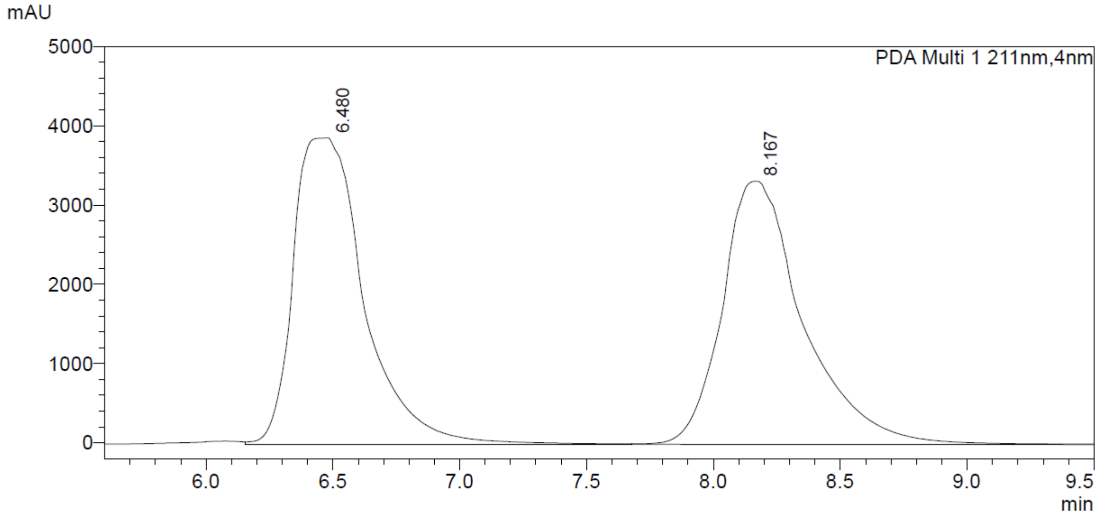

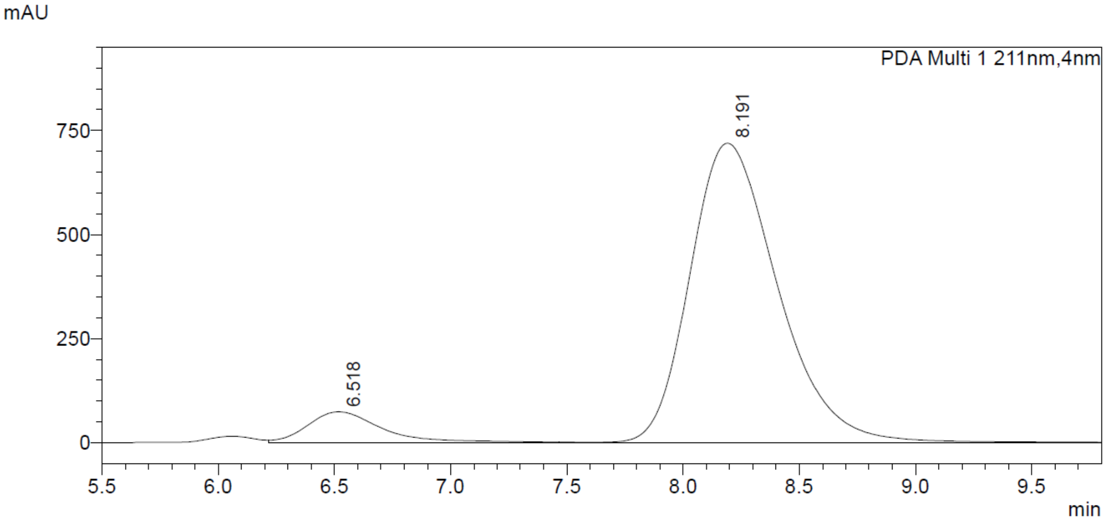

Racemic

<Peak Table>

PDA Ch1 211nm

Peak#	Ret. Time	Area%
1	6.480	50.392
2	8.167	49.608
Total		100.000

Enantioenriched

<Peak Table>

PDA Ch1 211nm

Peak#	Ret. Time	Area%
1	6.518	8.082
2	8.191	91.918
Total		100.000

Fig. S44: Chiral HPLC analysis for (*S*,*E*)-5-Fluoro-3-hydroxy-3-(3-phenylbut-2-en-1-yl)-1-tritylindolin-2-one (**17**), Chiralpak IA (90:10 hexane:IPA, flow rate 1mlmin[-1], 211 nm, 30 °C) t_R (*R*)-**17**: 8.7 min, t_R (*S*)-**17**: 10.7 min, 7:93 e.r.

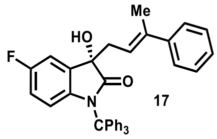

17

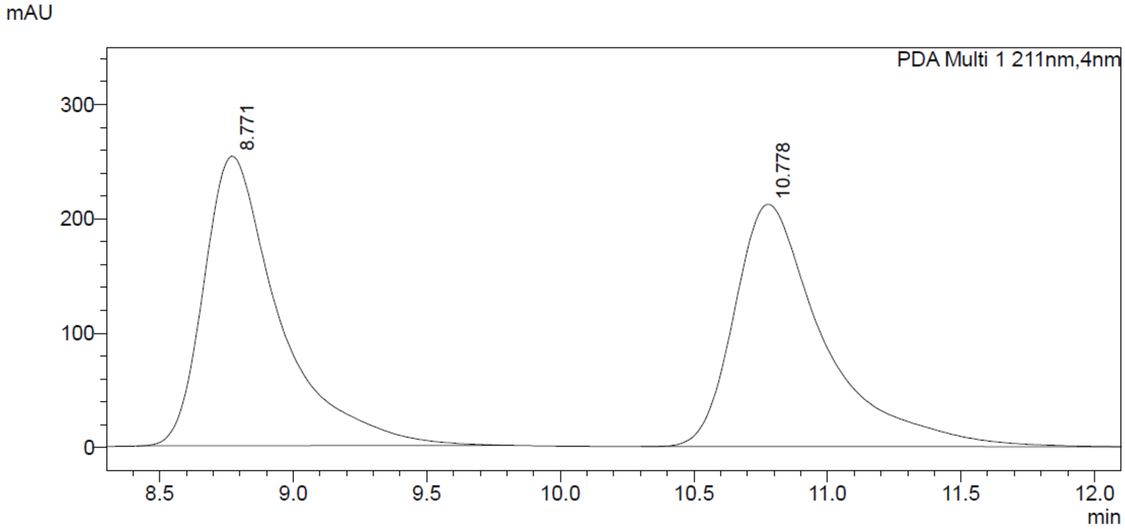

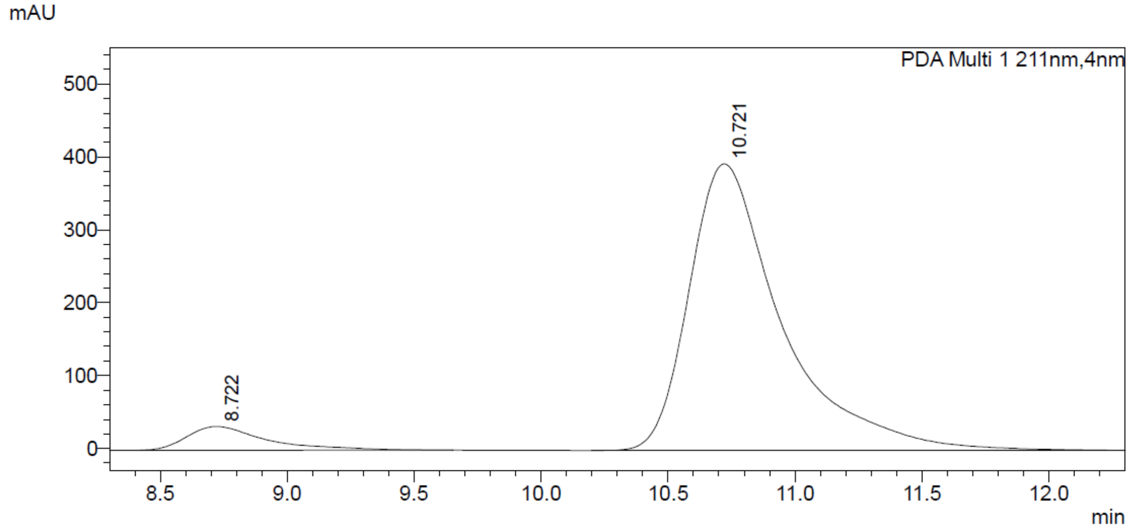

Racemic			Enantioenriched		
<Peak Table>			<Peak Table>		
PDA Ch1 211nm			PDA Ch1 211nm		
Peak#	Ret. Time	Area%	Peak#	Ret. Time	Area%
1	8.771	50.184	1	8.722	6.695
2	10.778	49.816	2	10.721	93.305
Total		100.000	Total		100.000

Fig. S45: Chiral HPLC analysis for (*S*,*E*)-5-Chloro-3-hydroxy-3-(3-phenylbut-2-en-1-yl)-1-tritylindolin-2-one (**33**), Chiralpak IA (90:10 hexane:IPA, flow rate 1mlmin^{-1}, 211 nm, 30 °C) t_R (*R*)-**33**: 8.9 min, t_R (*S*)-**33**: 10.9 min, 5:95 e.r.

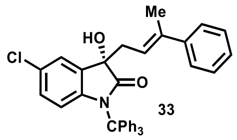

33

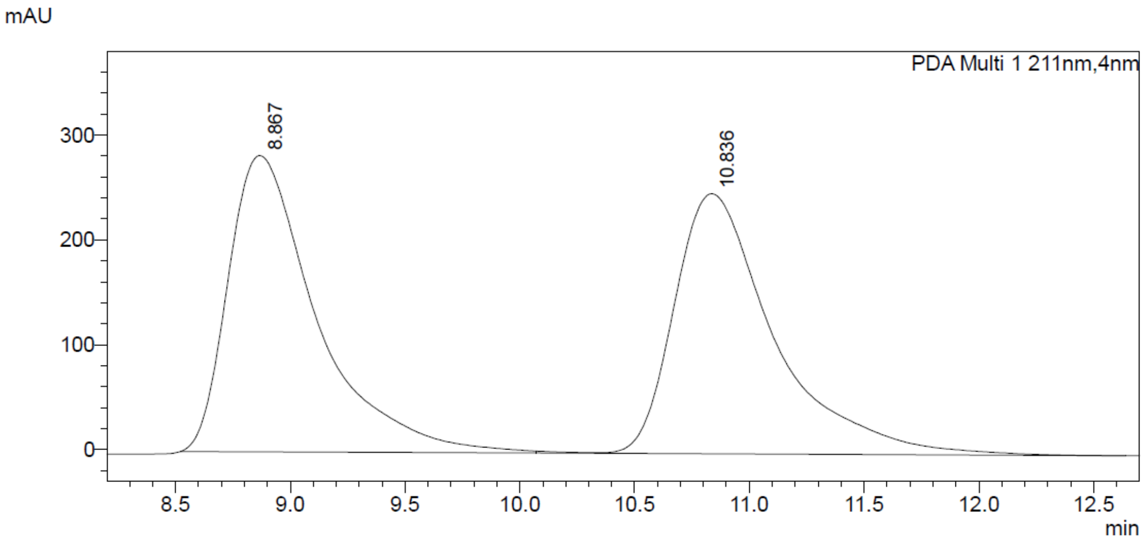

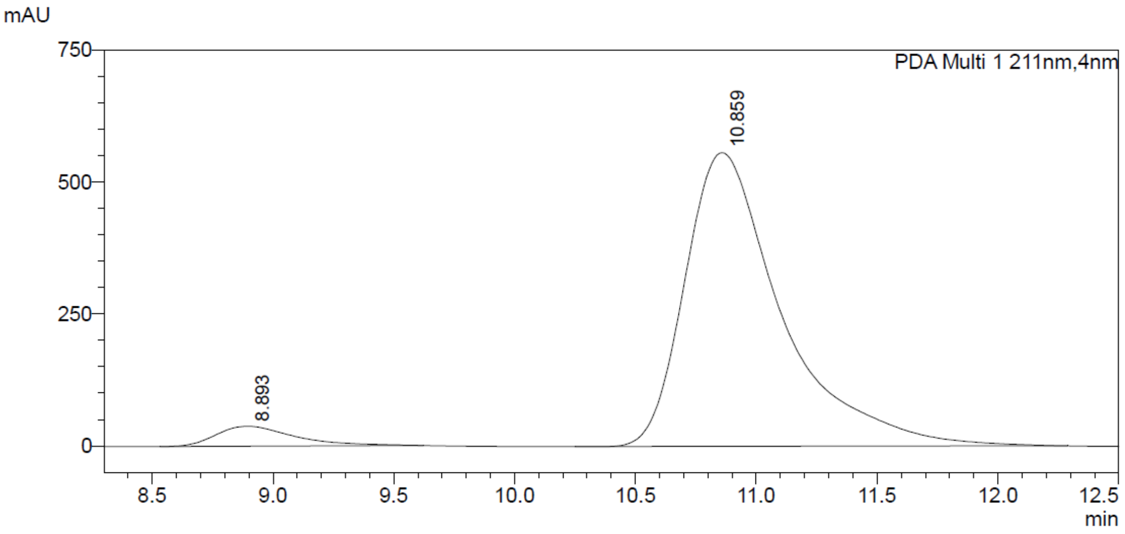

Racemic			Enantioenriched		
<Peak Table>			<Peak Table>		
PDA Ch1 211nm			PDA Ch1 211nm		
Peak#	Ret. Time	Area%	Peak#	Ret. Time	Area%
1	8.867	50.102	1	8.893	5.217
2	10.836	49.898	2	10.859	94.783
Total		100.000	Total		100.000

Fig. S46: Chiral HPLC analysis for (*S*,*E*)-3-Hydroxy-5-iodo-3-(3-phenylbut-2-en-1-yl)-1-tritylindolin-2-one (**34**), Chiralpak IA (90:10 hexane:IPA, flow rate 1mlmin^{-1}, 211 nm, 30 °C) t_R (*R*)-**34**: 9.8 min, t_R (*S*)-**34**: 11.8 min, 4:96 e.r.

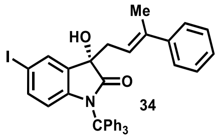

34

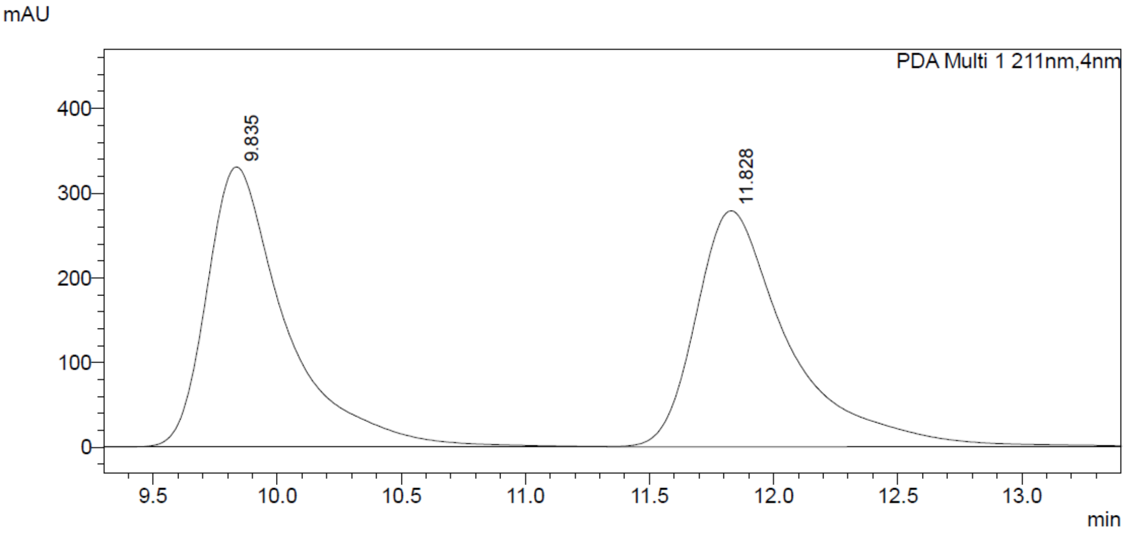

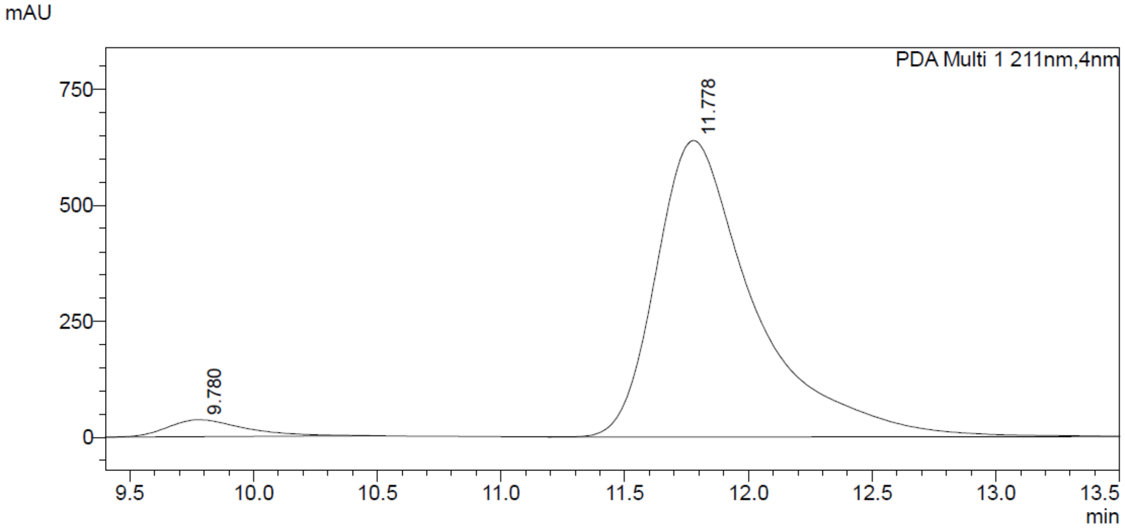

Racemic

<Peak Table>

PDA Ch1 211nm

Peak#	Ret. Time	Area%
1	9.835	50.078
2	11.828	49.922
Total		100.000

Enantioenriched

<Peak Table>

PDA Ch1 211nm

Peak#	Ret. Time	Area%
1	9.780	4.282
2	11.778	95.718
Total		100.000

Fig. S47: Chiral HPLC analysis for (*S*,*E*)-3-Hydroxy-5-nitro-3-(3-phenylbut-2-en-1-yl)-1-tritylindolin-2-one (**35**), Chiralpak IA (90:10 hexane:IPA, flow rate 1mlmin^{-1}, 211 nm, 30 °C) t_R (*R*)-**35**: 9.0 min, t_R (*S*)-**35**: 12.0 min, 7:93 e.r.

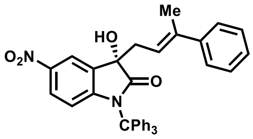

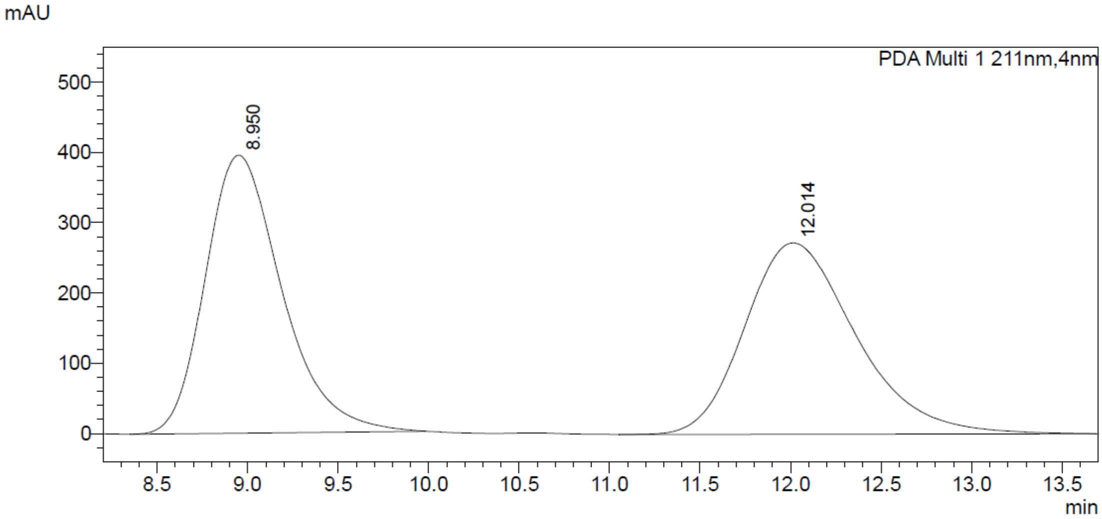

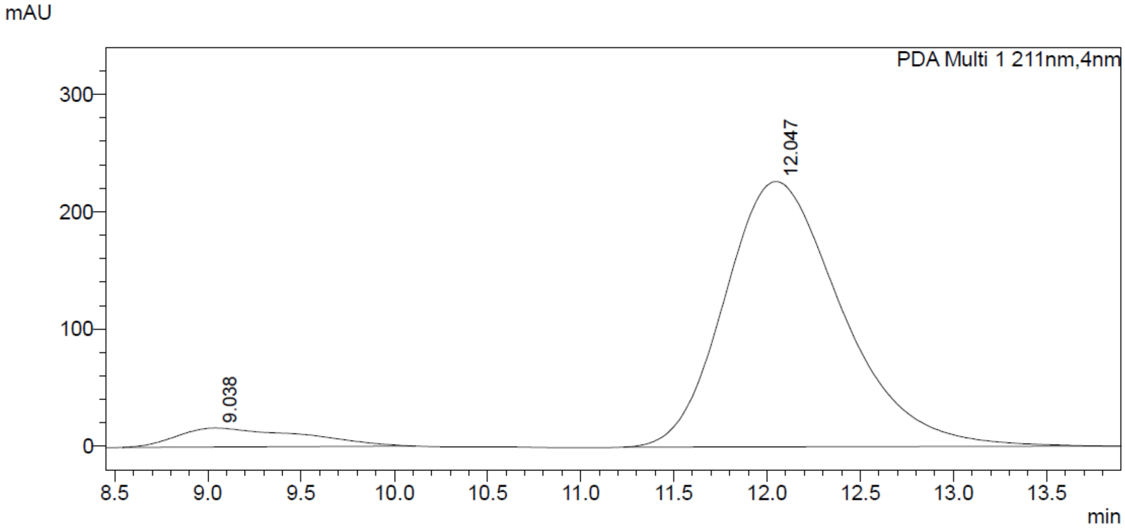

Racemic			Enantioenriched		
<Peak Table>			<Peak Table>		
PDA Ch1 211nm			PDA Ch1 211nm		
Peak#	Ret. Time	Area%	Peak#	Ret. Time	Area%
1	8.950	49.983	1	9.038	7.120
2	12.014	50.017	2	12.047	92.880
Total		100.000	Total		100.000

Fig. S48: Chiral HPLC analysis for (*S*,*E*)-3-Hydroxy-5-methyl-3-(3-phenylbut-2-en-1-yl)-1-tritylindolin-2-one (**36**), Chiralpak IA (90:10 hexane:IPA, flow rate 1mlmin^{-1}, 211 nm, 30 °C) t_R (*R*)-**36**: 9.1 min, t_R (*S*)-**36**: 11.1 min, 5:95 e.r.

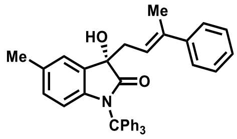

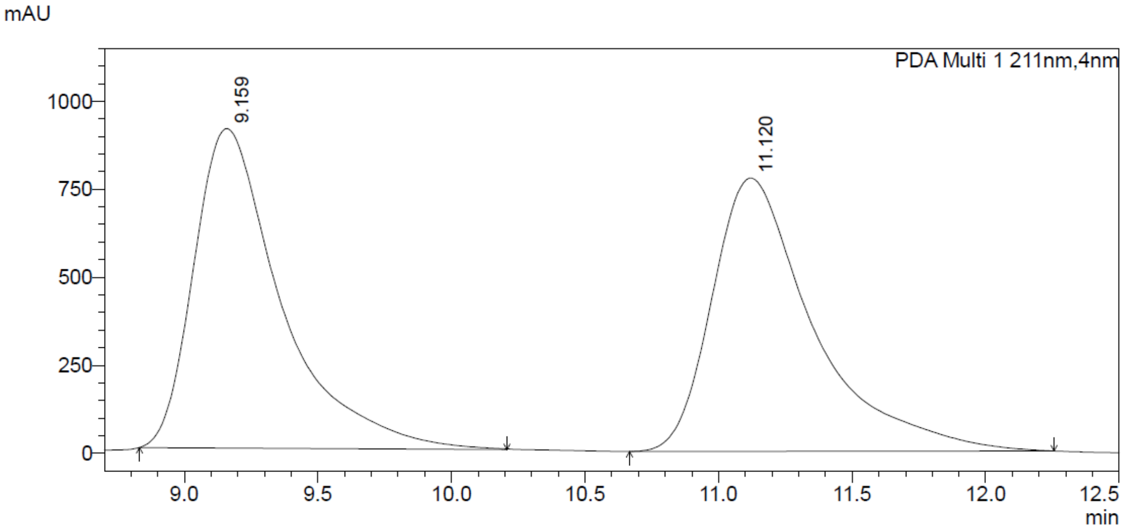

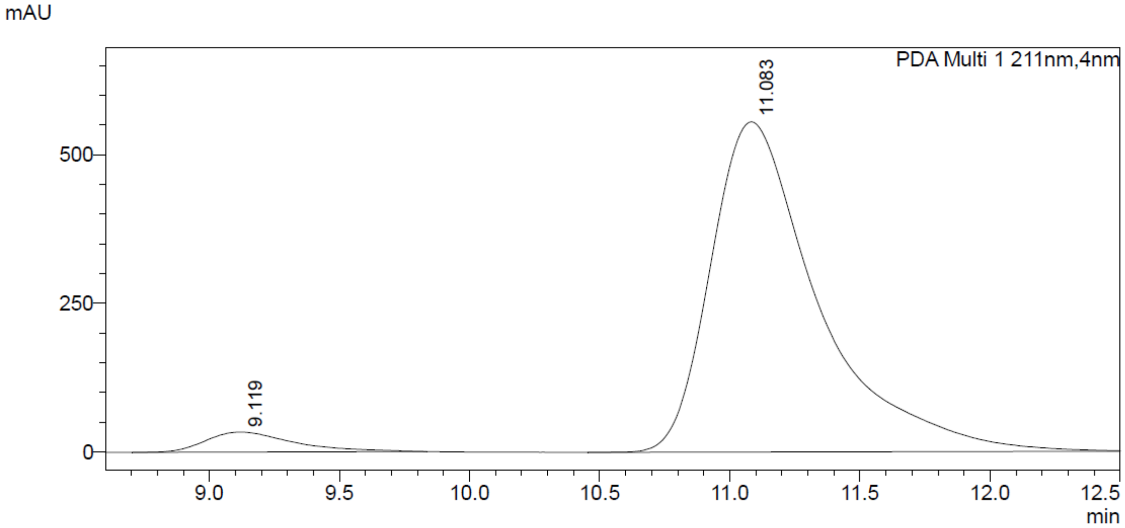

Racemic			Enantioenriched		
**<Peak Table>**			**<Peak Table>**		
PDA Ch1 211nm			PDA Ch1 211nm		
Peak#	Ret. Time	Area%	Peak#	Ret. Time	Area%
1	9.159	50.230	1	9.119	4.668
2	11.120	49.770	2	11.083	95.332
Total		100.000	Total		100.000

Fig. S49: Chiral HPLC analysis for (*S*,*E*)-3-Hydroxy-5-methoxy-3-(3-phenylbut-2-en-1-yl)-1-tritylindolin-2-one (**37**), Chiralpak IA (90:10 hexane:IPA, flow rate 1mlmin^{-1}, 211 nm, 30 °C) t_R (*R*)-**37**: 11.6 min, t_R (*S*)-**37**: 14.3 min, 5:95 e.r.

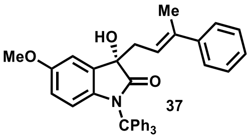

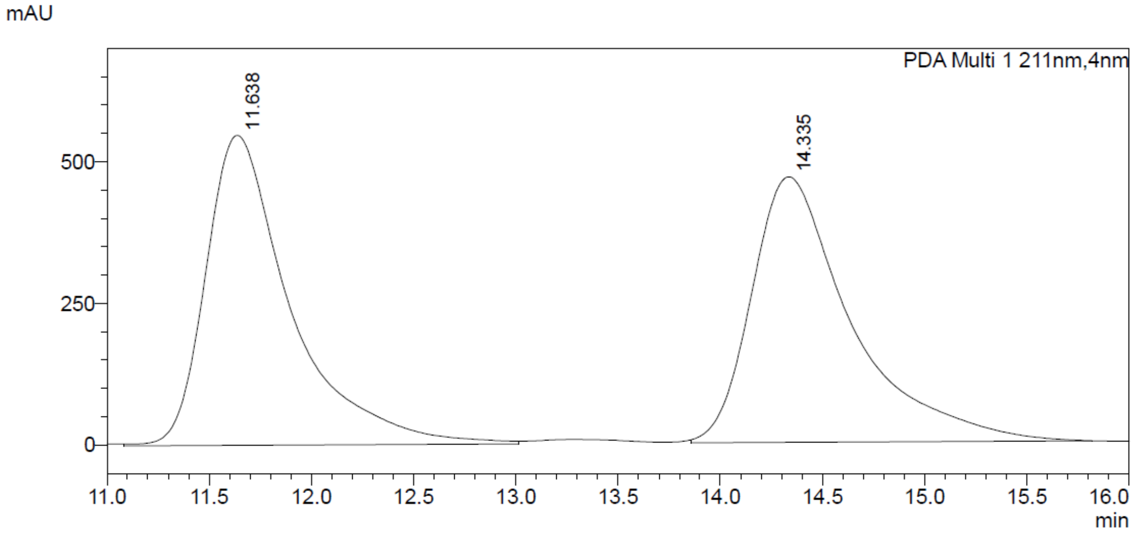

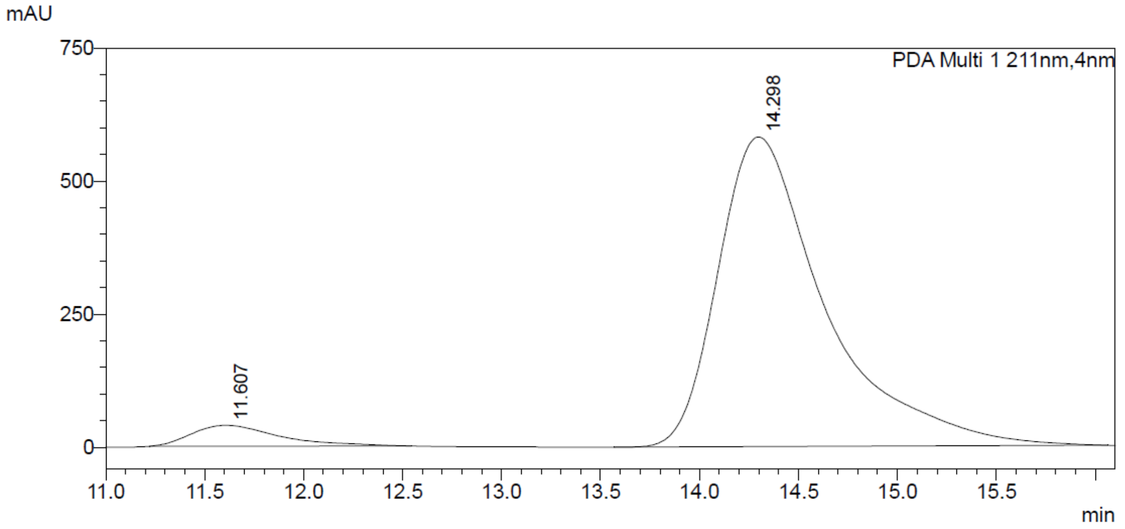

Racemic			Enantioenriched		
**<Peak Table>**			**<Peak Table>**		
PDA Ch1 211nm			PDA Ch1 211nm		
Peak#	Ret. Time	Area%	Peak#	Ret. Time	Area%
1	11.638	49.888	1	11.607	5.224
2	14.335	50.112	2	14.298	94.776
Total		100.000	Total		100.000

Fig. S50: Chiral HPLC analysis for (*S*,*E*)-6-Chloro-3-hydroxy-3-(3-phenylbut-2-en-1-yl)-1-tritylindolin-2-one (**38**), Chiralpak IA (90:10 hexane:IPA, flow rate 1mlmin^{-1}, 211 nm, 30 °C) t_R (*R*)-**38**: 9.4 min, t_R (*S*)-**38**: 11.5 min, 6:94 e.r.

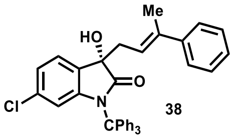

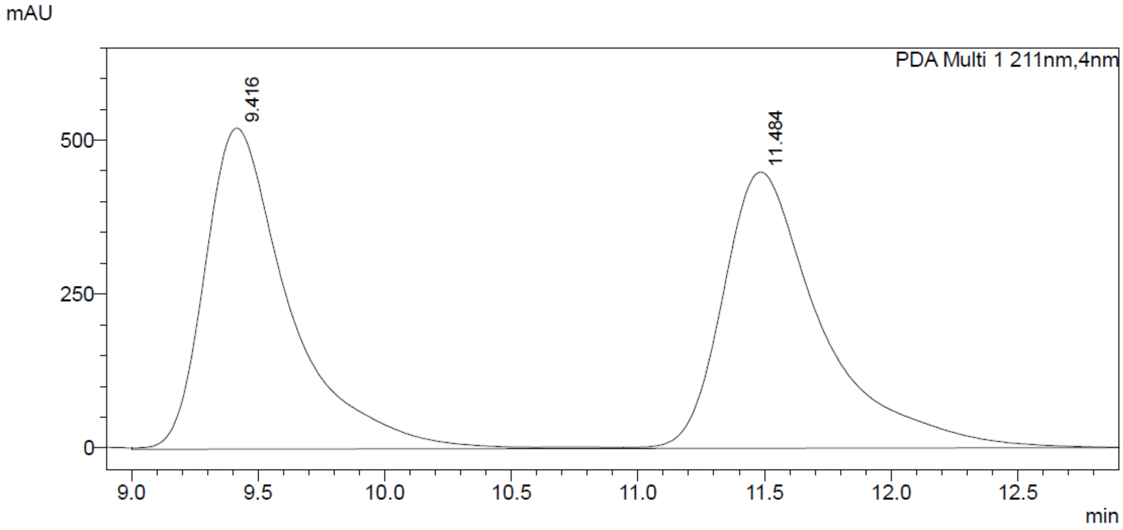

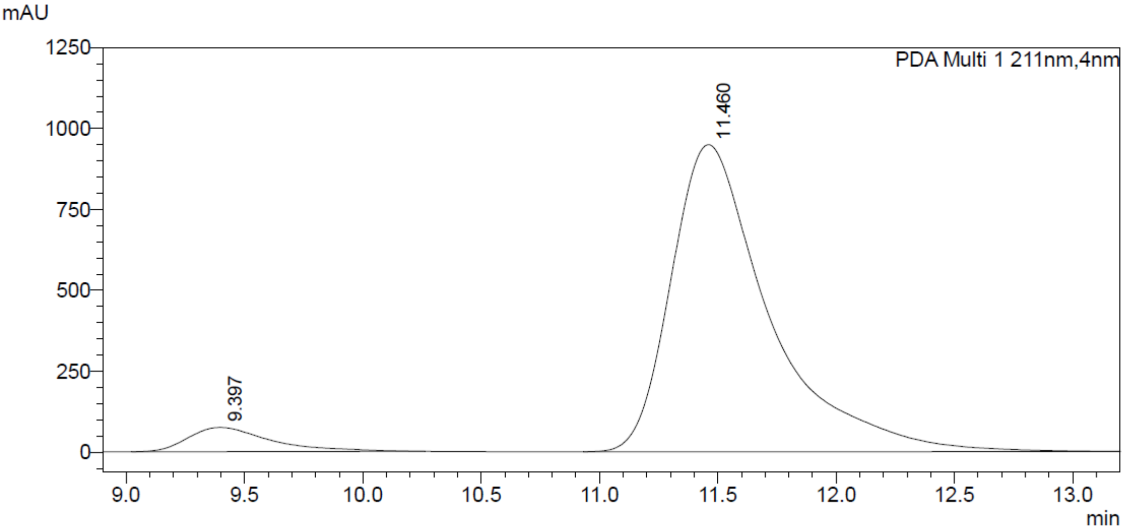

Racemic

<Peak Table>

PDA Ch1 211nm

Peak#	Ret. Time	Area%
1	9.416	49.913
2	11.484	50.087
Total		100.000

Enantioenriched

<Peak Table>

PDA Ch1 211nm

Peak#	Ret. Time	Area%
1	9.397	6.207
2	11.460	93.793
Total		100.000

Fig. S51: Chiral HPLC analysis for (*S*,*E*)-6-Bromo-3-hydroxy-3-(3-phenylbut-2-en-1-yl)-1-tritylindolin-2-one (**39**), Chiralpak IA (90:10 hexane:IPA, flow rate 1mlmin^{-1}, 211 nm, 30 °C) t_R (*R*)-**39**: 9.8 min, t_R (*S*)-**39**: 11.7 min, 6:94 e.r.

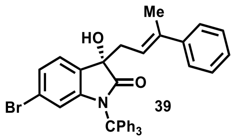

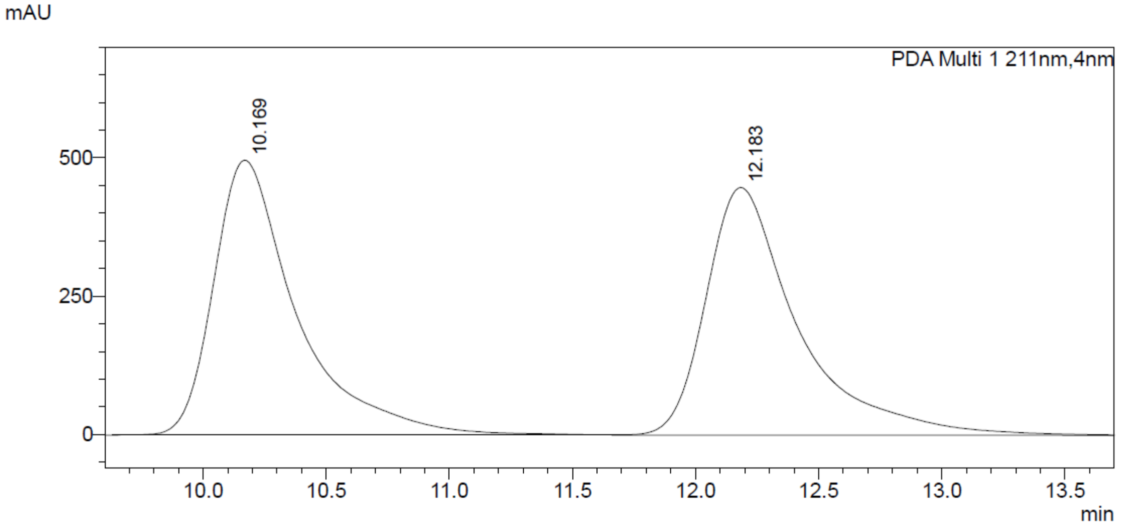

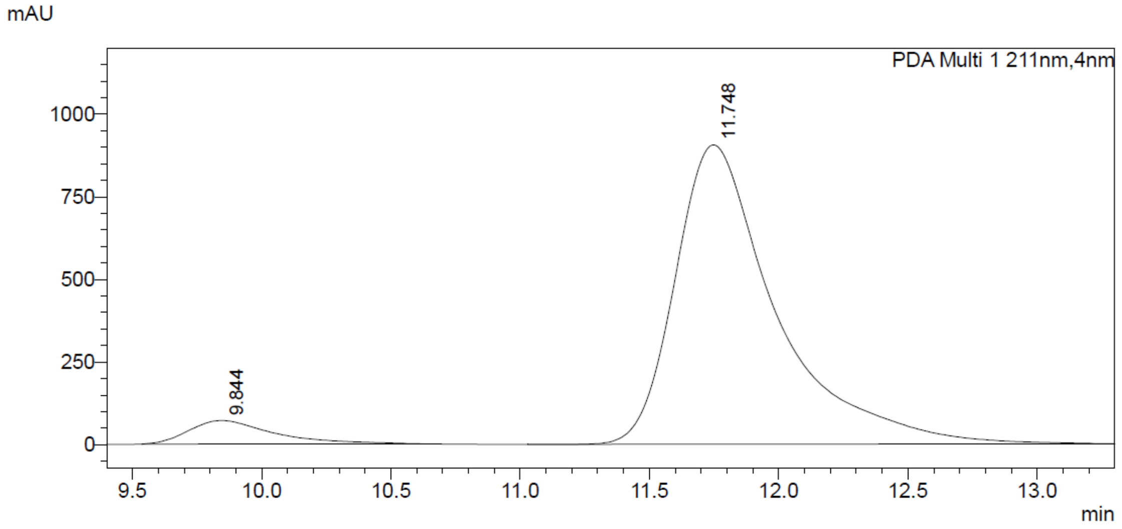

Racemic	Enantioenriched

Racemic

<Peak Table>

PDA Ch1 211nm

Peak#	Ret. Time	Area%
1	9.416	49.913
2	11.484	50.087
Total		100.000

Enantioenriched

<Peak Table>

PDA Ch1 211nm

Peak#	Ret. Time	Area%
1	9.844	6.154
2	11.748	93.846
Total		100.000

Fig. S52: Chiral HPLC analysis for (*S*,*E*)-1-Benzyl-7-chloro-3-hydroxy-3-(3-phenylbut-2-en-1-yl)indolin-2-one (**40**), Chiralpak IA (90:10 hexane:IPA, flow rate 1mlmin^{-1}, 211 nm, 30 °C) t_R (*R*)-**40**: 11.0 min, t_R (*S*)-**40**: 15.4 min, 27:73 e.r.

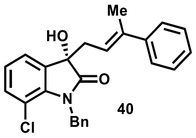

40

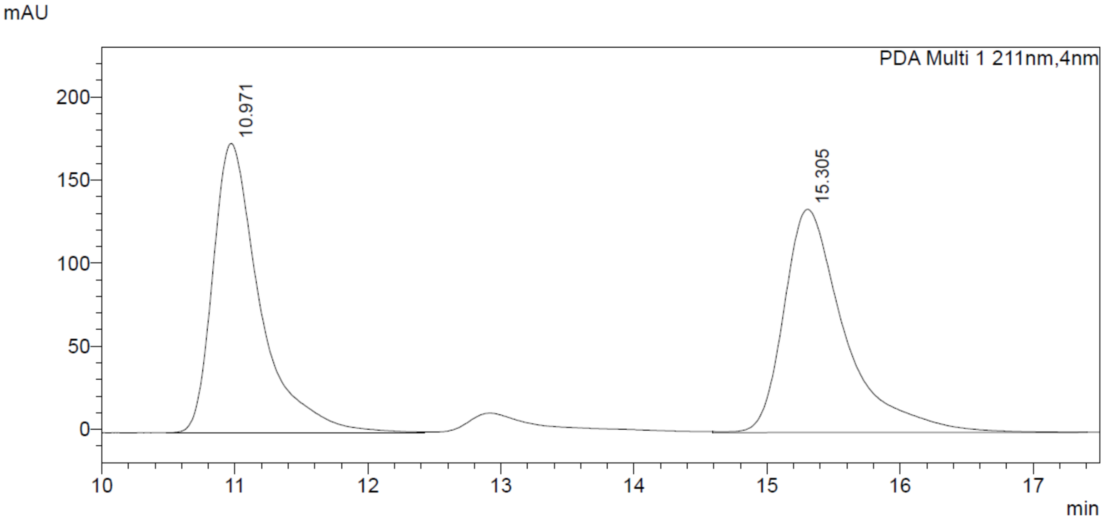

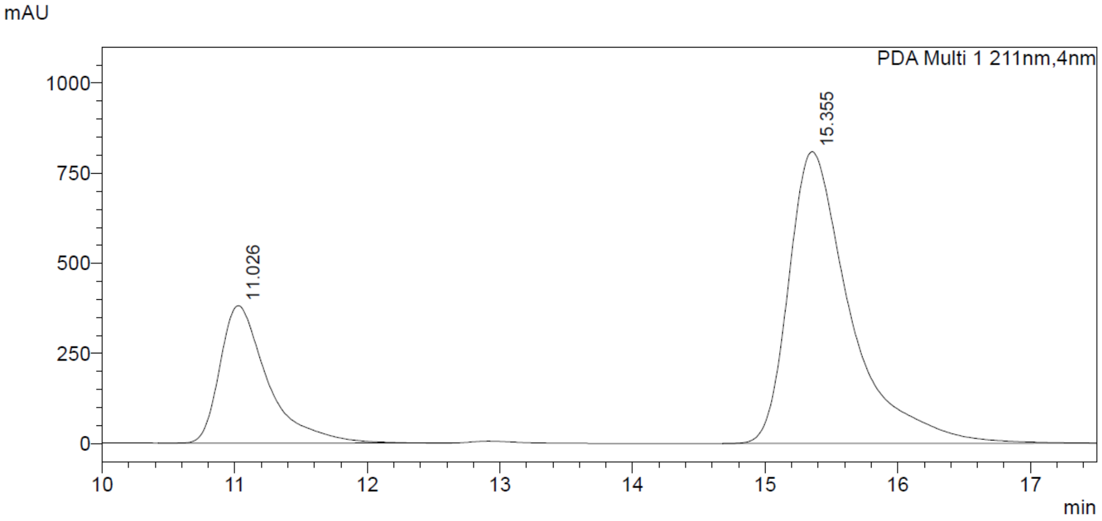

Racemic			Enantioenriched		
**<Peak Table>**			**<Peak Table>**		
PDA Ch1 211nm			PDA Ch1 211nm		
Peak#	Ret. Time	Area%	Peak#	Ret. Time	Area%
1	10.971	49.975	1	11.026	26.656
2	15.305	50.025	2	15.355	73.344
Total		100.000	Total		100.000

Fig. S53: Chiral HPLC analysis for 3-Hydroxy-3-(2-phenylbut-3-en-2-yl)-1-tritylindolin-2-one (**14**), Chiralpak IB (98:2 hexane:IPA, flow rate 1 ml·min^{-1}, 211 nm, 30 °C) major diastereomer: t_R (2``*R*,3*R*)-**14**: 8.5min, $t_R$ (2``*S*,3*S*)-**14**: 10.6min, 1:99 e.r.; minor diastereomer: t_R (2``*S*,3*R*)-**14**: 9.5min, $t_R$ (2``*R*,3*S*)-**14**: 11.6min, 1:99 e.r.

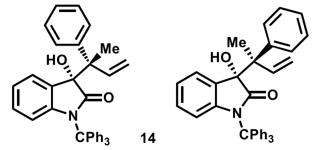

14

major diastereomer *minor diastereomer*

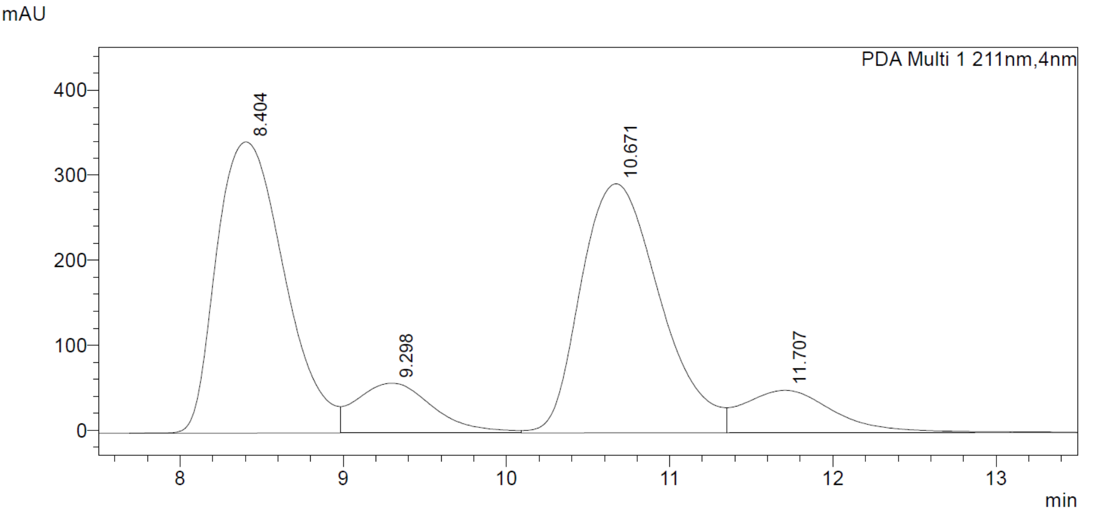

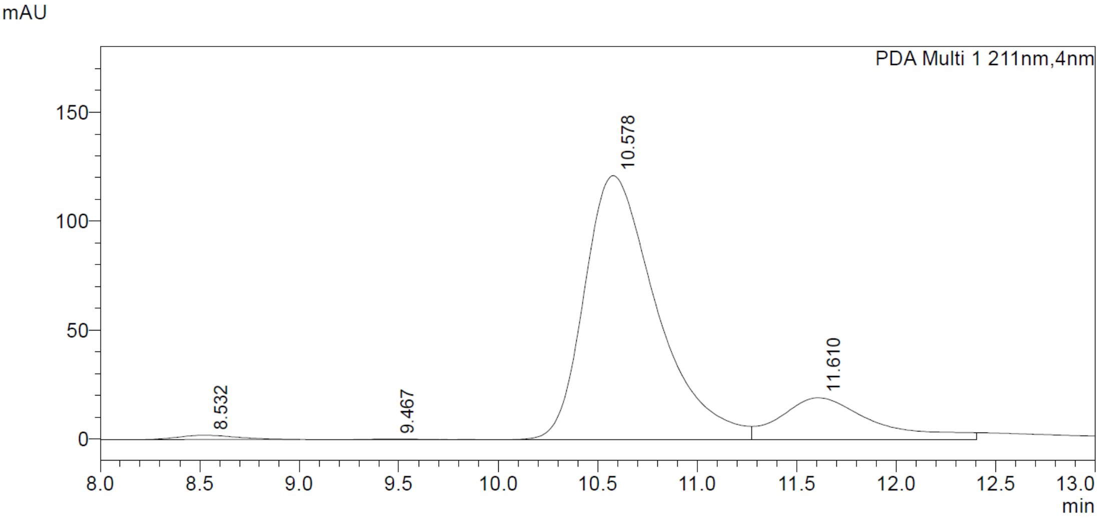

Racemic

<Peak Table>

PDA Ch1 211nm

Peak#	Ret. Time	Area%
1	8.404	41.841
2	9.298	8.114
3	10.671	41.848
4	11.707	8.196
Total		100.000

Enantioenriched

<Peak Table>

PDA Ch1 211nm

Peak#	Ret. Time	Area%
1	8.532	1.096
2	9.467	0.126
3	10.578	82.093
4	11.610	16.686
Total		100.000

Fig. S54: Chiral HPLC analysis for 3-hydroxy-3-(2-phenylbut-3-en-2-yl)indolin-2-one (**S24**), Chiralpak ID (95:5 hexane:IPA, flow rate 1 ml·min^{-1}, 211 nm, 30 °C), major diastereomer: t_R (2``*R*,3*R*)-**S24**: 20.5min, $t_R$ (2``*S*,3*S*)-**S24**: 23.8min, 2:98 e.r.; minor diastereomer: t_R (2``*S*,3*R*)-**S24**: 21.1min, $t_R$ (2``*R*,3*S*)-**S24**: 25.9min, 1:99 e.r.;

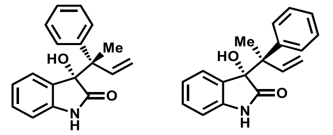

major diastereomer *minor diastereomer*

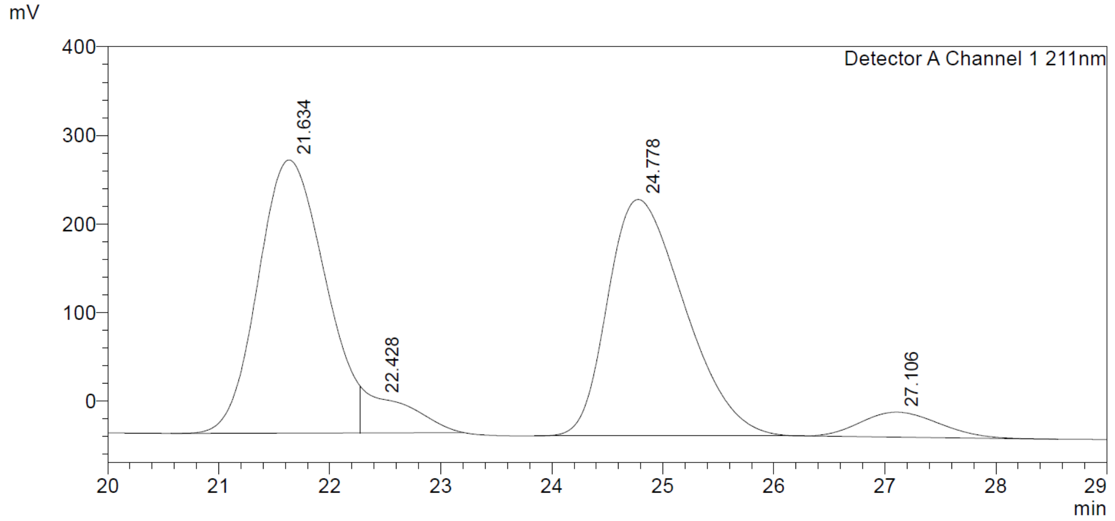

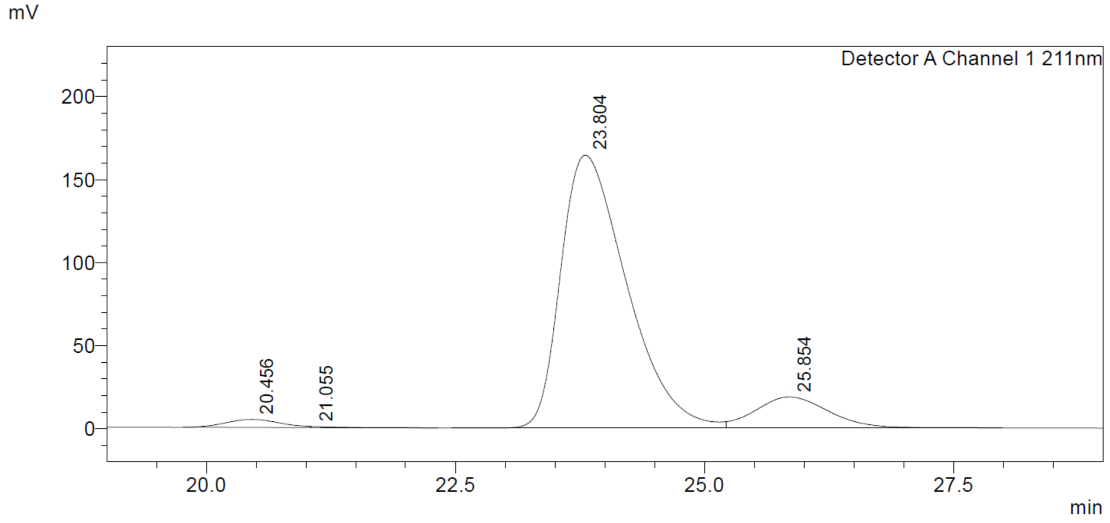

Racemic	Enantioenriched
<Peak Table>	<Peak Table>
Detector A Channel 1 211nm	Detector A Channel 1 211nm

Racemic:

Peak#	Ret. Time	Area%
1	21.634	45.115
2	22.428	4.862
3	24.778	45.129
4	27.106	4.893
Total		100.000

Enantioenriched:

Peak#	Ret. Time	Area%
1	20.456	2.092
2	21.055	0.124
3	23.804	86.867
4	25.854	10.918
Total		100.000

Fig. S55: Chiral HPLC analysis for 3-hydroxy-3-(2-phenylbut-3-en-2-yl)indolin-2-one (**S24**), Chiralpak AD-H (80:20 hexane:IPA, flow rate 1ml·min^{-1}, 211 nm, 30 °C) t_R (*S*)-**128**: 7.1min, t_R (*R*)-**128**: 9.2min, 96:4 e.r.

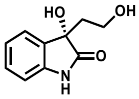

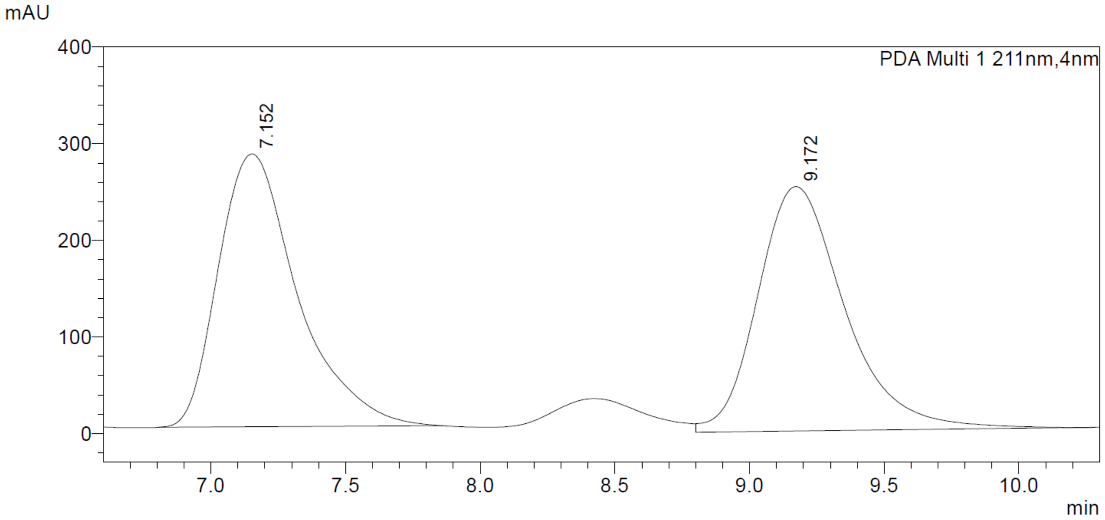

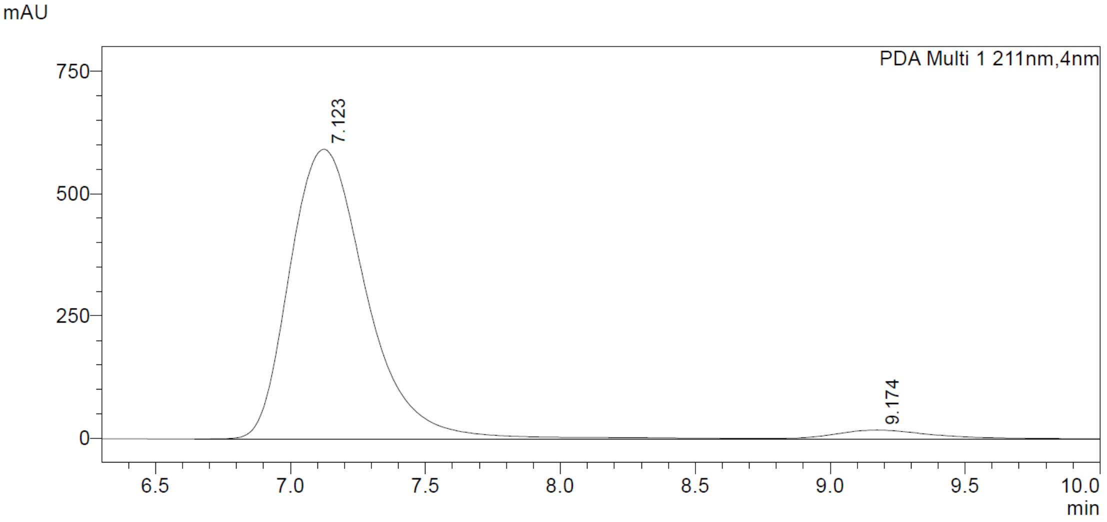

Racemic

<Peak Table>

PDA Ch1 211nm

Peak#	Ret. Time	Area%
1	7.152	50.265
2	9.172	49.735
Total		100.000

Enantioenriched

<Peak Table>

PDA Ch1 211nm

Peak#	Ret. Time	Area%
1	7.123	96.260
2	9.174	3.740
Total		100.000

Fig. S56: Chiral HPLC analysis for 5-phenyl-4,5-dihydro-3H-spiro[furan-2,3'-indolin]-2'-one (**S25**), Chiralcel OD-H (90:10 hexane:IPA, flow rate 1 ml·min^{-1}, 211 nm, 30 °C) t_R (2’*R*,3*S*)-**S25**: 11.7 min, t_R (2’*S*,3*R*)-**S25**: 15.5min, 92:8 e.r.

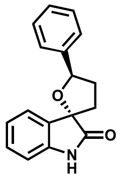

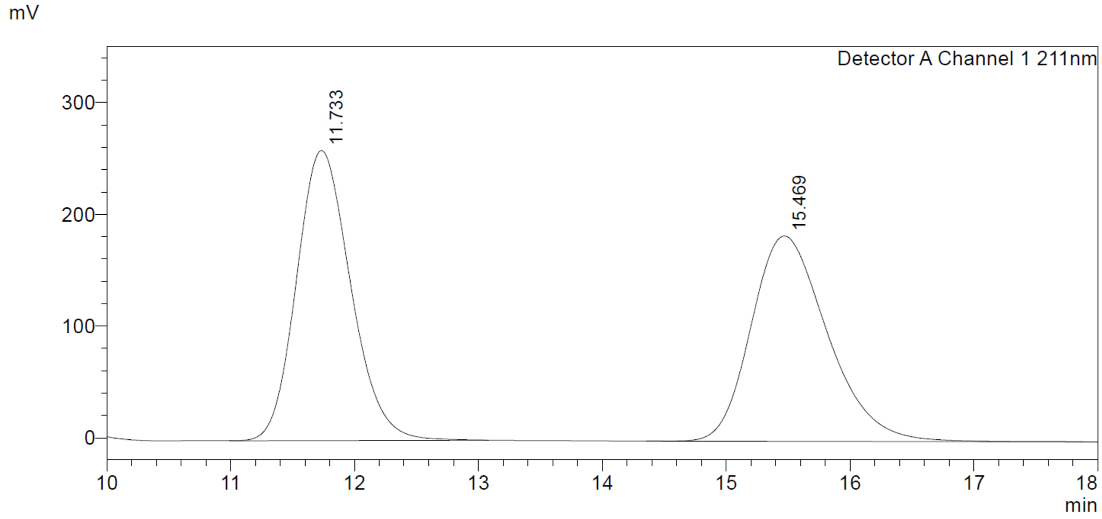

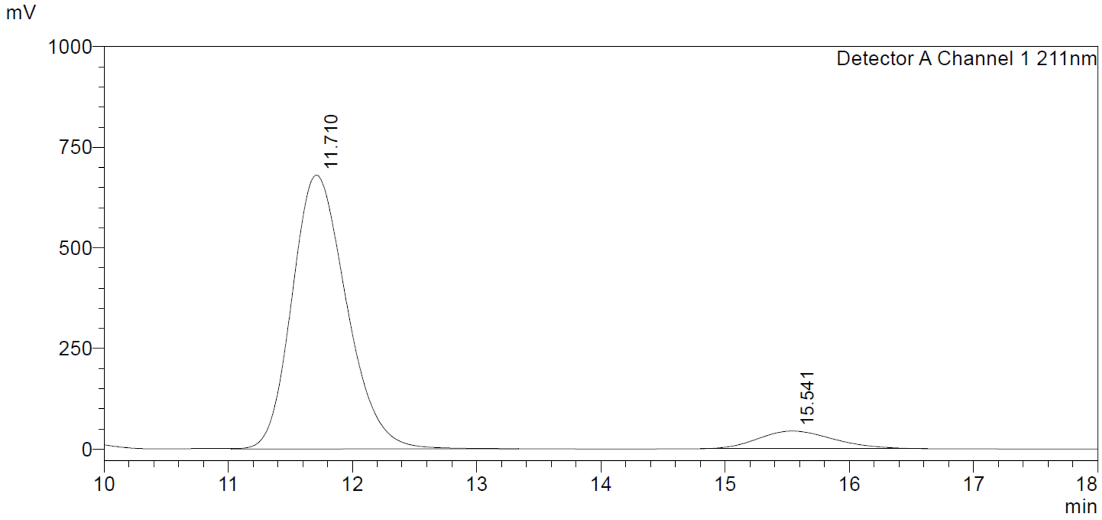

Racemic

<Peak Table>

Detector A Channel 1 211nm

Peak#	Ret. Time	Area%
1	11.733	50.112
2	15.469	49.888
Total		100.000

Enantioenriched

<Peak Table>

Detector A Channel 1 211nm

Peak#	Ret. Time	Area%
1	11.710	91.812
2	15.541	8.188
Total		100.000

Fig. S57: Chiral HPLC analysis for 3-(1-fluoro-1-phenylallyl)-3-hydroxy-1-tritylindolin-2-one (**42**), Chiralpak AD-H (90:10 hexane:IPA, flow rate 1ml·min^{-1}, 211 nm, 30 °C), major diastereomer: t_R (3*R*,1``*R*)-**42**: 7.56min, $t_R$ (3*S*,1``*S*)-**42**: 12.03min, 2:98 e.r.; minor diastereomer: t_R (3*S*,1``*R*)-**42**: 9.37min, $t_R$ (3*R*,1``*S*)-**42**: 10.11min, 91:9 e.r.

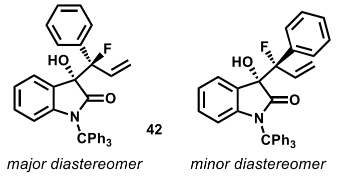

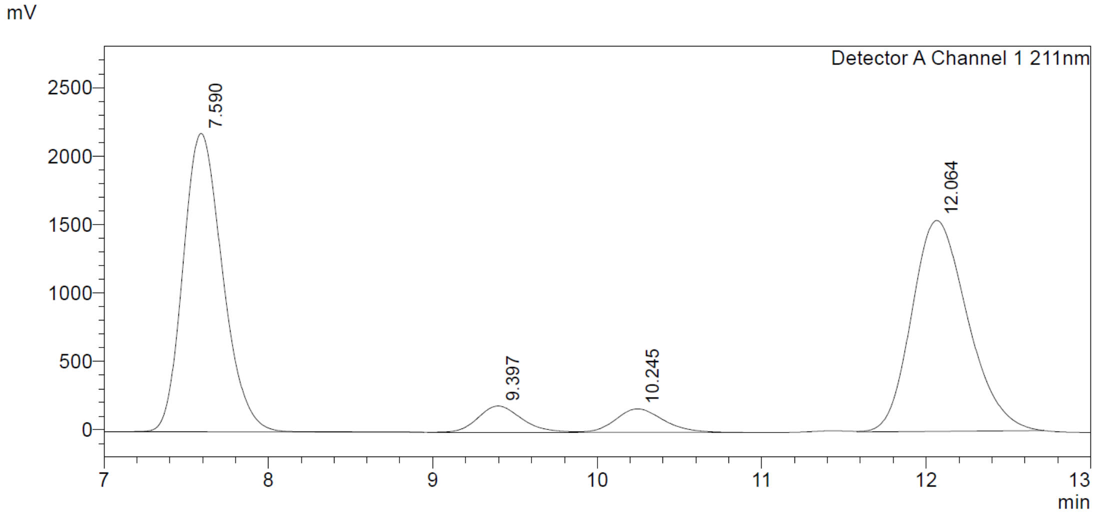

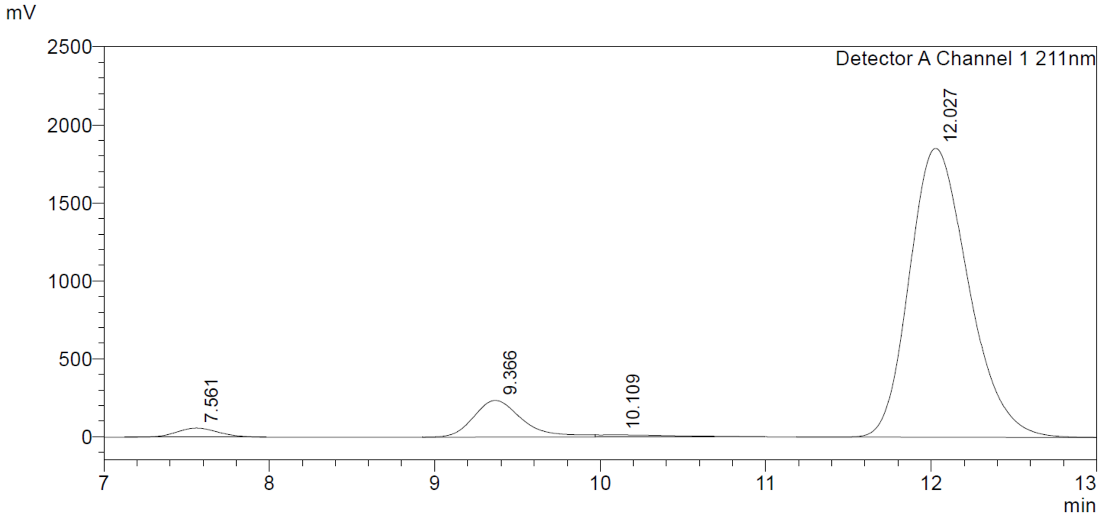

Racemic

<Peak Table>

Detector A Channel 1 211nm

Peak#	Ret. Time	Area%
1	7.590	45.643
2	9.397	4.390
3	10.245	4.327
4	12.064	45.641
Total		100.000

Enantioenriched

<Peak Table>

Detector A Channel 1 211nm

Peak#	Ret. Time	Area%
1	7.561	1.981
2	9.366	9.246
3	10.109	0.861
4	12.027	87.912
Total		100.000

Fig. S58: Chiral HPLC analysis for 3-hydroxy-3-(1-phenylallyl)-1-tritylindolin-2-one (**44**), Chiralpak AD-H (90:10 hexane/IPA, flow rate 1 mL·min^{-1}, 211 nm, 30 °C), major diastereomer: t_R (3*R*,1``*R*)-**44**: 8.5 min, $t_R$ (3*S*,1``*S*)-**44**: 27.5 min, 2:98 e.r.; minor diastereomer: t_R (3*R*,1``*S*)-**44**: 10.0 min, $t_R$ (3*S*,1``*R*)-**44**: 17.1 min, 3:97 e.r.

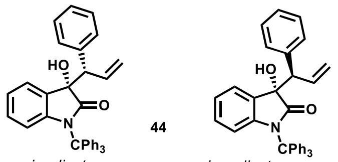

major diastereomer *minor diastereomer*

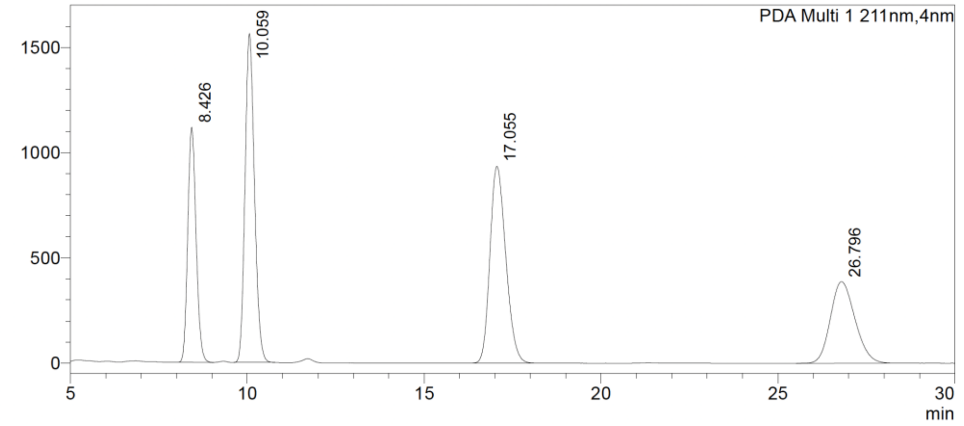

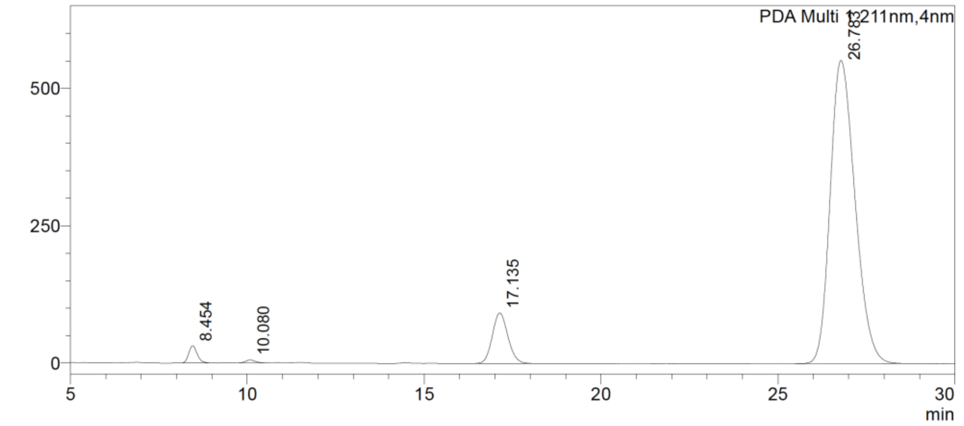

Racemic

PDA Ch1 211nm

Peak#	Ret. Time	Area%
1	8.426	19.279
2	10.059	30.319
3	17.055	30.698
4	26.796	19.704
Total		100.000

Enantioenriched

PDA Ch1 211nm

Peak#	Ret. Time	Area%
1	8.454	1.735
2	10.080	0.310
3	17.135	9.234
4	26.783	88.721
Total		100.000

Fig. S59: Chiral HPLC analysis for (*S*,*Z*)-3-(3-fluoro-3-phenylallyl)-3-hydroxy-1-tritylindolin-2-one (**46**), Chiralpak AD-H (90:10 hexane:IPA, flow rate 1 ml·min^{-1}, 211 nm, 30 °C) t_R (*R*)-**46**: 13.2 min, t_R (*S*)-**46**: 14.7 min, 5:95 e.r.

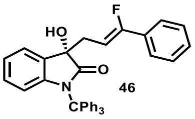

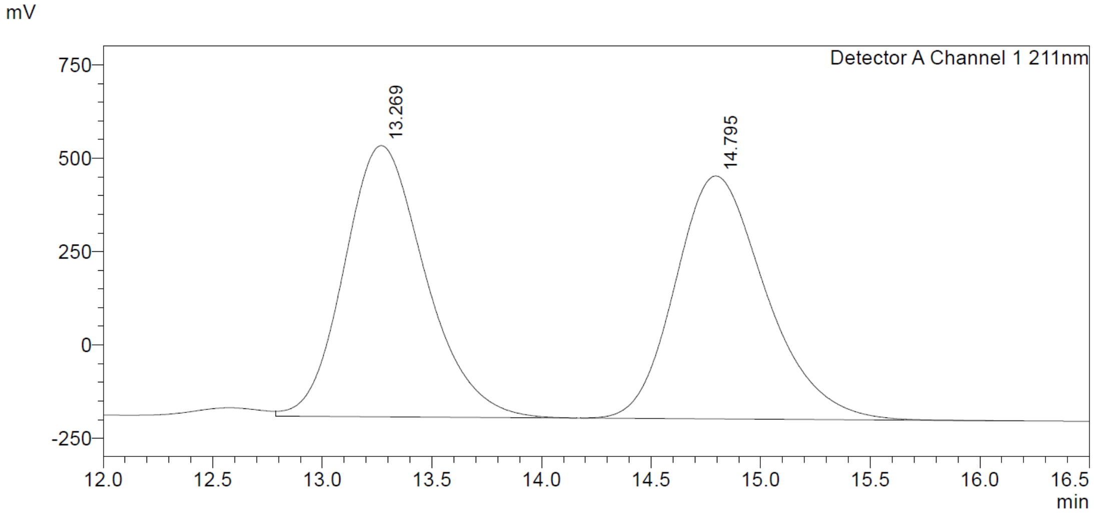

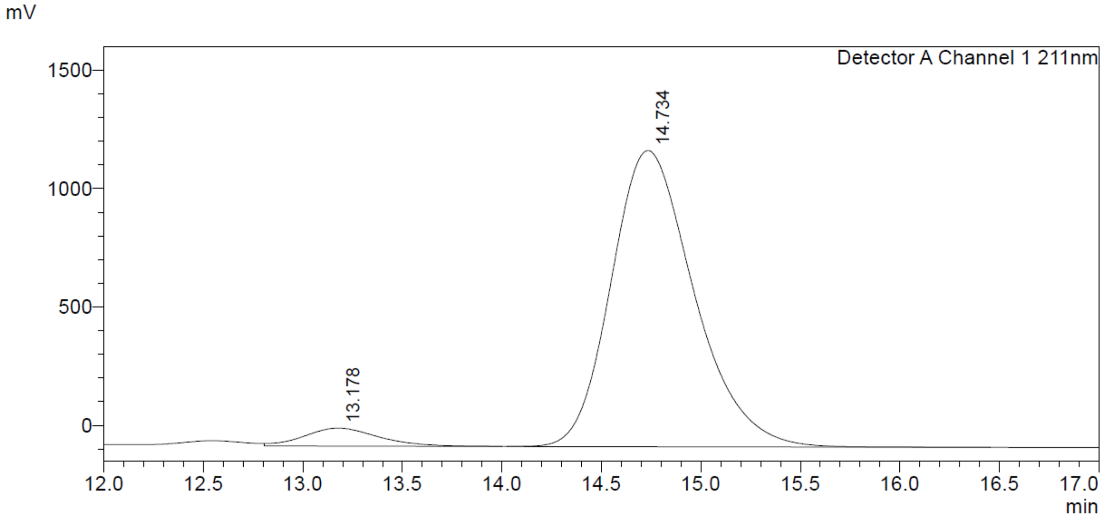

Racemic

<Peak Table>

Detector A Channel 1 211nm

Peak#	Ret. Time	Area%
1	13.269	49.984
2	14.795	50.016
Total		100.000

Enantioenriched

<Peak Table>

Detector A Channel 1 211nm

Peak#	Ret. Time	Area%
1	13.178	5.109
2	14.734	94.891
Total		100.000

Fig. S60: Chiral HPLC analysis for (*S*,*Z*)-3-(3-Fluoro-3-(p-tolyl)allyl)-3-hydroxy-1-tritylindolin-2-one (**47**), Chiralpak IB (95:5 hexane:IPA, flow rate 1 ml·min^{-1}, 211 nm, 30 °C) t_R (*R*)-**47**: 9.8 min, t_R (*S*)-**47**: 14.9 min, 7:93 e.r.

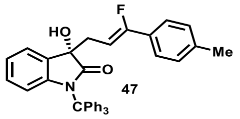

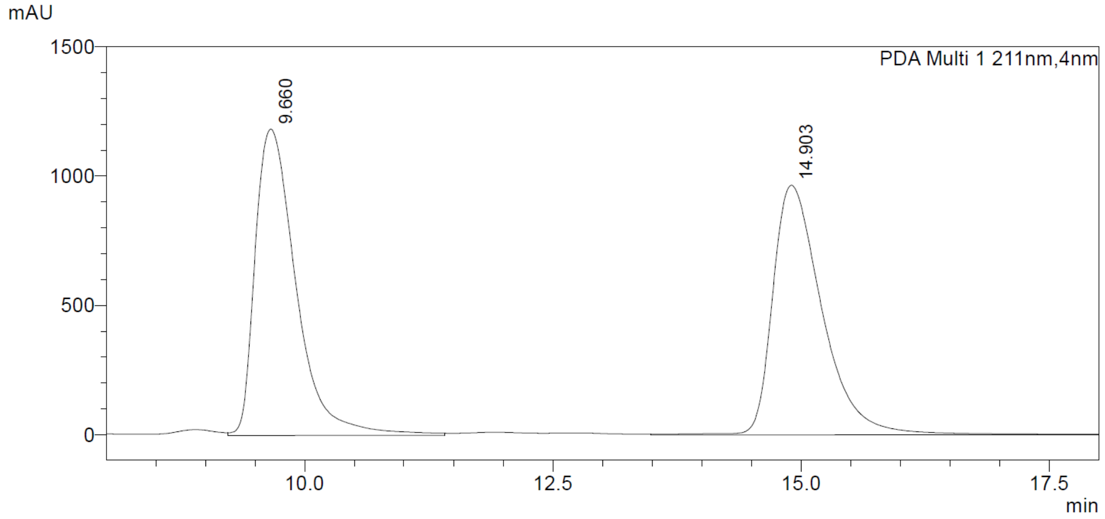

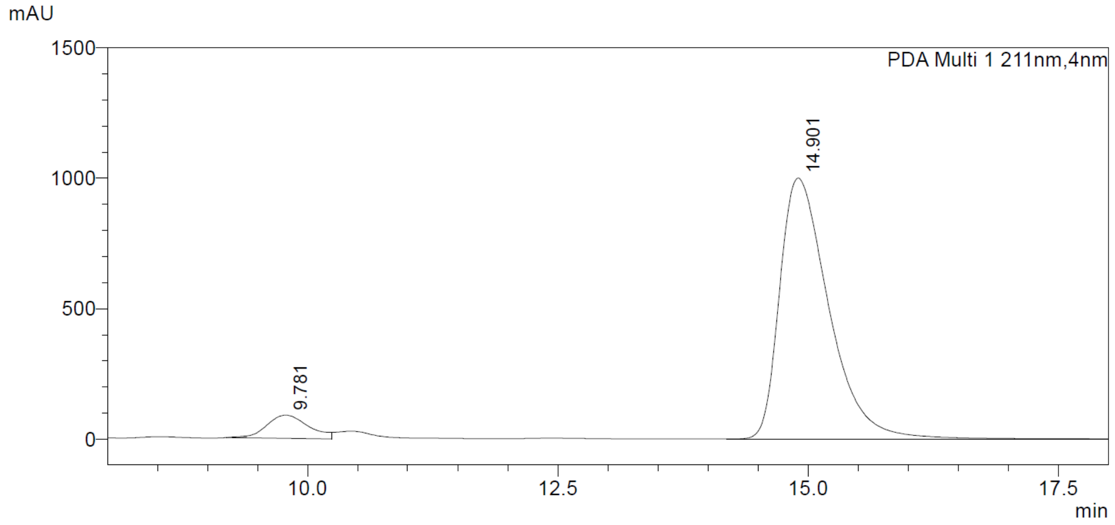

Racemic

<Peak Table>

PDA Ch1 211nm

Peak#	Ret. Time	Area%
1	9.660	50.283
2	14.903	49.717
Total		100.000

Enantioenriched

<Peak Table>

PDA Ch1 211nm

Peak#	Ret. Time	Area%
1	9.781	7.068
2	14.901	92.932
Total		100.000

Fig. S61: Chiral HPLC analysis for (*S*,*Z*)-3-[3-Fluoro-3-(4-methoxyphenyl)allyl]-3-hydroxy-1-tritylindolin-2-one (**48**), Chiralpak IB (90:10 hexane:IPA, flow rate 1 ml·min^{-1}, 211 nm, 30 °C) t_R (*R*)-**48**: 8.6 min, t_R (*S*)-**48**: 12.2 min, 7:93 e.r.

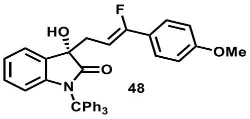

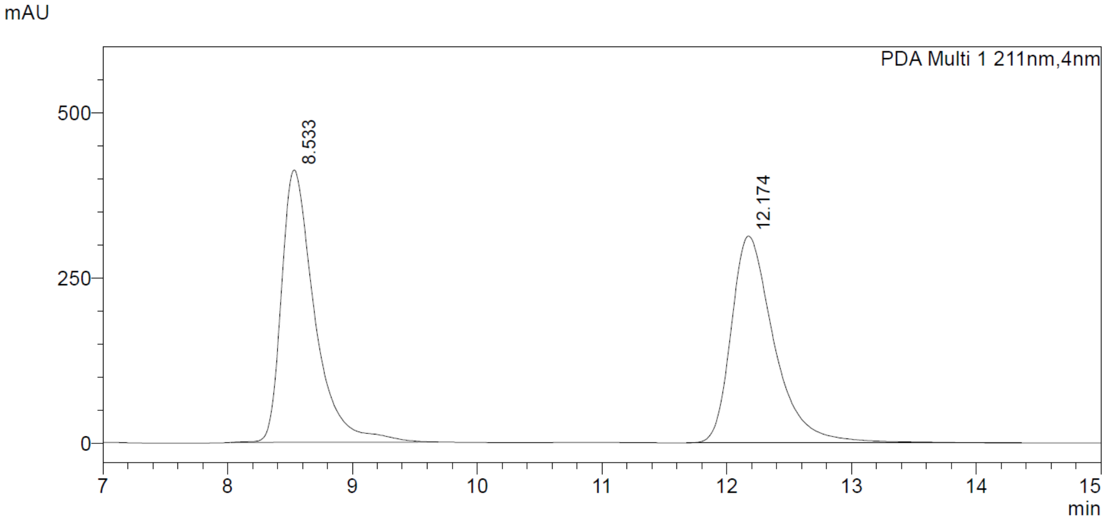

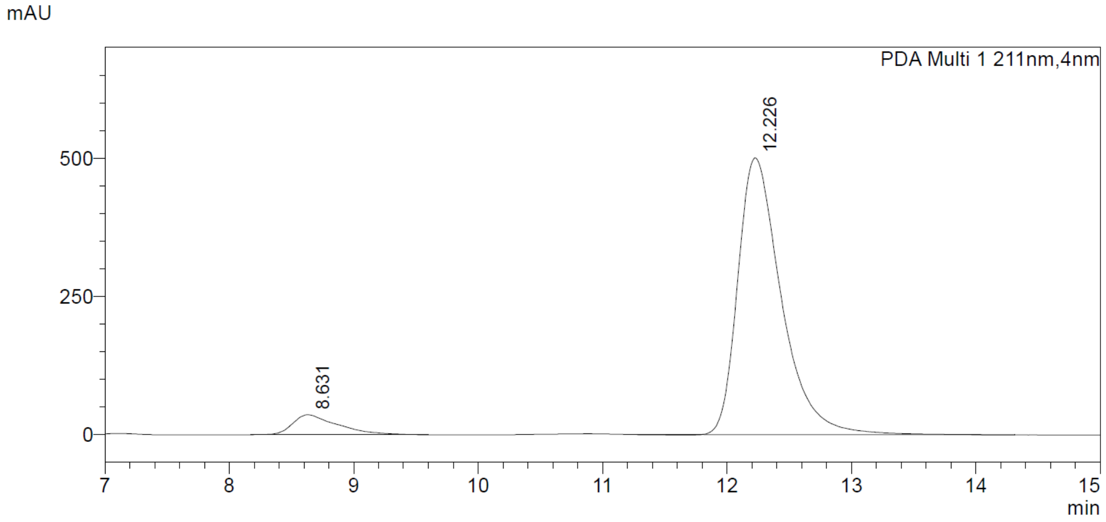

Racemic

<Peak Table>

PDA Ch1 211nm

Peak#	Ret. Time	Area%
1	8.533	50.346
2	12.174	49.654
Total		100.000

Enantioenriched

<Peak Table>

PDA Ch1 211nm

Peak#	Ret. Time	Area%
1	8.631	6.901
2	12.226	93.099
Total		100.000

Fig. S62: Chiral HPLC analysis for (*S*,*Z*)-3-{3-Fluoro-3-[4-(trifluoromethyl)phenyl]allyl}-3-hydroxy-1-tritylindolin-2-one (**49**), Chiralpak IB (95:5 hexane:IPA, flow rate 1 ml·min^{-1}, 211 nm, 30 °C) t_R (*R*)-**49**: 10.6 min, t_R (*S*)-**49**: 18.5 min, 9:91 e.r.

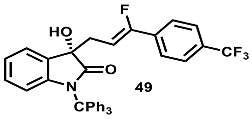

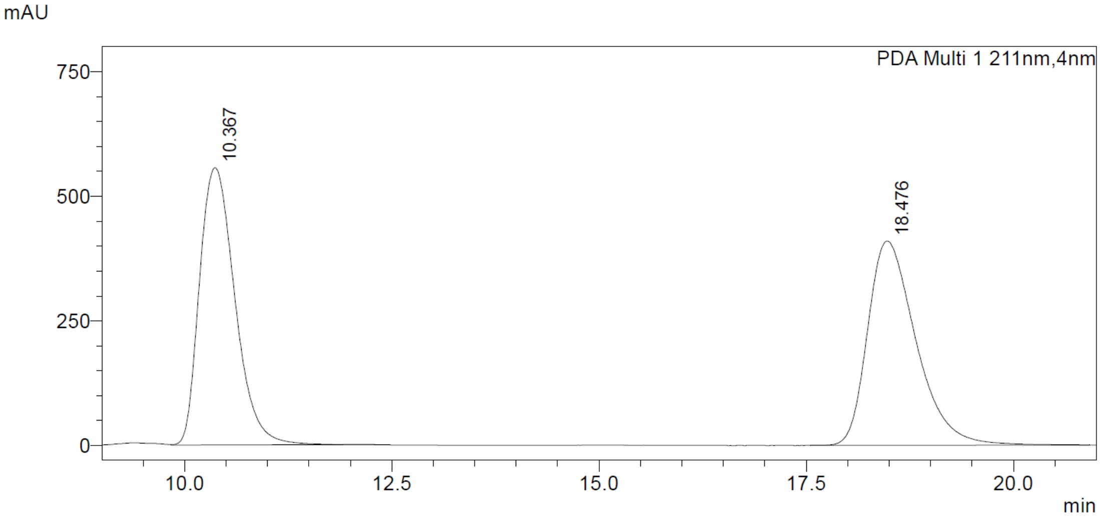

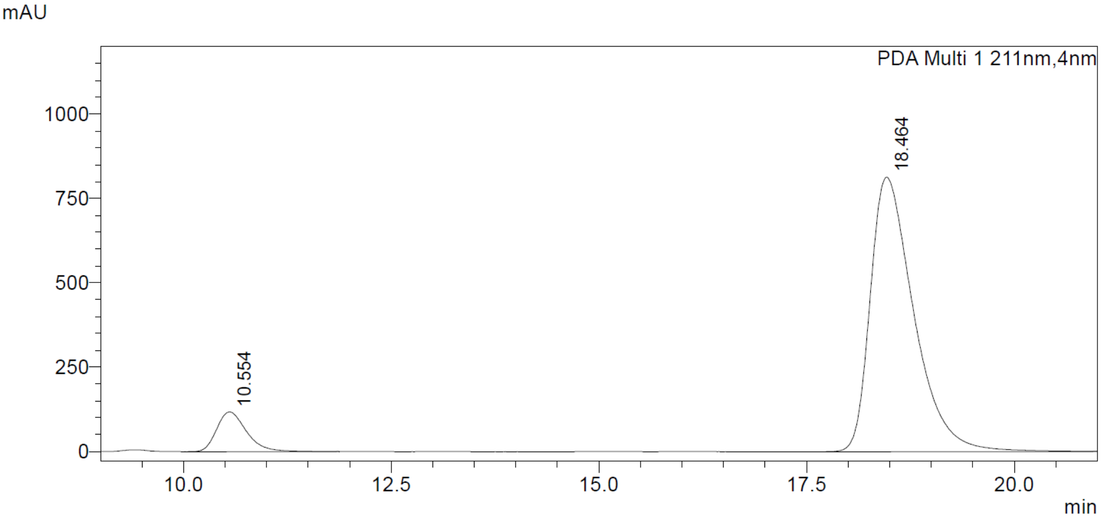

Racemic			Enantioenriched		
**<Peak Table>**			**<Peak Table>**		
PDA Ch1 211nm			PDA Ch1 211nm		
Peak#	Ret. Time	Area%	Peak#	Ret. Time	Area%
1	10.367	49.877	1	10.554	9.066
2	18.476	50.123	2	18.464	90.934
Total		100.000	Total		100.000

Fig. S63: Chiral HPLC analysis for (*S*,*Z*)-4-Chloro-3-(3-fluoro-3-phenylallyl)-3-hydroxy-1-tritylindolin-2-one (**50**), Chiralpak IB (95:5 hexane:IPA, flow rate 1 ml·min^{-1}, 211 nm, 30 °C) t_R (*R*)-**50**: 11.6 min, t_R (*S*)-**50**: 16.1 min, 4:96 e.r.

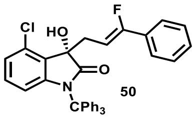

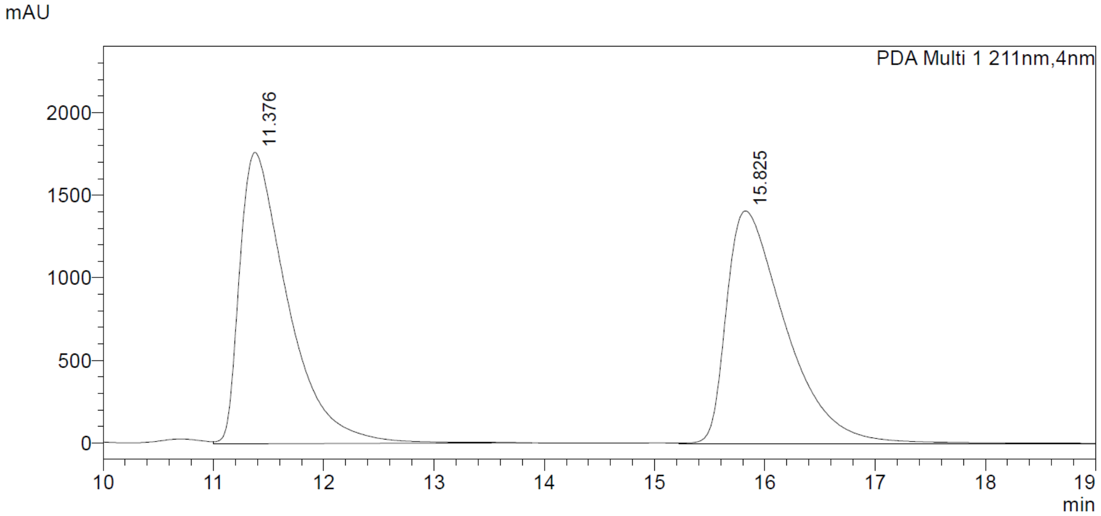

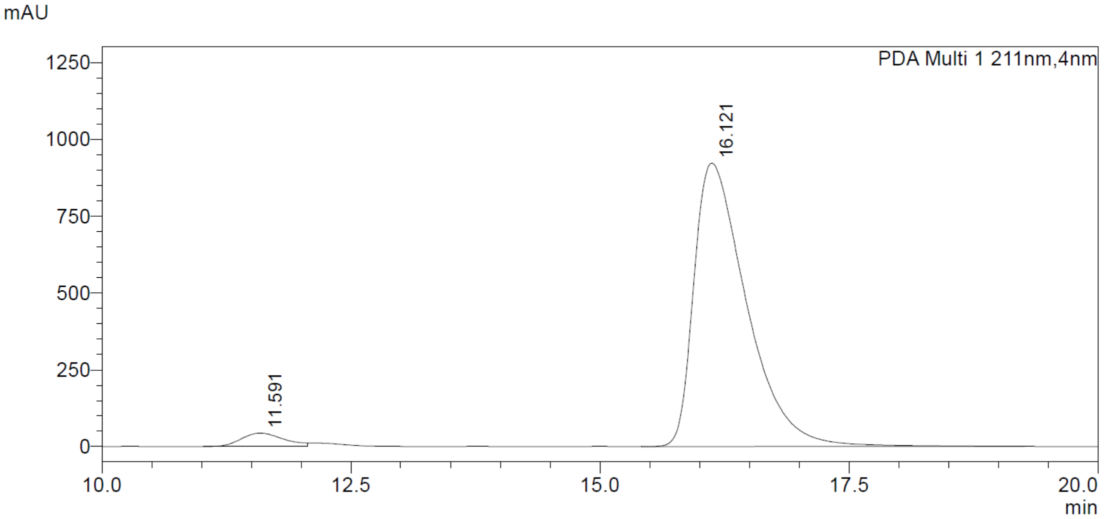

Racemic	Enantioenriched

Racemic — <Peak Table>

PDA Ch1 211nm

Peak#	Ret. Time	Area%
1	11.376	50.272
2	15.825	49.728
Total		100.000

Enantioenriched — <Peak Table>

PDA Ch1 211nm

Peak#	Ret. Time	Area%
1	11.591	3.709
2	16.121	96.291
Total		100.000

Fig. S64: Chiral HPLC analysis for (*S*,*Z*)-5-fluoro-3-(3-fluoro-3-phenylallyl)-3-hydroxy-1-tritylindolin-2-one (**51**), Chiralpak IB (95:5 hexane:IPA, flow rate 1 ml·min^{-1}, 211 nm, 30 °C) t_R (*R*)-**51**: 10.4 min, t_R (*S*)-**51**: 15.0 min, 4:96 e.r.

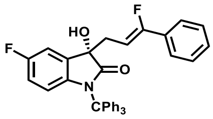

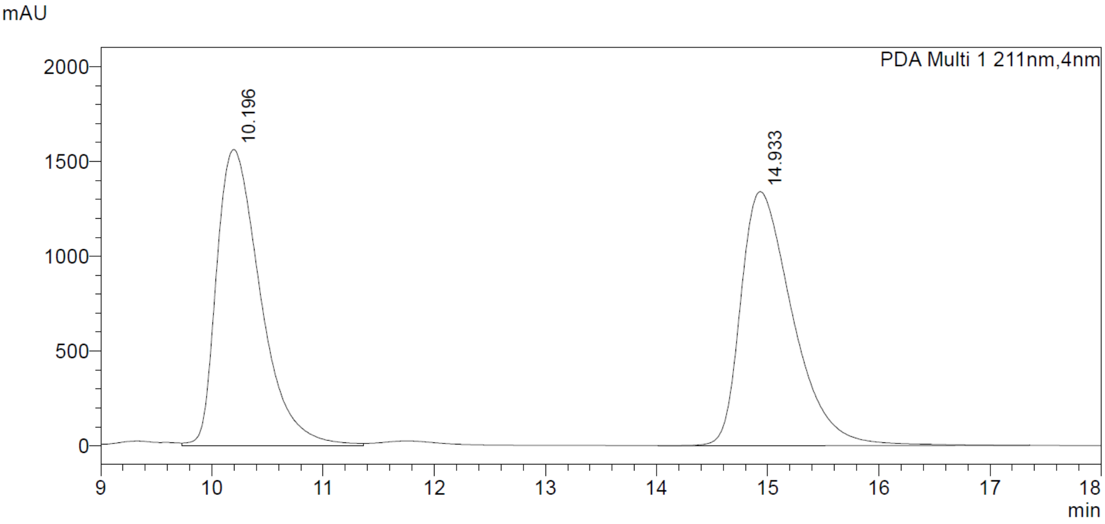

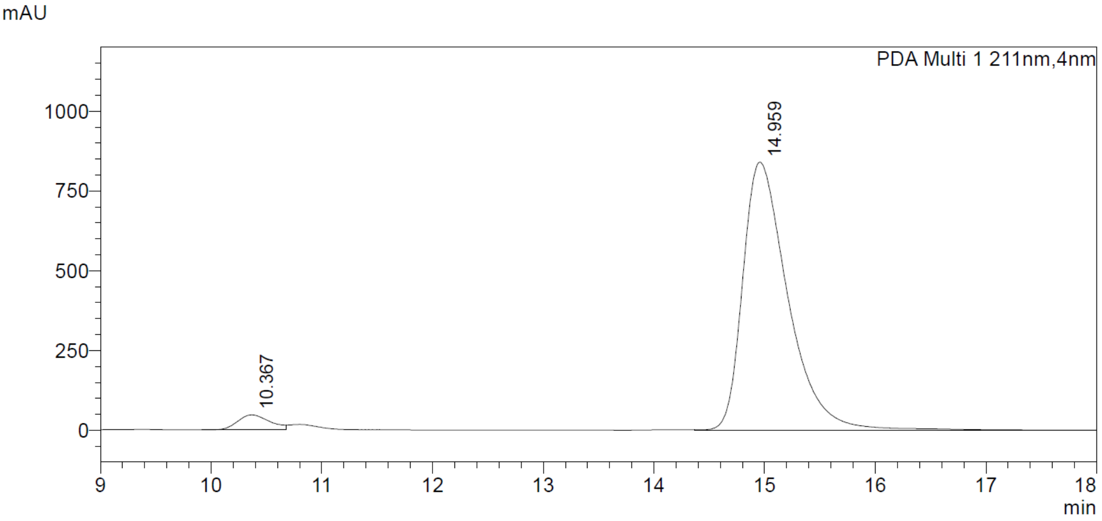

Racemic

<Peak Table>

PDA Ch1 211nm

Peak#	Ret. Time	Area%
1	10.196	49.704
2	14.933	50.296
Total		100.000

Enantioenriched

<Peak Table>

PDA Ch1 211nm

Peak#	Ret. Time	Area%
1	10.367	3.849
2	14.959	96.151
Total		100.000

Fig. S65: Chiral HPLC analysis for (*S*,*Z*)-3-(3-Fluoro-3-phenylallyl)-3-hydroxy-5-nitro-1-tritylindolin-2-one (**52**), Chiralpak IB (90:10 hexane:IPA, flow rate 1 ml·min^{-1}, 211 nm, 30 °C) t_R (*R*)-**52**: 9.1 min, t_R (*S*)-**52**: 13.4 min, 3:97 e.r.

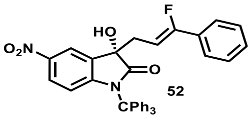

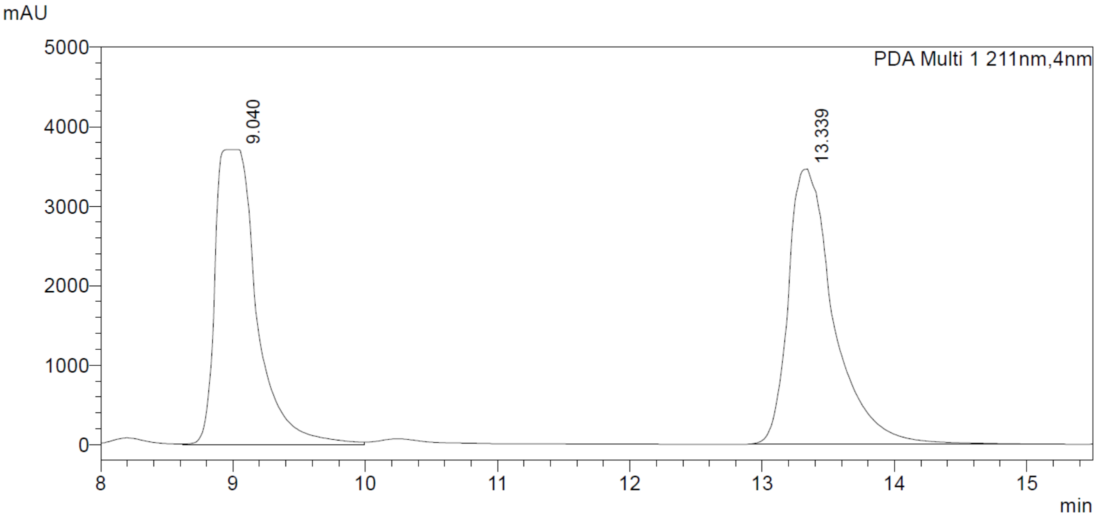

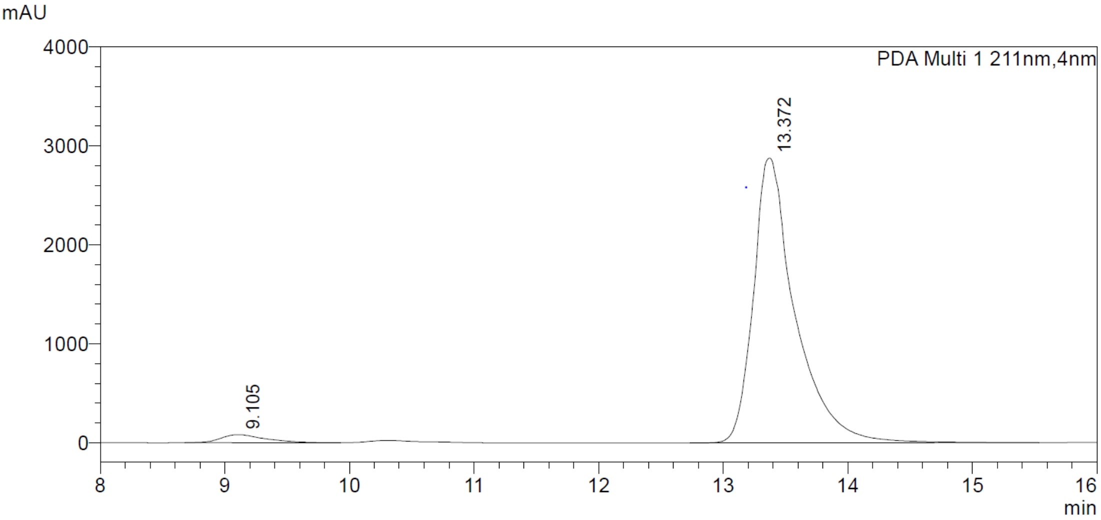

Racemic

<Peak Table>

PDA Ch1 211nm

Peak#	Ret. Time	Area%
1	9.040	49.438
2	13.339	50.562
Total		100.000

Enantioenriched

<Peak Table>

PDA Ch1 211nm

Peak#	Ret. Time	Area%
1	9.105	2.971
2	13.372	97.029
Total		100.000

Fig. S66: Chiral HPLC analysis for (*S*,*Z*)-3-(3-fluoro-3-phenylallyl)-3-hydroxy-5-methoxy-1-tritylindolin-2-one (**53**), Chiralpak IB (90:10 hexane:IPA, flow rate 1 ml·min^{-1}, 211 nm, 30 °C) t_R (*R*)-**53**: 8.5 min, t_R (*S*)-**53**: 10.1 min, 5:95 e.r.

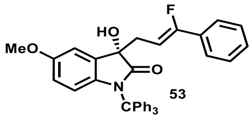

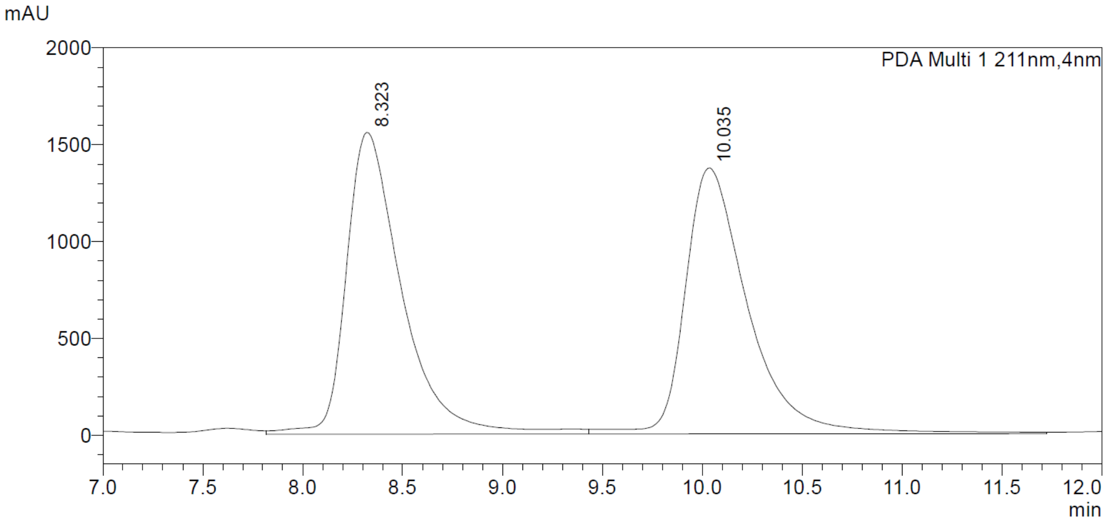

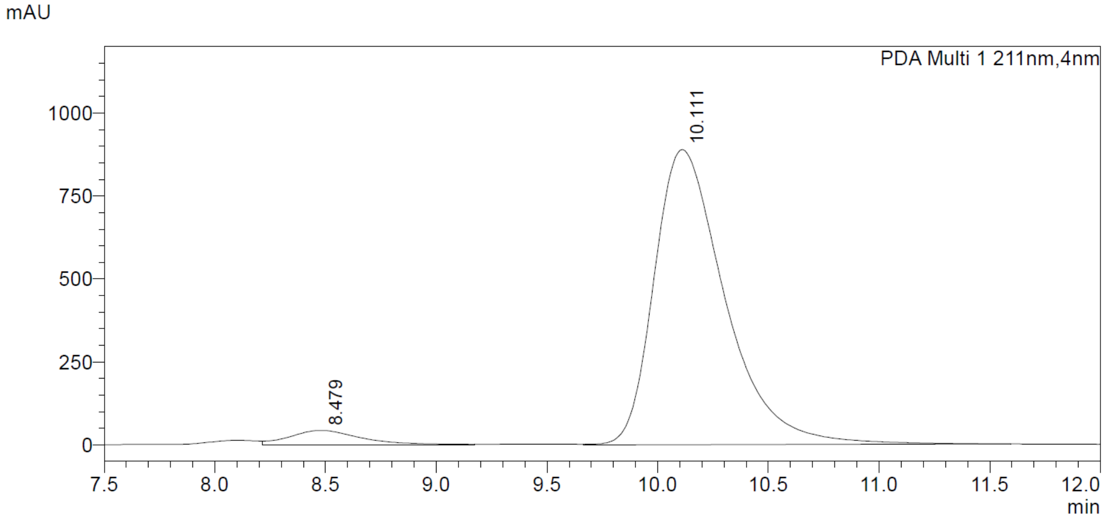

Racemic

<Peak Table>

PDA Ch1 211nm

Peak#	Ret. Time	Area%
1	8.323	49.999
2	10.035	50.001
Total		100.000

Enantioenriched

<Peak Table>

PDA Ch1 211nm

Peak#	Ret. Time	Area%
1	8.479	4.894
2	10.111	95.106
Total		100.000

Fig. S67: Chiral HPLC analysis for (*S*,*Z*)-6-Chloro-3-(3-fluoro-3-phenylallyl)-3-hydroxy-1-tritylindolin-2-one (**54**), Chiralpak IB (90:10 hexane:IPA, flow rate 1 ml·min^{-1}, 211 nm, 30 °C) t_R (*R*)-**54**: 7.2 min, t_R (*S*)-**54**: 9.4 min, 5:95 e.r.

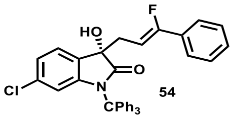

<Chromatogram>

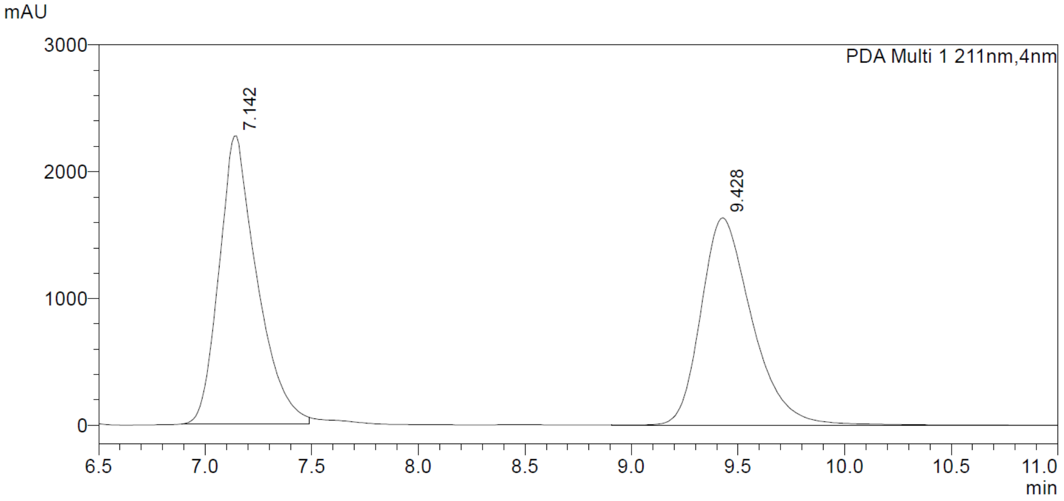

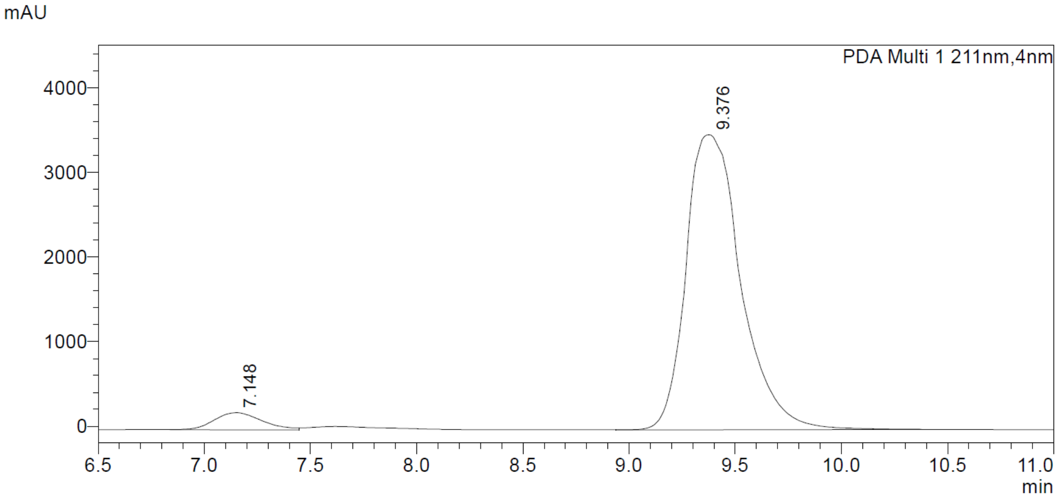

Racemic	Enantioenriched
**<Peak Table>** PDA Ch1 211nm	**<Peak Table>** PDA Ch1 211nm

Racemic — PDA Ch1 211nm

Peak#	Ret. Time	Area%
1	7.142	50.675
2	9.428	49.325
Total		100.000

Enantioenriched — PDA Ch1 211nm

Peak#	Ret. Time	Area%
1	7.148	4.830
2	9.376	95.170
Total		100.000

Fig. S68: Chiral HPLC analysis for (*S*,*E*)-3-Cinnamyl-3-hydroxy-1-tritylindolin-2-one (**45**), Chiralpak AD-H (90:10 hexane:IPA, flow rate 1 mL·min^{-1}, 211 nm, 30 °C) t_R (*R*)-**45**: 12.5 min, t_R (*S*)-**45**: 14.6 min, 5:95 e.r.

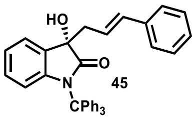

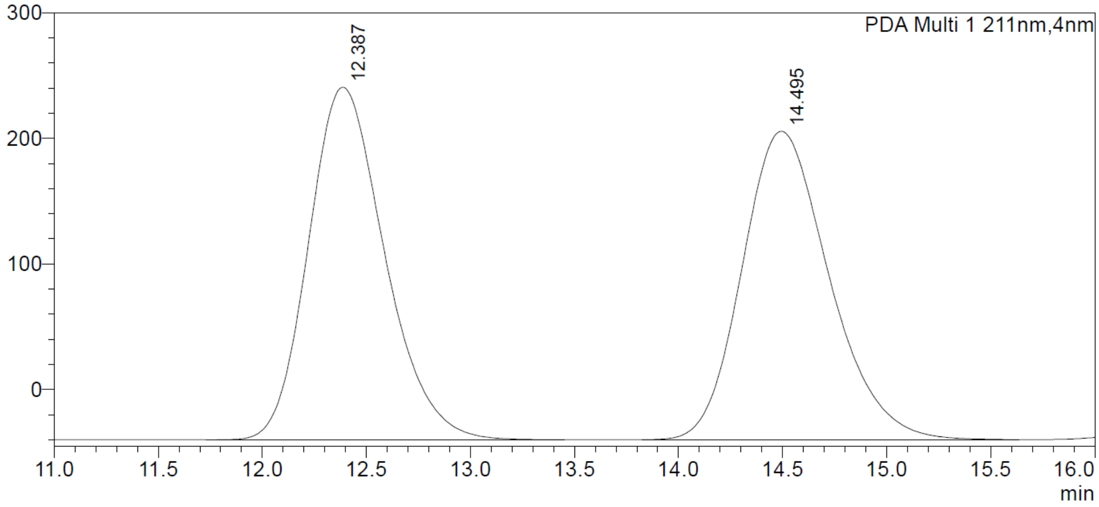

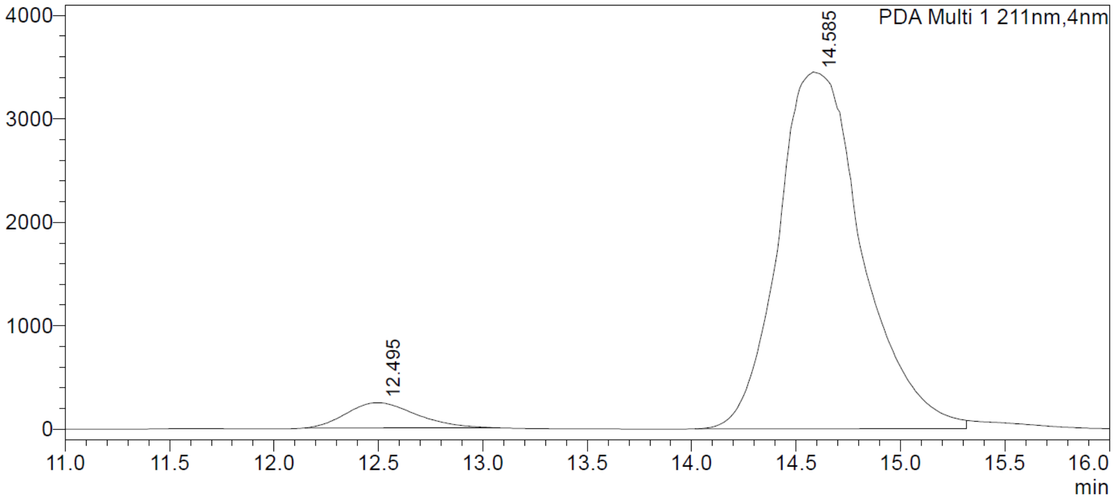

Racemic

PDA Ch1 211nm

Peak#	Ret. Time	Area%
1	12.387	49.951
2	14.495	50.049
Total		100.000

Enantioenriched

PDA Ch1 211nm

Peak#	Ret. Time	Area%
1	12.495	5.460
2	14.585	94.540
Total		100.000

Fig. S69: Chiral HPLC analysis for (*S*,*E*)-3-Hydroxy-3-[3-(4-methoxyphenyl)allyl]-1-tritylindolin-2-one (**55**), Chiralpak IB (90:10 hexane:IPA, flow rate 1 mL·min^{-1}, 211 nm, 30 °C) t_R (*R*)-**55**: 9.0 min, t_R (*S*)-**55**: 12.5 min, 11:89 e.r.

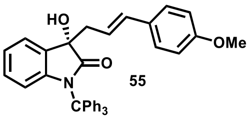

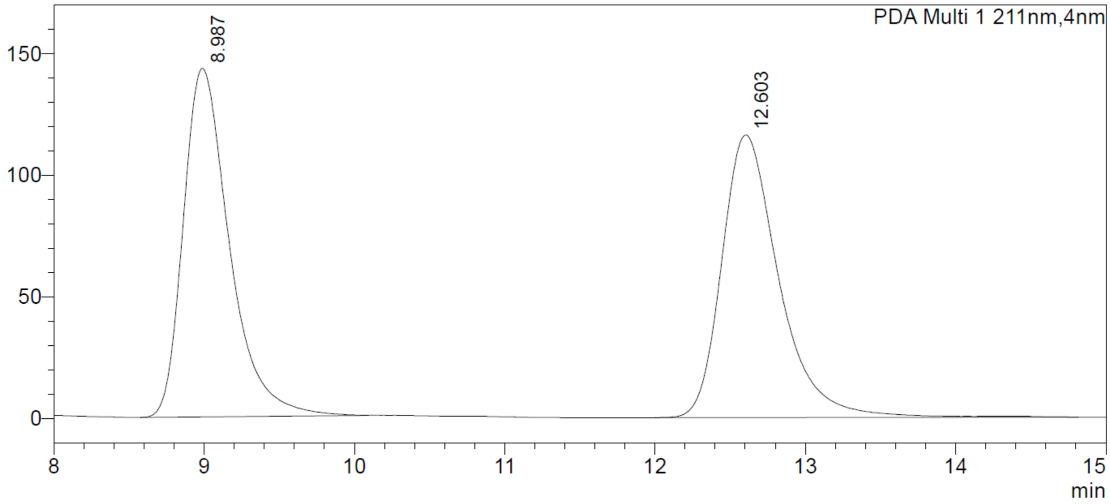

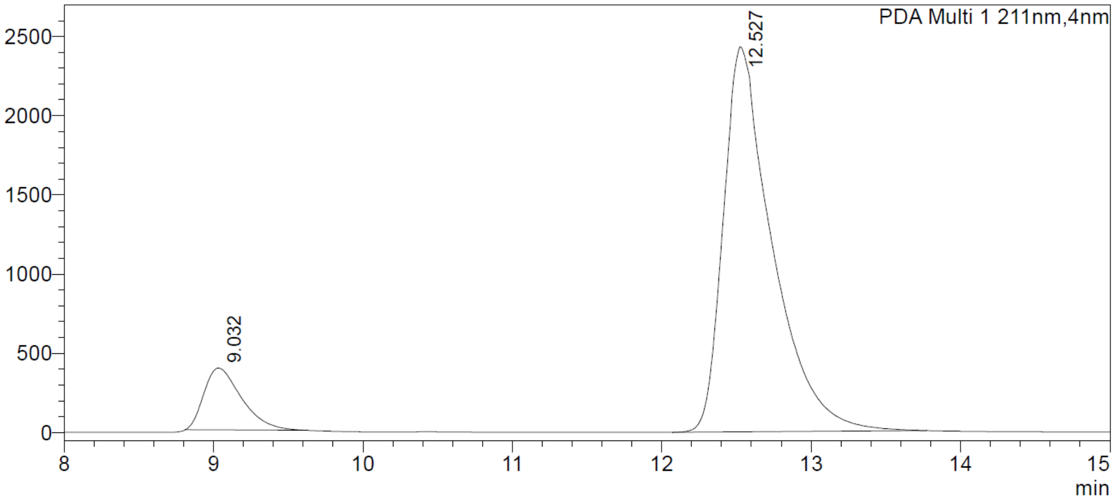

Racemic	Enantioenriched

Racemic — PDA Ch1 211nm

Peak#	Ret. Time	Area%
1	8.987	49.772
2	12.603	50.228
Total		100.000

Enantioenriched — PDA Ch1 211nm

Peak#	Ret. Time	Area%
1	9.032	11.001
2	12.527	88.999
Total		100.000

Fig. S70: Chiral HPLC analysis for (*S*,*E*)-3-Hydroxy-3-{3-[4-(trifluoromethyl)phenyl]allyl}-1-tritylindolin-2-one (**56**), Chiralcel OD-H (90:10 hexane:, flow rate 1 mLmin^{-1}, 211 nm, 30 °C) t_R (*R*)-**56**: 9.4 min, t_R (*S*)-**56**: 18.0 min, 8:92 e.r.

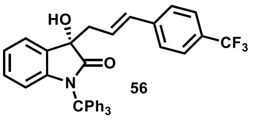

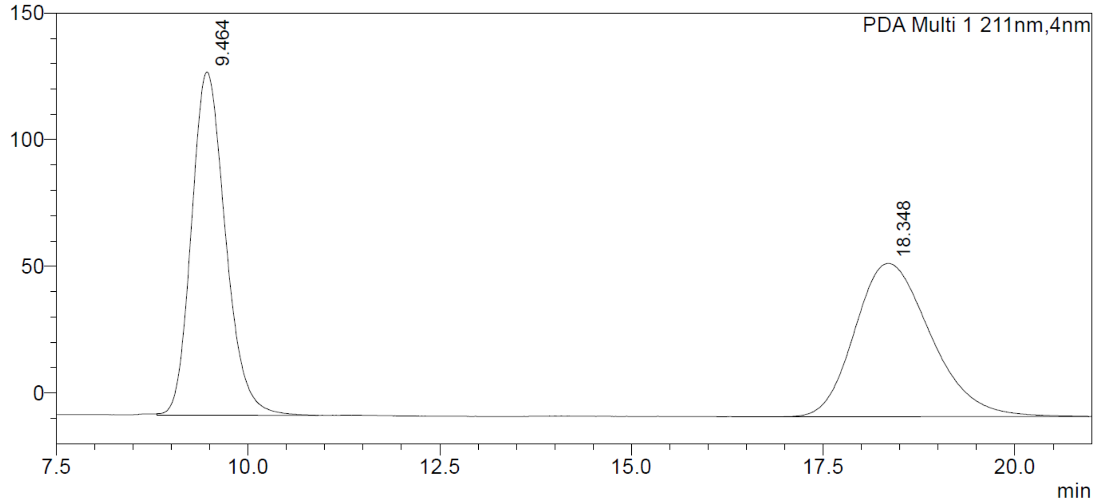

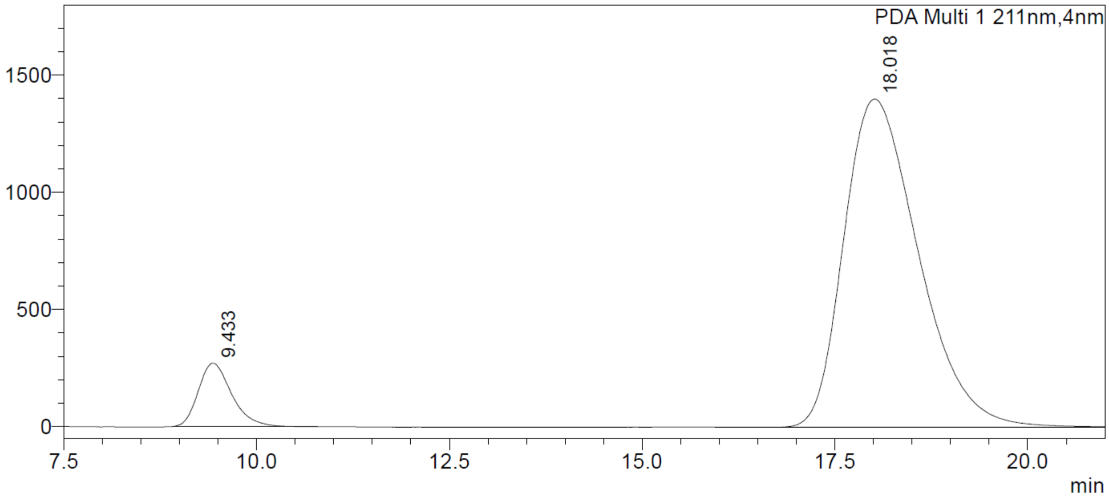

Racemic	Enantioenriched
PDA Ch1 211nm	PDA Ch1 211nm

Peak#	Ret. Time	Area%
1	9.464	50.079
2	18.348	49.921
Total		100.000

Peak#	Ret. Time	Area%
1	9.433	7.902
2	18.018	92.098
Total		100.000

Fig. S71: Chiral HPLC analysis for (*S*,*E*)-3-(3-(4-fluorophenyl)allyl)-3-hydroxy-1-tritylindolin-2-one (**57**), Chiralcel IB (90:10 hexane:IPA, flow rate 1 mLmin^{-1}, 211 nm, 30 °C) t_R (*R*)-**57**: 7.9 min, t_R (*S*)-**57**: 11.0 min, 9:91 e.r.

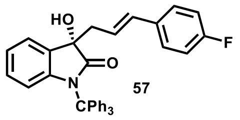

57

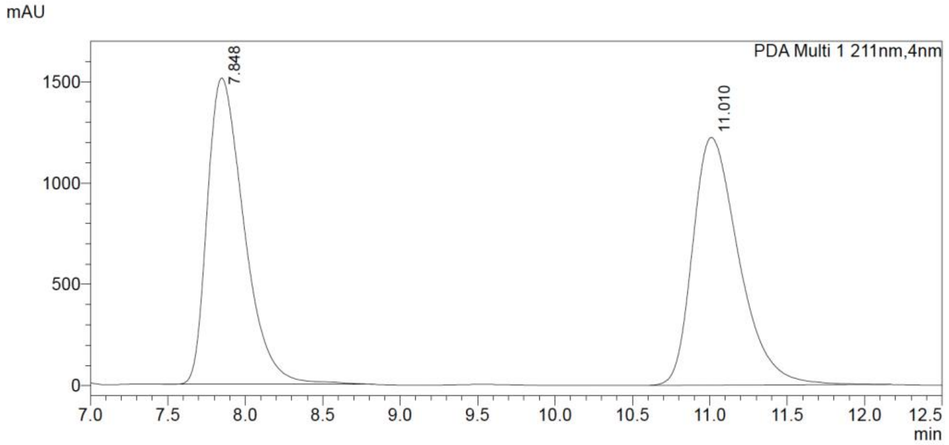

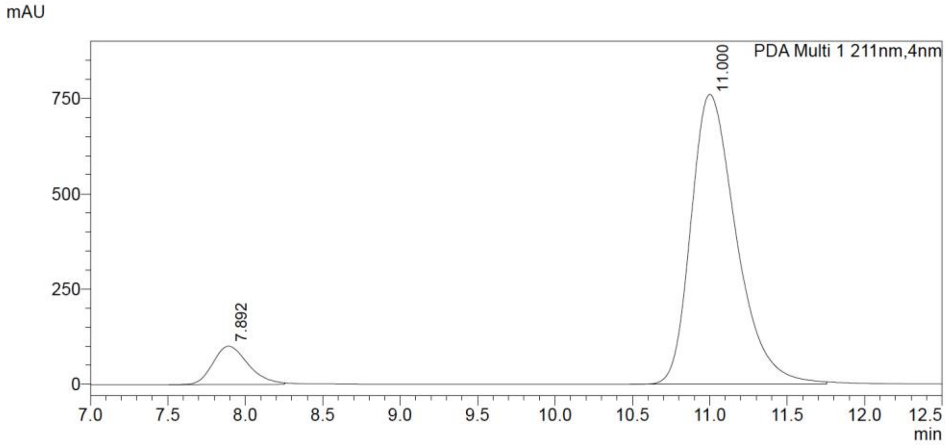

Racemic

PDA Ch1 211nm

Peak#	Ret. Time	Area%
1	7.848	49.670
2	11.010	50.330
Total		100.000

Enantioenriched

PDA Ch1 211nm

Peak#	Ret. Time	Area%
1	7.892	9.360
2	11.000	90.640
Total		100.000

Fig. S72: Chiral HPLC analysis for (*S*,*E*)-3-Hydroxy-3-[3-(2-methoxyphenyl)allyl]-1-tritylindolin-2-one (**58**), Chiralpak IB (95:5 hexane:IPA, flow rate 1 mL·min^{-1}, 211 nm, 30 °C) t_R (*R*)-**58**: 15.1 min, t_R (*S*)-**58**: 24.0 min, 9:91 e.r.

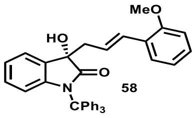

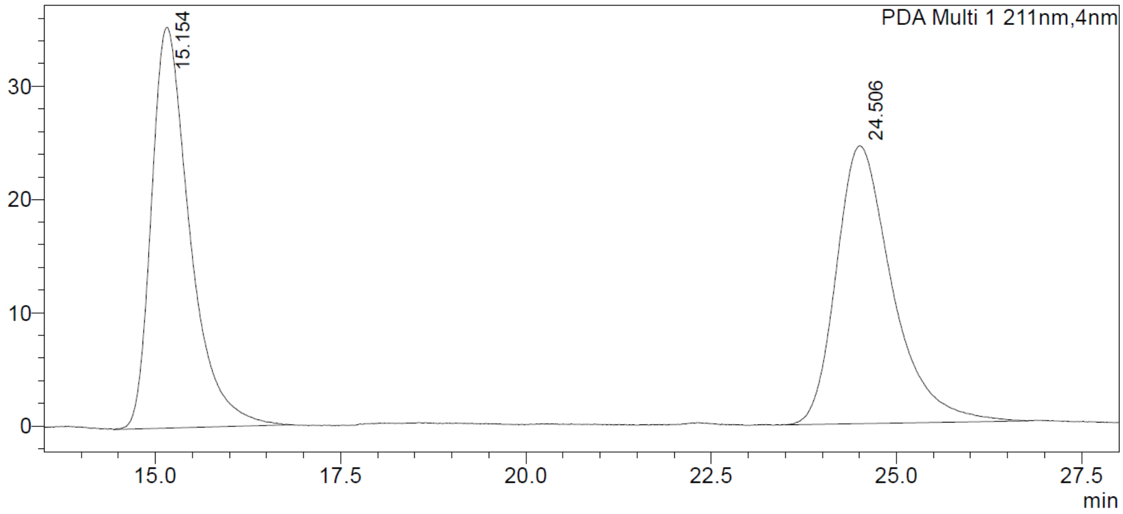

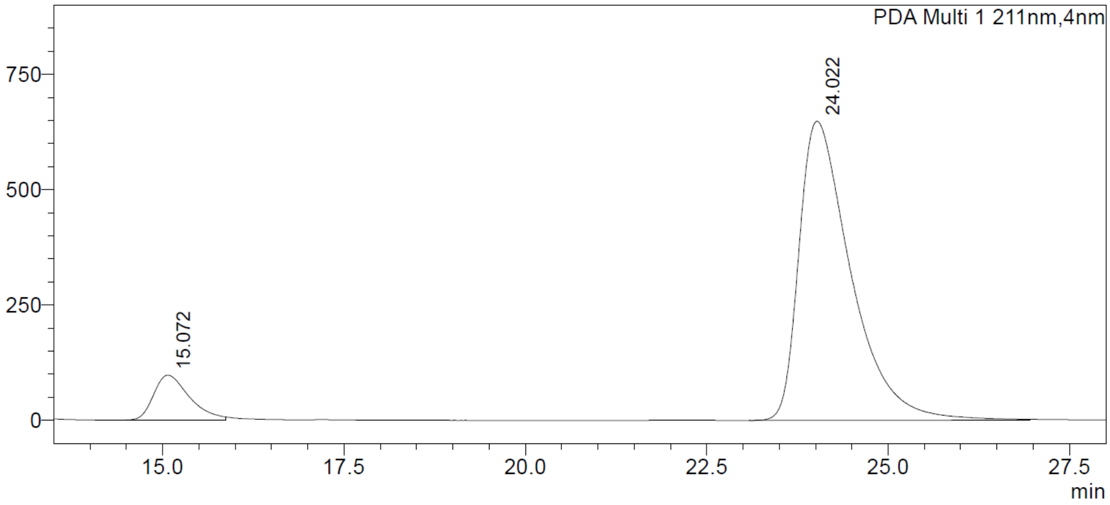

Racemic			Enantioenriched		
PDA Ch1 211nm			PDA Ch1 211nm		
Peak#	Ret. Time	Area%	Peak#	Ret. Time	Area%
1	15.154	49.704	1	15.072	9.396
2	24.506	50.296	2	24.022	90.604
Total		100.000	Total		100.000

Fig. S73: Chiral HPLC analysis for (*S*,*E*)-3-Hydroxy-3-[3-(1-naphthyl)allyl]-1-tritylindolin-2-one (**59**), Chiralcel OD-H (90:10 hexane:IPA, flow rate 1 mL·min^{-1}, 211 nm, 30 °C) t_R (*R*)-**59**: 10.4 min, t_R (*S*)-**59**: 14.9 min, 6:94 e.r.

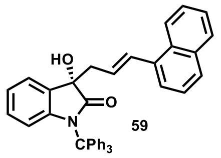

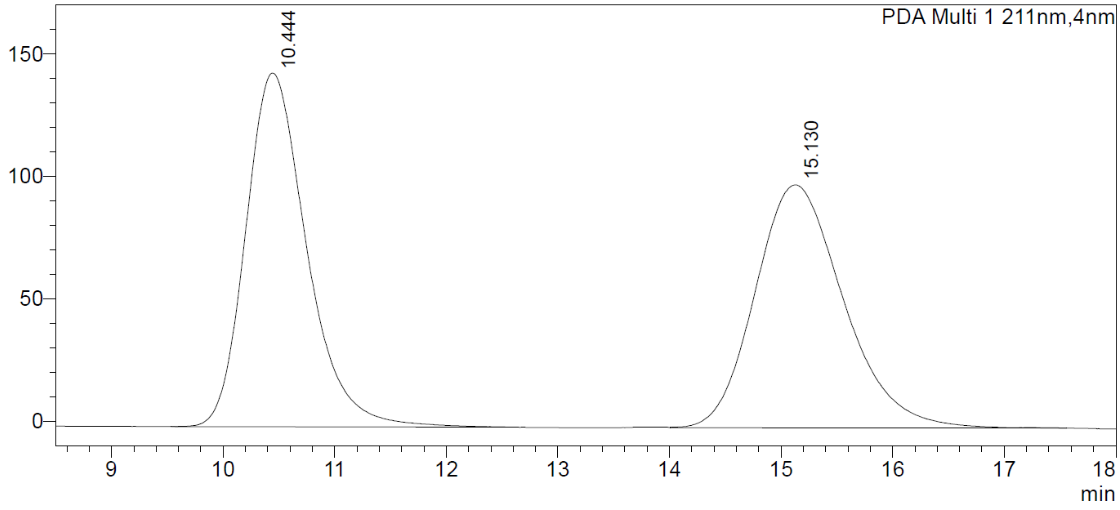

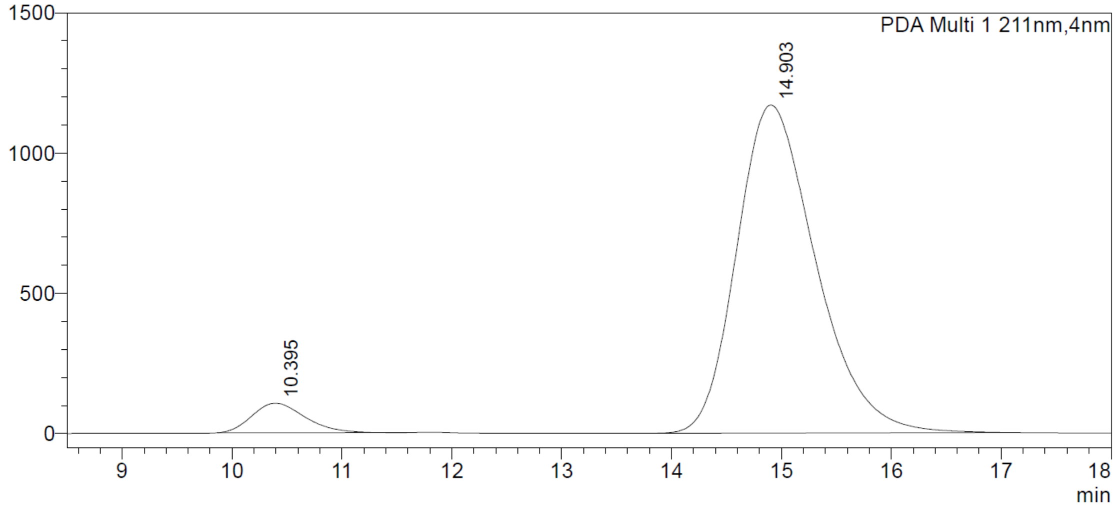

Racemic	Enantioenriched

Racemic — PDA Ch1 211nm

Peak#	Ret. Time	Area%
1	10.444	50.096
2	15.130	49.904
Total		100.000

Enantioenriched — PDA Ch1 211nm

Peak#	Ret. Time	Area%
1	10.395	5.628
2	14.903	94.372
Total		100.000

Fig. S74: Chiral HPLC analysis for (*S*,*E*)-3-Hydroxy-3-[3-(2-naphthyl)allyl]-1-tritylindolin-2-one (**60**), Chiralcel OD-H (90:10 hexane:IPA, flow rate 1 mL·min^{-1}, 211 nm, 30 °C) t_R (*R*)-**60**: 15.4 min, t_R (*S*)-**60**: 31.9 min, 9:91 e.r.

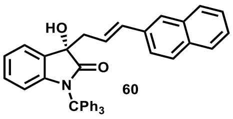

60

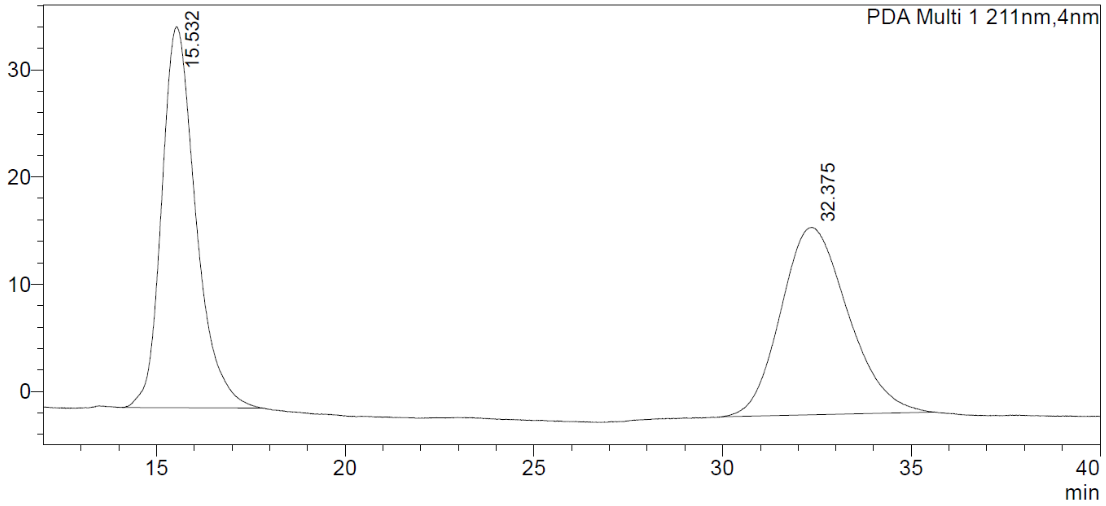

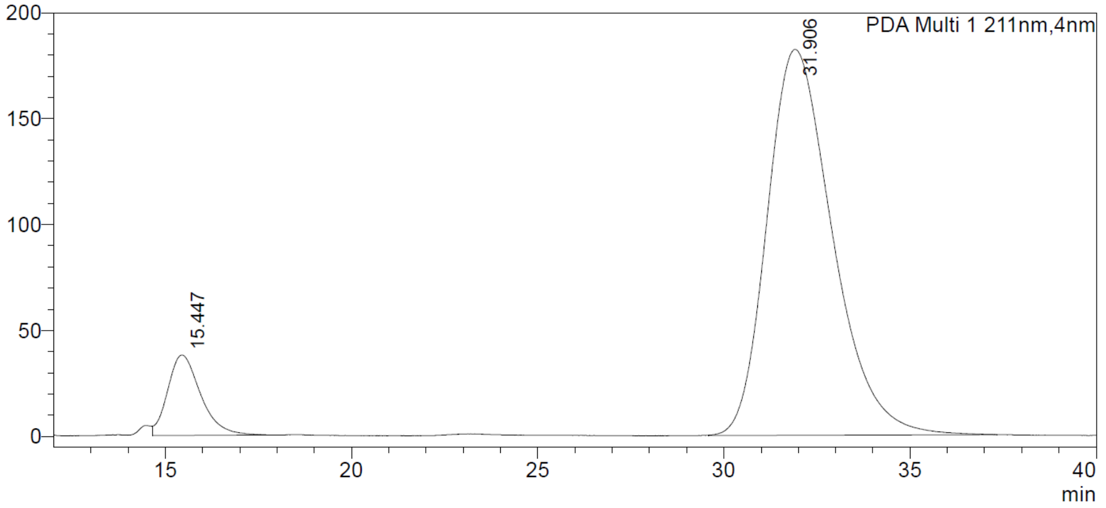

Racemic

PDA Ch1 211nm

Peak#	Ret. Time	Area%
1	15.532	50.835
2	32.375	49.165
Total		100.000

Enantioenriched

PDA Ch1 211nm

Peak#	Ret. Time	Area%
1	15.447	9.497
2	31.906	90.503
Total		100.000

Fig. S75: Chiral HPLC analysis for (*S*,*E*)-3-Hydroxy-3-[3-(thiophen-3-yl)allyl]-1-tritylindolin-2-one (**61**), Chiralpak AD-H (95:5 hexane:IPA, flow rate 1 mL·min^{-1}, 211 nm, 30 °C) t_R (*R*)-**61**: 15.2 min, t_R (*S*)-**61**: 17.3 min, 13:87 e.r.

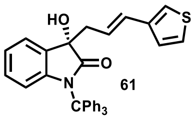

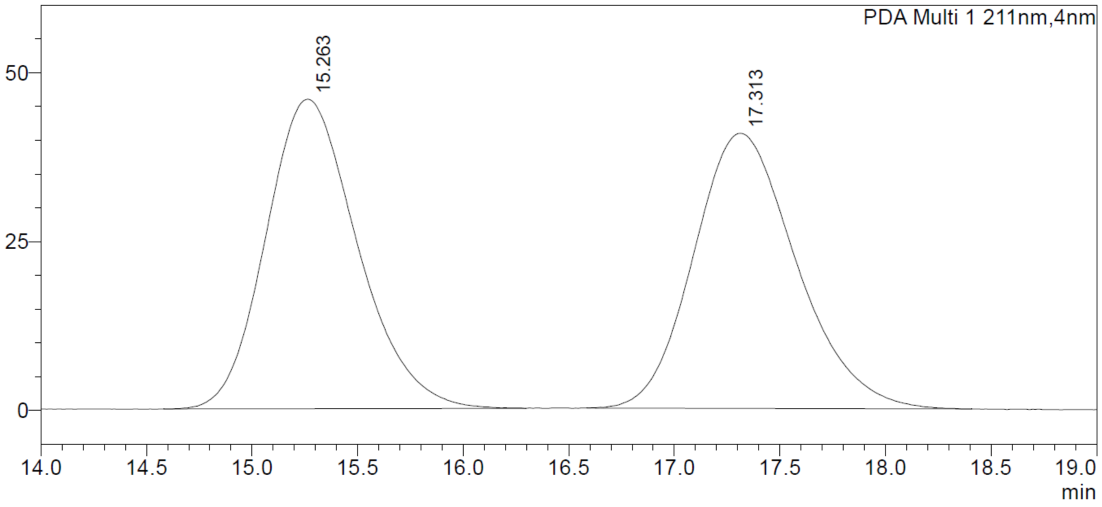

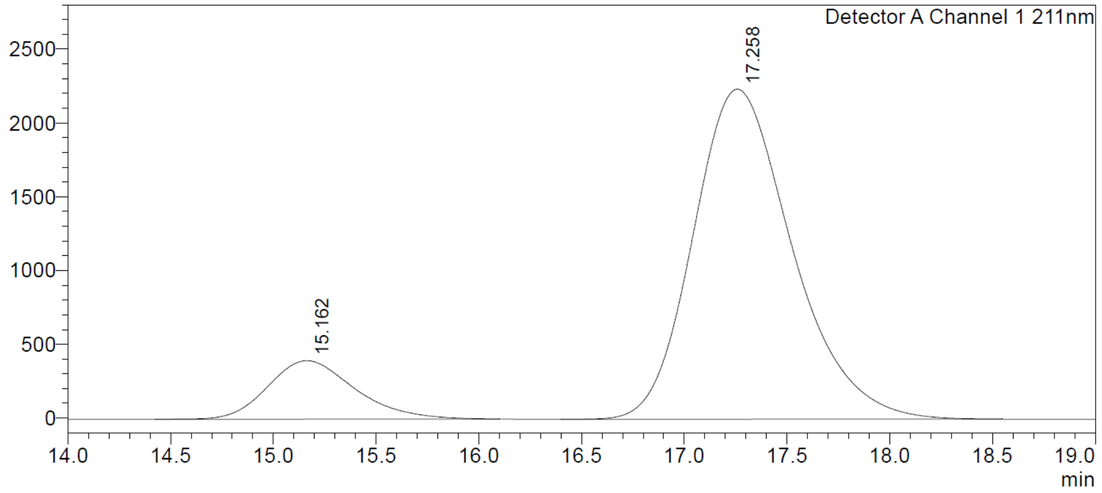

Racemic

PDA Ch1 211nm

Peak#	Ret. Time	Area%
1	15.263	50.007
2	17.313	49.993
Total		100.000

Enantioenriched

Detector A Channel 1 211nm

Peak#	Ret. Time	Area%
1	15.162	13.473
2	17.258	86.527
Total		100.000

Fig. S76: Chiral HPLC analysis for (*S*,*E*)-3-Hydroxy-3-(2-methyl-3-phenylallyl)-1-tritylindolin-2-one (**62**), Chiralpak AD-H (95:5 hexane:IPA, flow rate 1 mL·min^{-1}, 211 nm, 30 °C) t_R (*R*)-**62**: 17.4 min, t_R (*S*)-**62**: 21.8 min, 11:89 e.r.

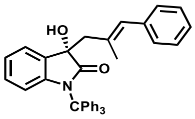

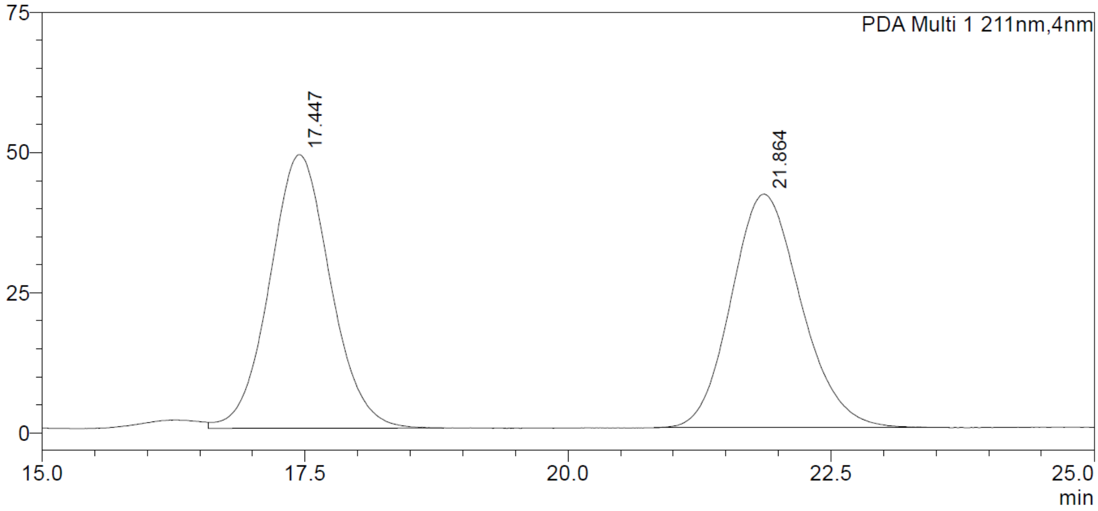

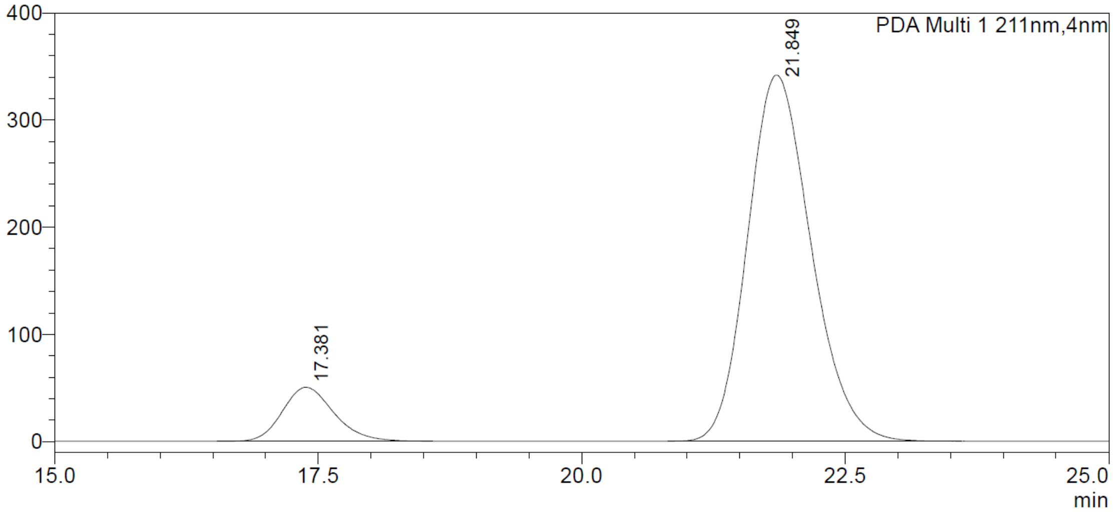

Racemic	Enantioenriched
PDA Ch1 211nm	PDA Ch1 211nm

Racemic — PDA Ch1 211nm

Peak#	Ret. Time	Area%
1	17.447	50.432
2	21.864	49.568
Total		100.000

Enantioenriched — PDA Ch1 211nm

Peak#	Ret. Time	Area%
1	17.381	10.719
2	21.849	89.281
Total		100.000

Fig. S77: Chiral HPLC analysis (*S*,*E*)-4-chloro-3-cinnamyl-3-hydroxy-1-tritylindolin-2-one (**63**), Chiralcel IB (90:10 hexane:IPA, flow rate 1 mLmin^{-1}, 211 nm, 30 °C) t_R (*R*)-**63**: 7.0 min, t_R (*S*)-**63**: 8.5 min, 13:87 e.r.

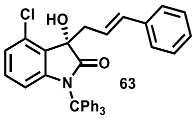

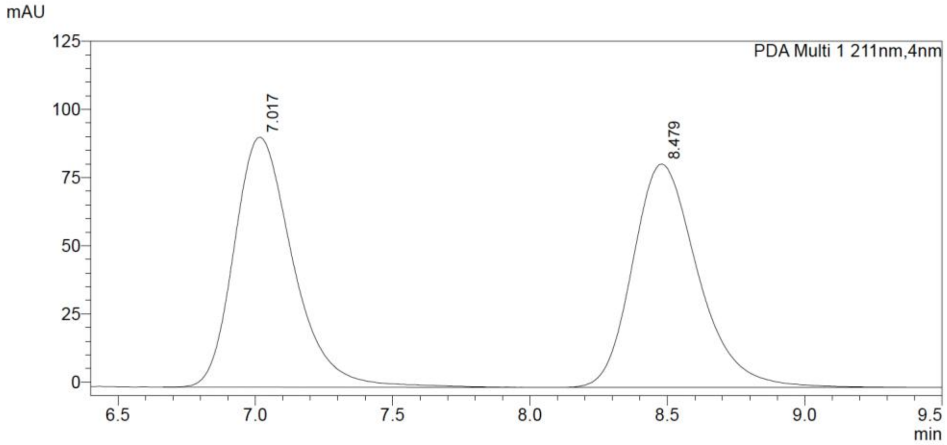

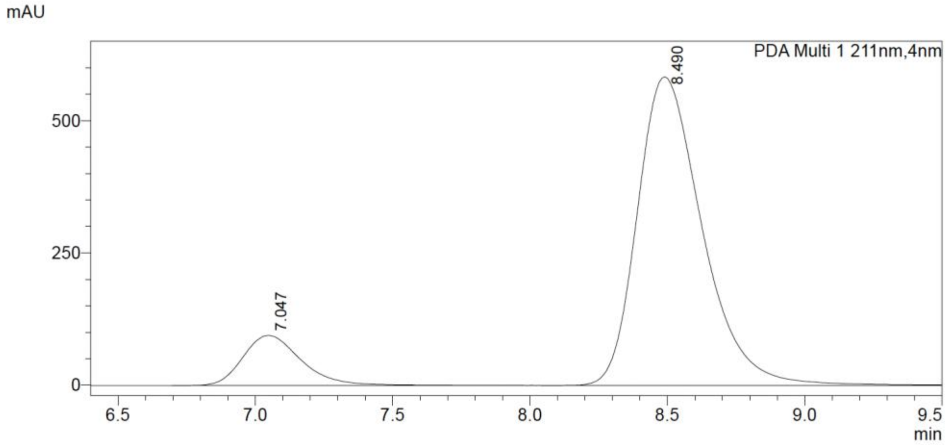

Racemic	Enantioenriched

Racemic — PDA Ch1 211nm

Peak#	Ret. Time	Area%
1	7.017	50.131
2	8.479	49.869
Total		100.000

Enantioenriched — PDA Ch1 211nm

Peak#	Ret. Time	Area%
1	7.047	12.816
2	8.490	87.184
Total		100.000

Fig. S78: Chiral HPLC analysis for (*S*,*E*)-3-cinnamyl-3-hydroxy-5-methoxy-1-tritylindolin-2-one (**64**), Chiralcel IB (90:10 hexane:IPA, flow rate 1 mLmin^{-1}, 211 nm, 30 °C) t_R (*R*)-**64**: 9.0 min, t_R (*S*)-**64**: 10.7 min, 11:89 e.r.

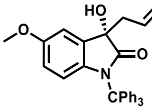

64

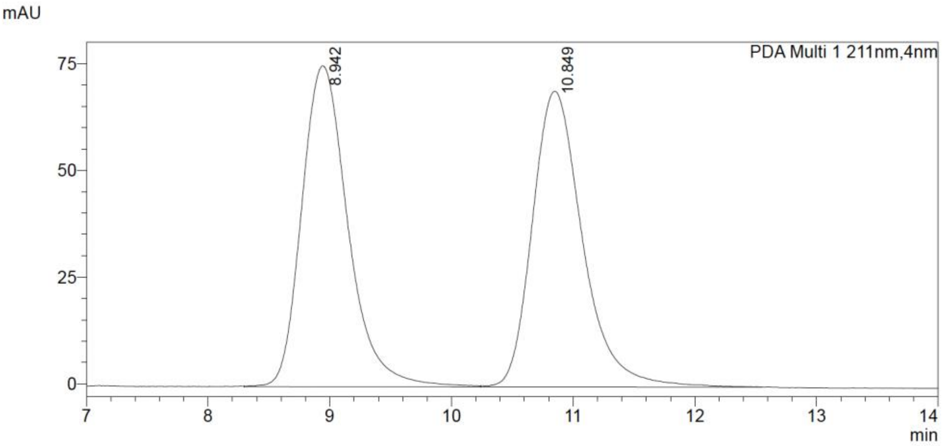

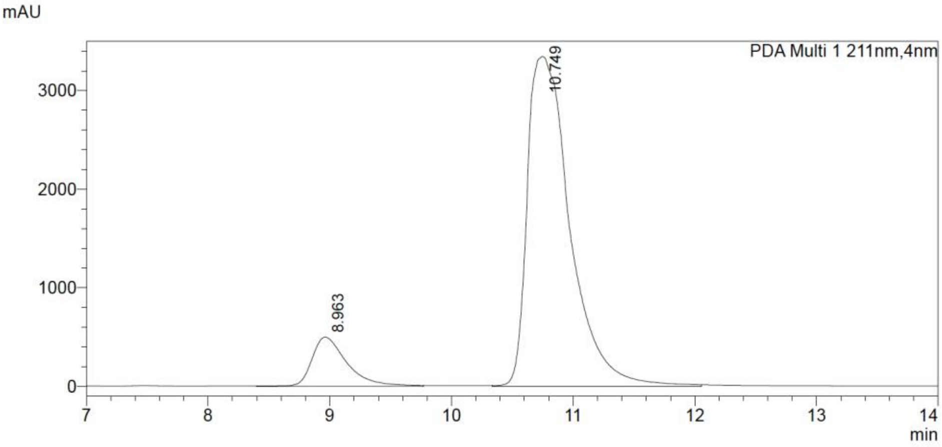

Racemic	Enantioenriched

Racemic:

PDA Ch1 211nm

Peak#	Ret. Time	Area%
1	8.942	49.975
2	10.849	50.025
Total		100.000

Enantioenriched:

PDA Ch1 211nm

Peak#	Ret. Time	Area%
1	8.963	10.978
2	10.749	89.022
Total		100.000

Fig. S79: Chiral HPLC analysis for (*S*,*E*)-6-bromo-3-cinnamyl-3-hydroxy-1-tritylindolin-2-one (**65**), Chiralcel IB (90:10 hexane:IPA, flow rate 1 mLmin^{-1}, 211 nm, 30 °C) t_R (*R*)-**65**: 8.4 min, t_R (*S*)-**65**: 10.7 min, 10:90 e.r.

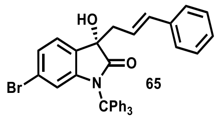

65

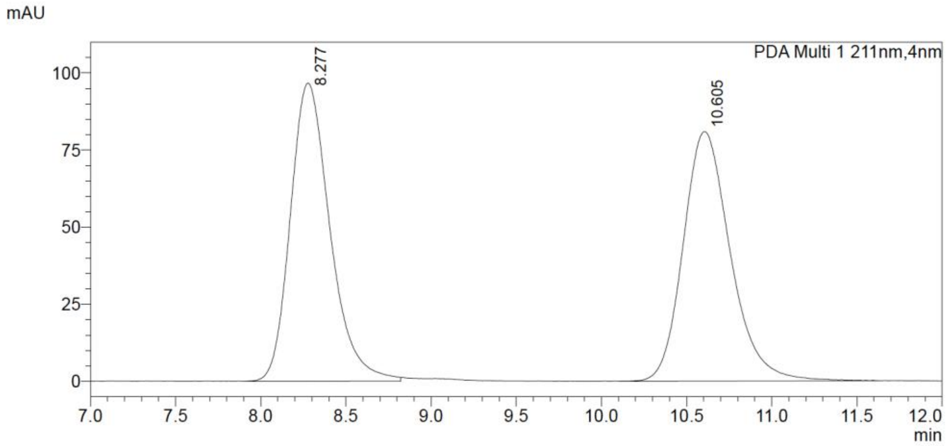

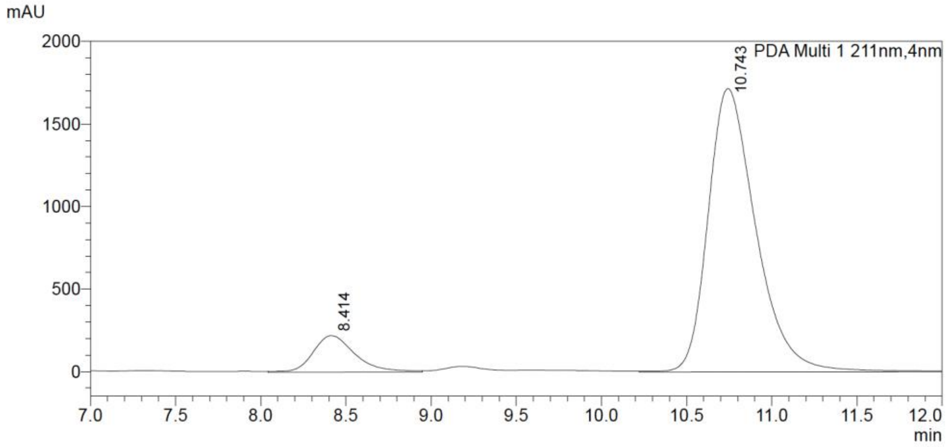

Racemic	Enantioenriched
PDA Ch1 211nm	PDA Ch1 211nm

Racemic — PDA Ch1 211nm

Peak#	Ret. Time	Area%
1	8.277	50.082
2	10.605	49.918
Total		100.000

Enantioenriched — PDA Ch1 211nm

Peak#	Ret. Time	Area%
1	8.414	10.156
2	10.743	89.844
Total		100.000

Fig. S80: Chiral HPLC analysis for ((*E*)-1-cyclopropyl-3-phenylbut-2-en-1-yl)-3-hydroxy-1-tritylindolin-2-one (**S127**), Chiralpak ID (95:5 hexane:IPA, flow rate 1 ml·min^{-1}, 211 nm, 30 °C) major diastereomer: t_R (*R*,*R*)-**S127**:13.3min, t_R (*S*,*S*)-**S127**: 23.1min; 21:79 e.r.; minor diastereomer t_R (*S*,*R*)-**S127**: 14.5min, t_R (*R*,*S*)-**S127**: 15.7min, 79:21 e.r.

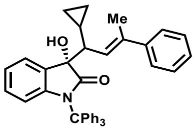

S127

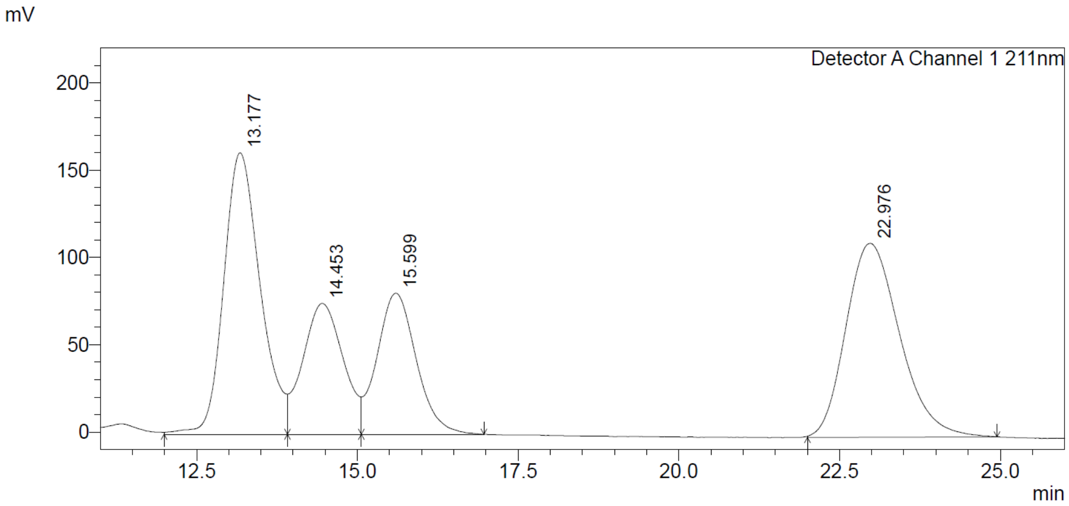

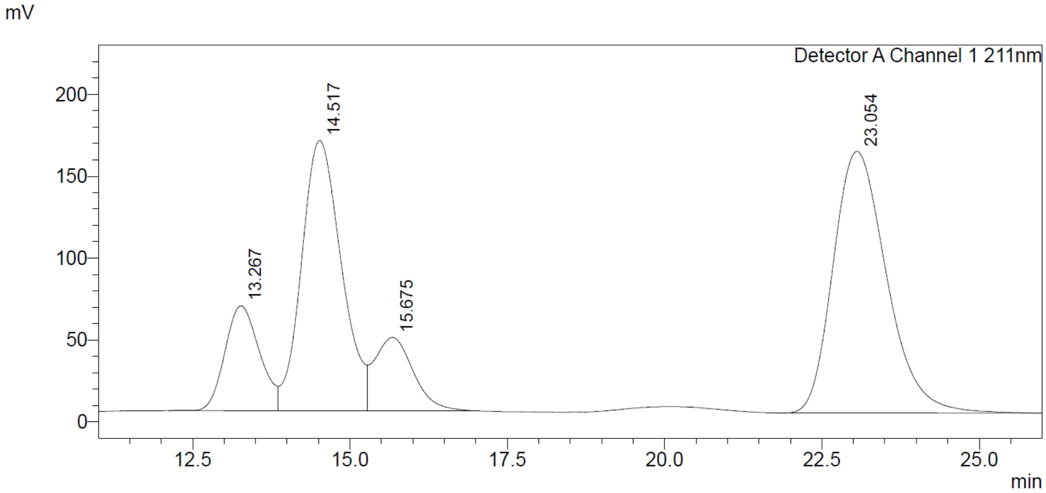

Racemic

<Peak Table>

Detector A Channel 1 211nm

Peak#	Ret. Time	Area%
1	13.177	32.599
2	14.453	16.792
3	15.599	17.553
4	22.976	33.056
Total		100.000

Enantioenriched

<Peak Table>

Detector A Channel 1 211nm

Peak#	Ret. Time	Area%
1	13.267	11.659
2	14.517	34.474
3	15.675	9.094
4	23.054	44.773
Total		100.000

Fig. S81: Chiral HPLC analysis ethyl (*E*)-4-(3-hydroxy-2-oxo-1-tritylindolin-3-yl)crotonate (**70**), Chiralpak IA (90:10 hexane:IPA, flow rate 1 mL·min^{-1}, 211 nm, 30 °C) t_R (*R*)-**70**: 13.3 min, t_R (*S*)-**70**: 15.0 min, 5:95 e.r.

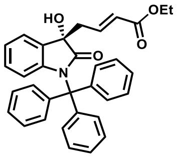

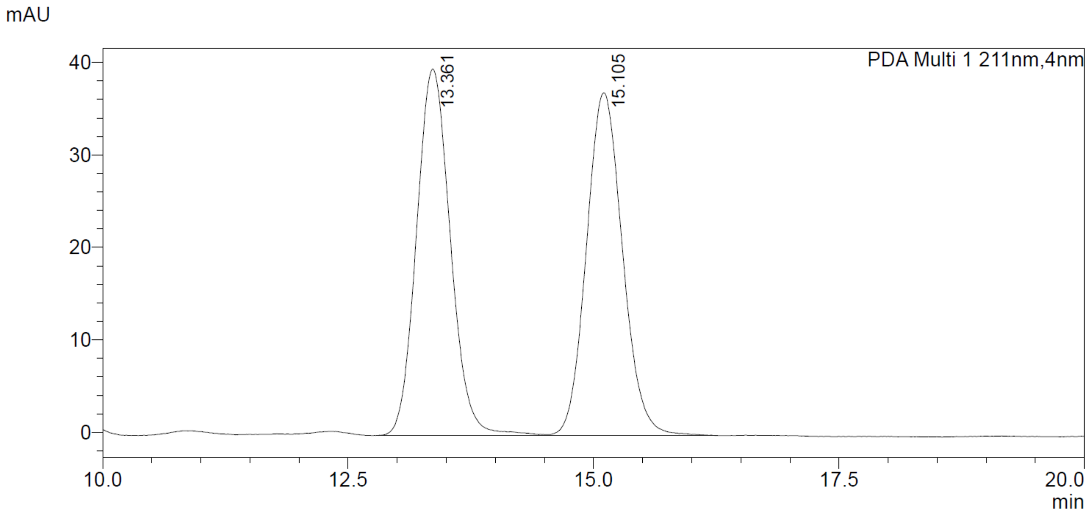

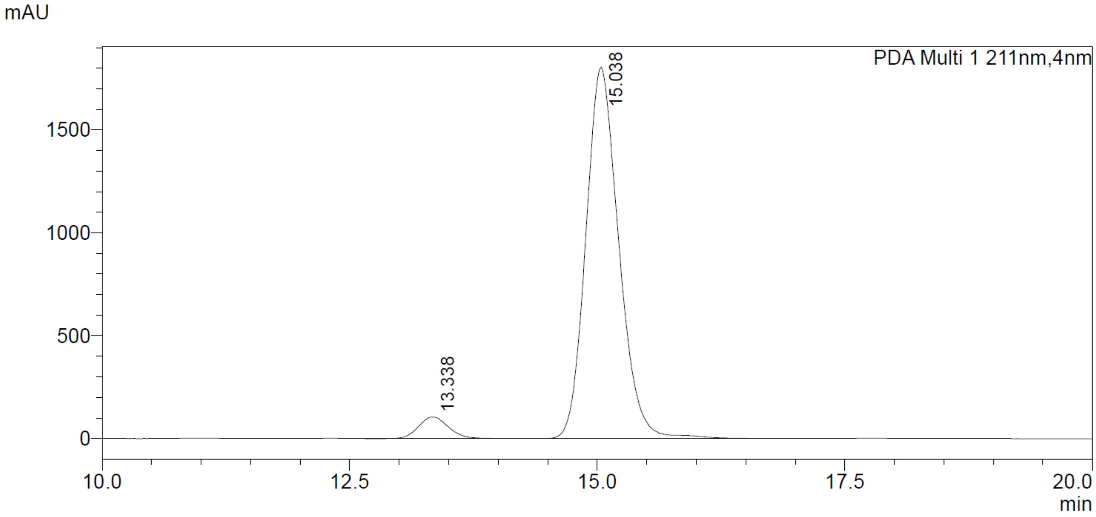

Racemic			Enantioenriched		
PDA Ch1 211nm			PDA Ch1 211nm		
Peak#	Ret. Time	Area%	Peak#	Ret. Time	Area%
1	13.361	50.098	1	13.338	4.947
2	15.105	49.902	2	15.038	95.053
Total		100.000	Total		100.000

Fig. S82: Chiral HPLC analysis for (*S*,*E*)-3-(3-(4-fluorophenyl)but-2-en-1-yl)-3-hydroxy-1-methylindolin-2-one (**S15**), ChiralCel® OD-H, 95:5 Hexane:IPA, flowrate 1 mL·min^{-1}, 254 nm, t_R (*R*)-**S15** 15.3 min, t_R (*S*)-**S15** 17.5 min, 35:65 e.r.

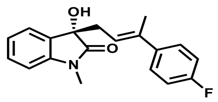

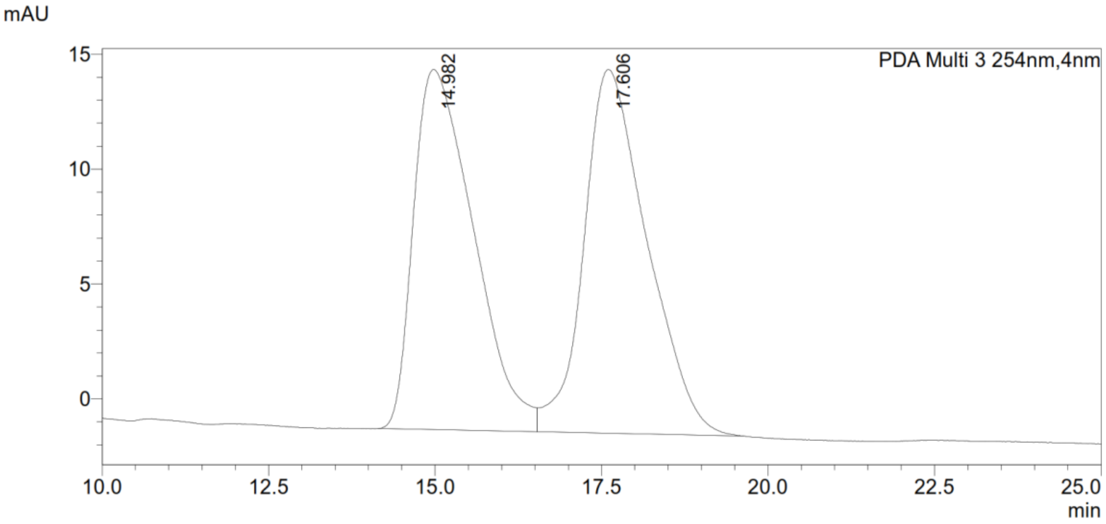

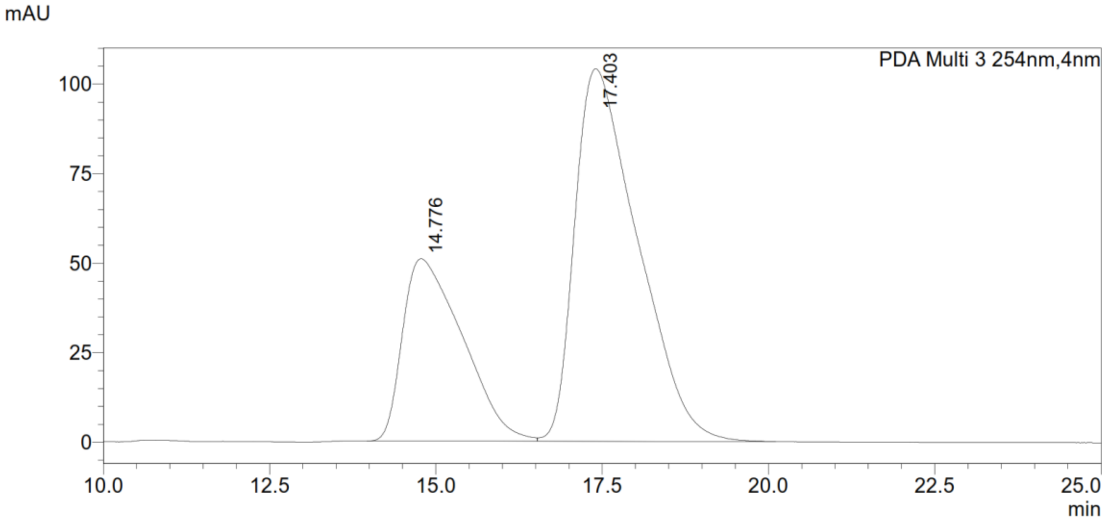

Racemic	Enantioenriched

Racemic — PDA Ch3 254nm

Peak#	Ret. Time	Area%
1	14.982	48.394
2	17.606	51.606
Total		100.000

Enantioenriched — PDA Ch3 254nm

Peak#	Ret. Time	Area%
1	14.776	31.787
2	17.403	68.213
Total		100.000

Fig. S83: Chiral HPLC analysis for (3*S*)-3-(2-(4-fluorophenyl)but-3-en-2-yl)-3-hydroxy-1-methylindolin-2-one (**S14**), ChiralCel® OJ-H, 94:6 Hexane:IPA flowrate 1 mL·min^{-1}, 211 nm, t_R (3*S*,2''*R*)-**S14** 13.0 min, t_R (3*R*,2''*S*)-**S14** 24.3 min, 71:29 er, t_R (3*R*,2''*R*)-**S14** 18.8 min, t_R (3*S*,2''*S*)-**S14** 27.9 min, 40:60 e.r.

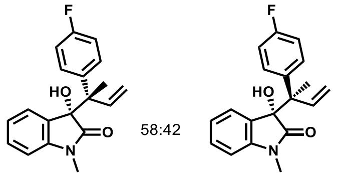

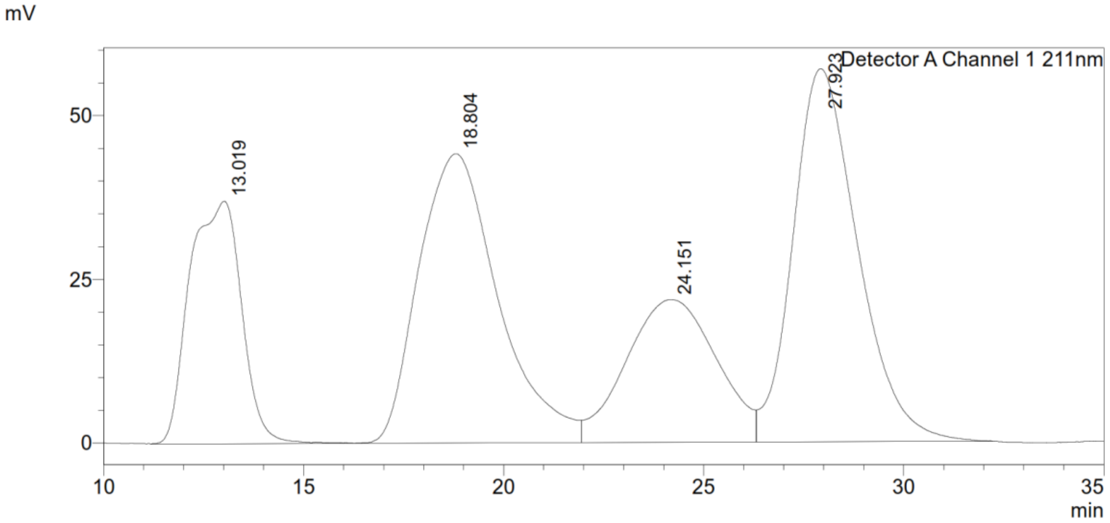

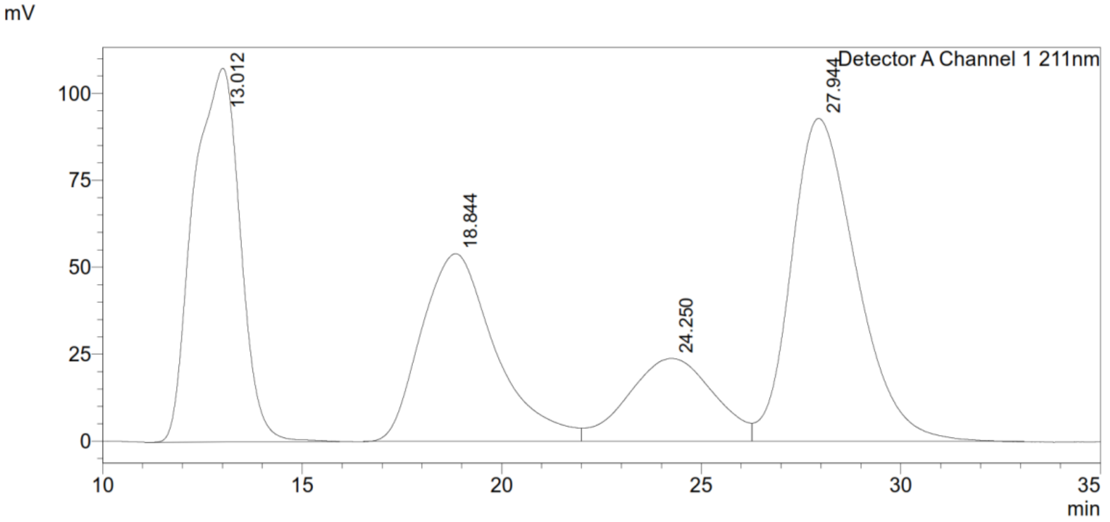

Racemic

Detector A Channel 1 211nm

Peak#	Ret. Time	Area%
1	13.019	17.362
2	18.804	31.654
3	24.151	17.707
4	27.923	33.277
Total		100.000

Enantioenriched

Detector A Channel 1 211nm

Peak#	Ret. Time	Area%
1	13.012	29.428
2	18.844	23.376
3	24.250	11.843
4	27.944	35.353
Total		100.000

Fig. S84: Chiral HPLC analysis for (*S*,*E*)-1-benzyl-3-(3-(4-fluorophenyl)but-2-en-1-yl)-3-hydroxyindolin-2-one (**S18**), ChiralCel® AS-H, 95:5 Hexanes:IPA, flowrate 1 mL·min^{-1}, 211 nm, t_R (*R*)-**S18** 15.9 min, t_R (*S*)-**S18** 21.2 min, 19:81 e.r.

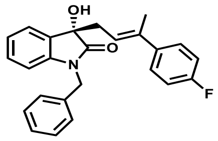

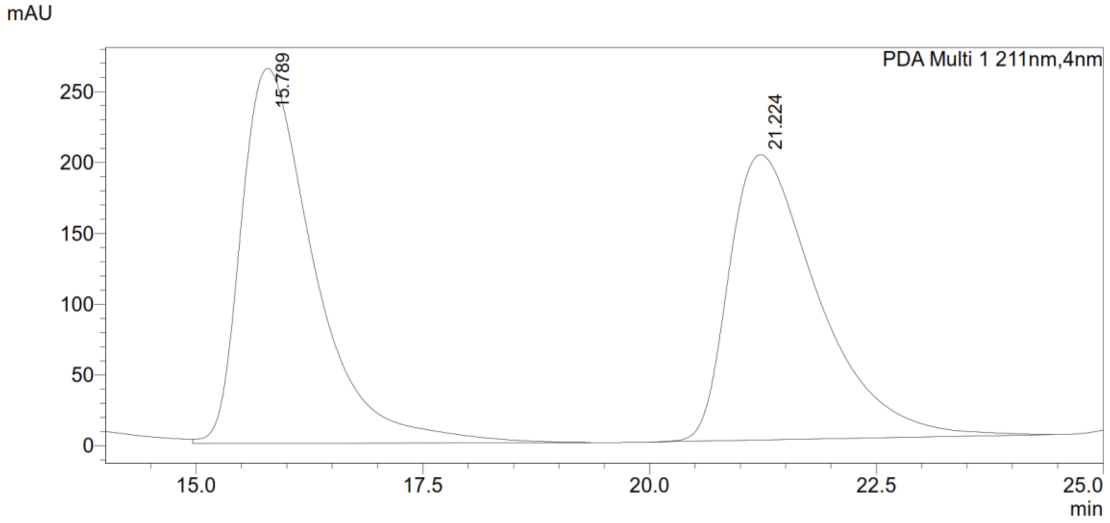

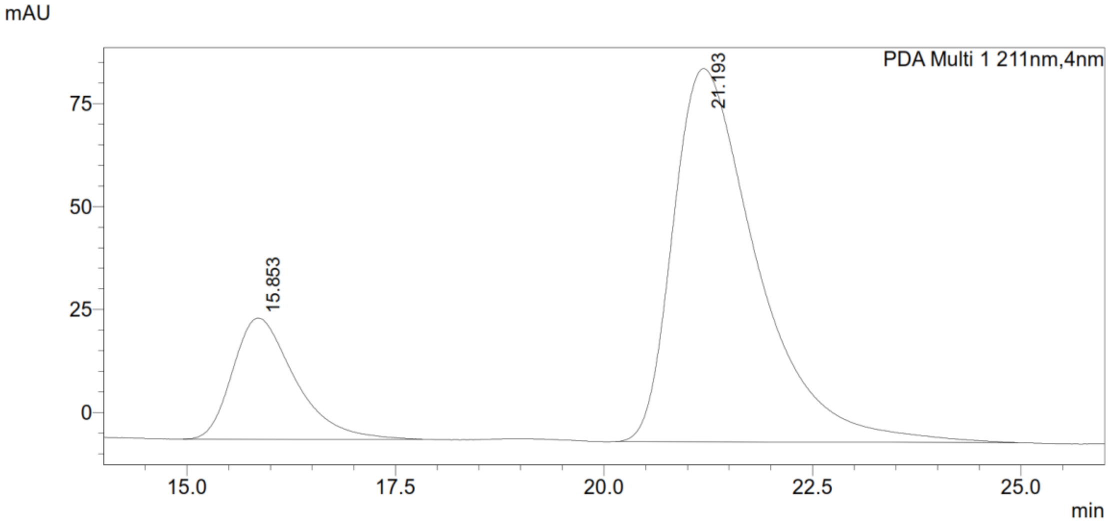

Racemic			Enantioenriched		
PDA Ch1 211nm			PDA Ch1 211nm		
Peak#	Ret. Time	Area%	Peak#	Ret. Time	Area%
1	15.789	51.669	1	15.853	19.346
2	21.224	48.331	2	21.193	80.654
Total		100.000	Total		100.000

Fig. S85: Chiral HPLC analysis for (3*S*)-1-benzyl-3-(2-(4-fluorophenyl)but-3-en-2-yl)-3-hydroxyindolin-2-one (**S17**), ChiralCel® AD-H, 95:5 Hexane:IPA, flowrate 1 mL·min^{-1}, 211 nm, t_R (3*R*,2''*R*)-**S17** 16.6 min, t_R (3*S*,2''*S*)-**S17** 24.7 min, 21:79 e.r.; t_R (3*R*,2''*S*)-**S17** 18.4 min, t_R (3*S*,2''*R*)-**S17** 21.6 min, 21:79 e.r.

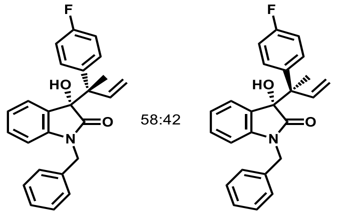

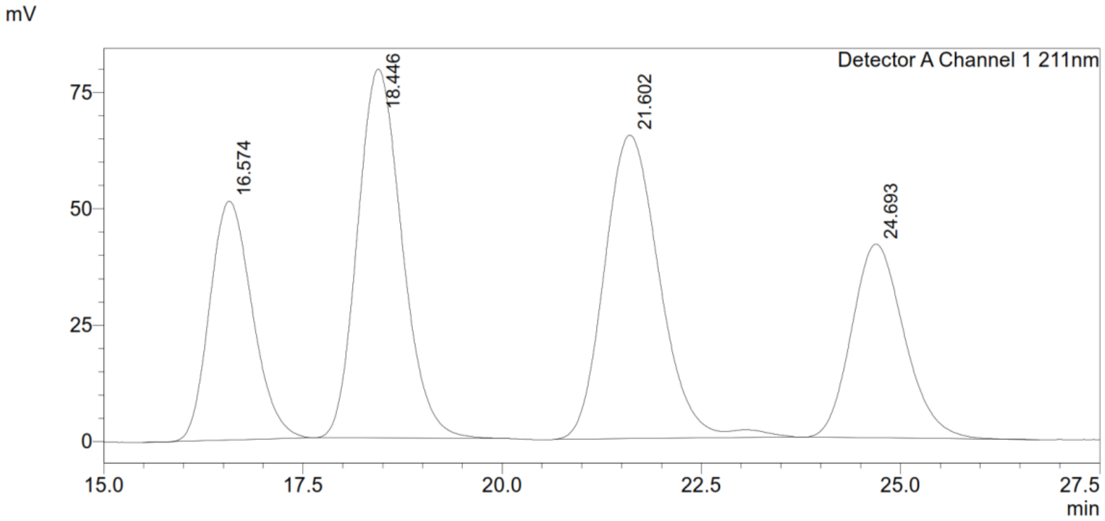

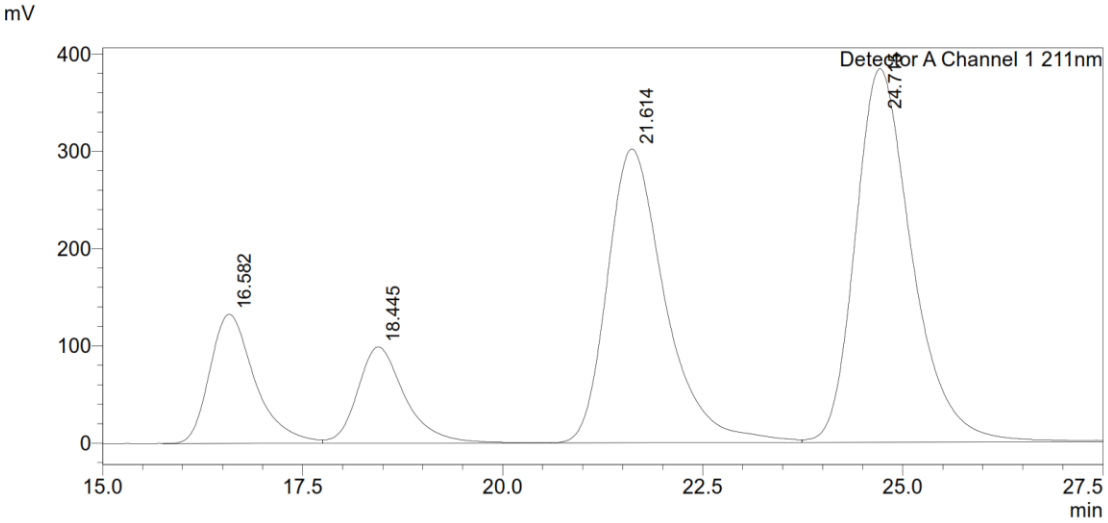

Racemic			Enantioenriched		
Detector A Channel 1 211nm			Detector A Channel 1 211nm		
Peak#	Ret. Time	Area%	Peak#	Ret. Time	Area%
1	16.574	18.967	1	16.582	11.968
2	18.446	30.668	2	18.445	9.372
3	21.602	31.323	3	21.614	34.871
4	24.693	19.042	4	24.715	43.789
Total		100.000	Total		100.000

Fig. S86: Chiral HPLC analysis for ethyl (3*R*,2''*S*)-2-(3-hydroxy-2-oxo-1-tritylindolin-3-yl)-2-methylbut-3-enoate (**S19**), ChiralPak® IC, 95:5 Hexane:IPA, flowrate 1 mL·min^{-1}, 254 nm, t_R (3S,2''*R*)-**S19** 11.0 min, t_R (3*R*,2''*S*)-**S19** 18.2 min, 11:89 e.r.

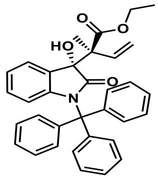

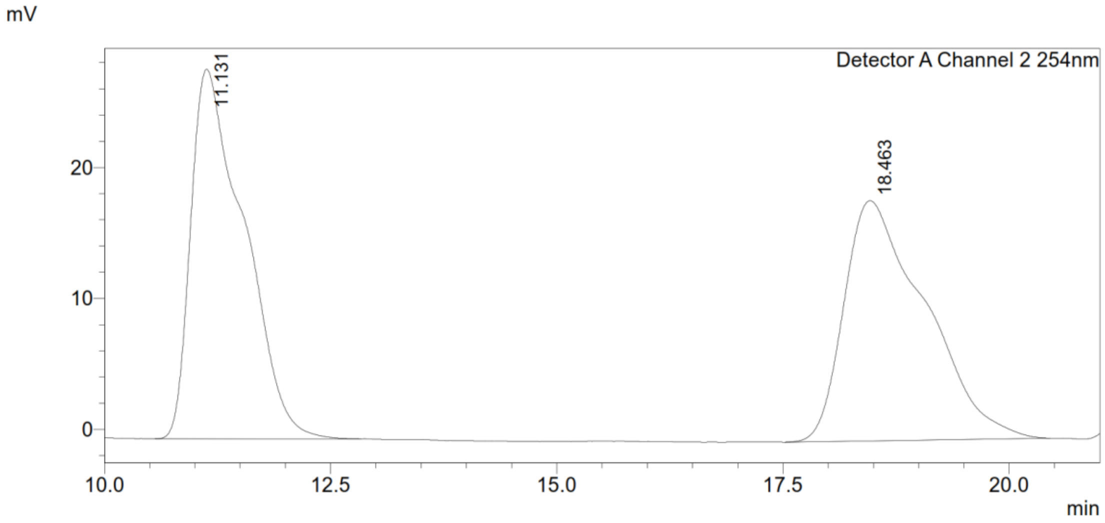

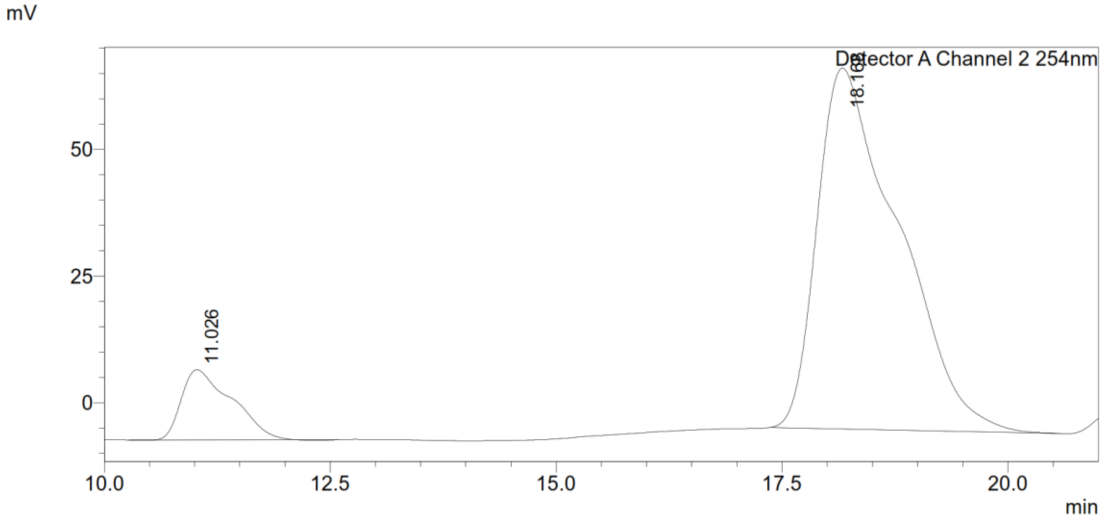

Racemic			Enantioenriched		
Detector A Channel 2 254nm			Detector A Channel 2 254nm		
Peak#	Ret. Time	Area%	Peak#	Ret. Time	Area%
1	11.131	50.307	1	11.026	10.714
2	18.463	49.693	2	18.168	89.286
Total		100.000	Total		100.000

Fig. S87: Chiral HPLC analysis for ethyl (*E*)-4-((3-hydroxy-2,2'-dioxo-1,1'-ditrityl-[3,3'-biindolin]-3'-yl)oxy)tiglate (**68**), ChiralCel® OD-H, 95:5 Hexane:IPA, flowrate 1 mL·min^{-1}, 211 nm, t_R major-**68** 7.7 min, t_R minor-**68** 16.7 min, 75:25 e.r.

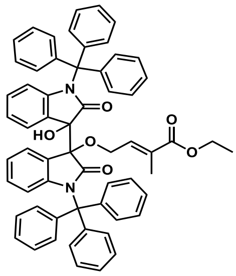

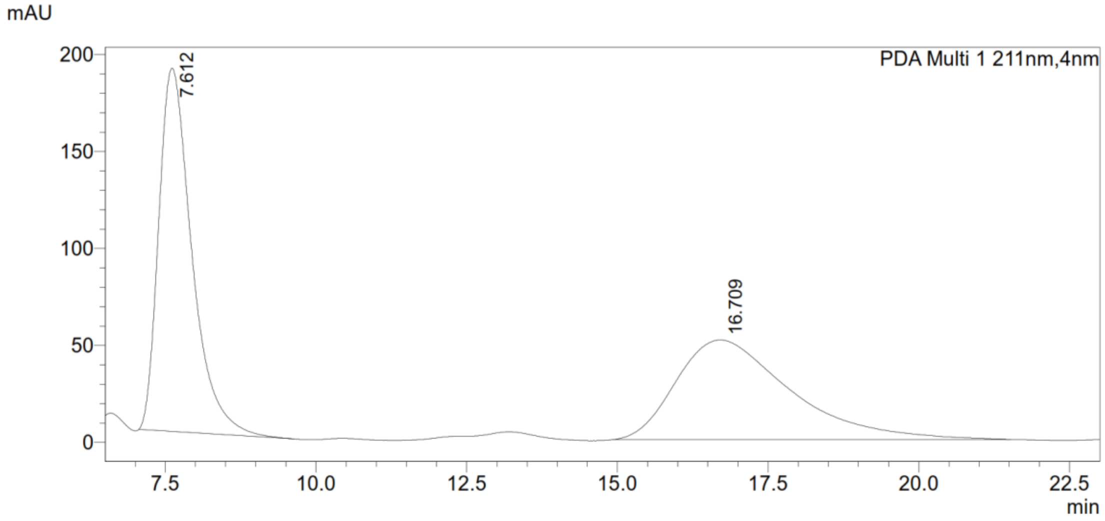

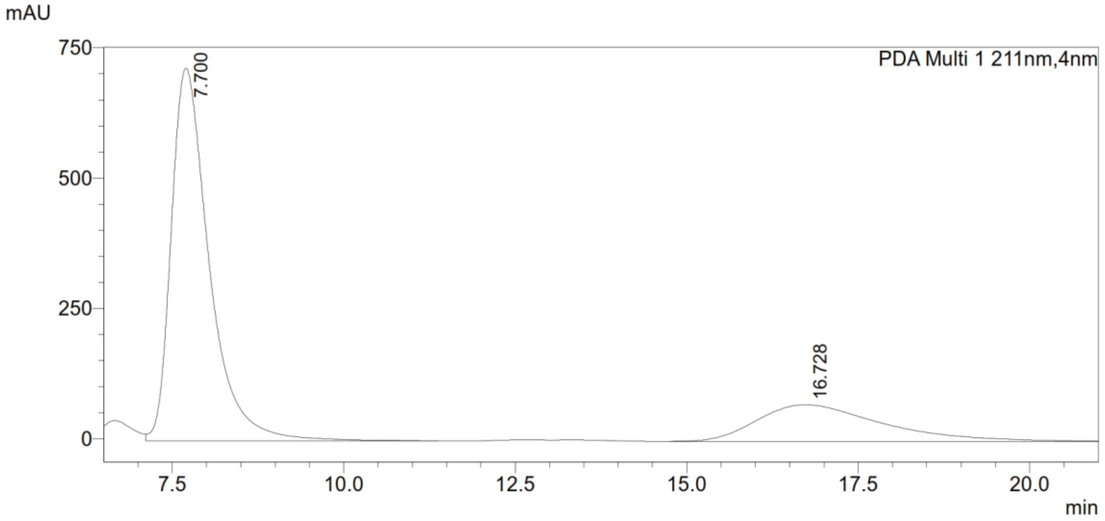

Racemic

PDA Ch1 211nm

Peak#	Ret. Time	Area%
1	7.612	51.367
2	16.709	48.633
Total		100.000

Enantioenriched

PDA Ch1 211nm

Peak#	Ret. Time	Area%
1	7.700	75.442
2	16.728	24.558
Total		100.000

Fig. S88: Chiral HPLC analysis for ethyl (*S*,*E*)-4-(3-hydroxy-2-oxo-1-tritylindolin-3-yl)tiglate (**67**), ChiralCel® OD-H, 95:5 Hexane:IPA, flowrate 1 mL·min^{-1}, 211 nm, t_R (*R*)-**67** 10.0 min, t_R (*S*)-**67** 14.4 min, 2:98 e.r.

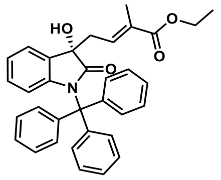

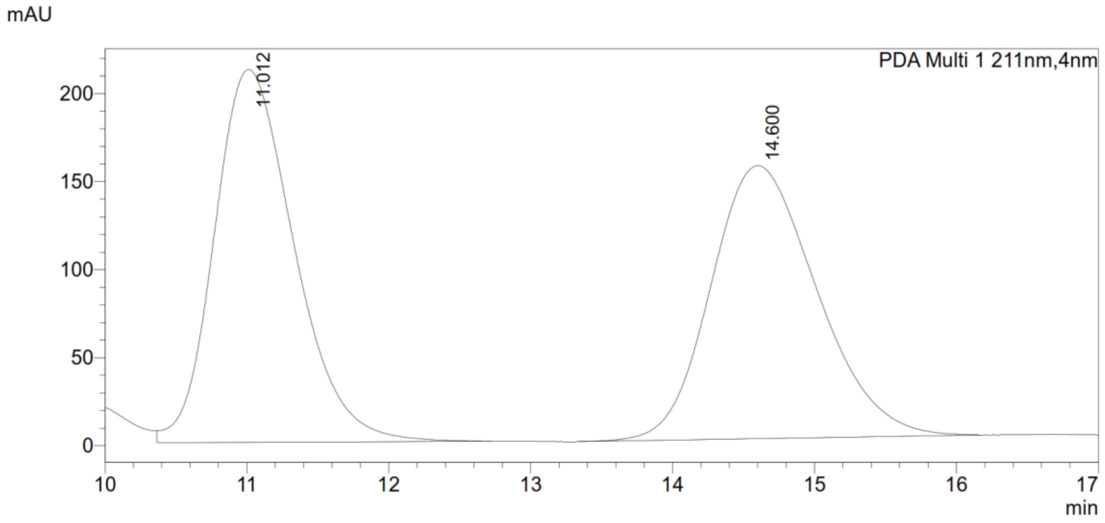

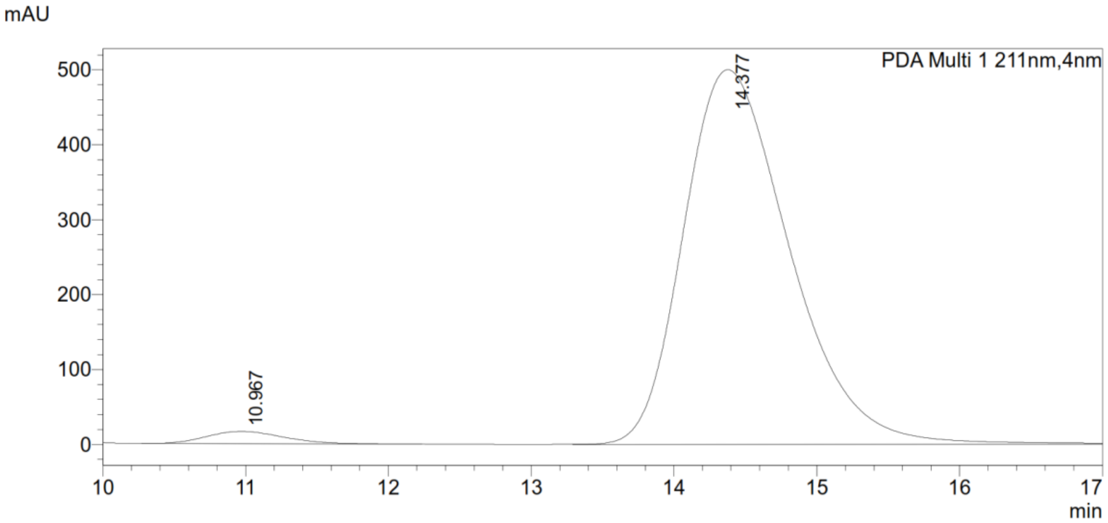

Racemic			Enantioenriched		
PDA Ch1 211nm			PDA Ch1 211nm		
Peak#	Ret. Time	Area%	Peak#	Ret. Time	Area%
1	11.012	50.692	1	10.967	2.335
2	14.600	49.308	2	14.377	97.665
Total		100.000	Total		100.000

10 Supplementary NMR spectral data of compounds

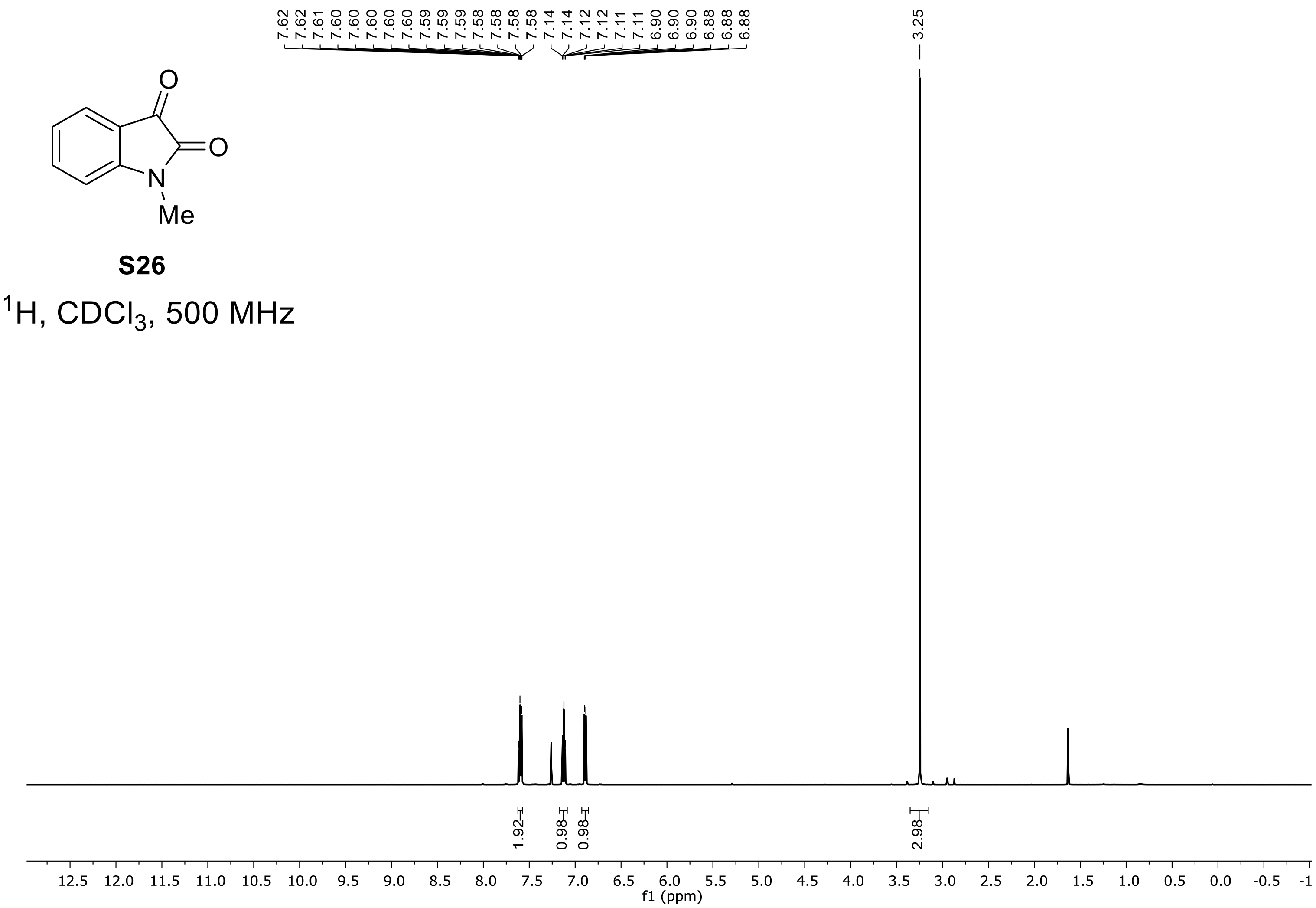

Fig. S89: ^{1}H NMR spectrum for 1-methylindoline-2,3-dione (**S26**).

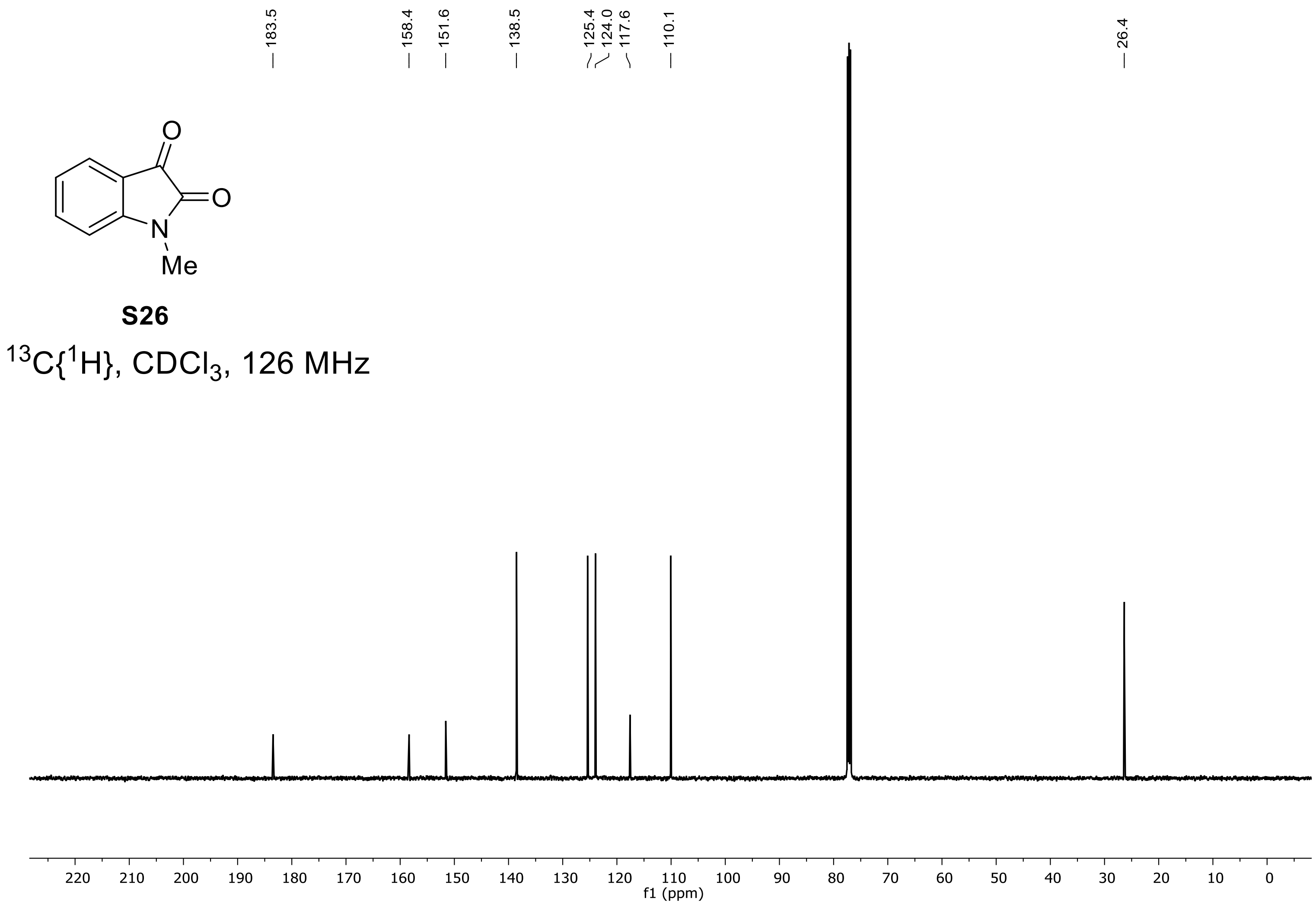

Fig. S90: ^{13}C NMR spectrum for 1-methylindoline-2,3-dione (**S26**).

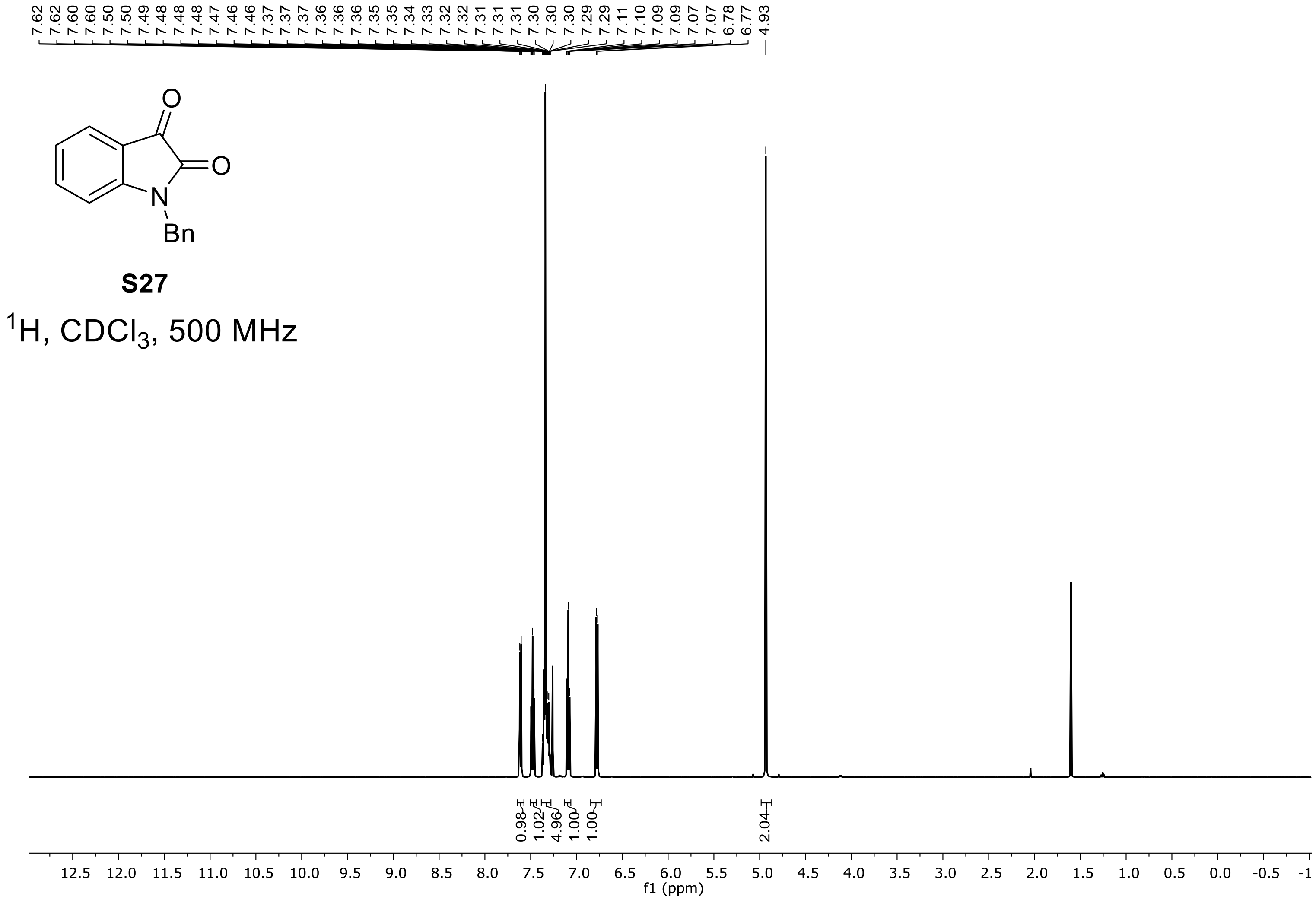

Fig. S91: ^{1}H NMR spectrum for 1-benzylindoline-2,3-dione (**S27**).

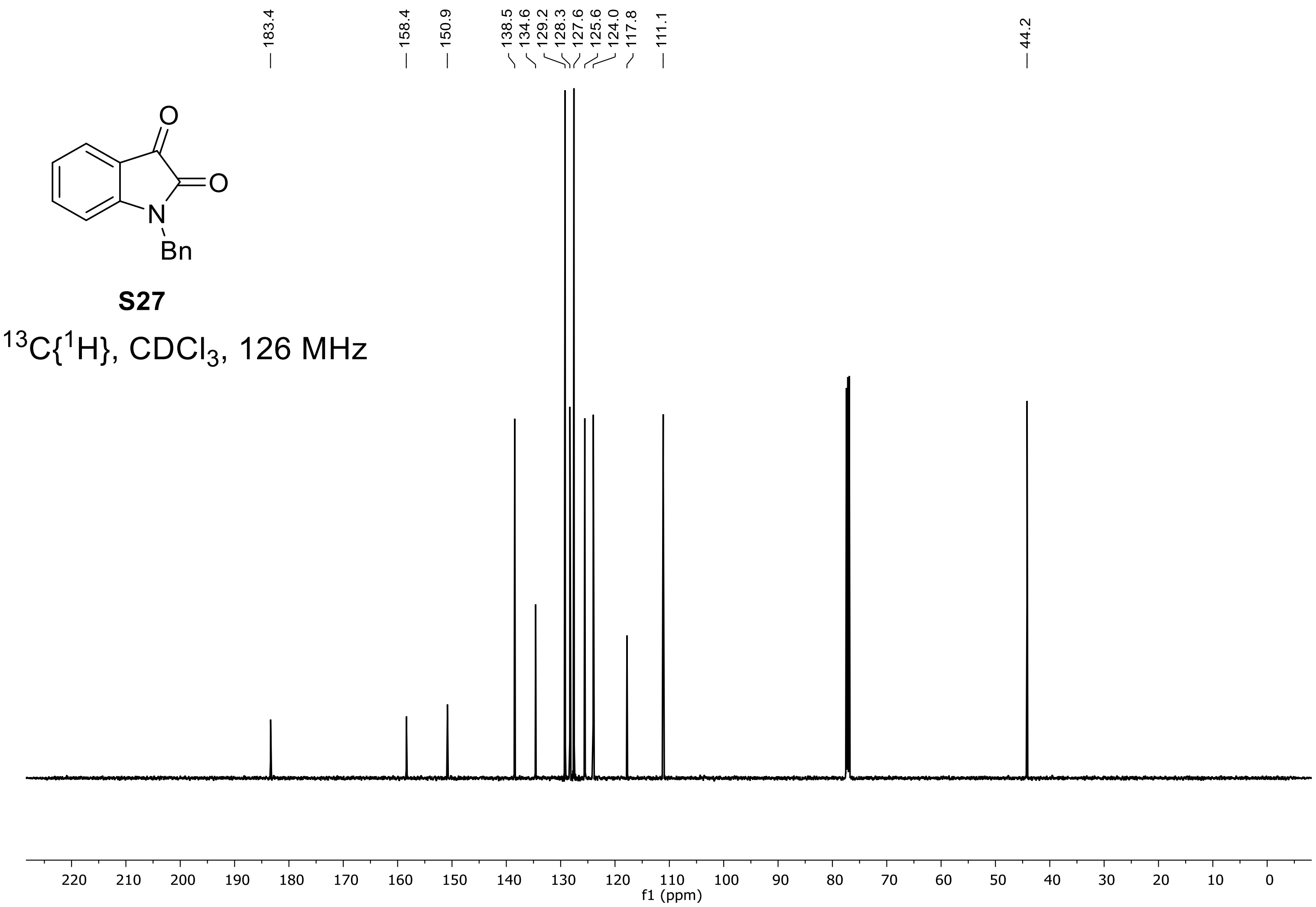

Fig. S92: ^{13}C NMR spectrum for 1-benzylindoline-2,3-dione (**S27**).

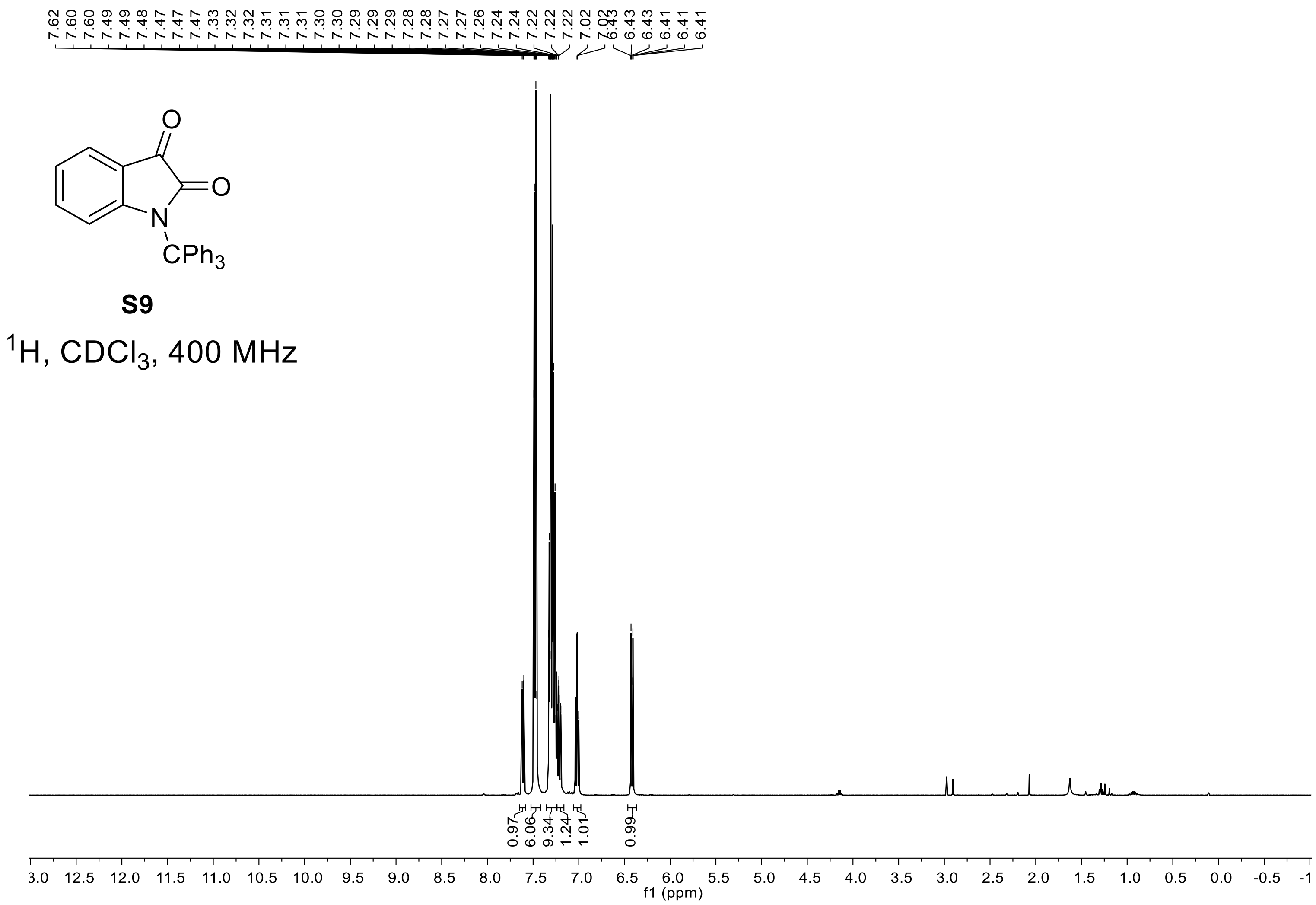

Fig. S93: ^{1}H NMR spectrum for 1-tritylindoline-2,3-dione (**S9**).

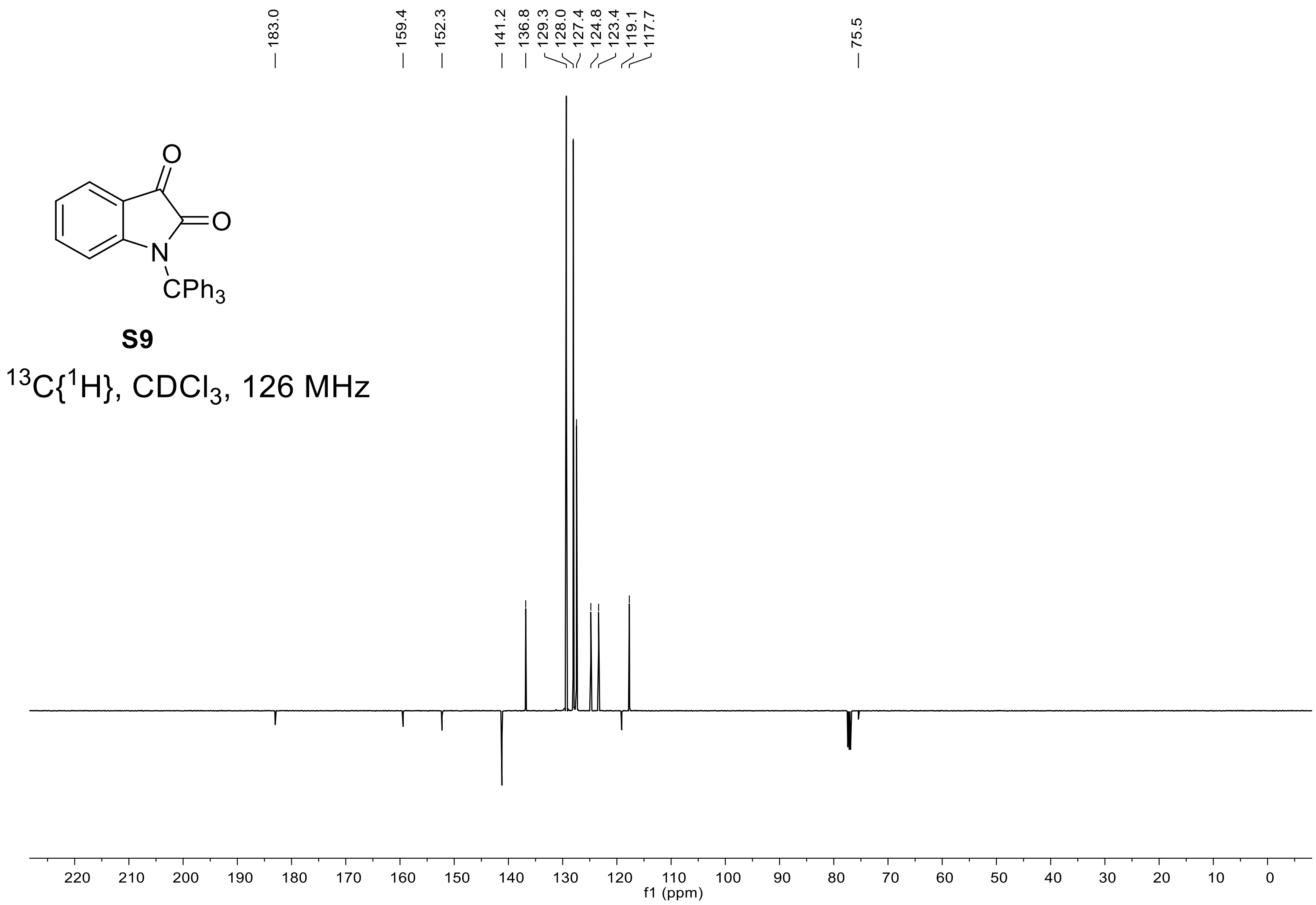

Fig. S94: ^{13}C NMR spectrum for 1-tritylindoline-2,3-dione (**S9**).

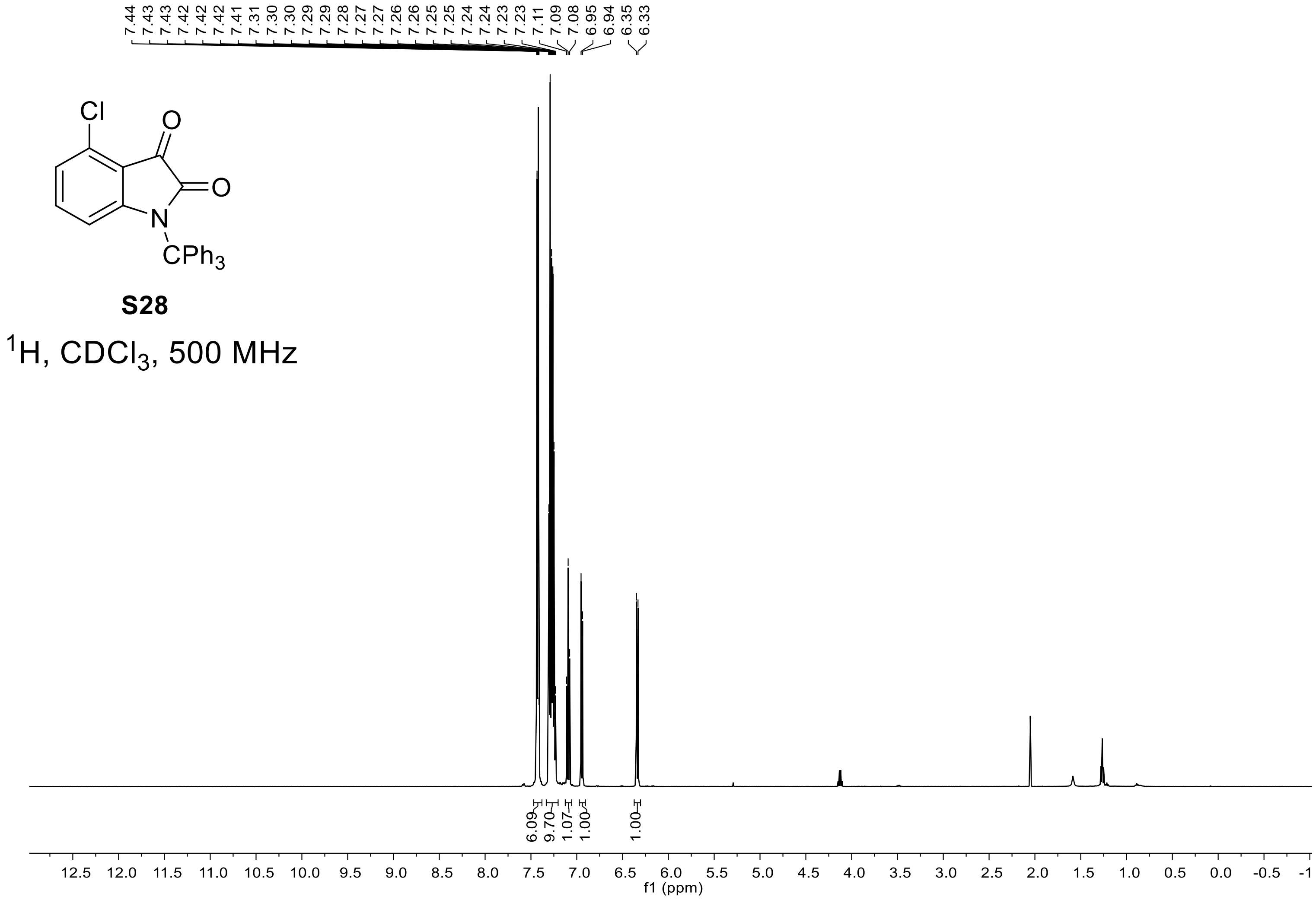

Fig. S95: ^{1}H NMR spectrum for 4-chloro-1-tritylindoline-2,3-dione (**S28**).

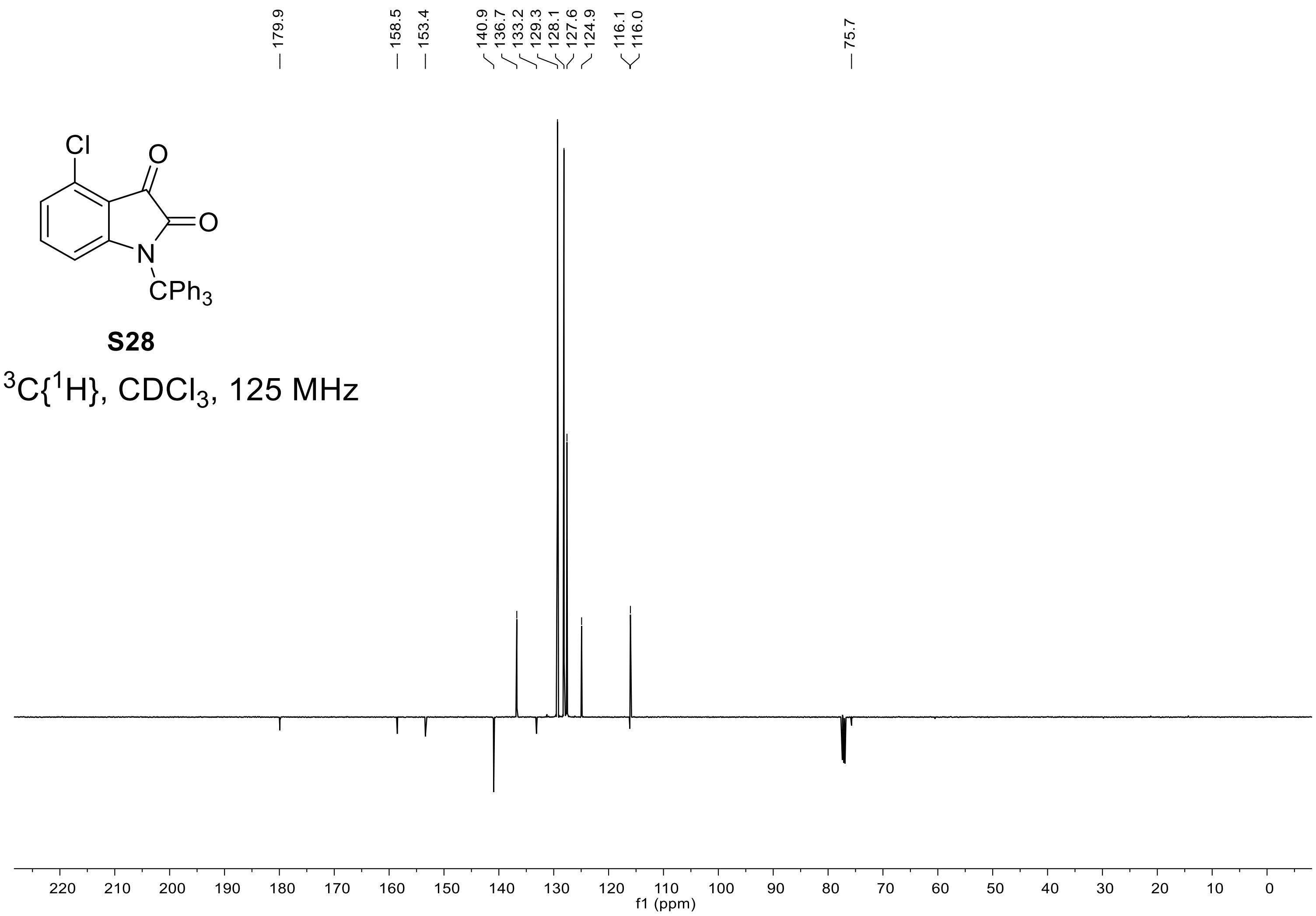

S28

$^{13}C\{^1H\}$, $CDCl_3$, 125 MHz

Fig. S96: ^{13}C NMR spectrum for 4-chloro-1-tritylindoline-2,3-dione (**S28**).

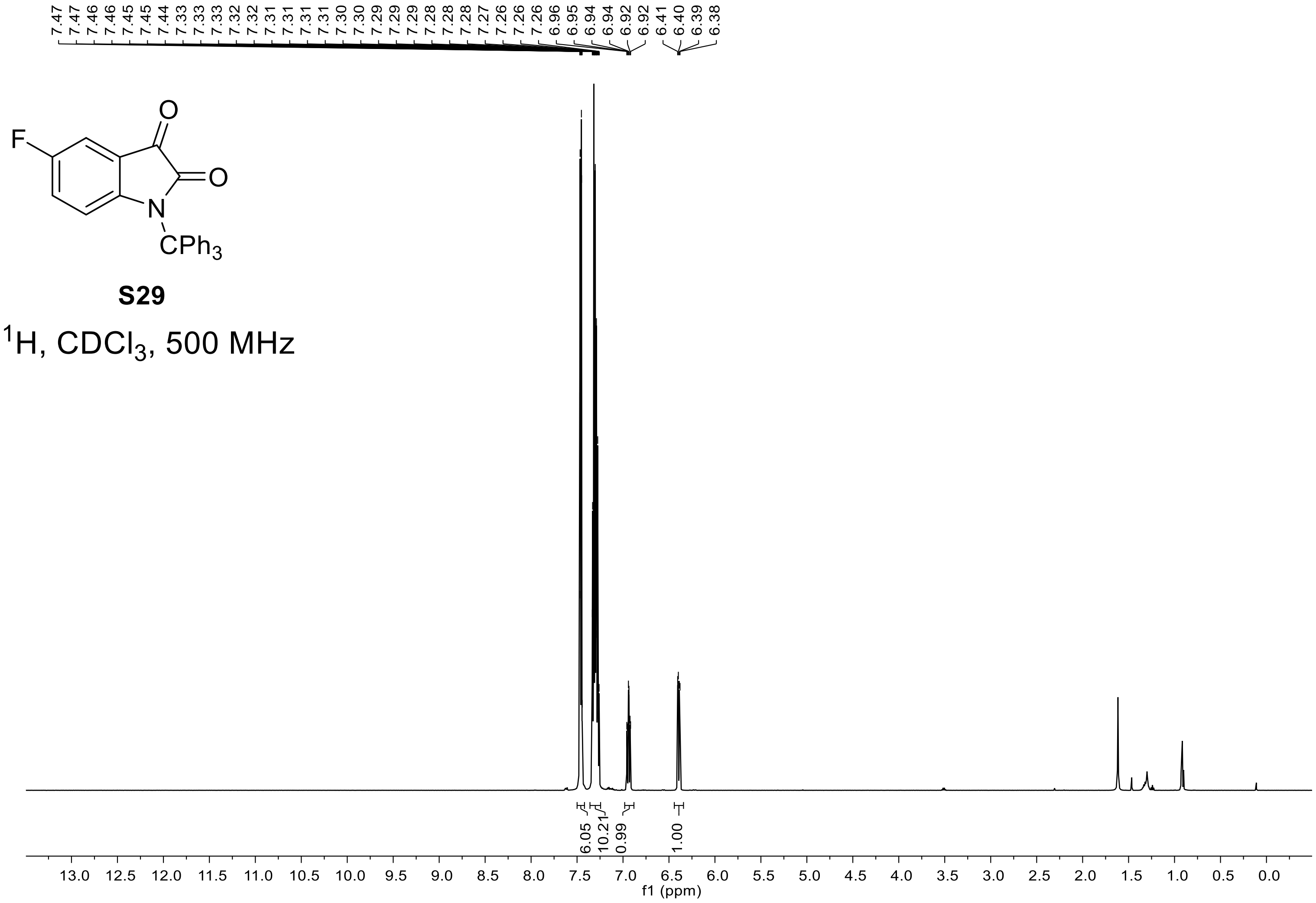

Fig. S97: ^{1}H NMR spectrum for 5-Fluoro-1-tritylindoline-2,3-dione (**S29**).

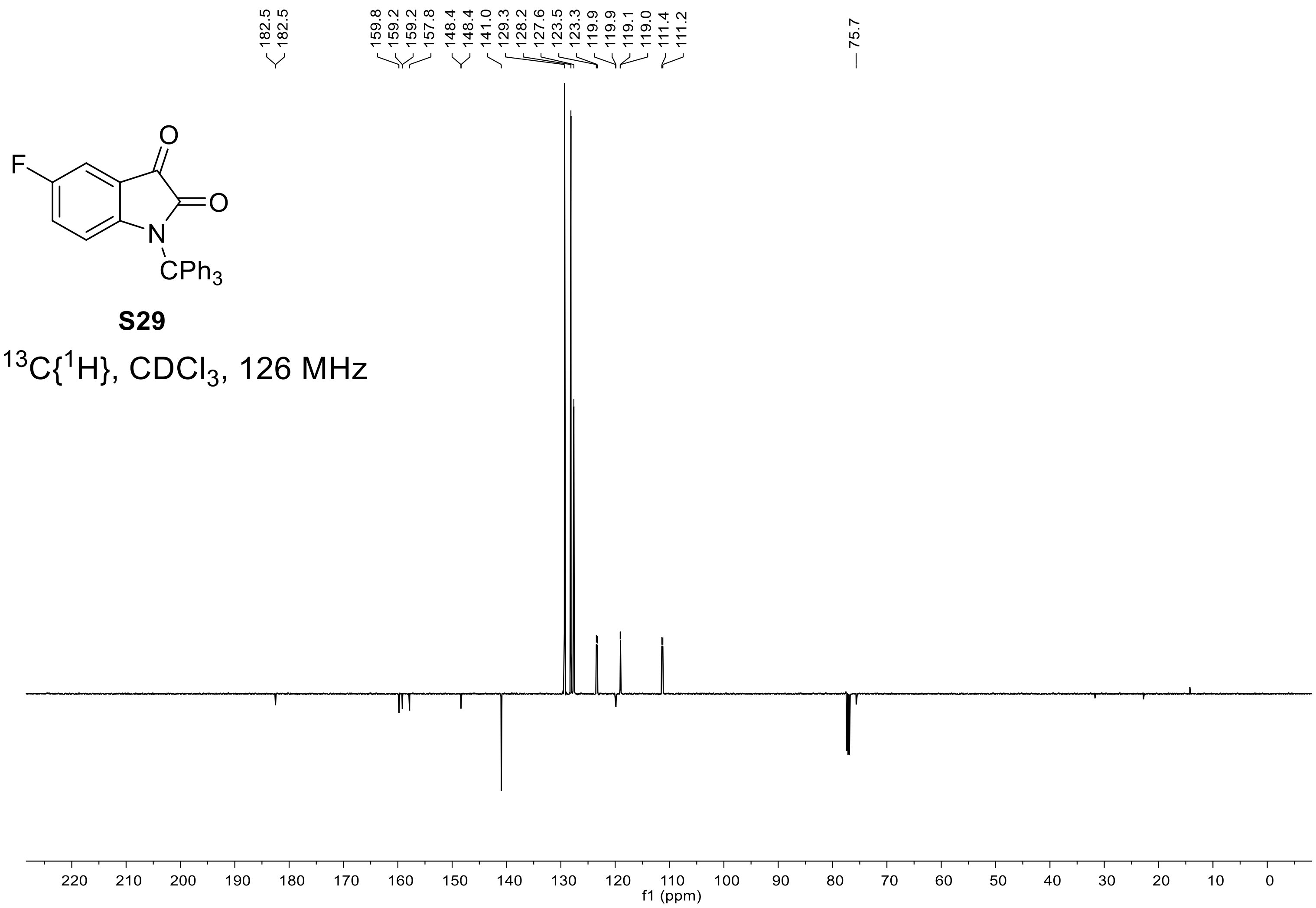

Fig. S98: $^{13}C\{^1H\}$ NMR spectrum for 5-Fluoro-1-tritylindoline-2,3-dione (**S29**).

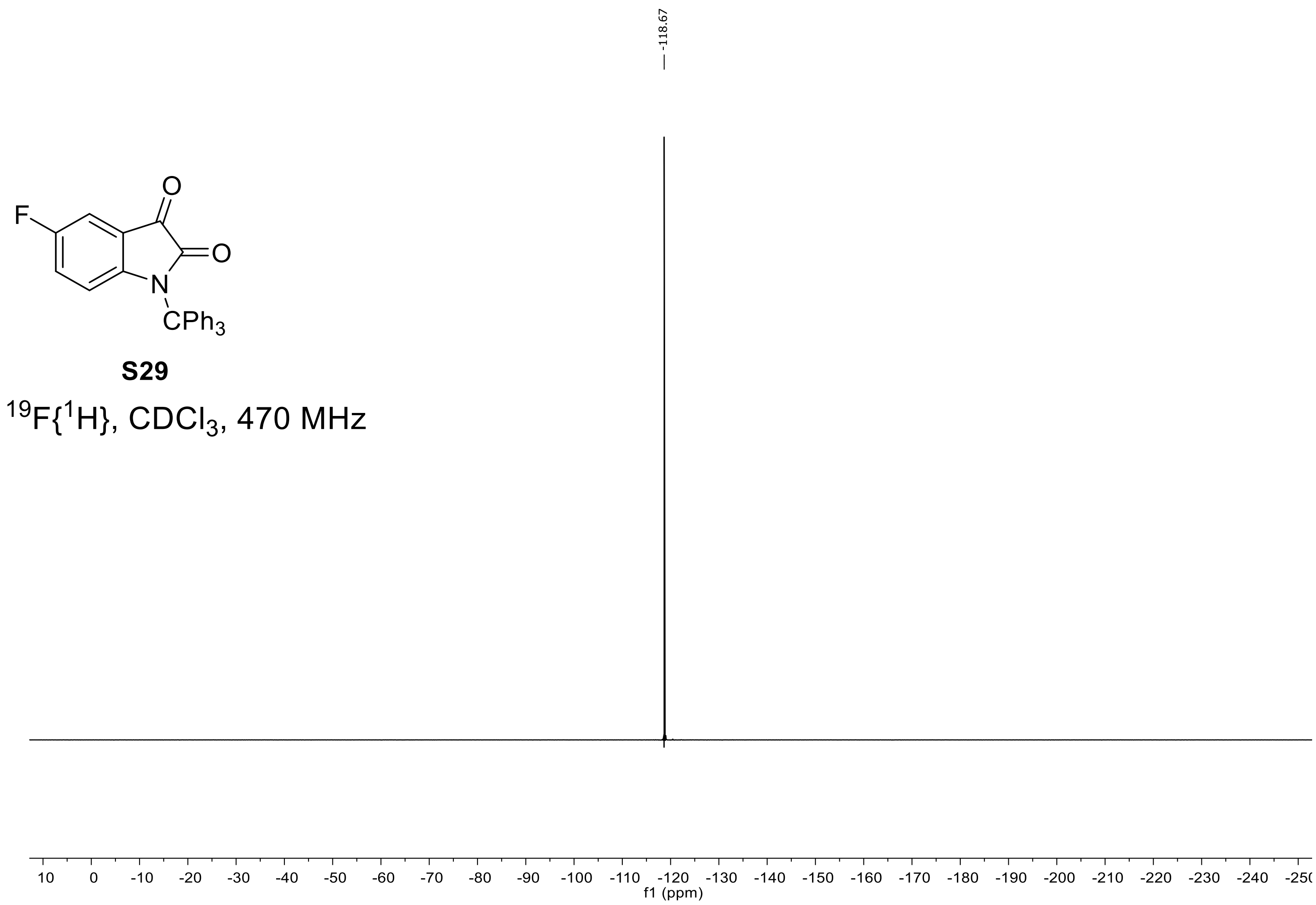

Fig. S99: $^{19}F\{^1H\}$ NMR spectrum for 5-Fluoro-1-tritylindoline-2,3-dione (**S29**).

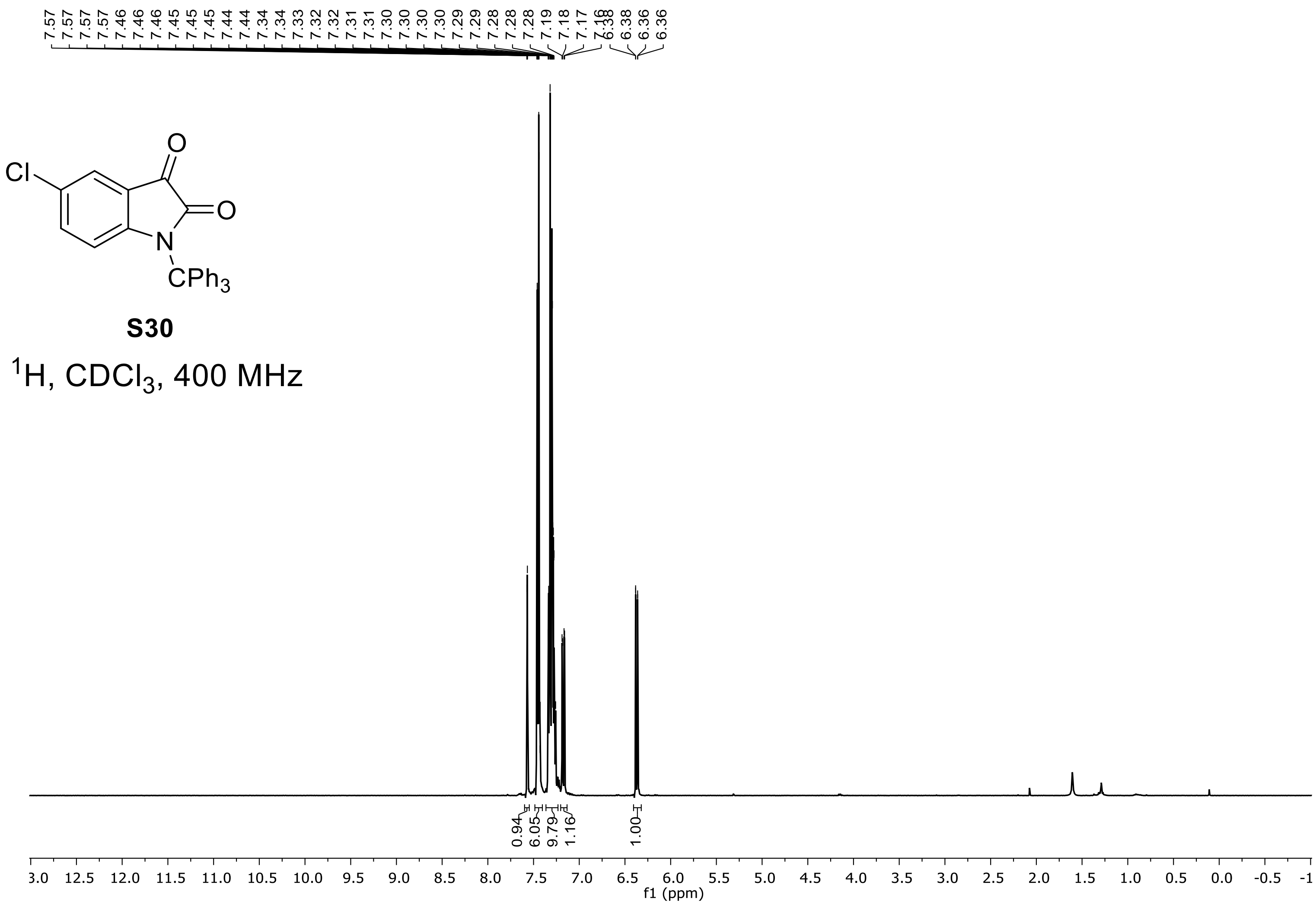

Fig. S100: ^{1}H NMR spectrum for 5-Chloro-1-tritylindoline-2,3-dione (**S30**).

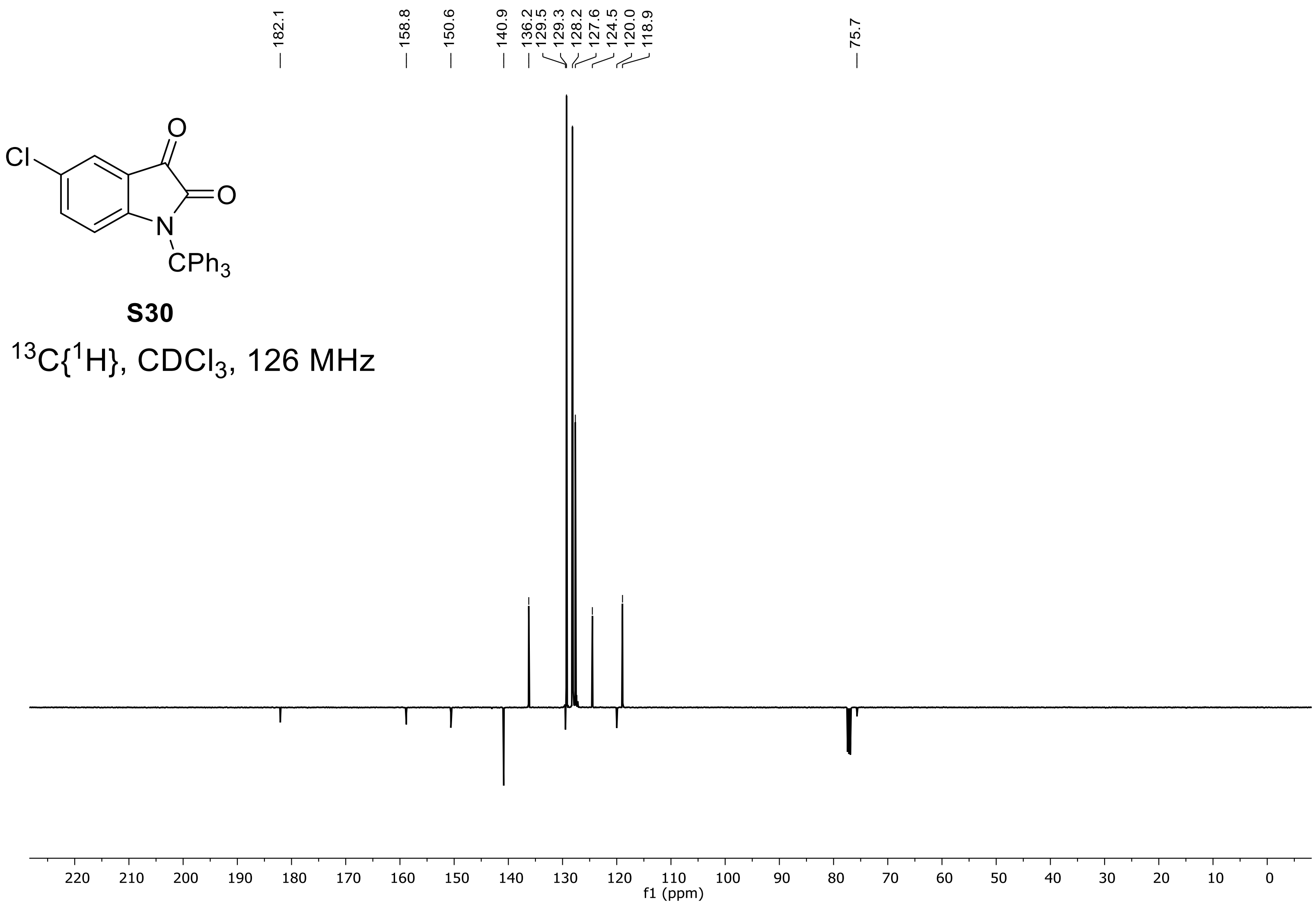

Fig. S101: $^{13}C\{^{1}H\}$ NMR spectrum for 5-Chloro-1-tritylindoline-2,3-dione (**S30**).

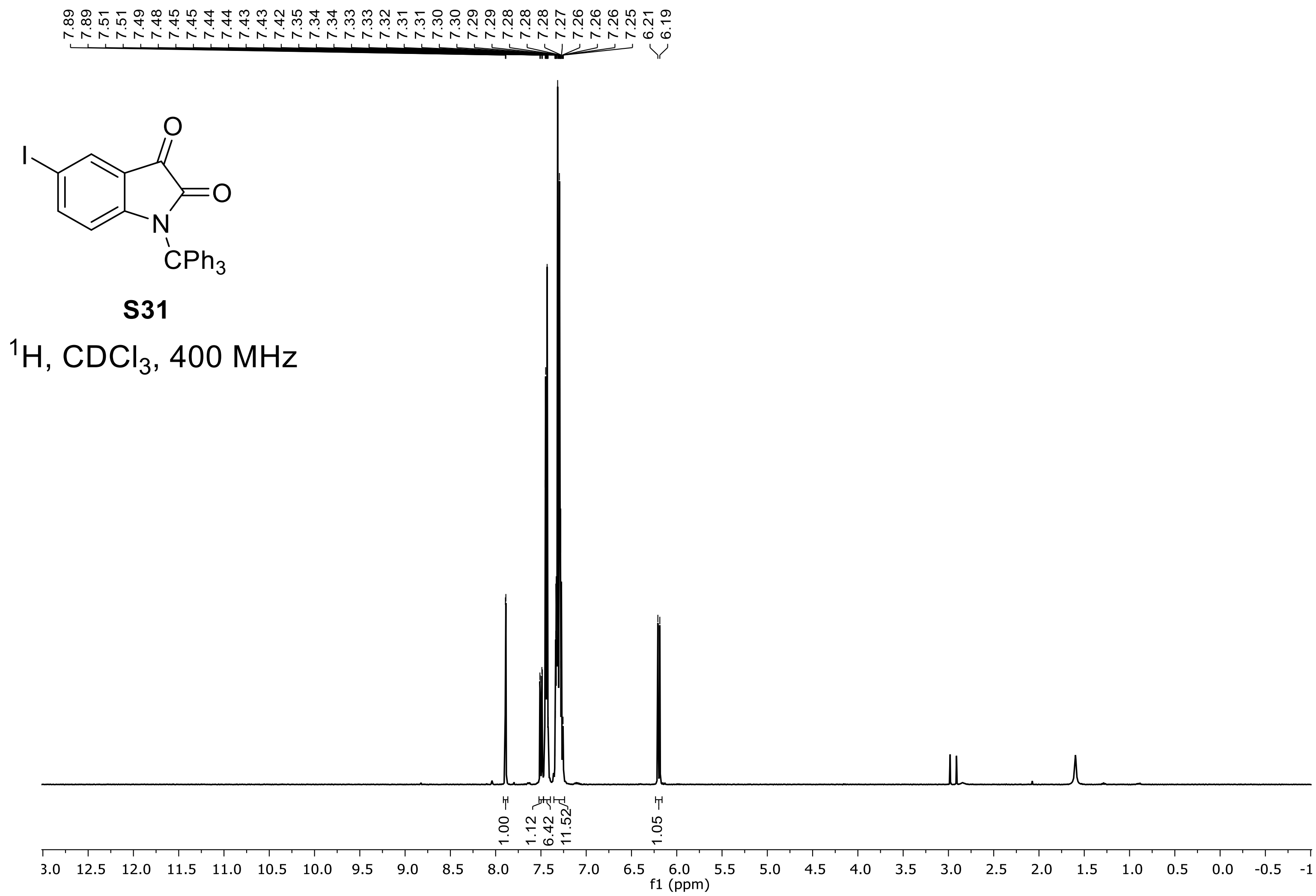

Fig. S102: ^{1}H NMR spectrum for 5-Iodo-1-tritylindoline-2,3-dione (**S31**).

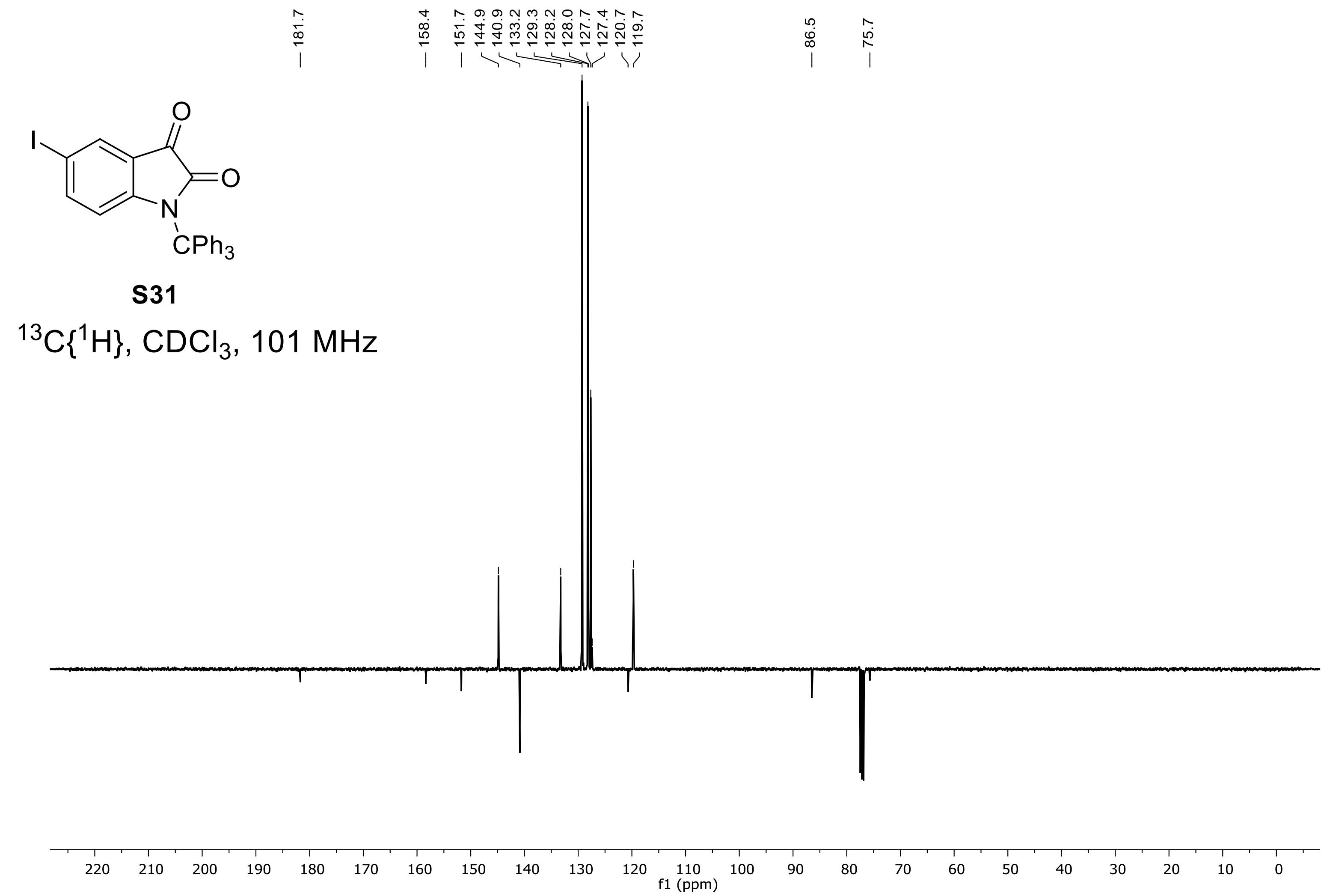

Fig. S103: $^{13}C\{^{1}H\}$ NMR spectrum for 5-Iodo-1-tritylindoline-2,3-dione (**S31**).

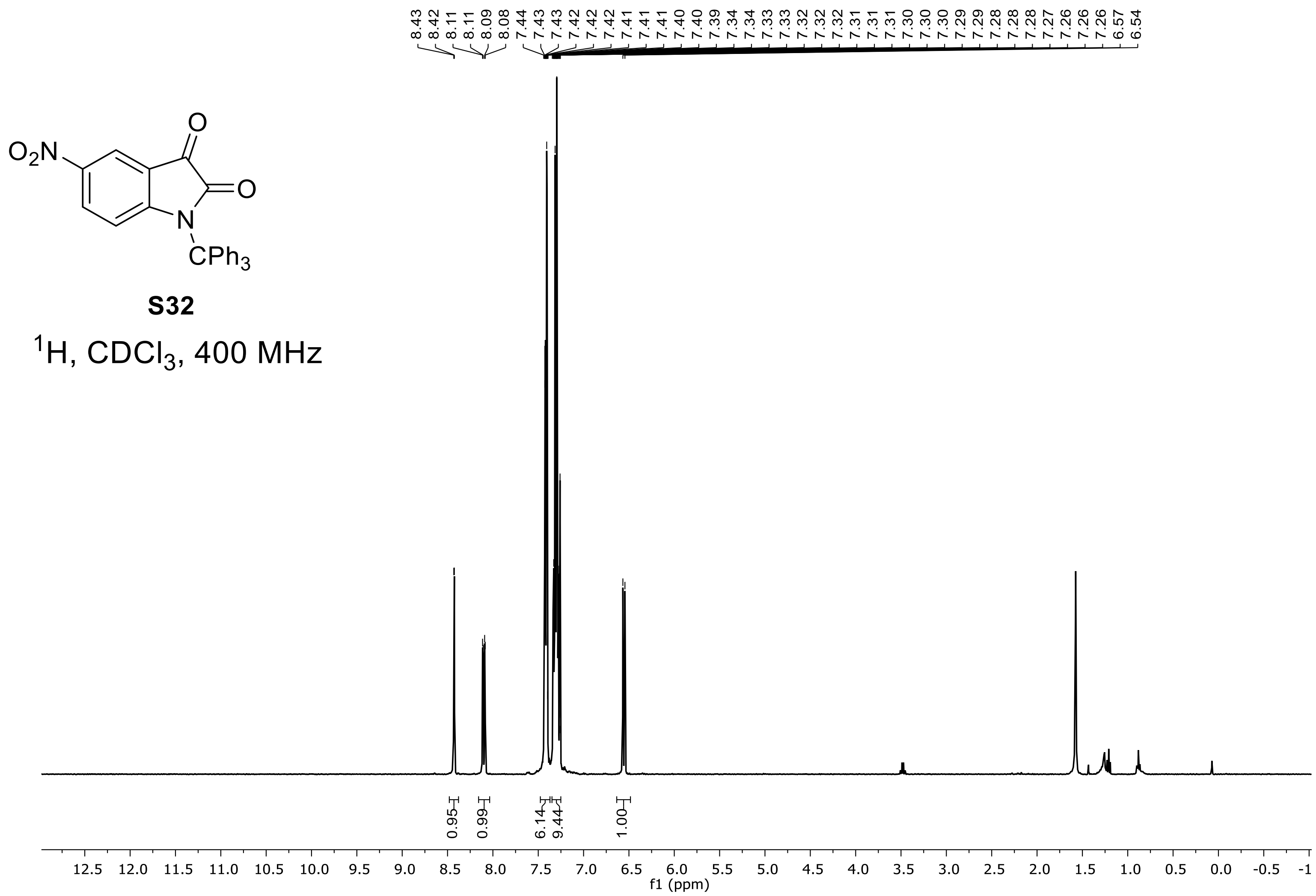

Fig. S104: ^{1}H NMR spectrum for 5-Nitro-1-tritylindoline-2,3-dione (**S32**).

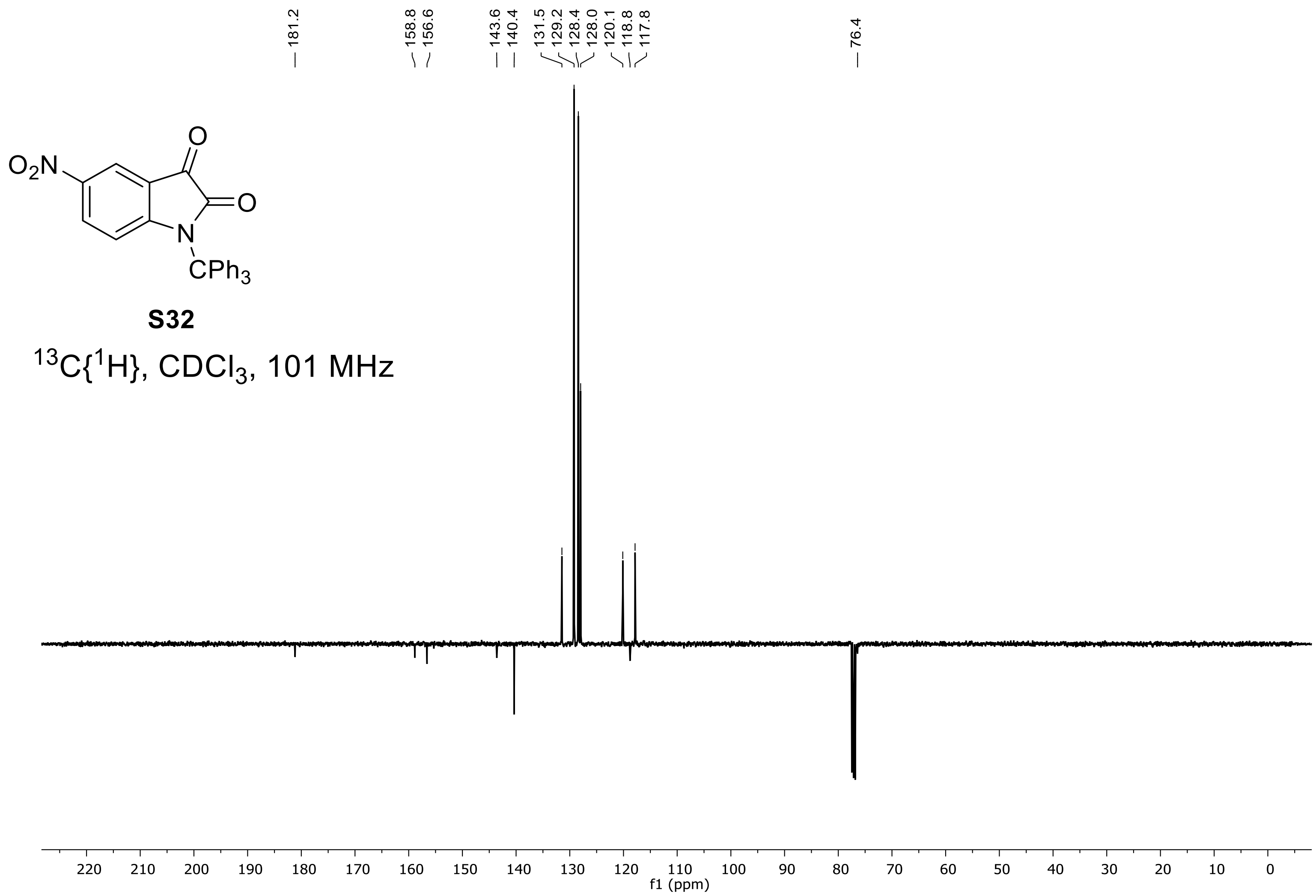

Fig. S105: $^{13}C\{^{1}H\}$ NMR spectrum for 5-Nitro-1-tritylindoline-2,3-dione (**S32**).

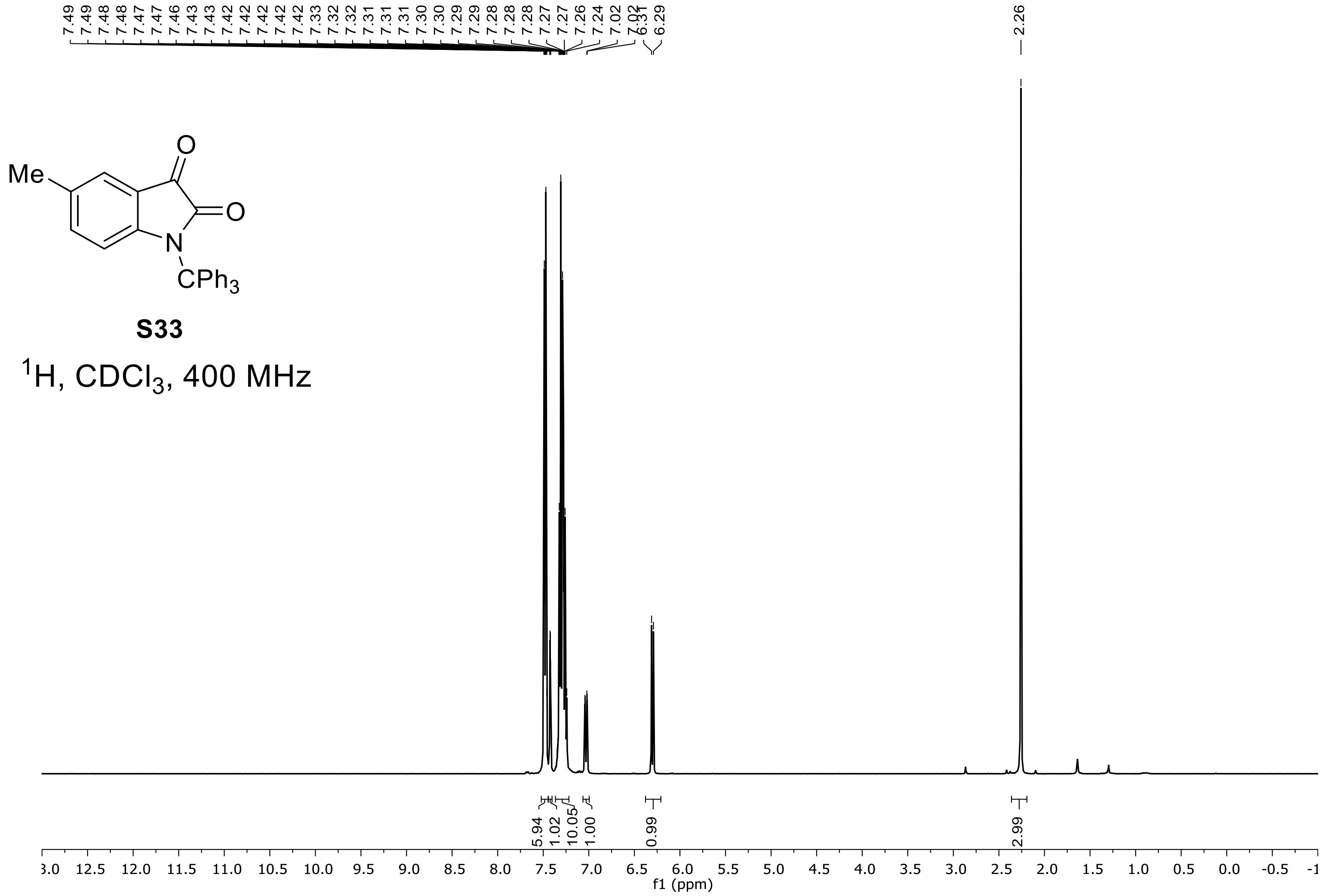

Fig. S106: ^{1}H NMR spectrum for 5-Methyl-1-tritylindoline-2,3-dione (**S33**).

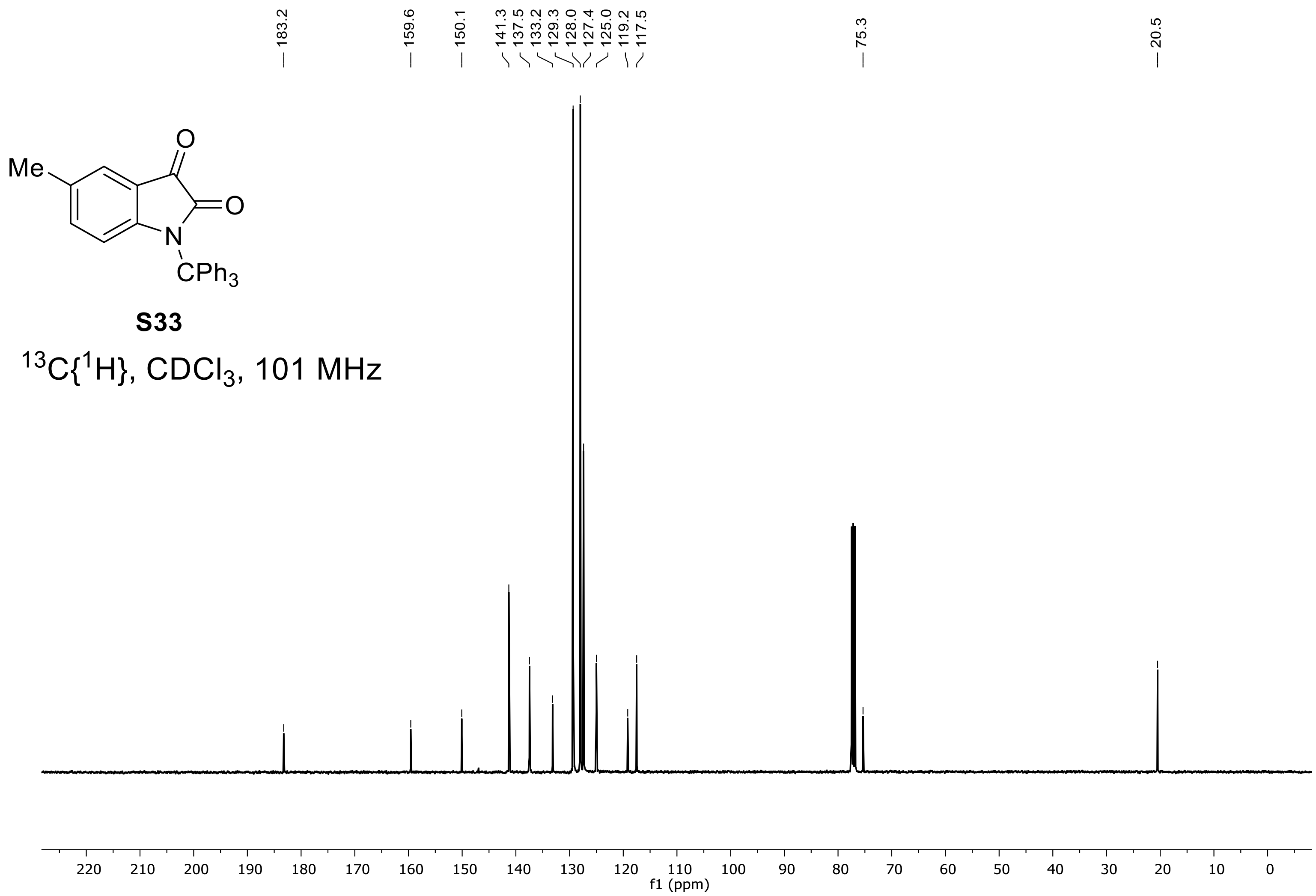

Fig. S107: $^{13}C\{^1H\}$ NMR spectrum for 5-Methyl-1-tritylindoline-2,3-dione (**S33**).

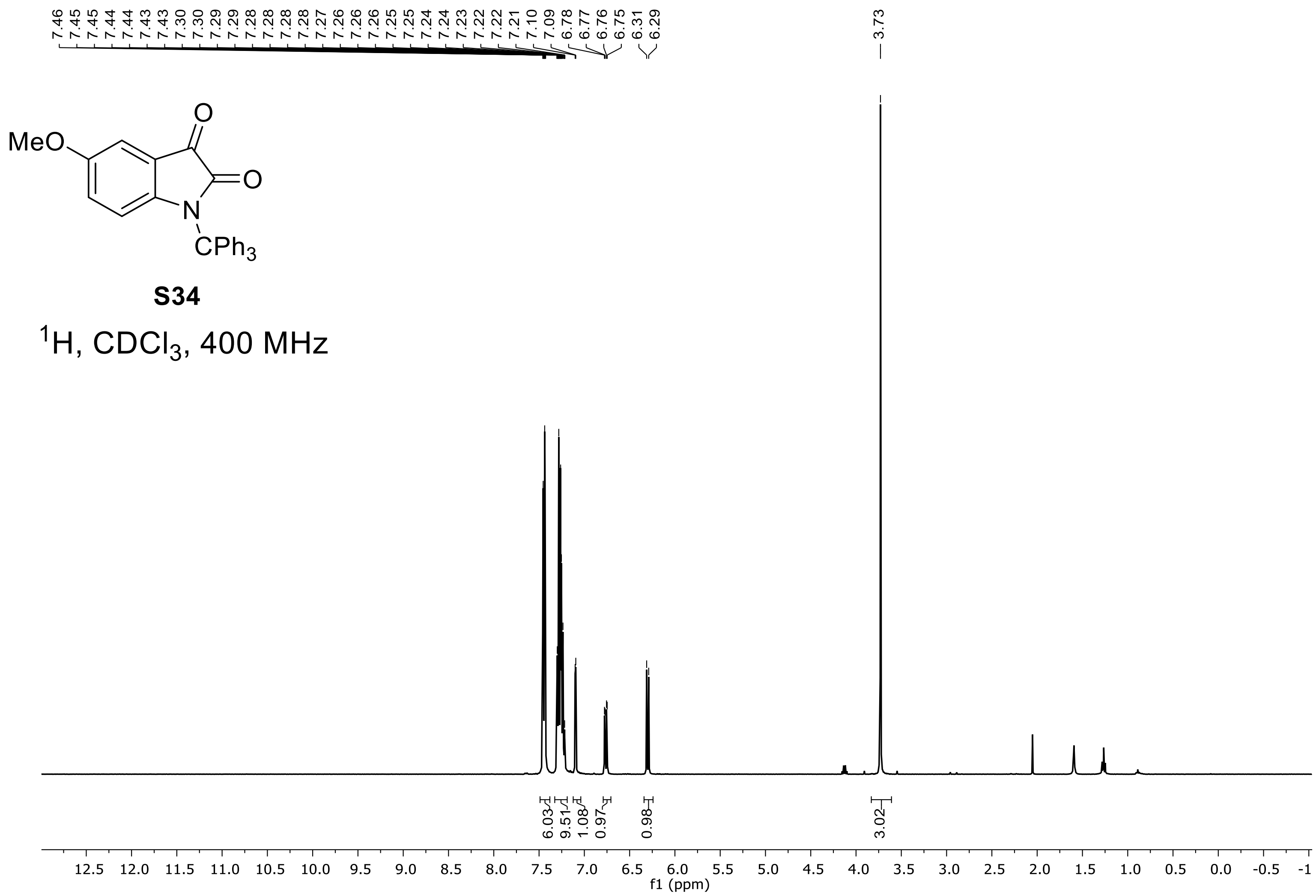

Fig. S108: ^{1}H NMR spectrum for 5-Methoxy-1-tritylindoline-2,3-dione (**S34**).

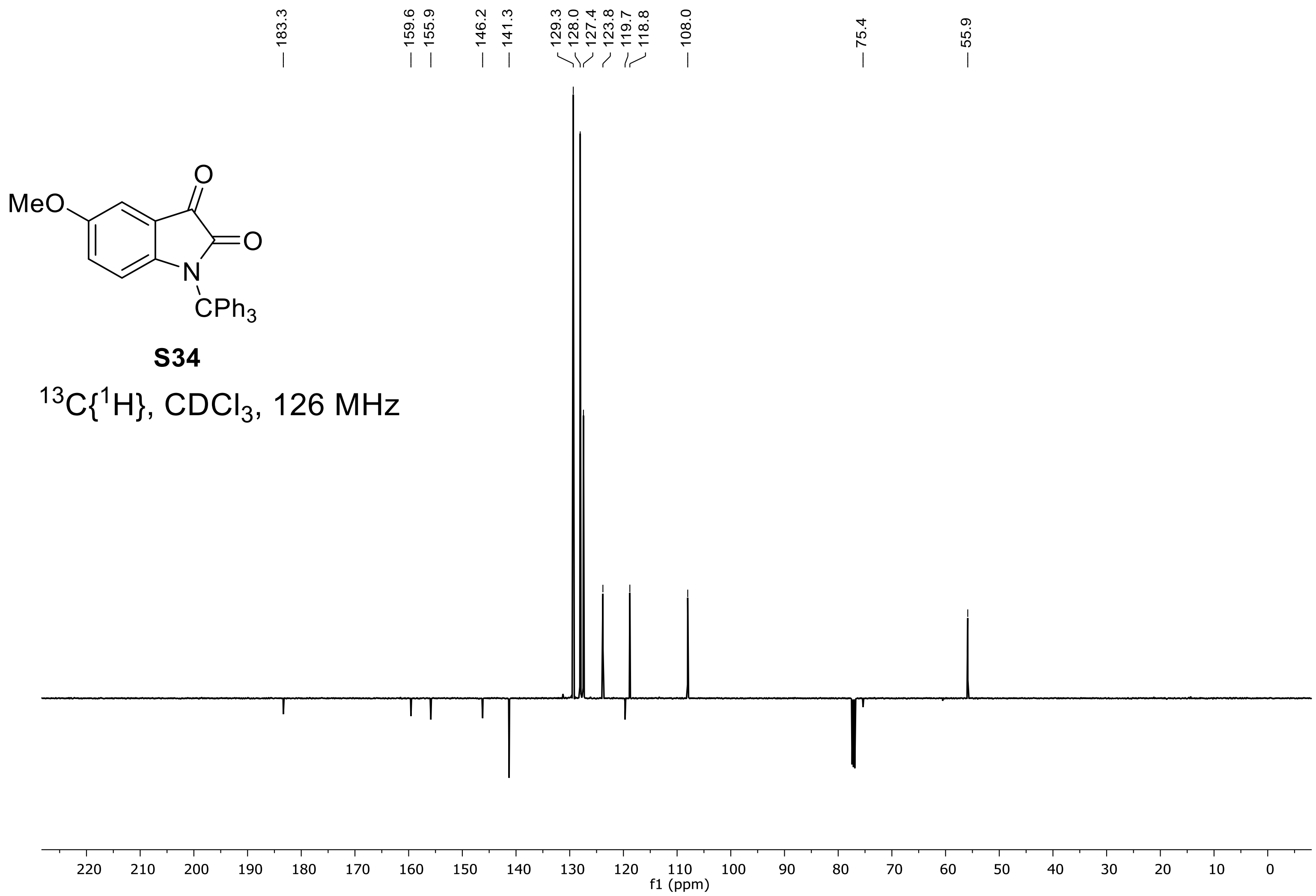

Fig. S109: $^{13}C\{^{1}H\}$ NMR spectrum for 5-Methoxy-1-tritylindoline-2,3-dione (**S34**).

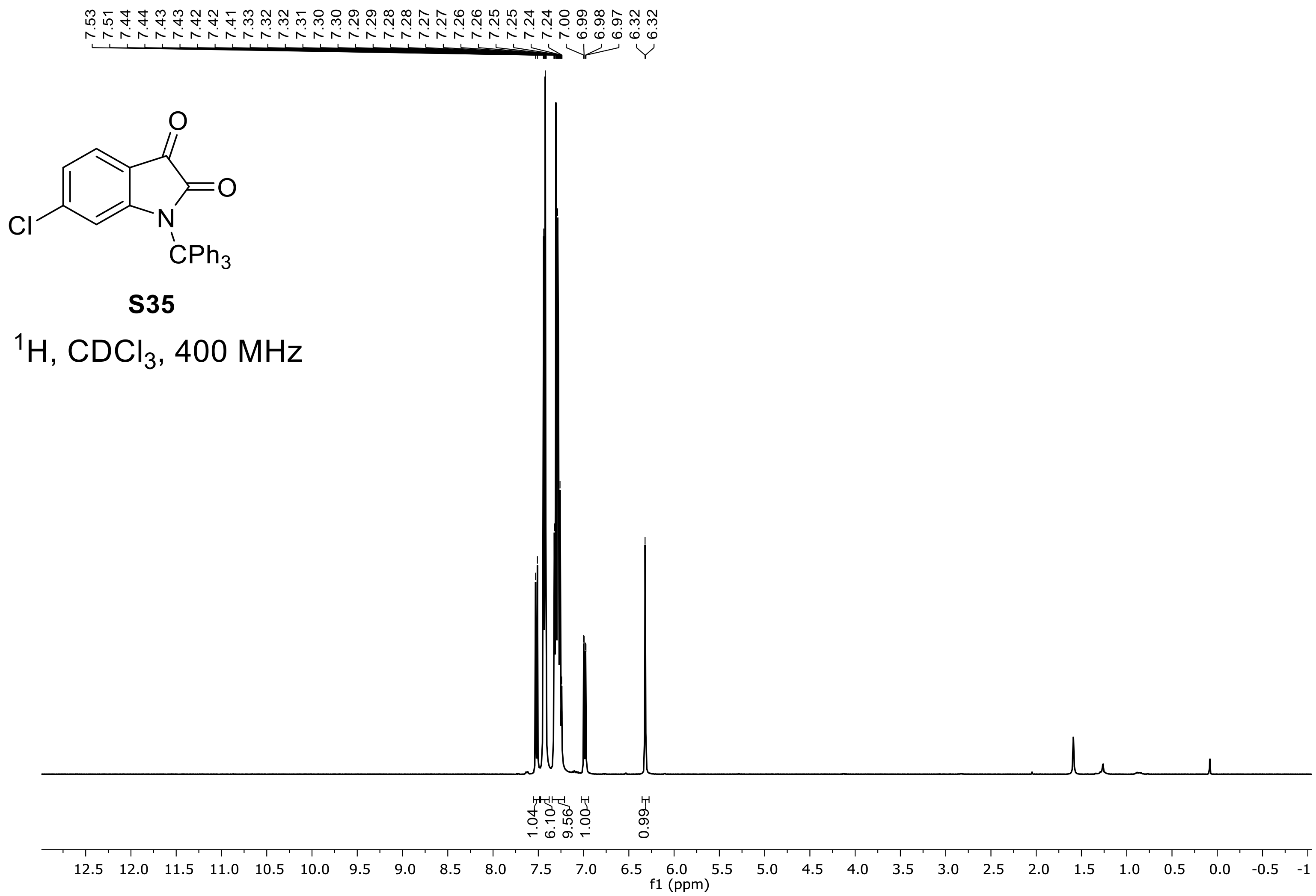

Fig. S110: ^{1}H NMR spectrum for 6-Chloro-1-tritylindoline-2,3-dione (**S35**).

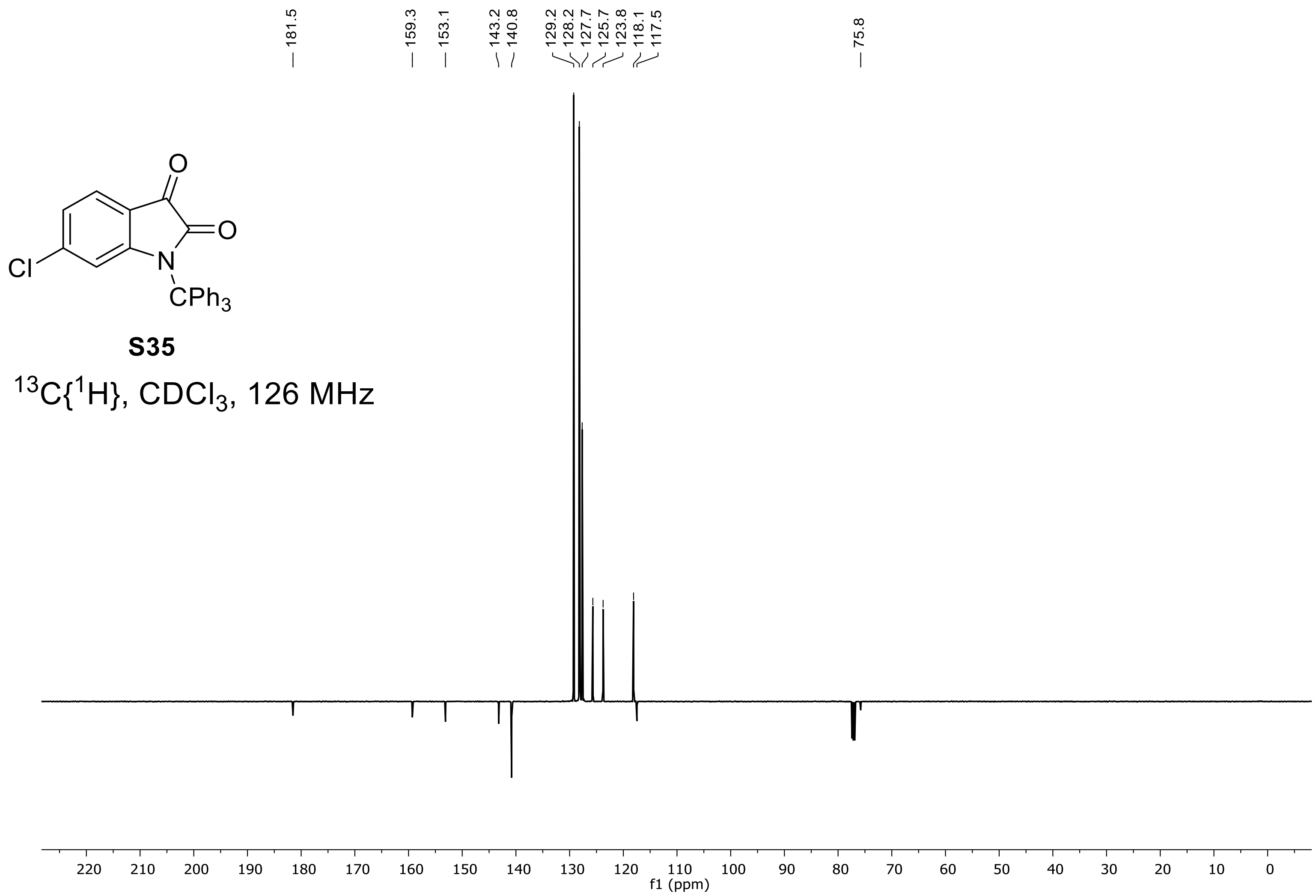

Fig. S111: $^{13}C\{^{1}H\}$ NMR spectrum for 6-Chloro-1-tritylindoline-2,3-dione (**S35**).

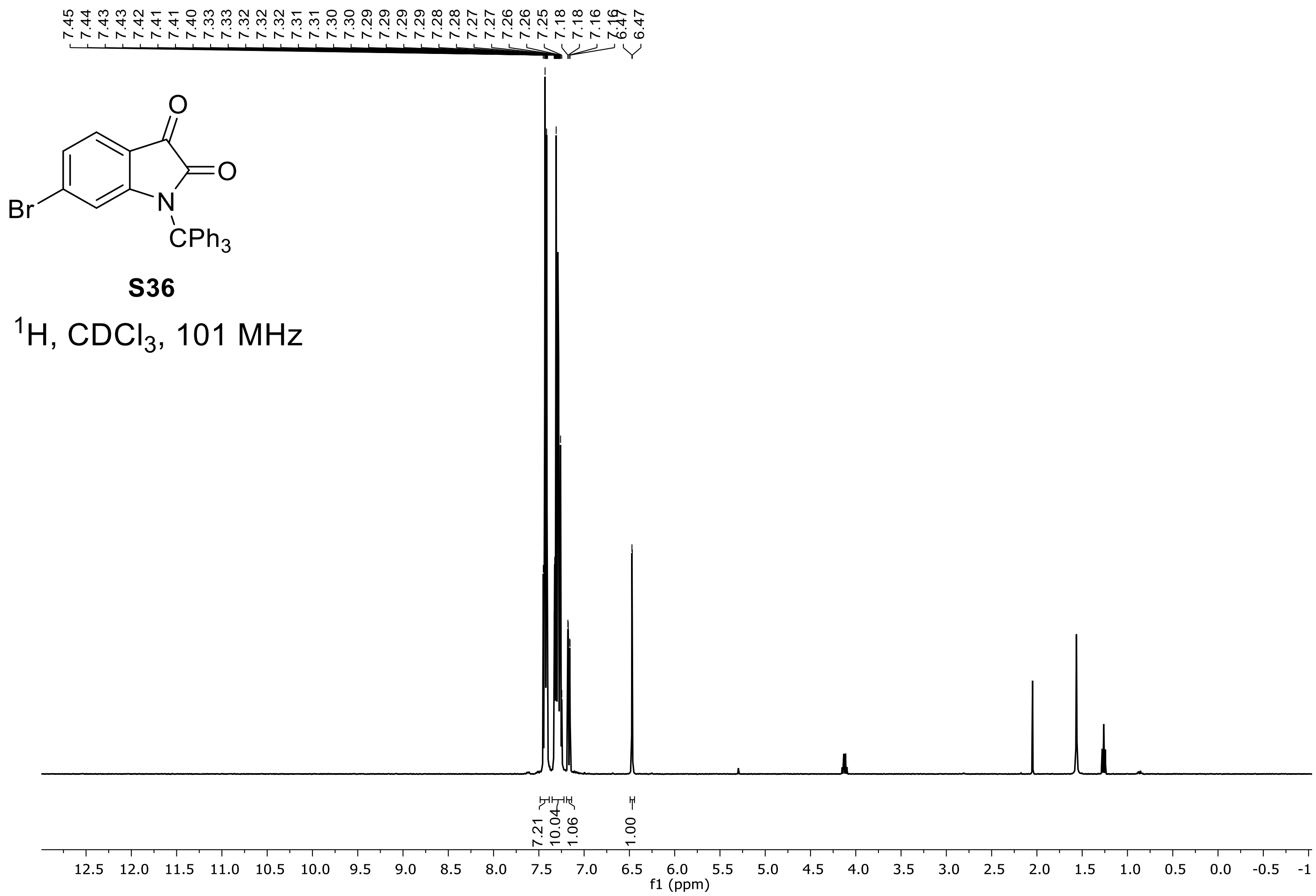

Fig. S112: ^{1}H NMR spectrum for 6-Bromo-1-tritylindoline-2,3-dione (**S36**).

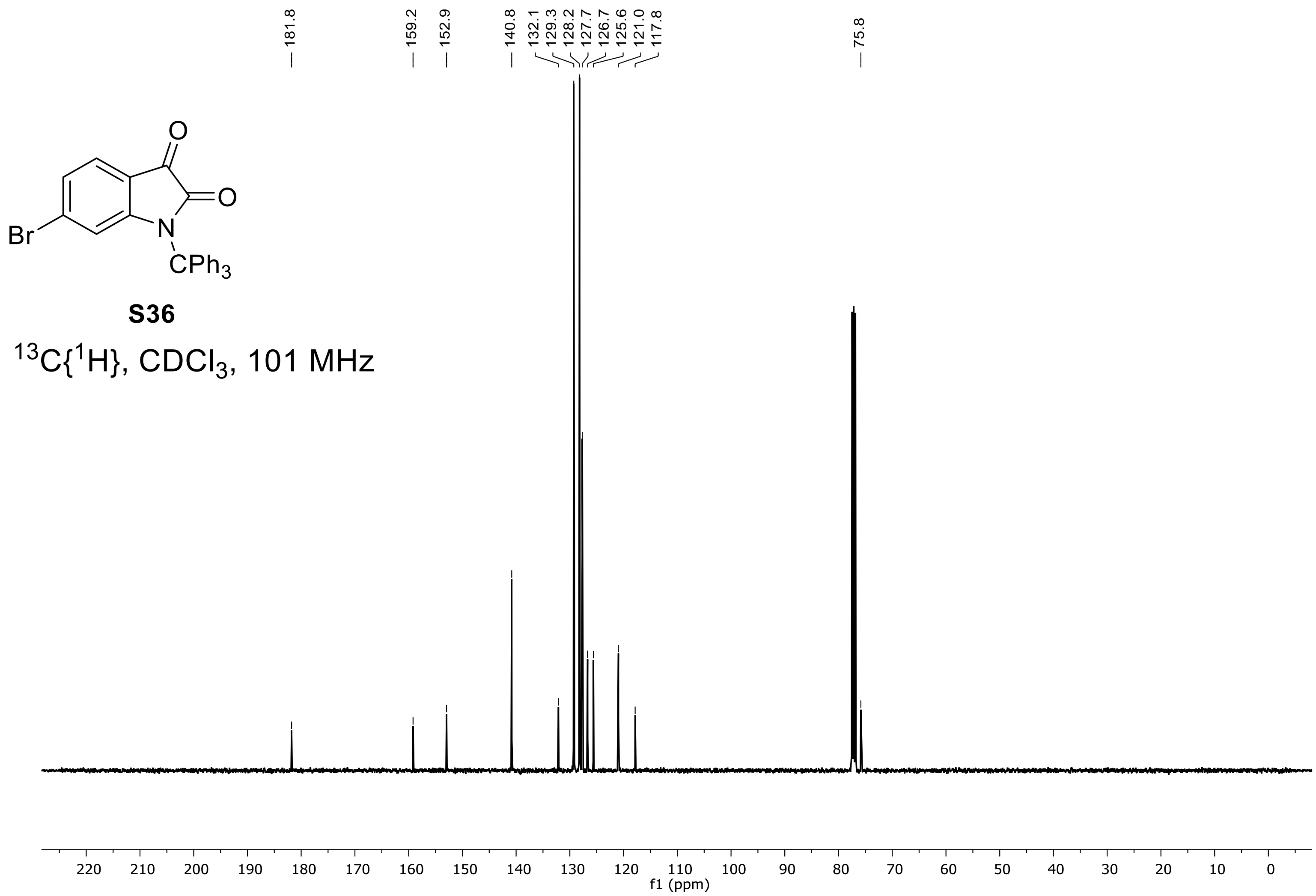

Fig. S113: $^{13}C\{^1H\}$ NMR spectrum for 6-Bromo-1-tritylindoline-2,3-dione (**S36**).

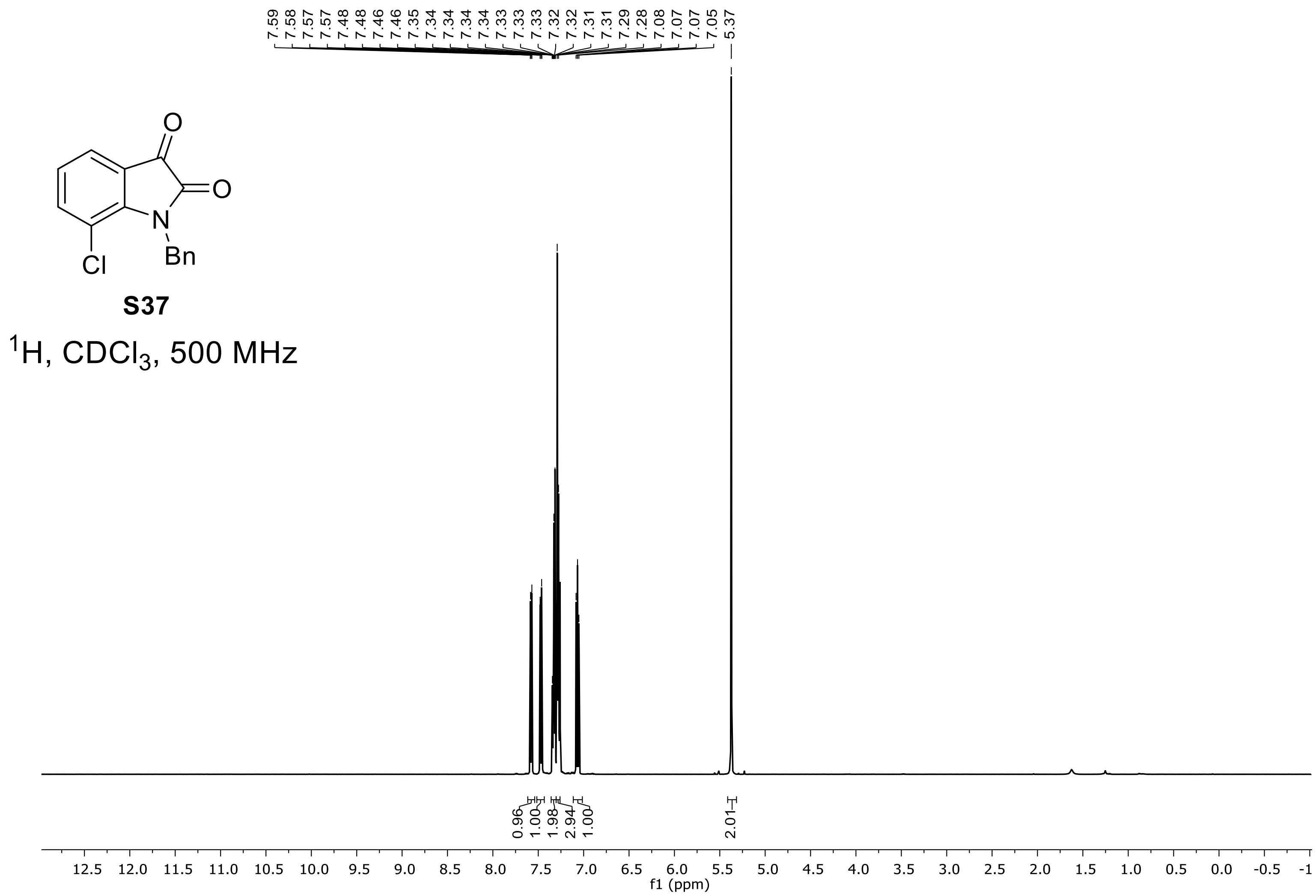

Fig. S114: ^{1}H NMR spectrum for 1-Benzyl-7-chloroindoline-2,3-dione (**S37**).

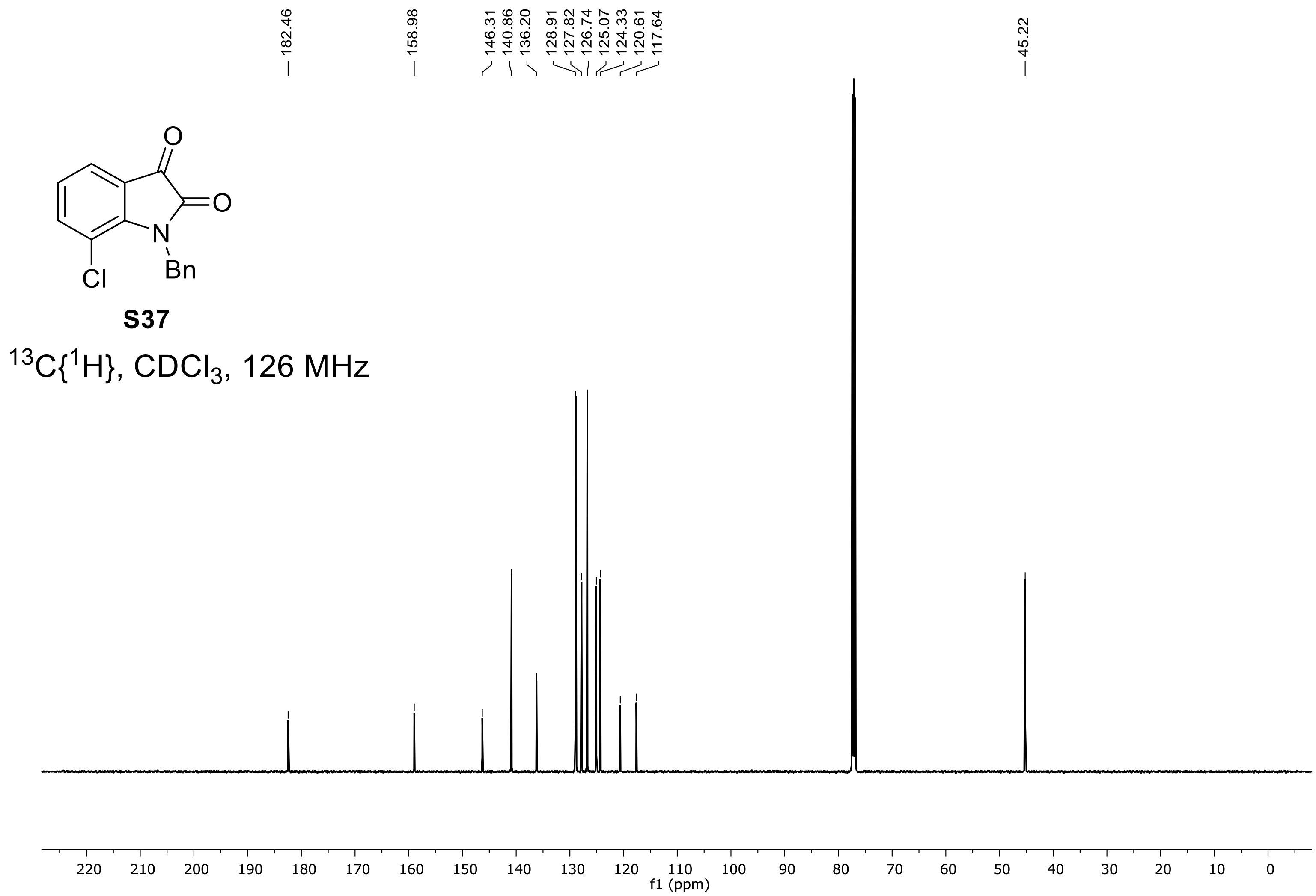

Fig. S115: $^{13}C\{^1H\}$ NMR spectrum for 1-Benzyl-7-chloroindoline-2,3-dione (**S37**).

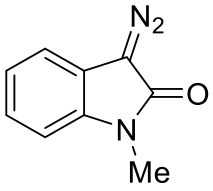

S38

^1H, $CDCl_3$, 500 MHz

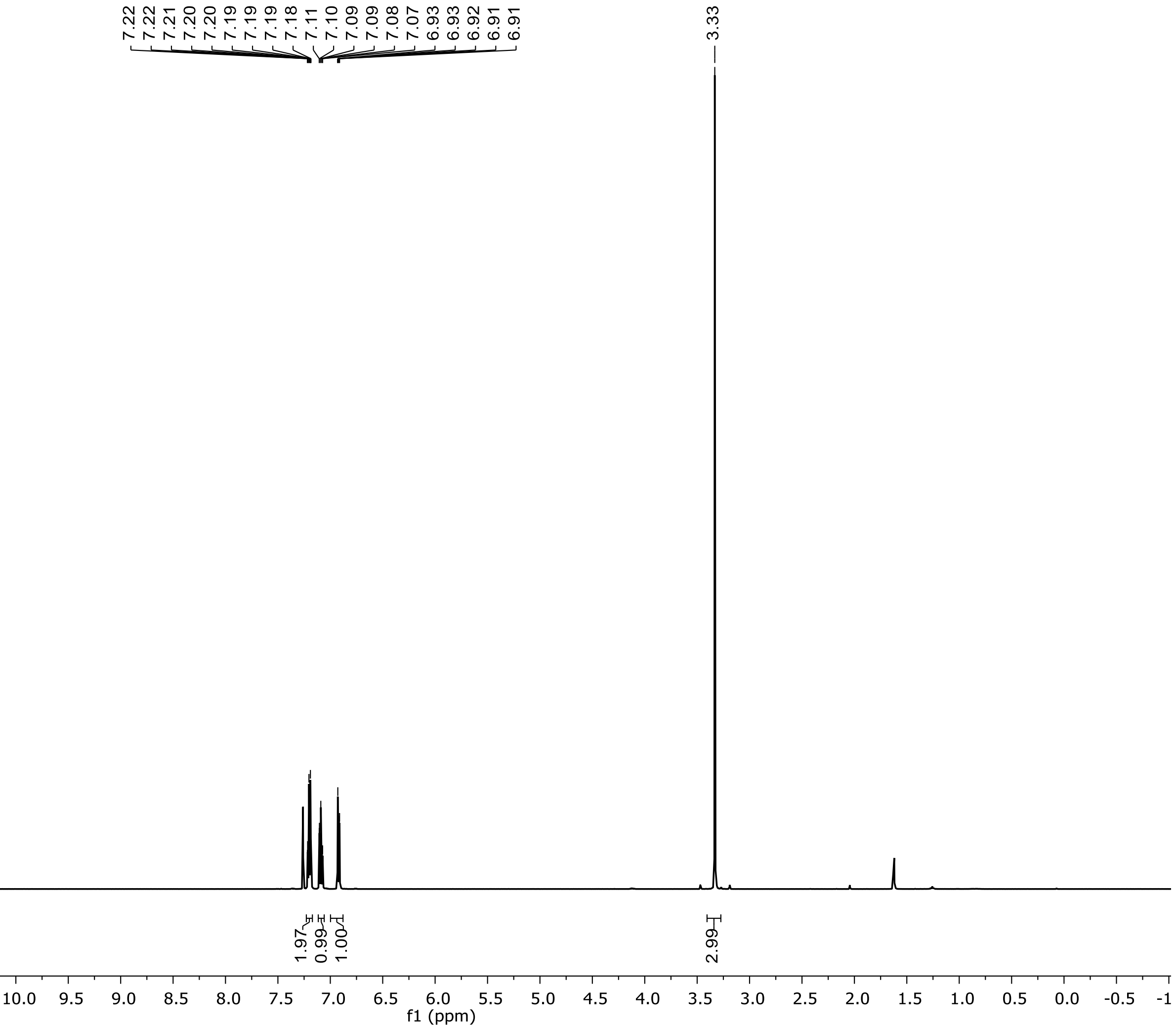

Fig. S116: ^1H NMR spectrum for 3-Diazo-1-methylindolin-2-one (**S38**).

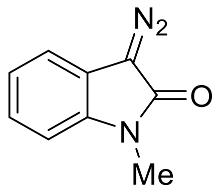

S38

$^{13}C\{^1H\}$, $CDCl_3$, 126 MHz

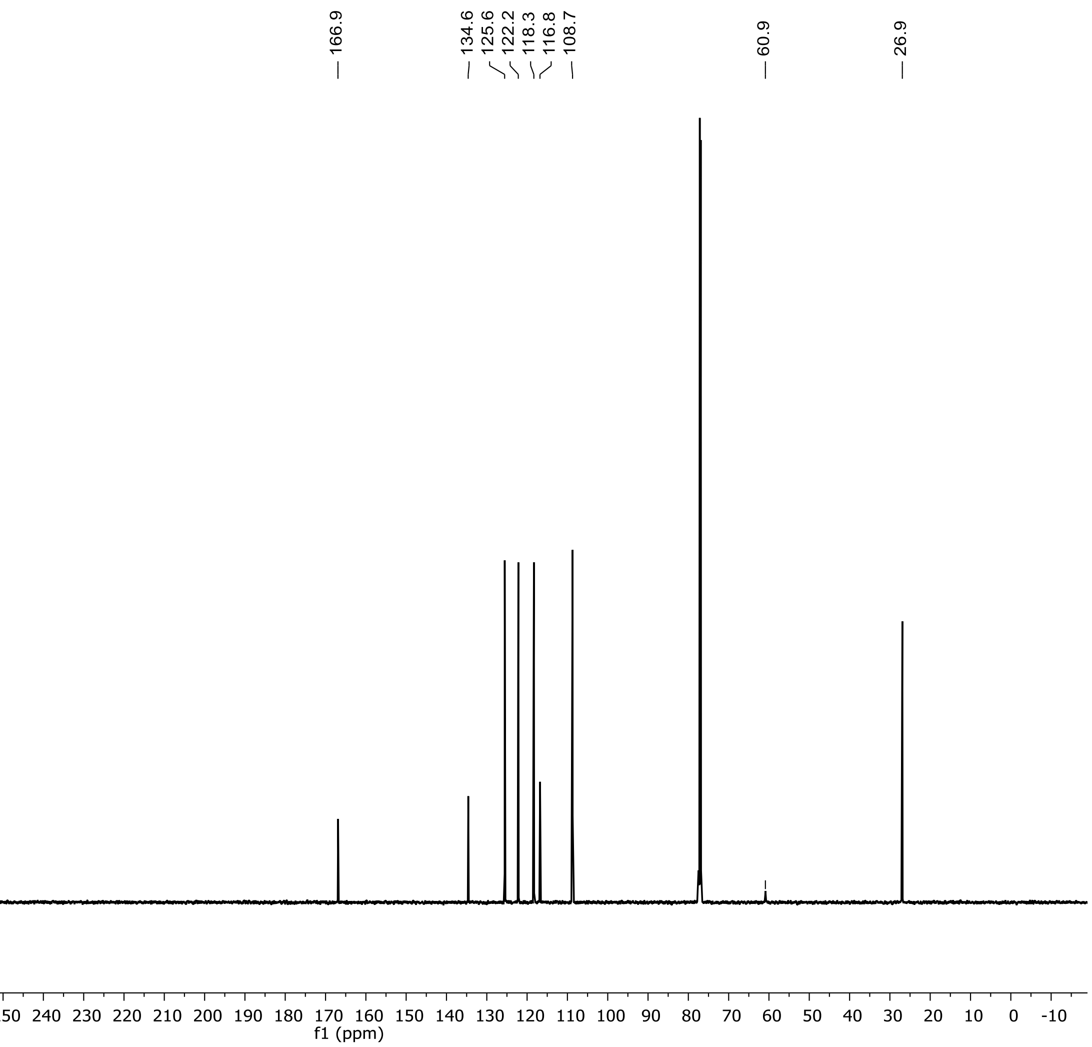

Fig. S117: $^{13}C\{^1H\}$ NMR spectrum for 3-Diazo-1-methylindolin-2-one (**S38**).

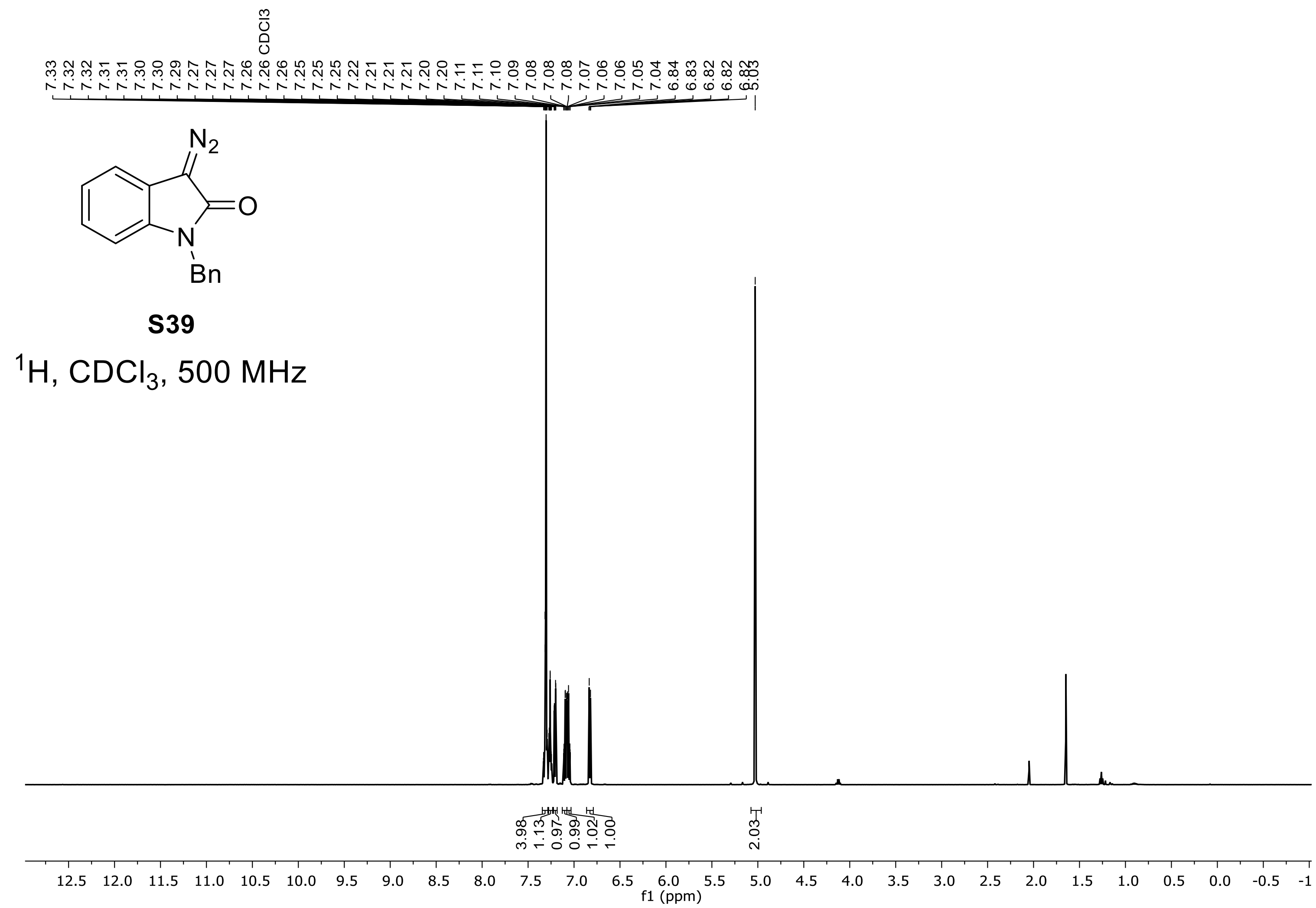

Fig. S118: ^{1}H NMR spectrum for 1-Benzyl-3-diazoindolin-2-one (**S39**).

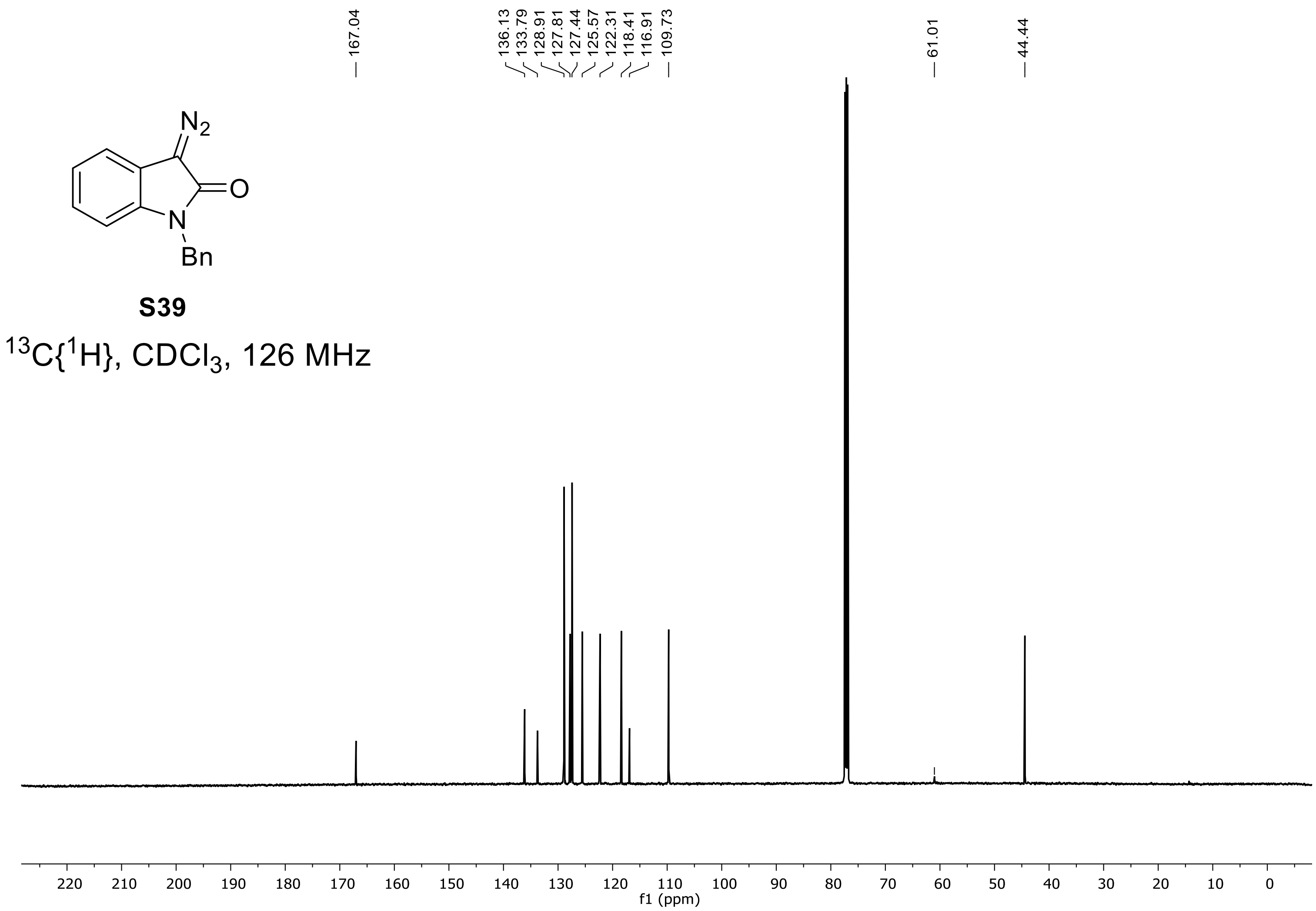

Fig. S119: $^{13}C\{^{1}H\}$ NMR spectrum for 1-Benzyl-3-diazoindolin-2-one (**S39**).

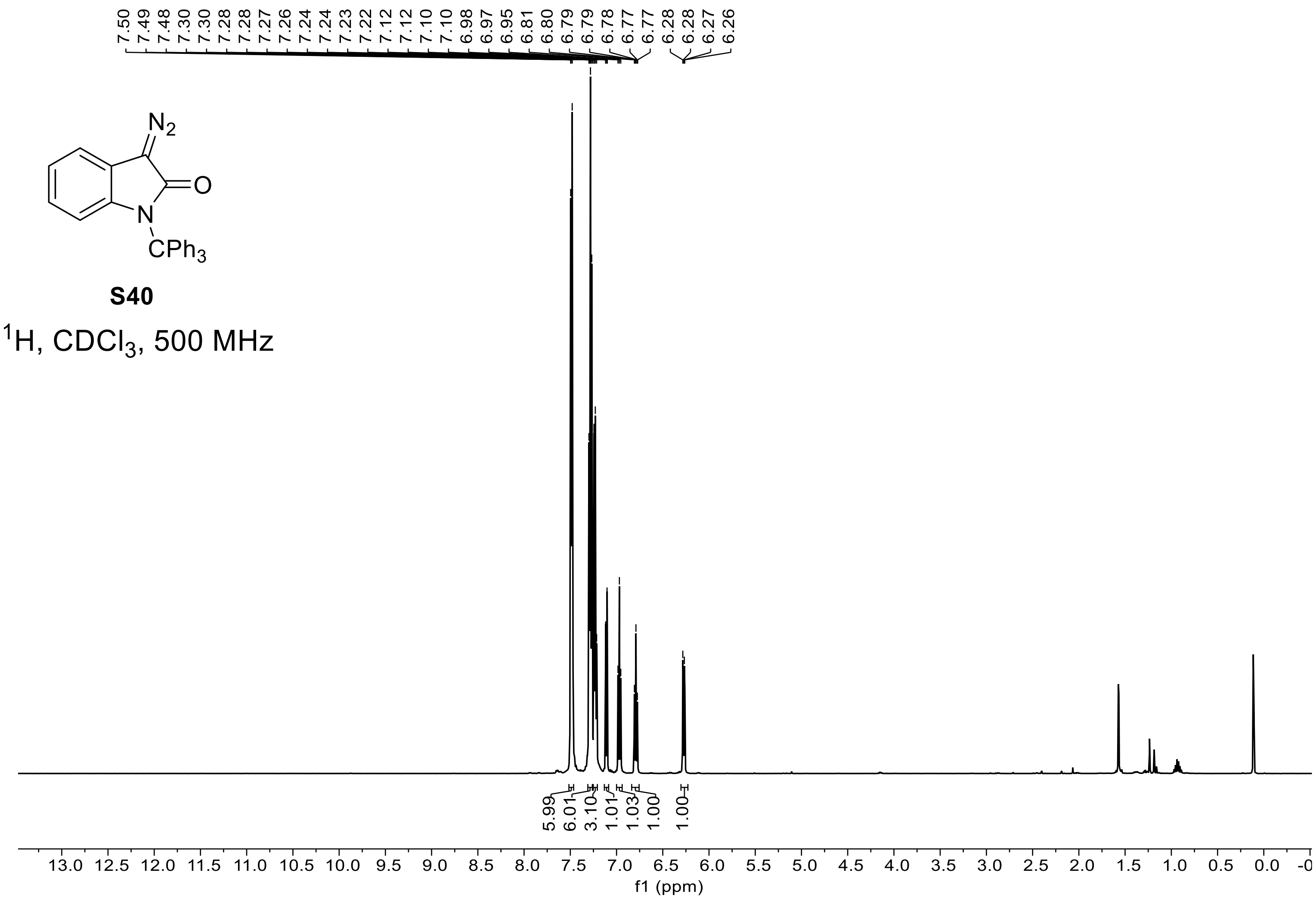

Fig. S120: ^{1}H NMR spectrum for 3-Diazo-1-tritylindolin-2-one (**S40**).

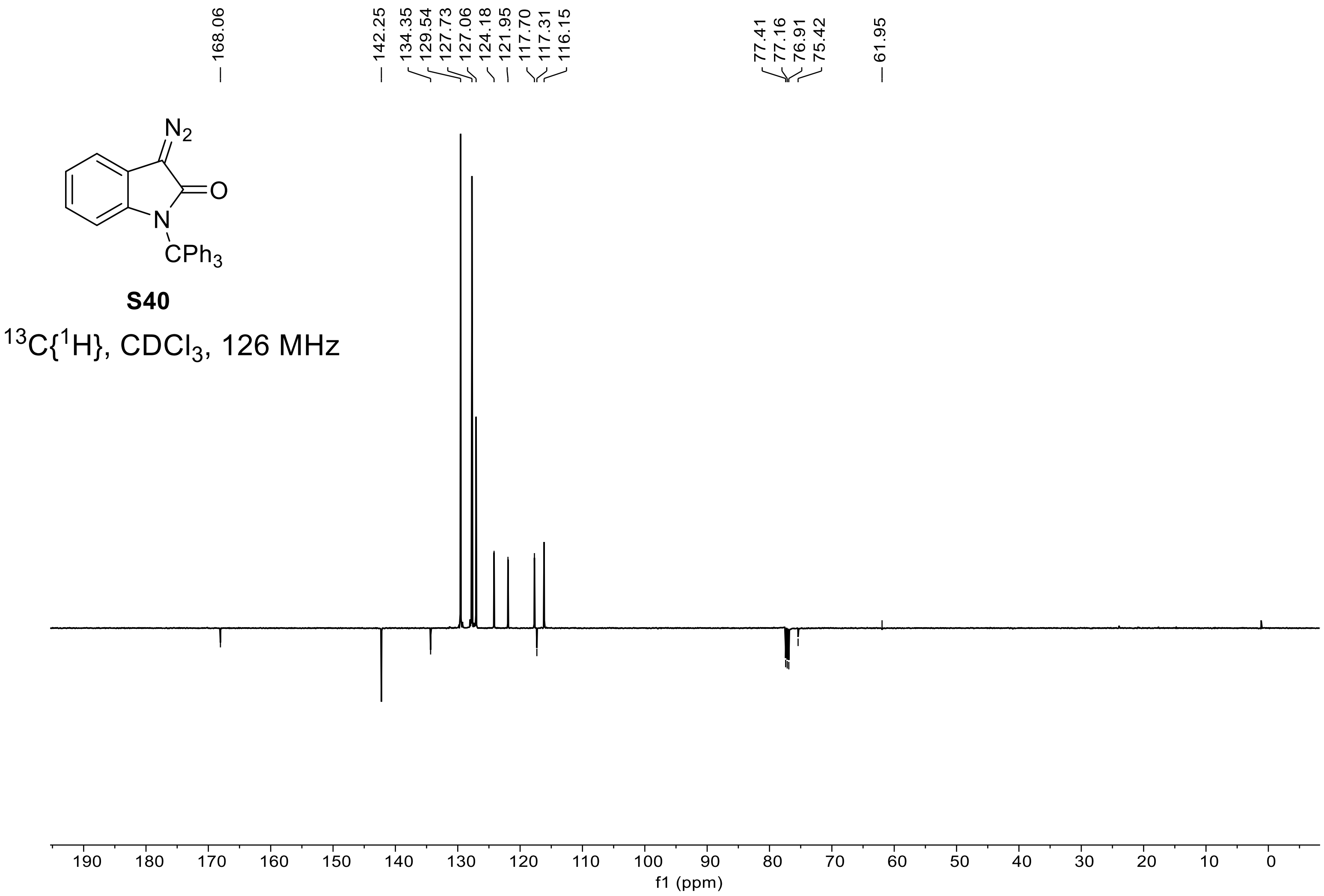

Fig. S121: $^{13}C\{^{1}H\}$ NMR spectrum for 3-Diazo-1-tritylindolin-2-one (**S40**).

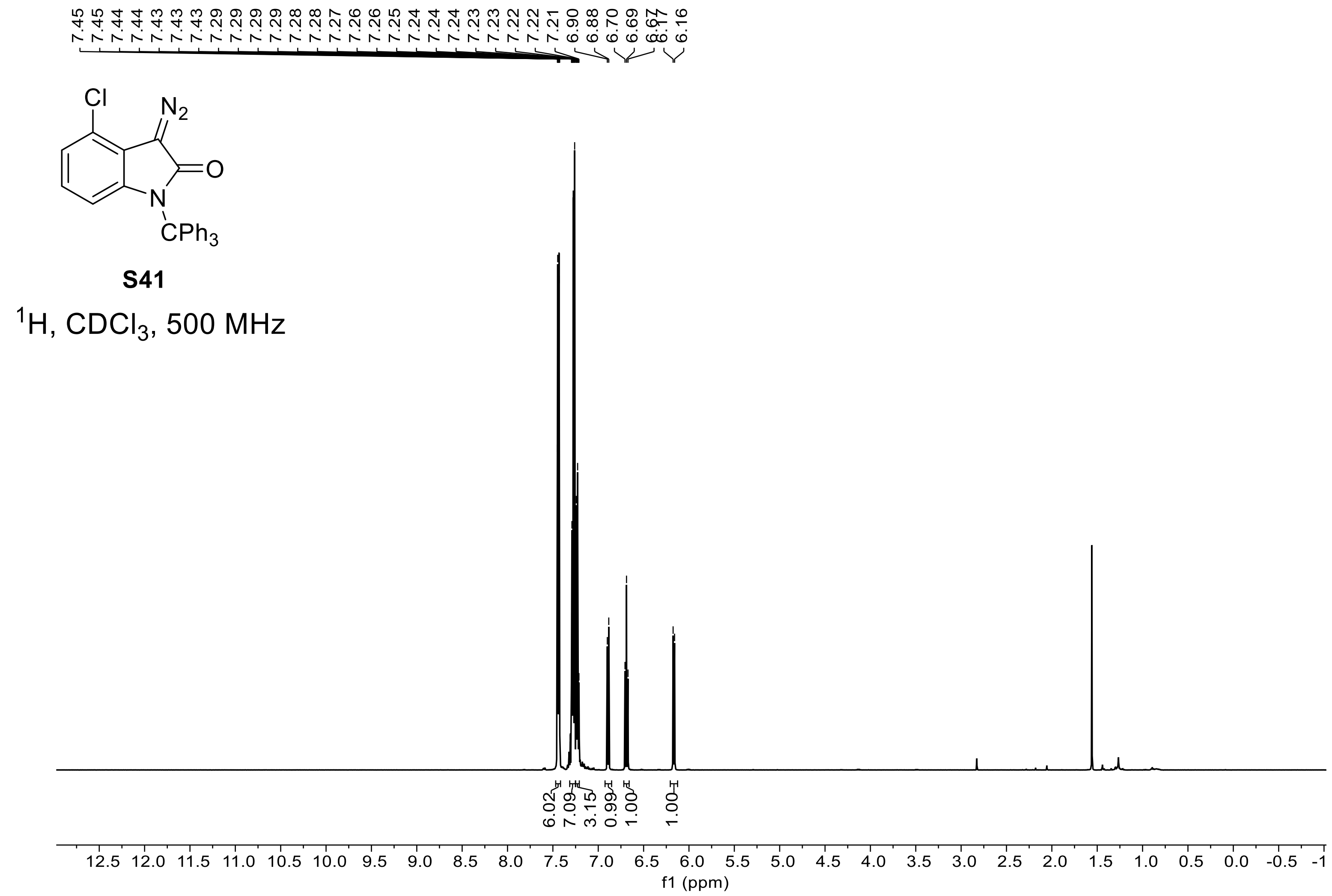

Fig. S122: ^{1}H NMR spectrum for 4-Chloro-3-diazo-1-tritylindolin-2-one (**S41**).

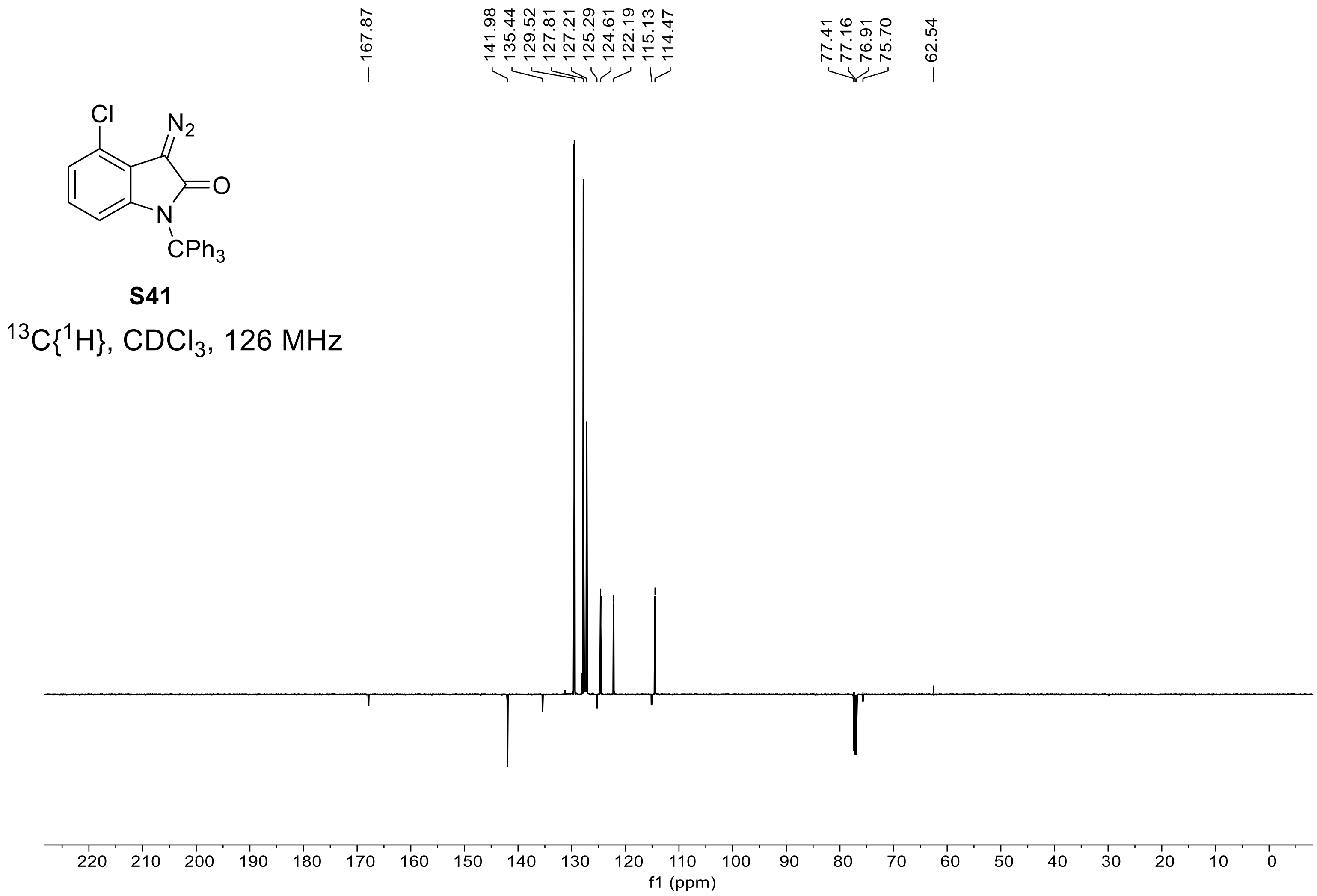

Fig. S123: $^{13}C\{^1H\}$ NMR spectrum for 4-Chloro-3-diazo-1-tritylindolin-2-one (**S41**).

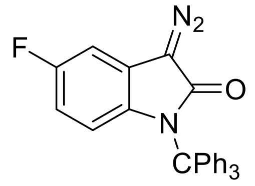

S42

^{1}H, $CDCl_3$, 500 MHz

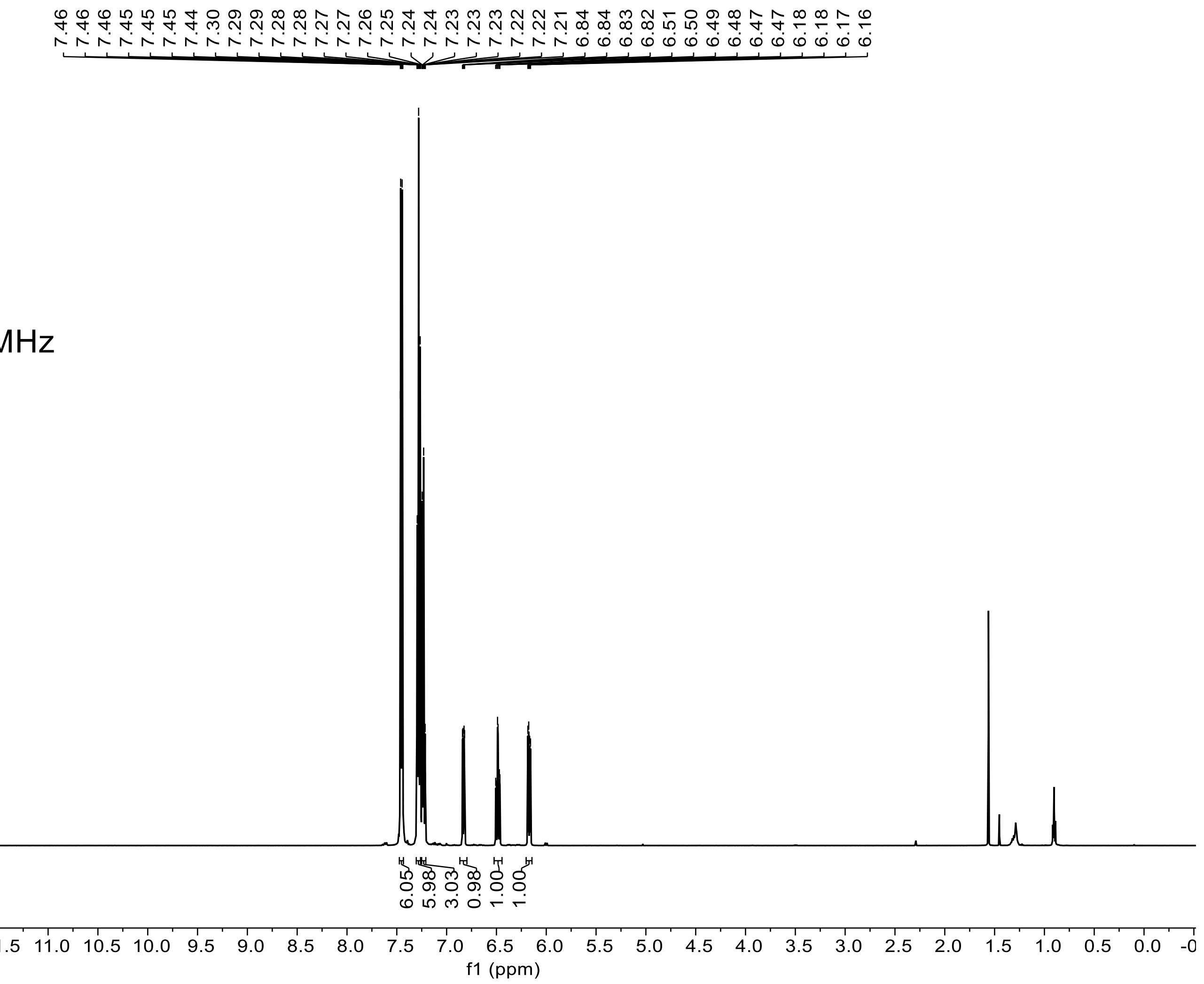

Fig. S124: ^{1}H NMR spectrum for 3-Diazo-5-fluoro-1-tritylindolin-2-one (**S42**).

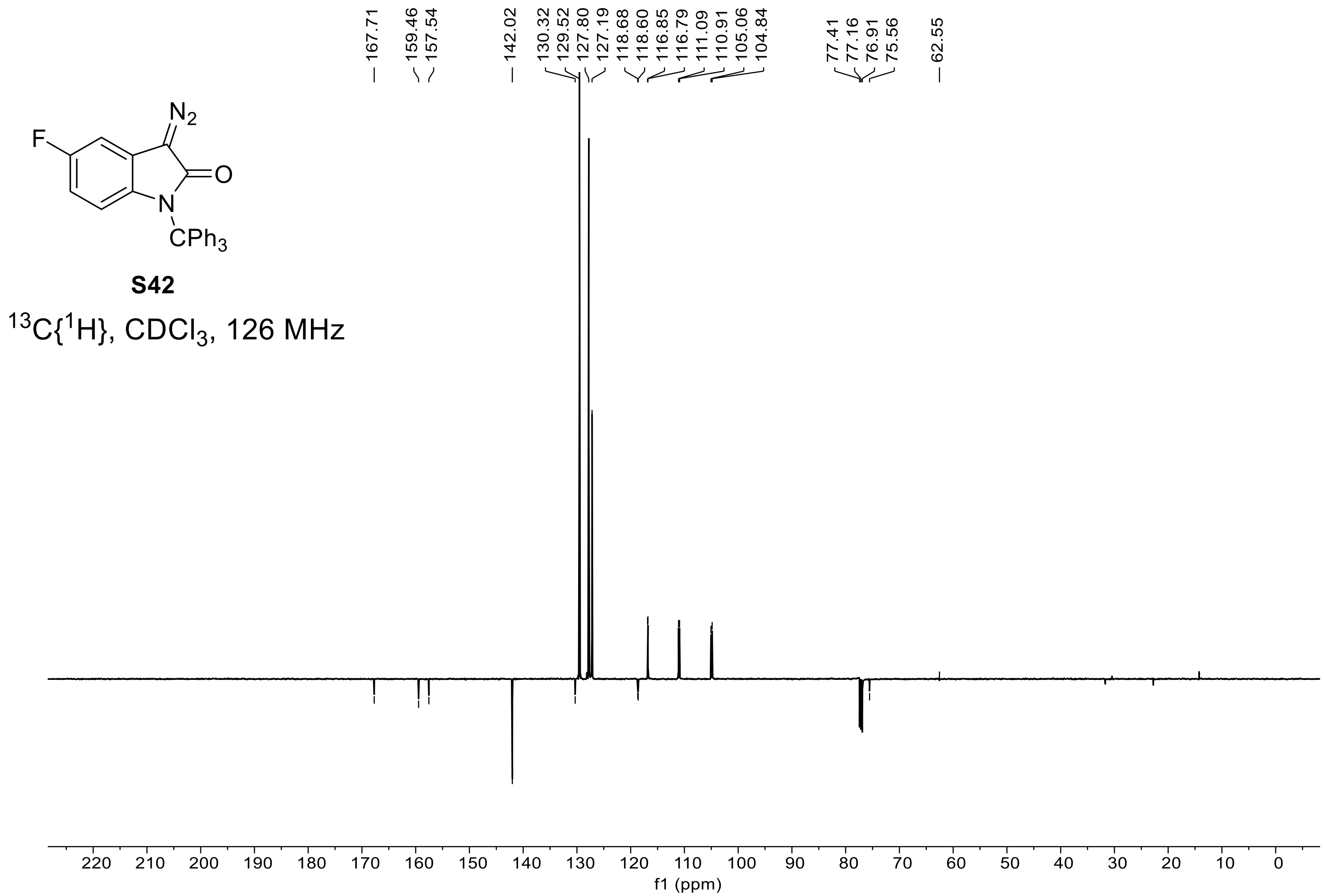

Fig. S125: $^{13}C\{^1H\}$ NMR spectrum for 3-Diazo-5-fluoro-1-tritylindolin-2-one (**S42**).

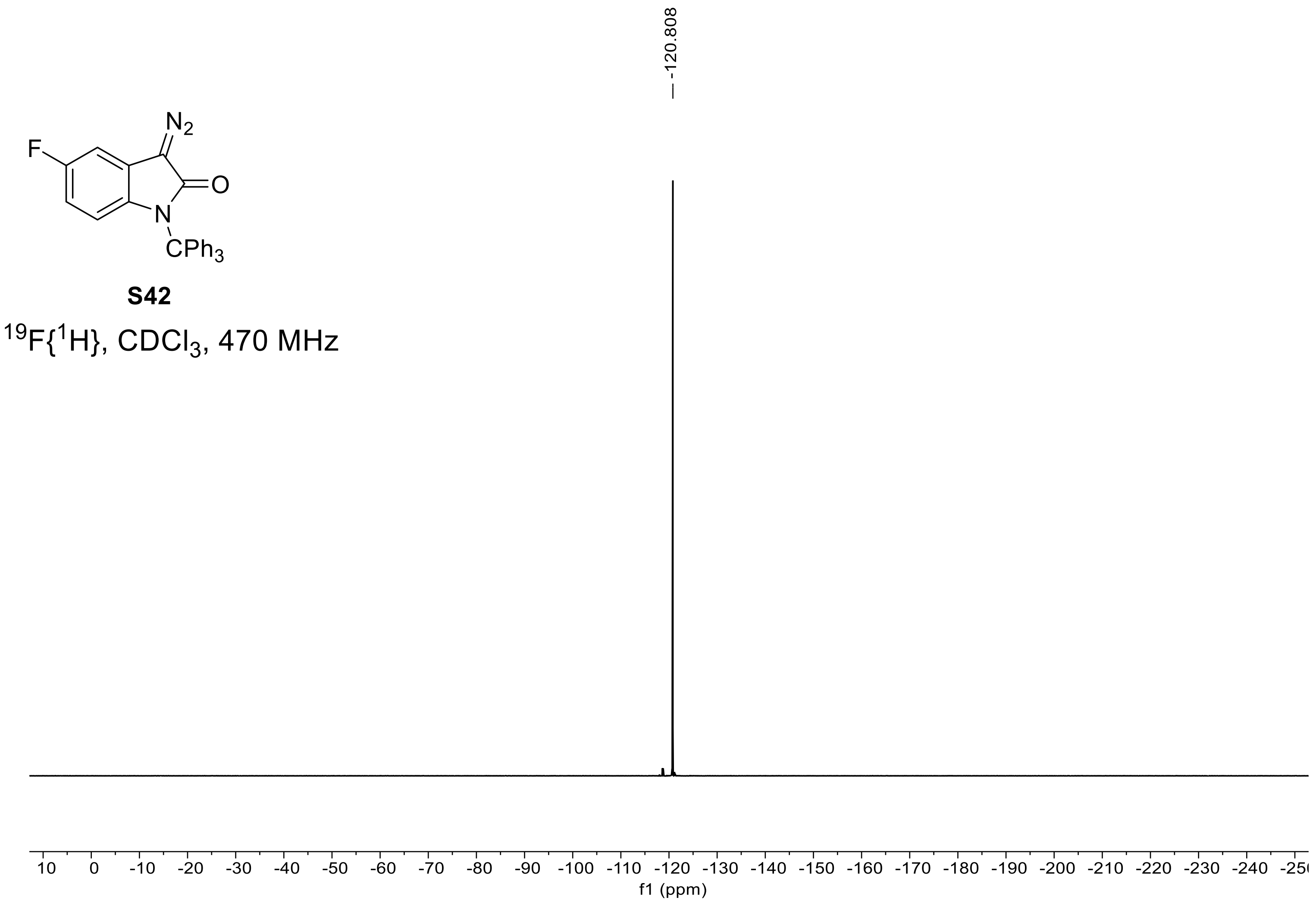

Fig. S126: $^{19}F\{^{1}H\}$ NMR spectrum for 3-Diazo-5-fluoro-1-tritylindolin-2-one (**S42**).

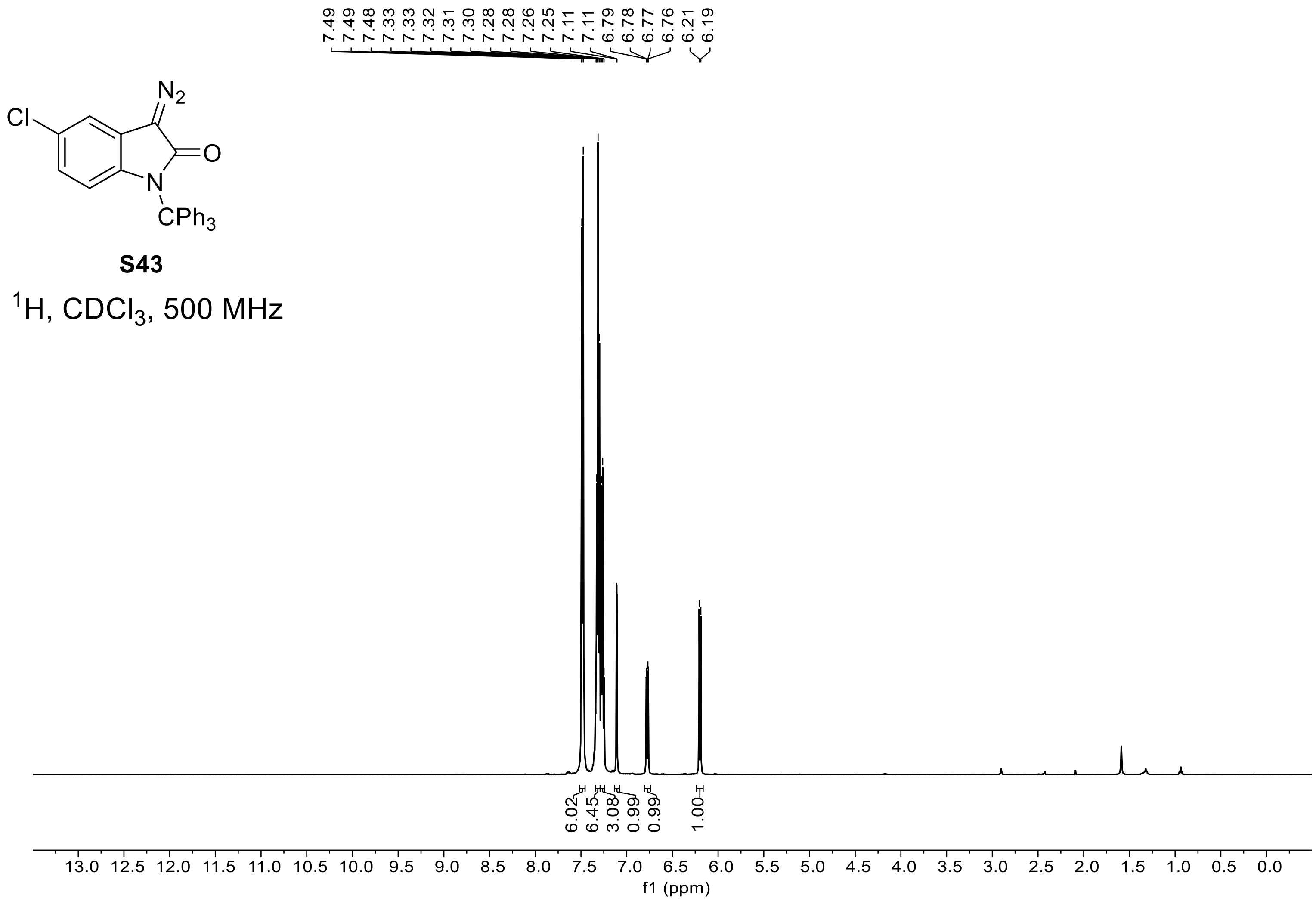

Fig. S127: ^{1}H NMR spectrum for 5-Chloro-3-diazo-1-tritylindolin-2-one (**S43**).

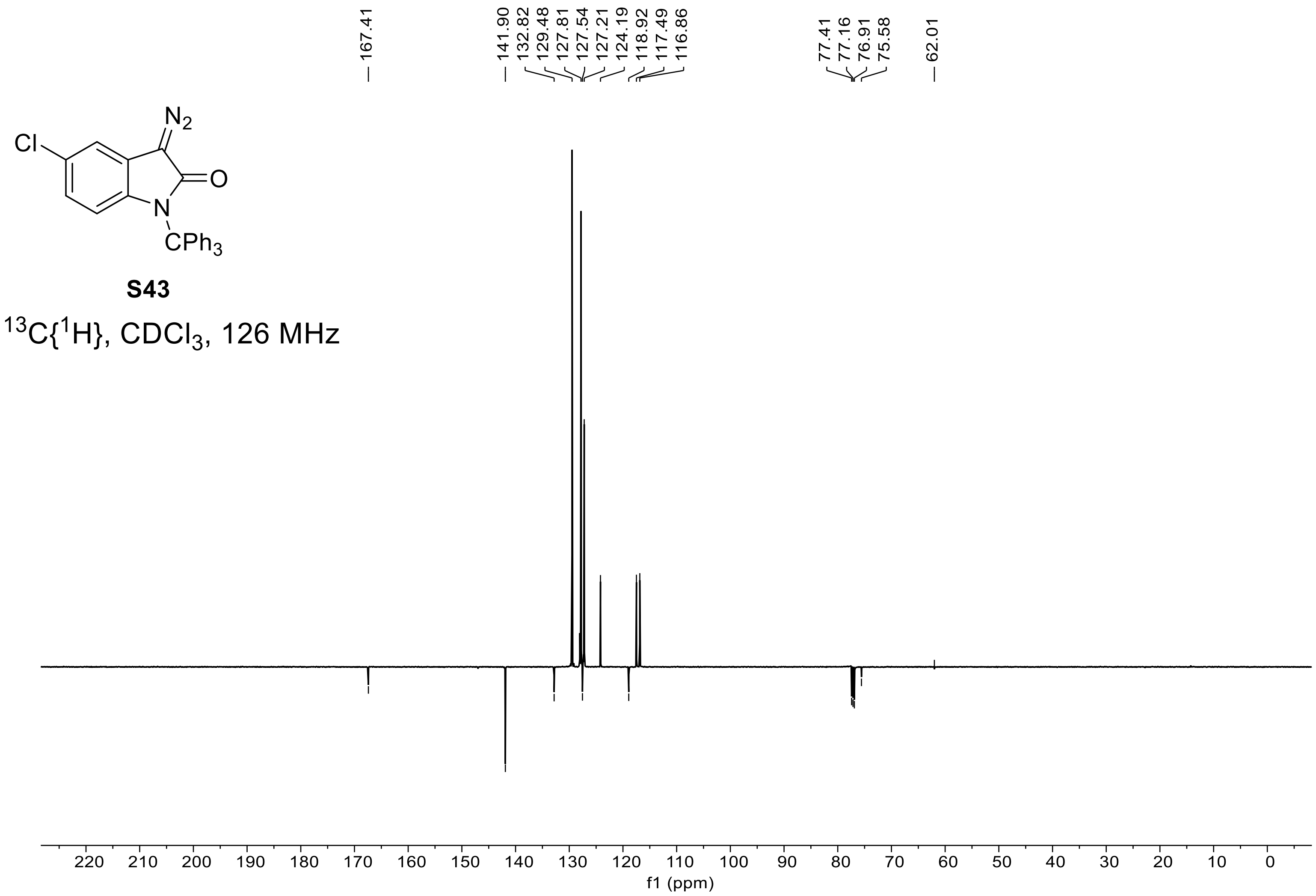

Fig. S128: $^{13}C\{^1H\}$ NMR spectrum for 5-Chloro-3-diazo-1-tritylindolin-2-one (**S43**).

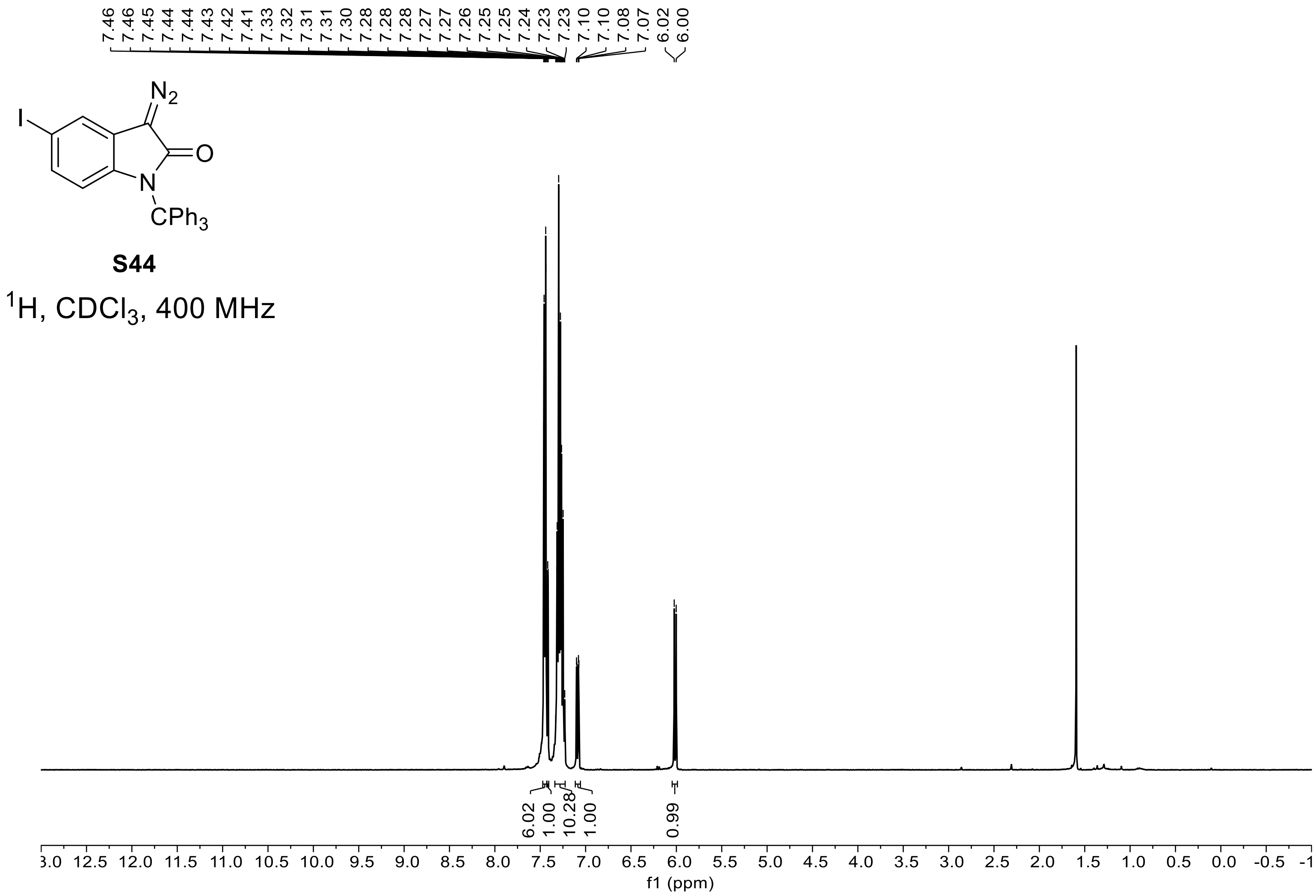

Fig. S129: ^{1}H NMR spectrum for 3-Diazo-5-iodo-1-tritylindolin-2-one (**S44**).

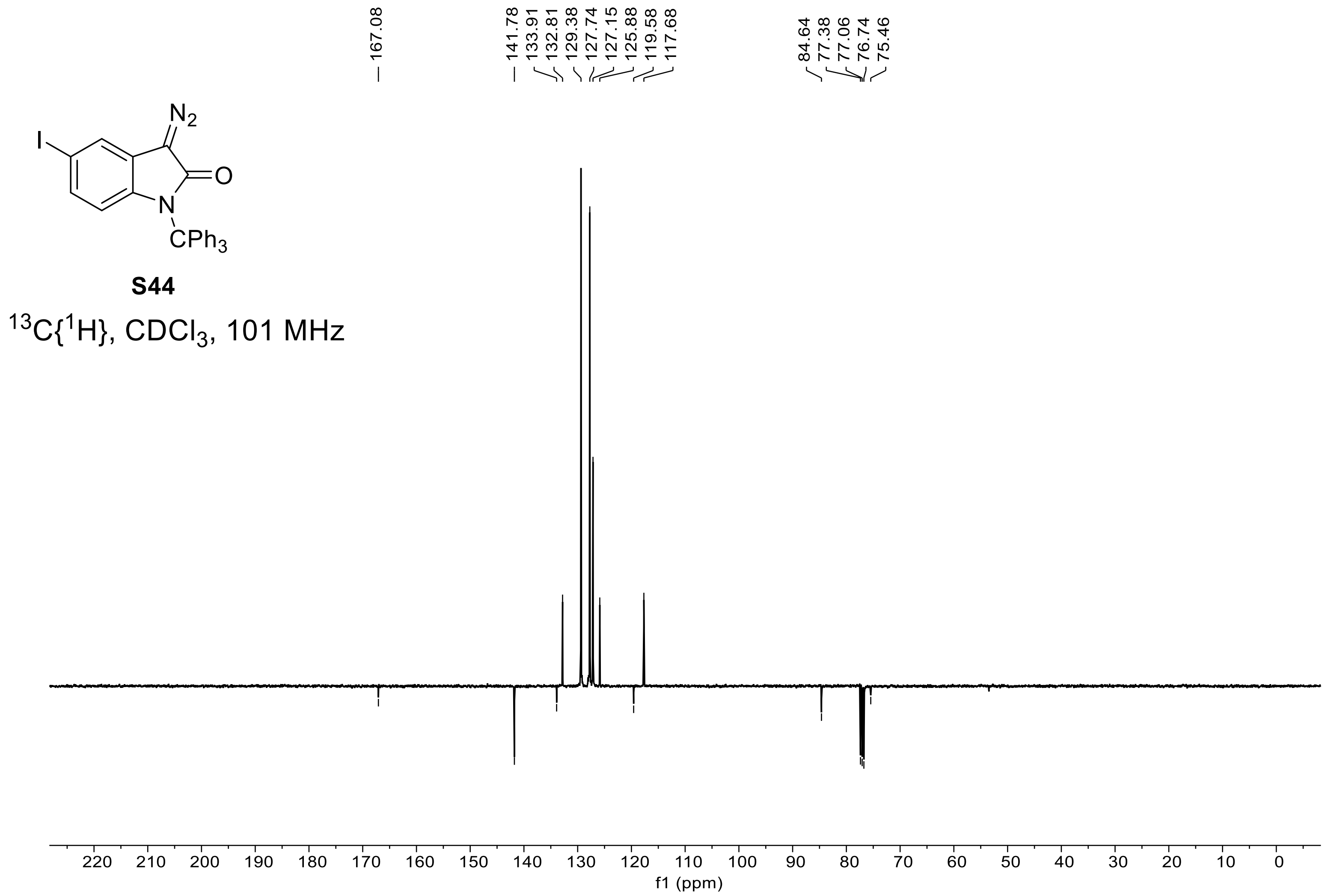

Fig. S130: $^{13}C\{^1H\}$ NMR spectrum for 3-Diazo-5-iodo-1-tritylindolin-2-one (**S44**).

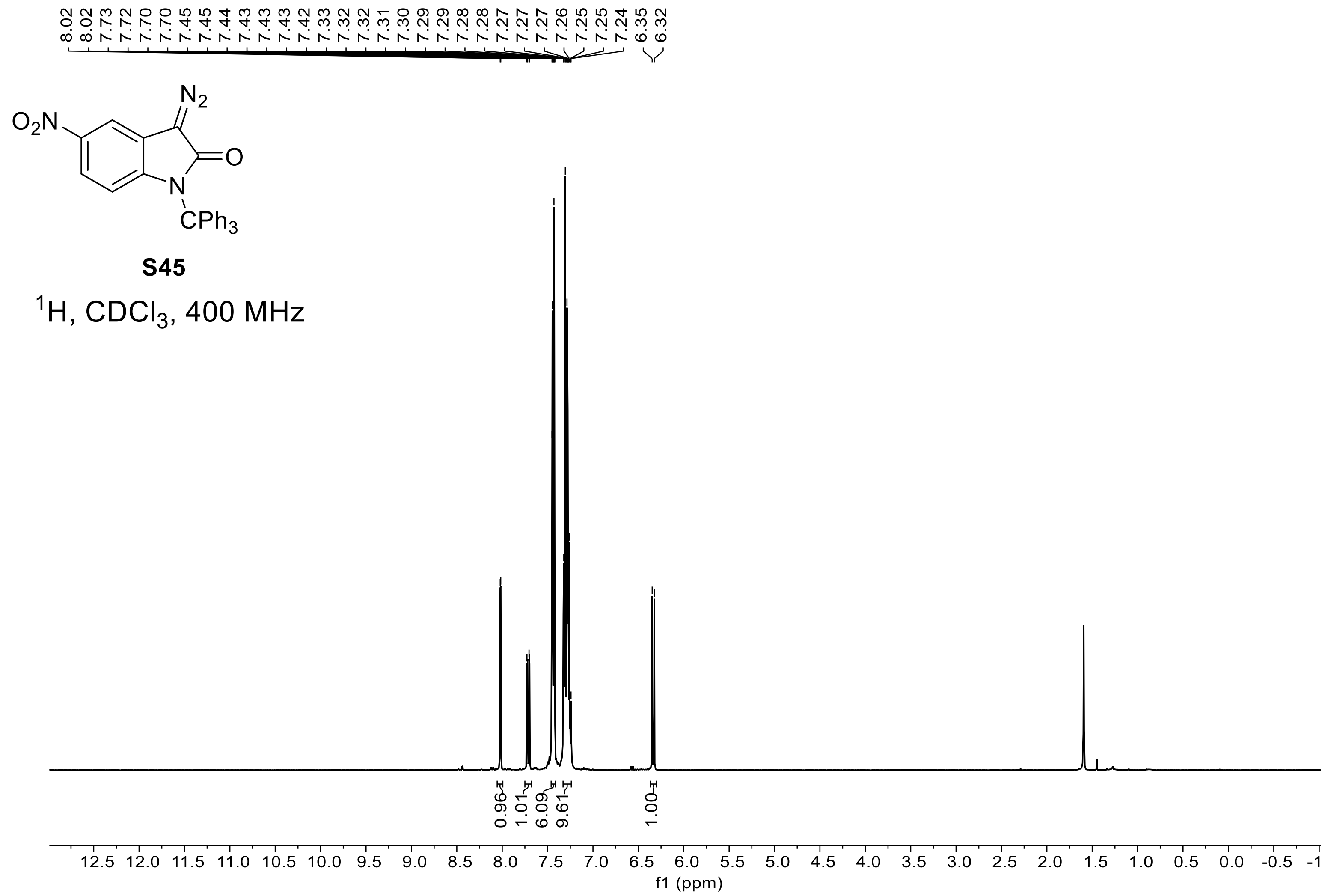

Fig. S131: ^{1}H NMR spectrum for 3-Diazo-5-nitro-1-tritylindolin-2-one (**S45**).

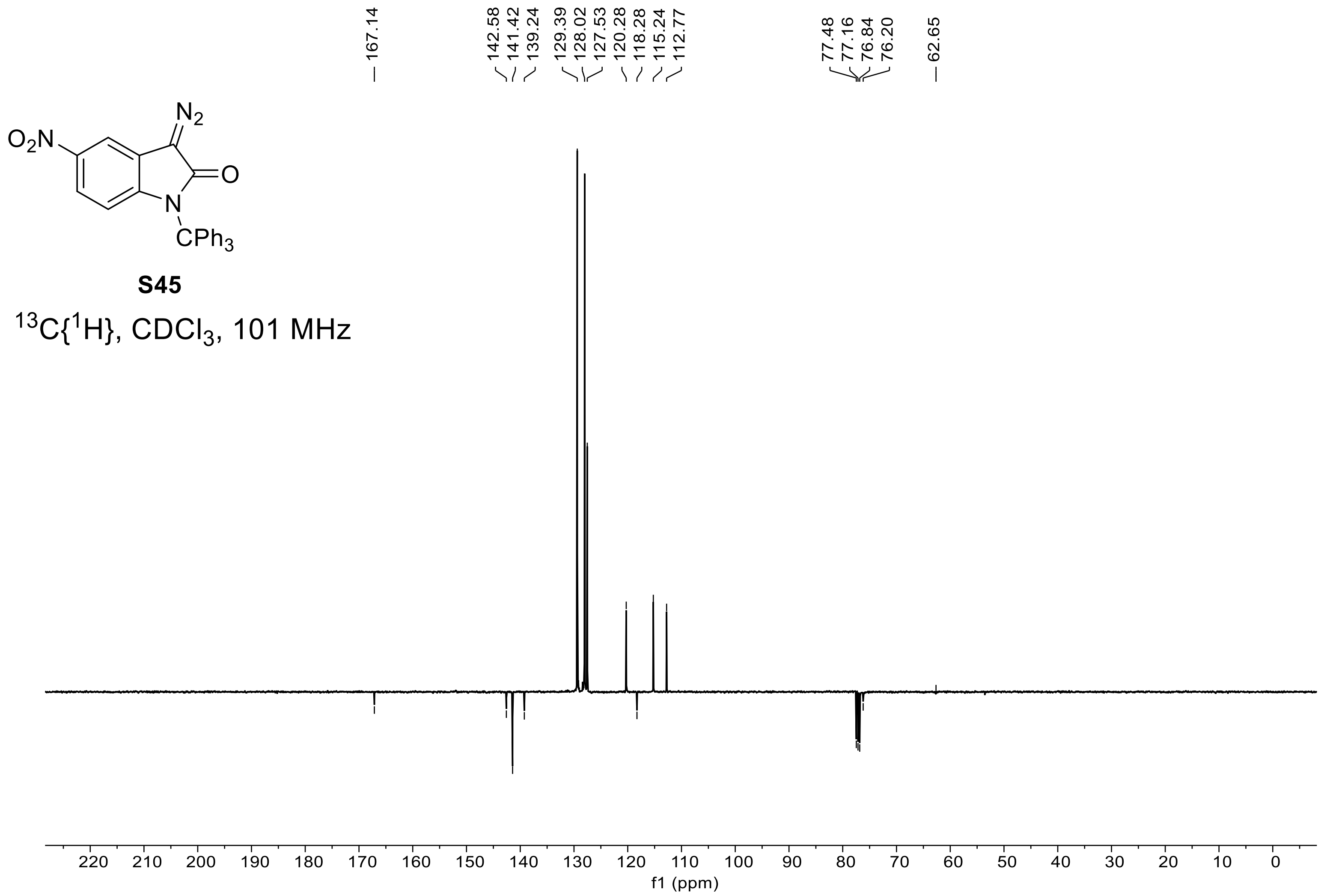

Fig. S132: $^{13}C\{^{1}H\}$ NMR spectrum for 3-Diazo-5-nitro-1-tritylindolin-2-one (**S45**).

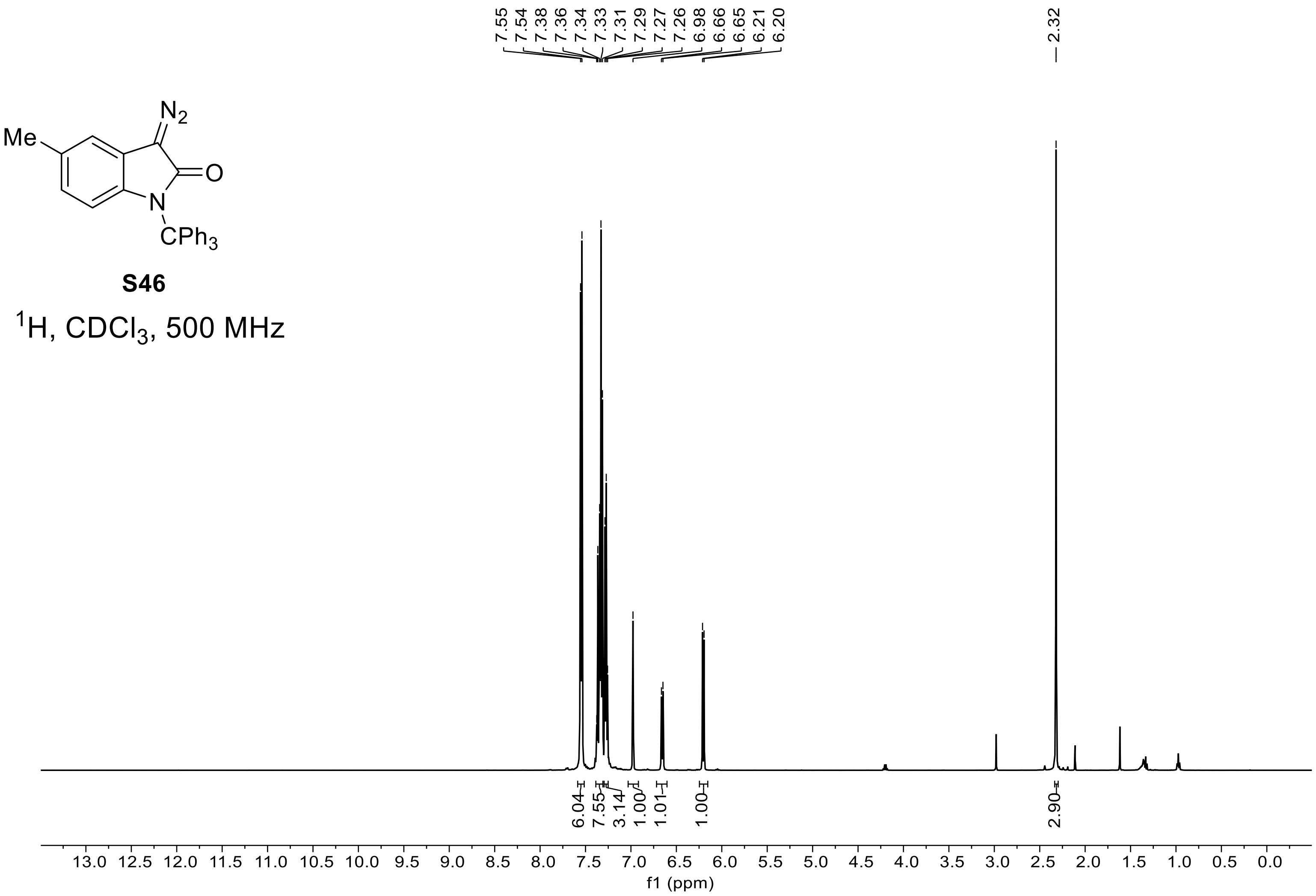

Fig. S133: ^{1}H NMR spectrum for 3-Diazo-5-methyl-1-tritylindolin-2-one (**S46**).

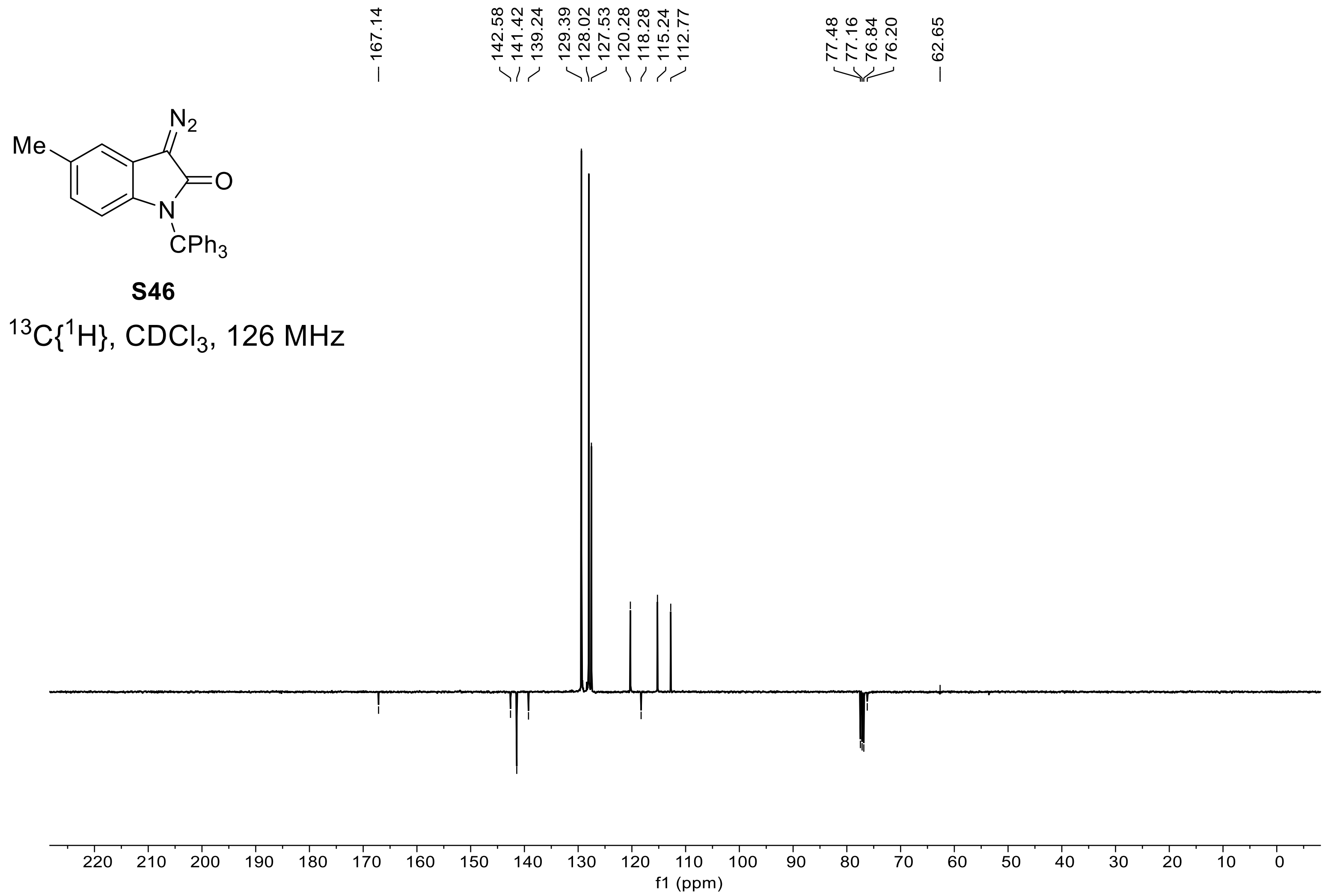

Fig. S134: $^{13}C\{^1H\}$ NMR spectrum for 3-Diazo-5-methyl-1-tritylindolin-2-one (**S46**).

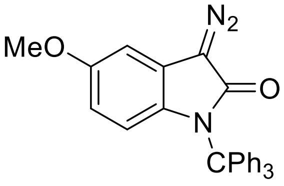

S47

^{1}H, $CDCl_3$, 500 MHz

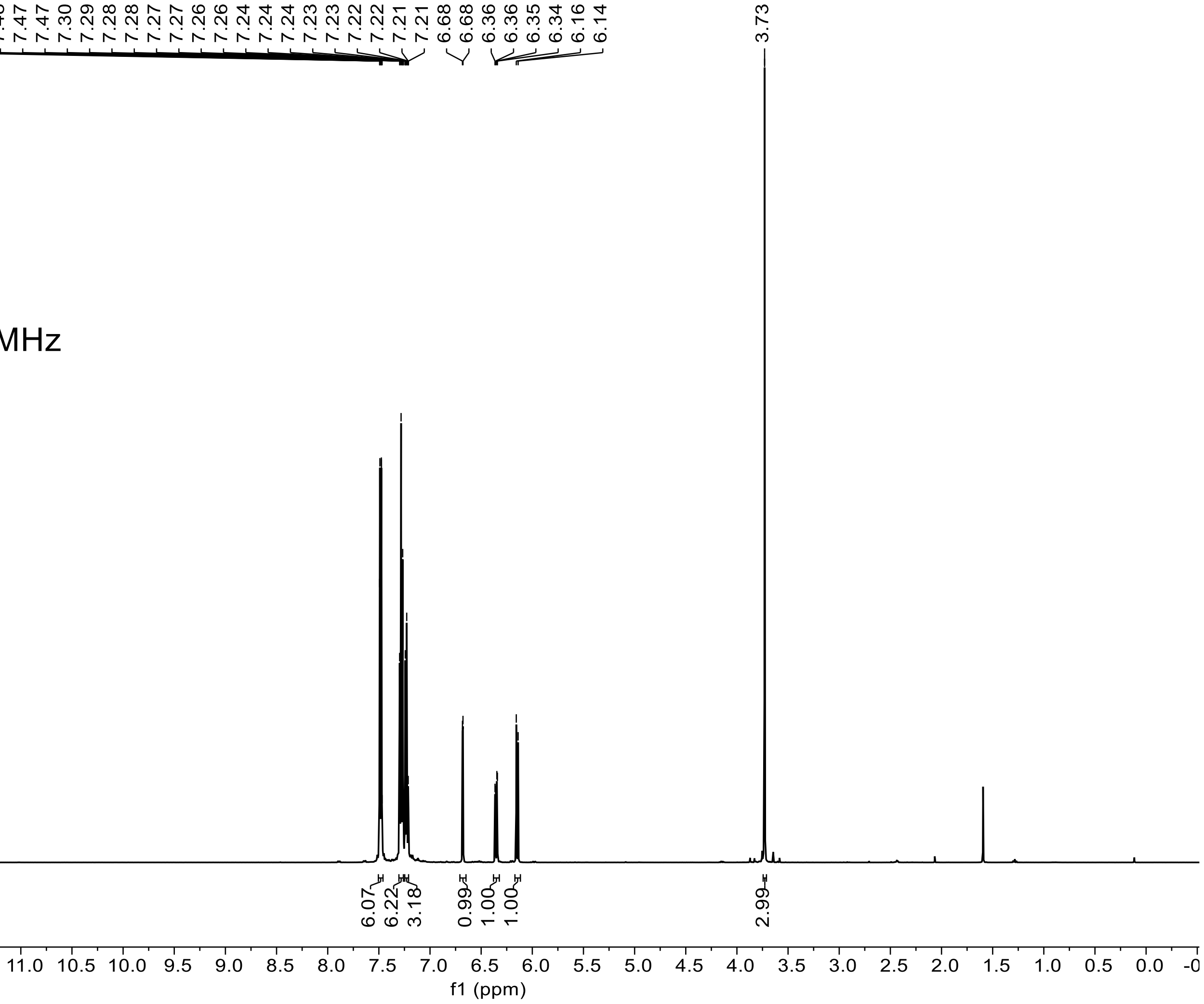

Fig. S135: ^{1}H NMR spectrum for 3-Diazo-5-methoxy-1-tritylindolin-2-one (**S47**).

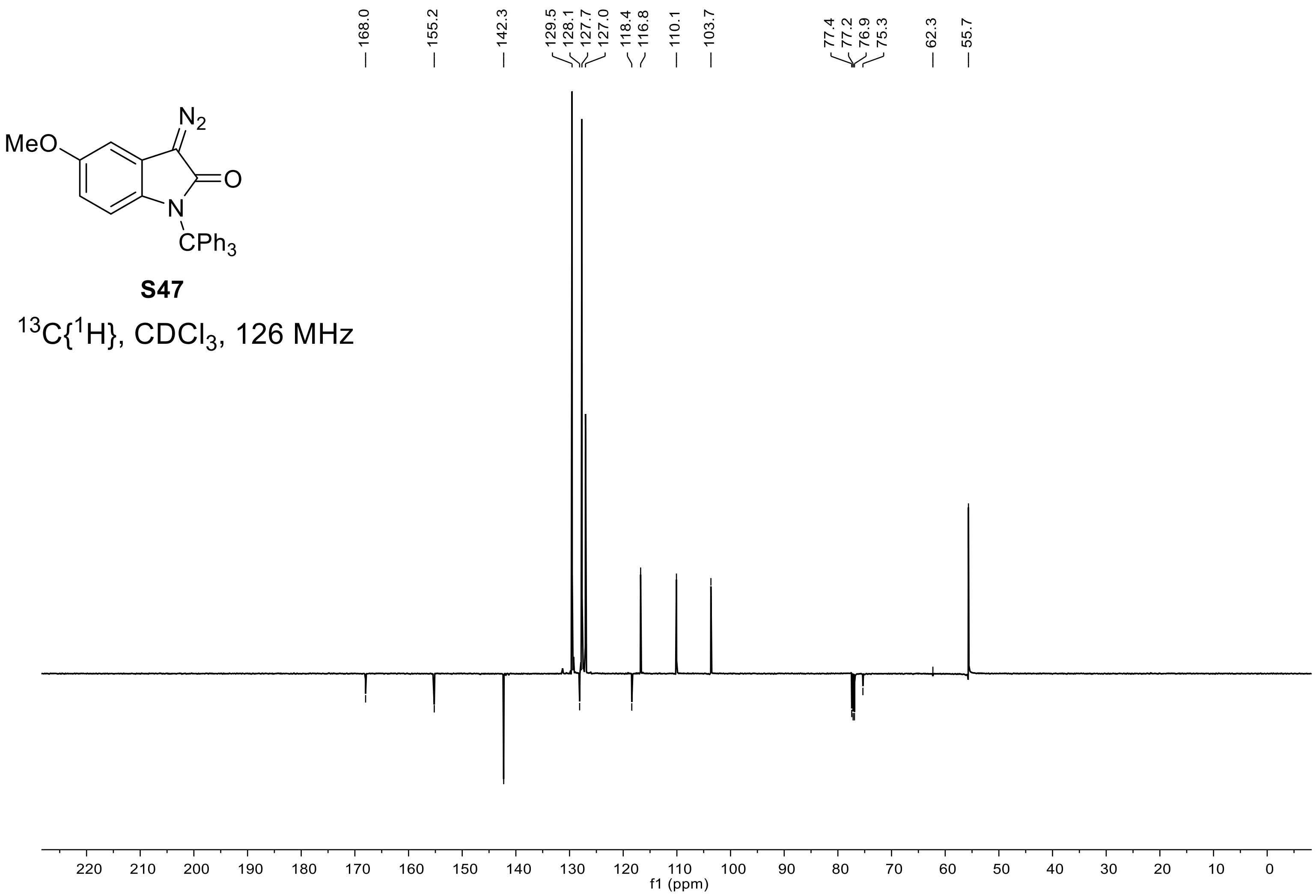

Fig. S136: $^{13}C\{^{1}H\}$ NMR spectrum for 3-Diazo-5-methoxy-1-tritylindolin-2-one (**S47**).

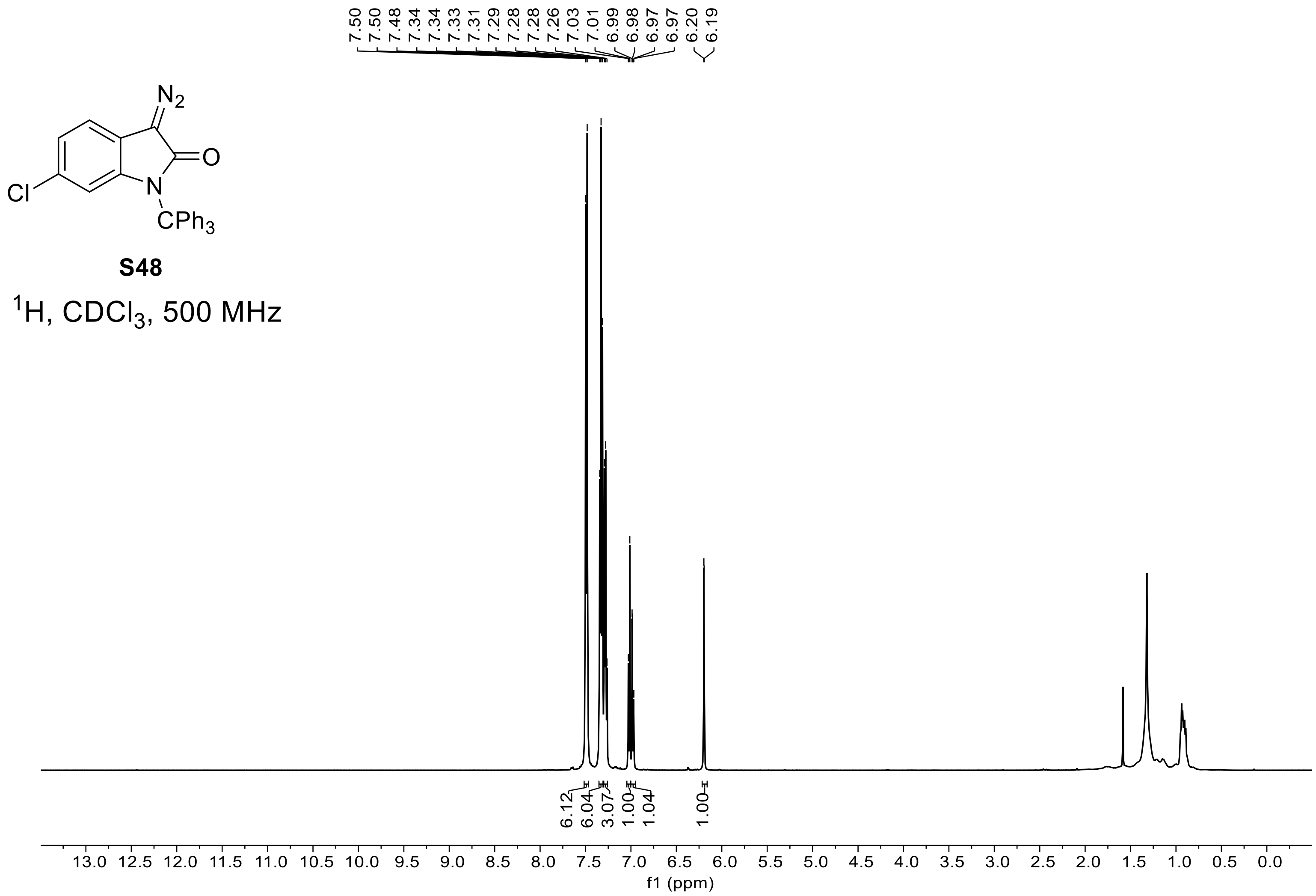

Fig. S137: ^{1}H NMR spectrum for 6-Chloro-3-diazo-1-tritylindolin-2-one (**S48**).

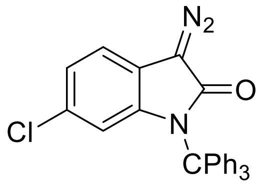

S48

$^{13}C\{^1H\}$, $CDCl_3$, 126 MHz

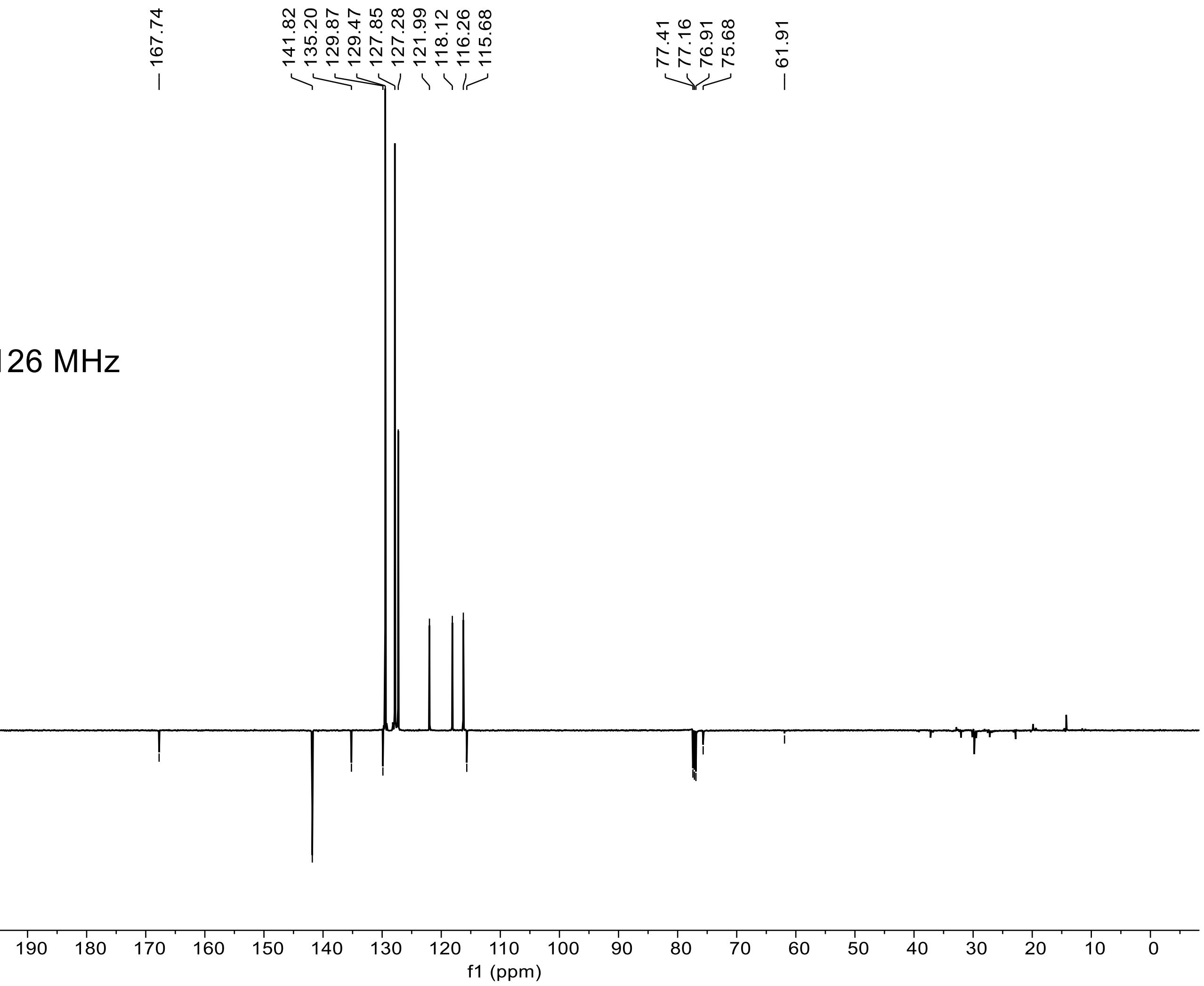

Fig. S138: $^{13}C\{^1H\}$ NMR spectrum for 6-Chloro-3-diazo-1-tritylindolin-2-one (**S48**).

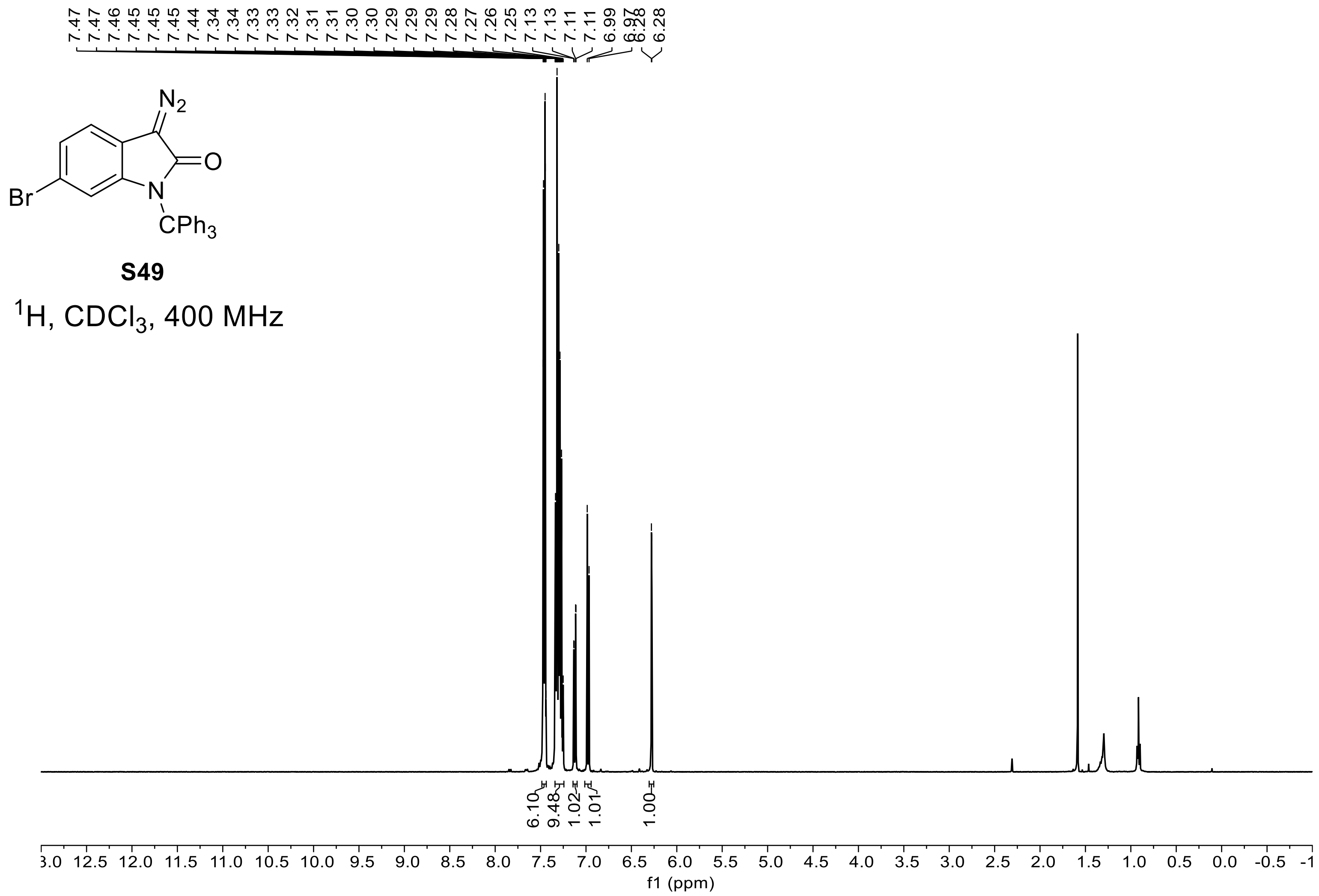

Fig. S139: ^{1}H NMR spectrum for 6-Bromo-3-diazo-1-tritylindolin-2-one (**S49**).

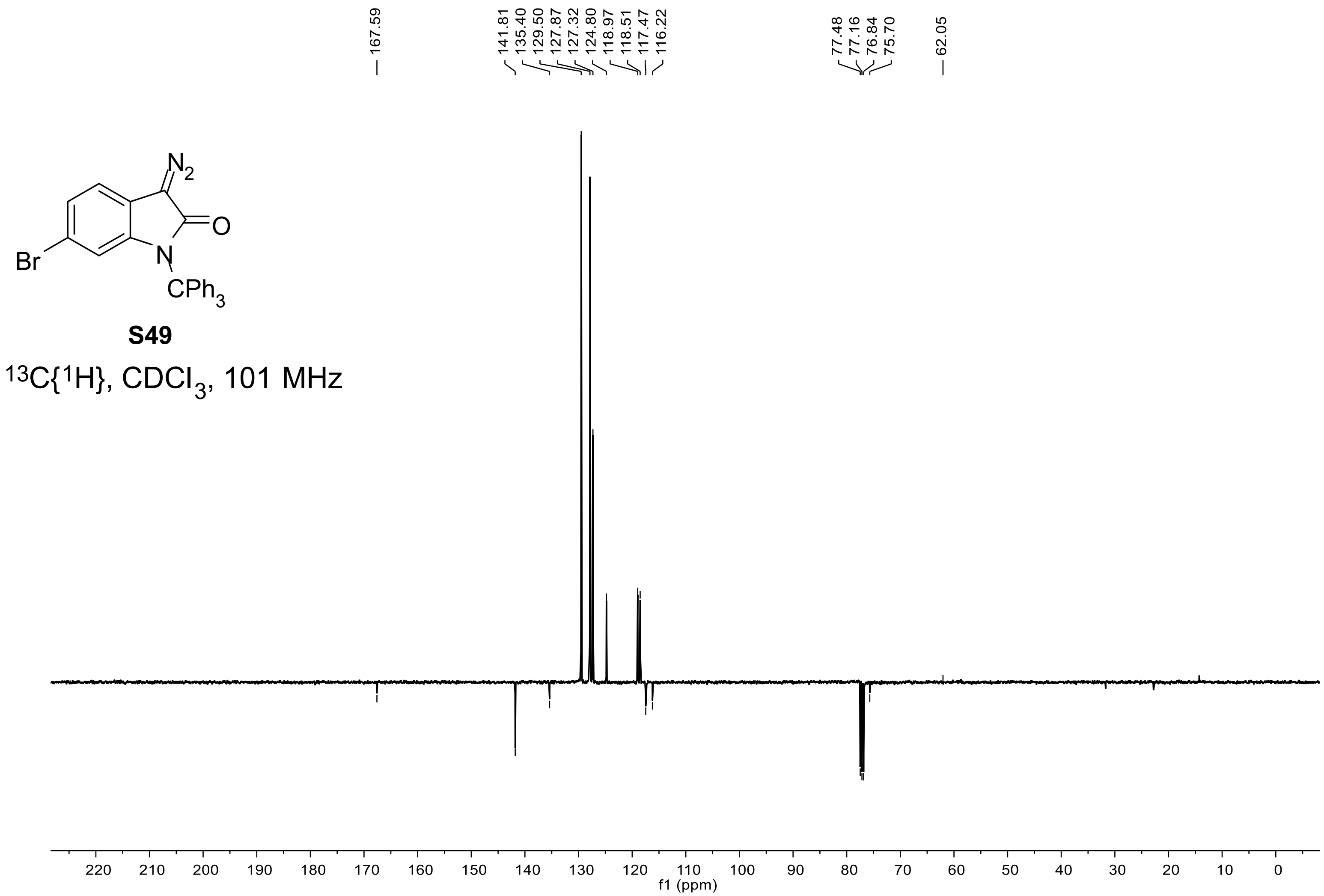

Fig. S140: $^{13}C\{^1H\}$ NMR spectrum for 6-Bromo-3-diazo-1-tritylindolin-2-one (**S49**).

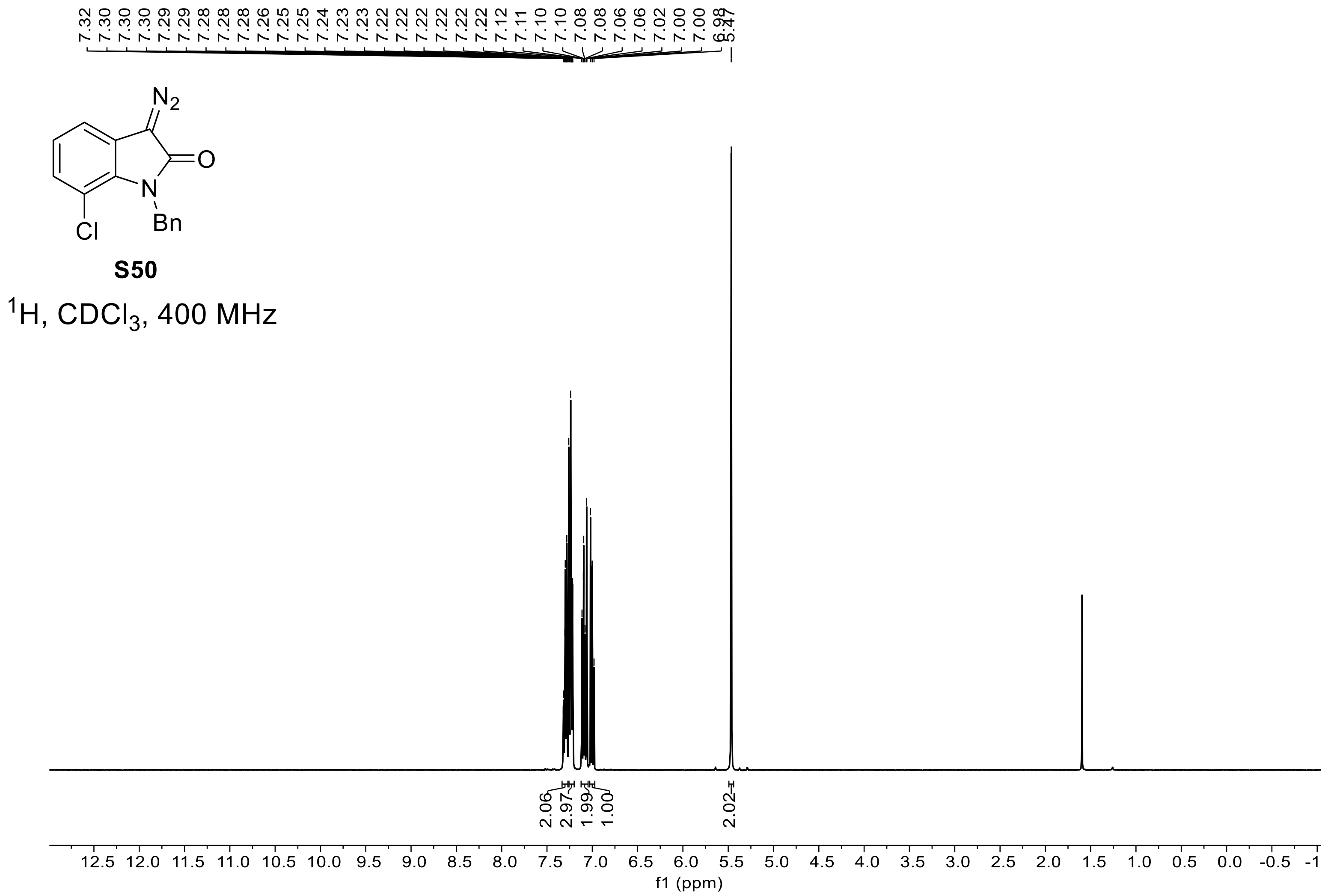

Fig. S141: ^{1}H NMR spectrum for 1-Benzyl-7-chloro-3-diazoindolin-2-one (**S50**).

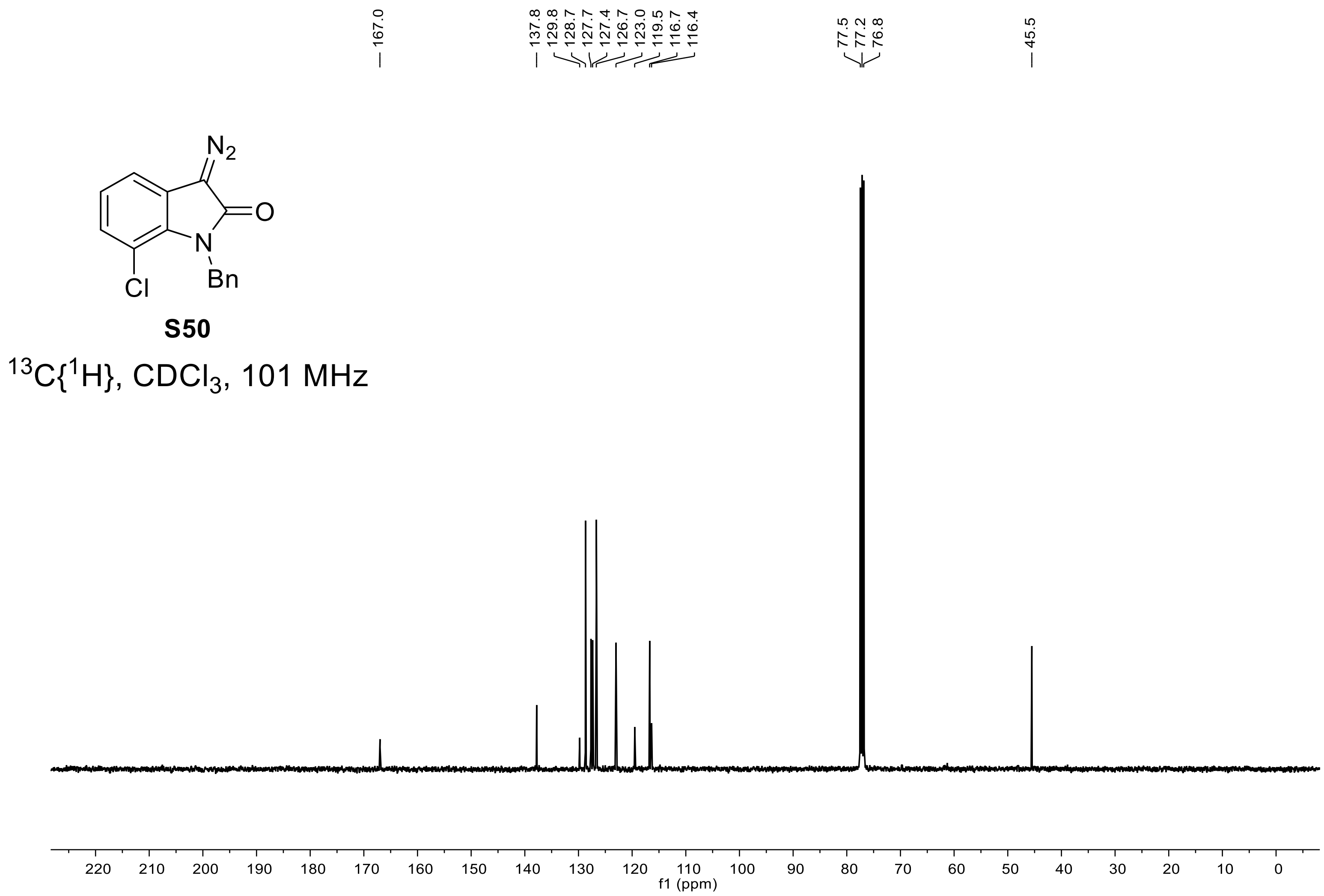

Fig. S142: $^{13}C\{^1H\}$ NMR spectrum for 1-Benzyl-7-chloro-3-diazoindolin-2-one (**S50**).

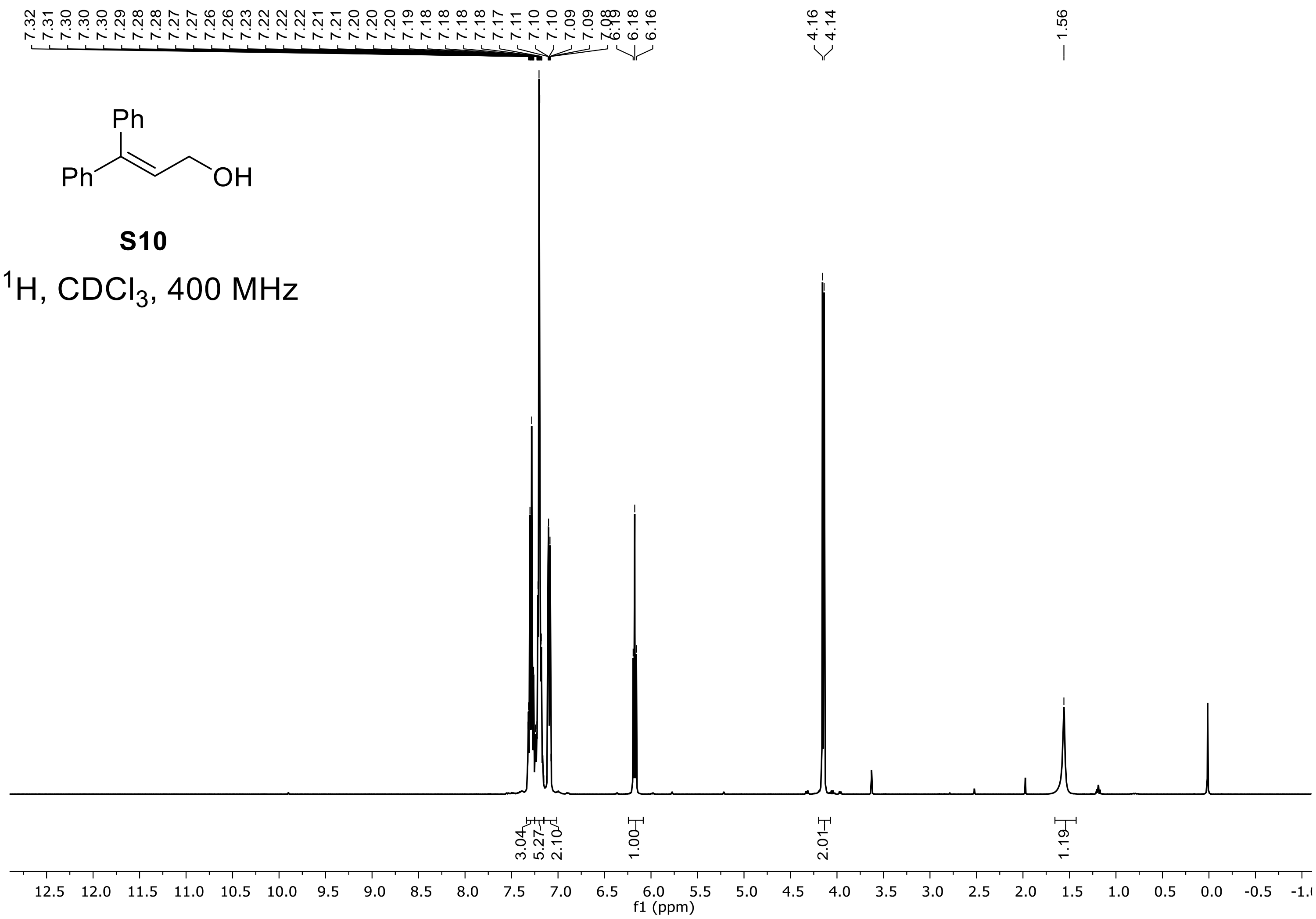

Fig. S143: ^{1}H NMR spectrum for 3,3-Diphenylprop-2-en-1-ol (**S10**).

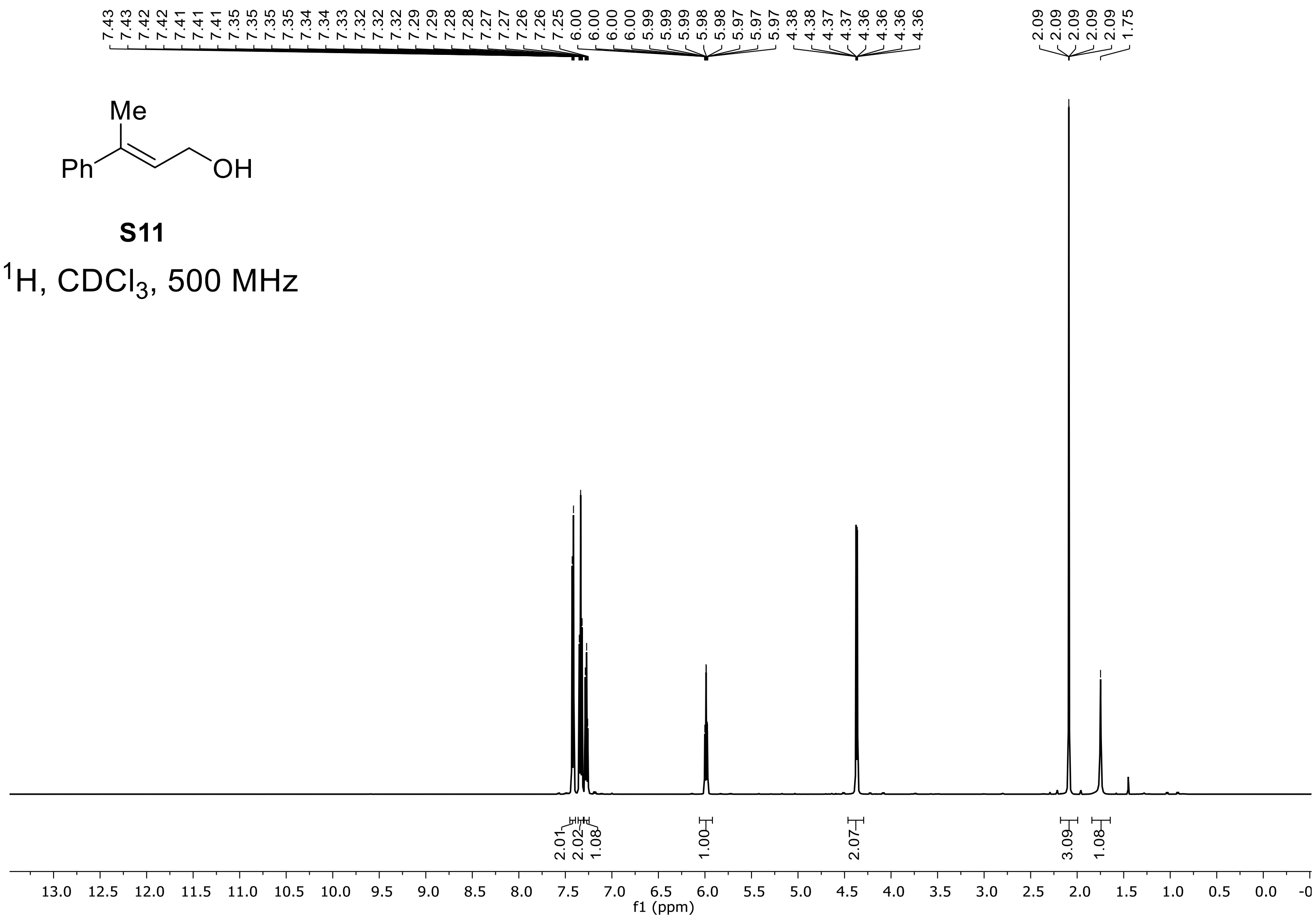

Fig. S144: ^{1}H NMR spectrum for (*E*)-3-Phenylbut-2-en-1-ol (**S11**).

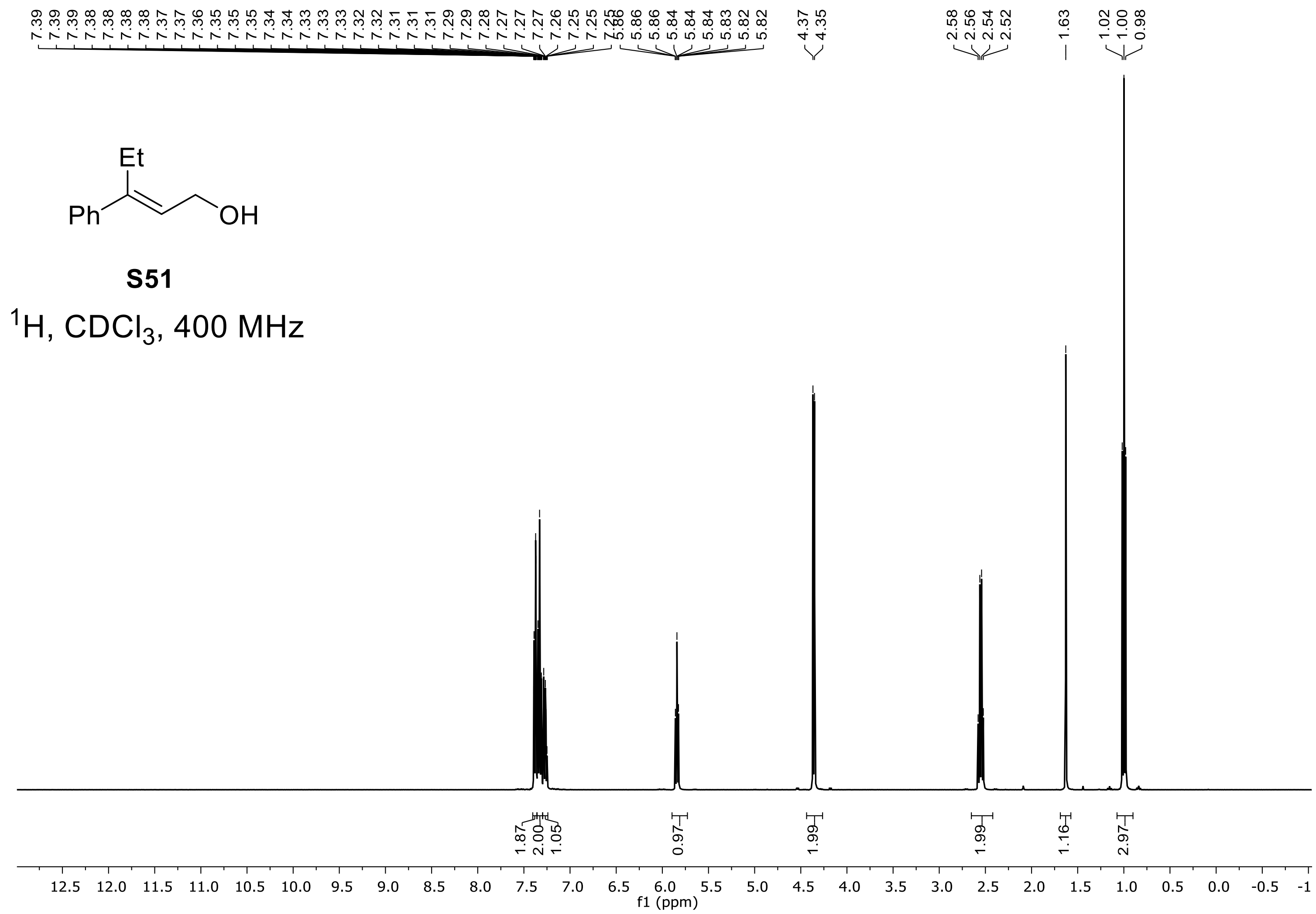

Fig. S145: ^{1}H NMR spectrum for (*E*)-3-Phenylpent-2-en-1-ol (**S51**).

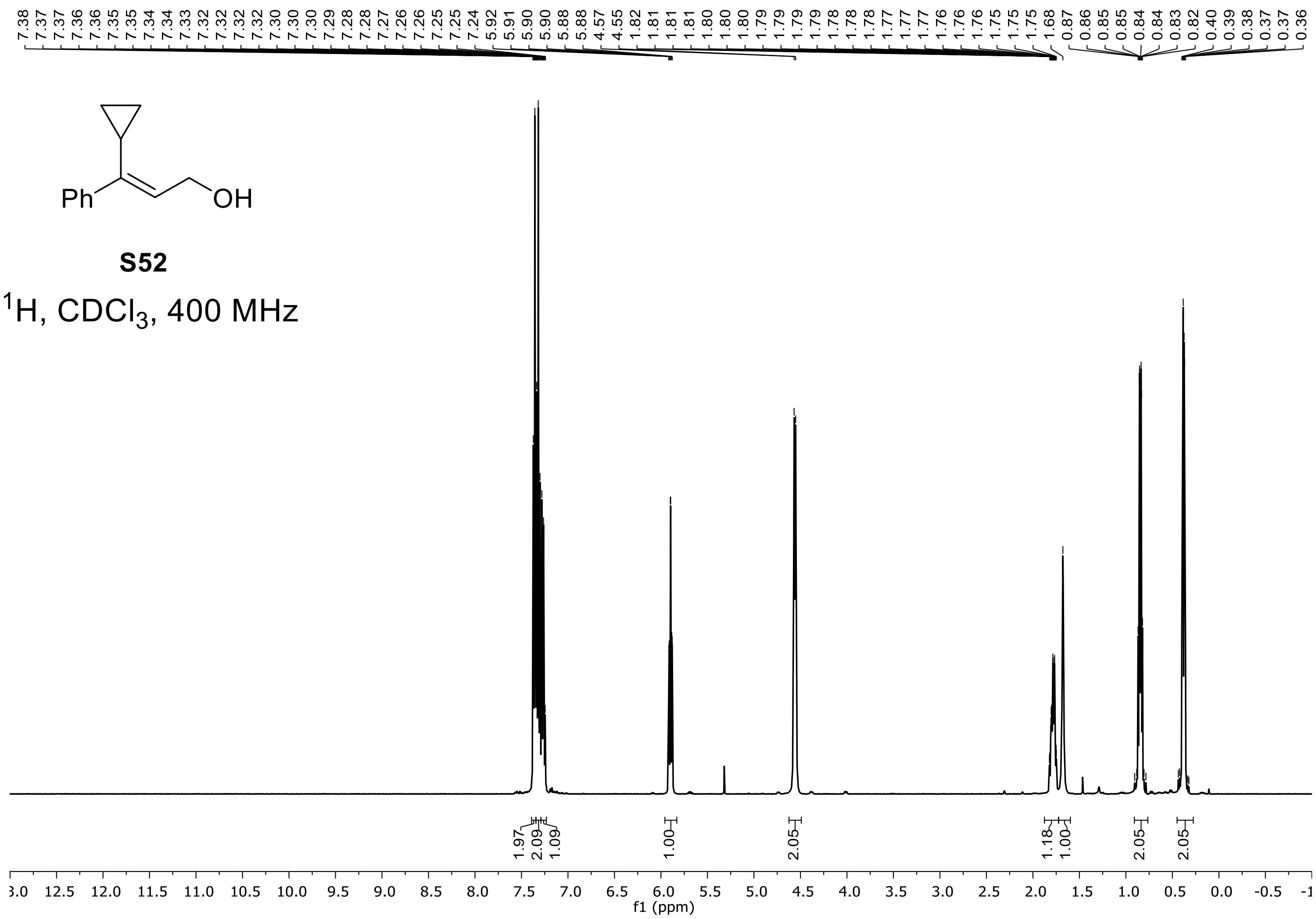

Fig. S146: ^{1}H NMR spectrum for (*E*)-3-Cyclopropyl-3-phenylprop-2-en-1-ol (**S52**).

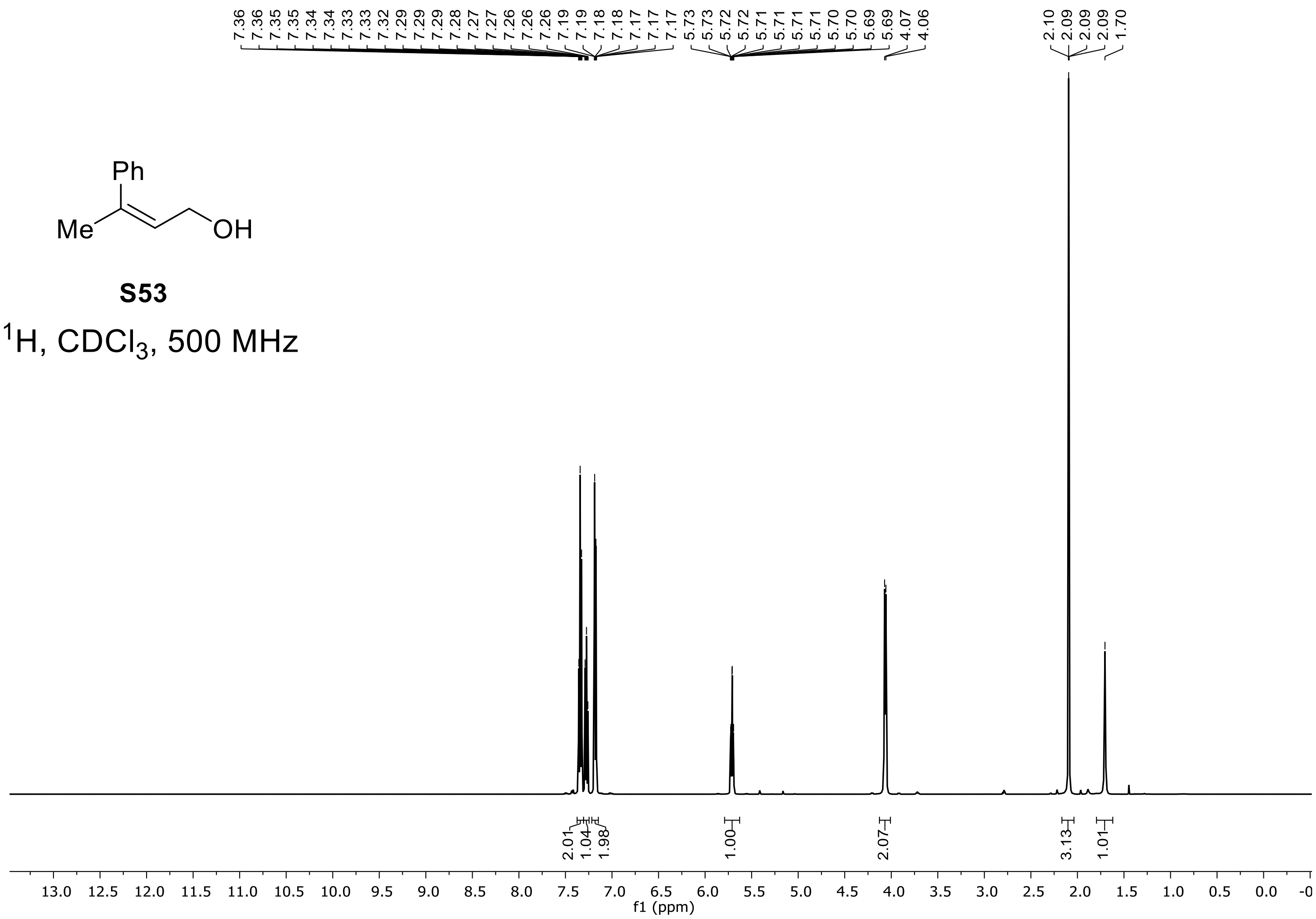

Fig. S147: ^{1}H NMR spectrum for (*Z*)-3-Phenylbut-2-en-1-ol (**S53**).

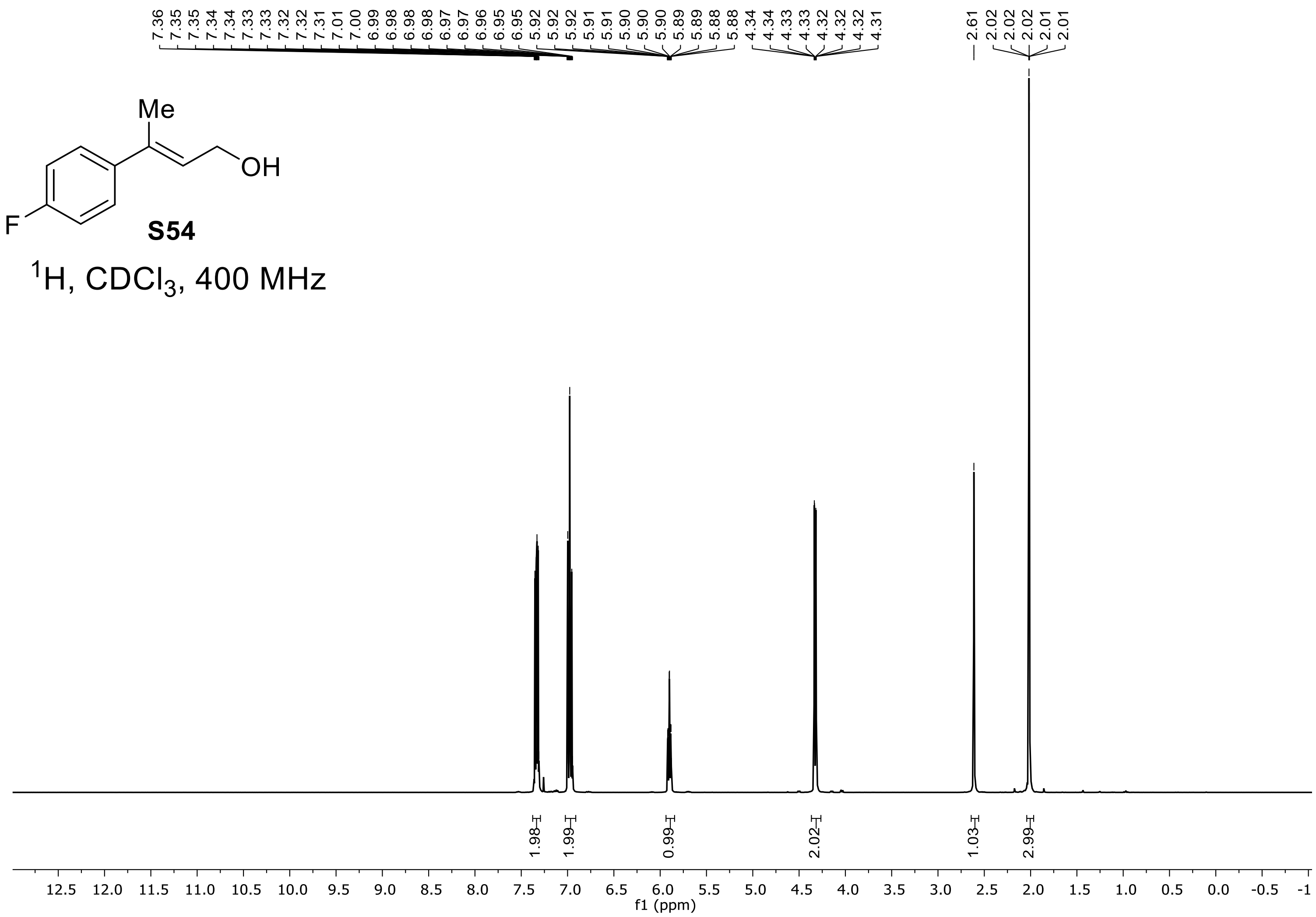

Fig. S148: ^{1}H NMR spectrum for (*E*)-3-(4-Fluorophenyl)but-2-en-1-ol (**S54**).

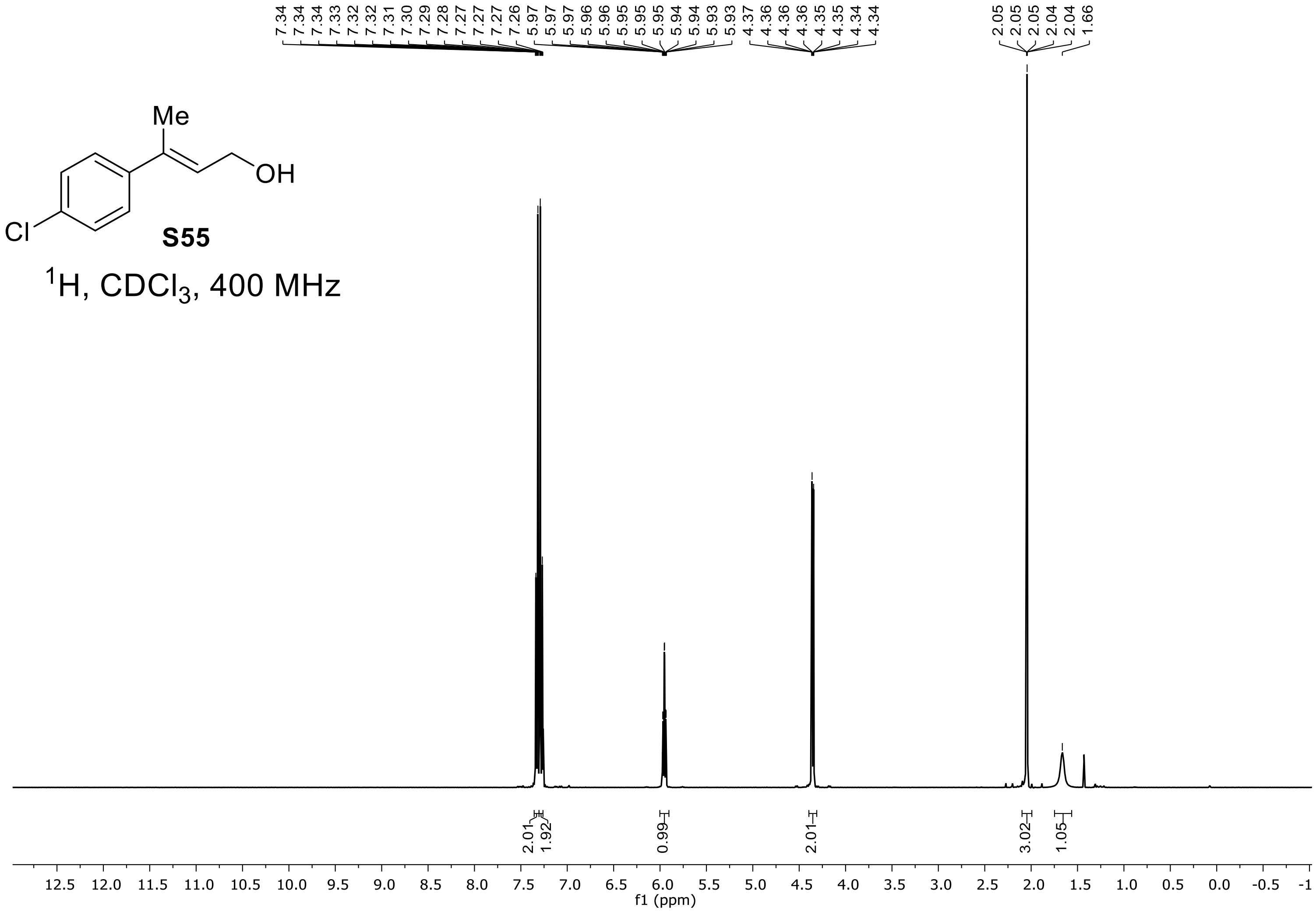

Fig. S149: ^{1}H NMR spectrum for (*E*)-3-(4-chlorophenyl)but-2-en-1-ol (**S55**).

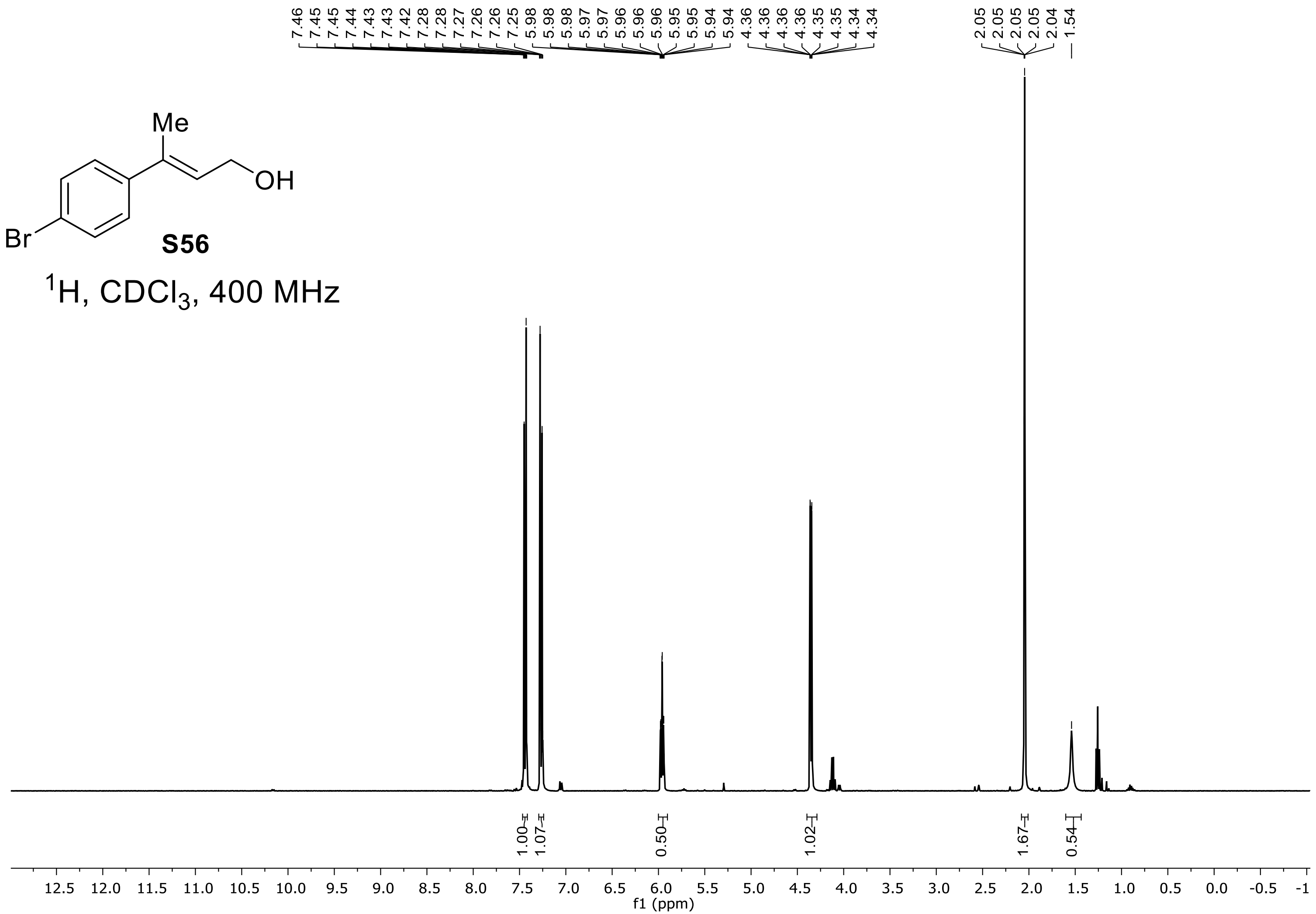

Fig. S150: ^{1}H NMR spectrum for (*E*)-3-(4-Bromophenyl)but-2-en-1-ol (**S56**).

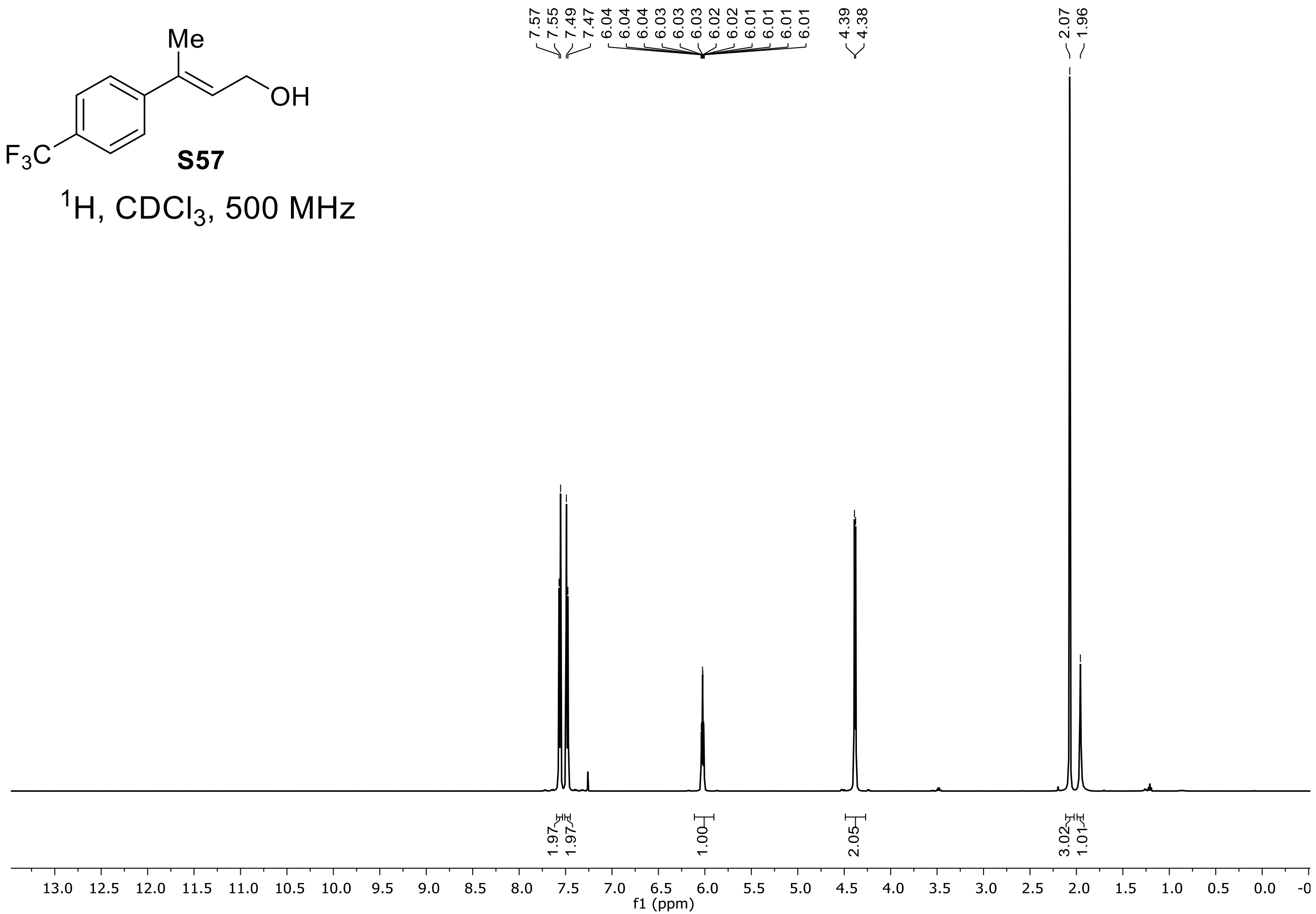

Fig. S151: ^{1}H NMR spectrum for (*E*)-3-(4-(Trifluoromethyl)phenyl)but-2-en-1-ol (**S57**).

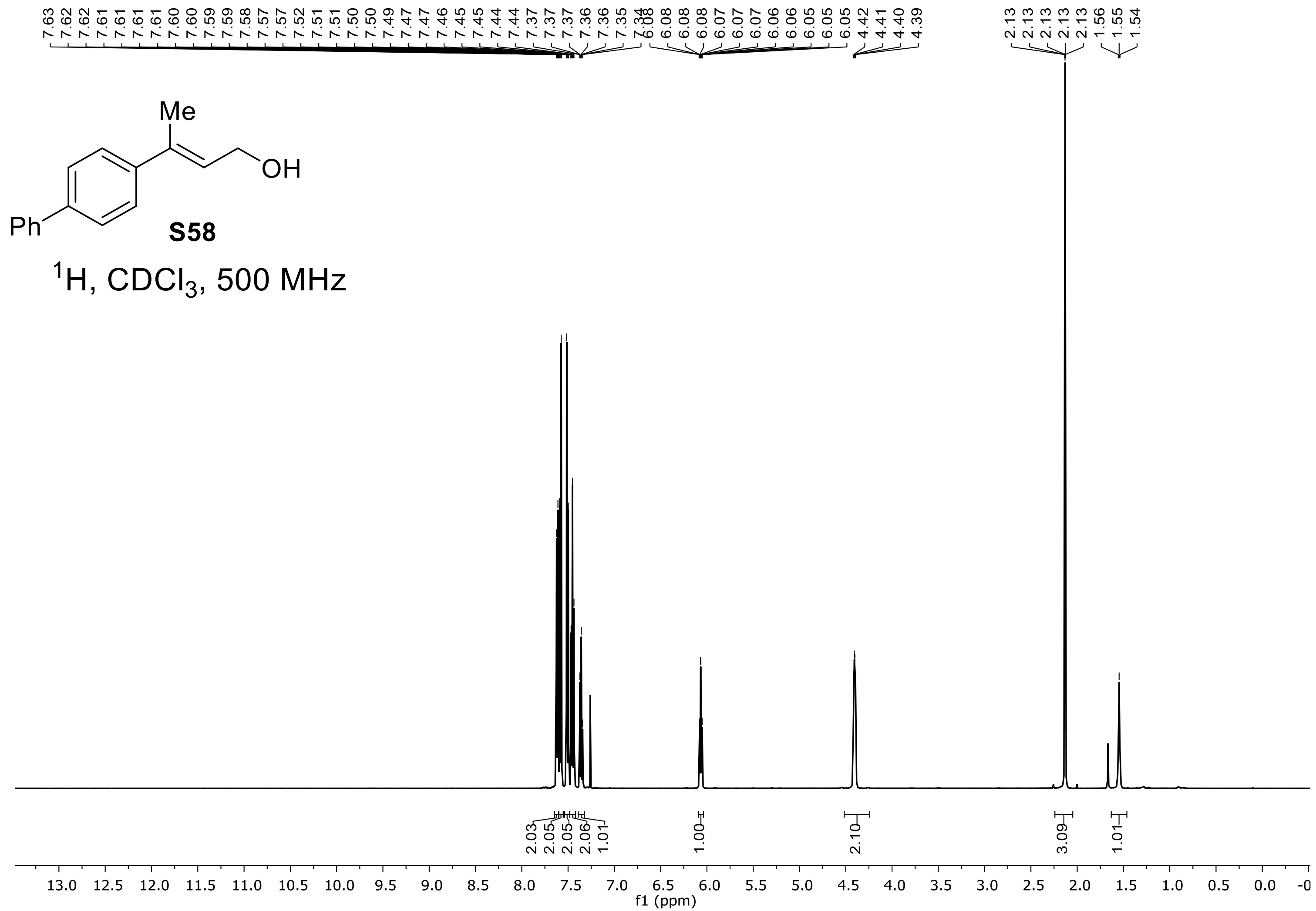

Fig. S152: ^{1}H NMR spectrum for (*E*)-3-([1,1'-Biphenyl]-4-yl)but-2-en-1-ol (**S58**).

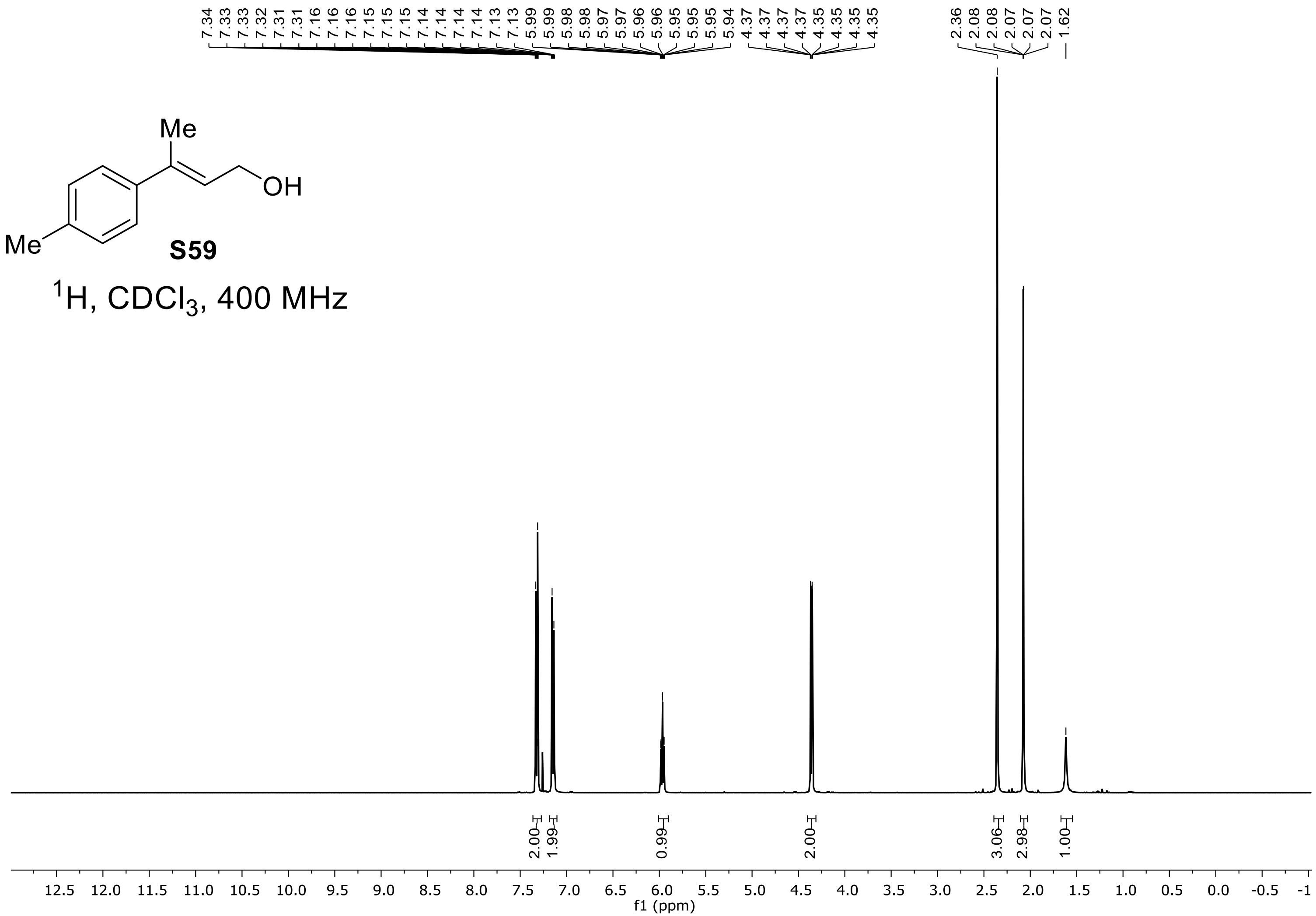

Fig. S153: ^{1}H NMR spectrum for (*E*)-3-(*p*-Tolyl)but-2-en-1-ol (**S59**).

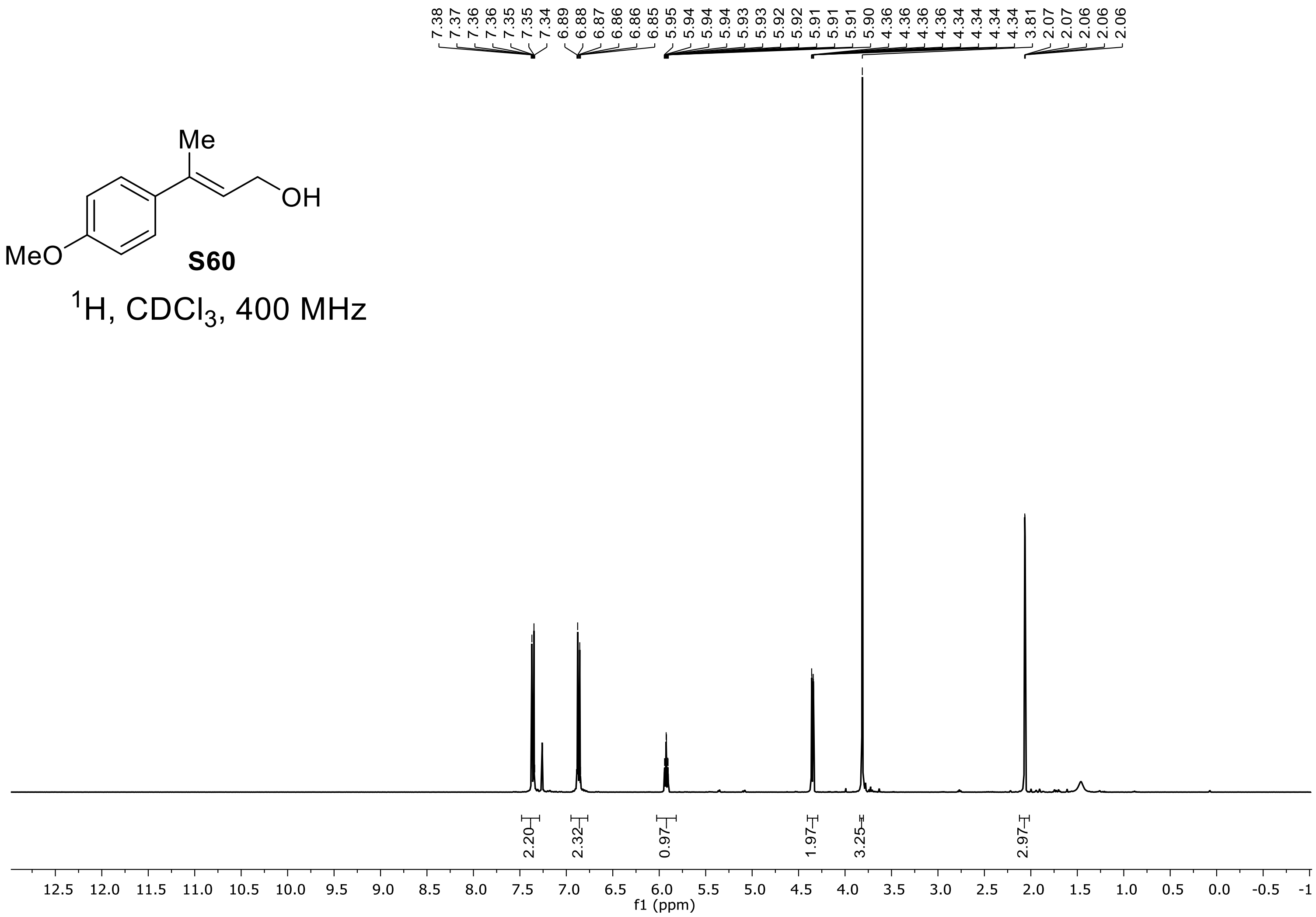

Fig. S154: ^{1}H NMR spectrum for (*E*)-3-(4-methoxyphenyl)but-2-en-1-ol (**S60**).

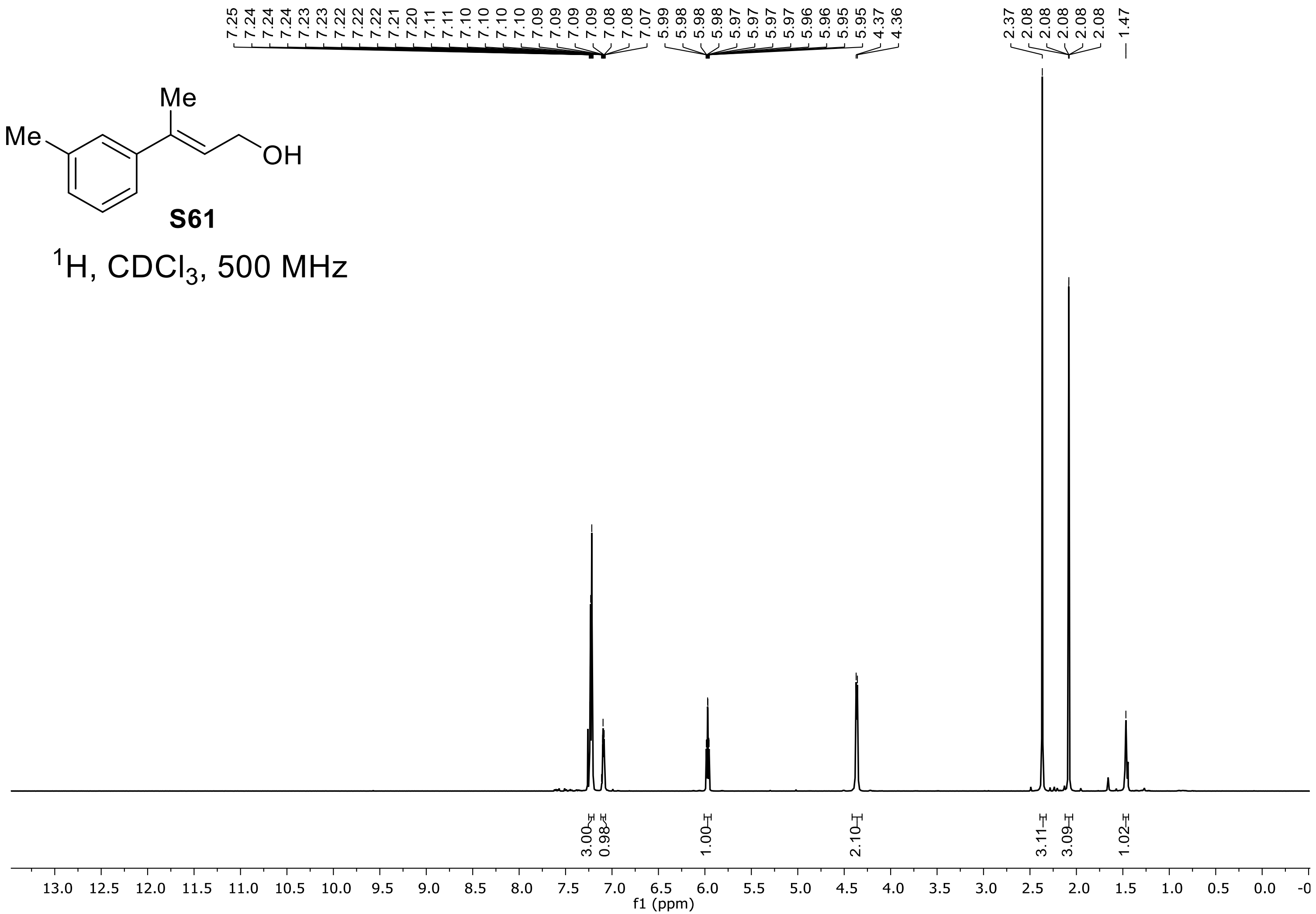

Fig. S155: ^{1}H NMR spectrum for (*E*)-3-(*m*-Tolyl)but-2-en-1-ol (**S61**).

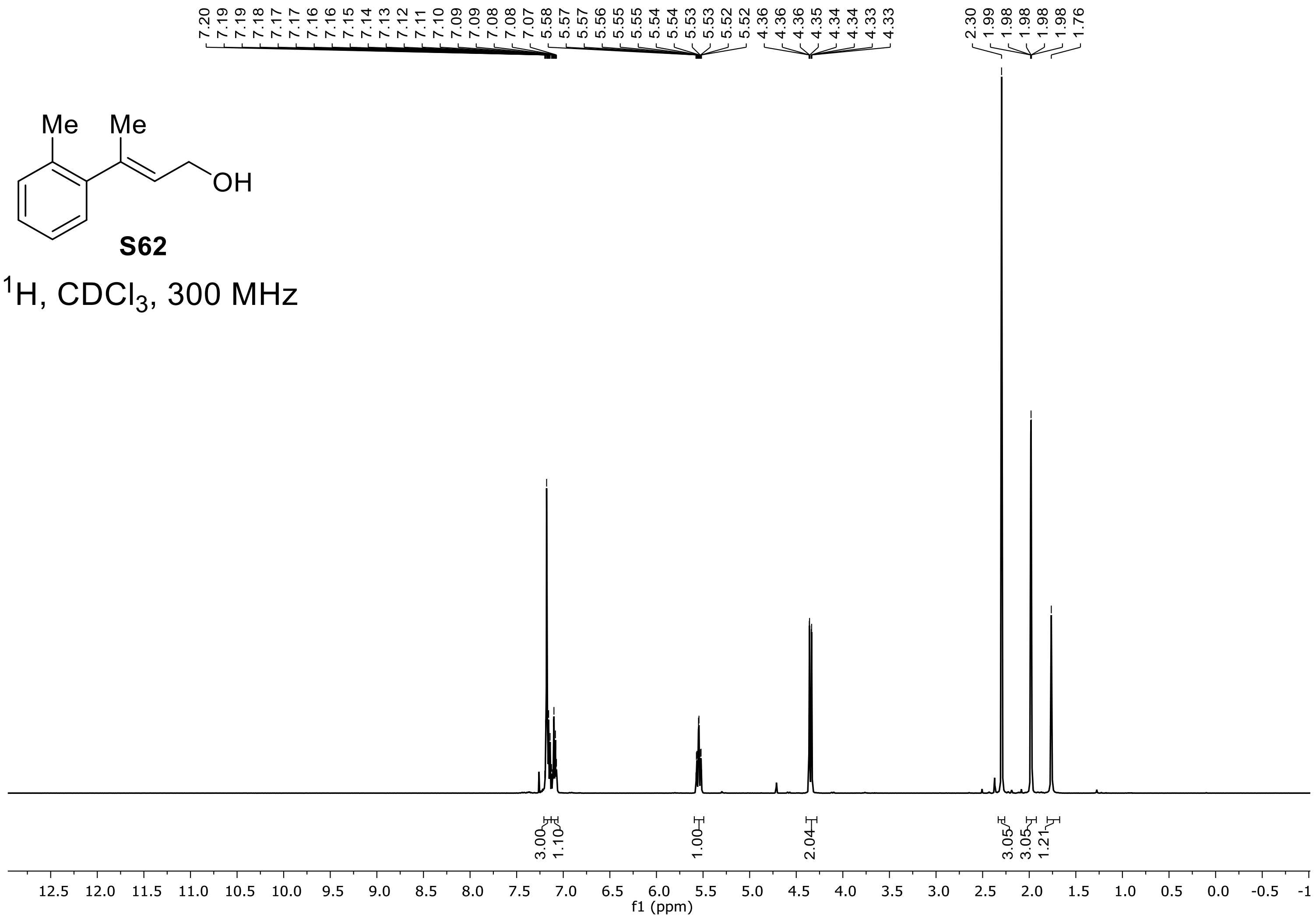

Fig. S156: ^{1}H NMR spectrum for (*E*)-3-(*o*-Tolyl)but-2-en-1-ol (**S62**).

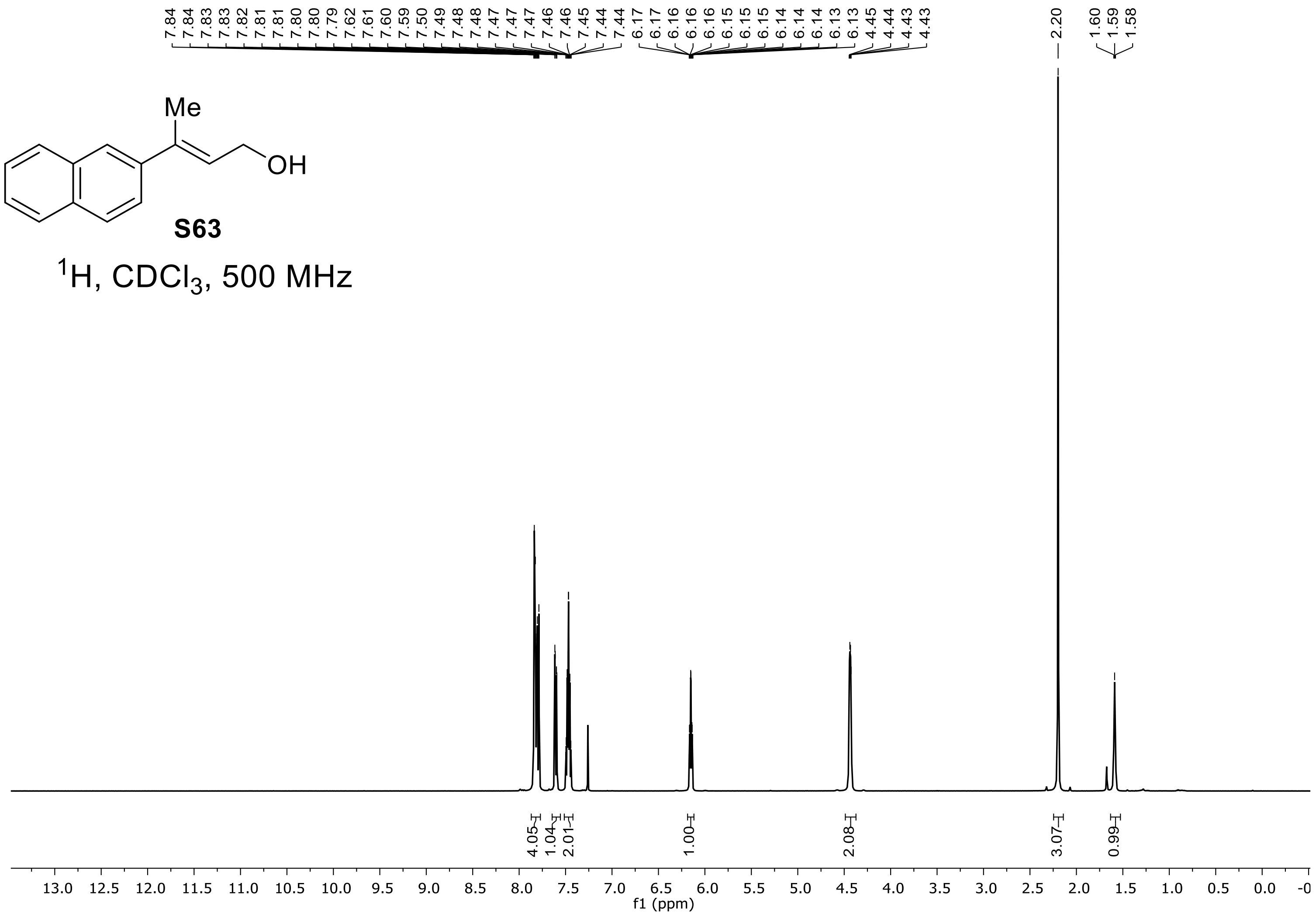

Fig. S157: ^{1}H NMR spectrum for (*E*)-3-(Naphth-2-yl)but-2-en-1-ol (**S63**).

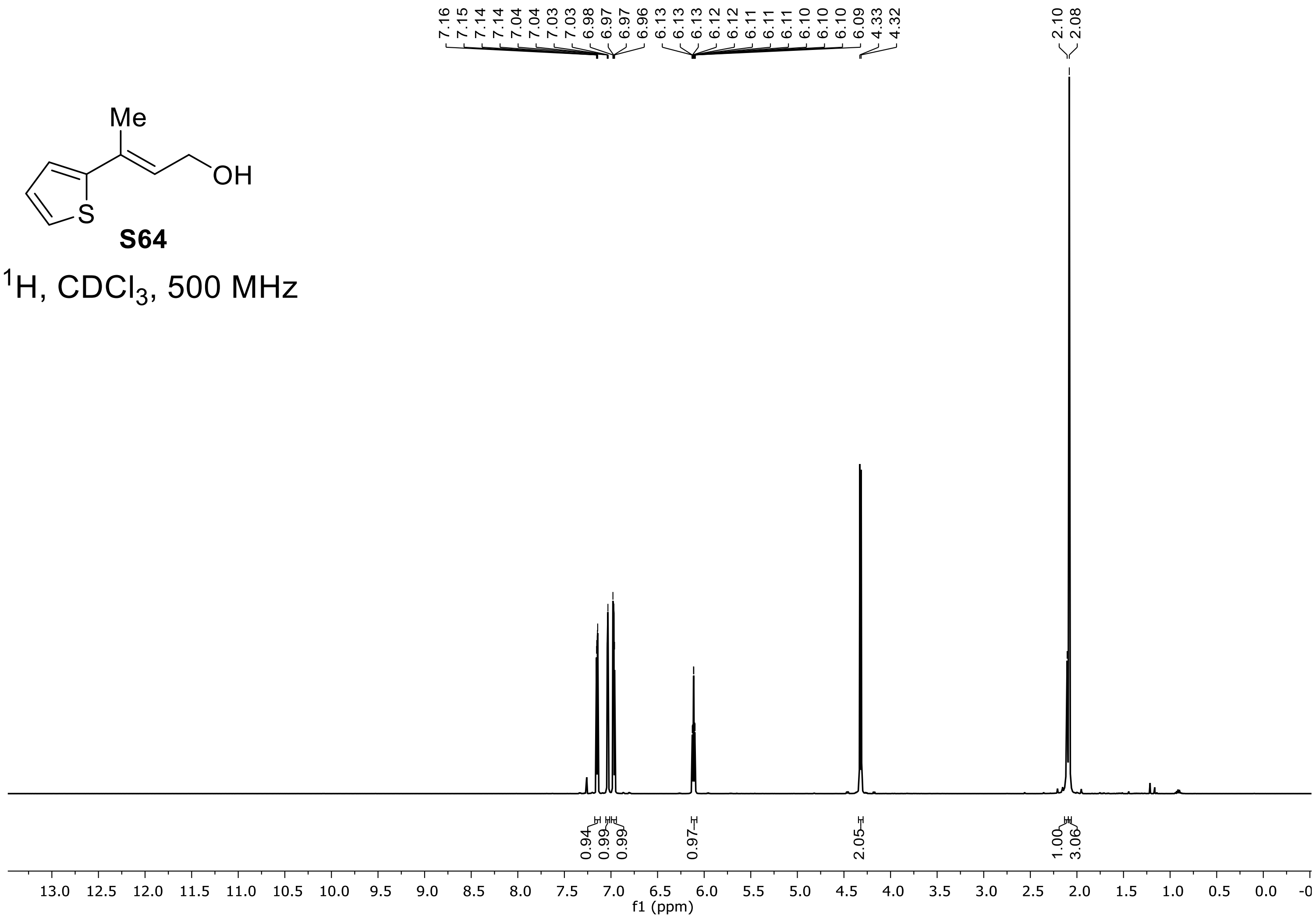

Fig. S158: ^{1}H NMR spectrum for (*E*)-3-(Thiophen-2-yl)but-2-en-1-ol (**S64**).

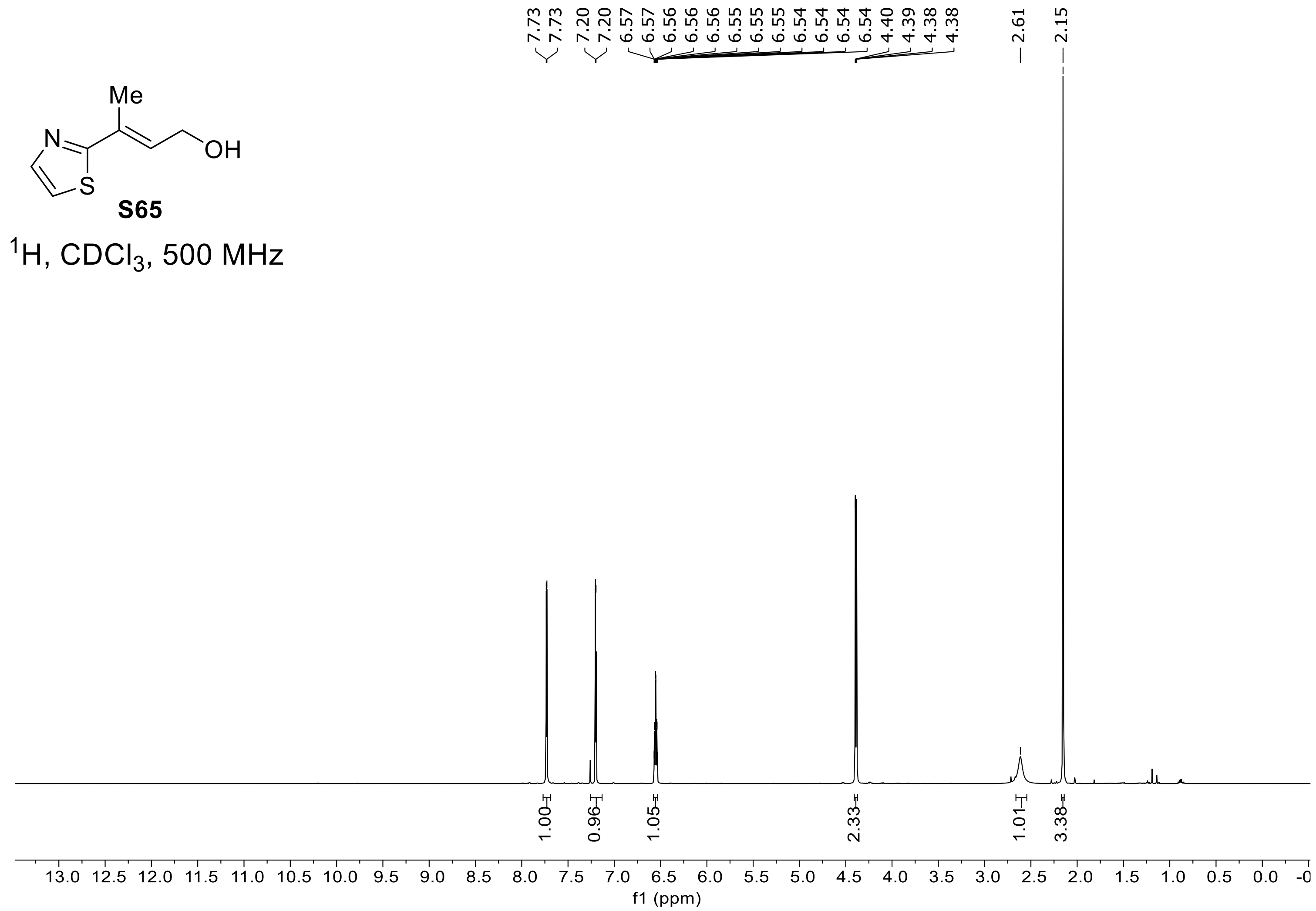

Fig. S159: ^{1}H NMR spectrum for (*E*)-3-(Thiazol-2-yl)but-2-en-1-ol (**S65**).

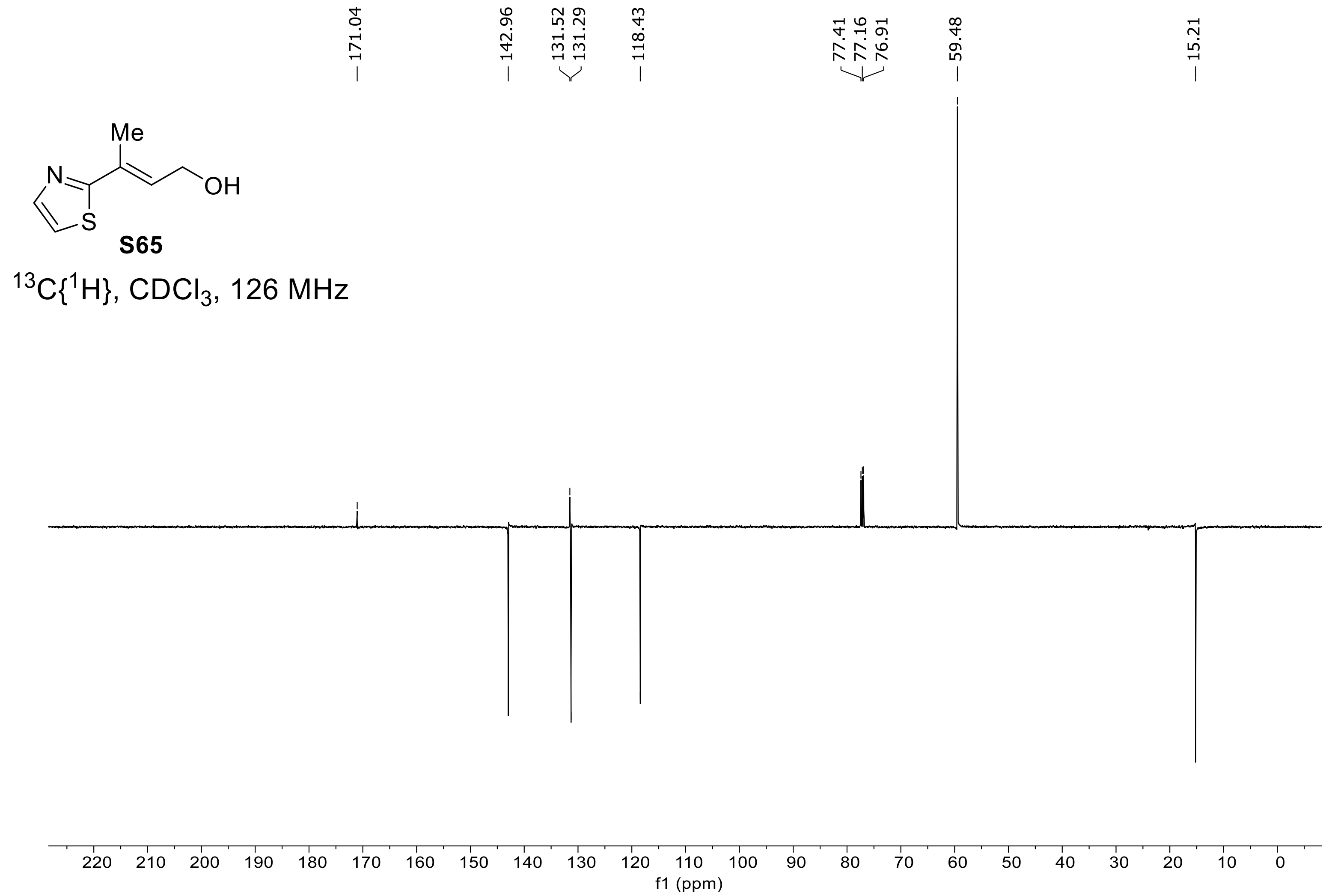

Fig. S160: ^{1}H NMR spectrum for (*E*)-3-(Thiazol-2-yl)but-2-en-1-ol (**S65**).

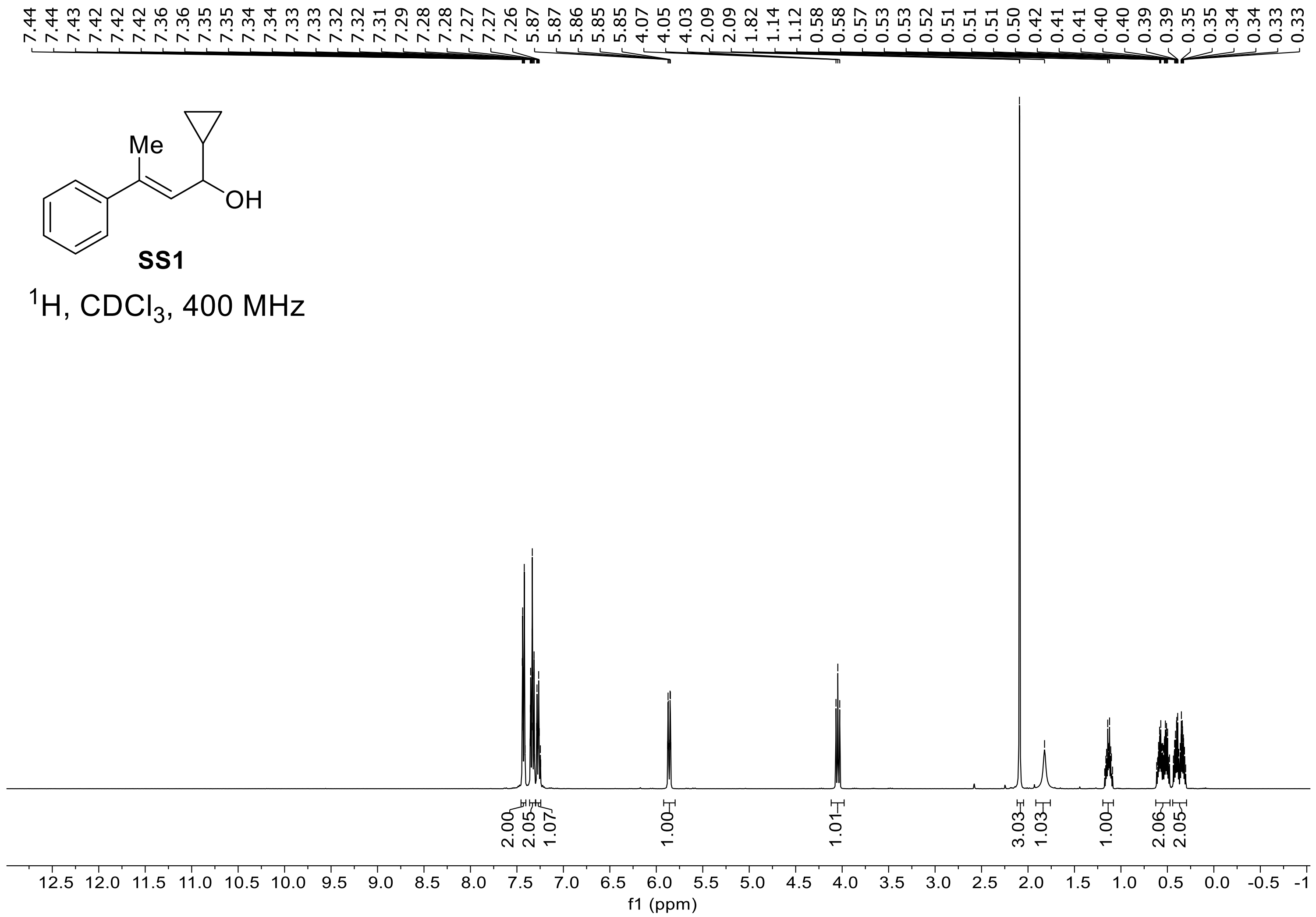

Fig. S161: ^{1}H NMR spectrum for (*E*)-1-cyclopropyl-3-methyl-3-phenylprop-2-en-1-ol (**SS1**).

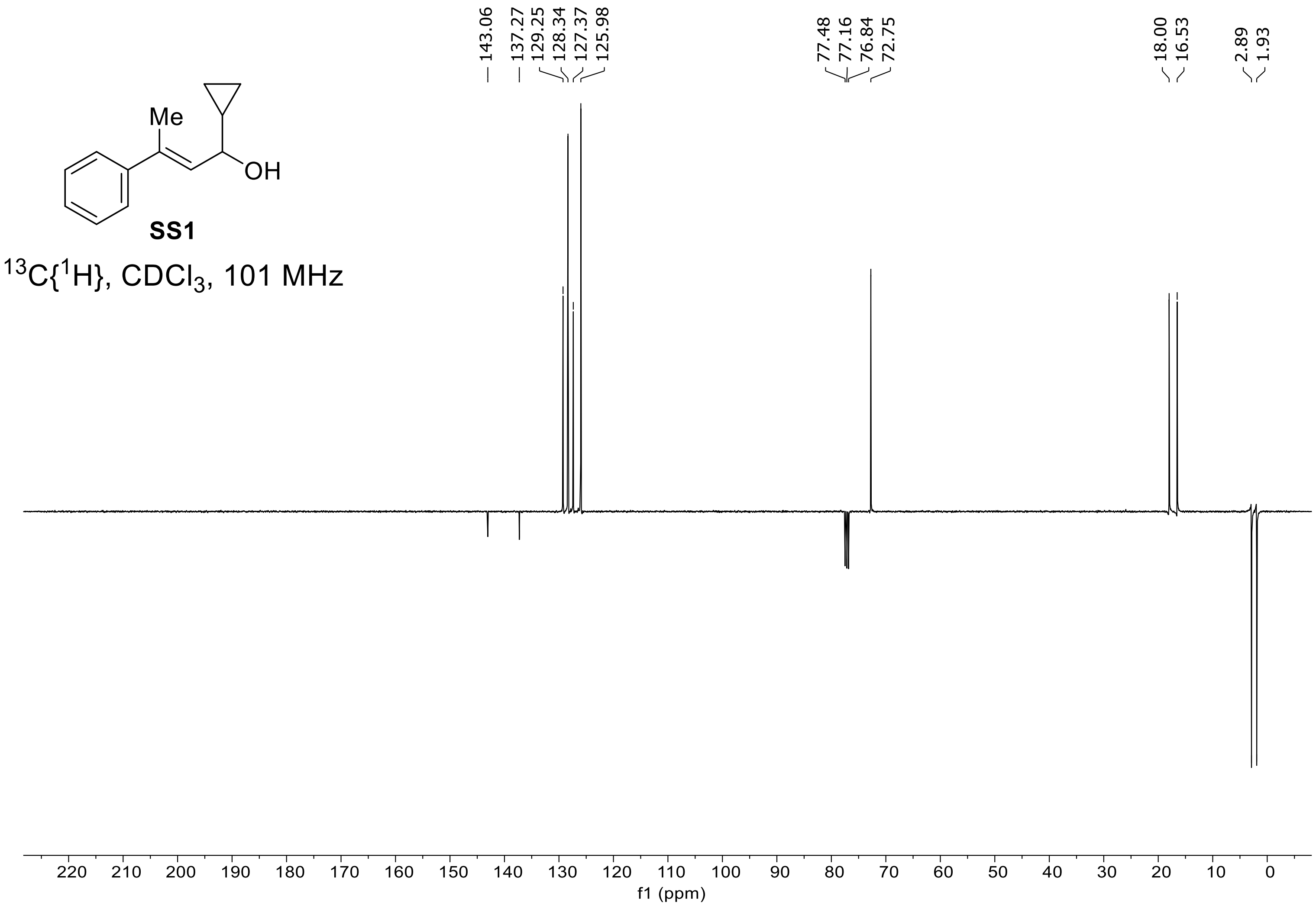

Fig. S162: $^{13}C\{^{1}H\}$ NMR spectrum for (*E*)-1-cyclopropyl-3-methyl-3-phenylprop-2-en-1-ol (**SS1**).

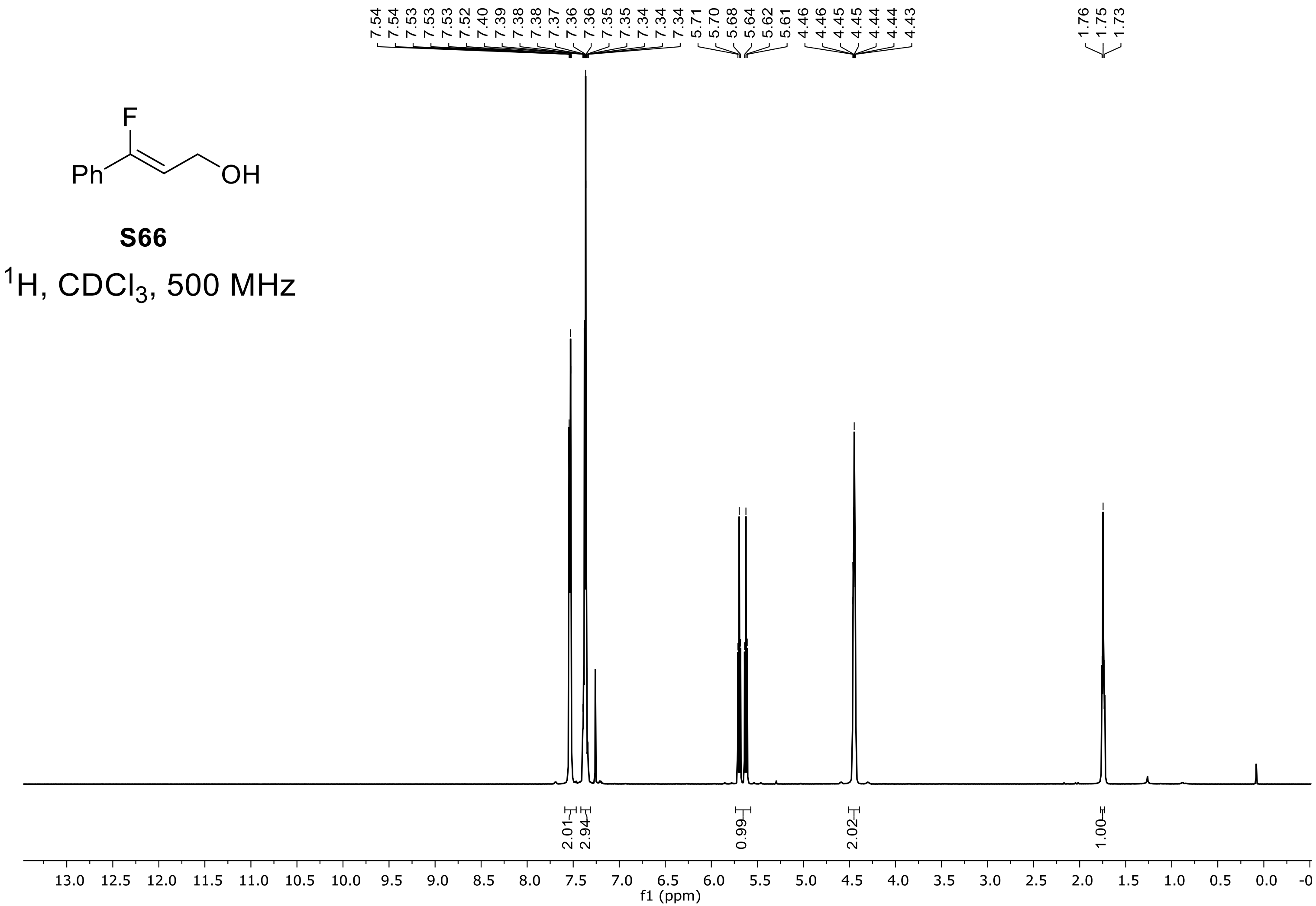

Fig. S163: ^{1}H NMR spectrum for (*Z*)-3-Fluoro-3-phenylprop-2-en-1-ol (**S66**).

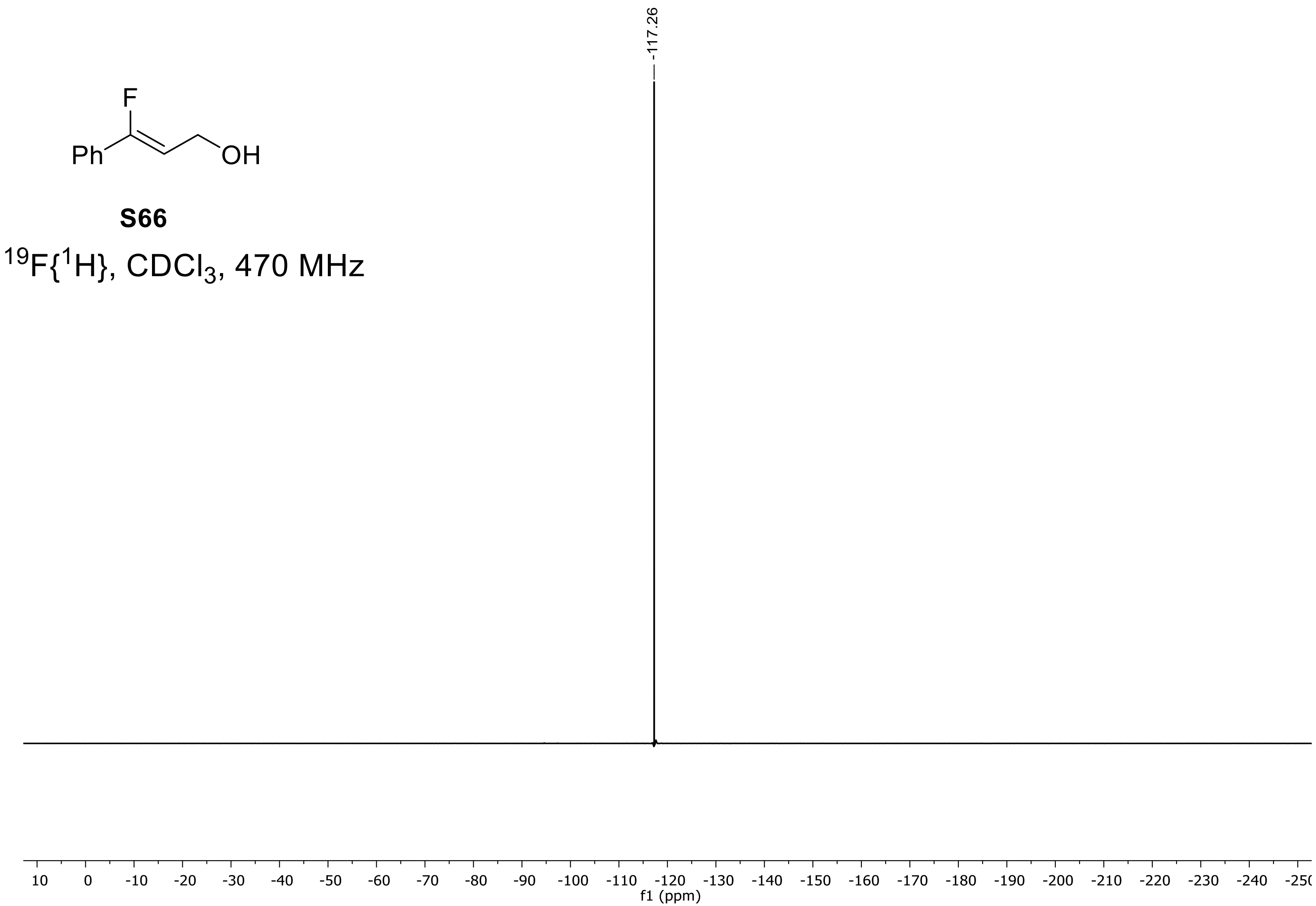

Fig. S164: $^{19}F\{^1H\}$ NMR spectrum for (*Z*)-3-Fluoro-3-phenylprop-2-en-1-ol (**S66**).

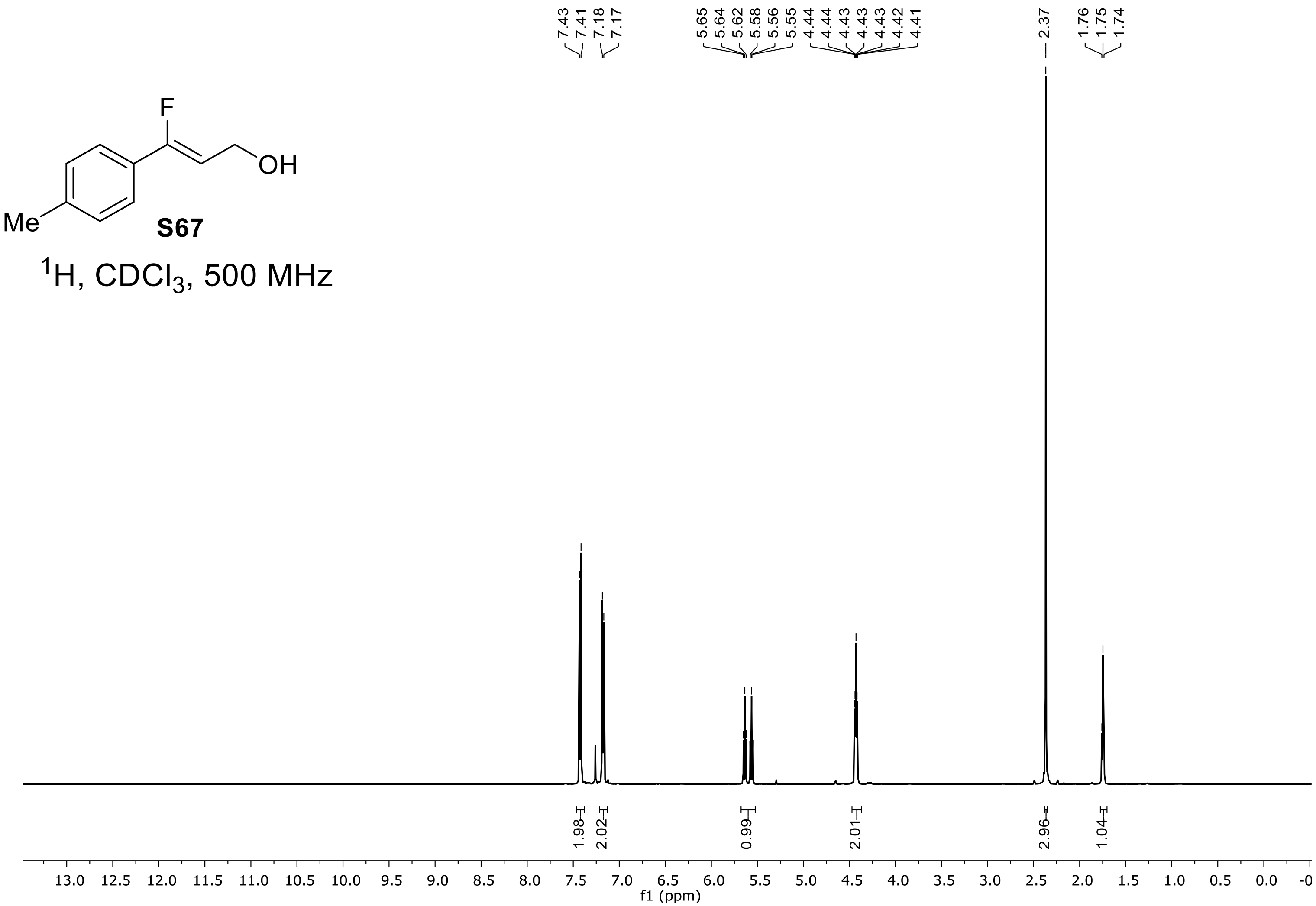

Fig. S165: ^{1}H NMR spectrum for (*Z*)-3-Fluoro-3-(*p*-tolyl)prop-2-en-1-ol (**S67**).

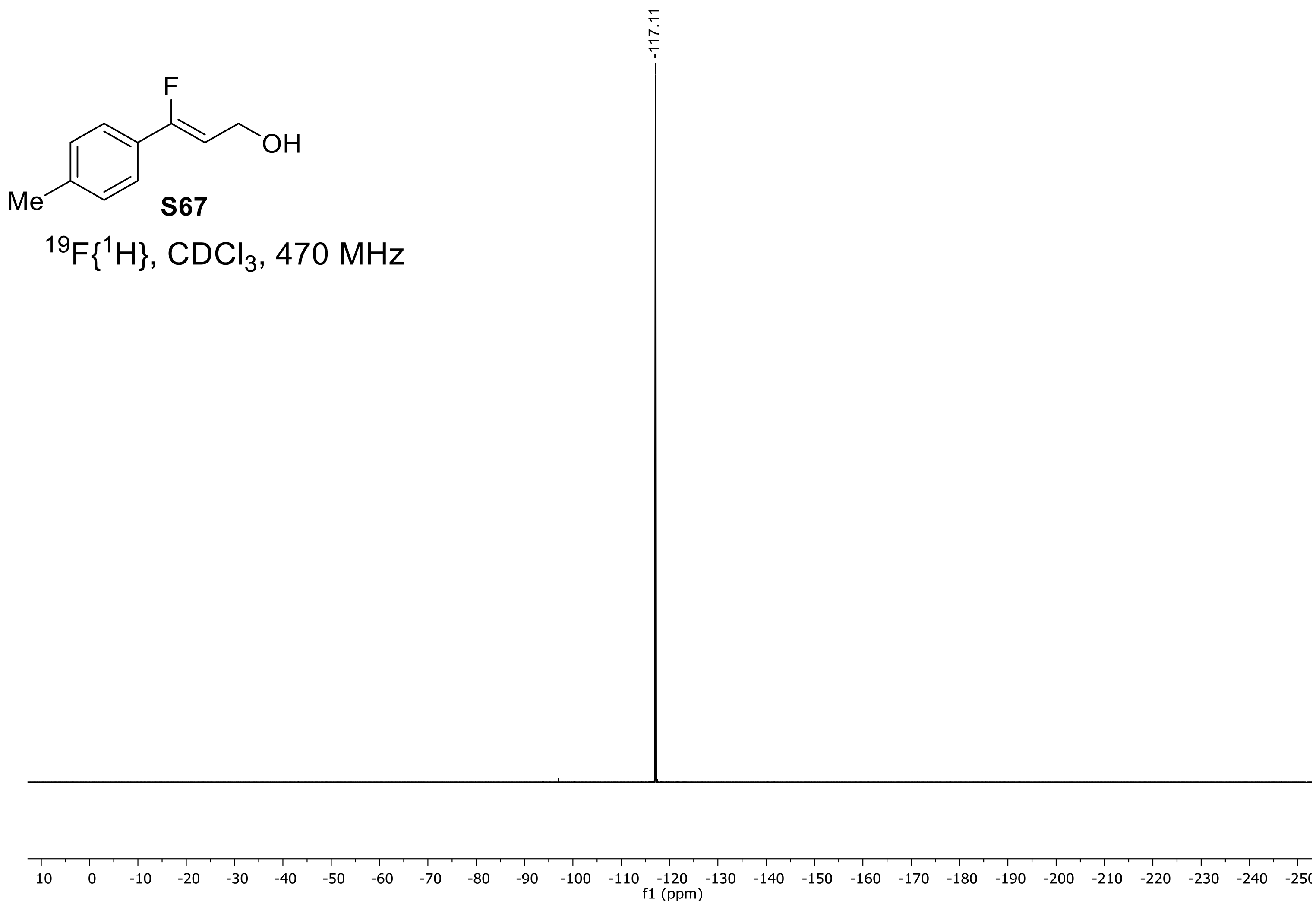

Fig. S166: $^{19}F\{^{1}H\}$ NMR spectrum for (*Z*)-3-Fluoro-3-(*p*-tolyl)prop-2-en-1-ol (**S67**).

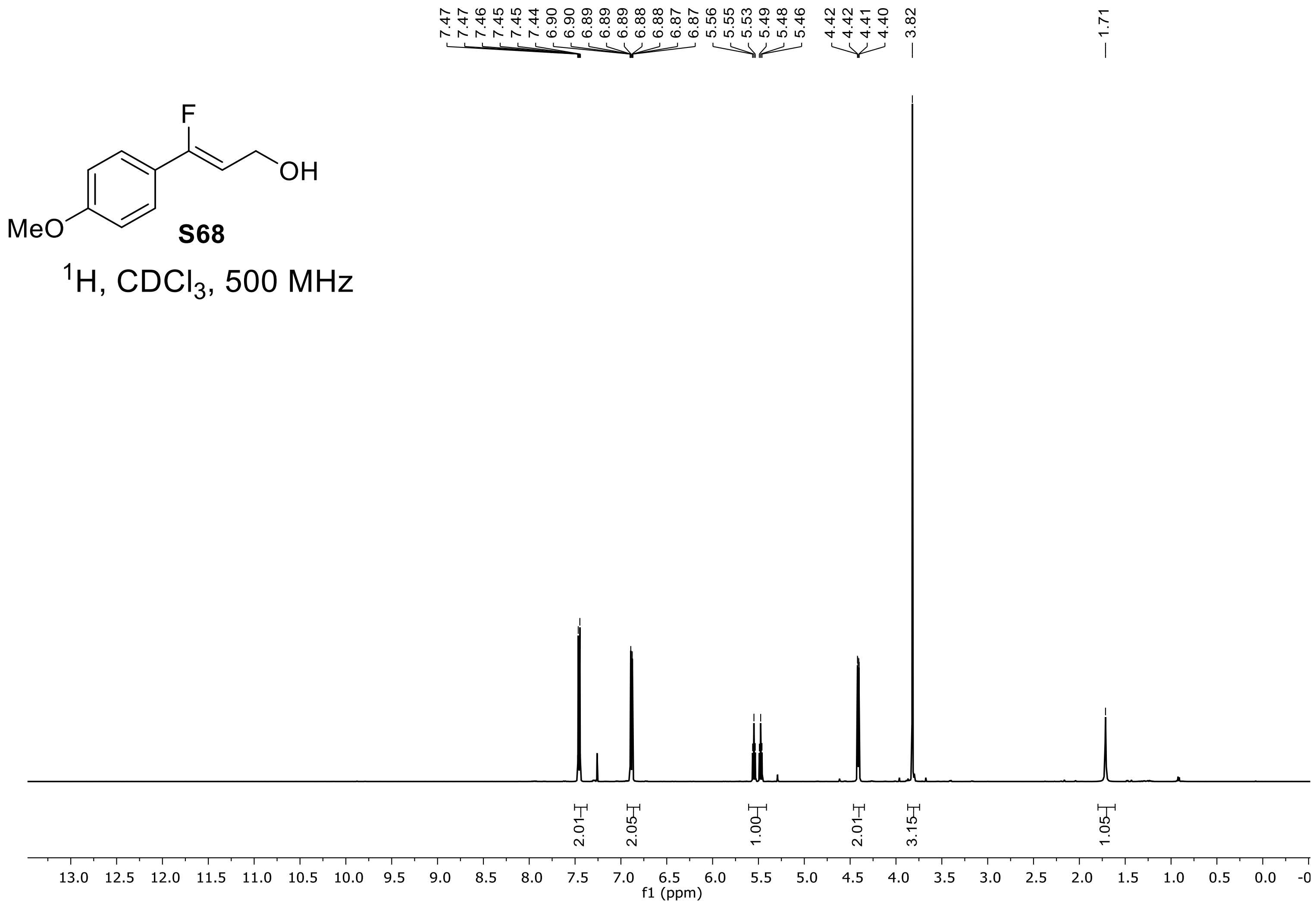

Fig. S167: ^{1}H NMR spectrum for (*Z*)-3-Fluoro-3-(4-methoxyphenyl)prop-2-en-1-ol (**S68**).

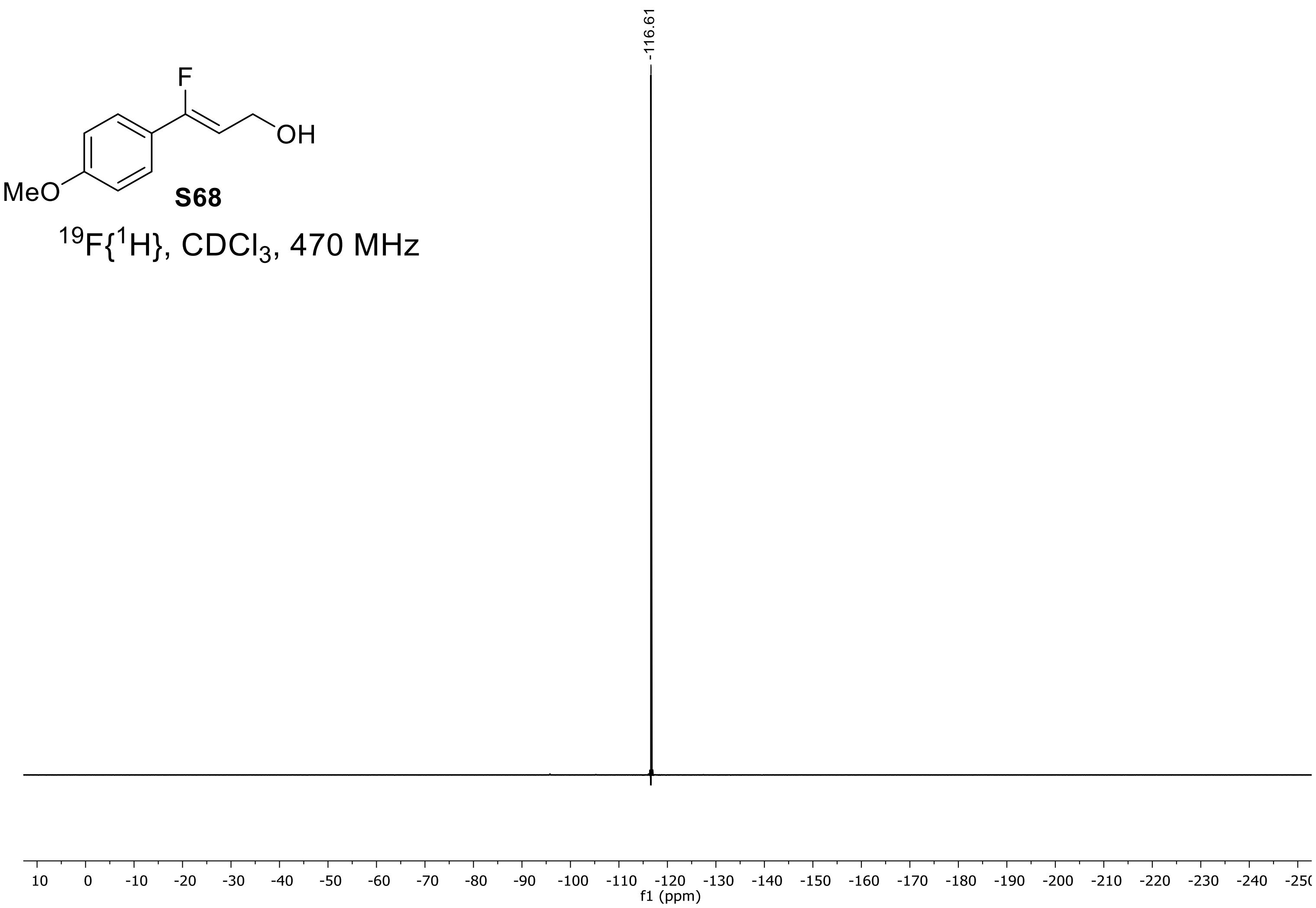

Fig. S168: ^{19}F{^{1}H} NMR spectrum for (*Z*)-3-Fluoro-3-(4-methoxyphenyl)prop-2-en-1-ol (**S68**).

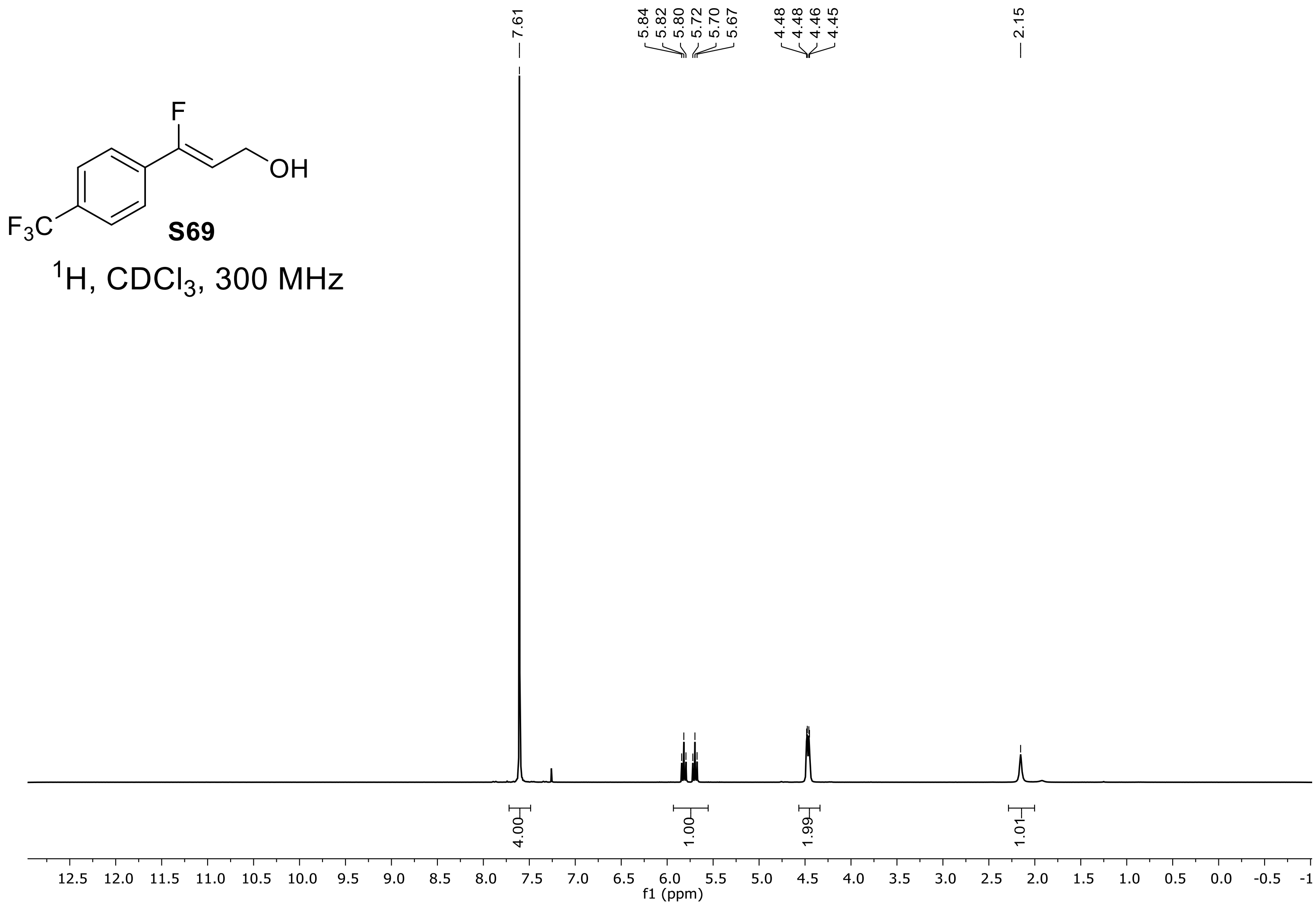

Fig. S169: ^{1}H NMR spectrum for (*Z*)-3-Fluoro-3-[4-(trifluoromethyl)phenyl]prop-2-en-1-ol (**S69**).

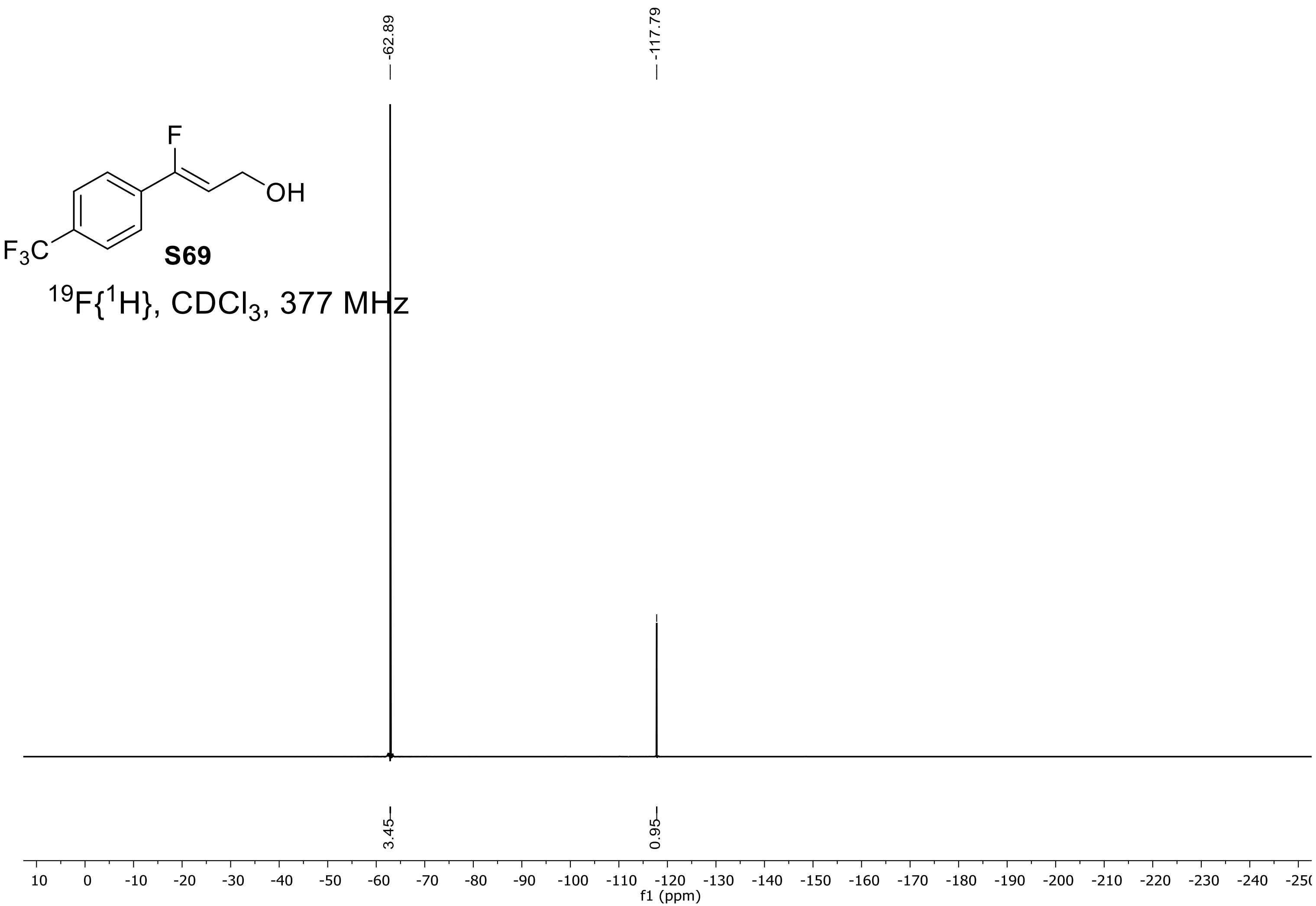

Fig. S170: $^{19}F\{^{1}H\}$ NMR spectrum for (*Z*)-3-Fluoro-3-[4-(trifluoromethyl)phenyl]prop-2-en-1-ol (**S69**).

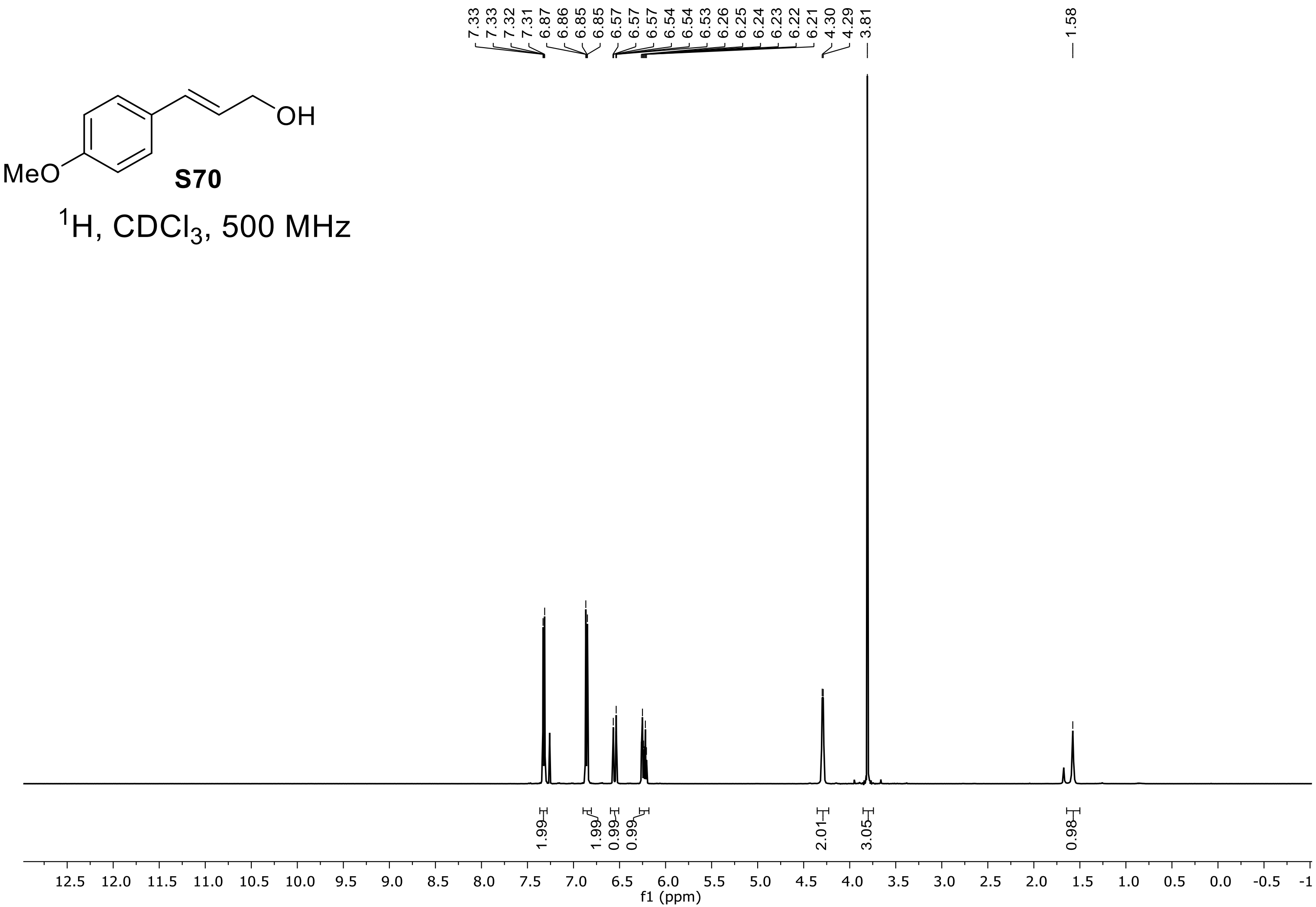

Fig. S171: ^{1}H NMR spectrum for (*E*)-3-(4-Methoxyphenyl)prop-2-en-1-ol (**S70**).

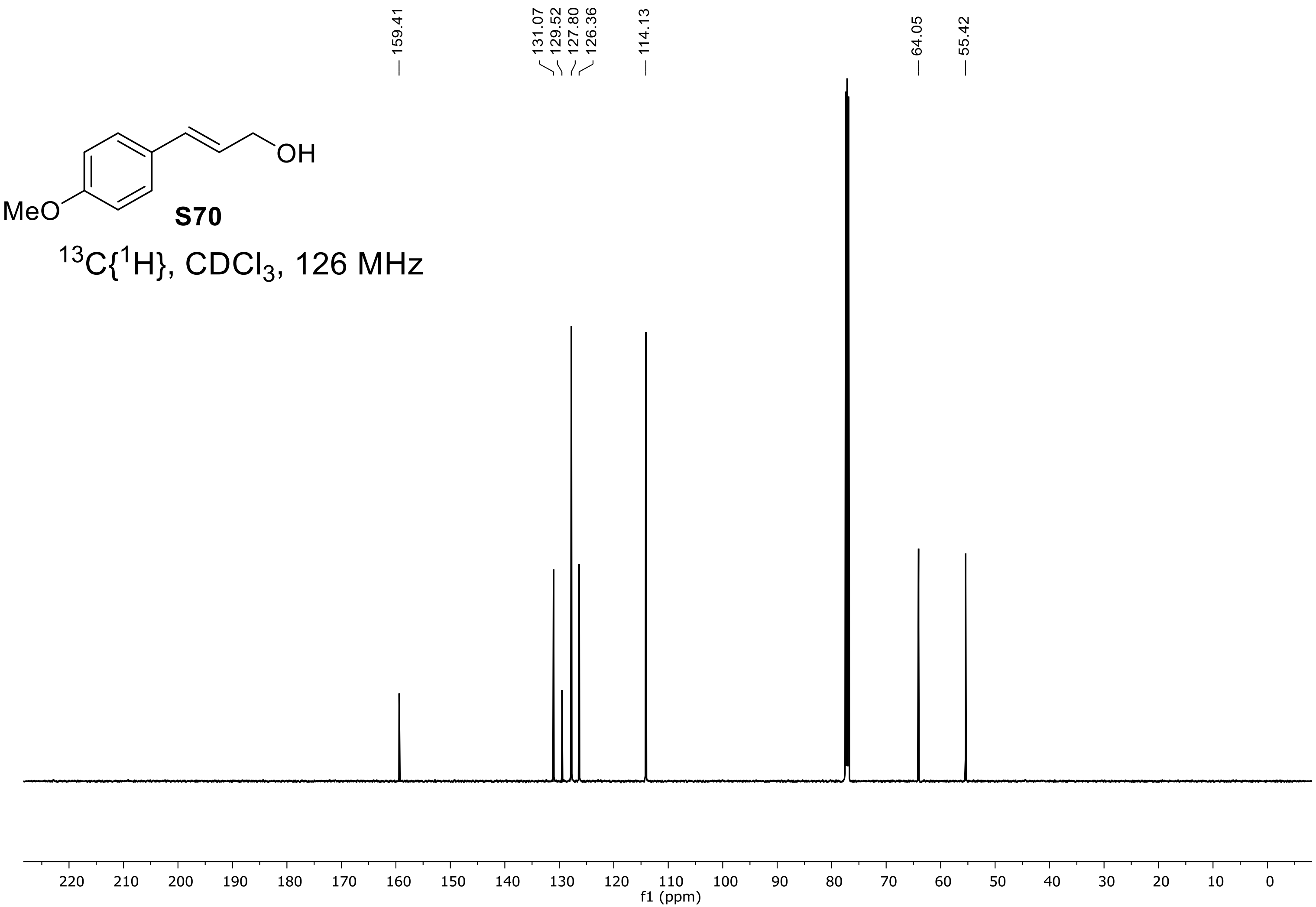

Fig. S172: $^{19}F\{^{1}H\}$ NMR spectrum for (E)-3-(4-Methoxyphenyl)prop-2-en-1-ol (**S70**).

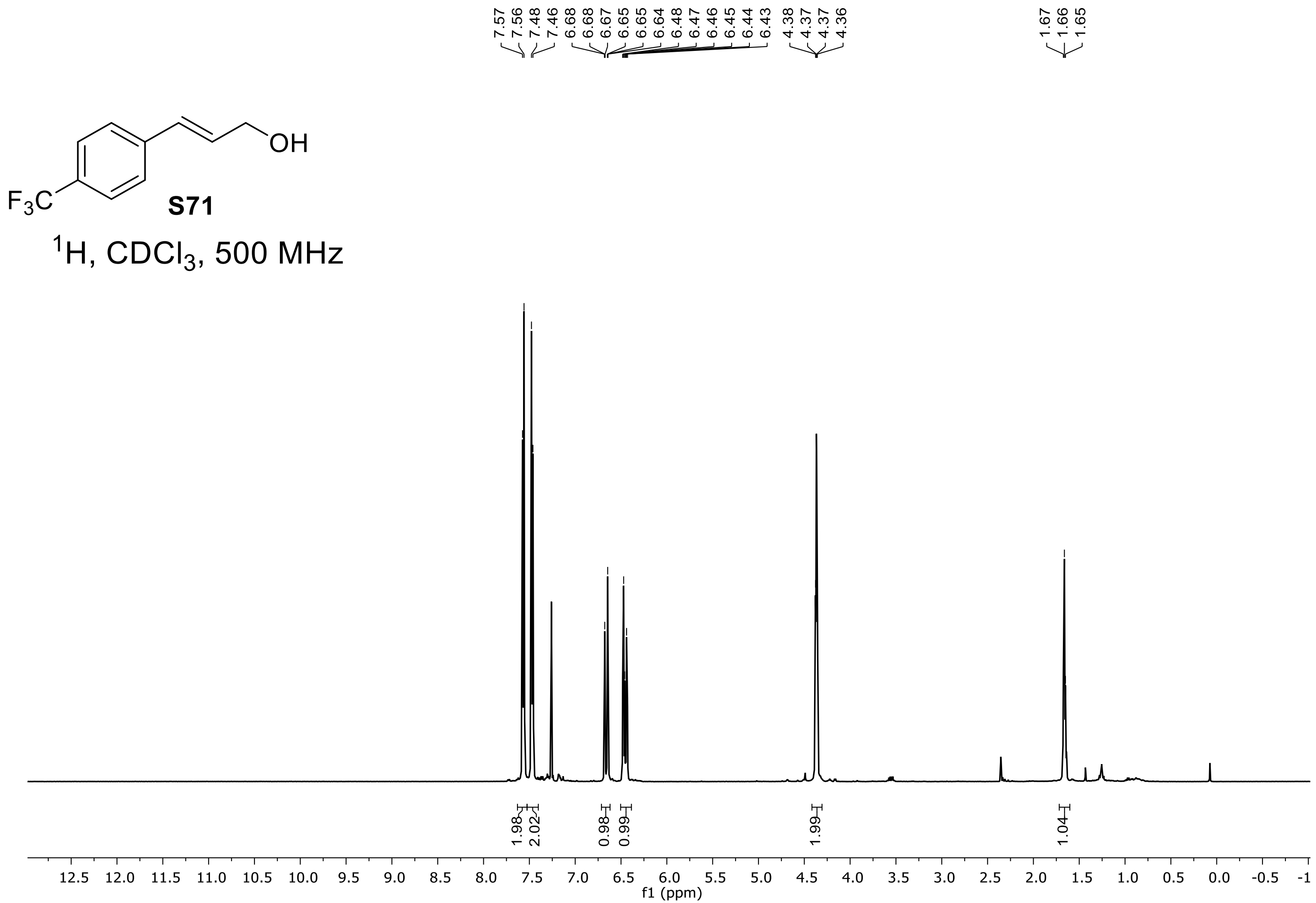

Fig. S173: ^{1}H NMR spectrum for (*E*)-3-[4-(Trifluoromethyl)phenyl]prop-2-en-1-ol (**S71**).

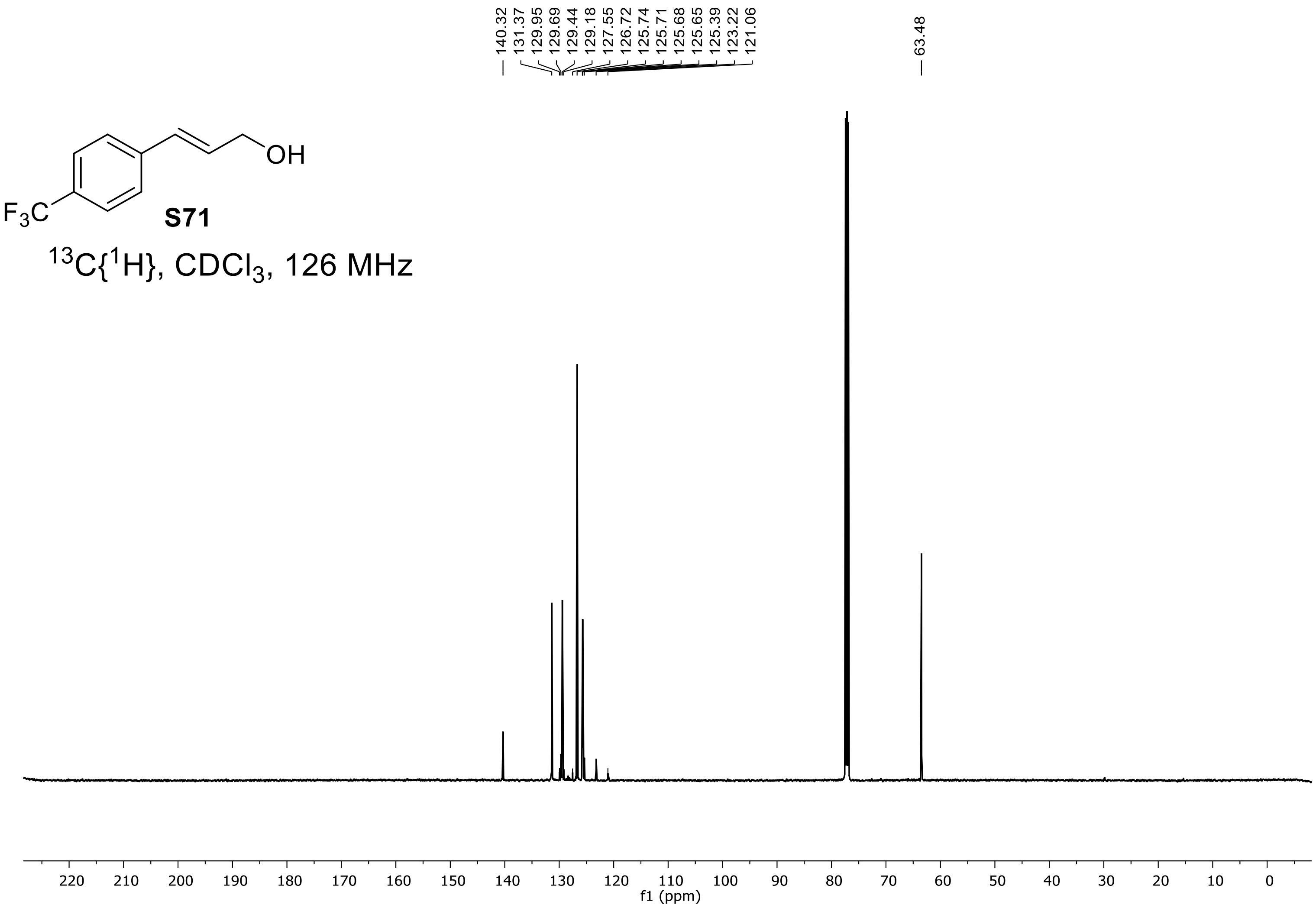

Fig. S174: $^{13}C\{^1H\}$ NMR spectrum for (*E*)-3-[4-(Trifluoromethyl)phenyl]prop-2-en-1-ol (**S71**).

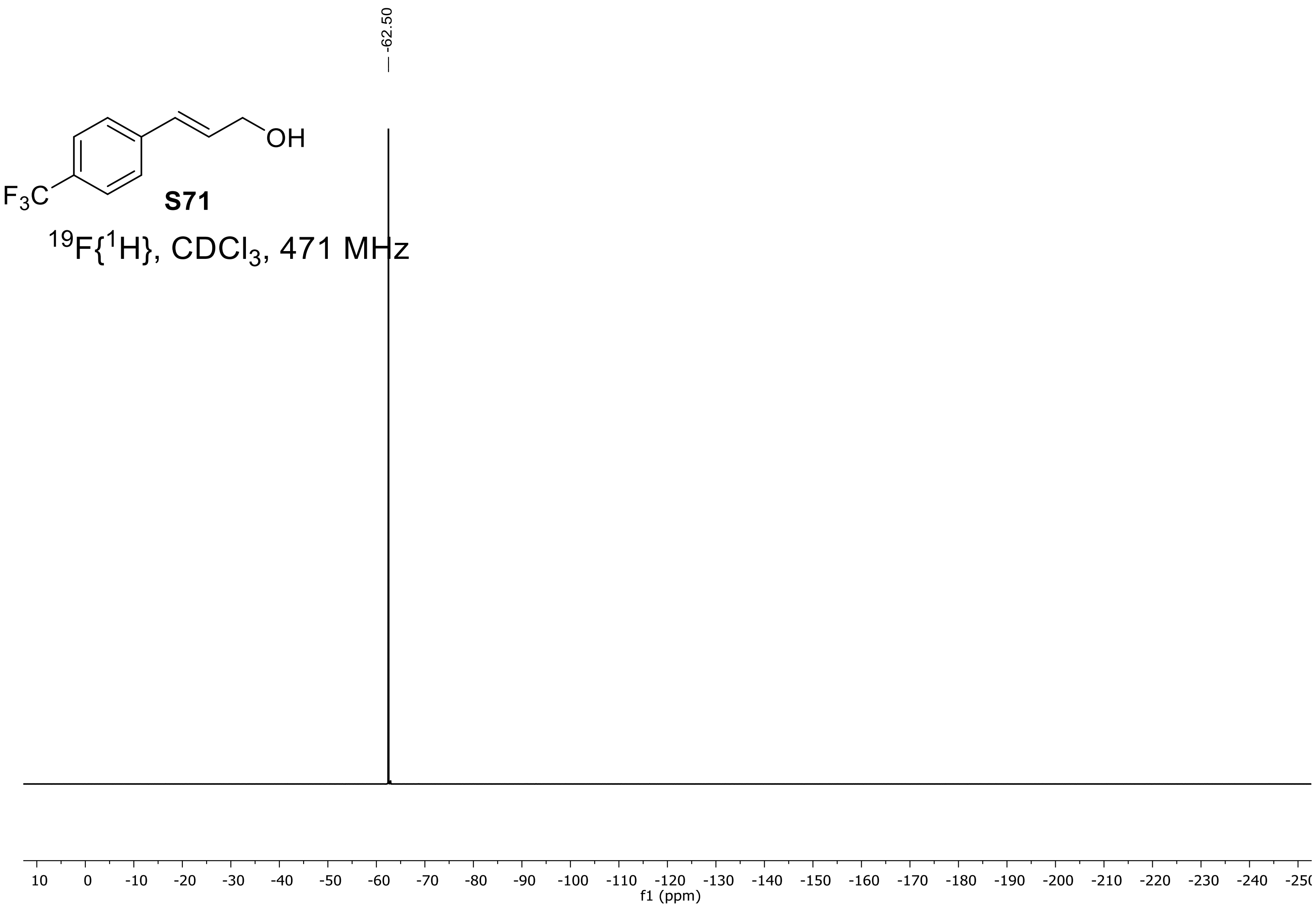

Fig. S175: $^{19}F\{^{1}H\}$ NMR spectrum for (*E*)-3-[4-(Trifluoromethyl)phenyl]prop-2-en-1-ol (**S71**).

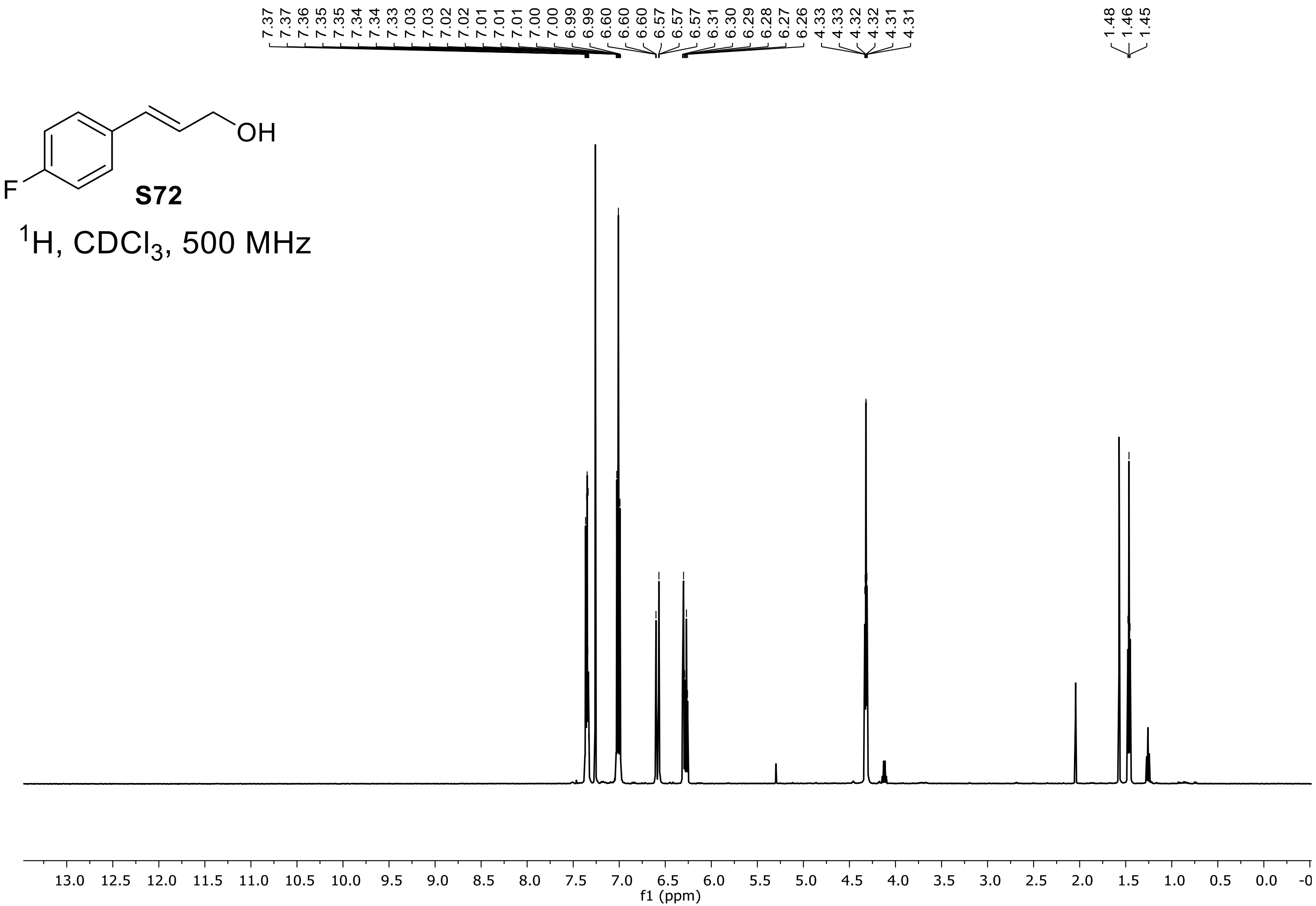

Fig. S176: ^{1}H NMR spectrum for (*E*)-3-(4-Fluorophenyl)prop-2-en-1-ol (**S72**).

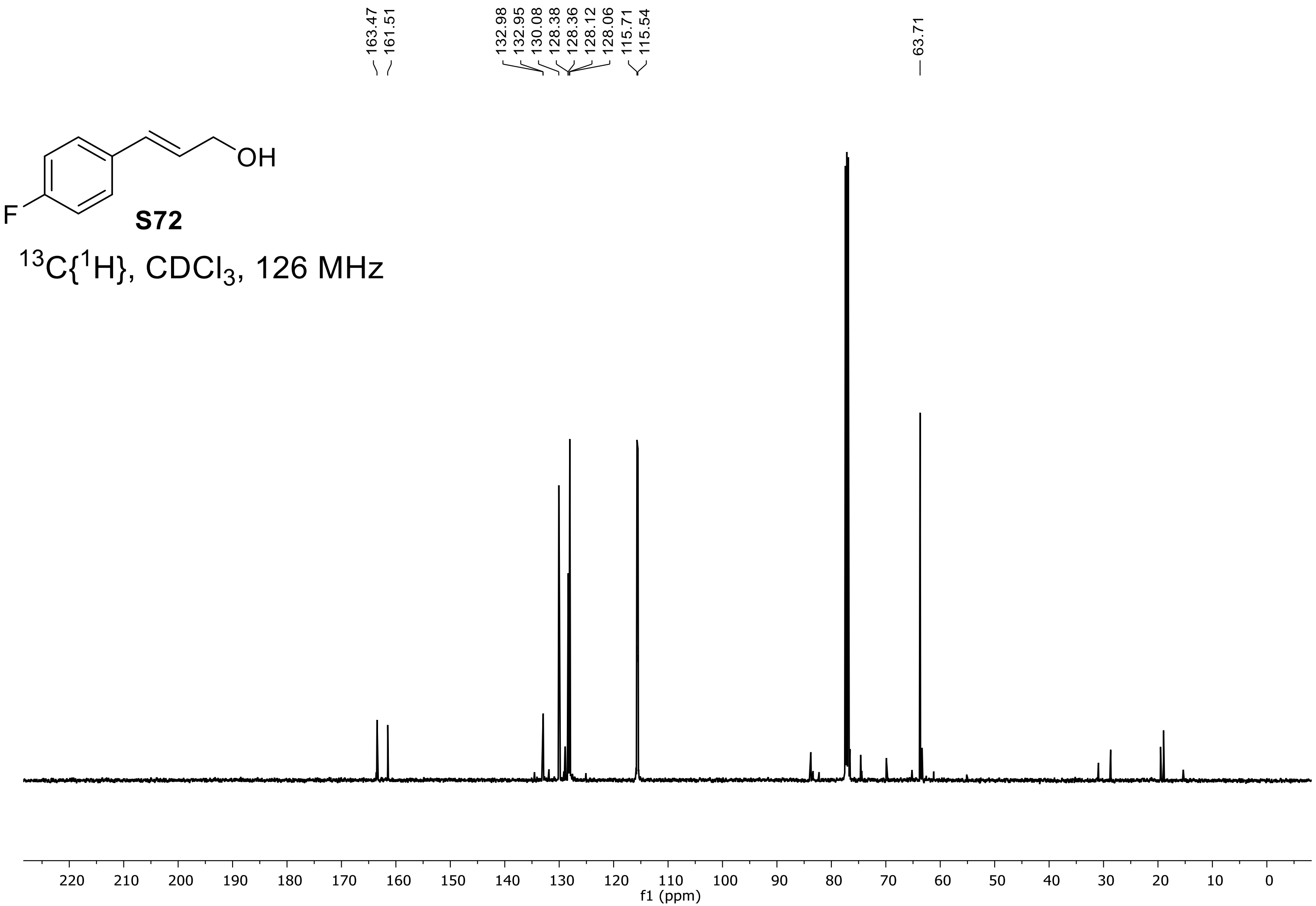

Fig. S177: $^{13}C\{^1H\}$ NMR spectrum for (*E*)-3-(4-Fluorophenyl)prop-2-en-1-ol (**S72**).

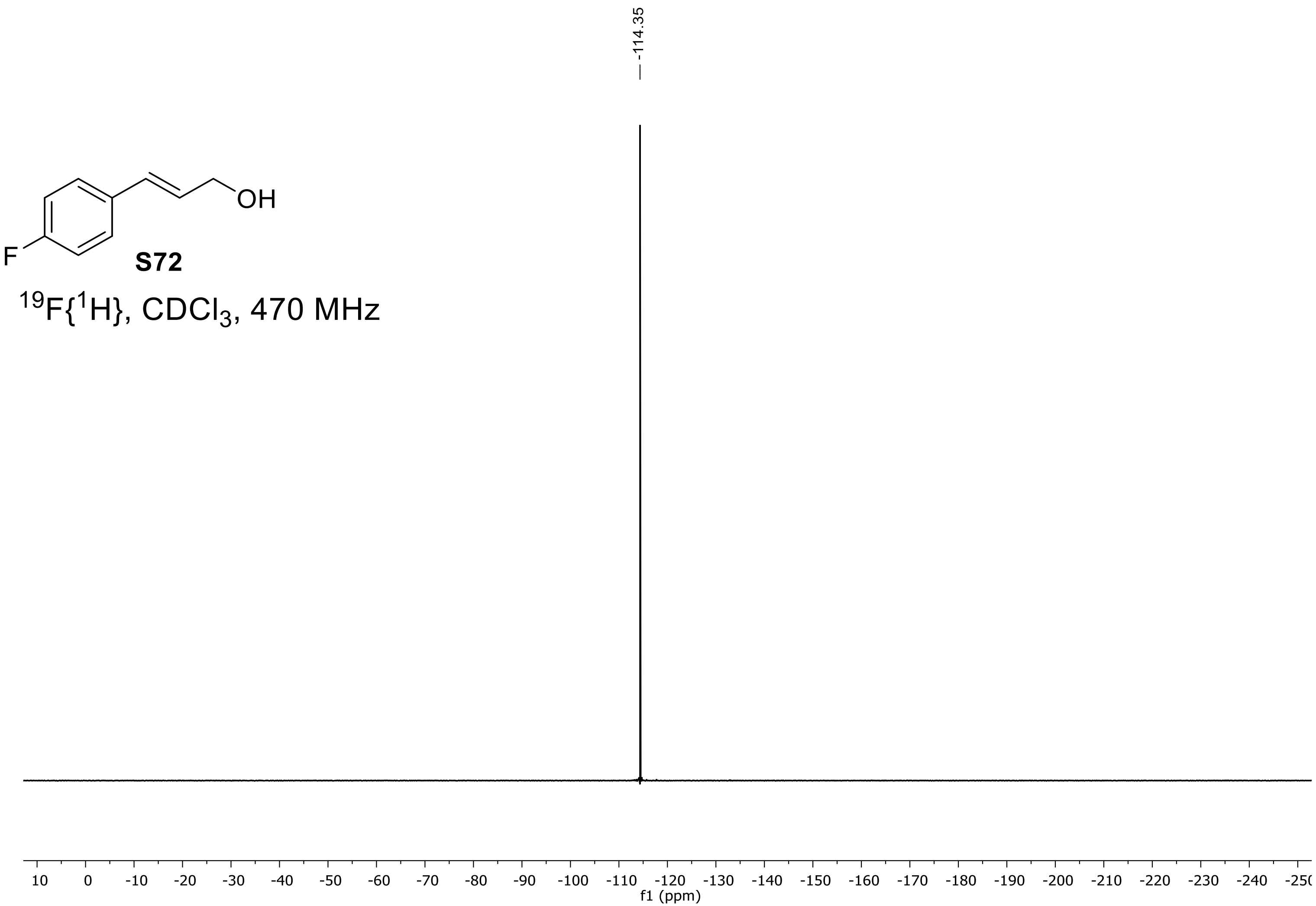

Fig. S180: $^{19}F\{^1H\}$ NMR spectrum for (*E*)-3-(4-Fluorophenyl)prop-2-en-1-ol (**S72**).

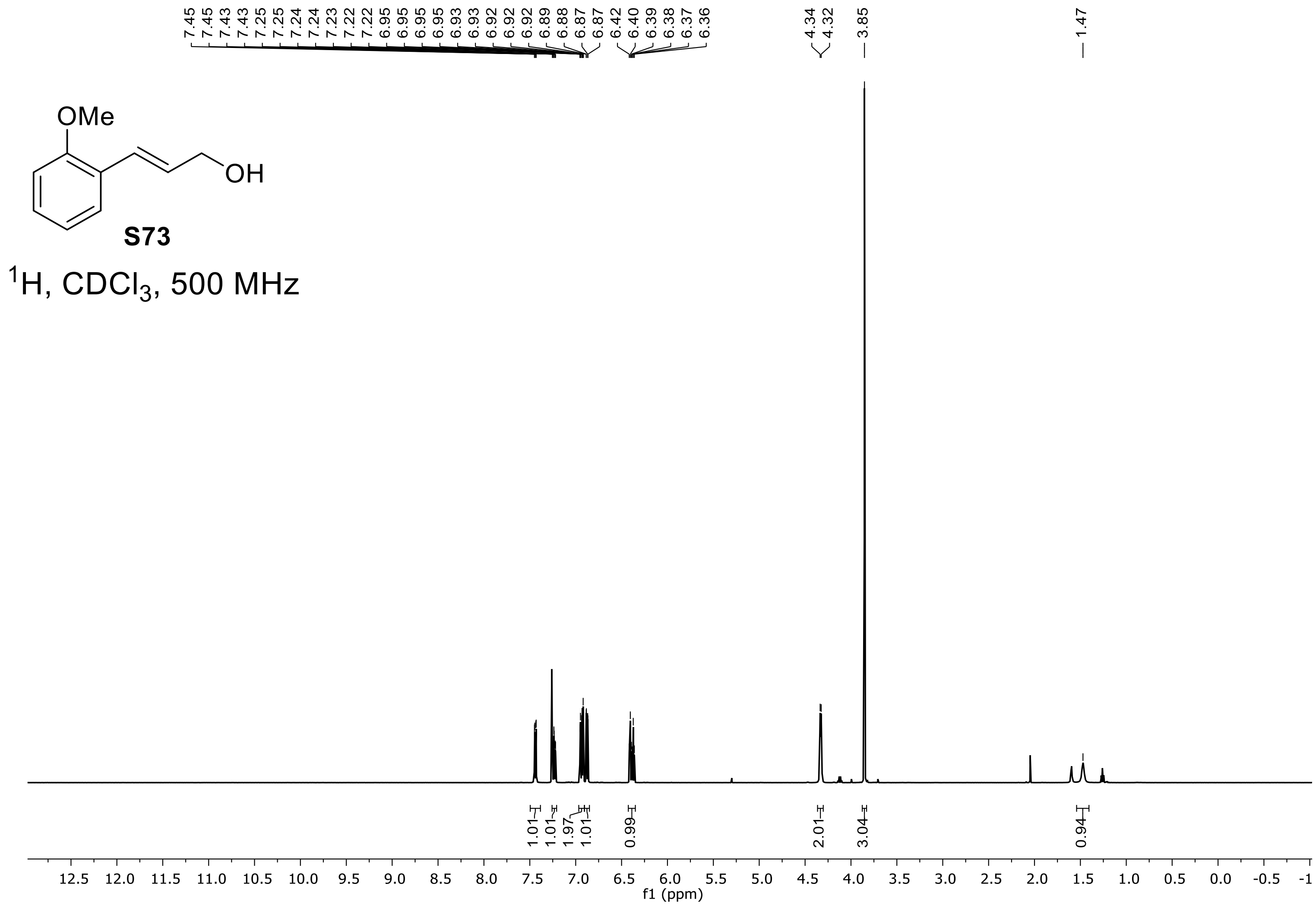

Fig. S179: ^{1}H NMR spectrum for (*E*)-3-(2-methoxyphenyl)prop-2-en-1-ol (**S73**).

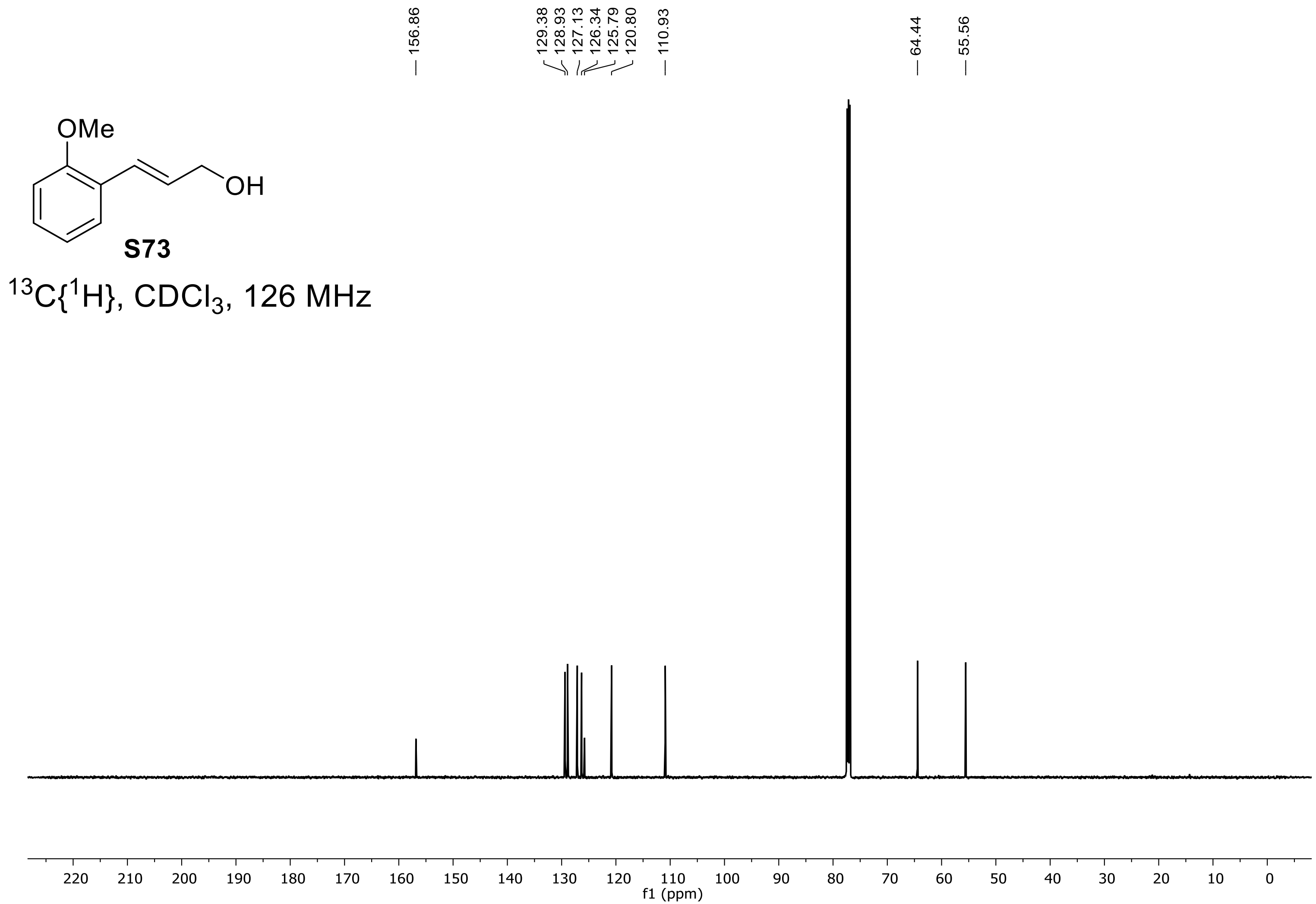

Fig. S180: $^{13}C\{^1H\}$ NMR spectrum for (*E*)-3-(2-methoxyphenyl)prop-2-en-1-ol (**S73**).

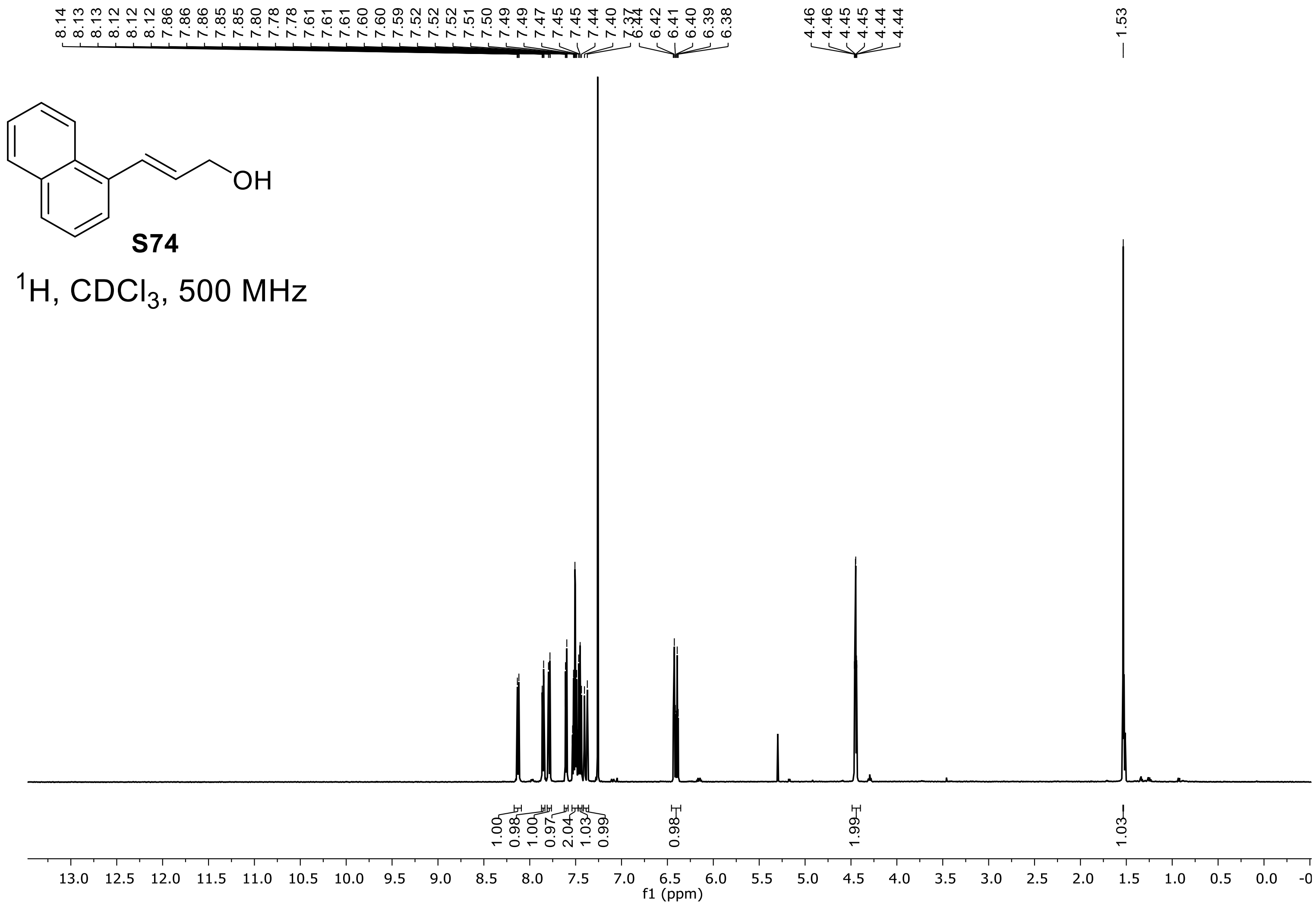

Fig. S181: ^{1}H NMR spectrum for (*E*)-3-(1-naphthyl)prop-2-en-1-ol (**S74**).

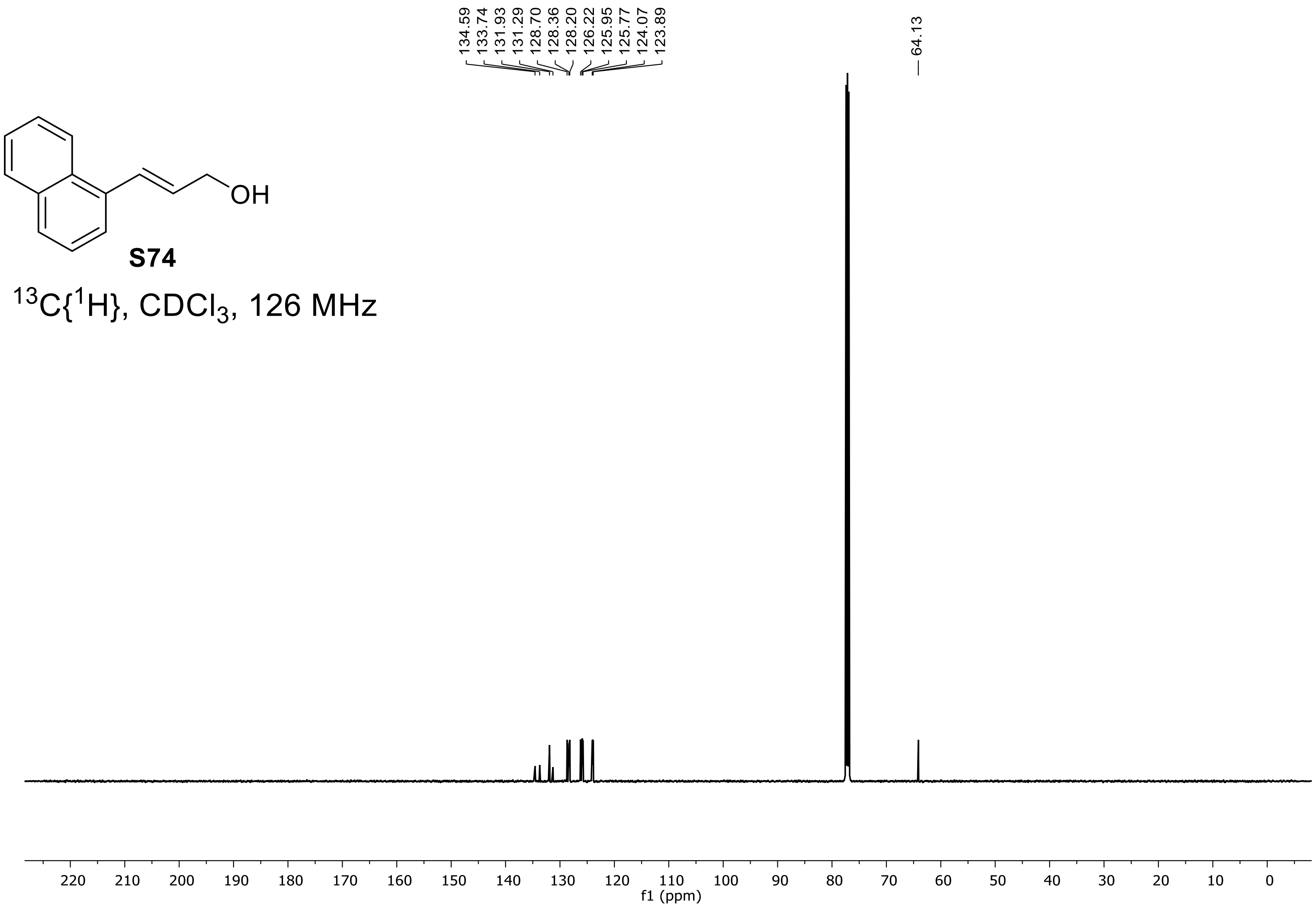

Fig. S182: $^{13}C\{^1H\}$ NMR spectrum for (*E*)-3-(1-naphthyl)prop-2-en-1-ol (**S74**).

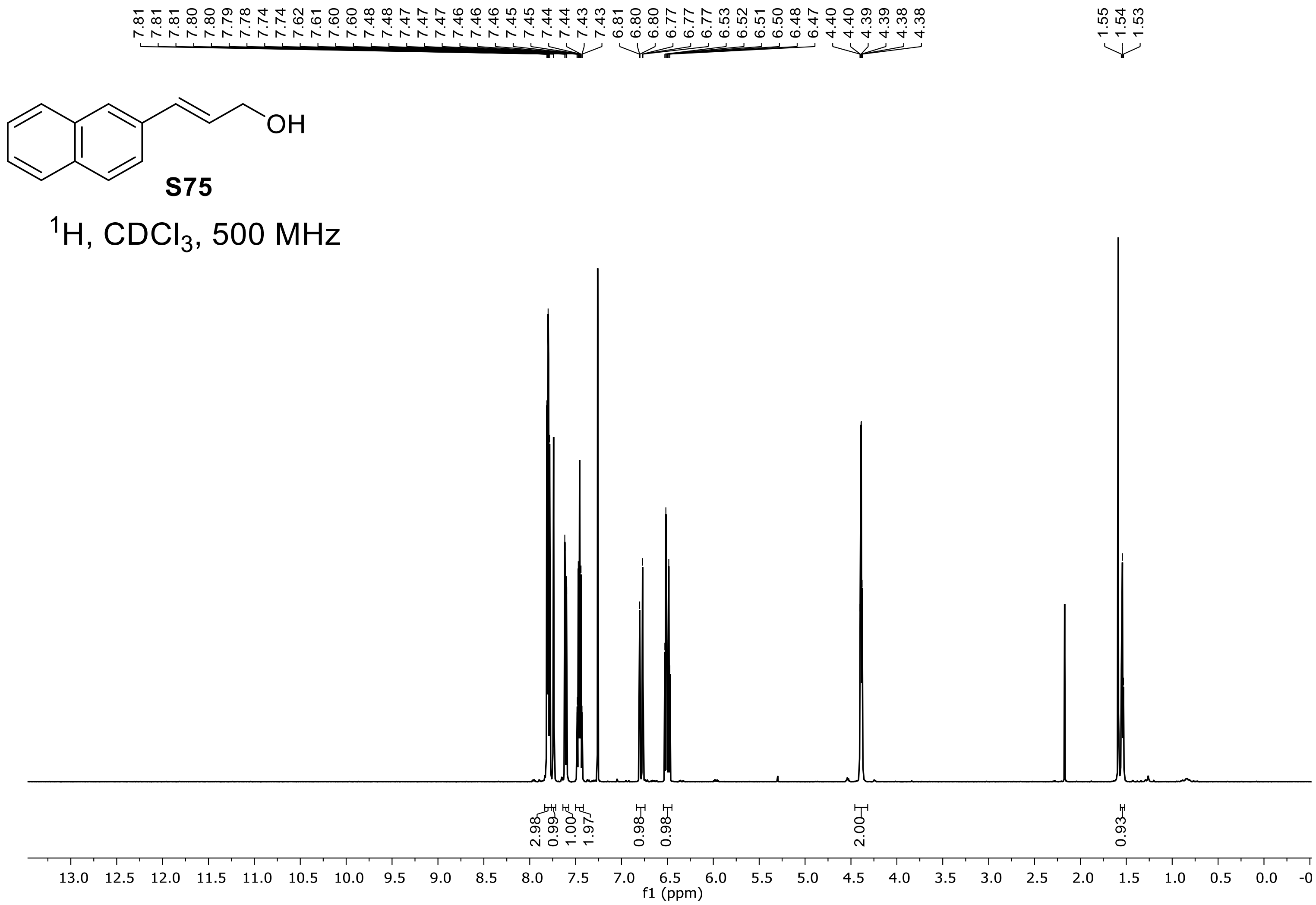

Fig. S183: ^{1}H NMR spectrum for (*E*)-3-(2-naphthyl)prop-2-en-1-ol (**S75**).

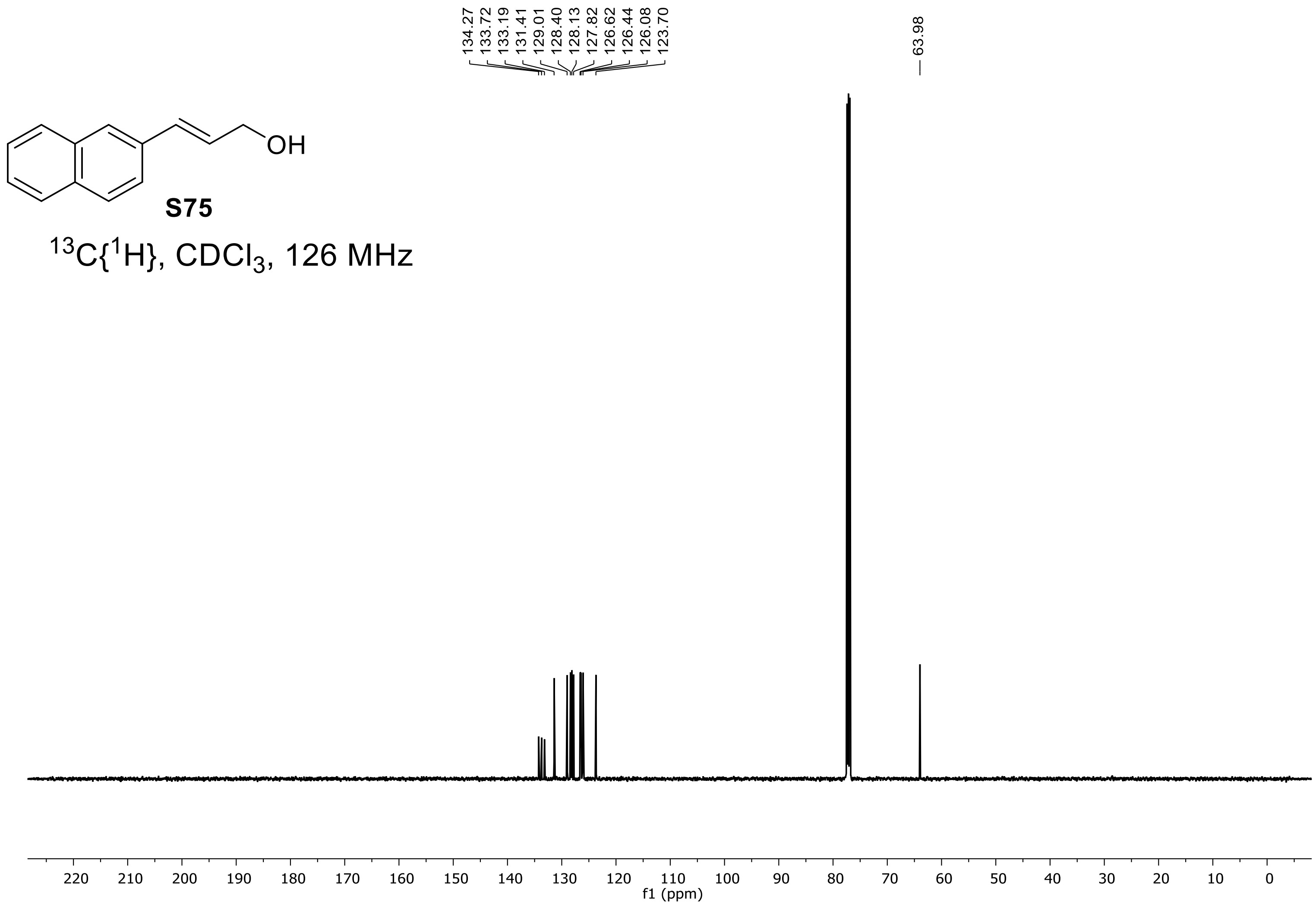

Fig. S184: ^{1}H NMR spectrum for (*E*)-3-(2-naphthyl)prop-2-en-1-ol (**S75**).

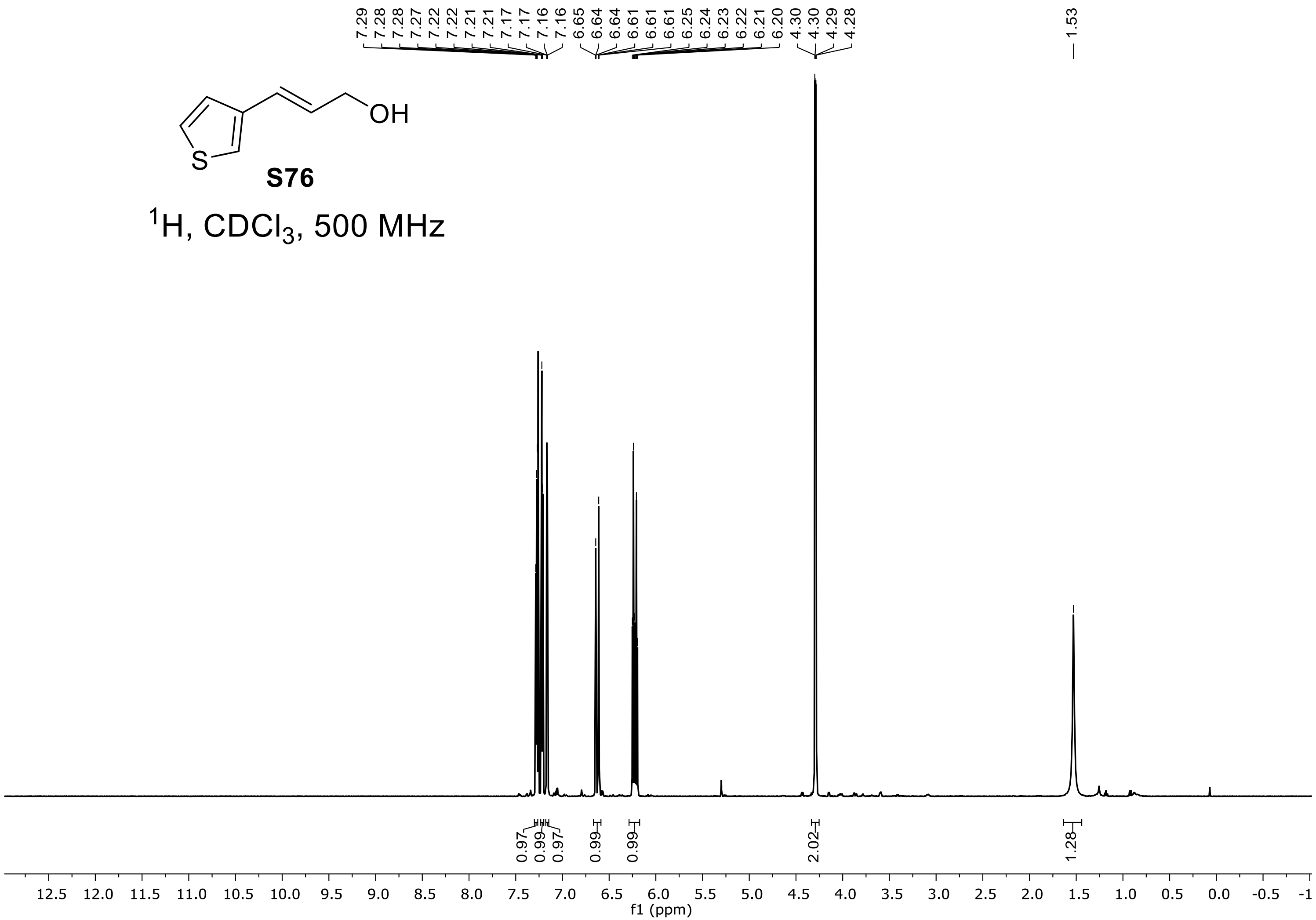

Fig. S185: ^{1}H NMR spectrum for (*E*)-3-(thiophen-3-yl)prop-2-en-1-ol (**S76**).

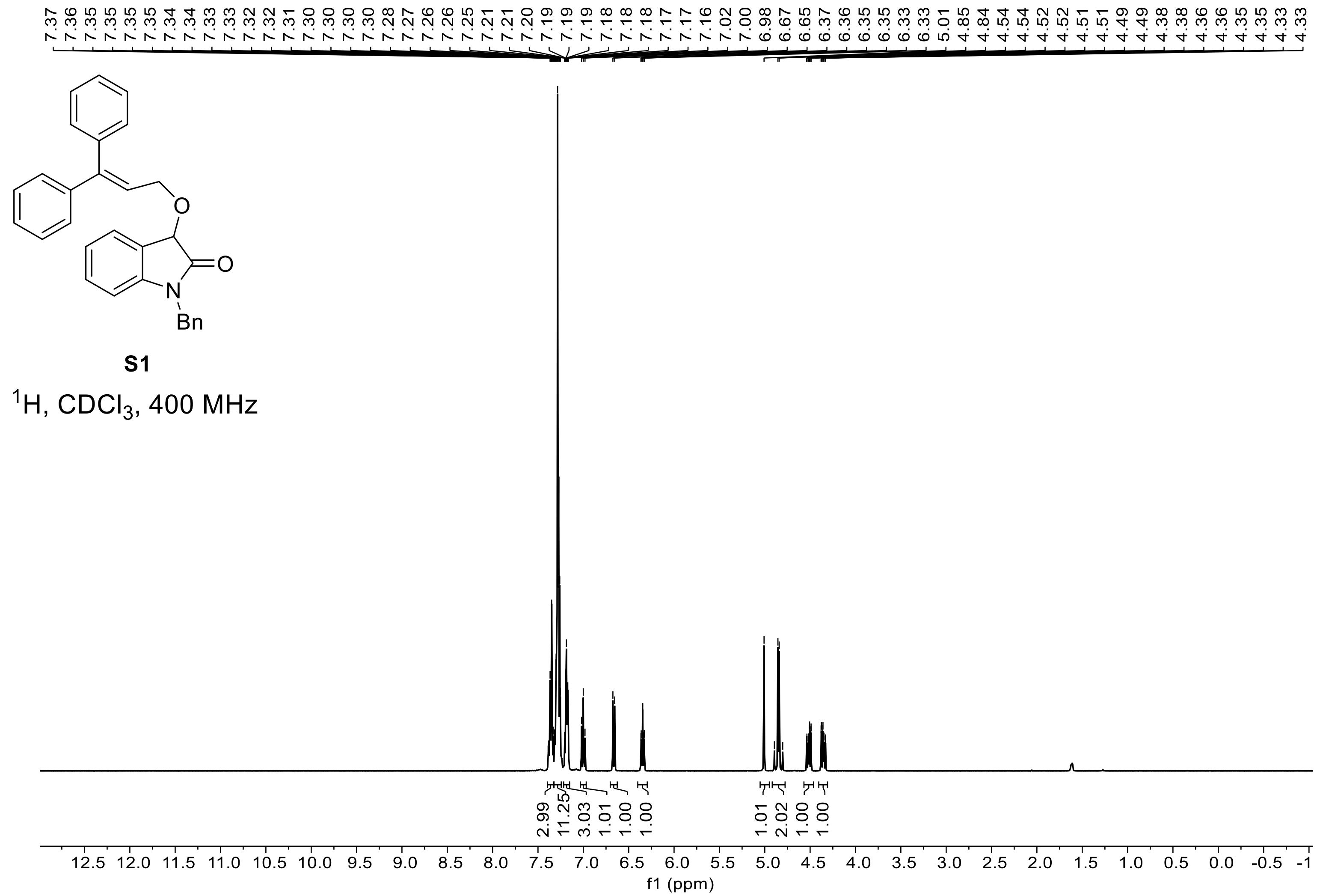

Fig. S186: ^{1}H NMR spectrum for 1-Benzyl-3-[(3,3-diphenylallyl)oxy]indolin-2-one (**S1**).

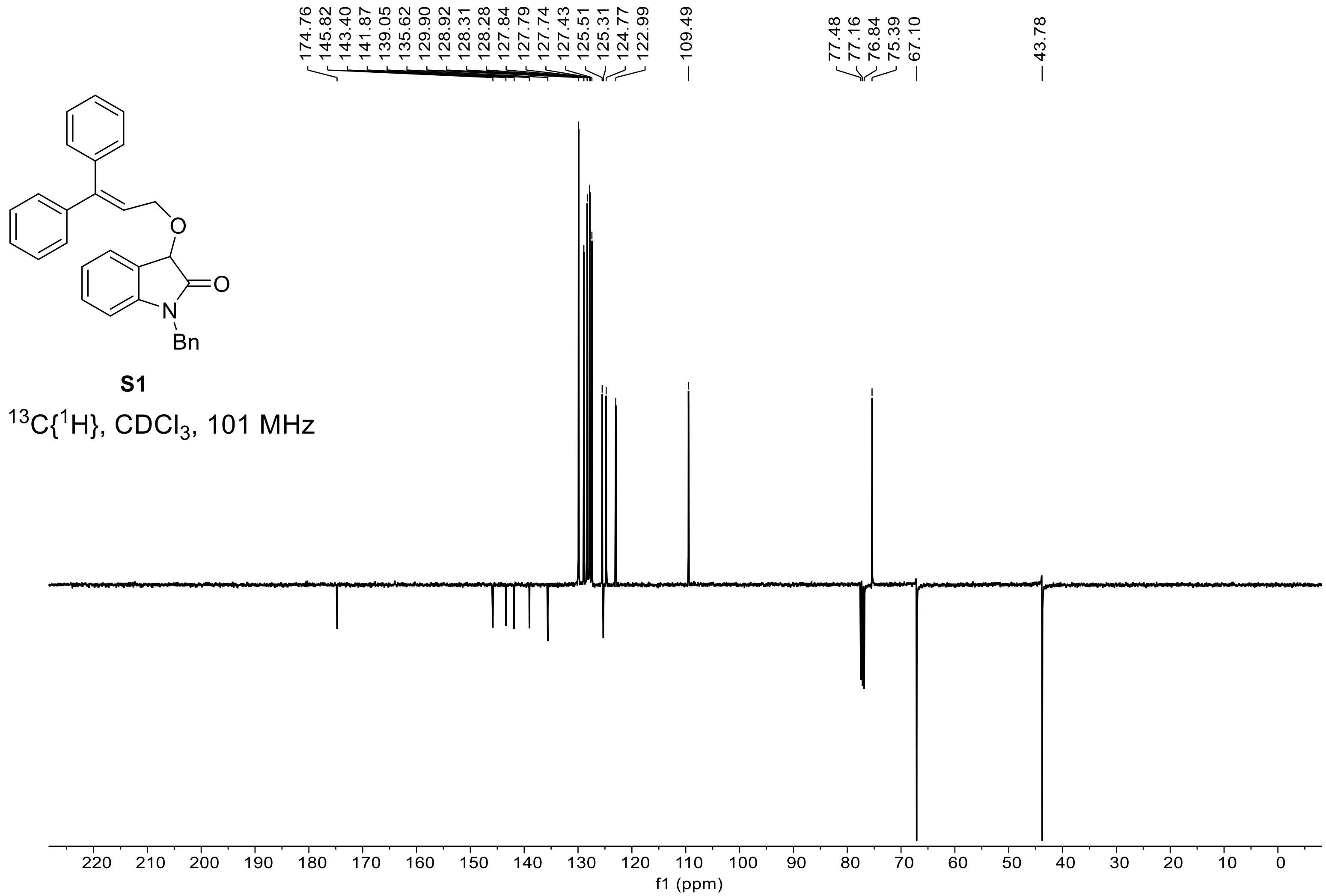

Fig. S187: $^{13}C\{^1H\}$ NMR spectrum for 1-Benzyl-3-[(3,3-diphenylallyl)oxy]indolin-2-one (**S1**).

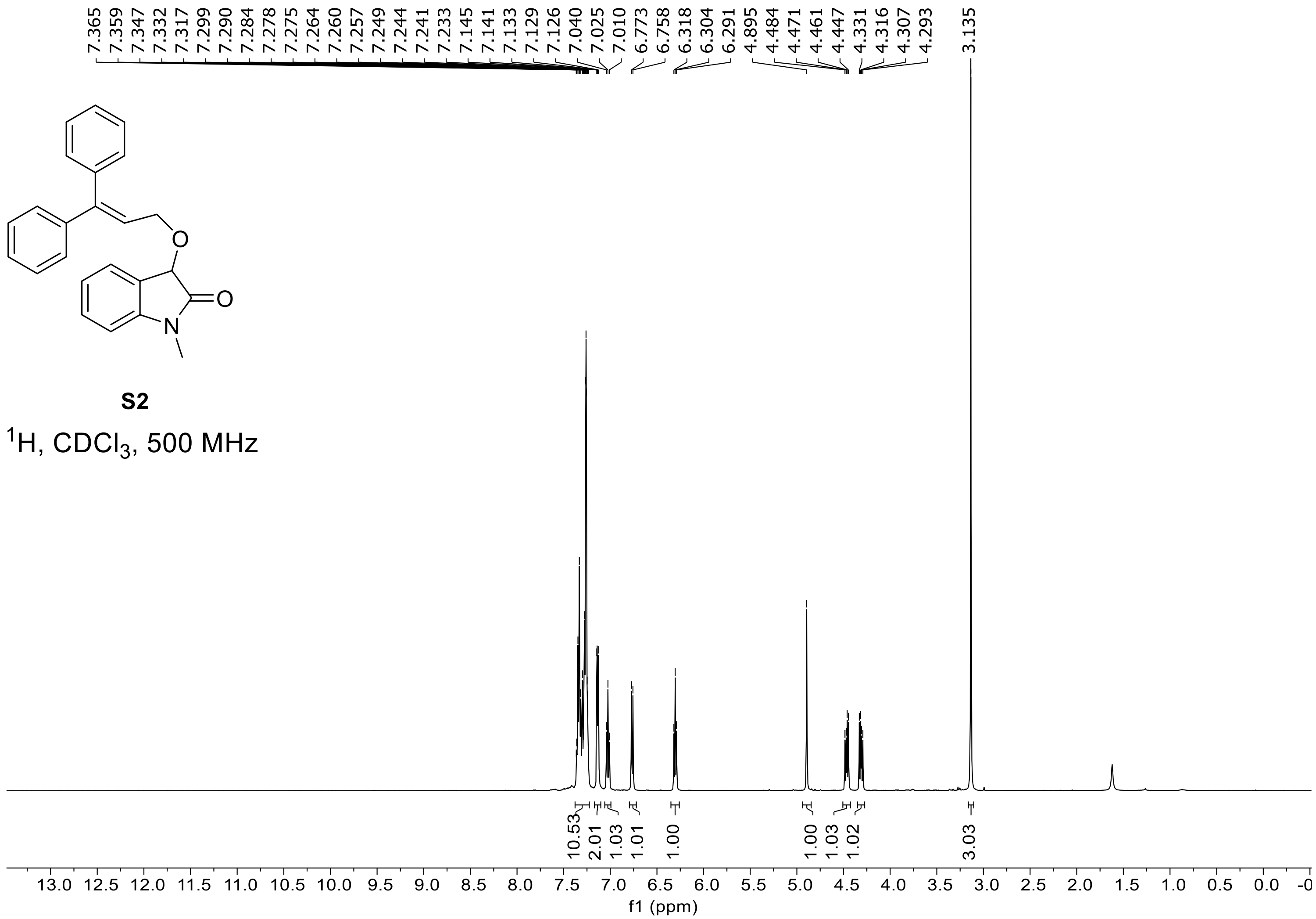

Fig. S188: ^{1}H NMR spectrum for 3-[(3,3-Diphenylallyl)oxy]-1-methylindolin-2-one (**S2**).

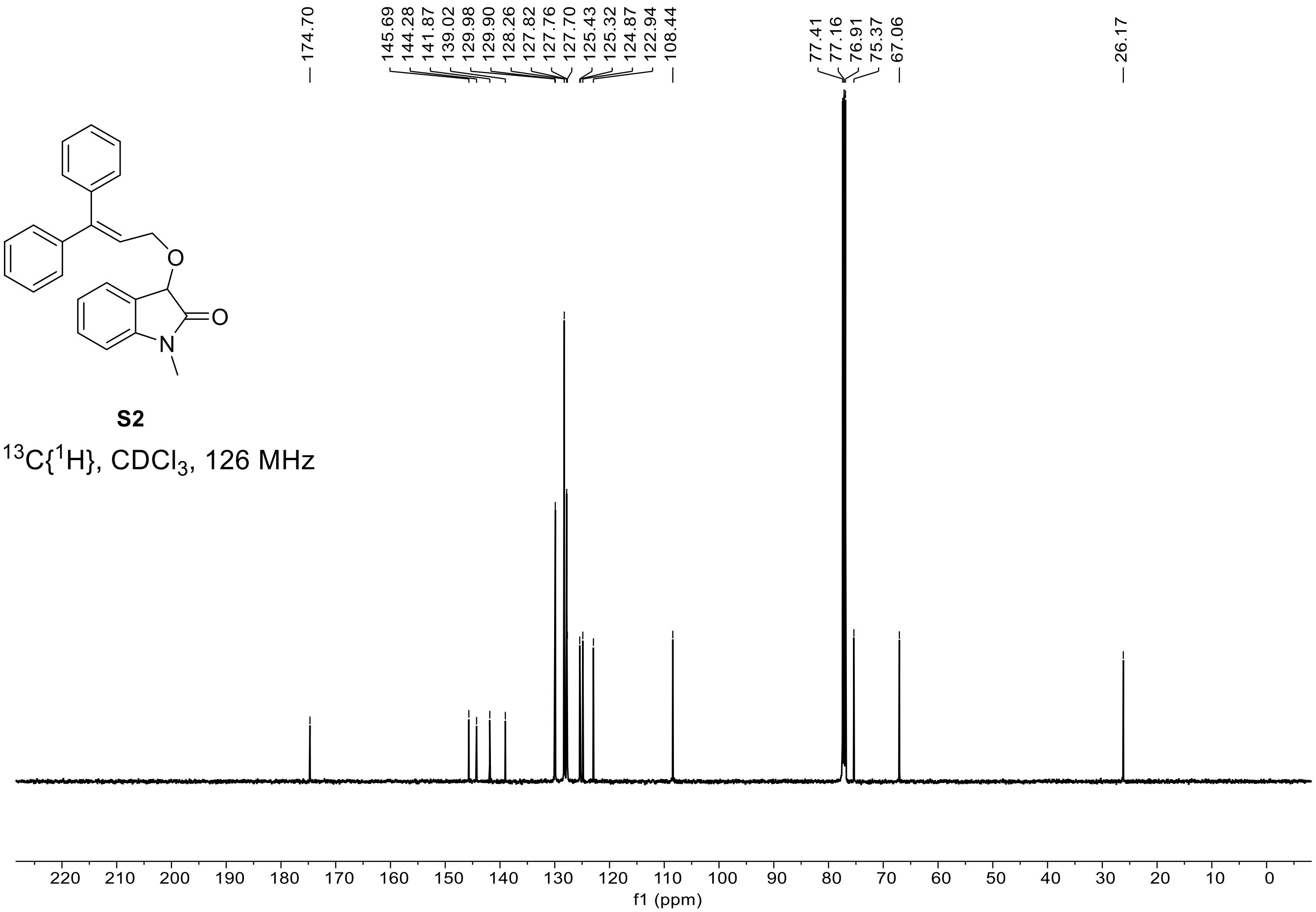

Fig. S189: $^{13}C\{^1H\}$ NMR spectrum for 3-[(3,3-Diphenylallyl)oxy]-1-methylindolin-2-one (**S2**).

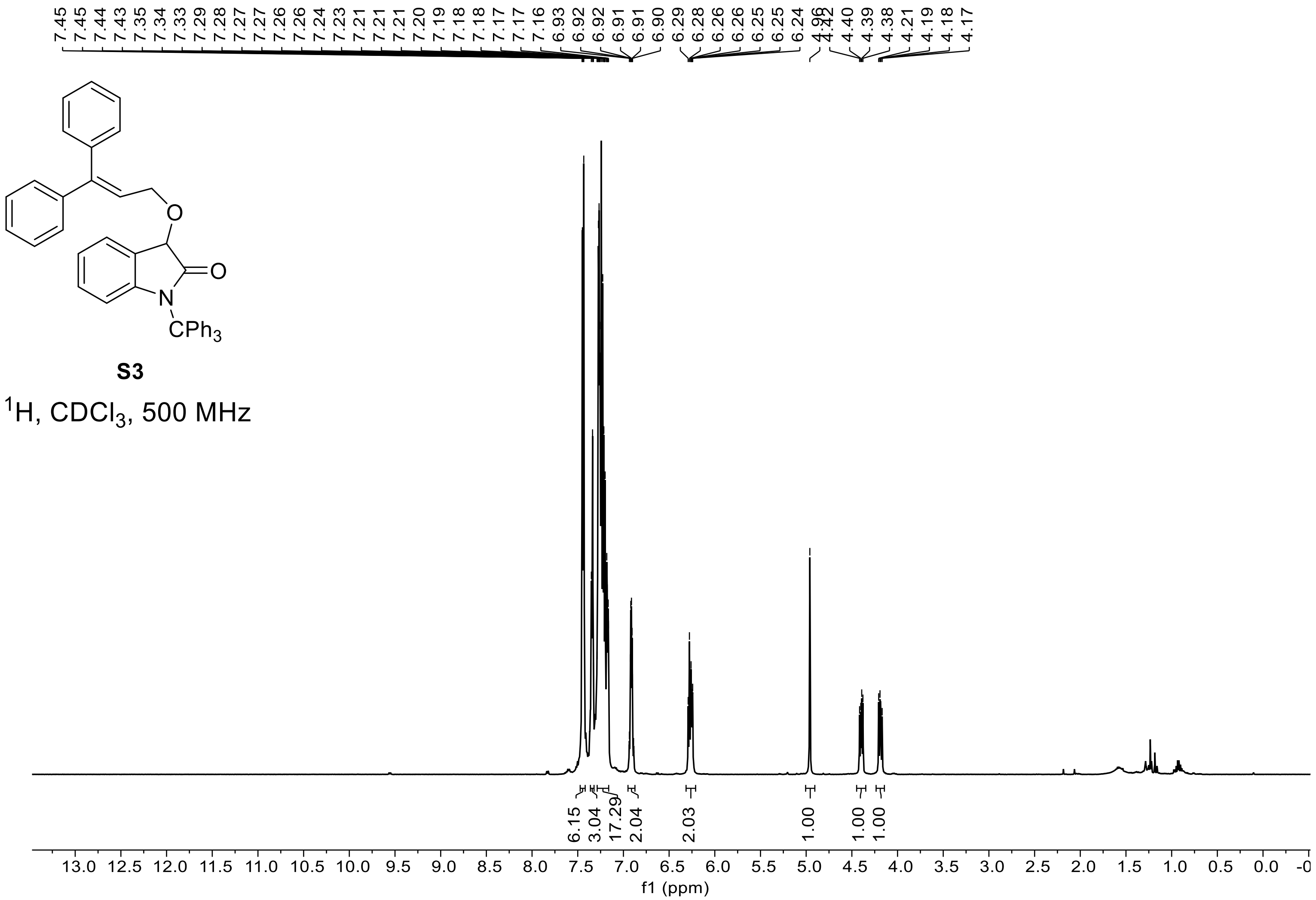

Fig. S190: ^{1}H NMR spectrum for 3-[(3,3-Diphenylallyl)oxy]-1-tritylindolin-2-one (**S3**).

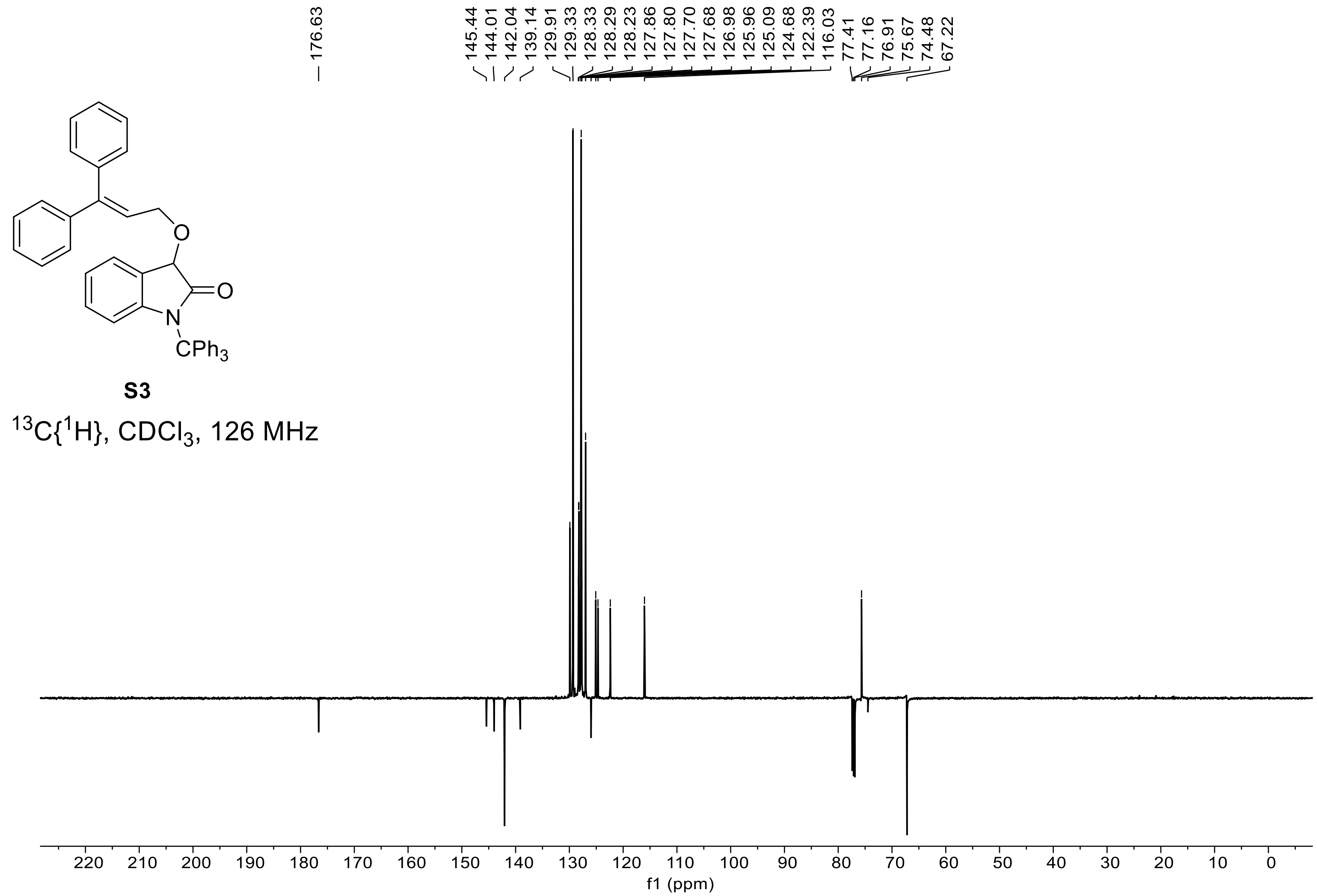

Fig. S191: $^{13}C\{^{1}H\}$ NMR spectrum for 3-[(3,3-Diphenylallyl)oxy]-1-tritylindolin-2-one (**S3**).

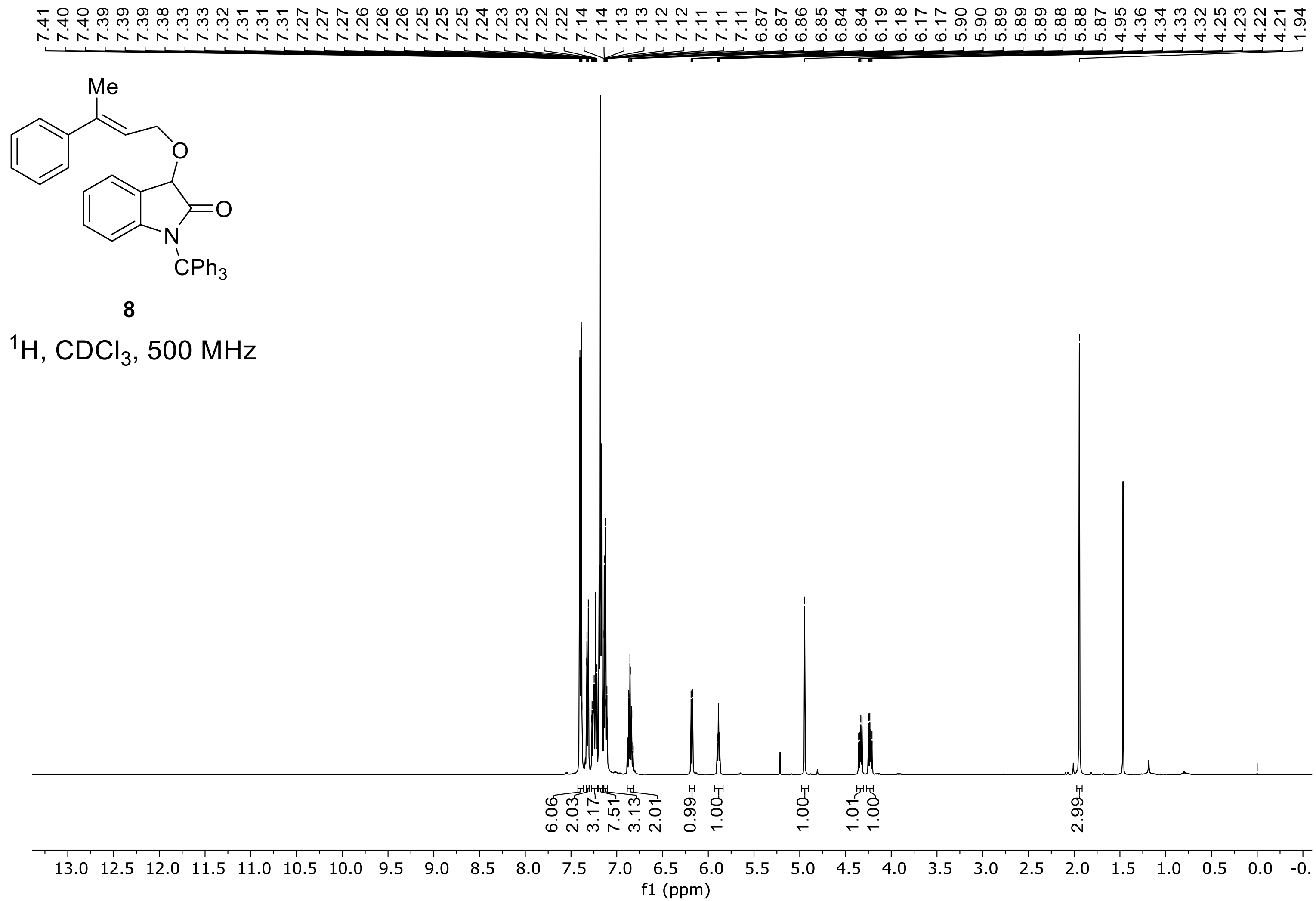

Fig. S192: ^{1}H NMR spectrum for (*E*)-3-[(3-Phenylbut-2-en-1-yl)oxy]-1-tritylindolin-2-one (**8**).

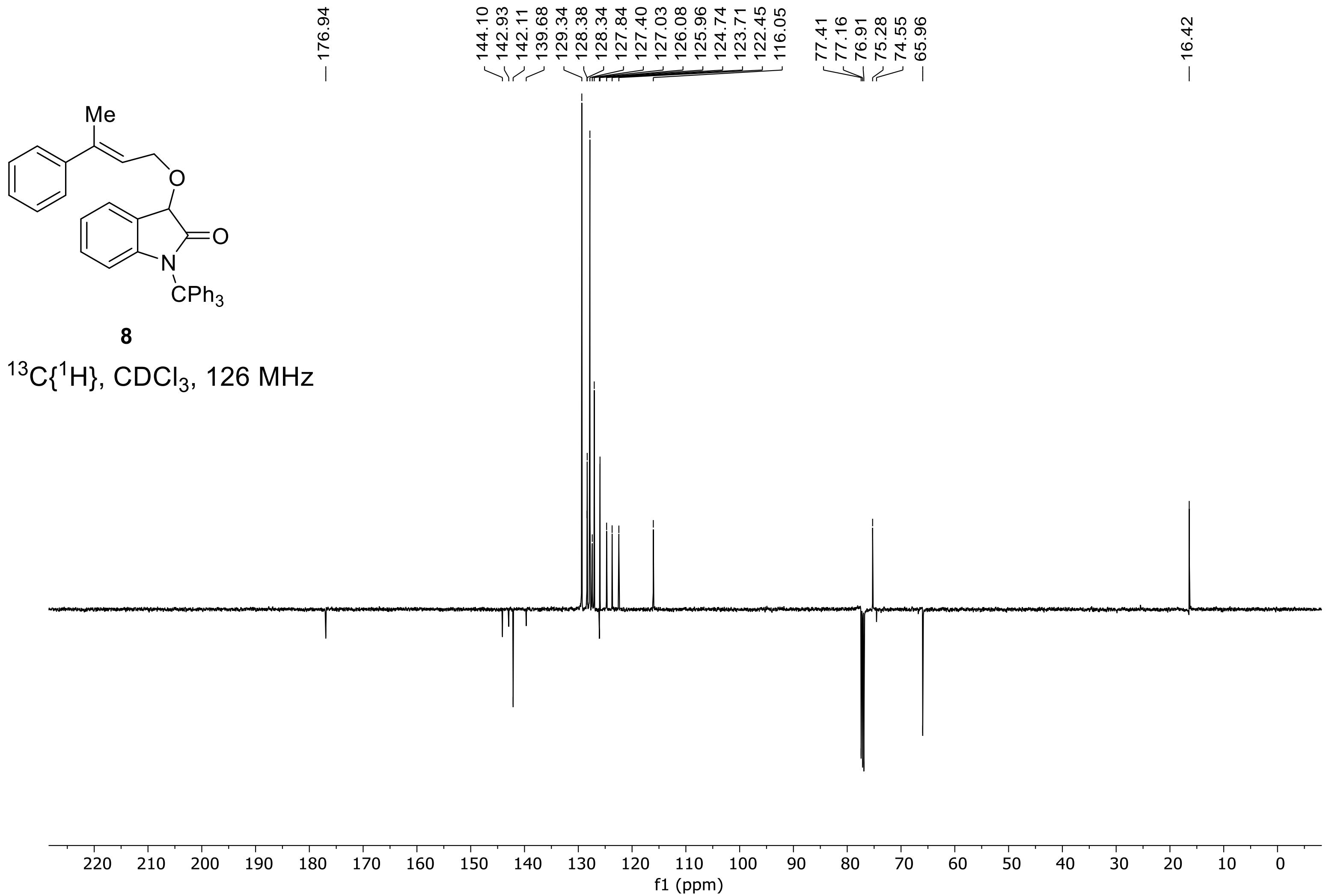

Fig. S193: $^{13}C\{^{1}H\}$ NMR spectrum for (E)-3-[(3-Phenylbut-2-en-1-yl)oxy]-1-tritylindolin-2-one (**8**).

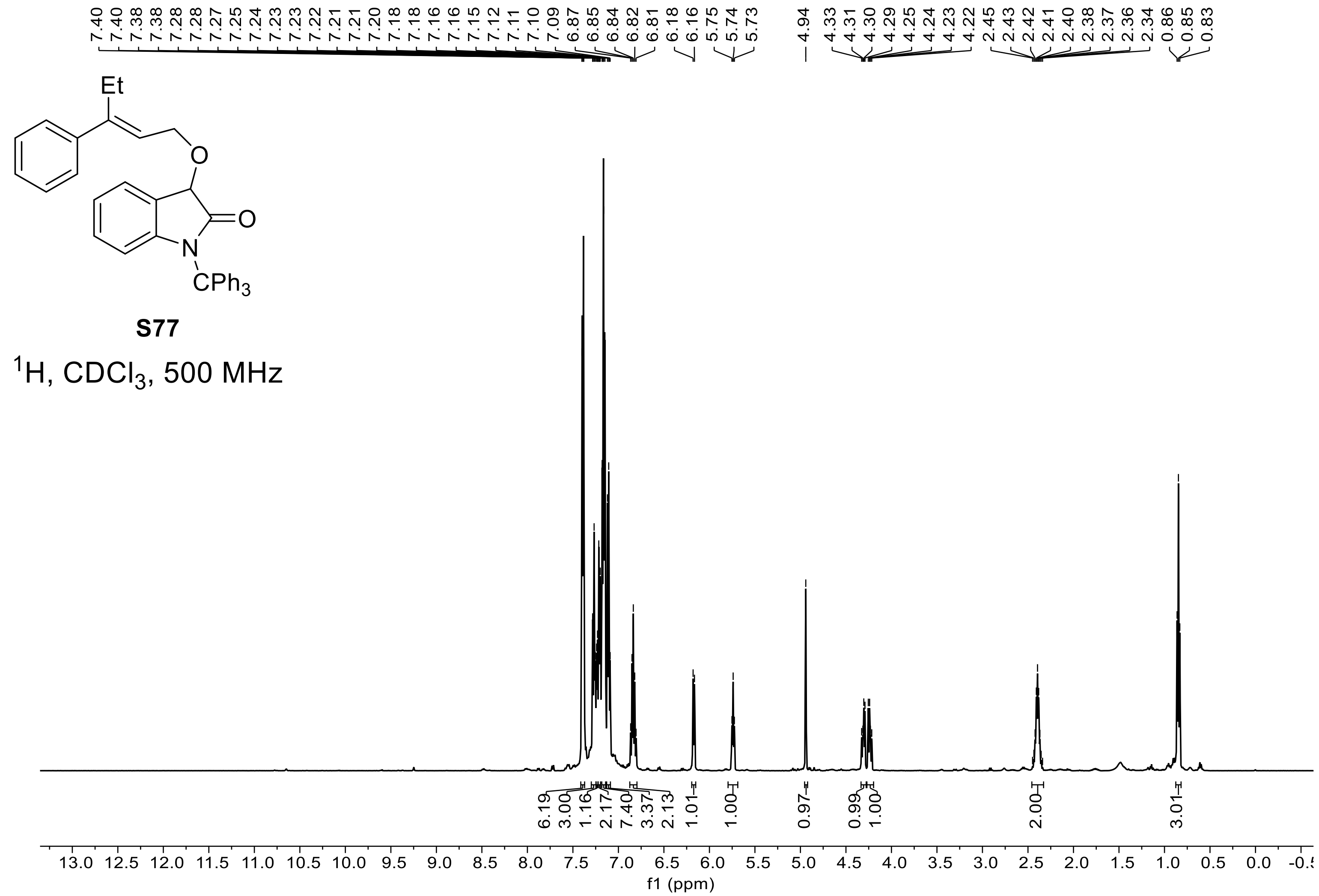

Fig. S194: ^{1}H NMR spectrum for (*E*)-3-[(3-Phenylpent-2-en-1-yl)oxy]-1-tritylindolin-2-one (**S77**).

S77

$^{13}C\{^1H\}$, $CDCl_3$, 126 MHz

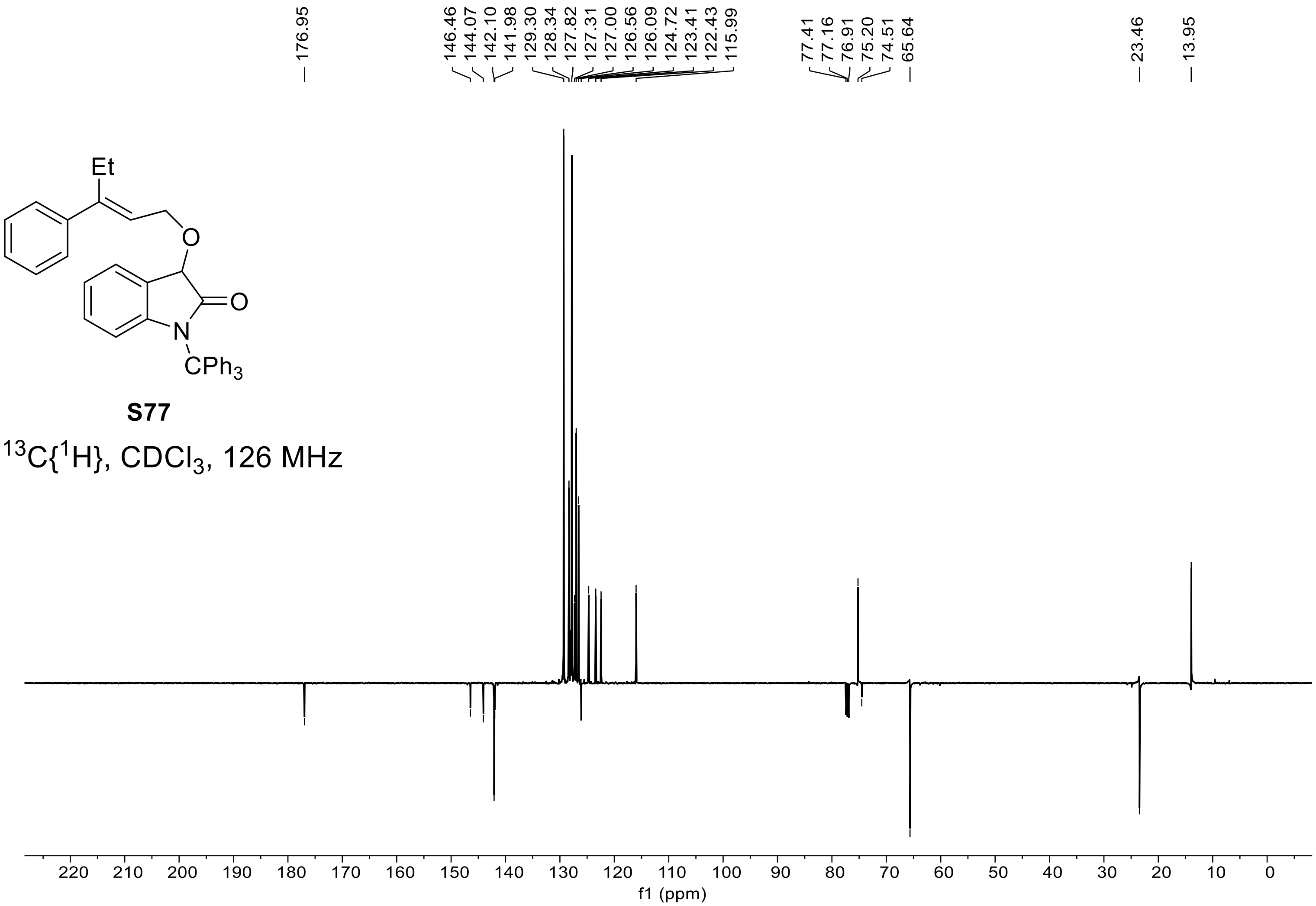

Fig. S195: $^{13}C\{^1H\}$ NMR spectrum for (*E*)-3-[(3-Phenylpent-2-en-1-yl)oxy]-1-tritylindolin-2-one (**S77**).

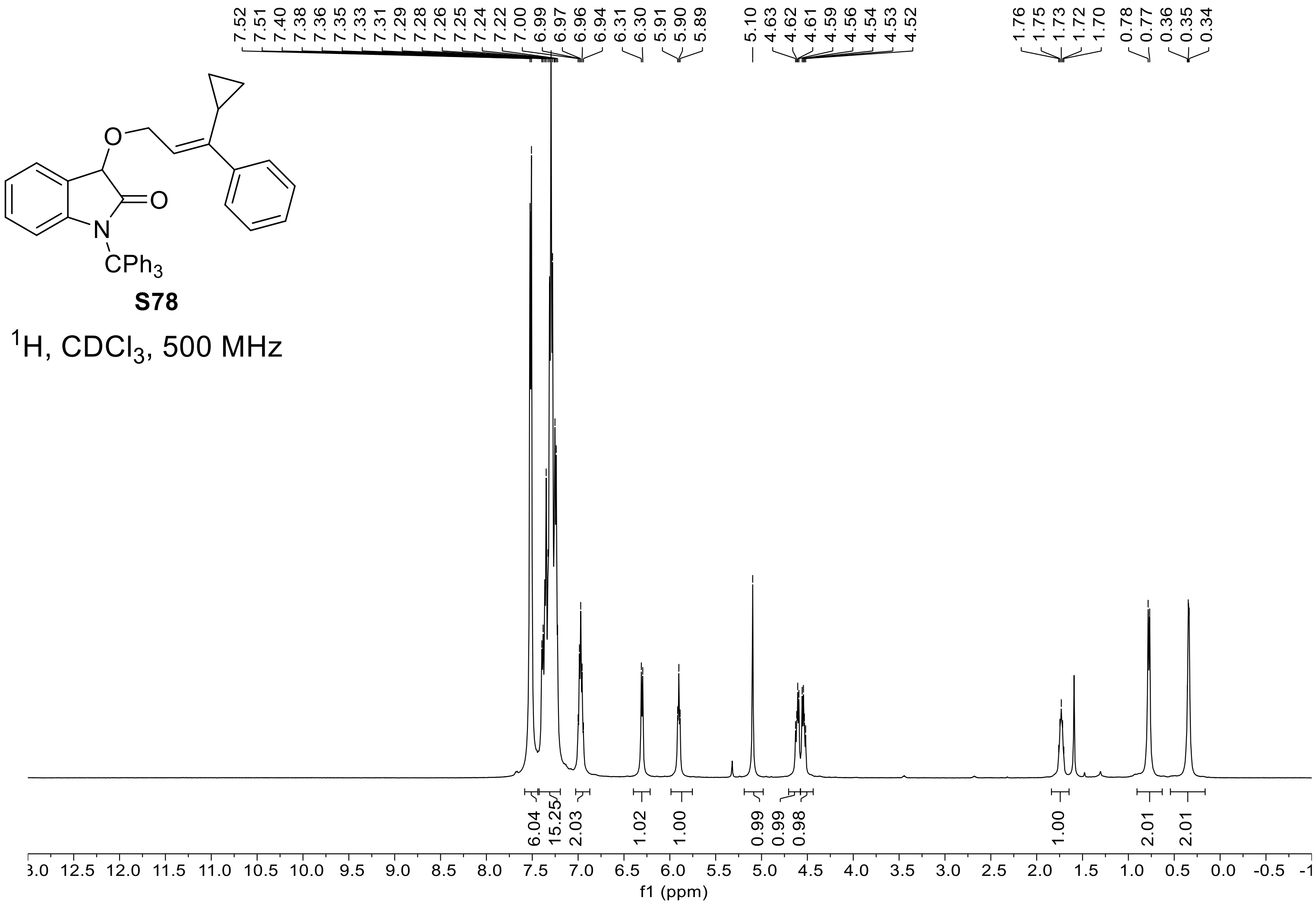

Fig. S196: ^{1}H NMR spectrum for (*E*)-3-[(3-Cyclopropyl-3-phenylallyl)oxy]-1-tritylindolin-2-one (**S78**).

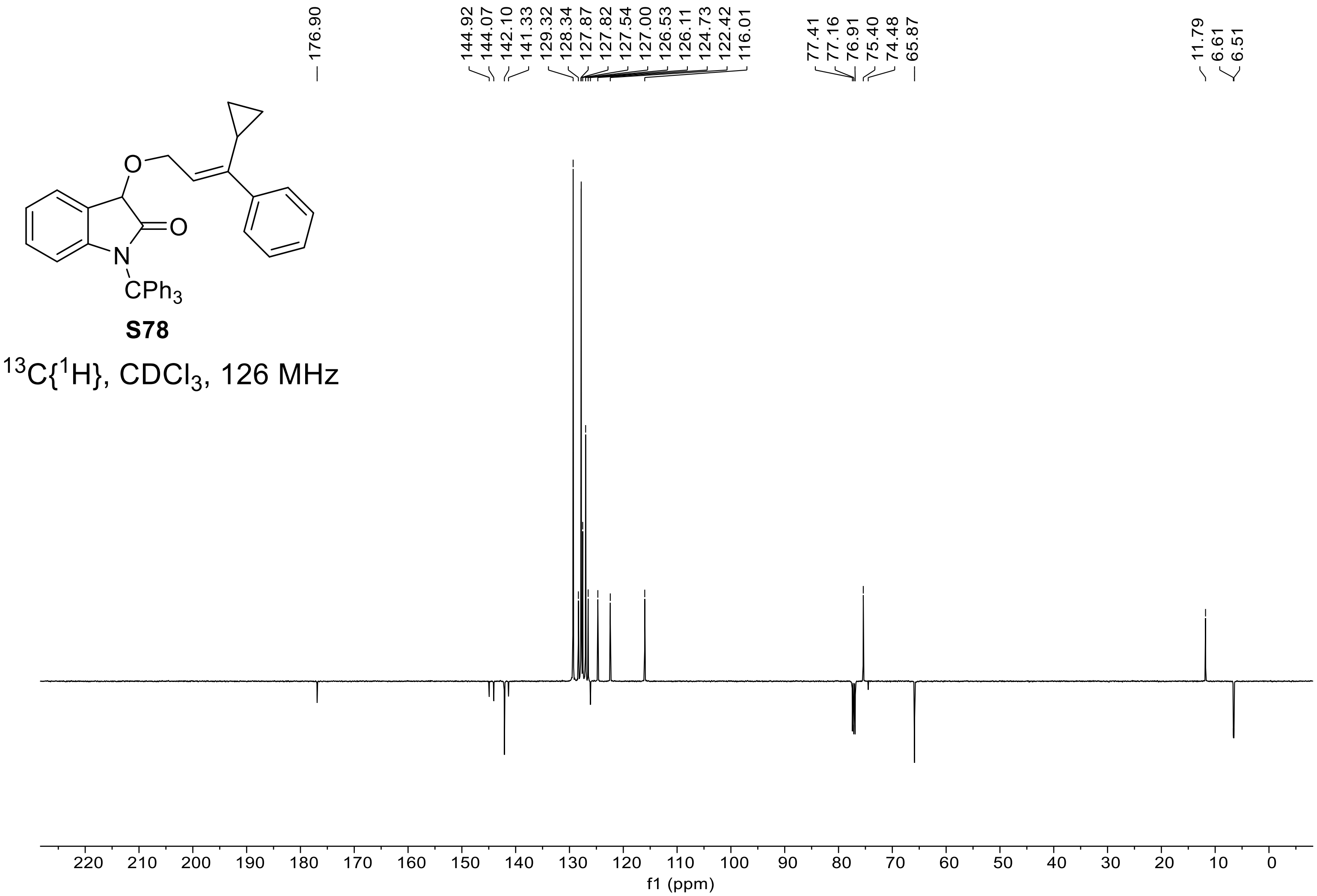

Fig. S197: $^{13}C\{^{1}H\}$ NMR spectrum for (*E*)-3-[(3-Cyclopropyl-3-phenylallyl)oxy]-1-tritylindolin-2-one (**S78**).

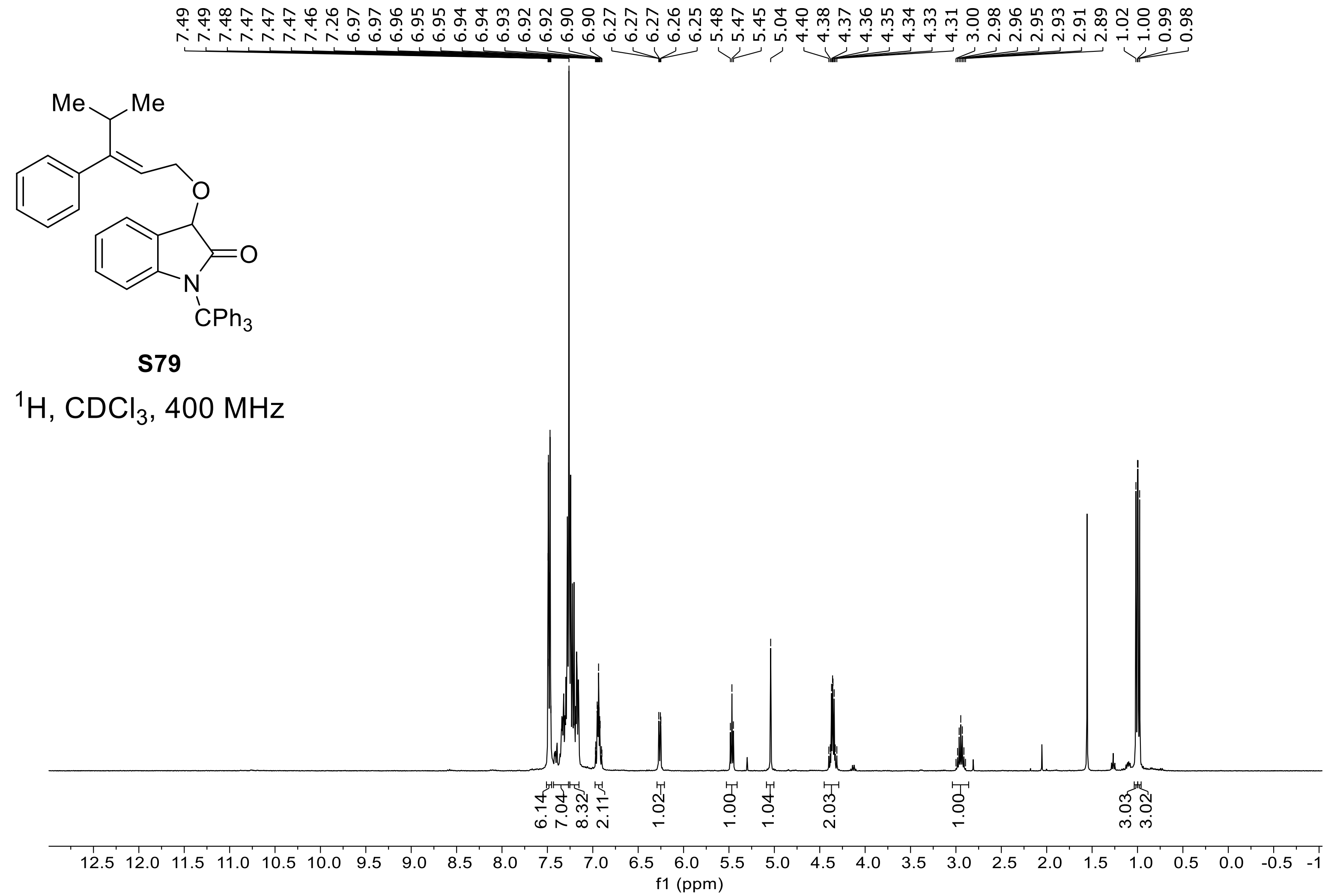

Fig. S198: ^{1}H NMR spectrum for (*E*)-3-[(4-Methyl-3-phenylpent-2-en-1-yl)oxy]-1-tritylindolin-2-one (**S79**).

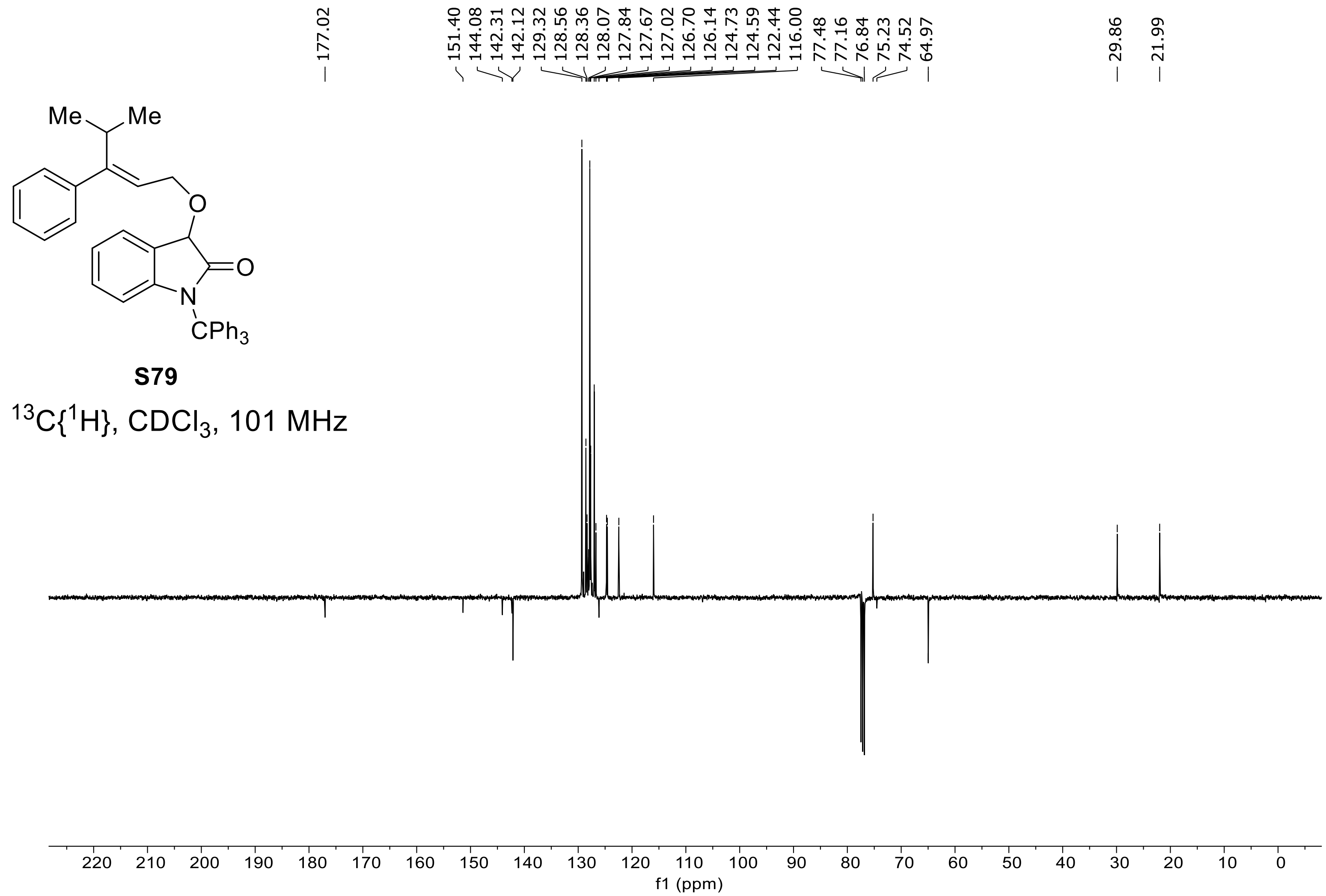

Fig. S199: $^{13}C\{^1H\}$ NMR spectrum for (*E*)-3-[(4-Methyl-3-phenylpent-2-en-1-yl)oxy]-1-tritylindolin-2-one (**S79**).

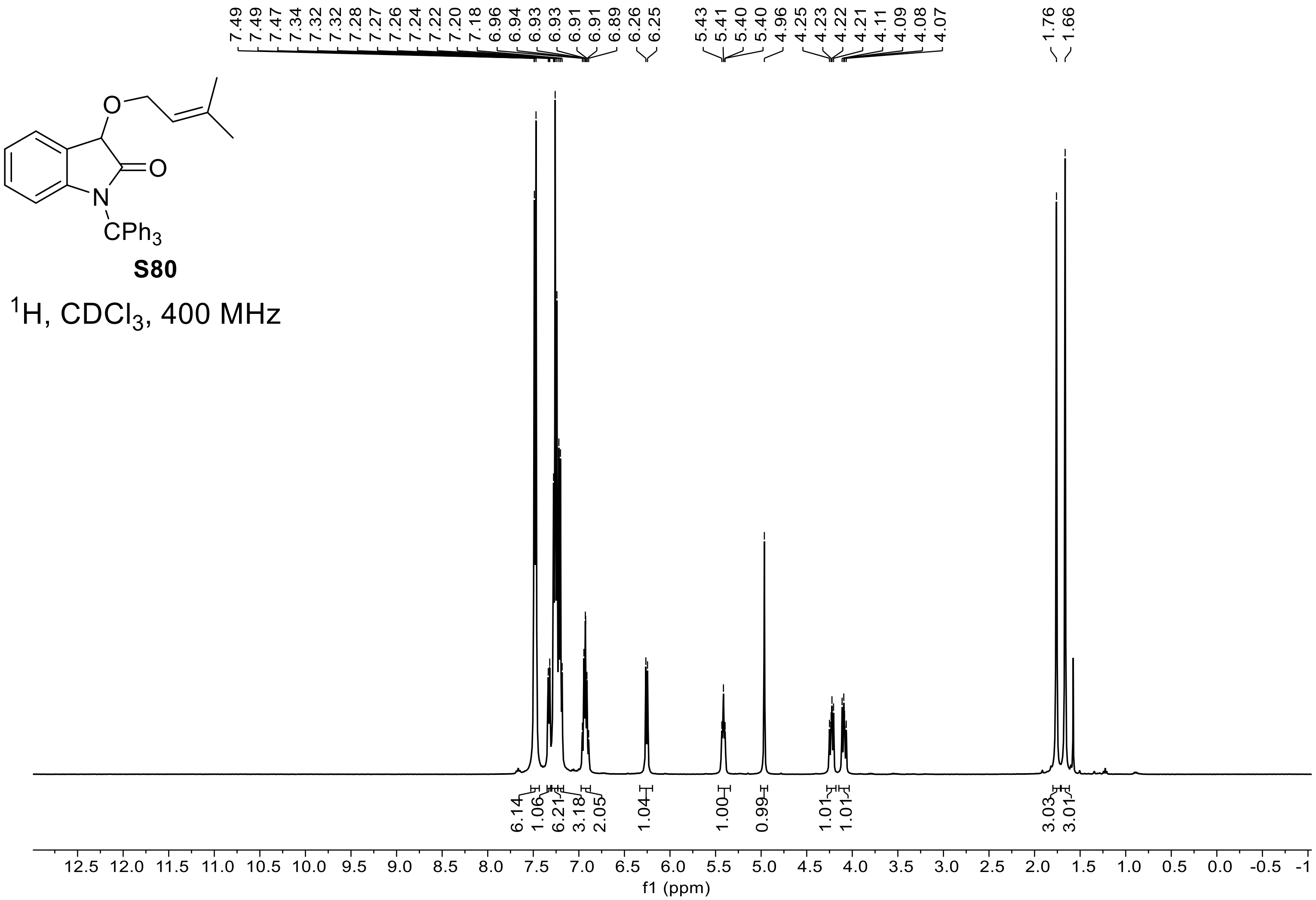

Fig. S200: ^{1}H NMR spectrum for 3-[(3-Methylbut-2-en-1-yl)oxy]-1-tritylindolin-2-one (**S80**).

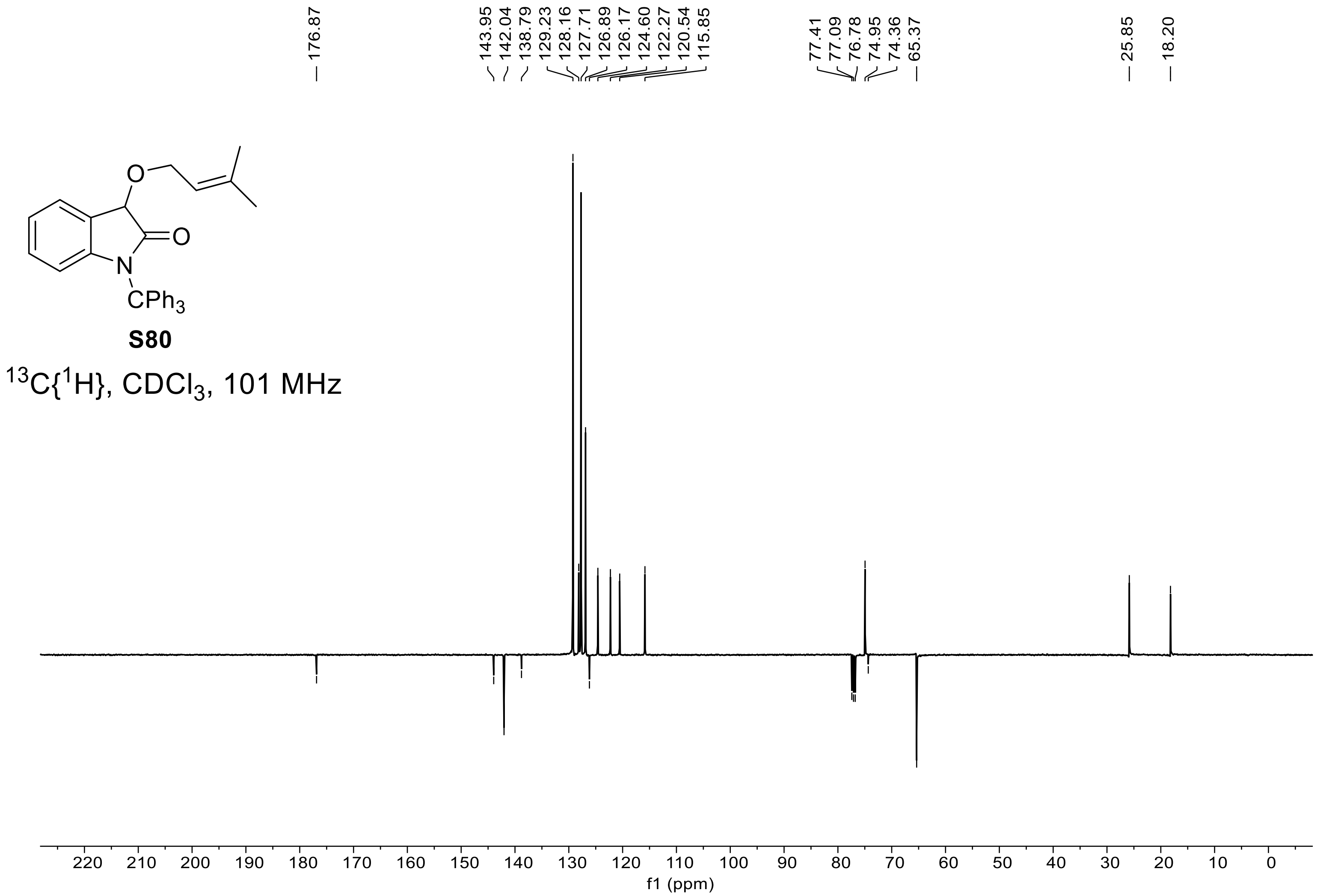

Fig. S201: $^{13}C\{^{1}H\}$ NMR spectrum for 3-[(3-Methylbut-2-en-1-yl)oxy]-1-tritylindolin-2-one (**S80**).

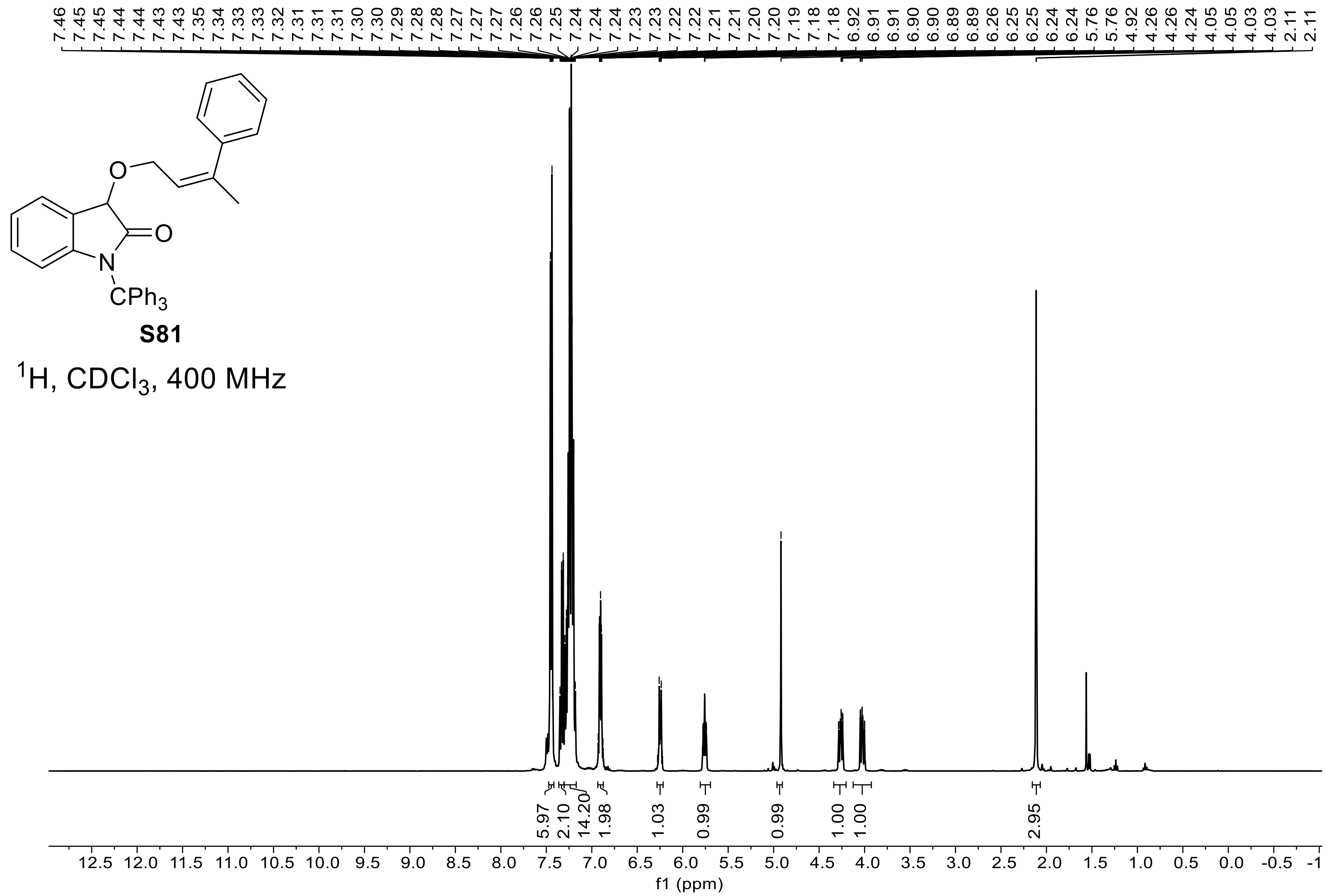

Fig. S202: ^{1}H NMR spectrum for (*Z*)-3-[(3-Phenylbut-2-en-1-yl)oxy]-1-tritylindolin-2-one (**S81**).

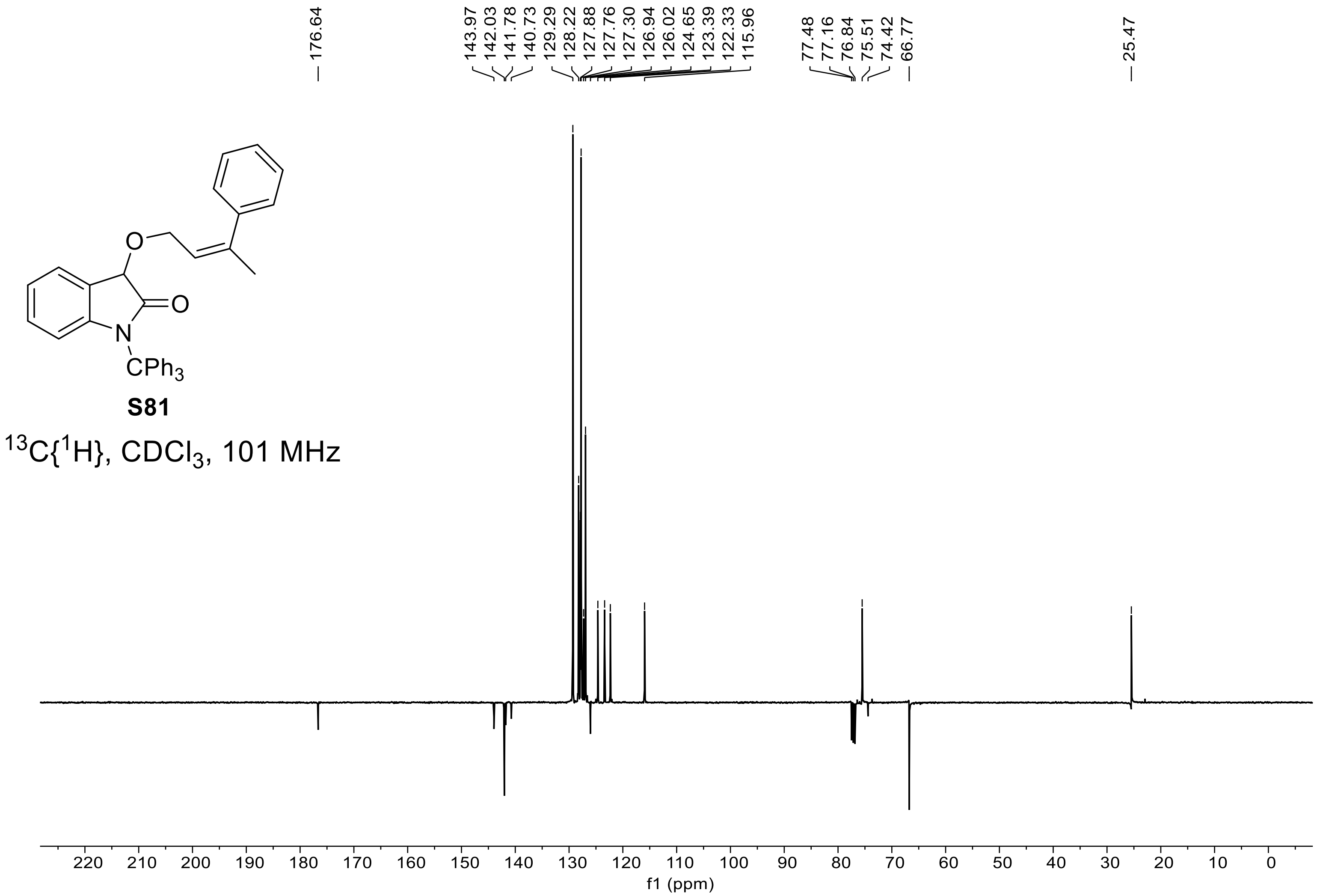

Fig. S203: $^{13}C\{^1H\}$ NMR spectrum for (*Z*)-3-[(3-Phenylbut-2-en-1-yl)oxy]-1-tritylindolin-2-one (**S81**).

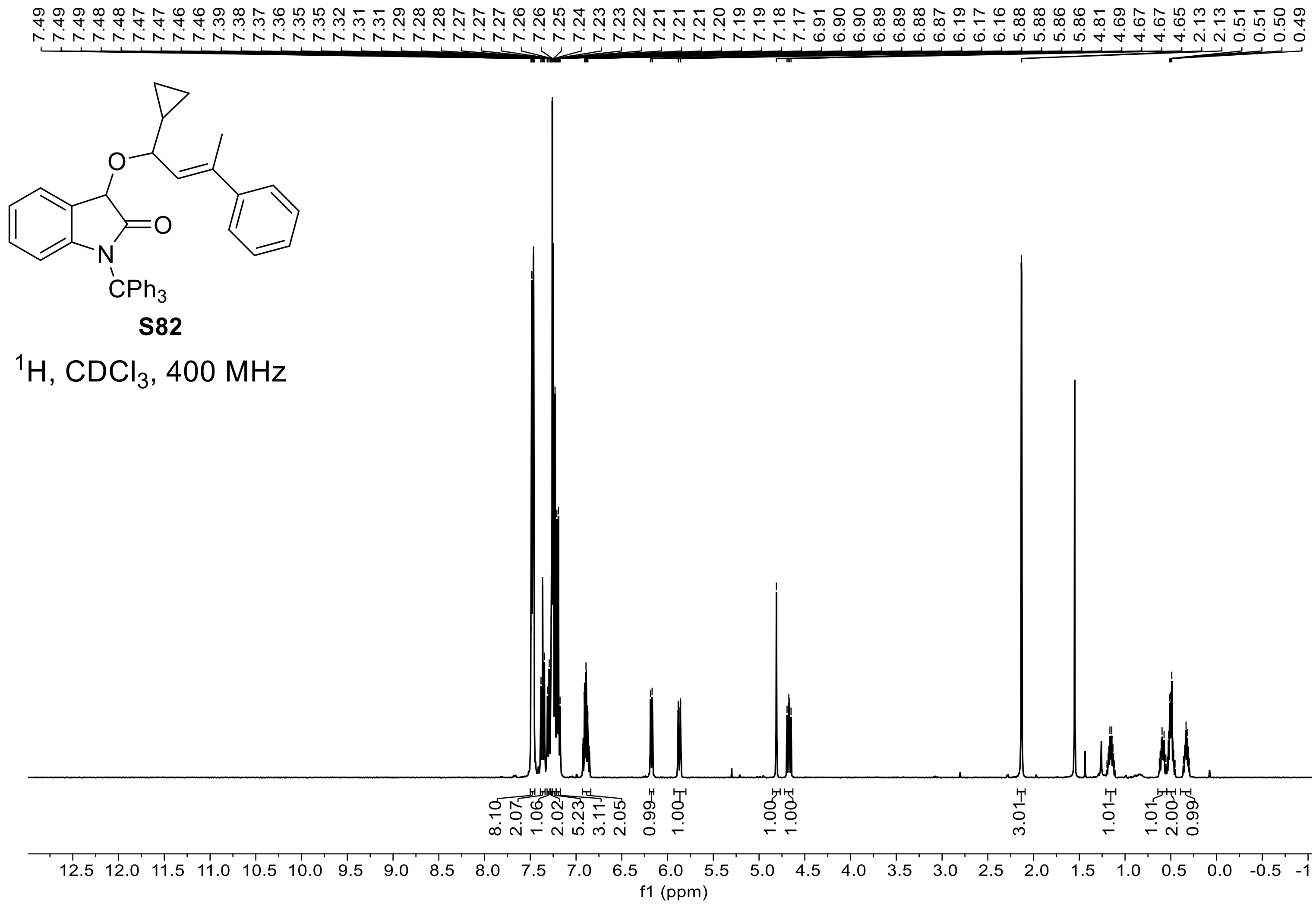

Fig. S204: ^{1}H NMR spectrum for (*E*)-3-[(1-Cyclopropyl-3-phenylbut-2-en-1-yl)oxy]-1-tritylindolin-2-one (**S82**).

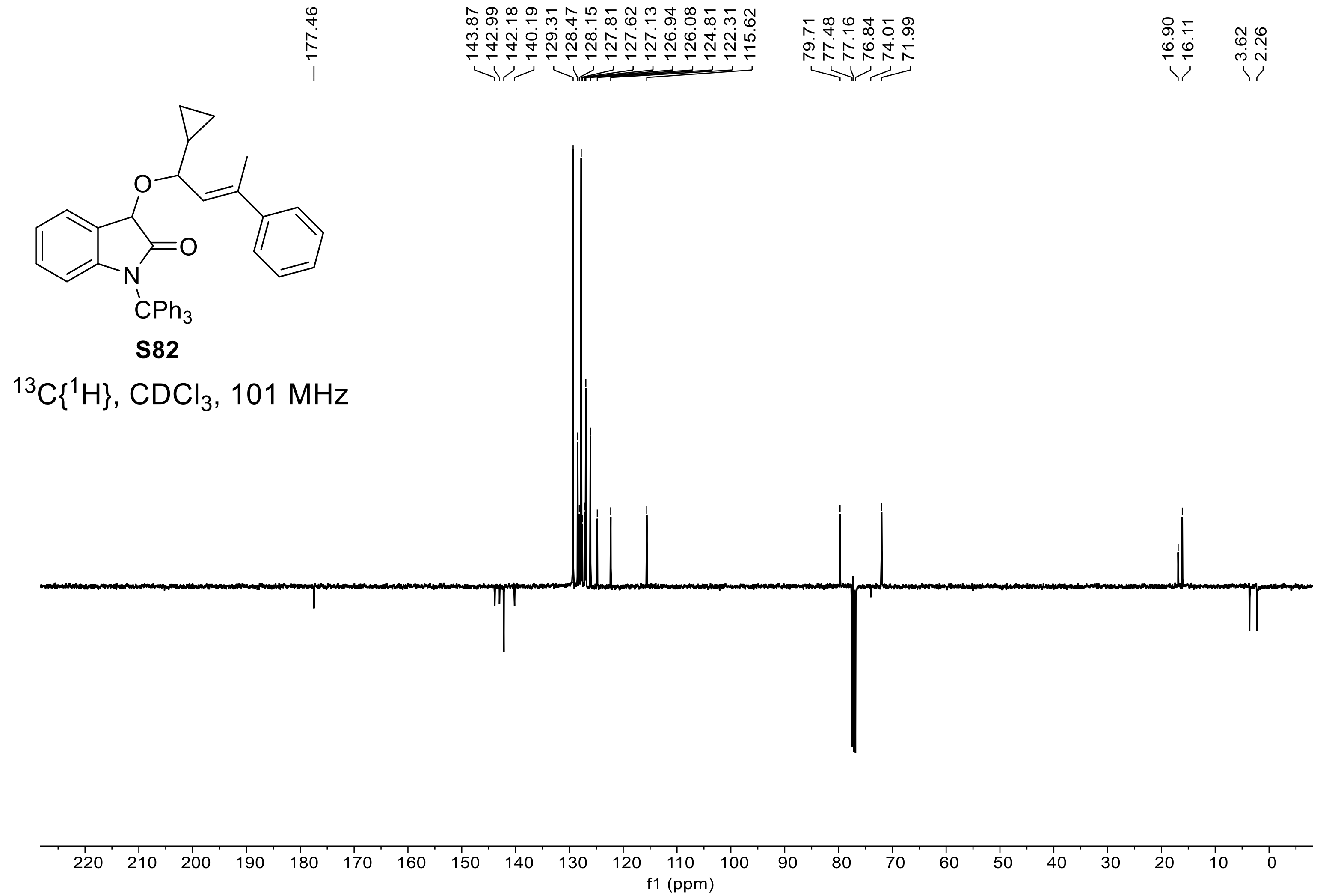

Fig. S205: $^{13}C\{^1H\}$ NMR spectrum for (*E*)-3-[(1-Cyclopropyl-3-phenylbut-2-en-1-yl)oxy]-1-tritylindolin-2-one (**S82**).

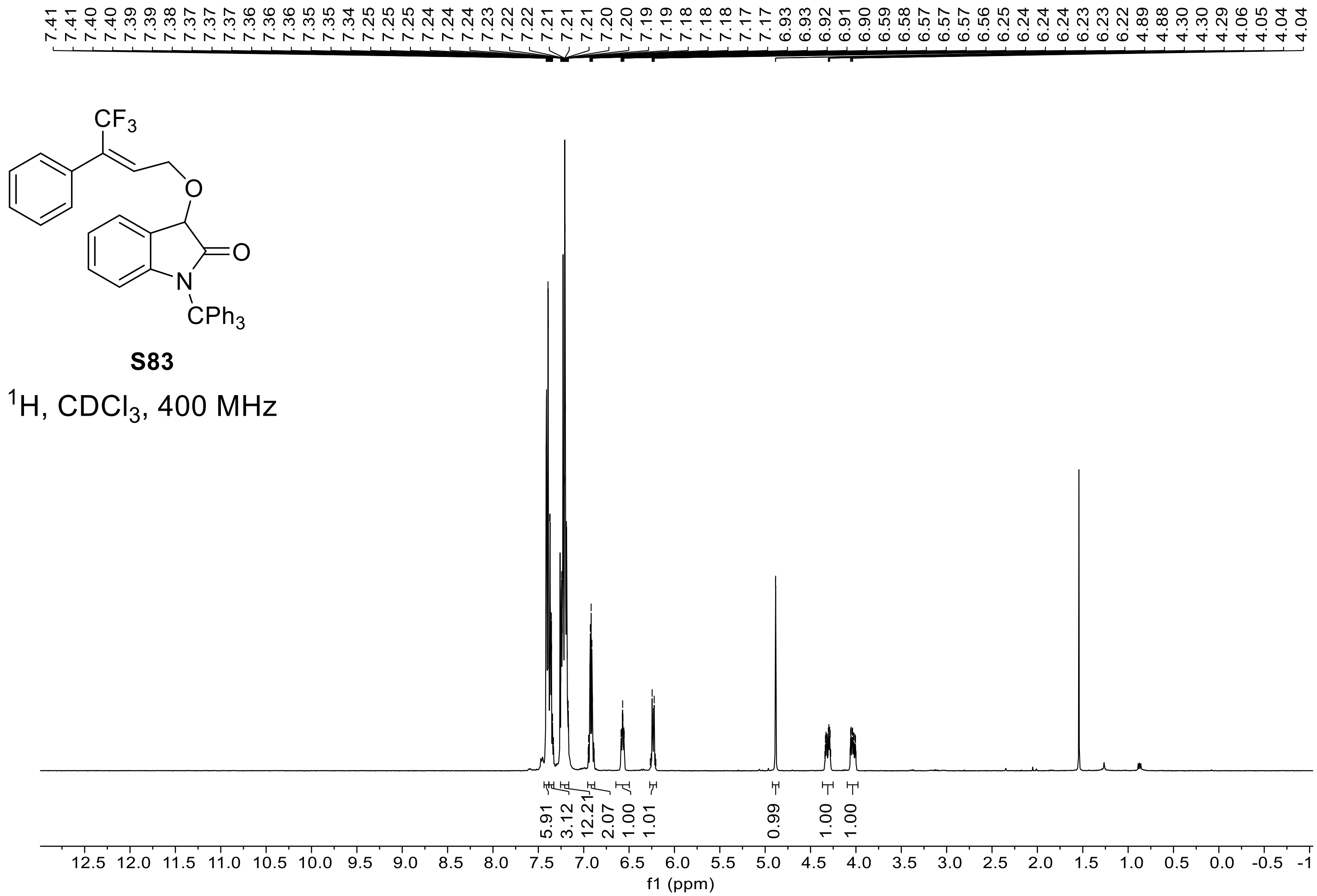

Fig. S206: ^{1}H NMR spectrum for (*Z*)-3-[(4,4,4-Trifluoro-3-phenylbut-2-en-1-yl)oxy]-1-tritylindolin-2-one (**S83**).

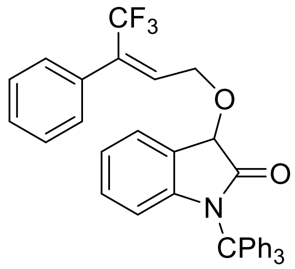

S83

$^{13}C\{^{1}H\}$, $CDCl_3$, 101 MHz

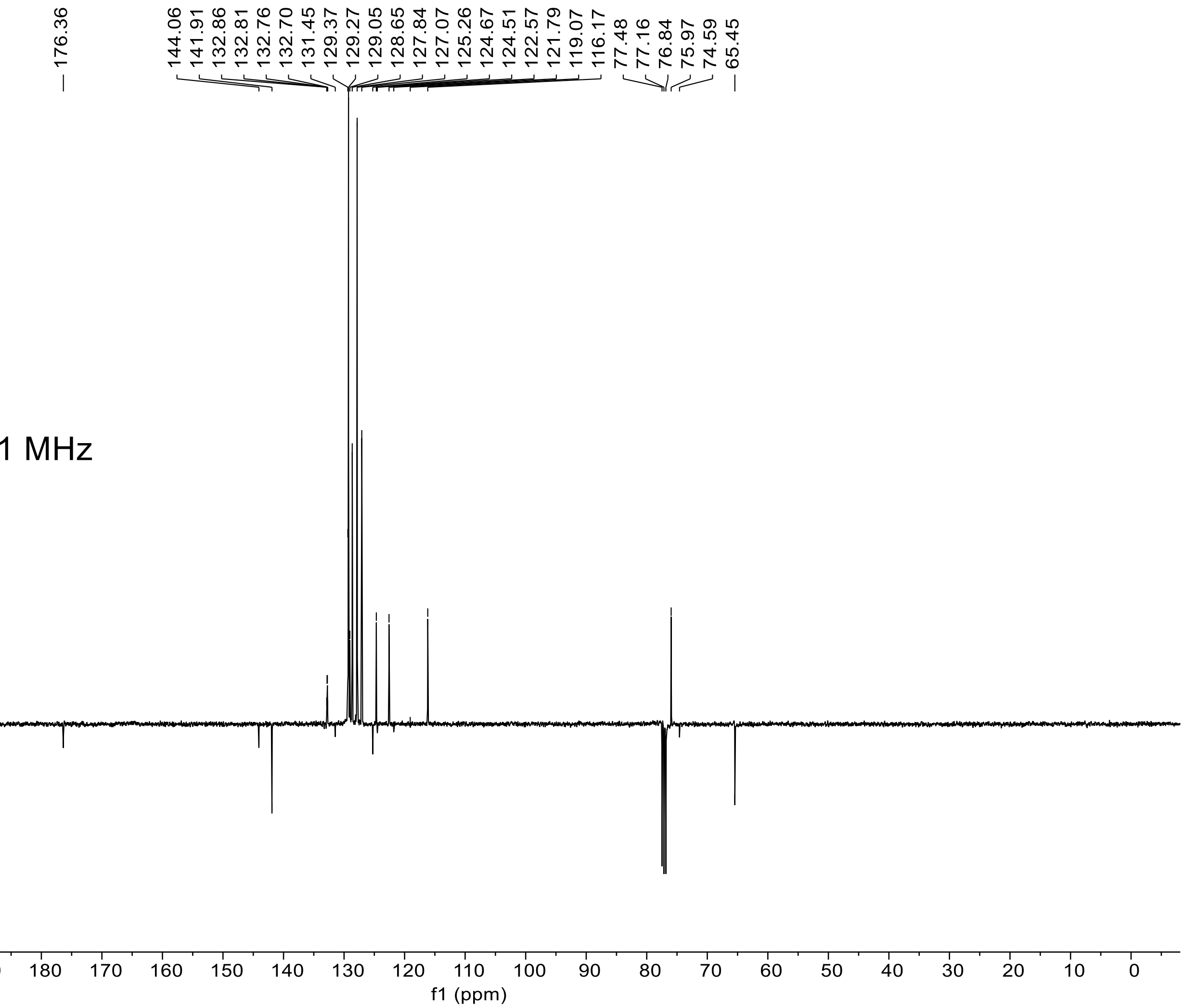

Fig. S207: $^{13}C\{^{1}H\}$ NMR spectrum for (*Z*)-3-[(4,4,4-Trifluoro-3-phenylbut-2-en-1-yl)oxy]-1-tritylindolin-2-one (**S83**).

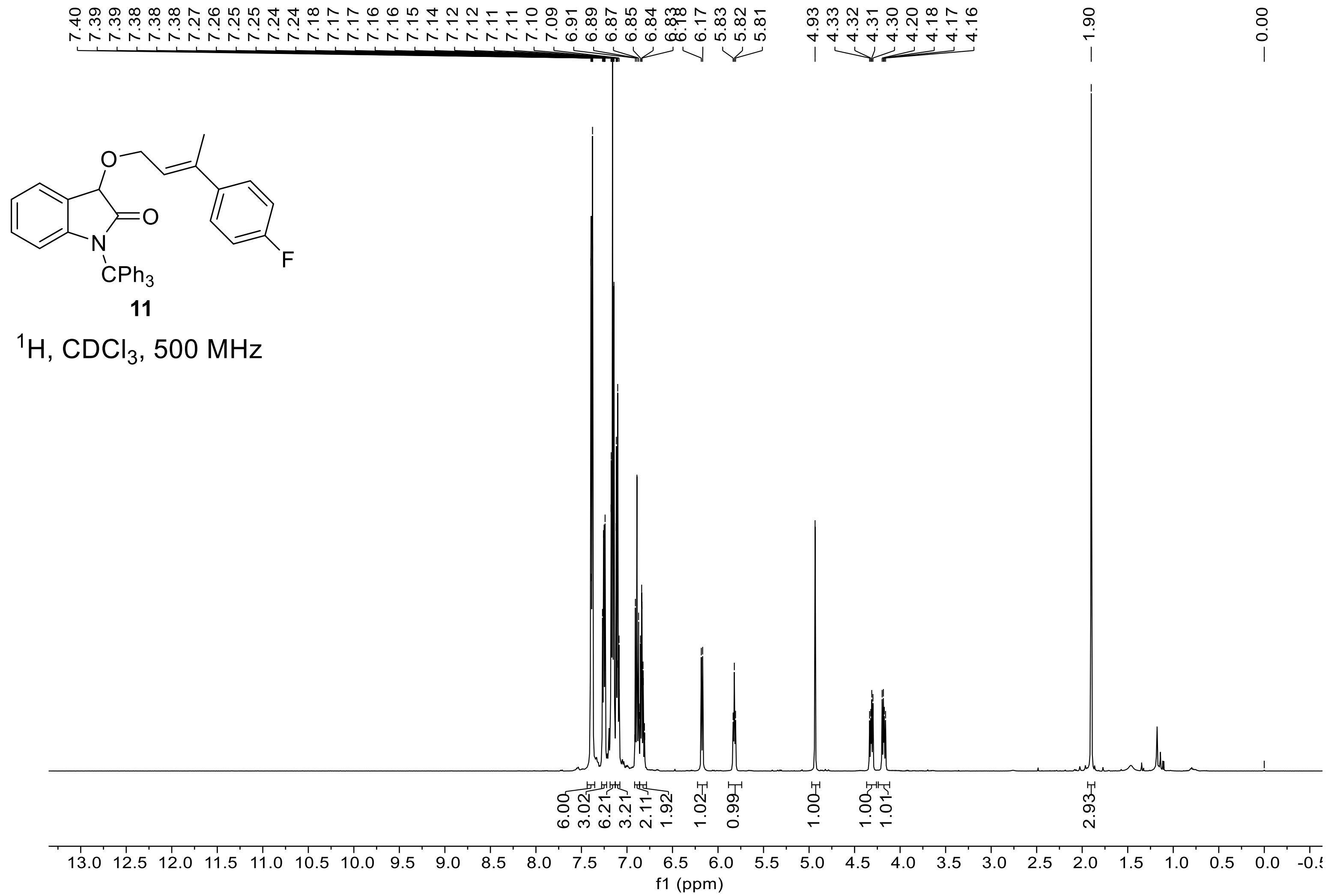

Fig. S208: ^{1}H NMR spectrum for (*E*)-3-{[3-(4-Fluorophenyl)but-2-en-1-yl]oxy}-1-tritylindolin-2-one (**11**).

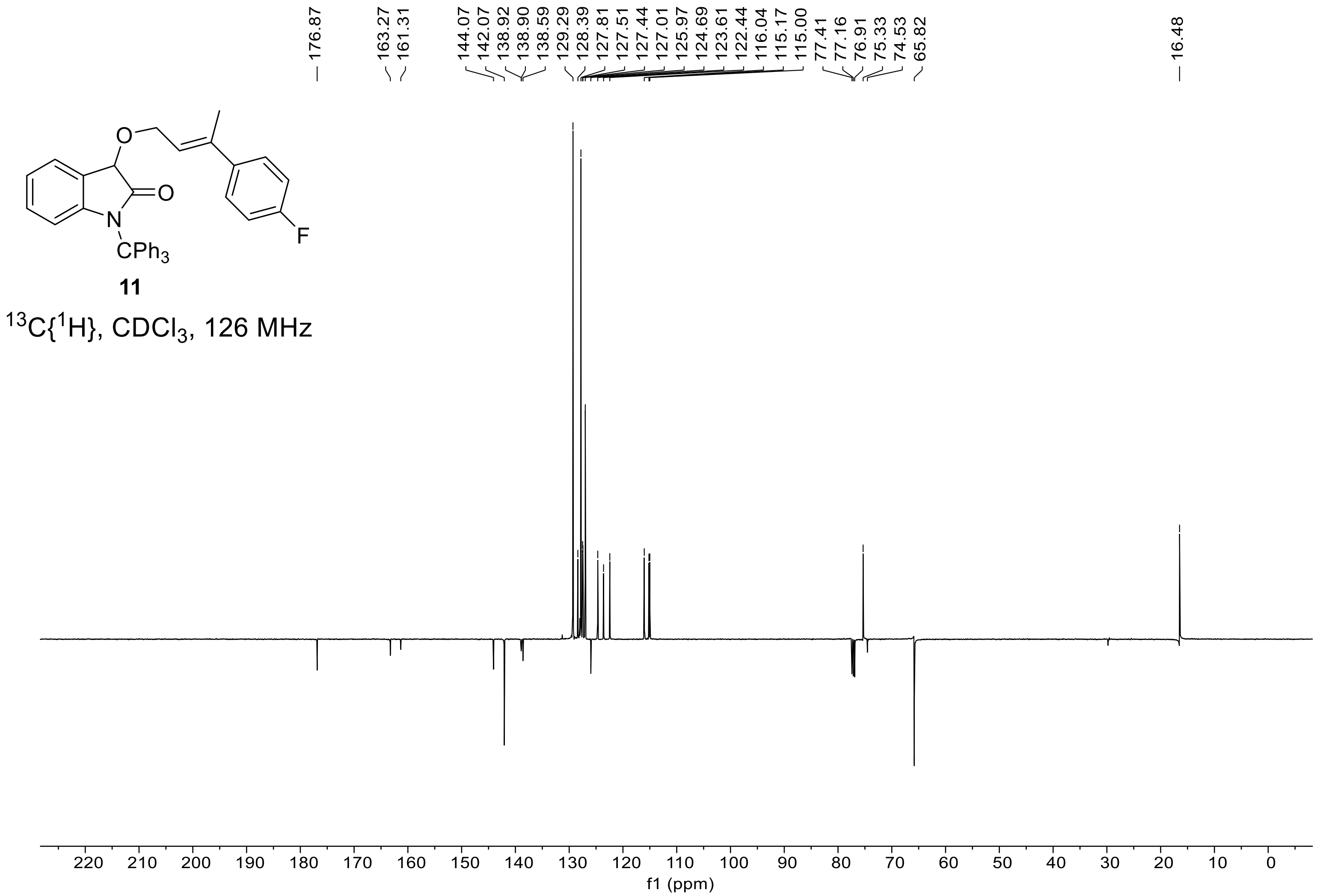

Fig. S209: $^{13}C\{^{1}H\}$ NMR spectrum for (*E*)-3-{[3-(4-Fluorophenyl)but-2-en-1-yl]oxy}-1-tritylindolin-2-one (**11**).

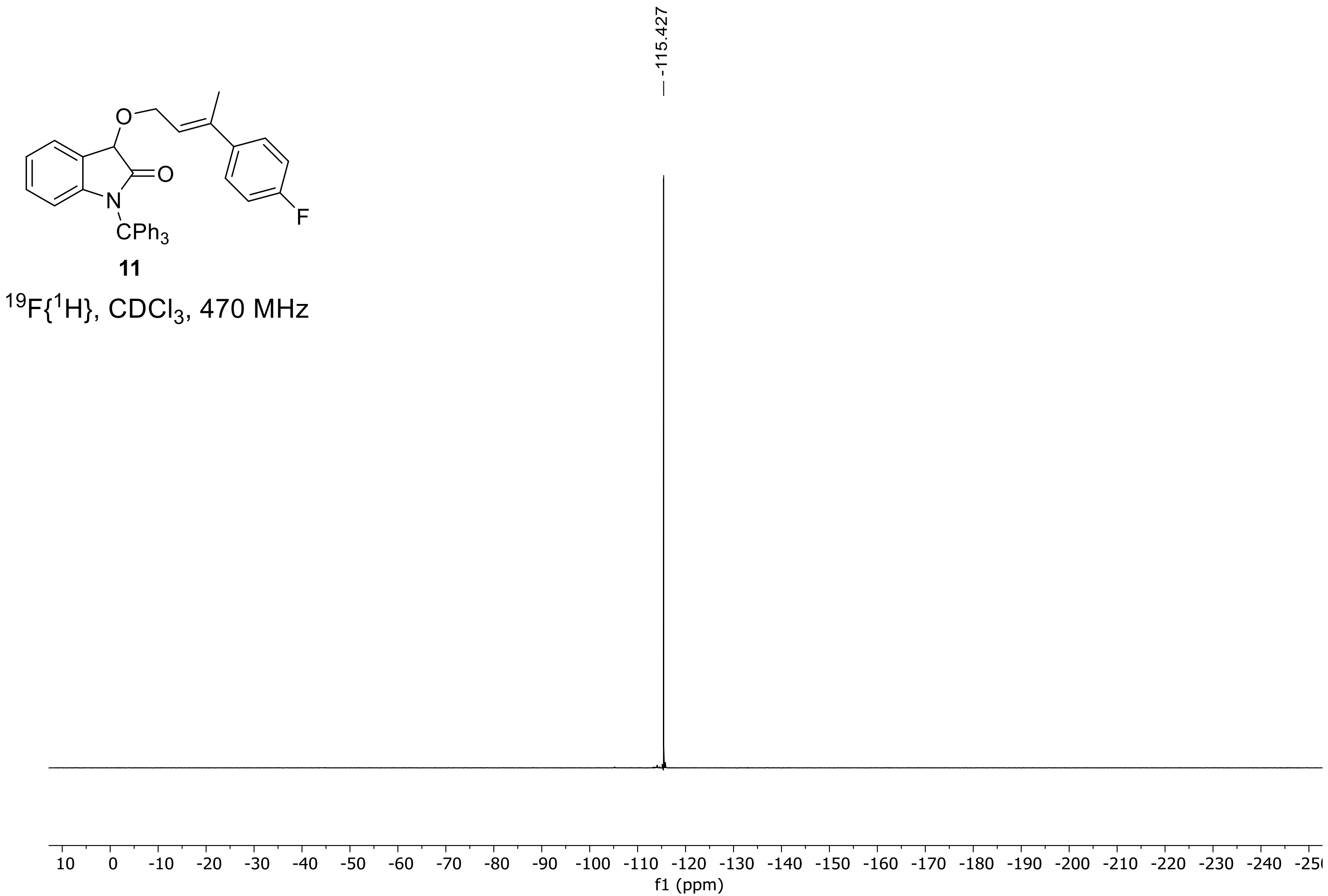

Fig. S210: $^{19}F\{^1H\}$ NMR spectrum for (*E*)-3-{[3-(4-Fluorophenyl)but-2-en-1-yl]oxy}-1-tritylindolin-2-one (**11**).

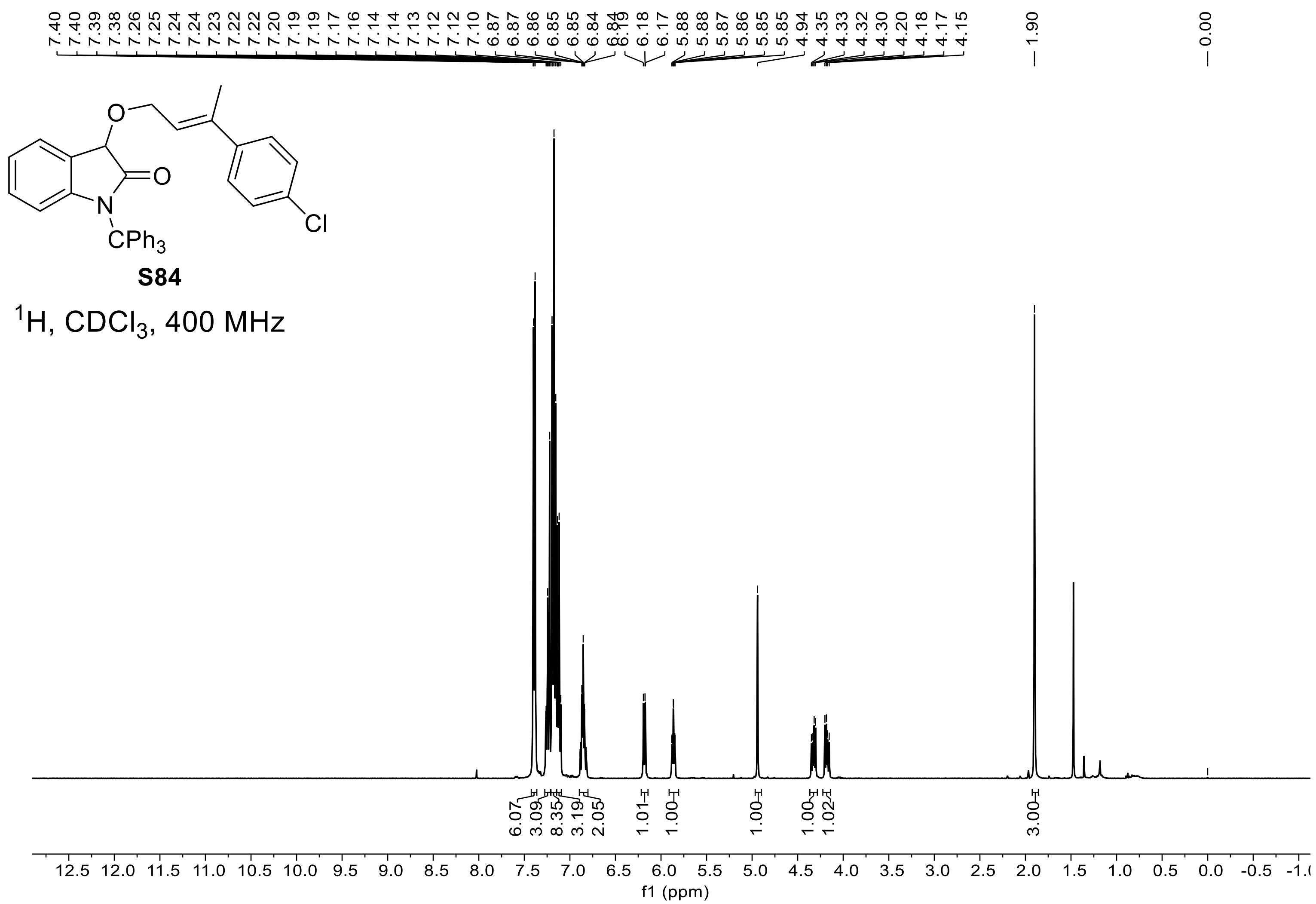

Fig. S211: ^{1}H NMR spectrum for (*E*)-3-{[3-(4-Chlorophenyl)but-2-en-1-yl]oxy}-1-tritylindolin-2-one (**S84**).

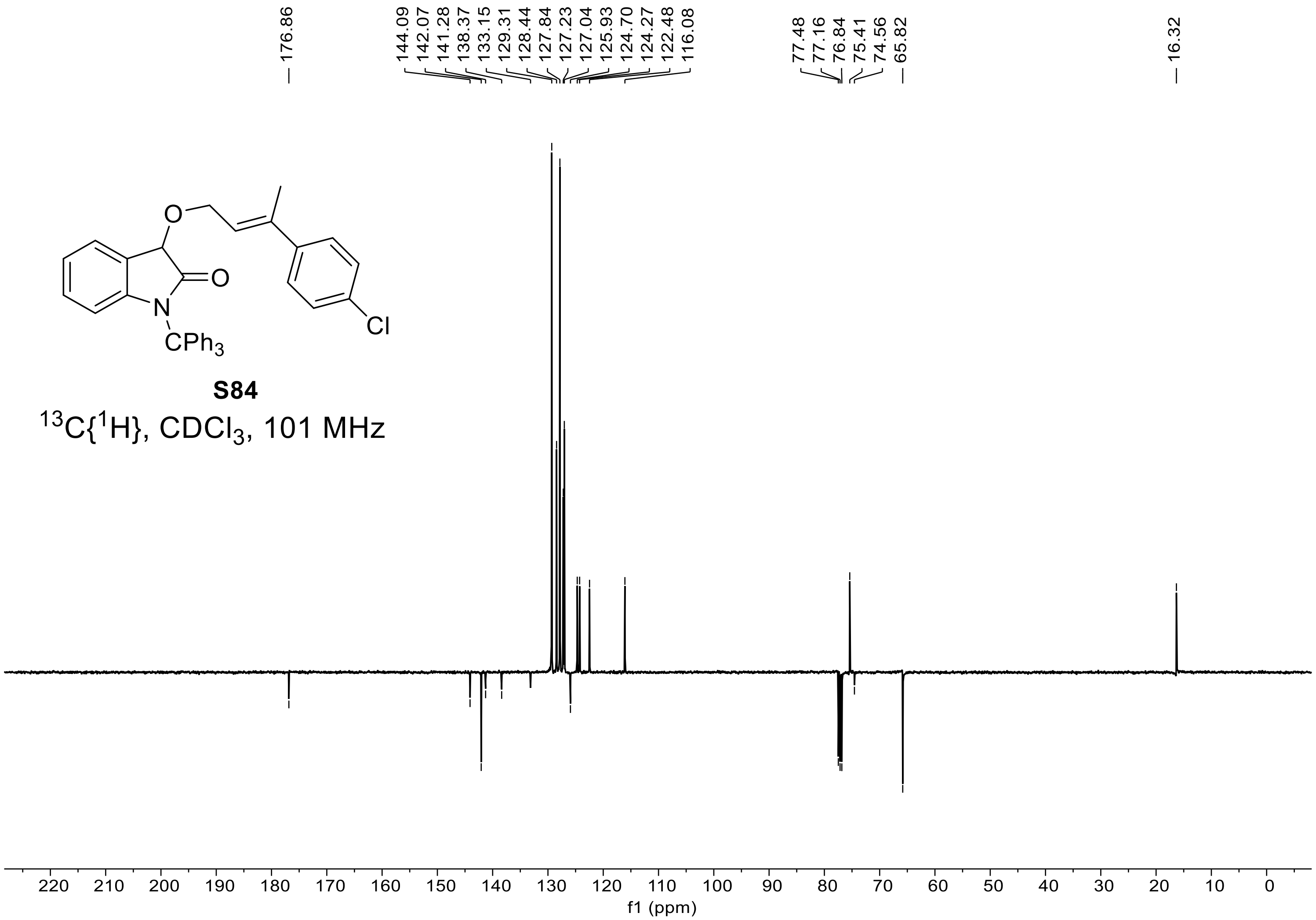

Fig. S212: $^{13}C\{^{1}H\}$ NMR spectrum for (*E*)-3-{[3-(4-Chlorophenyl)but-2-en-1-yl]oxy}-1-tritylindolin-2-one (**S84**).

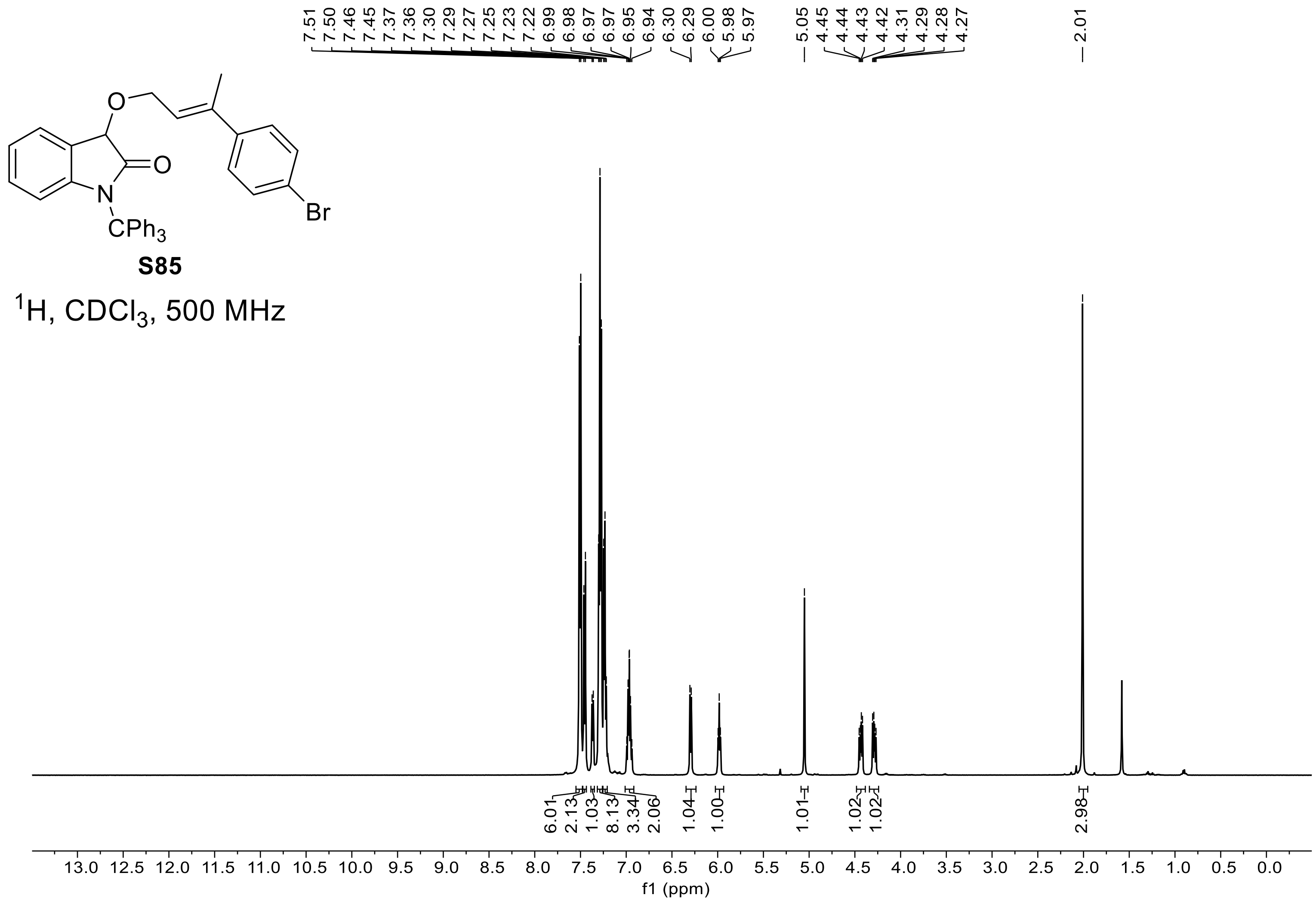

Fig. S213: ^{1}H NMR spectrum for (*E*)-3-{[3-(4-Bromophenyl)but-2-en-1-yl]oxy}-1-tritylindolin-2-one (**S85**).

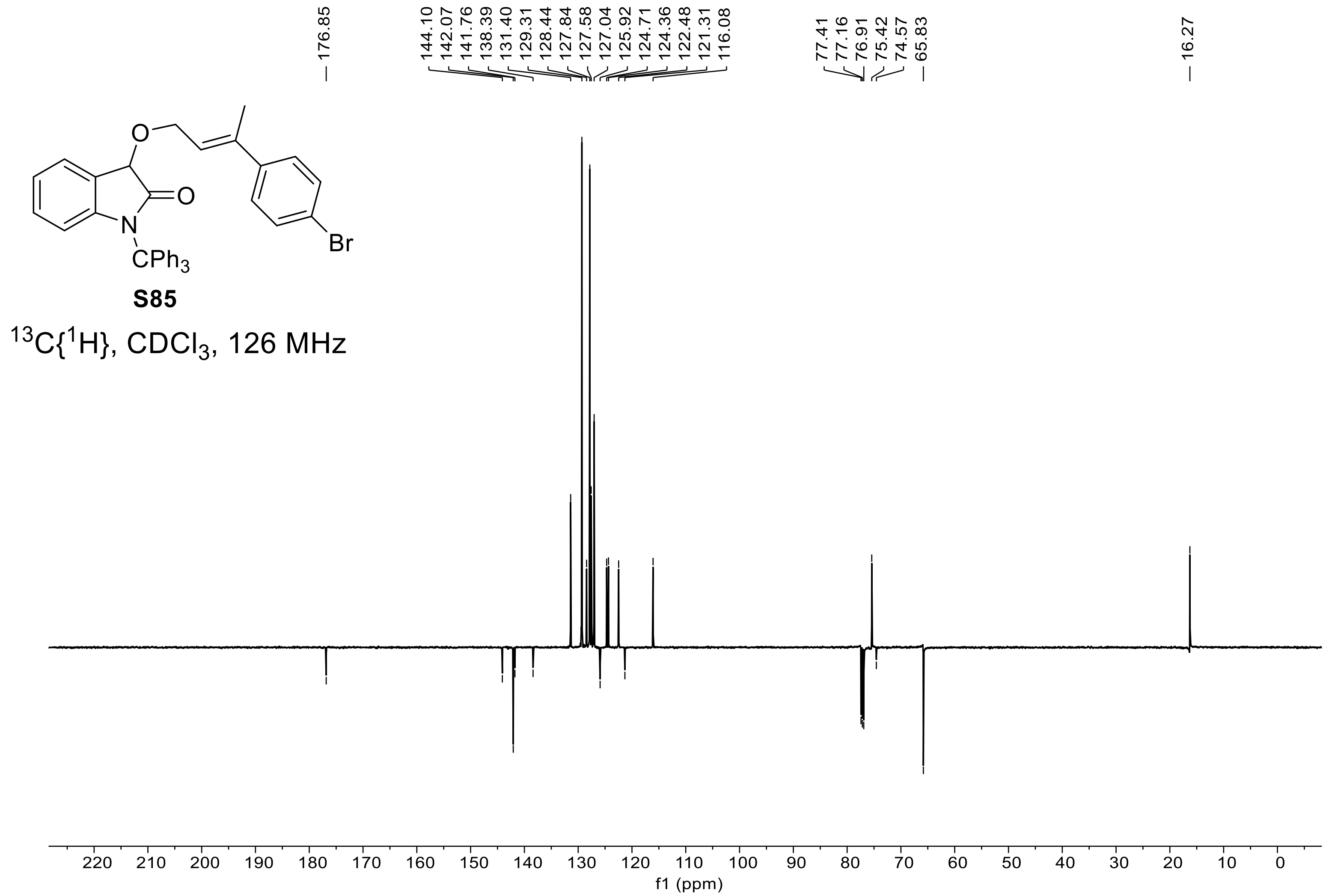

Fig. S214: ^{1}H NMR spectrum for (*E*)-3-{[3-(4-Bromophenyl)but-2-en-1-yl]oxy}-1-tritylindolin-2-one (**S85**).

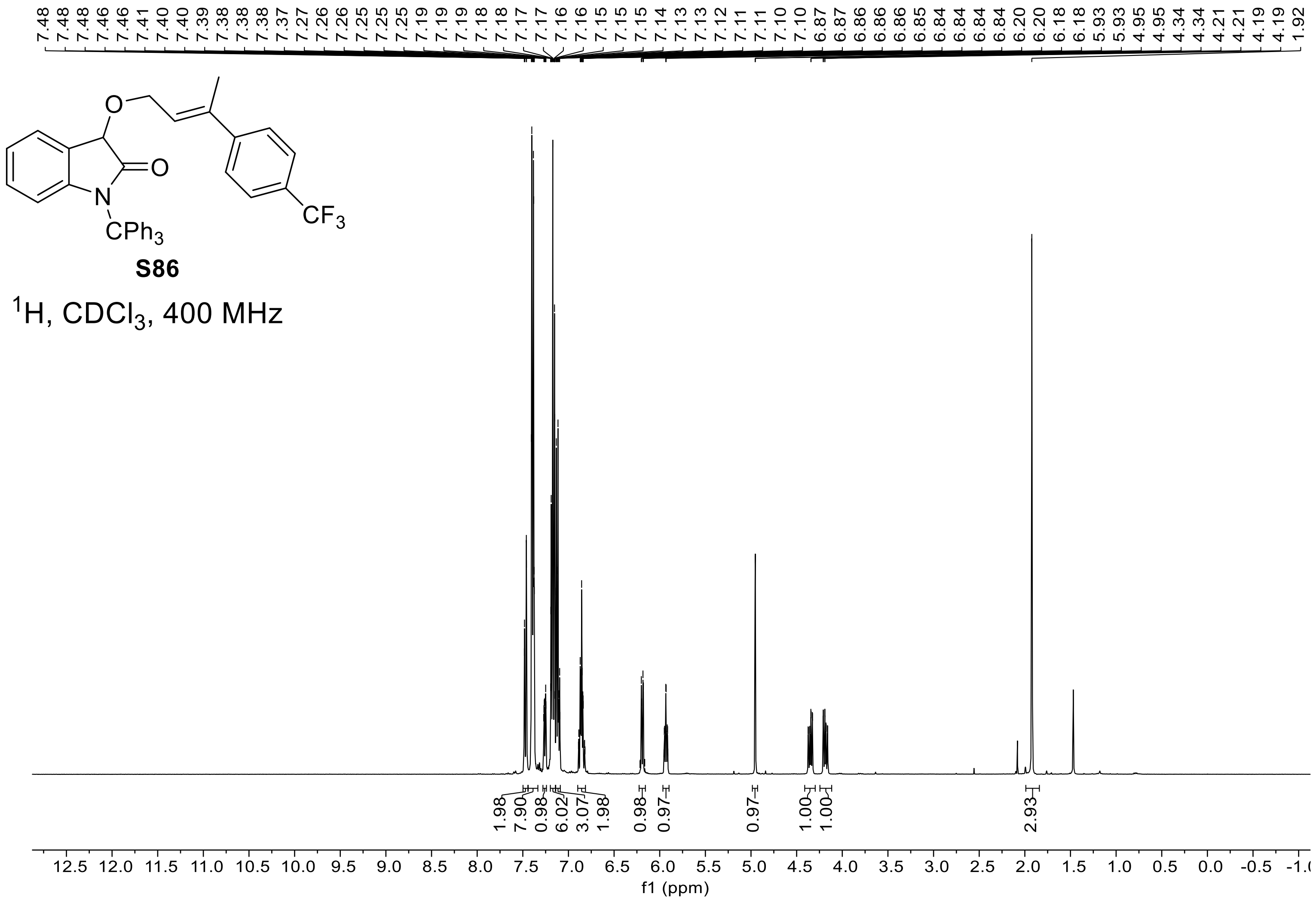

Fig. S215: ^{1}H NMR spectrum for (*E*)-3-{[3-(4-(Trifluoromethyl)phenyl)but-2-en-1-yl]oxy}-1-tritylindolin-2-one (**S86**).

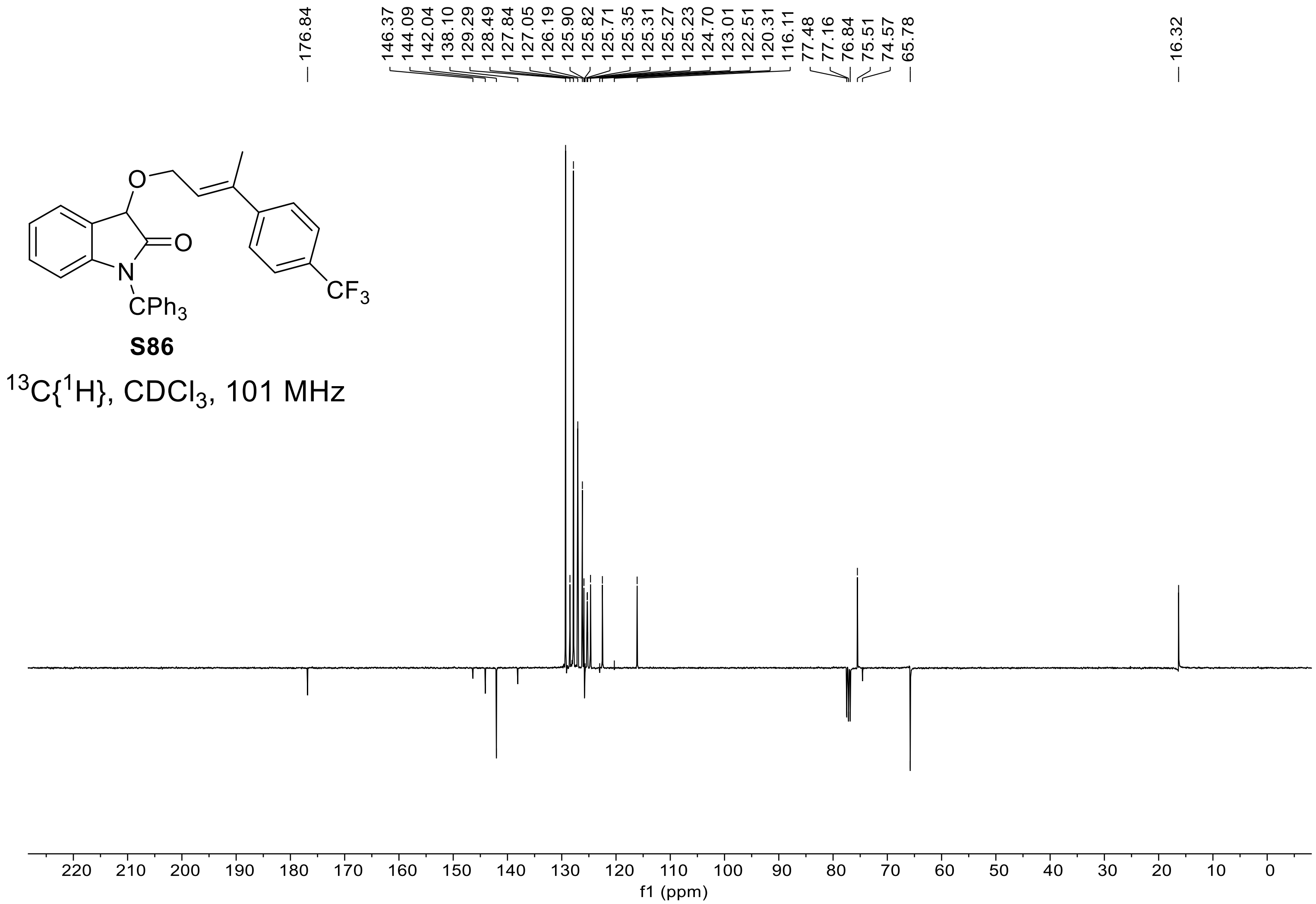

Fig. S216: $^{13}C\{^1H\}$ NMR spectrum for (*E*)-3-{[3-(4-(Trifluoromethyl)phenyl)but-2-en-1-yl]oxy}-1-tritylindolin-2-one (**S86**).

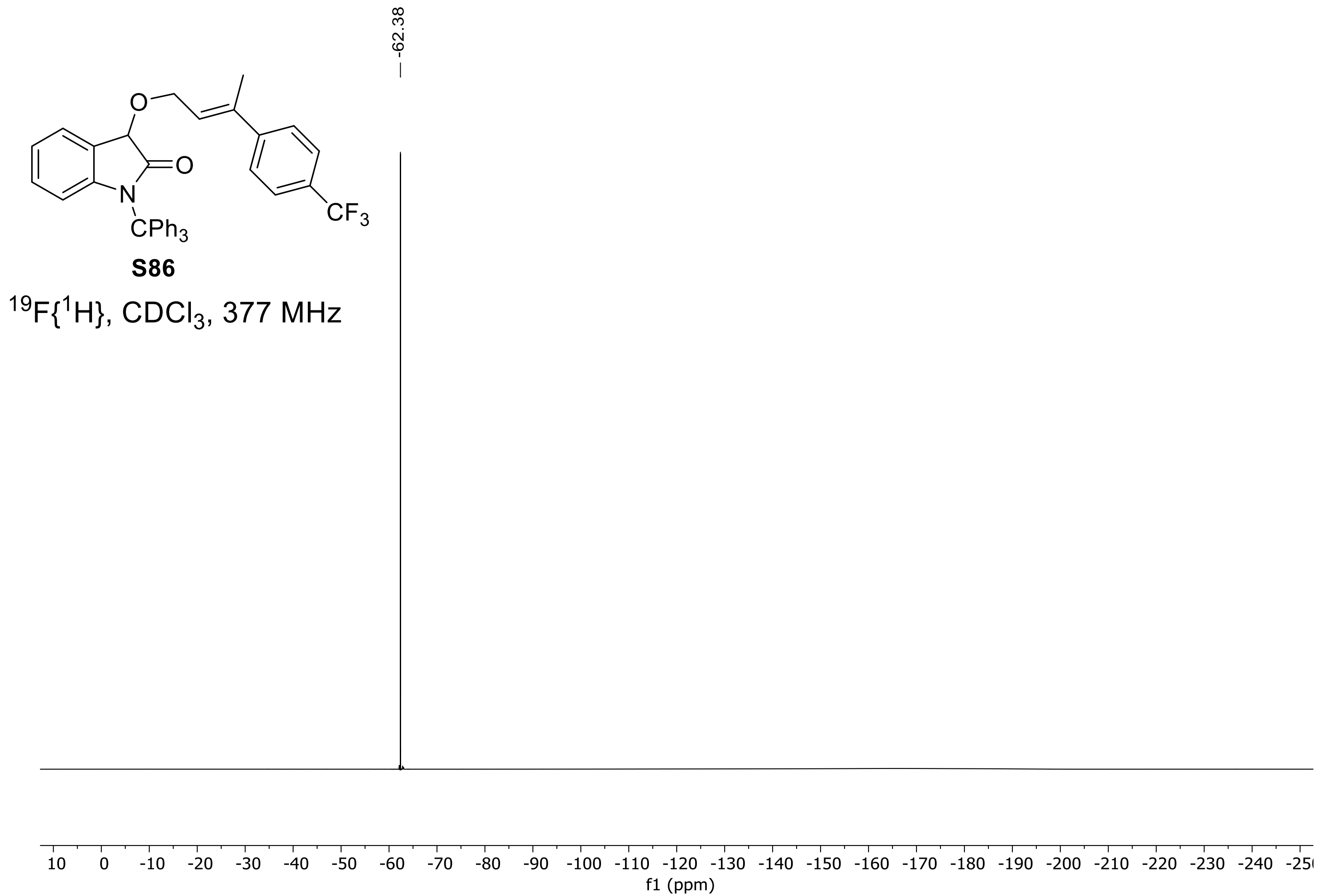

Fig. S217: $^{19}F\{^{1}H\}$ NMR spectrum for (*E*)-3-{[3-(4-(Trifluoromethyl)phenyl)but-2-en-1-yl]oxy}-1-tritylindolin-2-one (**S86**).

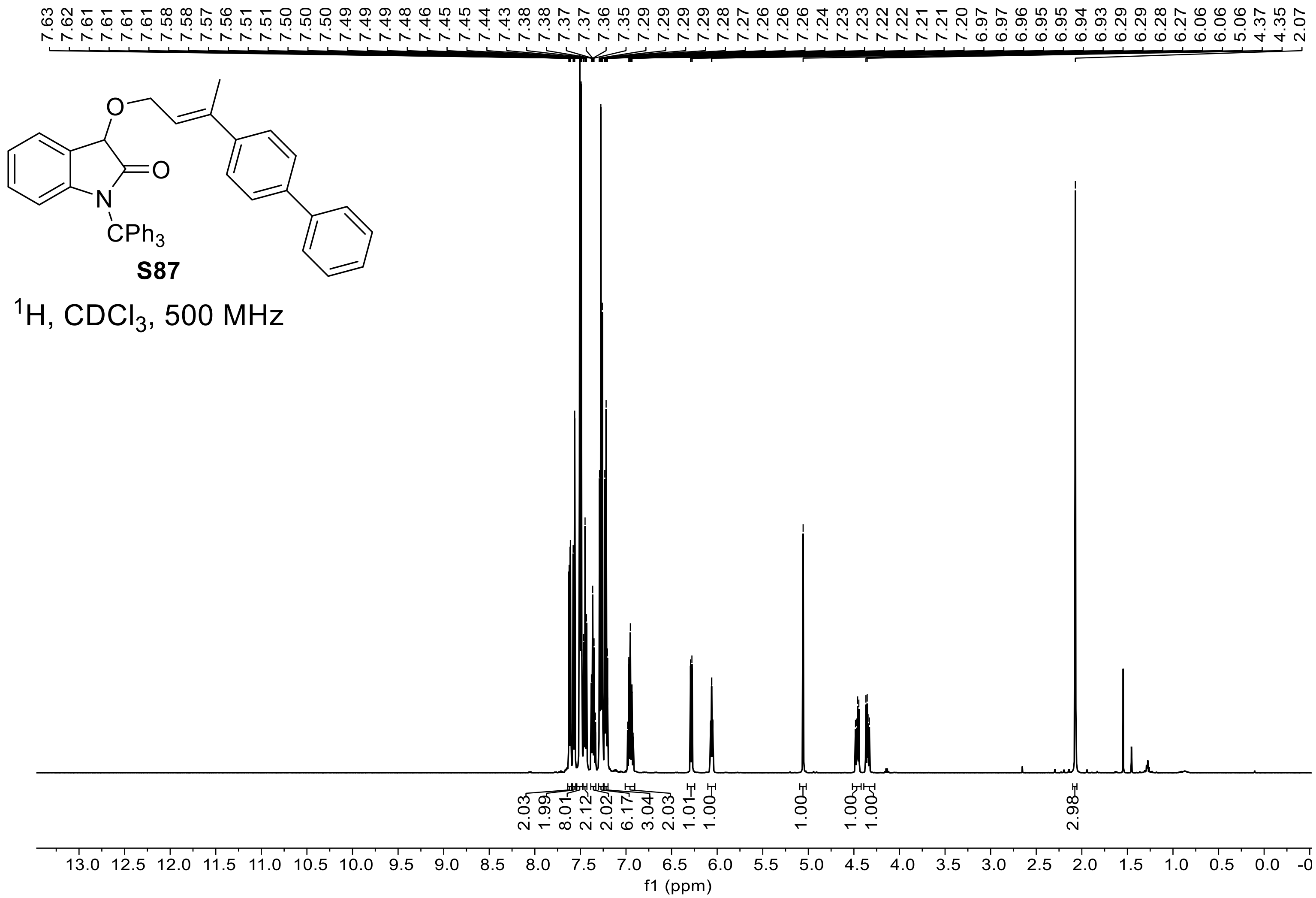

Fig. S218: ^{1}H NMR spectrum for (*E*)-3-{[3-((1,1'-Biphenyl)-4-yl)but-2-en-1-yl]oxy}-1-tritylindolin-2-one (**S87**).

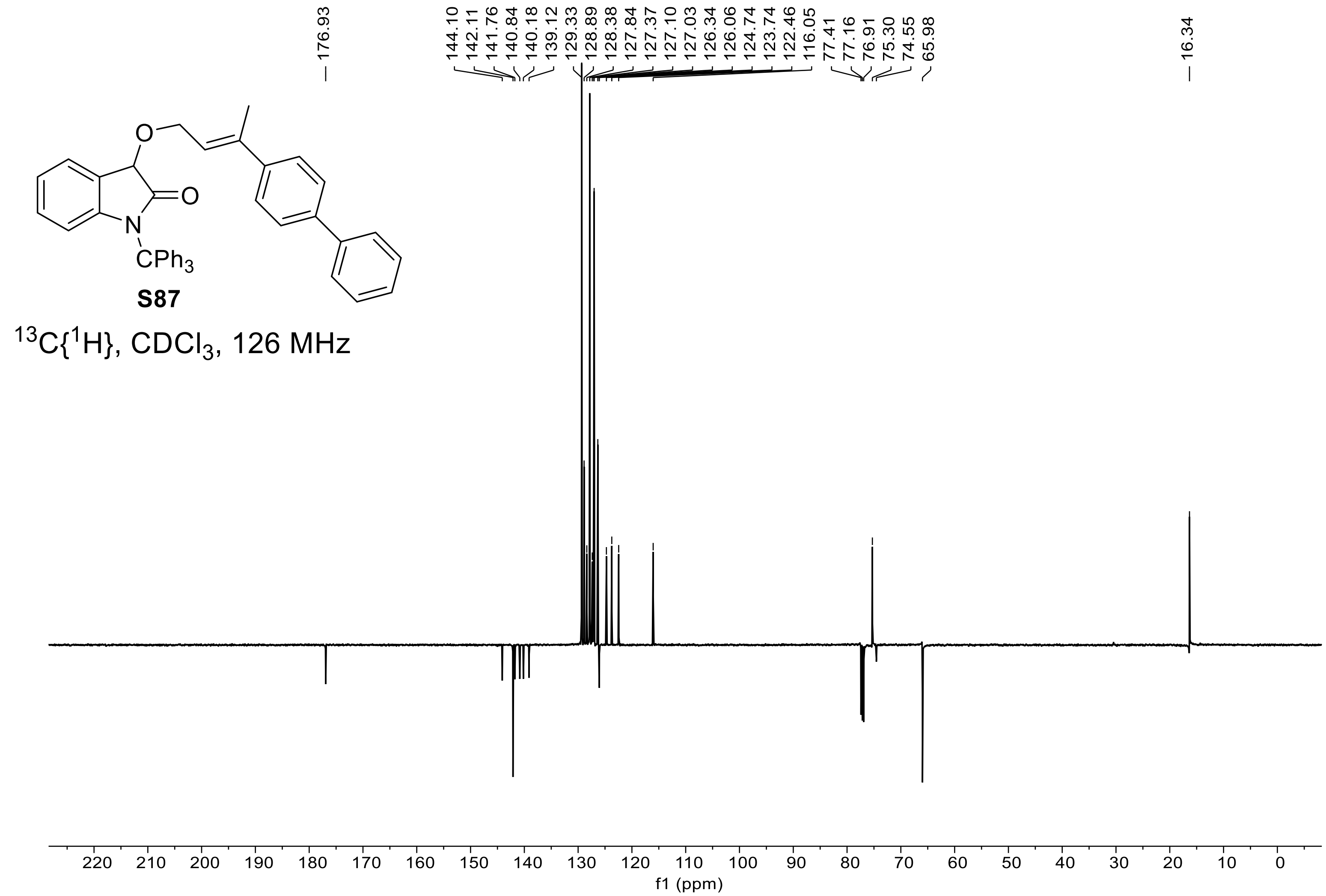

Fig. S219: $^{13}C\{^{1}H\}$ NMR spectrum for (*E*)-3-{[3-((1,1'-Biphenyl)-4-yl)but-2-en-1-yl]oxy}-1-tritylindolin-2-one (**S87**).

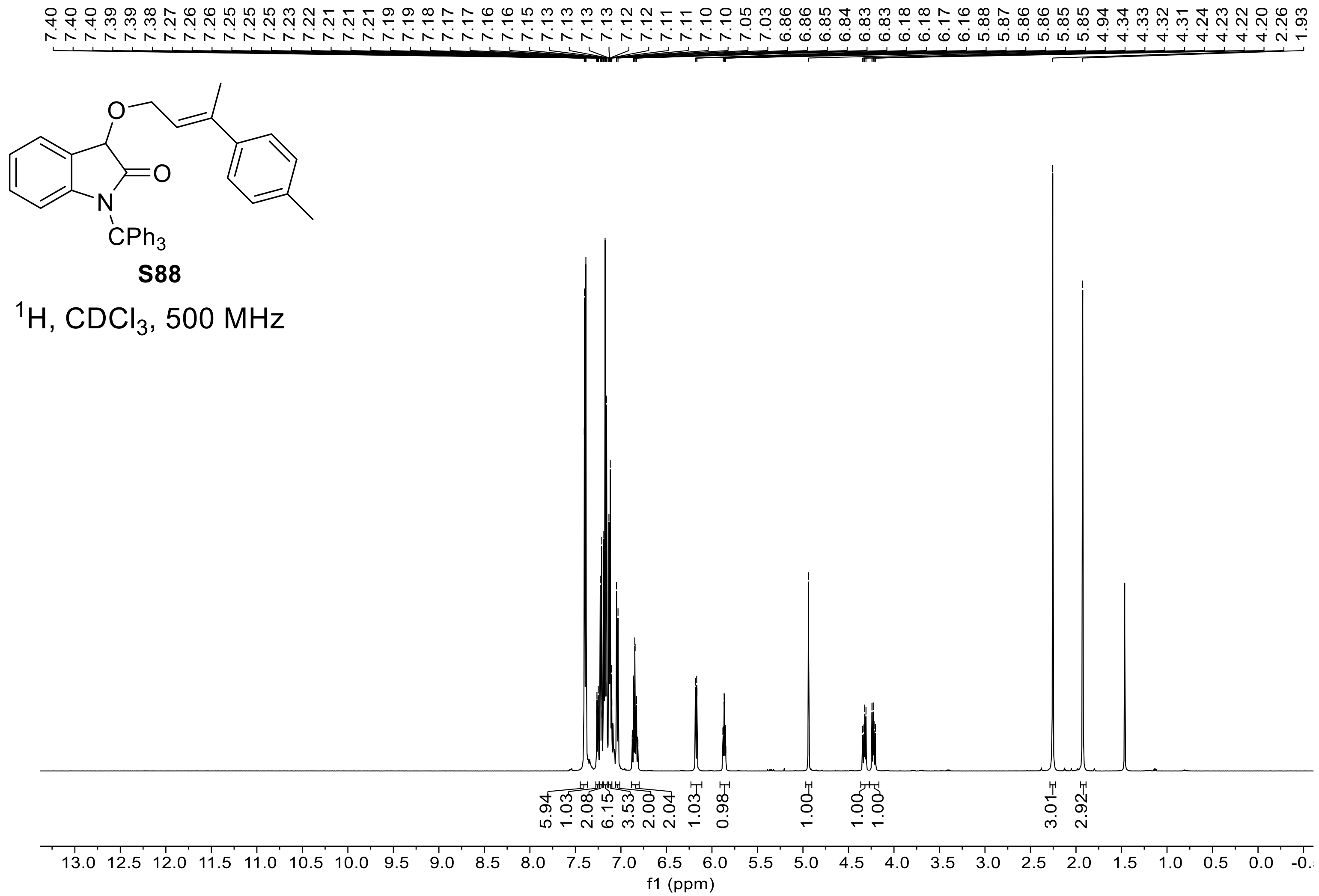

Fig. S220: ^{1}H NMR spectrum for (*E*)-3-{[3-(*p*-Tolyl)but-2-en-1-yl]oxy}-1-tritylindolin-2-one (**S88**).

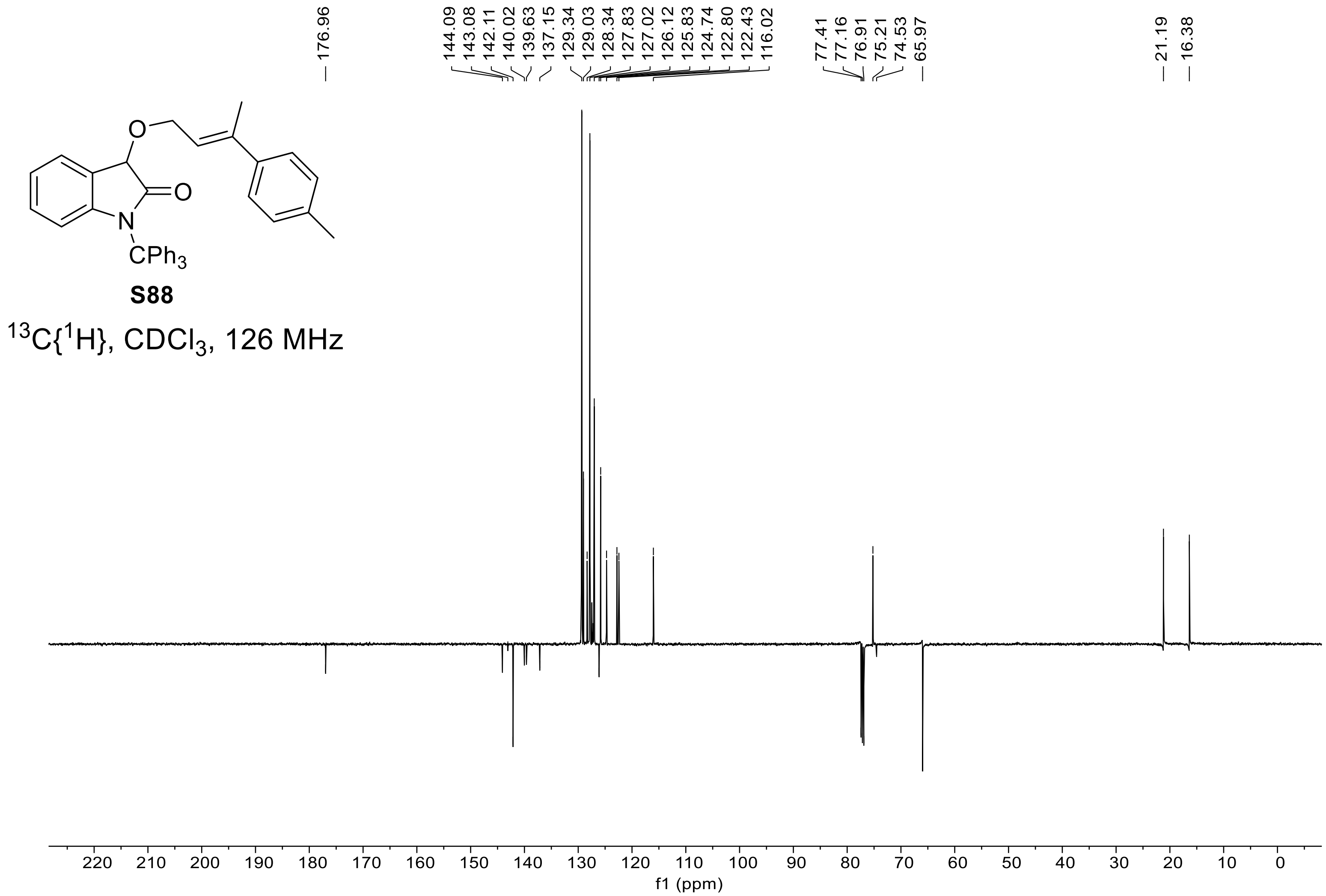

Fig. S221: $^{13}C\{^1H\}$ NMR spectrum for (*E*)-3-{[3-(*p*-Tolyl)but-2-en-1-yl]oxy}-1-tritylindolin-2-one (**S88**).

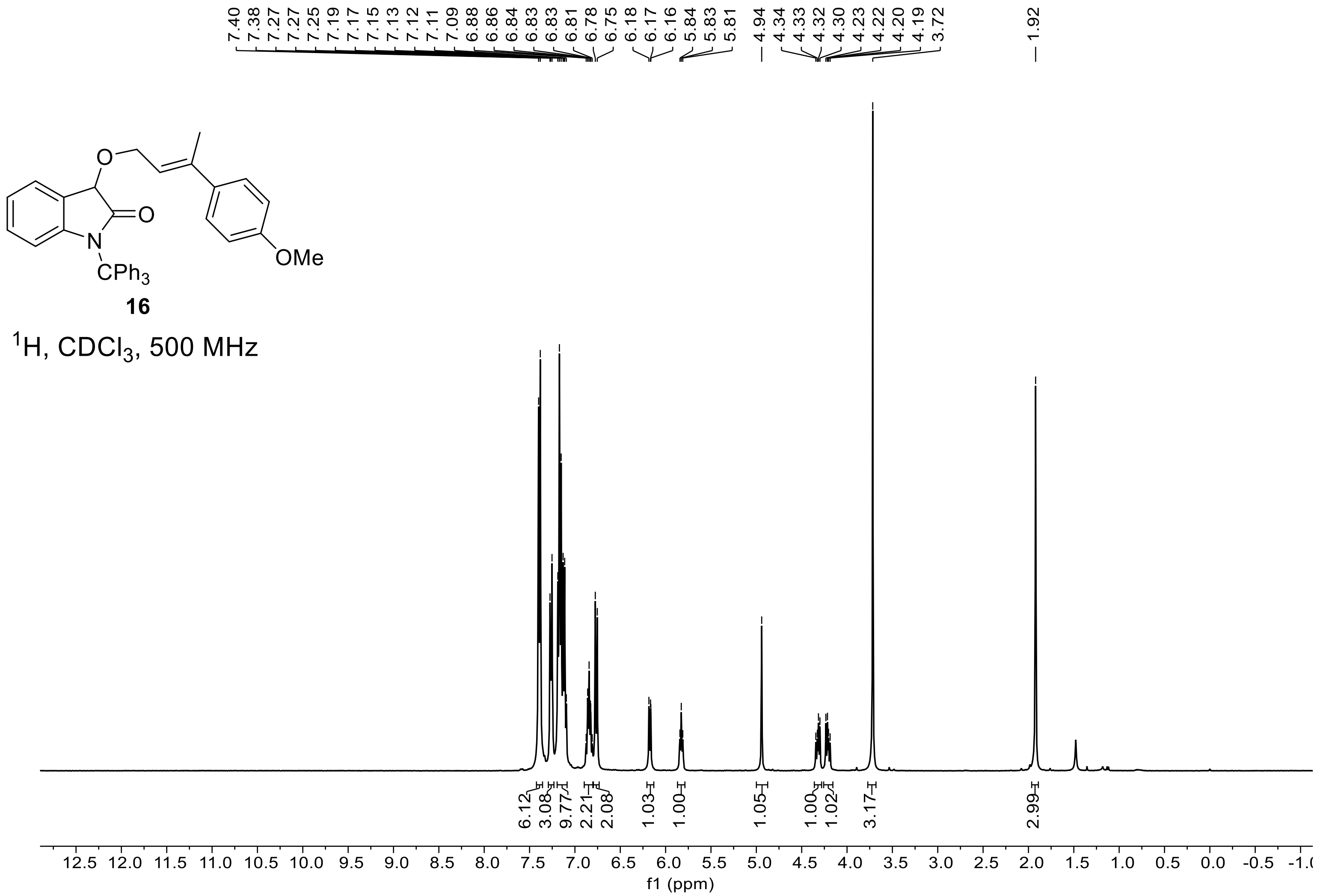

Fig. S222: ^{1}H NMR spectrum for (*E*)-3-{[3-(4-Methoxyphenyl)but-2-en-1-yl]oxy}-1-tritylindolin-2-one (**16**).

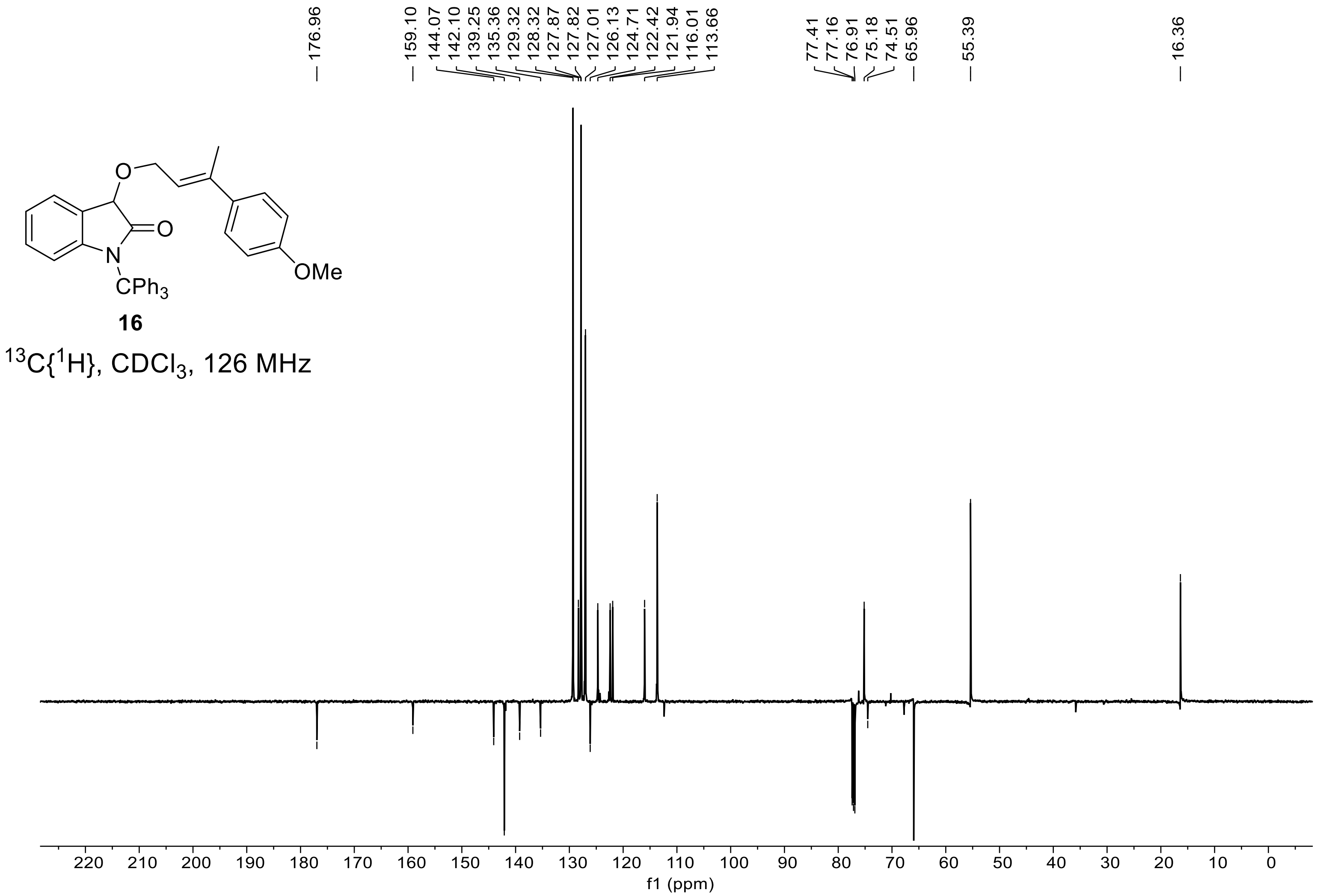

Fig. S223: $^{13}C\{^1H\}$ NMR spectrum for (*E*)-3-{[3-(4-Methoxyphenyl)but-2-en-1-yl]oxy}-1-tritylindolin-2-one (**16**).

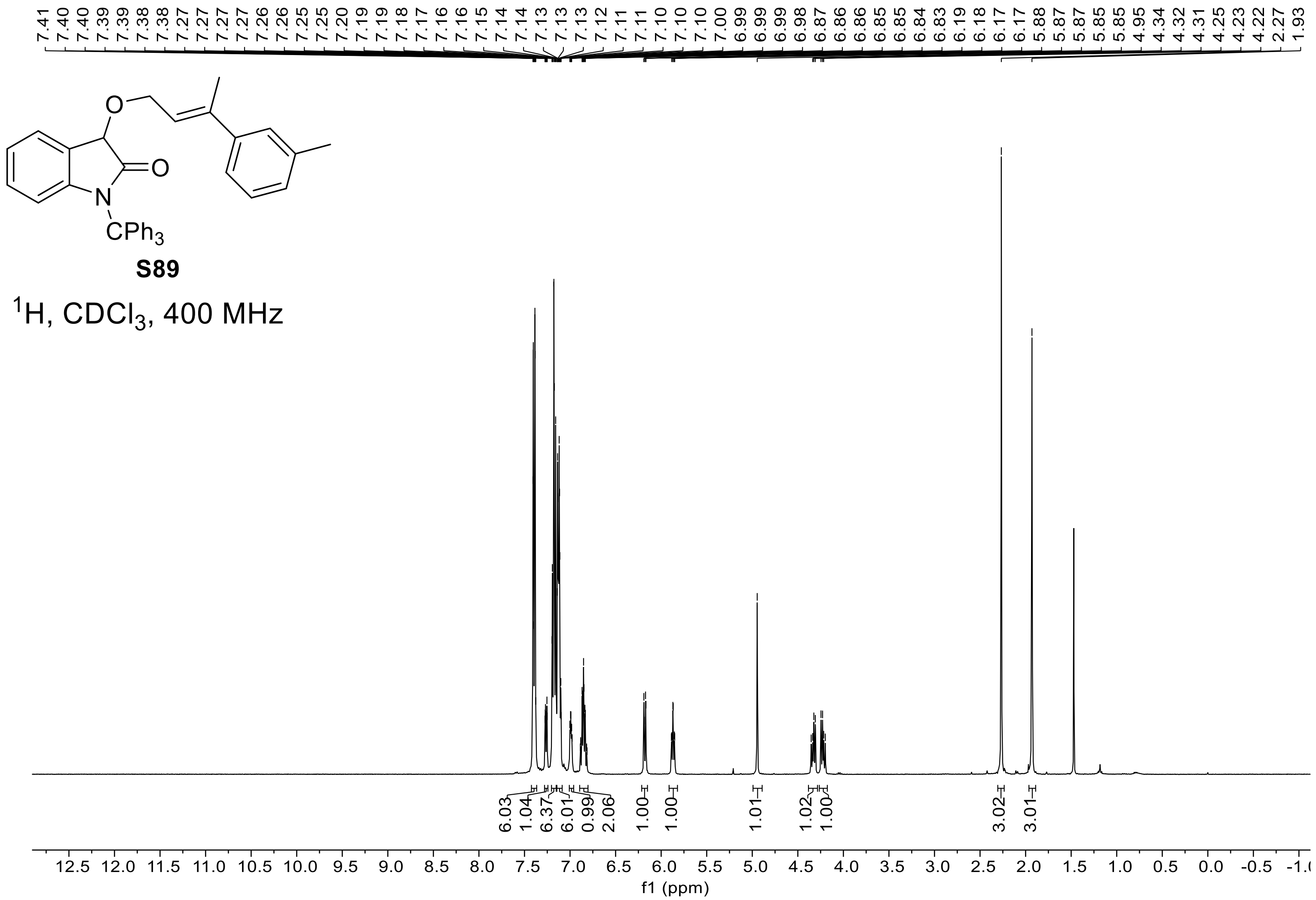

Fig. S224: ^{1}H NMR spectrum for (*E*)-3-{[3-(*m*-Tolyl)but-2-en-1-yl]oxy}-1-tritylindolin-2-one (**S89**).

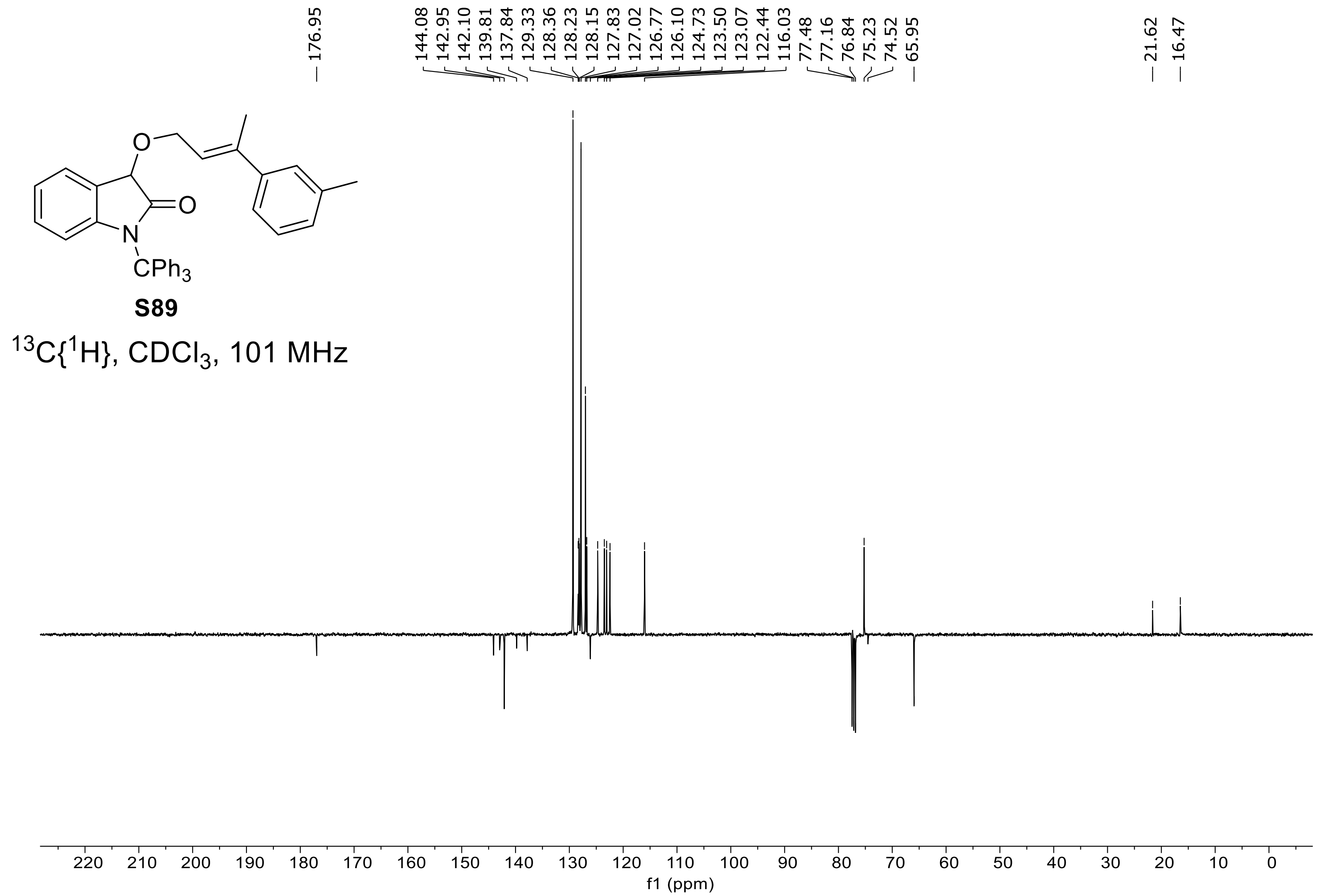

Fig. S225: $^{13}C\{^1H\}$ NMR spectrum for (*E*)-3-{[3-(*m*-Tolyl)but-2-en-1-yl]oxy}-1-tritylindolin-2-one (**S89**).

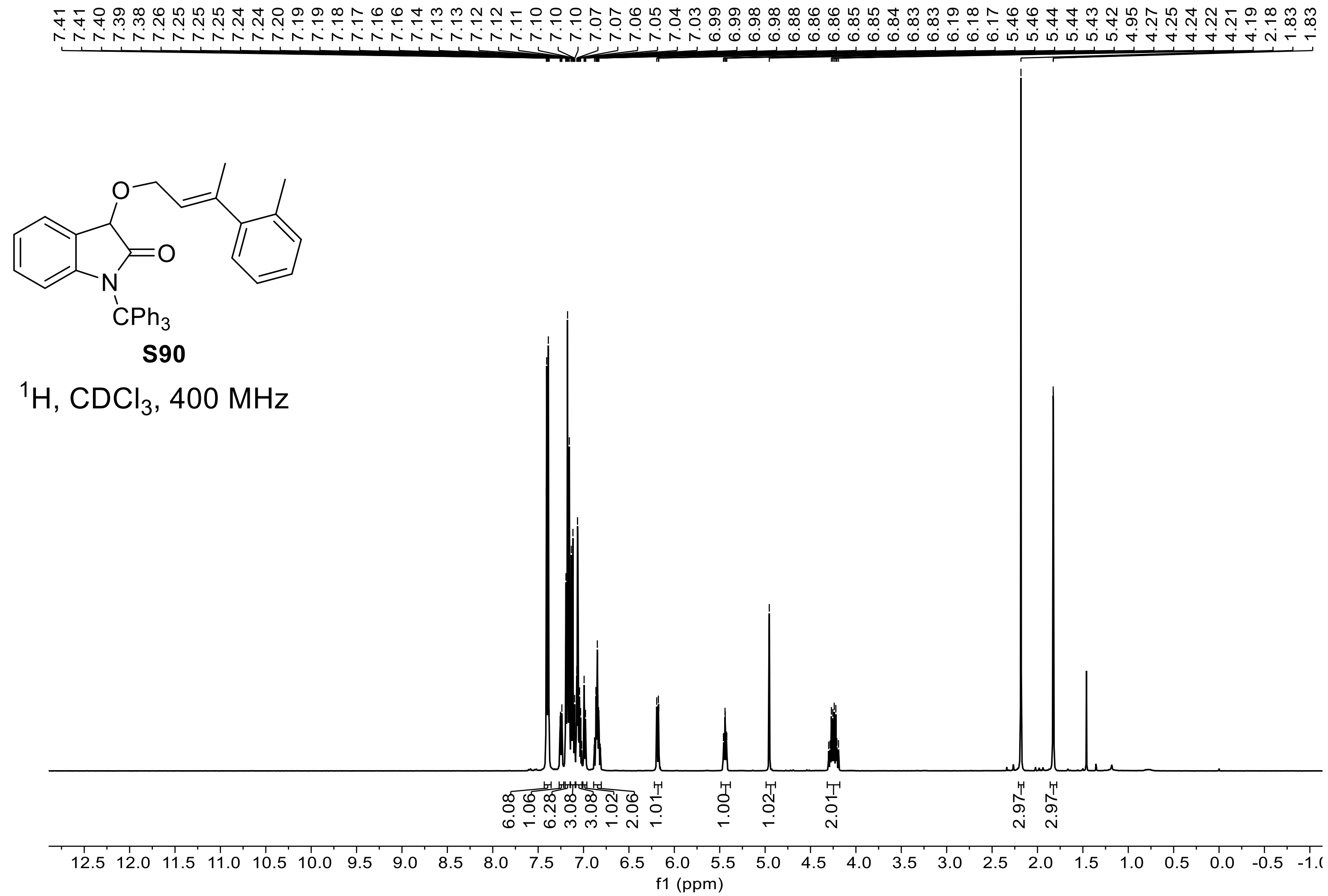

Fig. S226: ^{1}H NMR spectrum for (*E*)-3-{[3-(*o*-Tolyl)but-2-en-1-yl]oxy}-1-tritylindolin-2-one (**S90**).

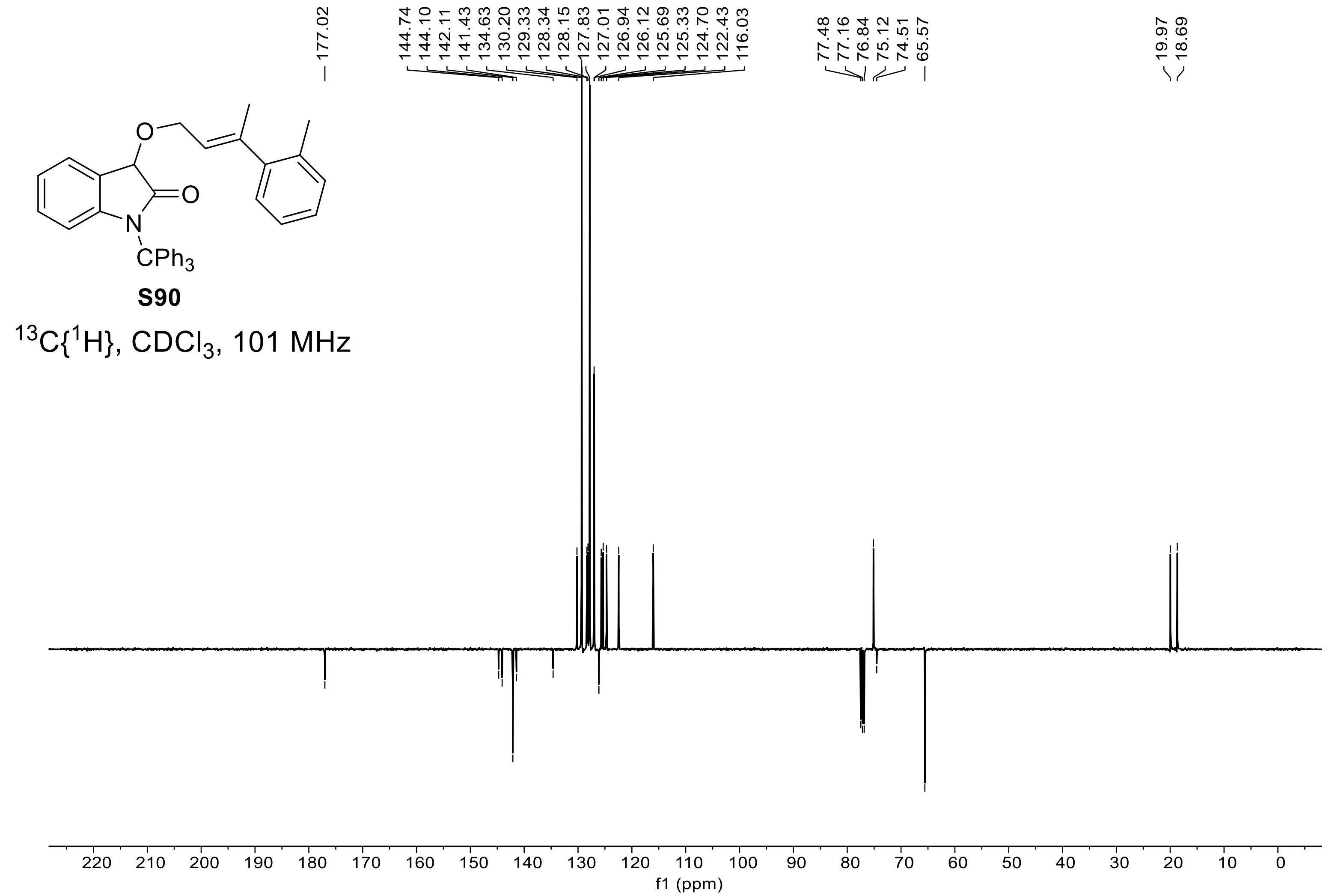

Fig. S227: ^{13}C{^{1}H} NMR spectrum for (*E*)-3-{[3-(*o*-Tolyl)but-2-en-1-yl]oxy}-1-tritylindolin-2-one (**S90**).

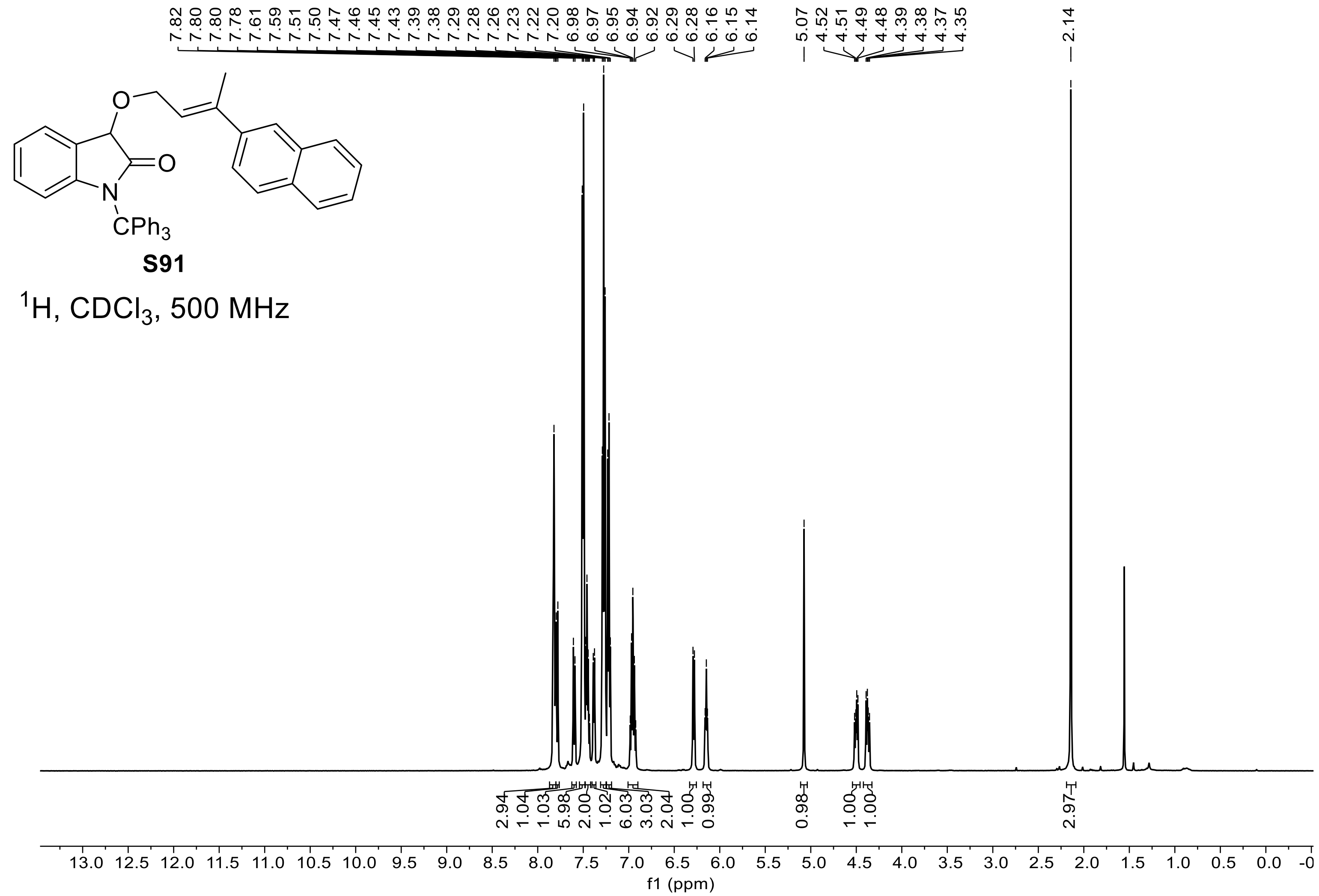

Fig. S228: ^{1}H NMR spectrum for (*E*)-3-{[3-(Naphtha-2-yl)but-2-en-1-yl]oxy}-1-tritylindolin-2-one (**S91**).

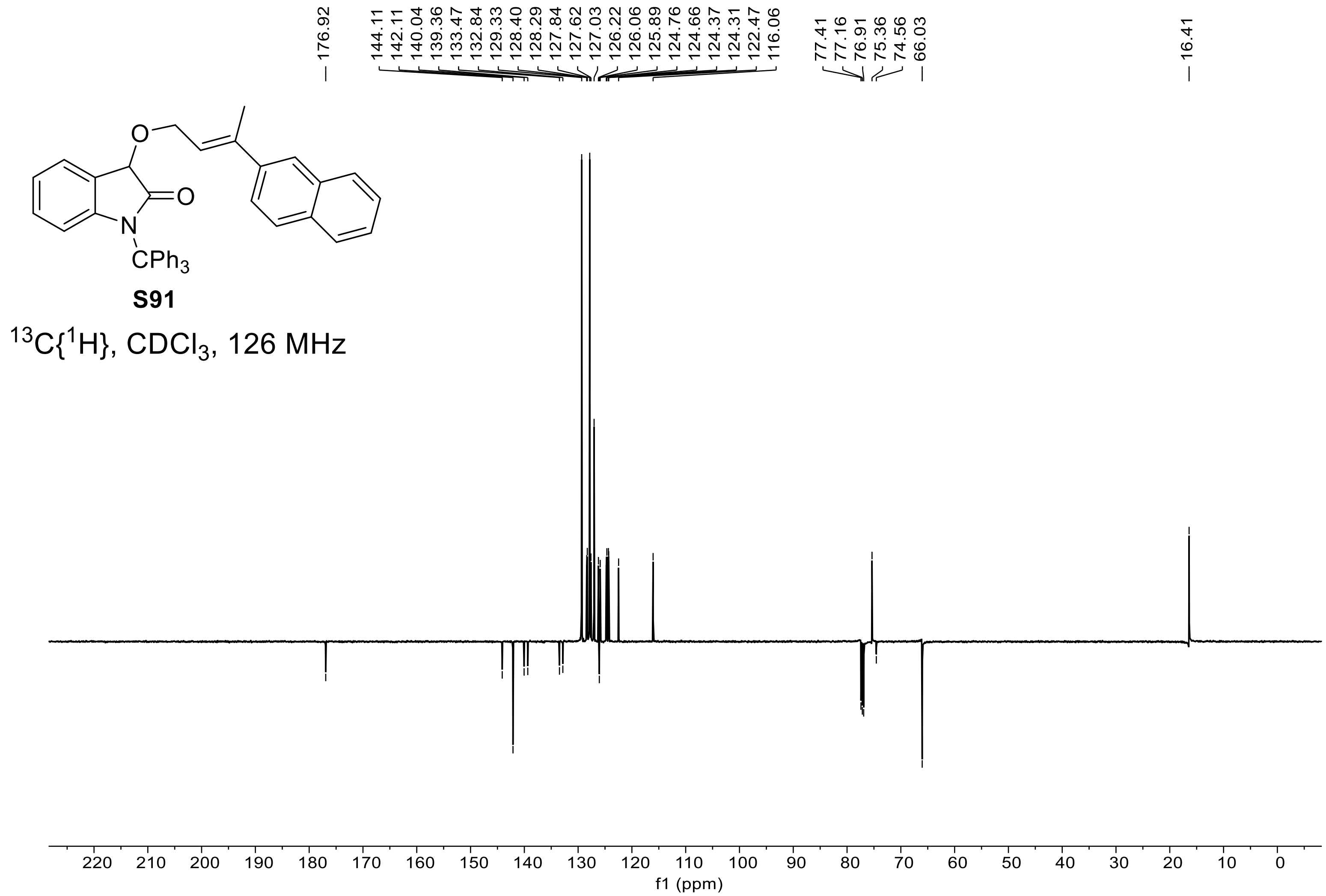

Fig. S229: $^{13}C\{^{1}H\}$ NMR spectrum for (*E*)-3-{[3-(Naphtha-2-yl)but-2-en-1-yl]oxy}-1-tritylindolin-2-one (**S91**).

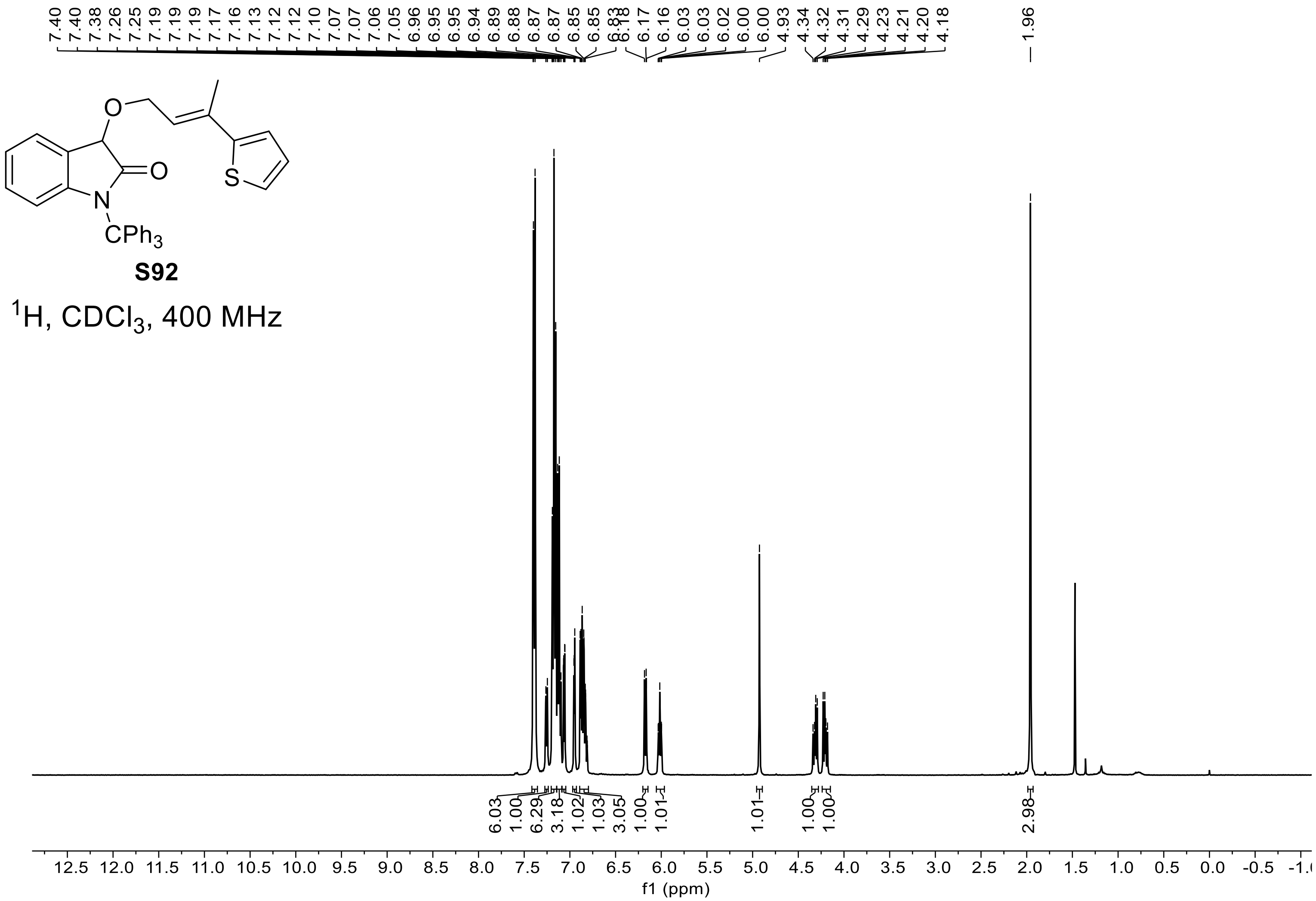

Fig. S230: ^{1}H NMR spectrum for (*E*)-3-{[3-(Thiophen-2-yl)but-2-en-1-yl]oxy}-1-tritylindolin-2-one (**S92**).

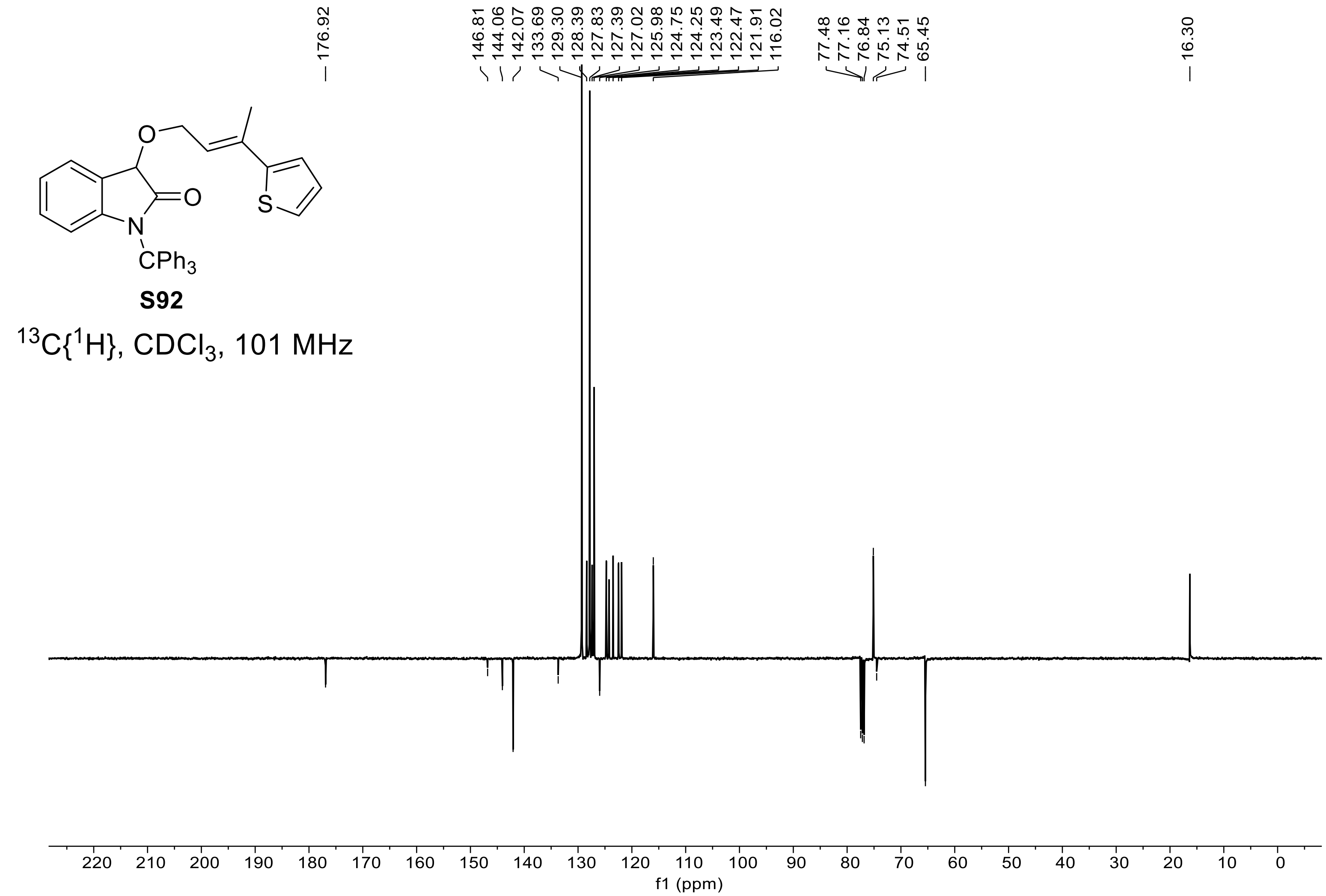

Fig. S231: $^{13}C\{^1H\}$ NMR spectrum for (*E*)-3-{[3-(Thiophen-2-yl)but-2-en-1-yl]oxy}-1-tritylindolin-2-one (**S92**).

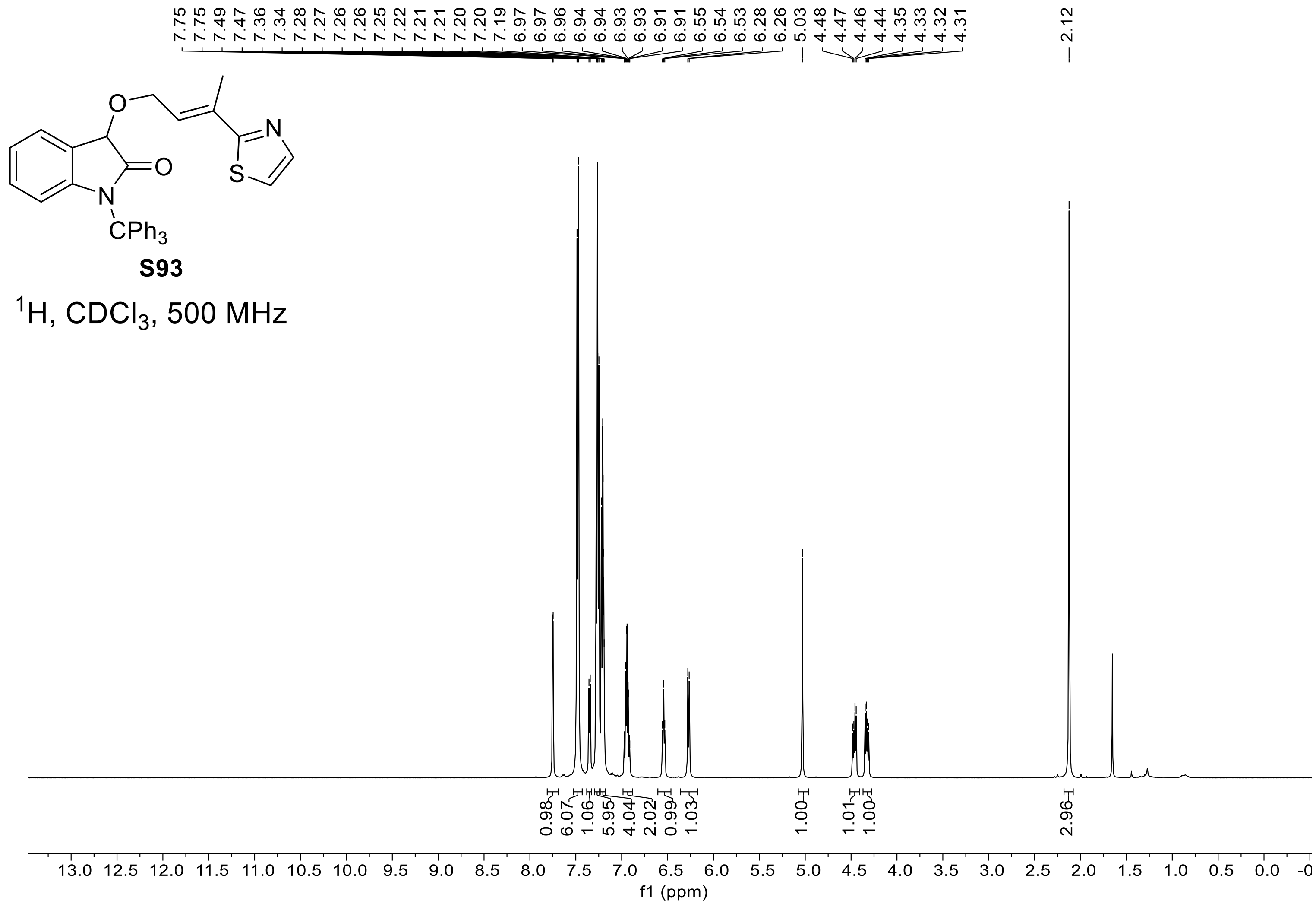

Fig. S232: ^1H NMR spectrum for (*E*)-3-{[3-(Thiazol-2-yl)but-2-en-1-yl]oxy}-1-tritylindolin-2-one (**S93**).

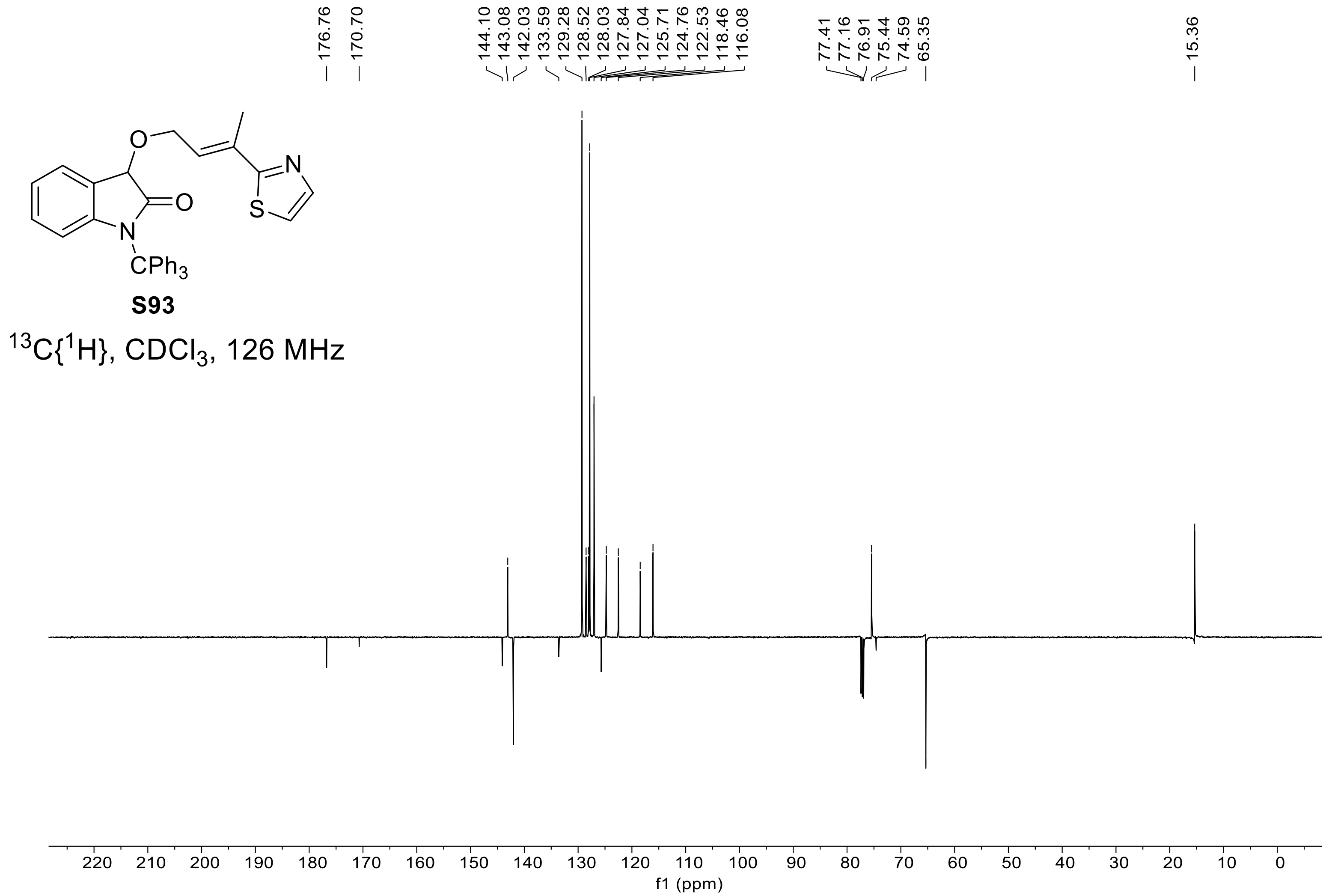

Fig. S233: $^{13}C\{^{1}H\}$ NMR spectrum for (*E*)-3-{[3-(Thiazol-2-yl)but-2-en-1-yl]oxy}-1-tritylindolin-2-one (**S93**).

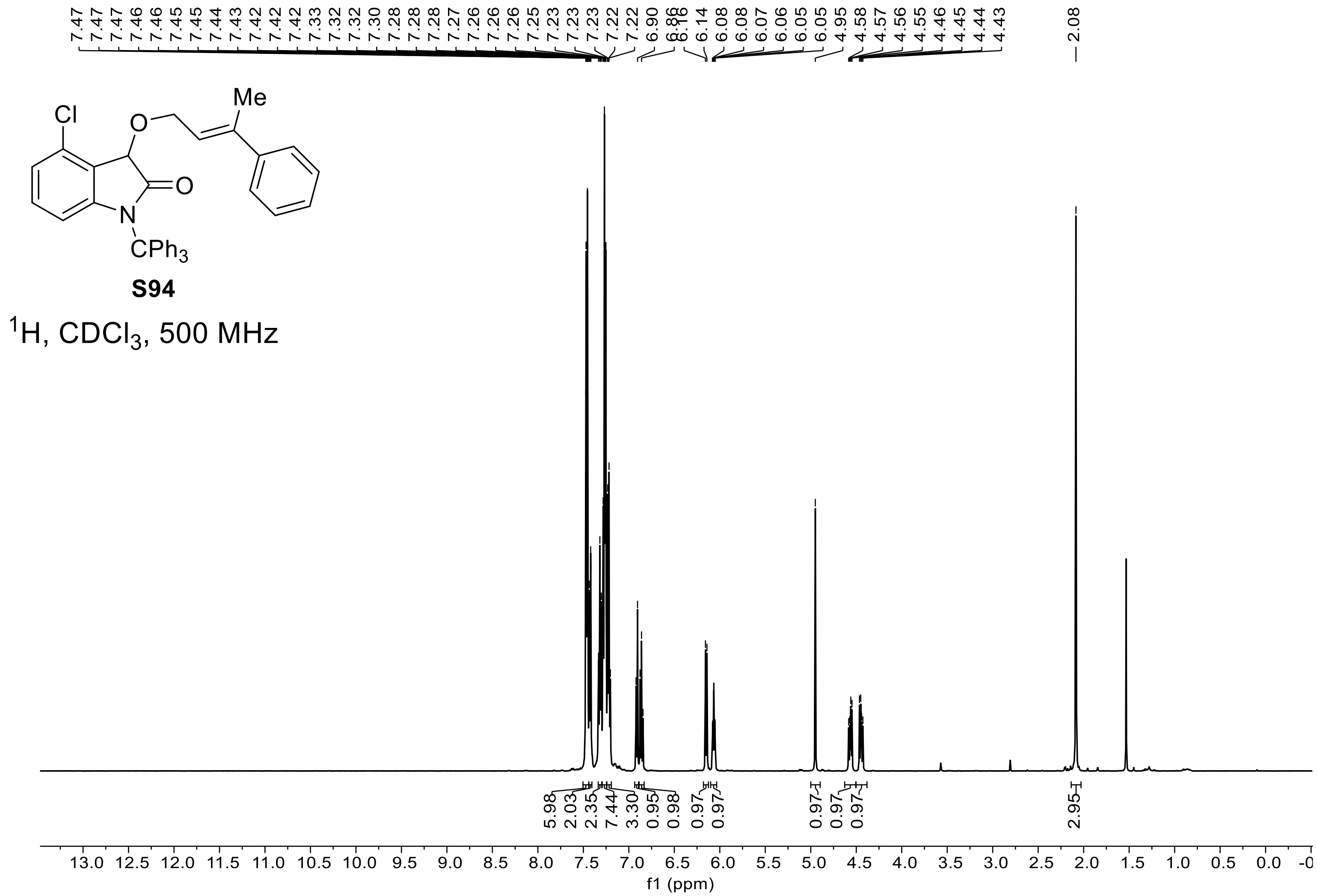

Fig. S234: ^{1}H NMR spectrum for (*E*)-4-Chloro-3-[(3-phenylbut-2-en-1-yl)oxy]-1-tritylindolin-2-one (**S94**).

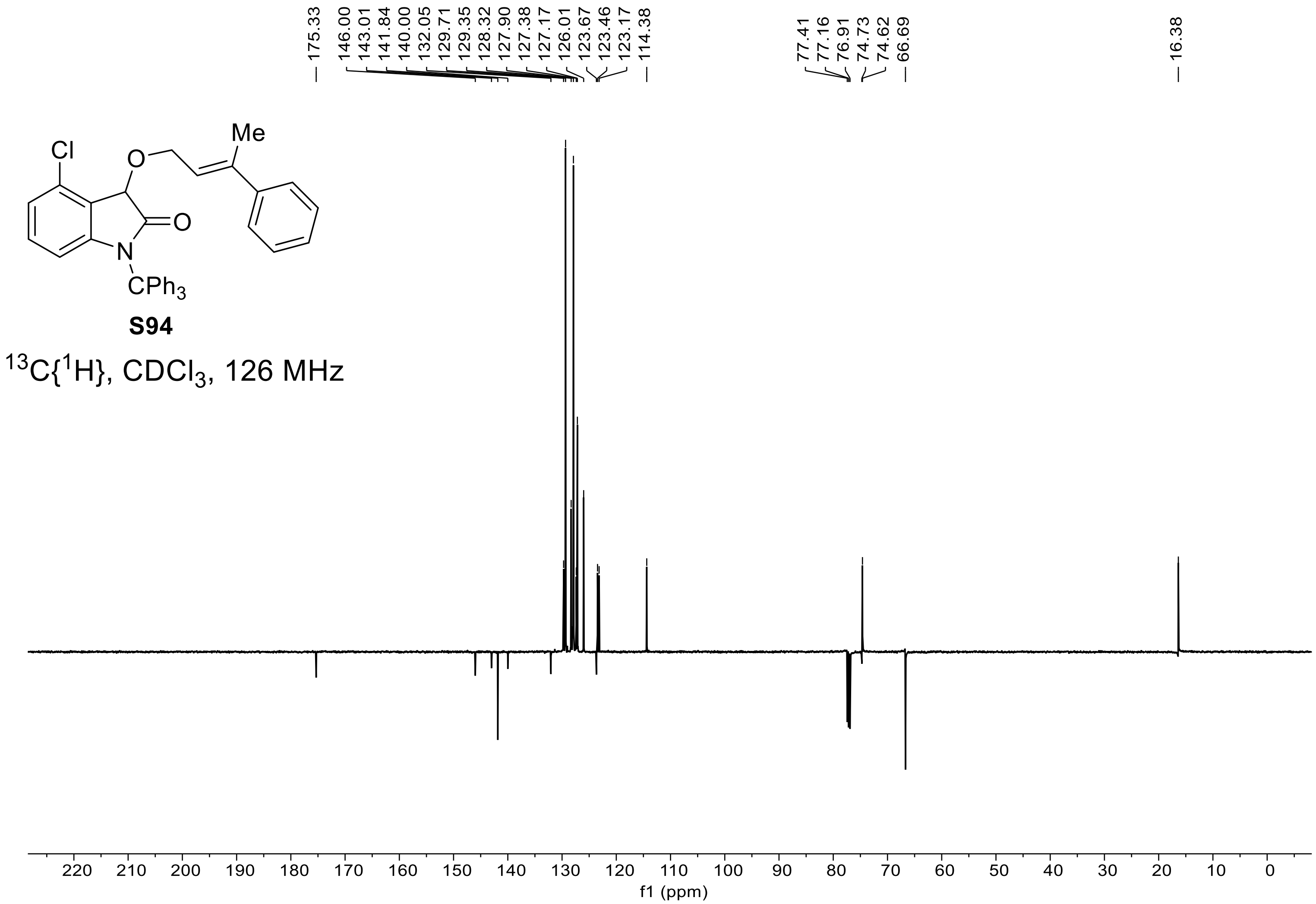

Fig. S235: $^{13}C\{^1H\}$ NMR spectrum for (*E*)-4-Chloro-3-[(3-phenylbut-2-en-1-yl)oxy]-1-tritylindolin-2-one (**S94**).

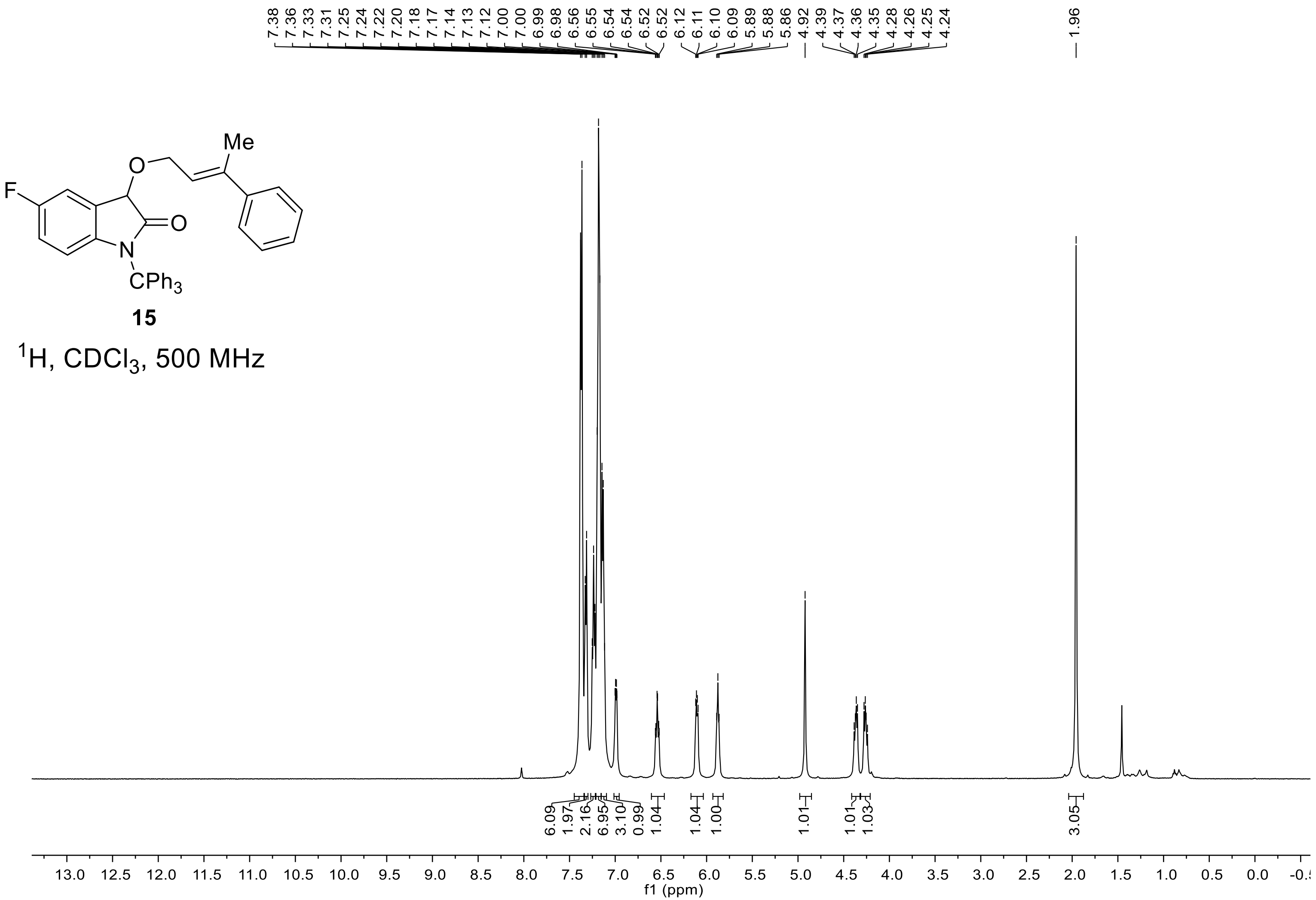

Fig. S236: ^{1}H NMR spectrum for (*E*)-5-Fluoro-3-[(3-phenylbut-2-en-1-yl)oxy]-1-tritylindolin-2-one (**15**).

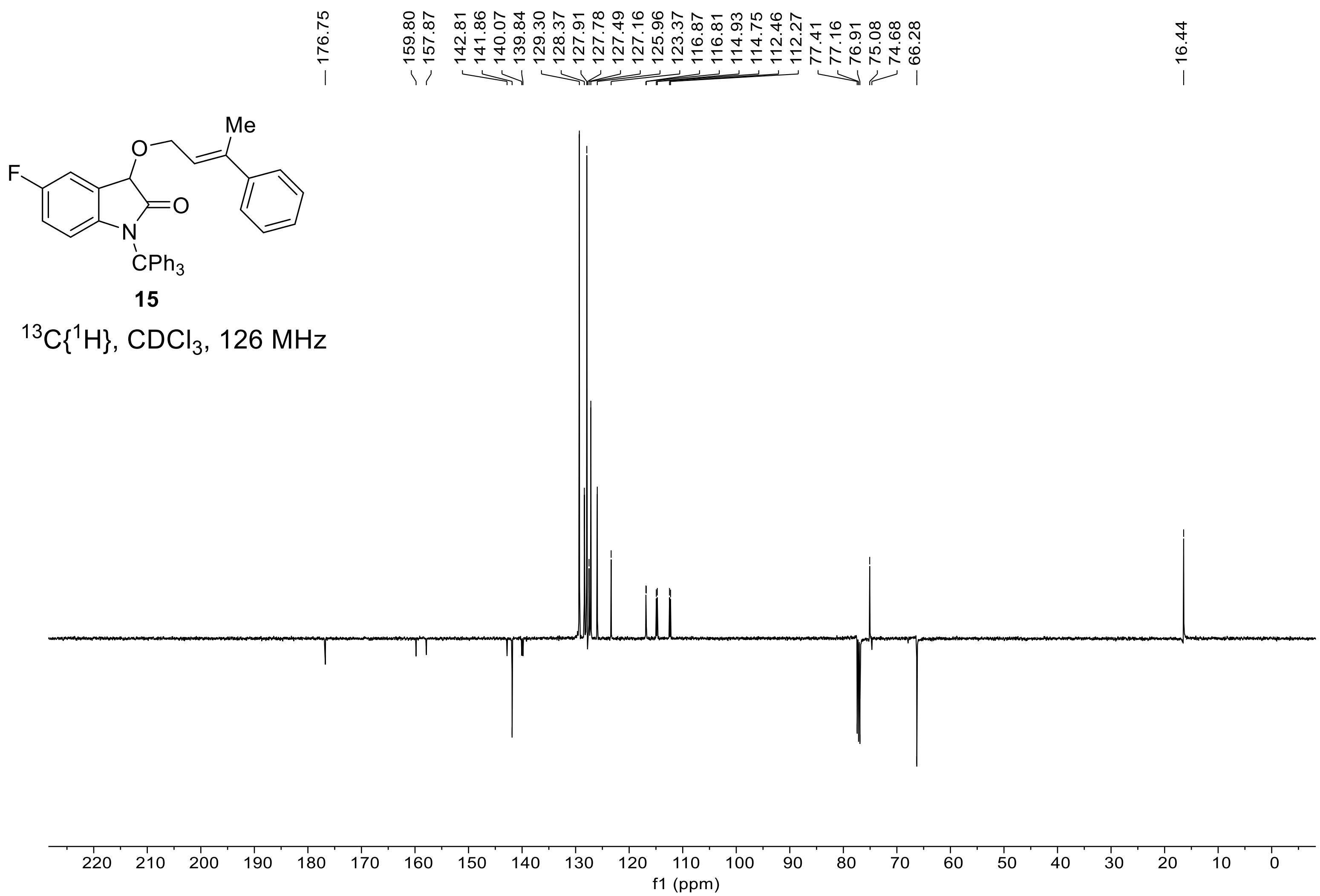

Fig. S237: $^{13}C\{^1H\}$ NMR spectrum for (*E*)-5-Fluoro-3-[(3-phenylbut-2-en-1-yl)oxy]-1-tritylindolin-2-one (**15**).

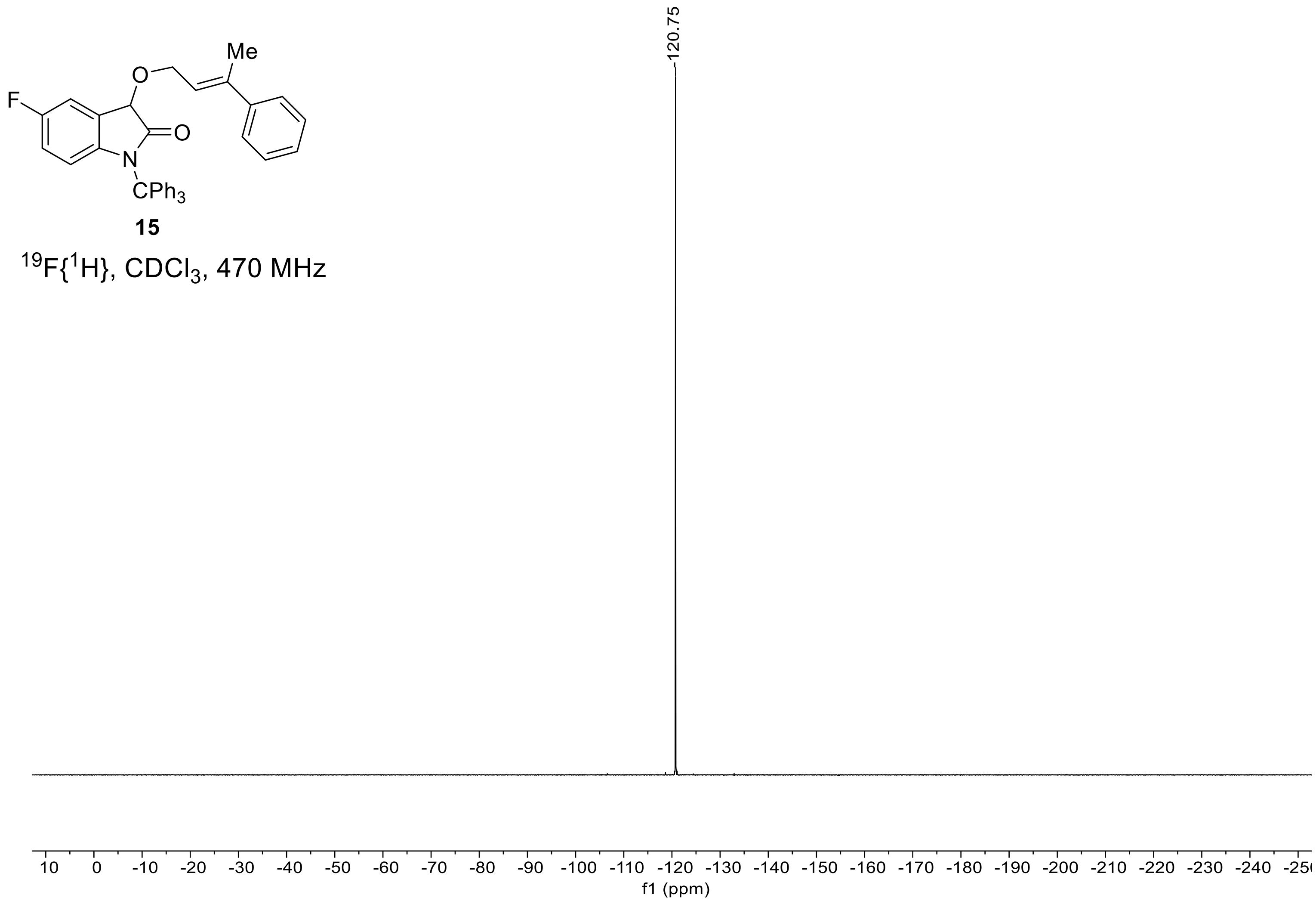

Fig. S238: $^{19}F\{^{1}H\}$ NMR spectrum for (*E*)-5-Fluoro-3-[(3-phenylbut-2-en-1-yl)oxy]-1-tritylindolin-2-one (**15**).

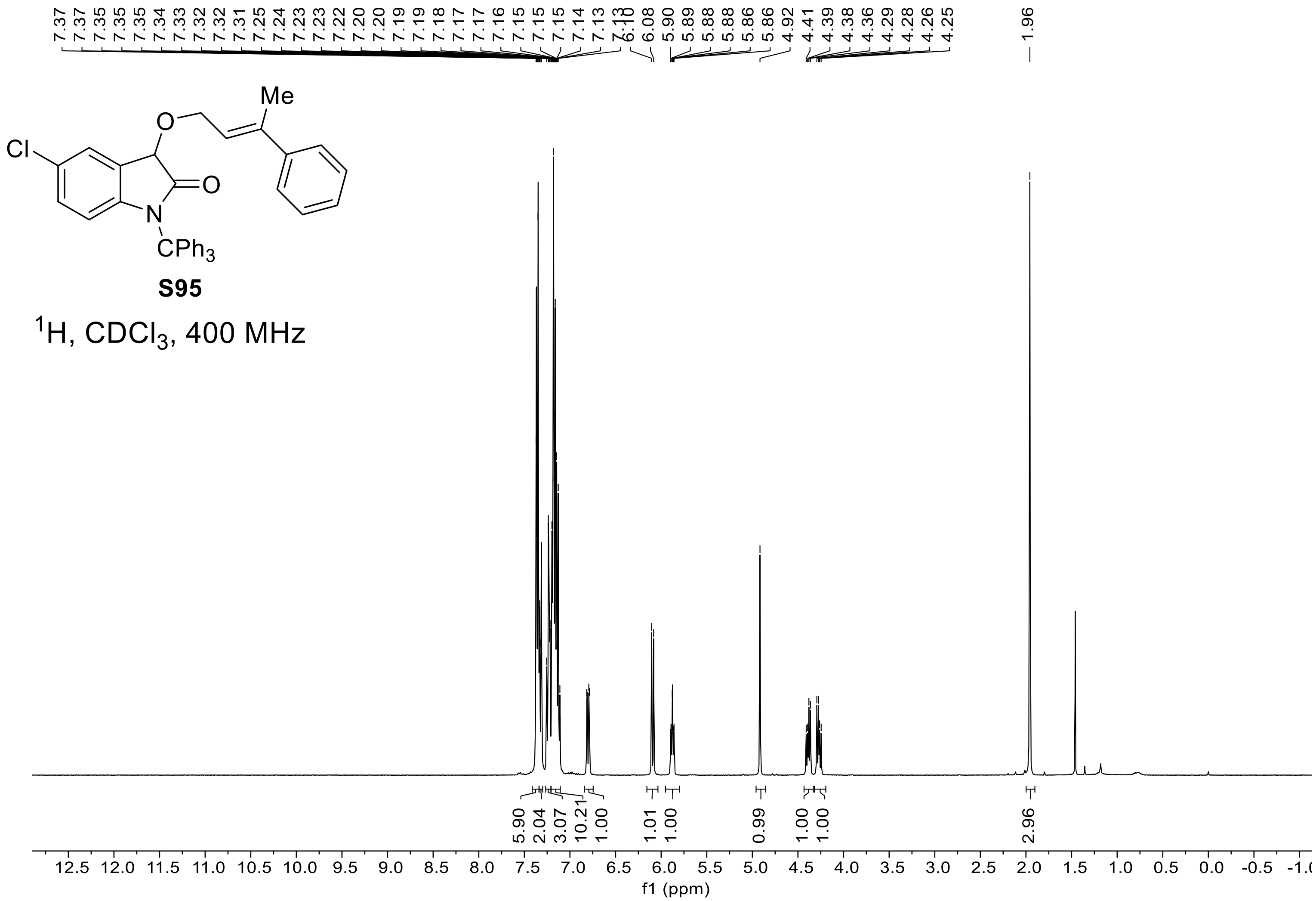

Fig. S239: ^{1}H} NMR spectrum for (*E*)-5-Chloro-3-[(3-phenylbut-2-en-1-yl)oxy]-1-tritylindolin-2-one (**S95**).

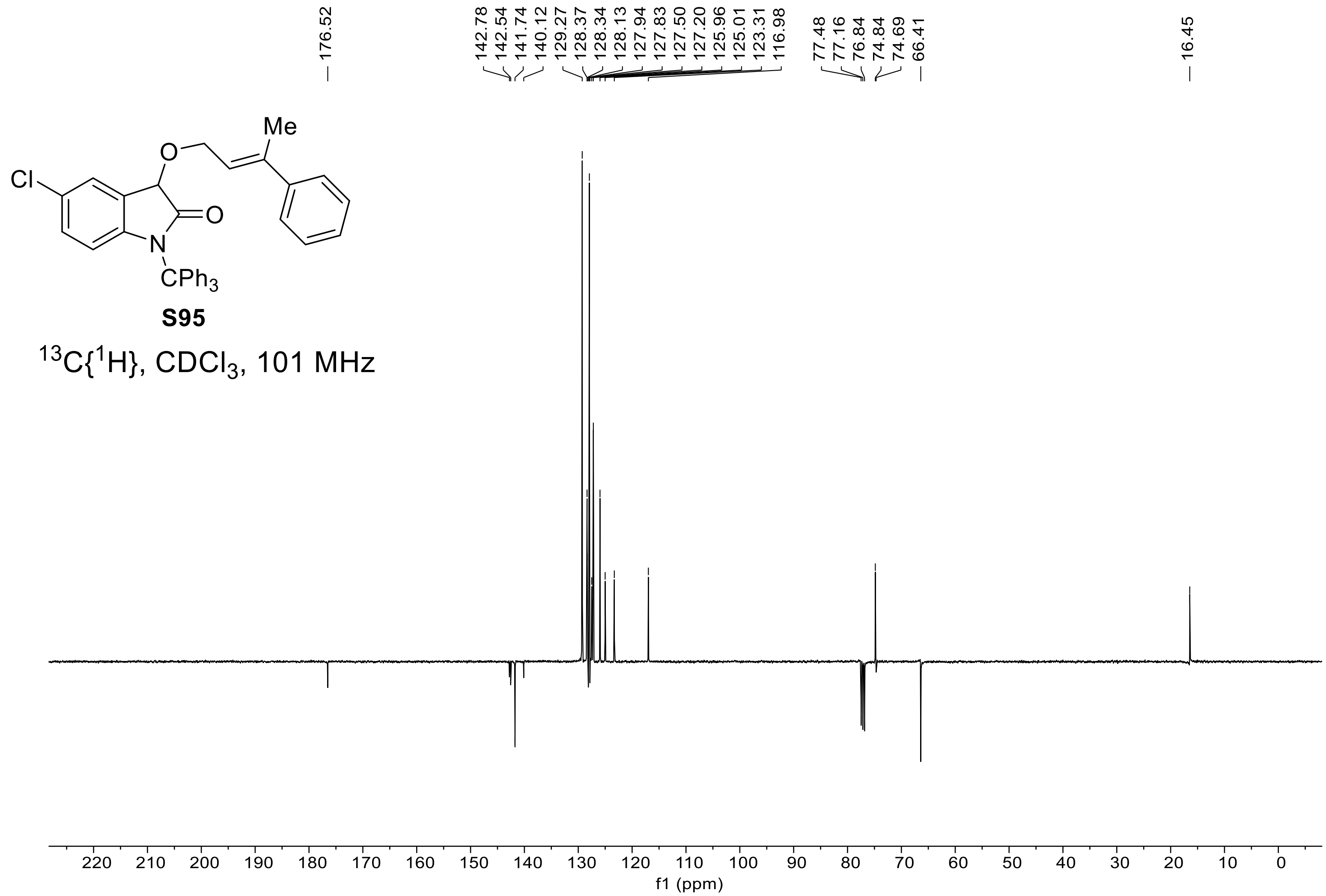

Fig. S240: $^{13}C\{^{1}H\}$ NMR spectrum for (*E*)-5-Chloro-3-[(3-phenylbut-2-en-1-yl)oxy]-1-tritylindolin-2-one (**S95**).

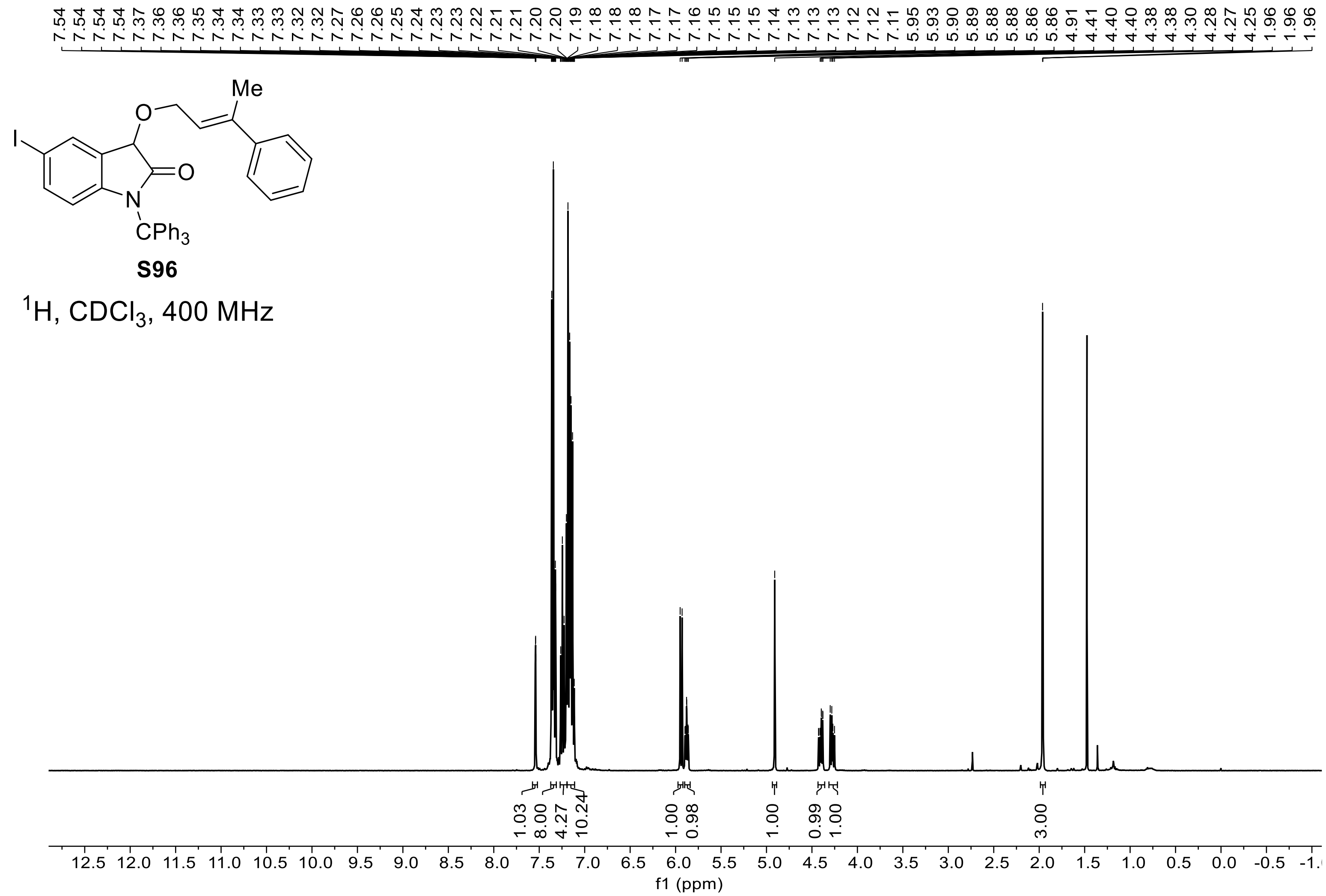

Fig. S241: ^{1}H NMR spectrum for (*E*)-5-Iodo-3-[(3-phenylbut-2-en-1-yl)oxy]-1-tritylindolin-2-one (**S96**).

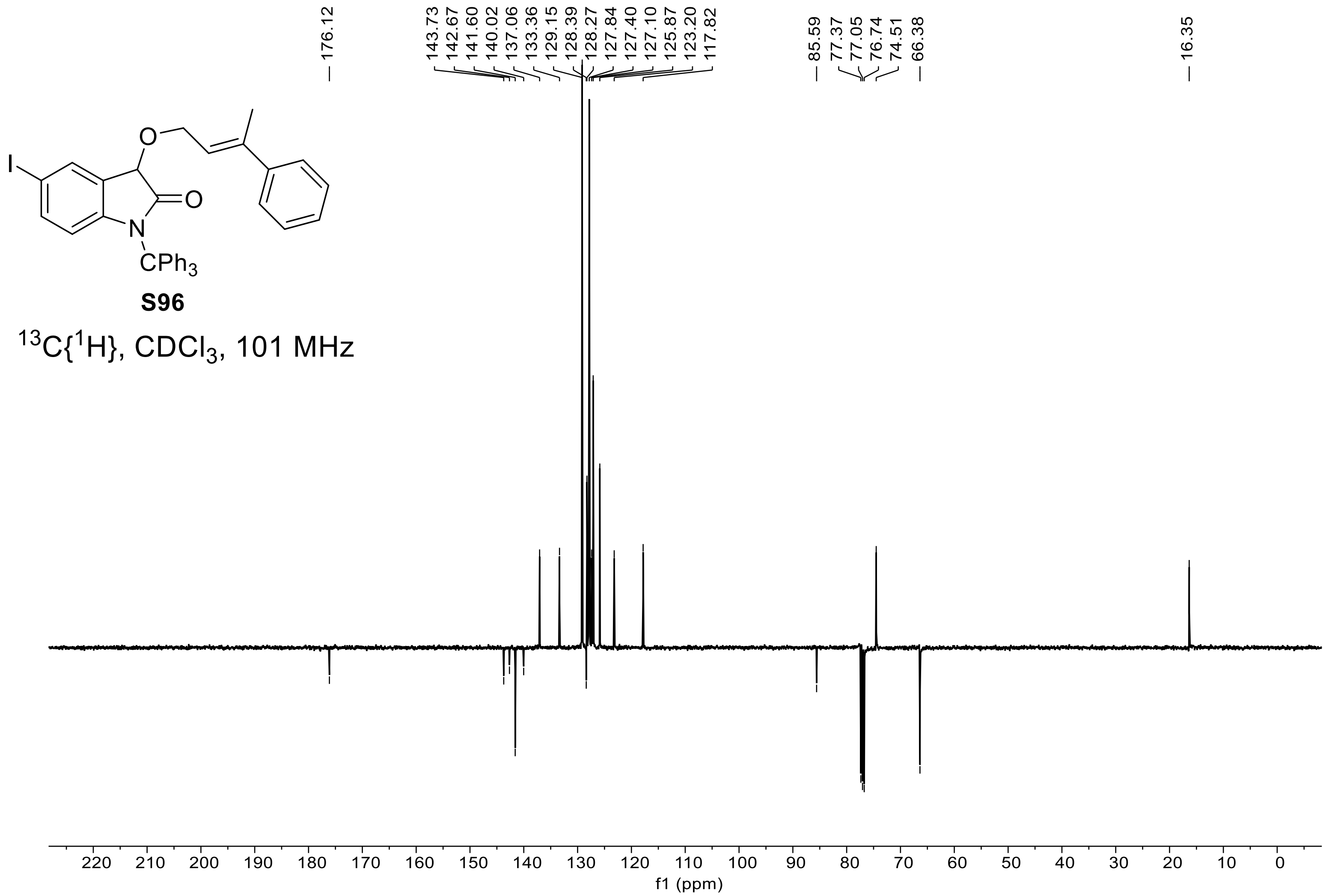

Fig. S242: $^{13}C\{^1H\}$ NMR spectrum for (*E*)-5-Iodo-3-[(3-phenylbut-2-en-1-yl)oxy]-1-tritylindolin-2-one (**S96**).

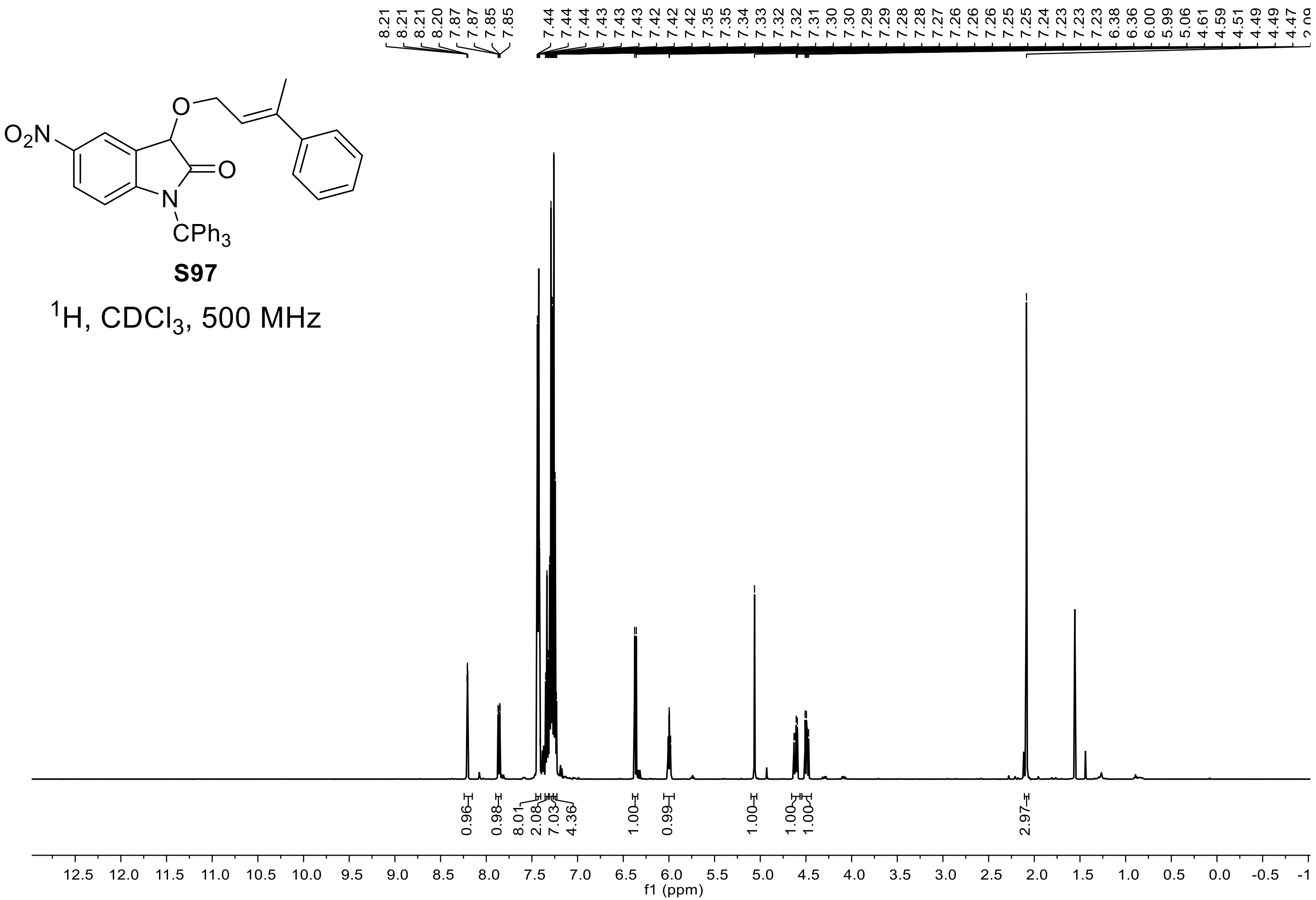

Fig. S243: ^{1}H NMR spectrum for (*E*)-5-Nitro-3-[(3-phenylbut-2-en-1-yl)oxy]-1-tritylindolin-2-one (**S97**).

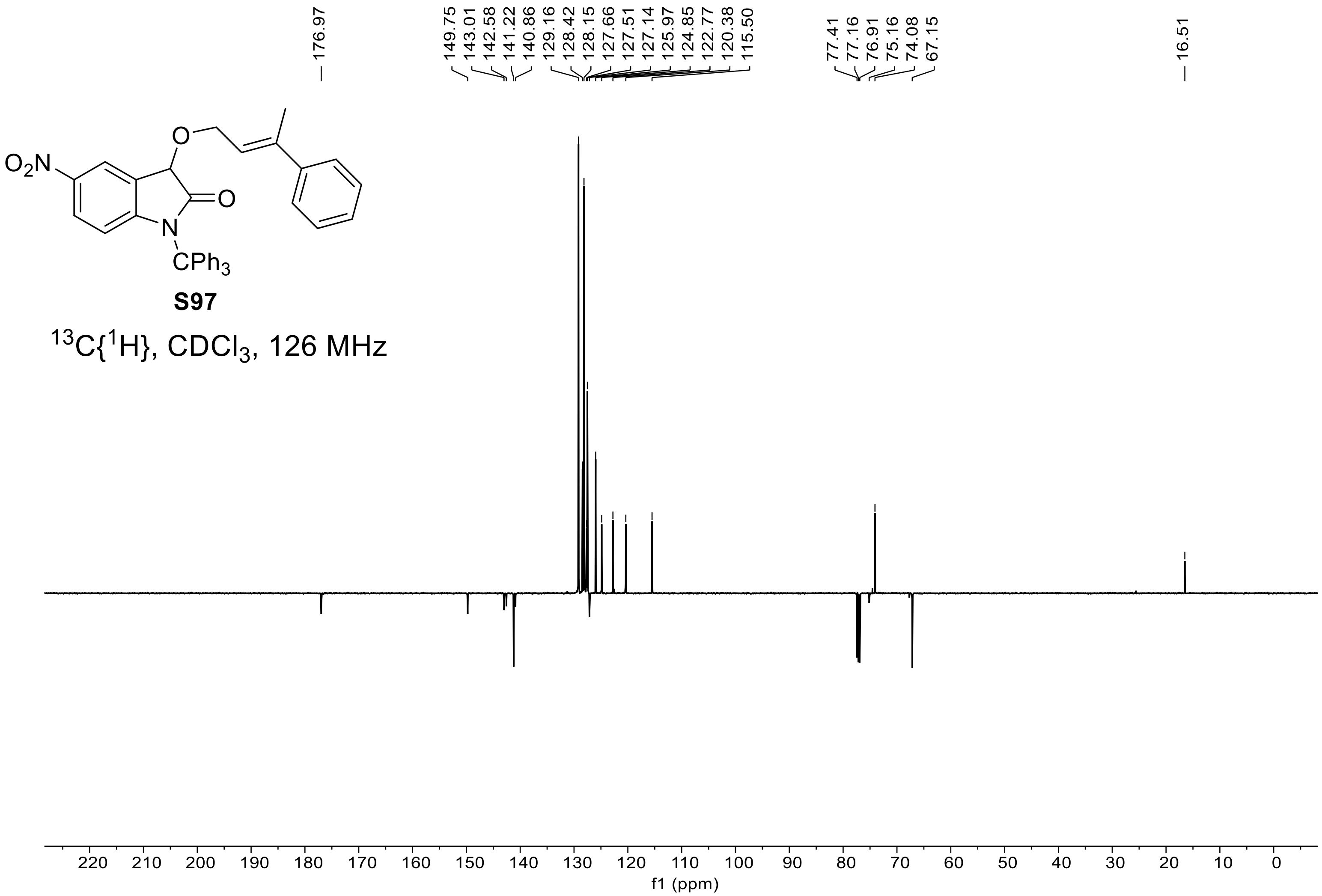

Fig. S244: $^{13}C\{^1H\}$ NMR spectrum for (*E*)-5-Nitro-3-[(3-phenylbut-2-en-1-yl)oxy]-1-tritylindolin-2-one (**S97**).

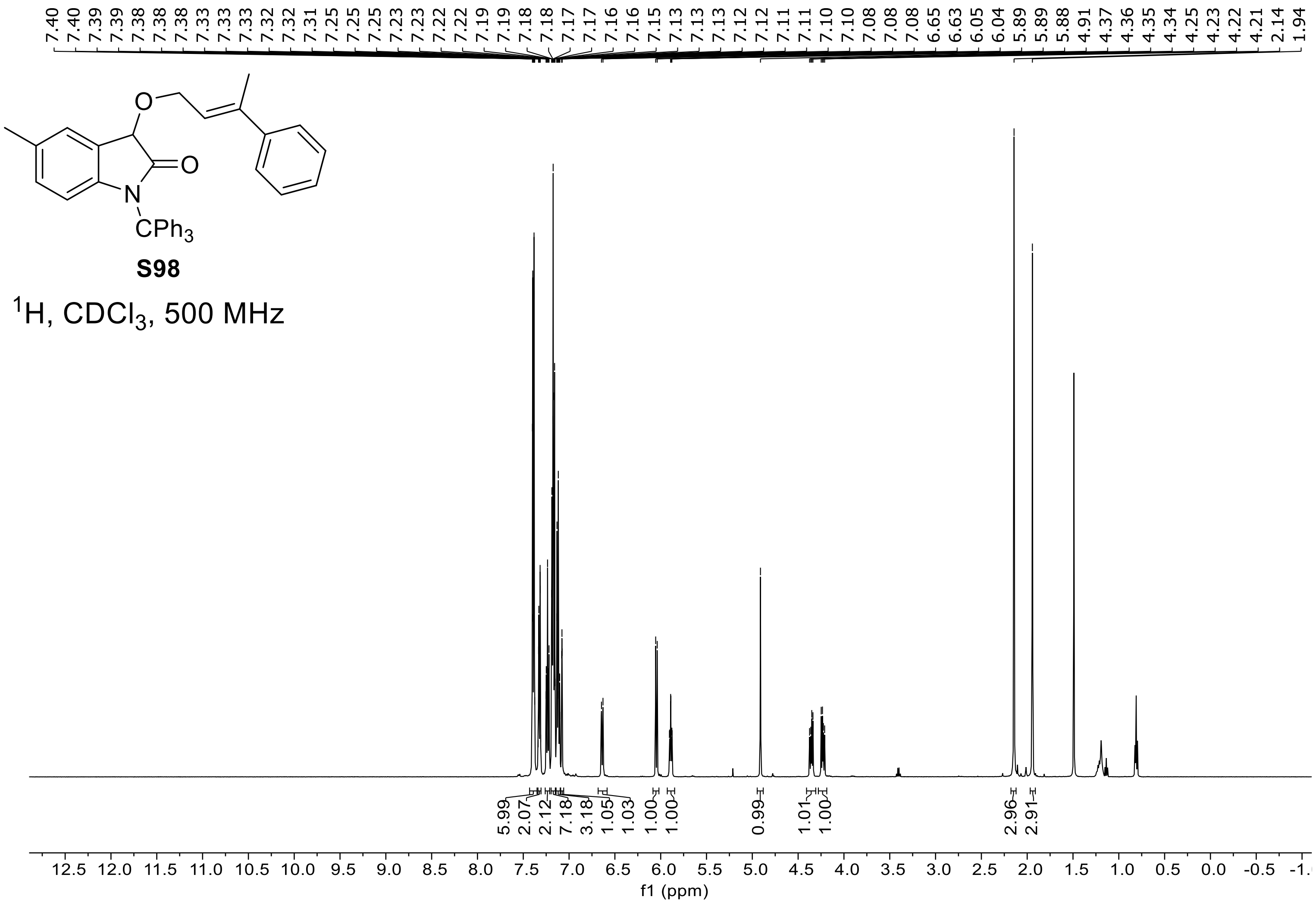

Fig. S245: ^{1}H NMR spectrum for (*E*)-5-Methyl-3-[(3-phenylbut-2-en-1-yl)oxy]-1-tritylindolin-2-one (**S98**).

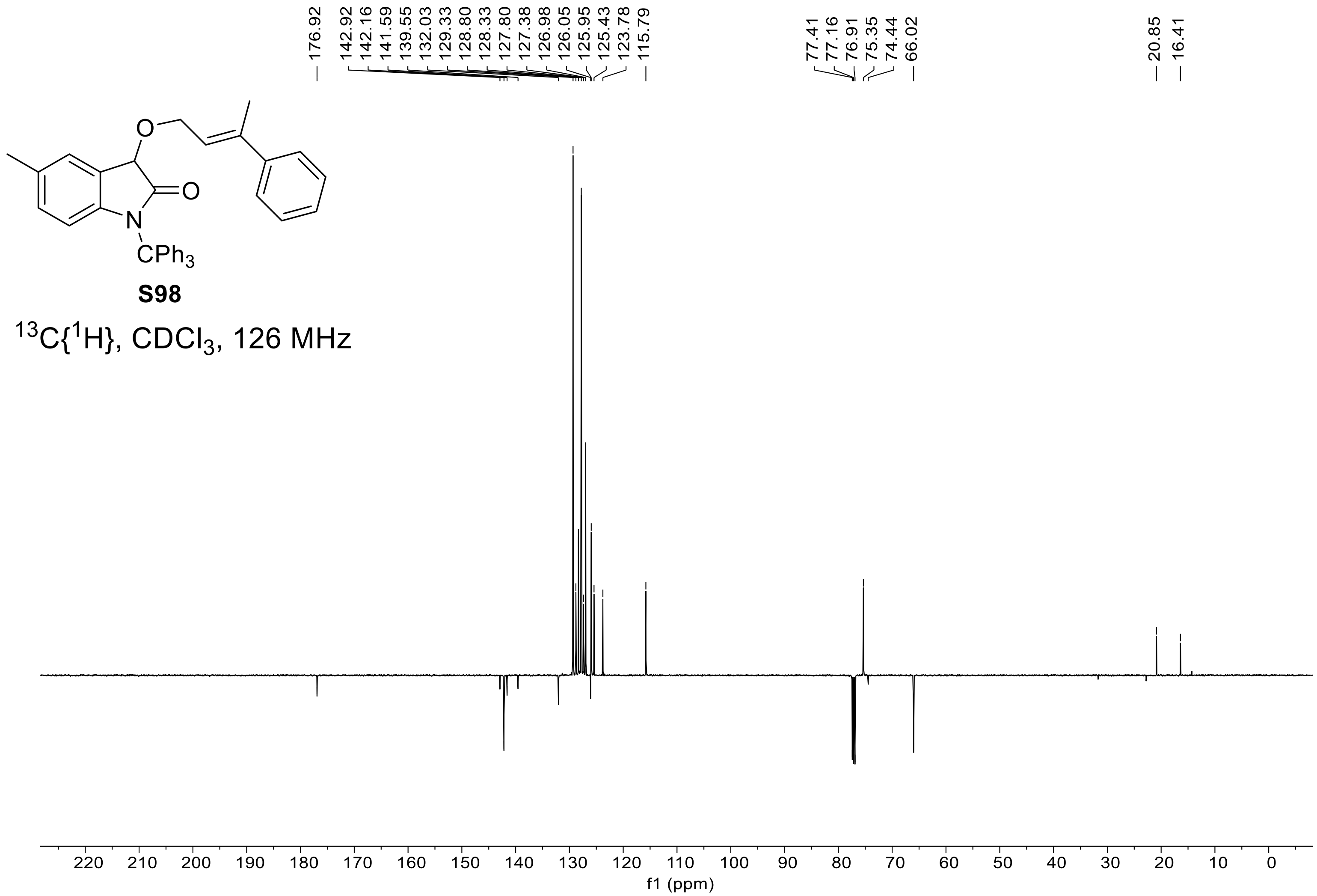

Fig. S246: ^{13}C{^{1}H} NMR spectrum for (*E*)-5-Methyl-3-[(3-phenylbut-2-en-1-yl)oxy]-1-tritylindolin-2-one (**S98**).

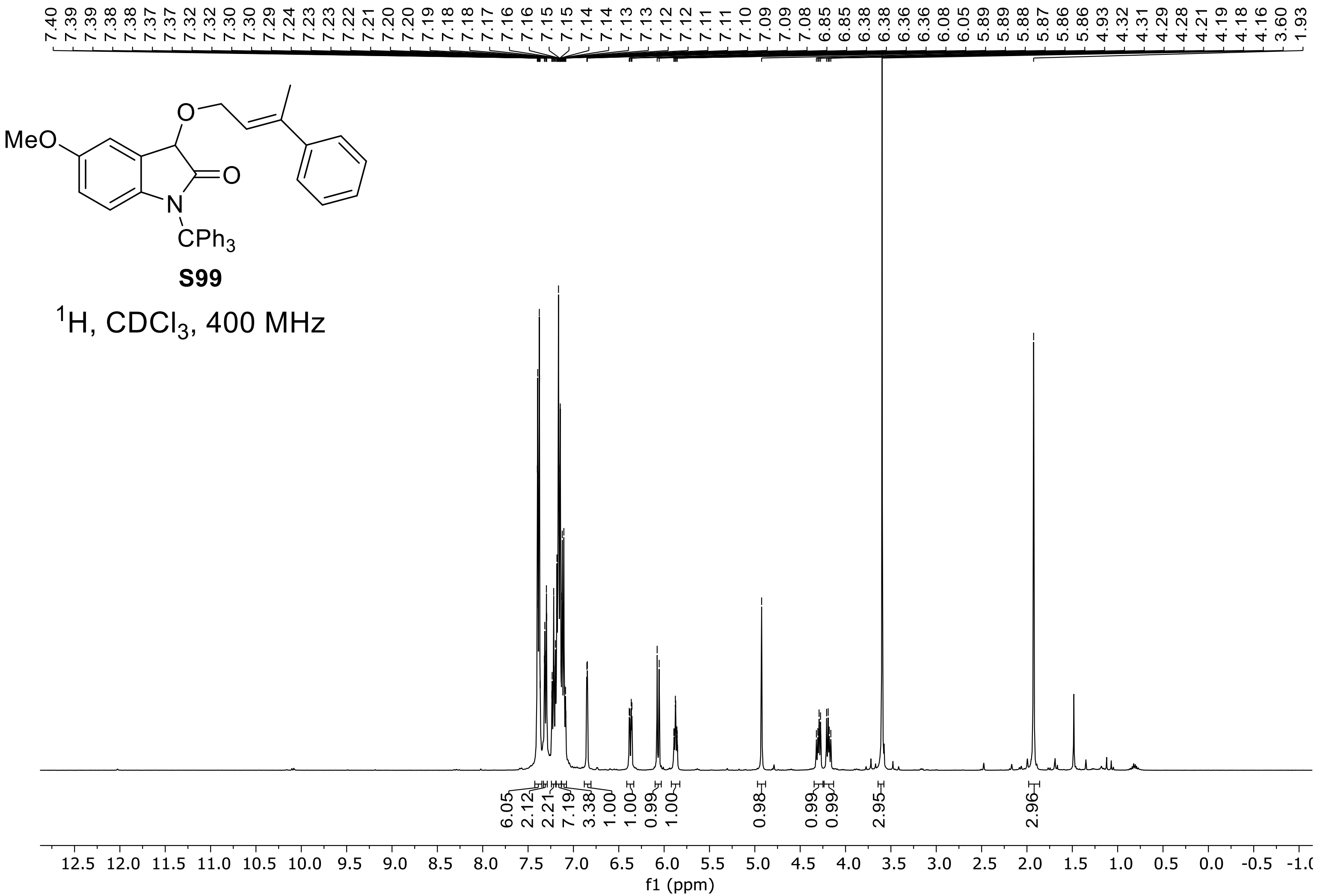

Fig. S247: ^{1}H NMR spectrum for (*E*)-5-Methoxy-3-[(3-phenylbut-2-en-1-yl)oxy]-1-tritylindolin-2-one (**S99**).

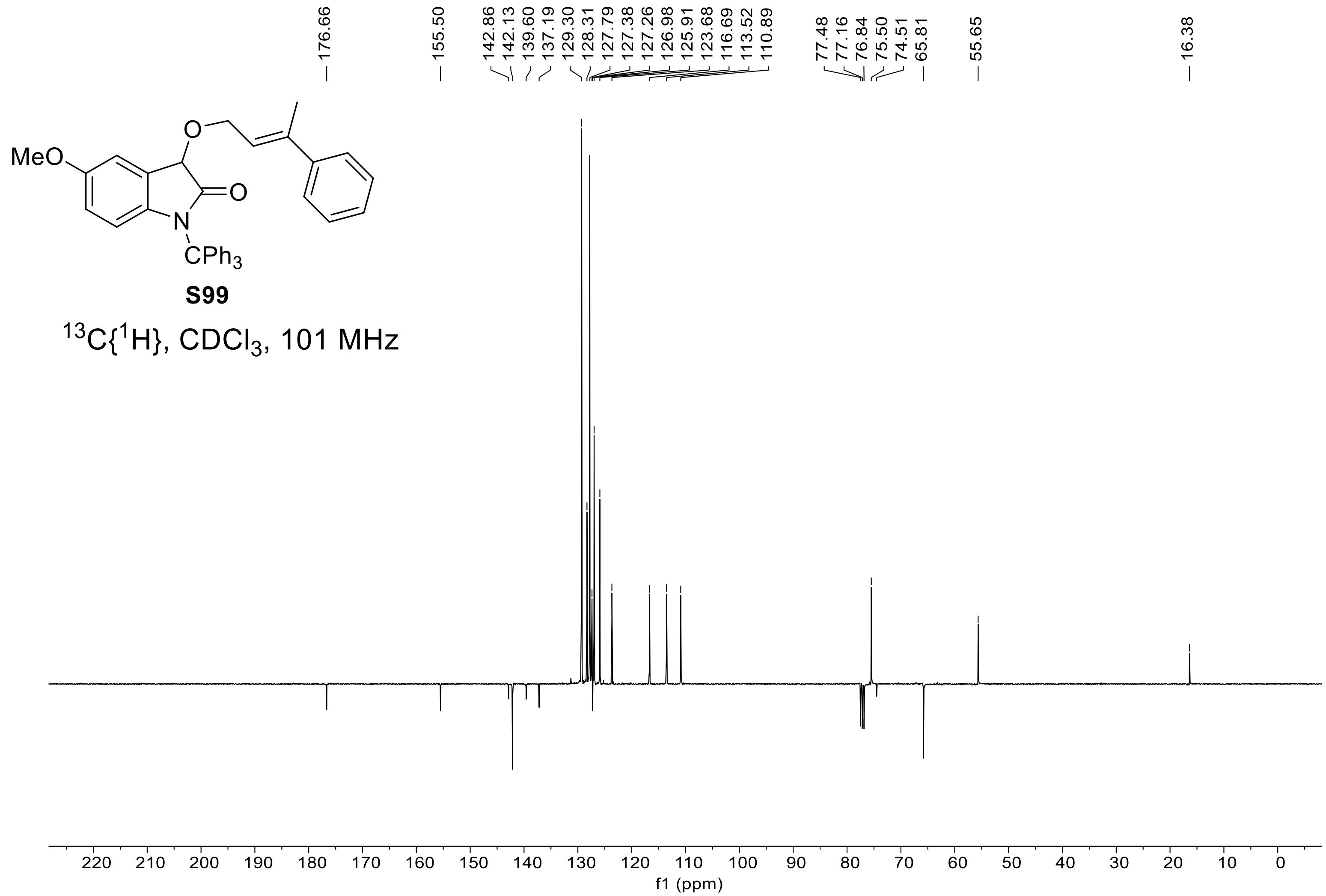

Fig. S248: $^{13}C\{^{1}H\}$ NMR spectrum for (*E*)-5-Methoxy-3-[(3-phenylbut-2-en-1-yl)oxy]-1-tritylindolin-2-one (**S99**).

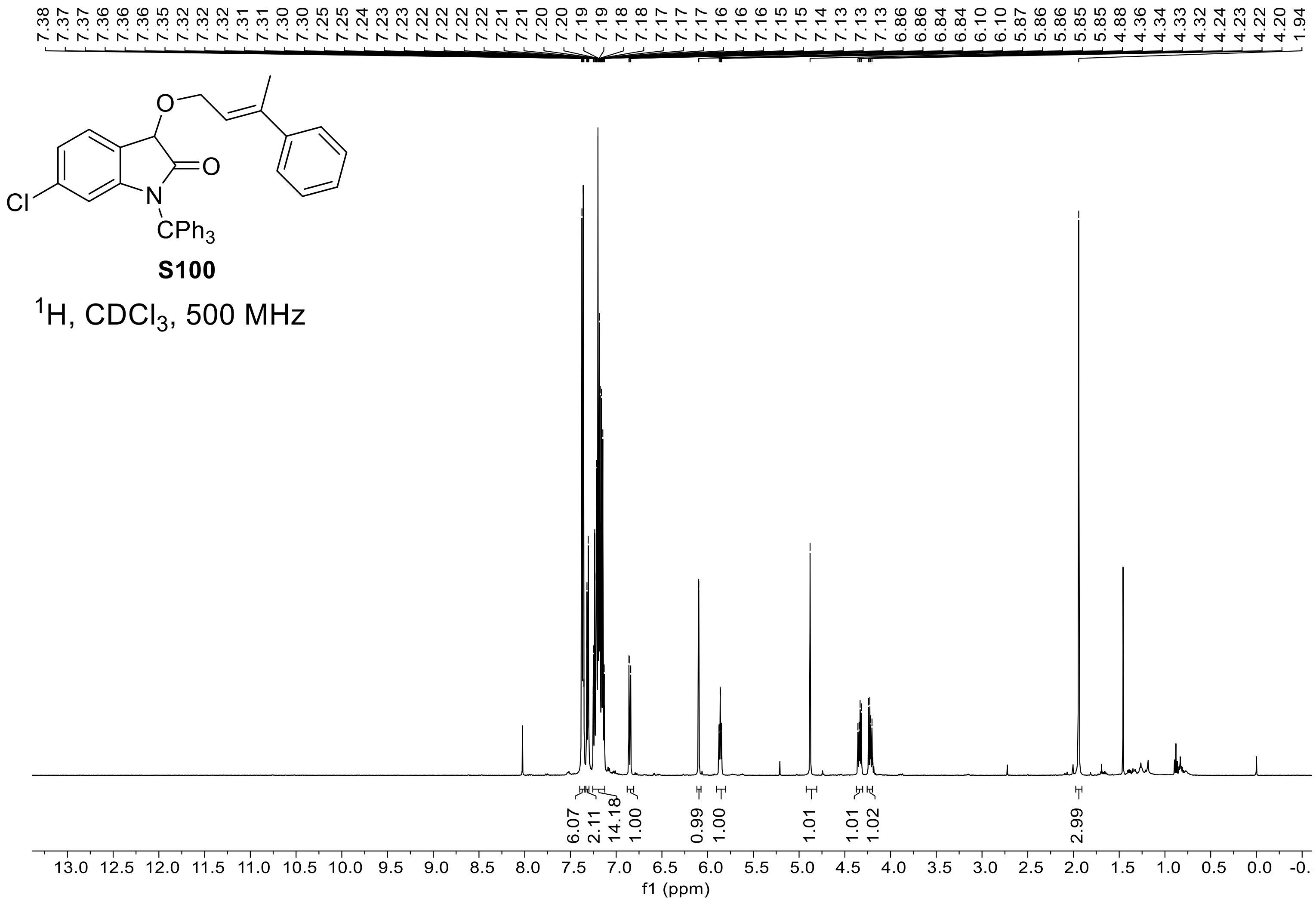

Fig. S249: ^{13}C{^{1}H} NMR spectrum for (*E*)-6-Chloro-3-[(3-phenylbut-2-en-1-yl)oxy]-1-tritylindolin-2-one (**S100**).

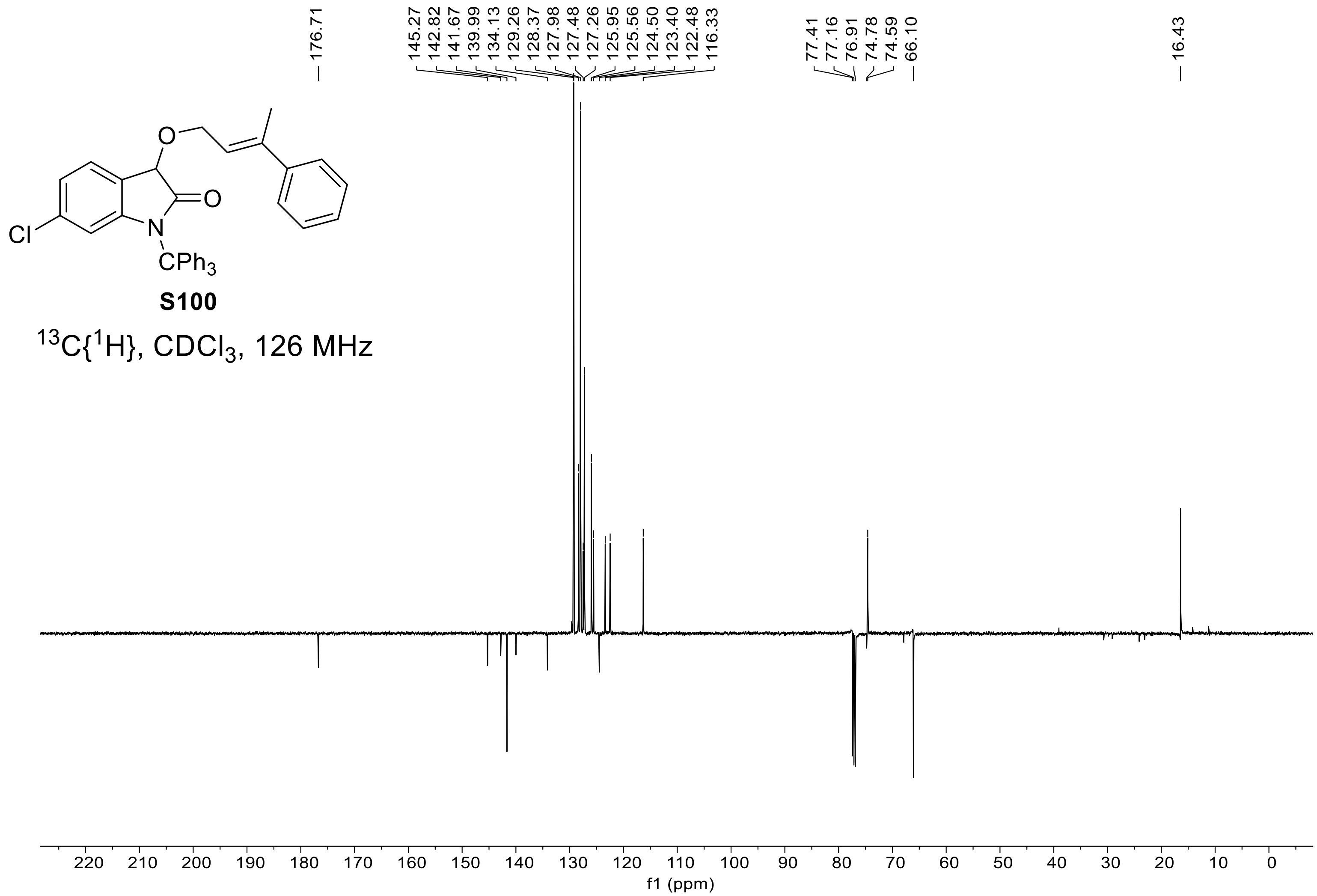

Fig. S250: $^{13}C\{^{1}H\}$ NMR spectrum for (*E*)-6-Chloro-3-[(3-phenylbut-2-en-1-yl)oxy]-1-tritylindolin-2-one (**S100**).

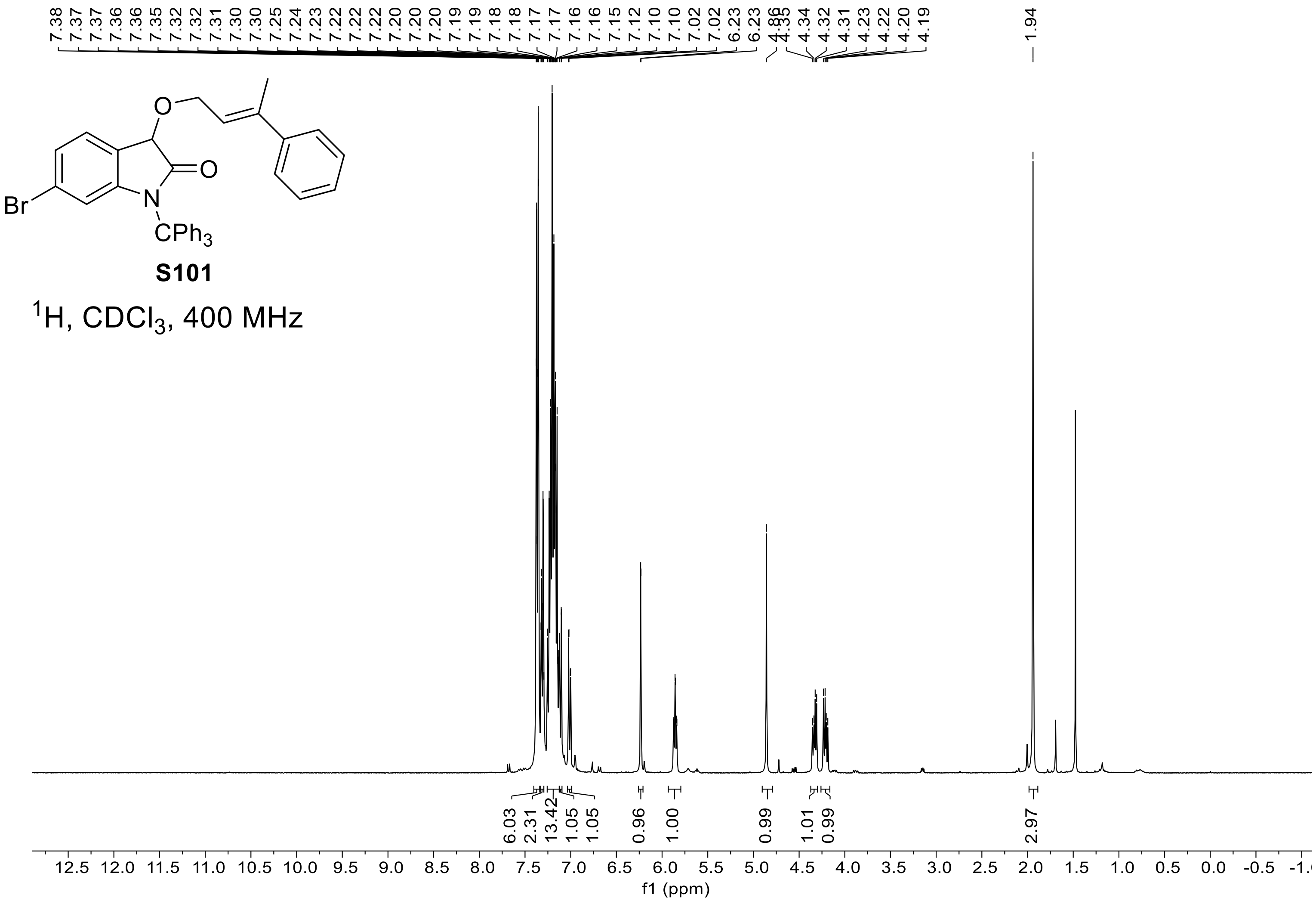

Fig. S251: ^{1}H NMR spectrum for (*E*)-6-Bromo-3-[(3-phenylbut-2-en-1-yl)oxy]-1-tritylindolin-2-one (**S101**).

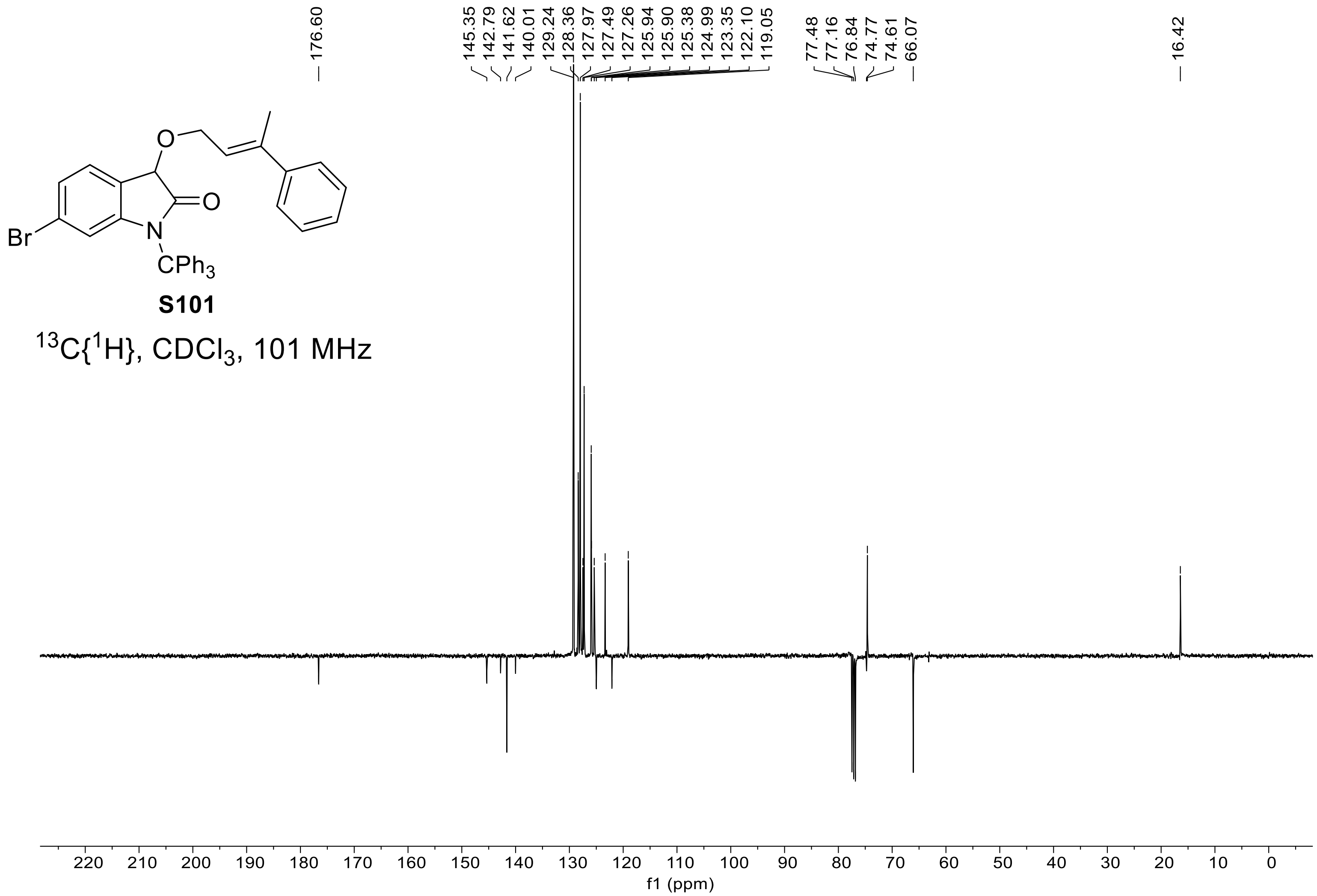

Fig. S252: $^{13}C\{^1H\}$ NMR spectrum for (*E*)-6-Bromo-3-[(3-phenylbut-2-en-1-yl)oxy]-1-tritylindolin-2-one (**S101**).

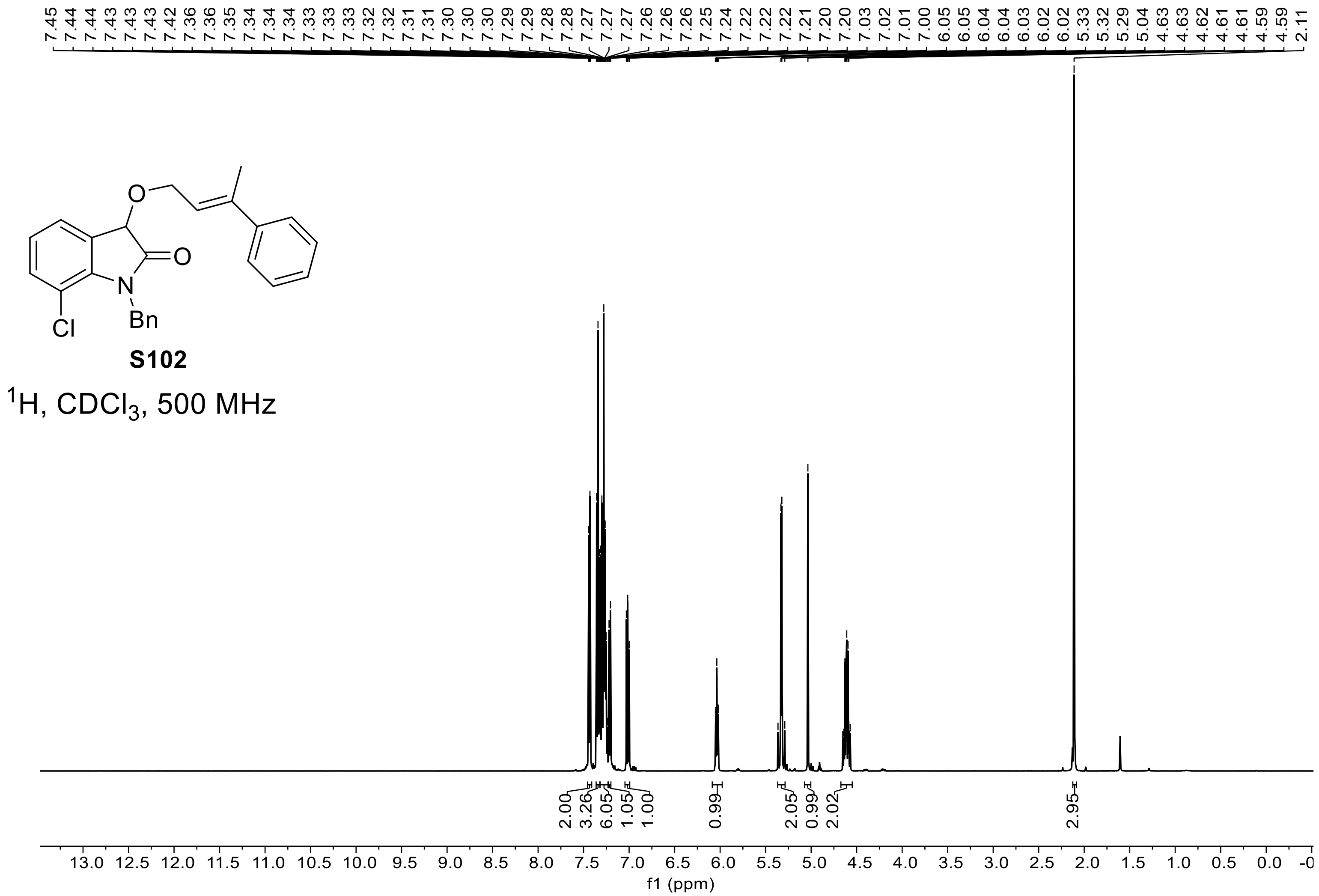

Fig. S253: ^{13}C{^{1}H} NMR spectrum for (*E*)-1-Benzyl-7-chloro-3-[(3-phenylbut-2-en-1-yl)oxy]indolin-2-one (**S102**).

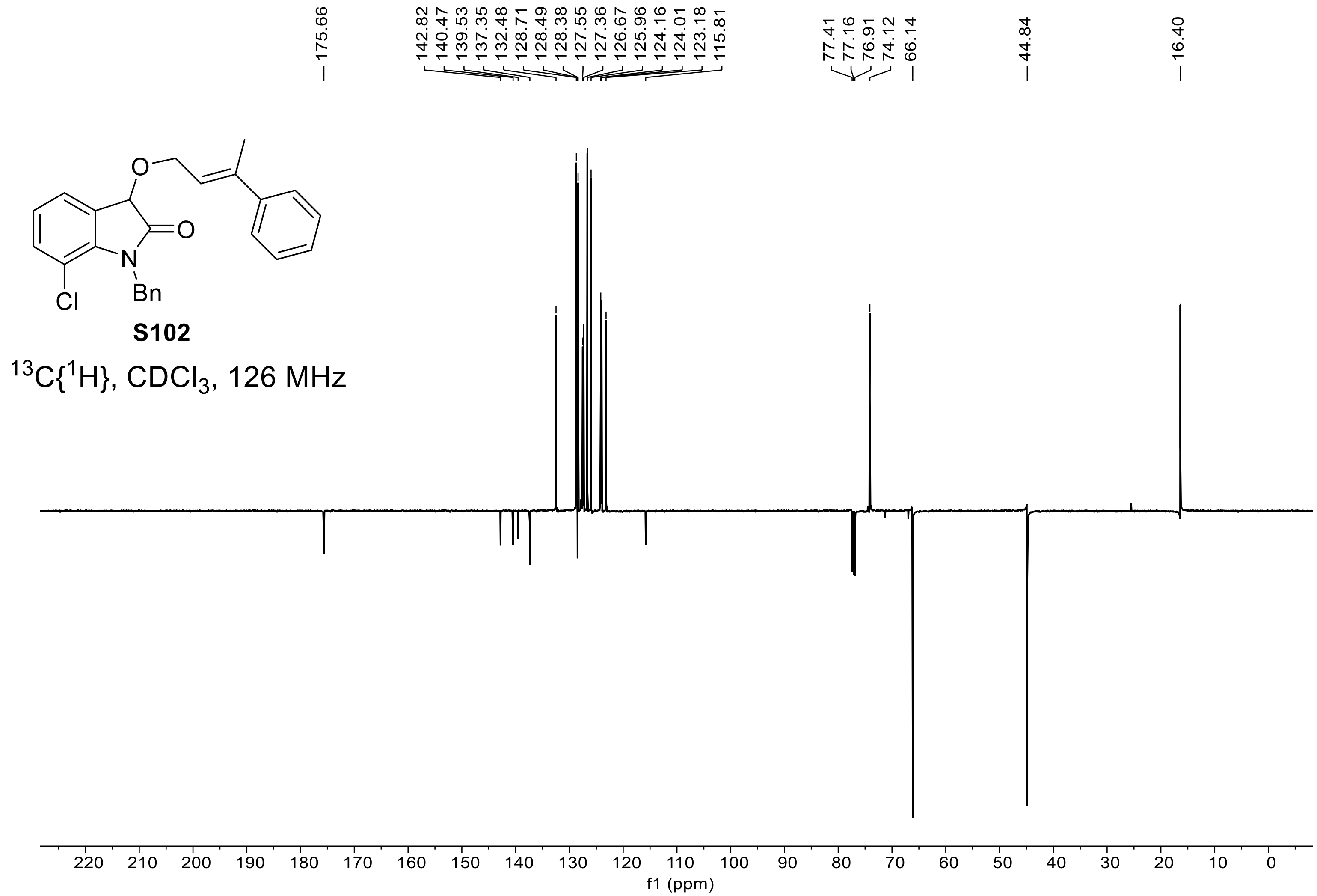

Fig. S254: $^{13}C\{^{1}H\}$ NMR spectrum for (*E*)-1-Benzyl-7-chloro-3-[(3-phenylbut-2-en-1-yl)oxy]indolin-2-one (**S102**).

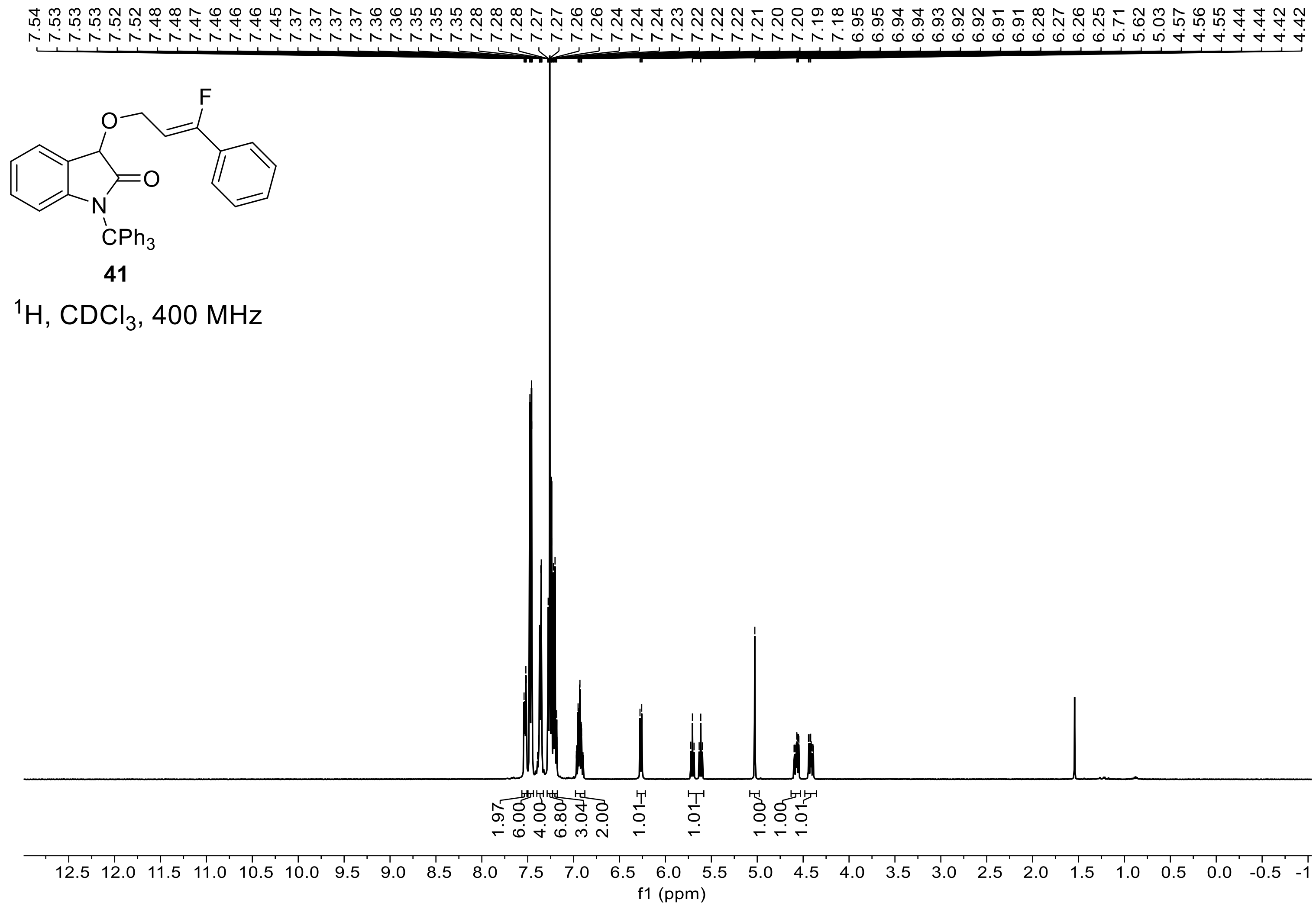

Fig. S255: ^{1}H NMR spectrum for (*Z*)-3-[(3-Fluoro-3-phenylallyl)oxy]-1-tritylindolin-2-one (**41**).

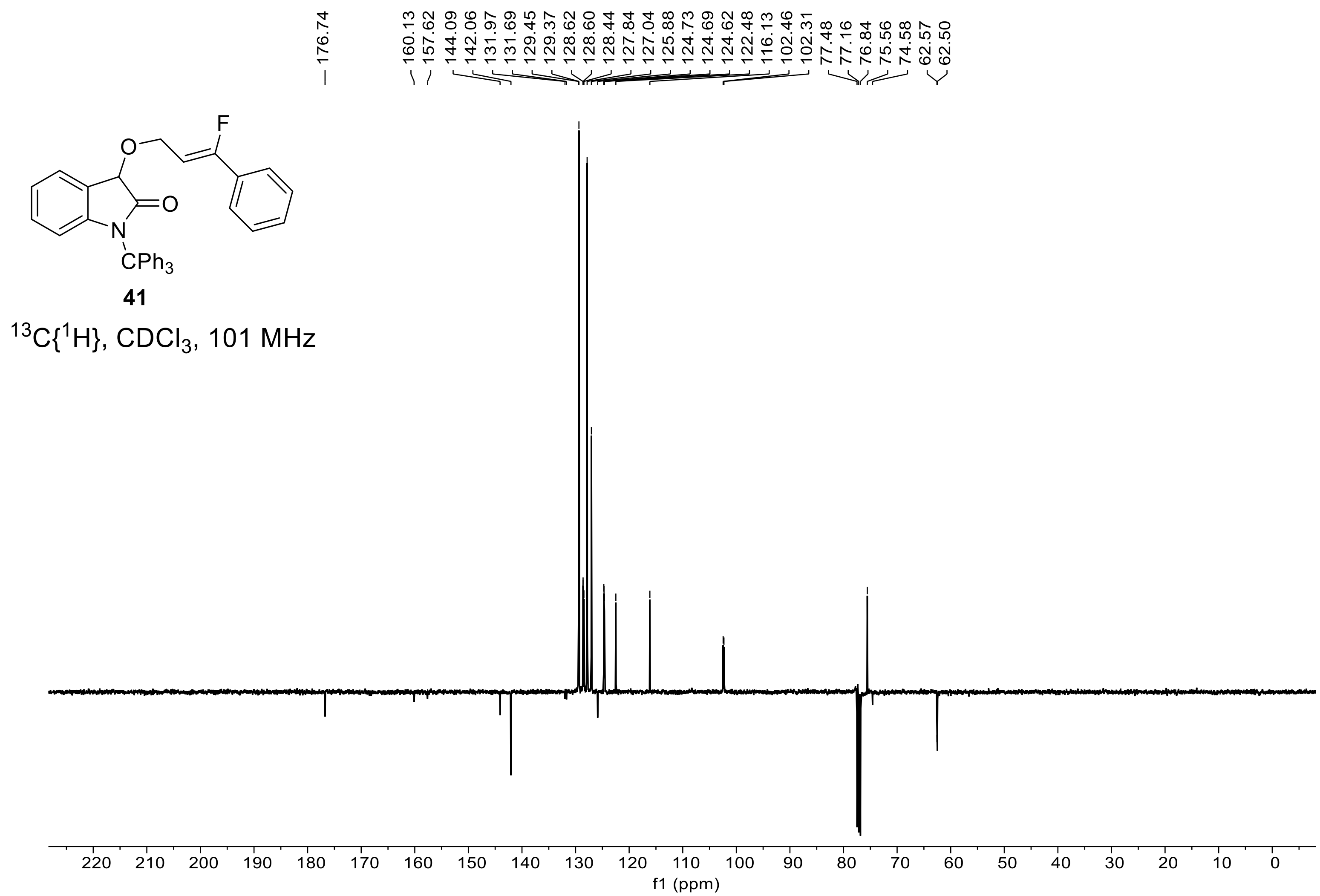

Fig. S256: $^{13}C\{^1H\}$ NMR spectrum for (*Z*)-3-[(3-Fluoro-3-phenylallyl)oxy]-1-tritylindolin-2-one (**41**).

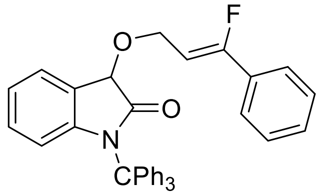

41

$^{19}F\{^1H\}$, $CDCl_3$, 377 MHz

-115.51

10 0 -10 -20 -30 -40 -50 -60 -70 -80 -90 -100 -110 -120 -130 -140 -150 -160 -170 -180 -190 -200 -210 -220 -230 -240 -25

f1 (ppm)

Fig. S257: $^{19}F\{^1H\}$ NMR spectrum for (*Z*)-3-[(3-Fluoro-3-phenylallyl)oxy]-1-tritylindolin-2-one (**41**).

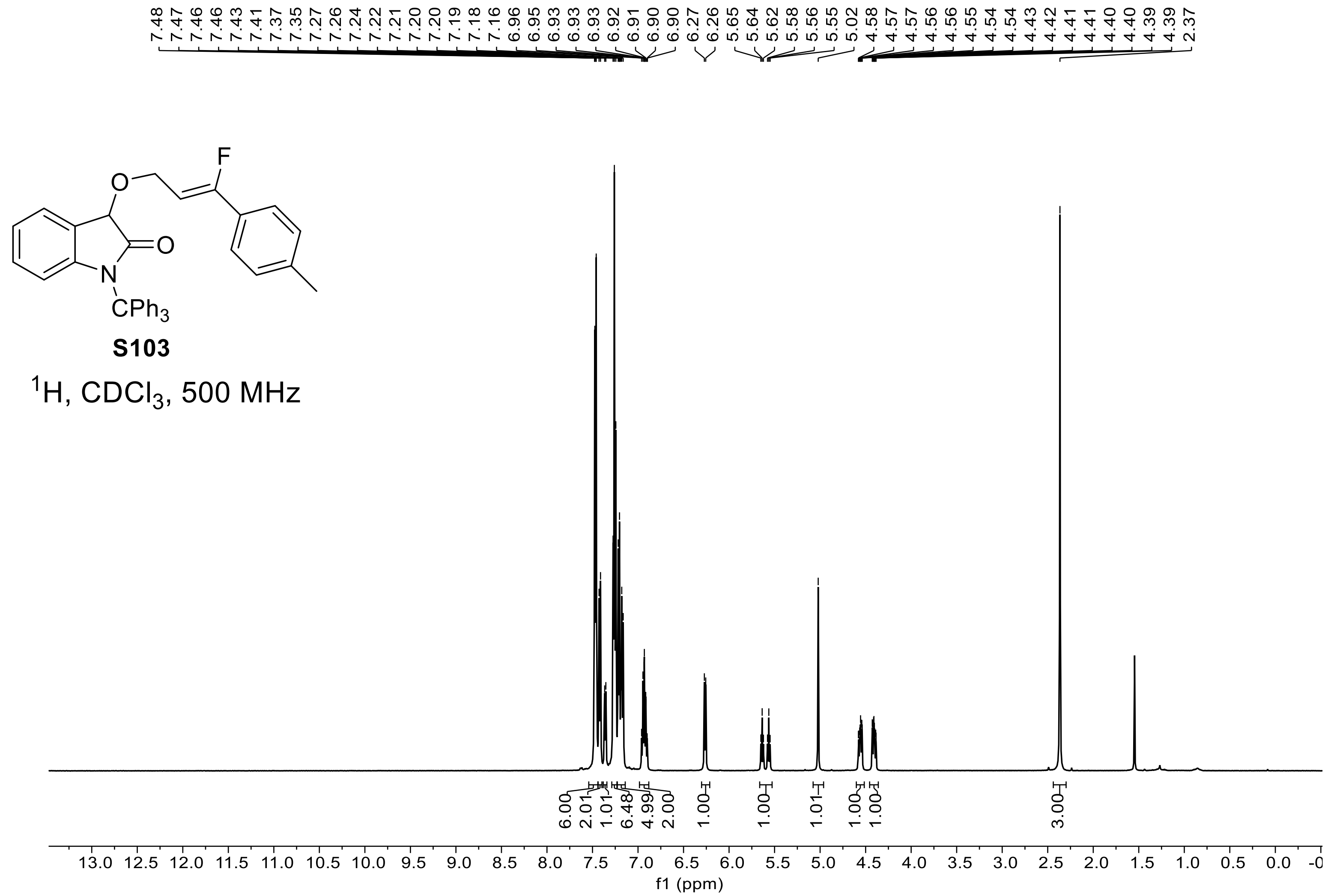

Fig. S257: ^{1}H NMR spectrum for (*Z*)-3-{[3-fluoro-3-(*p*-tolyl)allyl]oxy}-1-tritylindolin-2-one (**S103**).

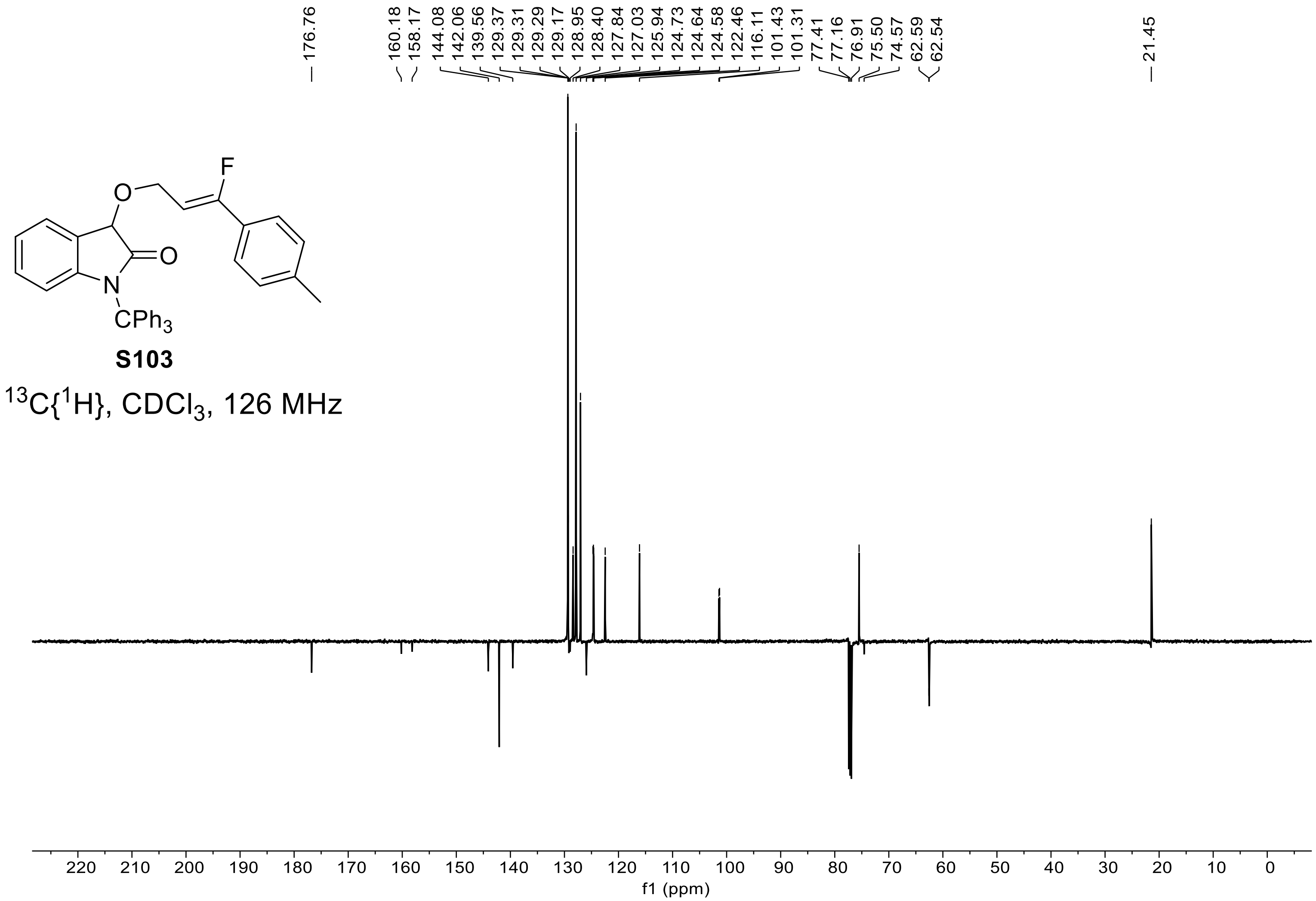

Fig. S258: $^{13}C\{^{1}H\}$ NMR spectrum for (*Z*)-3-{[3-fluoro-3-(*p*-tolyl)allyl]oxy}-1-tritylindolin-2-one (**S103**).

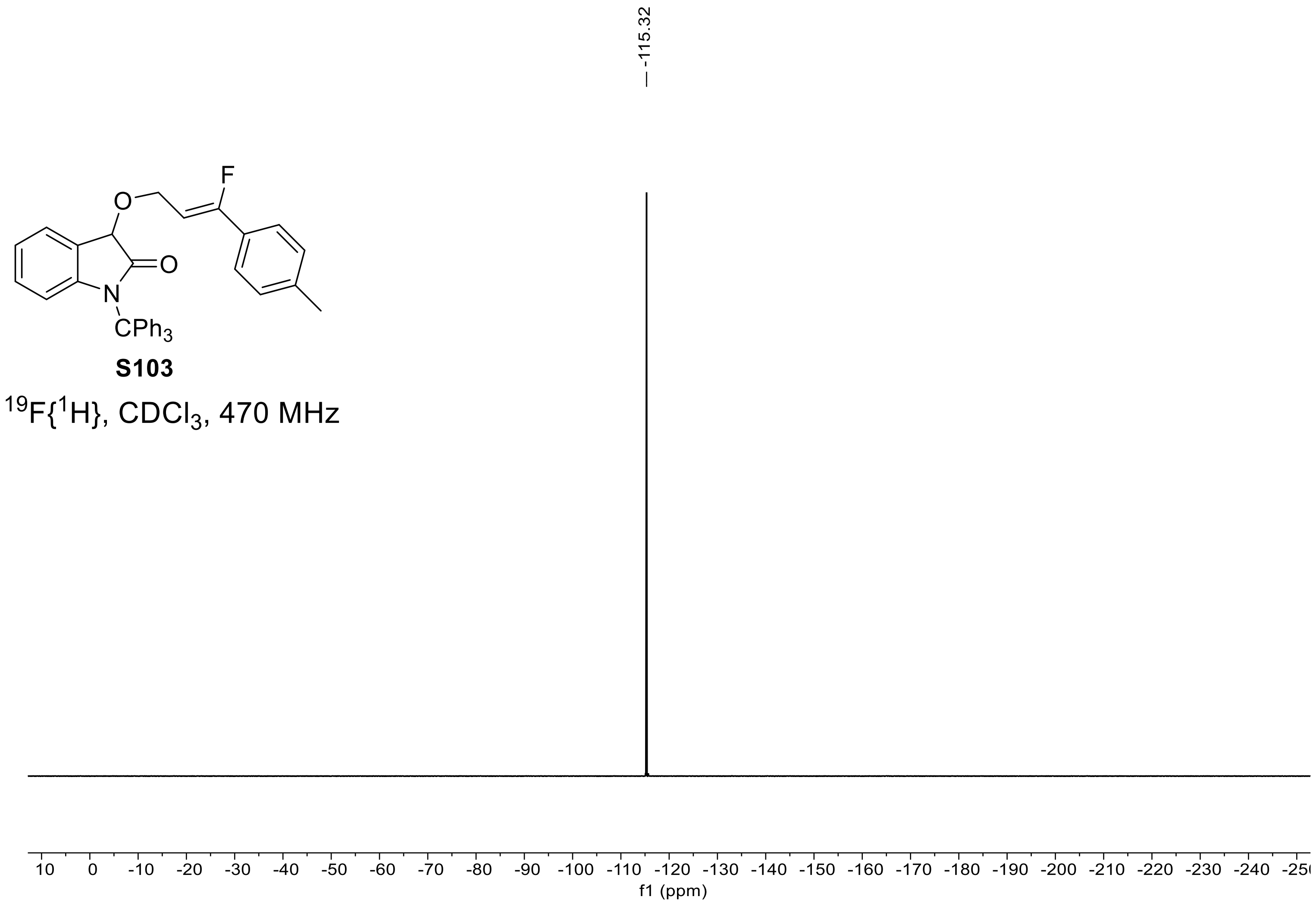

Fig. S259: $^{19}F\{^{1}H\}$ NMR spectrum for (*Z*)-3-{[3-fluoro-3-(*p*-tolyl)allyl]oxy}-1-tritylindolin-2-one (**S103**).

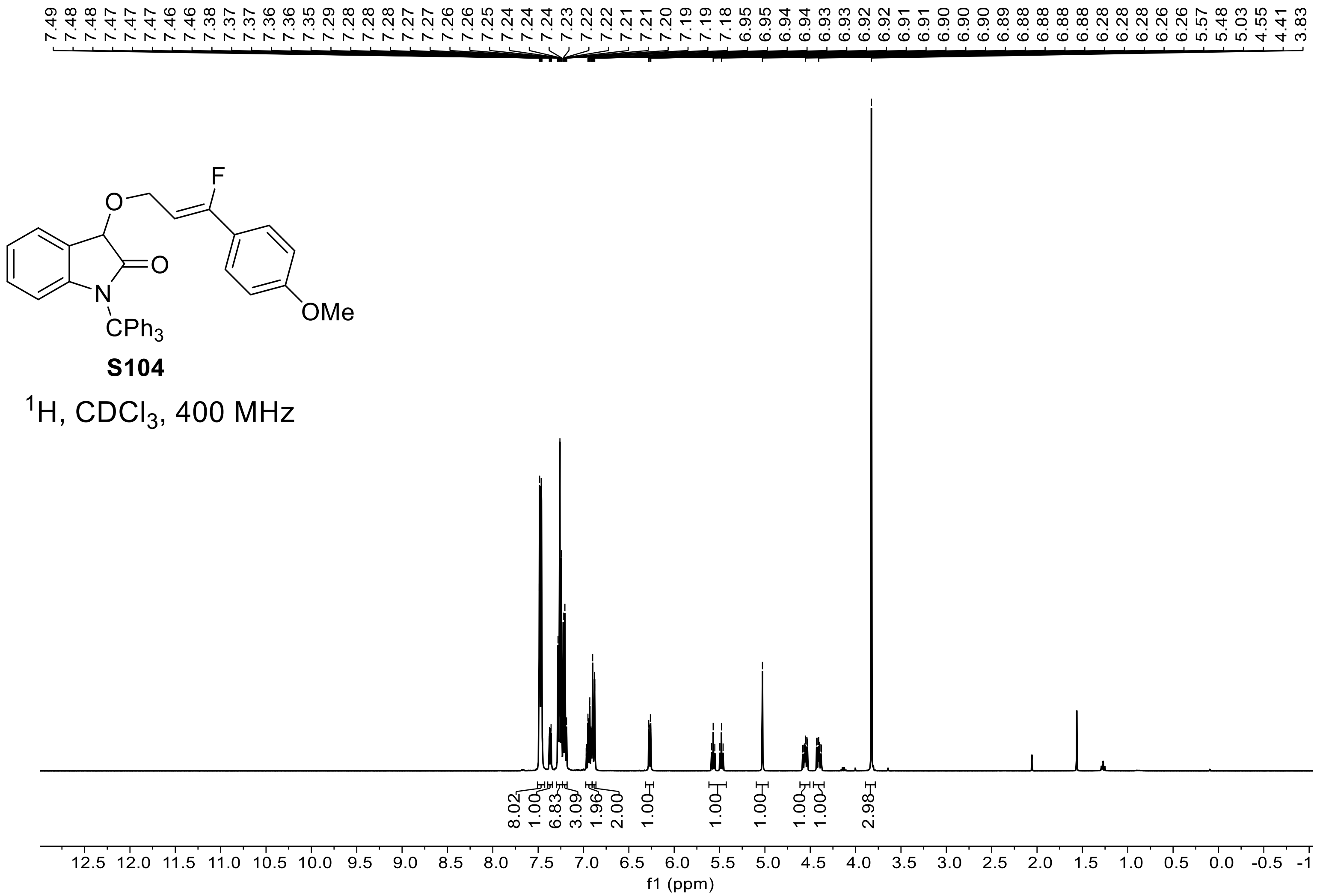

Fig. S260: ^{1}H NMR spectrum for (*Z*)-3-{[3-Fluoro-3-(4-methoxyphenyl)allyl]oxy}-1-tritylindolin-2-one (**S104**).

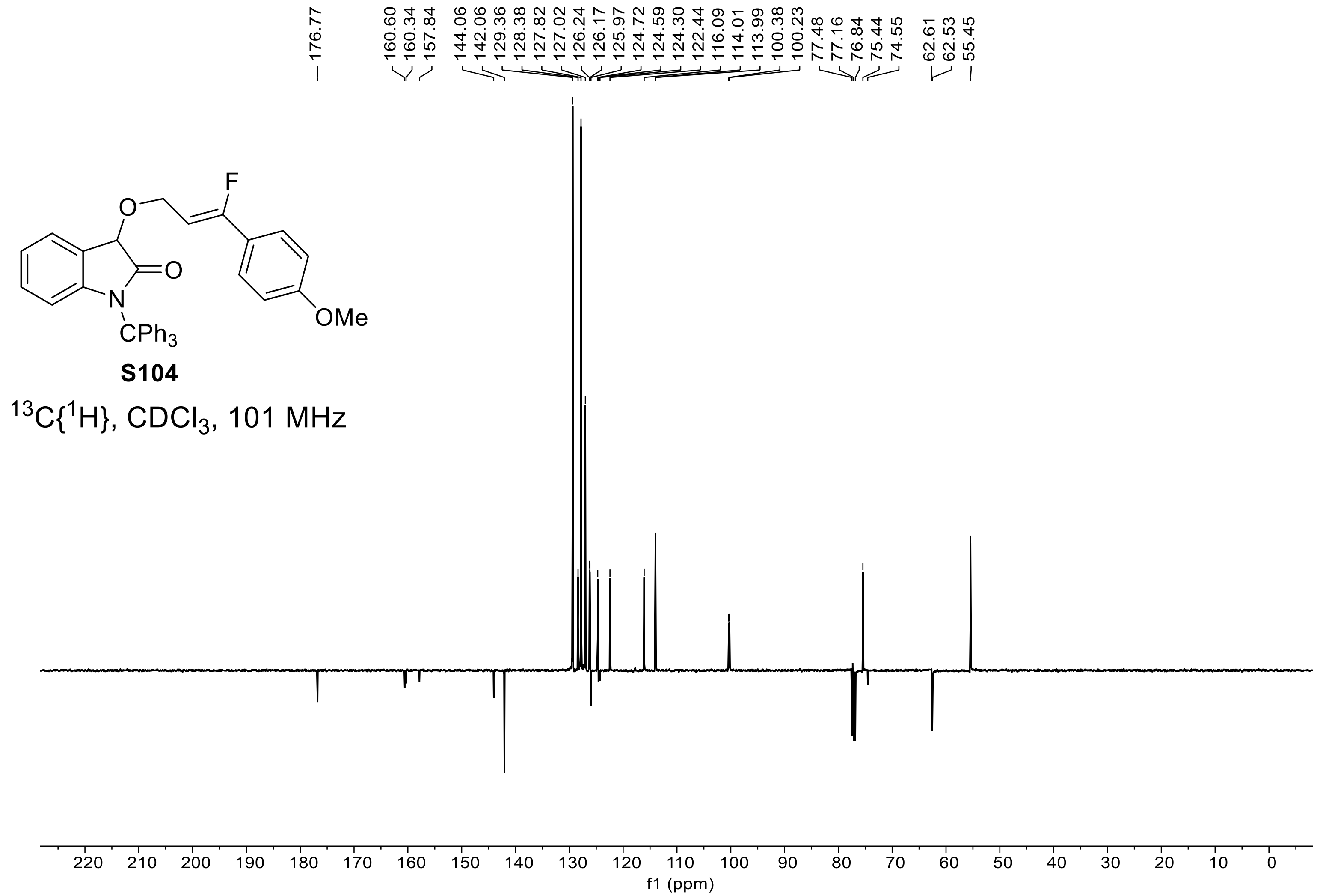

Fig. S261: $^{13}C\{^{1}H\}$ NMR spectrum for (*Z*)-3-{[3-Fluoro-3-(4-methoxyphenyl)allyl]oxy}-1-tritylindolin-2-one (**S104**).

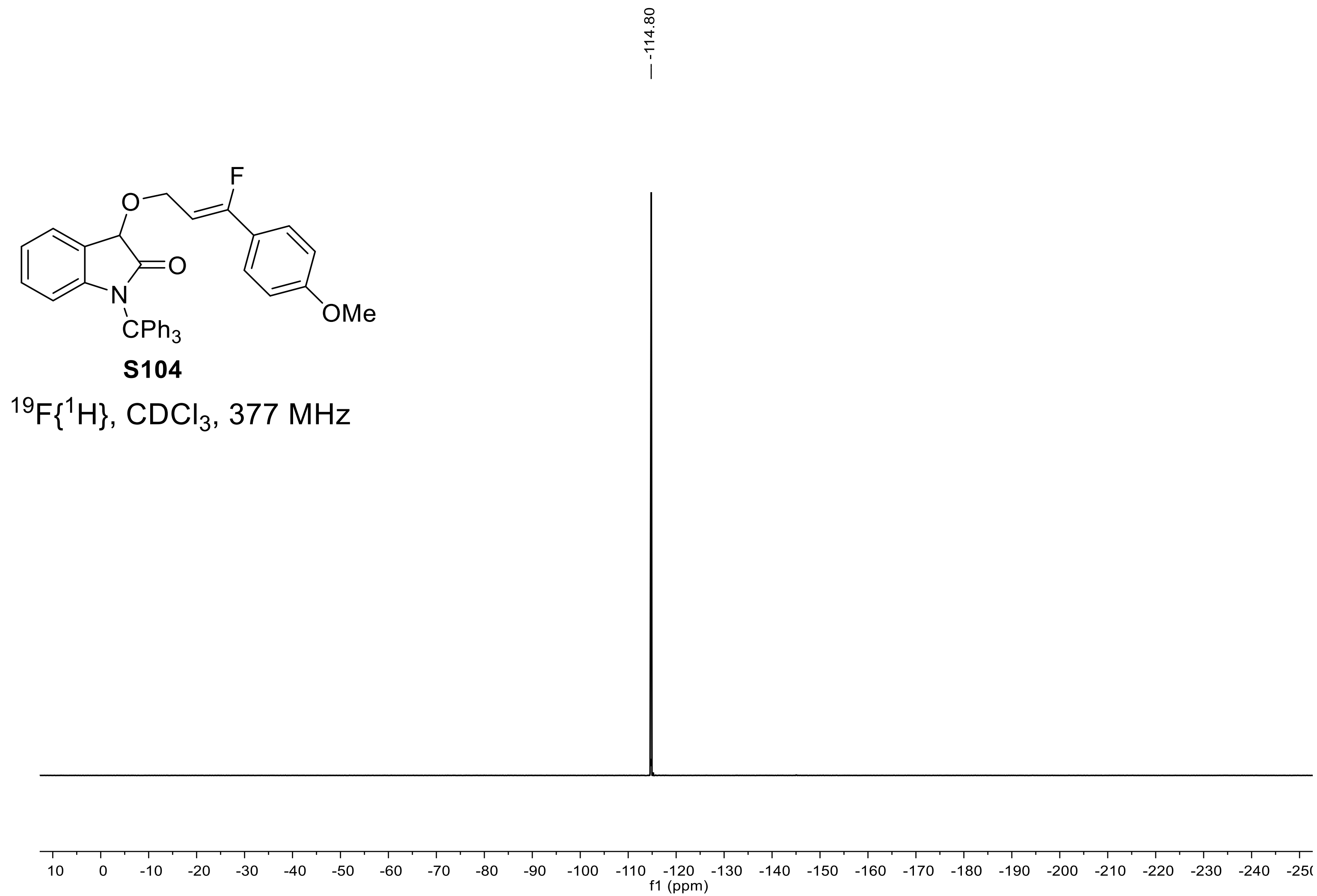

Fig. S262: $^{19}F\{^{1}H\}$ NMR spectrum for (*Z*)-3-{[3-Fluoro-3-(4-methoxyphenyl)allyl]oxy}-1-tritylindolin-2-one (**S104**).

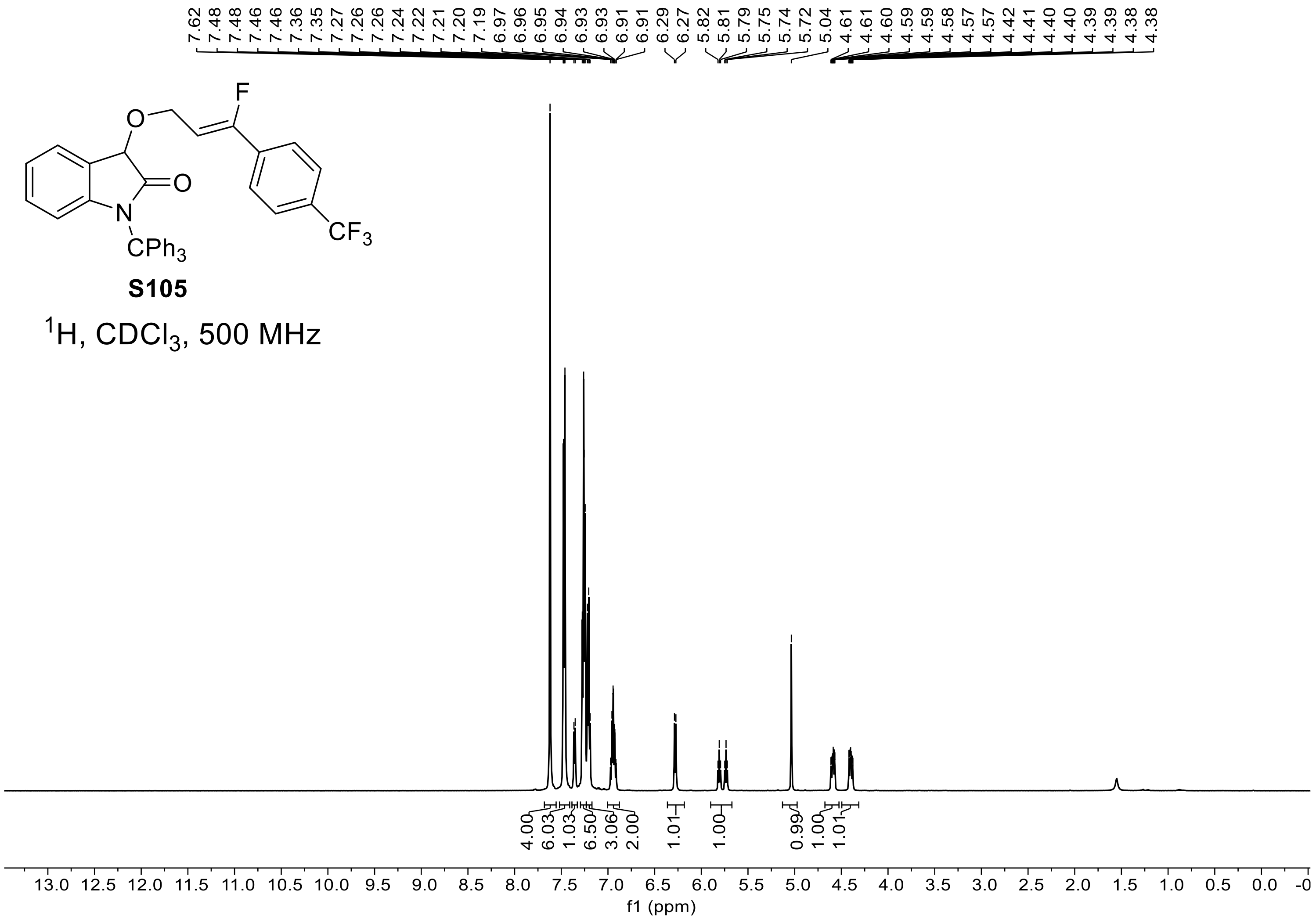

Fig. S263: ^{1}H NMR spectrum for (*Z*)-3-{[3-Fluoro-3-(4-(trifluoromethyl)phenyl)allyl]oxy}-1-tritylindolin-2-one (**S105**).

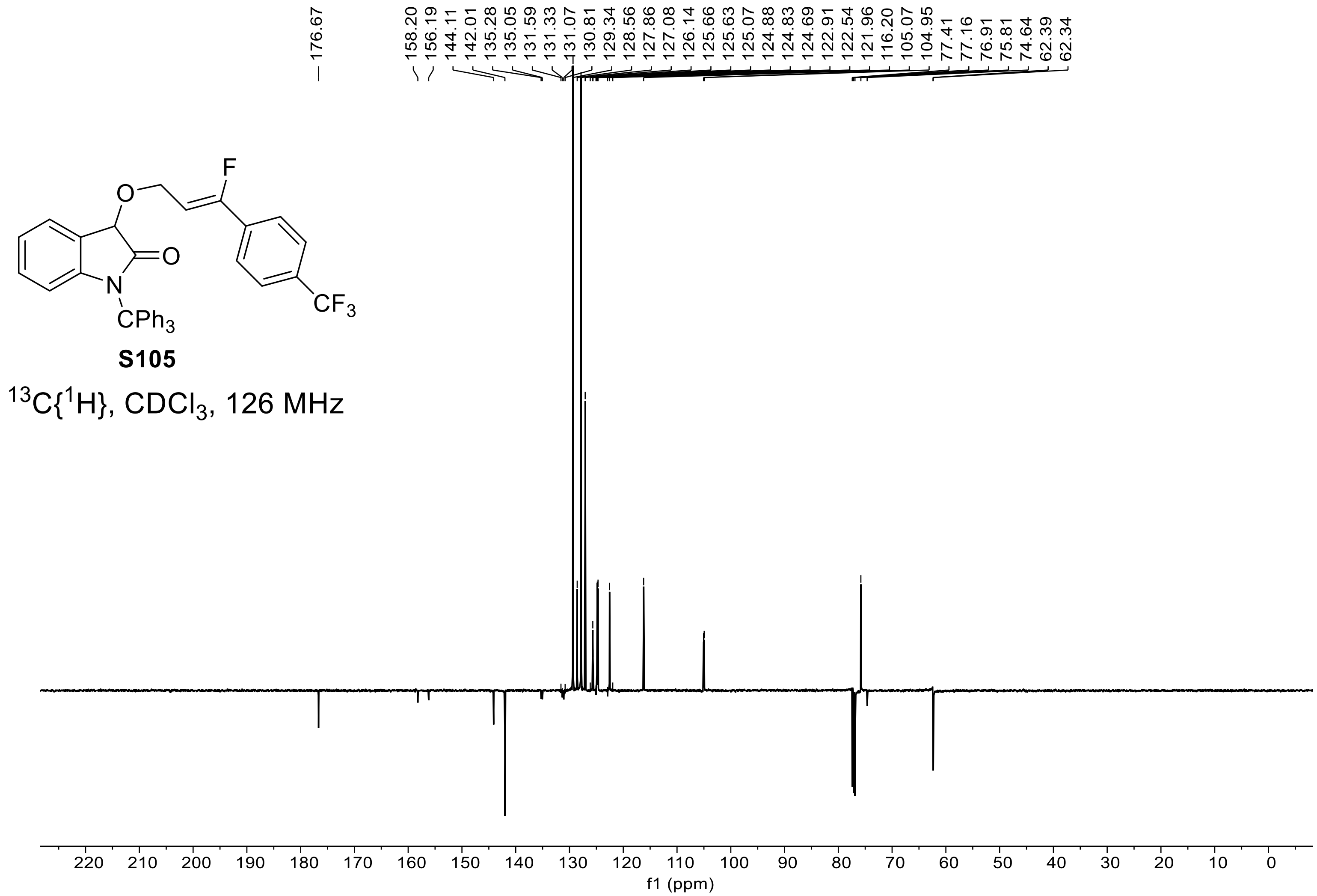

Fig. S264: ^{13}C{^{1}H} NMR spectrum for (*Z*)-3-{[3-Fluoro-3-(4-(trifluoromethyl)phenyl)allyl]oxy}-1-tritylindolin-2-one (**S105**).

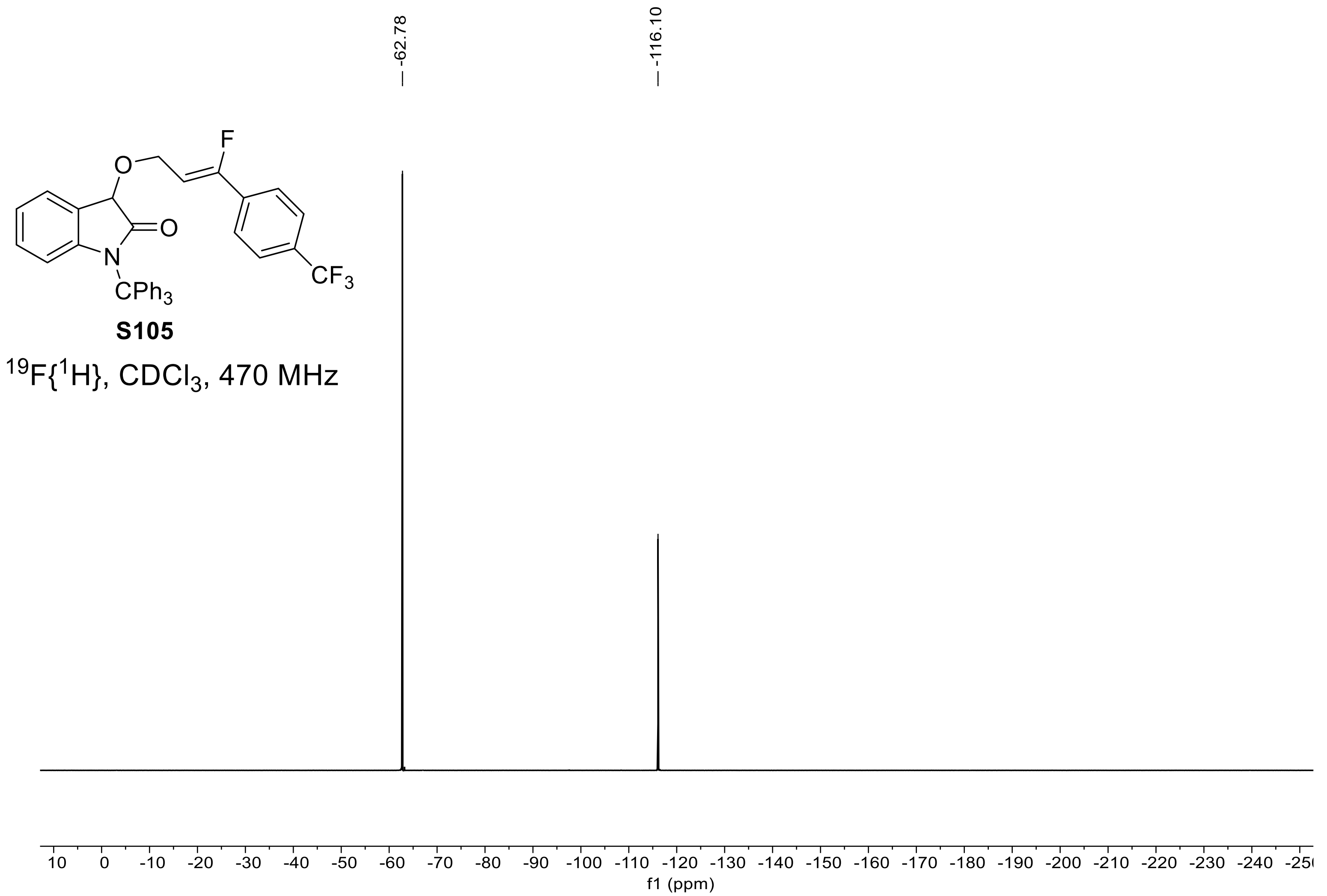

Fig. S265: $^{19}F\{^{1}H\}$ NMR spectrum for (*Z*)-3-{[3-Fluoro-3-(4-(trifluoromethyl)phenyl)allyl]oxy}-1-tritylindolin-2-one (**S105**).

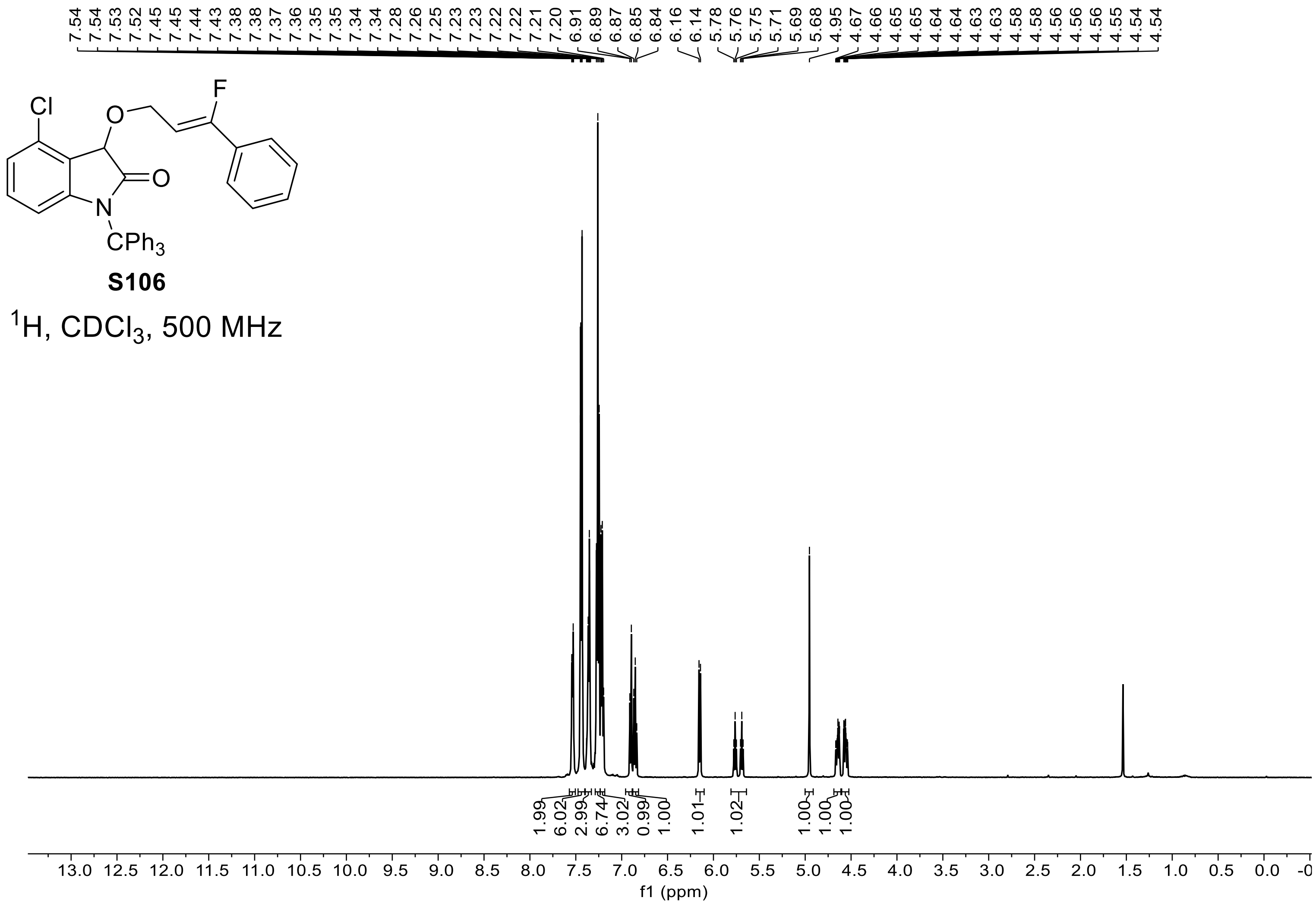

Fig. S266: ^{1}H NMR spectrum for (*Z*)-4-Chloro-3-[(3-fluoro-3-phenylallyl)oxy]-1-tritylindolin-2-one (**S106**).

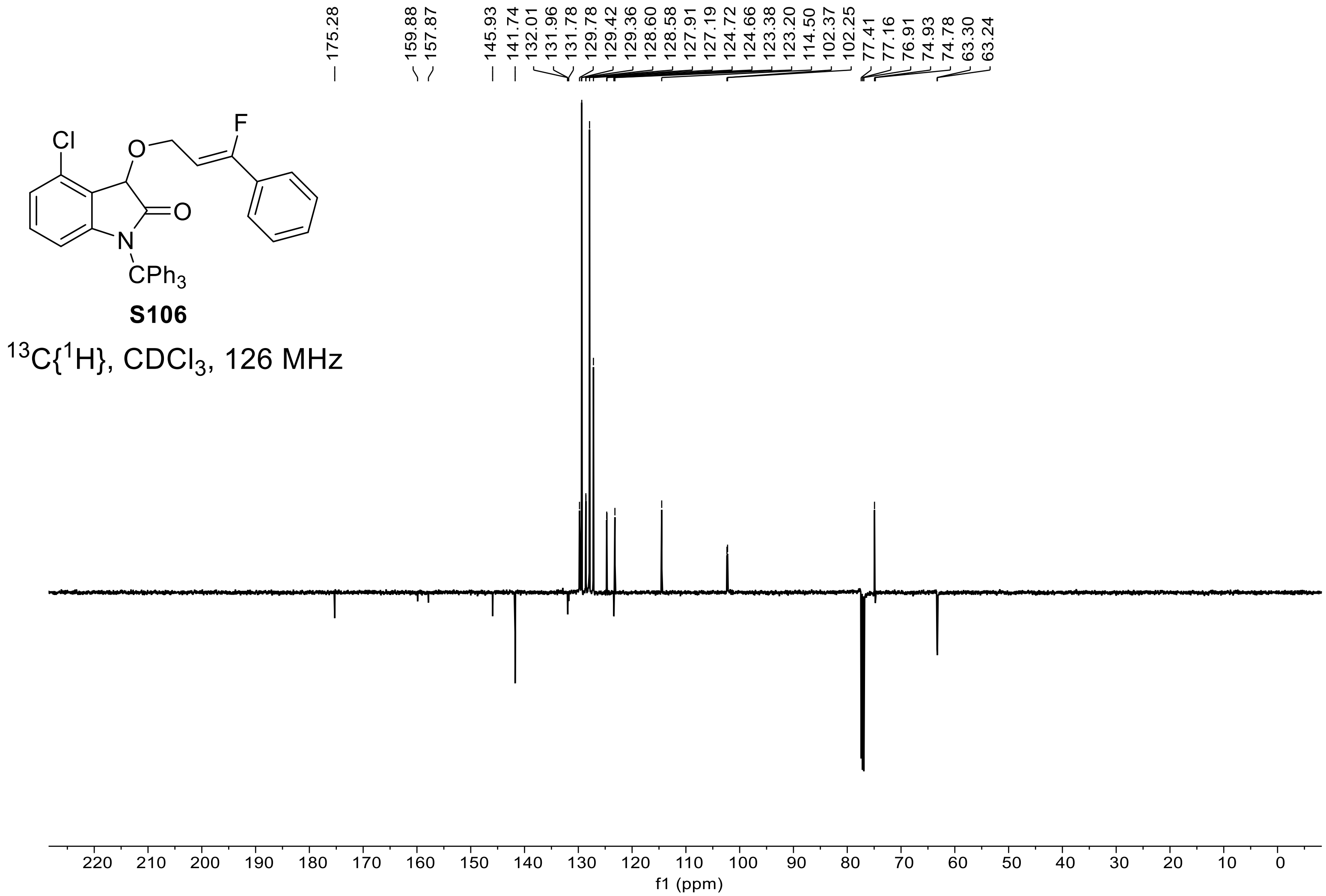

Fig. S267: $^{13}C\{^{1}H\}$ NMR spectrum for (*Z*)-4-Chloro-3-[(3-fluoro-3-phenylallyl)oxy]-1-tritylindolin-2-one (**S106**).

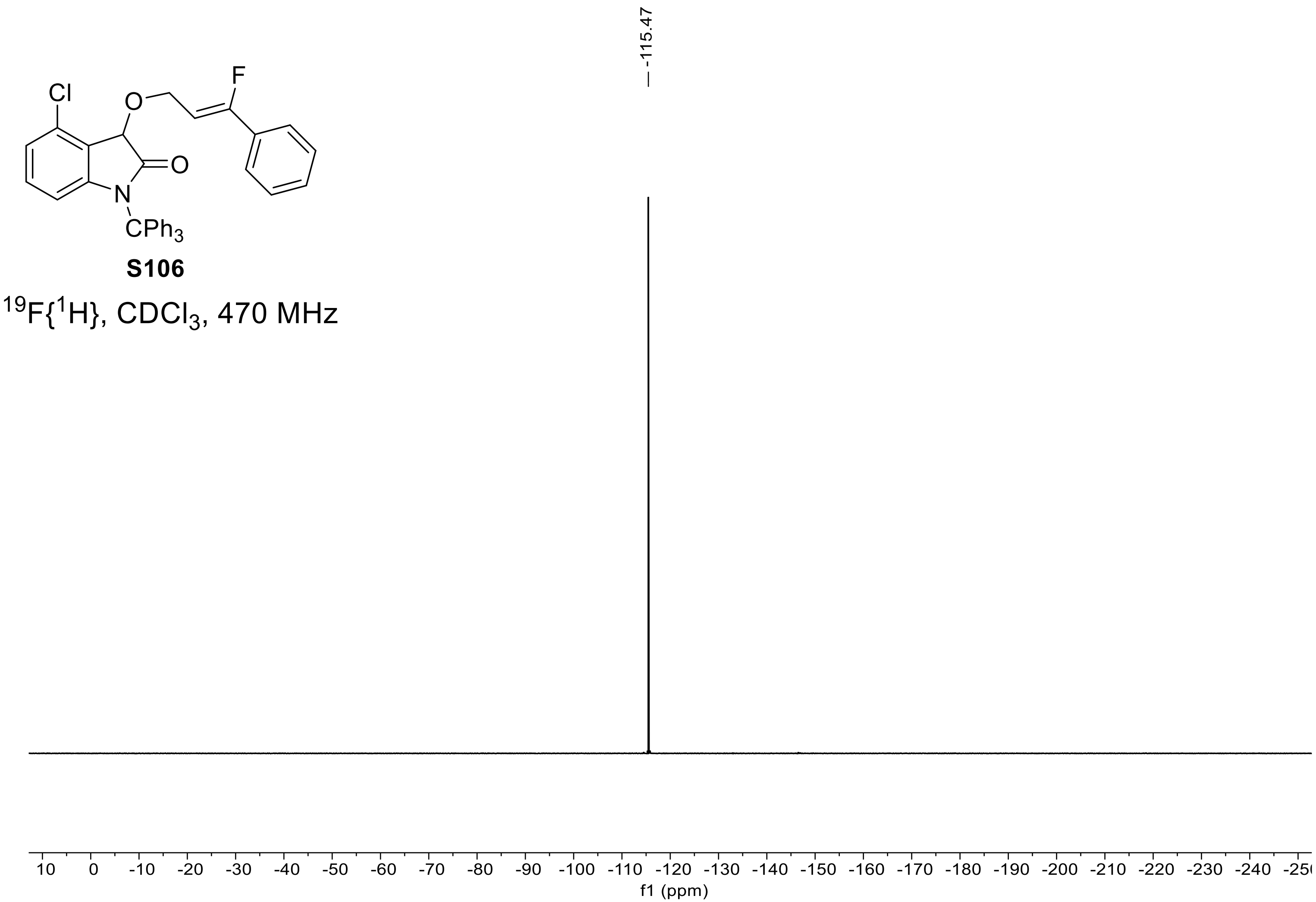

Fig. S268: $^{19}F\{^{1}H\}$ NMR spectrum for (*Z*)-4-Chloro-3-[(3-fluoro-3-phenylallyl)oxy]-1-tritylindolin-2-one (**S106**).

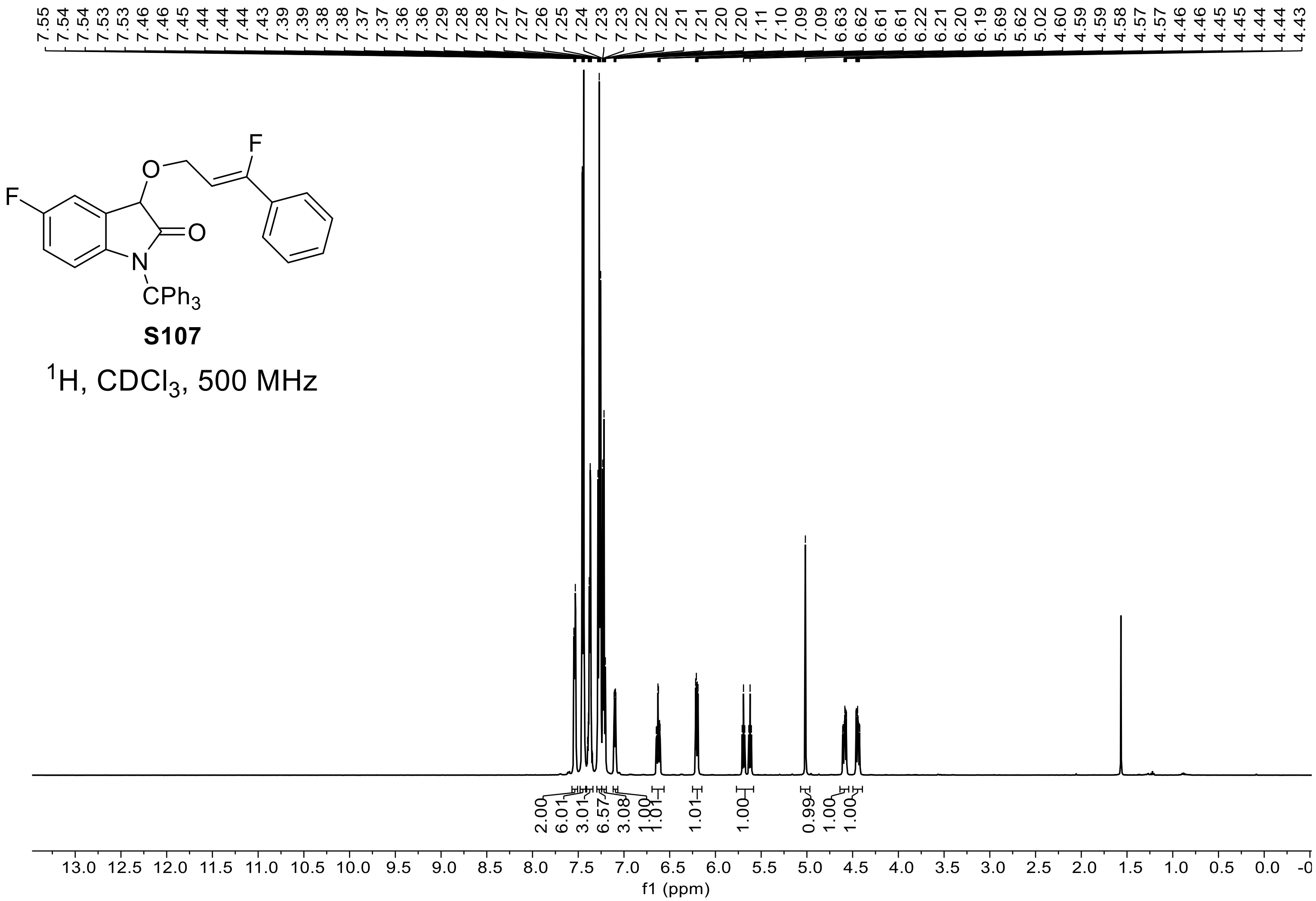

Fig. S269: ^{1}H NMR spectrum for (*Z*)-5-Fluoro-3-[(3-fluoro-3-phenylallyl)oxy]-1-tritylindolin-2-one (**S107**).

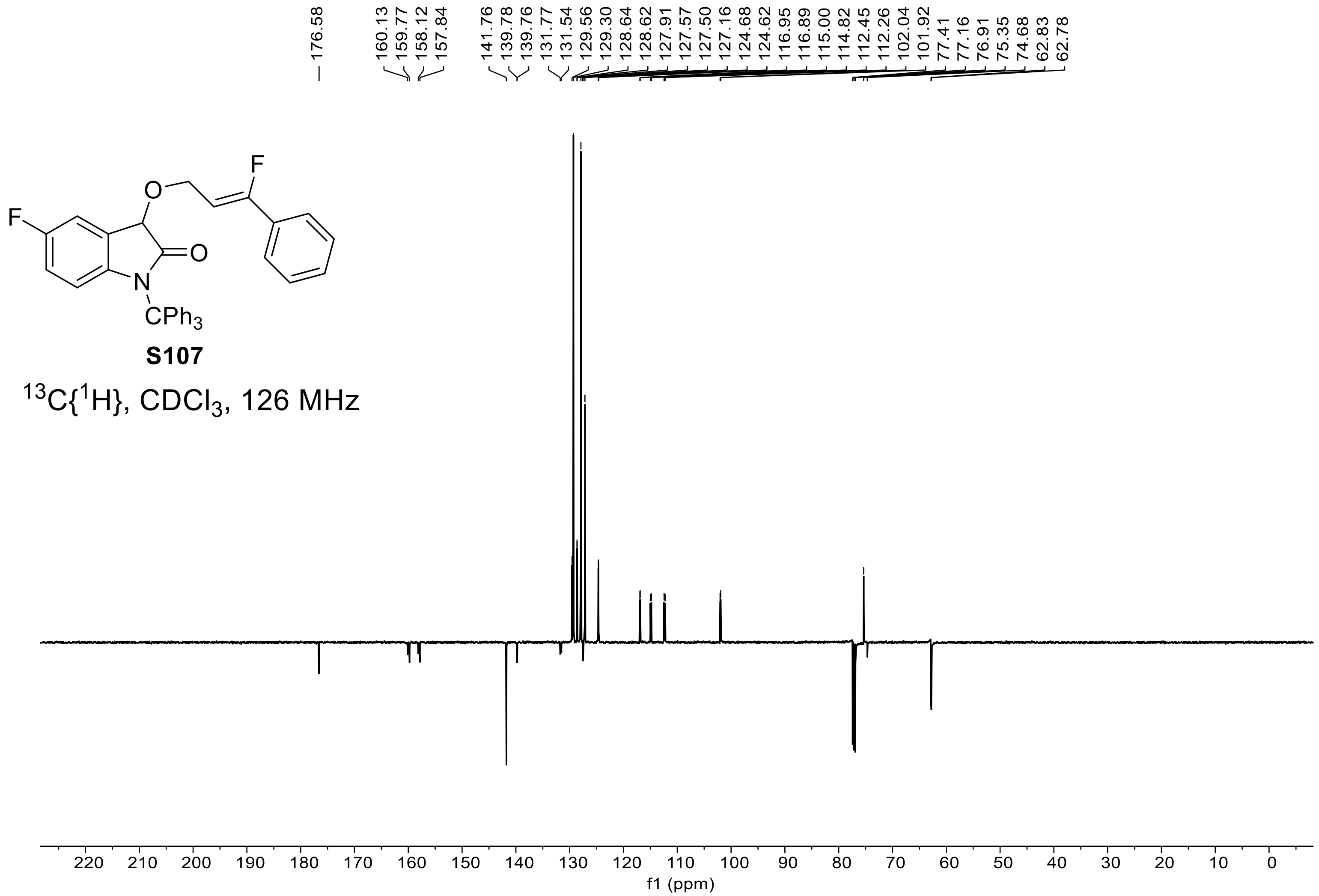

Fig. S270: $^{13}C\{^{1}H\}$ NMR spectrum for (*Z*)-5-Fluoro-3-[(3-fluoro-3-phenylallyl)oxy]-1-tritylindolin-2-one (**S107**).

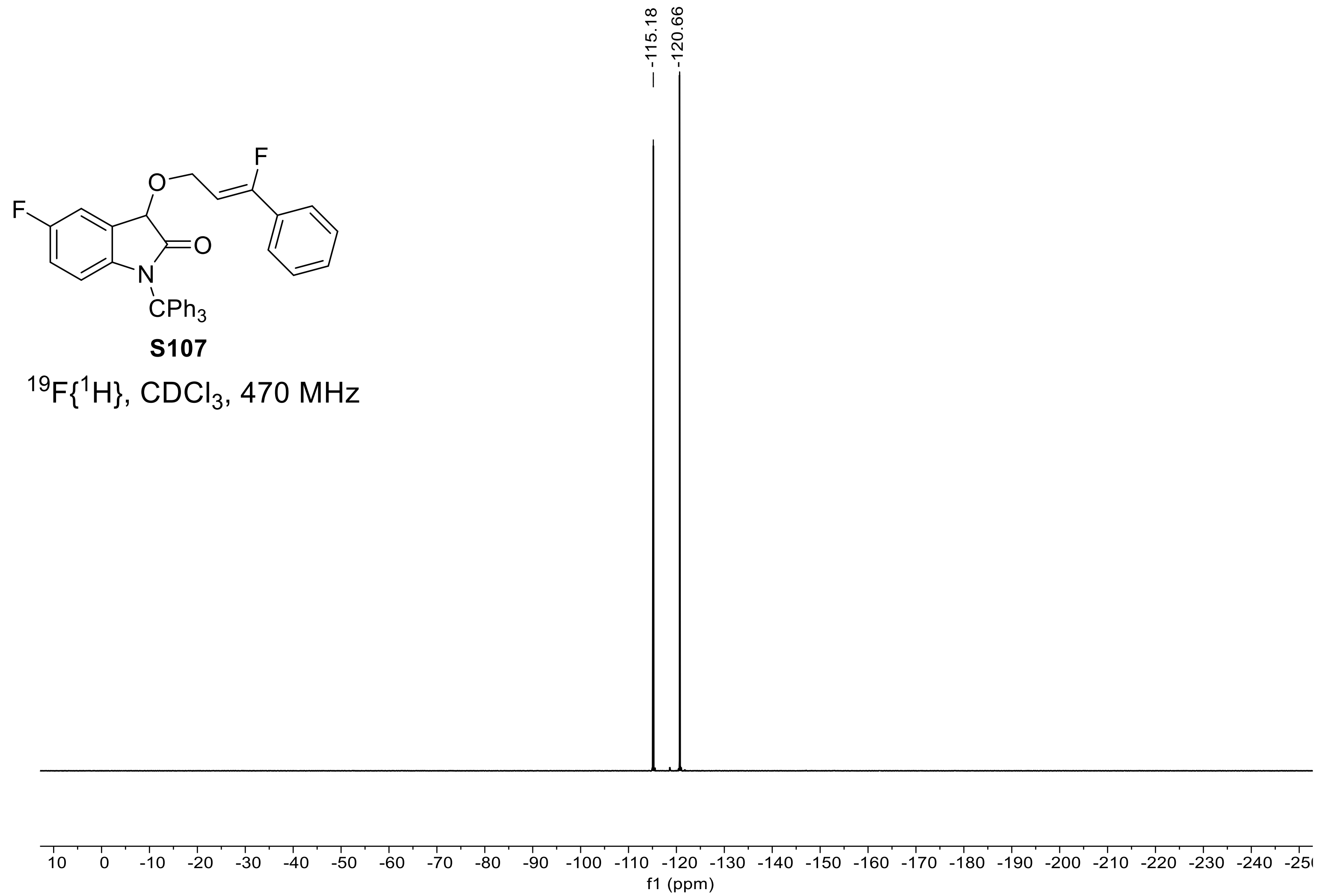

Fig. S271: $^{19}F\{^{1}H\}$ NMR spectrum for (*Z*)-5-Fluoro-3-[(3-fluoro-3-phenylallyl)oxy]-1-tritylindolin-2-one (**S107**).

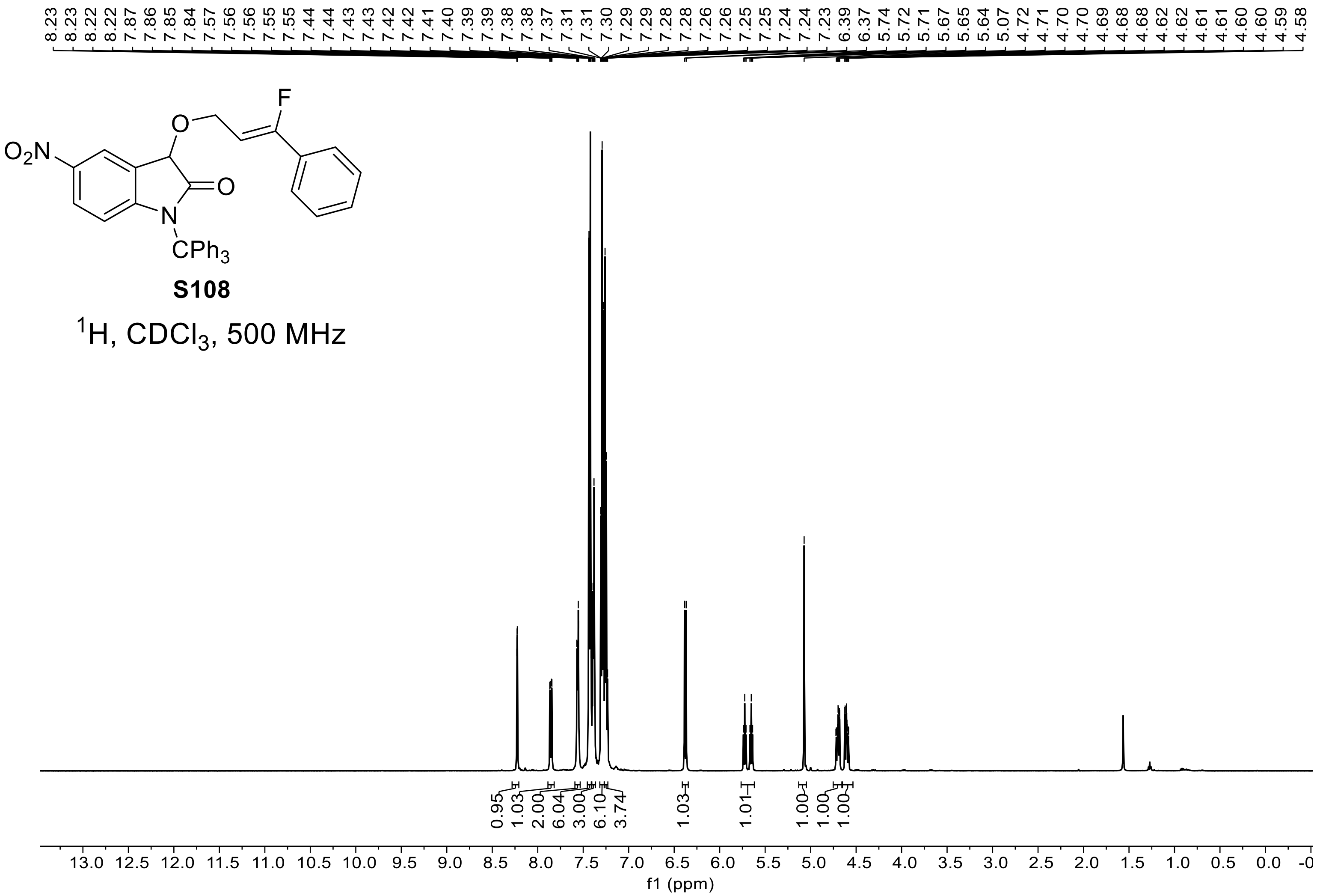

Fig. S272: ^{1}H NMR spectrum for (*Z*)-3-[(3-Fluoro-3-phenylallyl)oxy]-5-nitro-1-tritylindolin-2-one (**S108**).

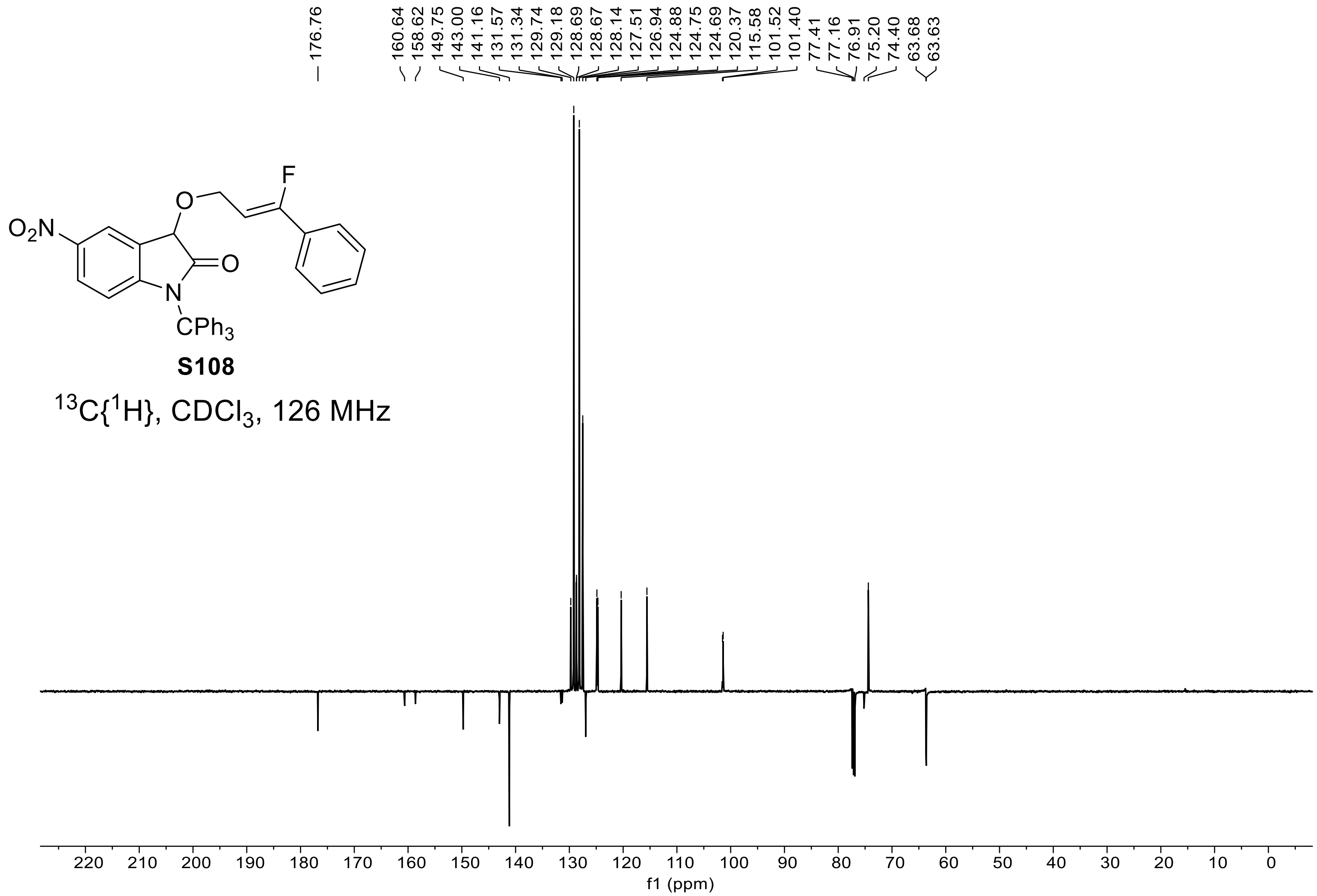

Fig. S273: $^{13}C\{^1H\}$ NMR spectrum for (*Z*)-3-[(3-Fluoro-3-phenylallyl)oxy]-5-nitro-1-tritylindolin-2-one (**S108**).

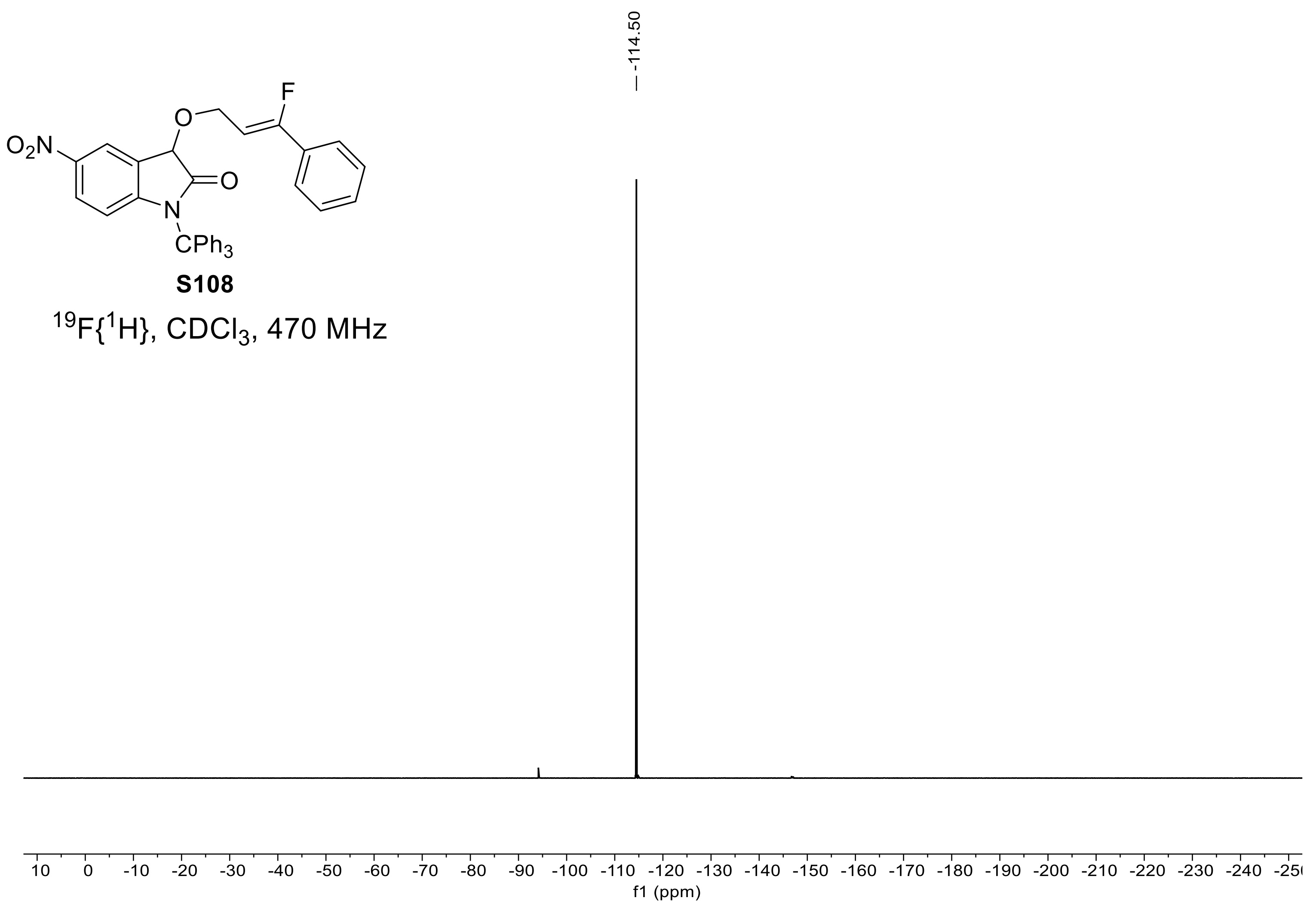

Fig. S274: $^{19}F\{^1H\}$ NMR spectrum for (*Z*)-3-[(3-Fluoro-3-phenylallyl)oxy]-5-nitro-1-tritylindolin-2-one (**S108**).

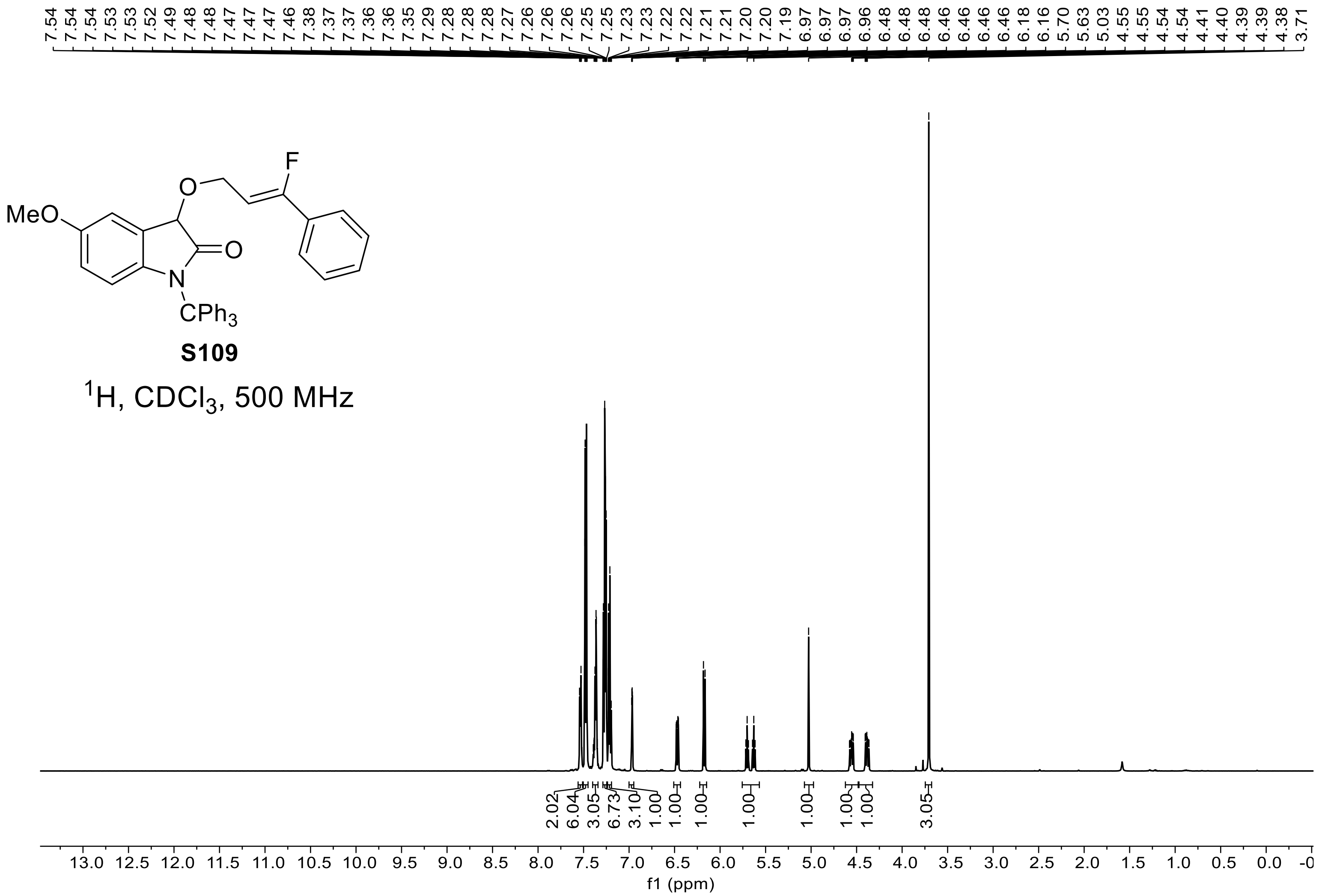

Fig. S275: ^{1}H NMR spectrum for (*Z*)-3-[(3-Fluoro-3-phenylallyl)oxy]-5-methoxy-1-tritylindolin-2-one (**S109**).

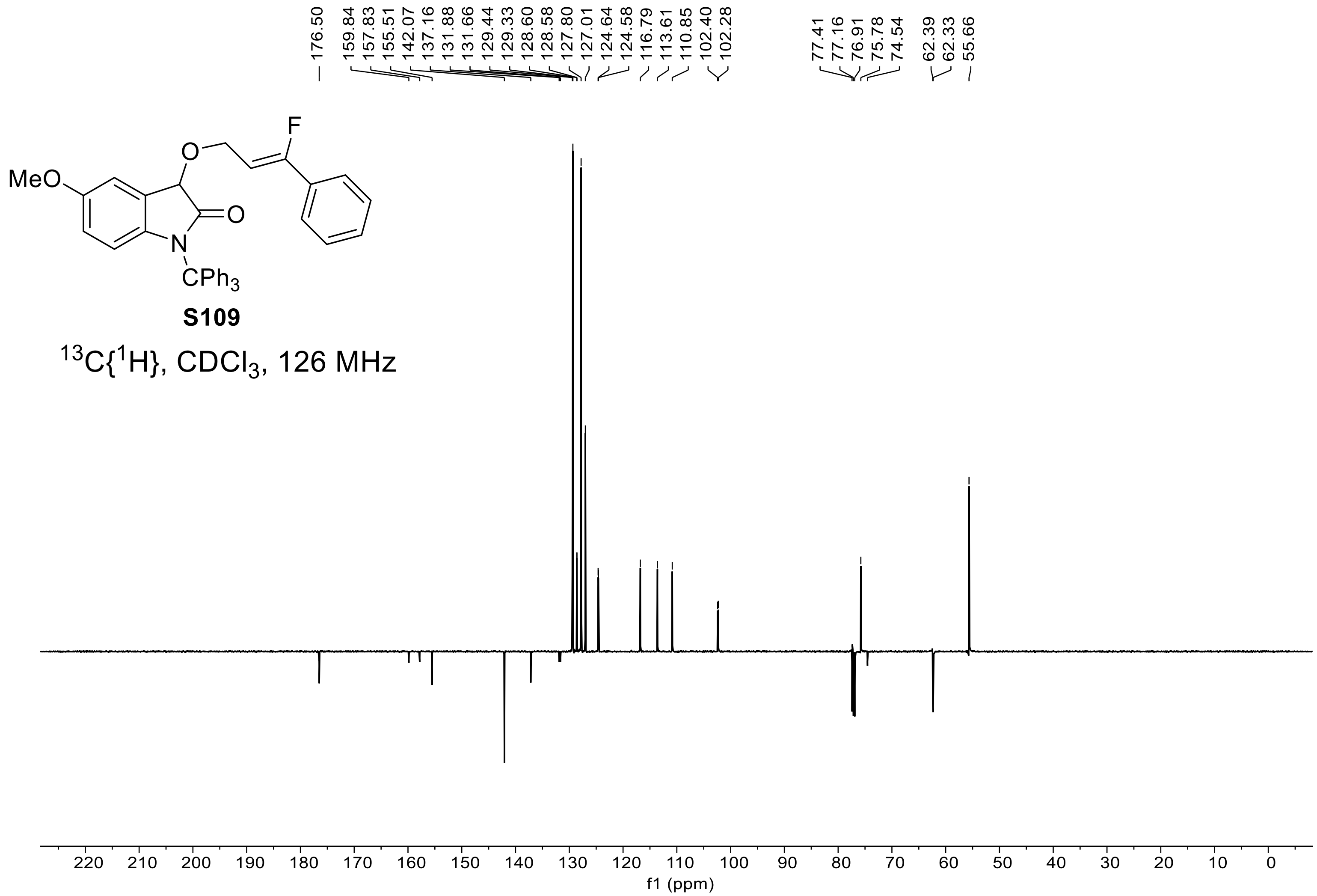

Fig. S276: $^{13}C\{^{1}H\}$ NMR spectrum for (*Z*)-3-[(3-Fluoro-3-phenylallyl)oxy]-5-methoxy-1-tritylindolin-2-one (**S109**).

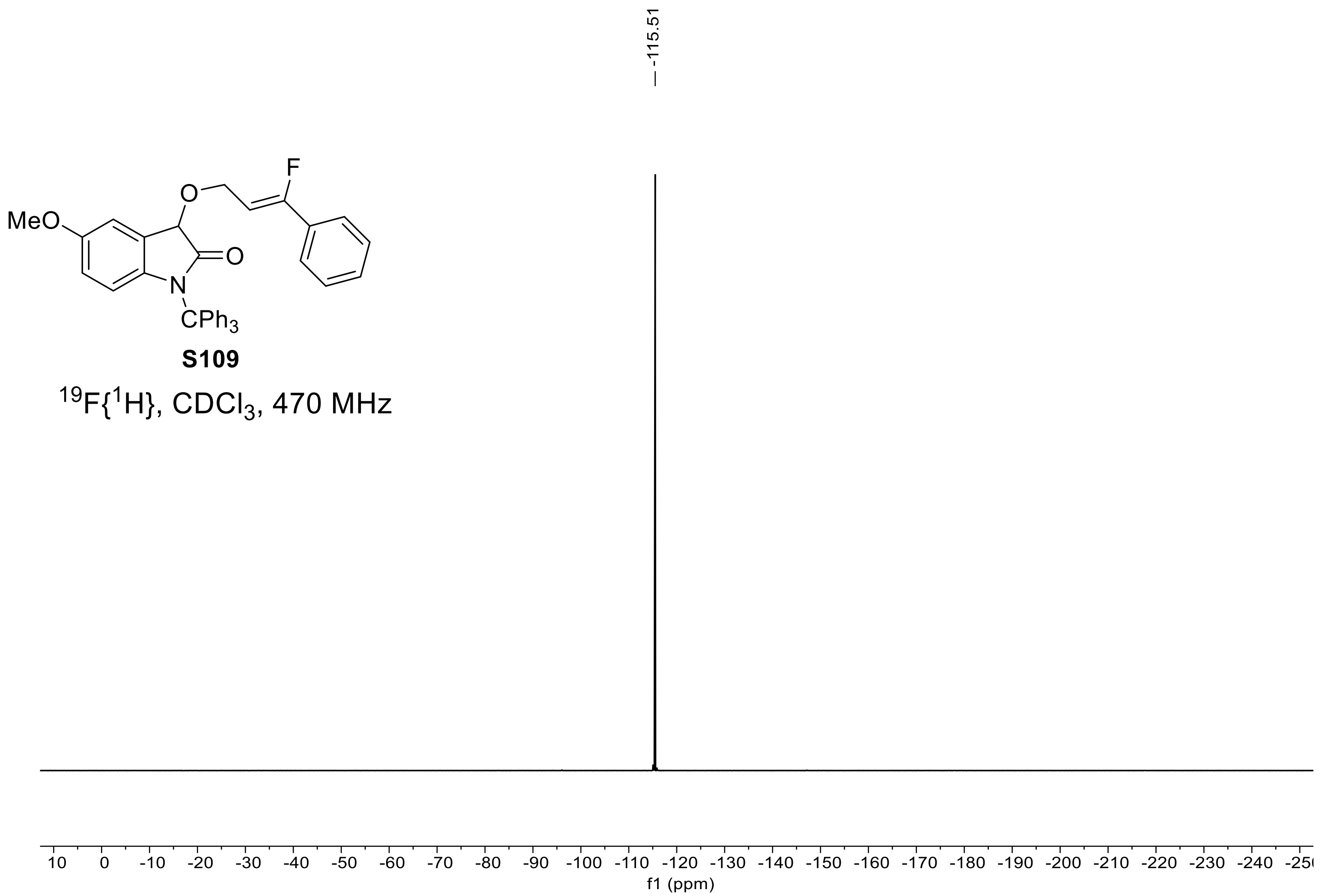

Fig. S277: $^{19}F\{^{1}H\}$ NMR spectrum for (*Z*)-3-[(3-Fluoro-3-phenylallyl)oxy]-5-methoxy-1-tritylindolin-2-one (**S109**).

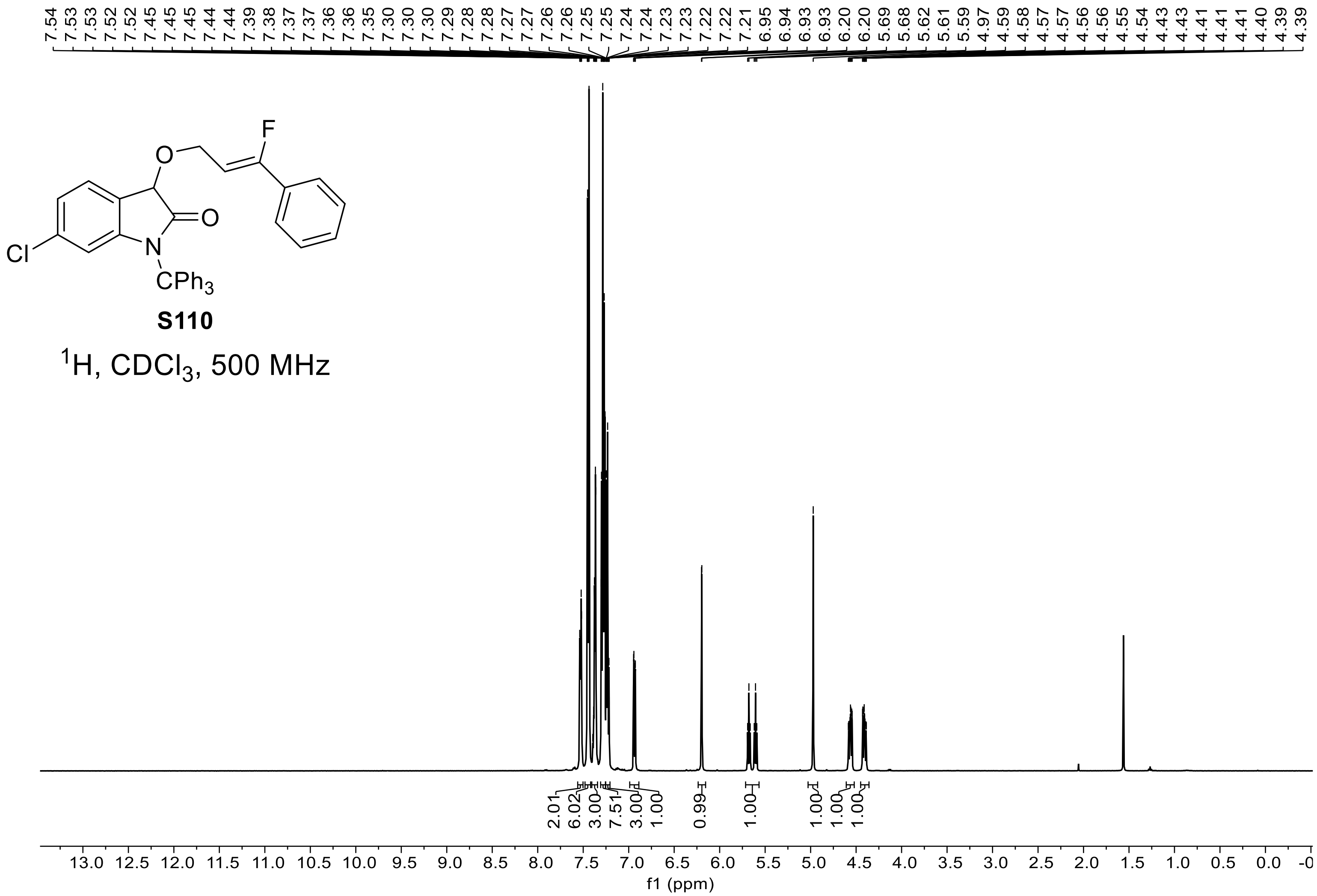

Fig. S278: ^{1}H NMR spectrum for (*Z*)-6-Chloro-3-[(3-fluoro-3-phenylallyl)oxy]-1-tritylindolin-2-one (**S110**).

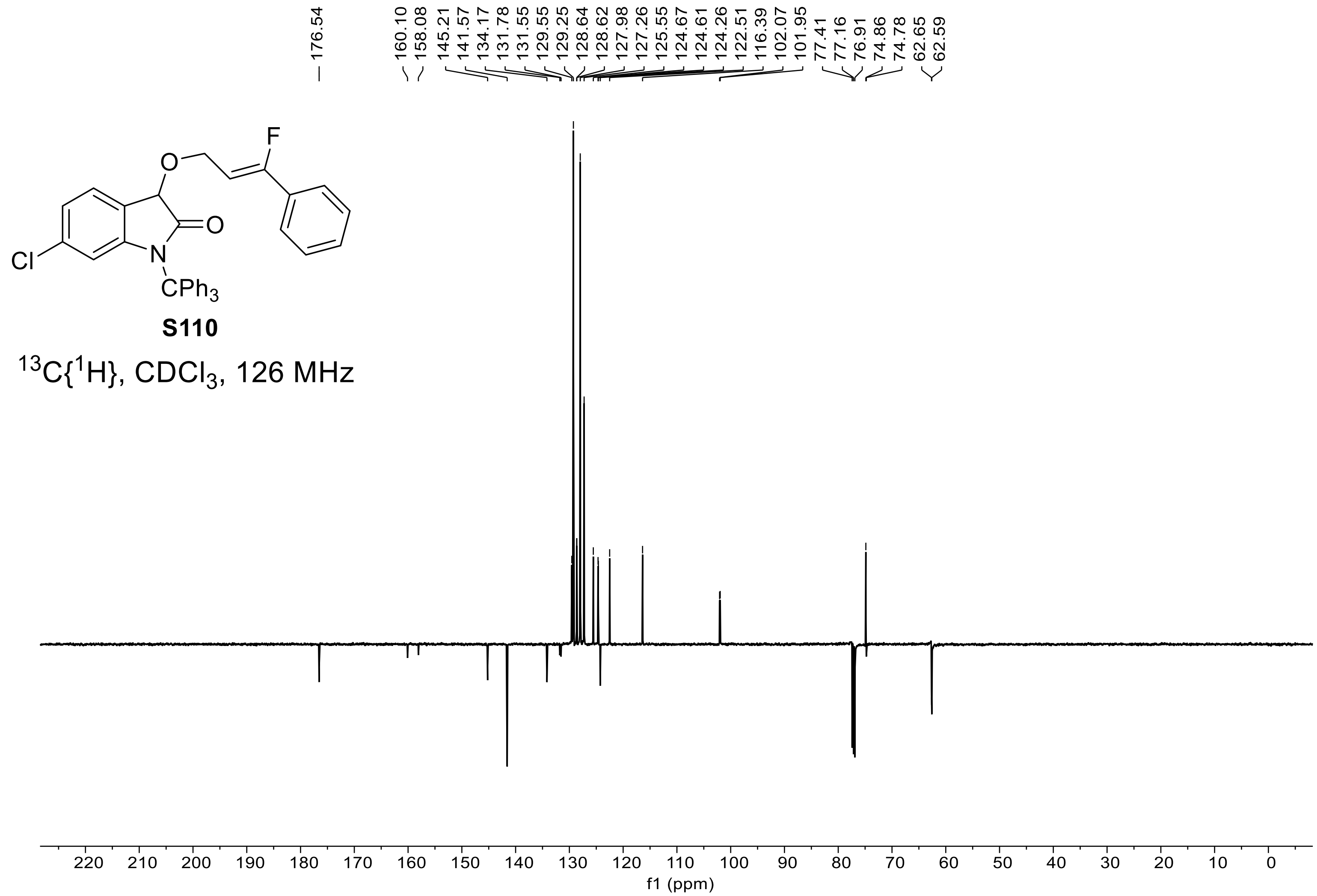

Fig. S279: $^{13}C\{^{1}H\}$ NMR spectrum for (*Z*)-6-Chloro-3-[(3-fluoro-3-phenylallyl)oxy]-1-tritylindolin-2-one (**S110**).

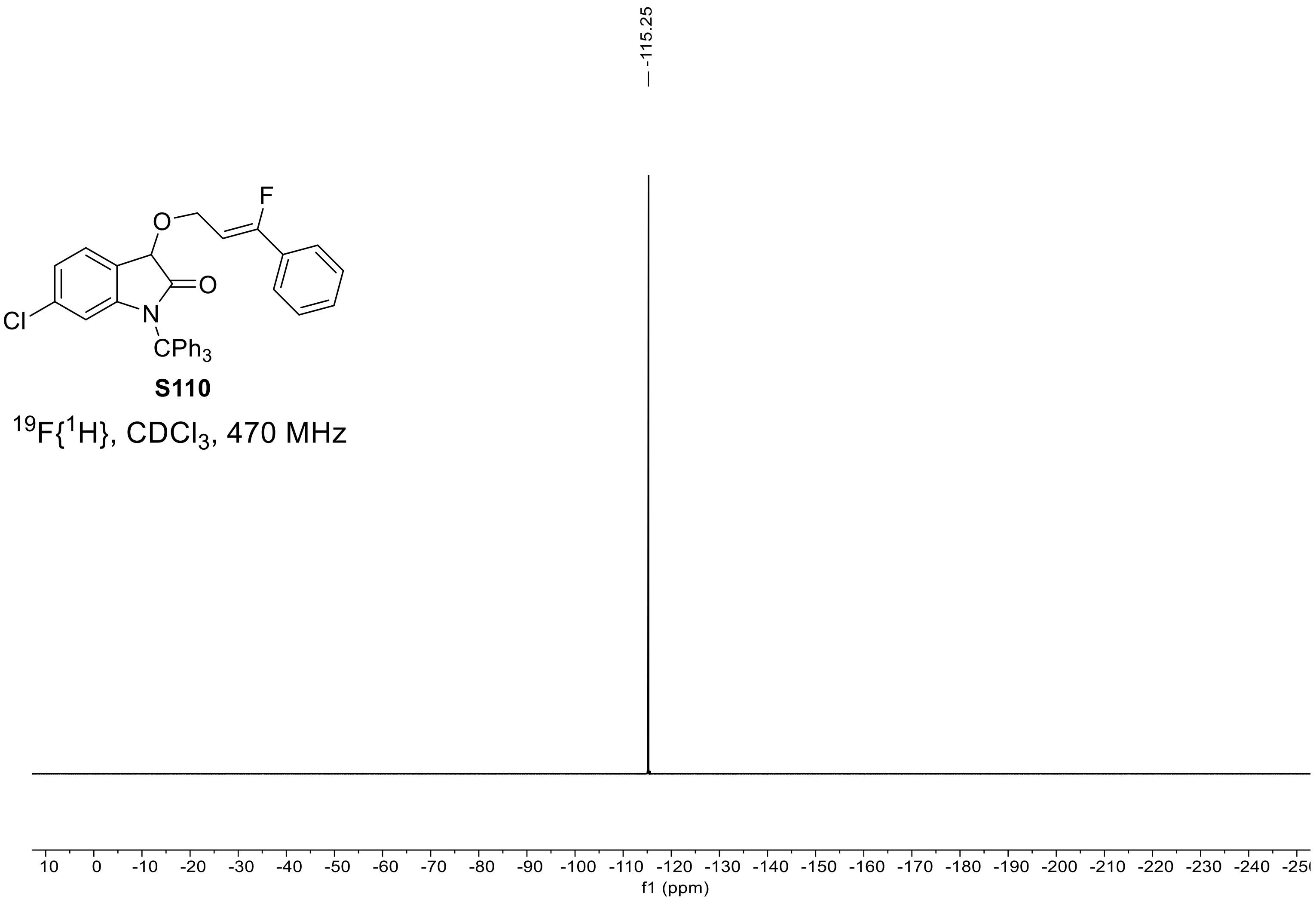

Fig. S280: ^{19}F{^{1}H} NMR spectrum for (*Z*)-6-Chloro-3-[(3-fluoro-3-phenylallyl)oxy]-1-tritylindolin-2-one (**S110**).

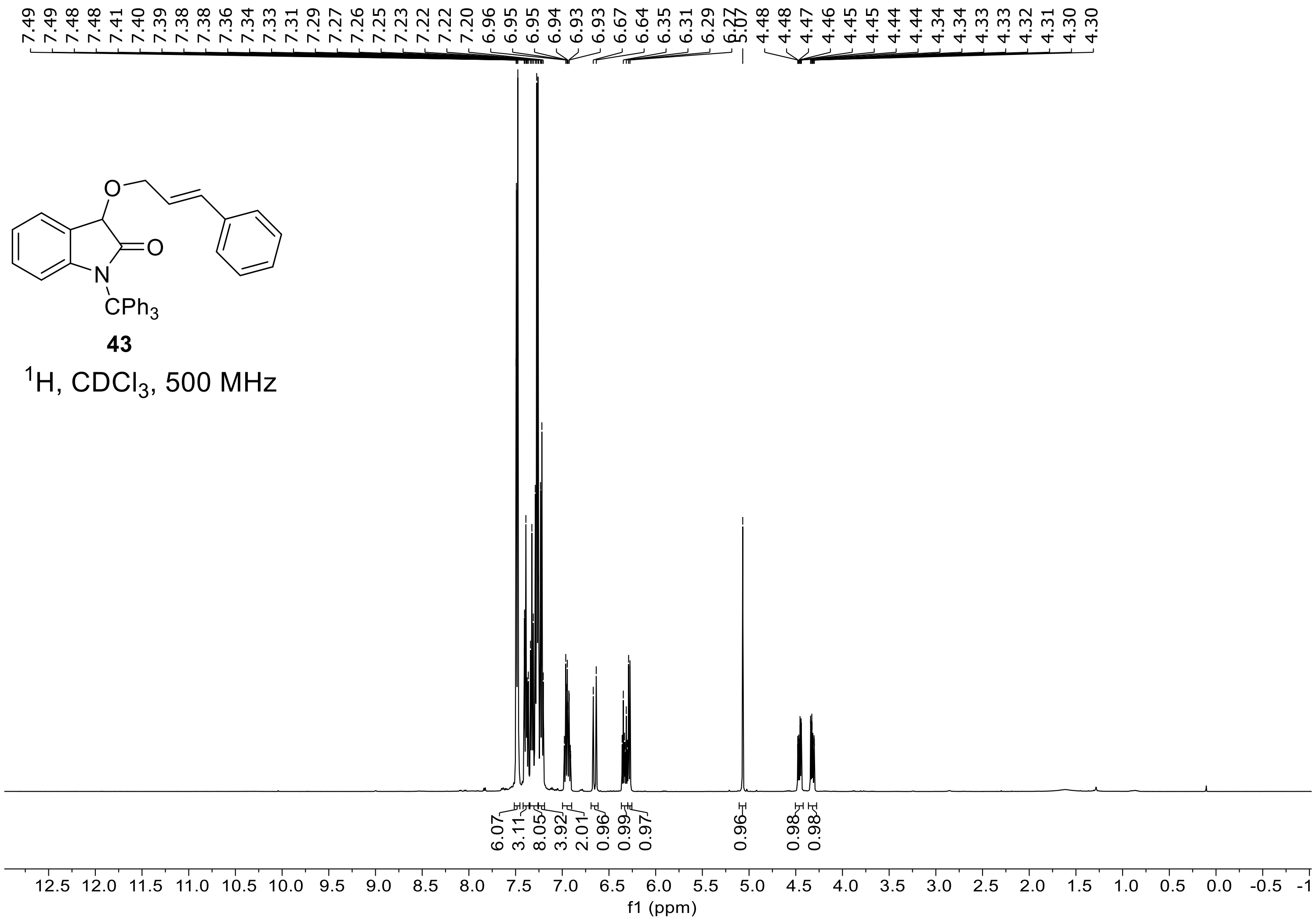

Fig. S281: ^{1}H NMR spectrum for (*E*)-3-(Cinnamyloxy)-1-tritylindolin-2-one (**43**).

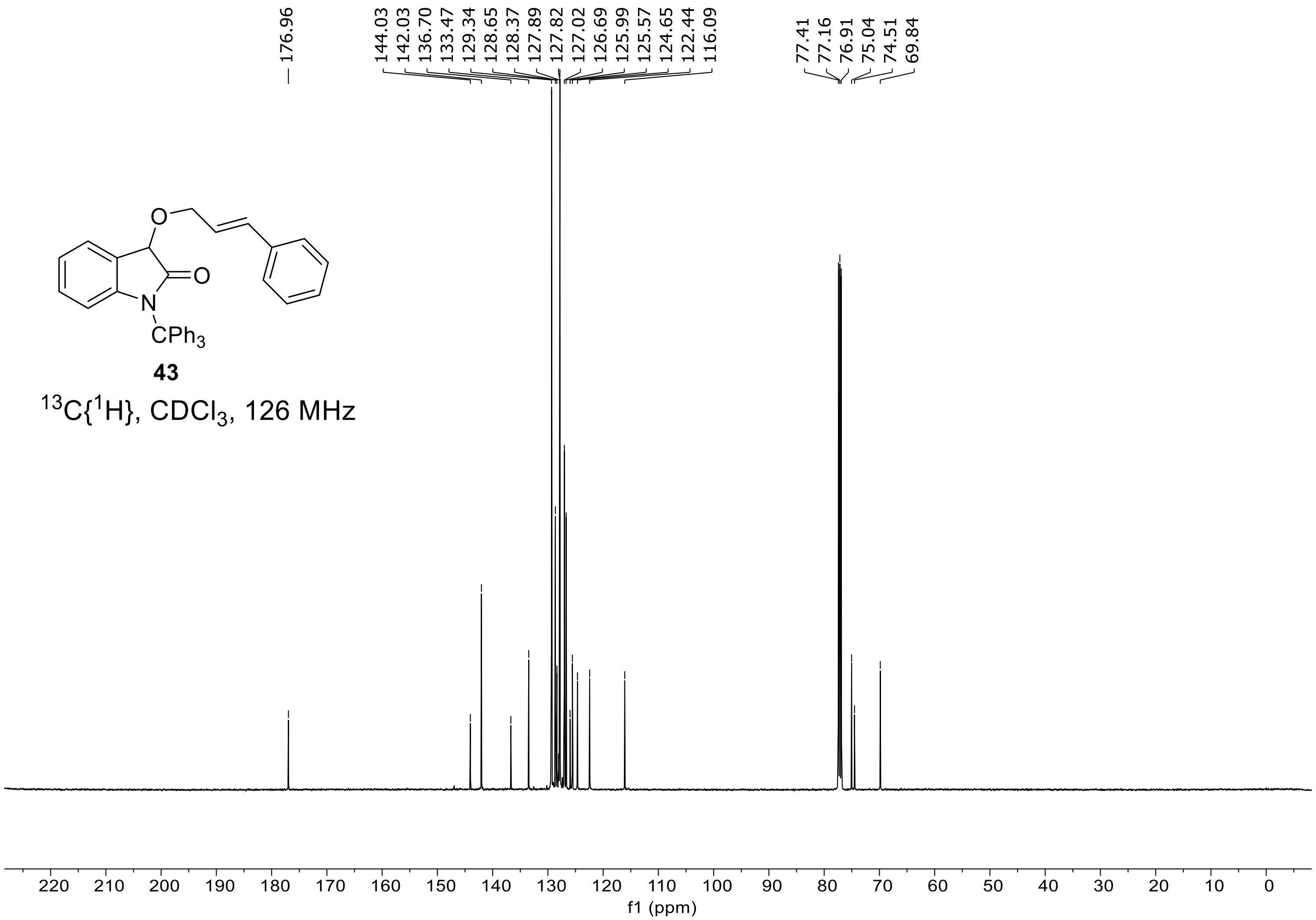

Fig. S282: $^{13}C\{^{1}H\}$ NMR spectrum for (*E*)-3-(Cinnamyloxy)-1-tritylindolin-2-one (**43**).

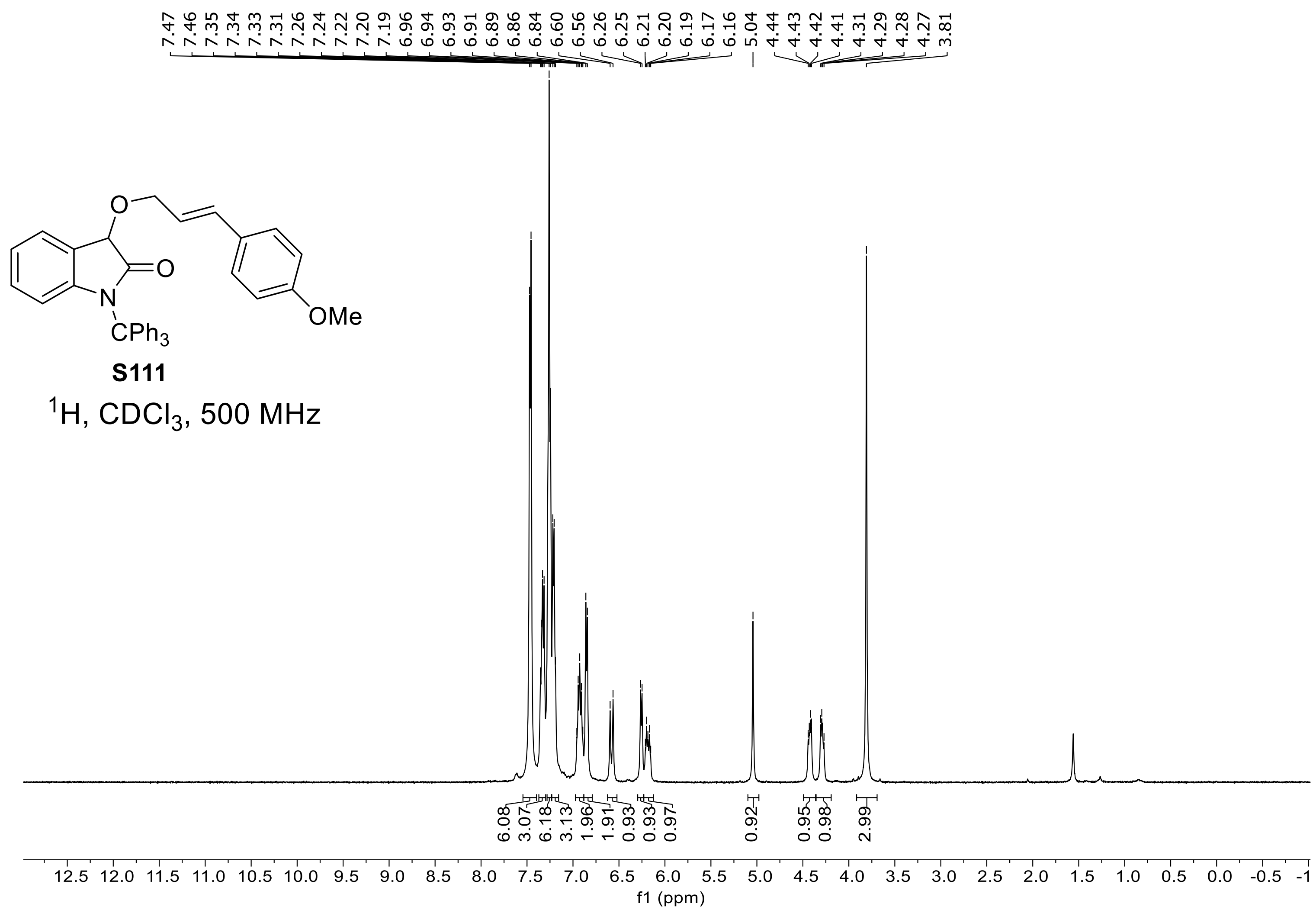

Fig. S283: ^{1}H NMR spectrum for (*E*)-3-{[3-(4-methoxyphenyl)allyl]oxy}-1-tritylindolin-2-one (**S111**).

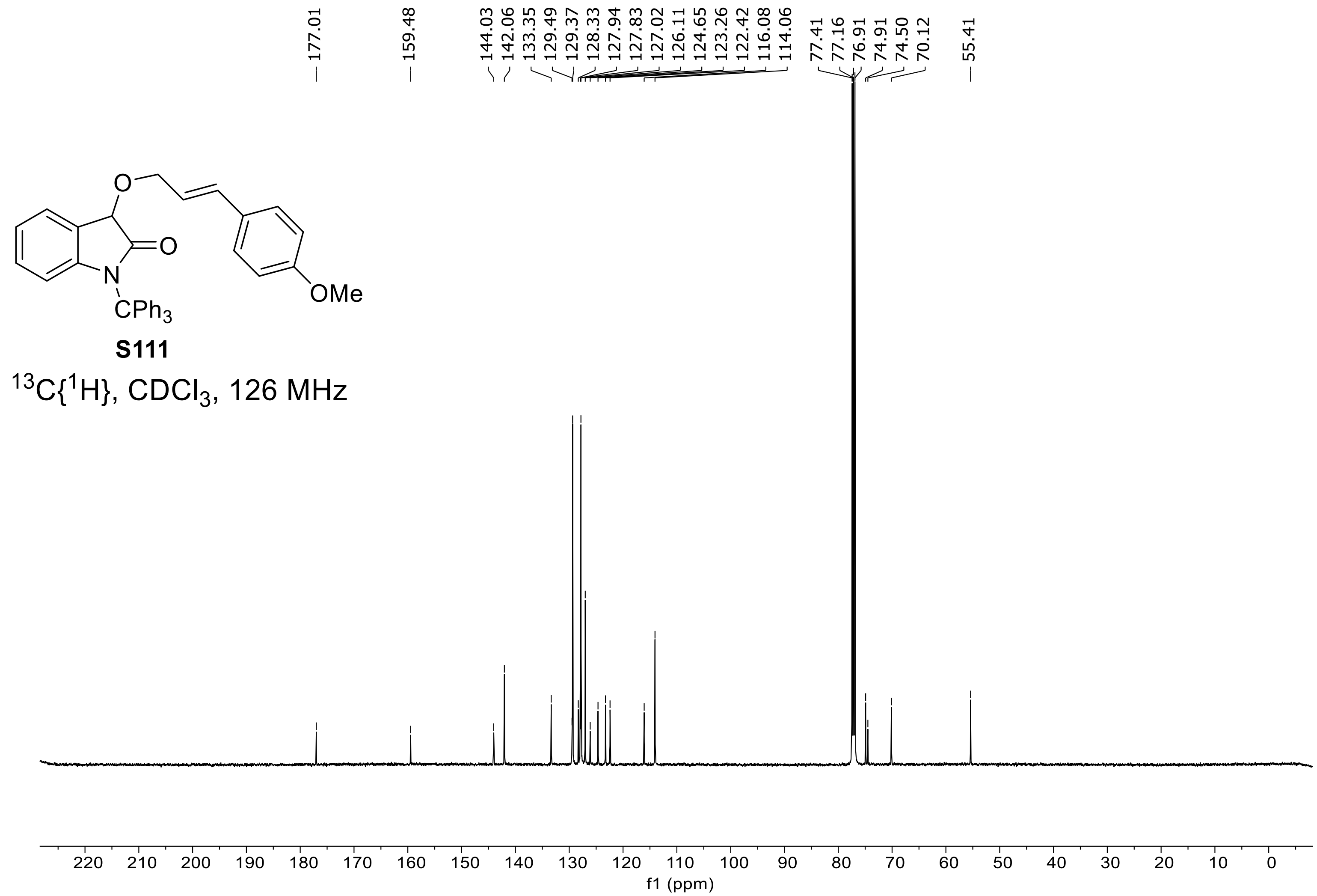

Fig. S284: $^{13}C\{^{1}H\}$ NMR spectrum for (*E*)-3-{[3-(4-methoxyphenyl)allyl]oxy}-1-tritylindolin-2-one (**S111**).

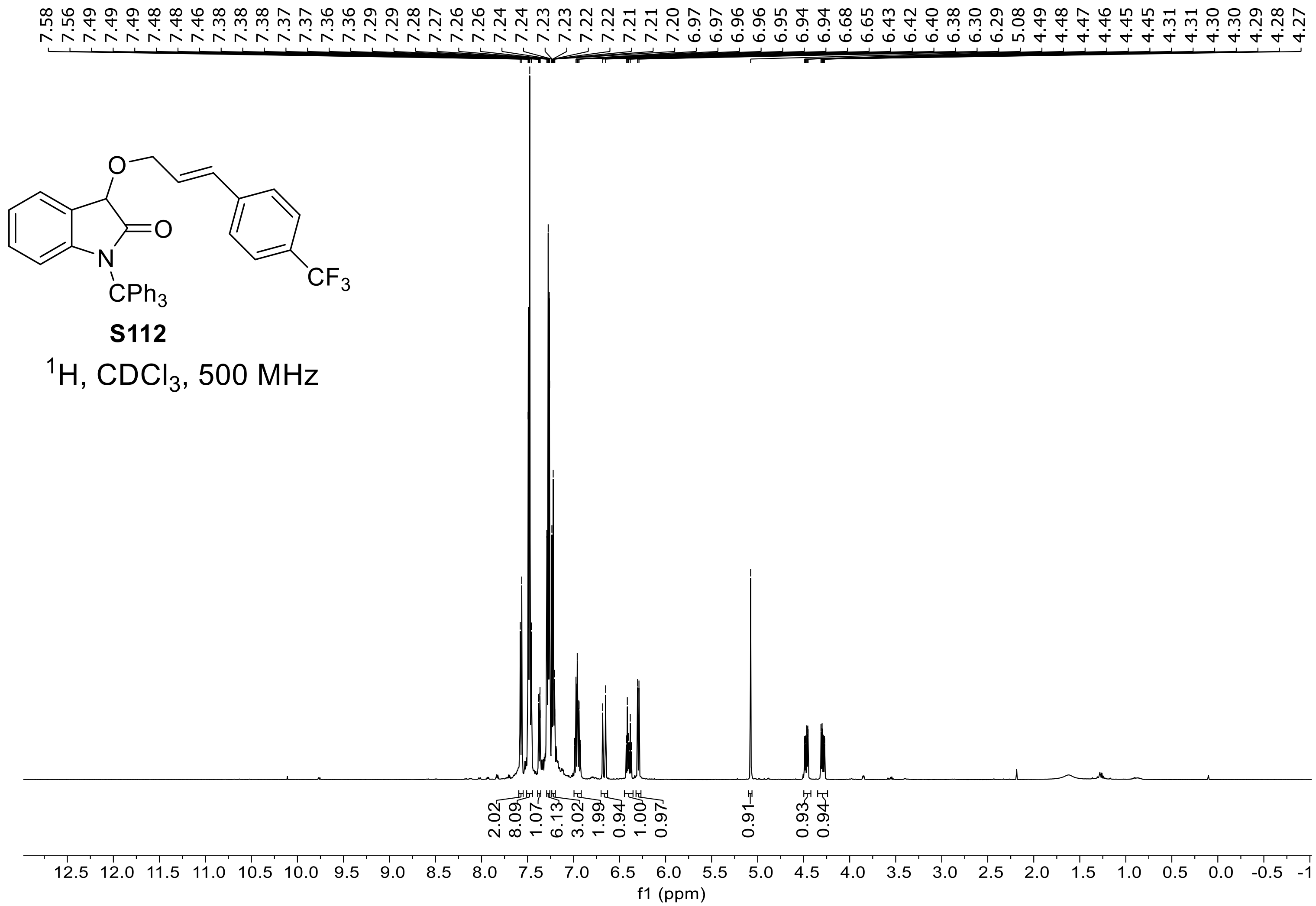

Fig. S285: ^{1}H NMR spectrum for (*E*)-3-{[3-(4-(trifluoromethyl)phenyl)allyl]oxy}-1-tritylindolin-2-one (**S112**).

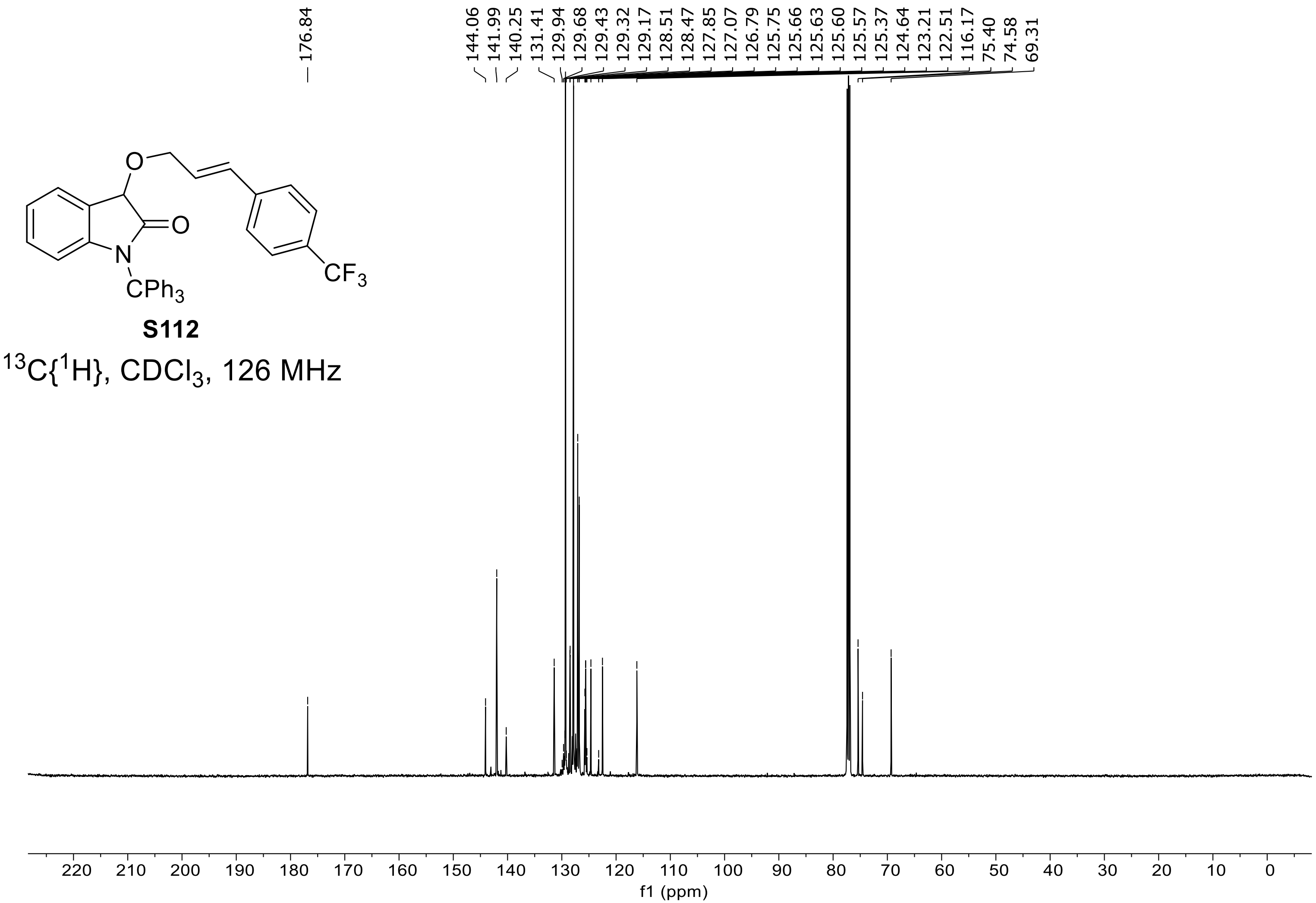

Fig. S286: $^{13}C\{^1H\}$ NMR spectrum for (*E*)-3-{[3-(4-(trifluoromethyl)phenyl)allyl]oxy}-1-tritylindolin-2-one (**S112**).

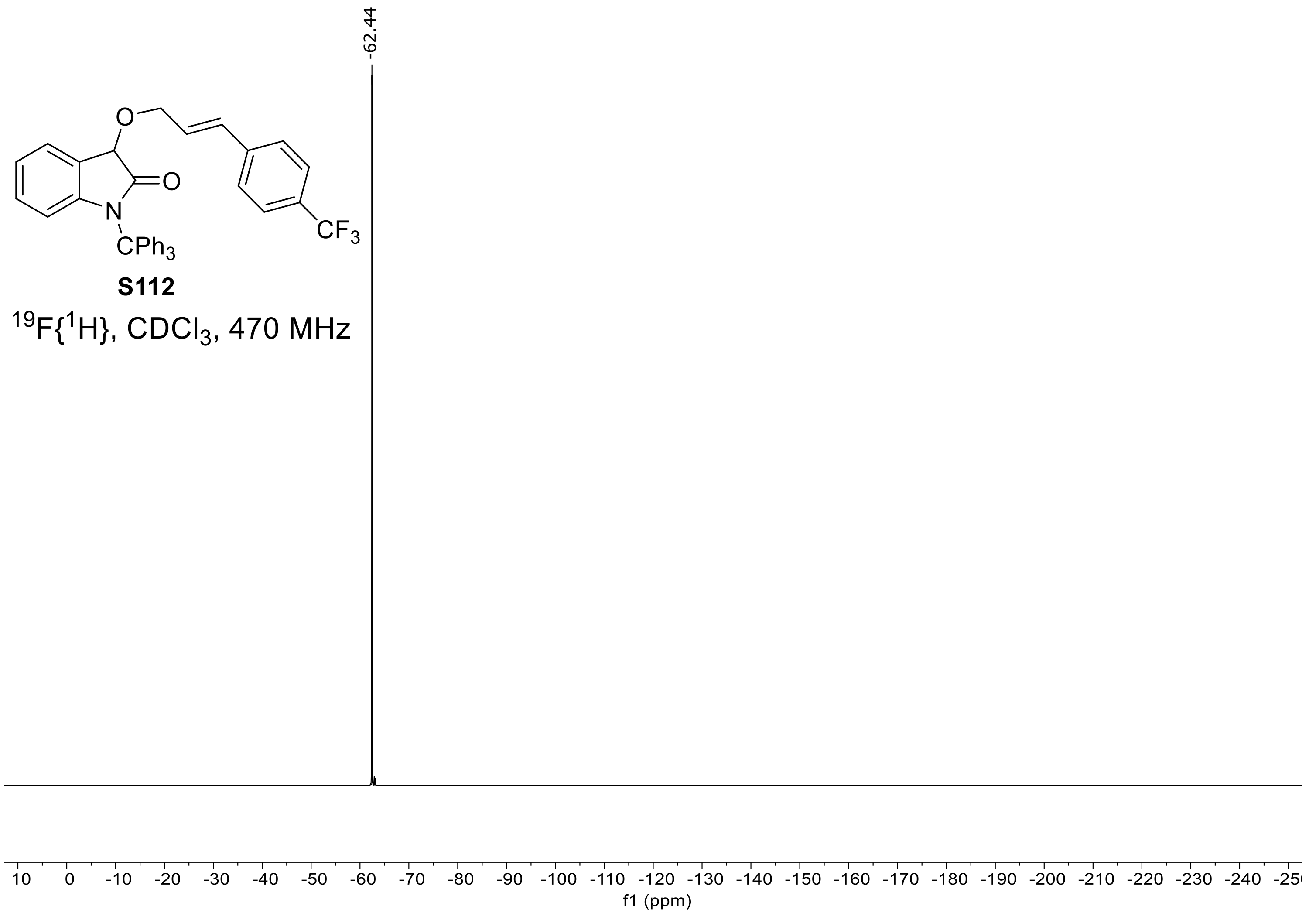

Fig. S287: $^{19}F\{^{1}H\}$ NMR spectrum for (*E*)-3-{[3-(4-(trifluoromethyl)phenyl)allyl]oxy}-1-tritylindolin-2-one (**S112**).

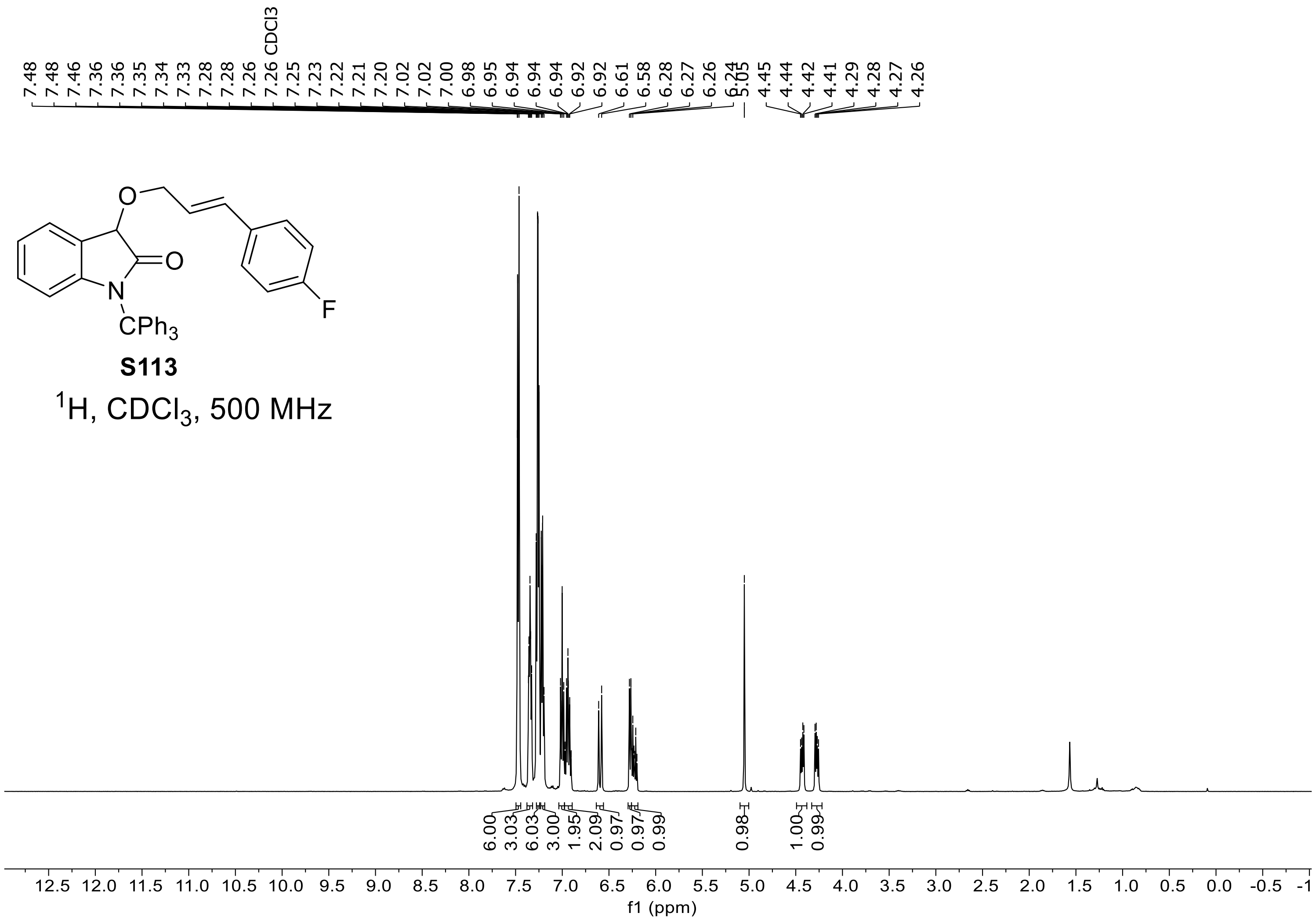

Fig. S288: ^{1}H NMR spectrum for (*E*)-3-{[3-(4-Fluorophenyl)allyl]oxy}-1-tritylindolin-2-one (**S113**).

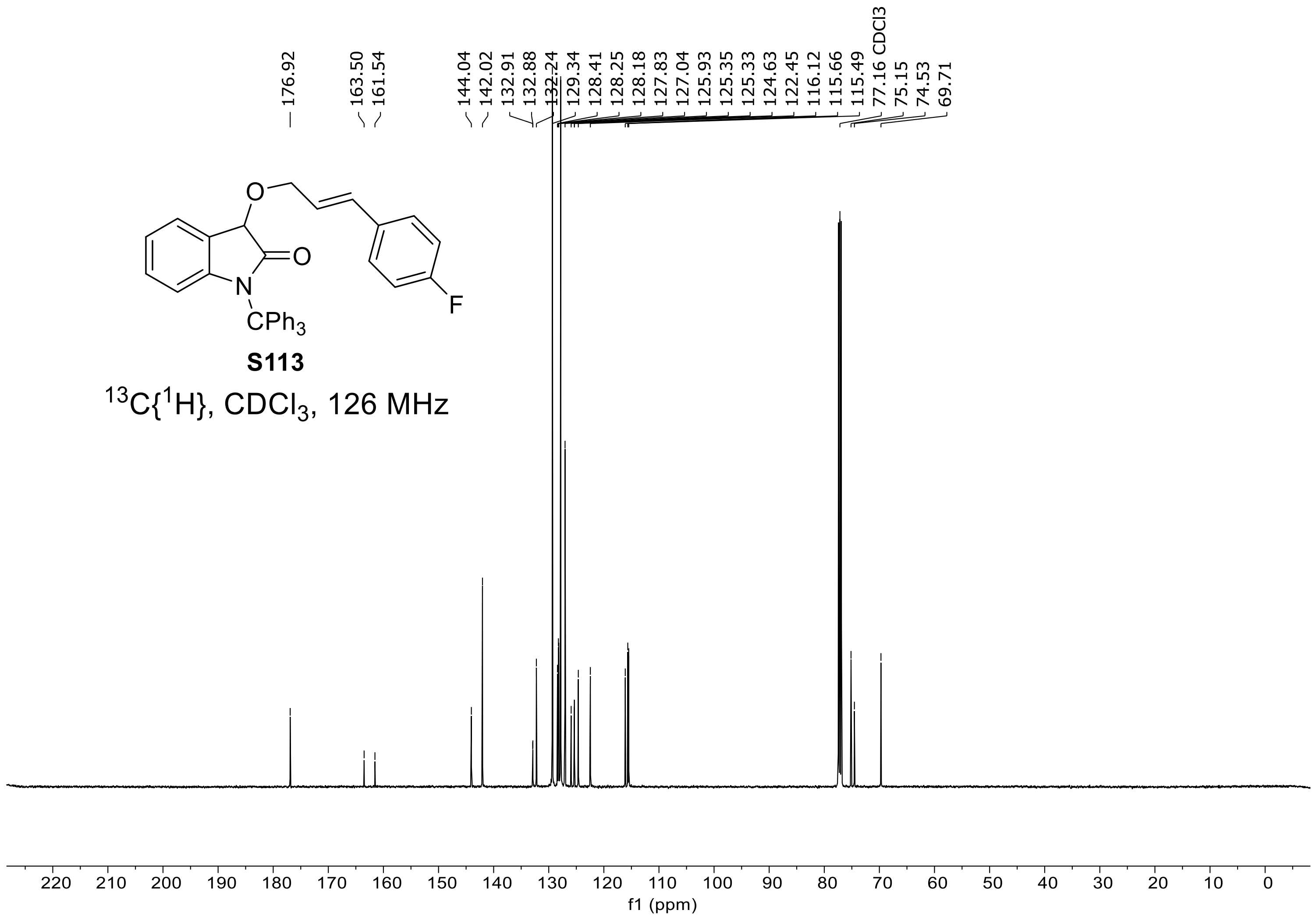

Fig. S289: $^{13}C\{^{1}H\}$ NMR spectrum (*E*)-3-{[3-(4-Fluorophenyl)allyl]oxy}-1-tritylindolin-2-one (**S113**).

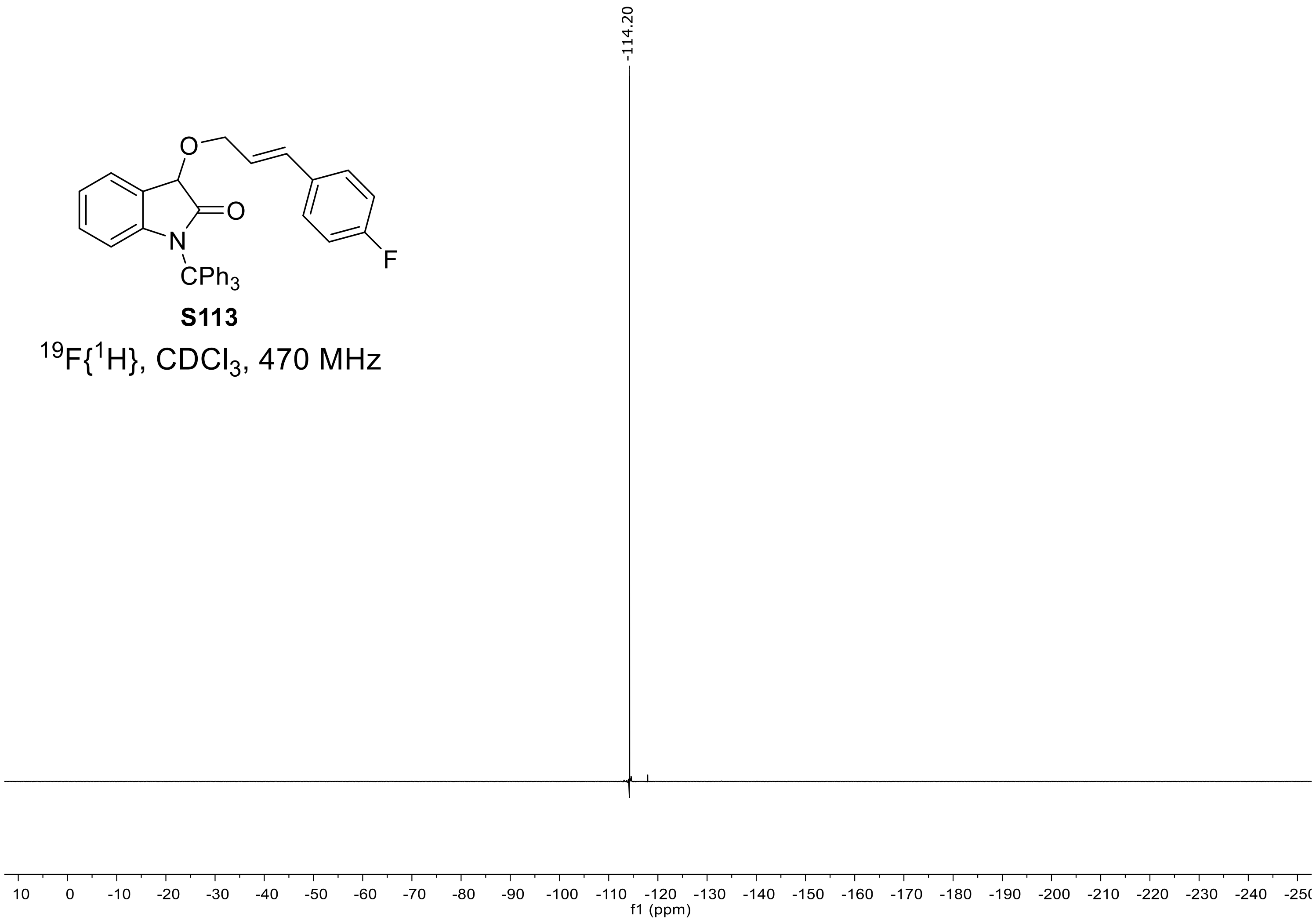

Fig. S290: $^{19}F\{^{1}H\}$ NMR spectrum for (*E*)-3-{[3-(4-Fluorophenyl)allyl]oxy}-1-tritylindolin-2-one (**S113**).

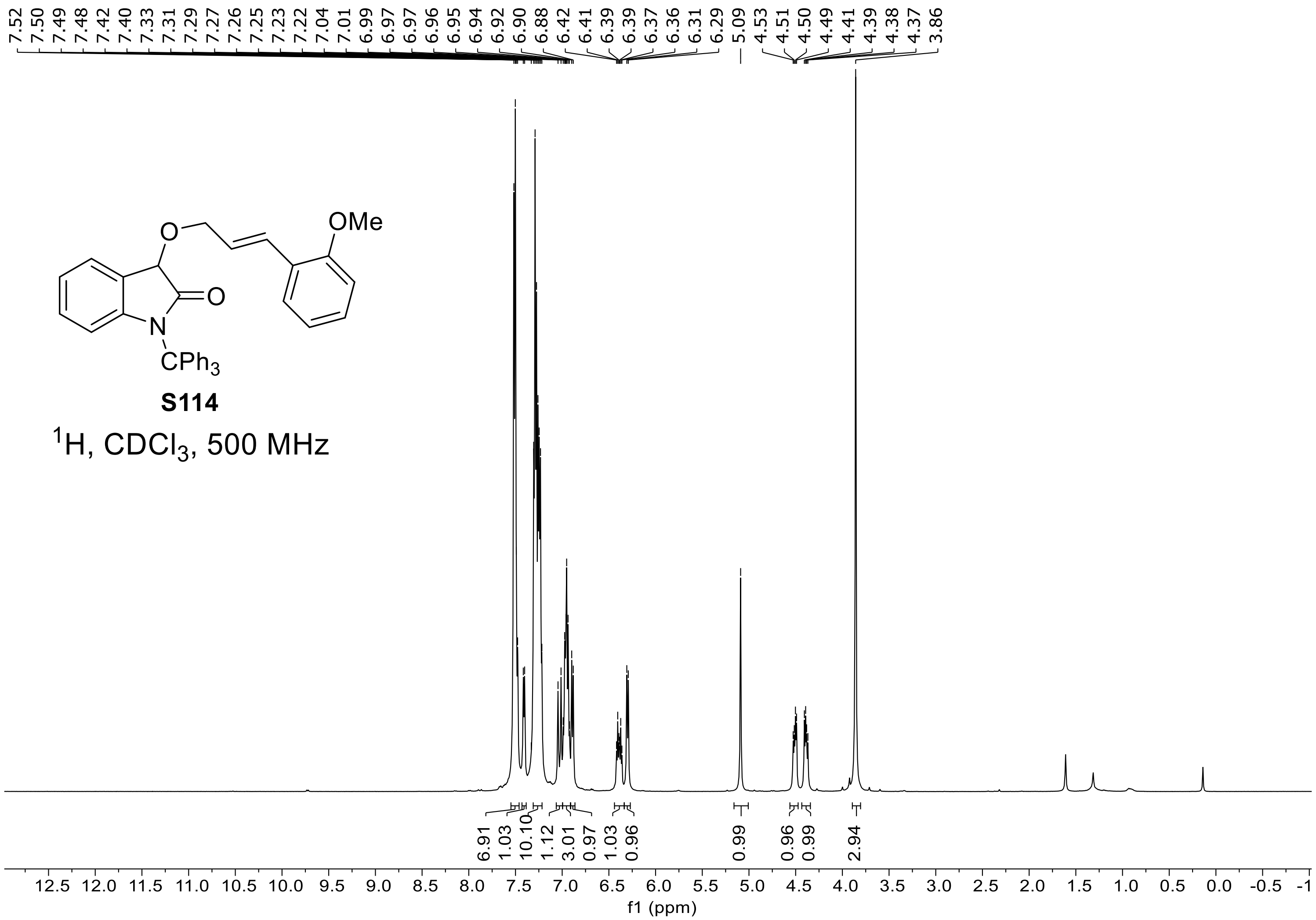

Fig. S291: ^{1}H NMR spectrum for (*E*)-3-{[3-(2-Methoxyphenyl)allyl]oxy}-1-tritylindolin-2-one (**S114**).

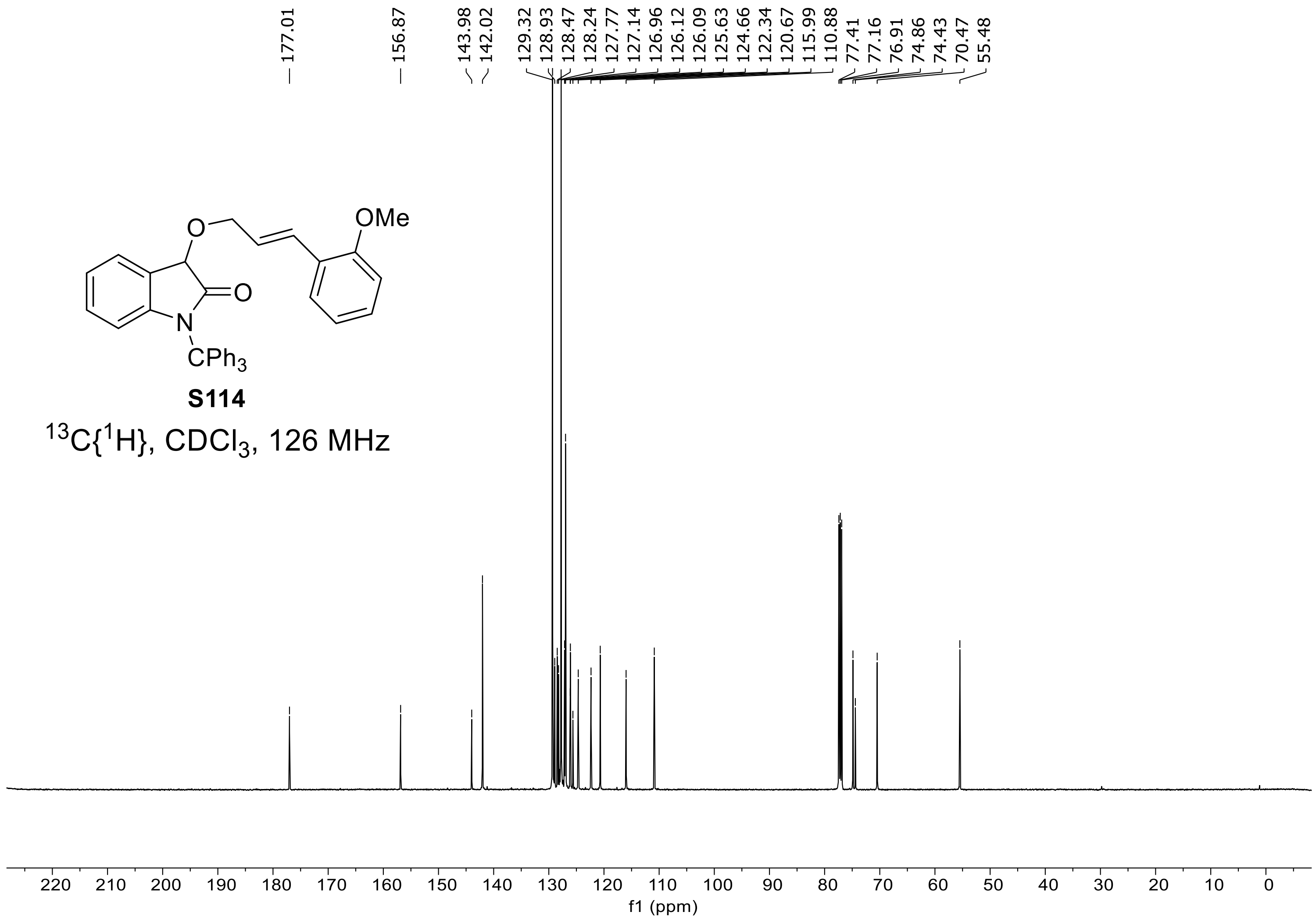

Fig. S292: $^{13}C\{^{1}H\}$ NMR spectrum for (*E*)-3-{[3-(2-Methoxyphenyl)allyl]oxy}-1-tritylindolin-2-one (**S114**).

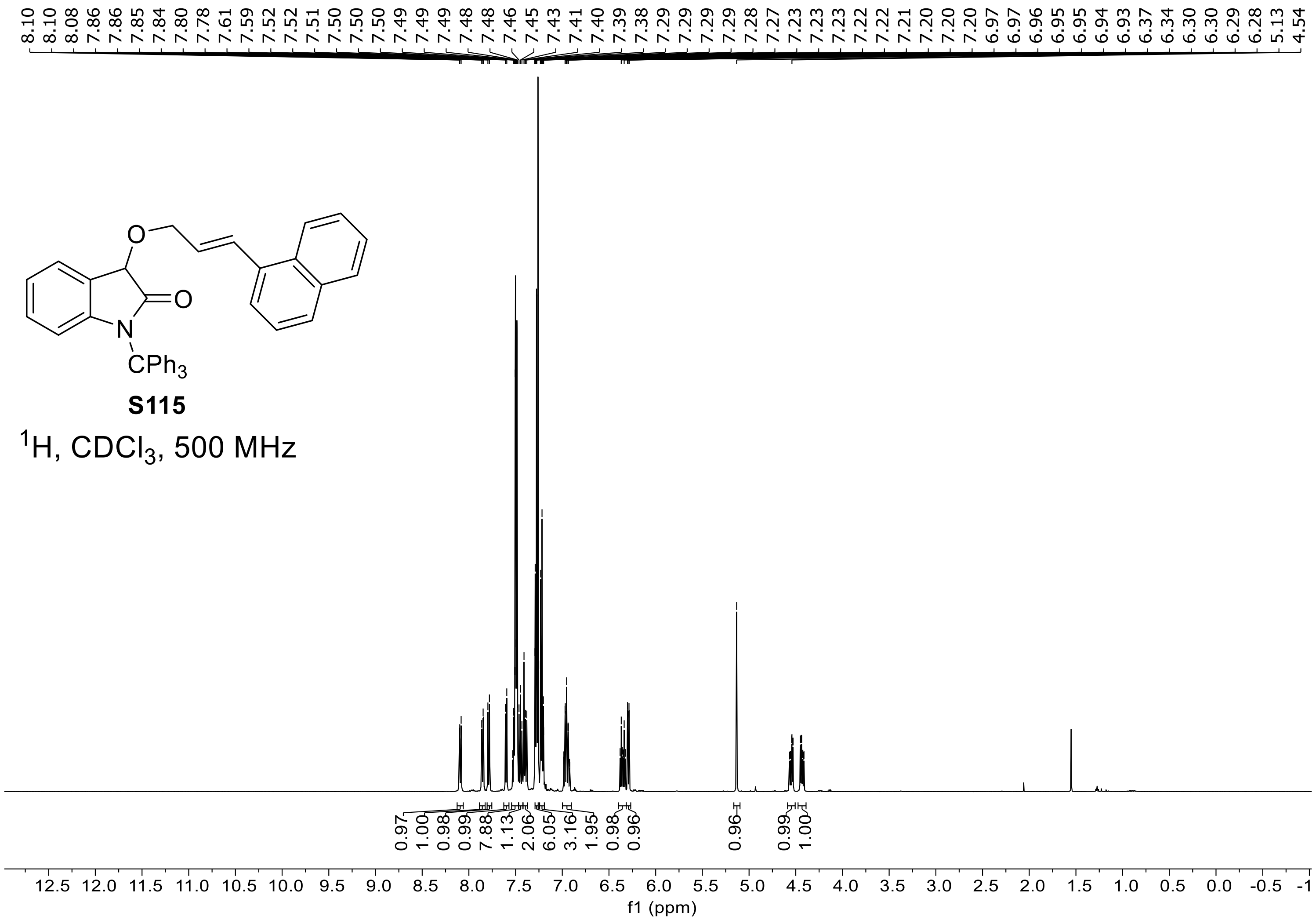

Fig. S293: ^{1}H NMR spectrum for (*E*)-3-{[3-(Naphthalen-1-yl)allyl]oxy}-1-tritylindolin-2-one (**S115**).

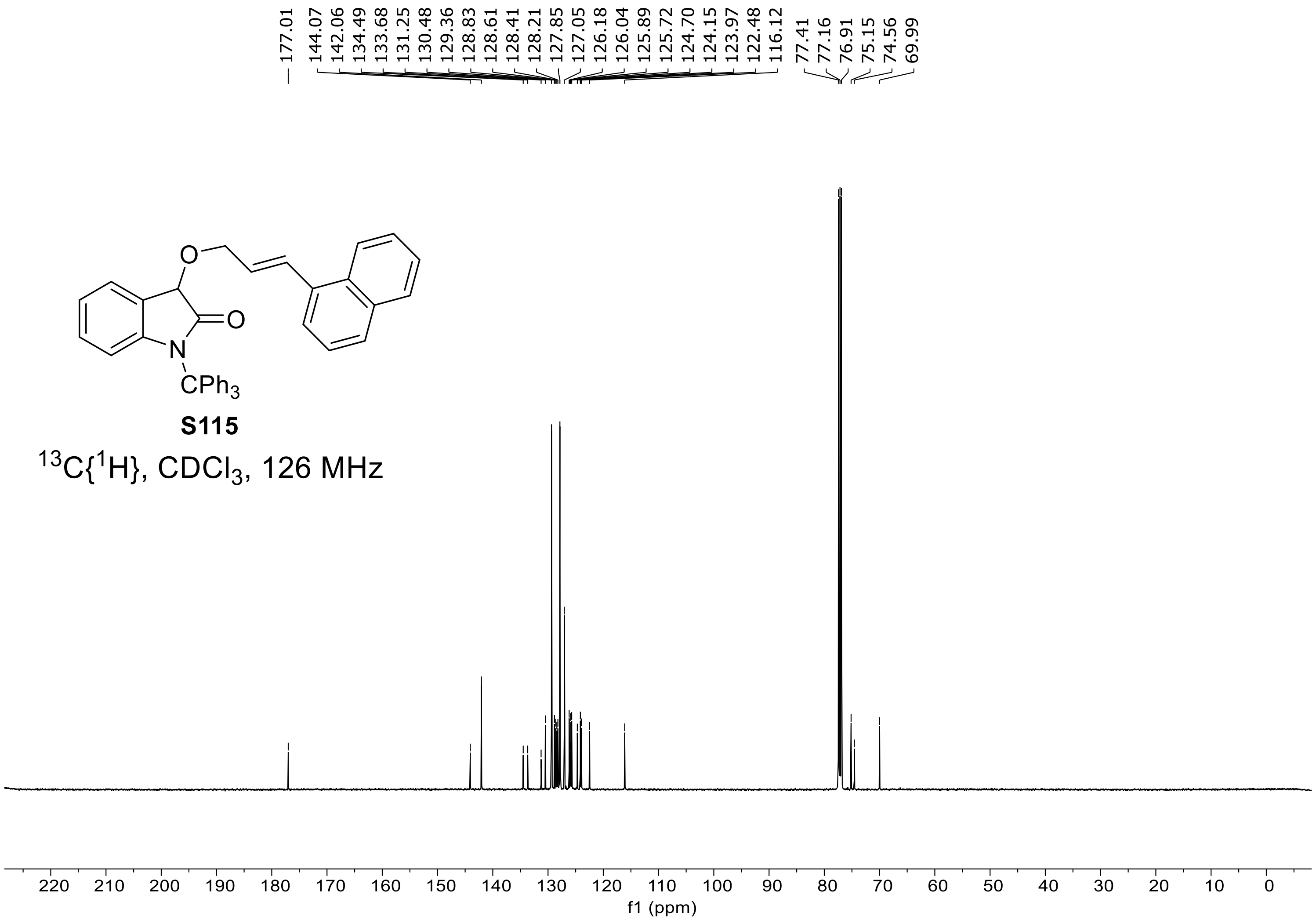

Fig. S294: $^{13}C\{^1H\}$ NMR spectrum for (*E*)-3-{[3-(Naphthalen-1-yl)allyl]oxy}-1-tritylindolin-2-one (**S115**).

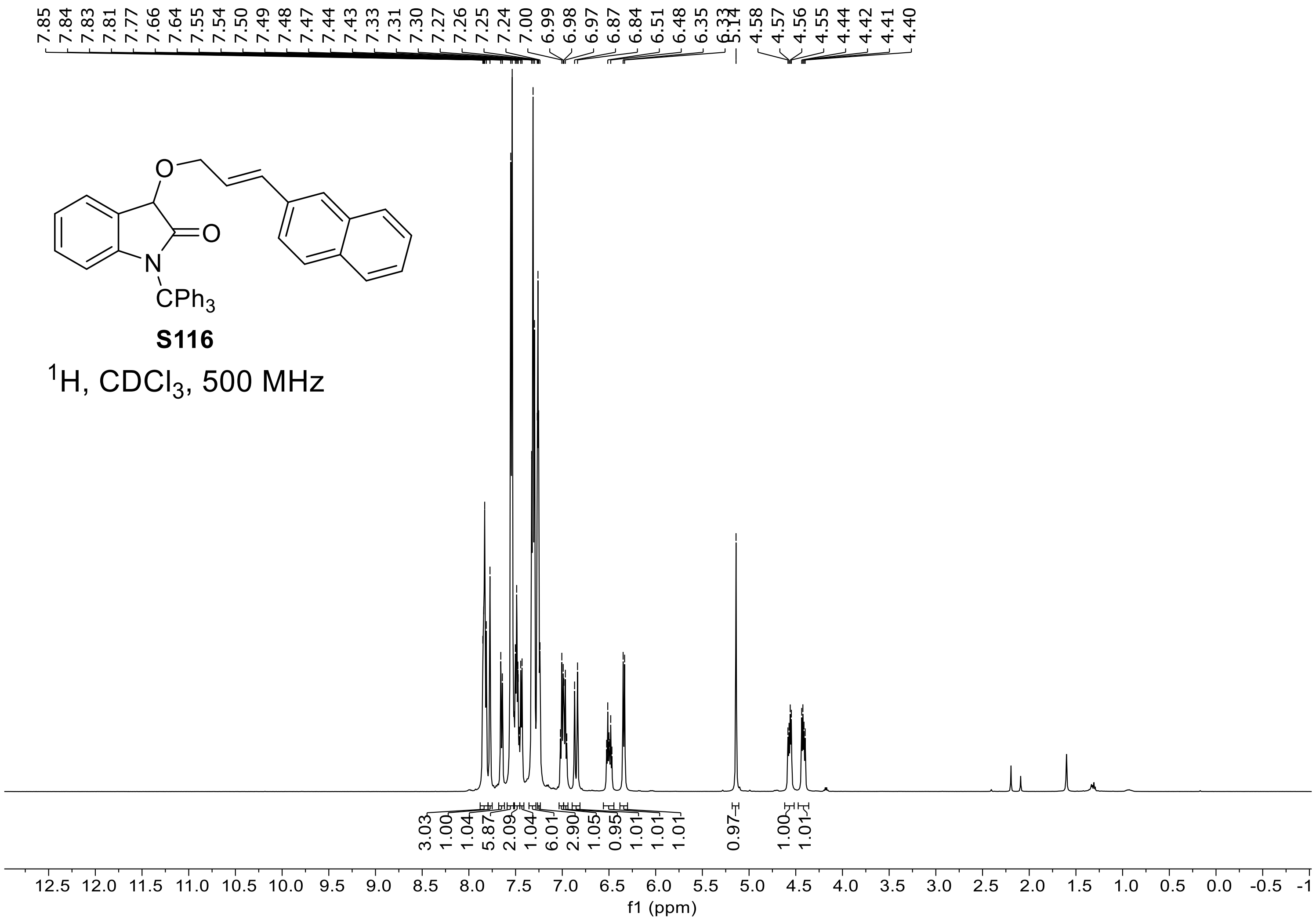

Fig. S295: ^{1}H NMR spectrum for (*E*)-3-{[3-(Naphthalen-2-yl)allyl]oxy}-1-tritylindolin-2-one (**S116**).

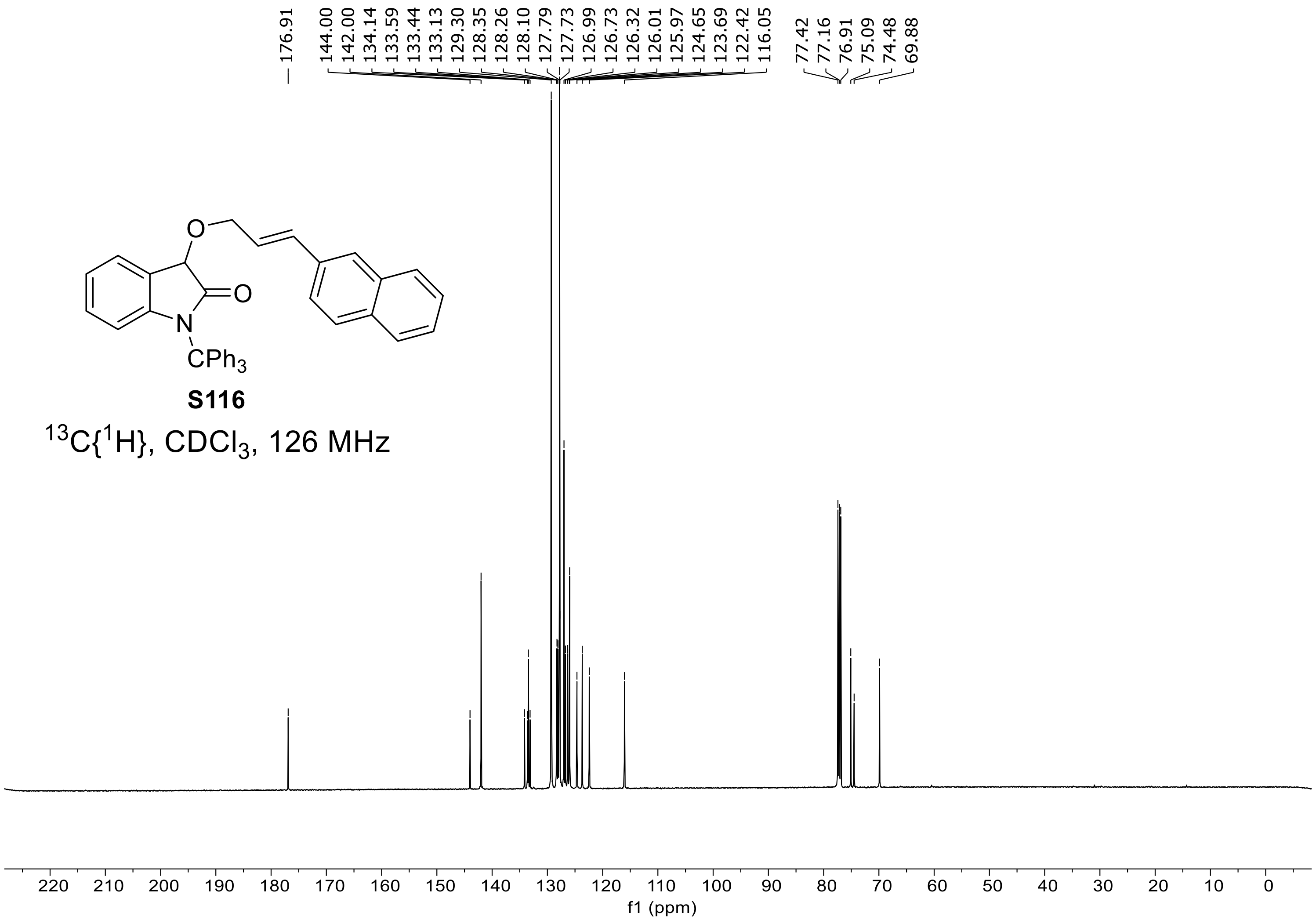

Fig. S296: $^{13}C\{^{1}H\}$ NMR spectrum for (*E*)-3-{[3-(Naphthalen-2-yl)allyl]oxy}-1-tritylindolin-2-one (**S116**).

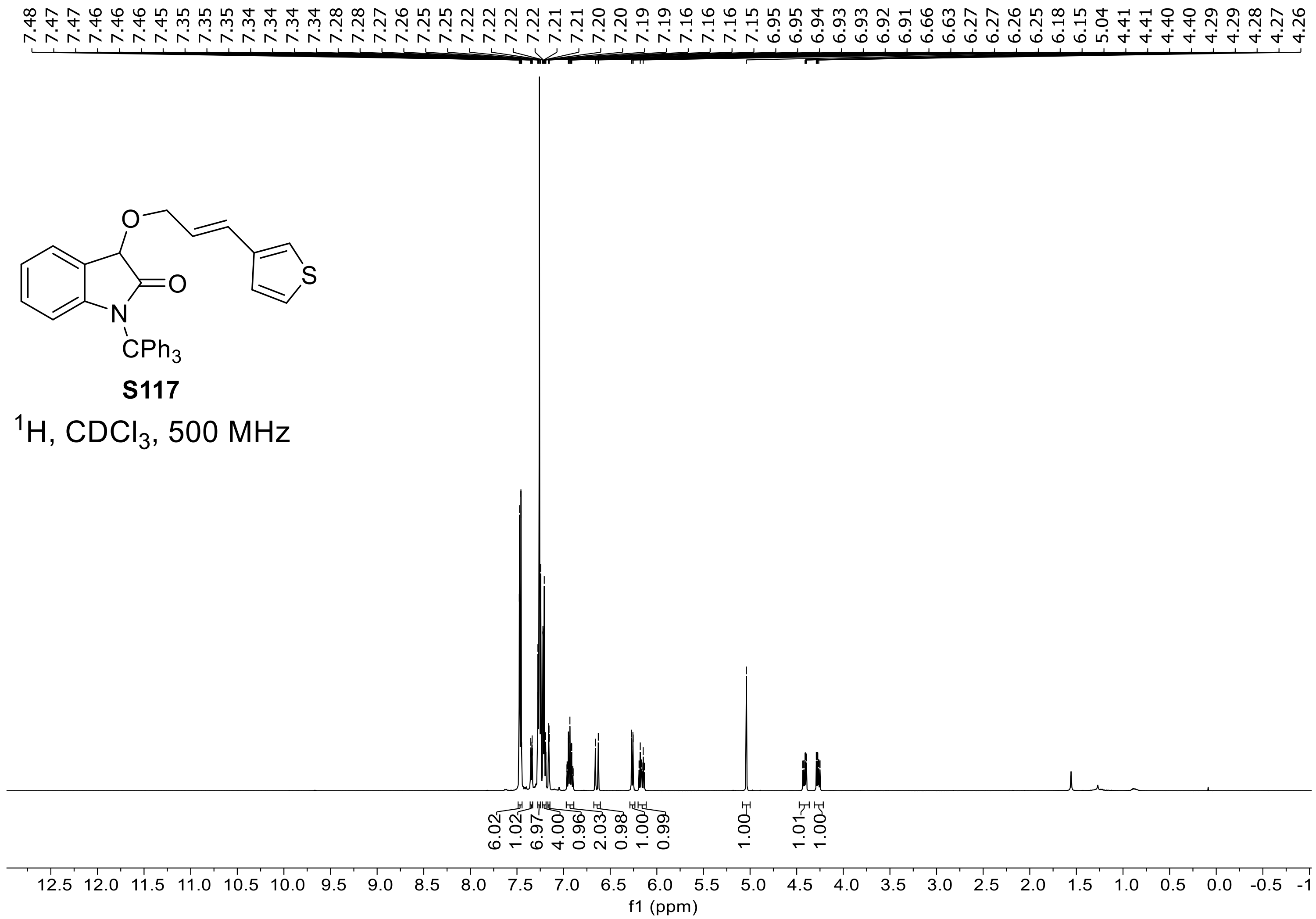

Fig. S297: ^{1}H NMR spectrum for (*E*)-3-{[3-(Thiophen-3-yl)allyl]oxy}-1-tritylindolin-2-one (**S117**).

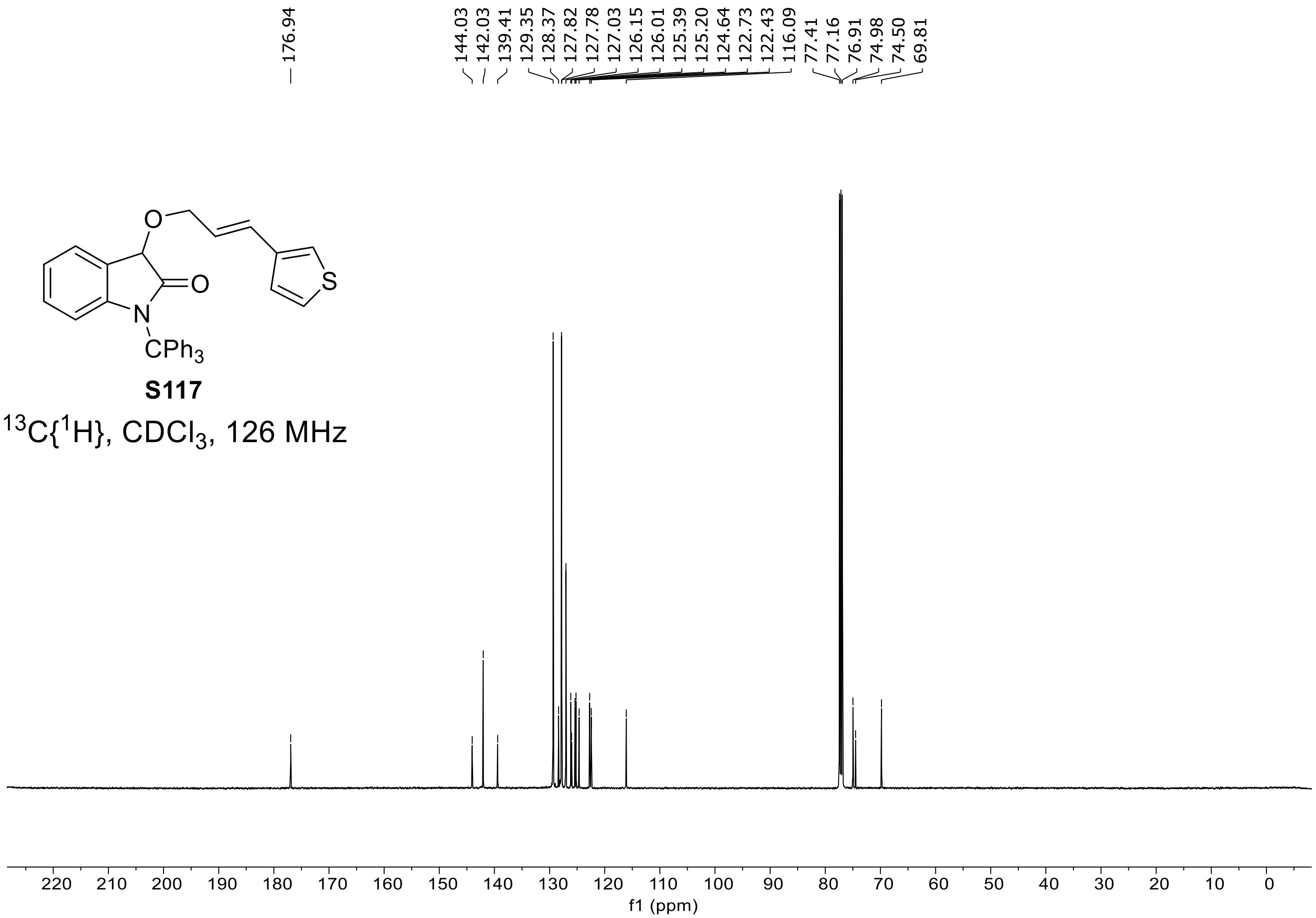

Fig. S298: $^{13}C\{^1H\}$ NMR spectrum for (*E*)-3-{[3-(Thiophen-3-yl)allyl]oxy}-1-tritylindolin-2-one (**S117**).

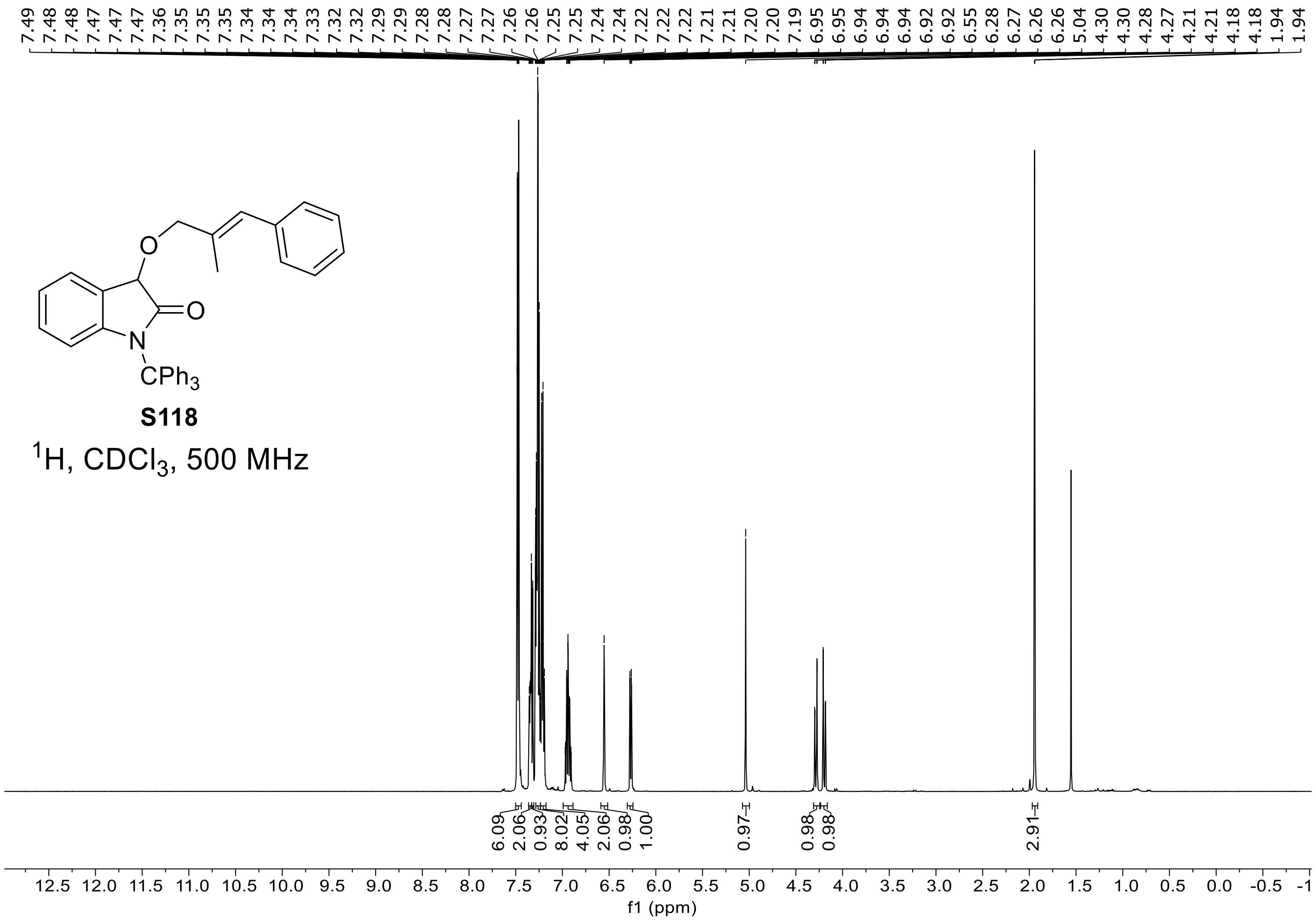

Fig. S299: ^{1}H NMR spectrum for (*E*)-3-[(2-Methyl-3-phenylallyl)oxy)-1-tritylindolin-2-one (**S118**).

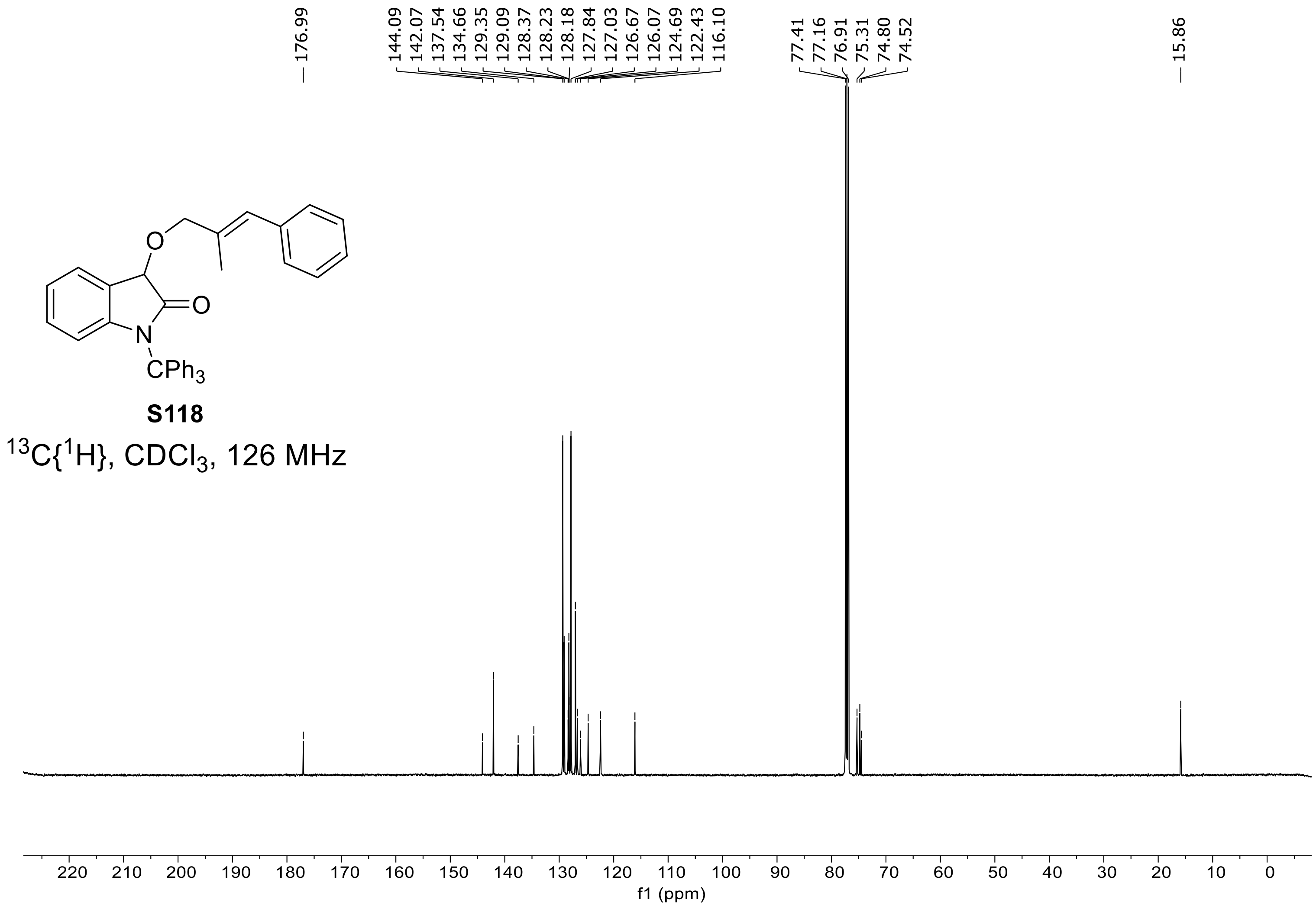

Fig. S300: $^{13}C\{^{1}H\}$ NMR spectrum for (*E*)-3-[(2-Methyl-3-phenylallyl)oxy)-1-tritylindolin-2-one (**S118**).

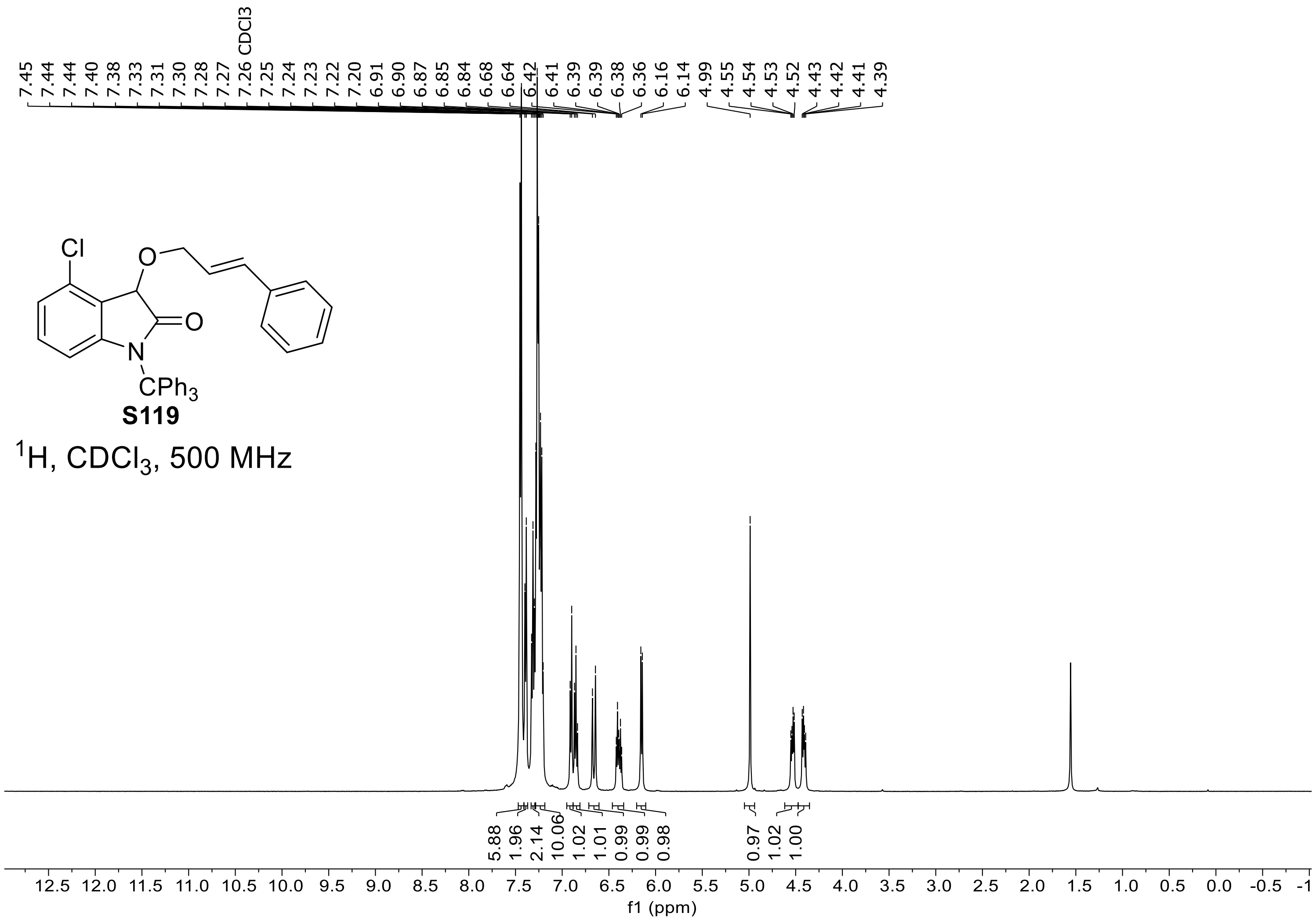

Fig. S301: ^{1}H NMR spectrum for (*E*)-4-Chloro-3-(cinnamyloxy)-1-tritylindolin-2-one (**S119**).

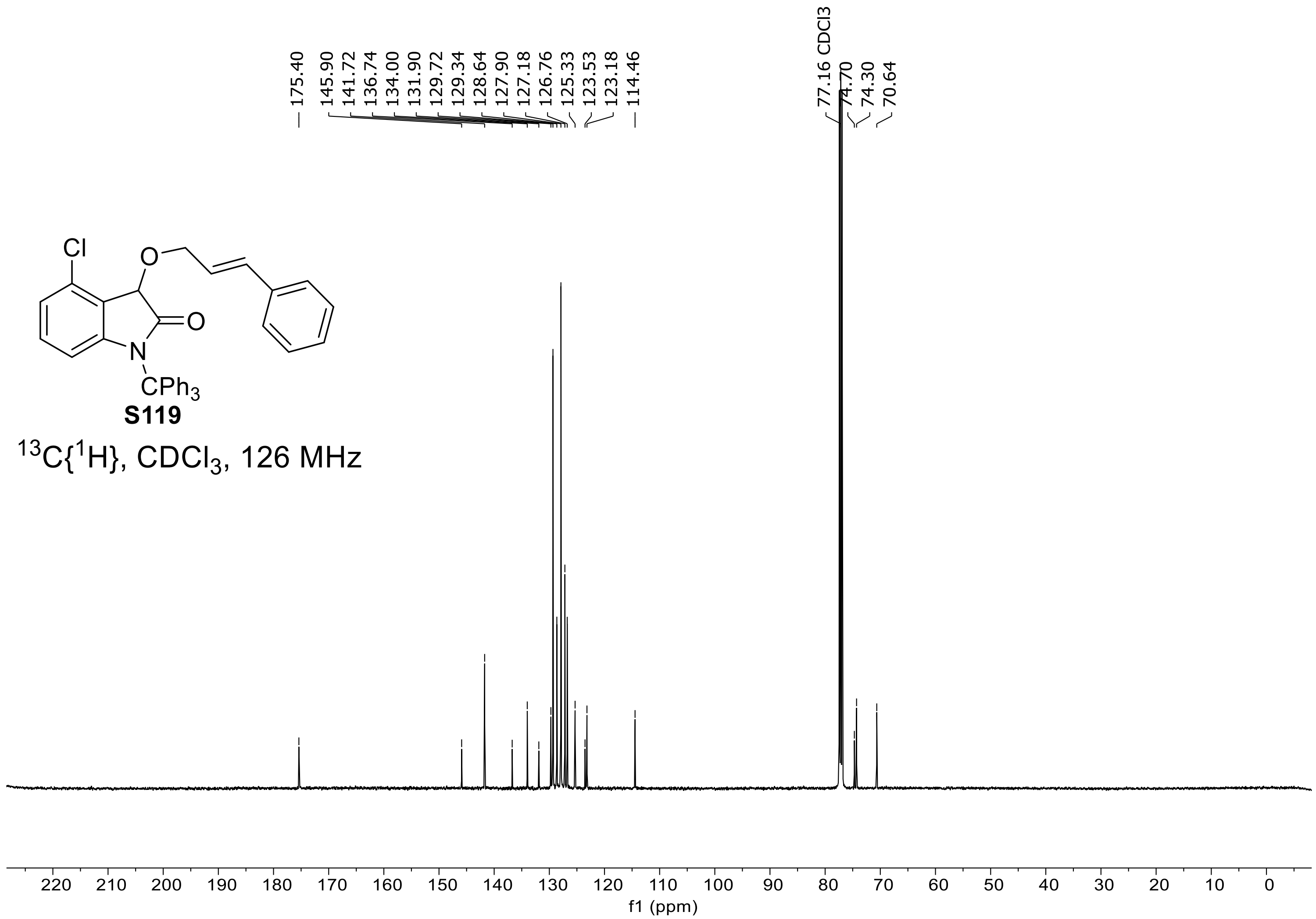

Fig. S302: $^{13}C\{^{1}H\}$ NMR spectrum for (*E*)-4-Chloro-3-(cinnamyloxy)-1-tritylindolin-2-one (**S119**).

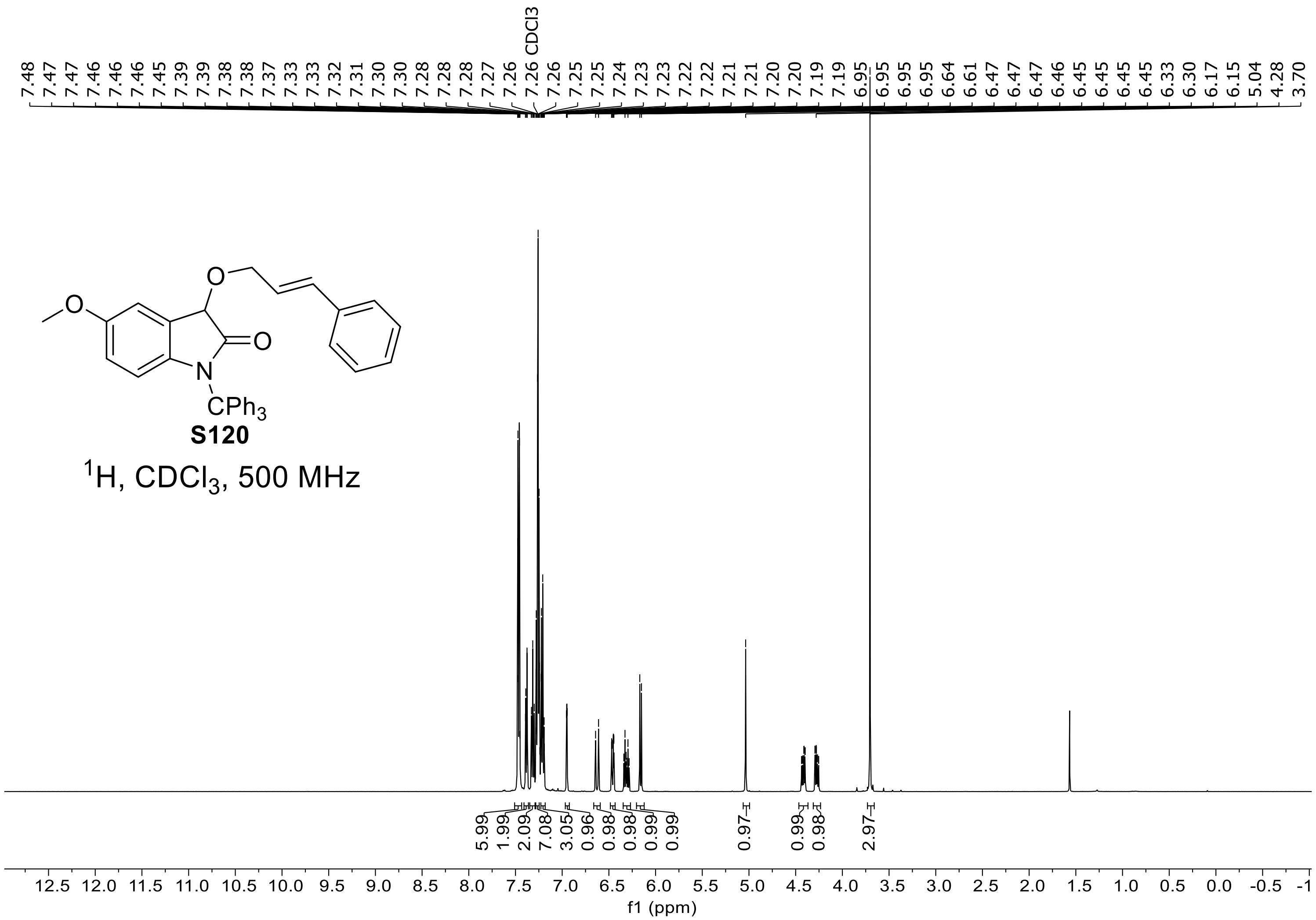

Fig. S303: ^{1}H NMR spectrum for (*E*)-3-(Cinnamyloxy)-5-methoxy-1-tritylindolin-2-one (**S120**).

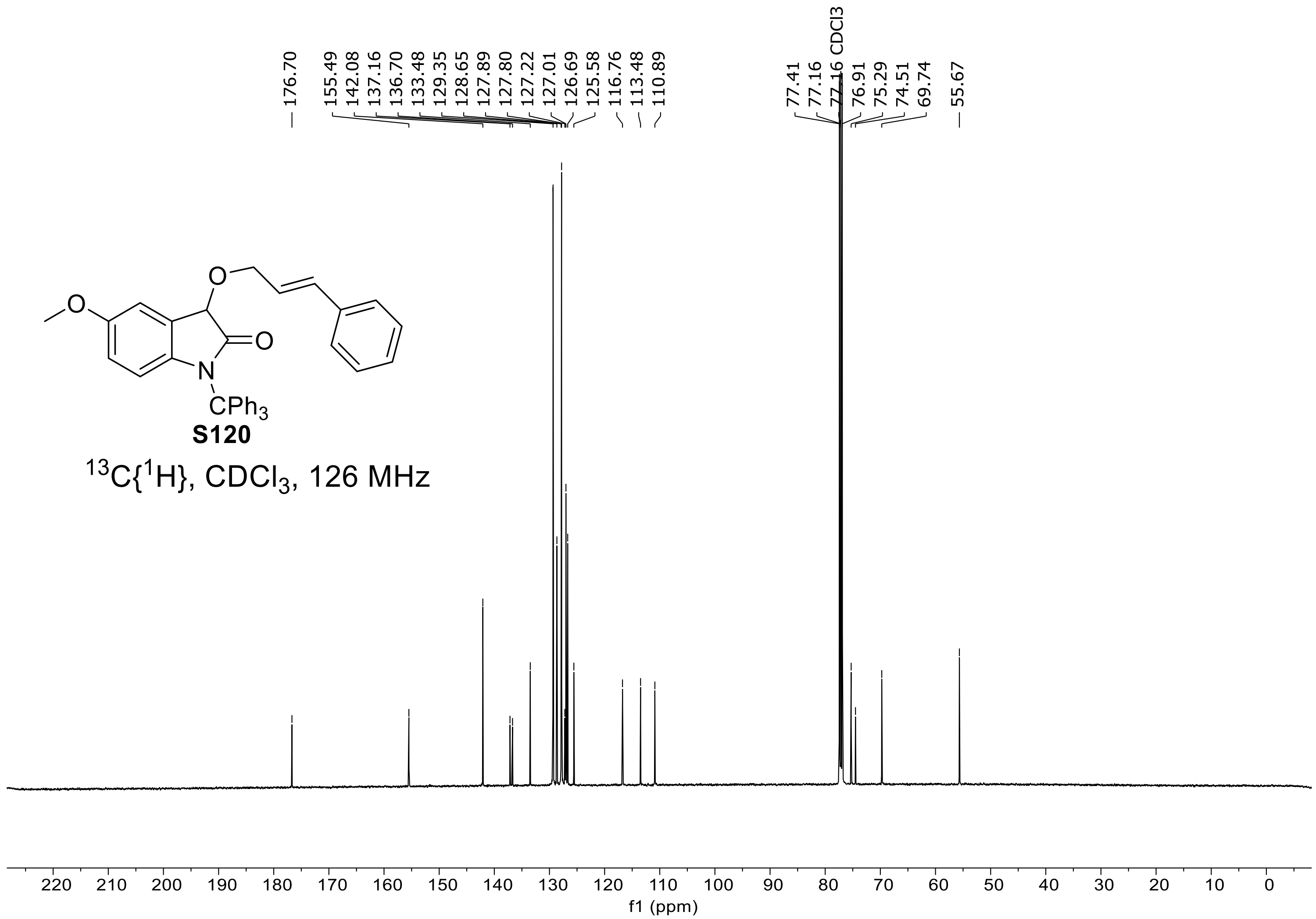

Fig. S304: $^{13}C\{^{1}H\}$ NMR spectrum for (*E*)-3-(Cinnamyloxy)-5-methoxy-1-tritylindolin-2-one (**S120**).

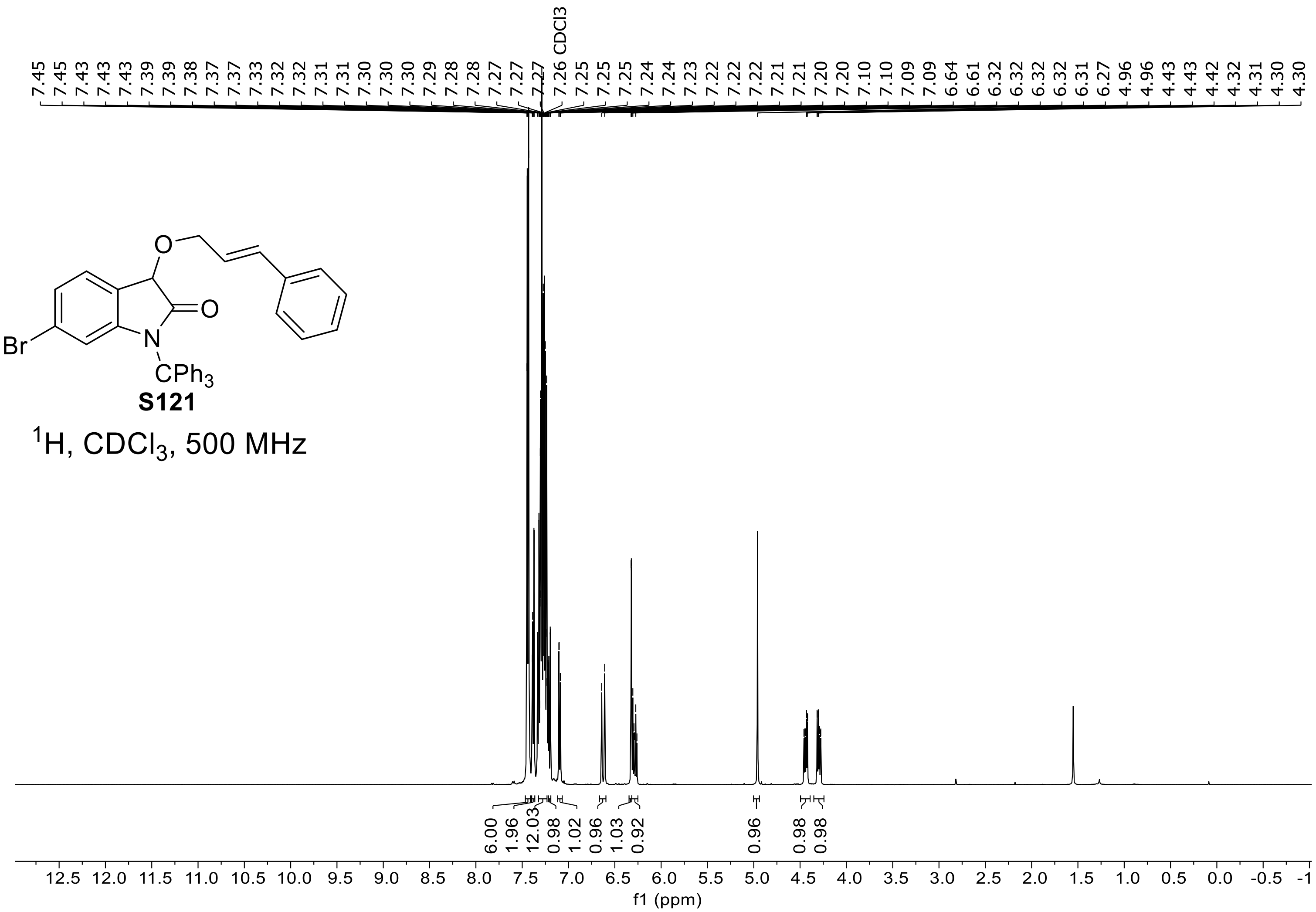

Fig. S305: ^{1}H NMR spectrum for (*E*)-6-Bromo-3-(cinnamyloxy)-1-tritylindolin-2-one (**S121**).

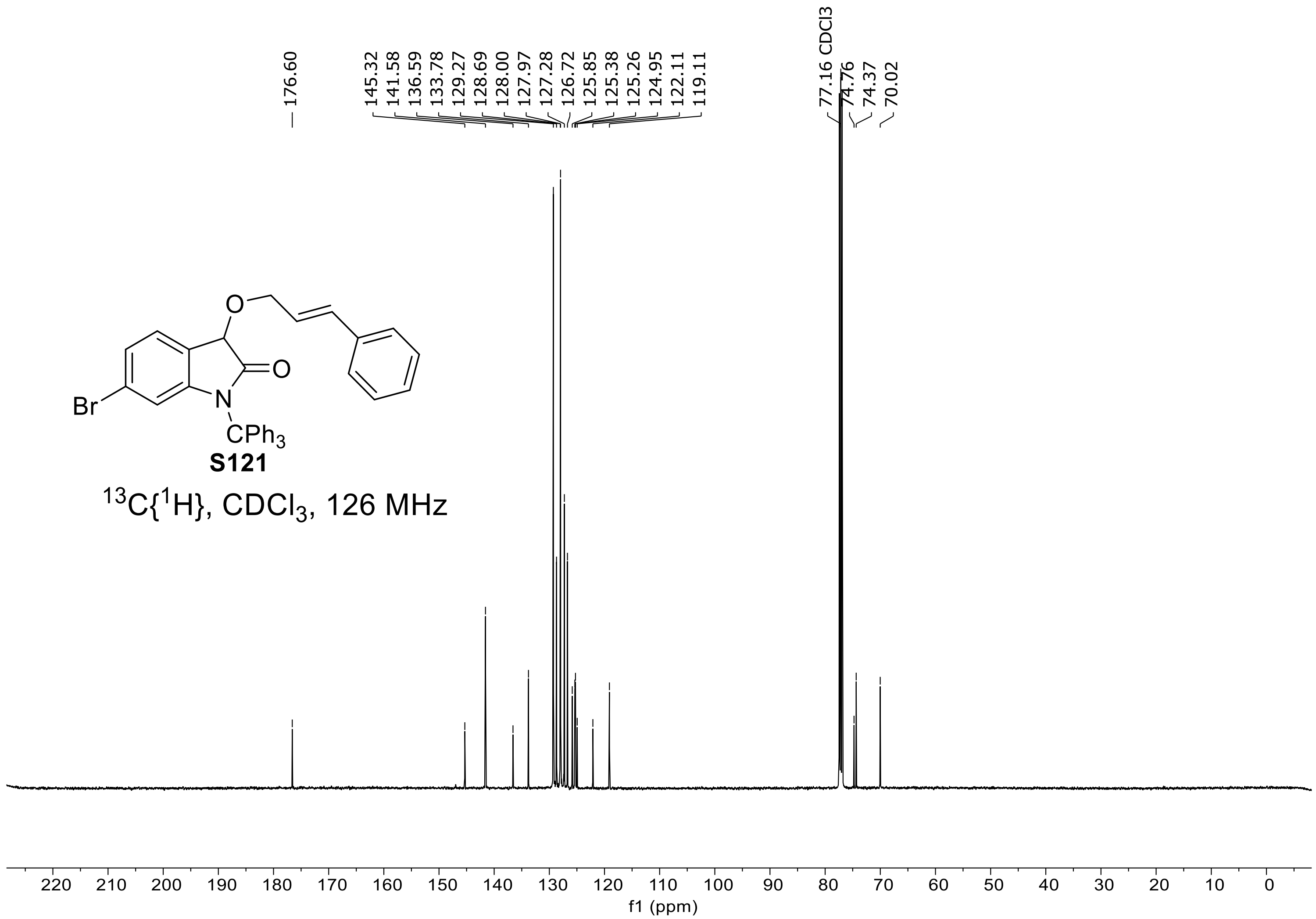

Fig. S306: $^{13}C\{^1H\}$ NMR spectrum for (*E*)-6-Bromo-3-(cinnamyloxy)-1-tritylindolin-2-one (**S121**).

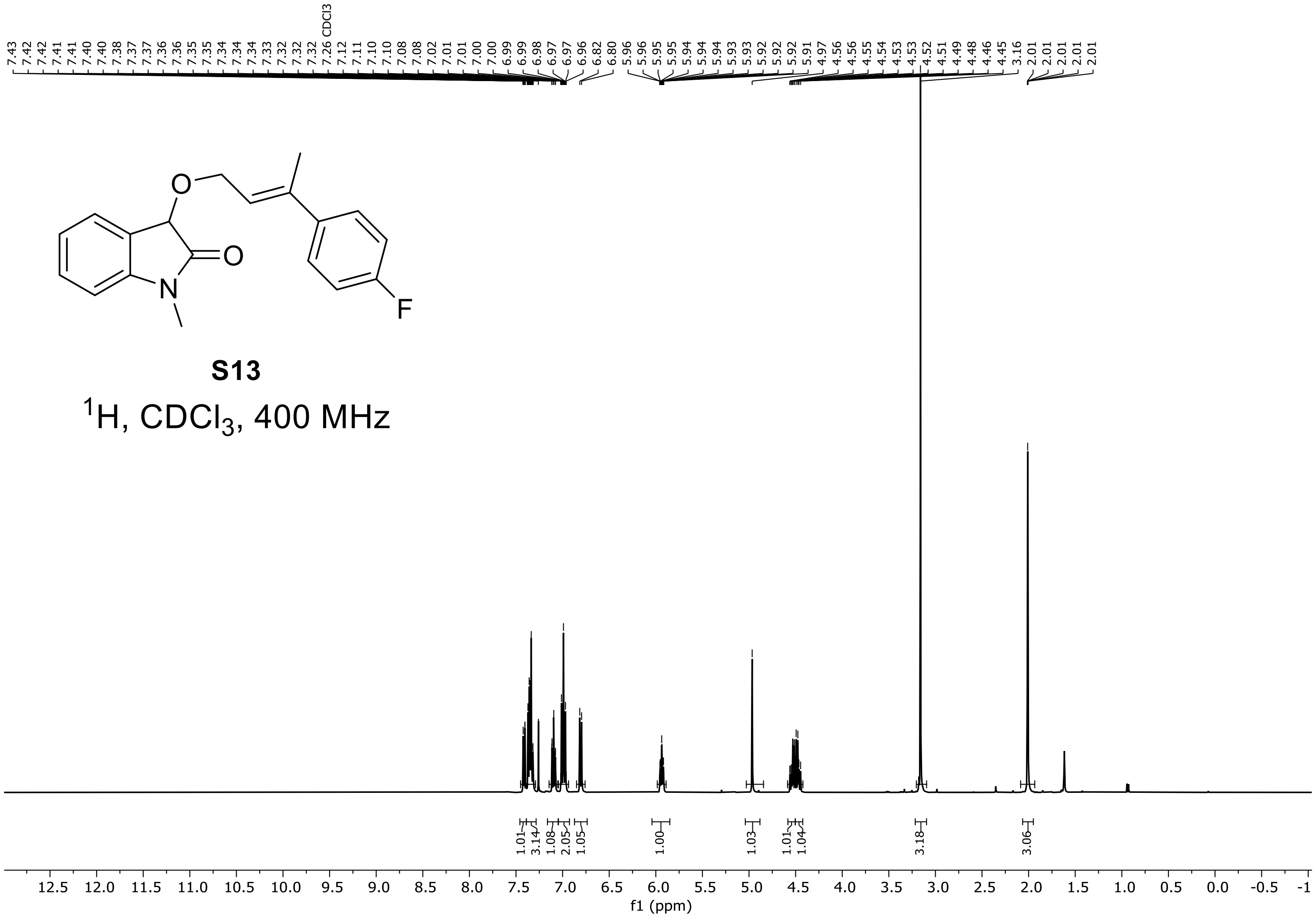

Fig. S307: ^{1}H NMR spectrum for (*E*)-3-((3-(4-fluorophenyl)but-2-en-1-yl)oxy)-1-methylindolin-2-one (**S13**).

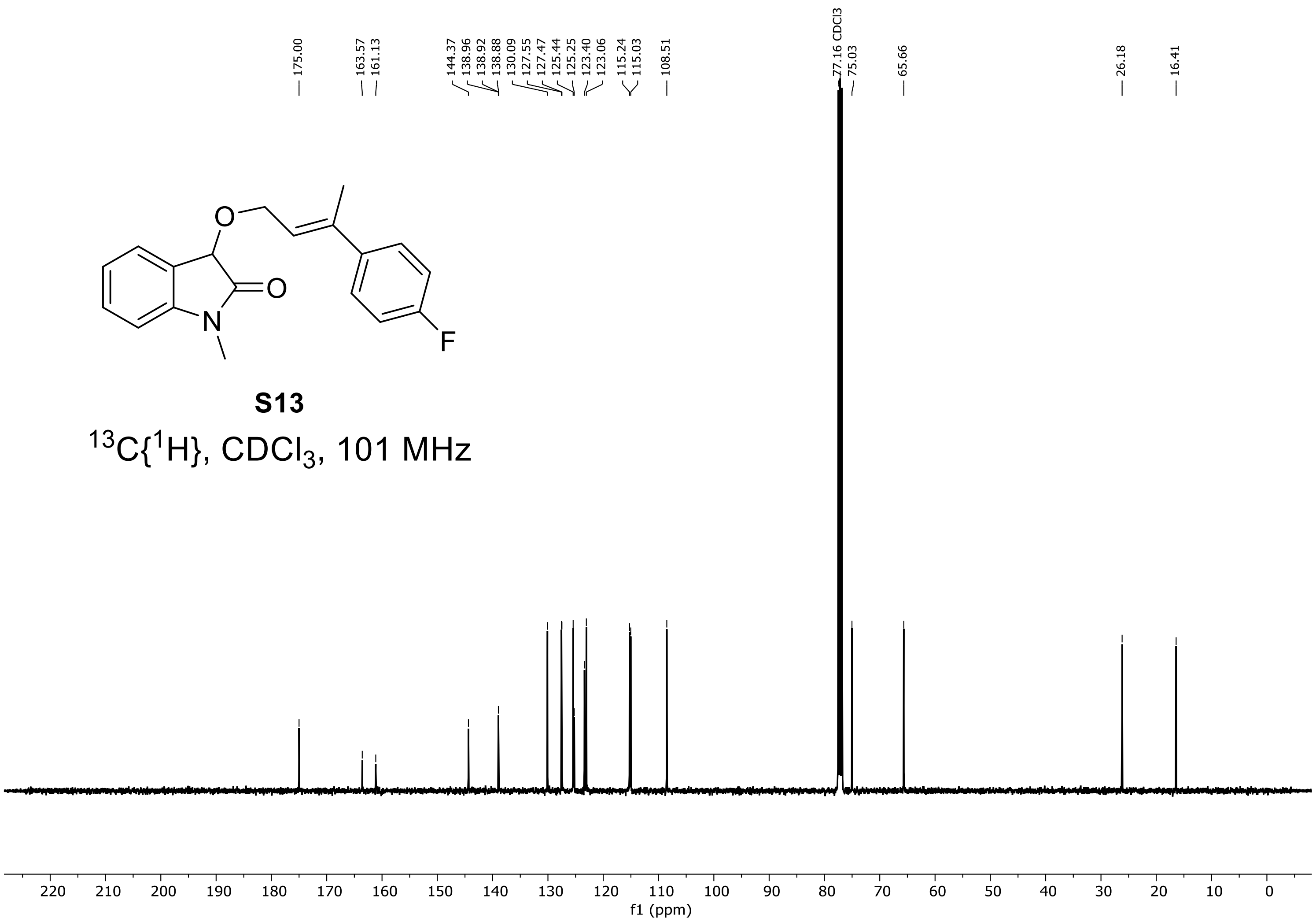

Fig. S308: $^{13}C\{^{1}H\}$ NMR spectrum for (*E*)-3-((3-(4-fluorophenyl)but-2-en-1-yl)oxy)-1-methylindolin-2-one (**S13**).

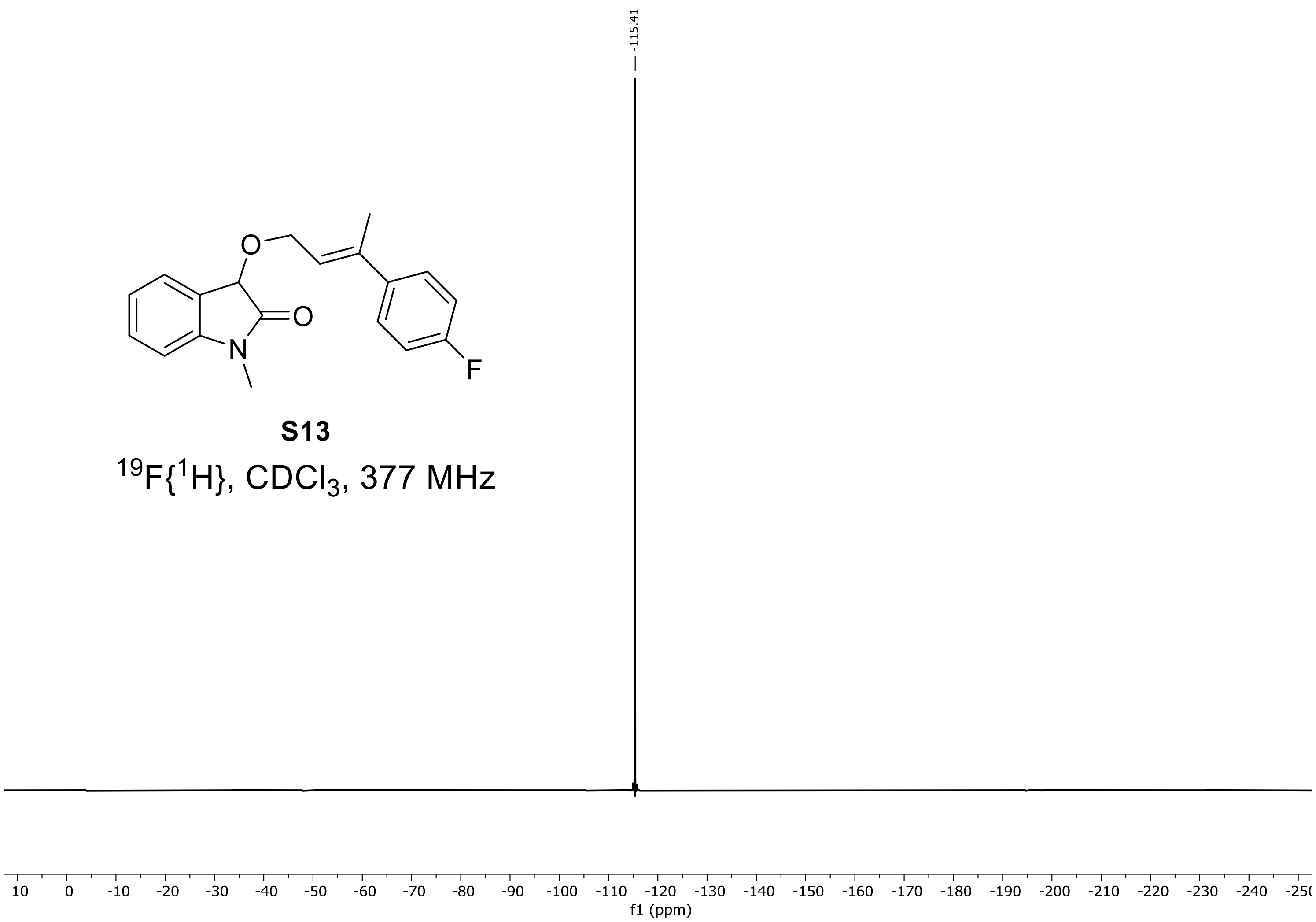

Fig. S309: $^{19}F\{^1H\}$ NMR spectrum for (*E*)-3-((3-(4-fluorophenyl)but-2-en-1-yl)oxy)-1-methylindolin-2-one (**S13**).

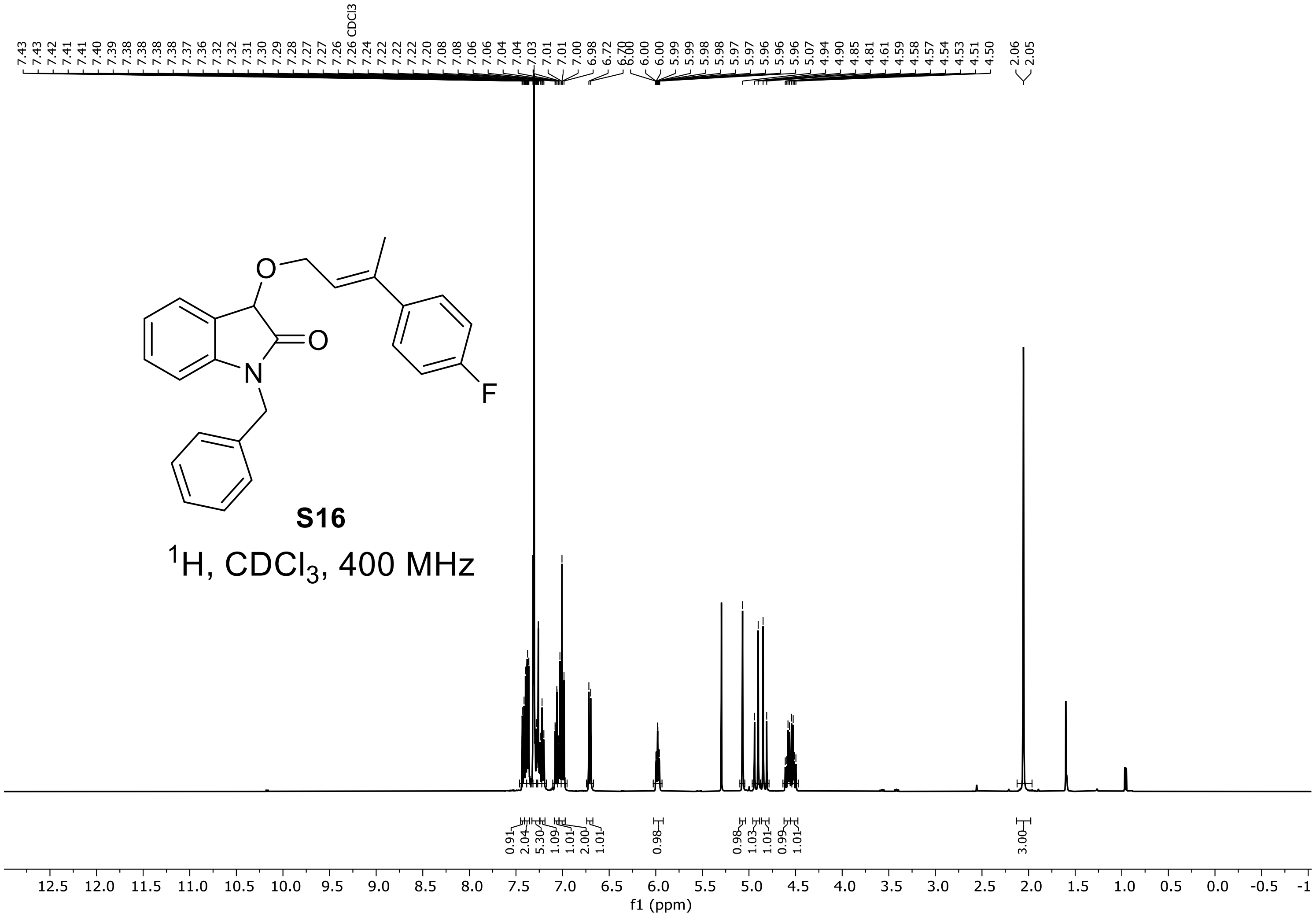

Fig. S310: ^{1}H NMR spectrum for (*E*)-1-benzyl-3-((3-(4-fluorophenyl)but-2-en-1-yl)oxy)indolin-2-one (**S16**).

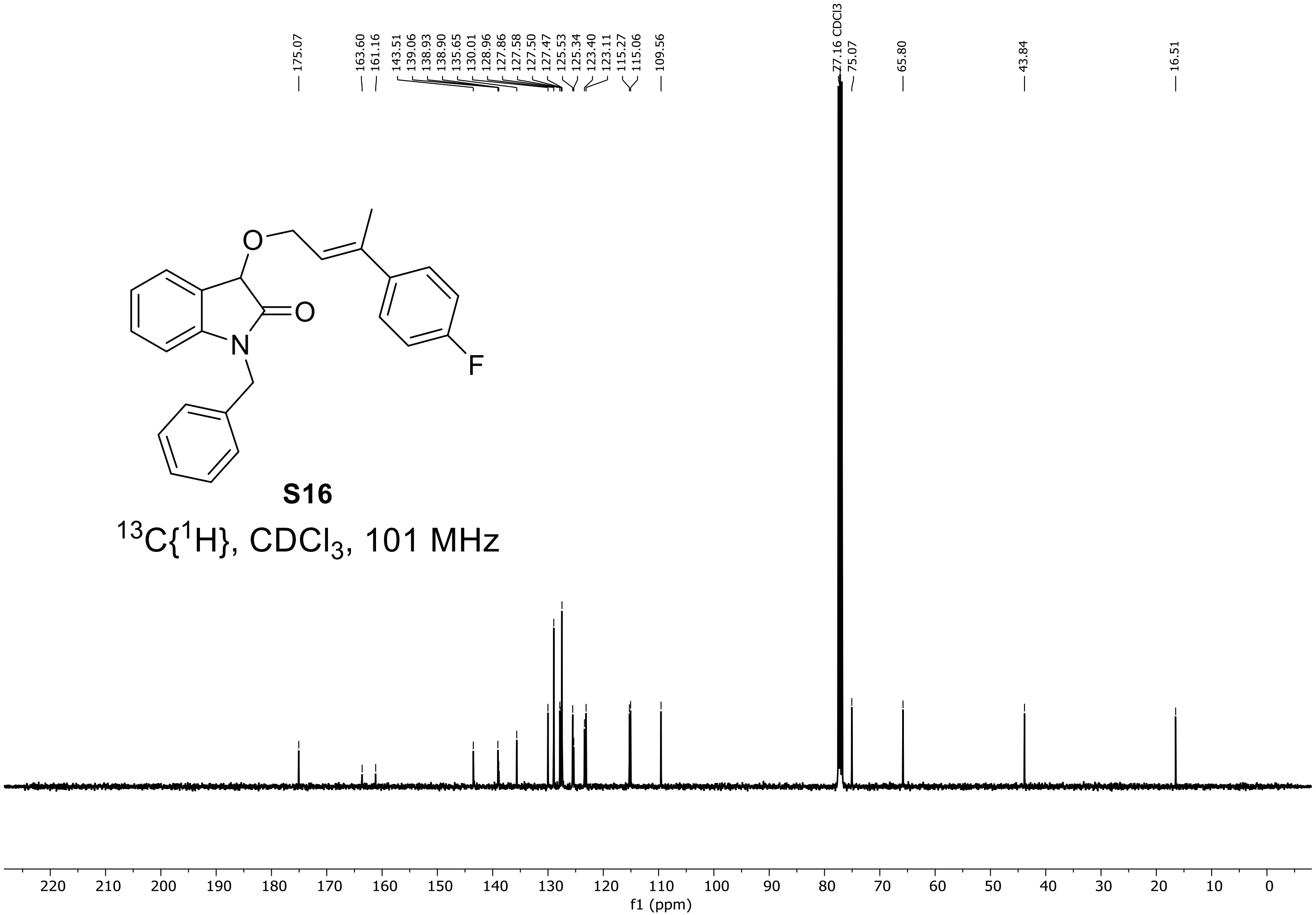

Fig. S311: $^{13}C\{^1H\}$ NMR spectrum for (*E*)-1-benzyl-3-((3-(4-fluorophenyl)but-2-en-1-yl)oxy)indolin-2-one (**S16**).

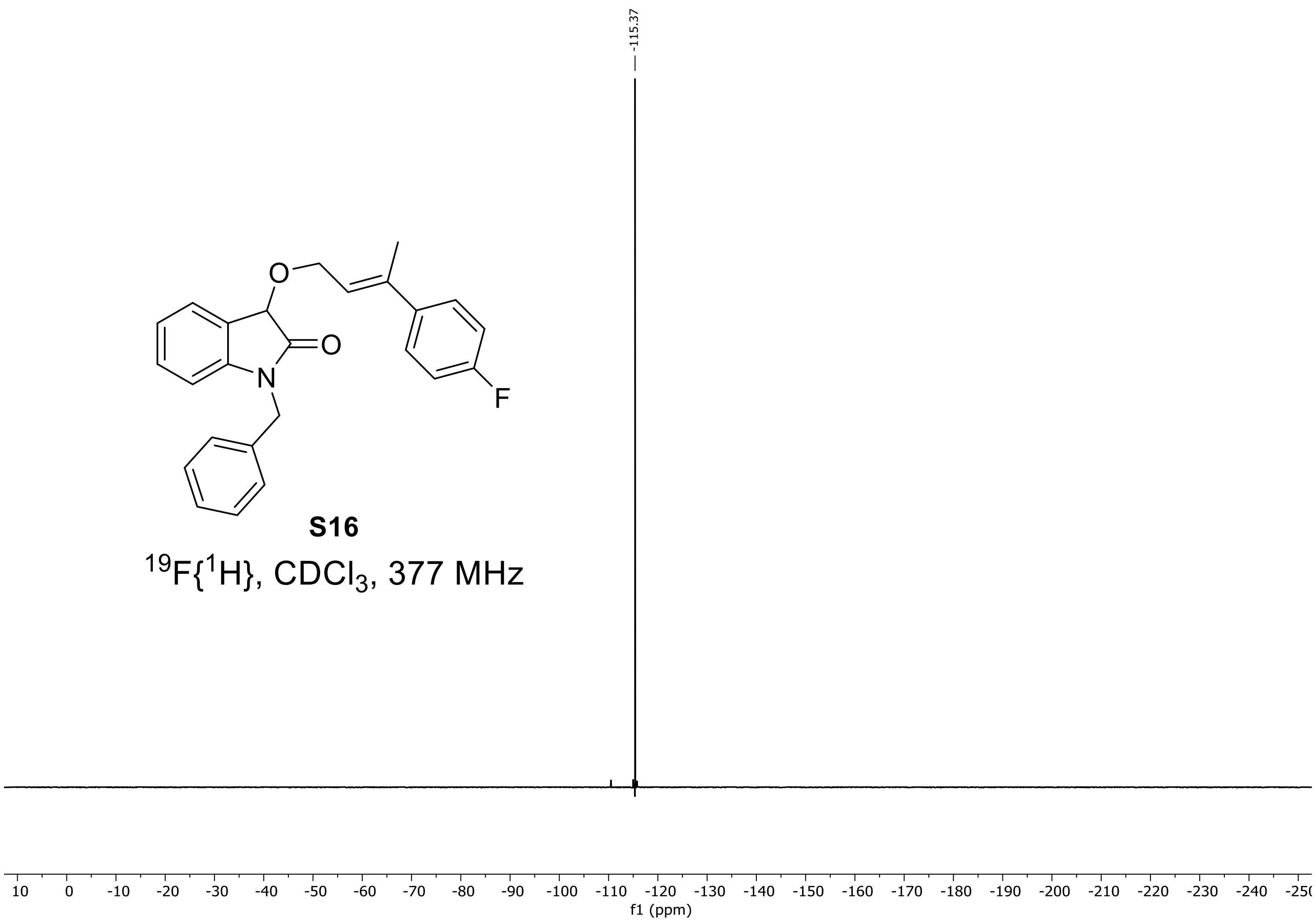

Fig. S312 $^{19}F\{^{1}H\}$ NMR spectrum for (*E*)-1-benzyl-3-((3-(4-fluorophenyl)but-2-en-1-yl)oxy)indolin-2-one (**S16**).

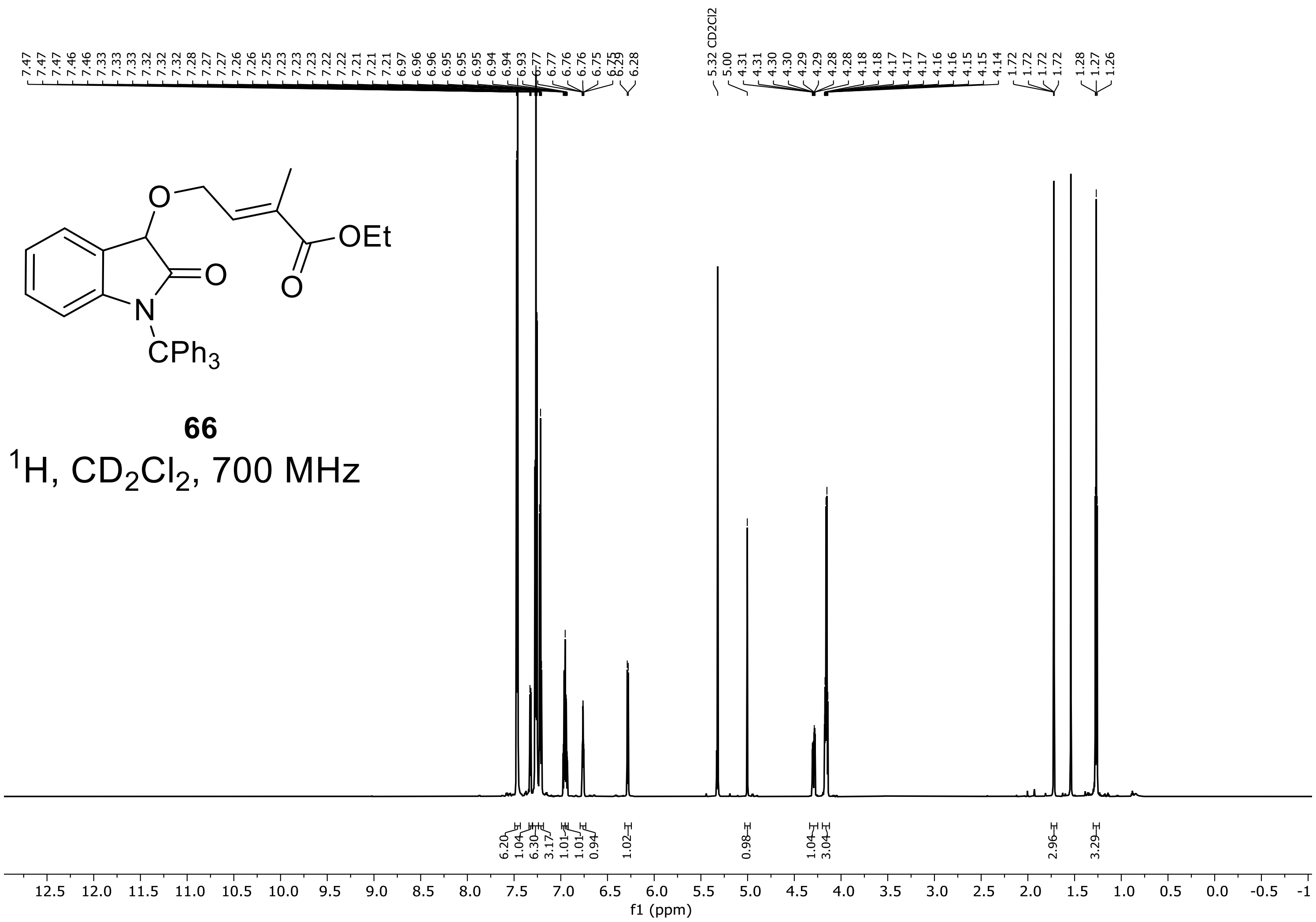

Fig. S313: ^{1}H NMR spectrum for ethyl 4-(1-trityloxindol-3-oxy)tiglate (**66**).

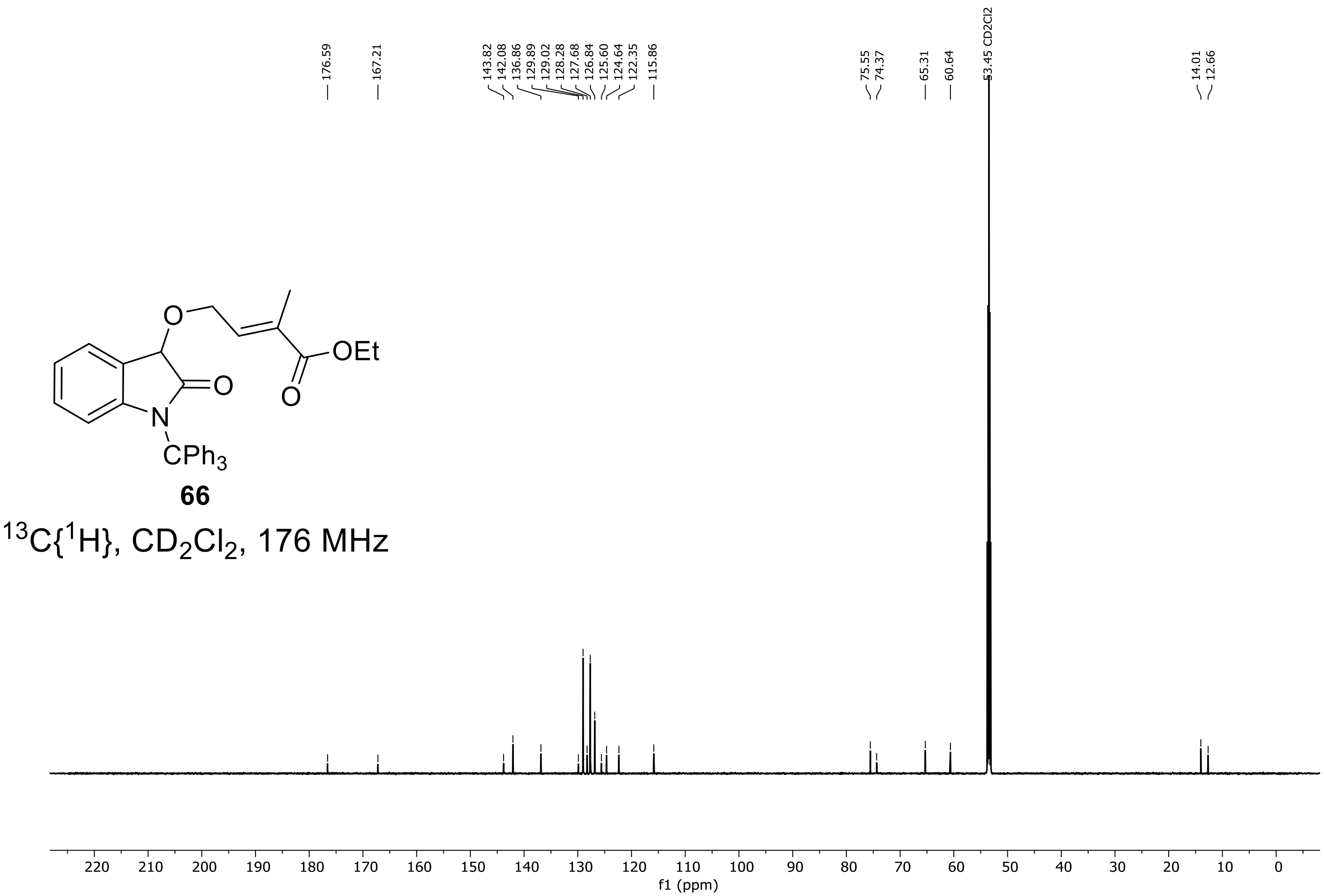

Fig. S314: $^{13}C\{^{1}H\}$ NMR spectrum for ethyl 4-(1-trityloxindol-3-oxy)tiglate (**66**).

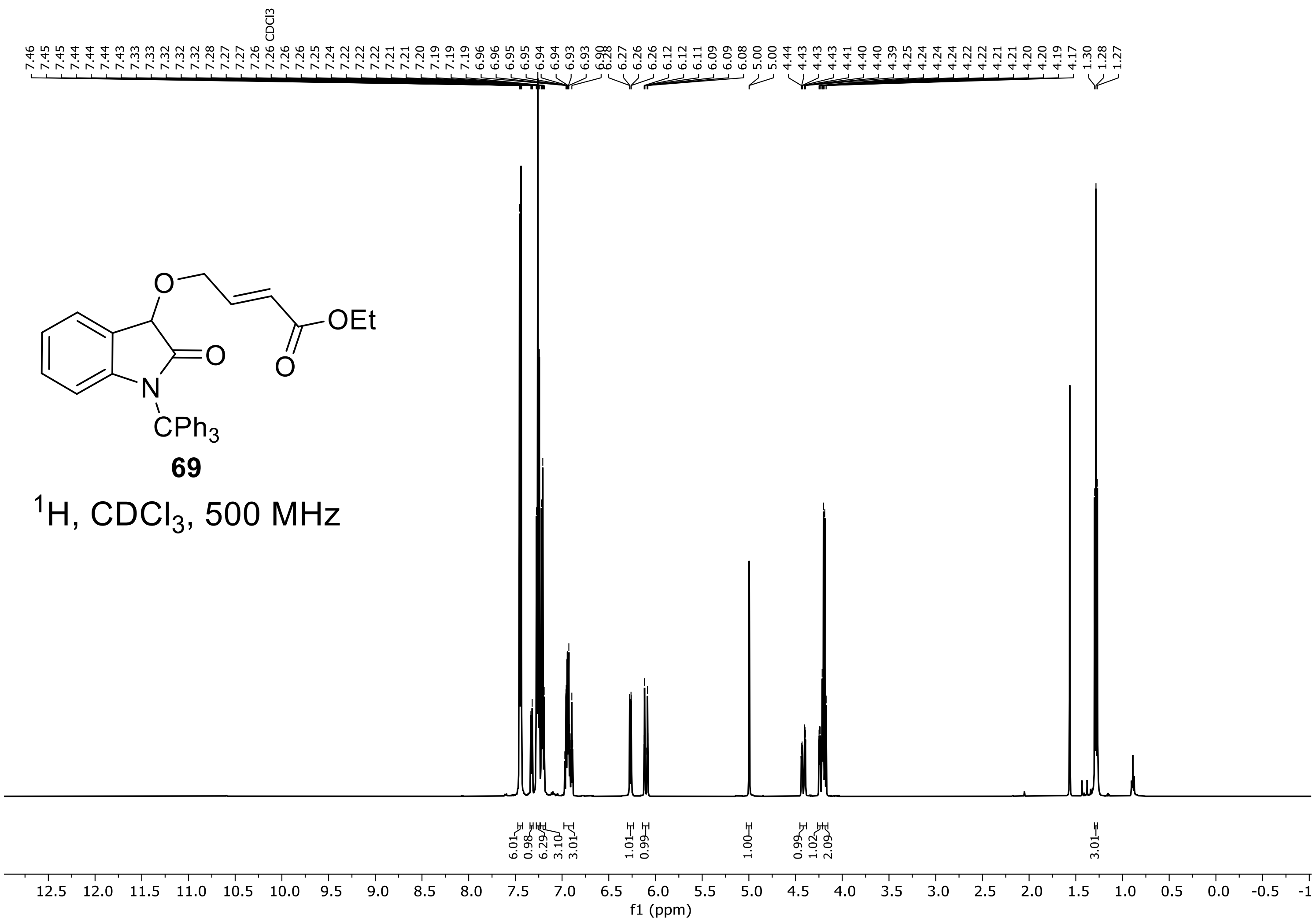

Fig. S315: ^{1}H NMR spectrum for ethyl (*E*)-4-((2-oxo-1-tritylindolin-3-yl)oxy)crotonate (**69**).

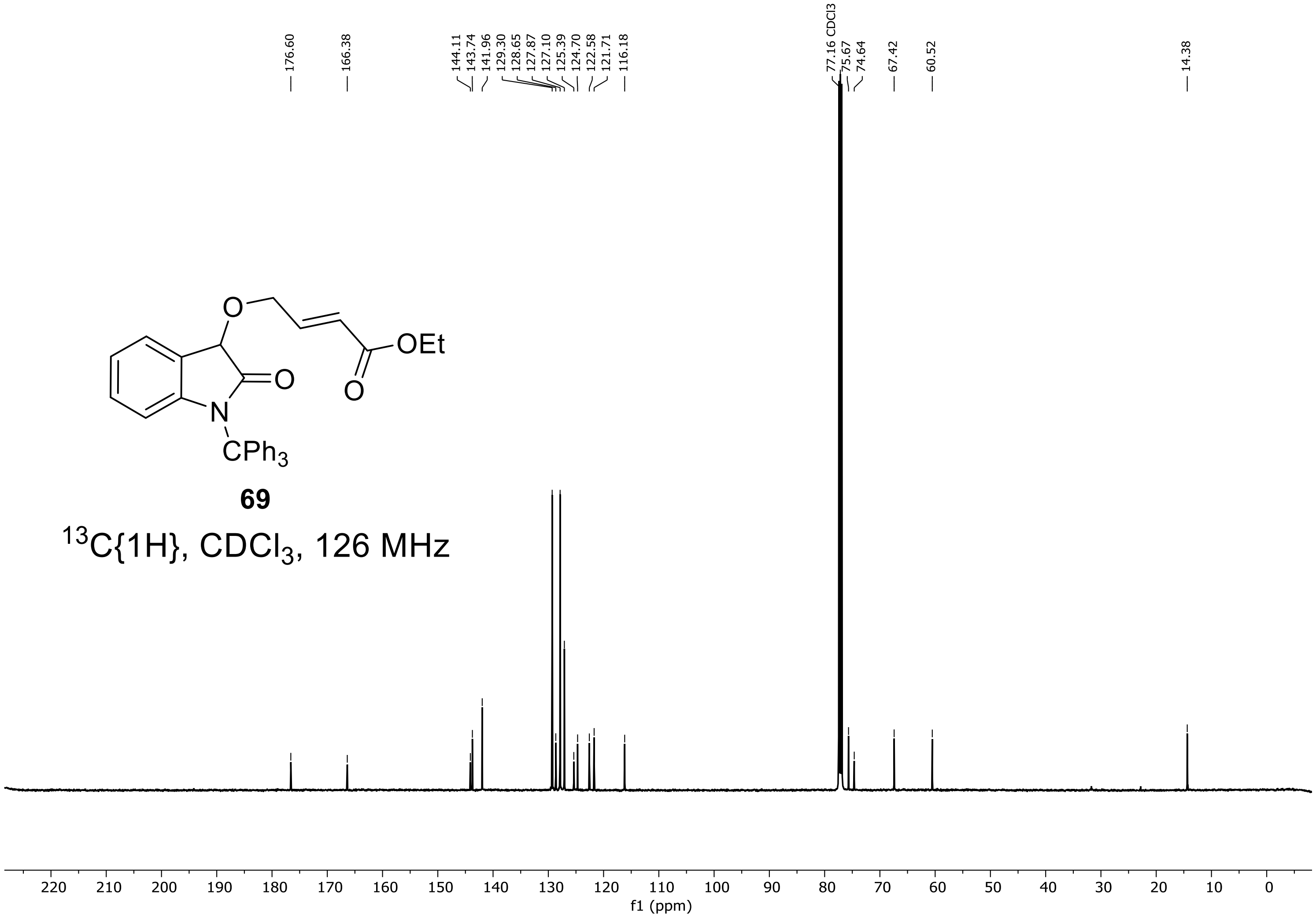

Fig. S316: $^{13}C\{^{1}H\}$ NMR spectrum for ethyl (*E*)-4-((2-oxo-1-tritylindolin-3-yl)oxy)crotonate (**69**).

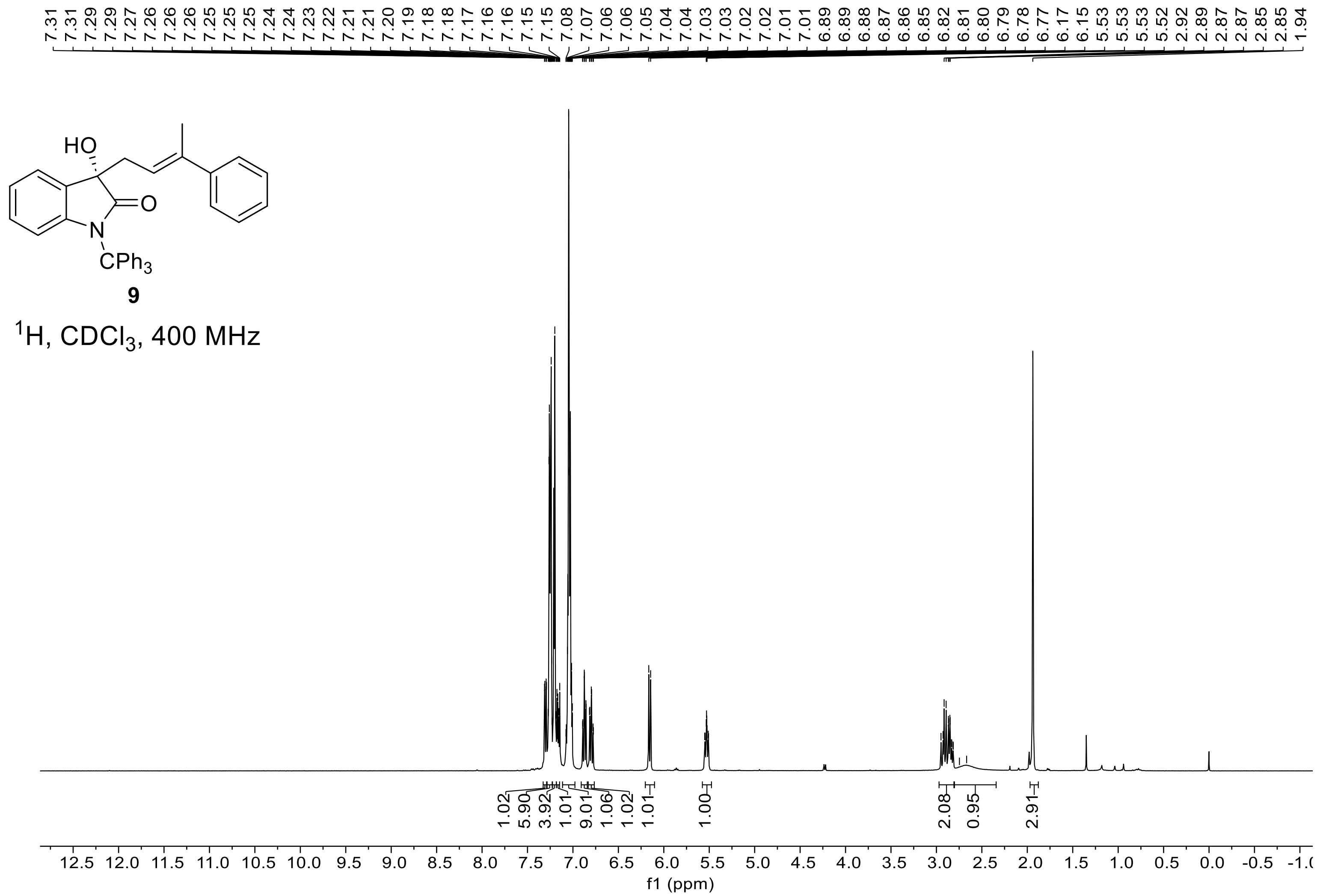

Fig. S317: ^{1}H NMR spectrum for (*S*,*E*)-3-Hydroxy-3-(3-phenylbut-2-en-1-yl)-1-tritylindolin-2-one (**9**).

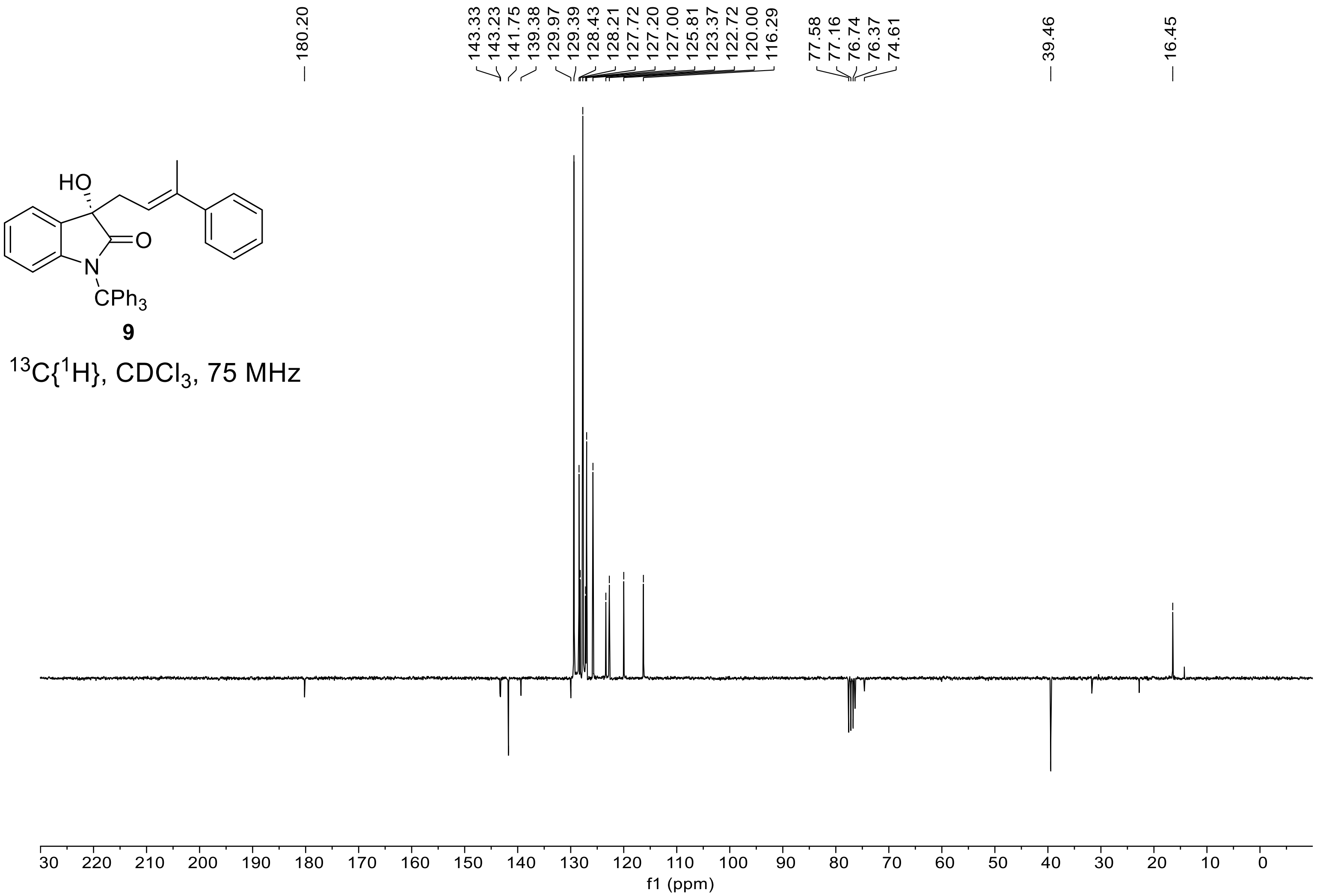

Fig. S318: $^{13}C\{^{1}H\}$ NMR spectrum for (*S*,*E*)-3-Hydroxy-3-(3-phenylbut-2-en-1-yl)-1-tritylindolin-2-one (**9**).

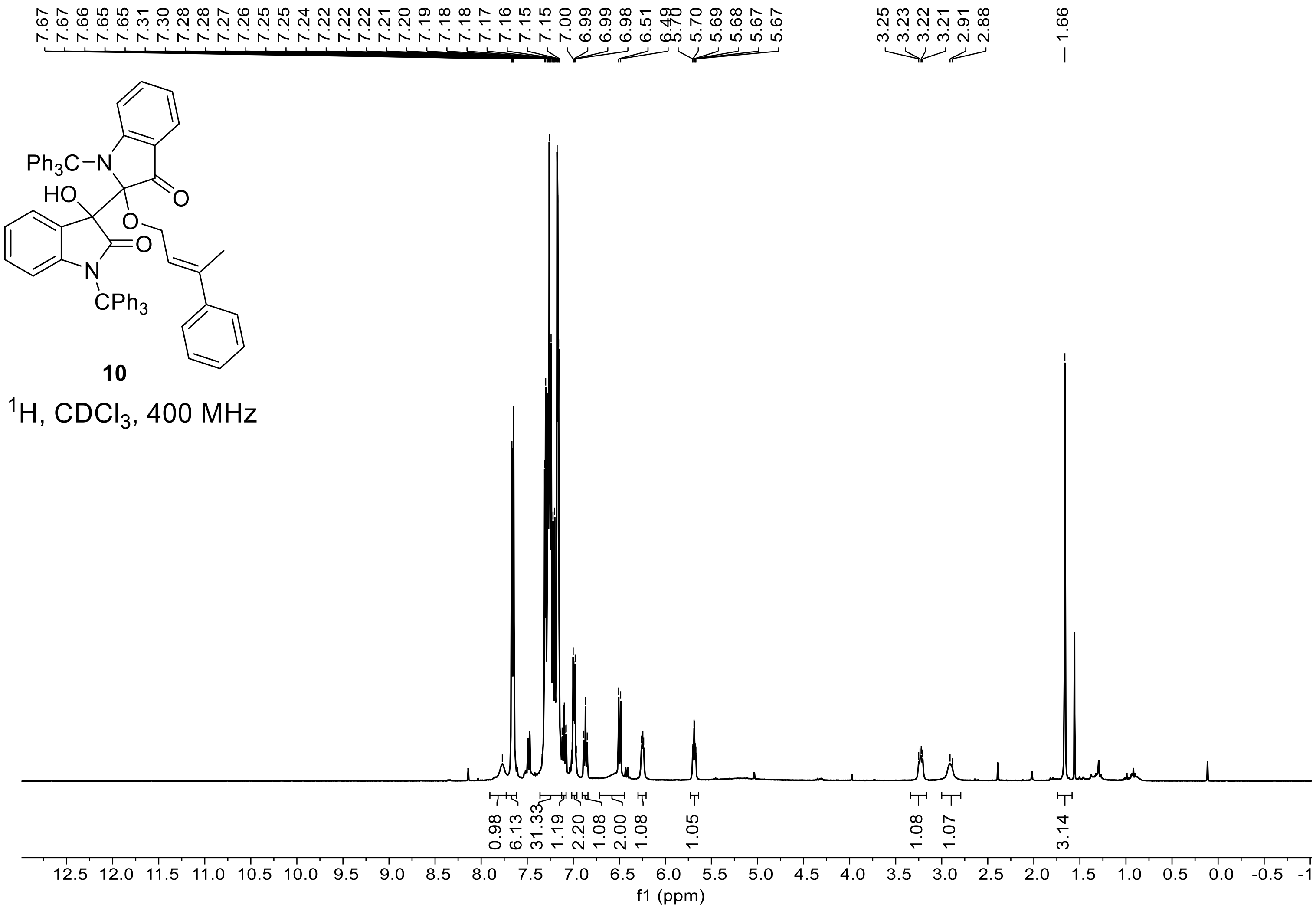

Fig. S319: ^{1}H NMR spectrum for (*E*)-3-Hydroxy-3'-[(3-phenylbut-2-en-1-yl)oxy]-1,1'-ditrityl-[3,3'-biindoline]-2,2'-dione (**10**).

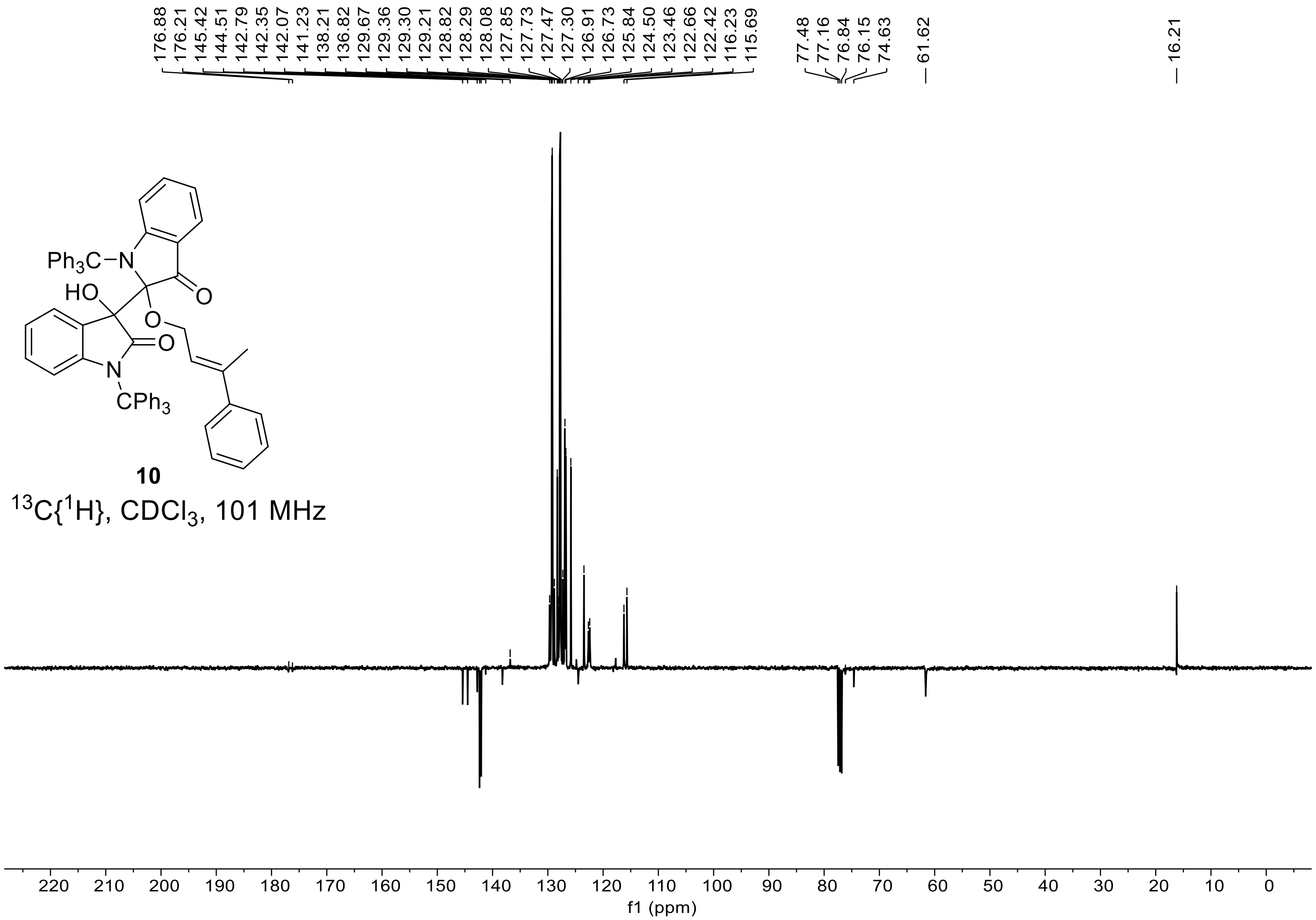

Fig. S320: $^{13}C\{^{1}H\}$ NMR spectrum for (*E*)-3-Hydroxy-3'-[(3-phenylbut-2-en-1-yl)oxy]-1,1'-ditrityl-[3,3'-biindoline]-2,2'-dione (**10**).

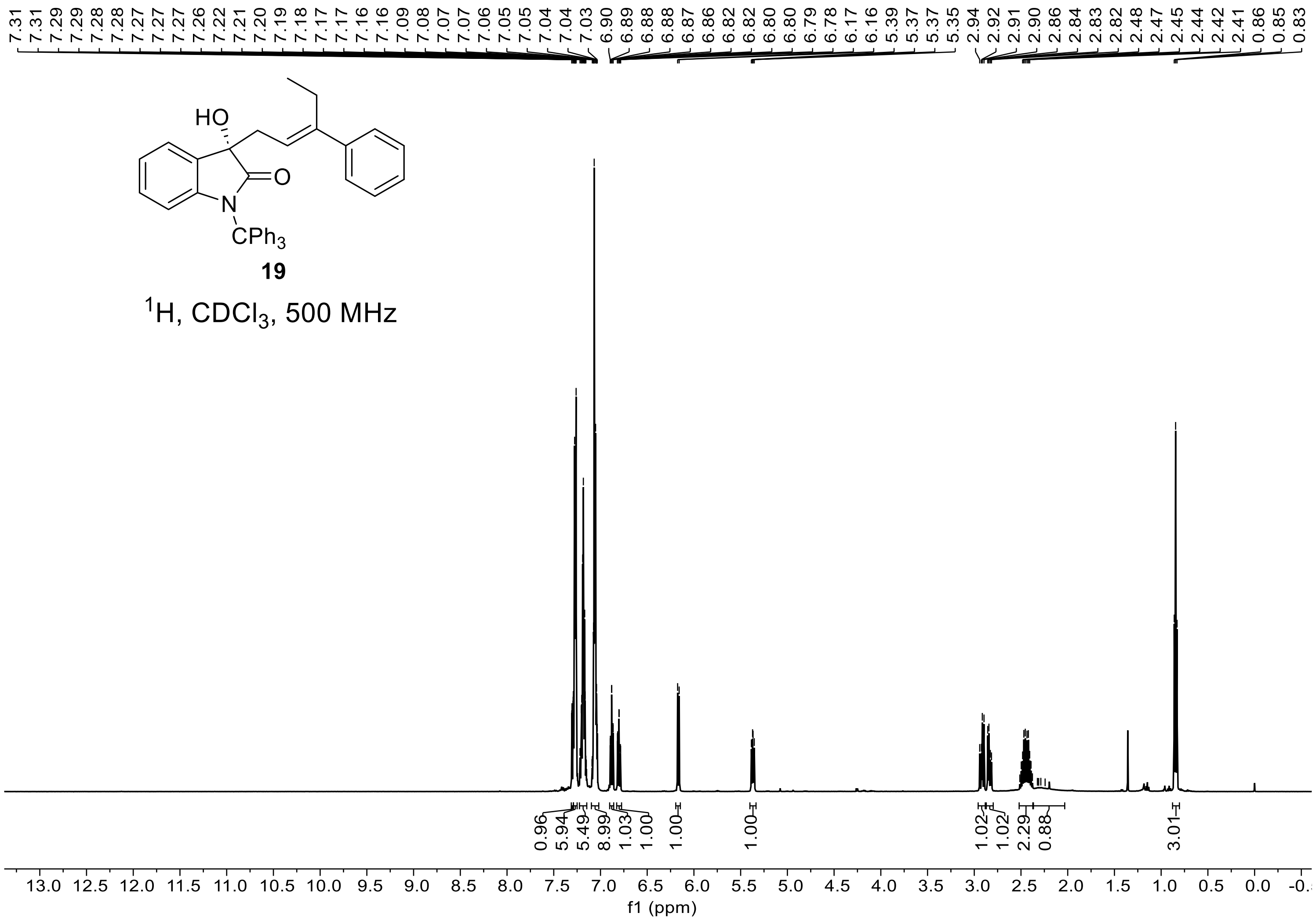

Fig. S321: ^{1}H NMR spectrum for (*S*,*E*)-3-Hydroxy-3-(3-phenylpent-2-en-1-yl)-1-tritylindolin-2-one (**19**).

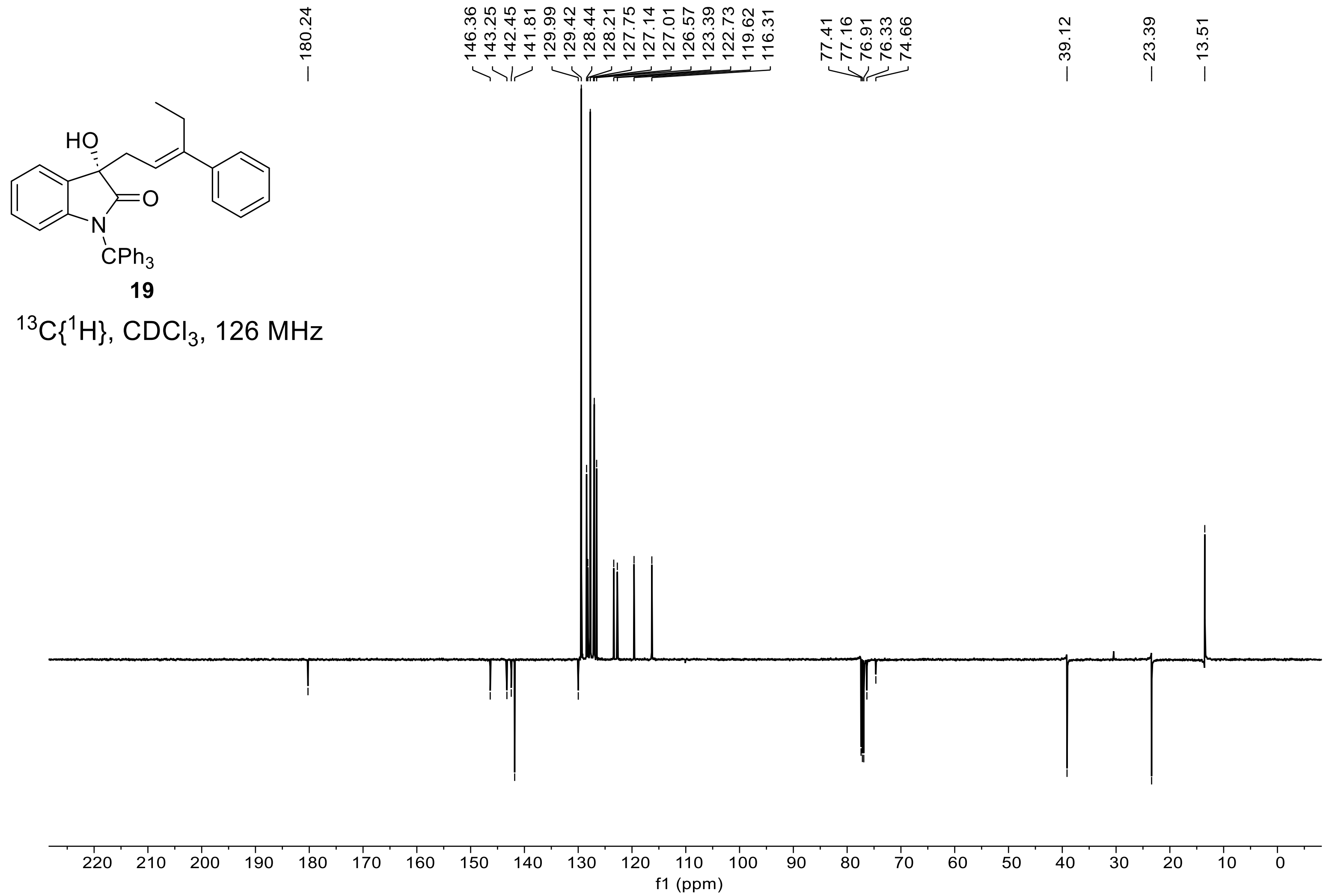

Fig. S322: $^{13}C\{^1H\}$ NMR spectrum for (*S*,*E*)-3-Hydroxy-3-(3-phenylpent-2-en-1-yl)-1-tritylindolin-2-one (**19**).

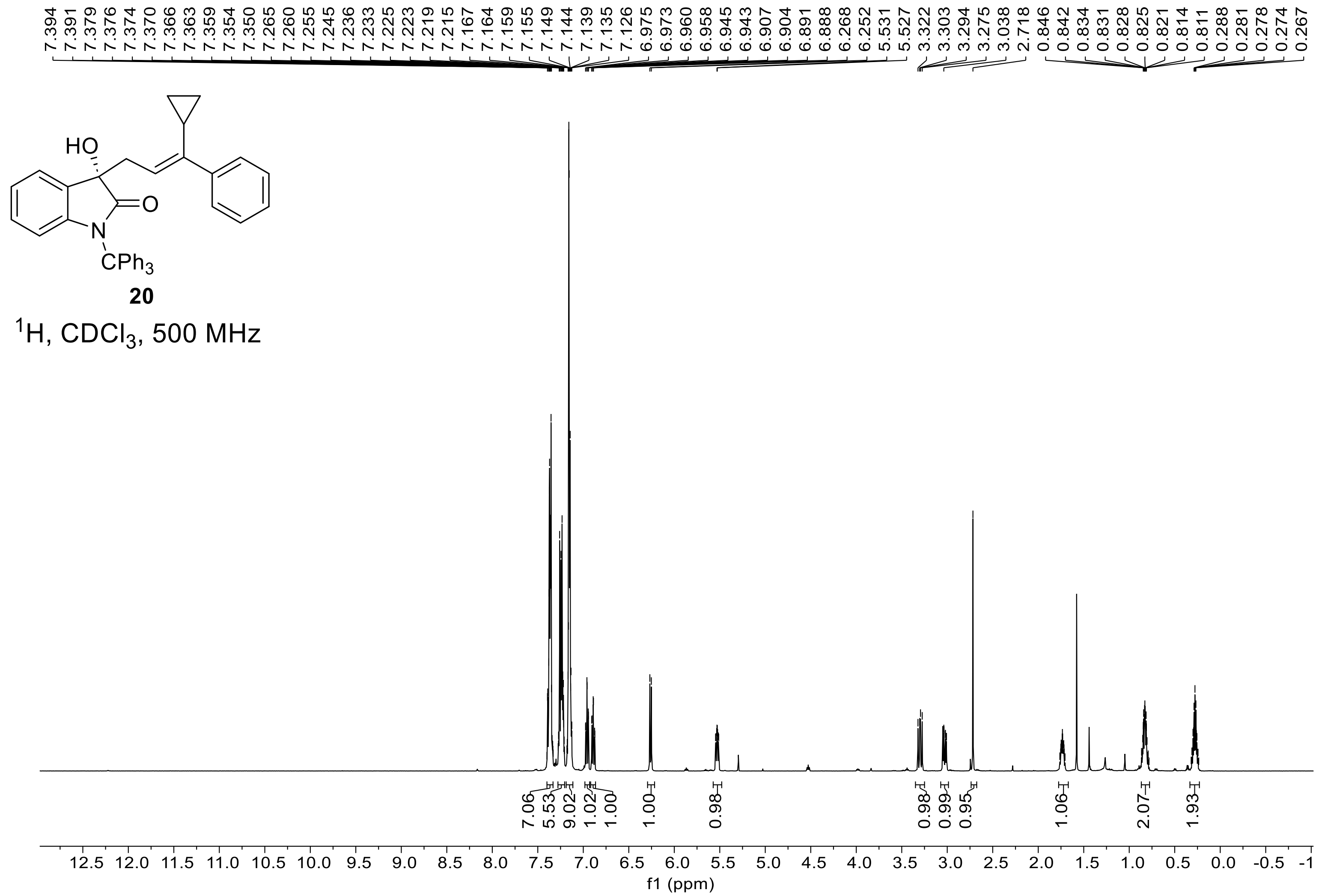

Fig. S323: ^{1}H NMR spectrum for (*S*,*E*)-3-(3-Cyclopropyl-3-phenylallyl)-3-hydroxy-1-tritylindolin-2-one (**20**).

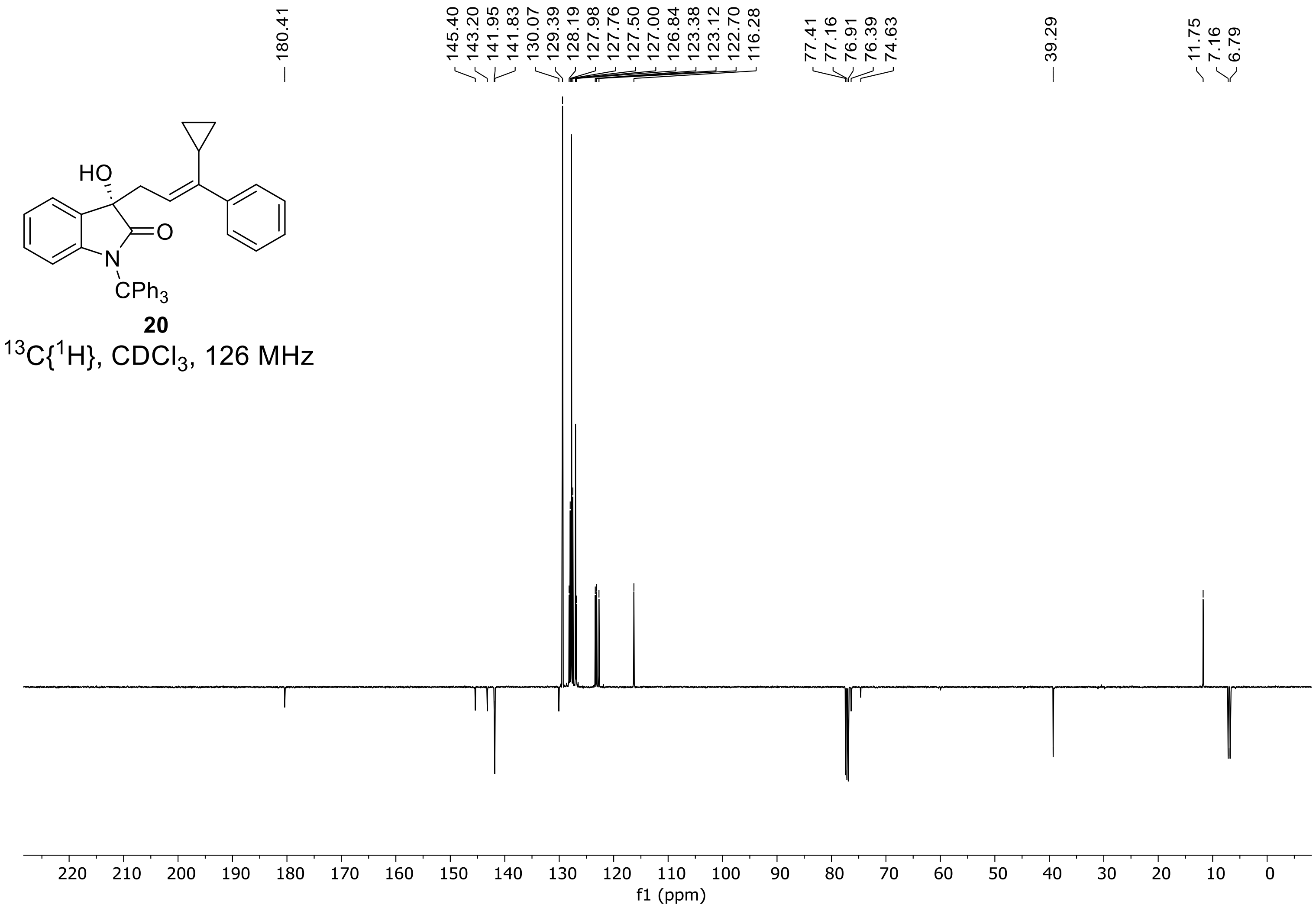

Fig. S324: $^{13}C\{^{1}H\}$ NMR spectrum for (*S*,*E*)-3-(3-Cyclopropyl-3-phenylallyl)-3-hydroxy-1-tritylindolin-2-one (**20**).

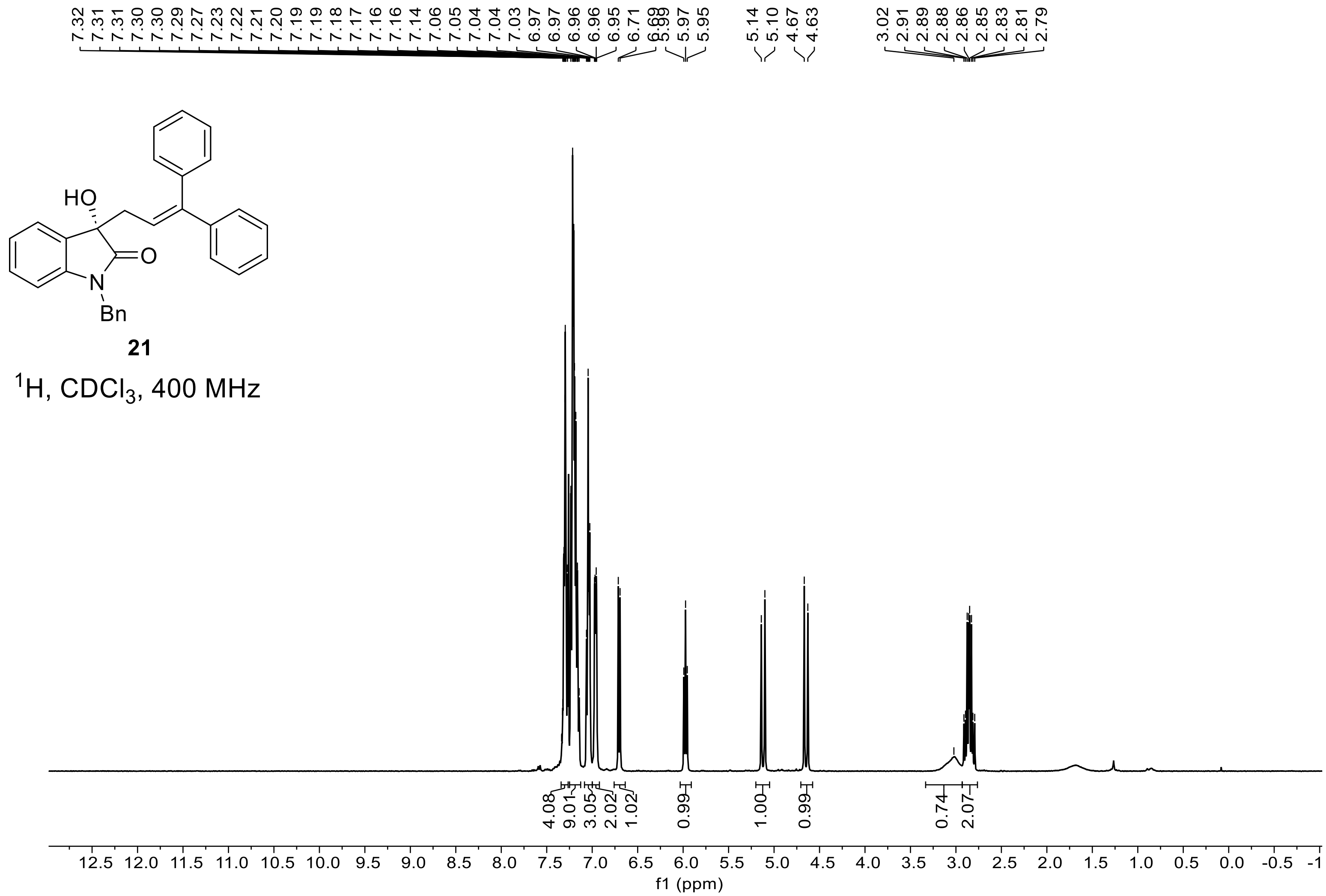

Fig. S325: ^{1}H NMR spectrum for (*S*)-1-Benzyl-3-(3,3-diphenylallyl)-3-hydroxyindolin-2-one (**21**).

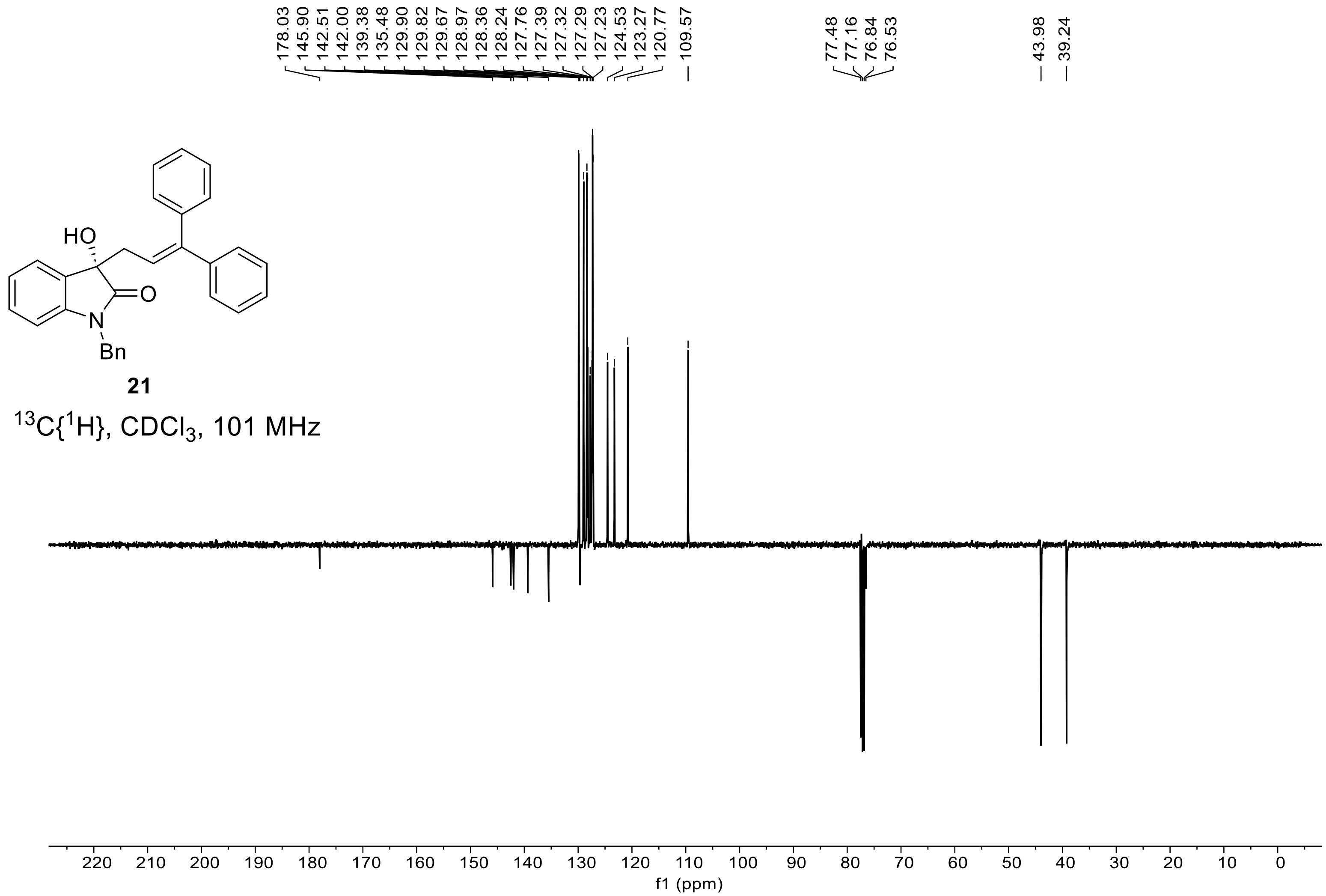

Fig. S326: $^{13}C\{^{1}H\}$ NMR spectrum for (*S*)-1-Benzyl-3-(3,3-diphenylallyl)-3-hydroxyindolin-2-one (**21**).

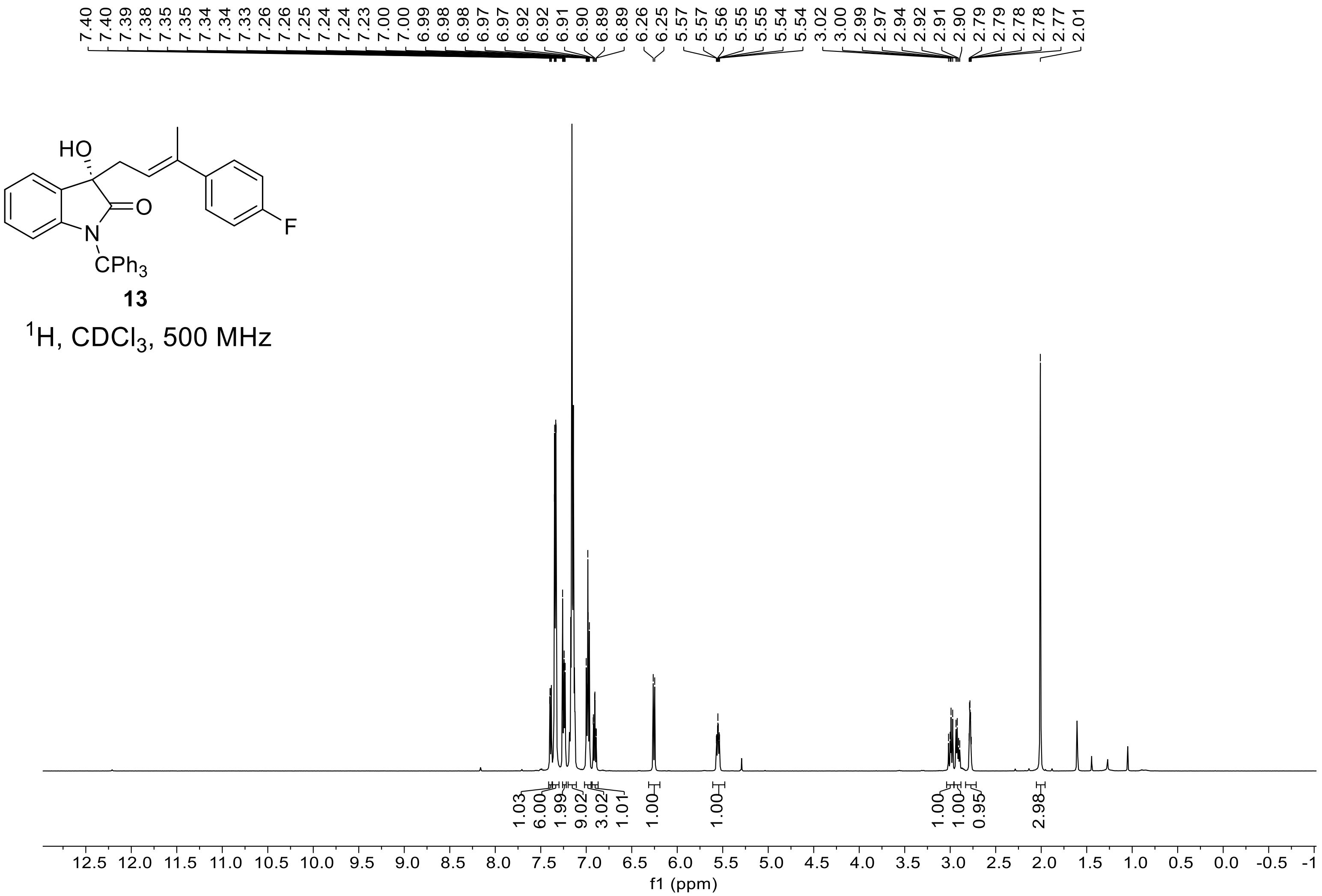

Fig. S327: ¹H NMR spectrum for (*S*,*E*)-3-[3-(4-Fluorophenyl)but-2-en-1-yl]-3-hydroxy-1-tritylindolin-2-one (**13**).

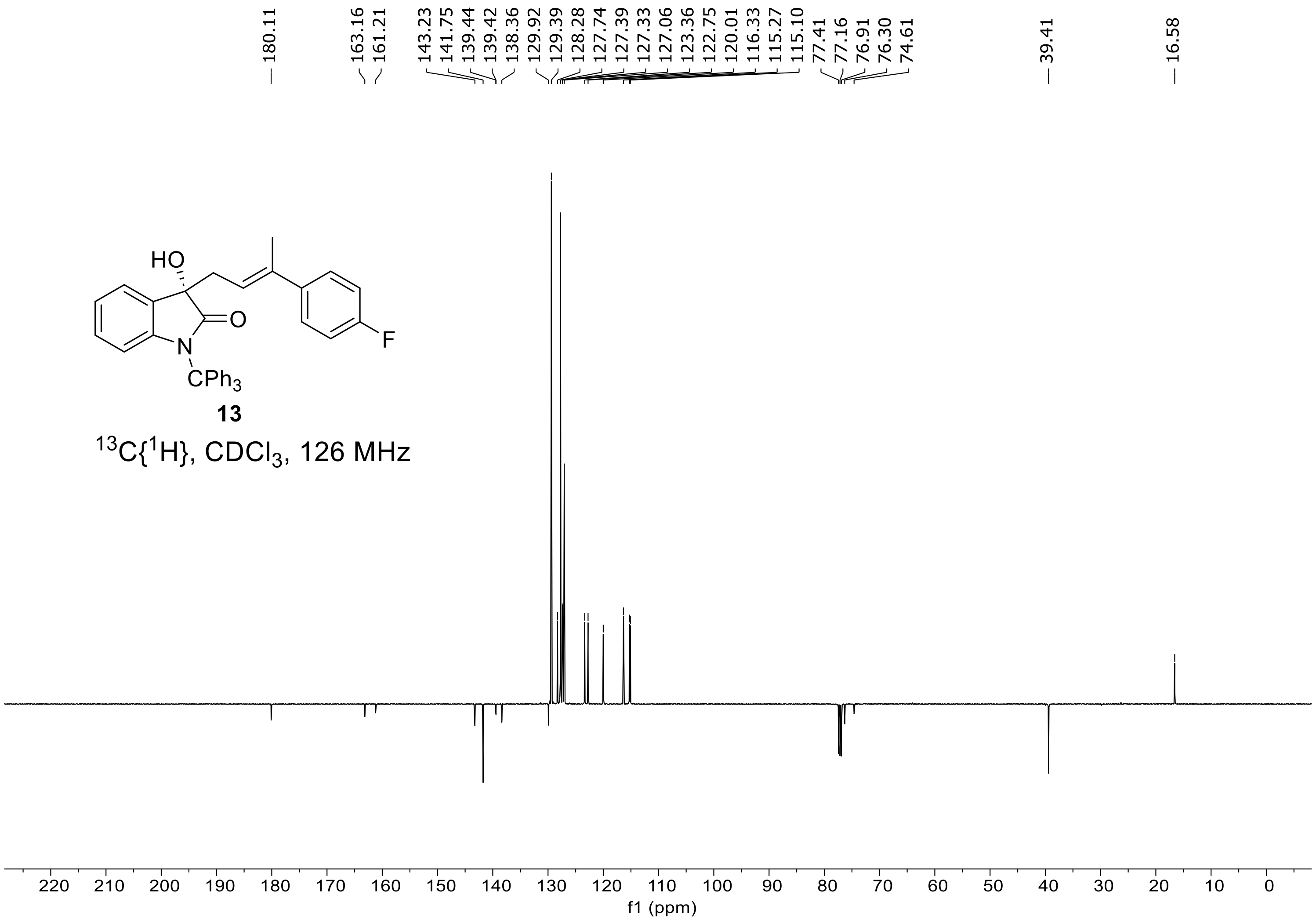

Fig. S328: $^{13}C\{^{1}H\}$ NMR spectrum for (*S*,*E*)-3-[3-(4-Fluorophenyl)but-2-en-1-yl]-3-hydroxy-1-tritylindolin-2-one (**13**).

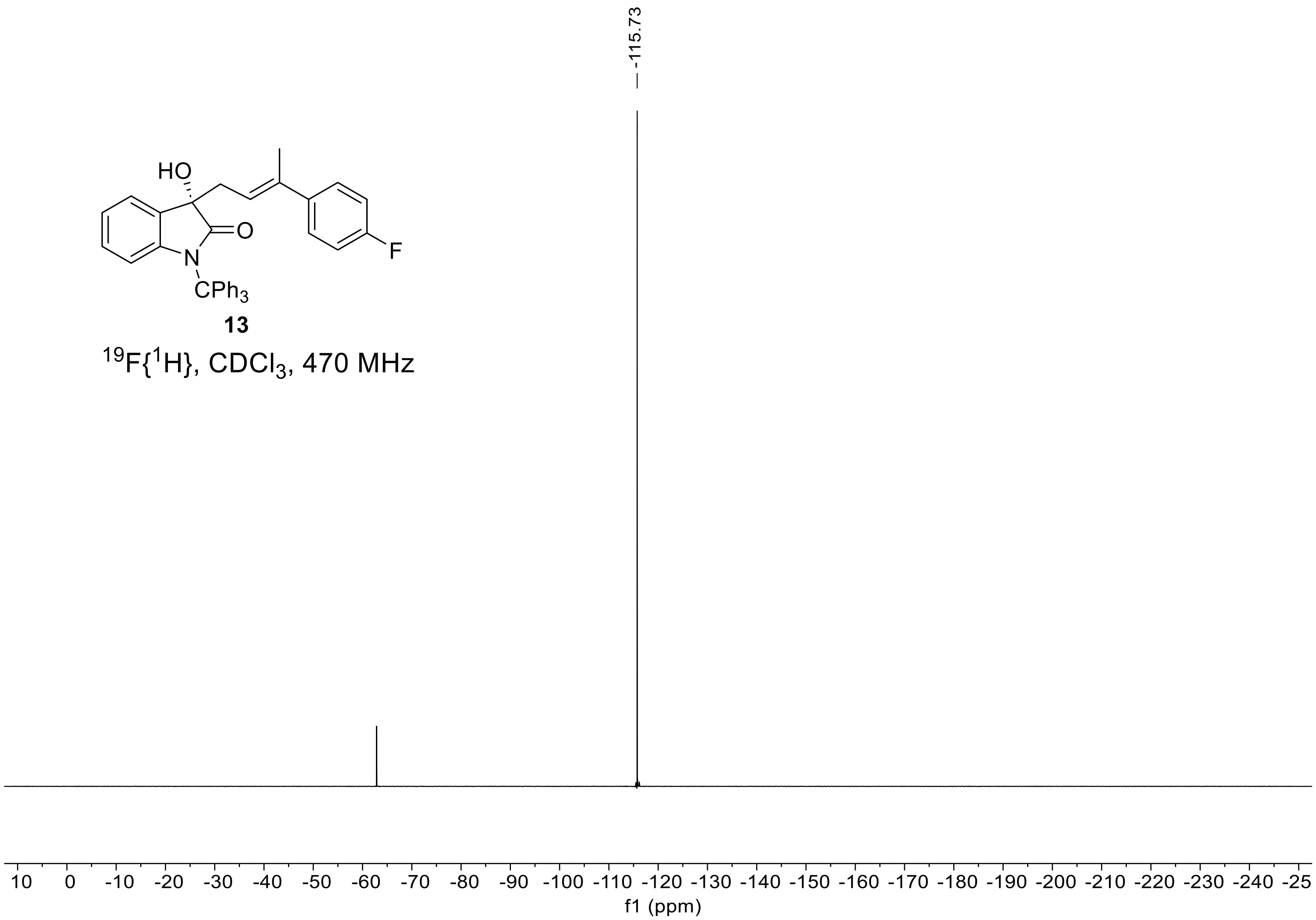

Fig. S329: $^{19}F\{^1H\}$ NMR spectrum for (*S*,*E*)-3-[3-(4-Fluorophenyl)but-2-en-1-yl]-3-hydroxy-1-tritylindolin-2-one (**13**).

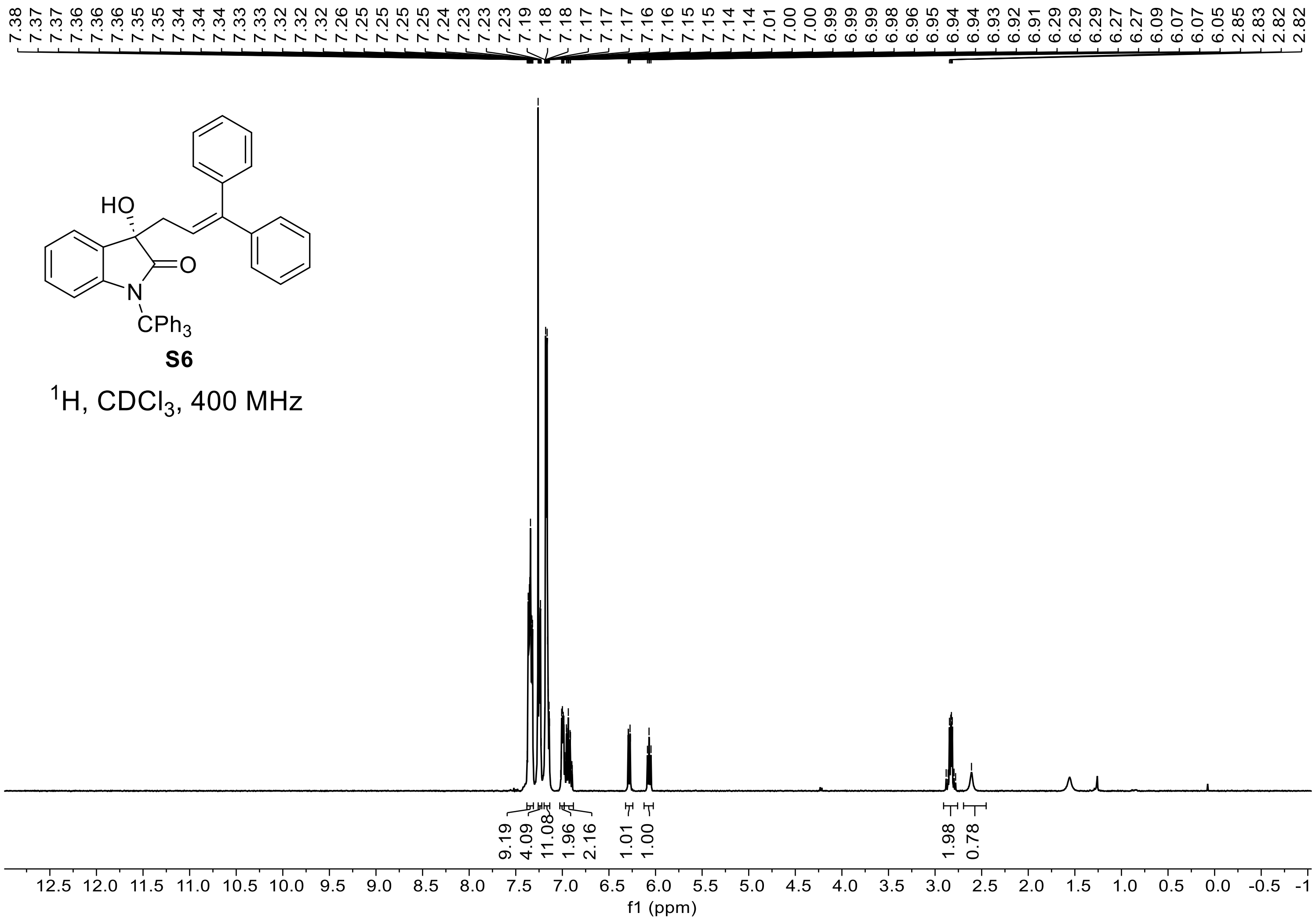

Fig. S330: ^{1}H NMR spectrum for (*S*)-3-(3,3-diphenylallyl)-3-hydroxy-1-tritylindolin-2-one (**S6**).

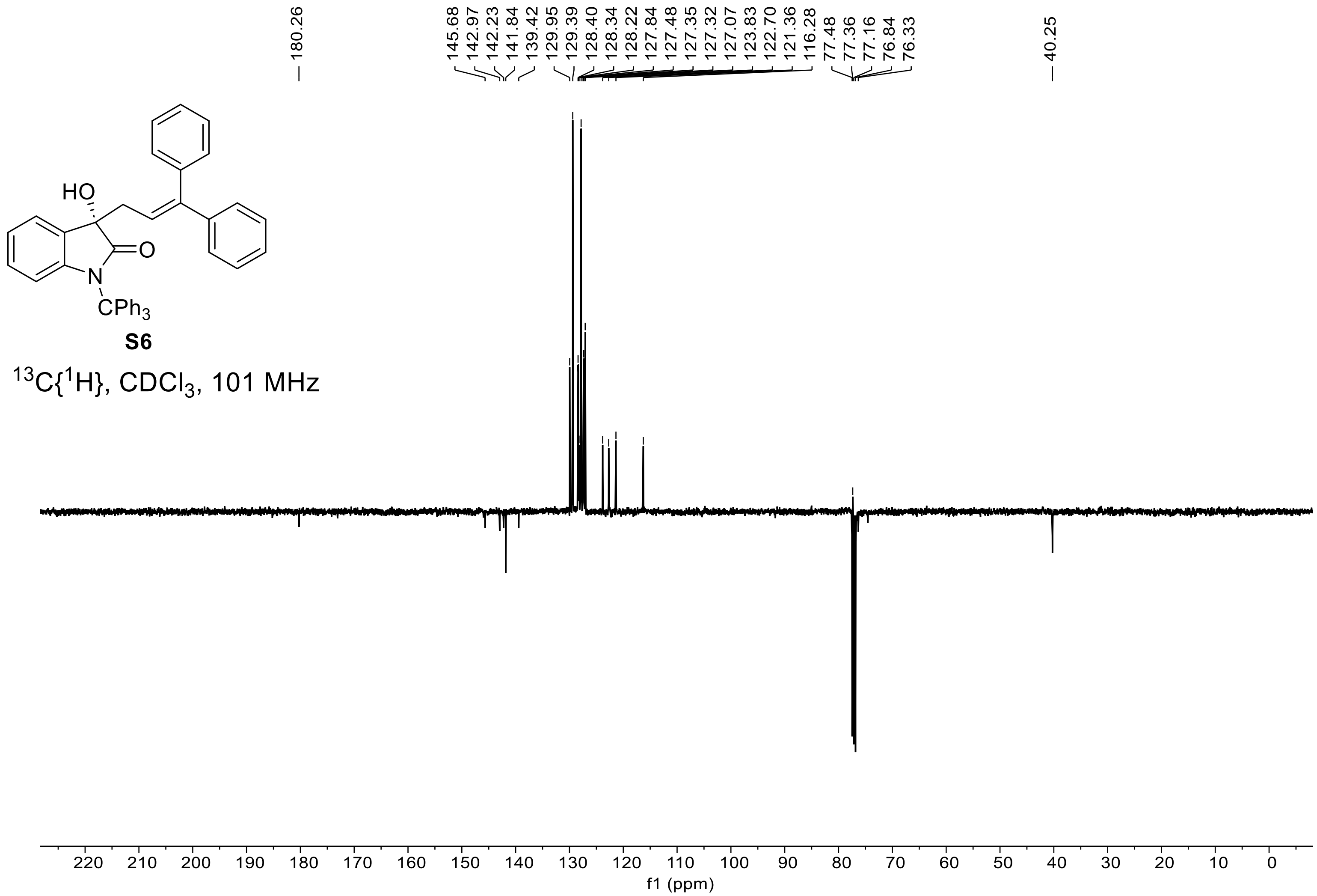

Fig. S331: $^{13}C\{^1H\}$ NMR spectrum for (*S*)-3-(3,3-diphenylallyl)-3-hydroxy-1-tritylindolin-2-one (**S6**).

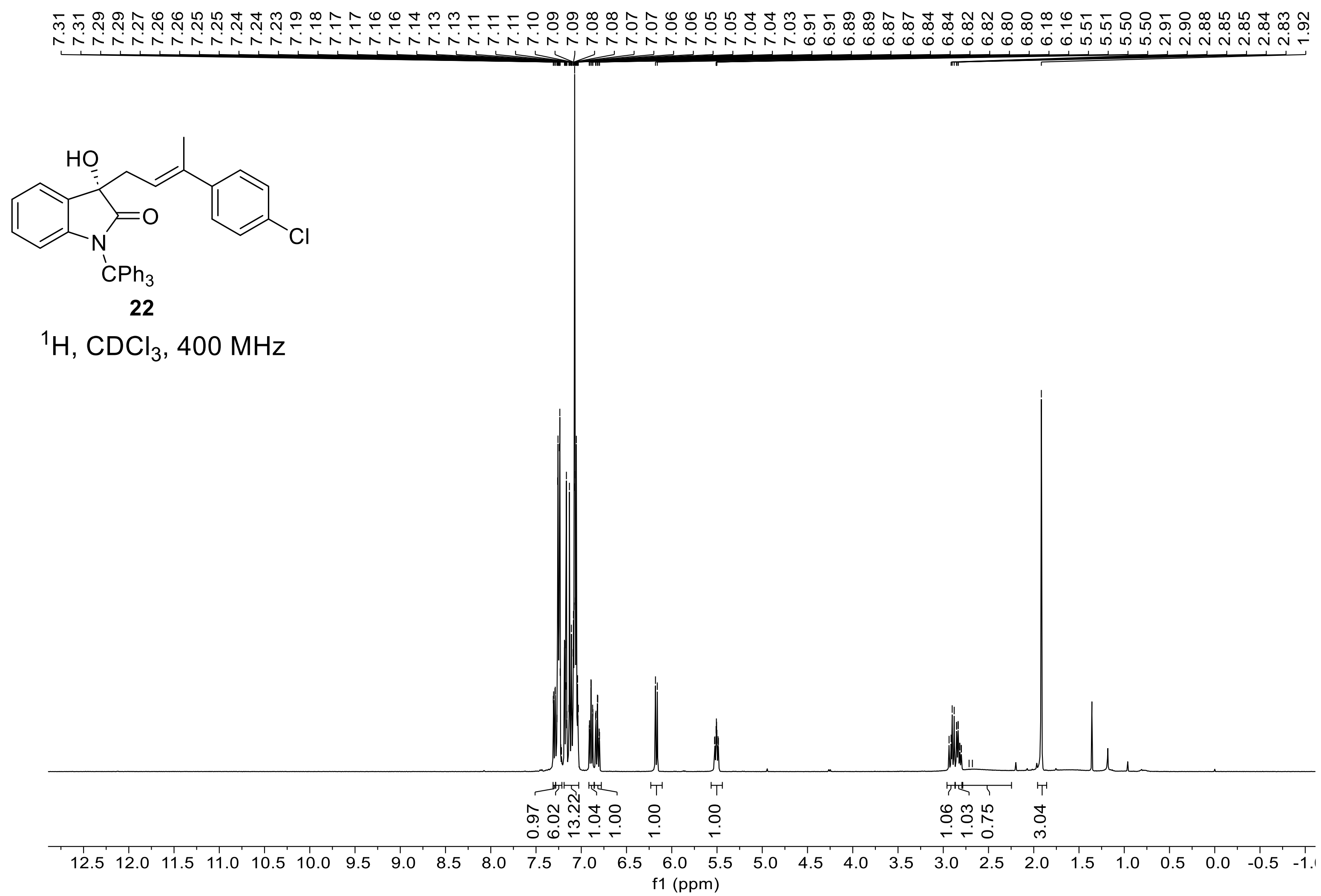

Fig. S332: ^{1}H NMR spectrum for (*S*,*E*)-3-[3-(4-Chlorophenyl)but-2-en-1-yl]-3-hydroxy-1-tritylindolin-2-one (**22**).

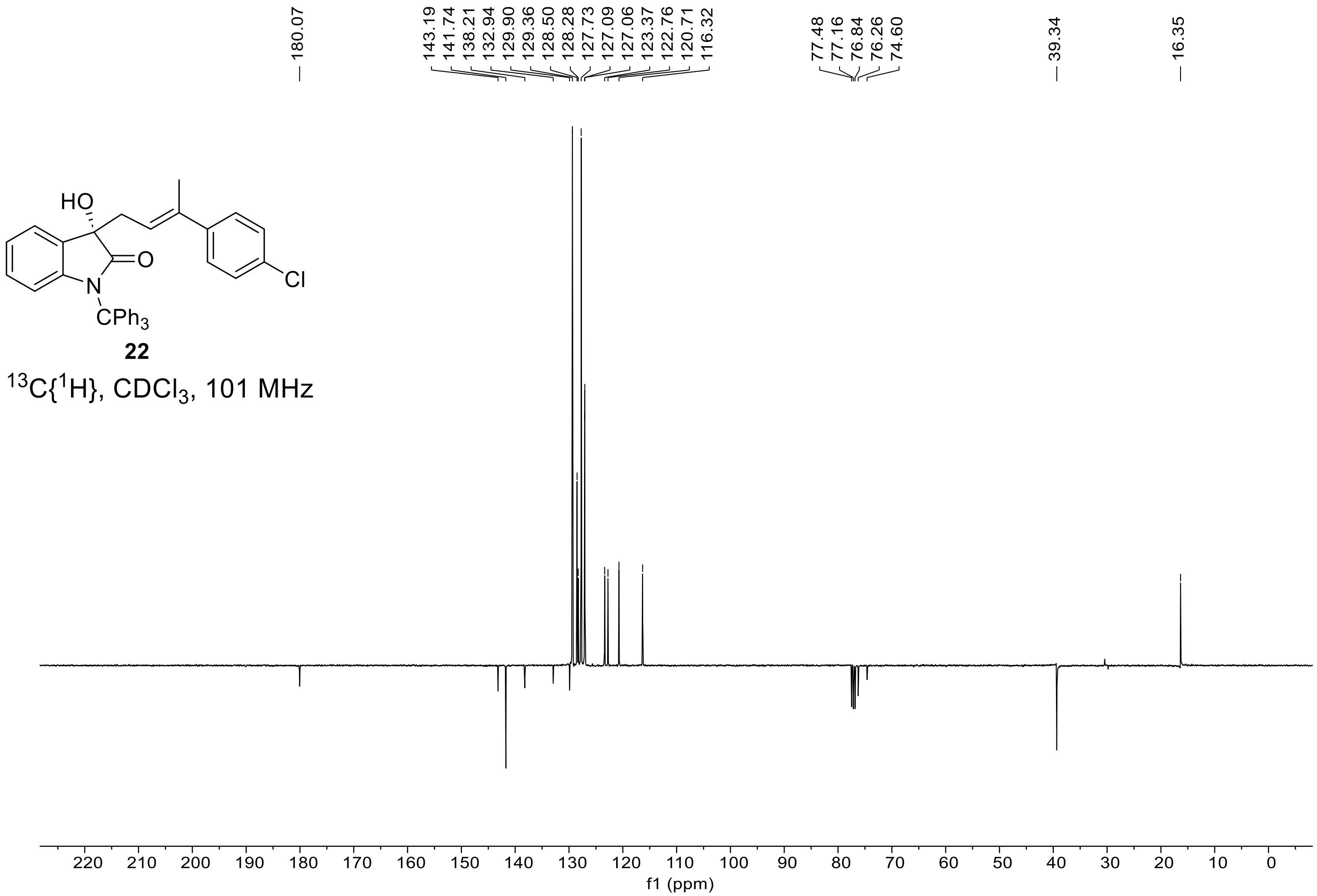

22

$^{13}C\{^1H\}$, $CDCl_3$, 101 MHz

Fig. S333: $^{13}C\{^1H\}$ NMR spectrum for (*S*,*E*)-3-[3-(4-Chlorophenyl)but-2-en-1-yl]-3-hydroxy-1-tritylindolin-2-one (**22**).

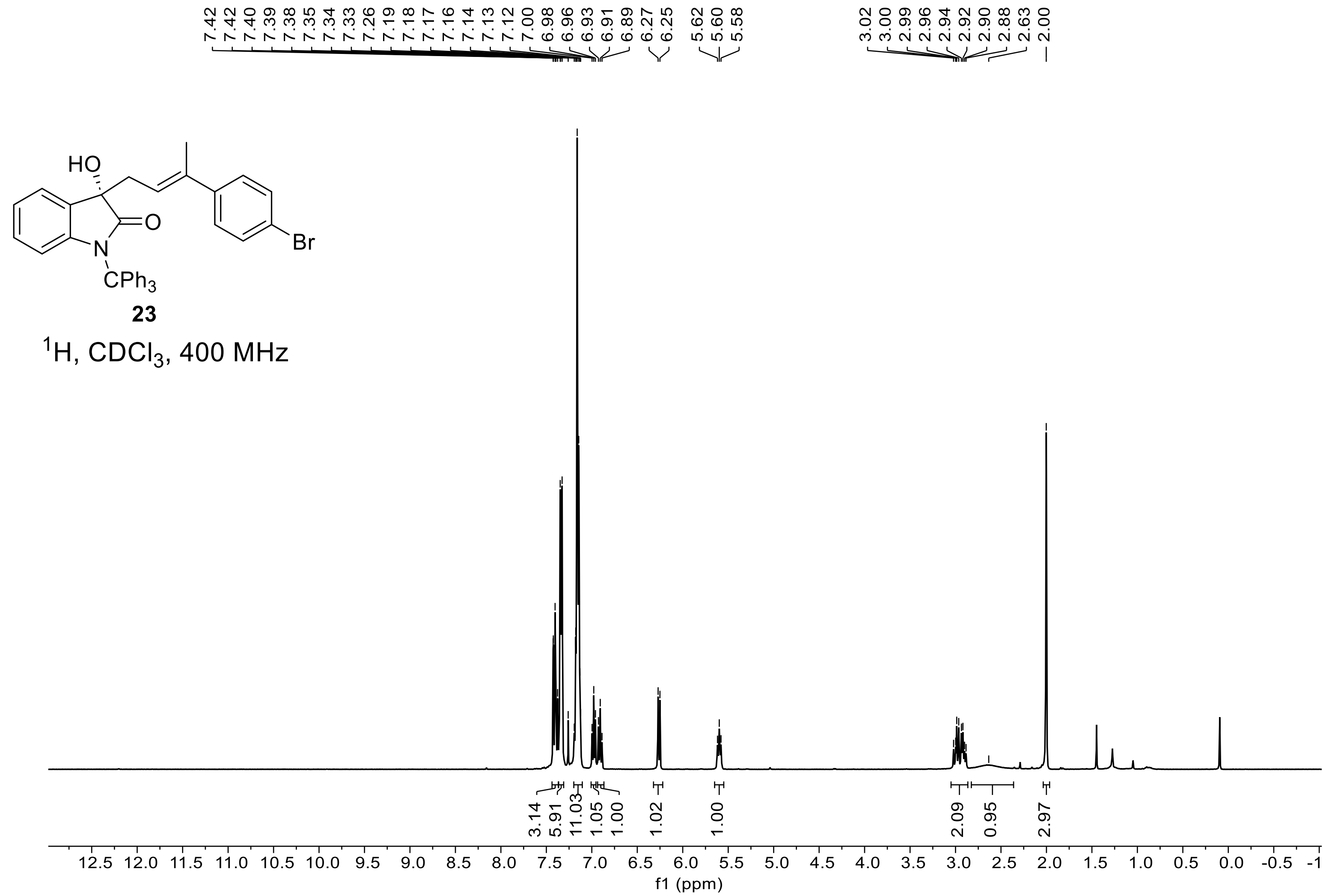

Fig. S334: $^{13}C\{^1H\}$ NMR spectrum for (*S*,*E*)-3-[3-(4-Bromophenyl)but-2-en-1-yl]-3-hydroxy-1-tritylindolin-2-one (**23**).

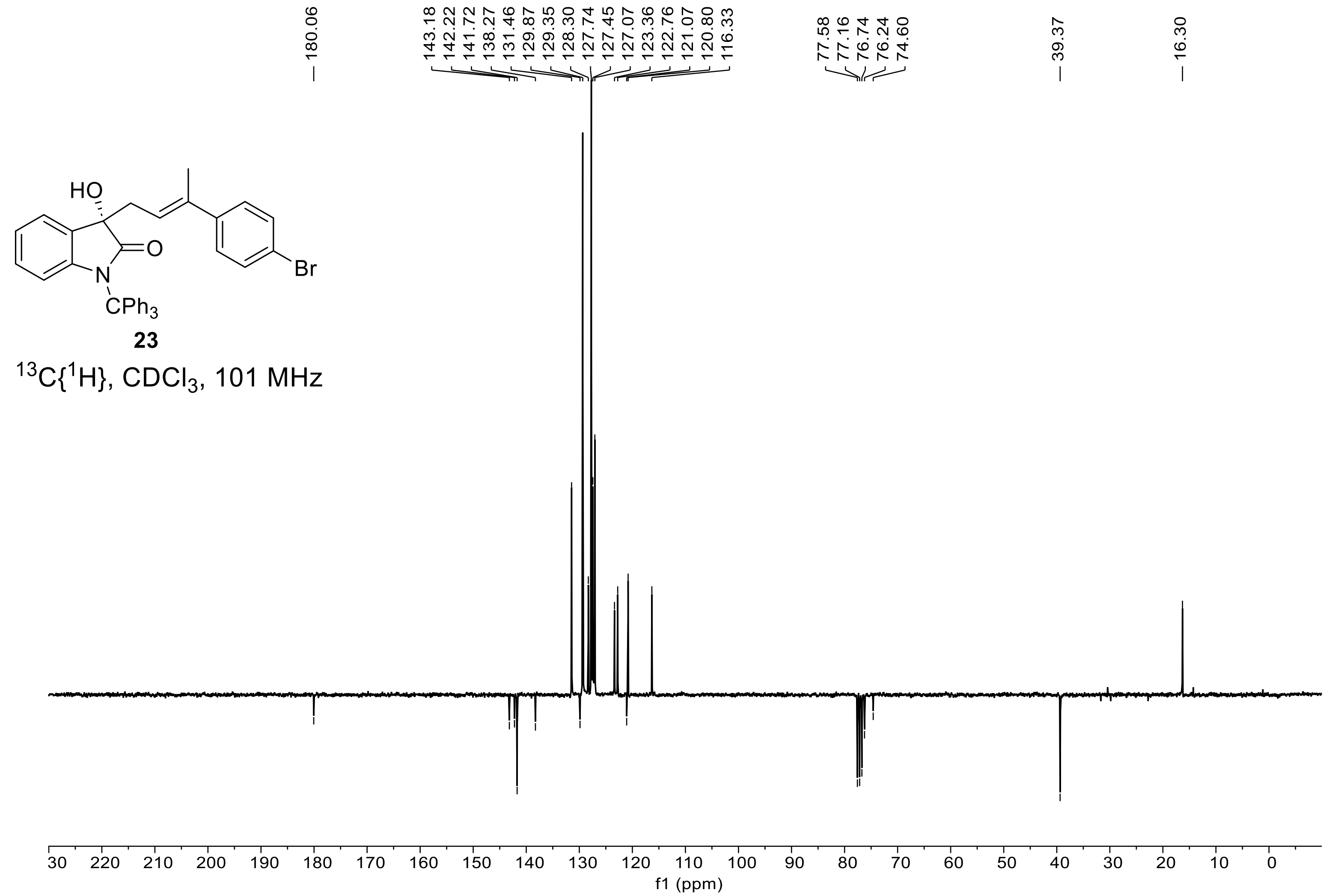

Fig. S335: $^{13}C\{^{1}H\}$ NMR spectrum for (*S*,*E*)-3-[3-(4-Bromophenyl)but-2-en-1-yl]-3-hydroxy-1-tritylindolin-2-one (**23**).

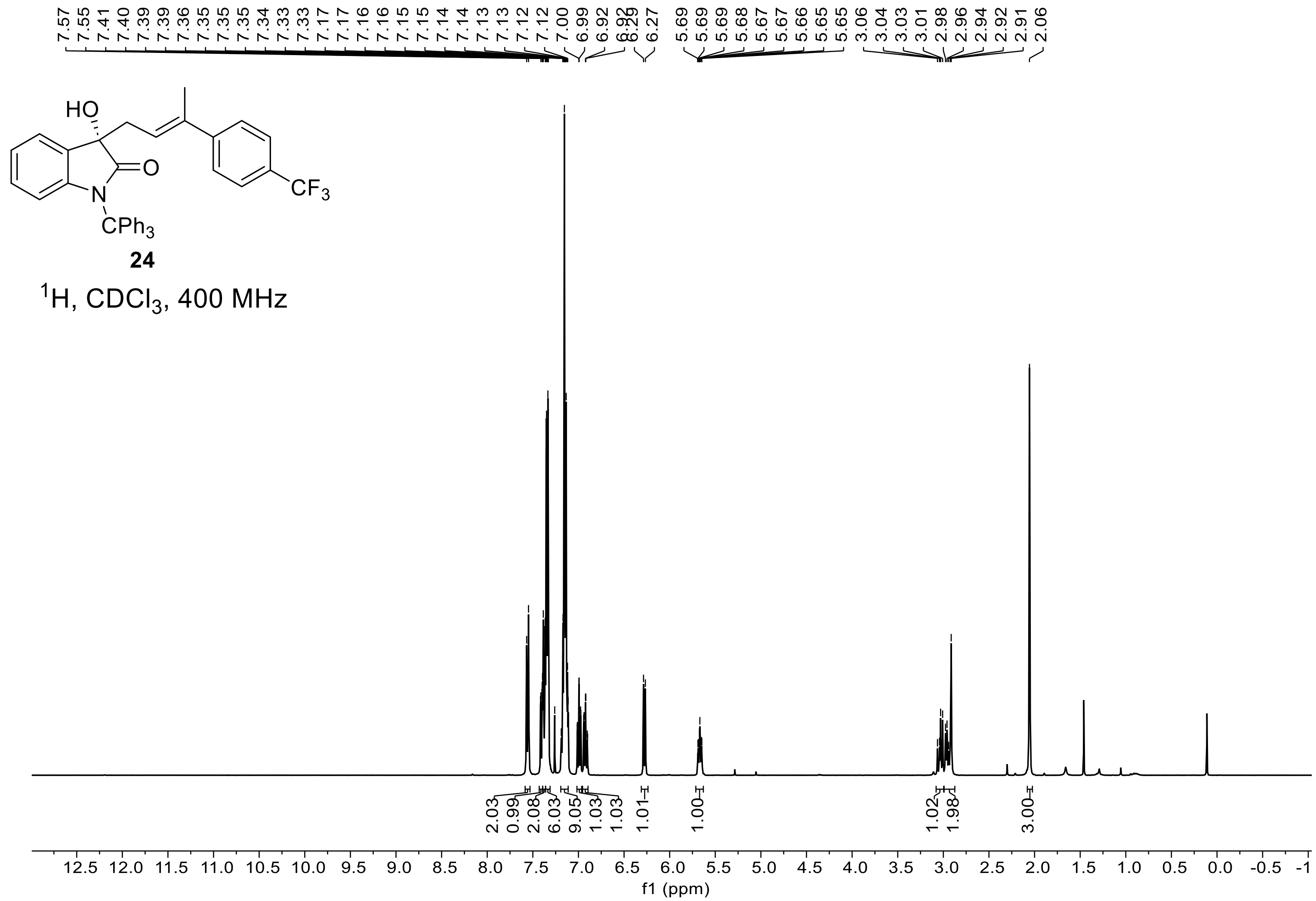

Fig. S336: ^{1}H NMR spectrum for (*S*,*E*)-3-Hydroxy-3-{3-[4-(trifluoromethyl)phenyl]but-2-en-1-yl}-1-tritylindolin-2-one (**24**).

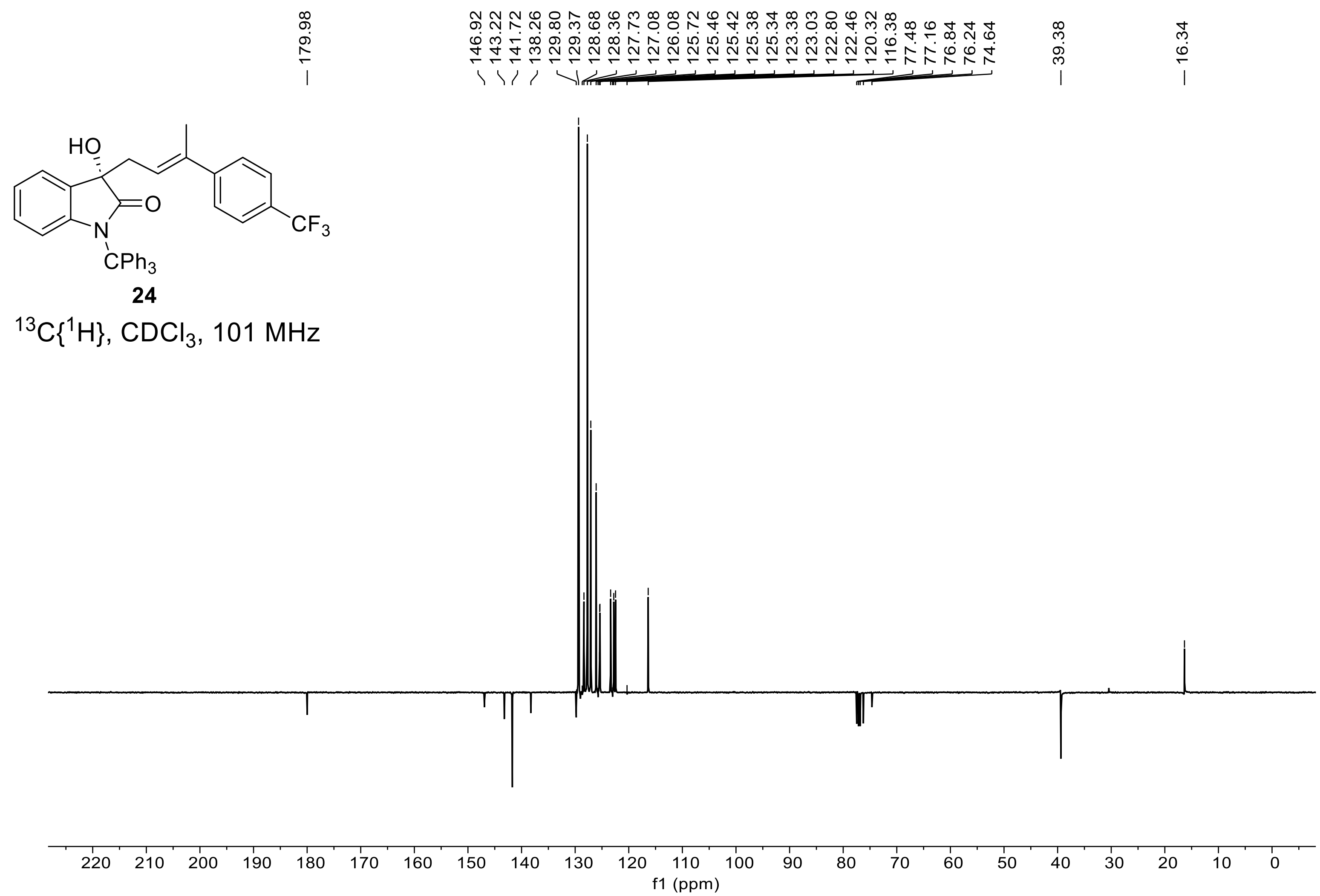

Fig. S337: $^{13}C\{^{1}H\}$ NMR spectrum for (*S*,*E*)-3-Hydroxy-3-{3-[4-(trifluoromethyl)phenyl]but-2-en-1-yl}-1-tritylindolin-2-one (**24**).

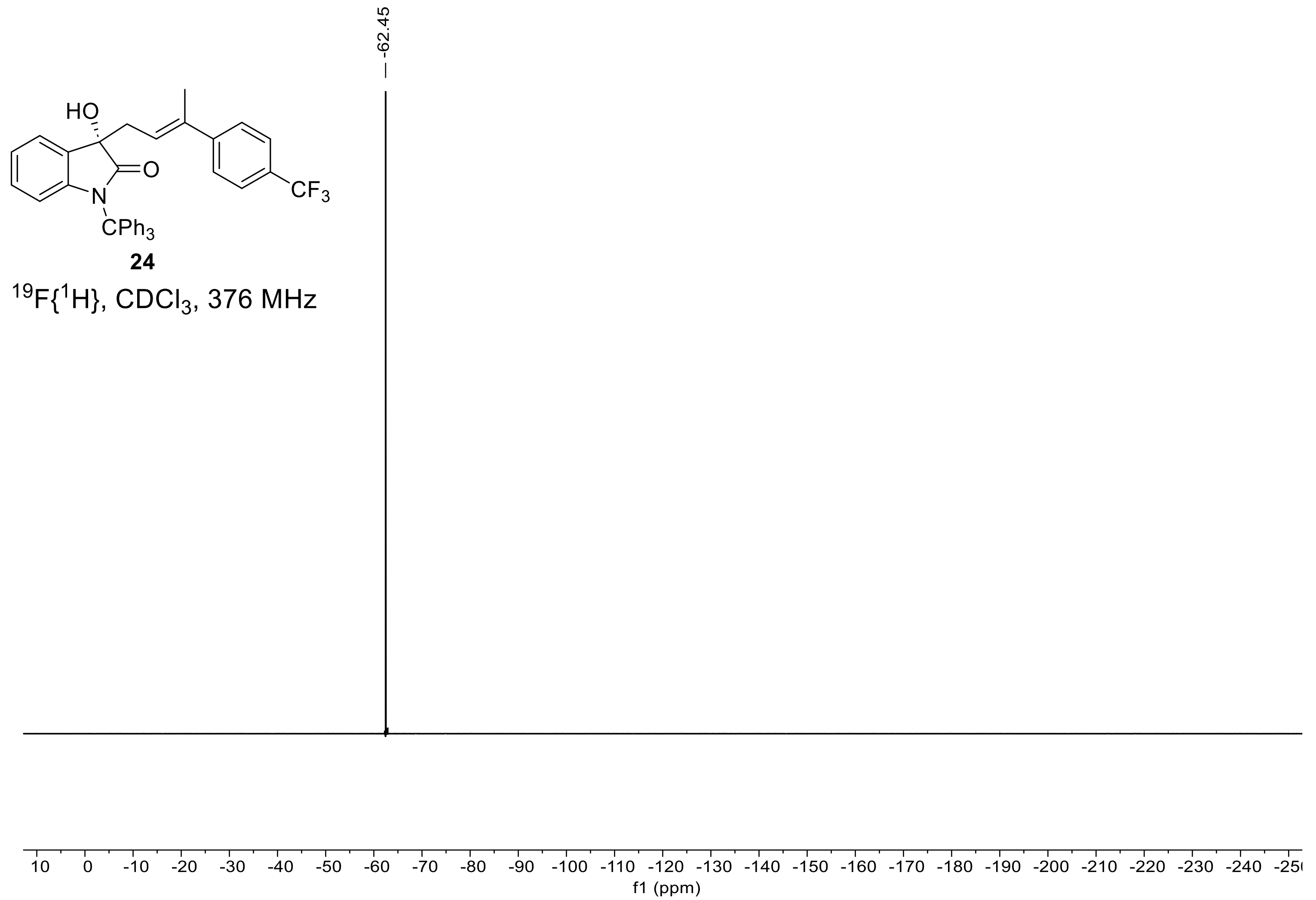

Fig. S338: $^{19}F\{^1H\}$ NMR spectrum for (*S*,*E*)-3-Hydroxy-3-{3-[4-(trifluoromethyl)phenyl]but-2-en-1-yl}-1-tritylindolin-2-one (**24**).

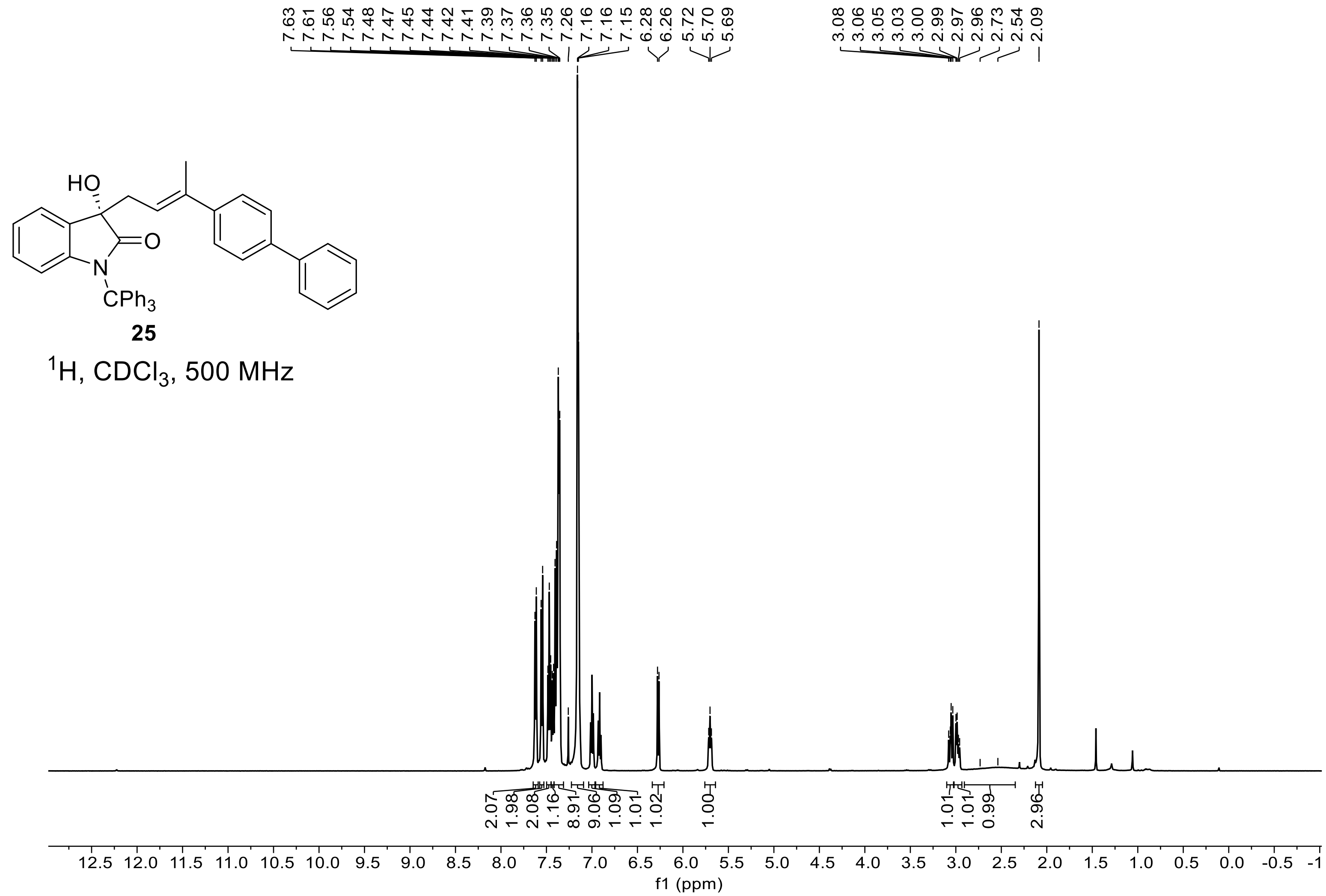

Fig. S339: ^{1}H NMR spectrum for (*S*,*E*)-3-{3-[(1,1'-biphenyl)-4-yl]but-2-en-1-yl}-3-hydroxy-1-tritylindolin-2-one (**25**).

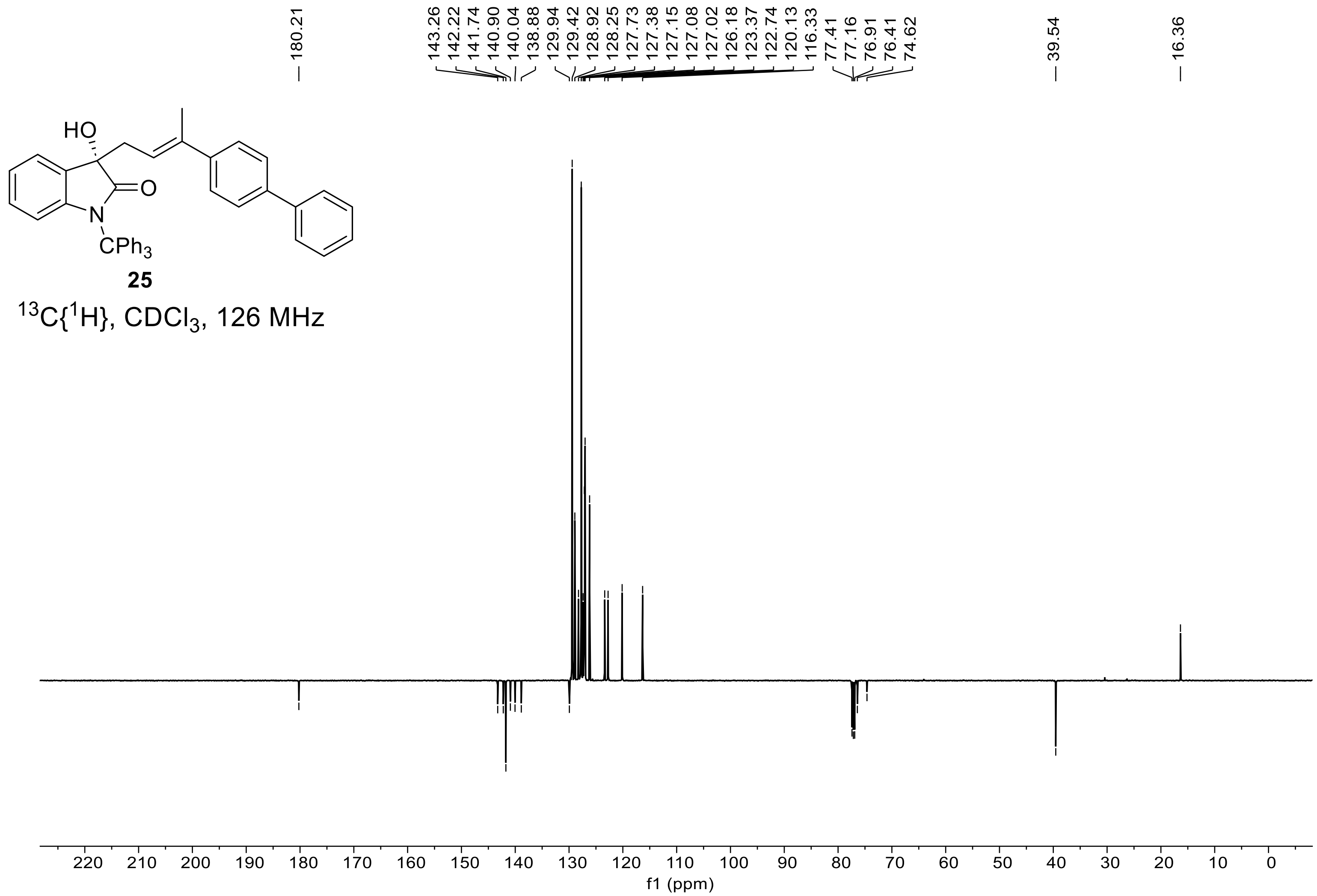

Fig. S340: $^{13}C\{^1H\}$ NMR spectrum for (*S*,*E*)-3-{3-[(1,1'-biphenyl)-4-yl]but-2-en-1-yl}-3-hydroxy-1-tritylindolin-2-one (**25**).

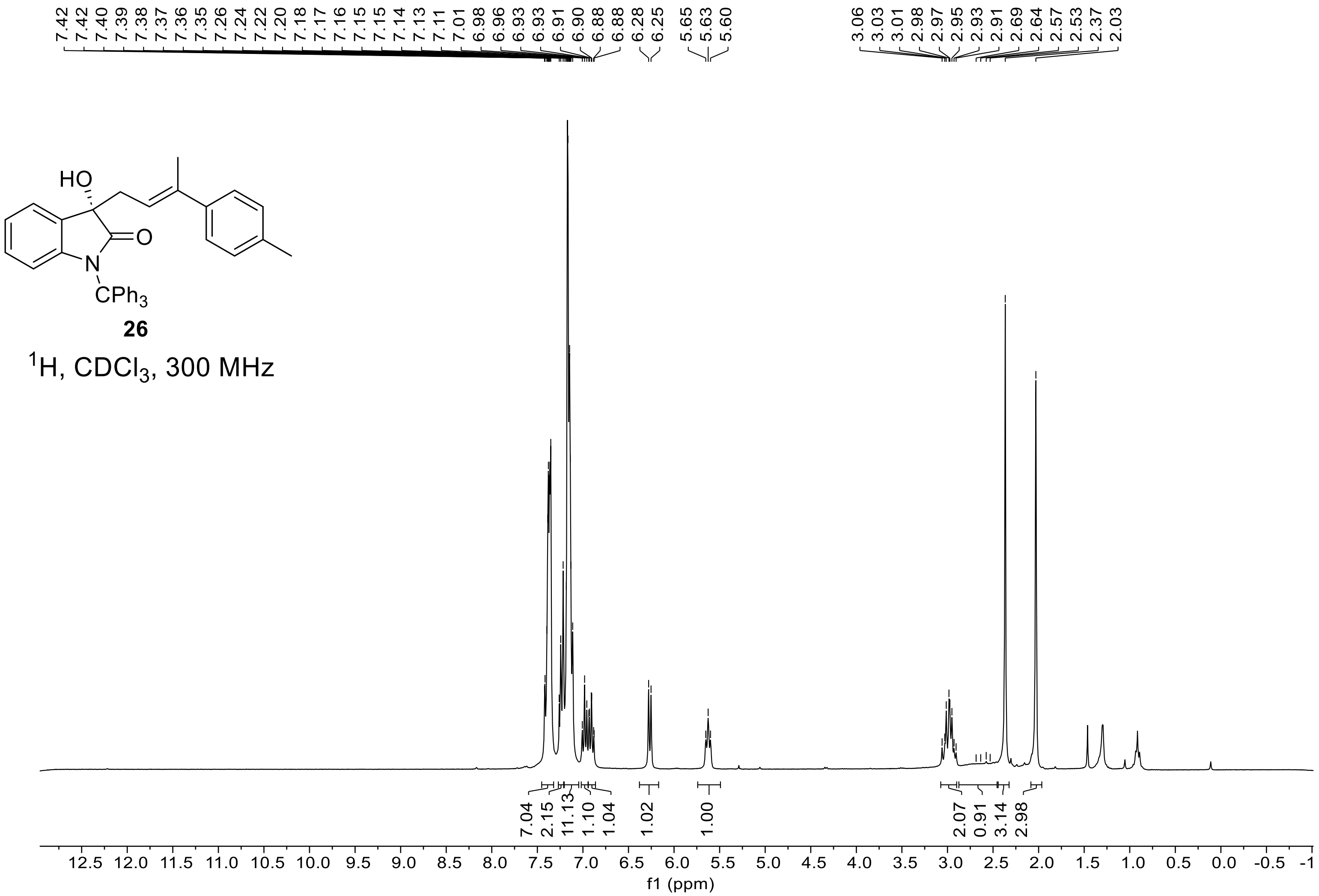

Fig. S341: ^{1}H NMR spectrum for (*S*,*E*)-3-Hydroxy-3-[3-(p-tolyl)but-2-en-1-yl]-1-tritylindolin-2-one (**26**).

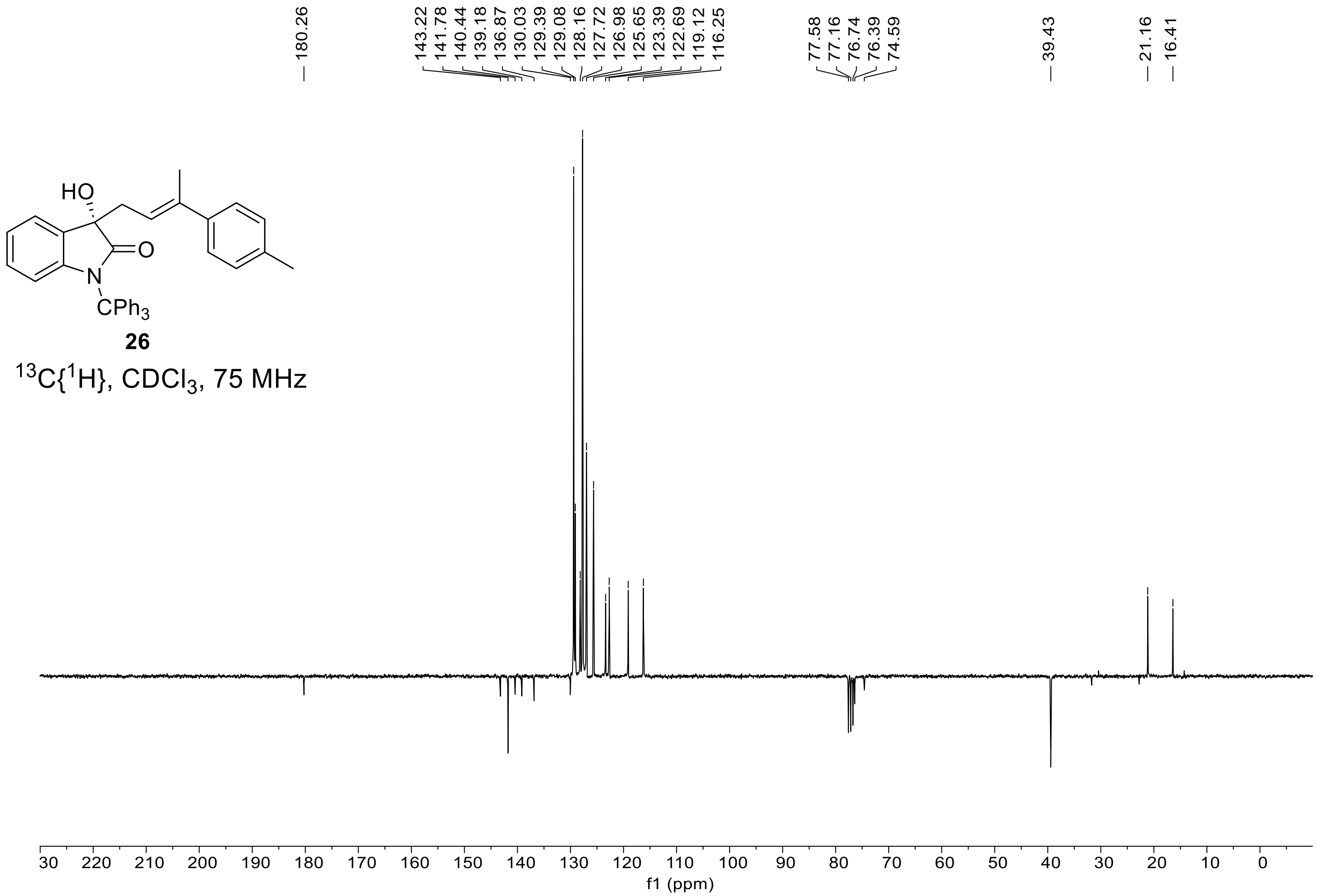

Fig. S342: $^{13}C\{^1H\}$ NMR spectrum for (*S*,*E*)-3-Hydroxy-3-[3-(p-tolyl)but-2-en-1-yl]-1-tritylindolin-2-one (**26**).

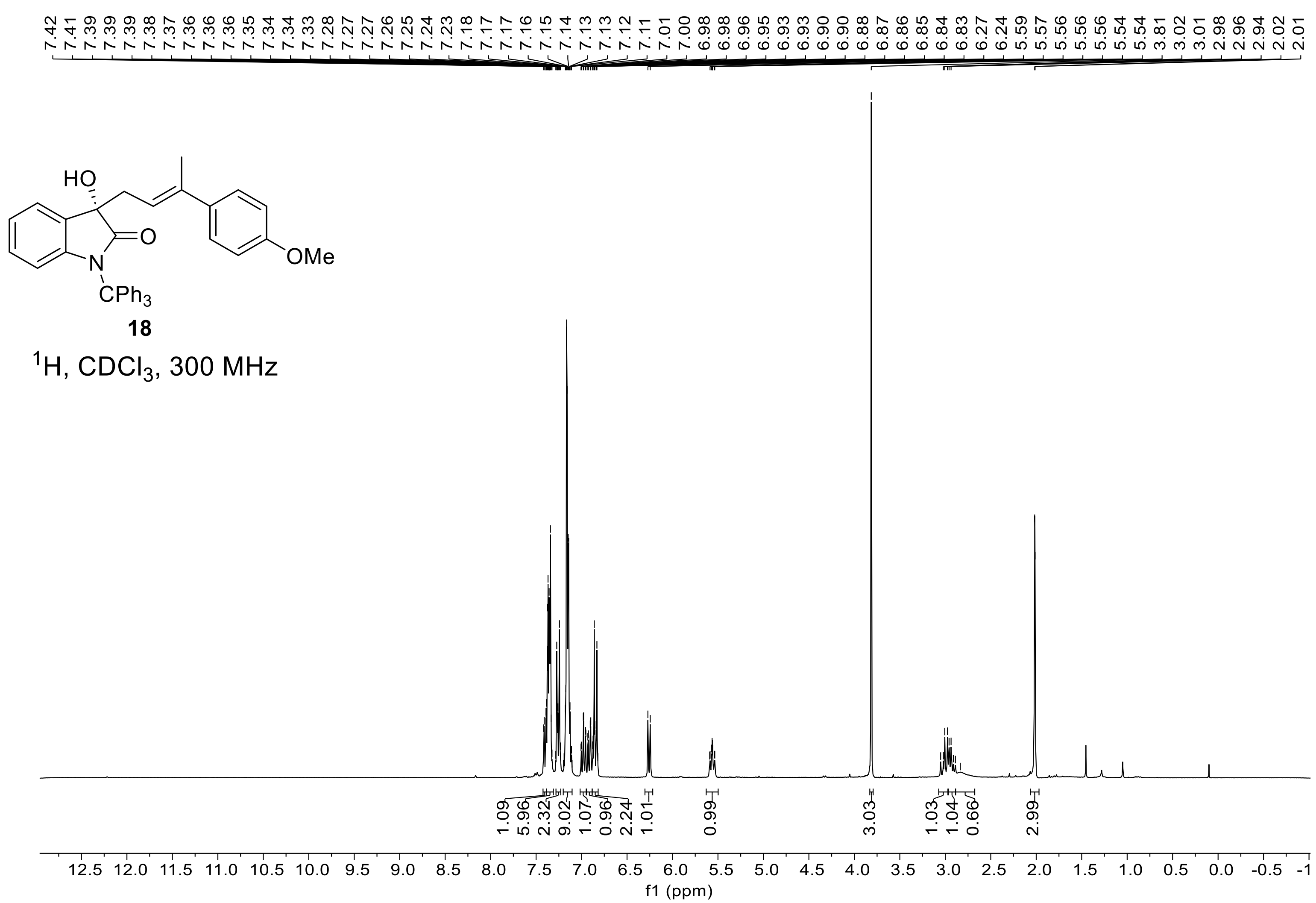

Fig. S343: ^{1}H NMR spectrum for (*S*,*E*)-3-Hydroxy-3-[3-(4-methoxyphenyl)but-2-en-1-yl]-1-tritylindolin-2-one (**18**).

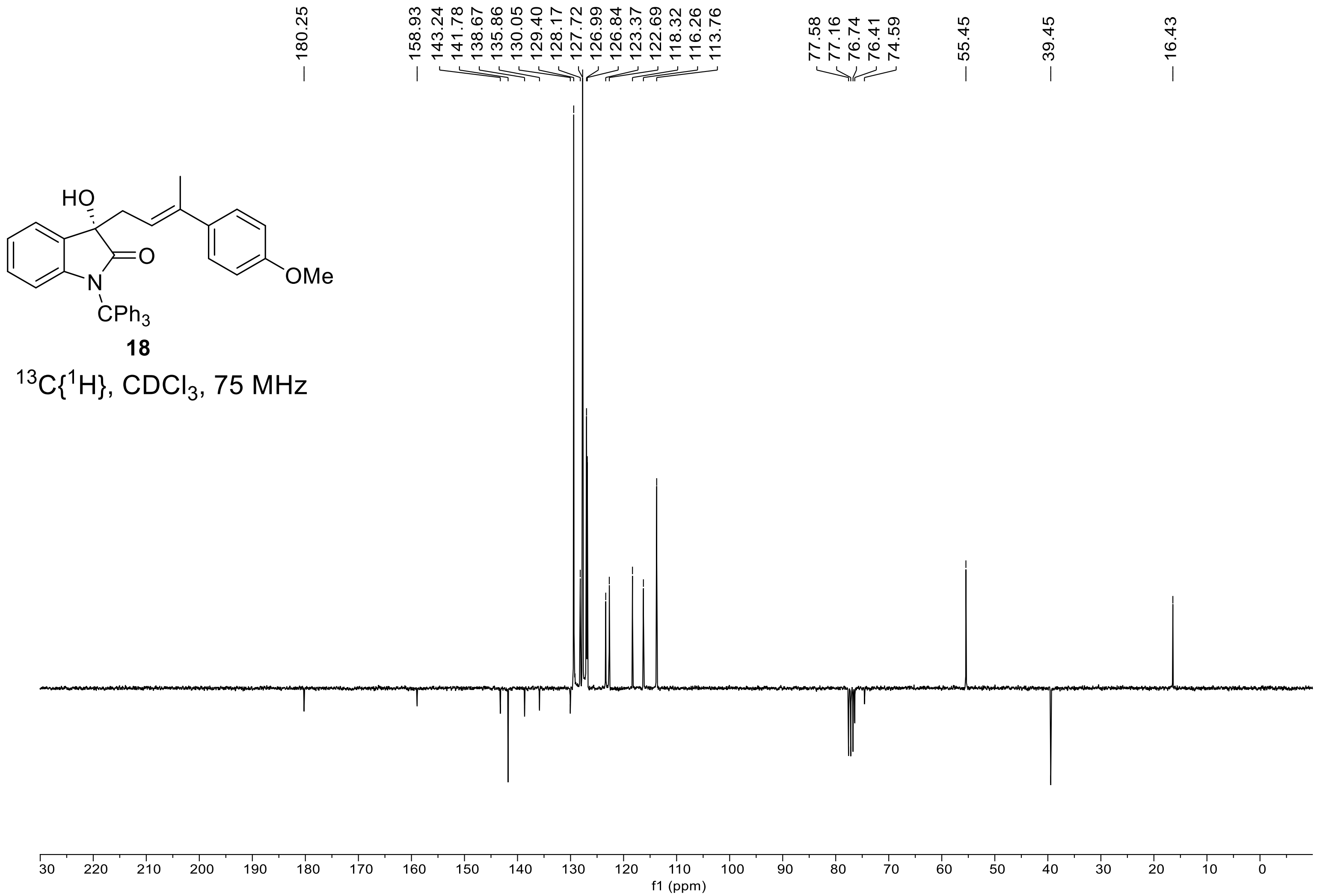

Fig. S344: $^{13}C\{^1H\}$ NMR spectrum for (*S*,*E*)-3-Hydroxy-3-[3-(4-methoxyphenyl)but-2-en-1-yl]-1-tritylindolin-2-one (**18**).

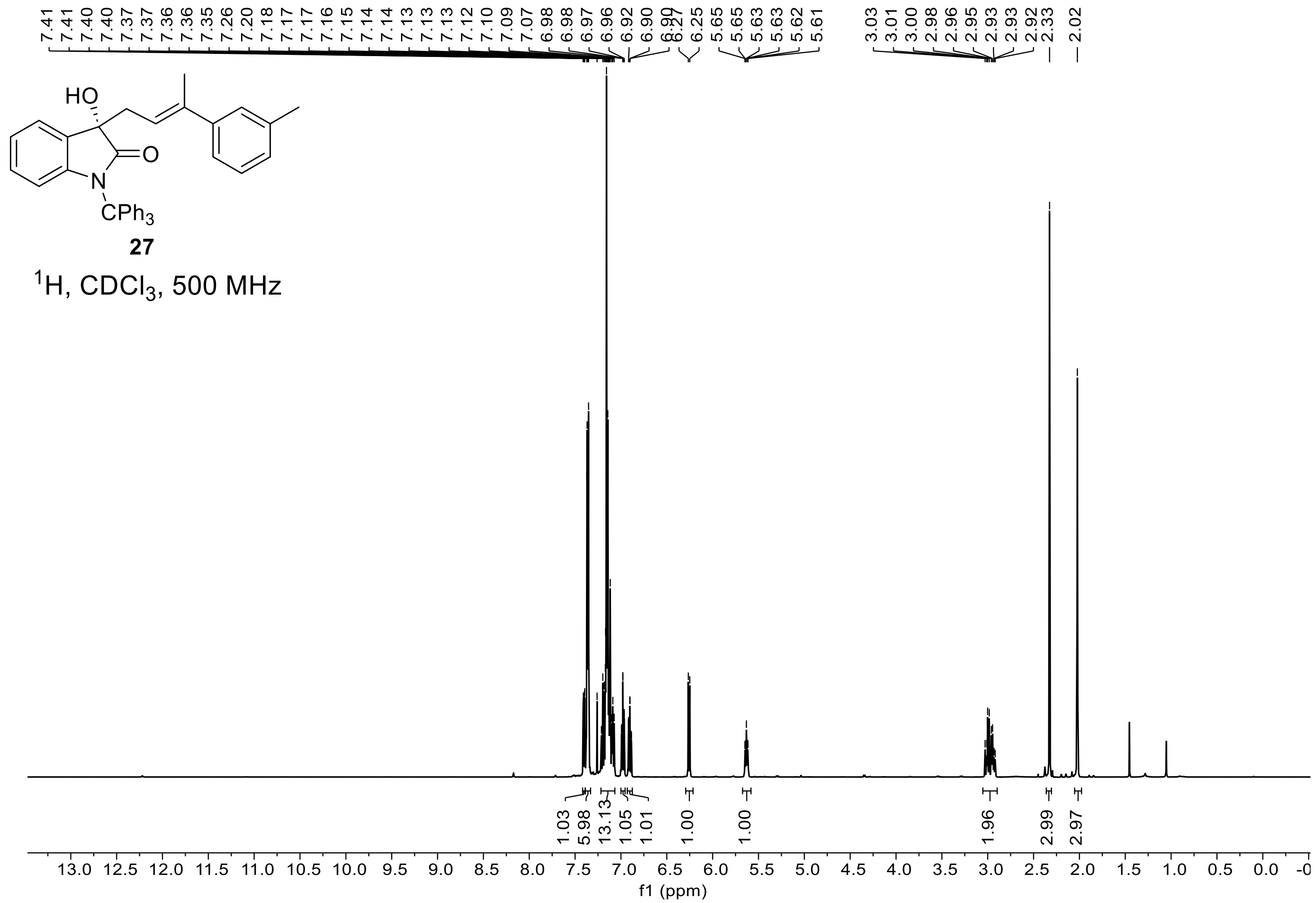

Fig. S345: ^{1}H NMR spectrum for (*S*,*E*)-3-Hydroxy-3-[3-(*m*-tolyl)but-2-en-1-yl]-1-tritylindolin-2-one (**27**).

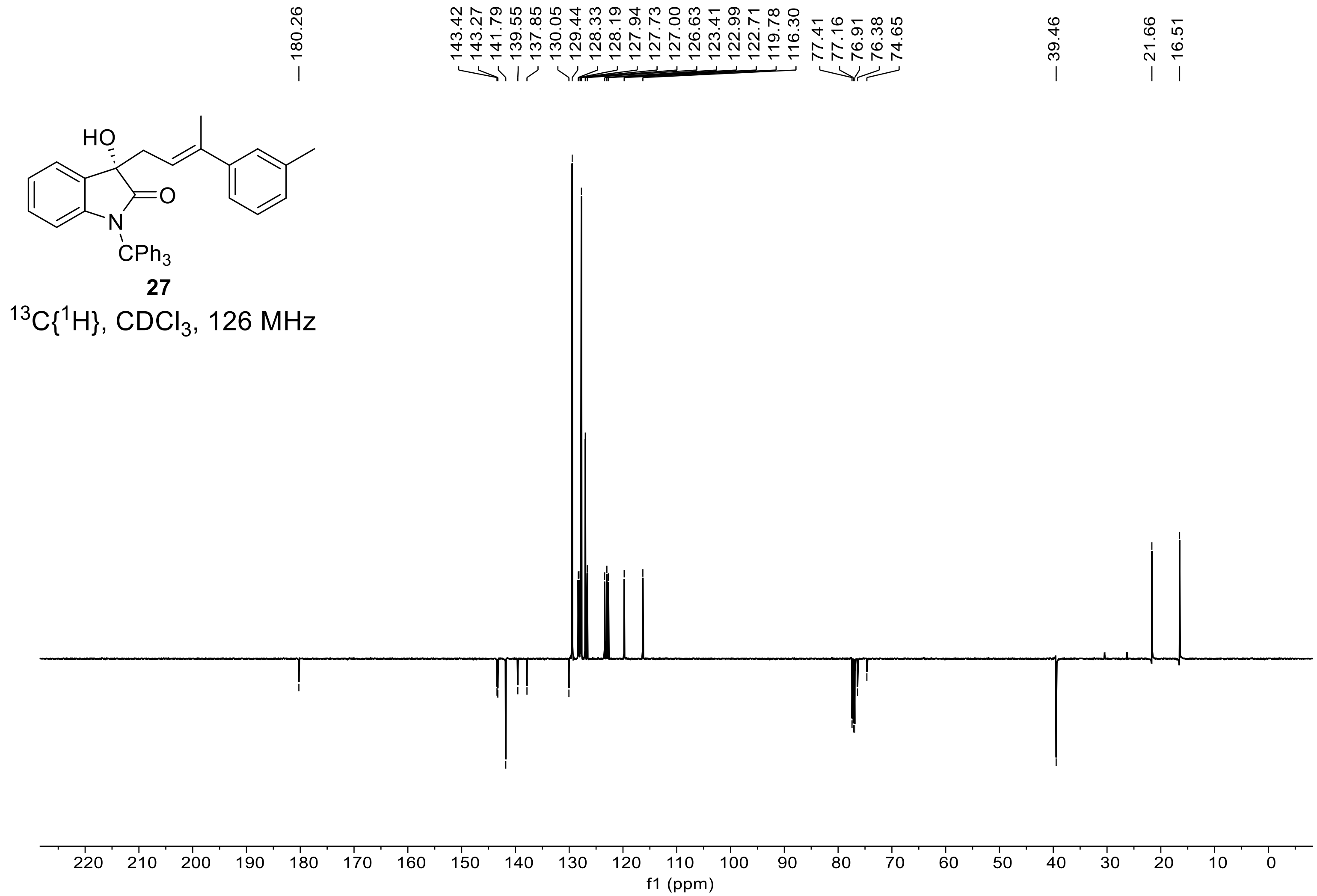

Fig. S346: $^{13}C\{^1H\}$ NMR spectrum for (*S*,*E*)-3-Hydroxy-3-[3-(*m*-tolyl)but-2-en-1-yl]-1-tritylindolin-2-one (**27**).

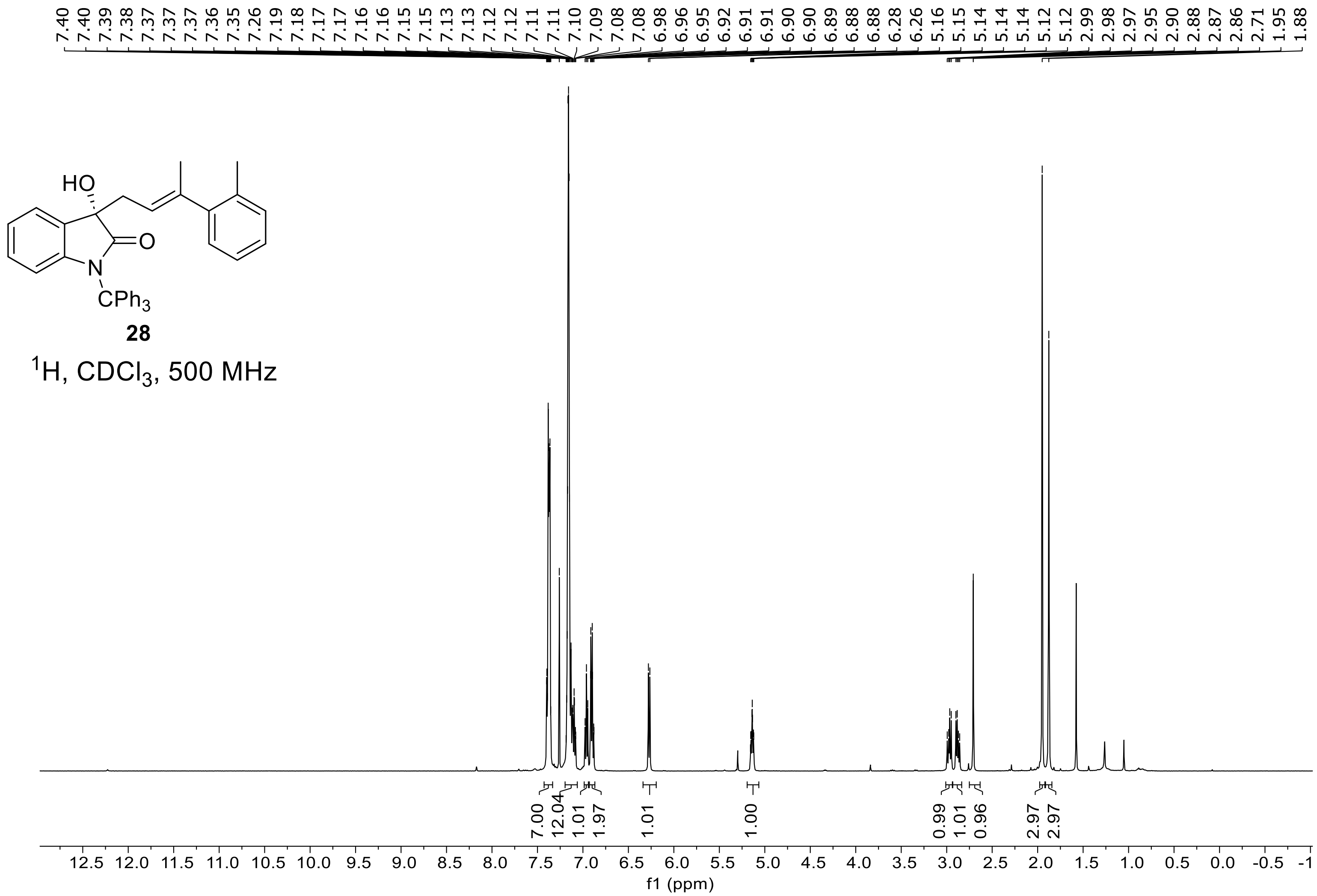

Fig. S347: ^{1}H NMR spectrum for (*S*,*E*)-3-Hydroxy-3-[3-(*o*-tolyl)but-2-en-1-yl]-1-tritylindolin-2-one (**28**).

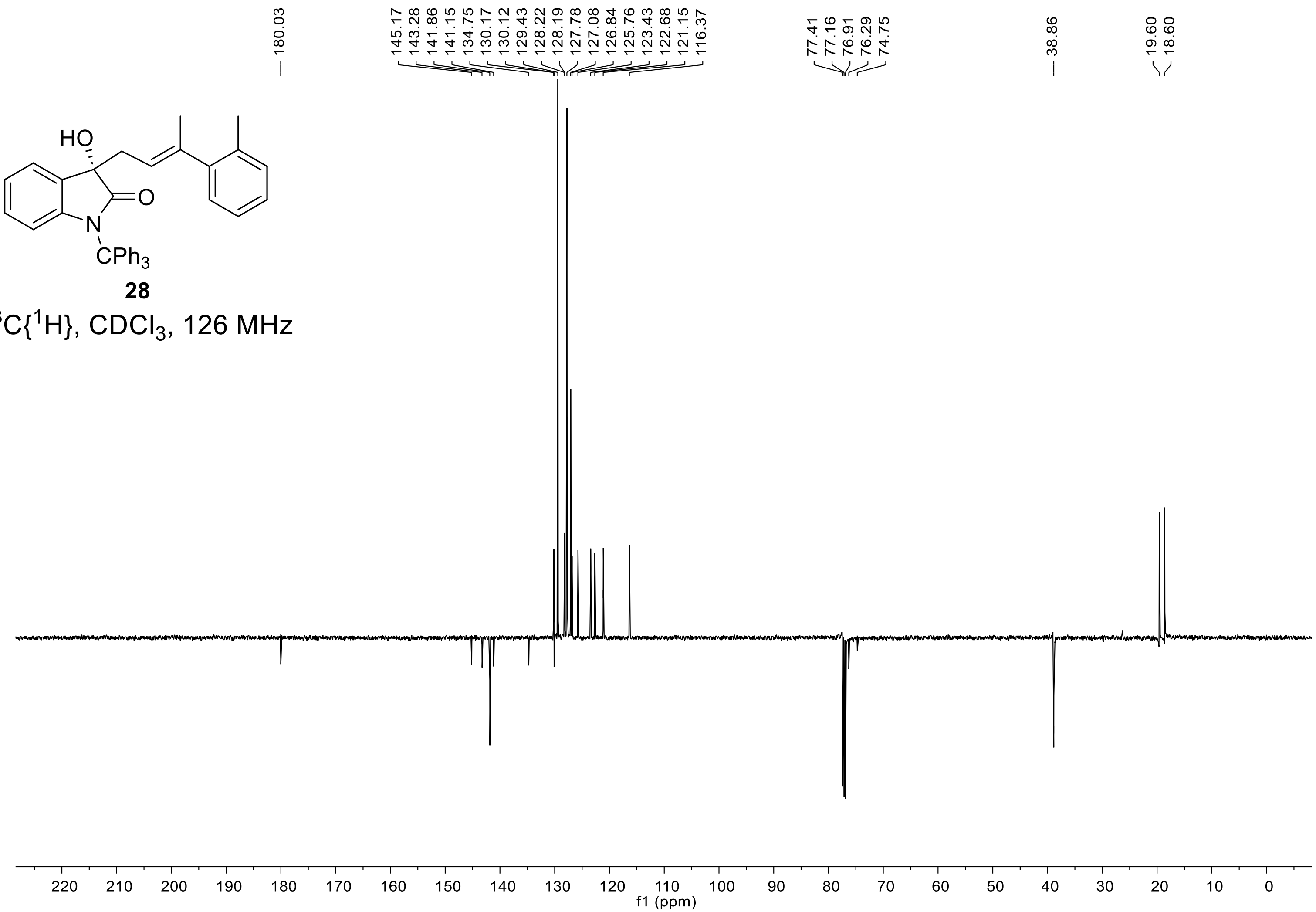

Fig. S348: $^{13}C\{^{1}H\}$ NMR spectrum for (*S*,*E*)-3-Hydroxy-3-[3-(*o*-tolyl)but-2-en-1-yl]-1-tritylindolin-2-one (**28**).

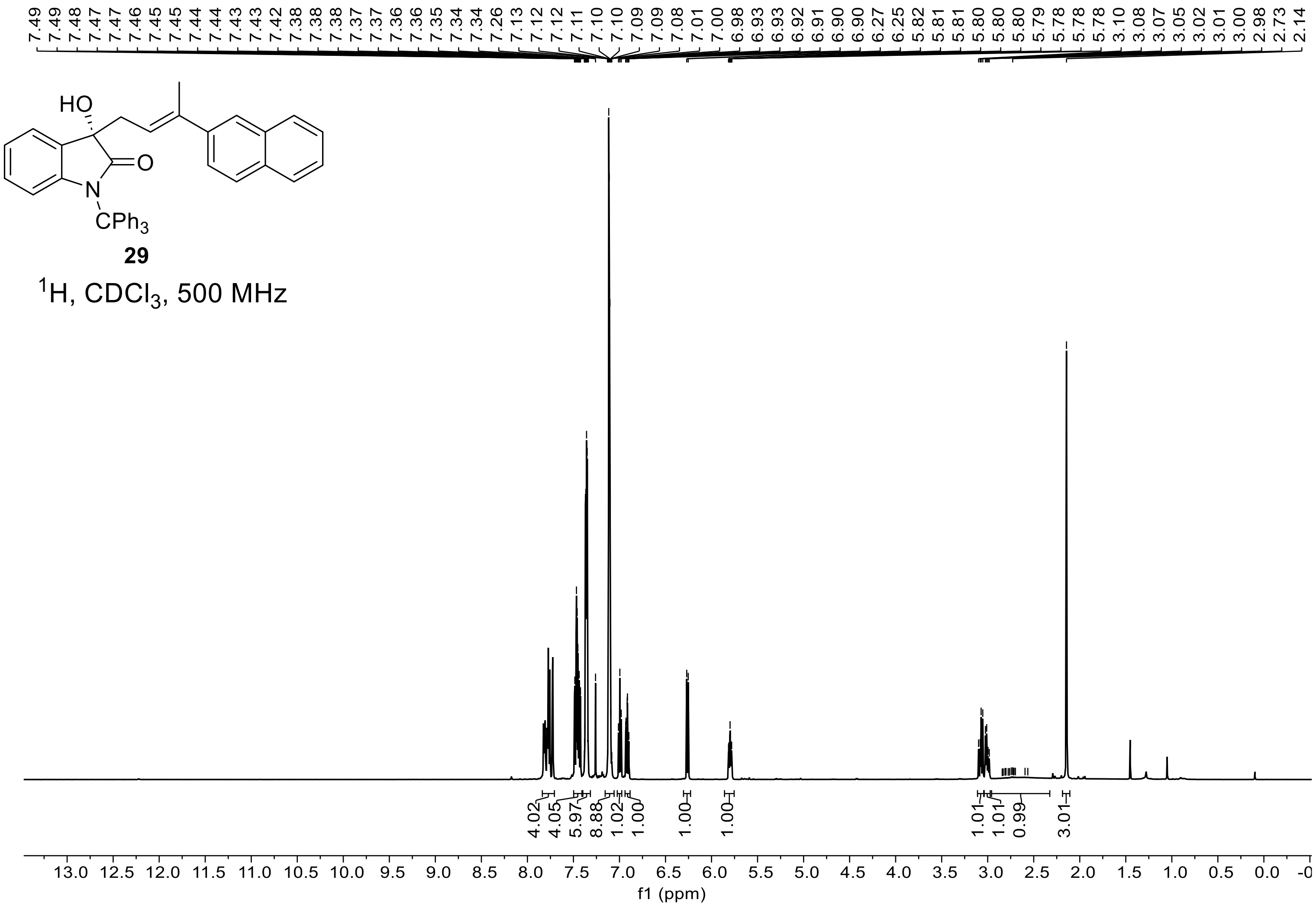

Fig. S349: ^{1}H NMR spectrum for (*S*,*E*)-3-Hydroxy-3-[3-(naphthalen-2-yl)but-2-en-1-yl]-1-tritylindolin-2-one (**29**).

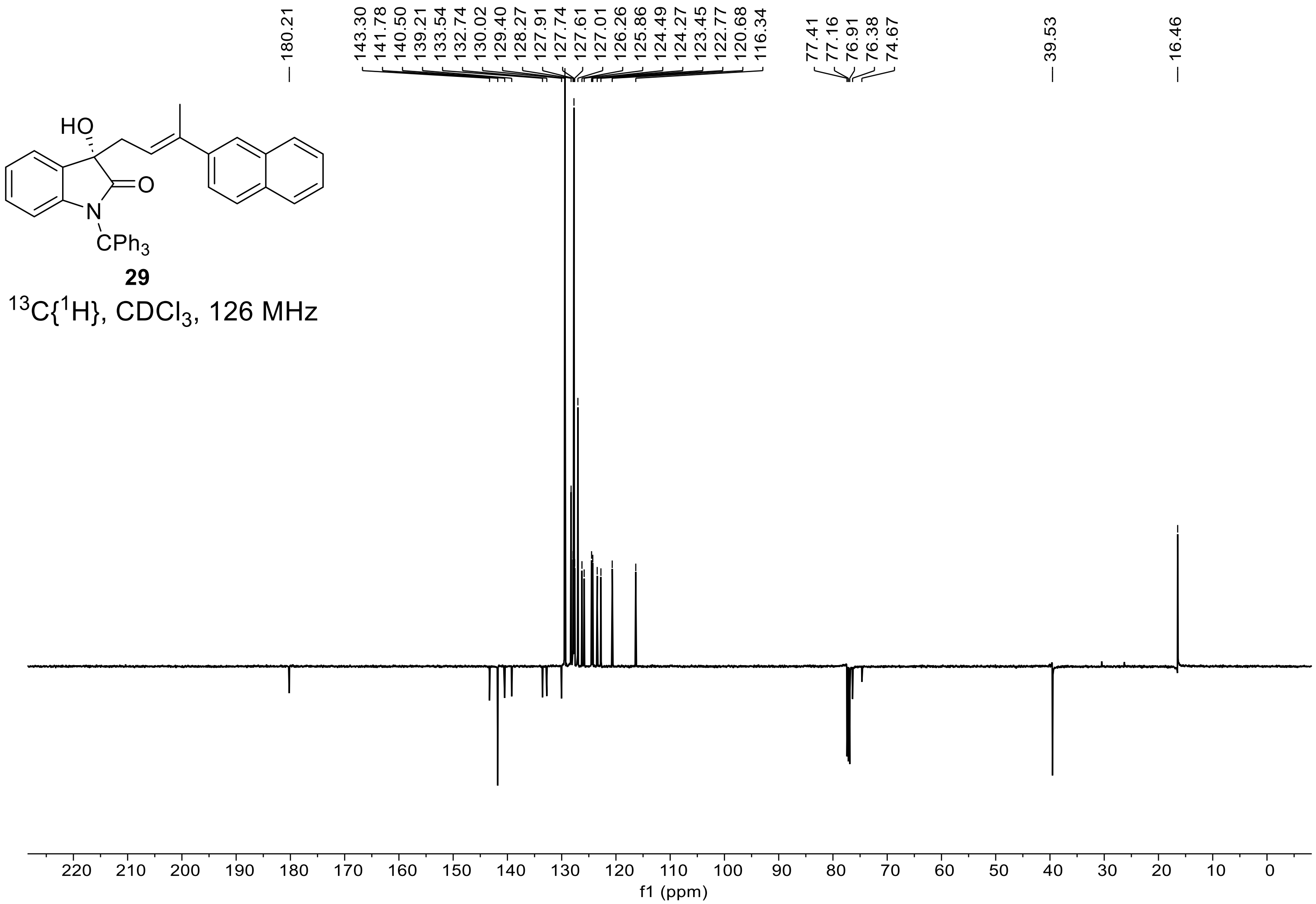

Fig. S350: $^{13}C\{^{1}H\}$ NMR spectrum for (*S*,*E*)-3-Hydroxy-3-[3-(naphthalen-2-yl)but-2-en-1-yl]-1-tritylindolin-2-one (**29**).

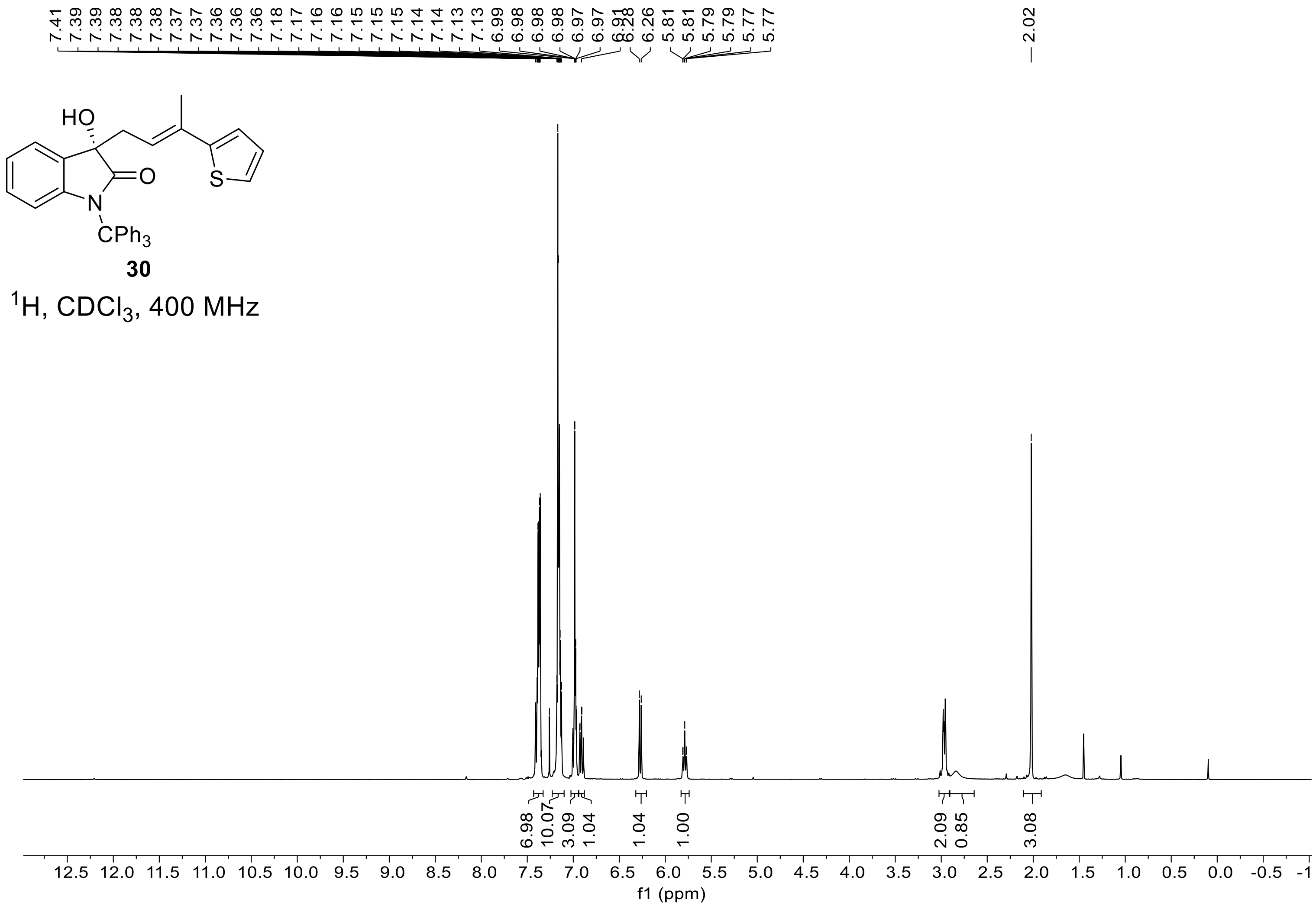

Fig. S351: ^{1}H NMR spectrum for (*S*,*E*)-3-Hydroxy-3-[3-(thiophen-2-yl)but-2-en-1-yl]-1-tritylindolin-2-one (**30**).

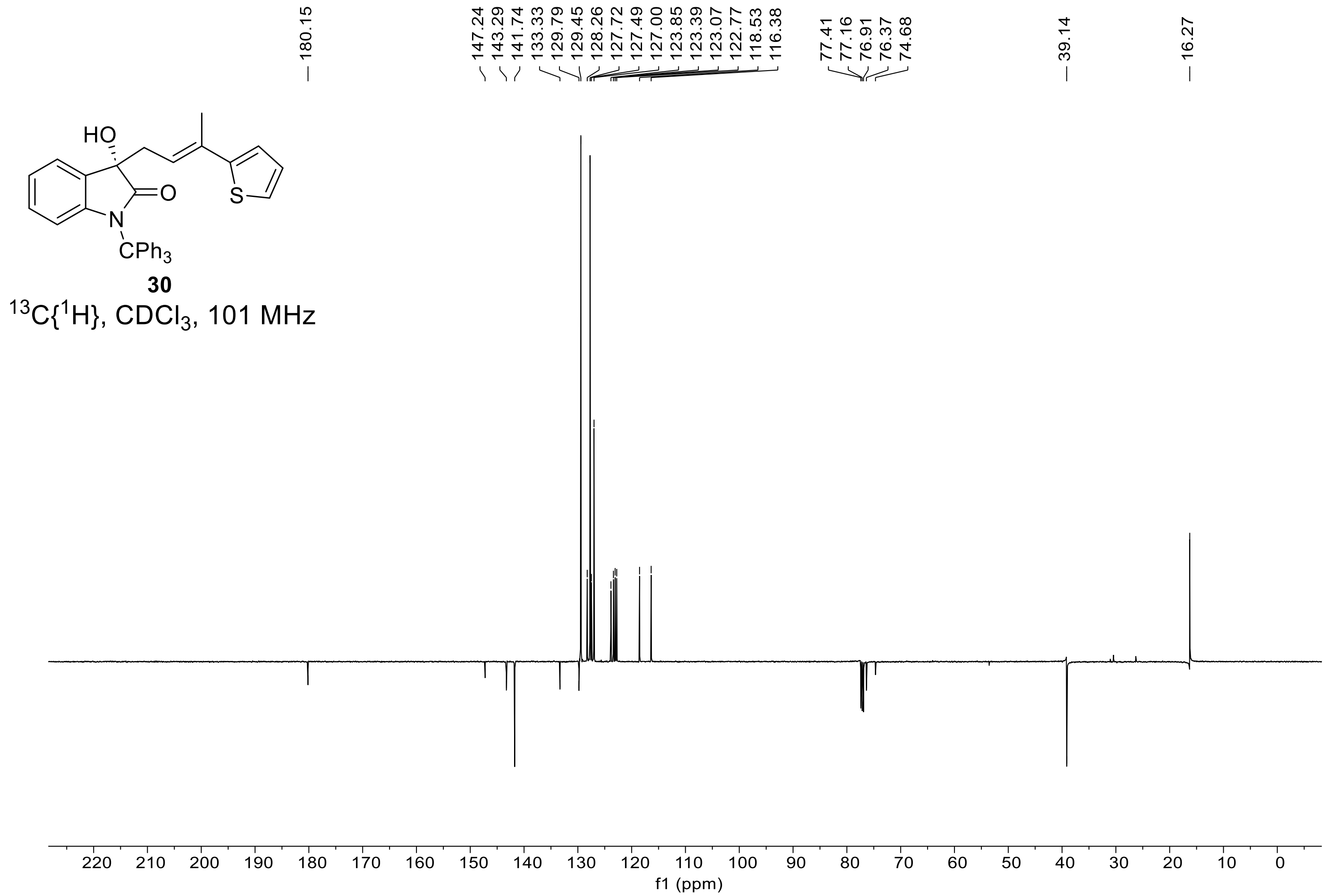

Fig. S352: $^{13}C\{^{1}H\}$ NMR spectrum for (*S*,*E*)-3-Hydroxy-3-[3-(thiophen-2-yl)but-2-en-1-yl]-1-tritylindolin-2-one (**30**).

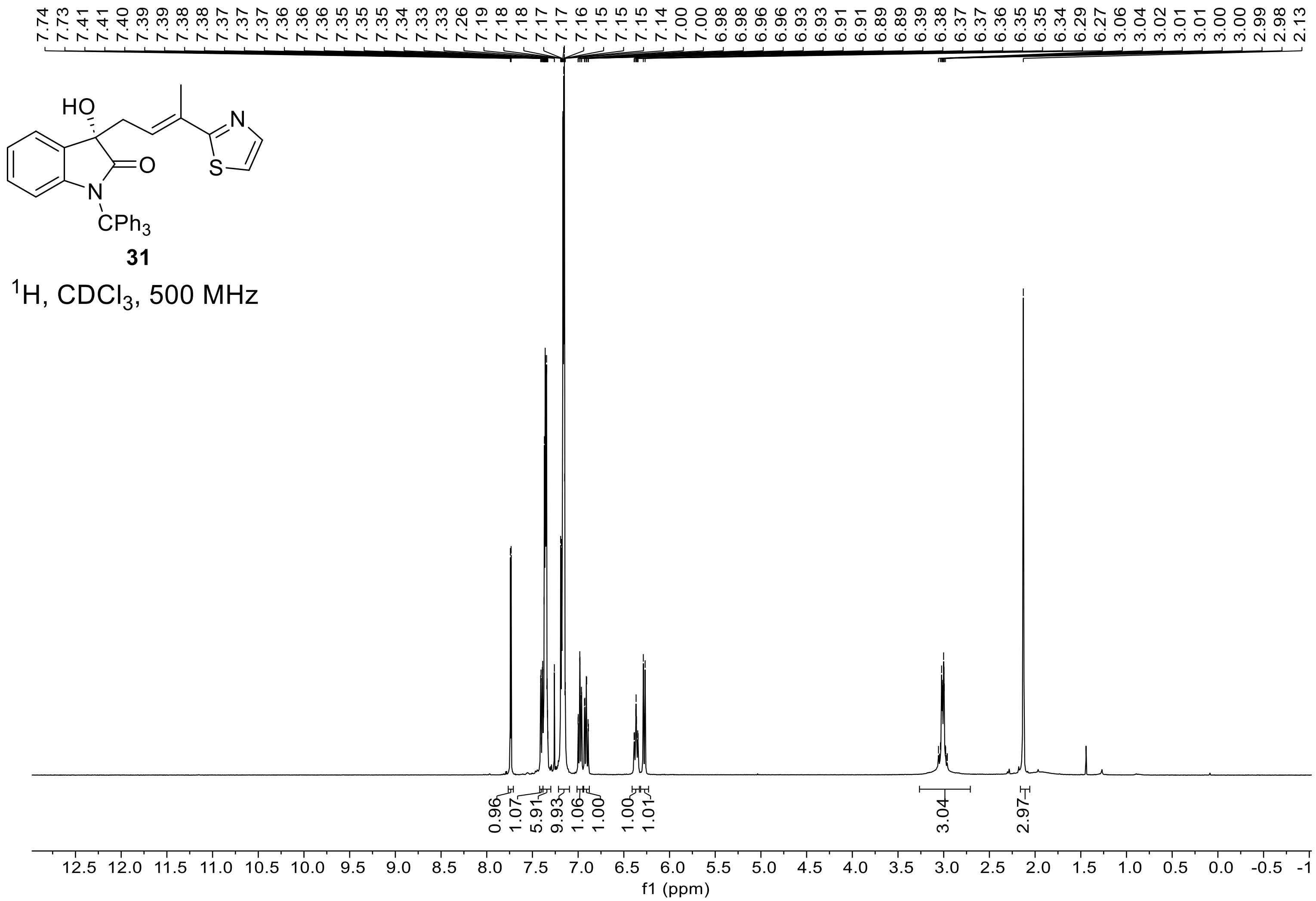

Fig. S353: ^{1}H NMR spectrum for (*S*,*E*)-3-Hydroxy-3-[3-(thiazol-2-yl)but-2-en-1-yl]-1-tritylindolin-2-one (**31**).

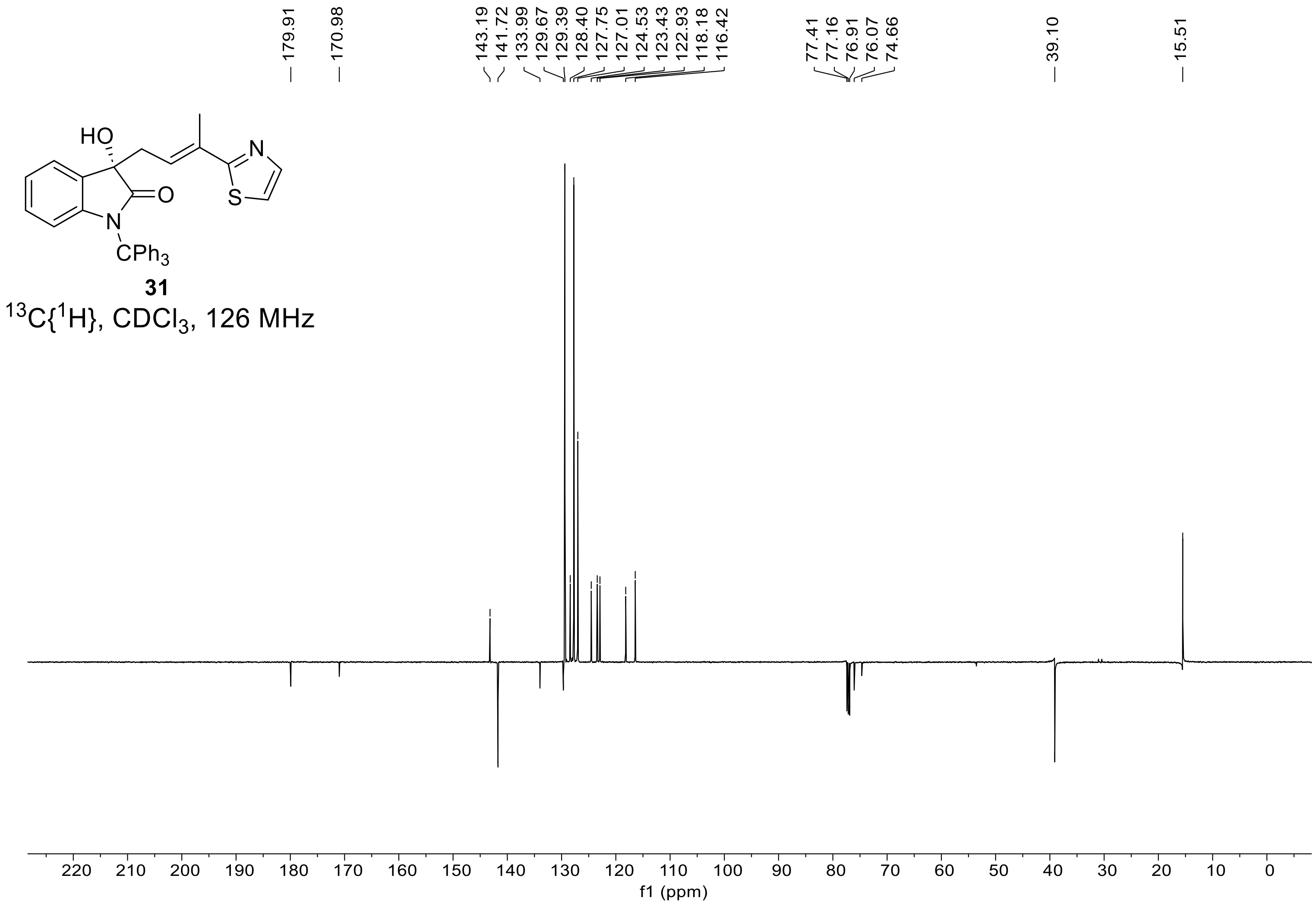

Fig. S354: $^{13}C\{^1H\}$ NMR spectrum for (*S*,*E*)-3-Hydroxy-3-[3-(thiazol-2-yl)but-2-en-1-yl]-1-tritylindolin-2-one (**31**).

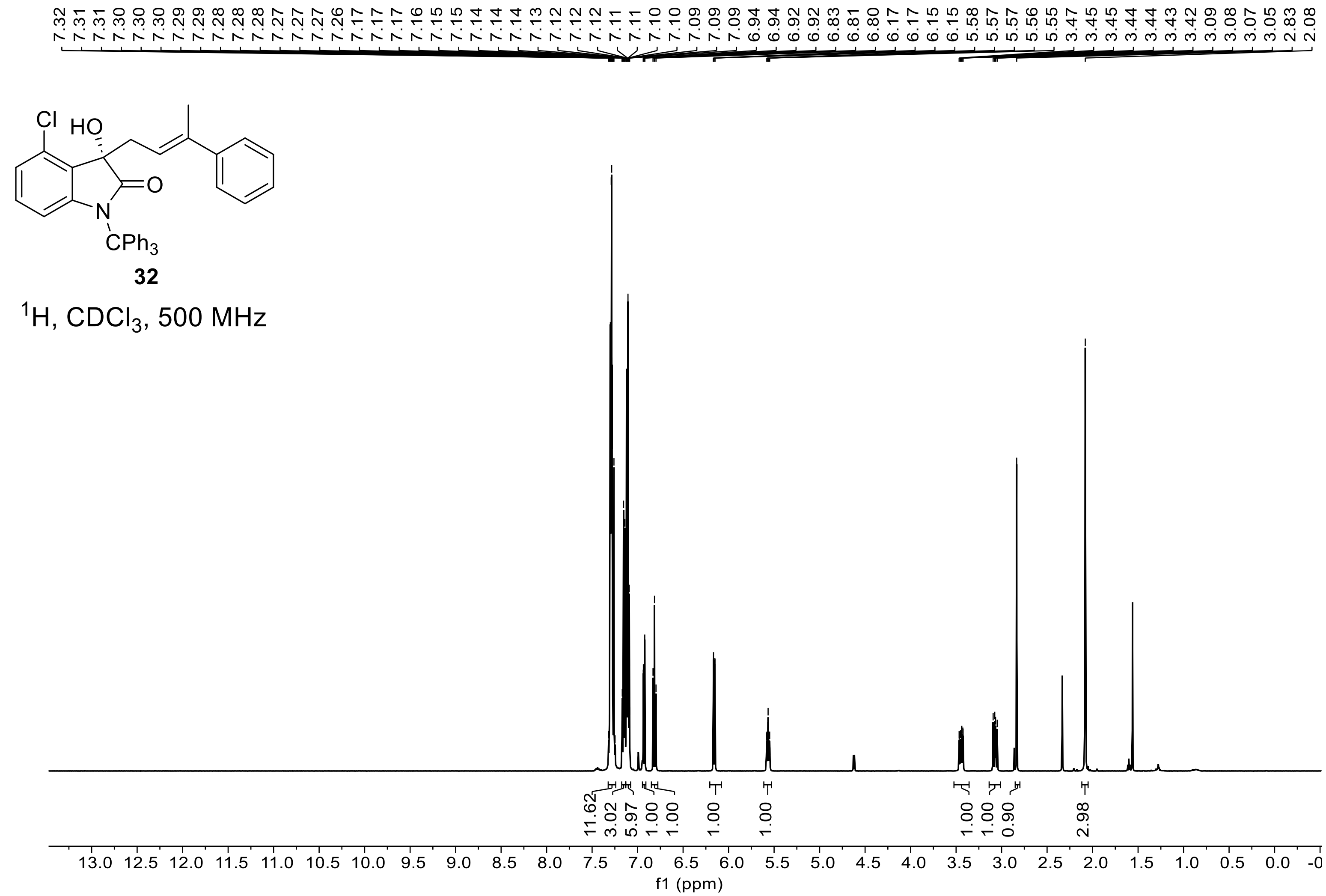

Fig. S355: ^{1}H NMR spectrum for (*S*,*E*)-4-Chloro-3-hydroxy-3-(3-phenylbut-2-en-1-yl)-1-tritylindolin-2-one (**32**).

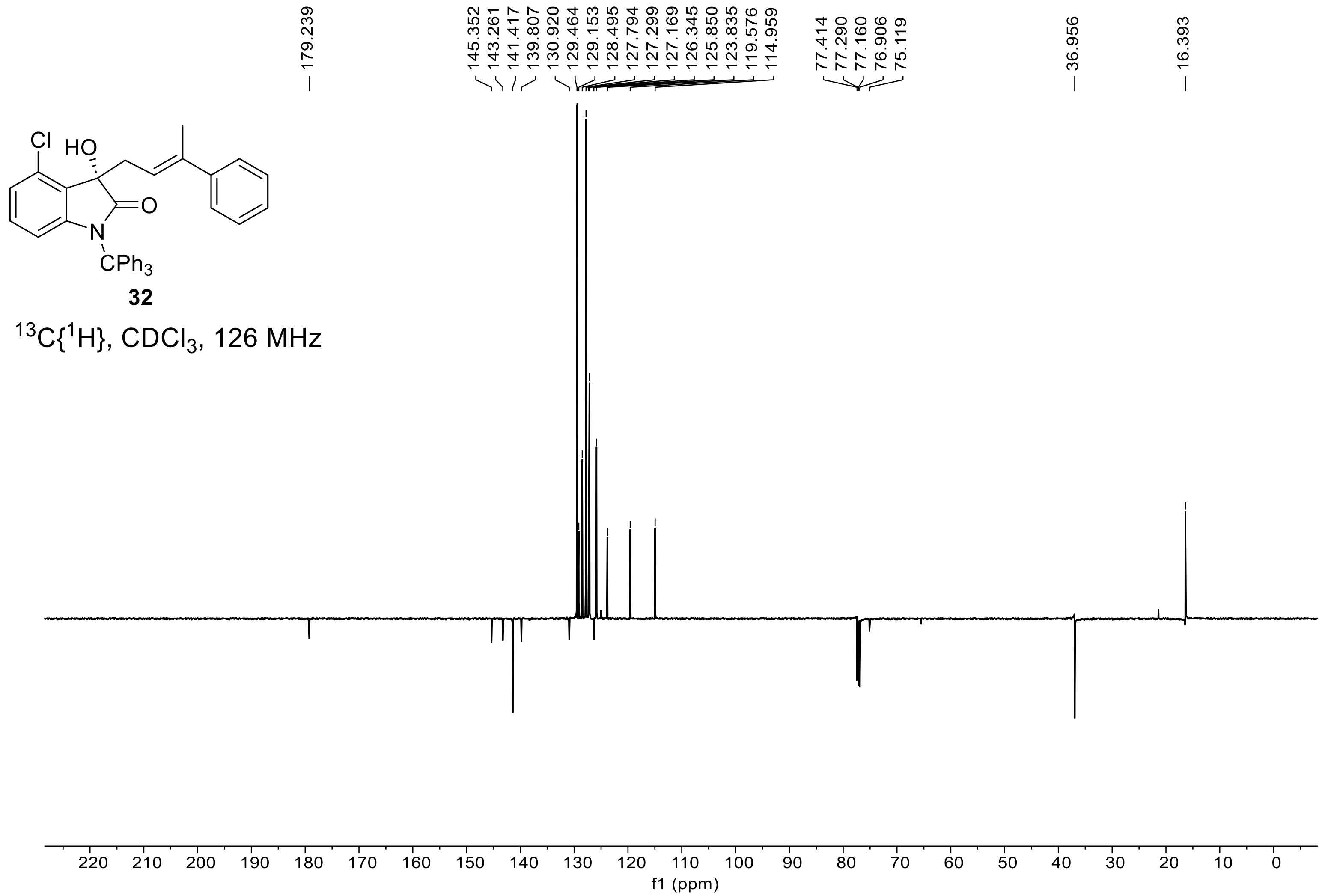

Fig. S356: $^{13}C\{^1H\}$ NMR spectrum for (*S*,*E*)-4-Chloro-3-hydroxy-3-(3-phenylbut-2-en-1-yl)-1-tritylindolin-2-one (**32**).

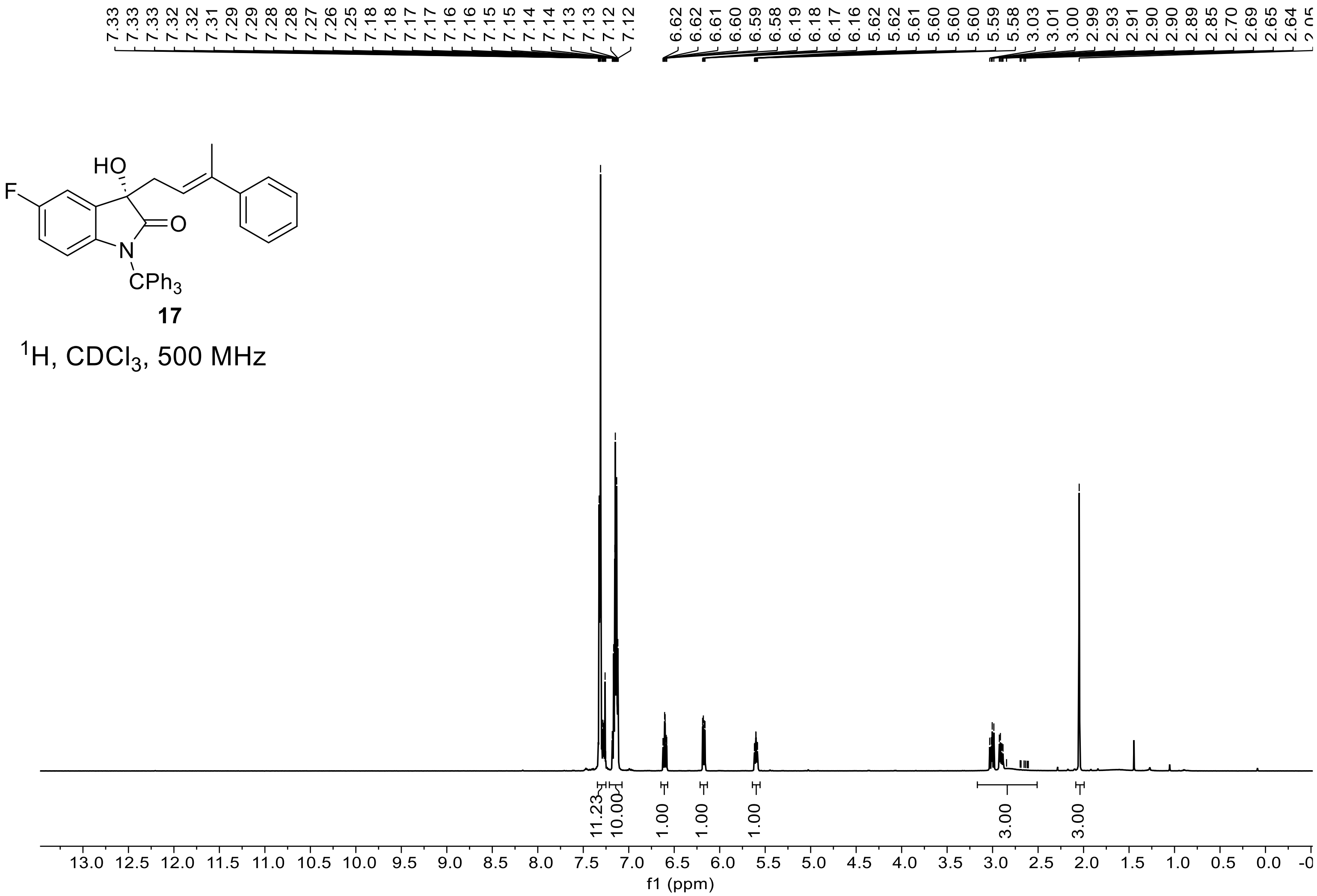

Fig. S357: $^{13}C\{^{1}H\}$ NMR spectrum for (*S*,*E*)-5-Fluoro-3-hydroxy-3-(3-phenylbut-2-en-1-yl)-1-tritylindolin-2-one (**17**).

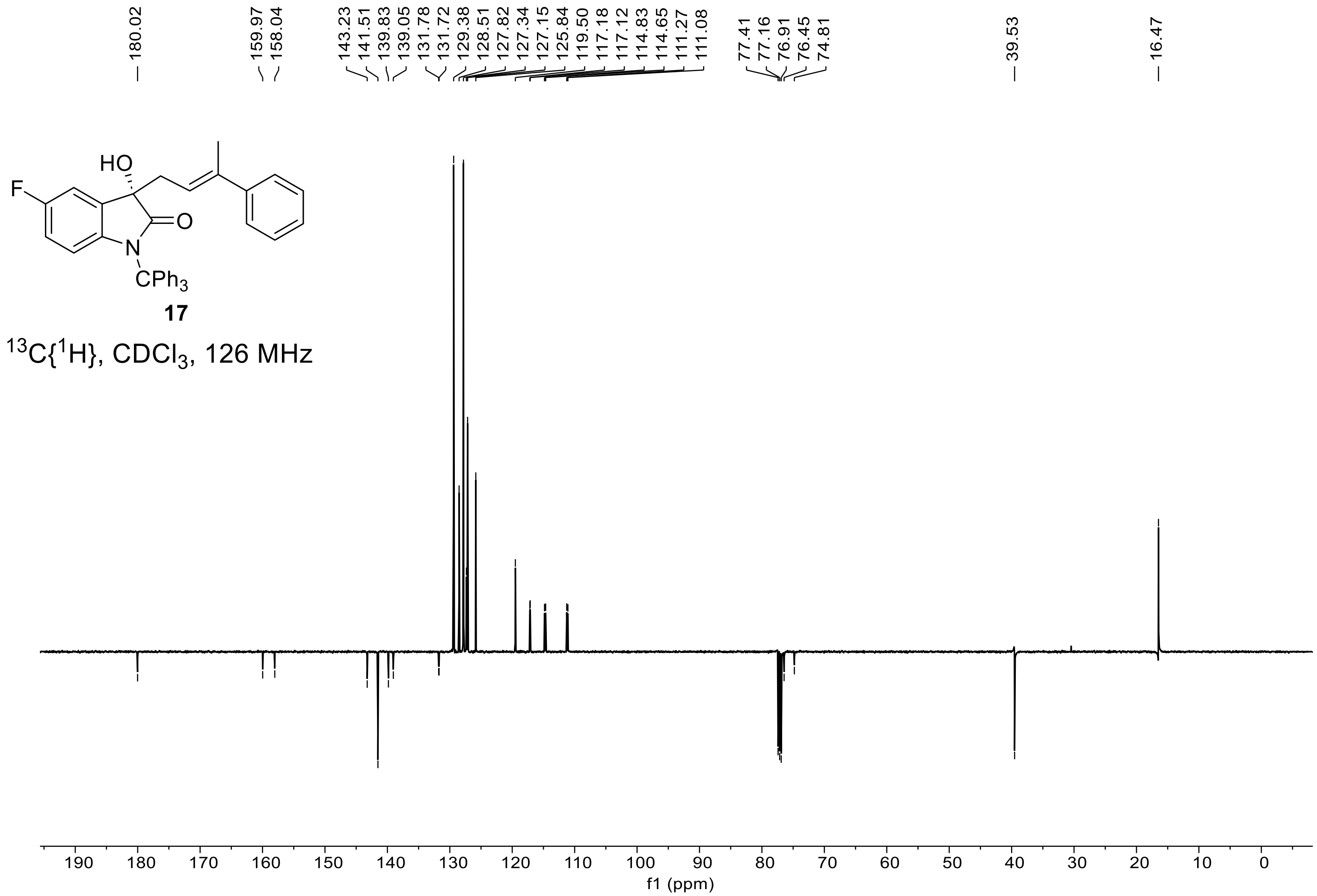

Fig. S358: $^{13}C\{^{1}H\}$ NMR spectrum for (*S*,*E*)-5-Fluoro-3-hydroxy-3-(3-phenylbut-2-en-1-yl)-1-tritylindolin-2-one (**17**).

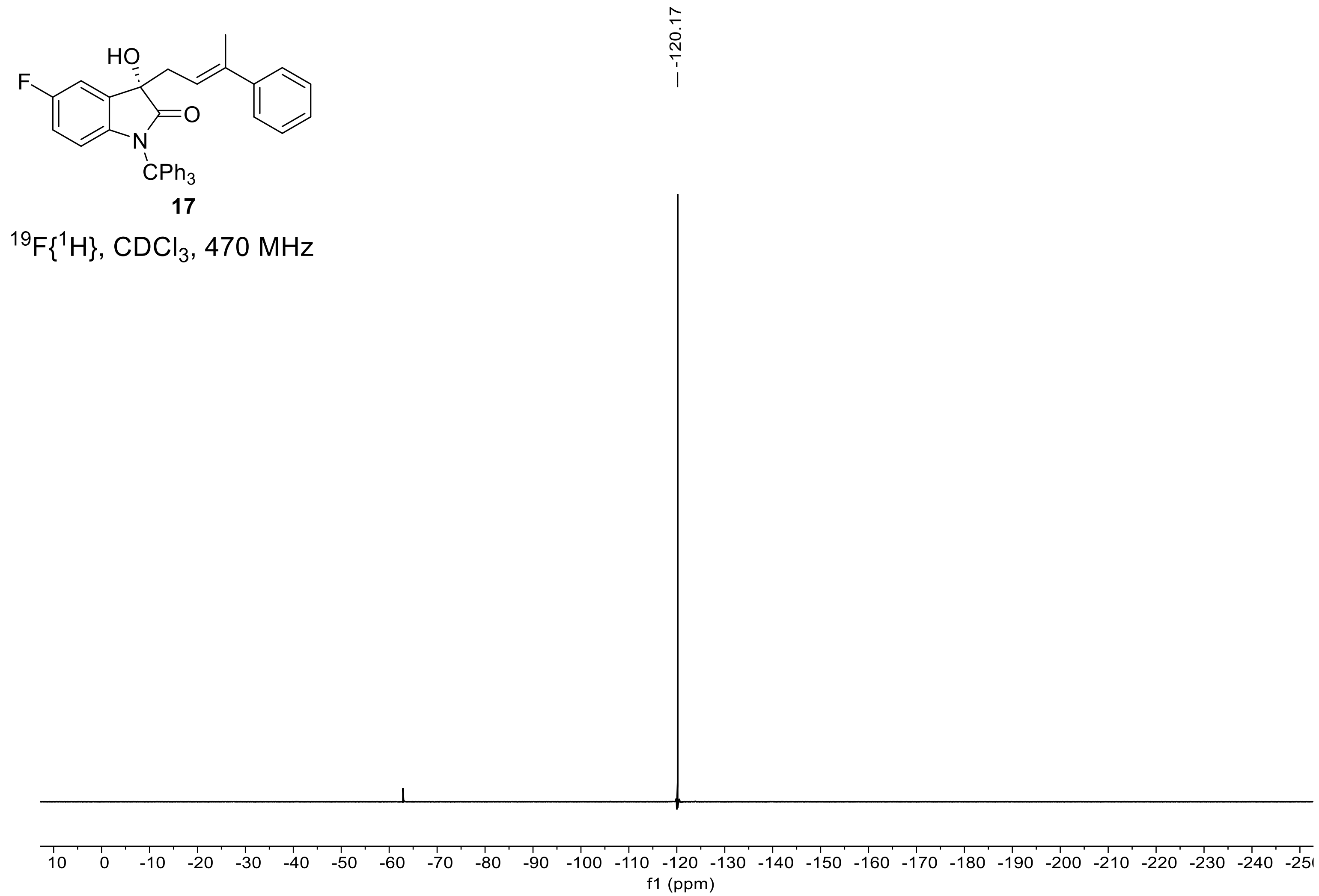

Fig. S359: $^{19}F\{^{1}H\}$ NMR spectrum for (*S*,*E*)-5-Fluoro-3-hydroxy-3-(3-phenylbut-2-en-1-yl)-1-tritylindolin-2-one (**17**).

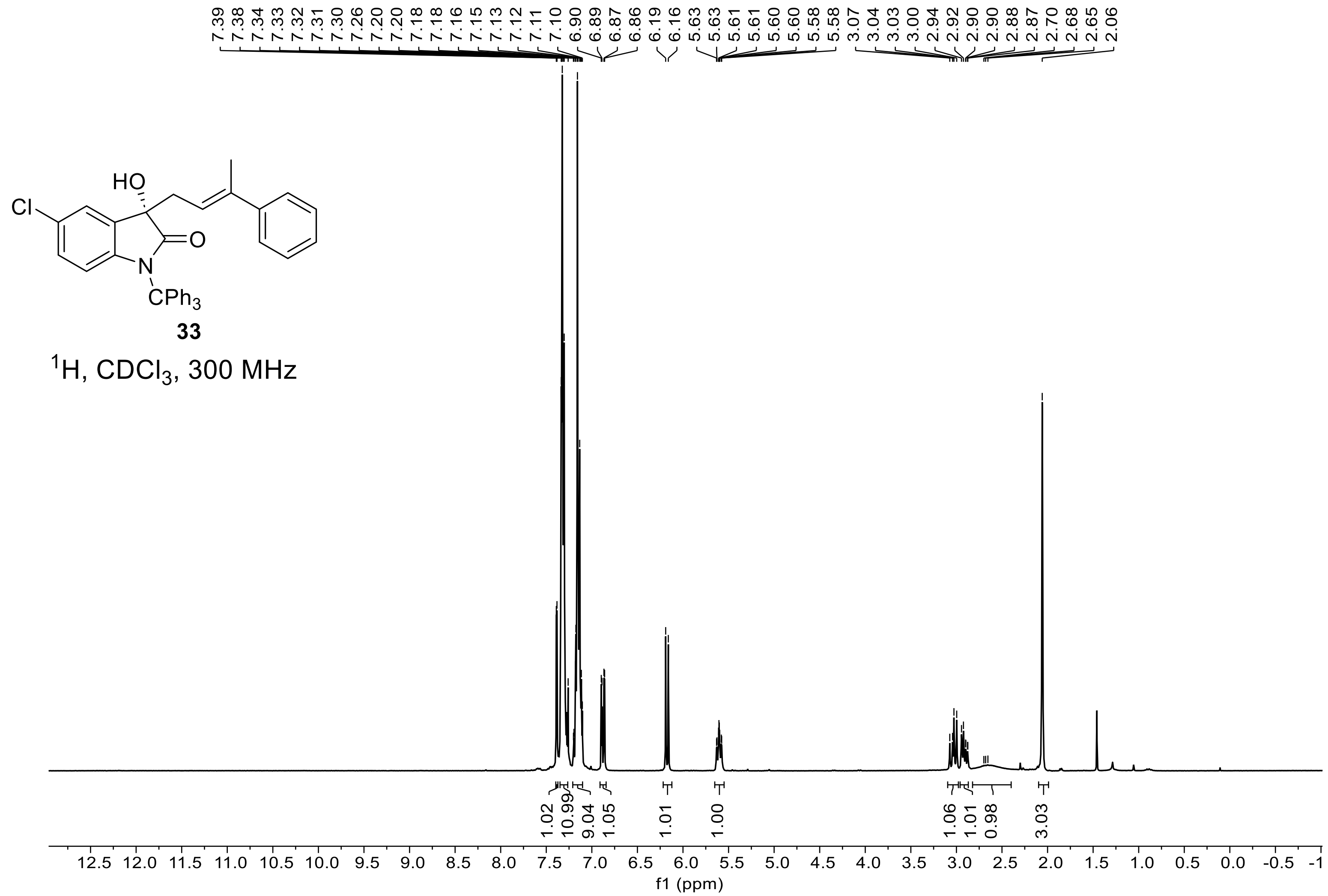

Fig. S360: ^{1}H NMR spectrum for (*S*,*E*)-5-Chloro-3-hydroxy-3-(3-phenylbut-2-en-1-yl)-1-tritylindolin-2-one (**33**).

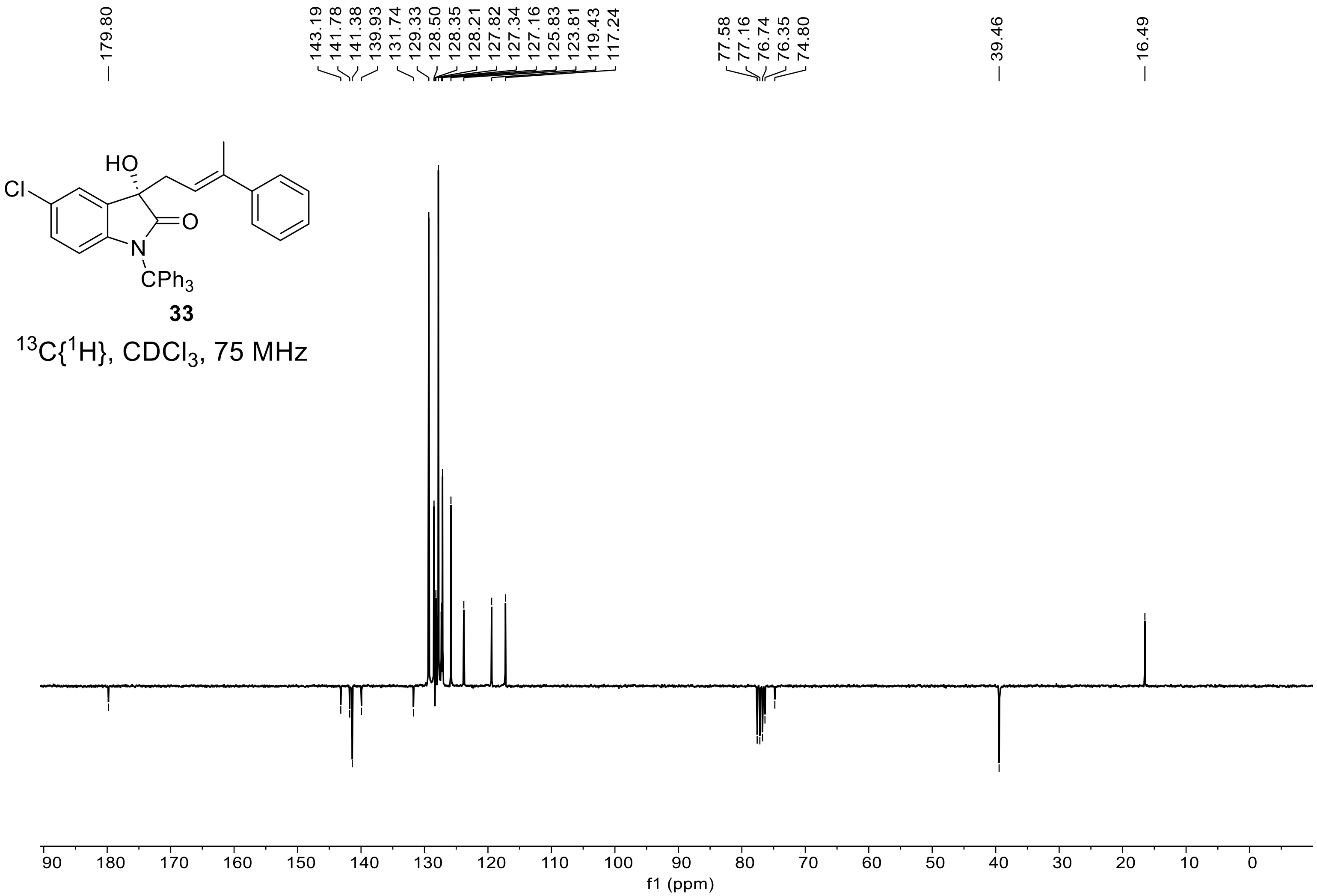

Fig. S361: $^{13}C\{^{1}H\}$ NMR spectrum for (*S*,*E*)-5-Chloro-3-hydroxy-3-(3-phenylbut-2-en-1-yl)-1-tritylindolin-2-one (**33**).

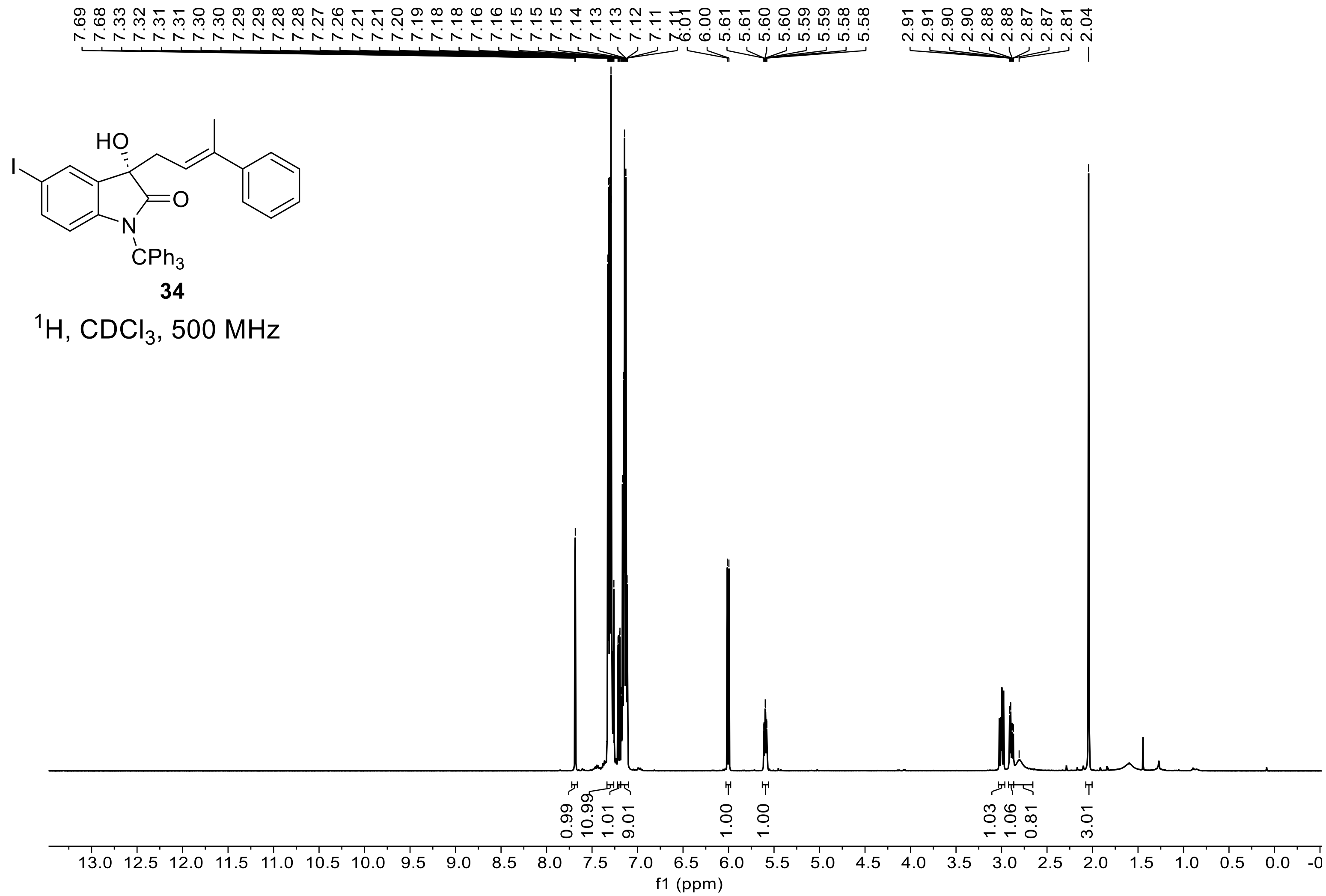

Fig. S362: ^{1}H NMR spectrum for (*S*,*E*)-3-Hydroxy-5-iodo-3-(3-phenylbut-2-en-1-yl)-1-tritylindolin-2-one (**34**).

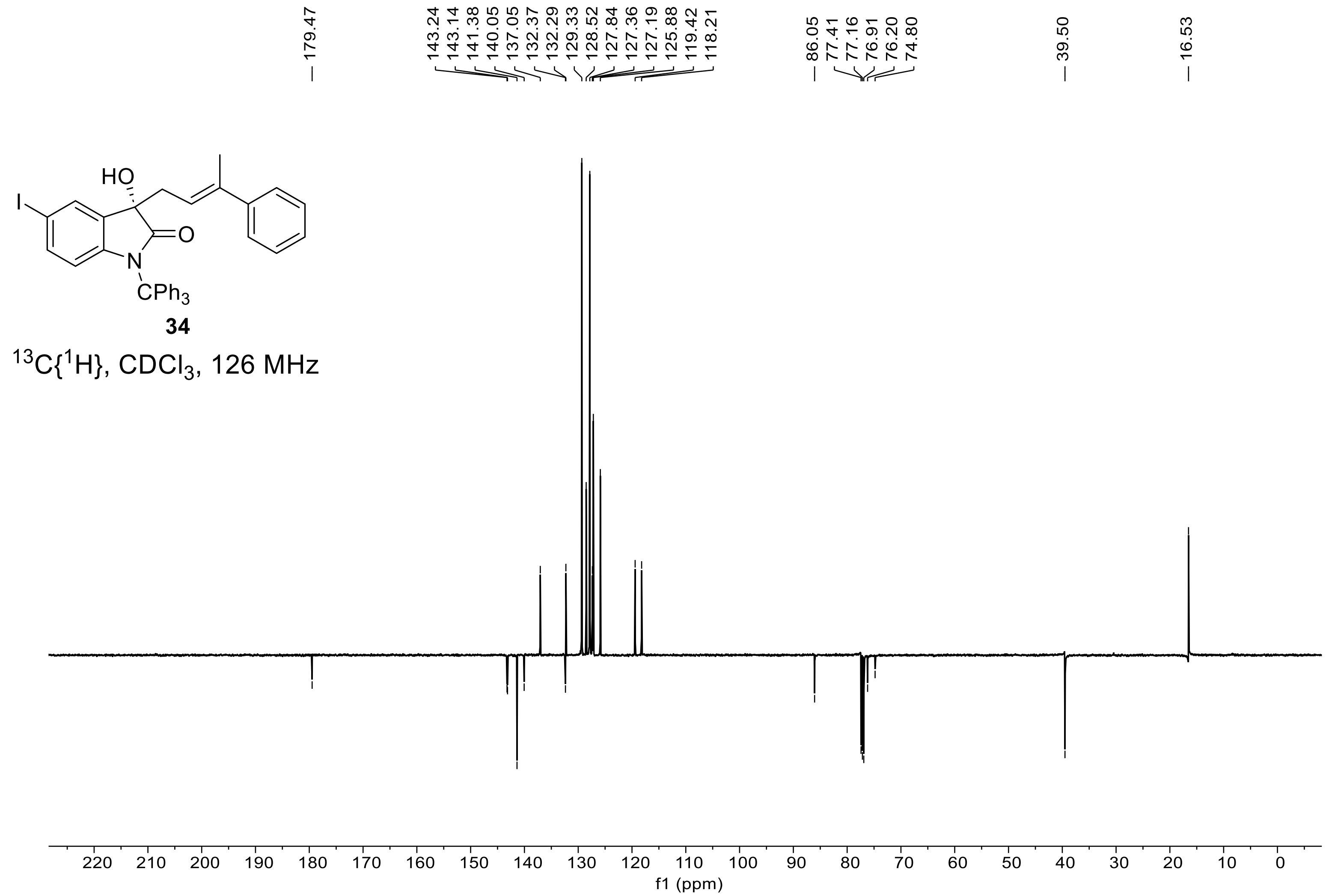

Fig. S363: $^{13}C\{^1H\}$ NMR spectrum for (*S*,*E*)-3-Hydroxy-5-iodo-3-(3-phenylbut-2-en-1-yl)-1-tritylindolin-2-one (**34**).

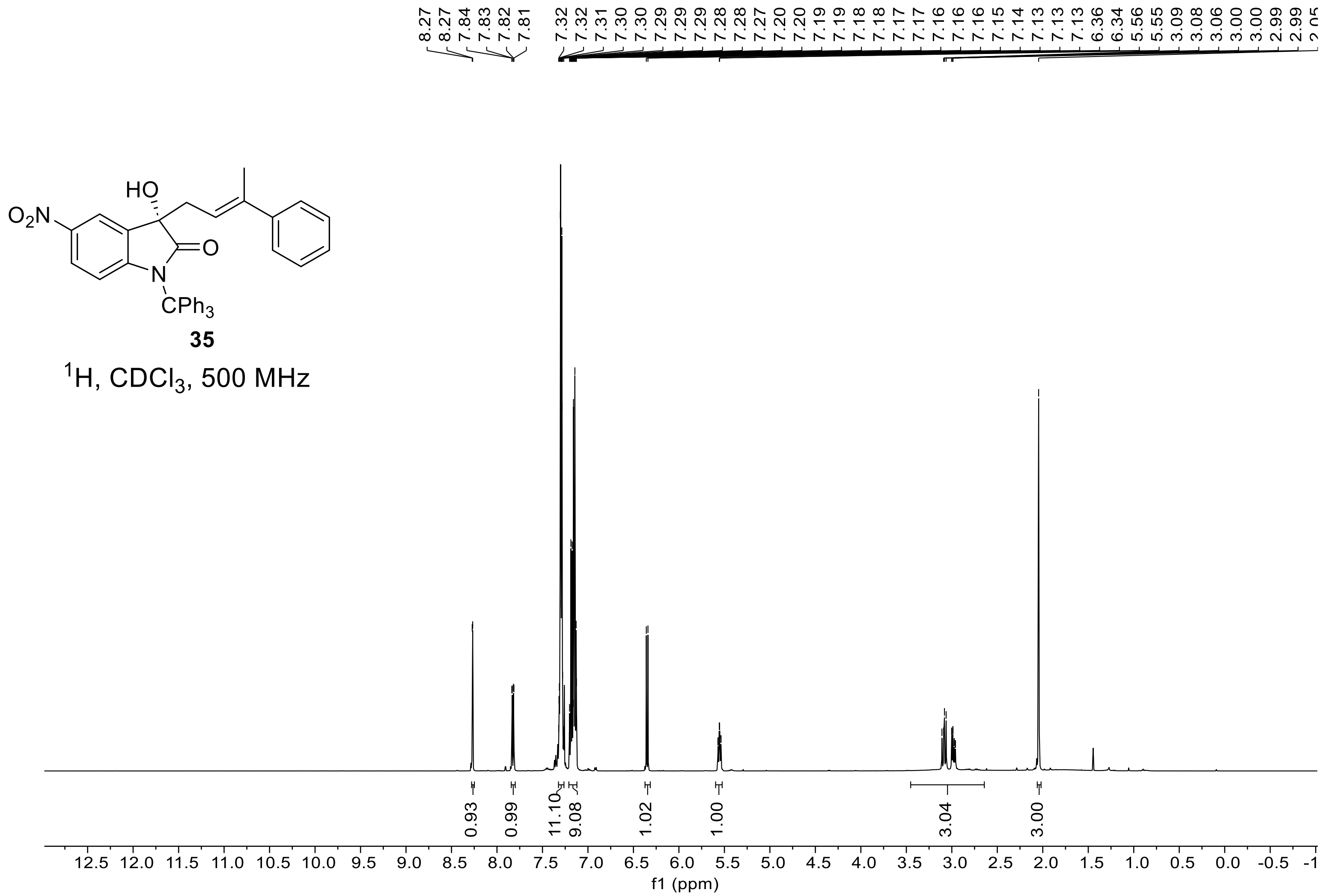

Fig. S364: ^{1}H NMR spectrum for (*S*,*E*)-3-Hydroxy-5-nitro-3-(3-phenylbut-2-en-1-yl)-1-tritylindolin-2-one (**35**).

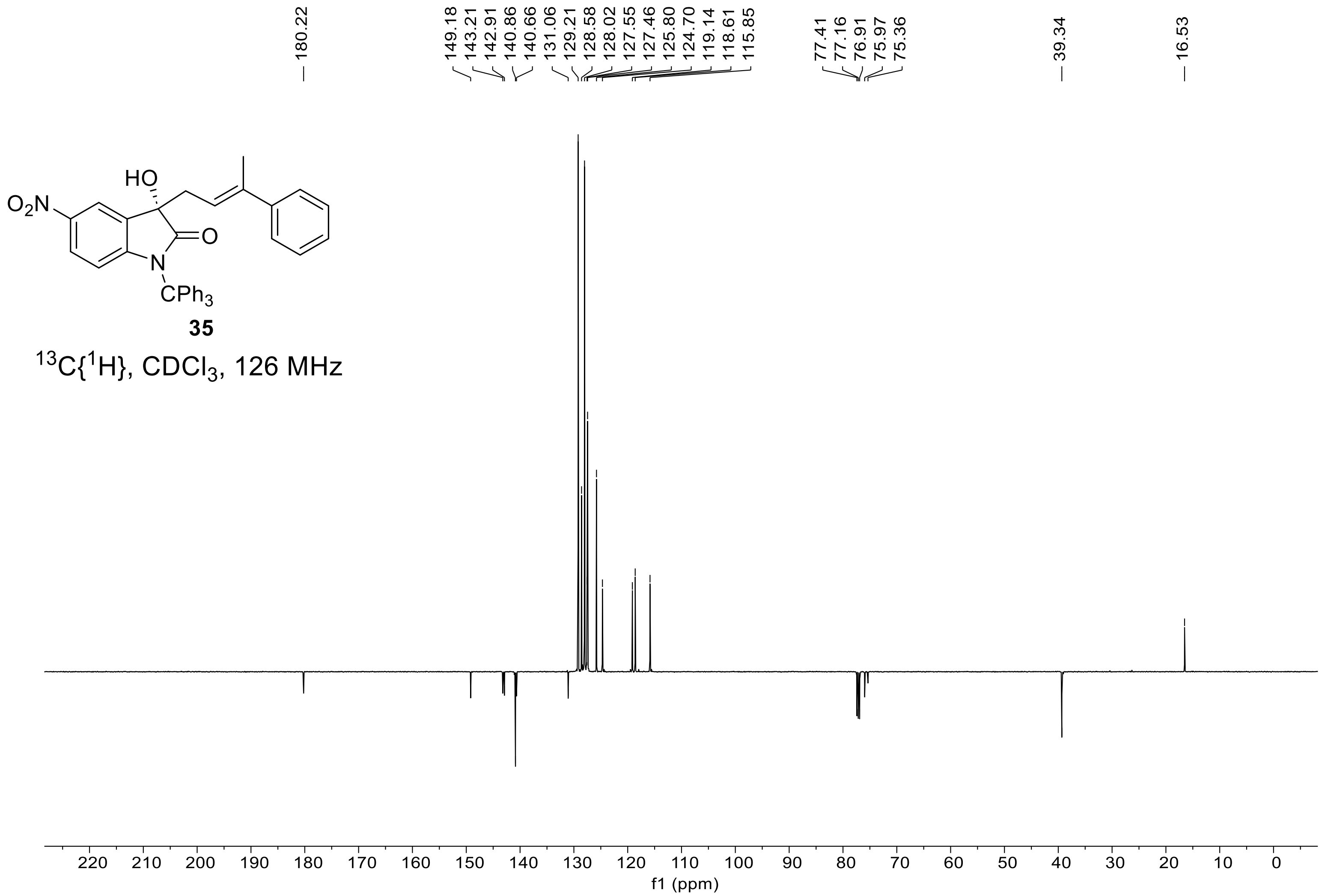

Fig. S365: $^{13}C\{^{1}H\}$ NMR spectrum for (*S*,*E*)-3-Hydroxy-5-nitro-3-(3-phenylbut-2-en-1-yl)-1-tritylindolin-2-one (**35**).

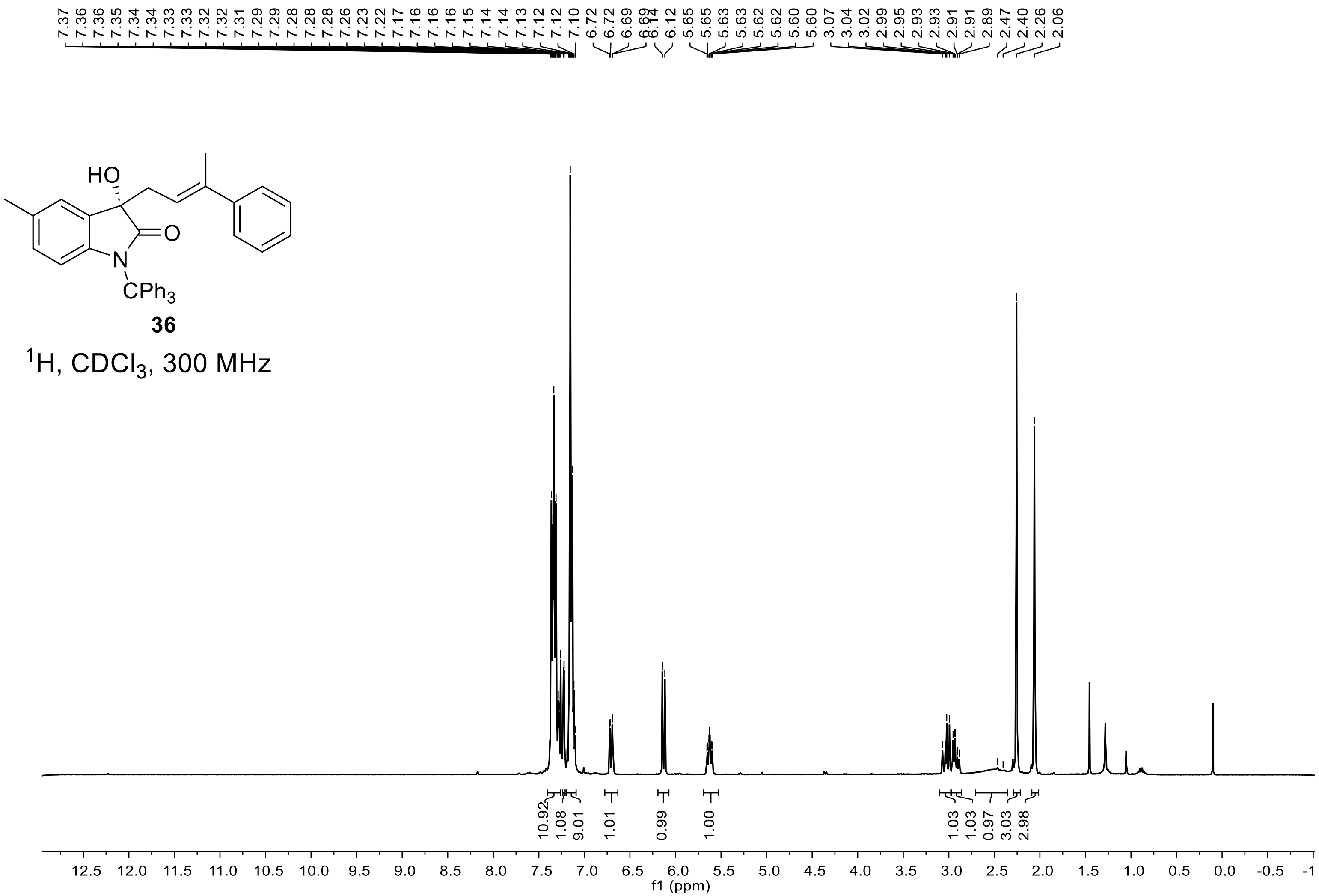

Fig. S366: ^{1}H NMR spectrum for (*S*,*E*)-3-Hydroxy-5-methyl-3-(3-phenylbut-2-en-1-yl)-1-tritylindolin-2-one (**36**).

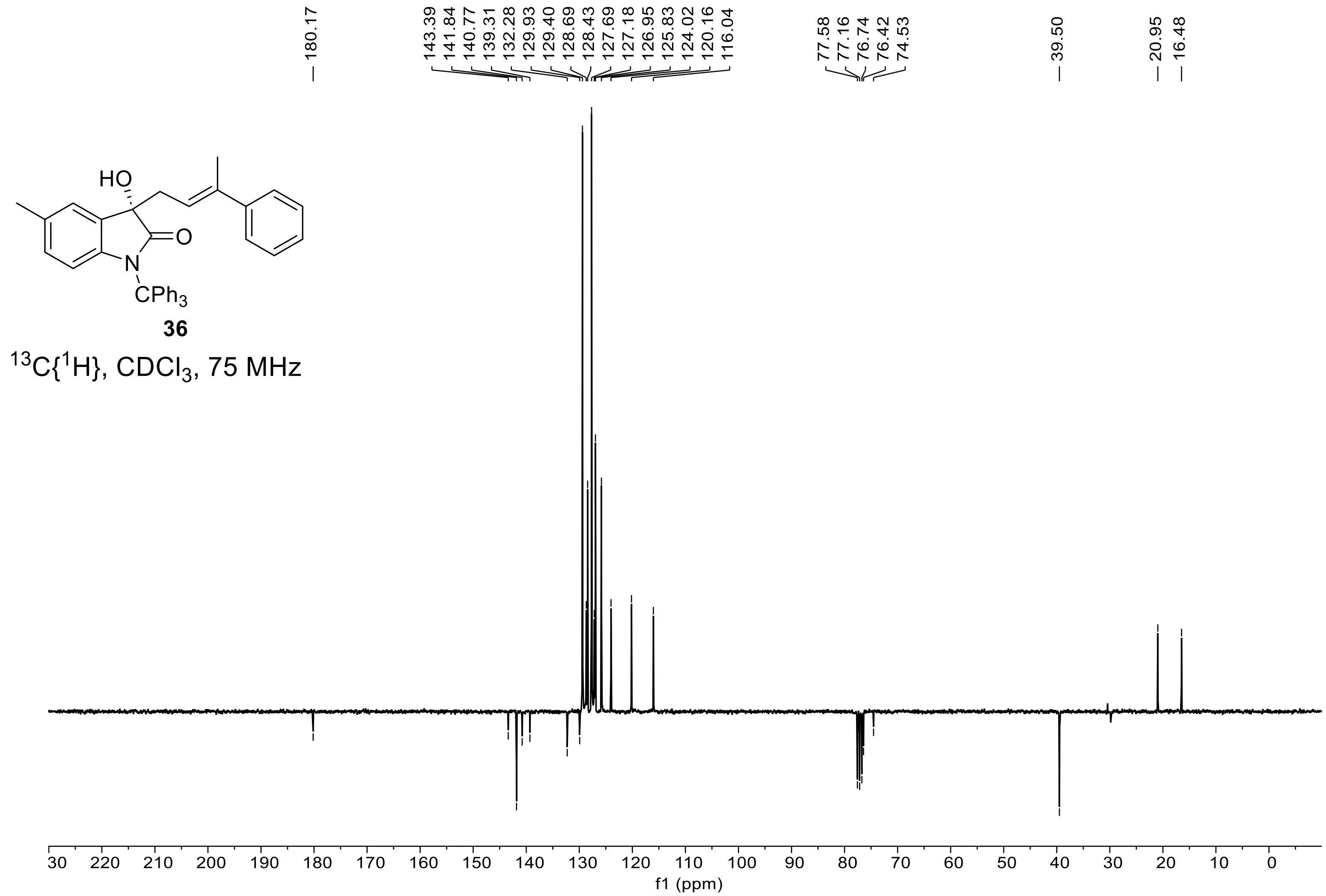

Fig. S367: $^{13}C\{^1H\}$ NMR spectrum for (*S*,*E*)-3-Hydroxy-5-methyl-3-(3-phenylbut-2-en-1-yl)-1-tritylindolin-2-one (**36**).

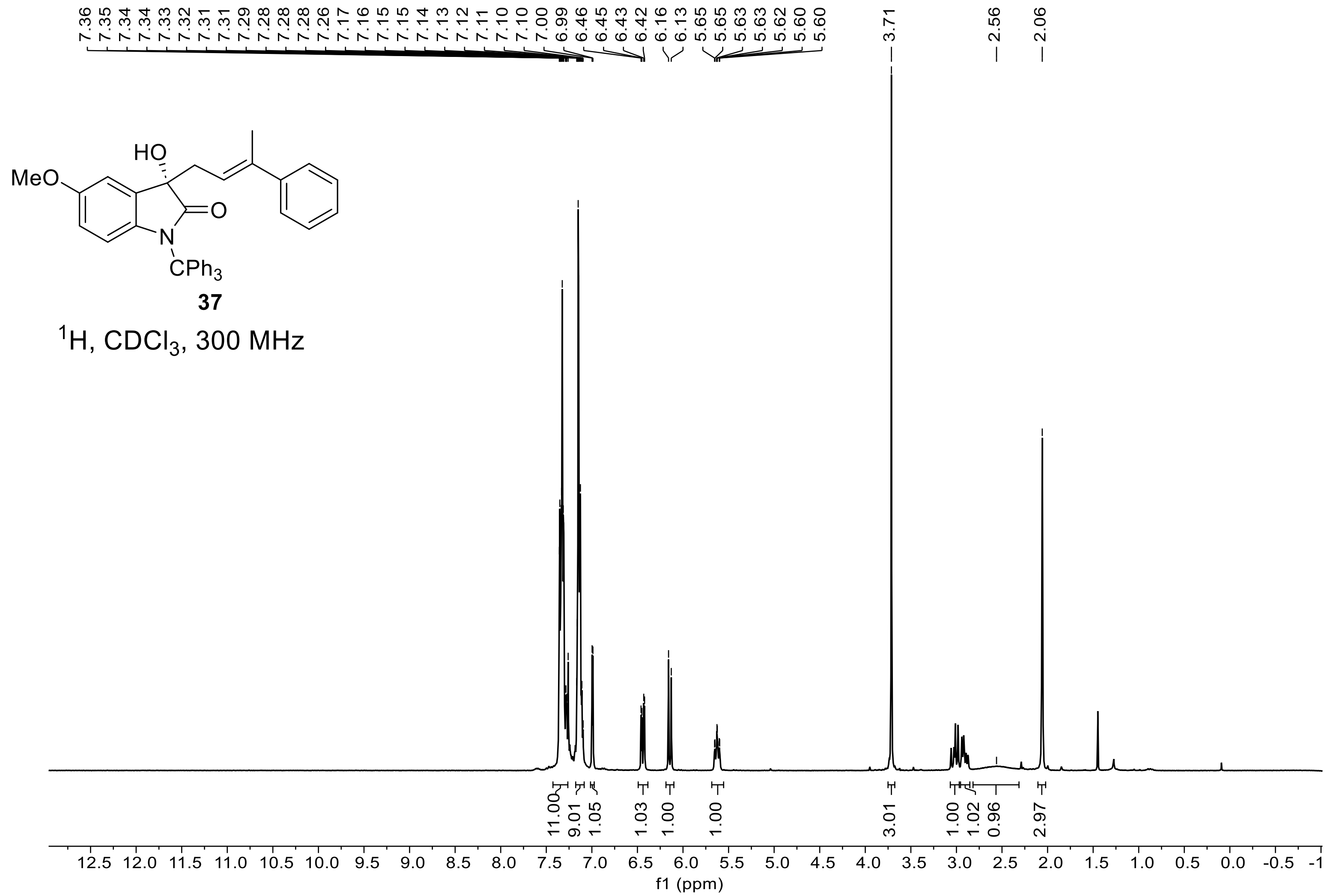

Fig. S368: ^{1}H NMR spectrum for (*S*,*E*)-3-Hydroxy-5-methoxy-3-(3-phenylbut-2-en-1-yl)-1-tritylindolin-2-one (**37**).

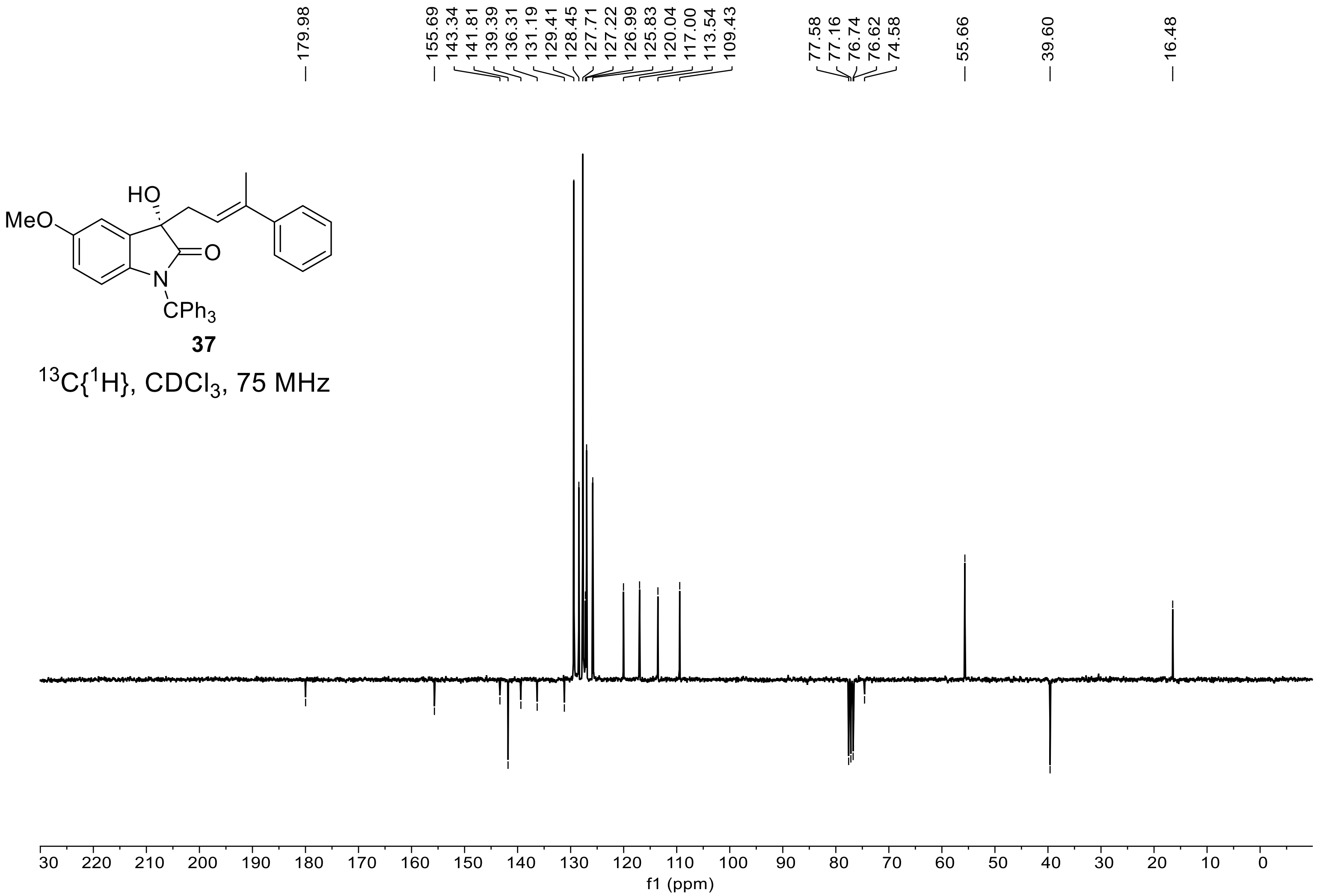

Fig. S369: $^{13}C\{^{1}H\}$ NMR spectrum for (*S*,*E*)-3-Hydroxy-5-methoxy-3-(3-phenylbut-2-en-1-yl)-1-tritylindolin-2-one (**37**).

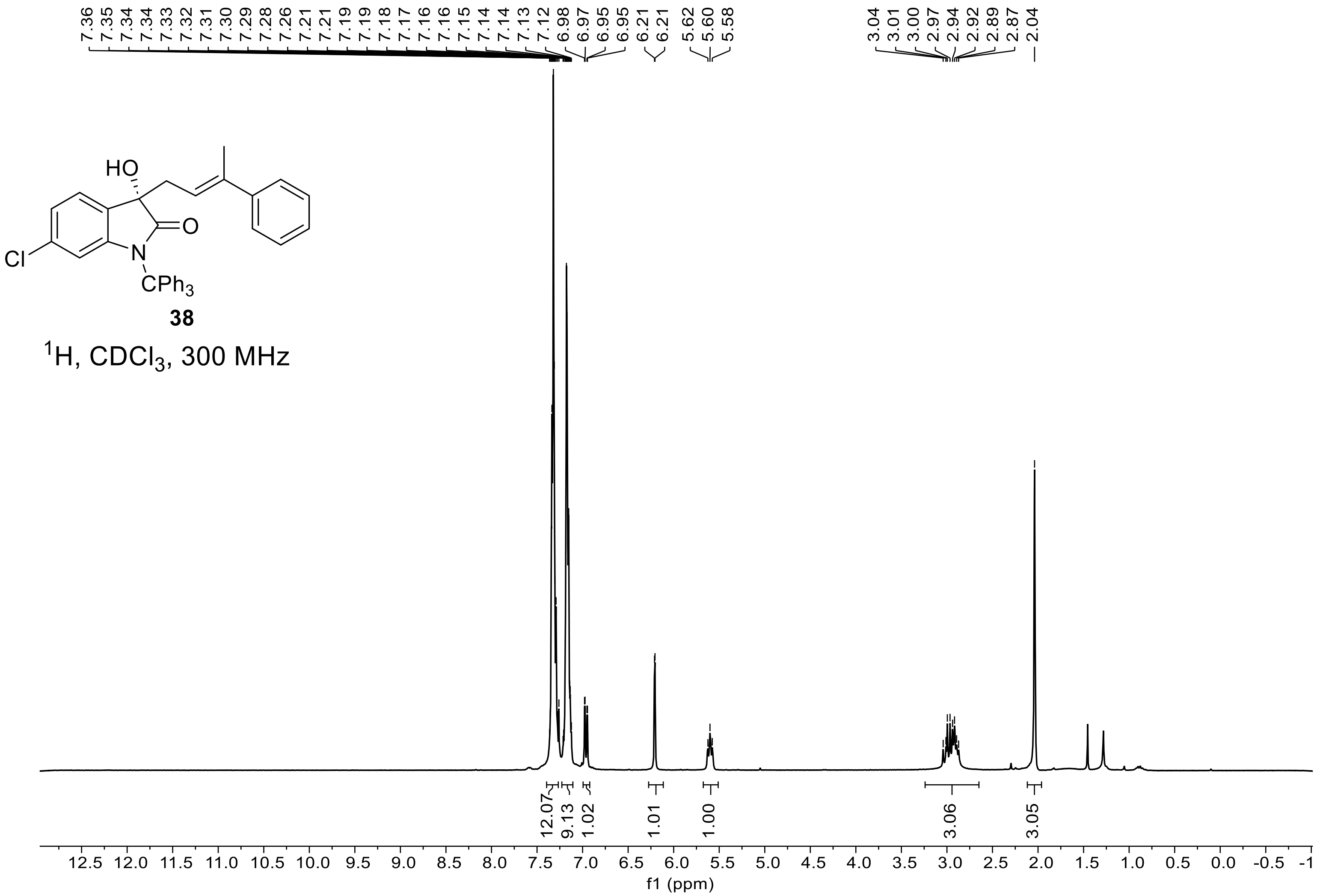

Fig. S370: ^{1}H NMR spectrum for (*S*,*E*)-6-Chloro-3-hydroxy-3-(3-phenylbut-2-en-1-yl)-1-tritylindolin-2-one (**38**).

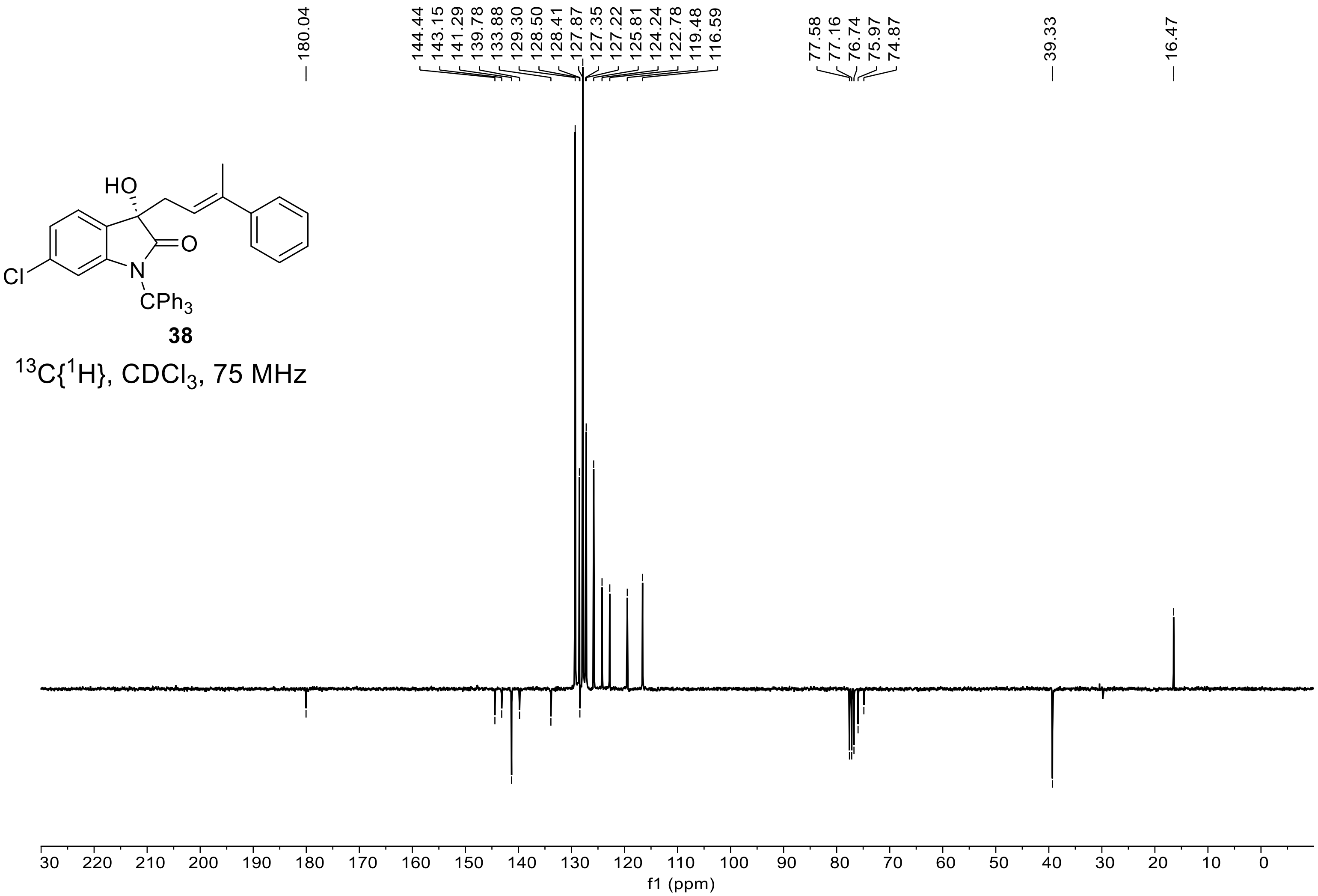

Fig. S371: $^{13}C\{^{1}H\}$ NMR spectrum for (*S*,*E*)-6-Chloro-3-hydroxy-3-(3-phenylbut-2-en-1-yl)-1-tritylindolin-2-one (**38**).

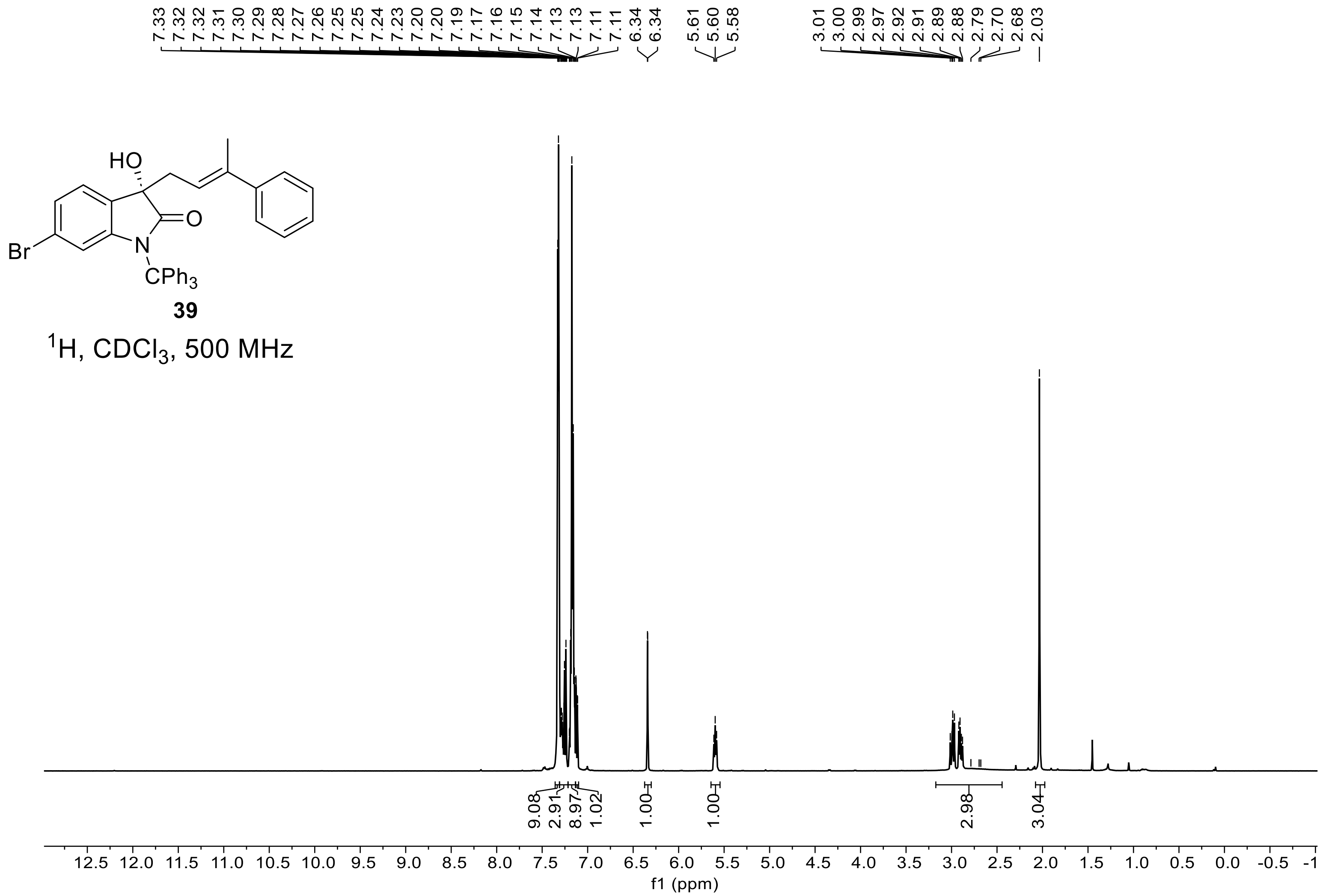

Fig. S372: ^{1}H NMR spectrum for (*S*,*E*)-6-Bromo-3-hydroxy-3-(3-phenylbut-2-en-1-yl)-1-tritylindolin-2-one (**39**).

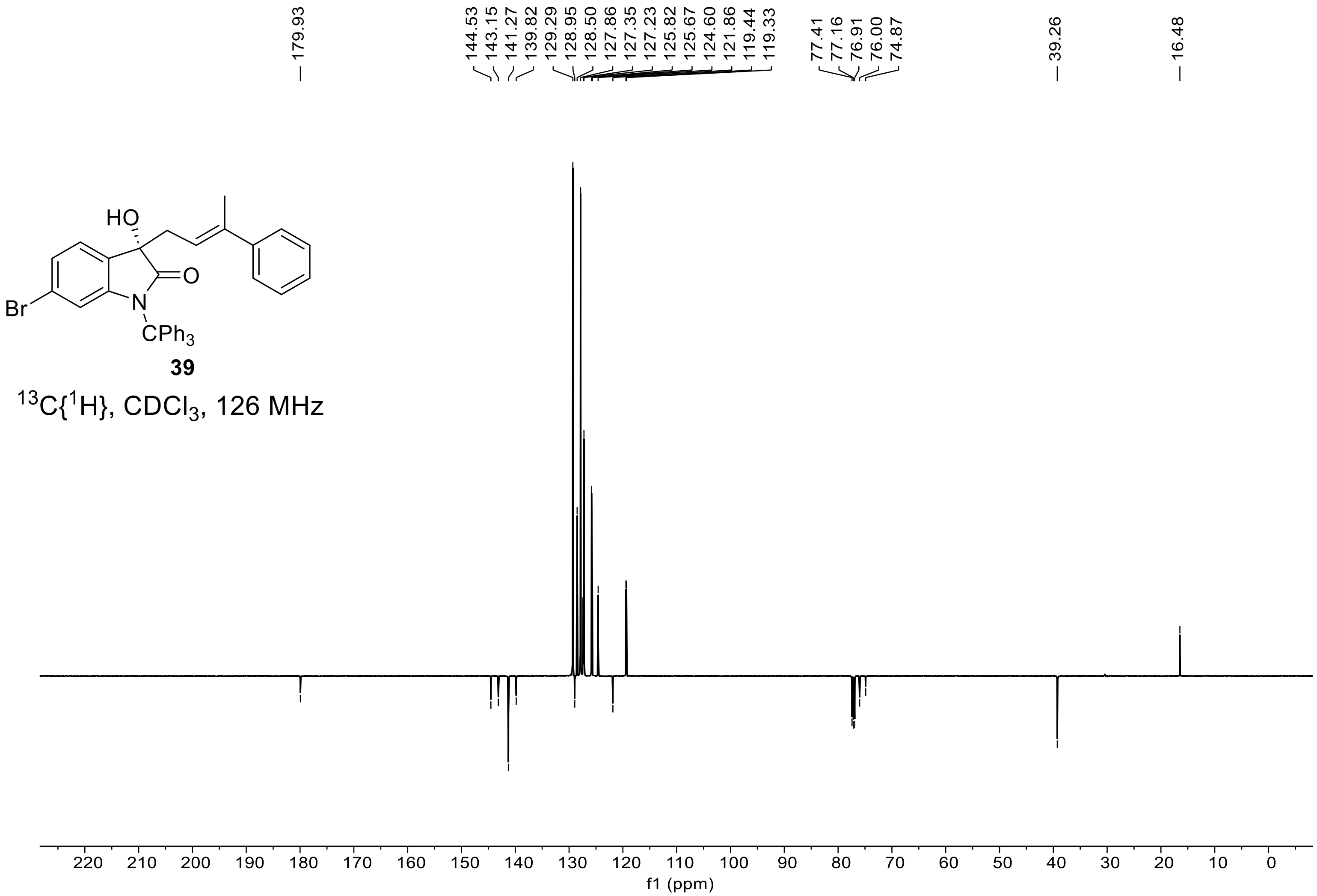

Fig. S373: $^{13}C\{^{1}H\}$ NMR spectrum for (*S*,*E*)-6-Bromo-3-hydroxy-3-(3-phenylbut-2-en-1-yl)-1-tritylindolin-2-one (**39**).

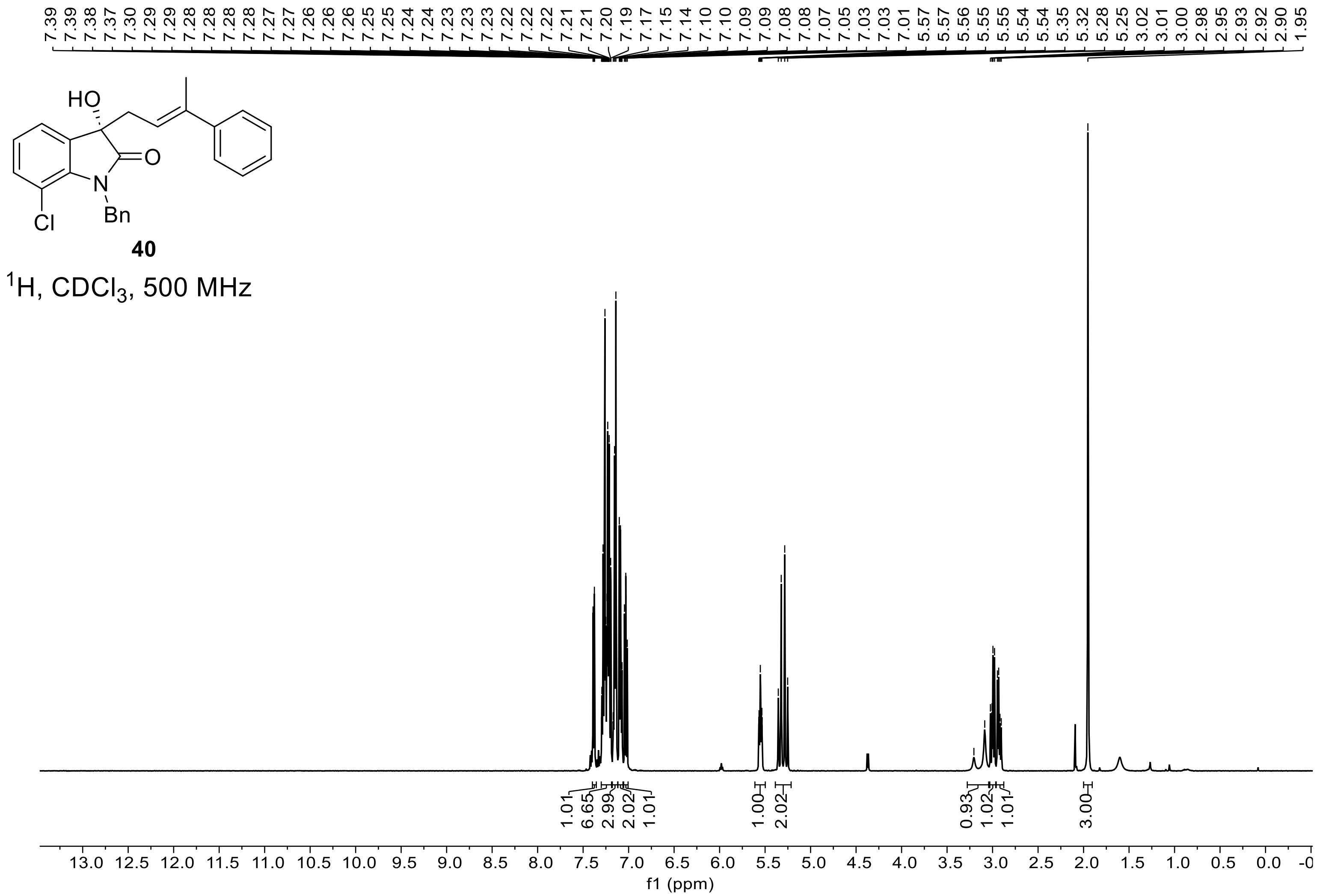

Fig. S374: ^{1}H NMR spectrum for (*S*,*E*)-1-Benzyl-7-chloro-3-hydroxy-3-(3-phenylbut-2-en-1-yl)indolin-2-one (**40**).

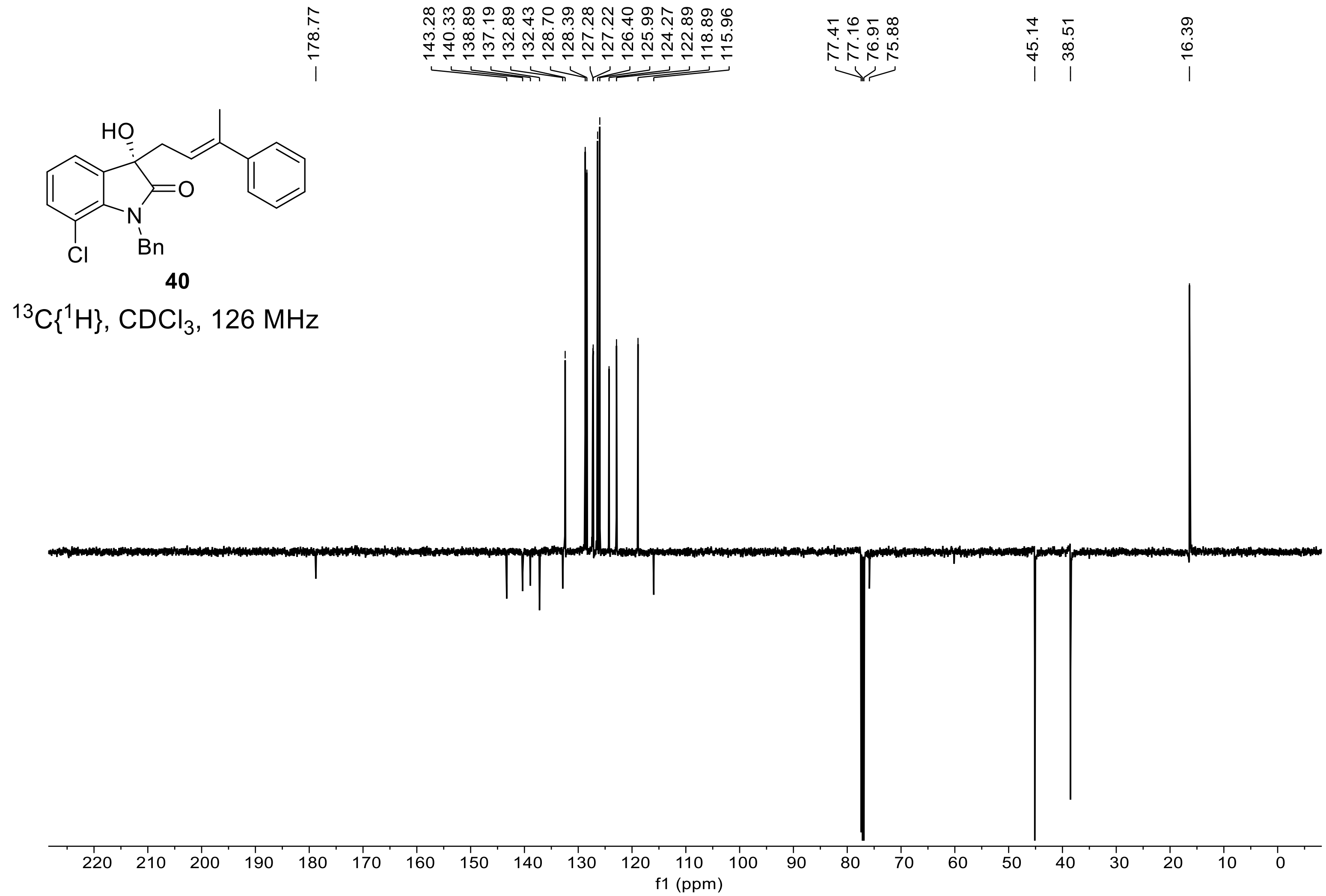

Fig. S375: $^{13}C\{^{1}H\}$ NMR spectrum for (*S*,*E*)-1-Benzyl-7-chloro-3-hydroxy-3-(3-phenylbut-2-en-1-yl)indolin-2-one (**40**).

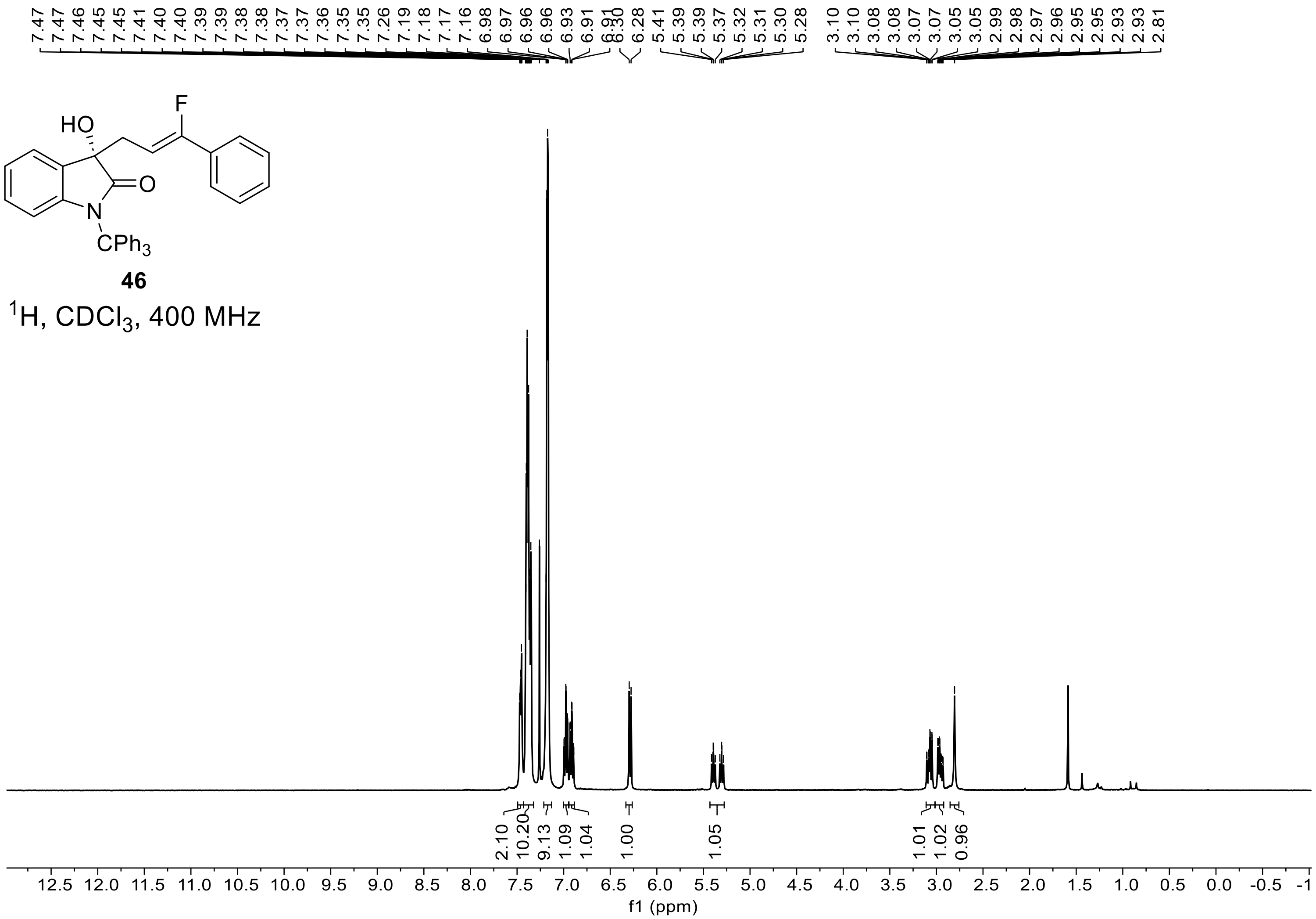

Fig. S376: ^{1}H NMR spectrum for (*S*,*Z*)-3-(3-fluoro-3-phenylallyl)-3-hydroxy-1-tritylindolin-2-one (**46**).

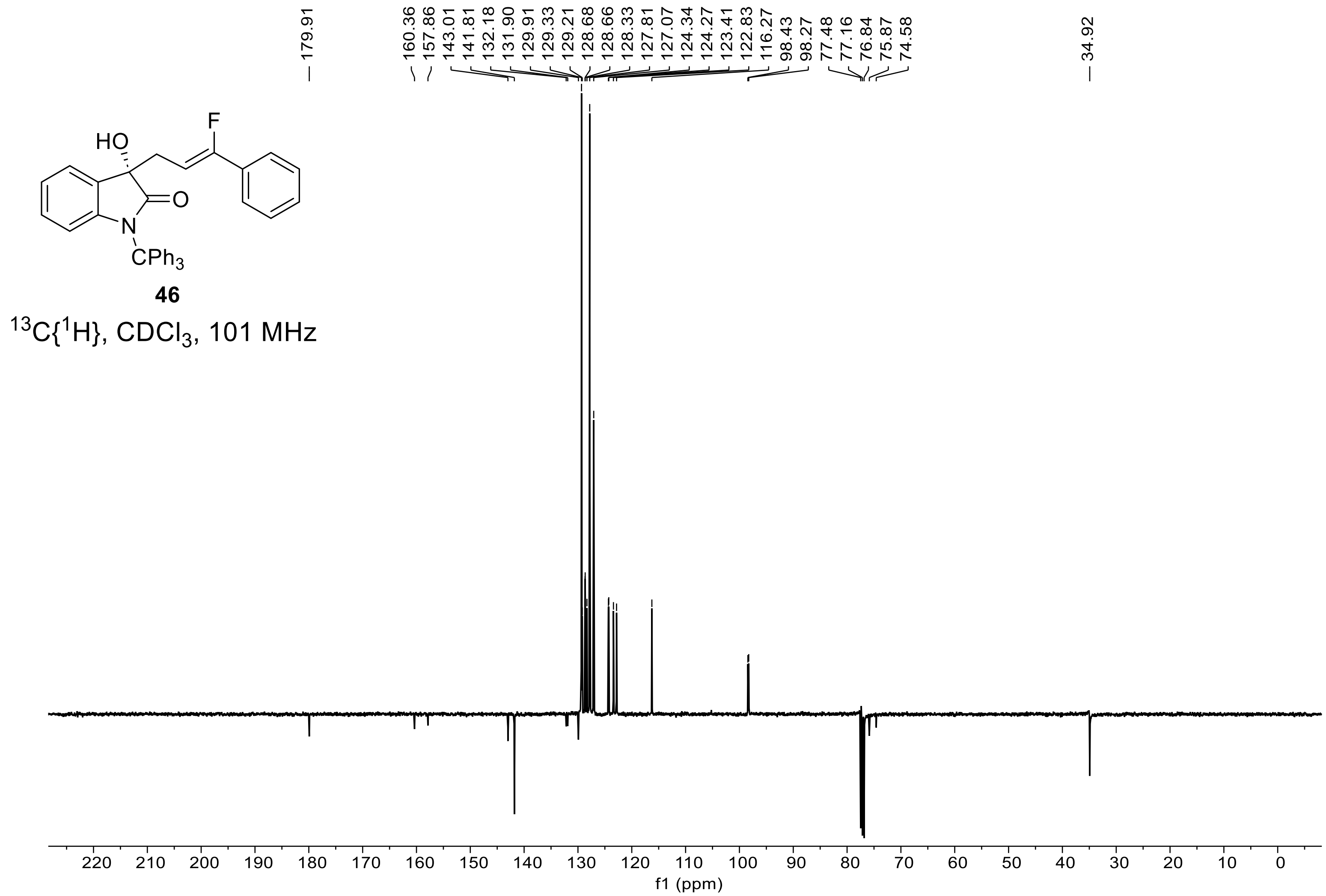

Fig. S378: $^{13}C\{^1H\}$ NMR spectrum for (*S*,*Z*)-3-(3-fluoro-3-phenylallyl)-3-hydroxy-1-tritylindolin-2-one (**46**).

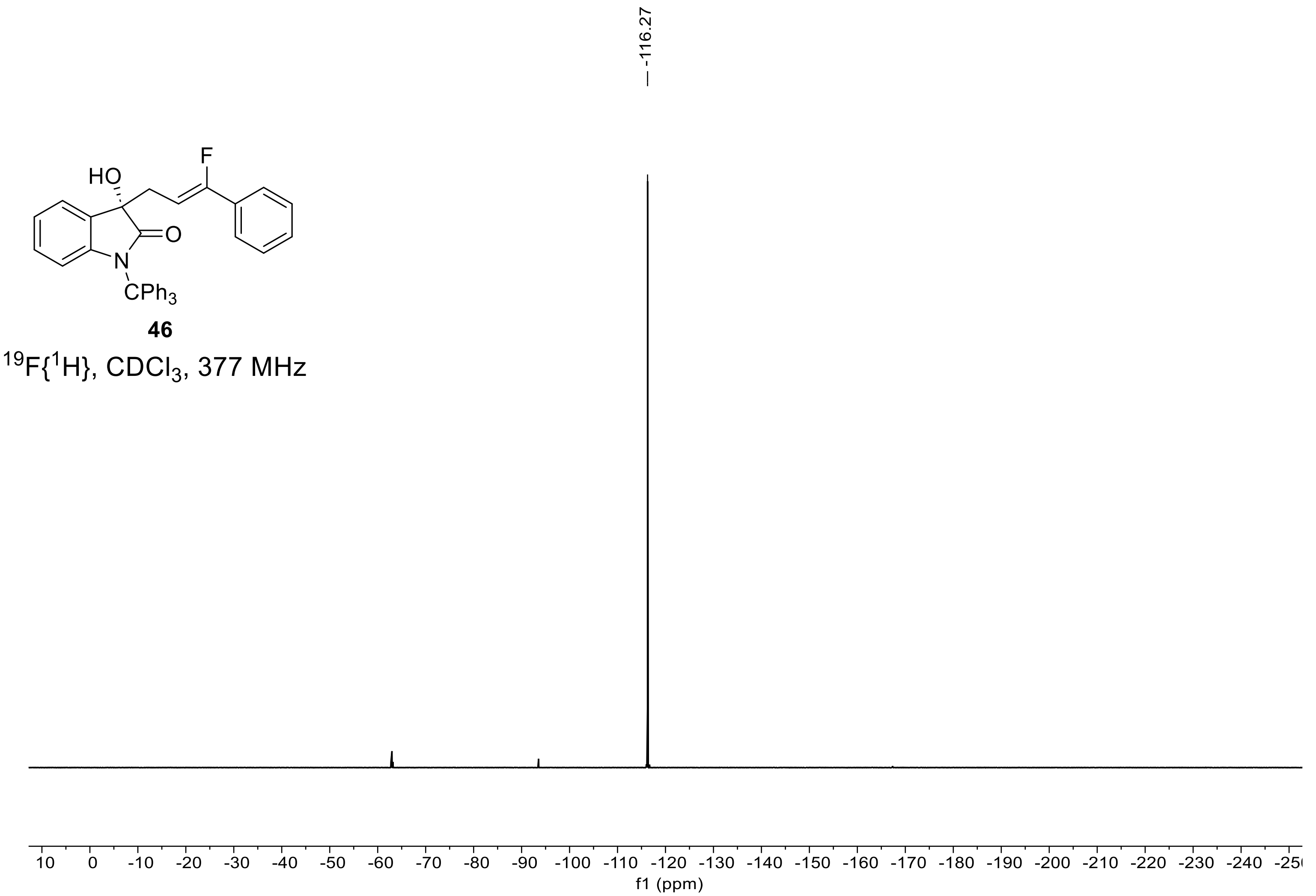

Fig. S378: $^{19}F\{^1H\}$ NMR spectrum for (*S*,*Z*)-3-(3-fluoro-3-phenylallyl)-3-hydroxy-1-tritylindolin-2-one (**46**).

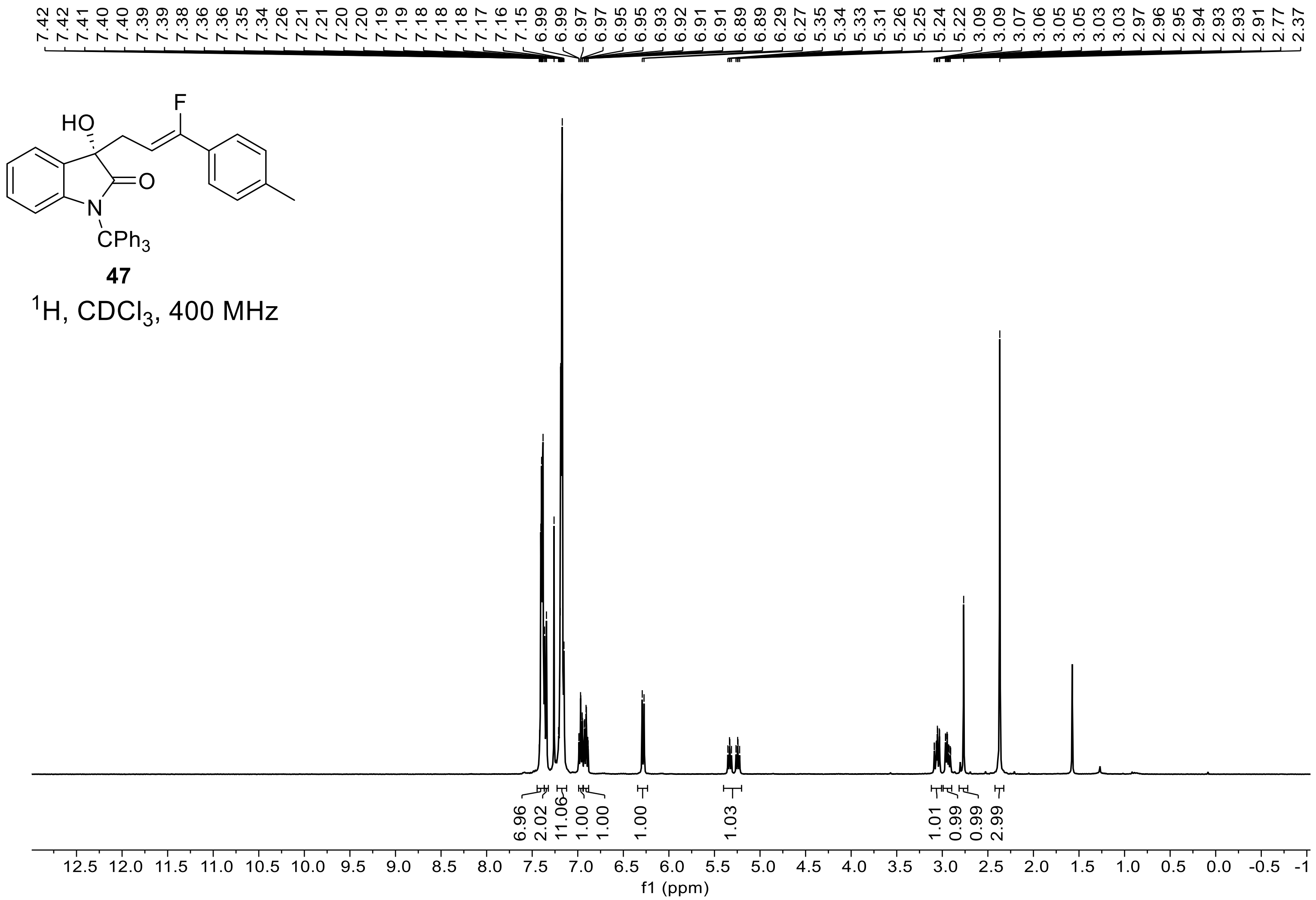

Fig. S379: ^{1}H NMR spectrum for (*S*,*Z*)-3-(3-Fluoro-3-(*p*-tolyl)allyl)-3-hydroxy-1-tritylindolin-2-one (**47**).

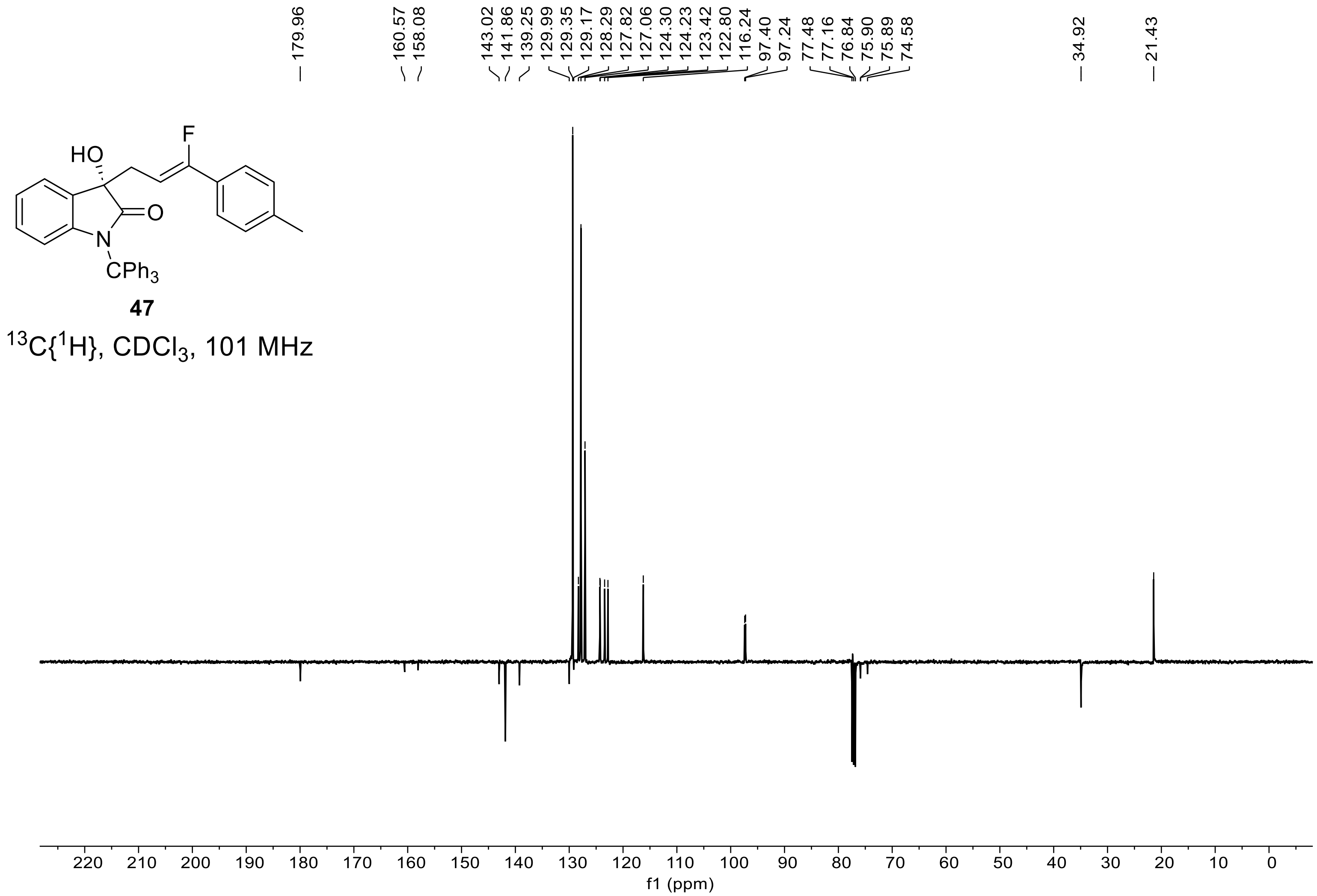

Fig. S380: $^{13}C\{^{1}H\}$ NMR spectrum for (*S*,*Z*)-3-(3-Fluoro-3-(*p*-tolyl)allyl)-3-hydroxy-1-tritylindolin-2-one (**47**).

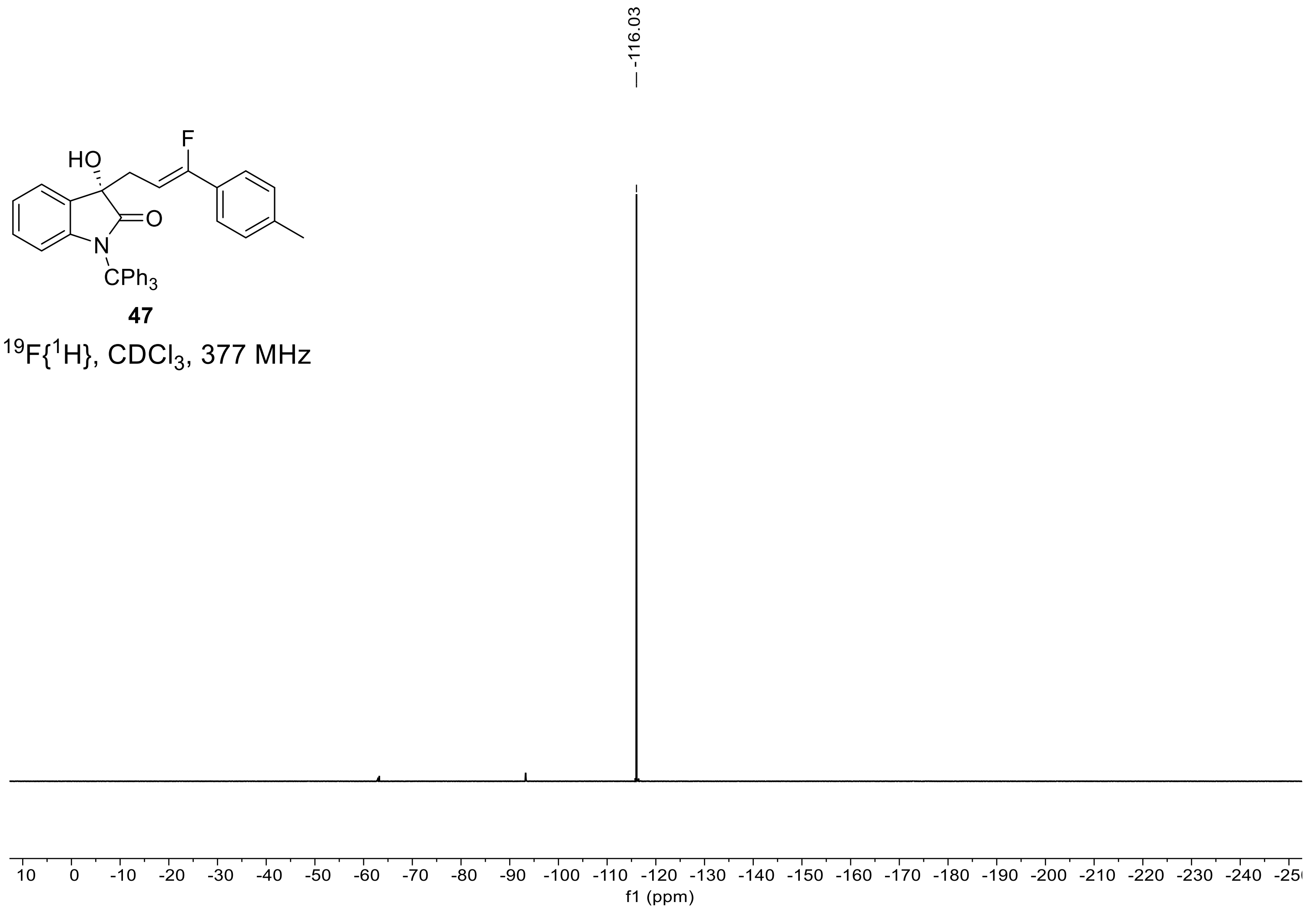

Fig. S381: $^{19}F\{^1H\}$ NMR spectrum for (*S*,*Z*)-3-(3-Fluoro-3-(*p*-tolyl)allyl)-3-hydroxy-1-tritylindolin-2-one (**47**).

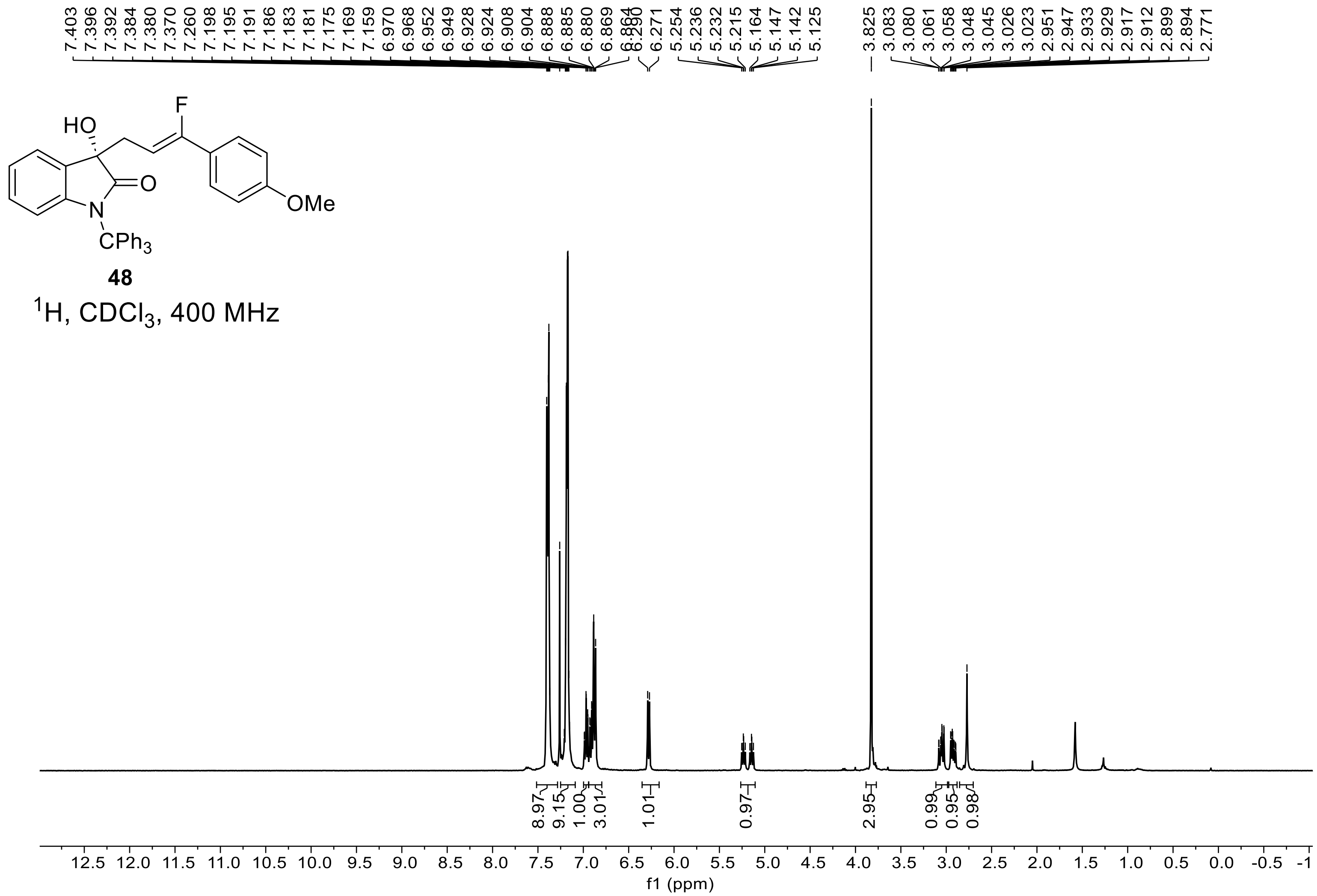

Fig. S382: ^{1}H NMR spectrum for (*S*,*Z*)-3-[3-Fluoro-3-(4-methoxyphenyl)allyl]-3-hydroxy-1-tritylindolin-2-one (**48**).

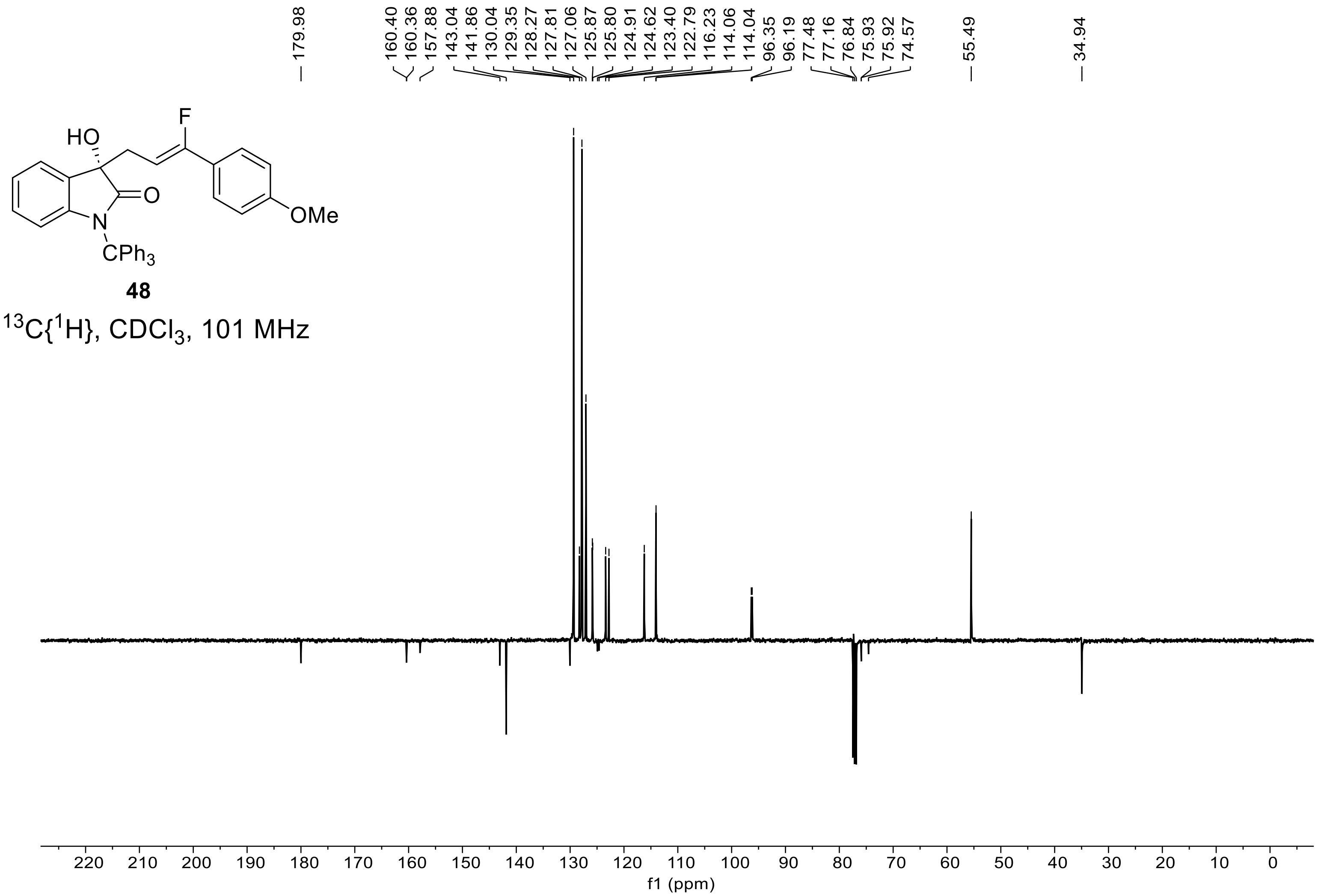

Fig. S383: $^{13}C\{^1H\}$ NMR spectrum for (*S*,*Z*)-3-[3-Fluoro-3-(4-methoxyphenyl)allyl]-3-hydroxy-1-tritylindolin-2-one (**48**).

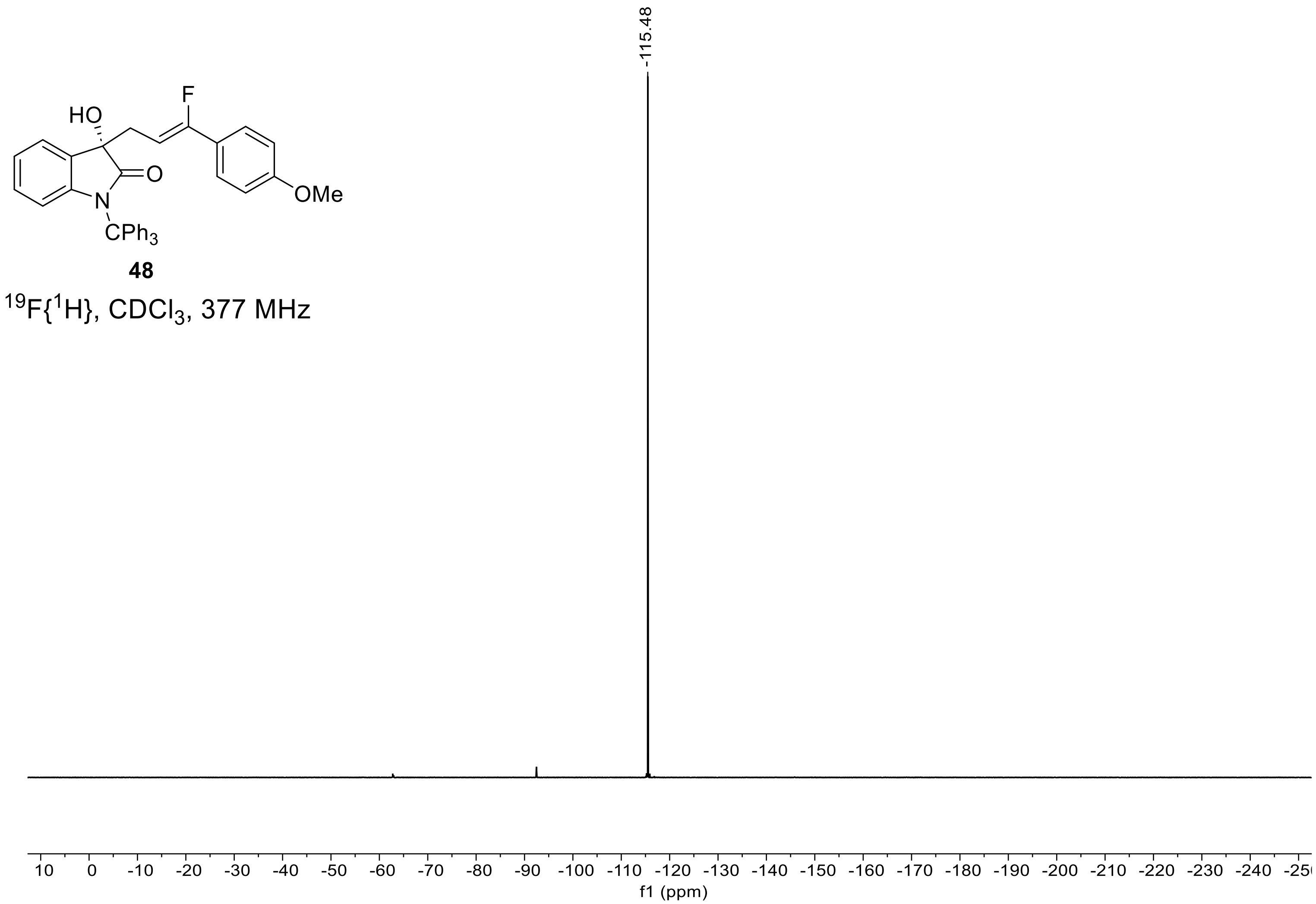

Fig. S384: $^{19}F\{^1H\}$ NMR spectrum for (*S*,*Z*)-3-[3-Fluoro-3-(4-methoxyphenyl)allyl]-3-hydroxy-1-tritylindolin-2-one (**48**).

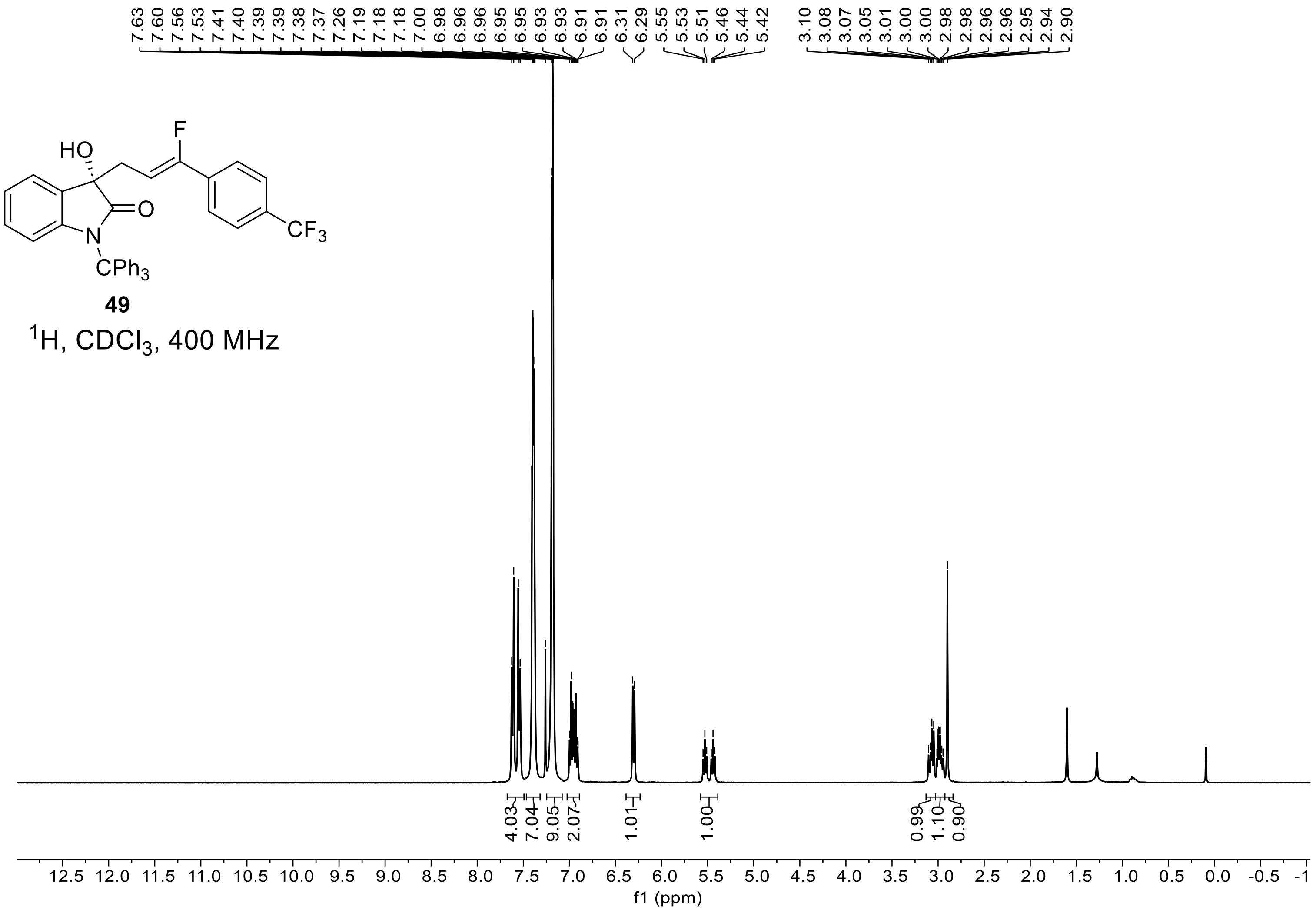

Fig. S385: ^{1}H NMR spectrum for (*S*,*Z*)-3-{3-Fluoro-3-[4-(trifluoromethyl)phenyl]allyl}-3-hydroxy-1-tritylindolin-2-one (**49**).

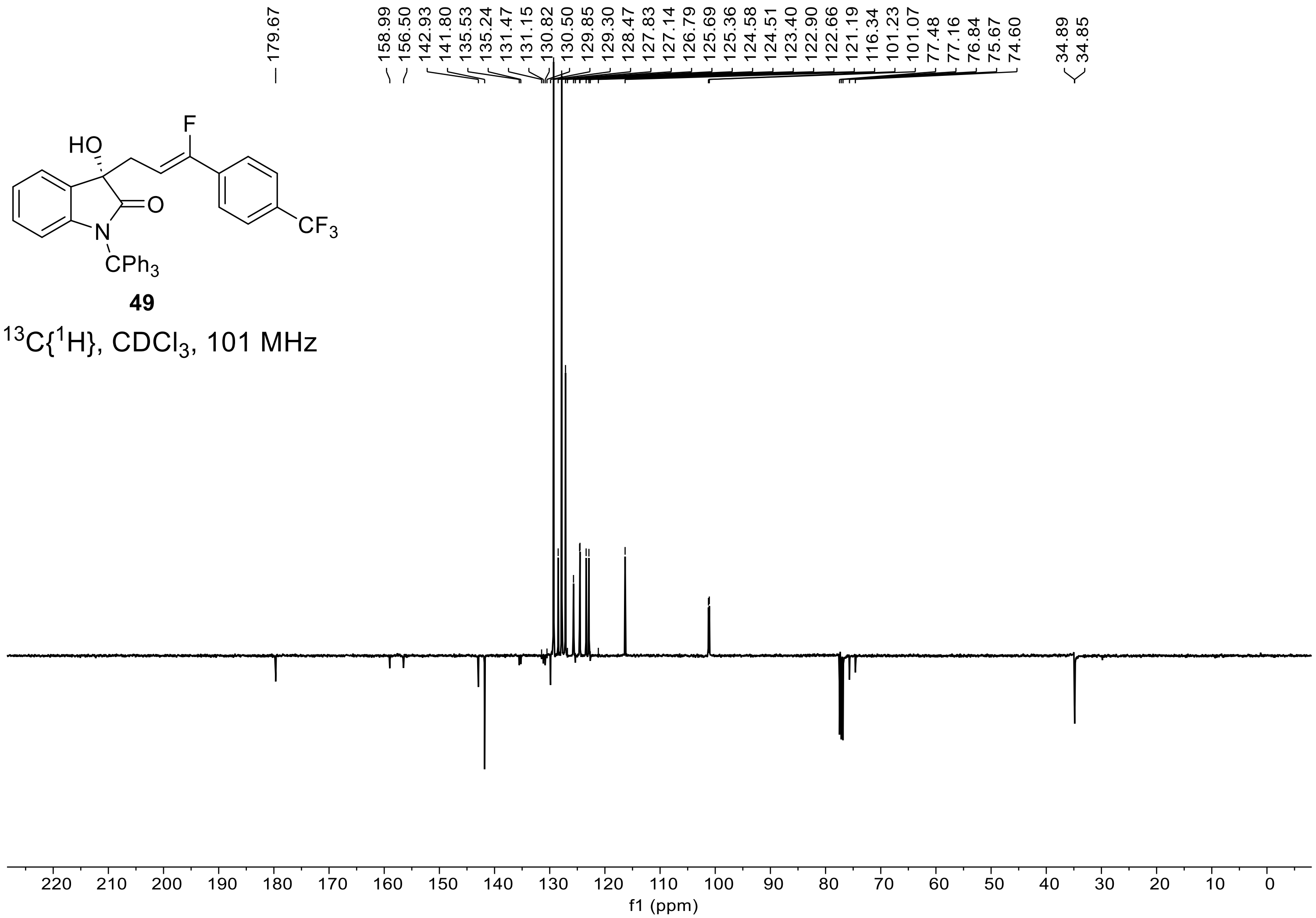

Fig. S386: $^{13}C\{^1H\}$ NMR spectrum for (*S*,*Z*)-3-{3-Fluoro-3-[4-(trifluoromethyl)phenyl]allyl}-3-hydroxy-1-tritylindolin-2-one (**49**).

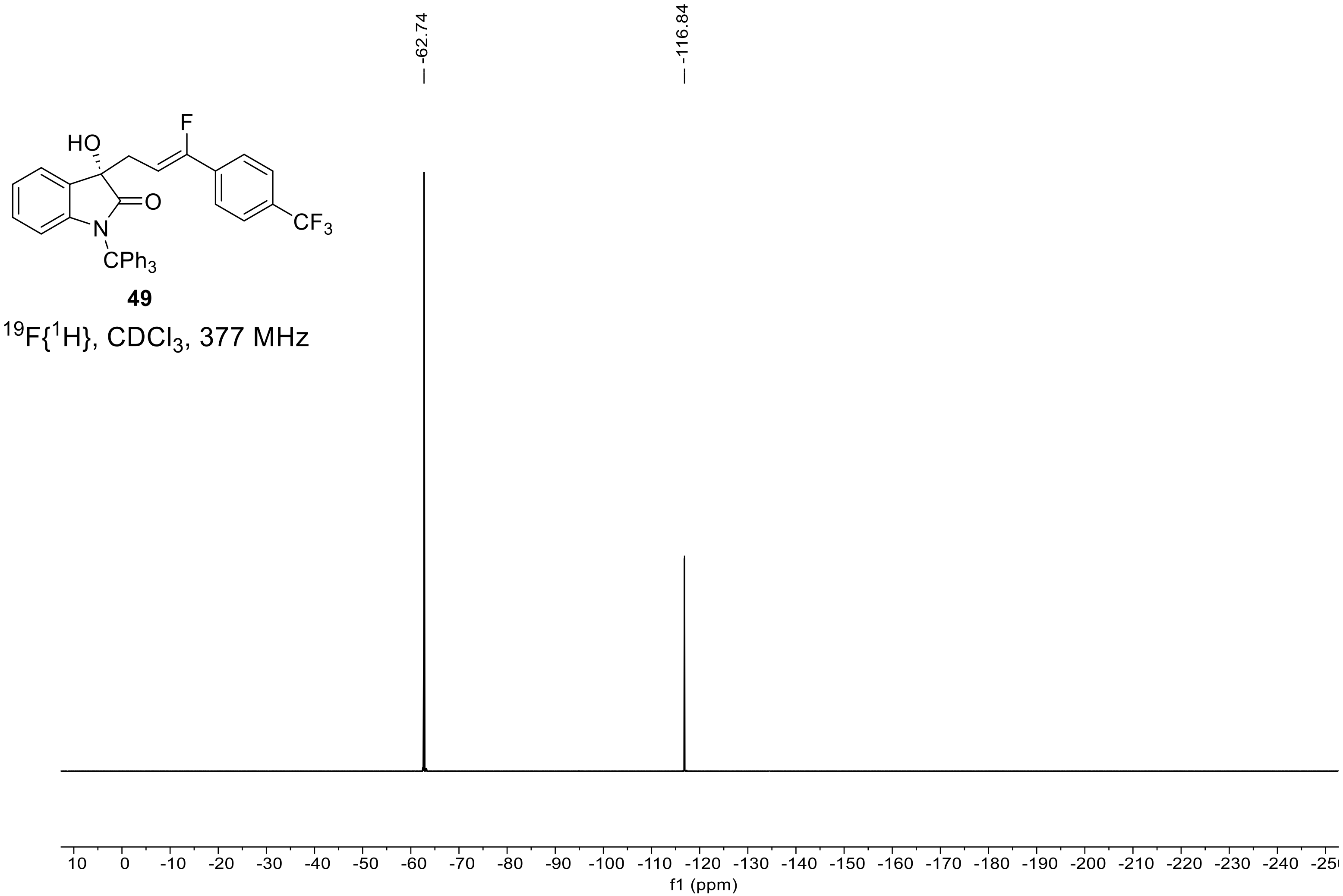

Fig. S387: $^{19}F\{^1H\}$ NMR spectrum for (*S*,*Z*)-3-{3-Fluoro-3-[4-(trifluoromethyl)phenyl]allyl}-3-hydroxy-1-tritylindolin-2-one (**49**).

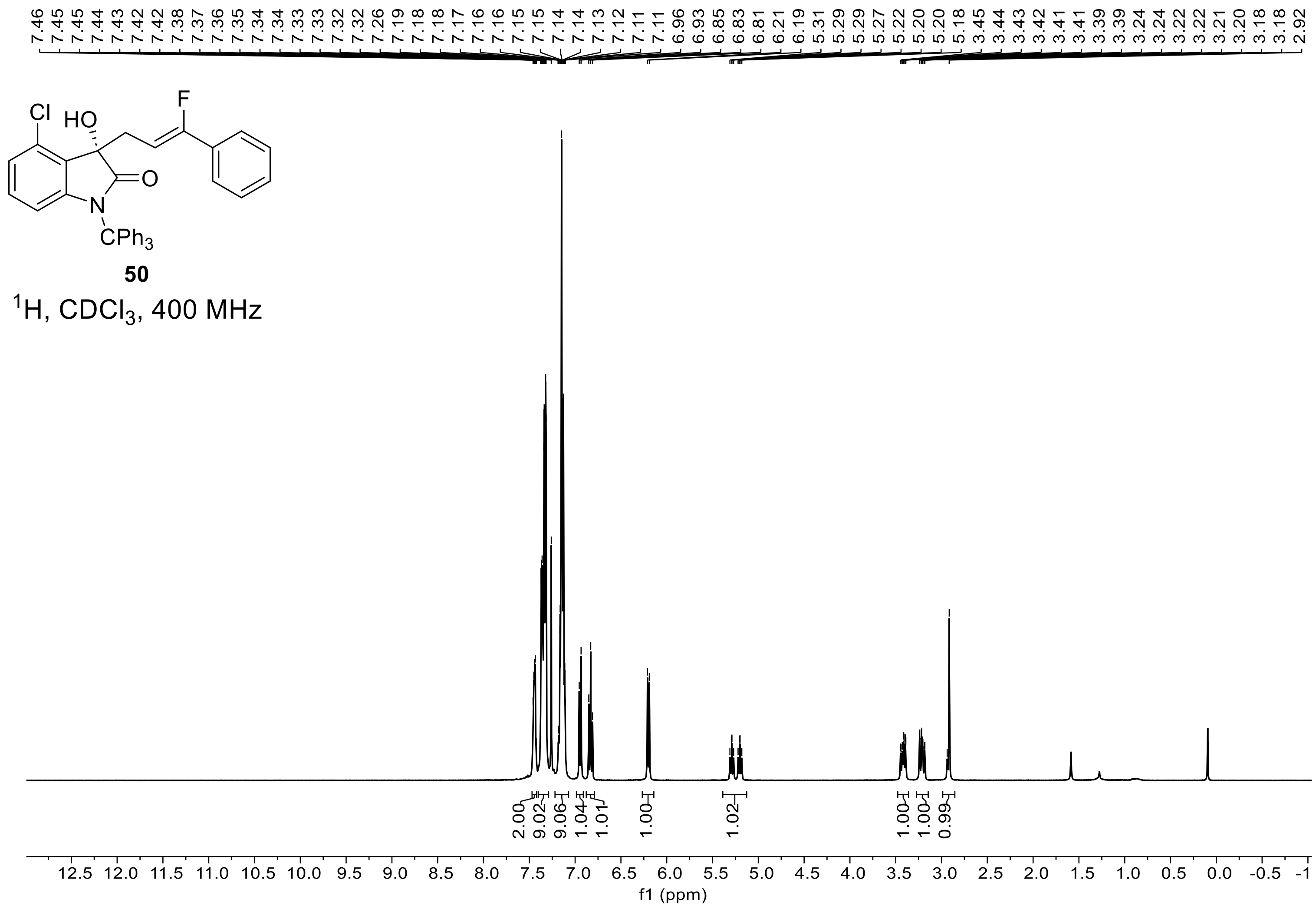

Fig. S388: ^{1}H NMR spectrum for (*S*,*Z*)-4-Chloro-3-(3-fluoro-3-phenylallyl)-3-hydroxy-1-tritylindolin-2-one (**50**).

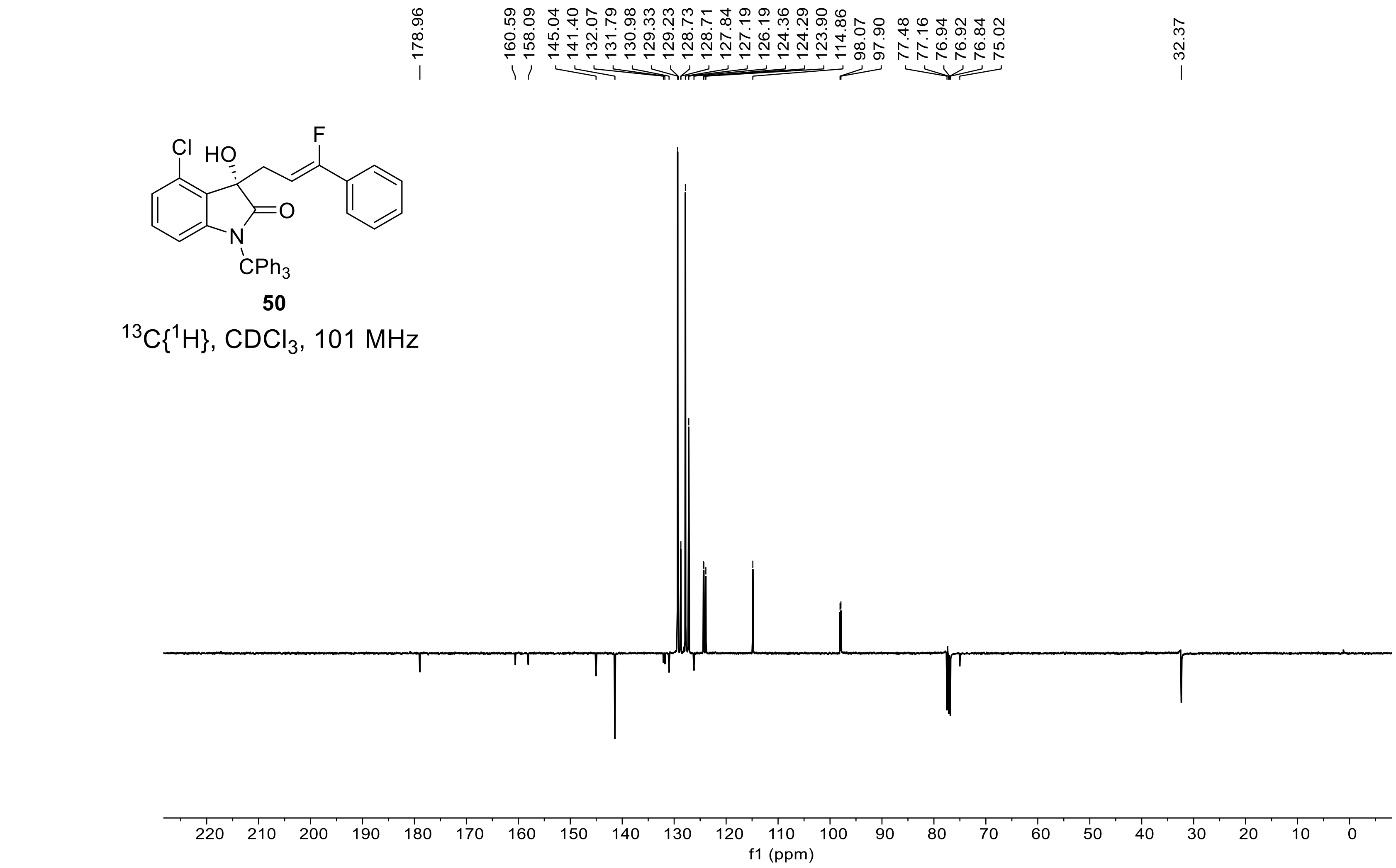

Fig. S389: $^{13}C\{^{1}H\}$ NMR spectrum for (*S*,*Z*)-4-Chloro-3-(3-fluoro-3-phenylallyl)-3-hydroxy-1-tritylindolin-2-one (**50**).

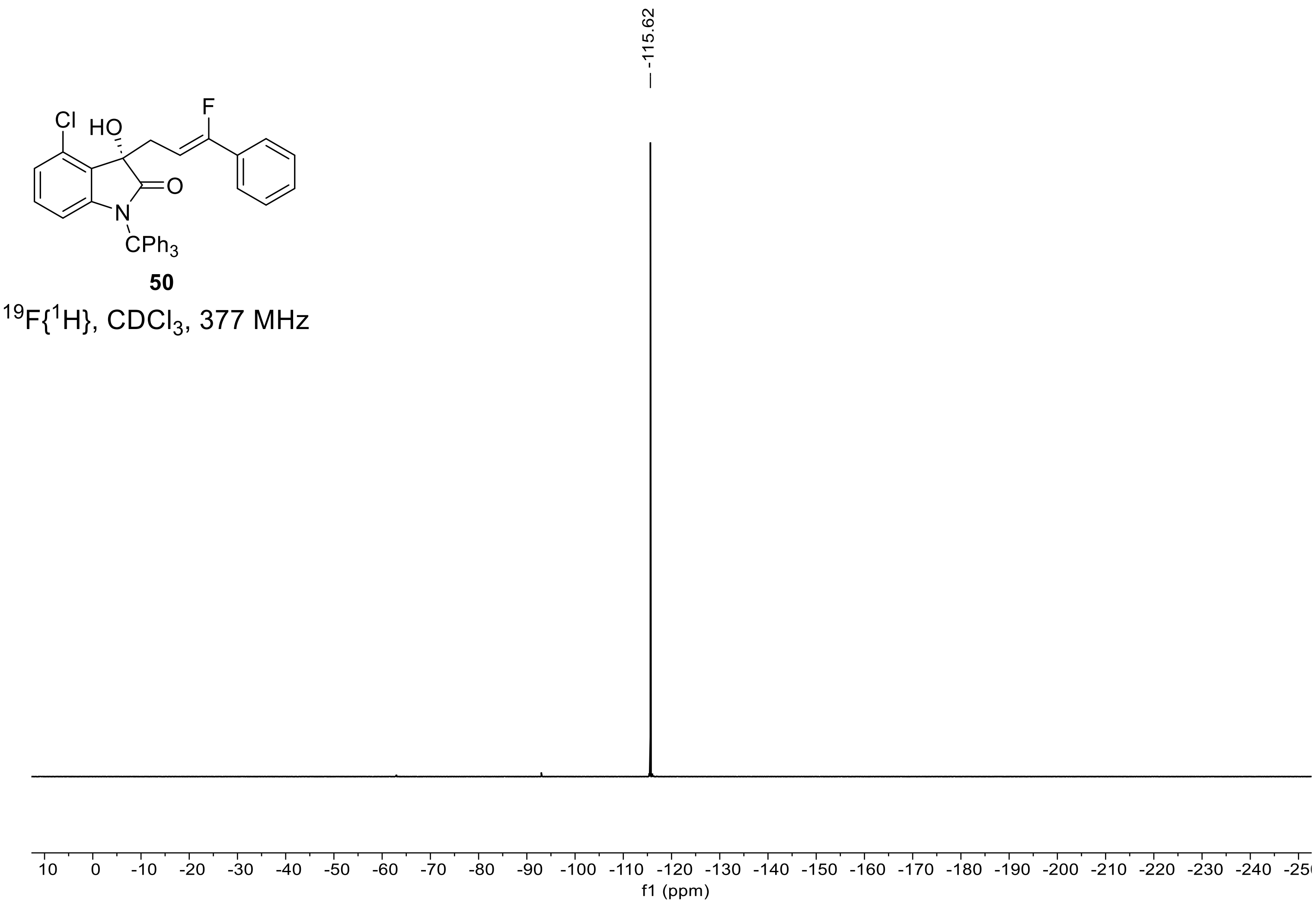

Fig. S390: $^{19}F\{^1H\}$ NMR spectrum for (*S*,*Z*)-4-Chloro-3-(3-fluoro-3-phenylallyl)-3-hydroxy-1-tritylindolin-2-one (**50**).

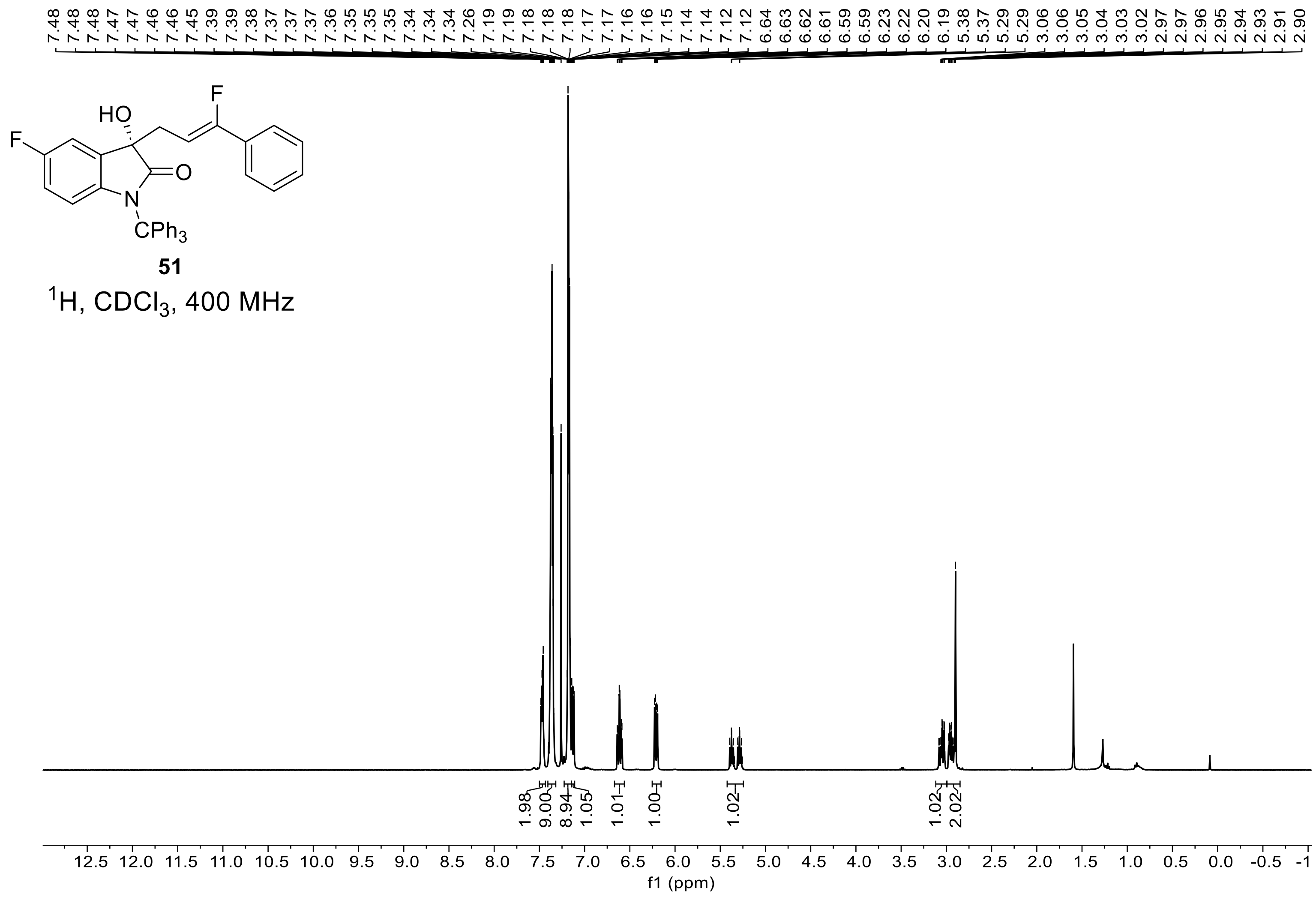

Fig. S391: ^{1}H NMR spectrum for (*S*,*Z*)-5-Fluoro-3-(3-fluoro-3-phenylallyl)-3-hydroxy-1-tritylindolin-2-one (**51**).

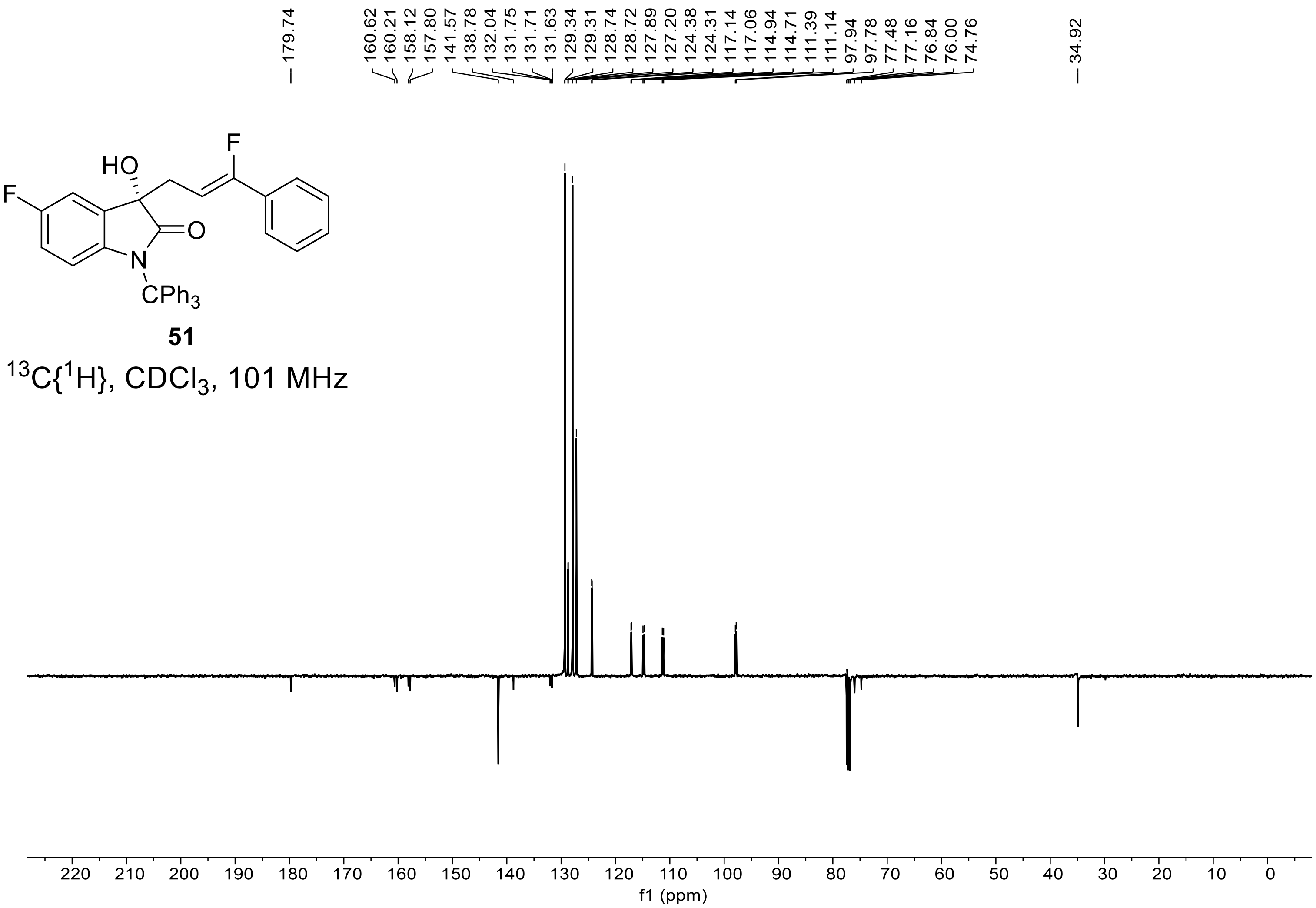

Fig. S392: $^{13}C\{^{1}H\}$ NMR spectrum for (*S*,*Z*)-5-Fluoro-3-(3-fluoro-3-phenylallyl)-3-hydroxy-1-tritylindolin-2-one (**51**).

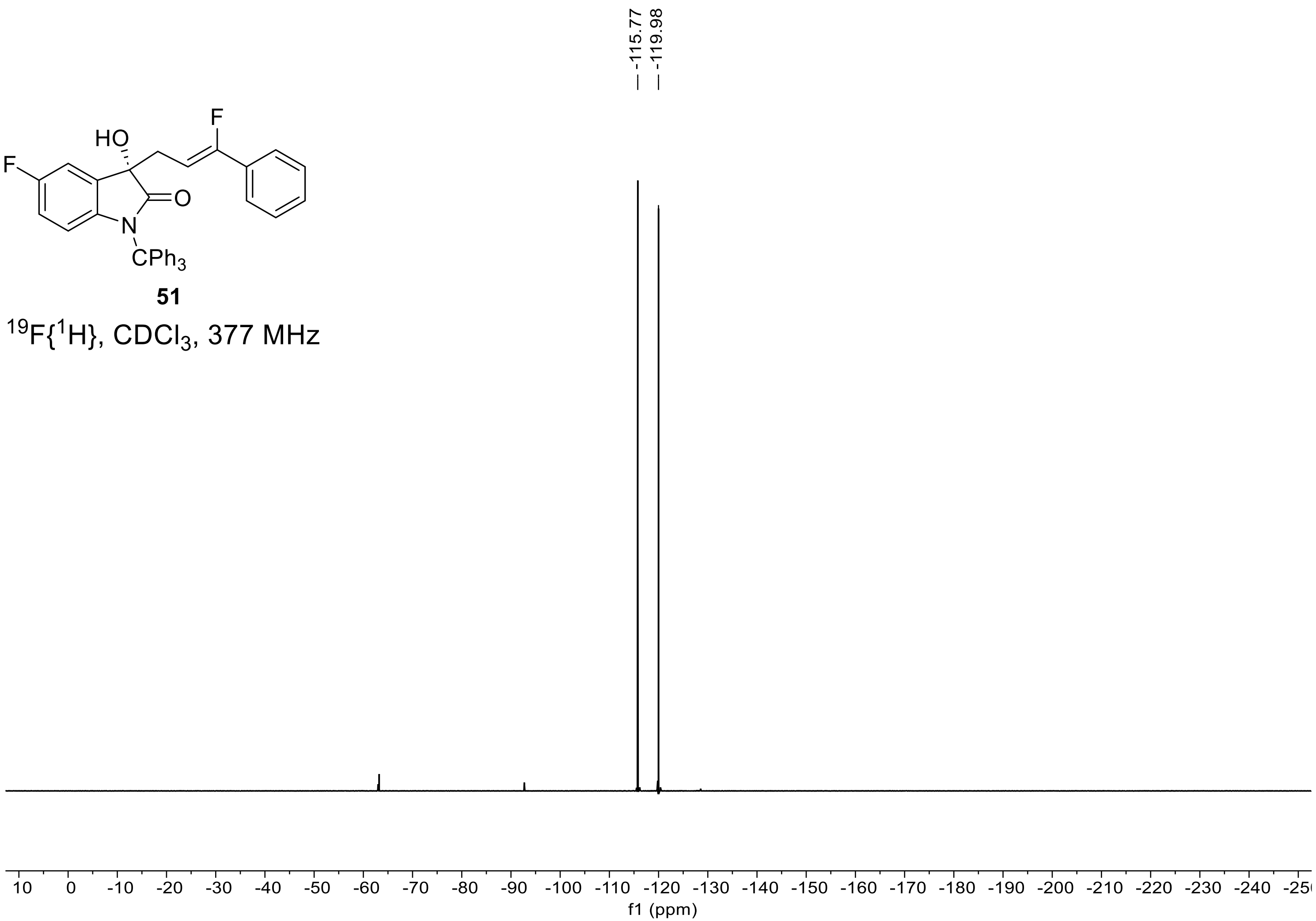

Fig. S393: $^{19}F\{^{1}H\}$ NMR spectrum for (*S*,*Z*)-5-Fluoro-3-(3-fluoro-3-phenylallyl)-3-hydroxy-1-tritylindolin-2-one (**51**).

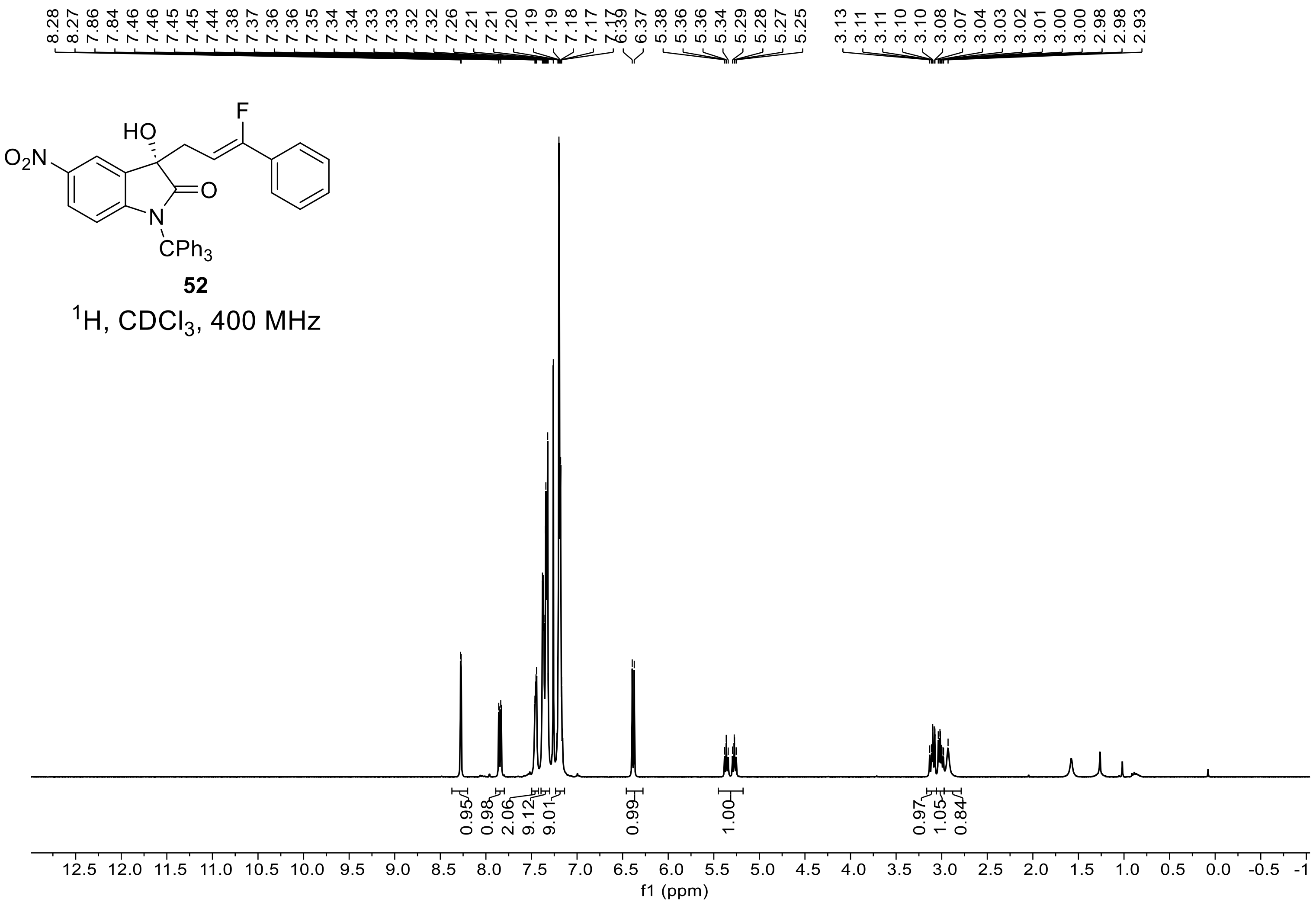

Fig. S394: ^{1}H NMR spectrum for (*S*,*Z*)-3-(3-Fluoro-3-phenylallyl)-3-hydroxy-5-nitro-1-tritylindolin-2-one (**52**).

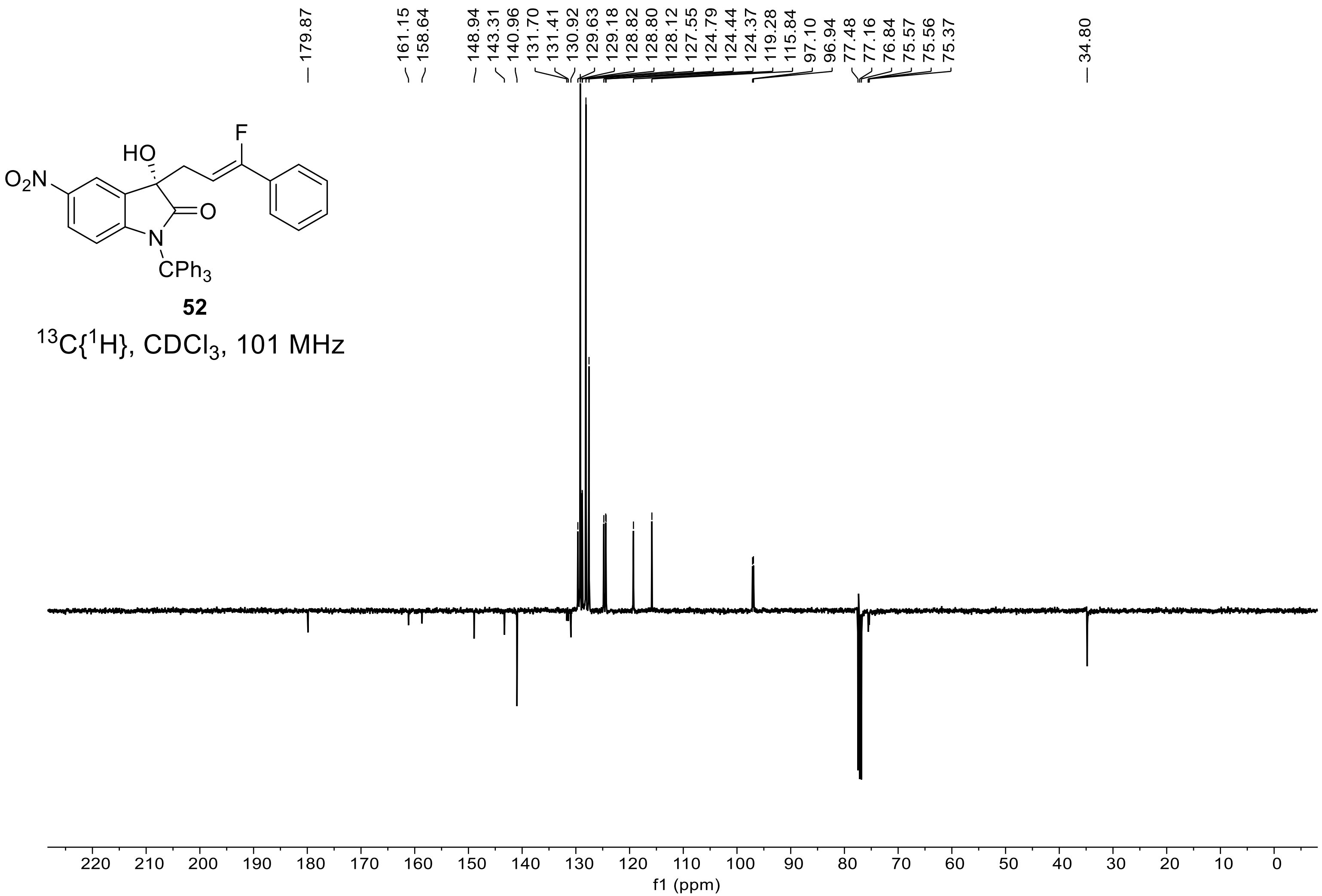

Fig. S395: $^{13}C\{^1H\}$ NMR spectrum for (*S*,*Z*)-3-(3-Fluoro-3-phenylallyl)-3-hydroxy-5-nitro-1-tritylindolin-2-one (**52**).

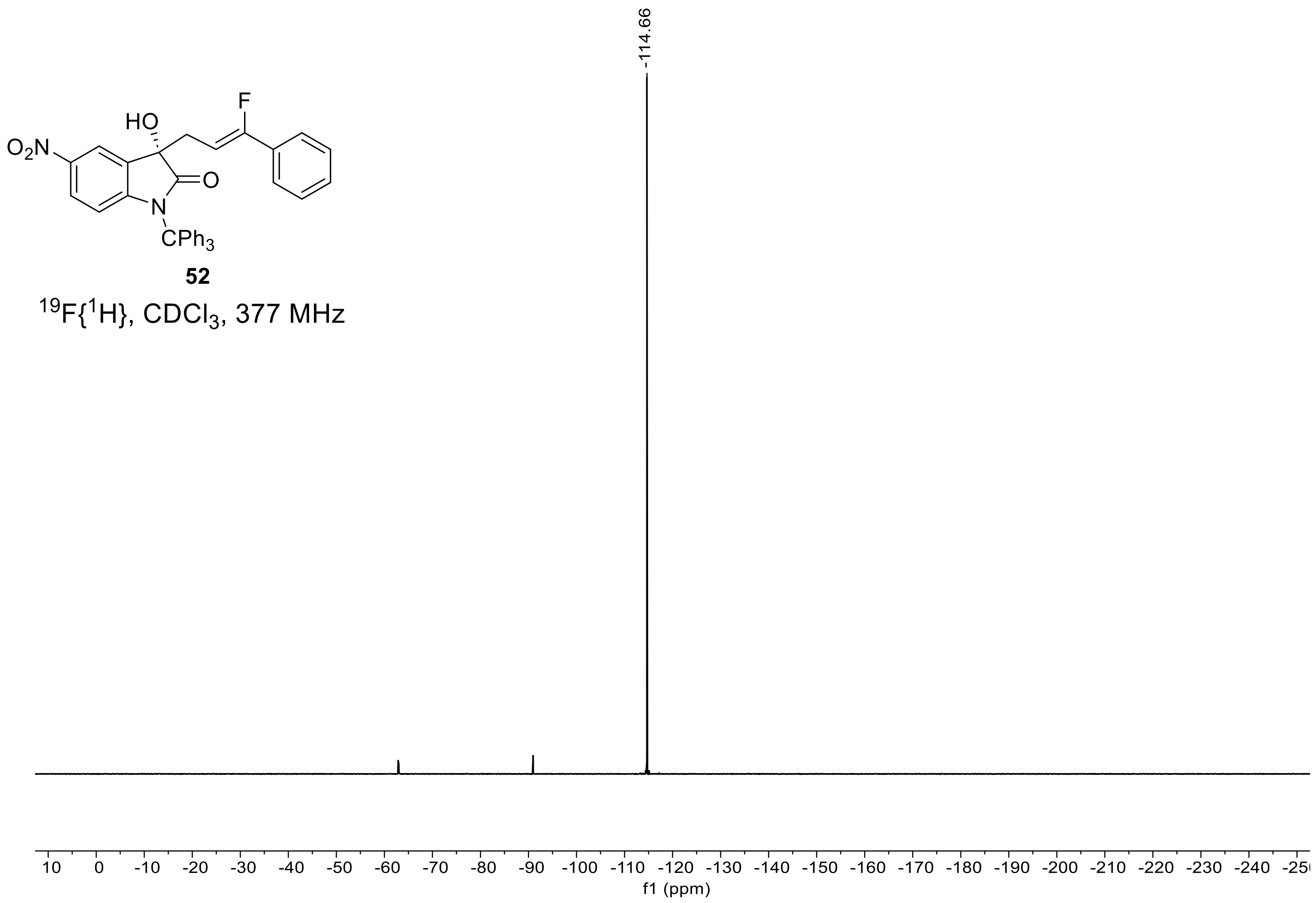

Fig. S396: $^{19}F\{^1H\}$ NMR spectrum for (*S*,*Z*)-3-(3-Fluoro-3-phenylallyl)-3-hydroxy-5-nitro-1-tritylindolin-2-one (**52**).

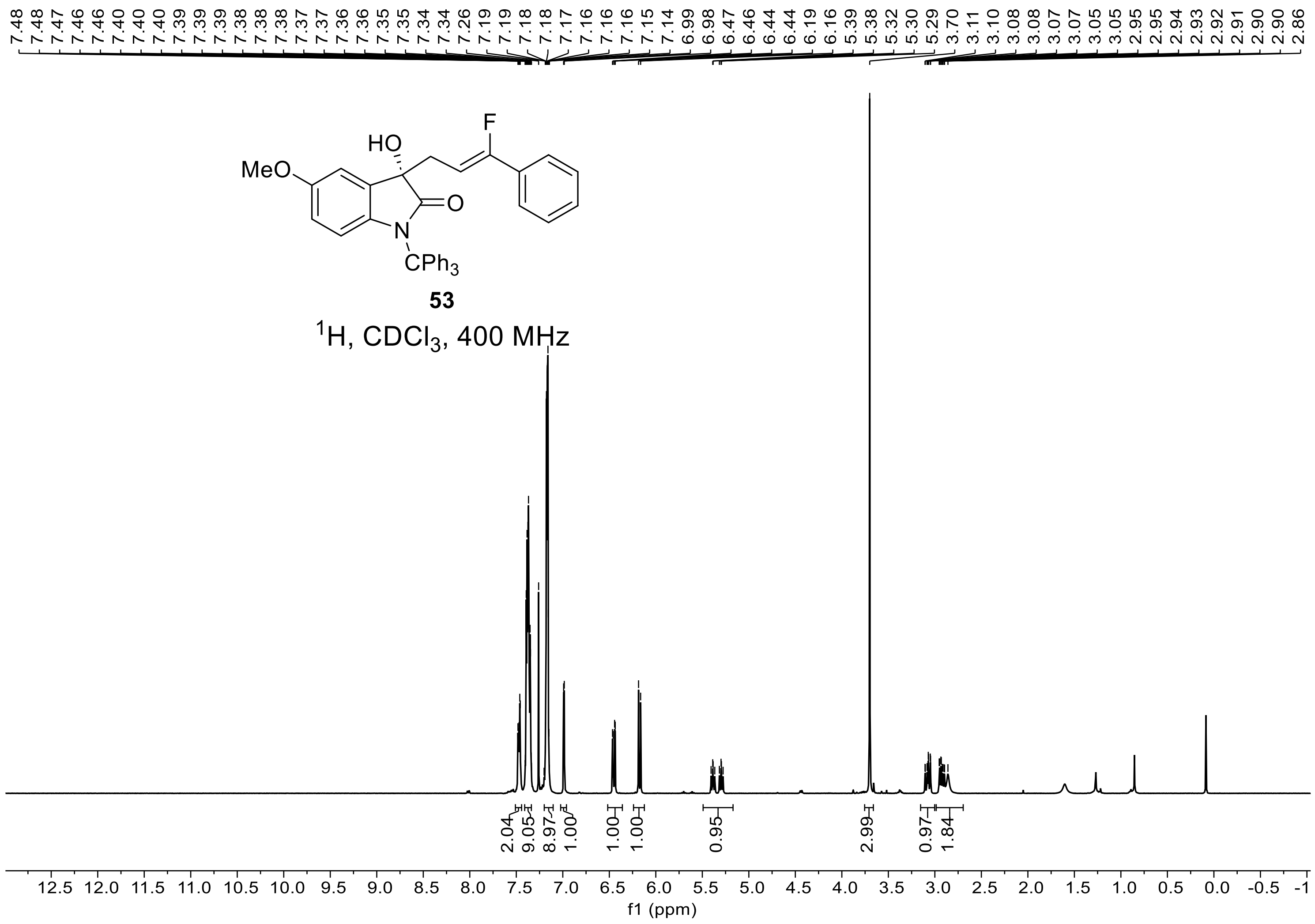

Fig. S397: ^{1}H NMR spectrum for (*S*,*Z*)-3-(3-Fluoro-3-phenylallyl)-3-hydroxy-5-methoxy-1-tritylindolin-2-one (**53**).

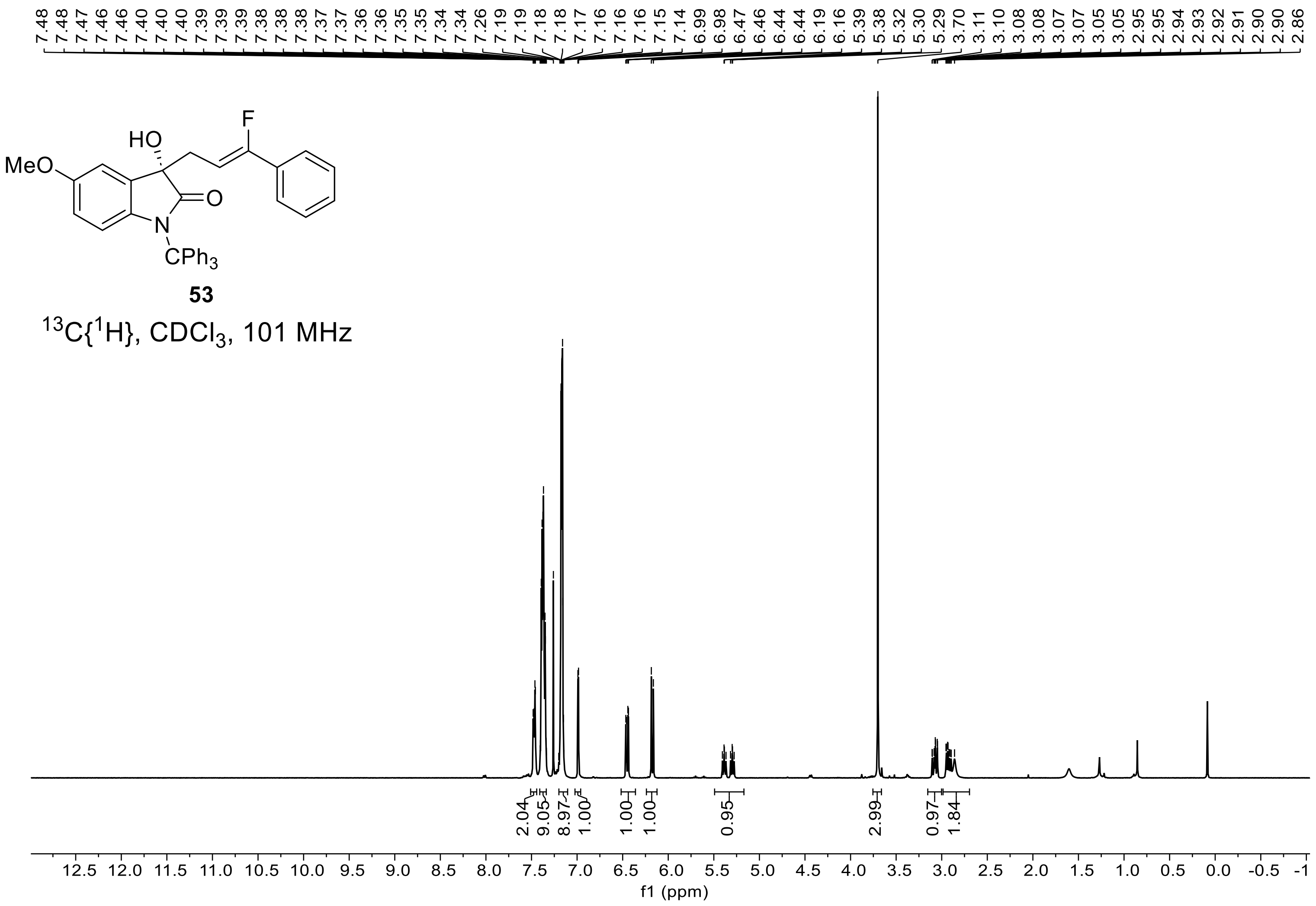

Fig. S398: $^{13}C\{^1H\}$ NMR spectrum for (*S*,*Z*)-3-(3-Fluoro-3-phenylallyl)-3-hydroxy-5-methoxy-1-tritylindolin-2-one (**53**).

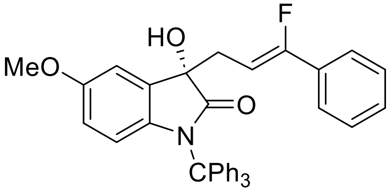

53

$^{19}F\{^1H\}$, $CDCl_3$, 377 MHz

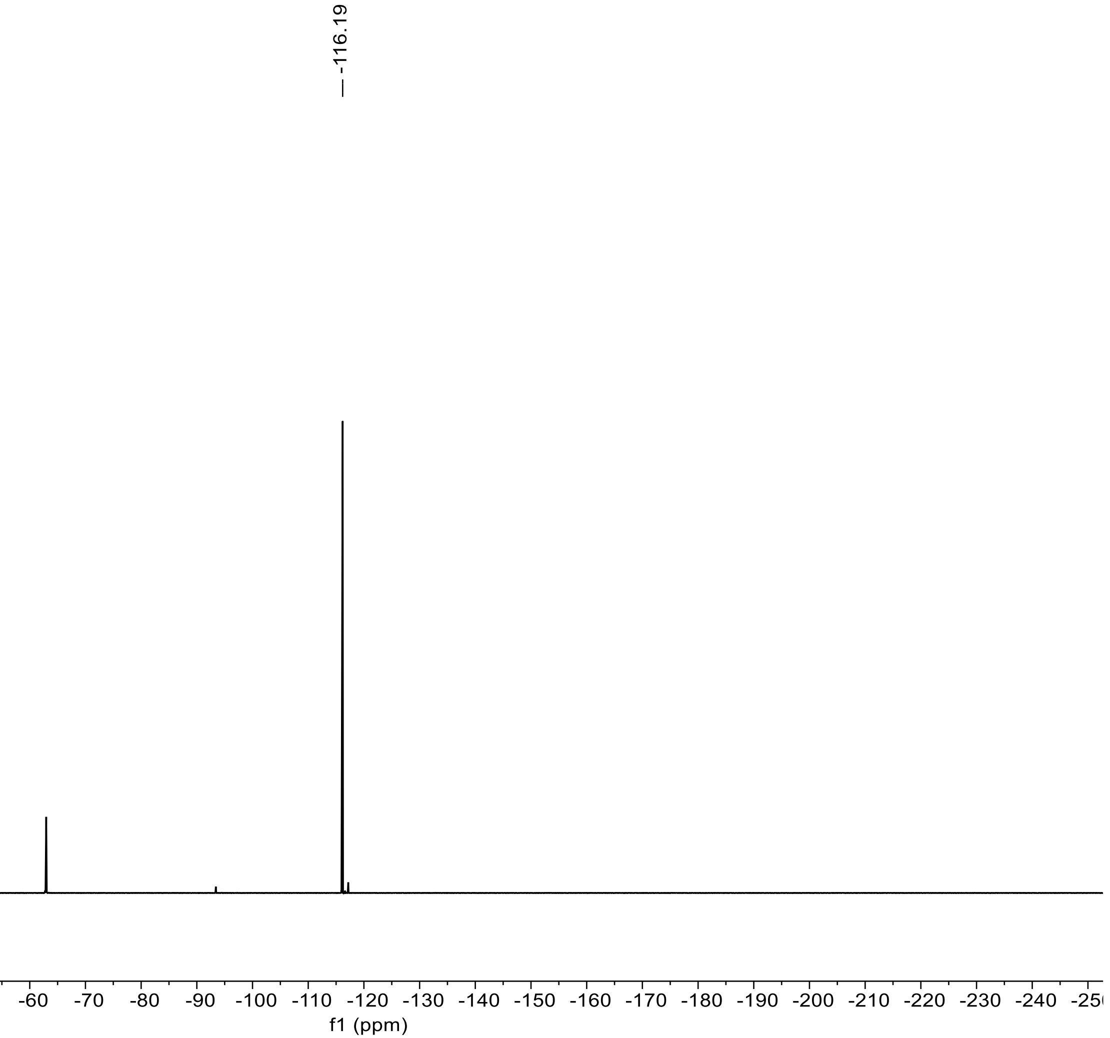

Fig. S399: $^{19}F\{^1H\}$ NMR spectrum for (*S*,*Z*)-3-(3-Fluoro-3-phenylallyl)-3-hydroxy-5-methoxy-1-tritylindolin-2-one (**53**).

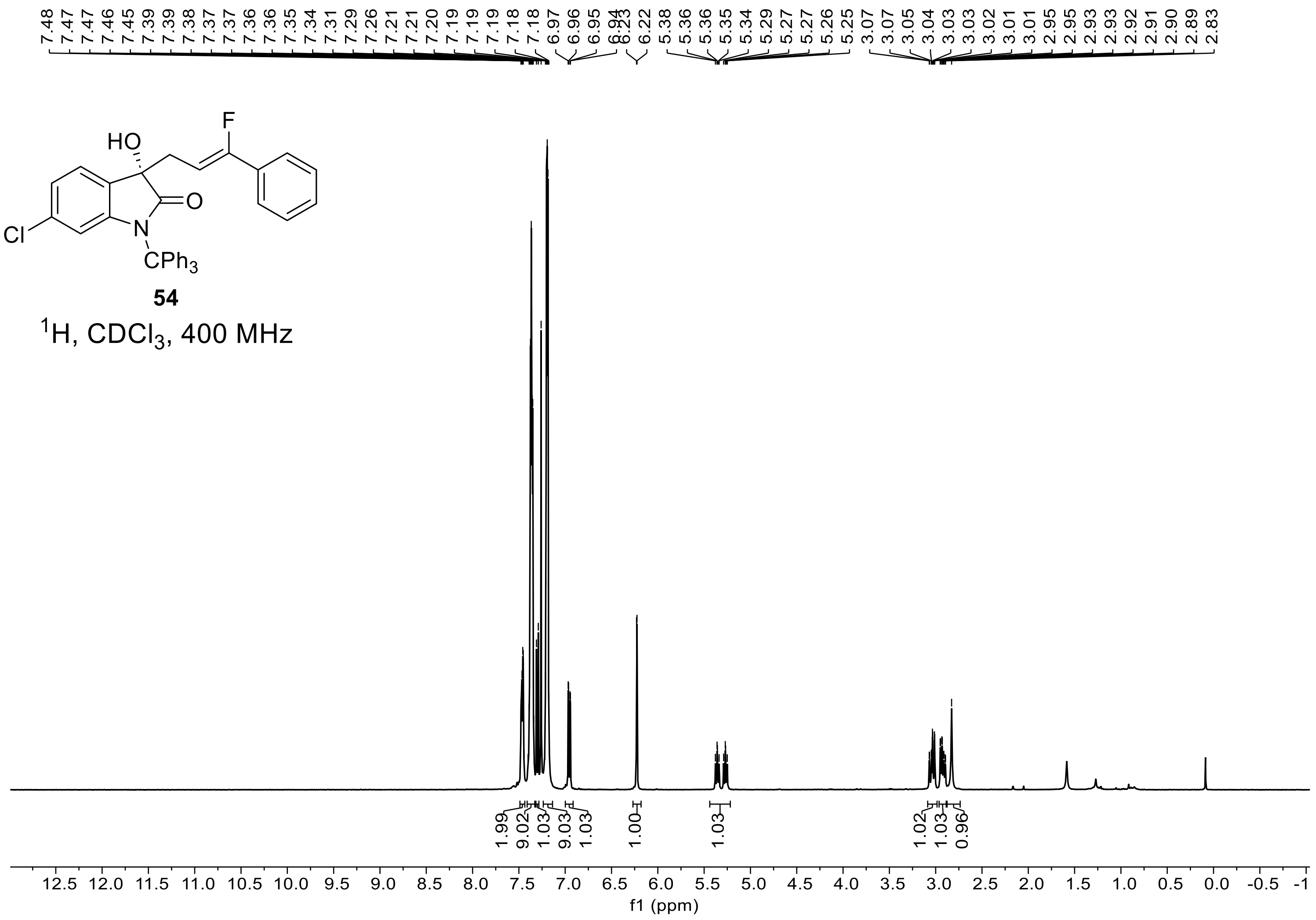

Fig. S400: ^{1}H NMR spectrum for (*S*,*Z*)-6-Chloro-3-(3-fluoro-3-phenylallyl)-3-hydroxy-1-tritylindolin-2-one (**54**).

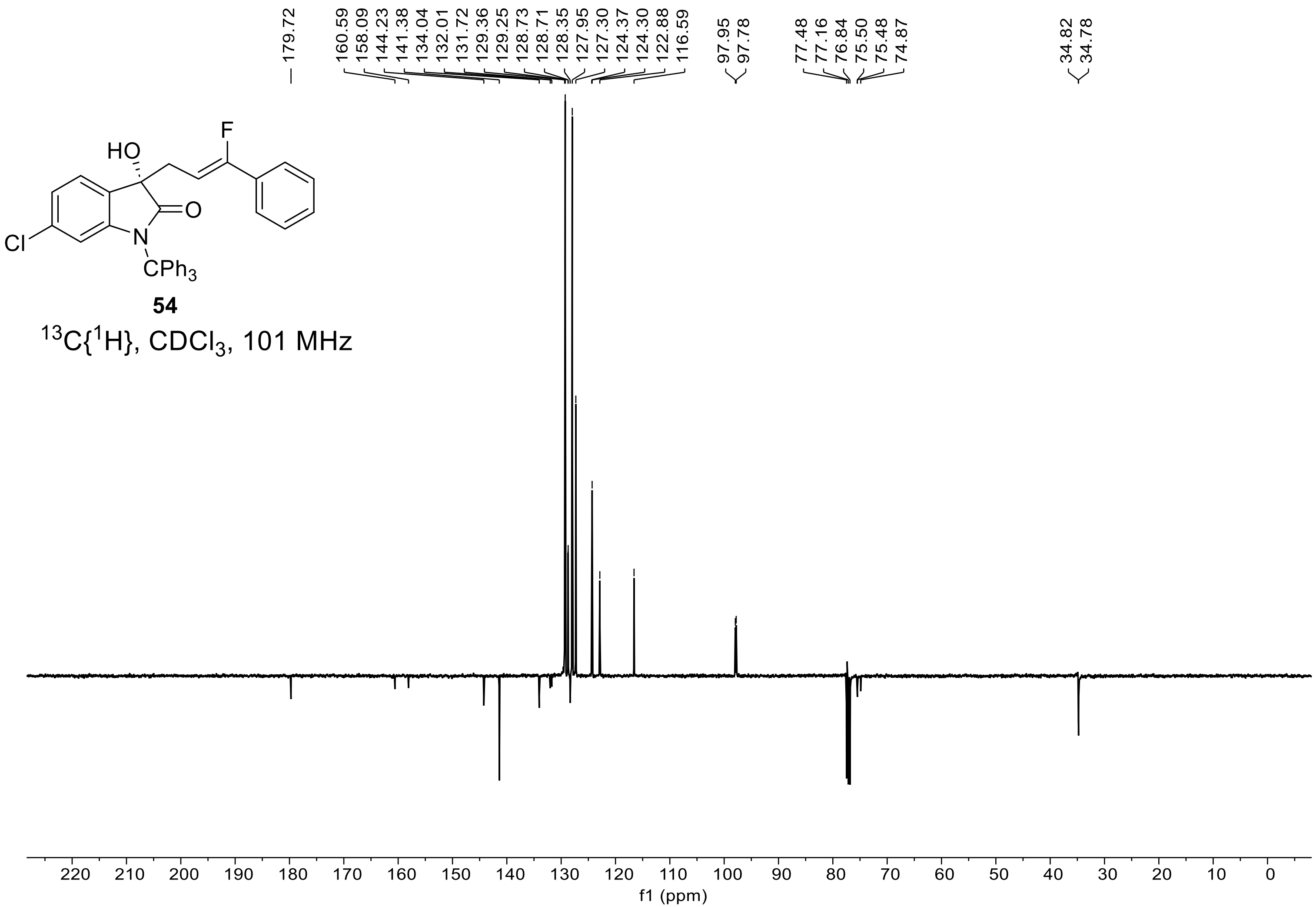

Fig. S401: $^{13}C\{^{1}H\}$ NMR spectrum for (*S*,*Z*)-6-Chloro-3-(3-fluoro-3-phenylallyl)-3-hydroxy-1-tritylindolin-2-one (**54**).

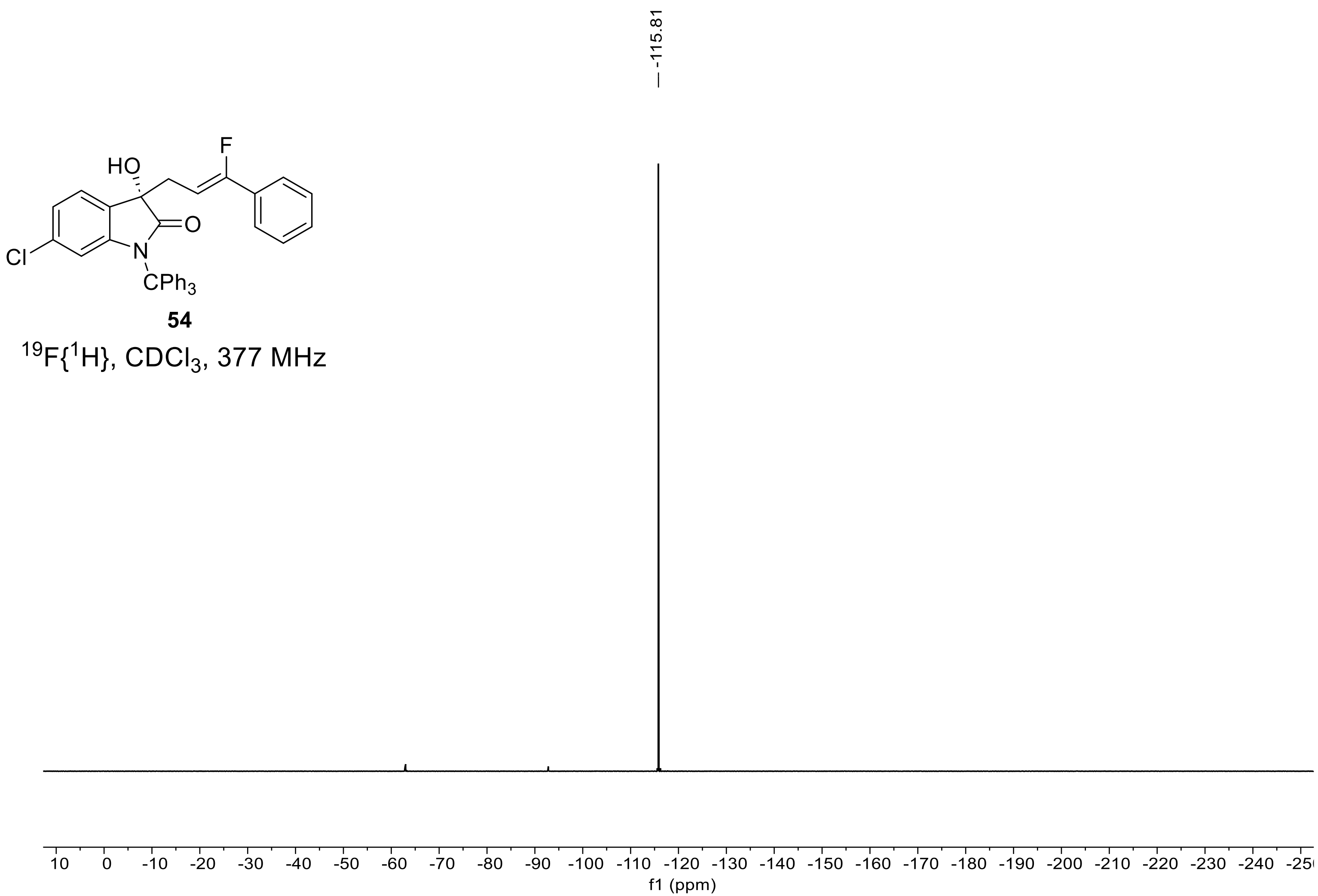

Fig. S402: $^{19}F\{^{1}H\}$ NMR spectrum for (*S*,*Z*)-6-Chloro-3-(3-fluoro-3-phenylallyl)-3-hydroxy-1-tritylindolin-2-one (**54**).

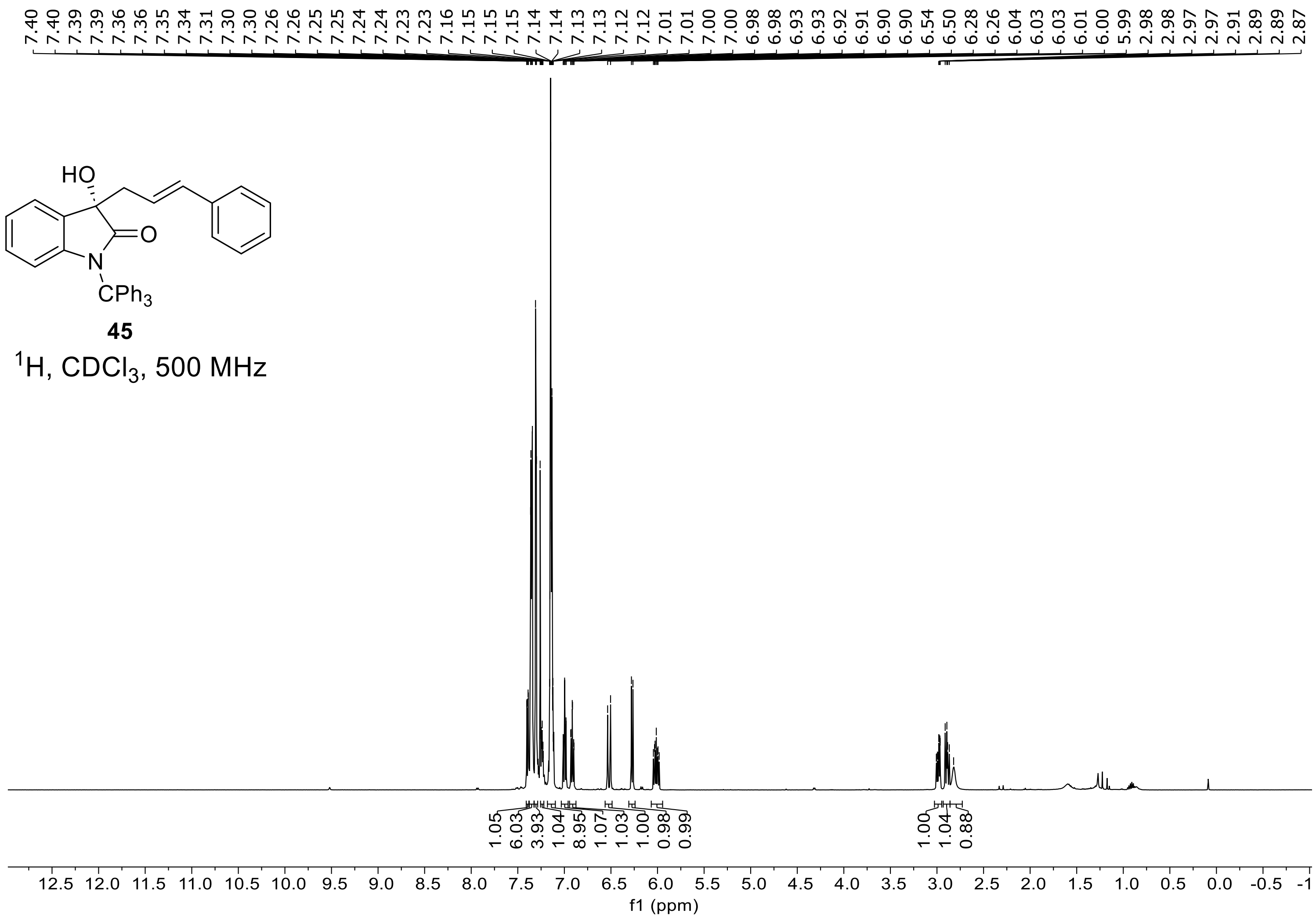

Fig. S403: ^{1}H NMR spectrum for (*S*,*E*)-3-Cinnamyl-3-hydroxy-1-tritylindolin-2-one (**45**).

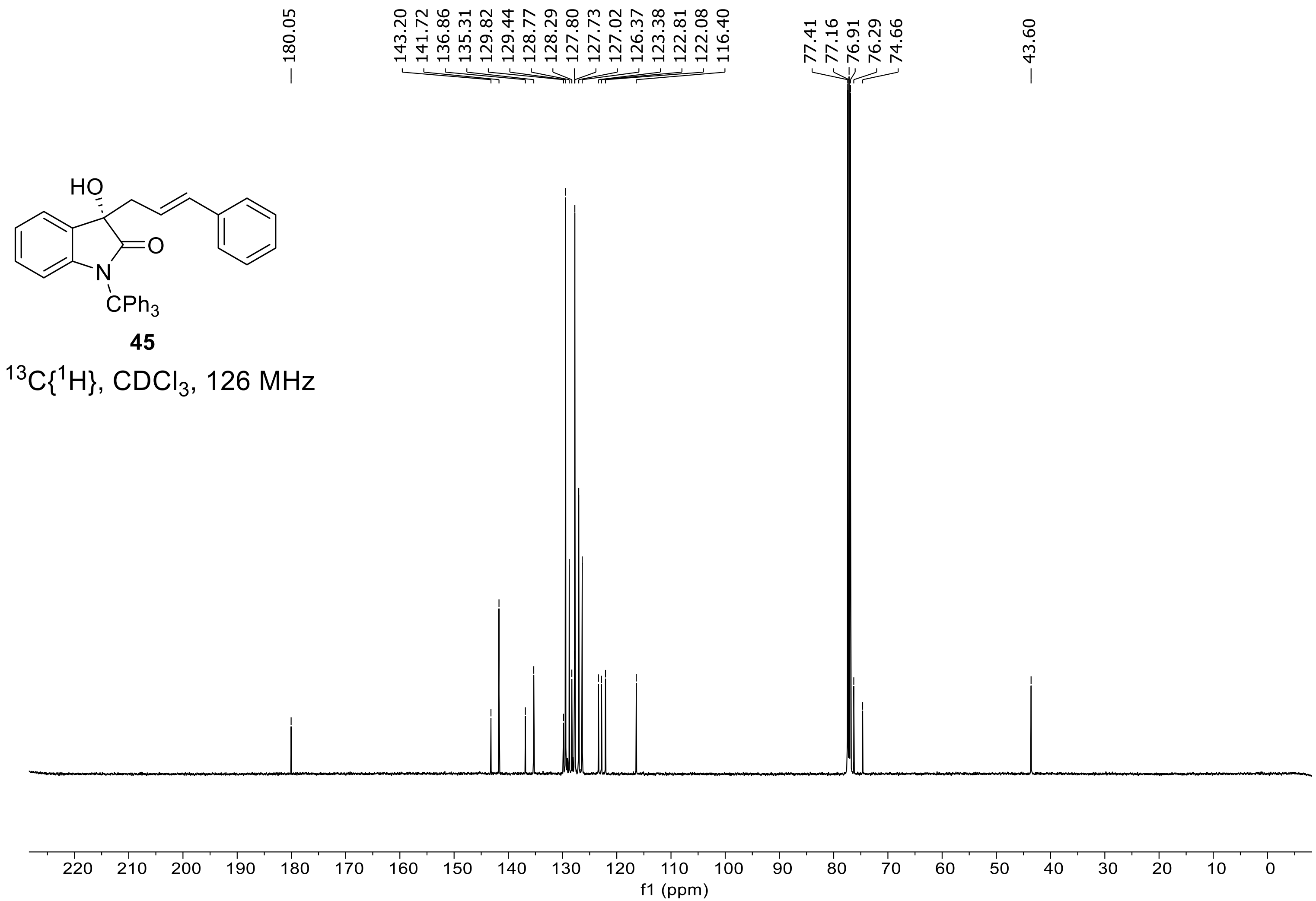

Fig. S404: $^{13}C\{^1H\}$ NMR spectrum for (*S*,*E*)-3-Cinnamyl-3-hydroxy-1-tritylindolin-2-one (**45**).

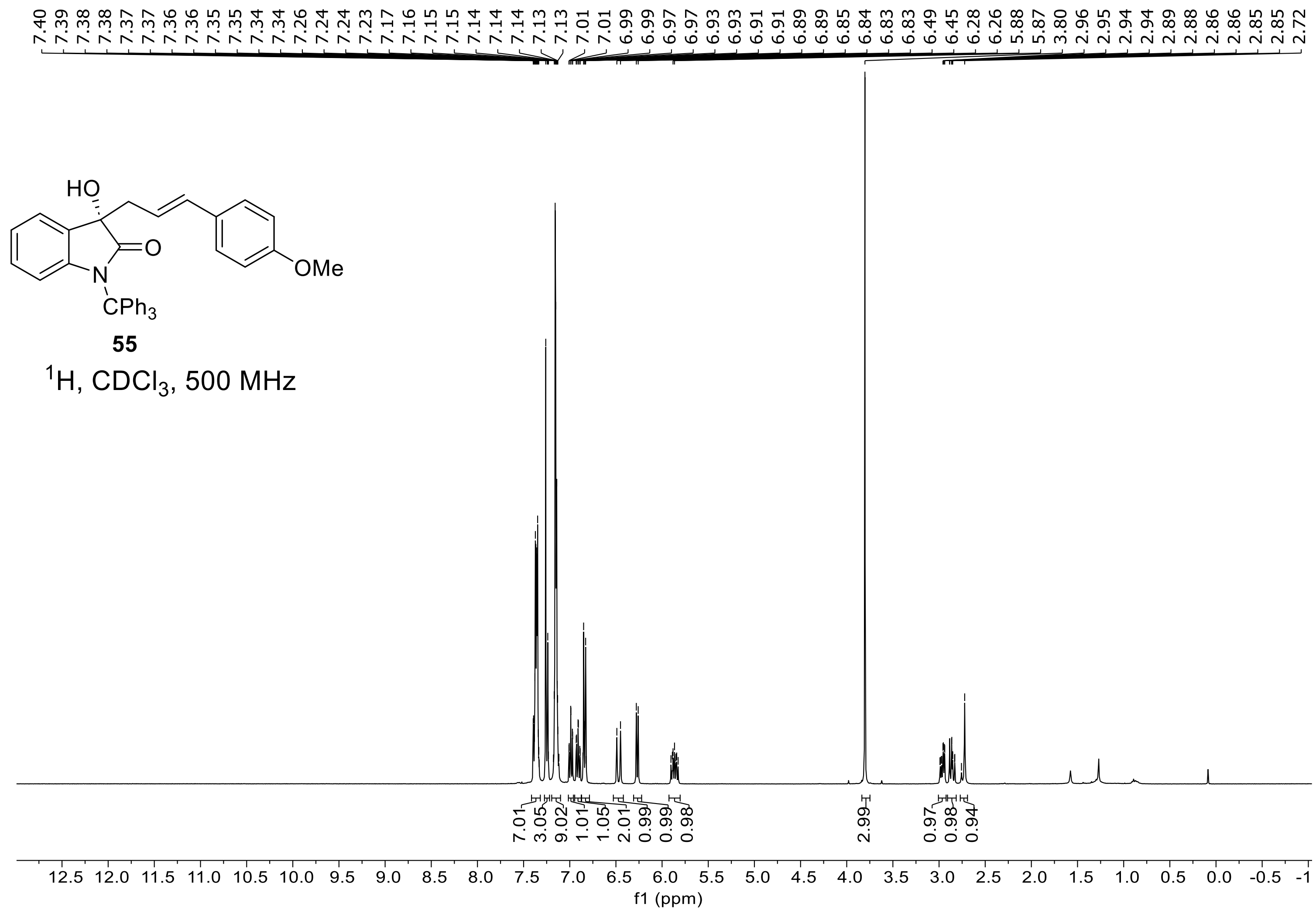

Fig. S405: ^{1}H NMR spectrum for (*S*,*E*)-3-Hydroxy-3-[3-(4-methoxyphenyl)allyl]-1-tritylindolin-2-one (**55**).

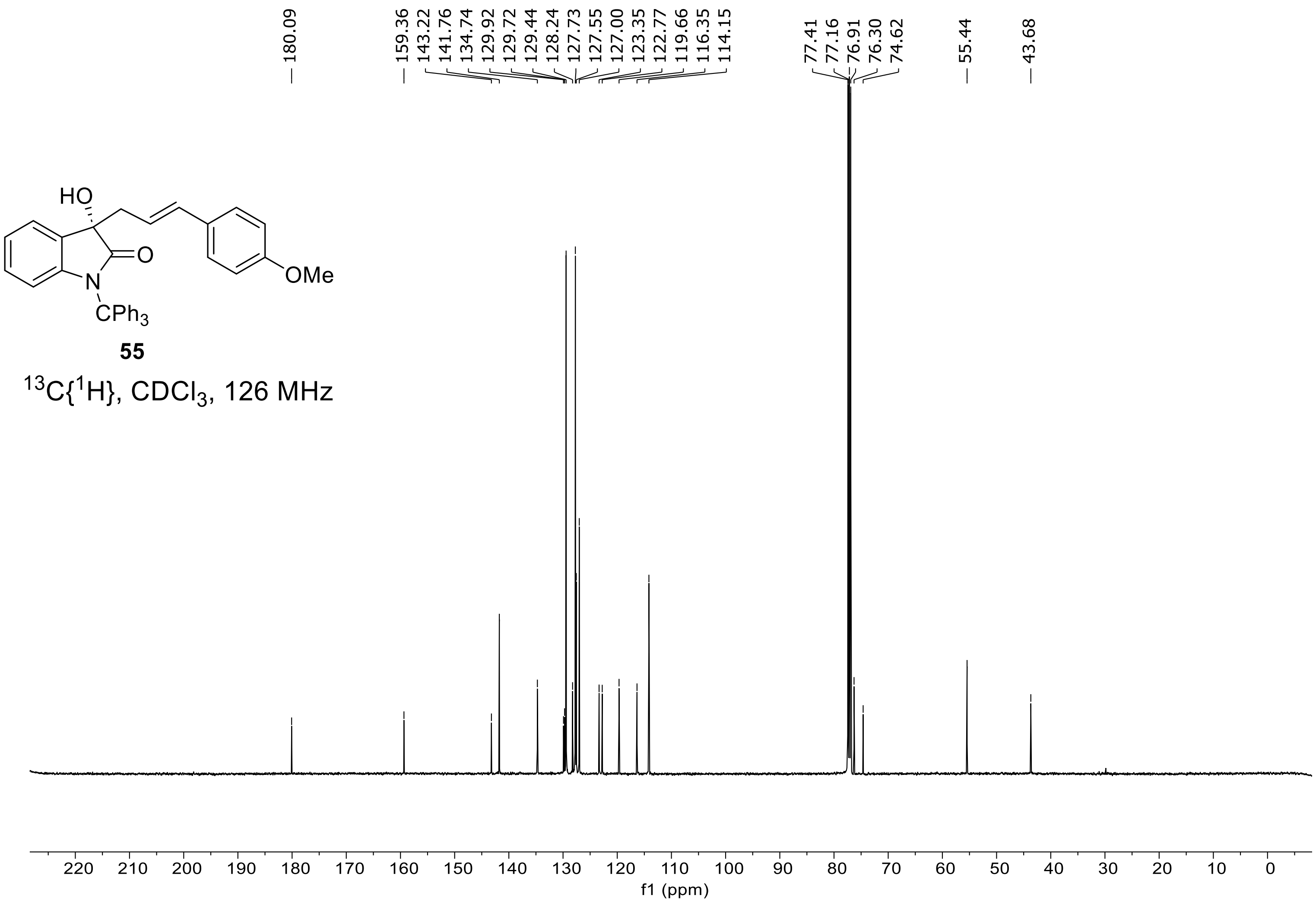

Fig. S406: $^{13}C\{^{1}H\}$ NMR spectrum for (*S*,*E*)-3-Hydroxy-3-[3-(4-methoxyphenyl)allyl]-1-tritylindolin-2-one (**55**).

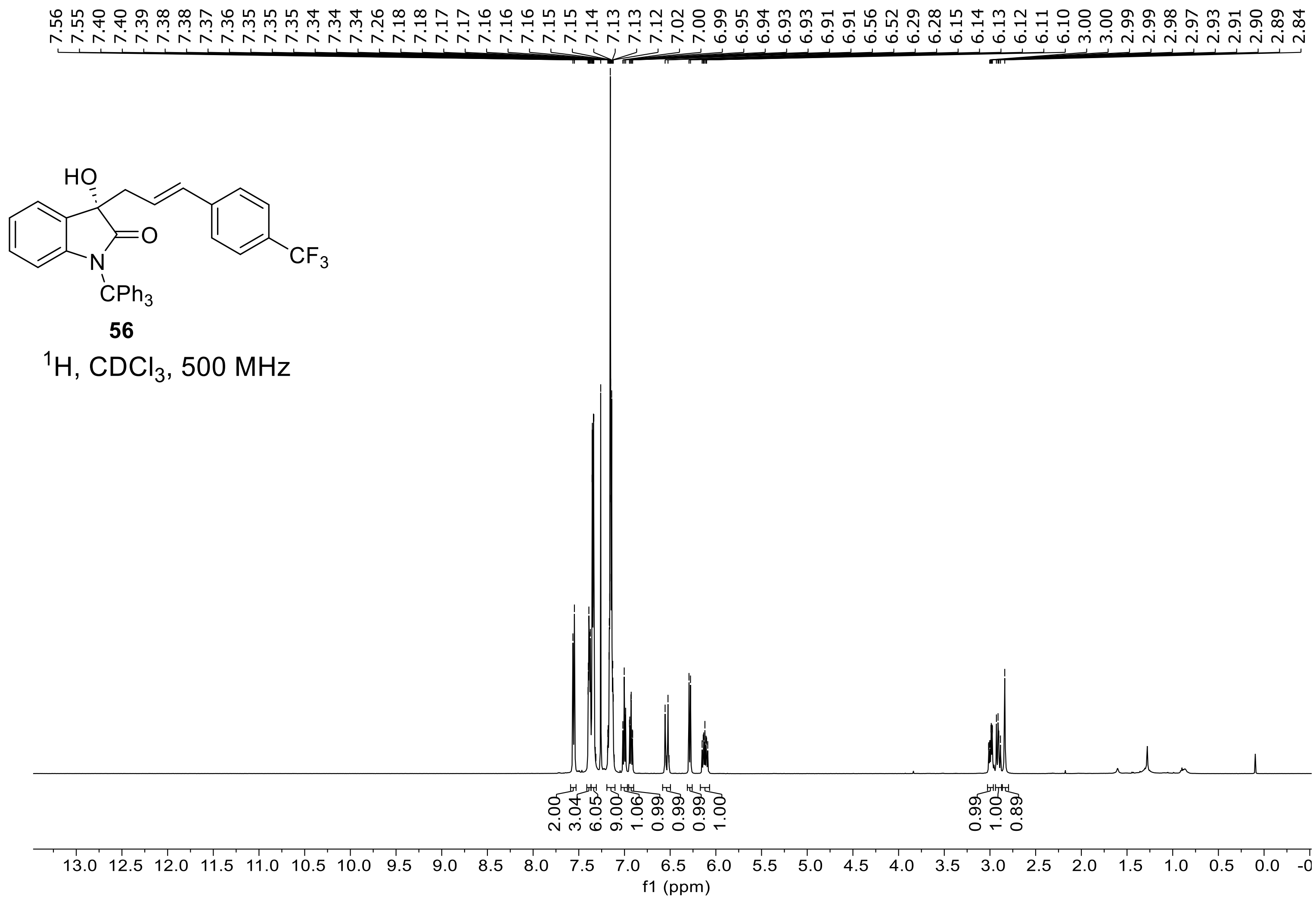

Fig. S407: ^{1}H NMR spectrum for (*S*,*E*)-3-Hydroxy-3-{3-[4-(trifluoromethyl)phenyl]allyl}-1-tritylindolin-2-one (**56**).

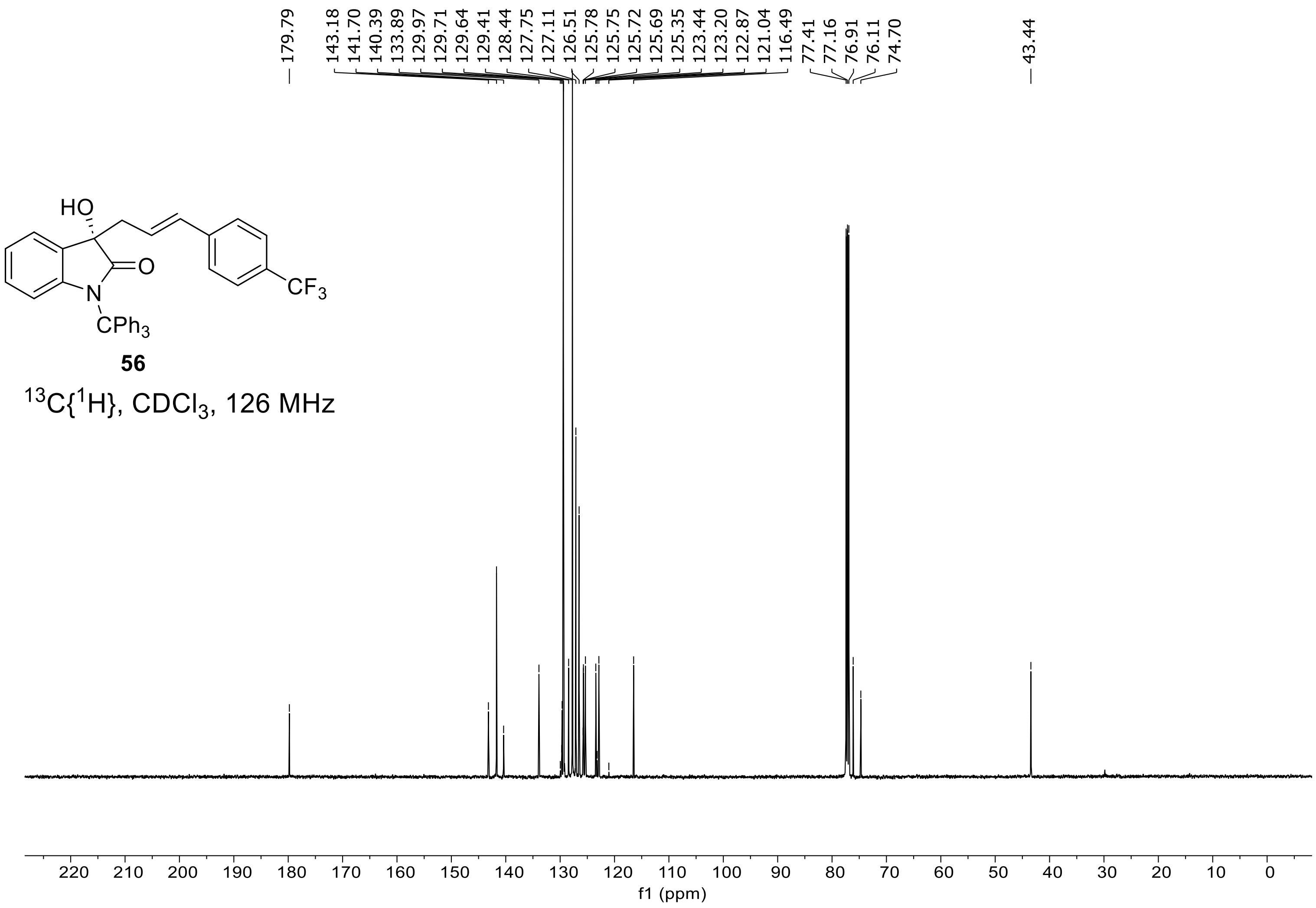

Fig. S408: $^{13}C\{^{1}H\}$ NMR spectrum for (*S*,*E*)-3-Hydroxy-3-{3-[4-(trifluoromethyl)phenyl]allyl}-1-tritylindolin-2-one (**56**).

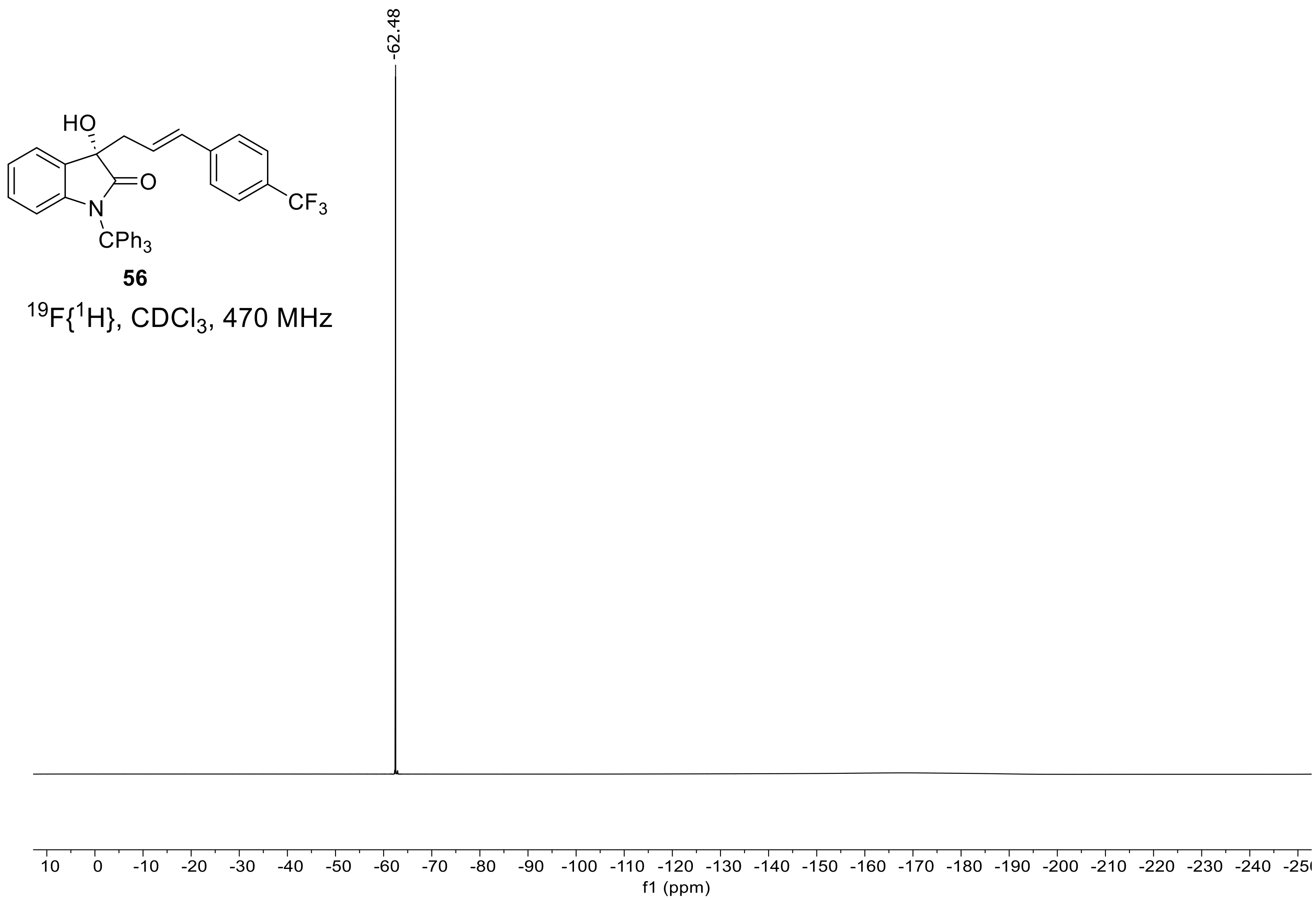

Fig. S409: $^{19}F\{^{1}H\}$ NMR spectrum for (*S*,*E*)-3-Hydroxy-3-{3-[4-(trifluoromethyl)phenyl]allyl}-1-tritylindolin-2-one (**56**).

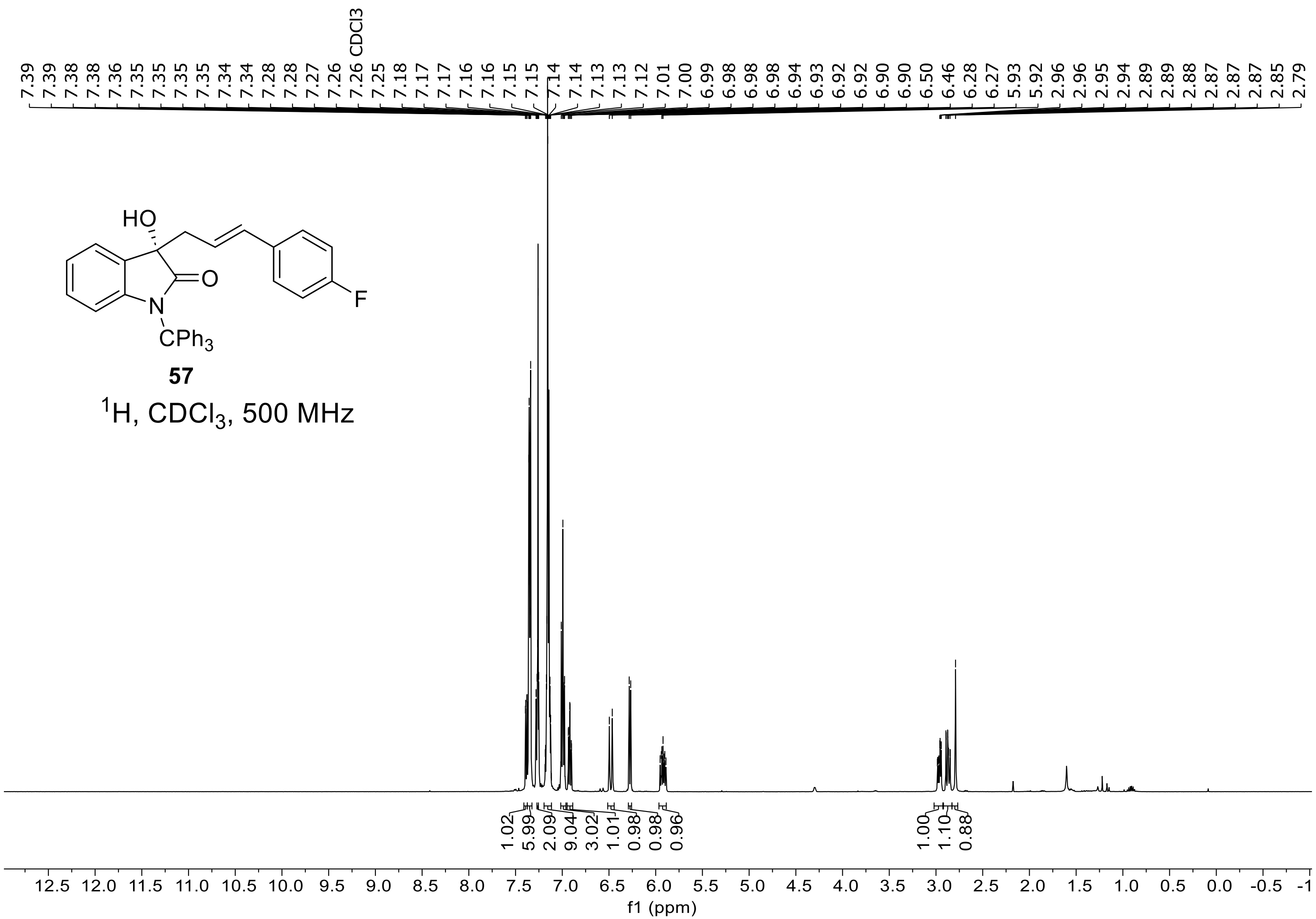

Fig. S410: ^{1}H NMR spectrum for (*S*,*E*)-3-(3-(4-fluorophenyl)allyl)-3-hydroxy-1-tritylindolin-2-one (**57**).

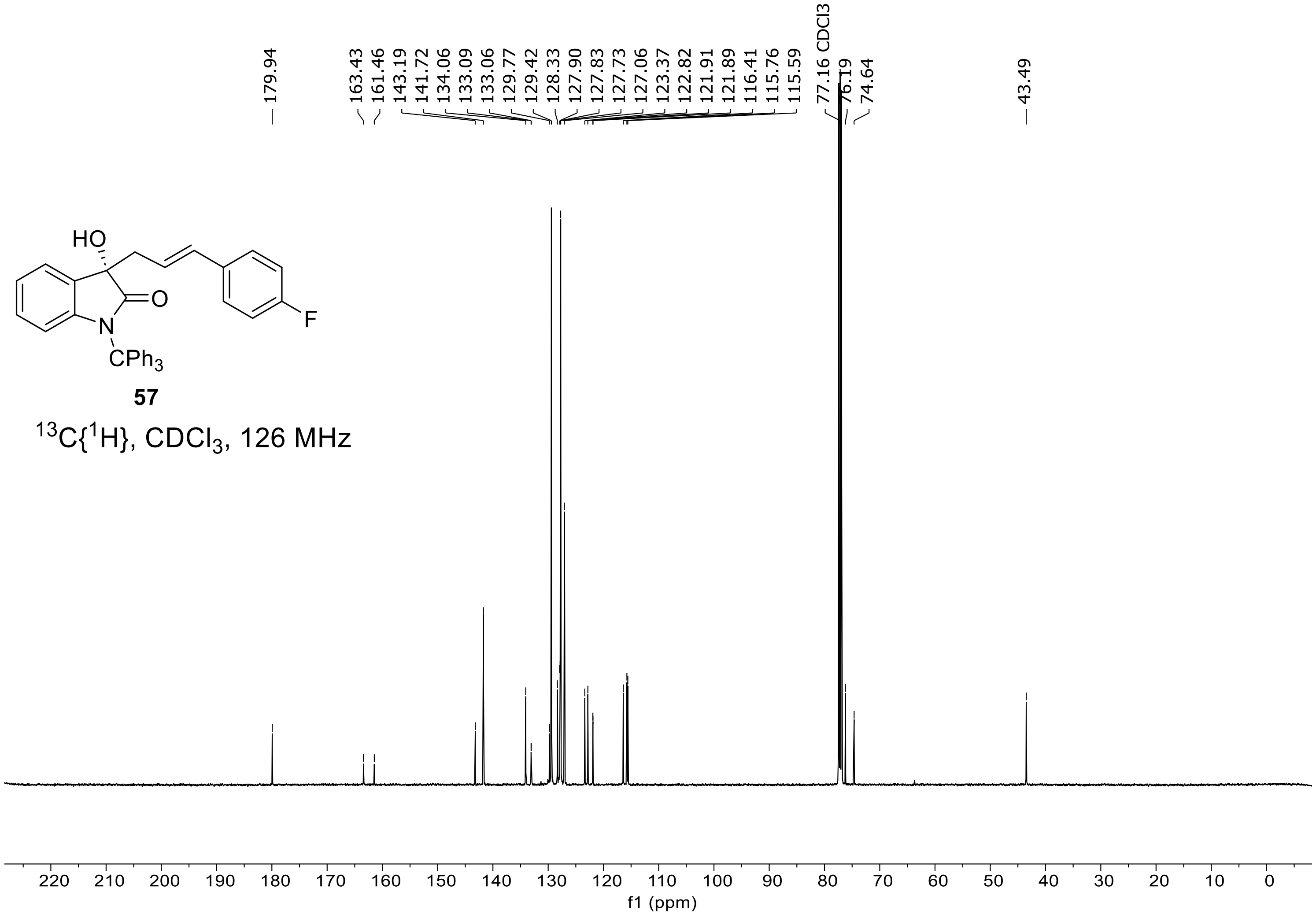

Fig. S411: $^{13}C\{^1H\}$ NMR spectrum for (*S*,*E*)-3-(3-(4-fluorophenyl)allyl)-3-hydroxy-1-tritylindolin-2-one (**57**).

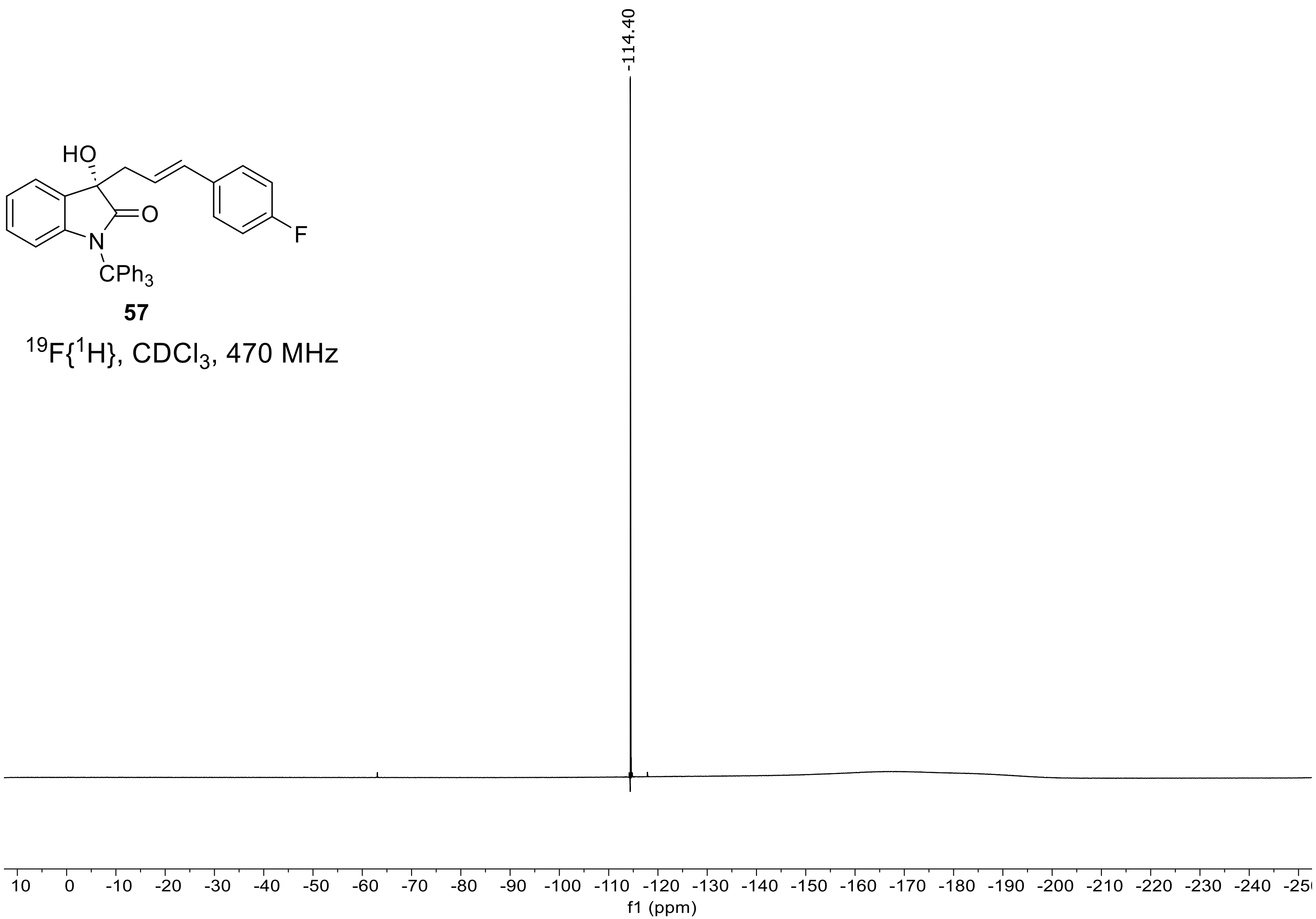

Fig. S412: $^{19}F\{^{1}H\}$ NMR spectrum for (*S*,*E*)-3-(3-(4-fluorophenyl)allyl)-3-hydroxy-1-tritylindolin-2-one (**57**).

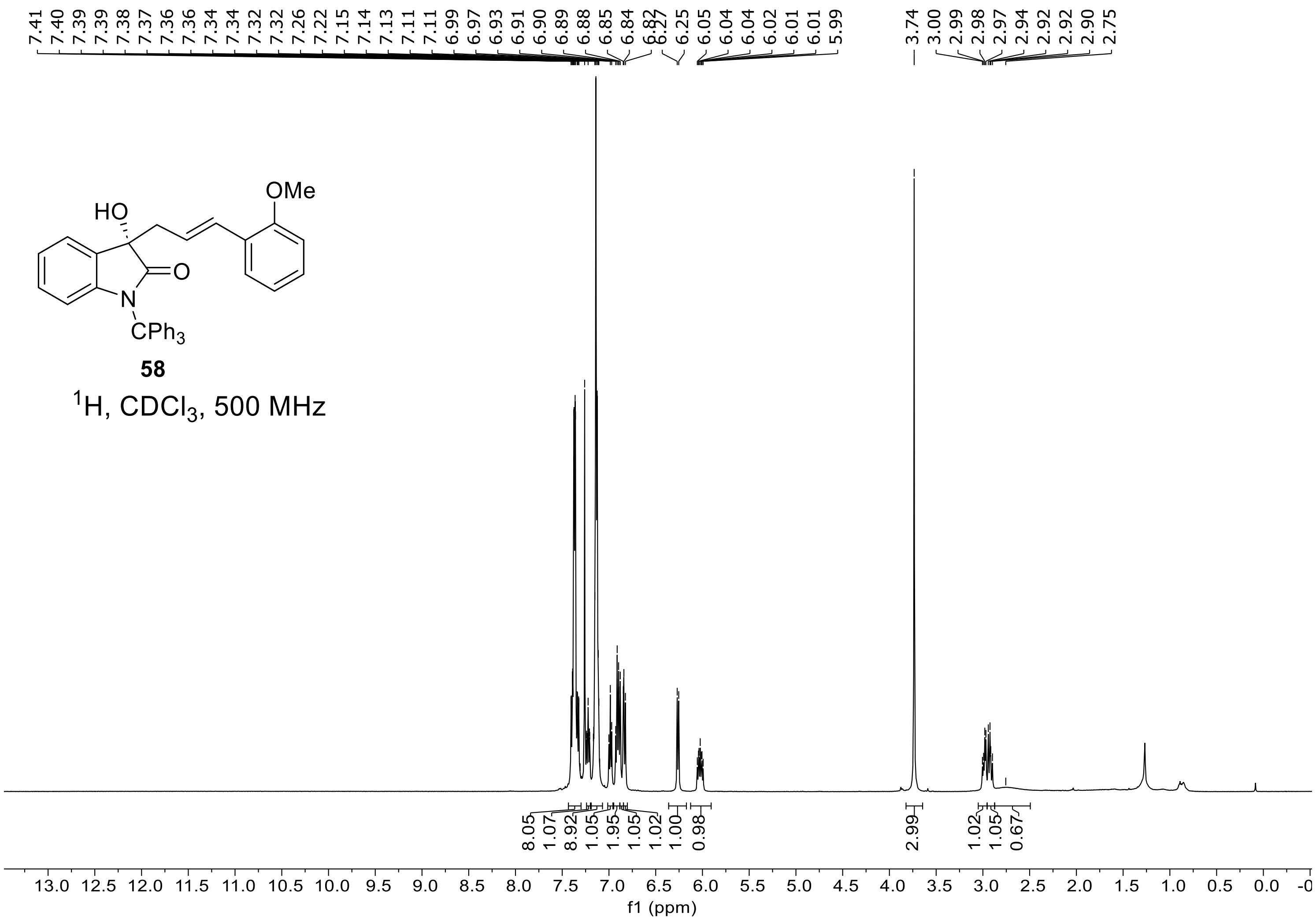

Fig. S413: ^{1}H NMR spectrum for (*S*,*E*)-3-Hydroxy-3-[3-(2-methoxyphenyl)allyl]-1-tritylindolin-2-one (**58**).

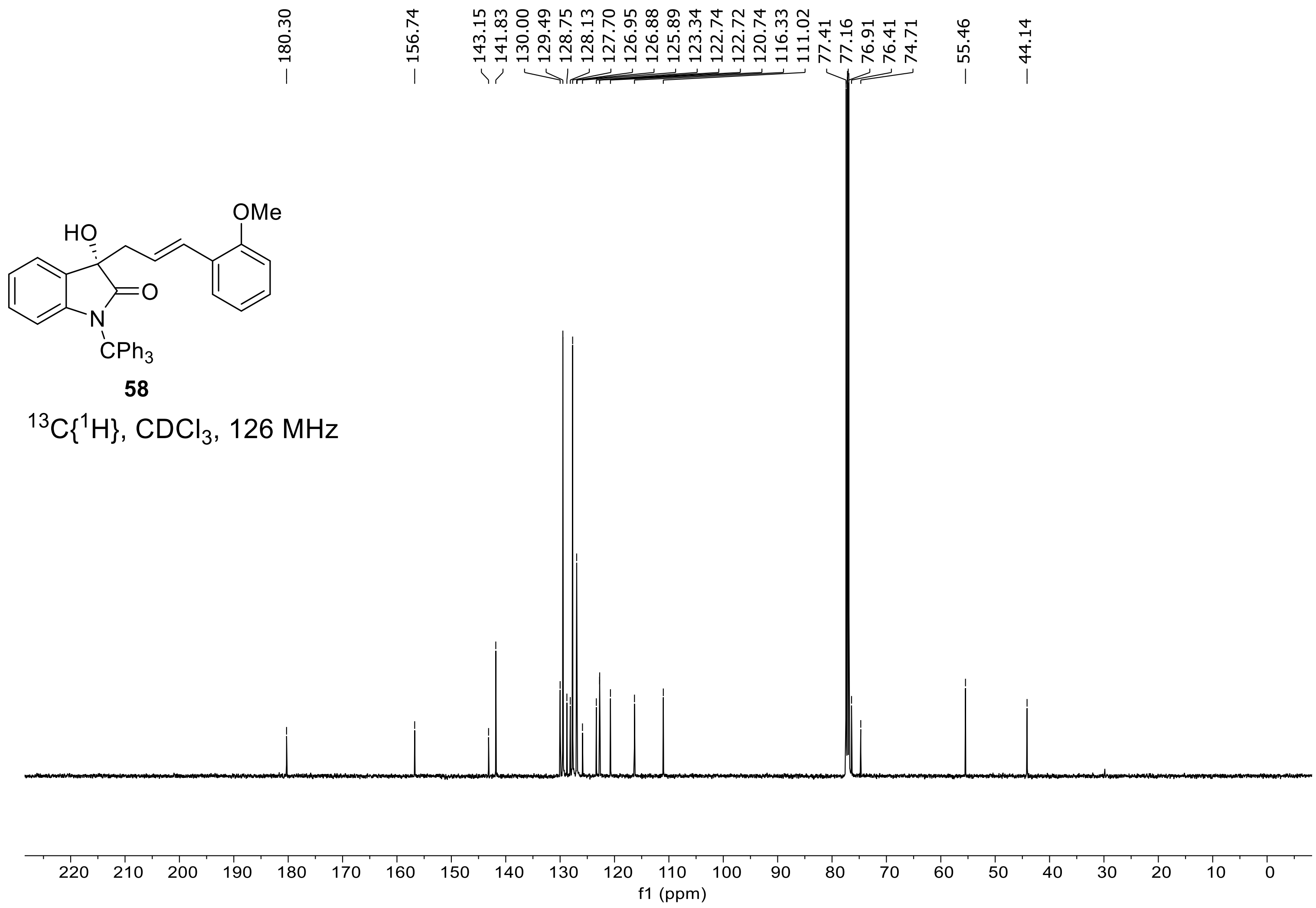

Fig. S414: $^{13}C\{^1H\}$ NMR spectrum for (*S*,*E*)-3-Hydroxy-3-[3-(2-methoxyphenyl)allyl]-1-tritylindolin-2-one (**58**).

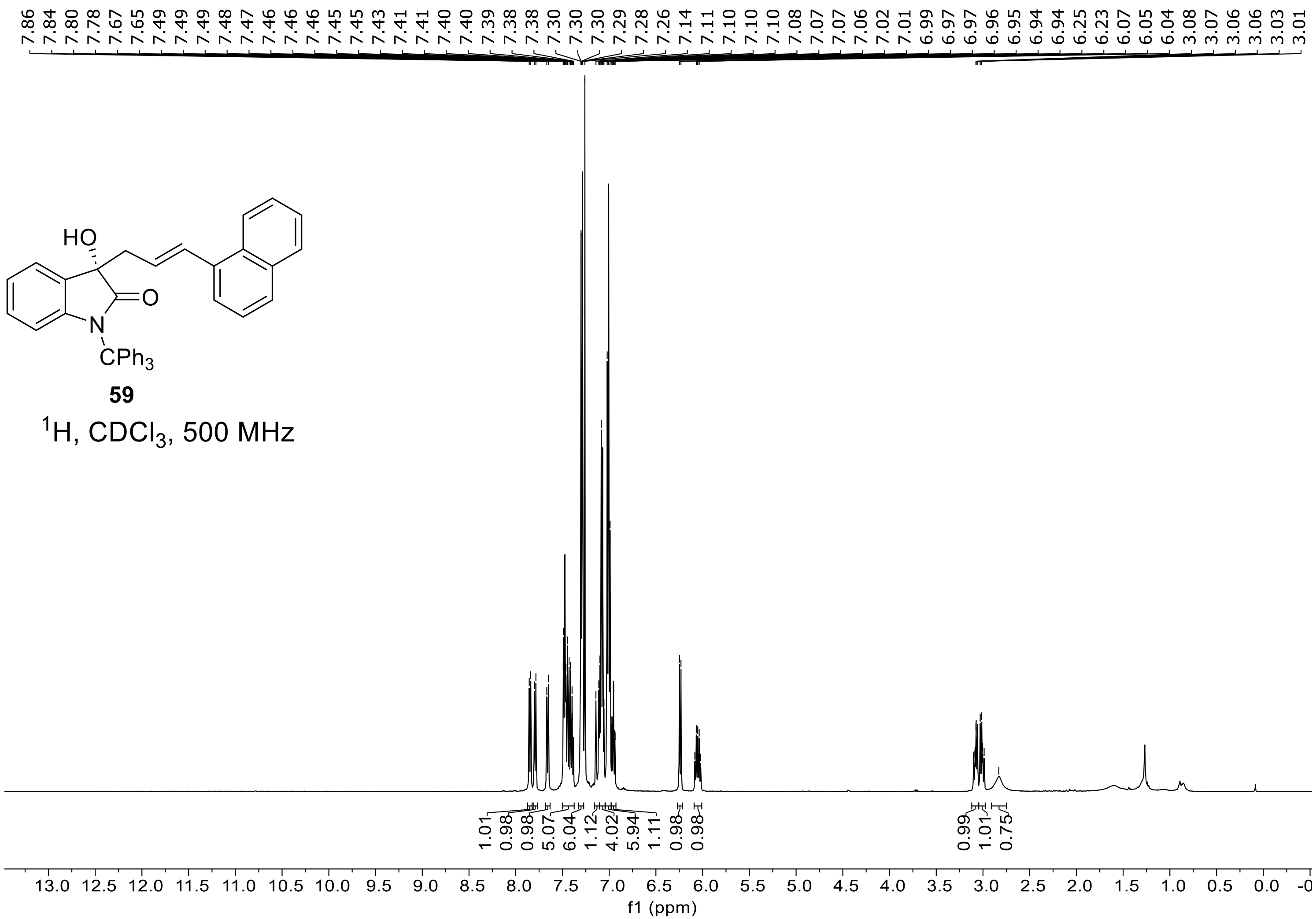

Fig. S415: ^{1}H NMR spectrum for (*S*,*E*)-3-Hydroxy-3-[3-(1-naphthyl)allyl]-1-tritylindolin-2-one (**59**).

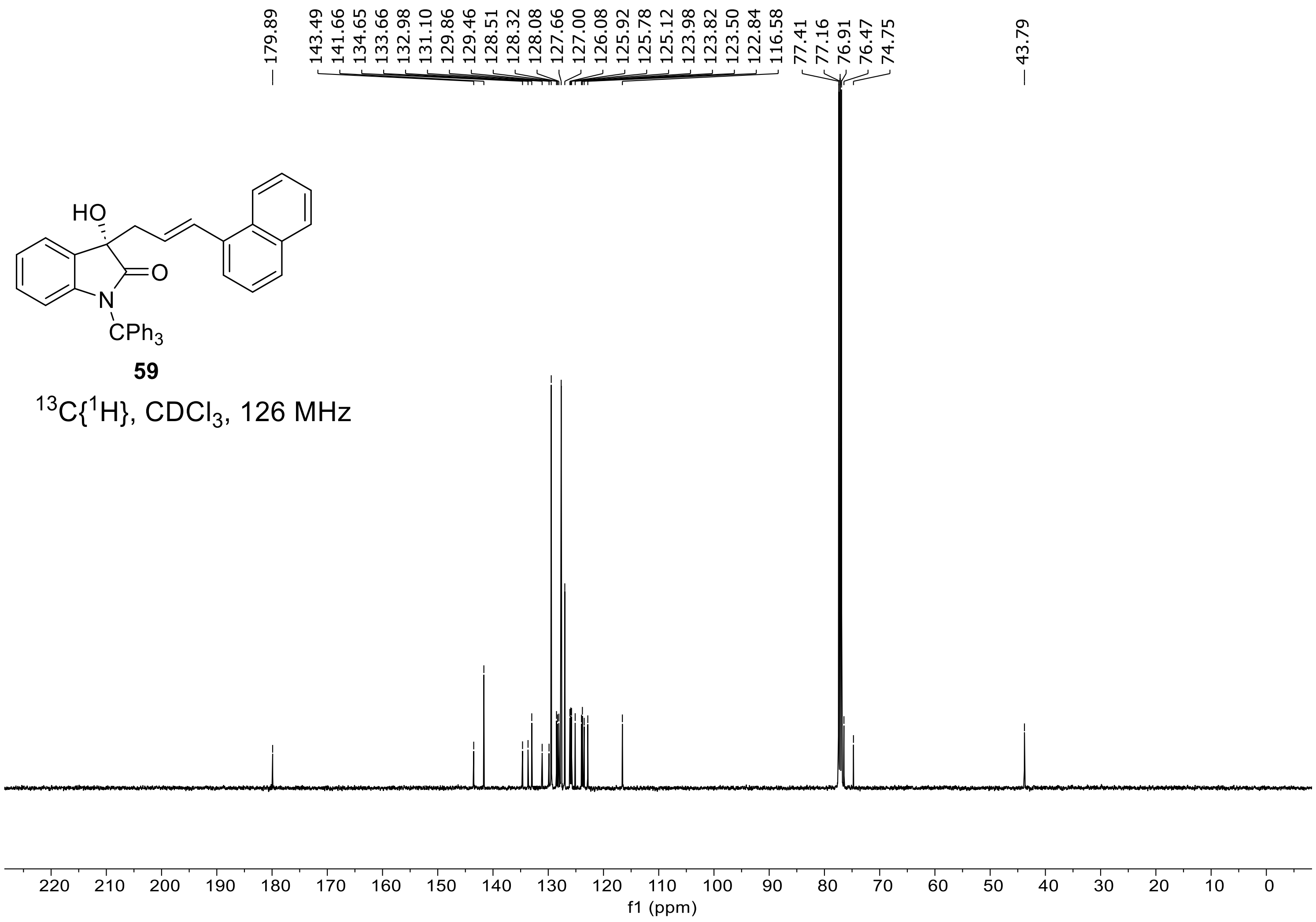

Fig. S416: $^{13}C\{^{1}H\}$ NMR spectrum for (*S*,*E*)-3-Hydroxy-3-[3-(1-naphthyl)allyl]-1-tritylindolin-2-one (**59**).

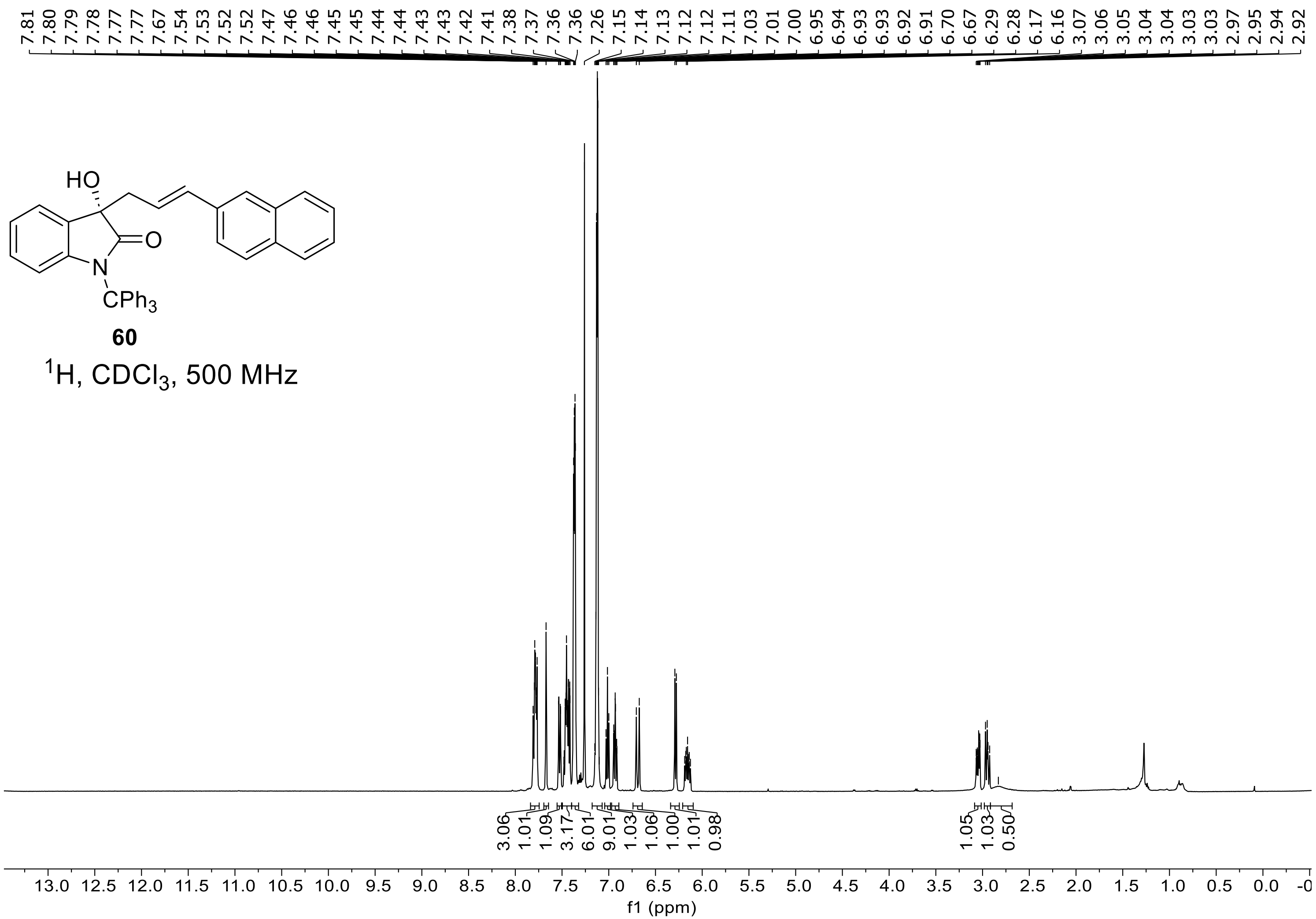

Fig. S417: ^{1}H NMR spectrum for (*S*,*E*)-3-Hydroxy-3-[3-(2-naphthyl)allyl]-1-tritylindolin-2-one (**60**).

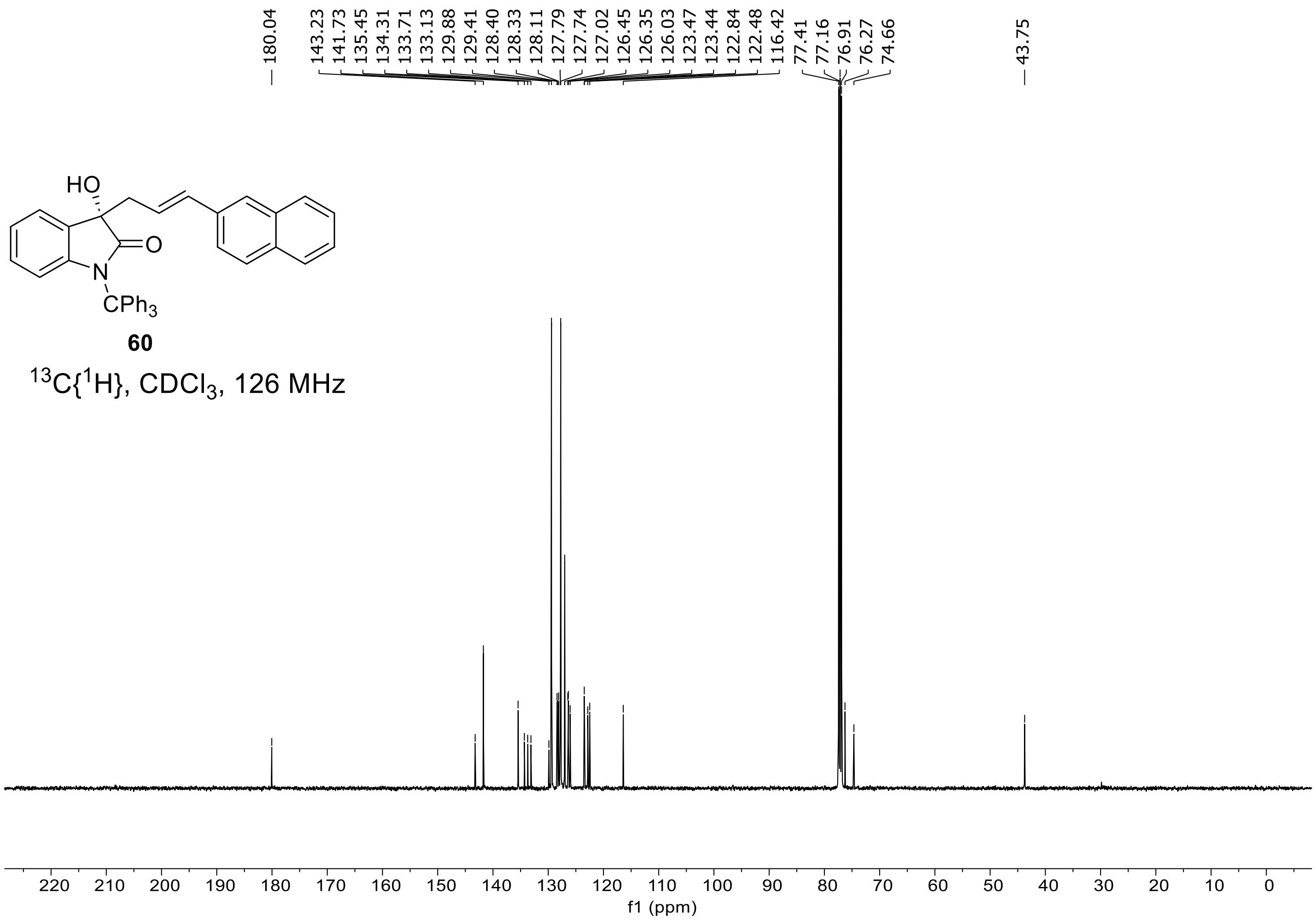

Fig. S418: $^{13}C\{^1H\}$ NMR spectrum for (*S*,*E*)-3-Hydroxy-3-[3-(2-naphthyl)allyl]-1-tritylindolin-2-one (**60**).

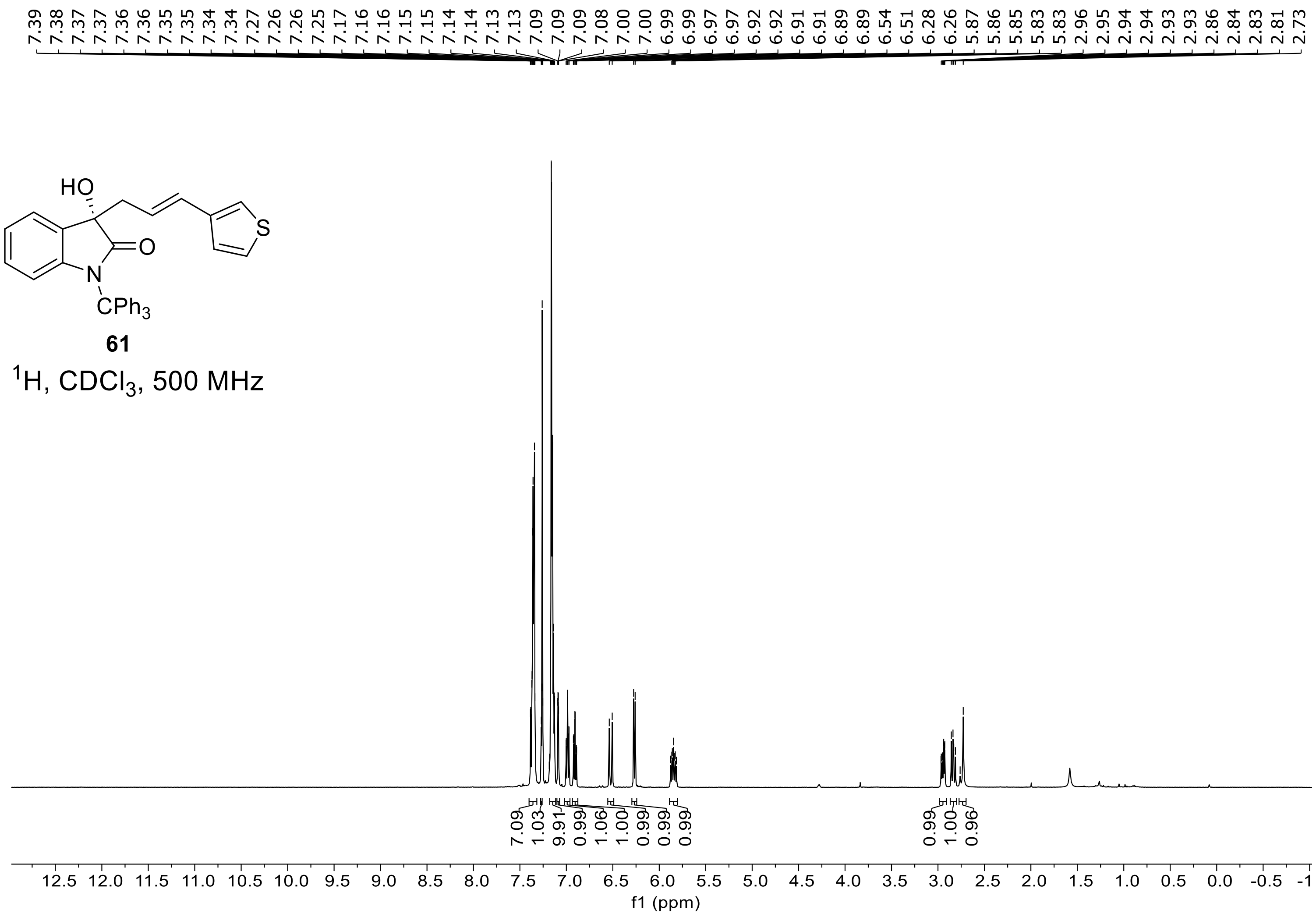

Fig. S419: ^{1}H NMR spectrum for (*S*,*E*)-3-Hydroxy-3-[3-(thiophen-3-yl)allyl]-1-tritylindolin-2-one (**61**).

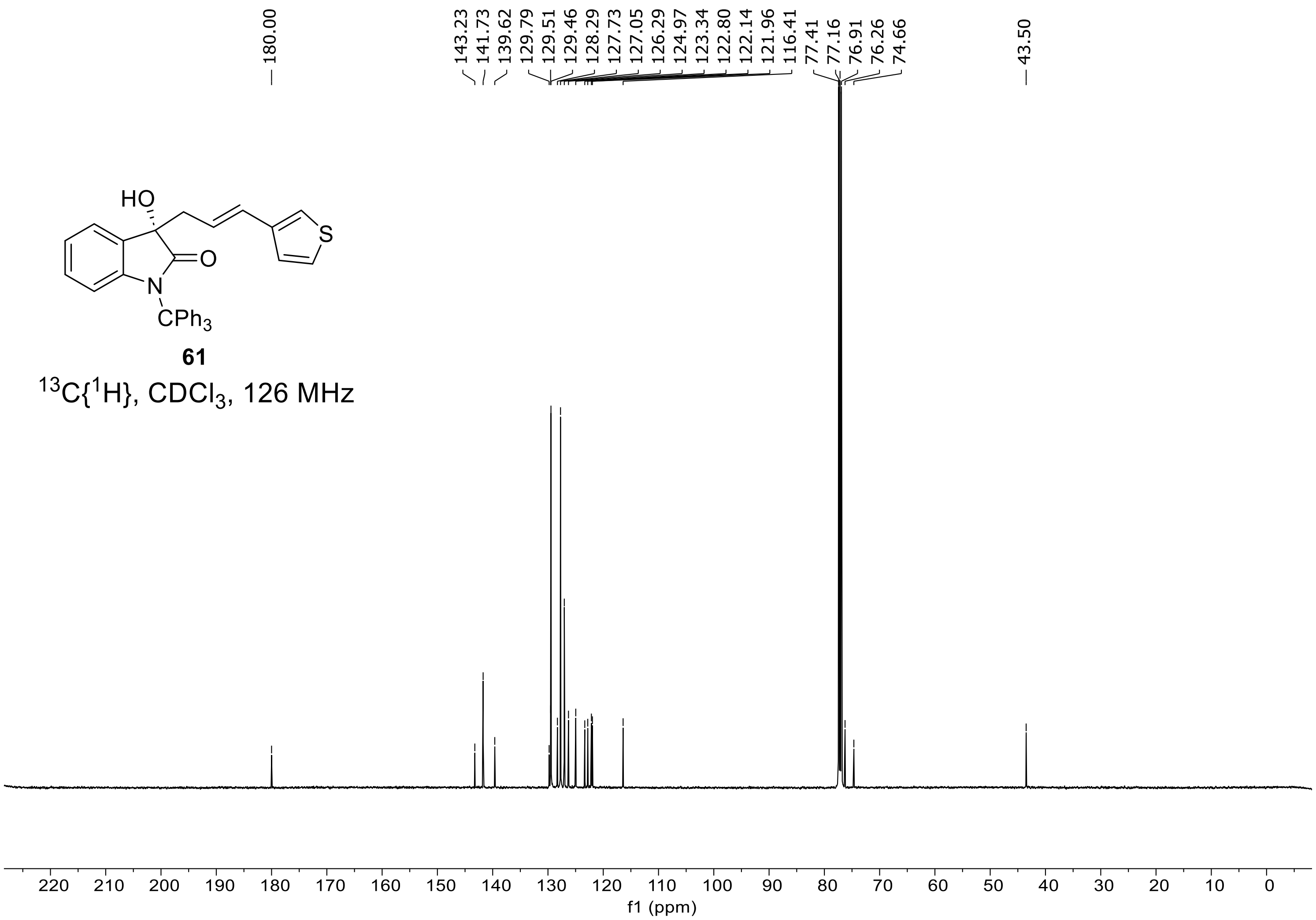

Fig. S420: $^{13}C\{^{1}H\}$ NMR spectrum for (*S*,*E*)-3-Hydroxy-3-[3-(thiophen-3-yl)allyl]-1-tritylindolin-2-one (**61**).

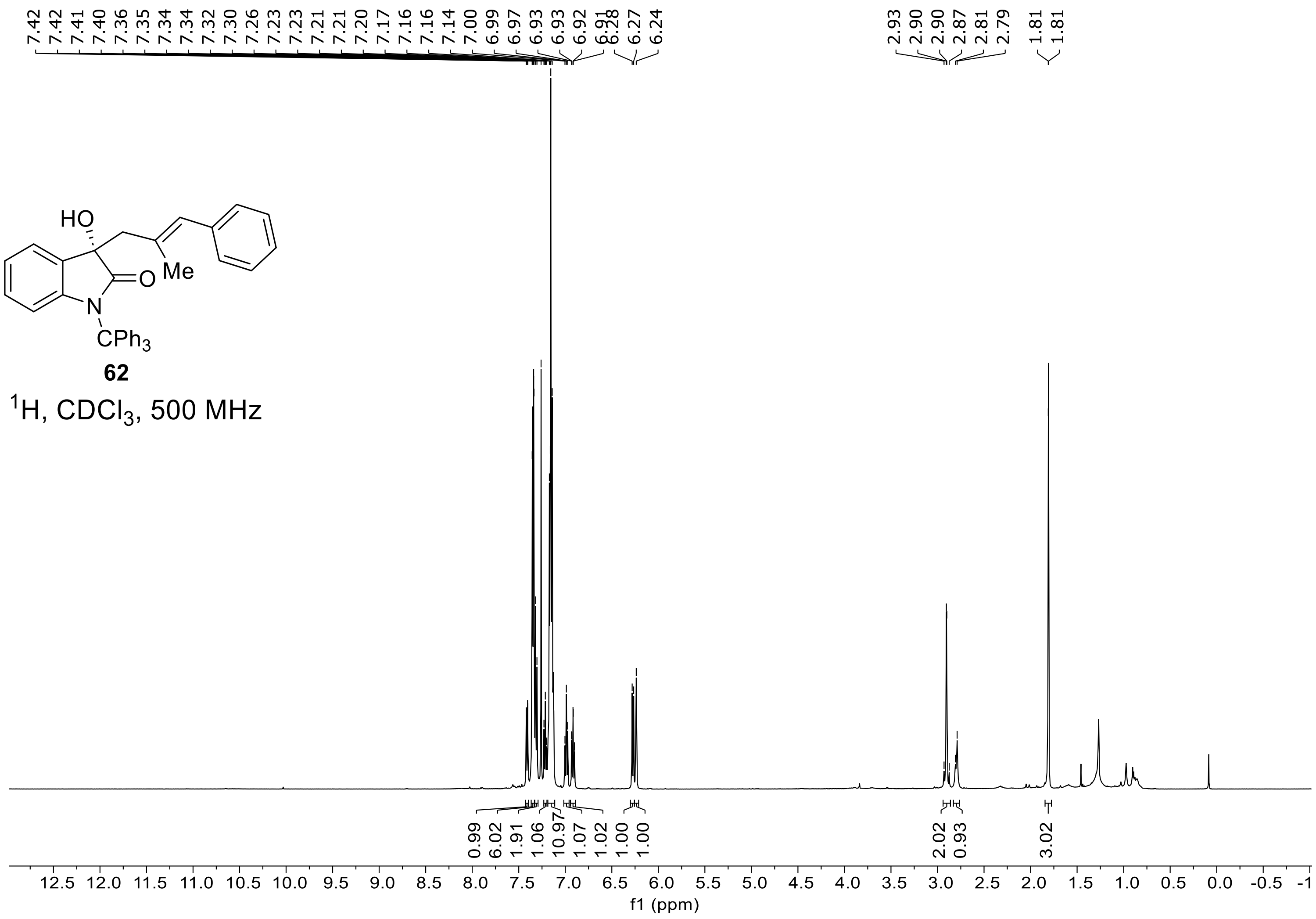

Fig. S421: ^{1}H NMR spectrum for (*S*,*E*)-3-Hydroxy-3-(2-methyl-3-phenylallyl)-1-tritylindolin-2-one (**62**).

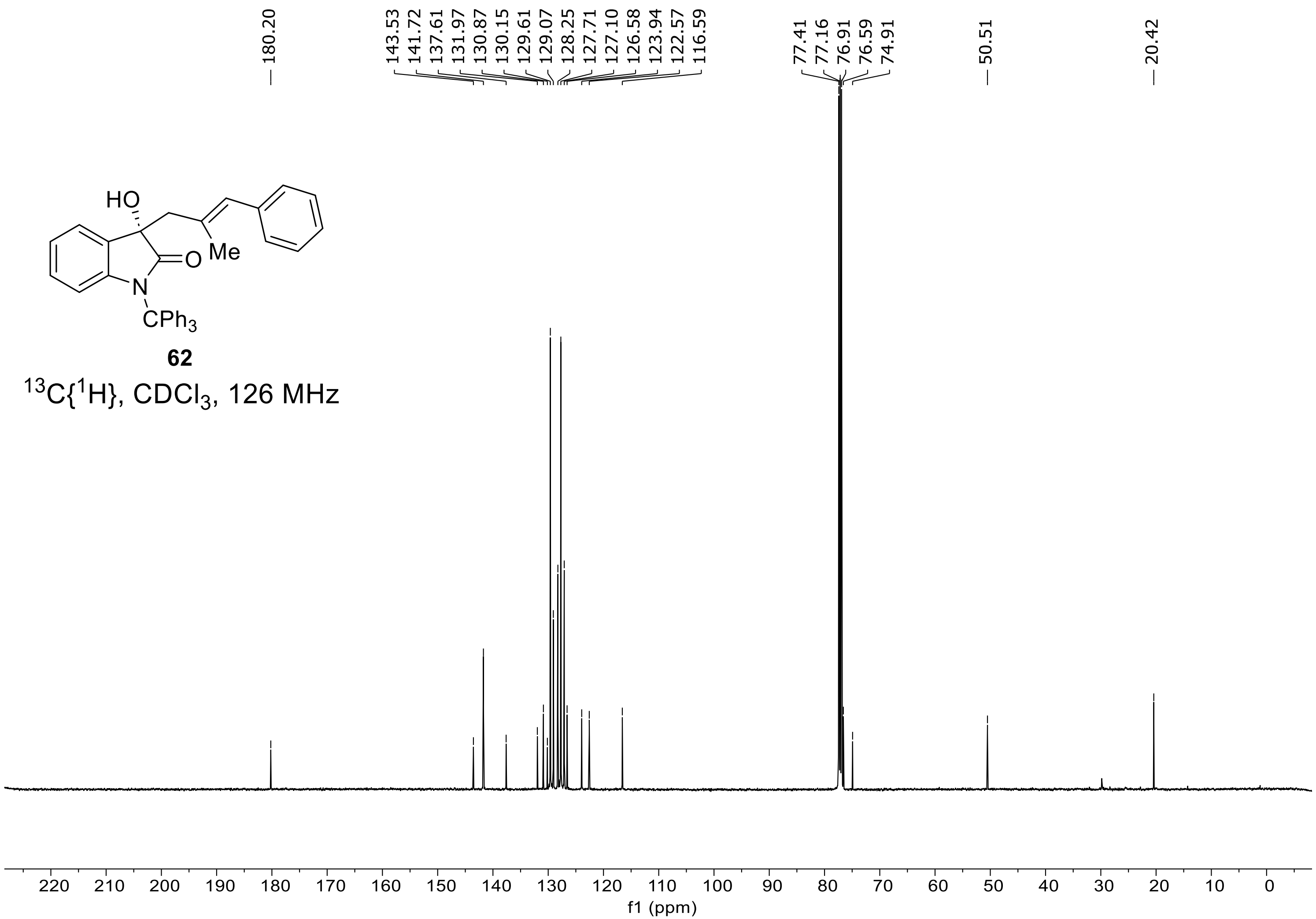

Fig. S422: $^{13}C\{^{1}H\}$ NMR spectrum for (*S*,*E*)-3-Hydroxy-3-(2-methyl-3-phenylallyl)-1-tritylindolin-2-one (**62**).

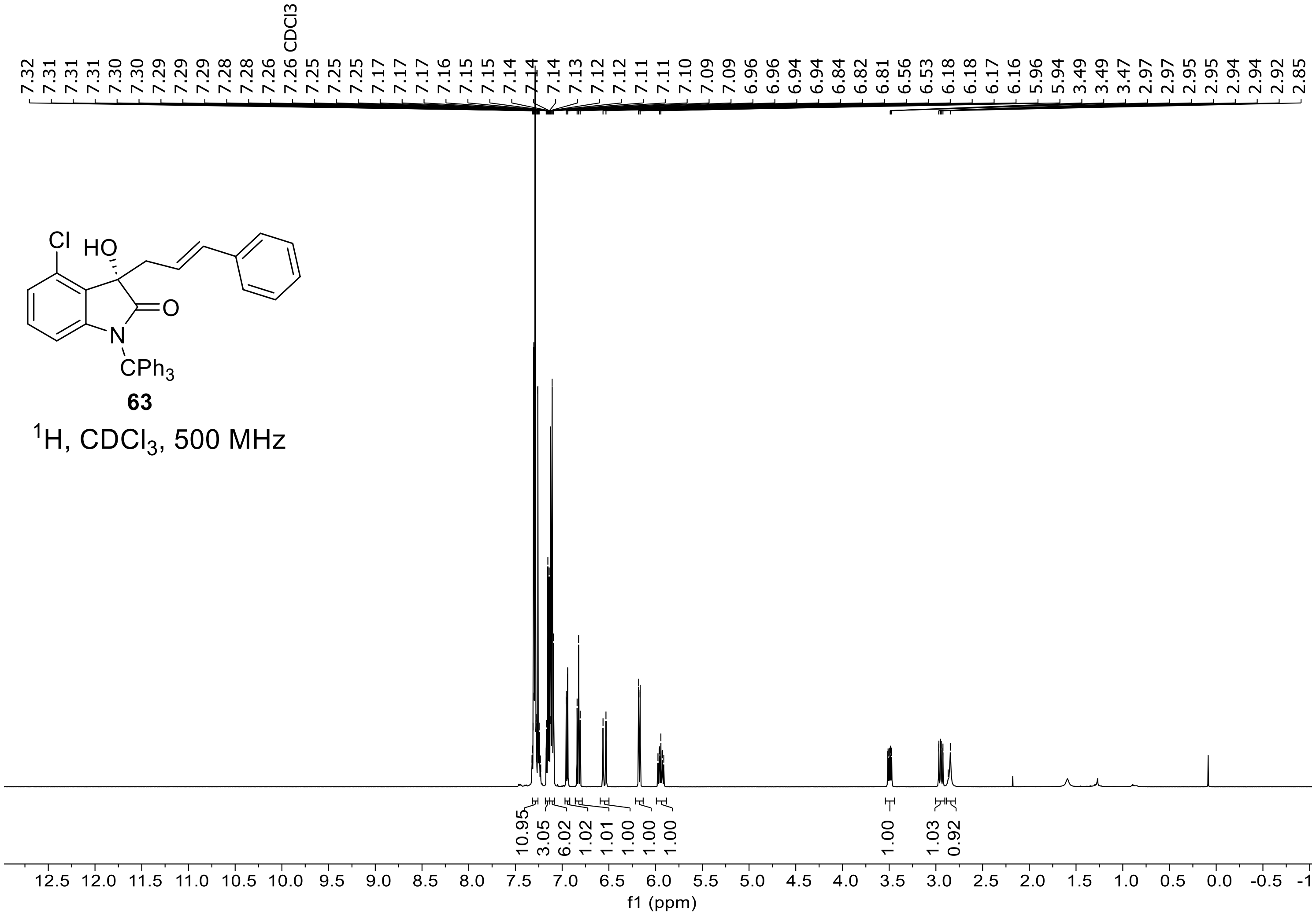

Fig. S423: ^{1}H NMR spectrum for (*S*,*E*)-4-Chloro-3-cinnamyl-3-hydroxy-1-tritylindolin-2-one (**63**).

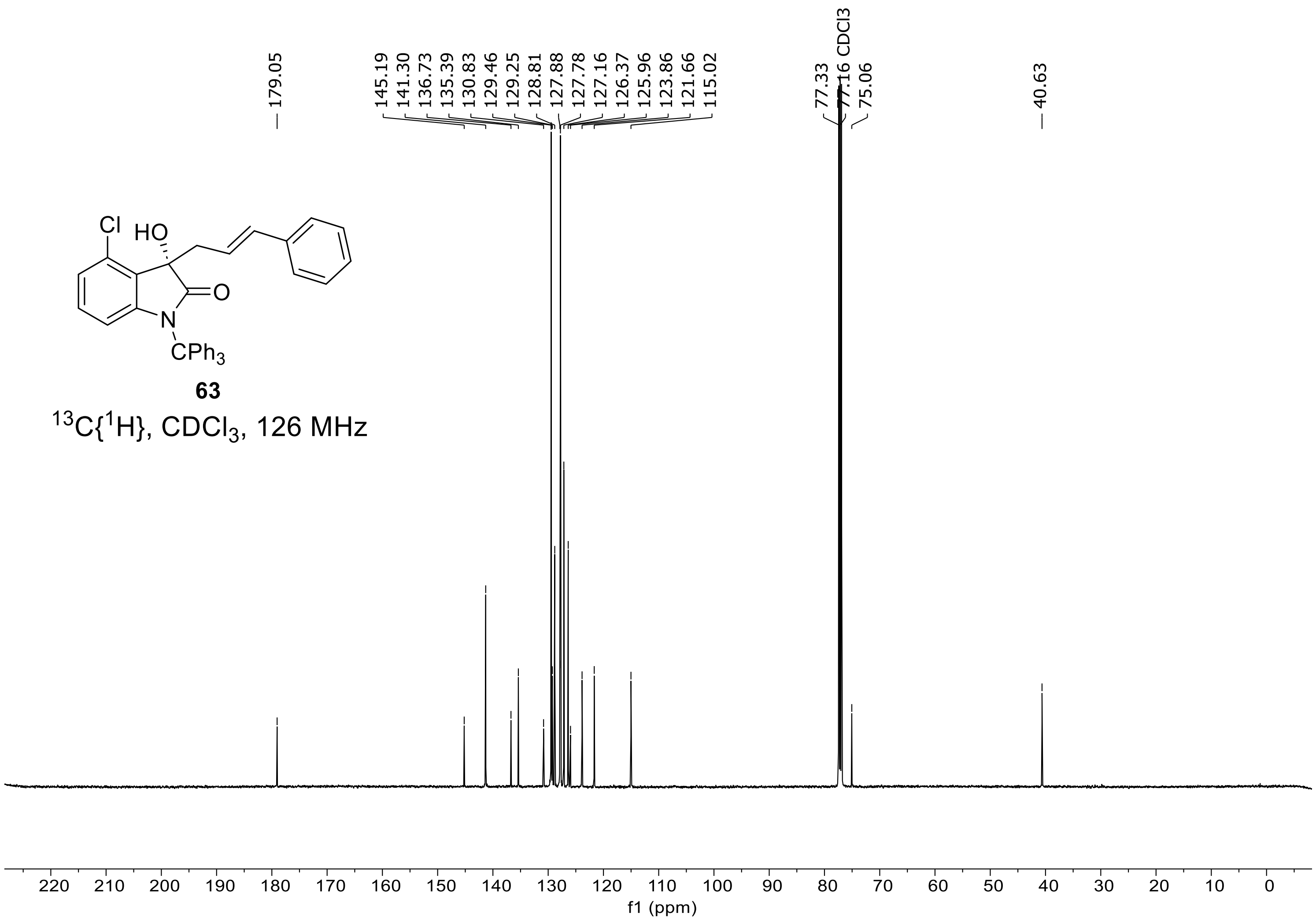

Fig. S424: $^{13}C\{^{1}H\}$ NMR spectrum for (*S*,*E*)-4-Chloro-3-cinnamyl-3-hydroxy-1-tritylindolin-2-one (**63**).

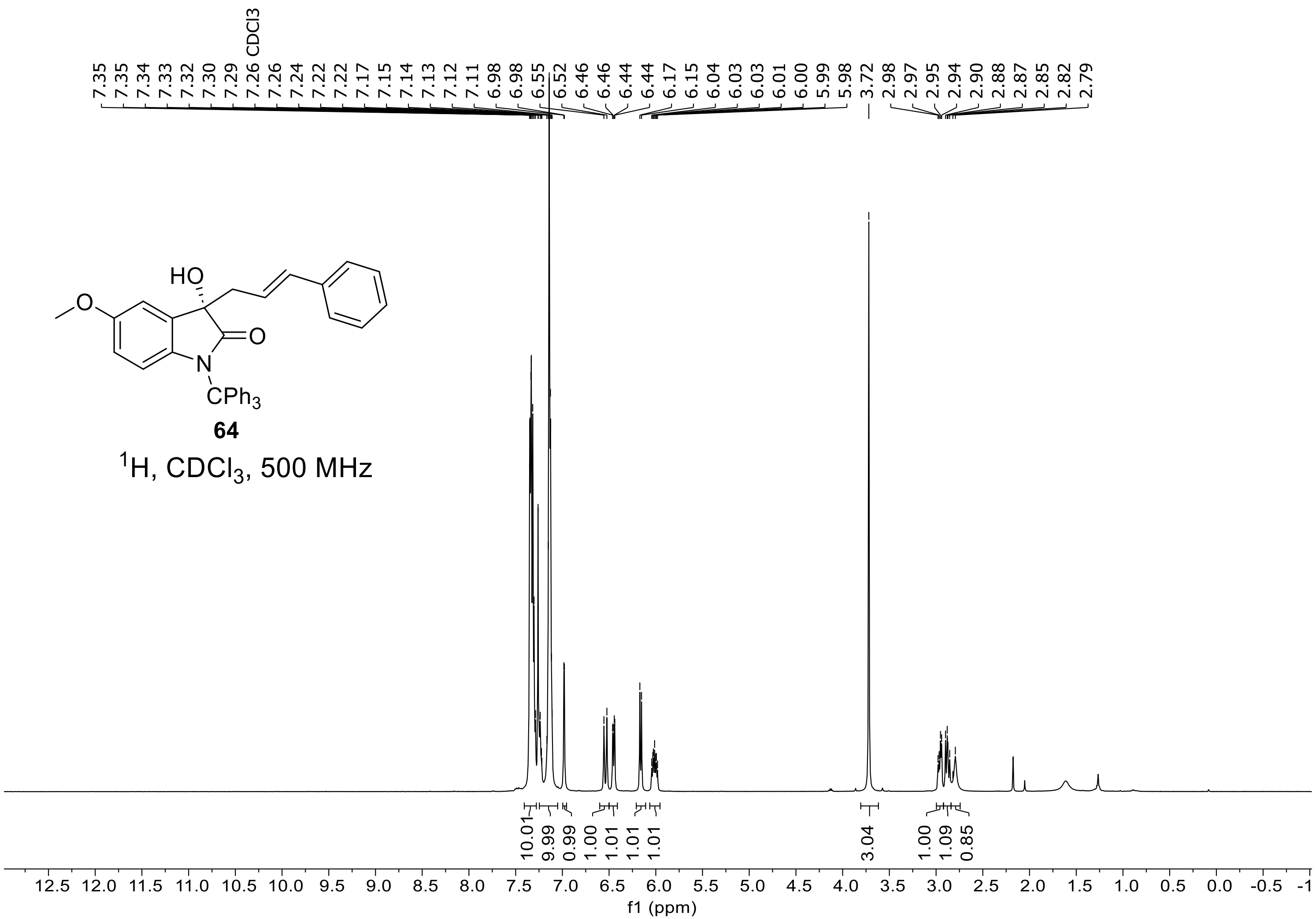

Fig. S425: ^{1}H NMR spectrum for (*S*,*E*)-3-cinnamyl-3-hydroxy-5-methoxy-1-tritylindolin-2-one (**64**).

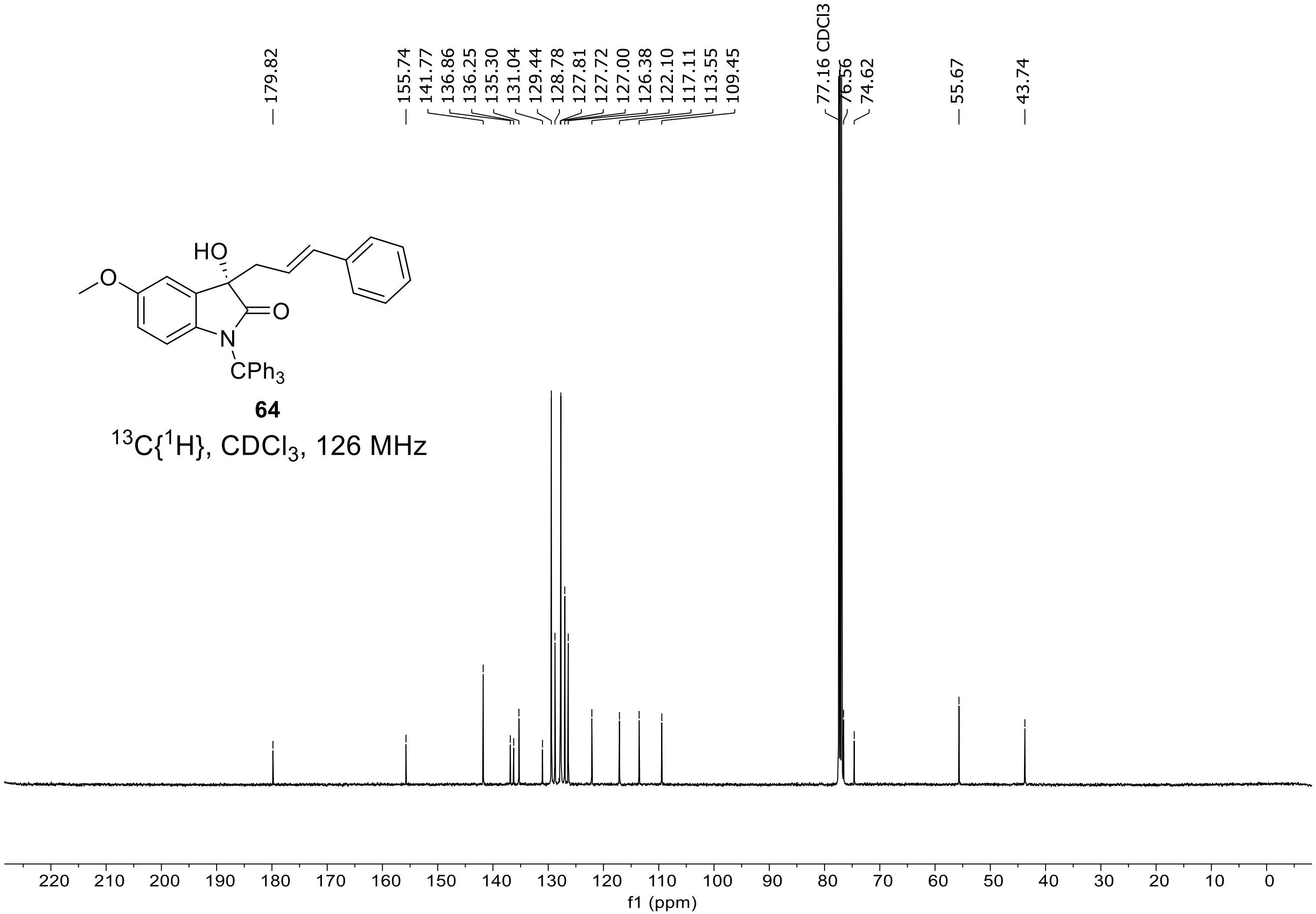

Fig. S426: $^{13}C\{^1H\}$ NMR spectrum for (*S*,*E*)-3-cinnamyl-3-hydroxy-5-methoxy-1-tritylindolin-2-one (**64**).

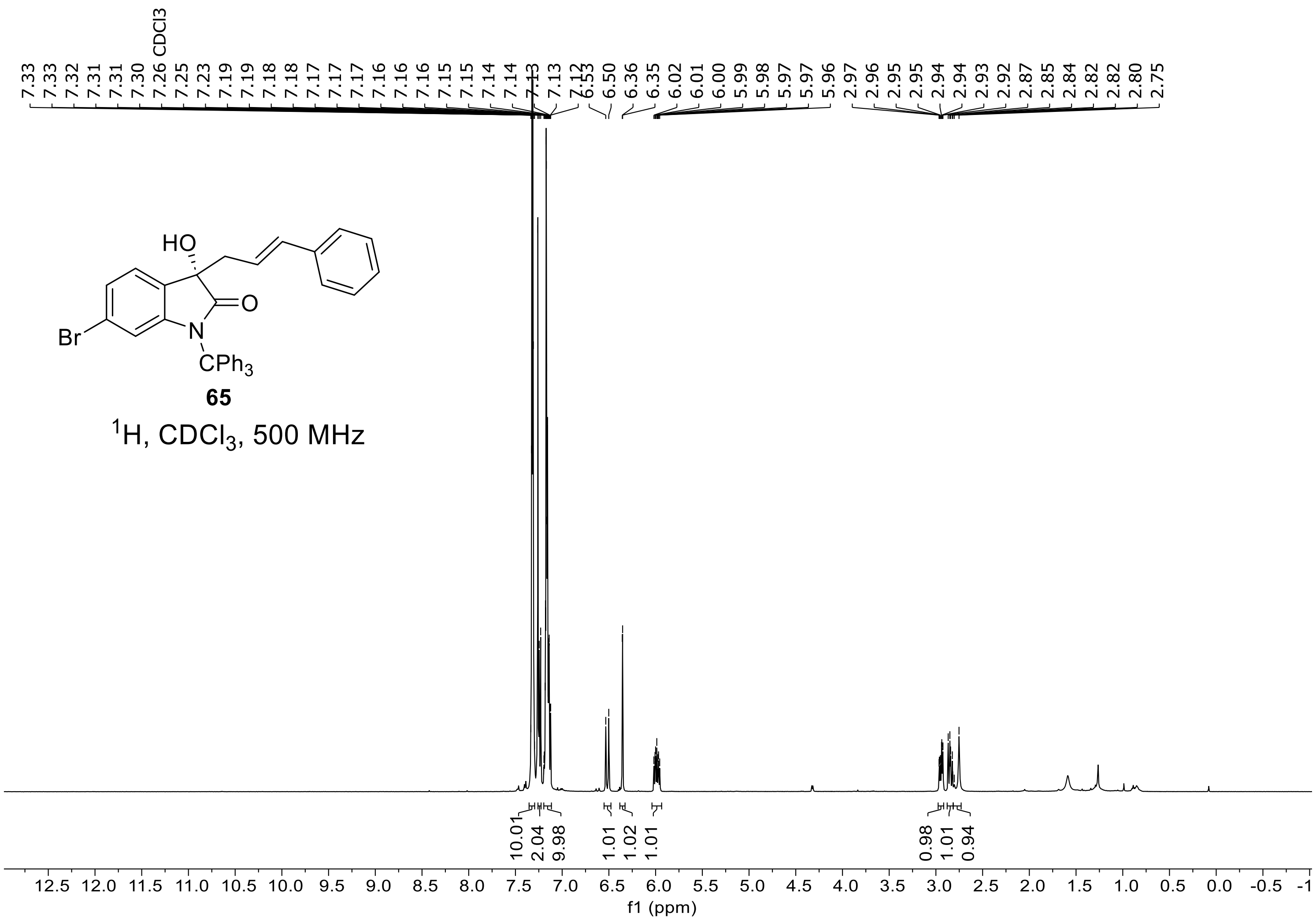

Fig. S427: ^{1}H NMR spectrum for (*S*,*E*)-6-bromo-3-cinnamyl-3-hydroxy-1-tritylindolin-2-one (**65**).

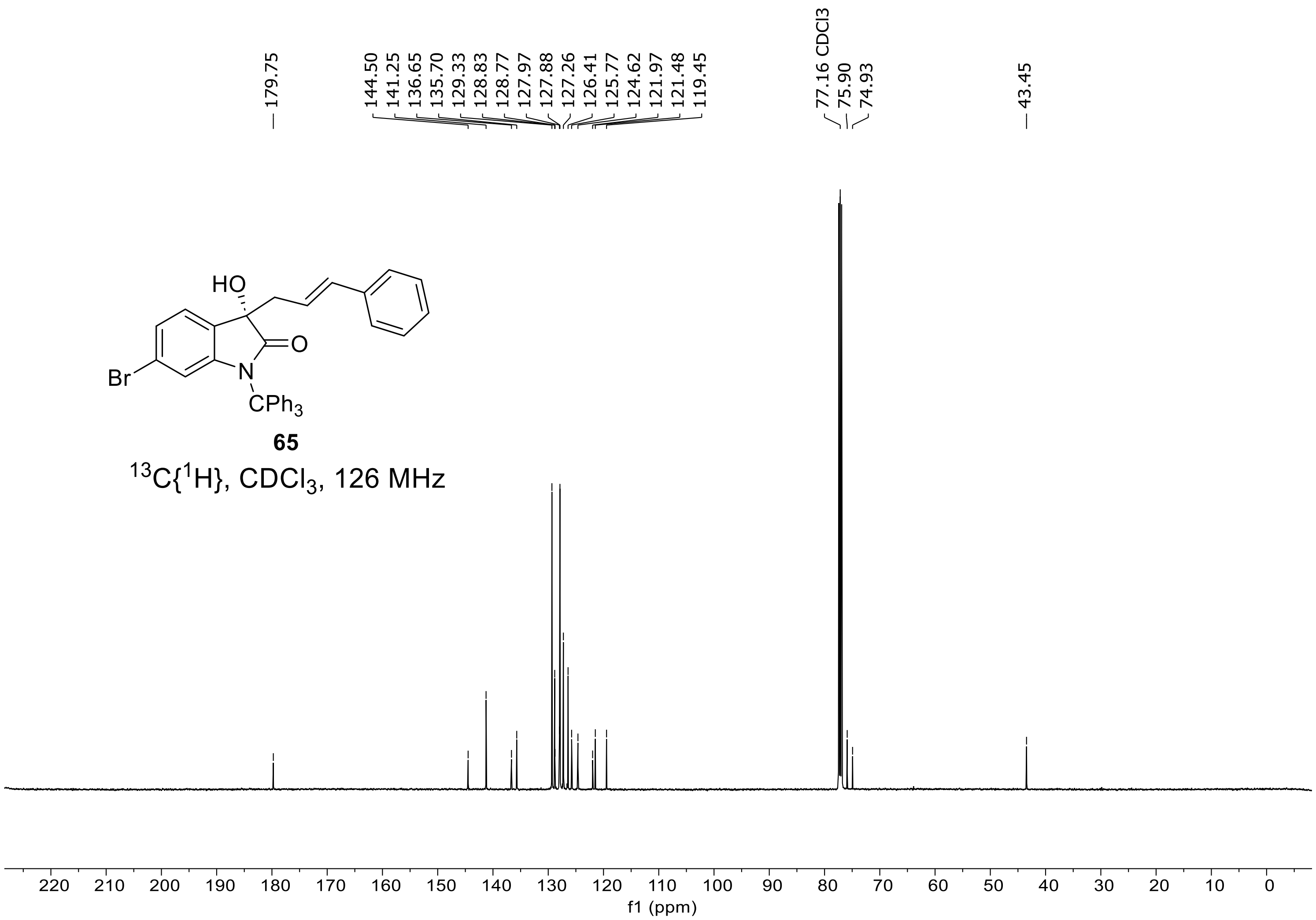

Fig. S428: $^{13}C\{^{1}H\}$ NMR spectrum for (*S*,*E*)-6-bromo-3-cinnamyl-3-hydroxy-1-tritylindolin-2-one (**65**).

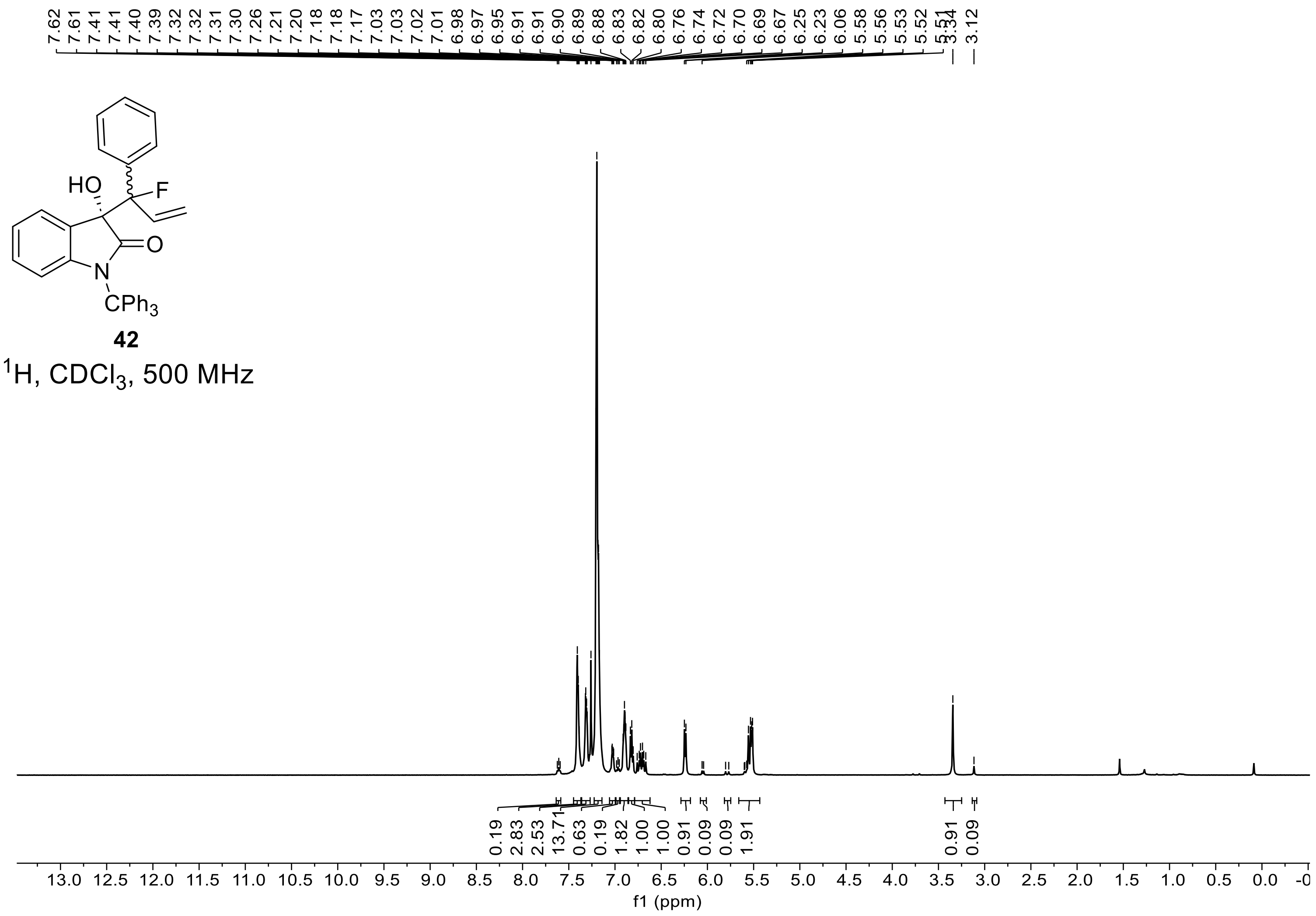

Fig. S429: ^{1}H NMR spectrum for (3*S*,1'*S*)- and (3*S*,1'*R*)-3-(1-Fluoro-1-phenylallyl)-3-hydroxy-1-tritylindolin-2-one (**42**).

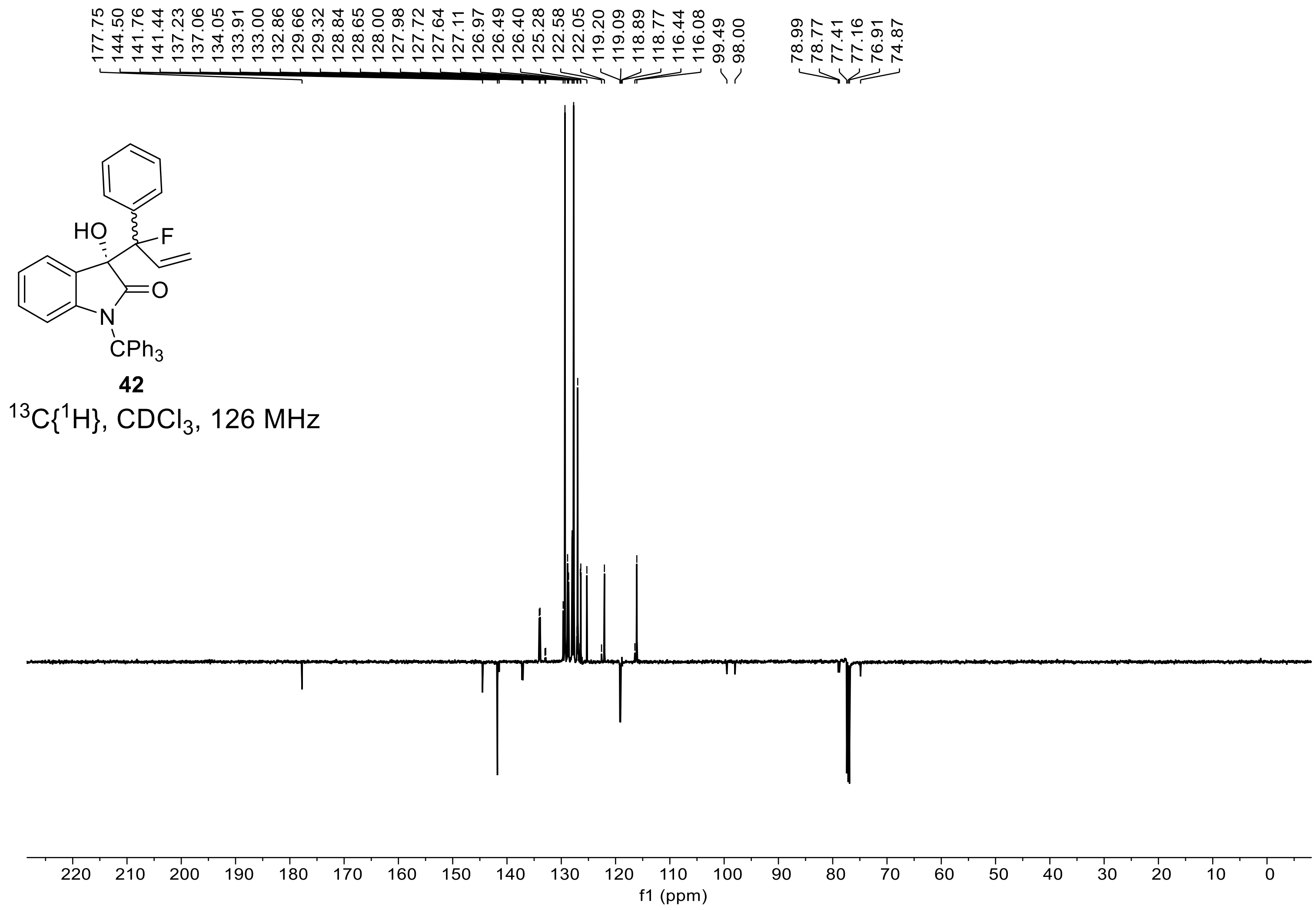

Fig. S430: $^{13}C\{^1H\}$ NMR spectrum for (3*S*,1'*S*)- and (3*S*,1'*R*)-3-(1-Fluoro-1-phenylallyl)-3-hydroxy-1-tritylindolin-2-one (**42**).

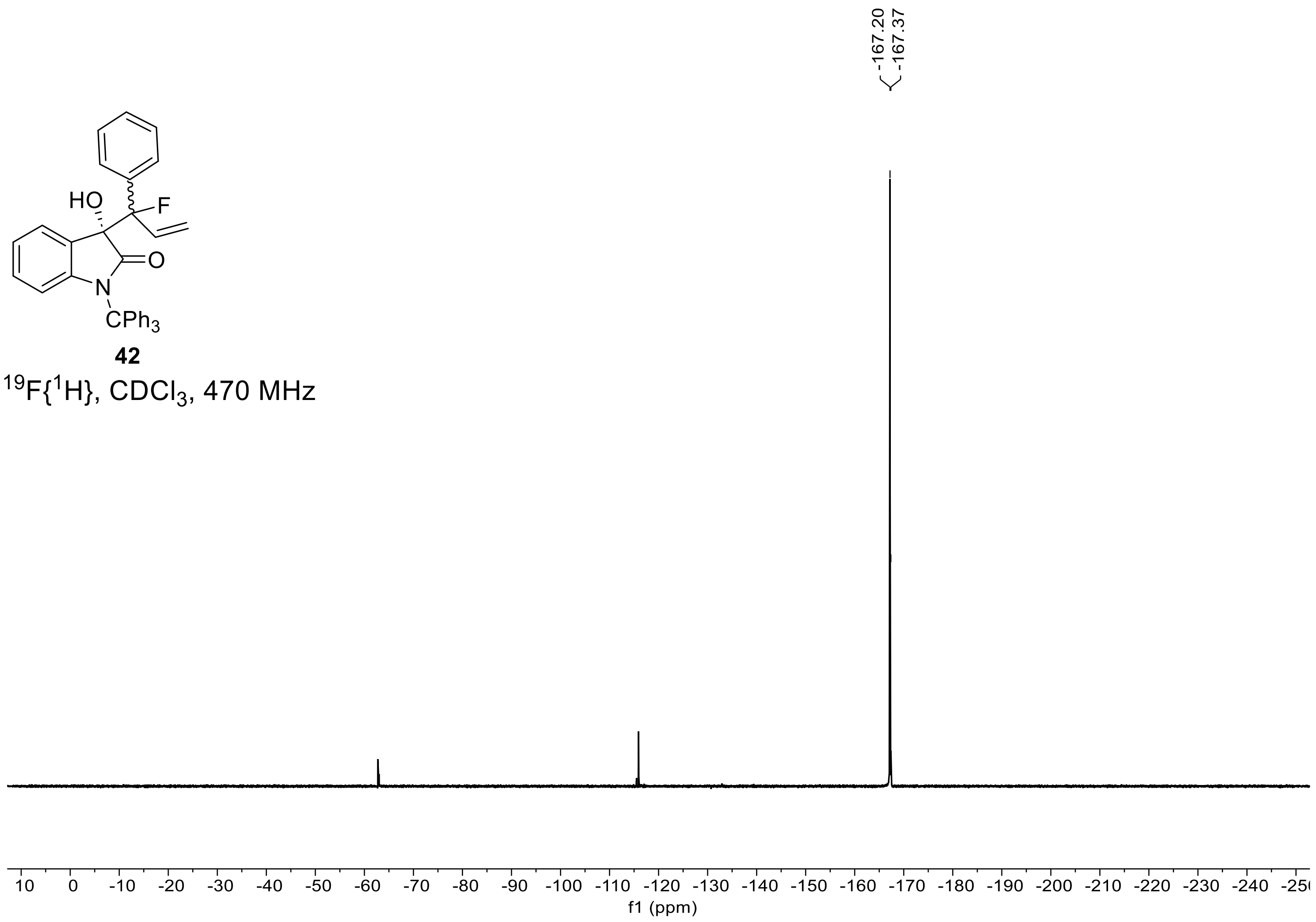

Fig. S431: $^{19}F\{^1H\}$ NMR spectrum for (3*S*,1'*S*)- and (3*S*,1'*R*)-3-(1-Fluoro-1-phenylallyl)-3-hydroxy-1-tritylindolin-2-one (**42**).

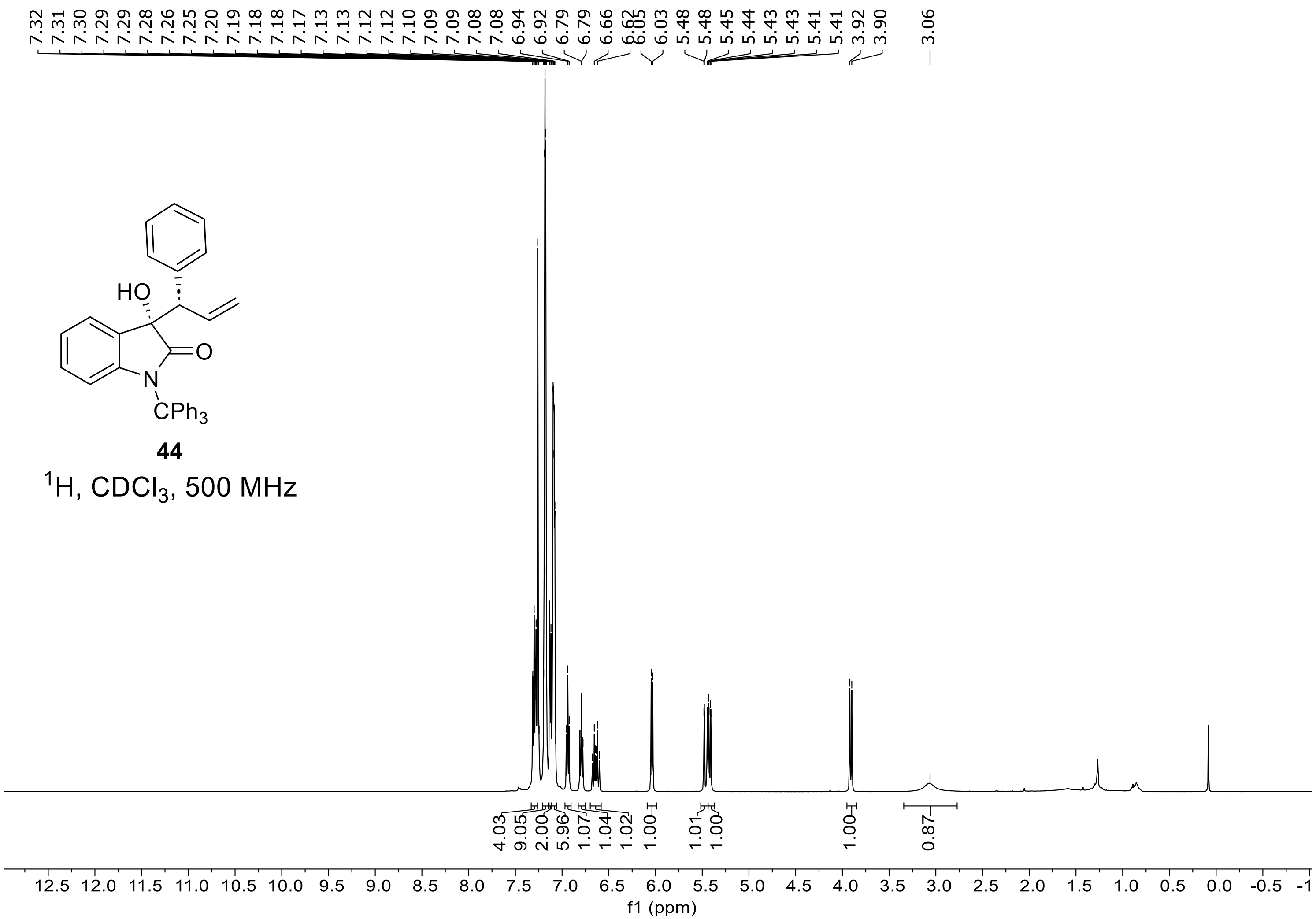

Fig. S432: ^{1}H NMR spectrum for (3*S*,1'*S*)-3-Hydroxy-3-(1-phenylallyl)-1-tritylindolin-2-one (**44**).

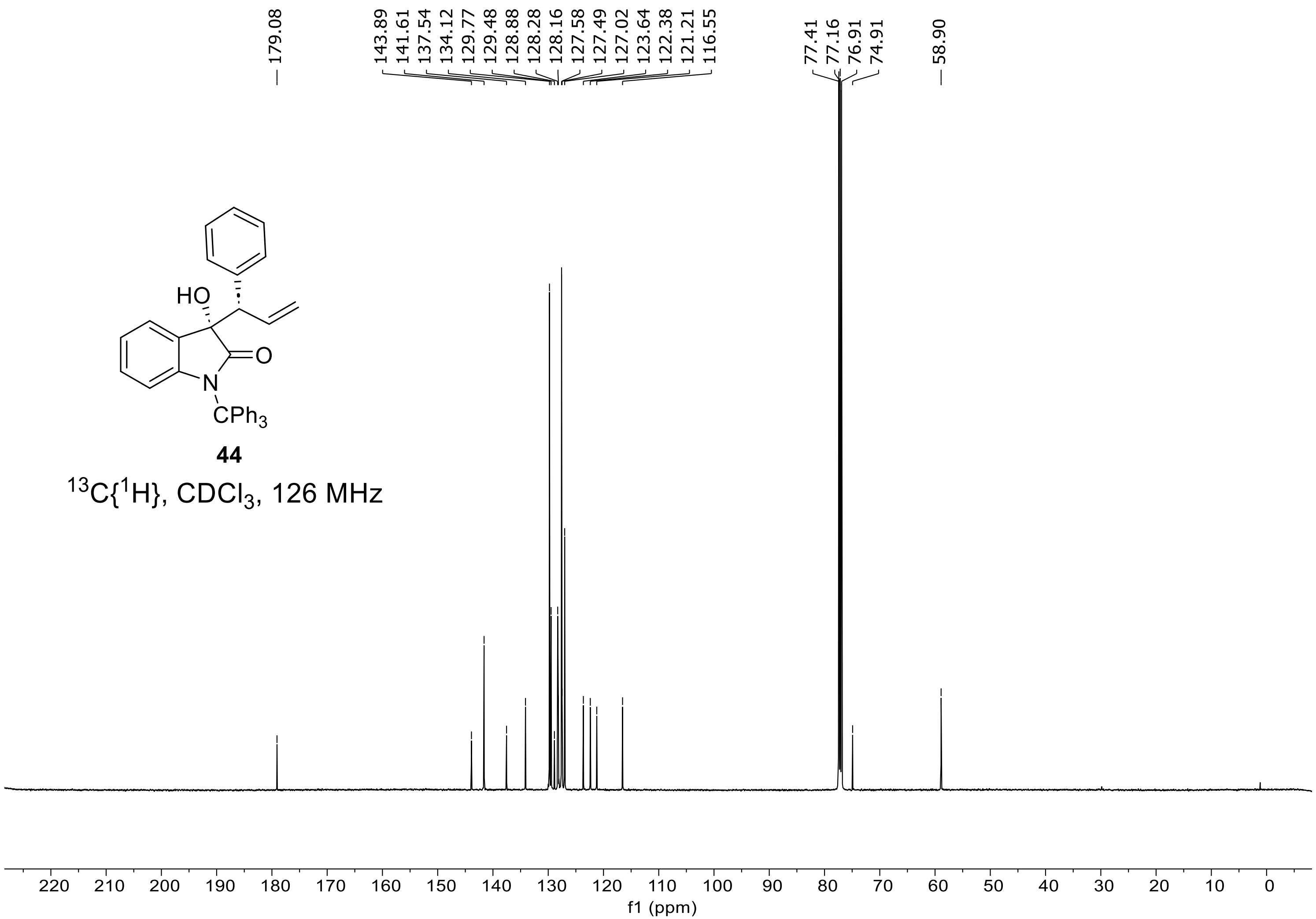

Fig. S433: ^{13}C{^{1}H} NMR spectrum for (3*S*,1'*S*)-3-Hydroxy-3-(1-phenylallyl)-1-tritylindolin-2-one (**44**).

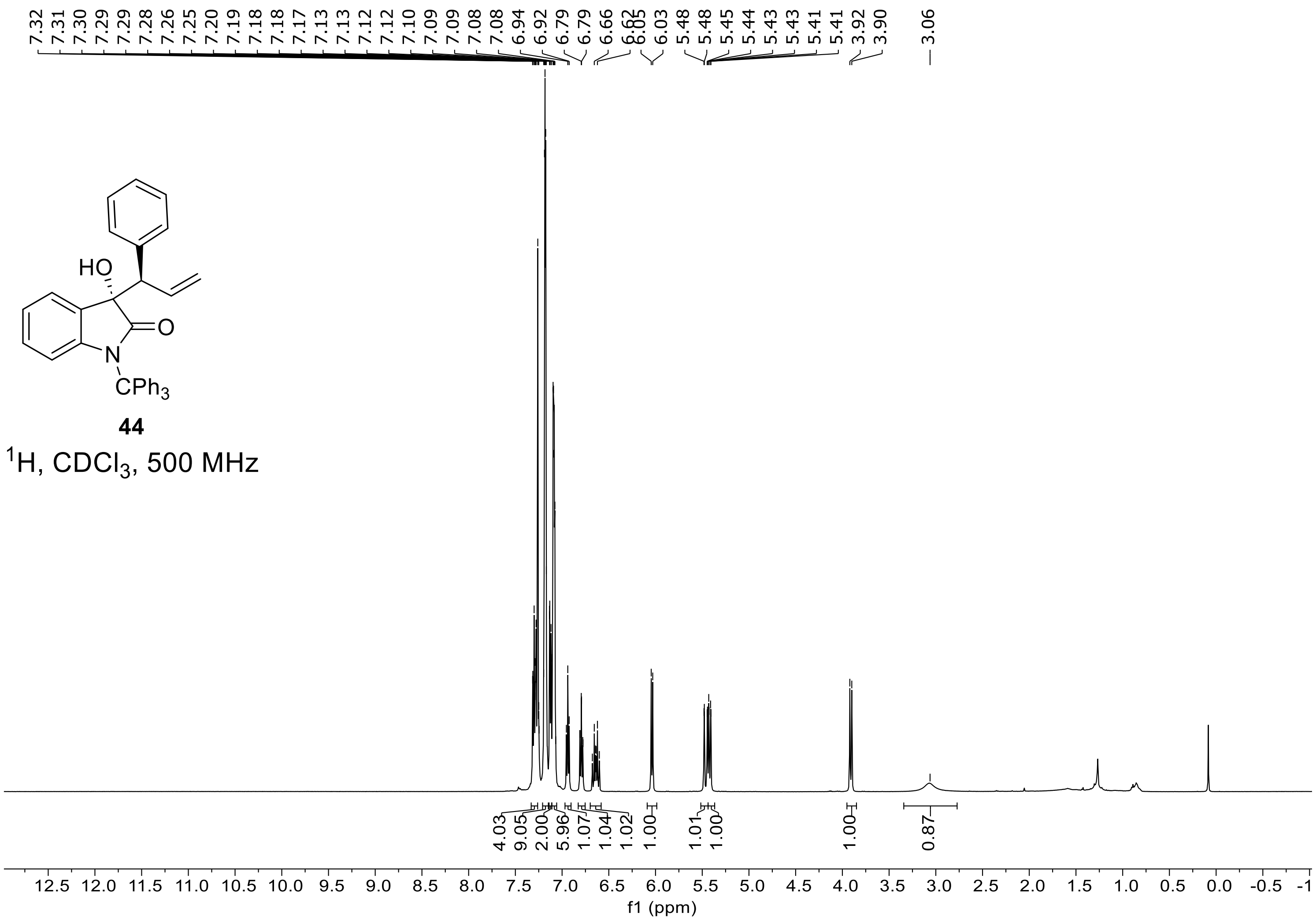

Fig. S434: ^{1}H NMR spectrum for (3*S*,1'*R*)-3-Hydroxy-3-(1-phenylallyl)-1-tritylindolin-2-one (**44**).

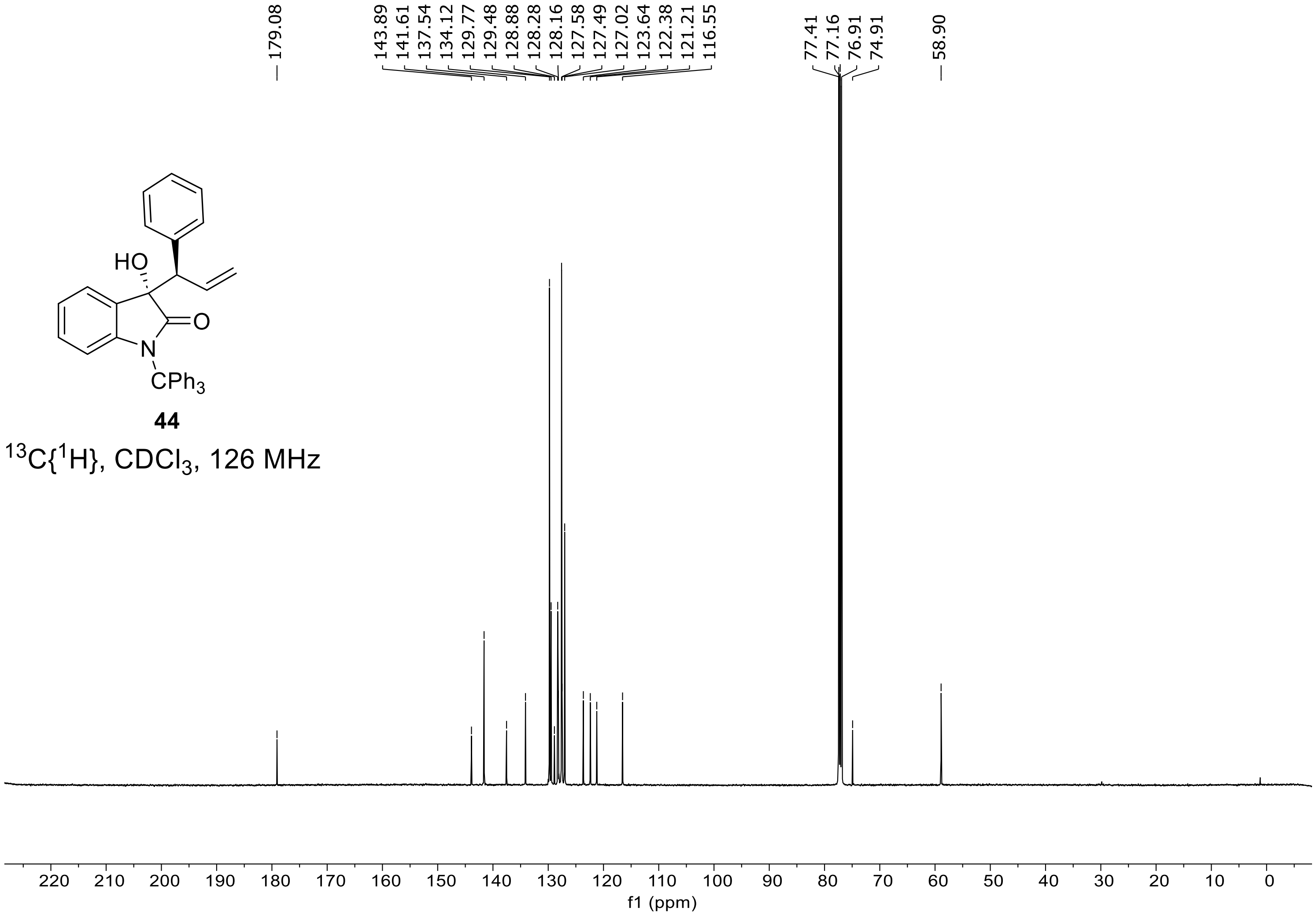

Fig. 5 $^{13}C\{^1H\}$ NMR spectrum for (3*S*,1'*R*)-3-Hydroxy-3-(1-phenylallyl)-1-tritylindolin-2-one (**44**).

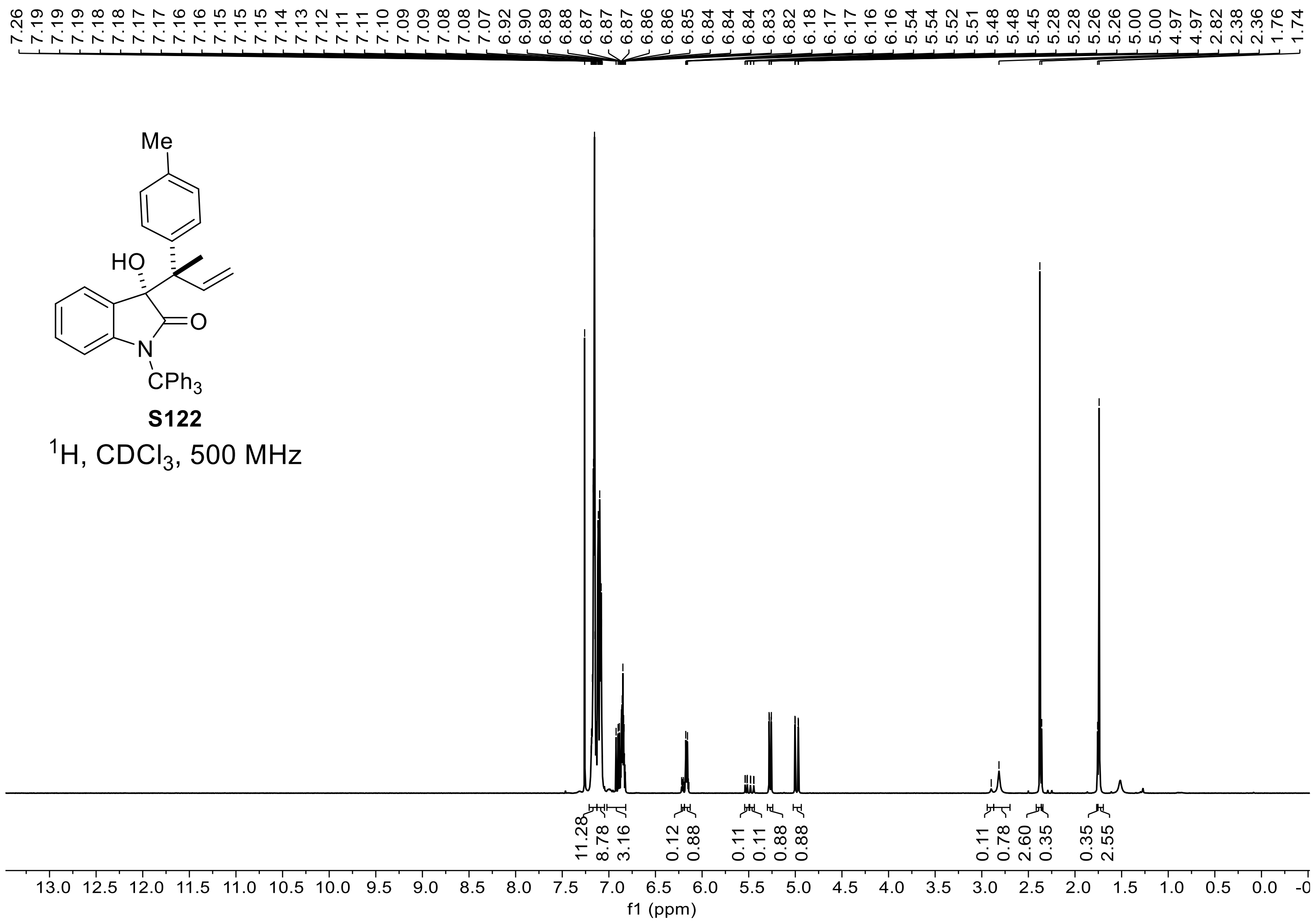

Fig. S436: ^{1}H NMR spectrum for (±)-(3*S*,2'*S*)-3-Hydroxy-3-[2-(*p*-tolyl)but-3-en-2-yl]-1-tritylindolin-2-one (**S122**).

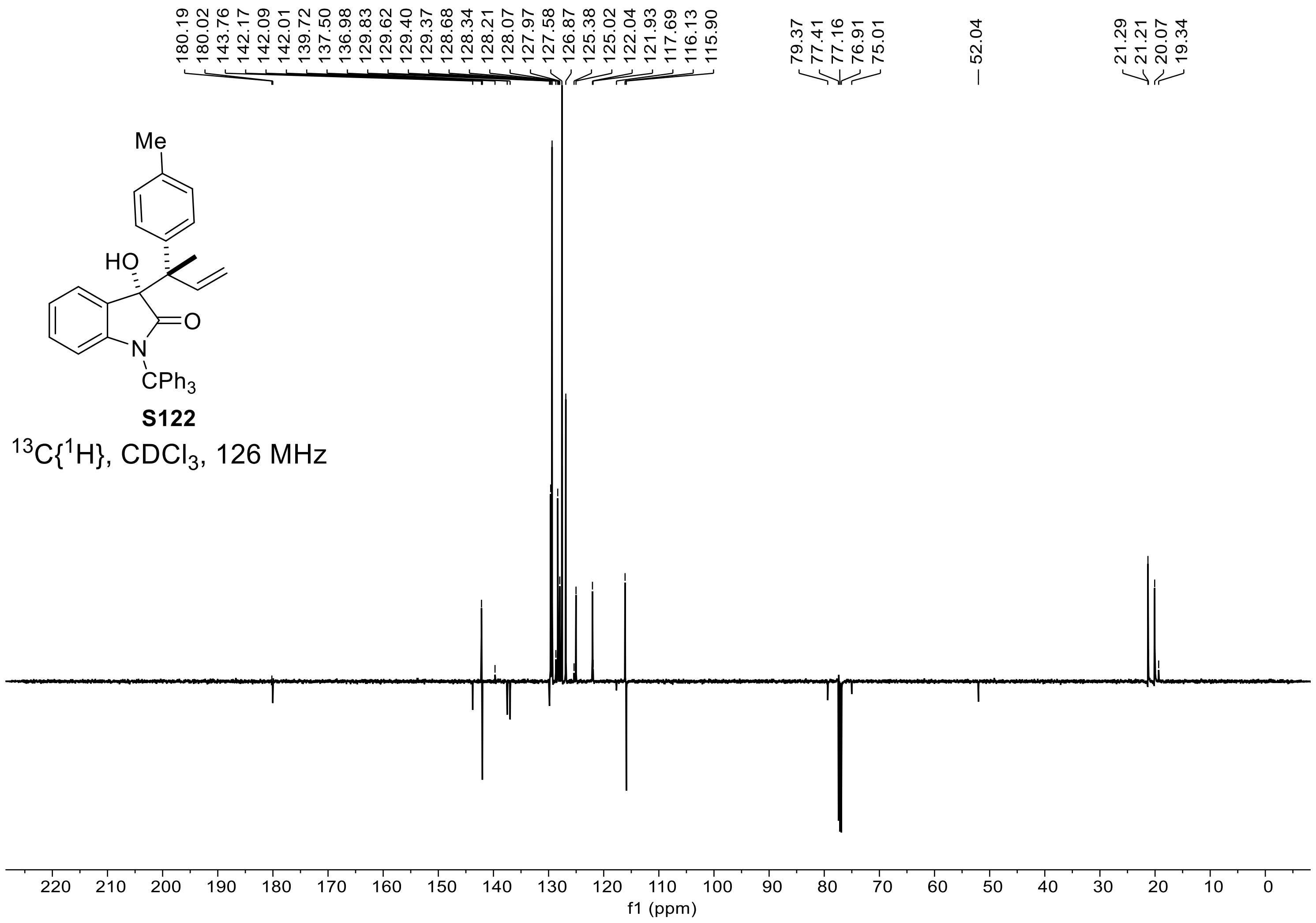

Fig. S437: $^{13}C\{^{1}H\}$ NMR spectrum for (±)-(3*S*,2’*S*)-3-Hydroxy-3-[2-(*p*-tolyl)but-3-en-2-yl]-1-tritylindolin-2-one (**S122**).

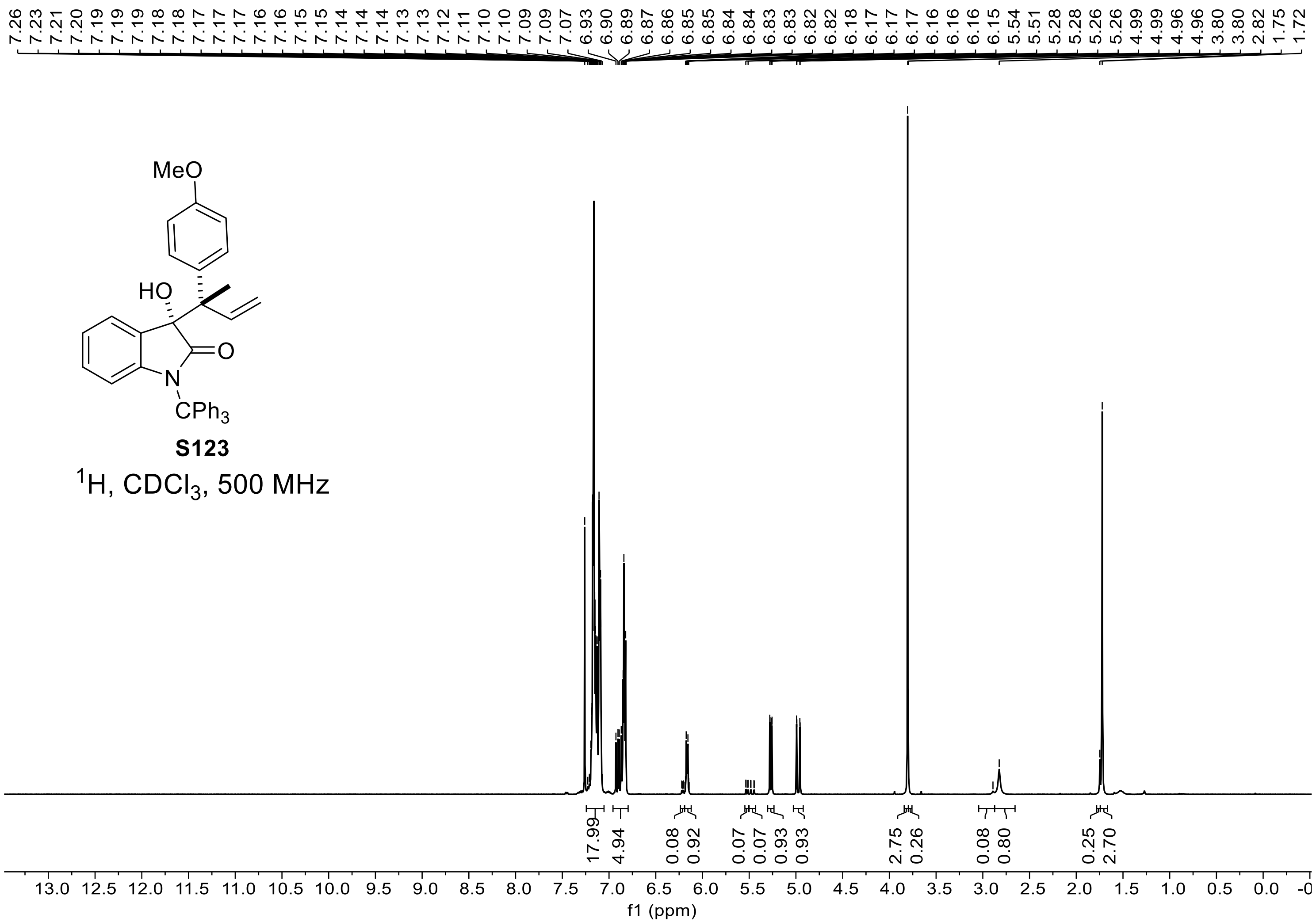

Fig. S438: ^{1}H NMR spectrum for (±)-(3*S*,2'*S*)-3-Hydroxy-3-[2-(4-methoxyphenyl)but-3-en-2-yl]-1-tritylindolin-2-one (**S123**).

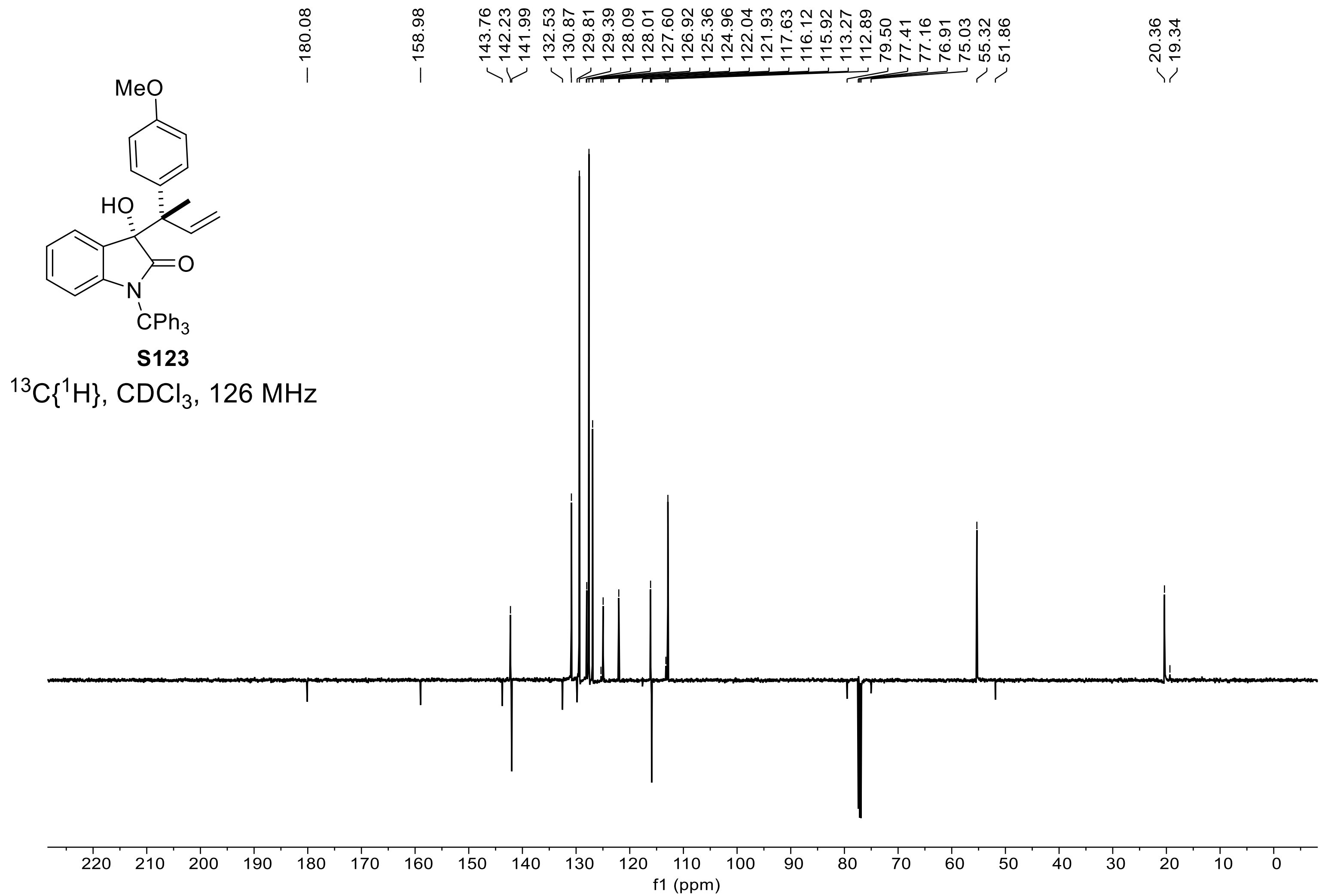

Fig. S439: $^{13}C\{^{1}H\}$ NMR spectrum for (±)-(3*S*,2'*S*)-3-Hydroxy-3-[2-(4-methoxyphenyl)but-3-en-2-yl]-1-tritylindolin-2-one (**S123**).

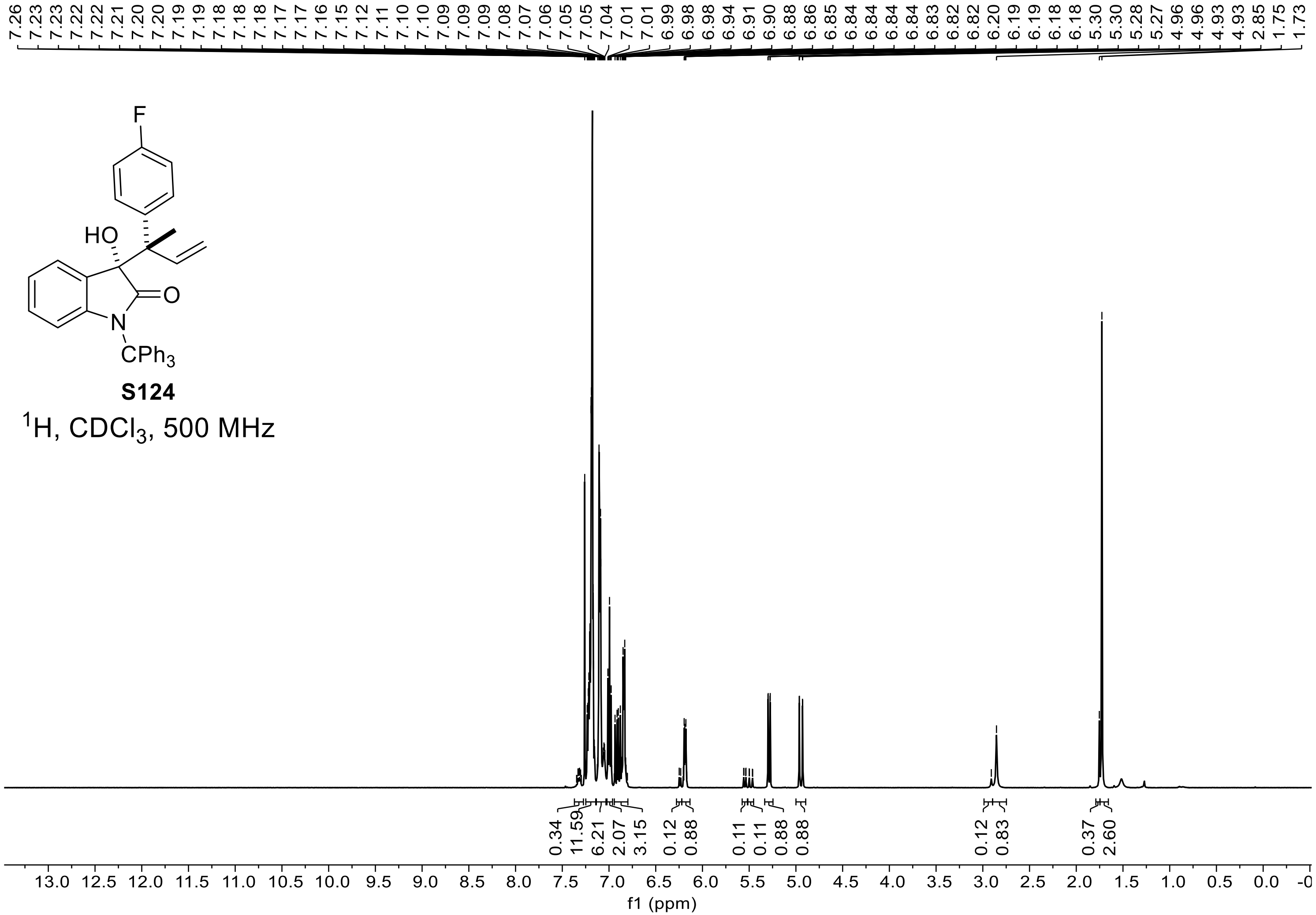

Fig. S440: $^{13}C\{^1H\}$ NMR spectrum for (±)-(3*S*,2'*S*)-3-[2-(4-Fluorophenyl)but-3-en-2-yl]-3-hydroxy-1-tritylindolin-2-one (**S124**).

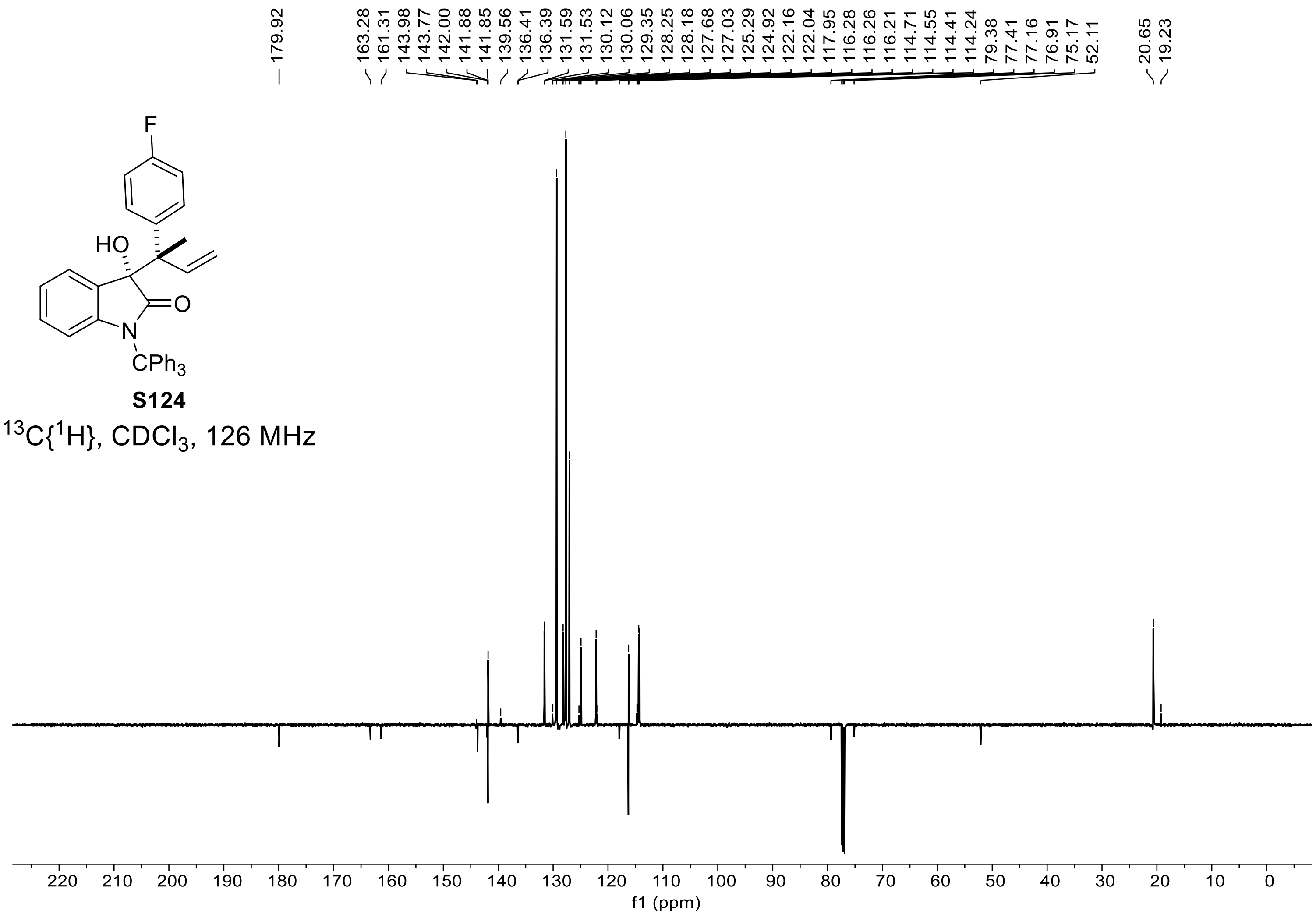

Fig. S441: $^{13}C\{^{1}H\}$ NMR spectrum for (±)-(3*S*,2'*S*)-3-[2-(4-Fluorophenyl)but-3-en-2-yl]-3-hydroxy-1-tritylindolin-2-one (**S124**).

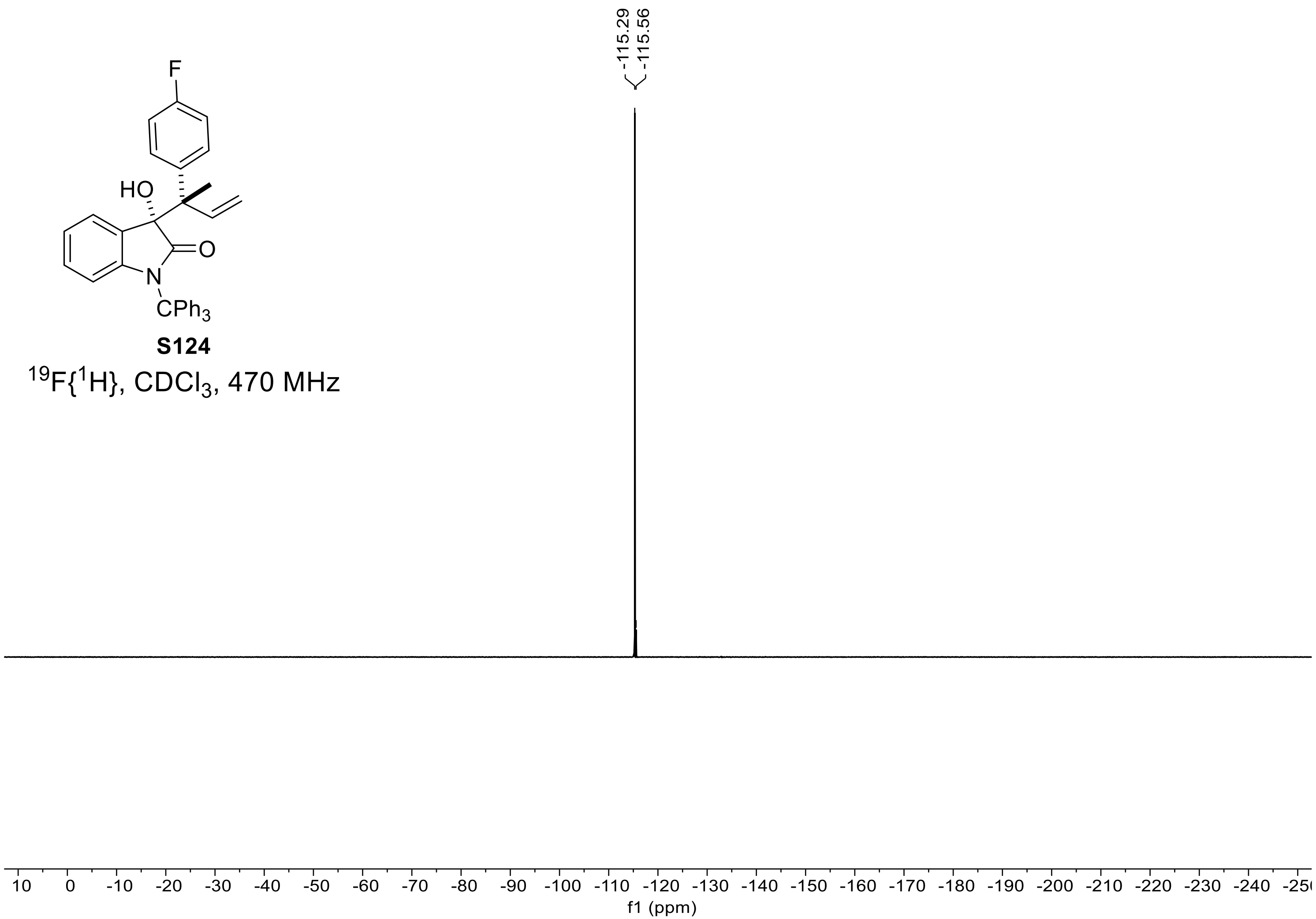

Fig. S442: $^{19}F\{^{1}H\}$ NMR spectrum for (±)-(3*S*,2'*S*)-3-[2-(4-Fluorophenyl)but-3-en-2-yl]-3-hydroxy-1-tritylindolin-2-one (**S124**).

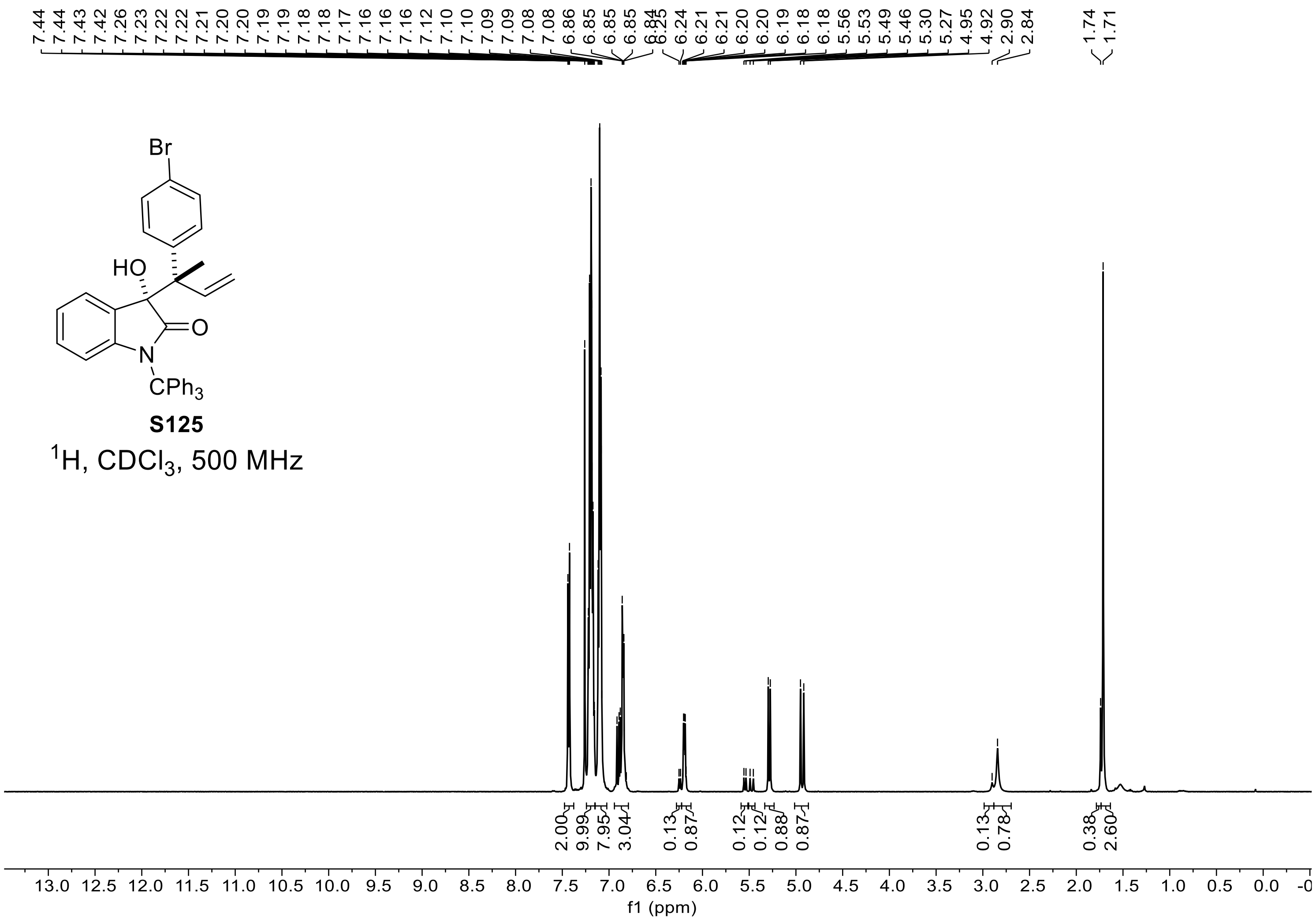

Fig. S443: ^{1}H NMR spectrum for (±)-(3*S*,2'*S*)-3-[2-(4-Bromophenyl)but-3-en-2-yl]-3-hydroxy-1-tritylindolin-2-one (**S125**).

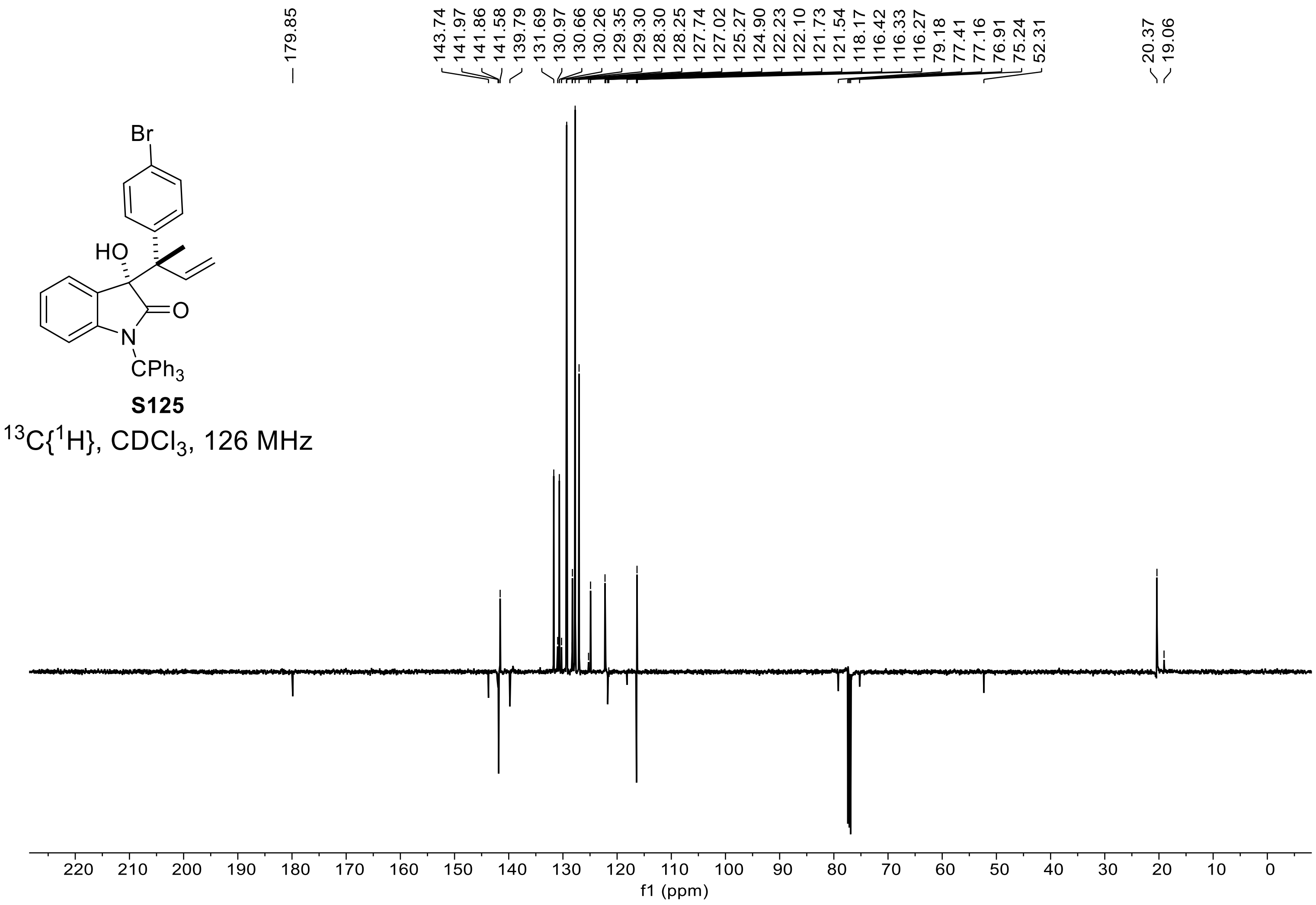

Fig. S444: $^{13}C\{^{1}H\}$ NMR spectrum for (±)-(3*S*,2’*S*)-3-[2-(4-Bromophenyl)but-3-en-2-yl]-3-hydroxy-1-tritylindolin-2-one (**S125**).

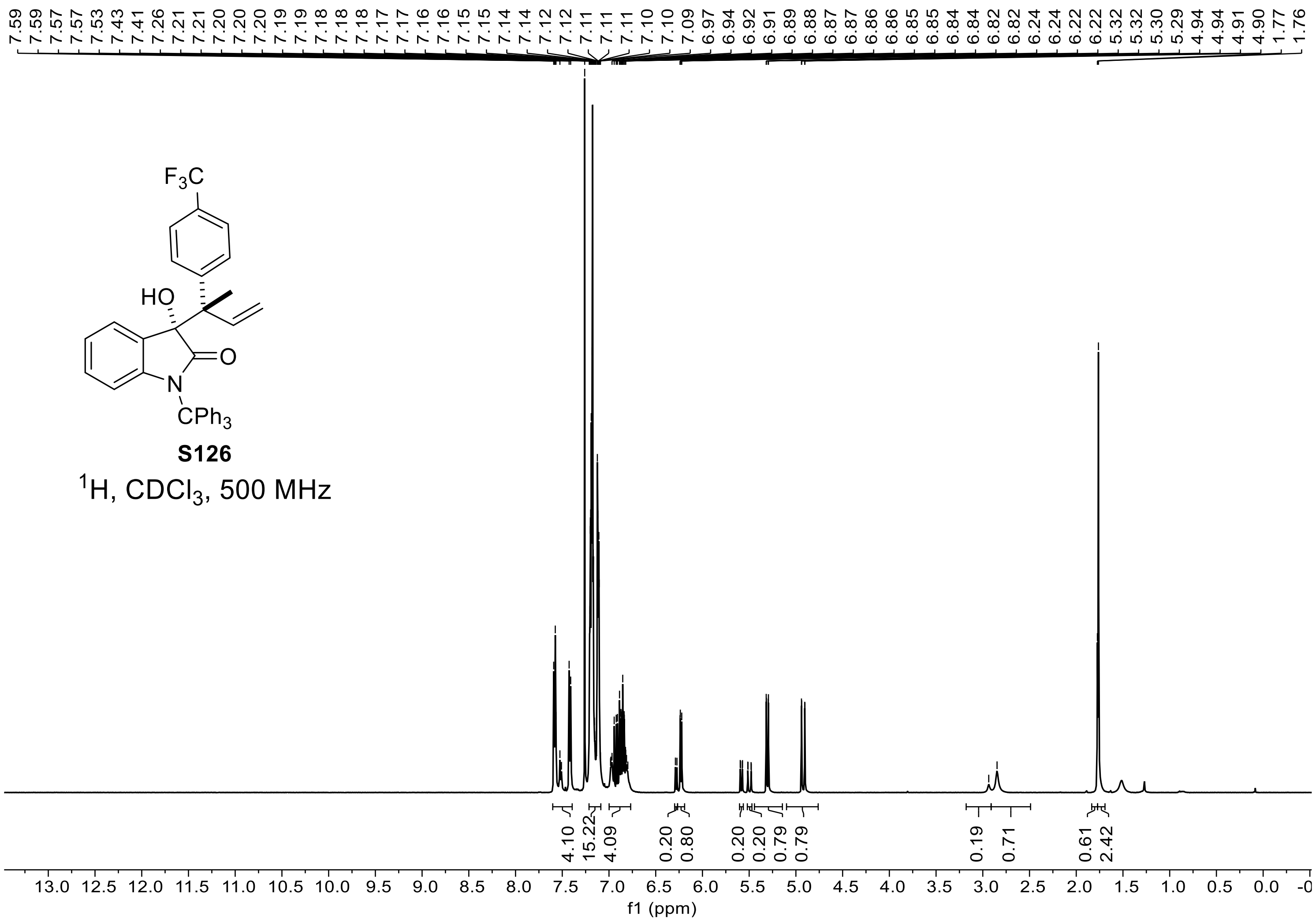

Fig. S445: ^{1}H NMR spectrum for (±)-(3*S*,2'*S*)-3-Hydroxy-3-{2-[4-(trifluoromethyl)phenyl]but-3-en-2-yl}-1-tritylindolin-2-one (**S126**).

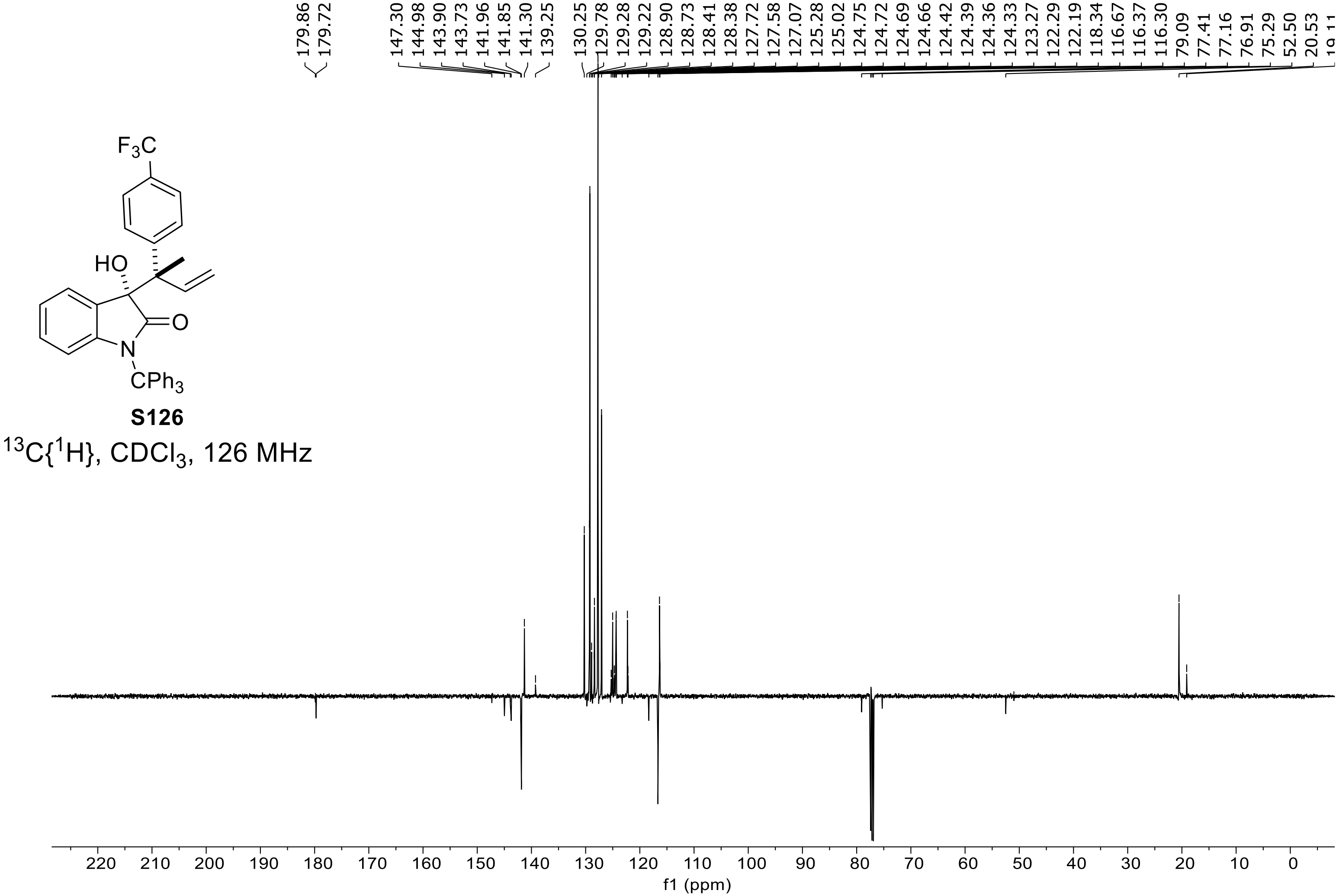

Fig. S446: $^{13}C\{^1H\}$ NMR spectrum for (±)-(3*S*,2'*S*)-3-Hydroxy-3-{2-[4-(trifluoromethyl)phenyl]but-3-en-2-yl}-1-tritylindolin-2-one (**S126**).

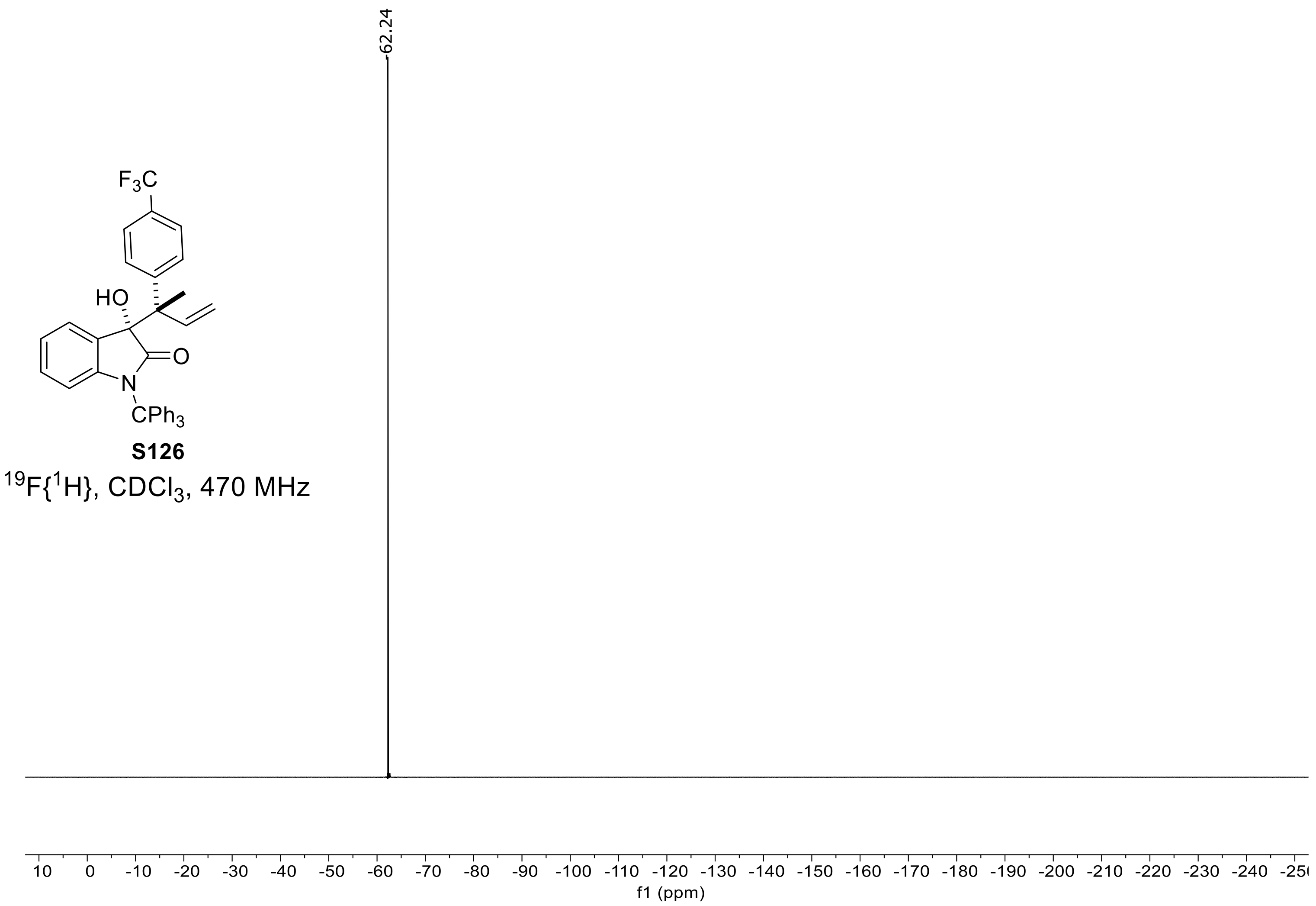

Fig. S447: $^{19}F\{^1H\}$ NMR spectrum for (±)-(3*S*,2'*S*)-3-Hydroxy-3-{2-[4-(trifluoromethyl)phenyl]but-3-en-2-yl}-1-tritylindolin-2-one (**S126**).

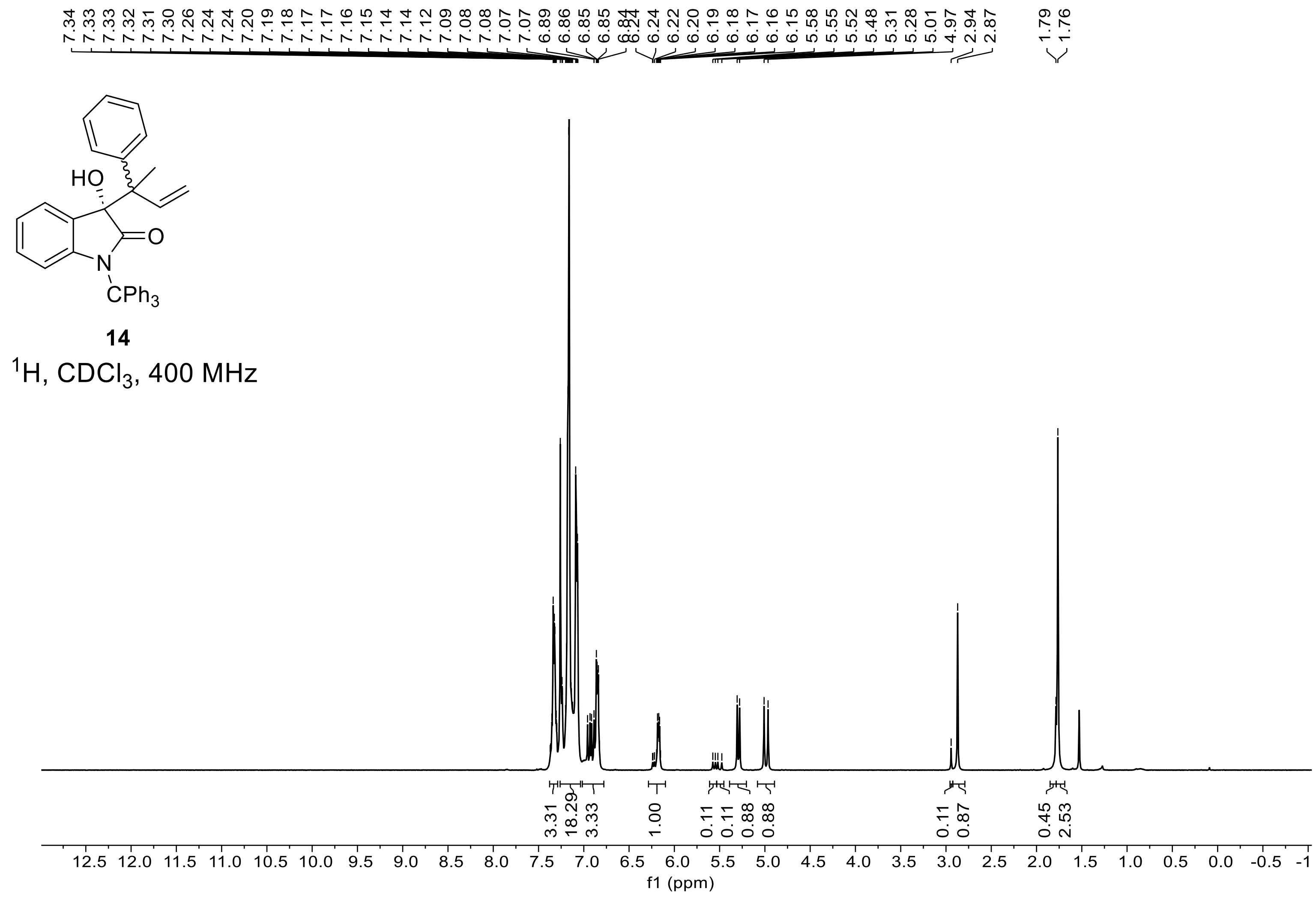

Fig. S448: ^{1}H NMR spectrum for (3*S*,2'*S*)- and (3*S*,2'*R*)- 3-Hydroxy-3-(2-phenylbut-3-en-2-yl)-1-tritylindolin-2-one (**14**).

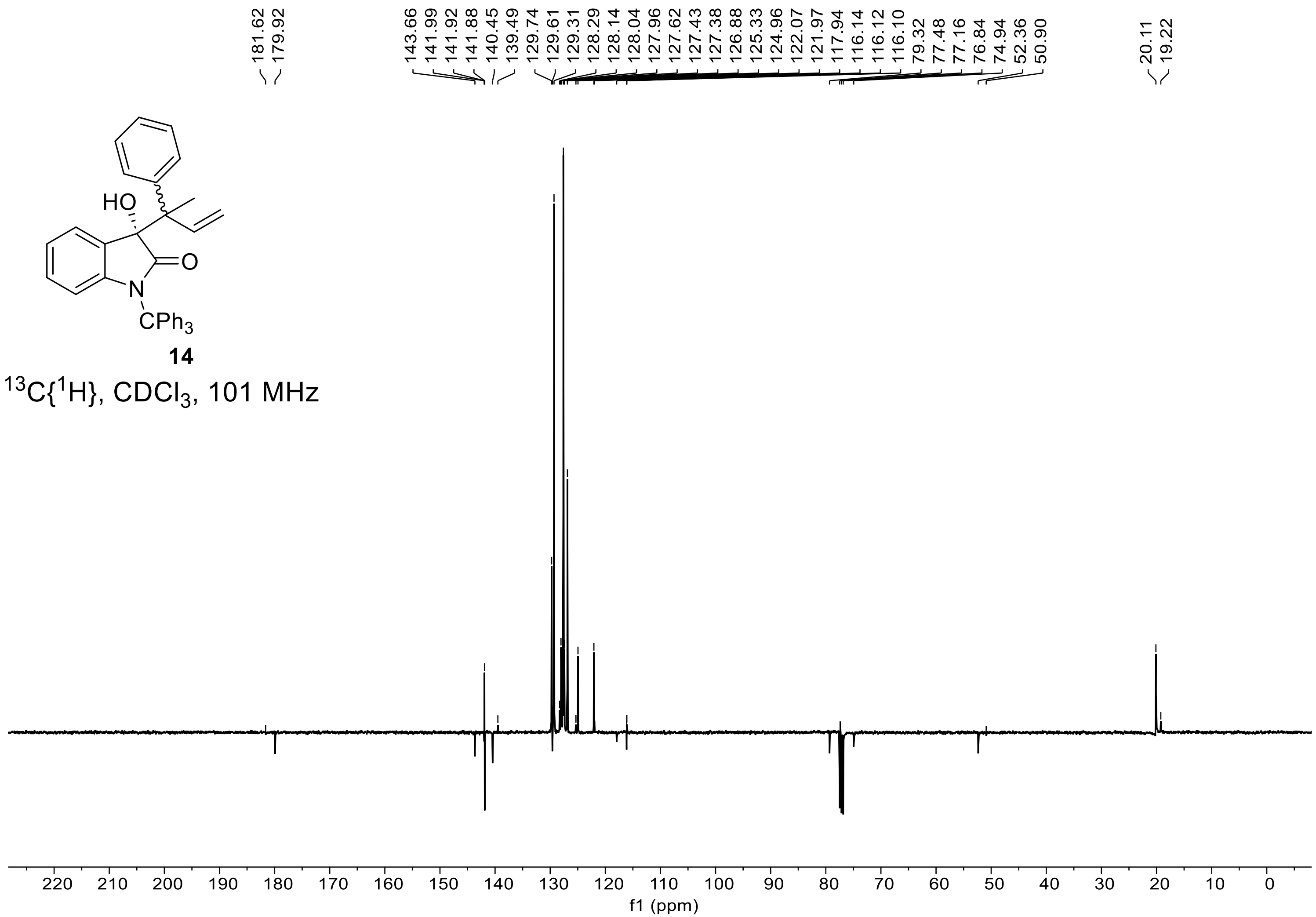

Fig. S449: $^{13}C\{^1H\}$ NMR spectrum for (3*S*,2'*S*)- and (3*S*,2'*R*)- 3-Hydroxy-3-(2-phenylbut-3-en-2-yl)-1-tritylindolin-2-one (**14**)..

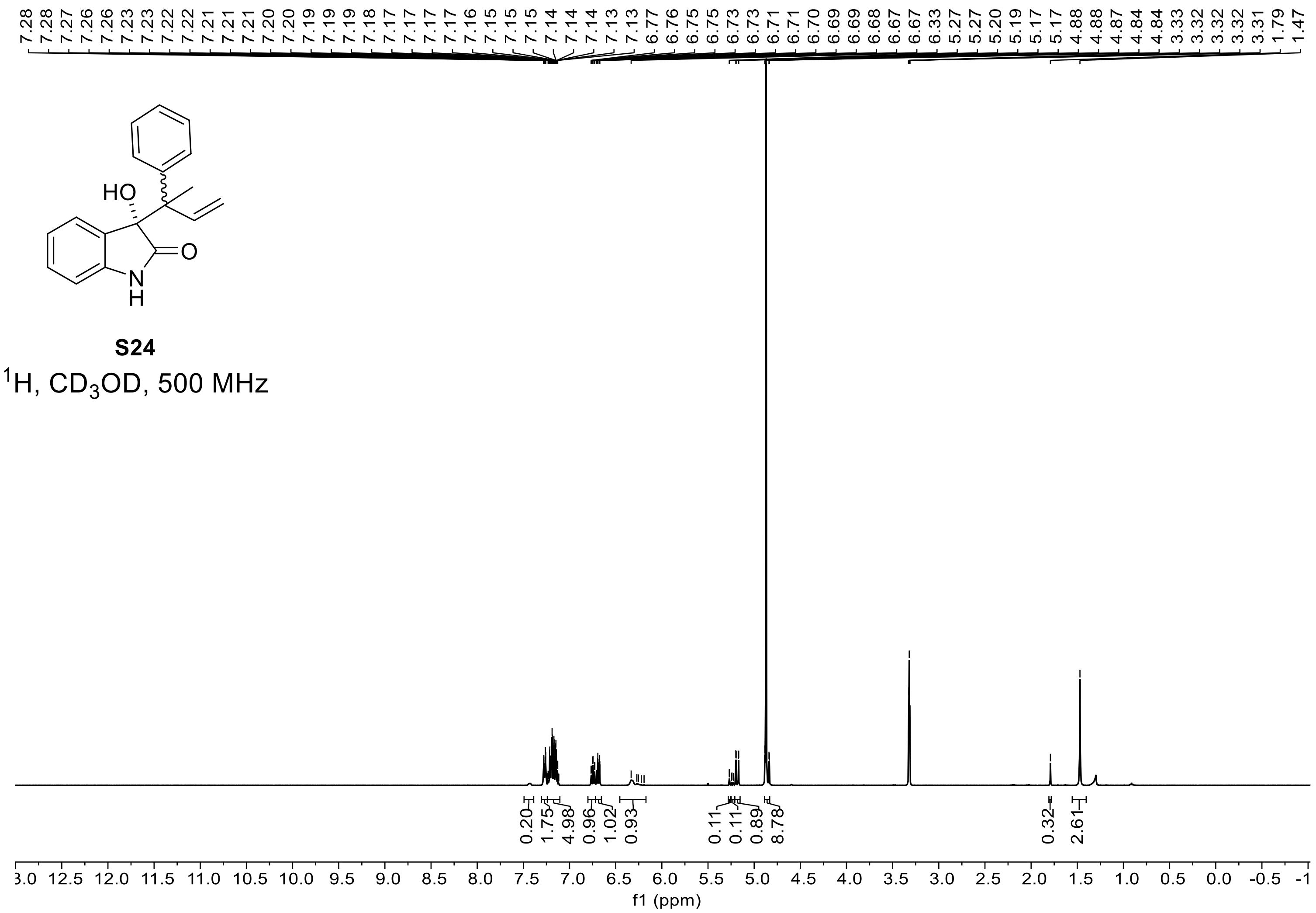

Fig. S450: ^{1}H NMR spectrum for (3*S*,2'*S*)- and (3*S*,2'*R*)-3-Hydroxy-3-(2-phenylbut-3-en-2-yl)indolin-2-one (**S24**).

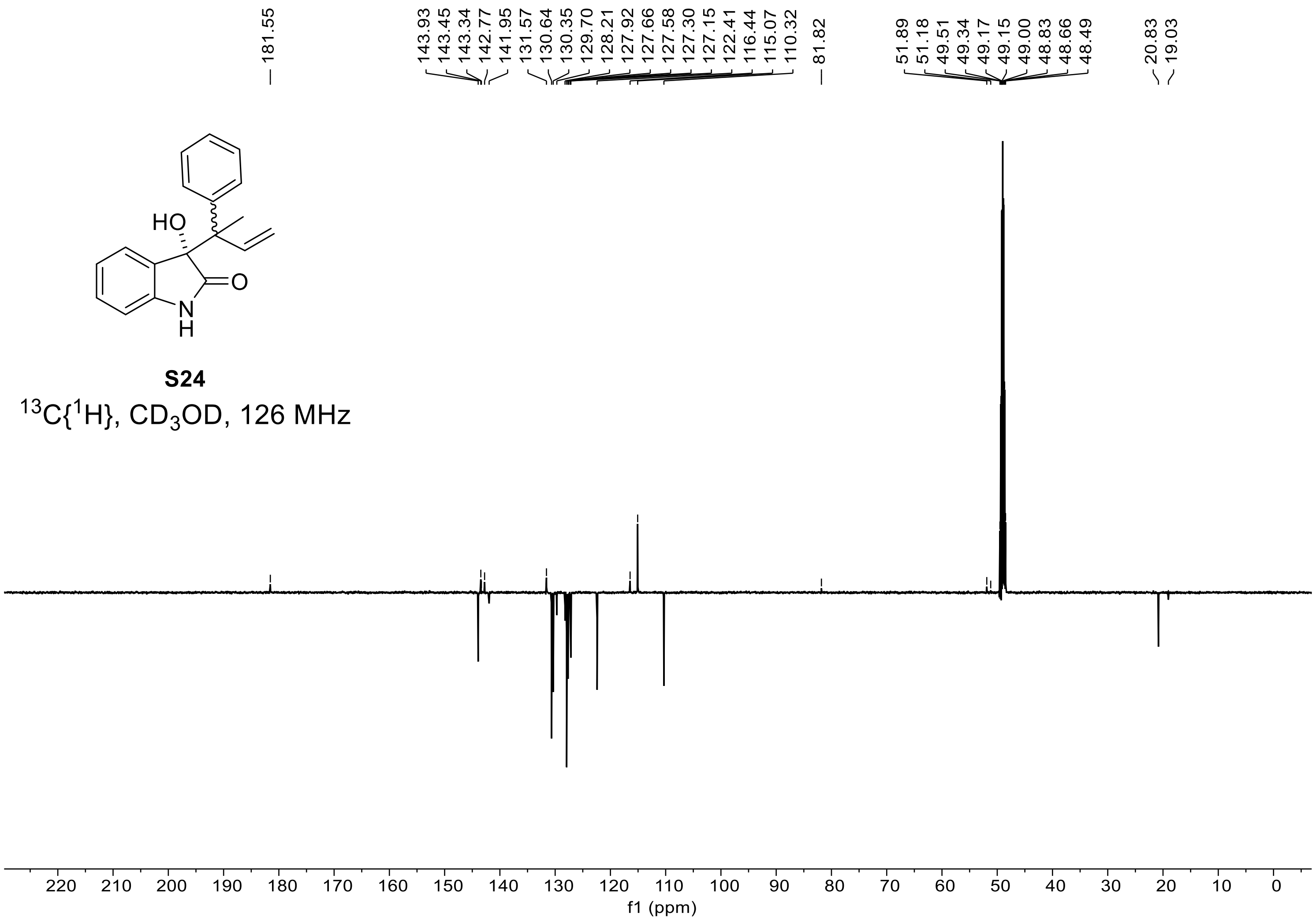

Fig. S451: $^{13}C\{^{1}H\}$ NMR spectrum for (3*S*,2'*S*)- and (3*S*,2'*R*)-3-Hydroxy-3-(2-phenylbut-3-en-2-yl)indolin-2-one (**S24**).

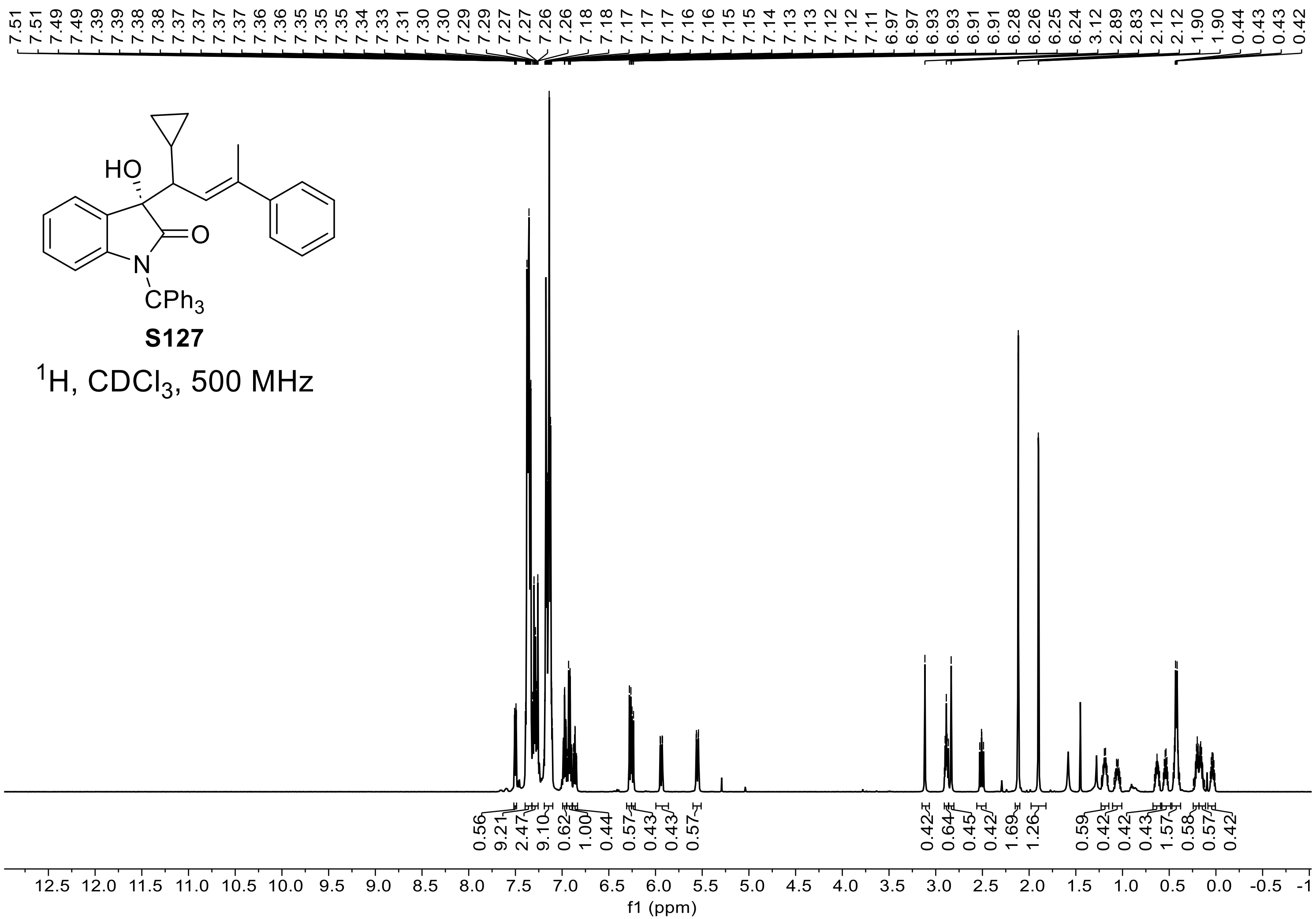

Fig. S452: ^{1}H NMR spectrum for ((*E*)-1-Cyclopropyl-3-phenylbut-2-en-1-yl)-3-hydroxy-1-tritylindolin-2-one (**S127**).

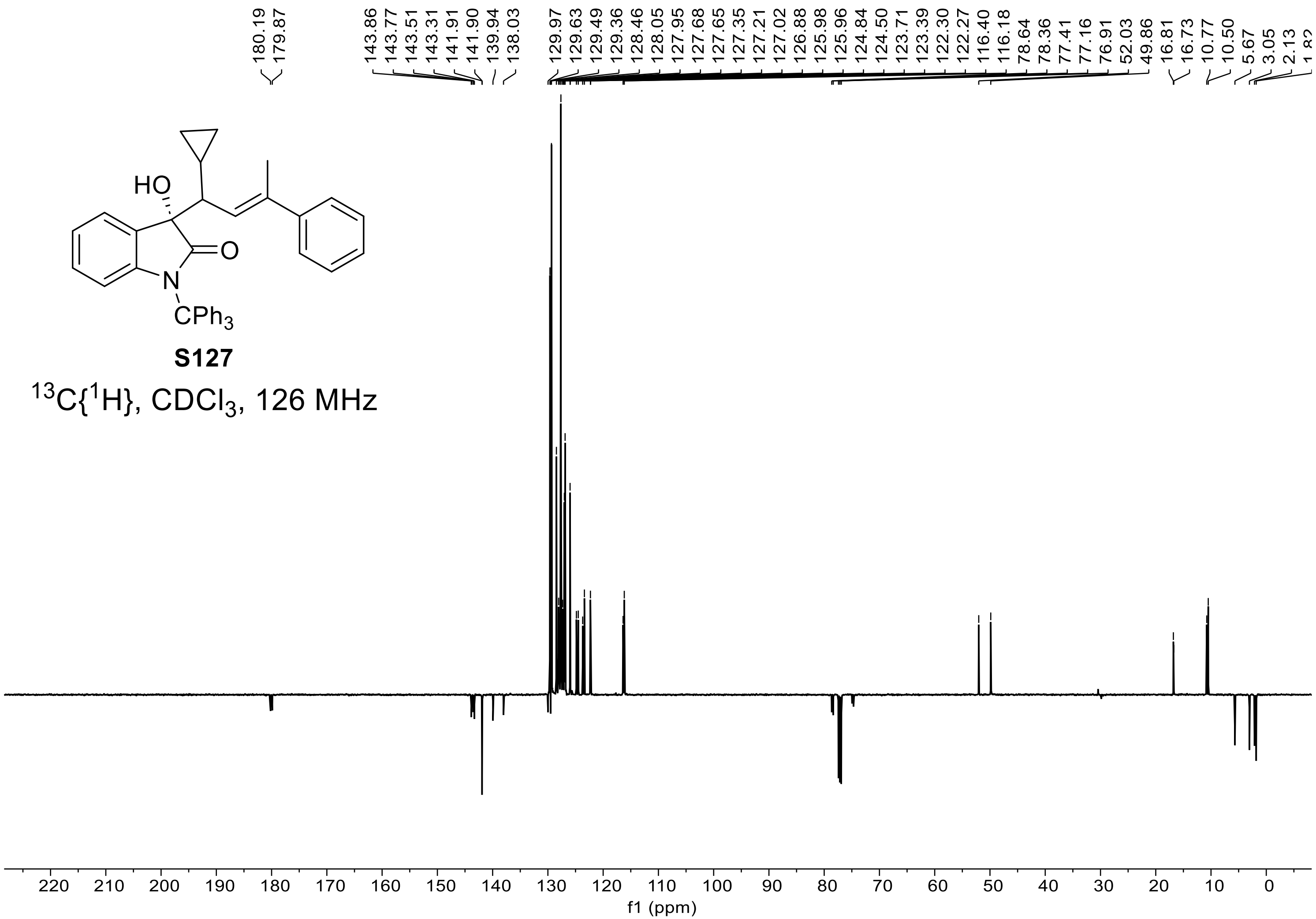

Fig. S453: $^{13}C\{^1H\}$ NMR spectrum for ((*E*)-1-Cyclopropyl-3-phenylbut-2-en-1-yl)-3-hydroxy-1-tritylindolin-2-one (**S127**).

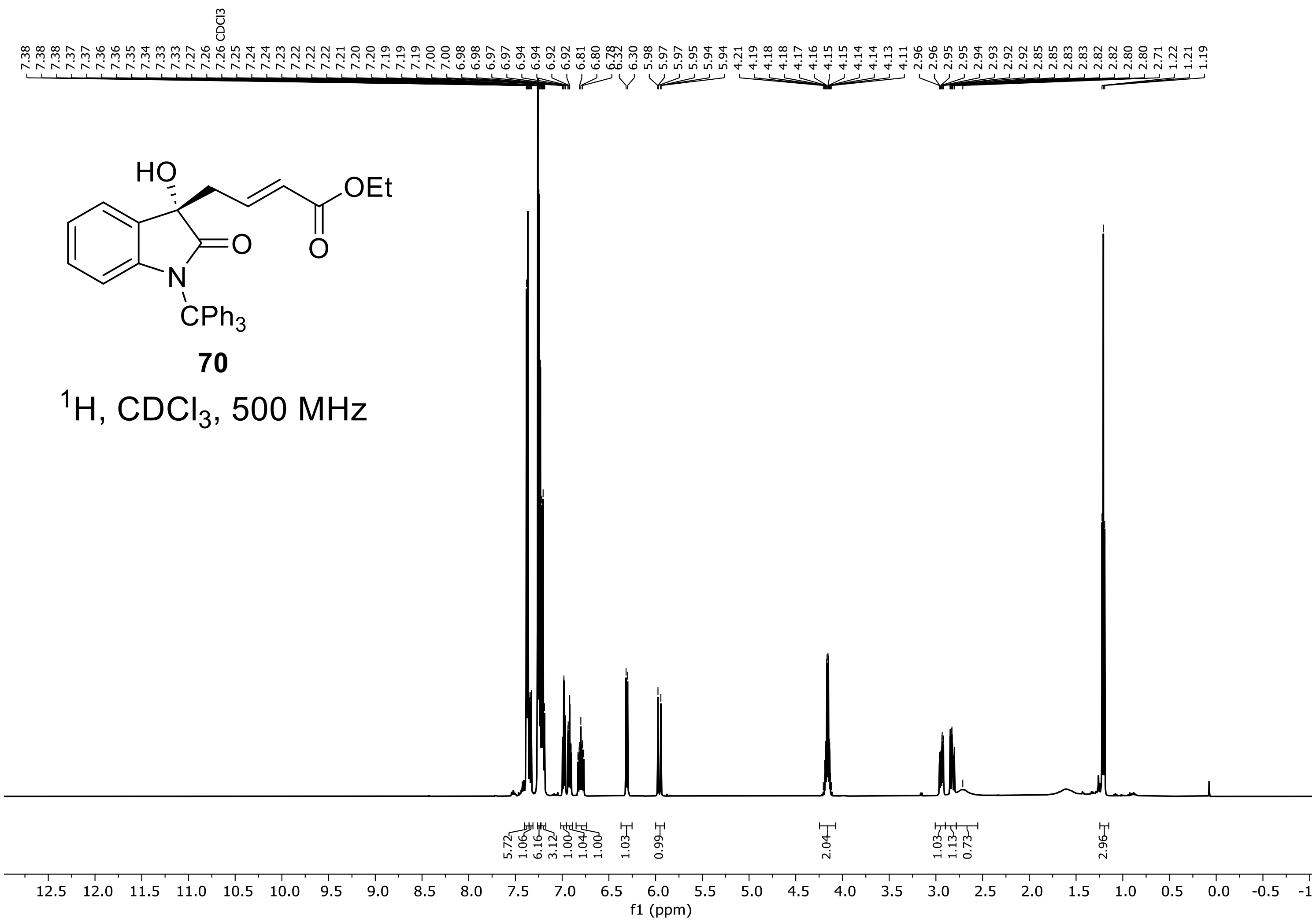

Fig. S454: ^{1}H NMR spectrum for ethyl (*E*)-4-(3-hydroxy-2-oxo-1-tritylindolin-3-yl)crotonate (**70**).

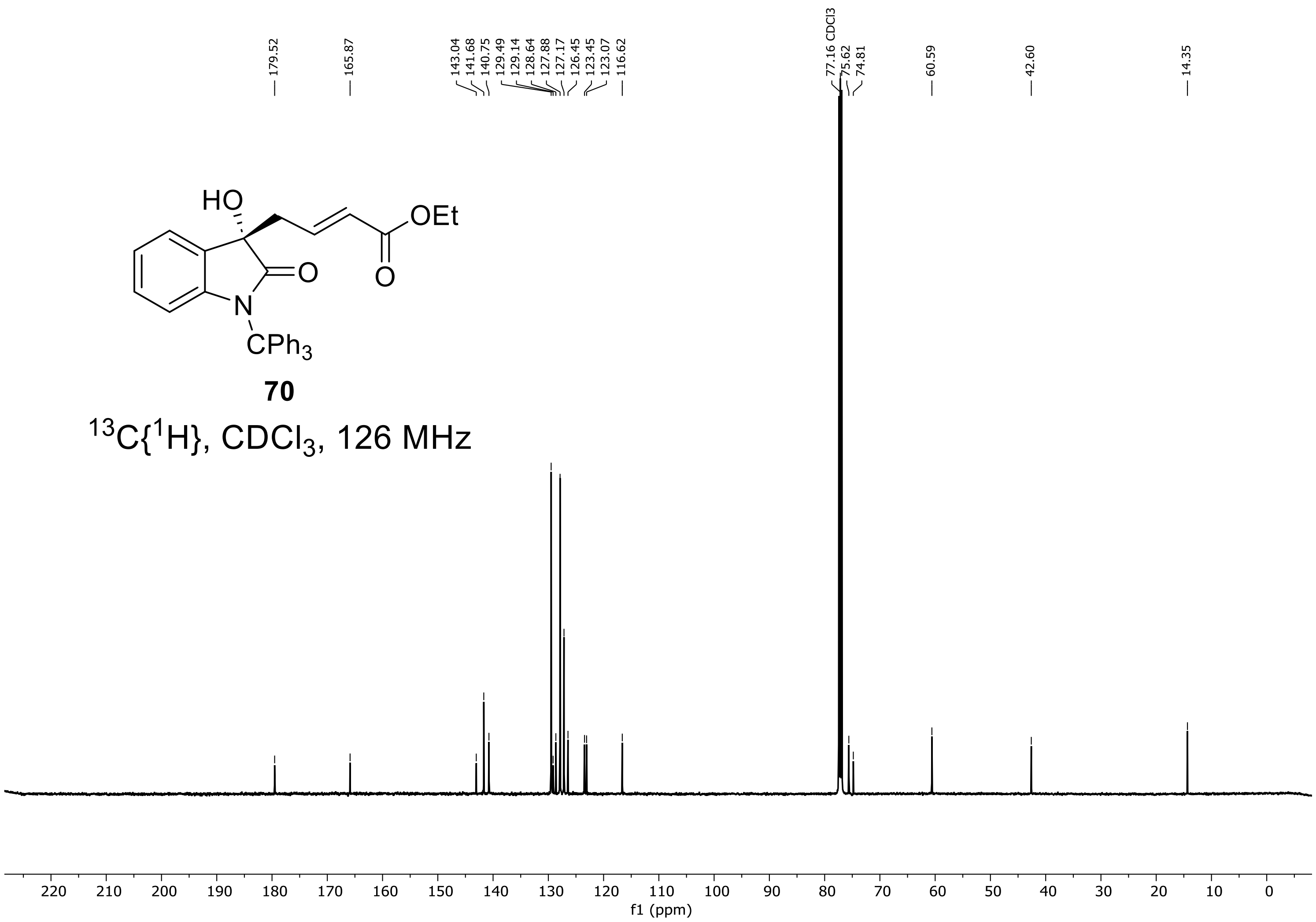

Fig. S455: $^{13}C\{^{1}H\}$ NMR spectrum for ethyl (*E*)-4-(3-hydroxy-2-oxo-1-tritylindolin-3-yl)crotonate (**70**).

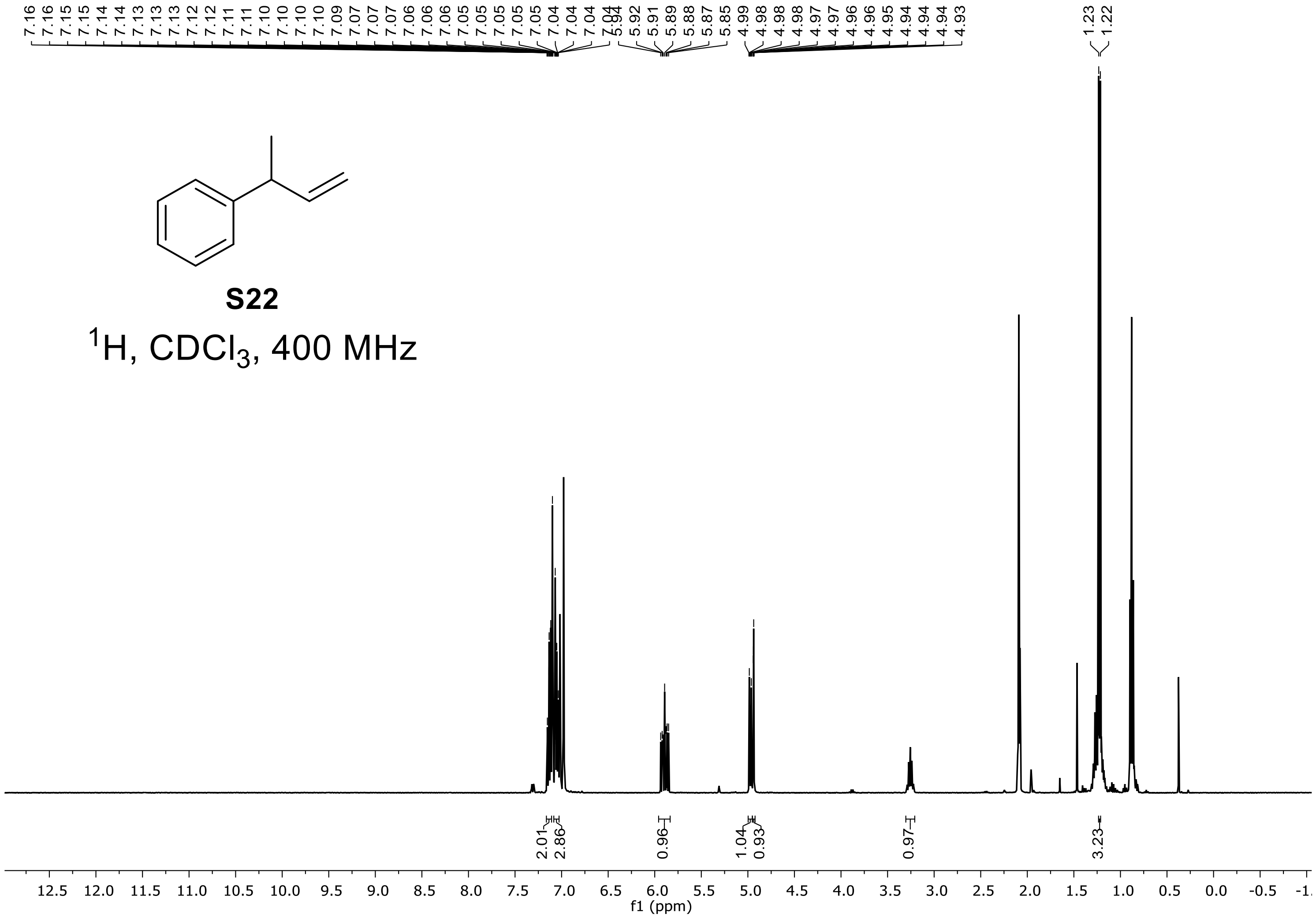

Fig. S456: ^{1}H NMR spectrum for But-3-en-2-ylbenzene (**S22**).

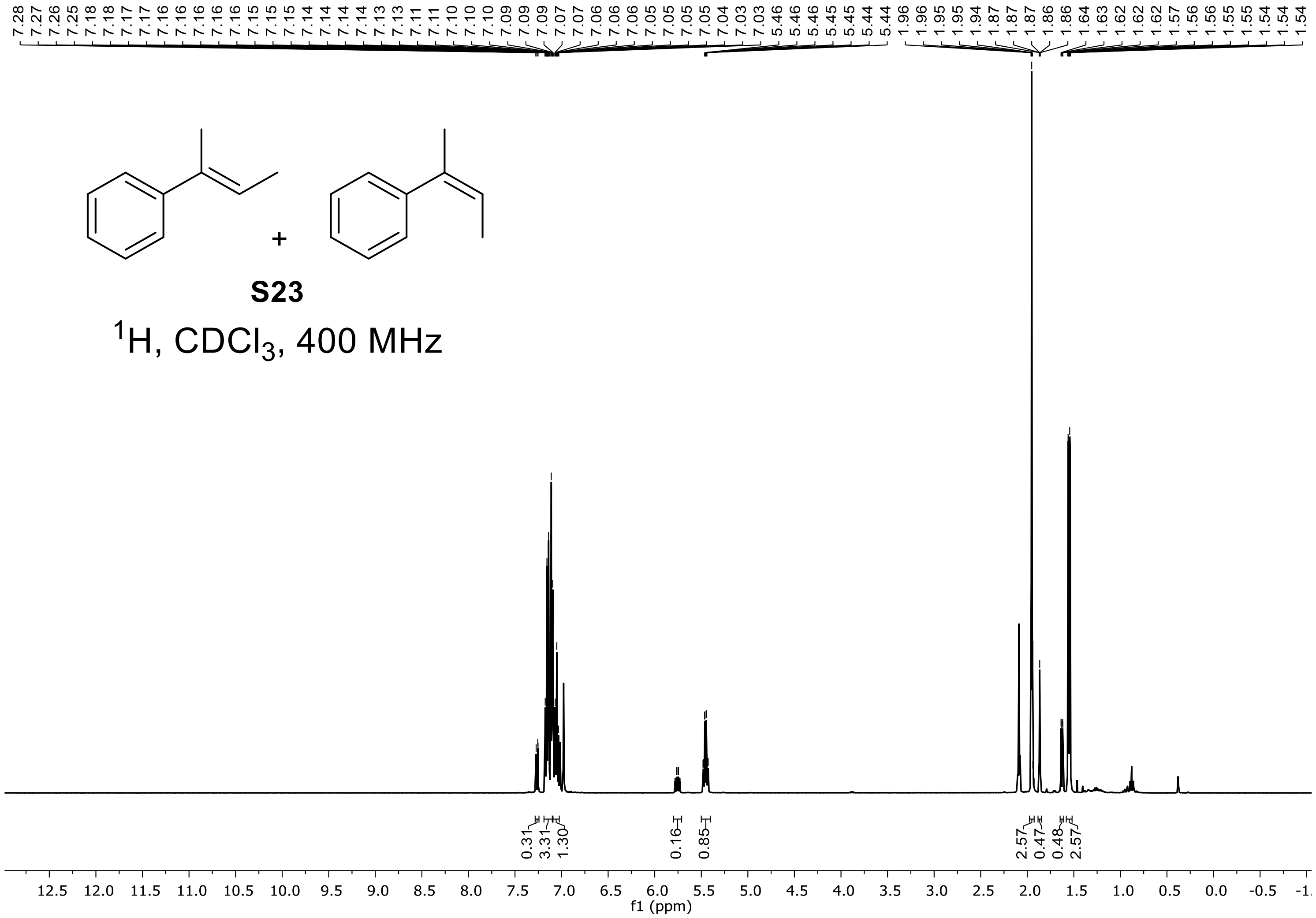

Fig. S457: ^{1}H NMR spectrum for But-2-en-2-ylbenzene (**S23**).

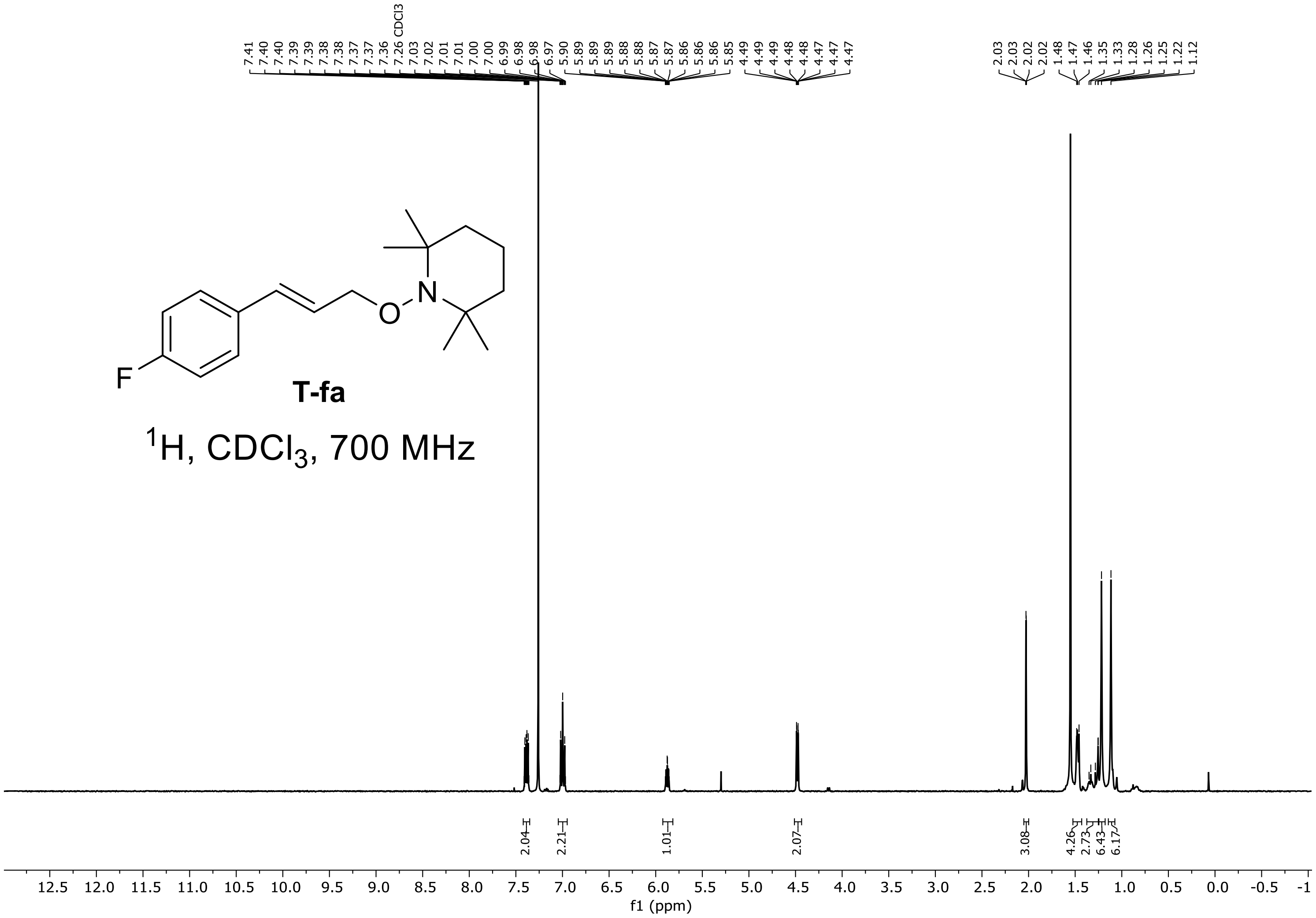

Fig. S458: ^{1}H NMR spectrum for (*E*)-2,2,6,6-tetramethyl-1-((3-(4-fluorophenyl)but-2-en-1-yl)oxy)piperidine (**T-fa**).

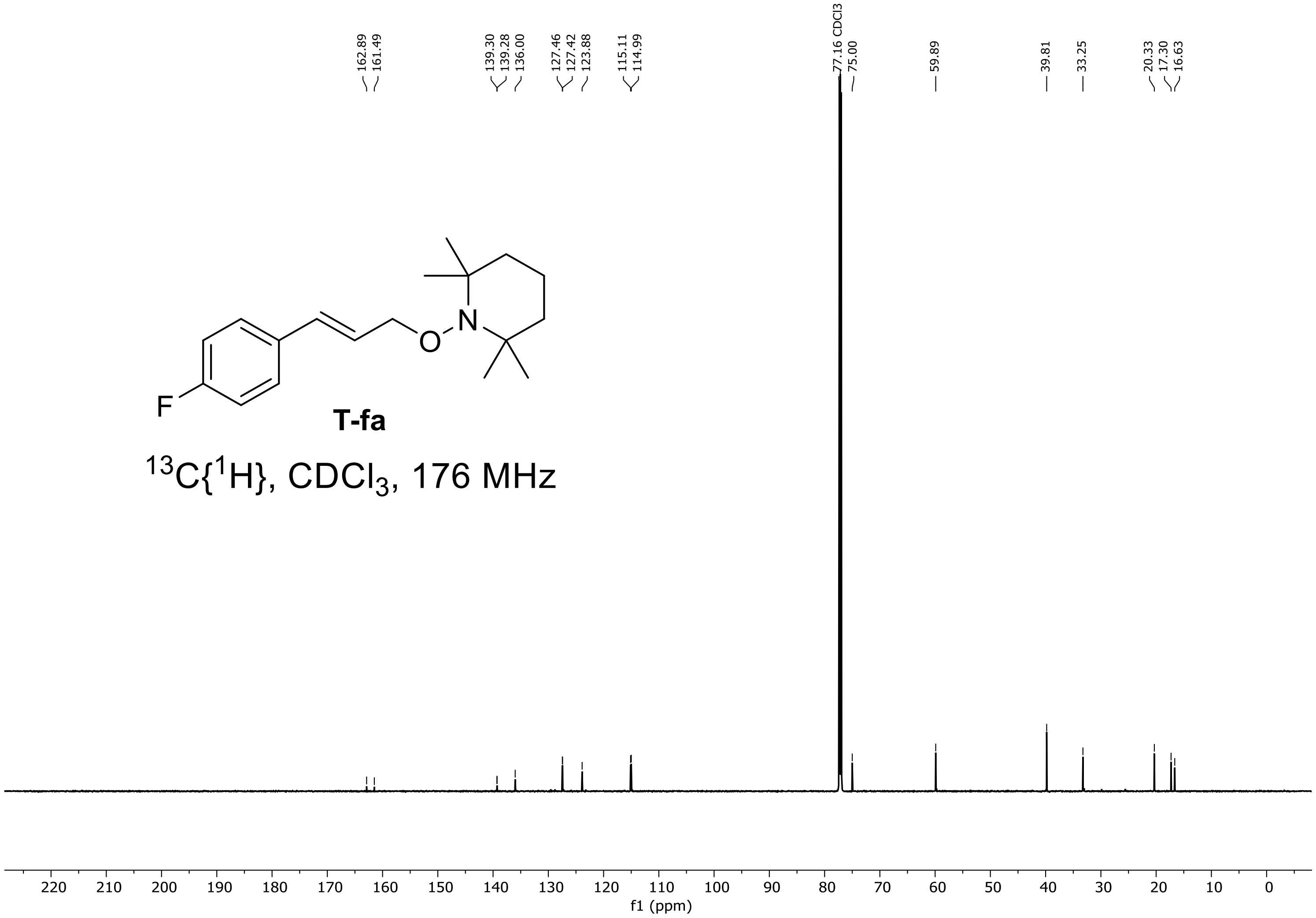

Fig. S459: $^{13}C\{^1H\}$ NMR spectrum for (*E*)-2,2,6,6-tetramethyl-1-((3-(4-fluorophenyl)but-2-en-1-yl)oxy)piperidine (**T-fa**).

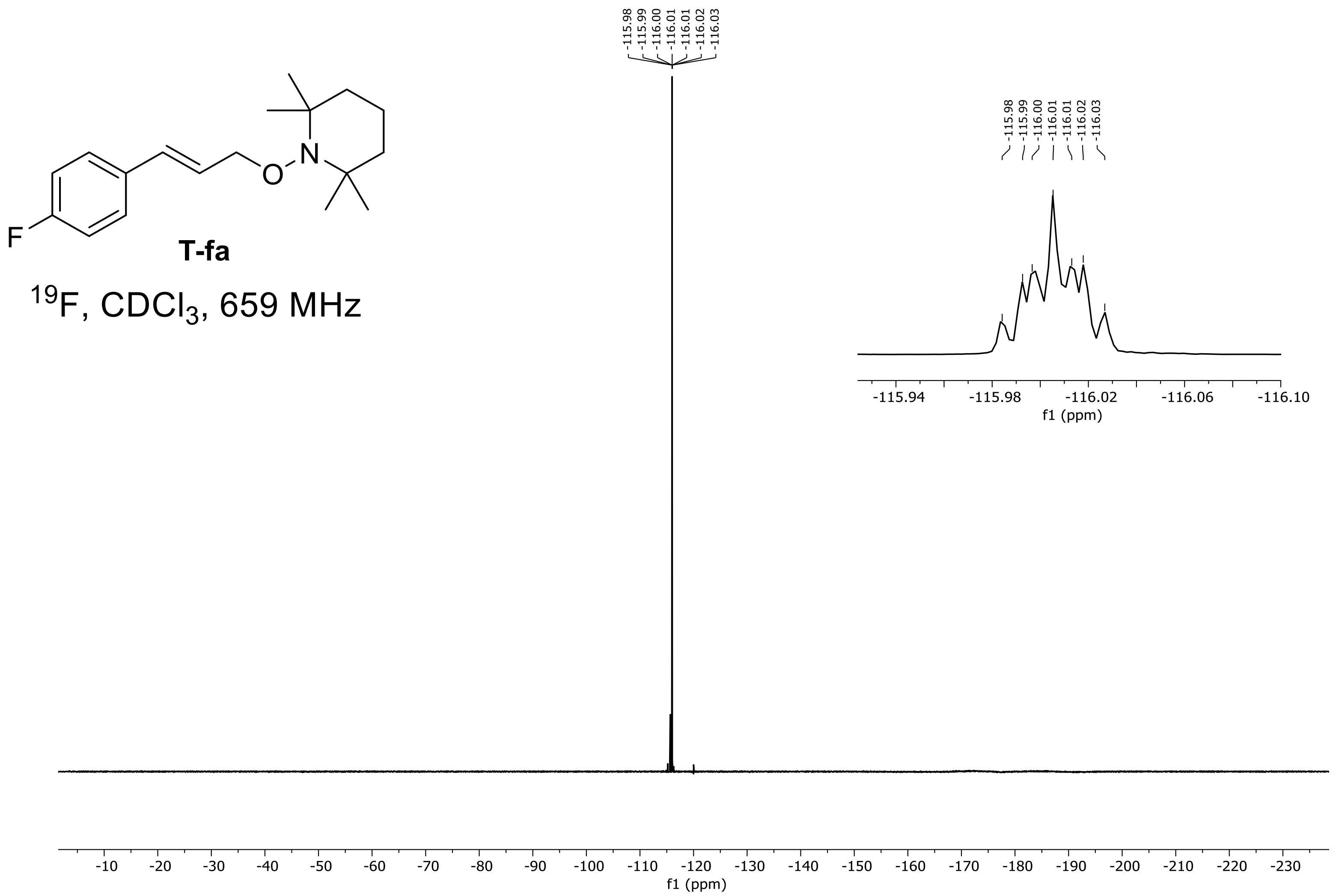

Fig. S460: ^{19}F NMR spectrum for (*E*)-2,2,6,6-tetramethyl-1-((3-(4-fluorophenyl)but-2-en-1-yl)oxy)piperidine (**T-fa**).

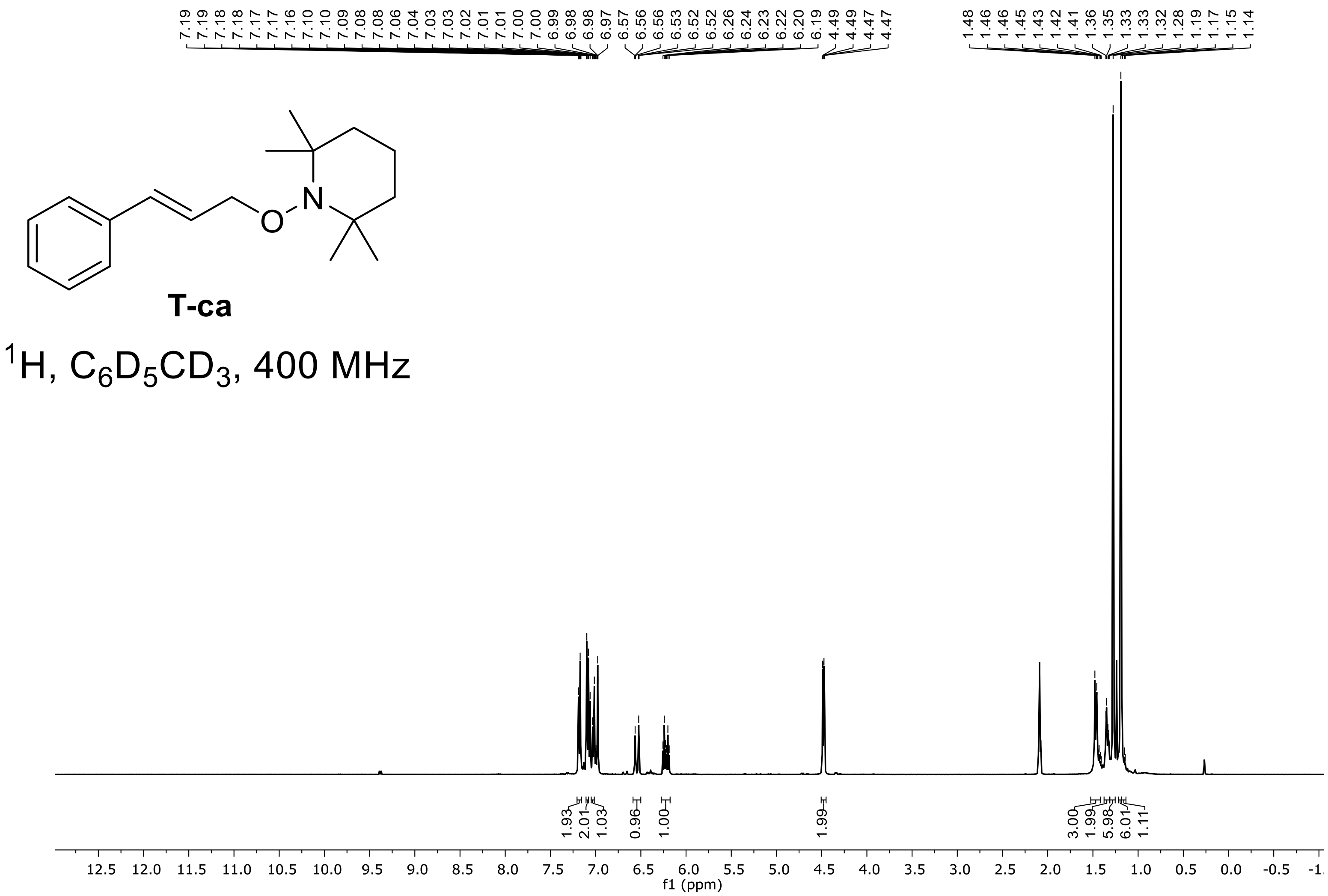

Fig. S461: ^1H NMR spectrum for (*E*)-1-(cinnamyloxy)-2,2,6,6-tetramethylpiperidine (**T-ca**).

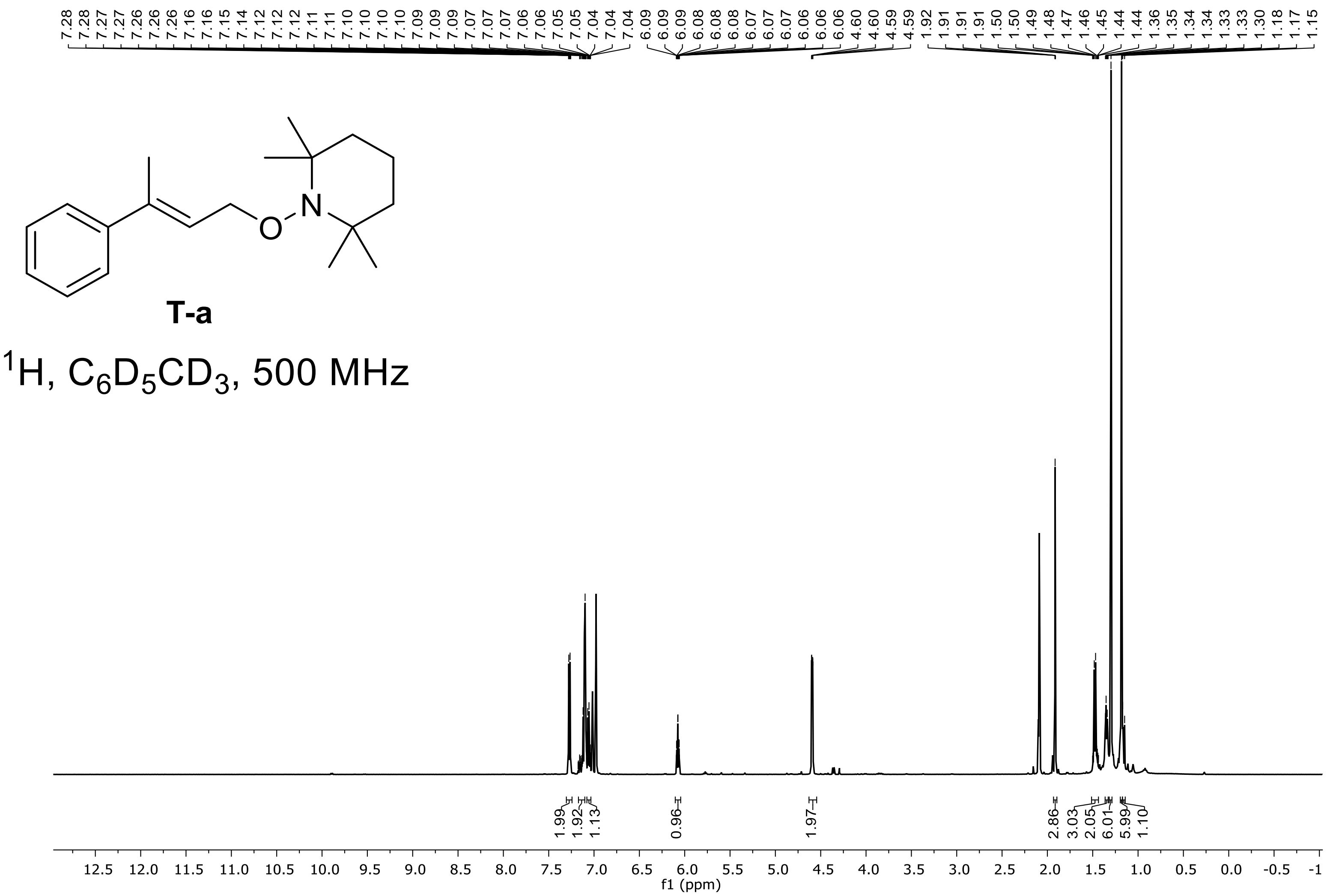

Fig. S462: ^{1}H NMR spectrum for (*E*)-2,2,6,6-tetramethyl-1-((3-phenylbut-2-en-1-yl)oxy)piperidine (**T-a**).

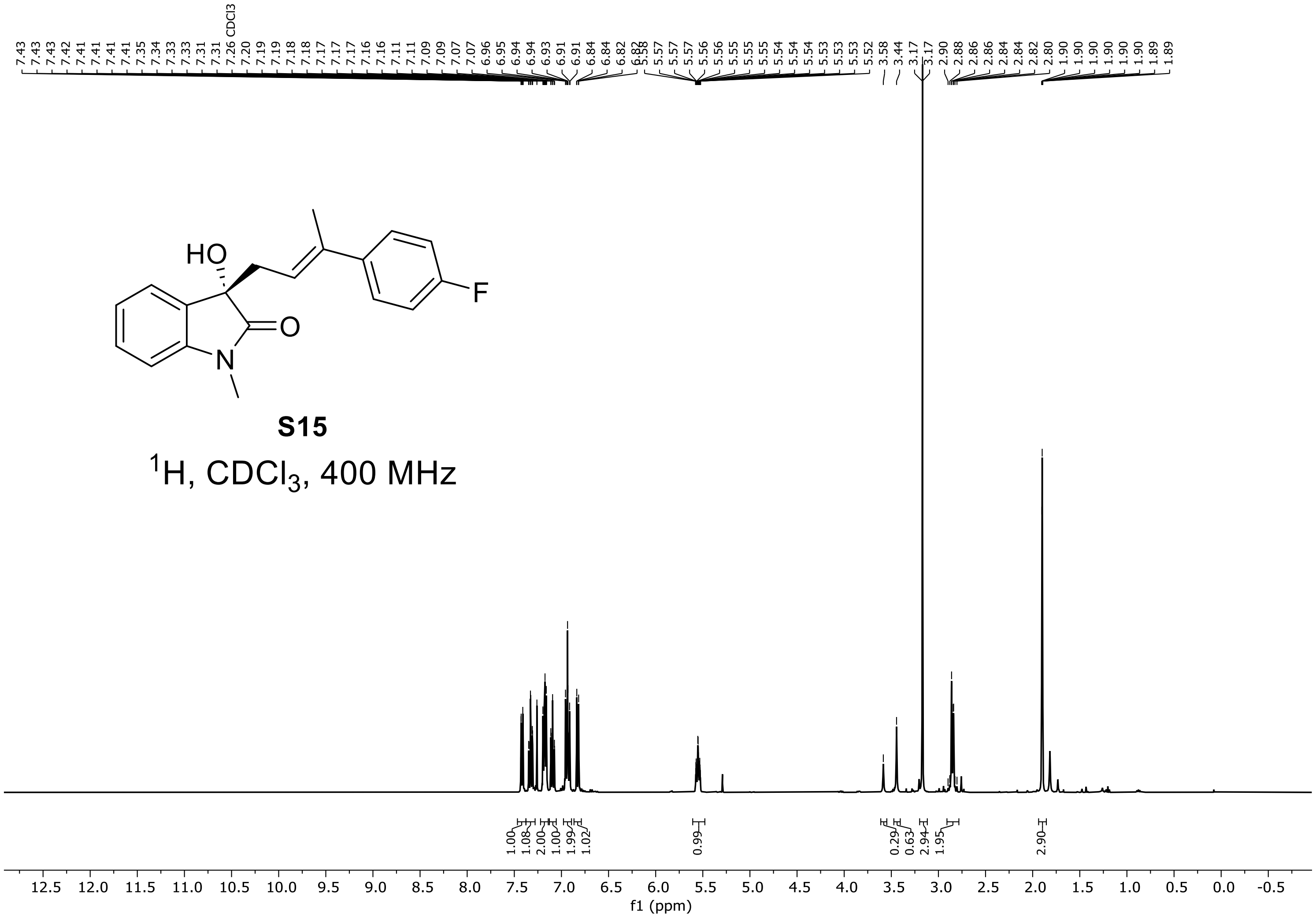

Fig. S463: $^{13}C\{^{1}H\}$ NMR spectrum for (*S*,*E*)-3-(3-(4-fluorophenyl)but-2-en-1-yl)-3-hydroxy-1-methylindolin-2-one (**S15**).

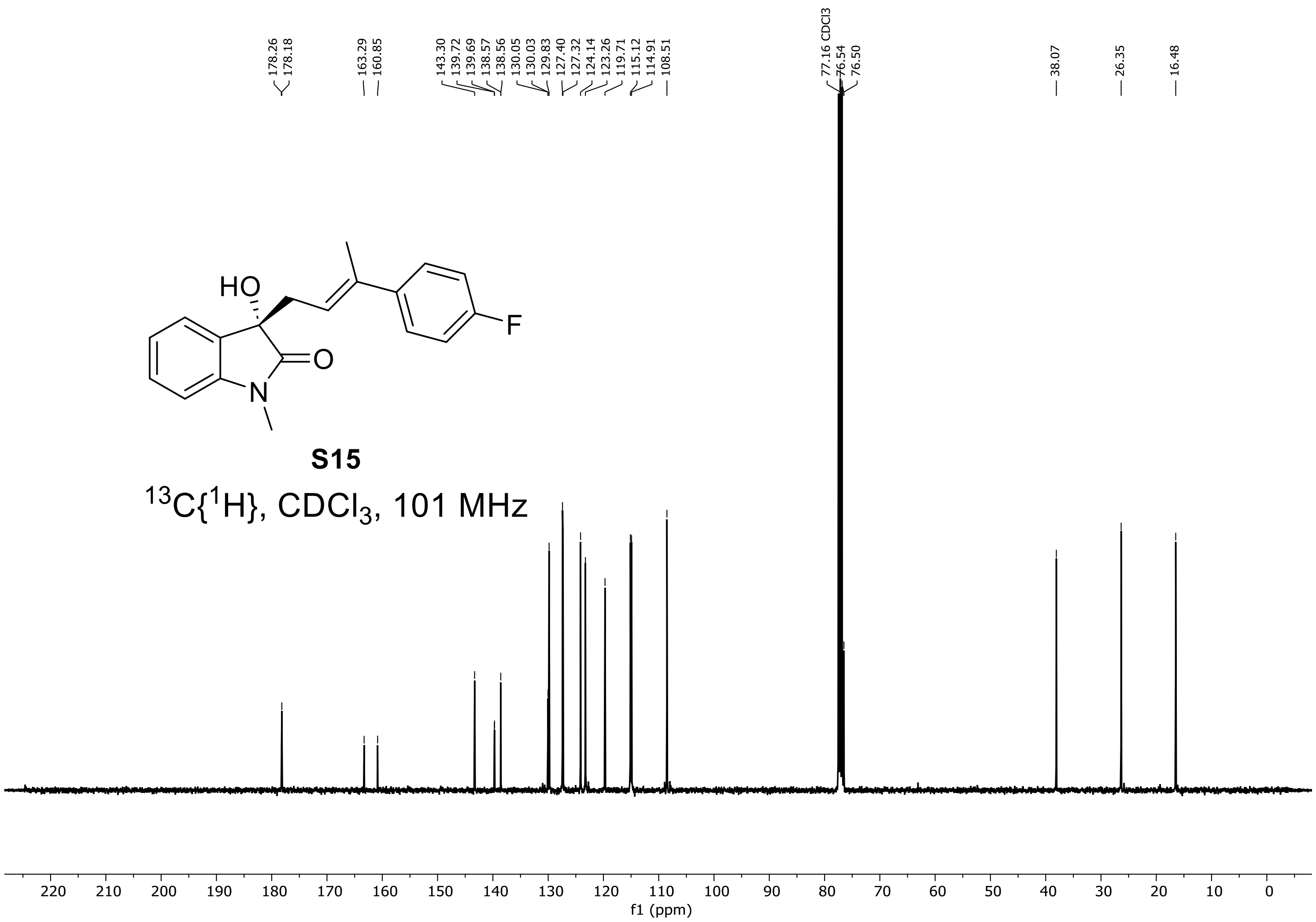

Fig. S464: $^{13}C\{^1H\}$ NMR spectrum for (*S*,*E*)-3-(3-(4-fluorophenyl)but-2-en-1-yl)-3-hydroxy-1-methylindolin-2-one (**S15**).

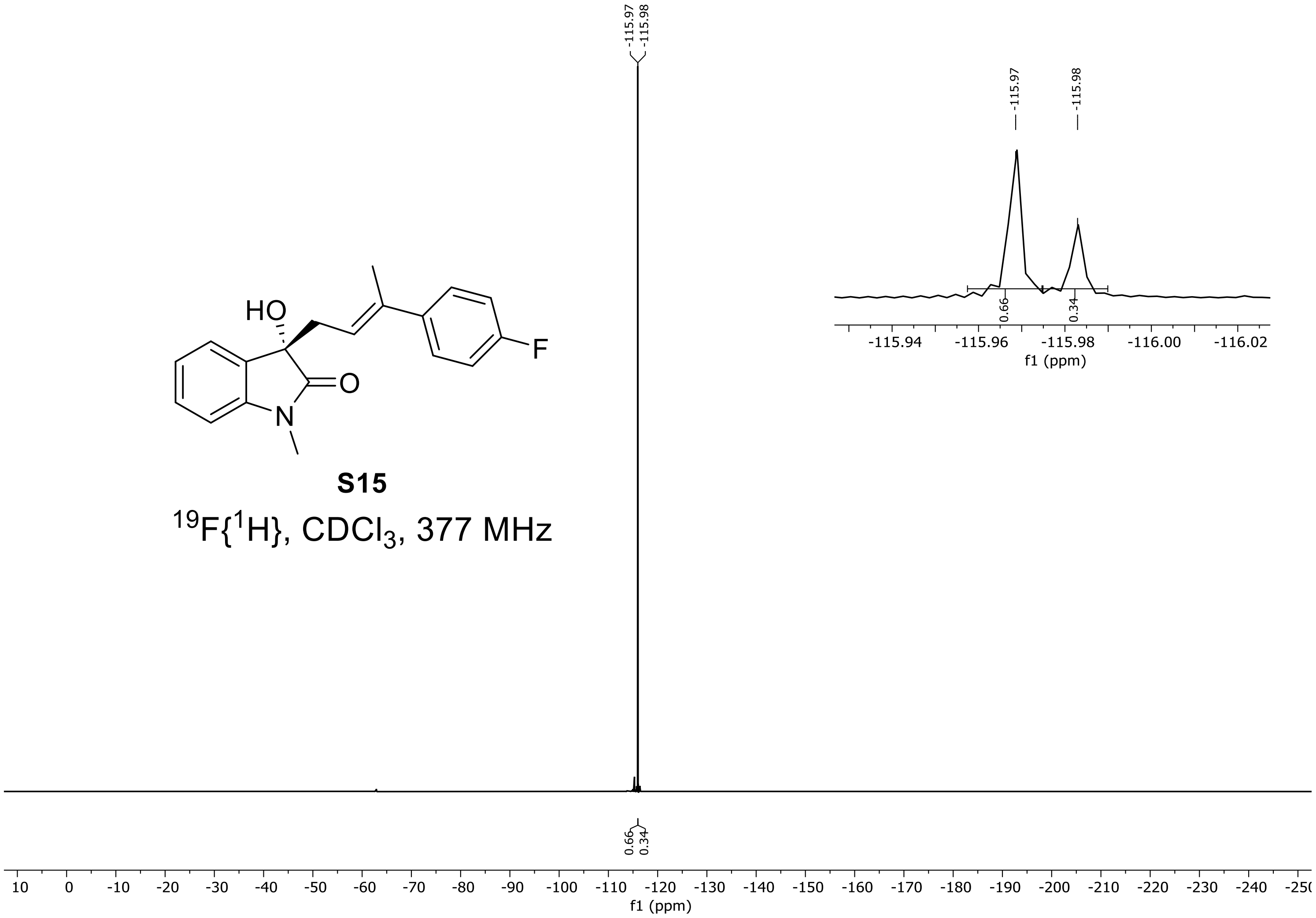

Fig. S465: $^{19}F\{^{1}H\}$ NMR spectrum for (*S*,*E*)-3-(3-(4-fluorophenyl)but-2-en-1-yl)-3-hydroxy-1-methylindolin-2-one (**S15**).

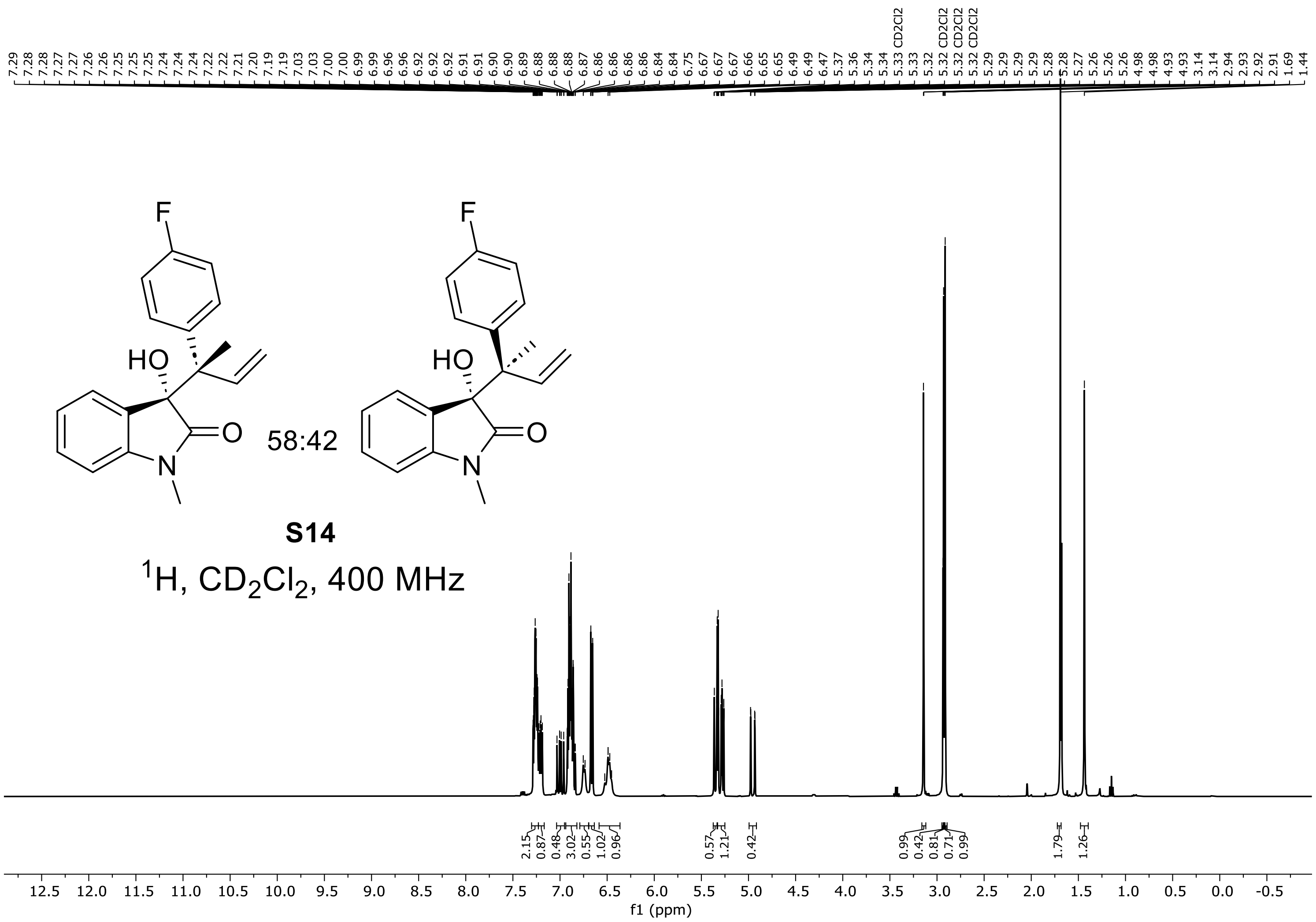

Fig. S468: ^{1}H NMR spectrum for (3S,2'*S*)- and (3*S*,2'*R*)-3-(2-(4-fluorophenyl)but-3-en-2-yl)-3-hydroxy-1-methylindolin-2-one (**S14**).

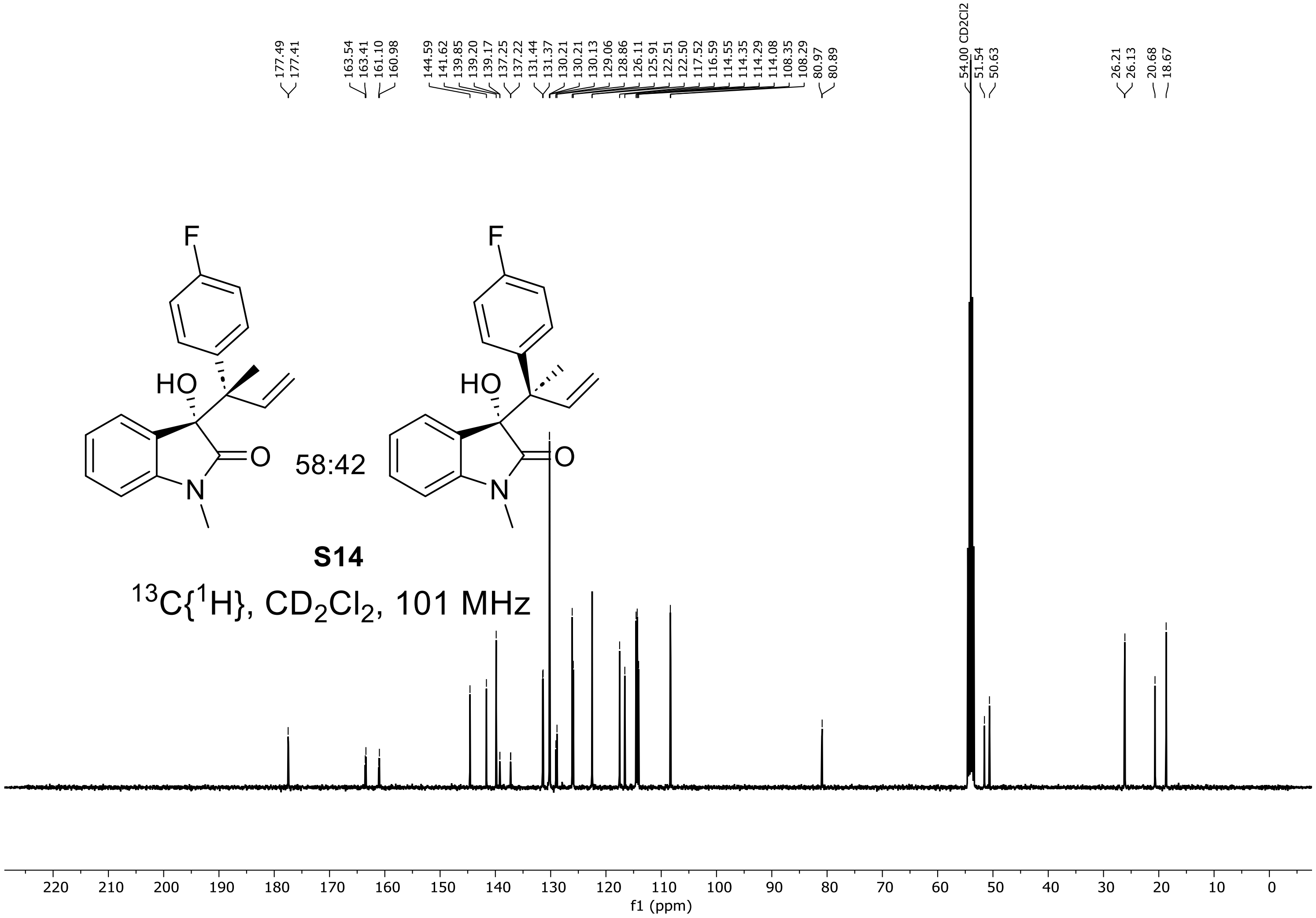

Fig. S467: $^{13}C\{^{1}H\}$ NMR spectrum for (3*S*,2'*S*)- and (3*S*,2'*R*)-3-(2-(4-fluorophenyl)but-3-en-2-yl)-3-hydroxy-1-methylindolin-2-one (**S14**).

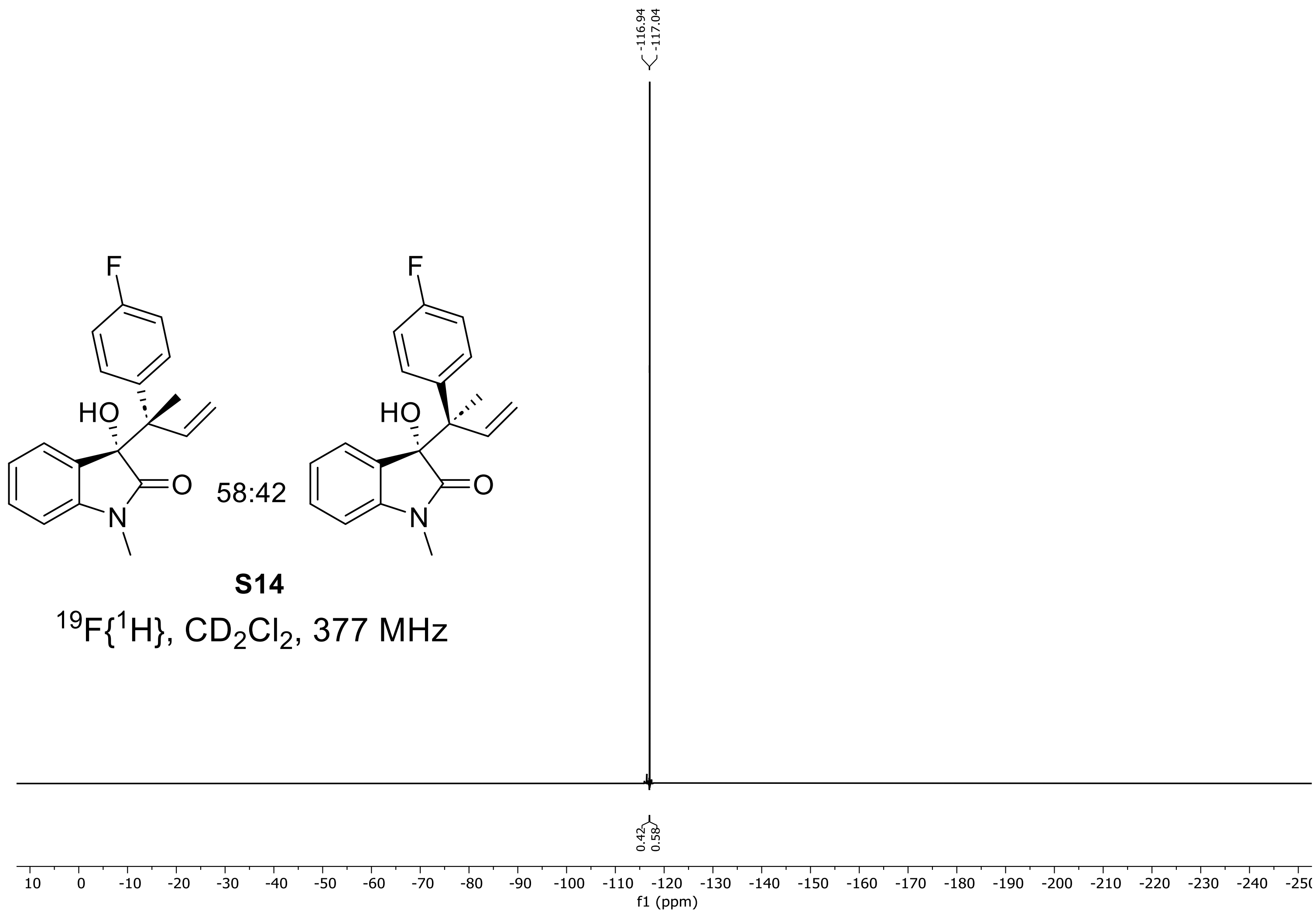

Fig. S468: $^{19}F\{^1H\}$ NMR spectrum for (3*S*,2'*S*)- and (3*S*,2'*R*)-3-(2-(4-fluorophenyl)but-3-en-2-yl)-3-hydroxy-1-methylindolin-2-one (**S14**).

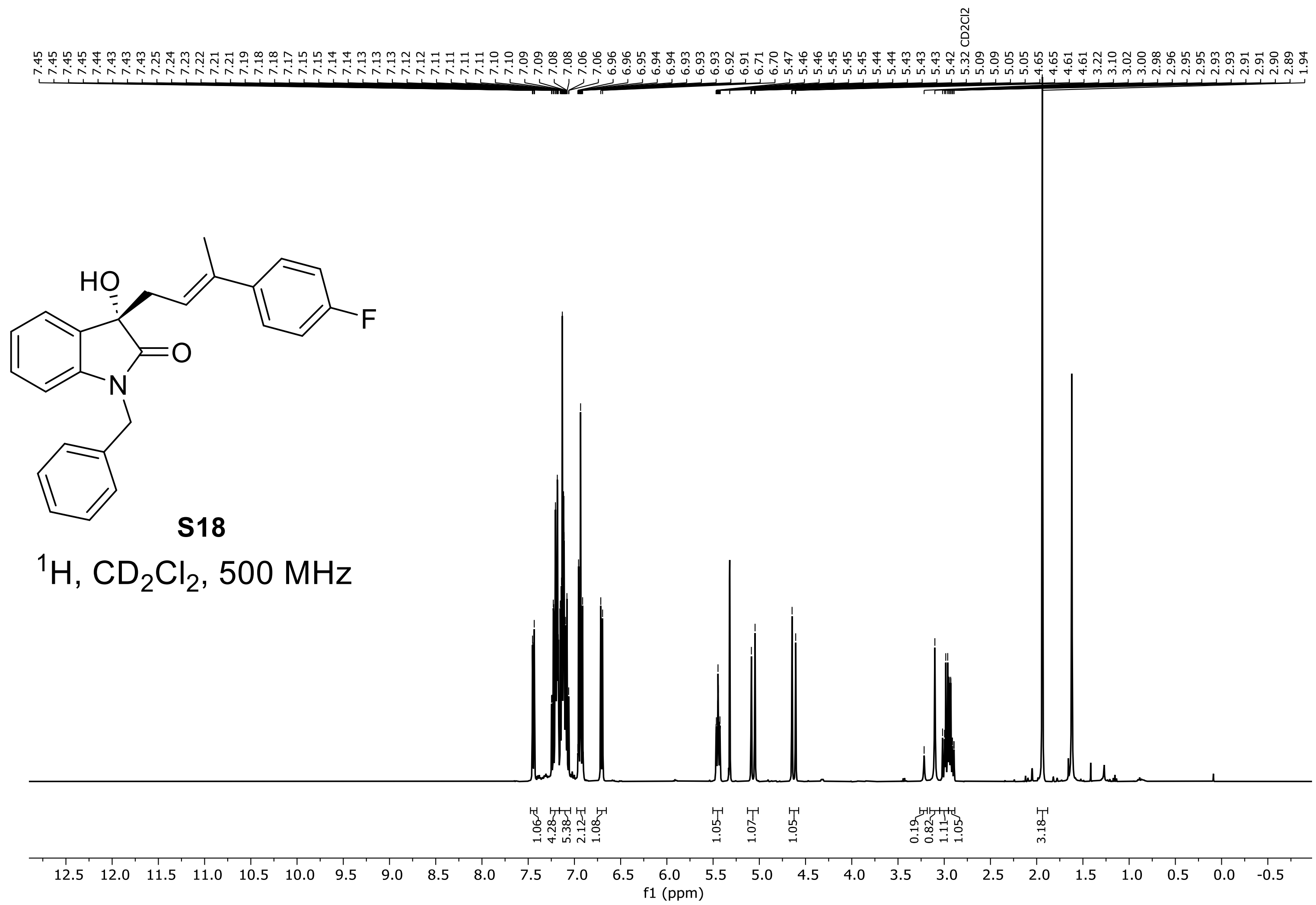

Fig. S469: ^{1}H NMR spectrum for (*S*,*E*)-1-benzyl-3-(3-(4-fluorophenyl)but-2-en-1-yl)-3-hydroxyindolin-2-one (**S18**).

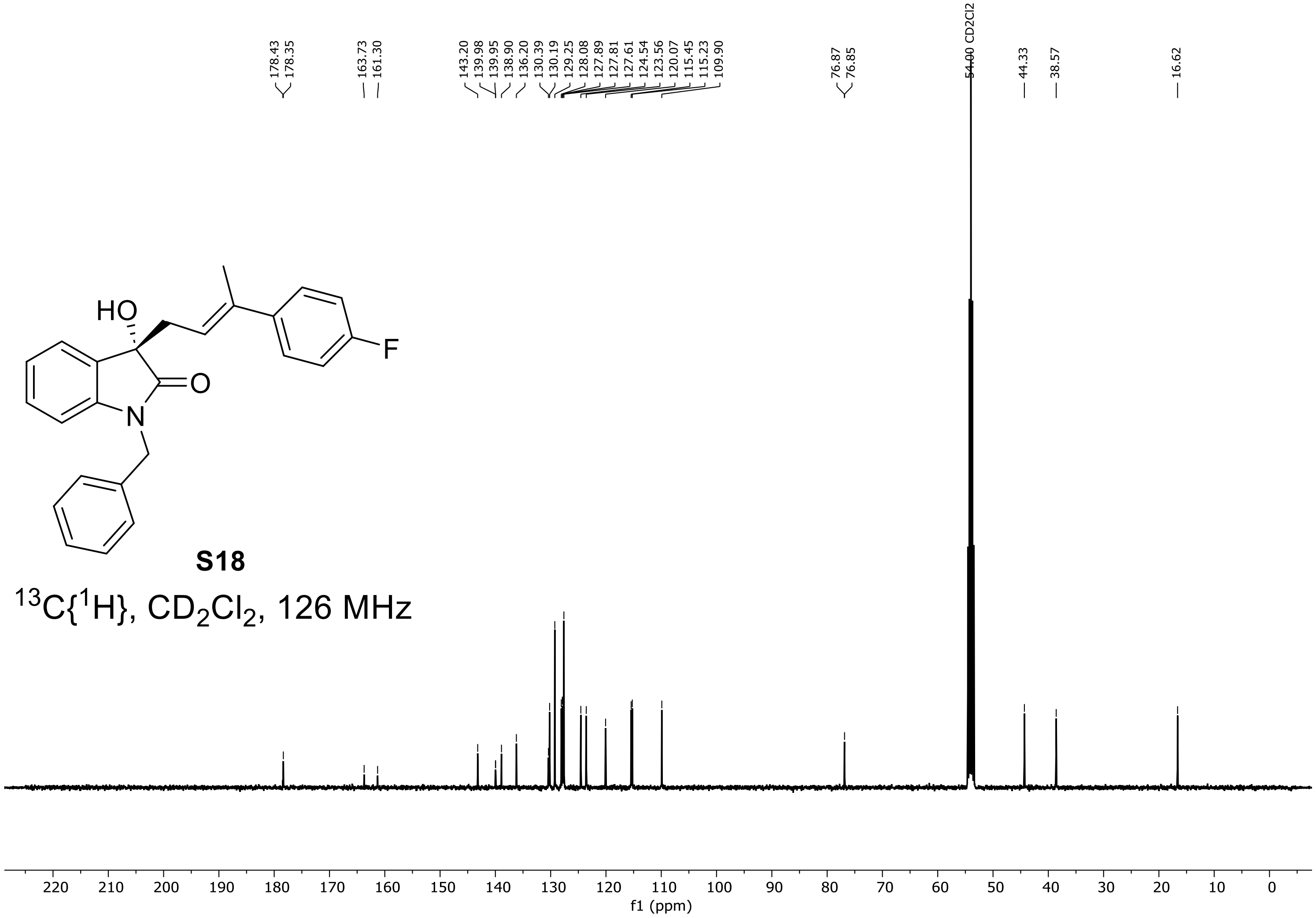

Fig. S470: $^{13}C\{^{1}H\}$ NMR spectrum for (*S*,*E*)-1-benzyl-3-(3-(4-fluorophenyl)but-2-en-1-yl)-3-hydroxyindolin-2-one (**S18**).

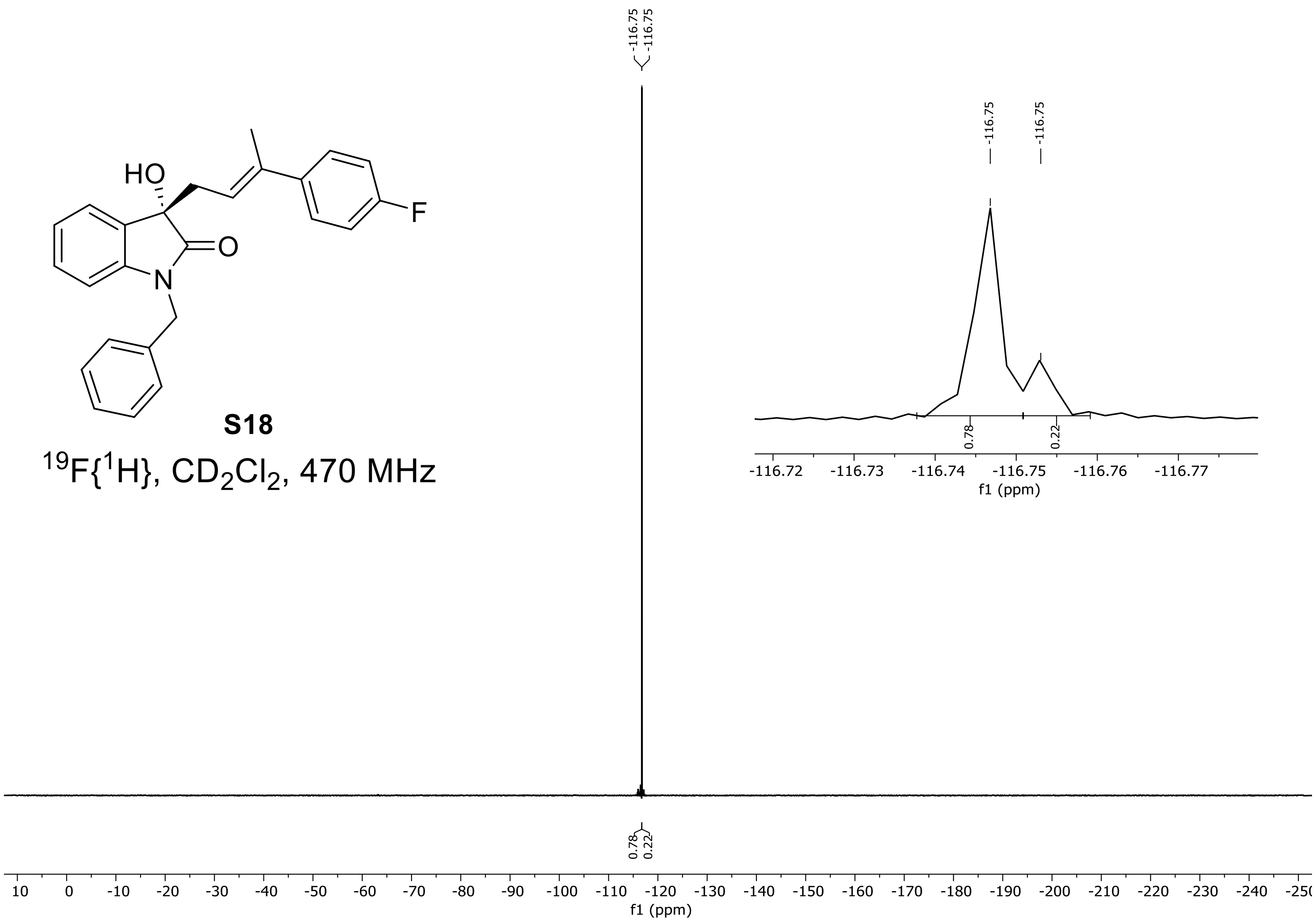

Fig. S471: $^{19}F\{^1H\}$ NMR spectrum for (*S*,*E*)-1-benzyl-3-(3-(4-fluorophenyl)but-2-en-1-yl)-3-hydroxyindolin-2-one (**S18**).

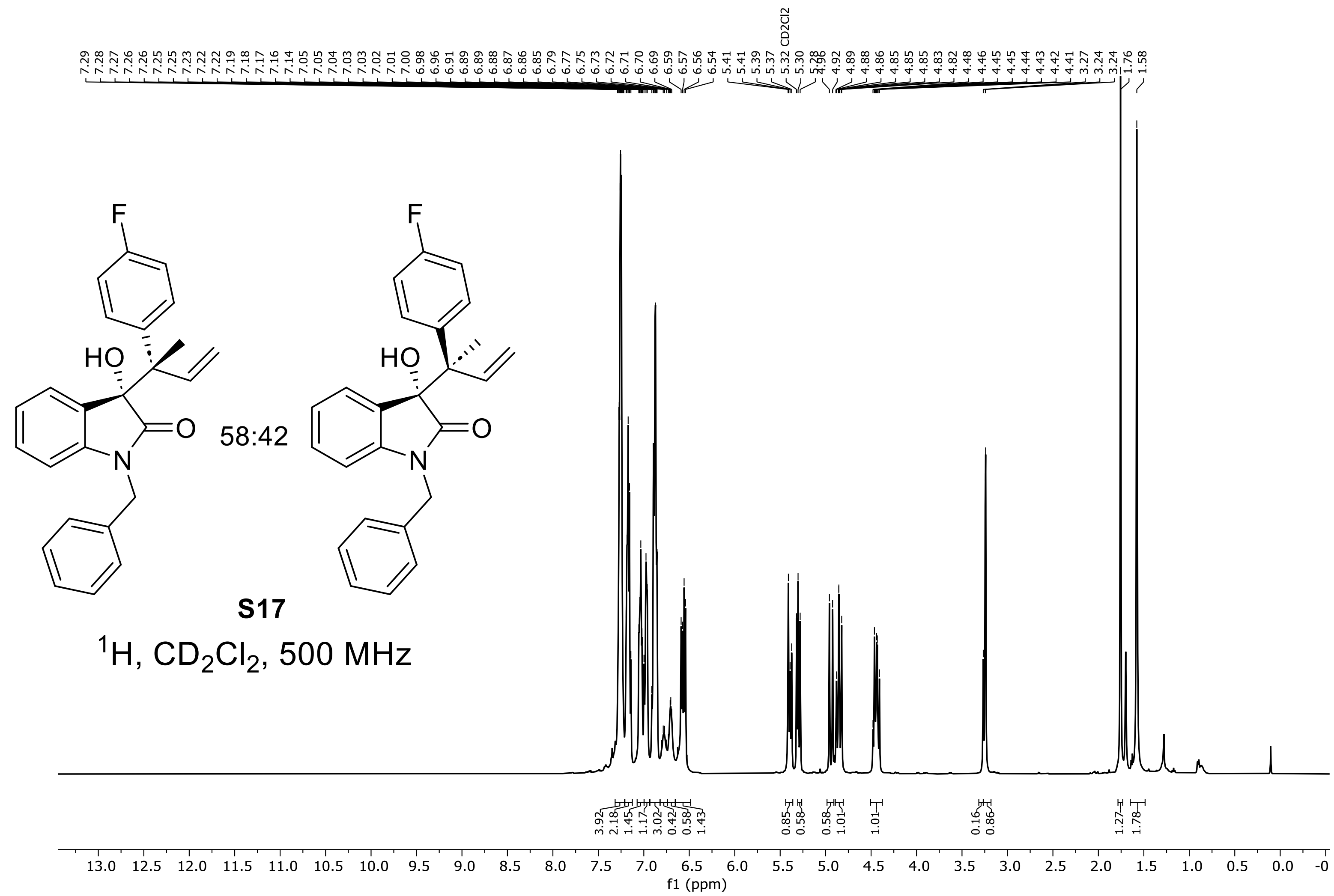

Fig. S472: ^{1}H NMR spectrum for (3*S*,2'*S*)- and (3*S*,2'*R*)-1-benzyl-3-(2-(4-fluorophenyl)but-3-en-2-yl)-3-hydroxyindolin-2-one (**S17**).

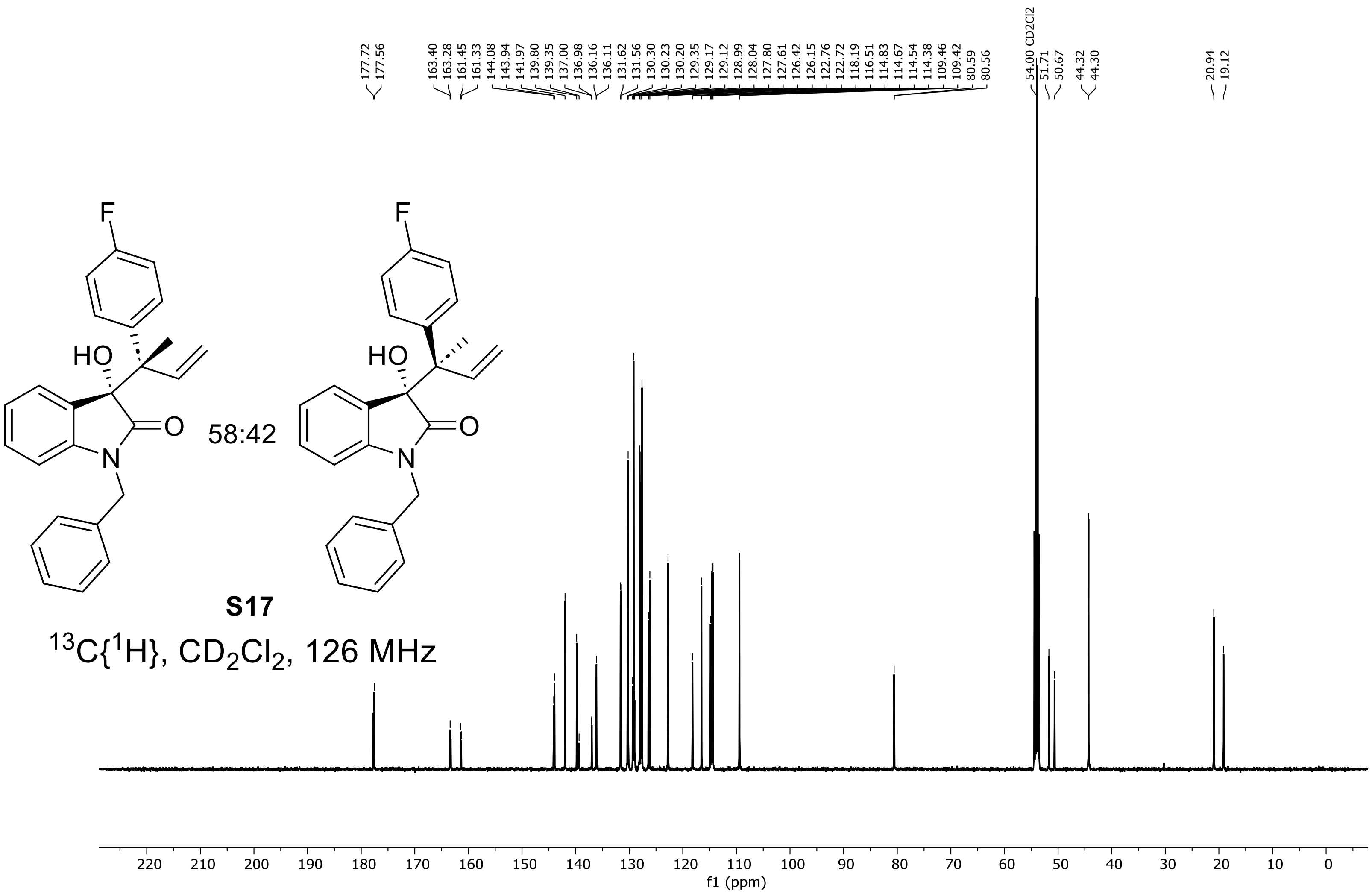

Fig. S473: $^{13}C\{^1H\}$ NMR spectrum for (3*S*,2'*S*)- and (3*S*,2'*R*)-1-benzyl-3-(2-(4-fluorophenyl)but-3-en-2-yl)-3-hydroxyindolin-2-one (**S17**).

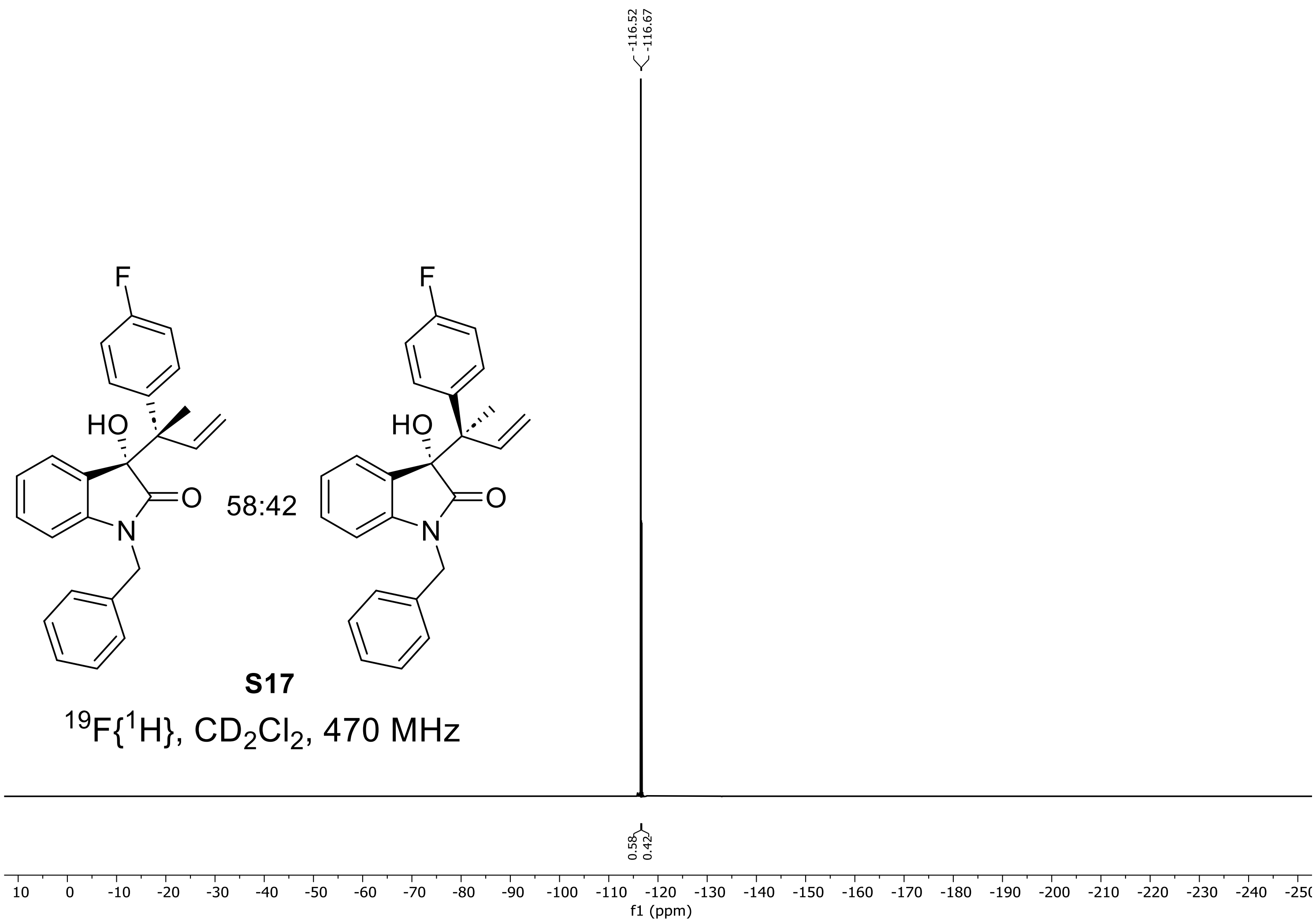

Fig. S474: $^{19}F\{^{1}H\}$ NMR spectrum for (3*S*,2'*S*)- and (3*S*,2'*R*)-1-benzyl-3-(2-(4-fluorophenyl)but-3-en-2-yl)-3-hydroxyindolin-2-one (**S17**).

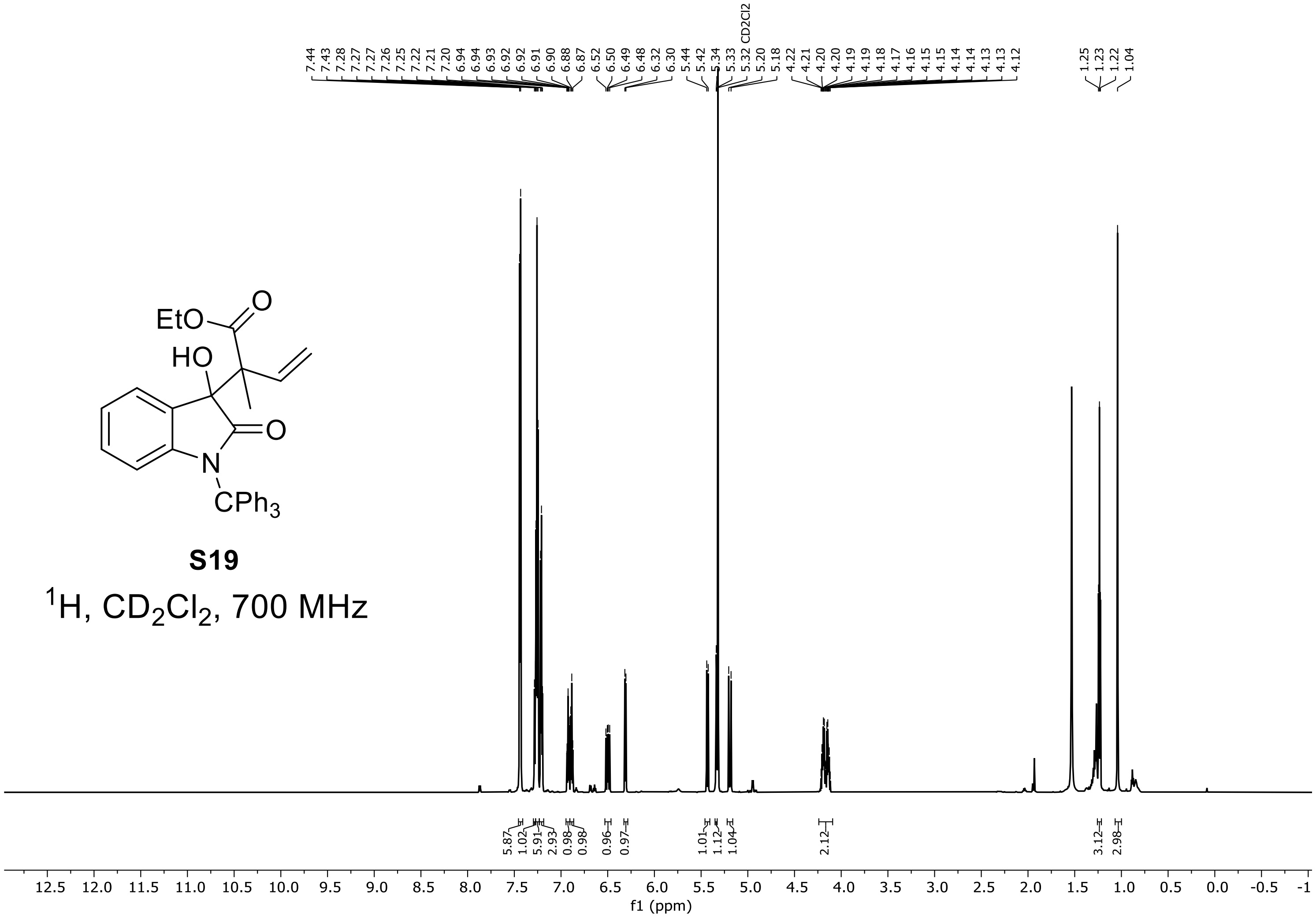

Fig. S475: ^{1}H NMR spectrum for ethyl (3*R*,2'*S*)-2-(3-hydroxy-2-oxo-1-tritylindolin-3-yl)-2-methylbut-3-enoate (**S19**).

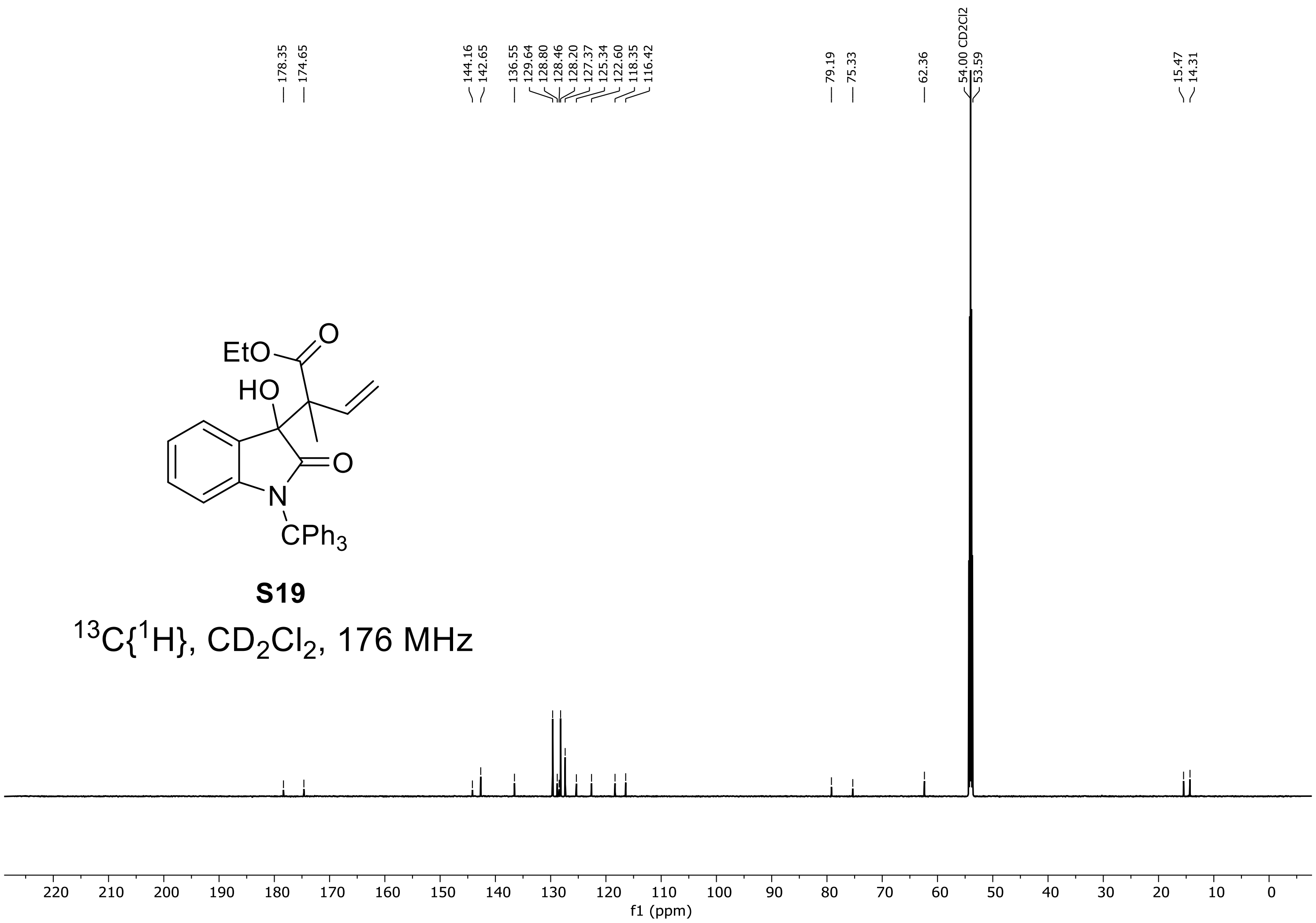

Fig. S476: $^{13}C\{^1H\}$ NMR spectrum for ethyl (3*R*,2''*S*)-2-(3-hydroxy-2-oxo-1-tritylindolin-3-yl)-2-methylbut-3-enoate (**S19**).

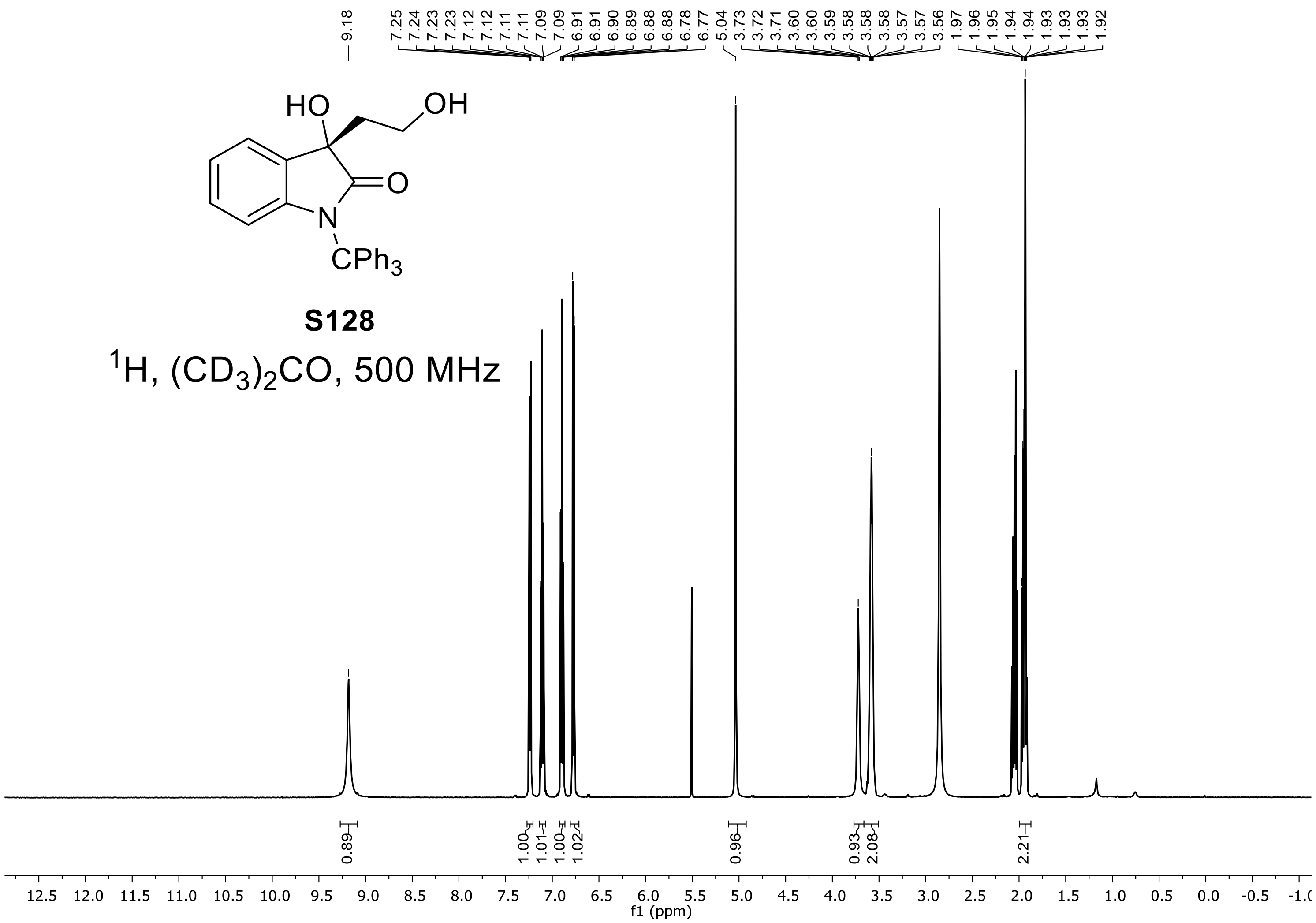

Fig. S477: ^{1}H NMR spectrum for (S)-3-Hydroxy-3-(2-hydroxyethyl)indolin-2-one (**S128**).

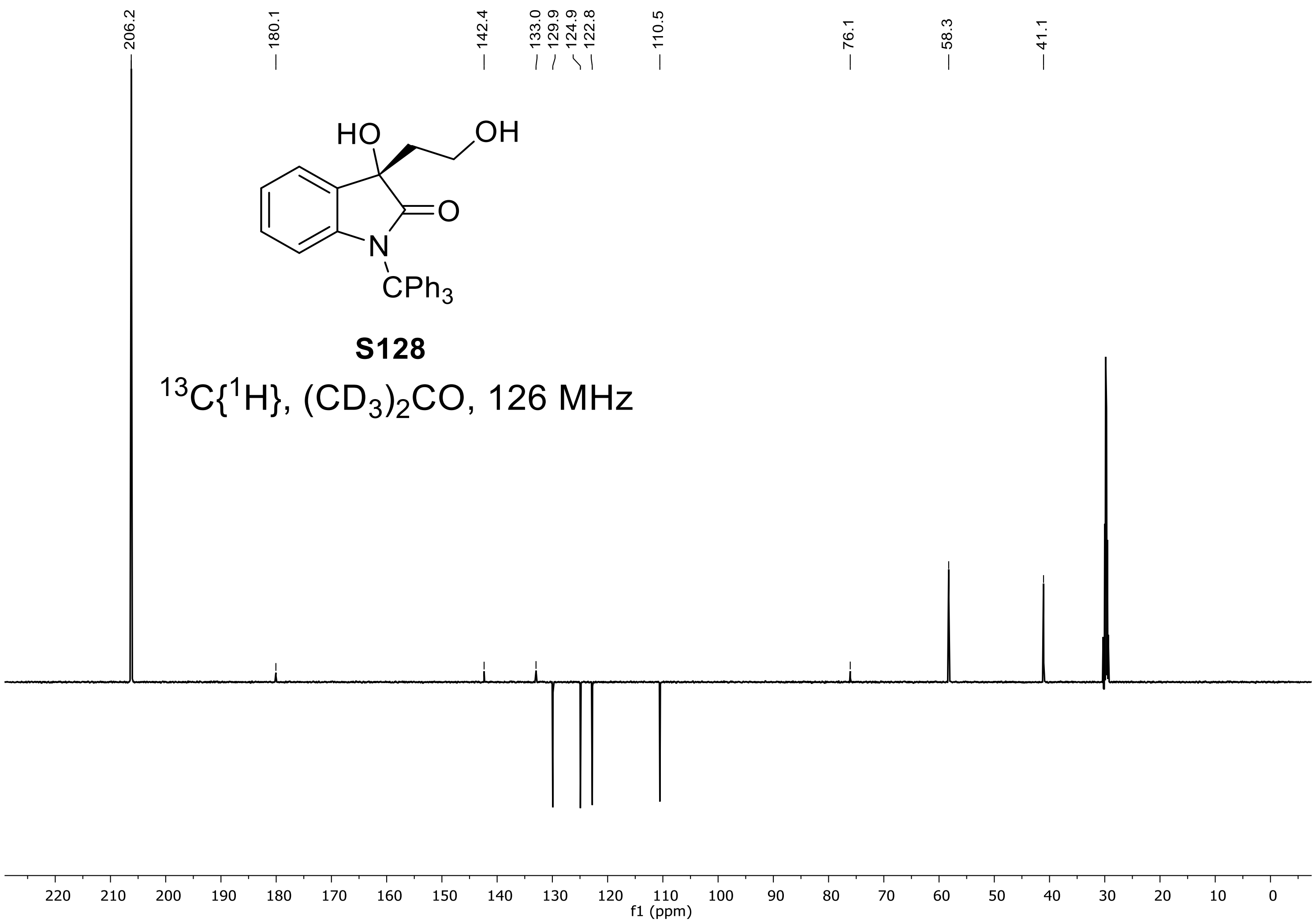

Fig. S478: $^{13}C\{^1H\}$ NMR spectrum for (*S*)-3-Hydroxy-3-(2-hydroxyethyl)indolin-2-one (**S128**).

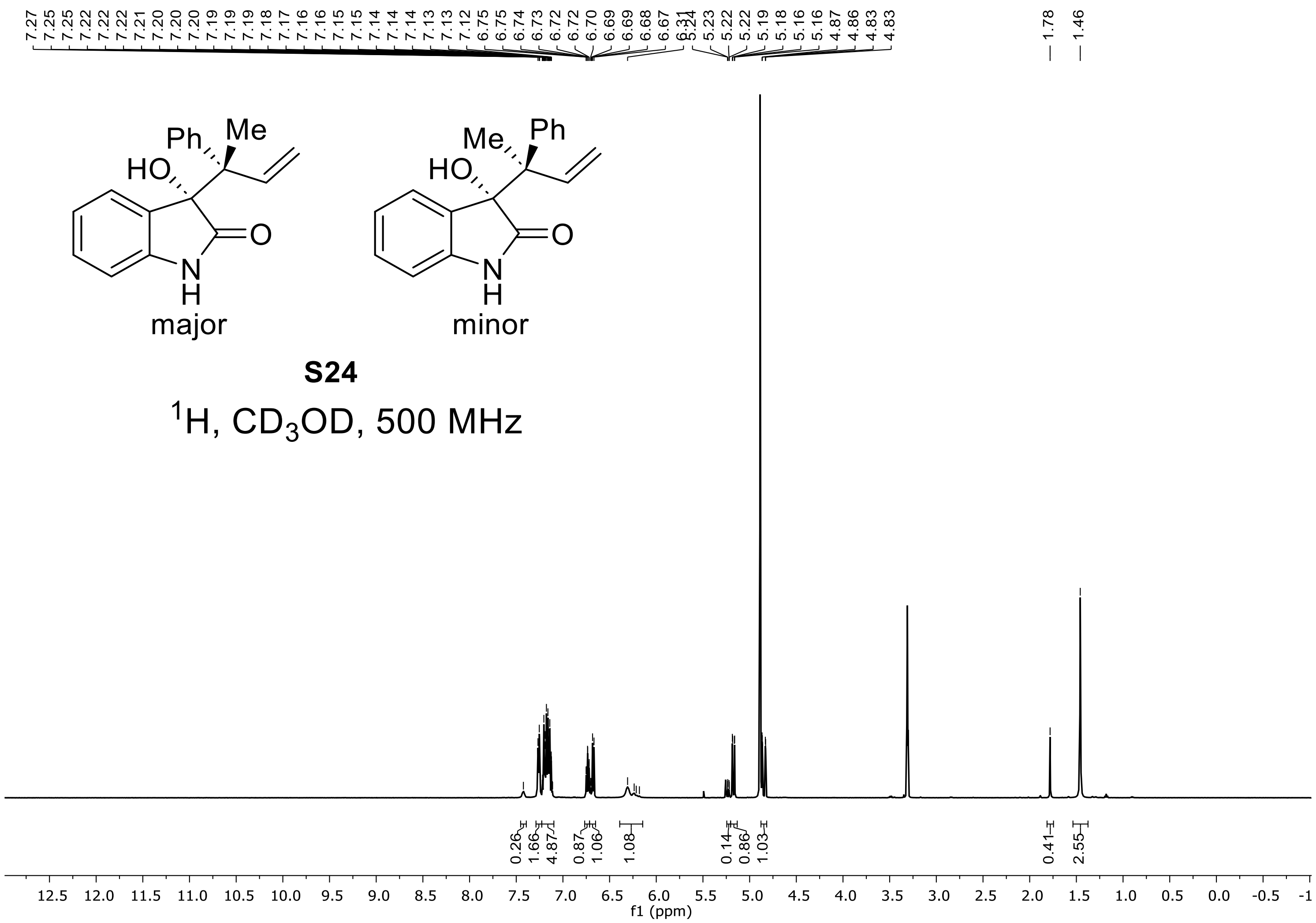

Fig. S479: ^{1}H NMR spectrum for (3*S*, 2'*S*)- and (3*S*,2'*R*)-3-Hydroxy-3-(2-phenylbut-3-en-2-yl)indolin-2-one (**S24**).

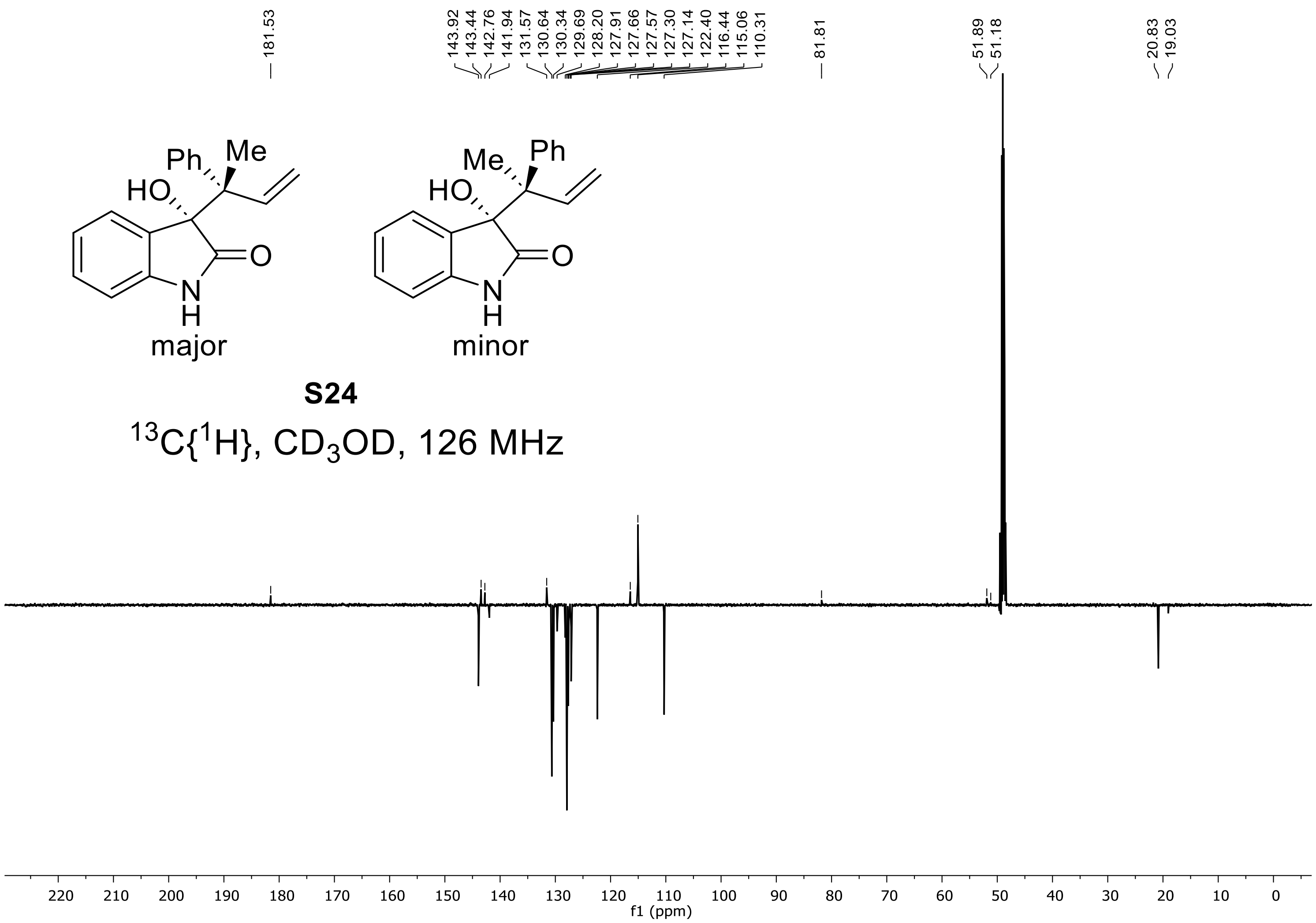

Fig. S480: $^{13}C\{^{1}H\}$ NMR spectrum for (3*S*, 2'*S*)- and (3*S*,2'*R*)-3-Hydroxy-3-(2-phenylbut-3-en-2-yl)indolin-2-one (**S24**).

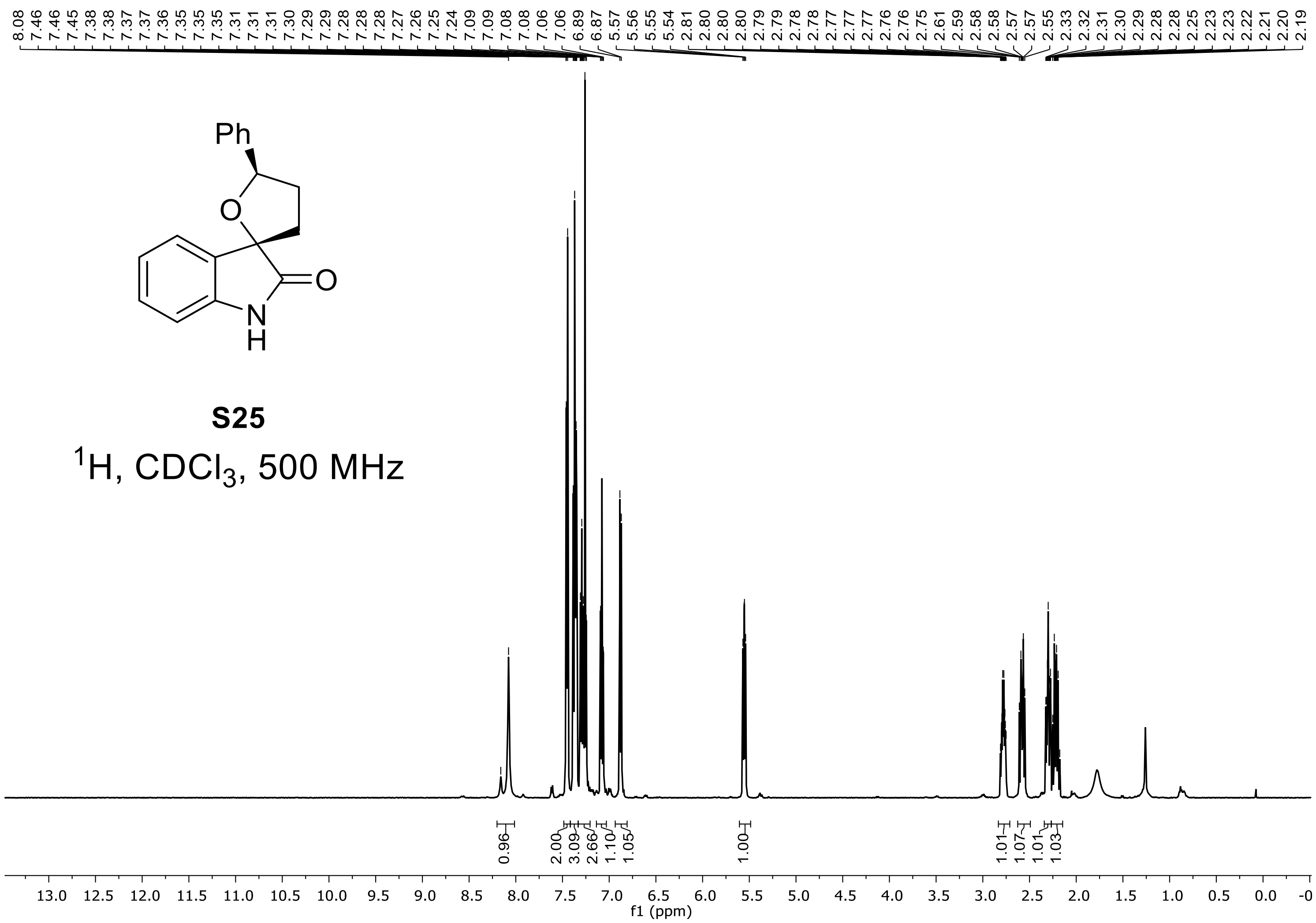

Fig. S481: ^{1}H NMR spectrum for (5*R*,3'*S*)-5-Phenyl-4,5-dihydro-3H-spiro[furan-2,3'-indolin]-2'-one (**S25**).

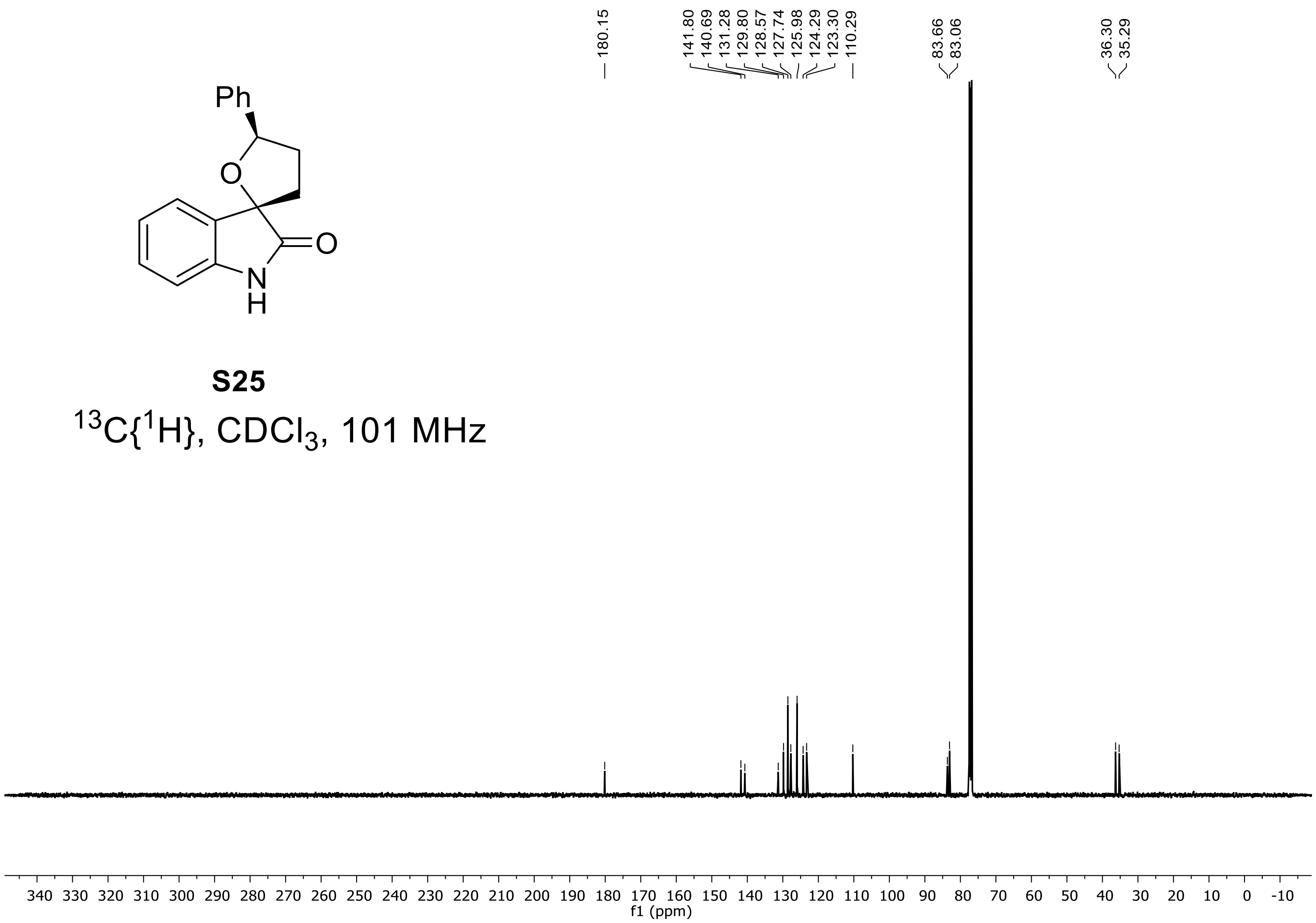

S25

$^{13}C\{^1H\}$, $CDCl_3$, 101 MHz

Fig. S482: $^{13}C\{^1H\}$ NMR spectrum for (5*R*,3’*S*)-5-Phenyl-4,5-dihydro-3H-spiro[furan-2,3'-indolin]-2'-one (**S25**).

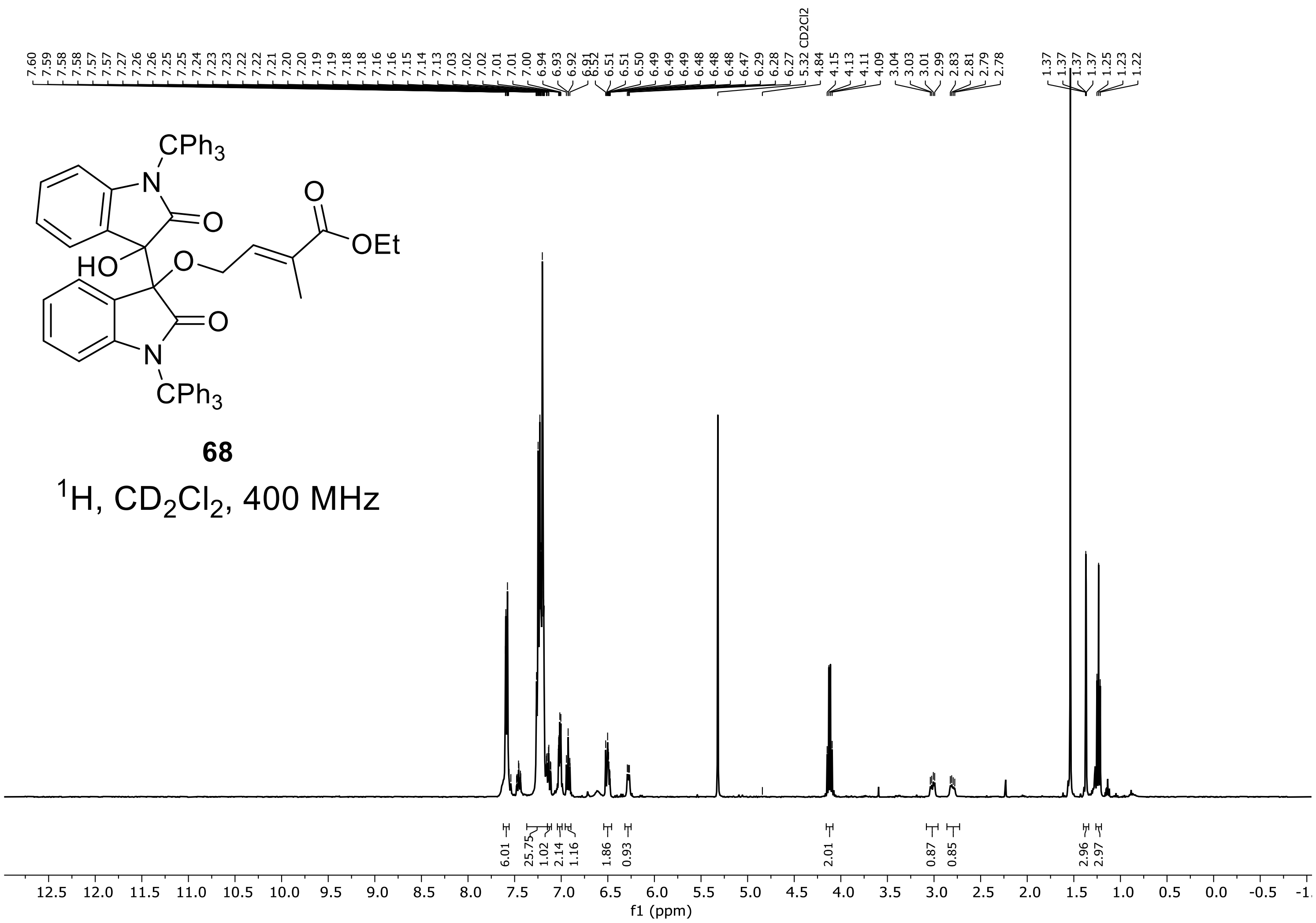

Fig. S483: ^{1}H NMR spectrum for Ethyl (*E*)-4-((3-hydroxy-2,2'-dioxo-1,1'-ditrityl-[3,3'-biindolin]-3'-yl)oxy)tiglate (**68**).

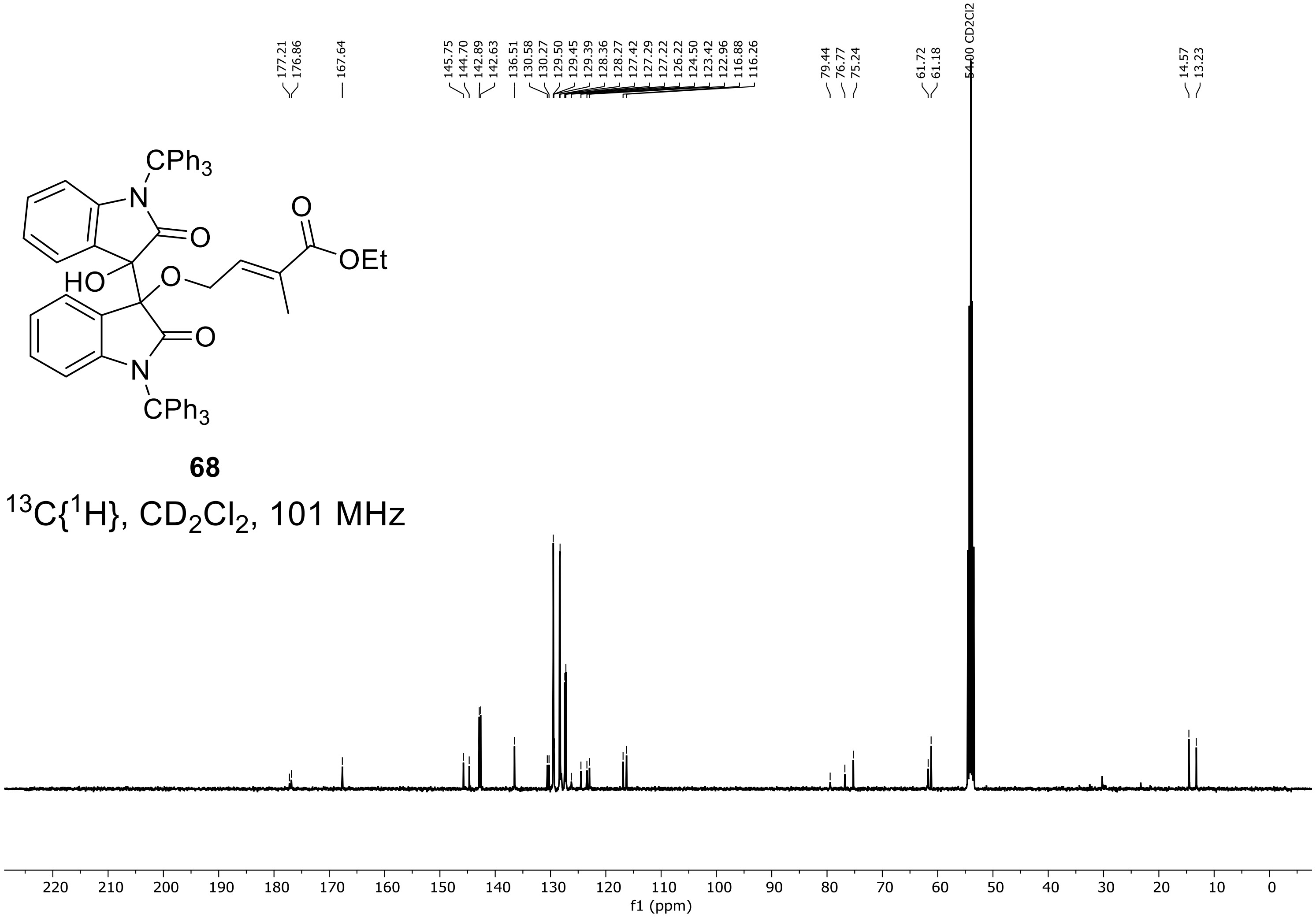

Fig. S484: $^{13}C\{^{1}H\}$ NMR spectrum for Ethyl (*E*)-4-((3-hydroxy-2,2'-dioxo-1,1'-ditrityl-[3,3'-biindolin]-3'-yl)oxy)tiglate (**68**).

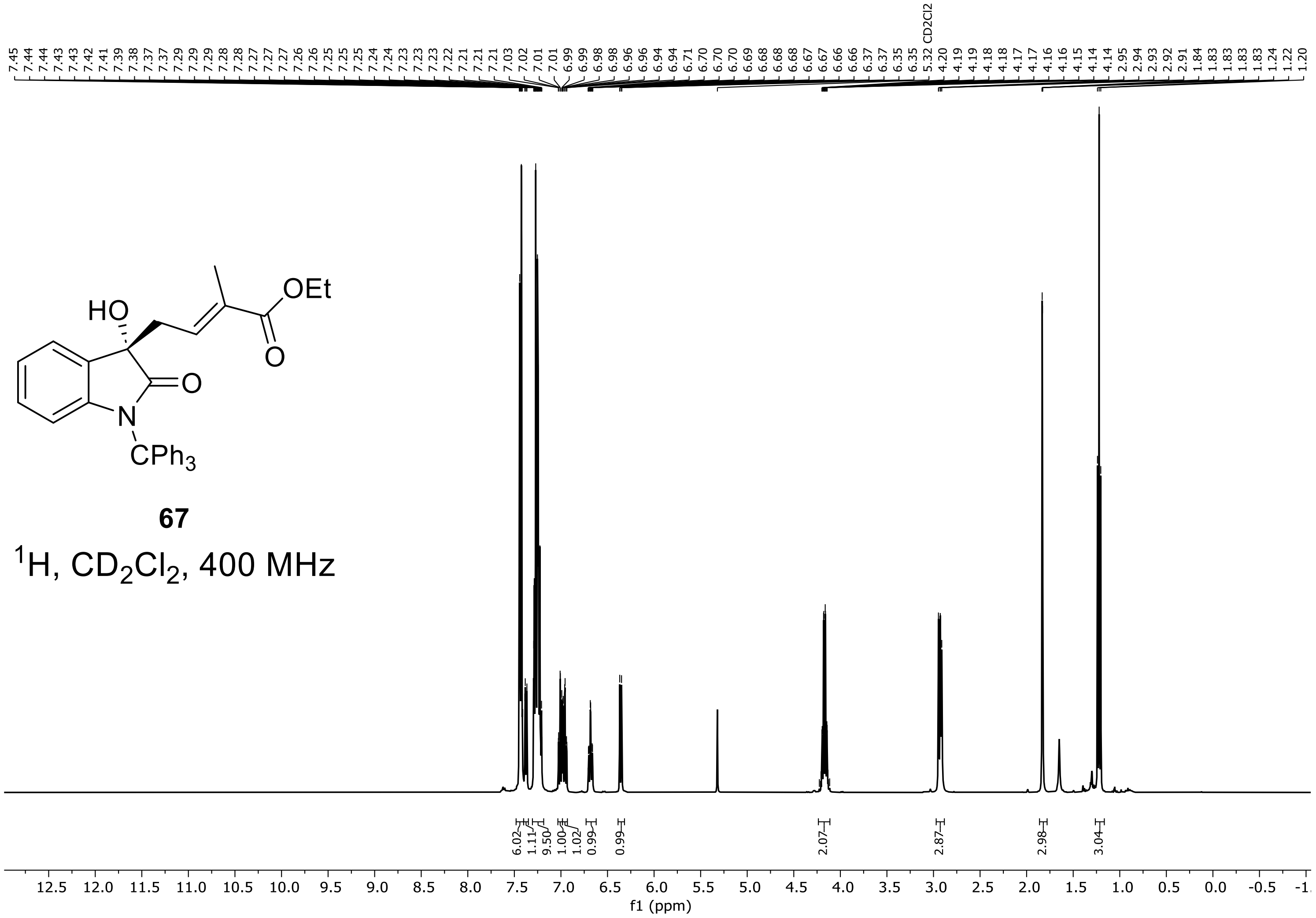

Fig. S485: ^{1}H NMR spectrum for ethyl (*S*,*E*)-4-(3-hydroxy-2-oxo-1-tritylindolin-3-yl)tiglate (**67**).

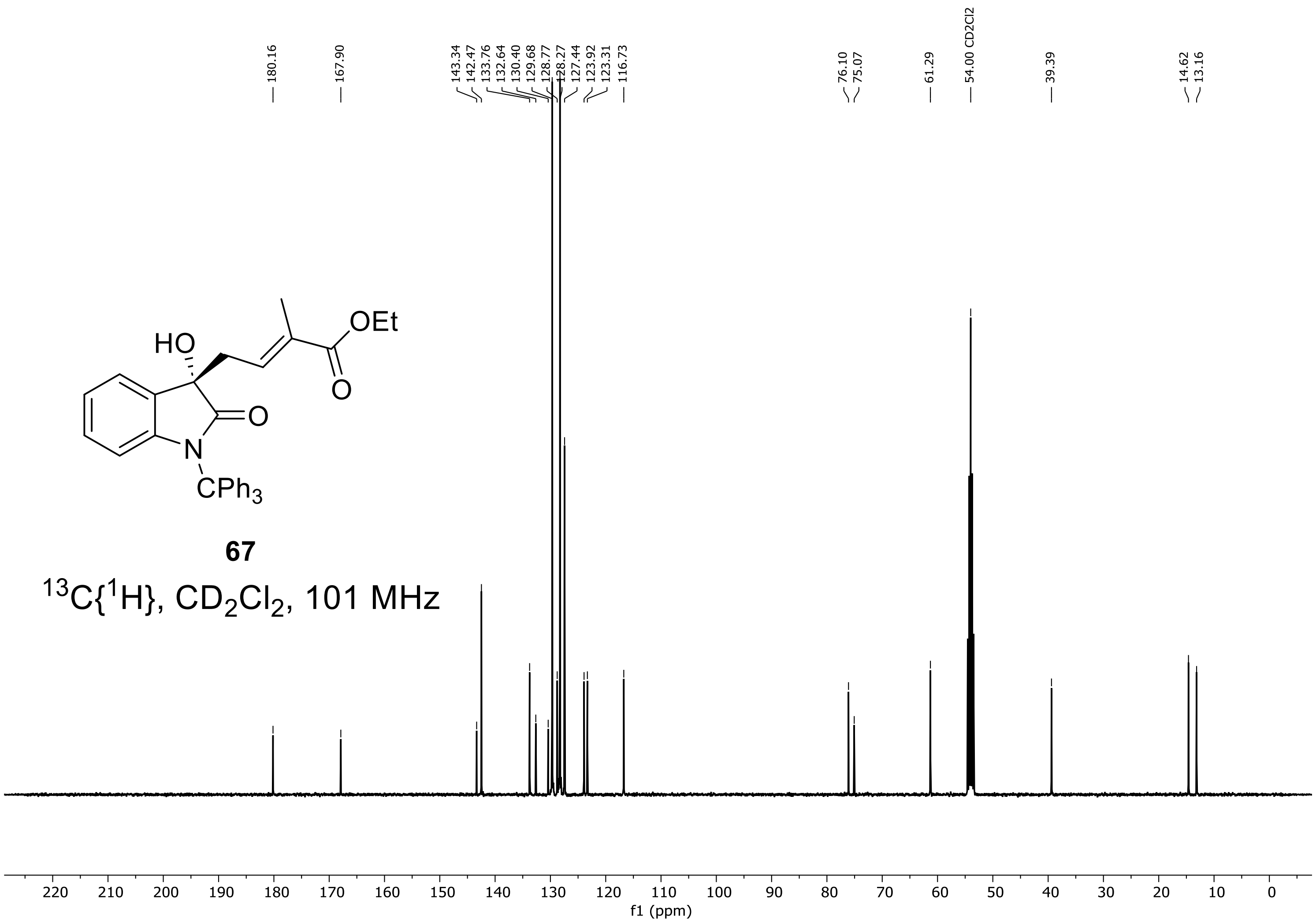

Fig. S486: $^{13}C\{^{1}H\}$ NMR spectrum for ethyl (*S*,*E*)-4-(3-hydroxy-2-oxo-1-tritylindolin-3-yl)tiglate (**67**).

11 Supplementary Reference

1 Núñez, M. G., Farley, A. J. M. & Dixon, D. J. Bifunctional Iminophosphorane Organocatalysts for Enantioselective Synthesis: Application to the Ketimine Nitro-Mannich Reaction. *J. Am. Chem. Soc.* **135**, 16348-16351 (2013). https://doi.org/10.1021/ja409121s

2 Robertson, G. P., Farley, A. J. M. & Dixon, D. J. Bifunctional Iminophosphorane Catalyzed Enantioselective Ketimine Phospha-Mannich Reaction. *Synlett* **27**, 21-24 (2016).

3 Martín, A. *et al.* TensorFlow: Large-Scale Machine Learning on Heterogeneous Systems. (2015).

4 CrystalClear-SM Expert v2.1. Rigaku Americas, The Woodlands, Texas, USA, and Rigaku Corporation, Tokyo, Japan. (2015).

5 CrysAlisPro v1.171.41.93a. or v1.171.42.82a Rigaku Oxford Diffraction, Rigaku Corporation, Oxford, U.K. (2020-2023).

6 Sheldrick, G. SHELXT - Integrated space-group and crystal-structure determination. *Acta Crystallogr., Sect. A: Found. Adv.* **71**, 3-8 (2015). https://doi.org/10.1107/S2053273314026370

7 Sheldrick, G. Crystal structure refinement with SHELXL. *Acta Crystallogr., Sect. C: Struct. Chem.* **71**, 3-8 (2015). https://doi.org/10.1107/S2053229614024218

8 Dolomanov, O. V., Bourhis, L. J., Gildea, R. J., Howard, J. A. K. & Puschmann, H. OLEX2: a complete structure solution, refinement and analysis program. *J. Appl. Crystallogr.* **42**, 339-341 (2009). https://doi.org/10.1107/S0021889808042726

9 Pracht, P., Bohle, F. & Grimme, S. Automated exploration of the low-energy chemical space with fast quantum chemical methods. *Phys. Chem. Chem. Phys.* **22**, 7169-7192 (2020). https://doi.org/10.1039/C9CP06869D

10 Bannwarth, C., Ehlert, S. & Grimme, S. GFN2-xTB—An Accurate and Broadly Parametrized Self-Consistent Tight-Binding Quantum Chemical Method with Multipole Electrostatics and Density-Dependent Dispersion Contributions. *J. Chem. Theory Comput.* **15**, 1652-1671 (2019). https://doi.org/10.1021/acs.jctc.8b01176

11 Chung, L. W. *et al.* The ONIOM Method and Its Applications. *Chem. Rev.* **115**, 5678-5796 (2015). https://doi.org/10.1021/cr5004419

12 Svensson, M. *et al.* ONIOM: A Multilayered Integrated MO + MM Method for Geometry Optimizations and Single Point Energy Predictions. A Test for Diels−Alder Reactions and Pt(P(t-Bu)3)2 + H2 Oxidative Addition. *J. Phys. Chem.* **100**, 19357-19363 (1996). https://doi.org/10.1021/jp962071j

13 Gaussian 16 Rev. C.01, Frisch, M. J., Trucks, G. W., Schlegel, H. B., Scuseria, G. E., Robb, M. A., Cheeseman, J. R., Scalmani, G., Barone, V., Petersson, G. A., Nakatsuji, H., Li, X., Caricato, M., Marenich, A. V., Bloino, J., Janesko, B. G., Gomperts, R., Mennucci, B., Hratchian, H. P., Ortiz, J. V., Izmaylov, A. F., Sonnenberg, J. L., Williams, Ding, F., Lipparini, F., Egidi, F., Goings, J., Peng, B., Petrone, A., Henderson, T., Ranasinghe, D., Zakrzewski, V. G., Gao, J., Rega, N., Zheng, G., Liang, W., Hada, M., Ehara, M., Toyota, K., Fukuda, R., Hasegawa, J., Ishida, M., Nakajima, T., Honda, Y., Kitao, O., Nakai, H., Vreven, T., Throssell, K., Montgomery Jr., J. A., Peralta, J. E., Ogliaro, F., Bearpark, M. J., Heyd, J. J., Brothers, E. N., Kudin, K. N., Staroverov, V. N., Keith, T. A., Kobayashi, R., Normand, J., Raghavachari, K., Rendell, A. P., Burant, J. C., Iyengar, S. S., Tomasi, J., Cossi, M., Millam, J. M., Klene, M.,

Adamo, C., Cammi, R., Ochterski, J. W., Martin, R. L., Morokuma, K., Farkas, O ., Foresman, J. B. & Fox, D. J. Gaussian, Inc., Wallingford CT, 2016.
14 Zhao, Y. & Truhlar, D. G. The M06 suite of density functionals for main group thermochemistry, thermochemical kinetics, noncovalent interactions, excited states, and transition elements: two new functionals and systematic testing of four M06-class functionals and 12 other functionals. *Theor. Chem. Acc.* **120**, 215-241 (2008). https://doi.org/10.1007/s00214-007-0310-x
15 Hehre, W. J., Ditchfield, R. & Pople, J. A. Self—Consistent Molecular Orbital Methods. XII. Further Extensions of Gaussian—Type Basis Sets for Use in Molecular Orbital Studies of Organic Molecules. *J. Chem. Phys.* **56**, 2257-2261 (1972). https://doi.org/10.1063/1.1677527
16 Dewar, M. J., Zoebisch, E. G., Healy, E. F. & Stewart, J. J. Development and use of quantum mechanical molecular models. 76. AM1: a new general purpose quantum mechanical molecular model. *J. Am. Chem. Soc.* **107**, 3902-3909 (1985).
17 Grayson, M. N., Pellegrinet, S. C. & Goodman, J. M. Mechanistic Insights into the BINOL-Derived Phosphoric Acid-Catalyzed Asymmetric Allylboration of Aldehydes. *J. Am. Chem. Soc.* **134**, 2716-2722 (2012). https://doi.org/10.1021/ja210200d
18 Lam, C. C. & Goodman, J. M. Computational insights on the origin of enantioselectivity in reactions with diarylprolinol silyl ether catalysts via a radical pathway. *Org. Chem. Front.* **9**, 3730-3738 (2022). https://doi.org/10.1039/D2QO00354F
19 Simón, L. Enantioselectivity in CPA-catalyzed Friedel–Crafts reaction of indole and N-tosylimines: a challenge for guiding models. *Org. Biomol. Chem.* **16**, 2225-2238 (2018). https://doi.org10.1039/C7OB02875J
20 Simón, L. & Goodman, J. M. Theoretical Study of the Mechanism of Hantzsch Ester Hydrogenation of Imines Catalyzed by Chiral BINOL-Phosphoric Acids. *J. Am. Chem. Soc.* **130**, 8741-8747 (2008). https://doi.org/10.1021/ja800793t
21 Simón, L. & Goodman, J. M. Mechanism of BINOL−Phosphoric Acid-Catalyzed Strecker Reaction of Benzyl Imines. *J. Am. Chem. Soc.* **131**, 4070-4077 (2009). https://doi.org/10.1021/ja808715j
22 Simón, L. & Goodman, J. M. What is the mechanism of amine conjugate additions to pyrazole crotonate catalyzed by thiourea catalysts? *Org. Biomol. Chem.* **7**, 483-487 (2009). https://doi.org/10.1039/B817283H
23 Simón, L. & Goodman, J. M. A Model for the Enantioselectivity of Imine Reactions Catalyzed by BINOL−Phosphoric Acid Catalysts. *J. Org. Chem.* **76**, 1775-1788 (2011). https://doi.org/10.1021/jo102410r
24 Simón, L. & Goodman, J. M. How reliable are DFT transition structures? Comparison of GGA, hybrid-meta-GGA and meta-GGA functionals. *Org. Biomol. Chem.* **9**, 689-700 (2011). https://doi.org/10.1039/C0OB00477D
25 Weigend, F. & Ahlrichs, R. Balanced basis sets of split valence, triple zeta valence and quadruple zeta valence quality for H to Rn: Design and assessment of accuracy. *Phys. Chem. Chem. Phys.* **7**, 3297-3305 (2005). https://doi.org/10.1039/B508541A
26 Cancès, E., Mennucci, B. & Tomasi, J. A new integral equation formalism for the polarizable continuum model: Theoretical background and applications to isotropic and anisotropic dielectrics. *J. Chem. Phys.* **107**, 3032-3041 (1997). https://doi.org/10.1063/1.474659

27 Bauzá, A., Quiñonero, D., Deyà, P. M. & Frontera, A. Is the Use of Diffuse Functions Essential for the Properly Description of Noncovalent Interactions Involving Anions? *J. Phys. Chem. A* **117**, 2651-2655 (2013). https://doi.org/10.1021/jp312755z

28 Luchini, G., Alegre-Requena, J. V., Funes-Ardoiz, I. & Paton, R. S. GoodVibes: automated thermochemistry for heterogeneous computational chemistry data. *F1000Research* **9**, 291 (2020). https://doi.org/10.12688/f1000research.22758.1

29 Ribeiro, R. F., Marenich, A. V., Cramer, C. J. & Truhlar, D. G. Use of Solution-Phase Vibrational Frequencies in Continuum Models for the Free Energy of Solvation. *J. Phys. Chem. B* **115**, 14556-14562 (2011). https://doi.org/10.1021/jp205508z

30 Li, Y.-P., Gomes, J., Mallikarjun Sharada, S., Bell, A. T. & Head-Gordon, M. Improved Force-Field Parameters for QM/MM Simulations of the Energies of Adsorption for Molecules in Zeolites and a Free Rotor Correction to the Rigid Rotor Harmonic Oscillator Model for Adsorption Enthalpies. *J. Phys. Chem. C* **119**, 1840-1850 (2015). https://doi.org/10.1021/jp509921r

31 Alecu, I. M., Zheng, J., Zhao, Y. & Truhlar, D. G. Computational Thermochemistry: Scale Factor Databases and Scale Factors for Vibrational Frequencies Obtained from Electronic Model Chemistries. *J. Chem. Theory Comput.* **6**, 2872-2887 (2010). https://doi.org/10.1021/ct100326h

32 GaussView, Version 6, Dennington, Roy; Keith, Todd A.; Millam, John M. Semichem Inc., Shawnee Mission, KS, 2016.

33 CYLview20, Legault, C.,(Université de Sherbrooke, 2020), http://www.cylview.org.

34 Zhang, H.-h. *et al.* NaI-mediated divergent synthesis of isatins and isoindigoes: a new protocol enabled by an oxidation relay strategy. *Chem. Commun.* **54**, 8265-8268 (2018). https://doi.org/10.1039/C8CC04471F

35 Ryu, H., Seo, J. & Ko, H. M. Synthesis of Spiro[oxindole-3,2′-pyrrolidine] Derivatives from Benzynes and Azomethine Ylides through 1,3-Dipolar Cycloaddition Reactions. *J. Org. Chem.* **83**, 14102-14109 (2018). https://doi.org/10.1021/acs.joc.8b02117

36 Shintani, R., Takatsu, K. & Hayashi, T. Copper-catalyzed asymmetric addition of arylboronates to isatins: a catalytic cycle involving alkoxocopper intermediates. *Chem. Commun.* **46**, 6822-6824 (2010). https://doi.org/10.1039/C0CC01635G

37 Yamamoto, Y., Yohda, M., Shirai, T., Ito, H. & Miyaura, N. Me-BIPAM for the Synthesis of Optically Active 3-Aryl-3-hydroxy-2-oxindoles by Ruthenium-catalyzed Addition of Arylboronic Acids to Isatins. *Chem. – Asian J.* **7**, 2446-2449 (2012). https://doi.org/10.1002/asia.201200481

38 Zhu, J. *et al.* Enantioselective Rhodium-Catalyzed Addition of Arylboroxines to N-Unprotected Ketimines: Efficient Synthesis of Cipargamin. *Angew. Chem. Int. Ed.* **58**, 16119-16123 (2019). https://doi.org/10.1002/anie.201910008

39 Greenhalgh, M. D. *et al.* A C=O···Isothiouronium Interaction Dictates Enantiodiscrimination in Acylative Kinetic Resolutions of Tertiary Heterocyclic Alcohols. *Angew. Chem. Int. Ed.* **57**, 3200-3206 (2018). https://doi.org/10.1002/anie.201712456

40 Reddy, A. C. S., Reddy, P. M. & Anbarasan, P. Diastereoselective Palladium Catalyzed Carbenylative Amination of ortho-Vinylanilines with 3-Diazoindolin-2-ones. *Adv. Synth. Catal.* **362**, 801-806 (2020). https://doi.org/10.1002/adsc.201901286

41 Yao, X., Wang, T. & Zhang, Z. Gold(I)-Catalyzed Dimerization of 3-Diazooxindoles towards Isoindigos. *Eur. J. Org. Chem.* **2018**, 4475-4478 (2018). https://doi.org/10.1002/ejoc.201800809

42 Li, P.-F., Wang, H.-L. & Qu, J. 1,n-Rearrangement of Allylic Alcohols Promoted by Hot Water: Application to the Synthesis of Navenone B, a Polyene Natural Product. *J. Org. Chem.* **79**, 3955-3962 (2014). https://doi.org/10.1021/jo5004086

43 Huang, Y.-K., Zhang, W.-Z., Zhang, K., Wang, W.-L. & Lu, X.-B. Carbon dioxide-promoted palladium-catalyzed dehydration of primary allylic alcohols: access to substituted 1,3-dienes. *Org. Chem. Front.* **8**, 941-946 (2021). https://doi.org/10.1039/D0QO01465F

44 Nguyen, T. N. T., Thiel, N. O. & Teichert, J. F. Copper(i)-catalysed asymmetric allylic reductions with hydrosilanes. *Chem. Commun.* **53**, 11686-11689 (2017). https://doi.org/10.1039/C7CC07008J

45 Mantilli, L., Gérard, D., Torche, S., Besnard, C. & Mazet, C. Iridium-Catalyzed Asymmetric Isomerization of Primary Allylic Alcohols. *Angew. Chem. Int. Ed.* **48**, 5143-5147 (2009). https://doi.org/10.1002/anie.200901863

46 Zi, W., Wang, Y.-M. & Toste, F. D. An In Situ Directing Group Strategy for Chiral Anion Phase-Transfer Fluorination of Allylic Alcohols. *J. Am. Chem. Soc.* **136**, 12864-12867 (2014). https://doi.org/10.1021/ja507468u

47 Zhou, X., Zhang, G., Huang, R. & Huang, H. Palladium-Catalyzed Allyl–Allyl Reductive Coupling of Allylamines or Allylic Alcohols with H2 as Sole Reductant. *Org. Lett.* **23**, 365-369 (2021). https://doi.org/10.1021/acs.orglett.0c03865

48 Moghadam, F. A. *et al.* Ir-Catalyzed Asymmetric Allylic Alkylation of Dialkyl Malonates Enabling the Construction of Enantioenriched All-Carbon Quaternary Centers. *J. Am. Chem. Soc.* **144**, 7983-7987 (2022). https://doi.org/10.1021/jacs.2c02960

49 Bernasconi, M., Ramella, V., Tosatti, P. & Pfaltz, A. Iridium-Catalyzed Asymmetric Hydrogenation of 3,3-Disubstituted Allylic Alcohols in Ethereal Solvents. *Chem. – Eur. J.* **20**, 2440-2444 (2014). https://doi.org/10.1002/chem.201303915

50 Li, H. *et al.* Selective Synthesis of Z-Cinnamyl Ethers and Cinnamyl Alcohols through Visible Light-Promoted Photocatalytic E to Z Isomerization. *Chem. – Asian J.* **15**, 555-559 (2020). https://doi.org/10.1002/asia.201901778

51 Kasten, K., Slawin, A. M. Z. & Smith, A. D. Enantioselective Synthesis of β-Fluoro-β-aryl-α-aminopentenamides by Organocatalytic [2,3]-Sigmatropic Rearrangement. *Org. Lett.* **19**, 5182-5185 (2017). https://doi.org/10.1021/acs.orglett.7b02452

52 Lölsberg, W., Ye, S. & Schmalz, H.-G. Enantioselective Copper-Catalysed Allylic Alkylation of Cinnamyl Chlorides by Grignard Reagents using Chiral Phosphine-Phosphite Ligands. *Adv. Synth. Catal.* **352**, 2023-2031 (2010). https://doi.org/10.1002/adsc.201000213

53 Wang, G., Gan, Y. & Liu, Y. Nickel-Catalyzed Direct Coupling of Allylic Alcohols with Organoboron Reagents. *Chin. J. Chem.* **36**, 916-920 (2018). https://doi.org/10.1002/cjoc.201800237

54 Guo, Y. & Shen, Z. Palladium-catalyzed allylic C–H oxidation under simple operation and mild conditions. *Org. Biomol. Chem.* **17**, 3103-3107 (2019). https://doi.org/10.1039/C9OB00209J

55 Craig, D. & Slavov, N. K. A quantitative structure–reactivity relationship in decarboxylative Claisen rearrangement reactions of allylic tosylmalonate esters. *Chem. Commun.*, 6054-6056 (2008). https://doi.org/10.1039/B812306C

56 Papa Spadafora, B. *et al.* Regio- and diastereoselective Pd-catalyzed aminochlorocyclization of allylic carbamates: scope, derivatization, and mechanism. *Org. Biomol. Chem.* **19**, 5595-5606 (2021). https://doi.org/10.1039/D1OB00670C

57 Kotoku, N. *et al.* Concise synthesis and structure–activity relationship of furospinosulin-1, a hypoxia-selective growth inhibitor from marine sponge. *Tetrahedron* **67**, 6673-6678 (2011). https://doi.org/10.1016/j.tet.2011.05.009

58 Nguyen, T. N. T., Thiel, N. O., Pape, F. & Teichert, J. F. Copper(I)-Catalyzed Allylic Substitutions with a Hydride Nucleophile. *Org. Lett.* **18**, 2455-2458 (2016). https://doi.org/10.1021/acs.orglett.6b00941

59 Rodríguez-Fernández, L., Lavandera, I. & Gotor-Fernández, V. Photocatalytic Oxidative Cleavage of Alkenes Followed by Carbonyl Stereoselective Bioreduction for the Synthesis of Enantioenriched Secondary Alcohols. *Adv. Synth. Catal.* **366**, 900-908 (2024). https://doi.org/10.1002/adsc.202301325

60 Mo, H. *et al.* Metal-Free, One-Pot Synthesis of Allylic and Benzylic Esters via Decarboxylation and C–H Bond Activation. *Synthesis* **47**, 209-215 (2015). https://doi.org/10.1055/s-0034-1379142

61 Hartley, W. C. *et al.* In-Cage Recombination Facilitates the Enantioselective Organocatalytic [1,2]-Rearrangement of Allylic Ammonium Ylides. *J. Am. Chem. Soc.* **147**, 1101-1111 (2025). https://doi.org/10.1021/jacs.4c14516
